# Identification of Differentially Expressed circRNAs, miRNAs, and Genes in Patients Associated with Cartilaginous Endplate Degeneration

**DOI:** 10.1155/2021/2545459

**Published:** 2021-05-18

**Authors:** Haiwei Xu, Yongjin Li, Jianhua Li, Zhenxin Huo, Guowang Li, Lilong Du, Lijun Tian, Baoshan Xu

**Affiliations:** ^1^Department of Minimally Invasive Spine Surgery, Tianjin Hospital, No. 406, Jiefangnan Road, Hexi District, Tianjin 300211, China; ^2^Graduate School, Tianjin Medical University, 22 Qixiangtai Road, Tianjin, China

## Abstract

**Background:**

Intervertebral disc degeneration (IDD) disease is a global challenge because of its predominant pathogenic factor in triggering low back pain, whereas cartilaginous endplate degeneration (CEPD) is the main cause of IDD. Accumulating evidence have indicated that the differentially expressed microRNAs (DEMs) and differentially expressed genes (DEGs) have been determined to be involved in multiple biological processes to mediate CEPD progression. However, the differentially expressed circular RNAs (DECs) and their potential biofunctions in CEPD have not been identified.

**Methods:**

GSE153761 dataset was analyzed using R software to predict DECs, DEMs, and DEGs. Pathway enrichment analysis of DEGs and host genes of DECs and protein-protein interaction network of DEGs were conducted to explore their potential biofunctions. Furthermore, we explore the potential relationship between DEGs and DECs.

**Results:**

There were 74 DECs, 17 DEMs, and 68 DEGs upregulated whereas 50 DECs, 16 DEMs, and 67 DEGs downregulated in CEPD group. Pathway analysis unveiled that these RNAs might regulate CEPD via mediating inflammatory response, ECM metabolism, chondrocytes apoptosis, and chondrocytes growth. A total of 17 overlapping genes were predicted between the host genes of DEGs and DECs, such as SDC1 and MAOA. Moreover, 6 upregulated DECs, of which hsa_circ_0052830 was the most upregulated circRNA in CEPD, were derived from the host genes SDC1, whereas 8 downregulated DECs were derived from the host genes MAOA.

**Conclusion:**

This will provide novel clues for future experimental studies to elucidate the pathomechanism of CEPD and therapeutic targets for CEPD-related diseases.

## 1. Introduction

Intervertebral disc degeneration (IDD) diseases are a global challenge that contributed to dyskinesia in patients and the increased of socioeconomic burden [1–3]. The intervertebral disc (IVD) is divided into 4 regions: the annulus fibrosus, the transitional zone, the central nucleus pulposus (NP), and up-down cartilaginous endplates (CEP) [4, 5]. IVD is an avascular structure on account of the blood vessels derived from the lumbar artery terminate at the CEP [5]. The CEP acts as an exchange channel, which is responsible for the metabolic waste transportation and nutrient intake of IVD [5–7]. During IDD, the water in NP tissue continually decreases, causing the pyknotic substances deposited in the CEP and gradually calcifies, then the calcified CEP leads to the decrease of CEP permeability [8].CEP degeneration (CEPD) caused by endplate chondrocyte death and calcification can block this exchange channel, which further aggravates IDD and the failure of endogenous repair, and ultimately forms a vicious cycle [9, 10]. Additionally, the abnormal increase of proinflammatory cytokine secretion and extracellular matrix (ECM) degradation is another characteristic of CEPD [9, 11]. Thus, it is very necessary to find a novel treatment method to mitigate CEPD.

In recent years, noncoding RNAs (ncRNAs) have drawn growing attention because of the role which they play in a spectrum of chronic degenerative diseases of the spine and joints, encompassing IDD [12–16], ankylosing spondylitis [17], osteoarthritis [18, 19], and rheumatoid arthritis [20]. Circular RNAs (circRNAs), microRNAs (miRNAs), and long ncRNAs are the members of the family of ncRNAs. MiRNAs bind to their target mRNAs to initiate degradation or repress their translation [21].CircRNAs have been determined to be involved in multiple biological processes to mediate IDD progression, such as cell death and growth, as well as inflammatory response and ECM metabolism [12–16]. Furthermore, circRNAs have also been demonstrated to have the therapeutic effect on IDD model rats [14–16]. Mechanistically, circRNAs acts as a competitive endogenous RNA (ceRNA) to sponge miRNAs, thereby positively regulating target mRNAs expression [12]. However, the potential biofunctions of circRNAs in CEPD of cervical vertebra is still a blank. Thus, this study aims to explore new circRNAs and mRNAs that are relevant to CEPD.

In our study, we downloaded the CEPD-related microarray dataset from public Gene Expression Omnibus (GEO) database (http://www.ncbi.nlm.nih.gov/geo) [22] to analyze and select the differentially expressed circRNAs, miRNAs, and genes (DECs, DEMs, and DEGs) associated with chondrocytes metabolism. Integrated bioinformatics methods, containing Gene Ontology (GO), Kyoto Encyclopedia of Gene and Genomes (KEGG), and protein-protein interaction (PPI) network analysis, were conducted to explore the potential biofunctions of DECs, DEMs, and DEGs, which will provide novel clues to elucidate the pathomechanism of CEPD for future experimental studies.

## 2. Methods

### 2.1. Analysis of GSE153761 Microarray Dataset

Opening the accession number GSE153761 in GEO database, then these raw data were normalized and log2-transformed using R software package limma [23]. The differentially expressed circRNAs (DECs) and differentially expressed genes (DEGs) were identified using the criterion of ∣log_2_ (fold − change) | >2 and *p* value < 0.05, while the differentially expressed microRNAs (DEMs) were selected based on the criterion of ∣log_2_ (fold − change) | >1 and *p* value < 0.05. The box plot is performed using the R software package ggplot2; the heatmap is performed using the R software package pheatmap. Volcano plot, bubble diagram, and chord plot were performed using the Sangerbox tools, a free online platform for data analysis (http://www.sangerbox.com/tool). All the above analysis methods and R package were performed by R foundation for statistical computing.

### 2.2. Venn Analysis

The DEGs and the host genes of DECs was intersected to select overlapping genes using Venn plot analysis by Sangerbox tools.

### 2.3. Pathways Enrichment Analysis

GO, KEGG, and Reactome pathway enrichment analysis were conducted by the Database for Annotation, Visualization, and Integrated Discovery (DAVID) online tools (https://david-d.ncifcrf.gov/) [24]. The analytical results were visualized by bubble and chord plot using Sangerbox tool. A value of *p* < 0.05 was set as a statistically significant difference.

### 2.4. PPI Network Construction

The Search Tool for the Retrieval of Interacting Genes (STRING) database was utilized to seek the interactions between multiple proteins [25]. The PPI network was constructed by STRING to predict the potential relationship among those DEGs. Furthermore, those proteins that interact with each other were extracted from the PPI network and visualized by Cytoscape software [26]. The CytoHubba plugin in Cytoscape software was utilized to find the hub genes [27].

### 2.5. Statistical Analysis

Statistical analysis was conducted by R (version 3.6.2) software. For all data analyses, a value of *p* < 0.05 was set as a statistically significant difference.

## 3. Results

### 3.1. Identification of the DECs, DEMs, and DEGs from GEO Database

**Figure 1:**
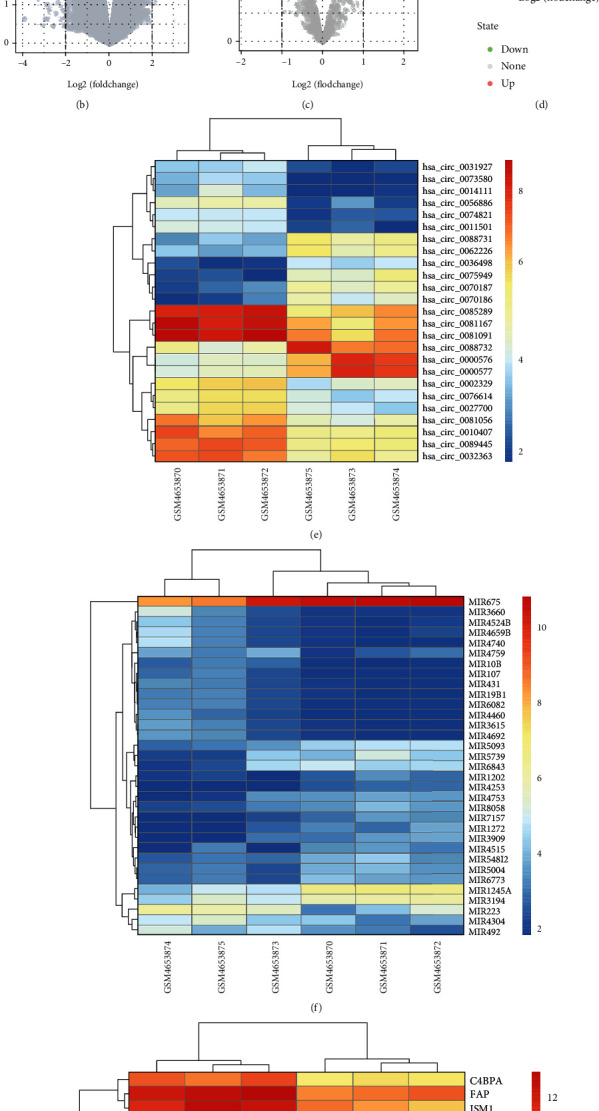
Identification of the DECs, DEMs, and DEGs in GSE153761 dataset. (a) After normalization, the box plot revealed the distribution trends among six samples were nearly the same. The row represents the sample, and the column represents the data expression value in the sample. (b–d) Volcano plot visualizing DECs, DEMs, and DEGs expression between the two groups. Green points represent downregulation (left side), gray points represent undifferential expression (middle), and red points represent upregulation (right side). (e, f) Clustering heatmaps for the DECs, DEMs, and DEGs, with rows representing genes and columns representing samples. The color scale varies from red to blue; red points represent downregulation, and blue points represent downregulation.

GSE153761 microarray dataset was downloaded from GEO database, which included circRNA, miRNA, and gene expression data. The fundamental information of GSE153761 dataset was shown in Table 1. As shown in Figure 1(a), the box plot revealed the distribution trends of the 6 different samples were the same, that is, they are all on the same line, suggesting the data have already been standardized. Then, the R software limma package was utilized to identify DECs, DEMs, and DEGs. The results of analysis unveiled that a total of 88731 circRNAs, 1309 miRNAs, and 18655 genes were identified (Supplementary Table 1). Compared with the expression of circRNAs, miRNAs, and genes in the normal CEP group, a volcano plot reflected that there were 74 DECs, 17 DEMs, and 68 DEGs upregulated whereas 50 DECs, 16 DEMs, and 67 DEGs downregulated in CEPD group with the screening criteria of ∣log_2_ (fold − change) | >2 and *p* value < 0.05 (Figures 1(b)–1(d), Supplementary Table 2). Furthermore, we displayed the top 25 DECs and DEGs as well as the 33 DEMs through heatmap (Figures 1(e)–1(g)), from which we found that the 3 CEPD samples can be remarkably separated from the 3 control samples, suggesting that the results of analysis were trustable.

### 3.2. Functional Enrichment Analysis of the DEGs

**Figure 2:**
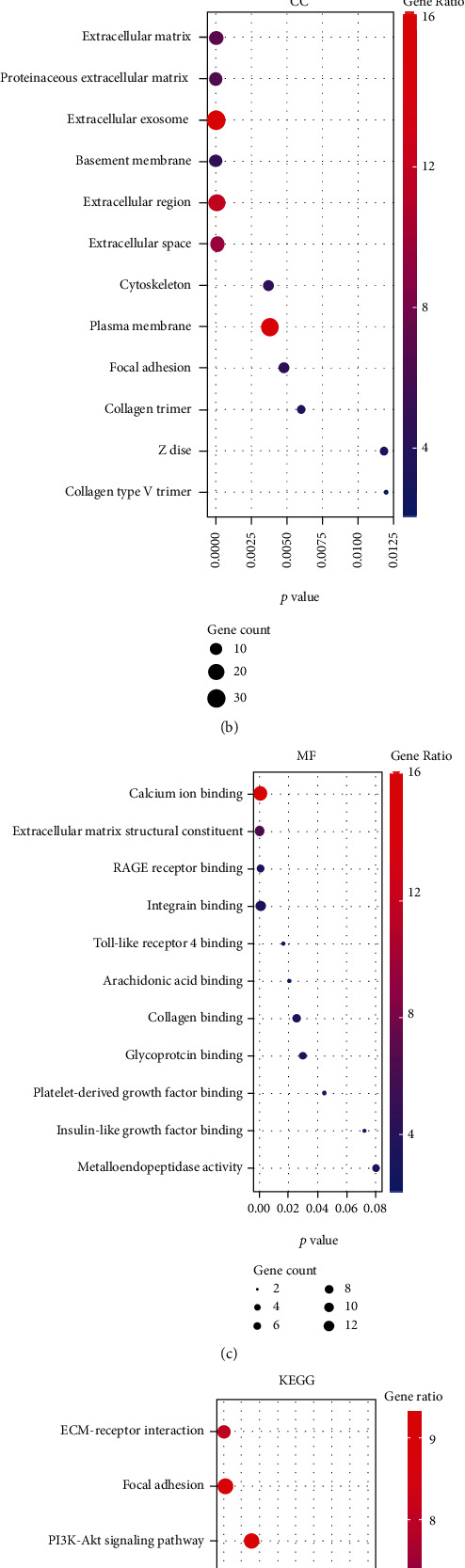
Functional enrichment analysis of the DEGs. (a–c) Most enriched Gene Ontology (GO) terms in biological process (BP), cellular component (CC), and molecular function (MF). The *x*-axis represents the *p* value, the left *y*-axis represents the GO terms, and the right *y*-axis represents the gene ratio (up) and gene count (down). (d) Bubble diagram exhibited the Kyoto Encyclopedia of Genes and Genomes (KEGG) enrichment analysis results of the DEGs. (e) The results of reactome pathway analysis of the DEG was shown by bubble diagram.

To find the potential etiological factors and key genes that correlated with the pathomechanism of CEPD, the analysis of GO, KEGG, and reactome enrichment pathways was conducted for DEGs. GO function annotations included terms in the biological process (BP), molecular function (MF), and cellular component (CC) categories. As shown in Figure 2(a), the 19 BP terms were enriched, such as positive regulation of apoptotic cell clearance, inflammatory response, neutrophil chemotaxis, endochondral ossification, angiogenesis, chemokine production, regulation of endothelial cell proliferation, and collagen catabolic process. Extracellular space, extracellular exosome, proteinaceous extracellular matrix, blood microparticle secretory granule, and cell junction were the enriched important CC terms (Figure 2(b)). The MF were predicted to be mainly enriched in the following terms: receptor for advanced glycosylation end products (RAGE) receptor binding, serine-type endopeptidase activity, arachidonic acid binding, endopeptidase inhibitor activity, growth factor activity, and Toll-like receptor 4 binding (Figure 2(c)). KEGG analysis revealed those DEGs were predominantly enriched in the salivary secretion, complement and coagulation cascades, systemic lupus erythematosus, amoebiasis, and staphylococcus aureus infection (Figure 2(d)). Moreover, the reactome pathways in which these genes were enriched included regulation of complement cascade and activation of C3 and C5, alpha-defensins, aefensins, and erythrocytes take up oxygen and release carbon dioxide (Figure 2(e)). It is not hard to see that those DEGs were predicted to participate in inflammatory response, ossification, angiogenesis, ECM catabolic process, cell proliferation, and apoptosis, while these pathological processes were the crucial pathogenesis of CEPD.

### 3.3. Construction of PPI Network

**Figure 3:**
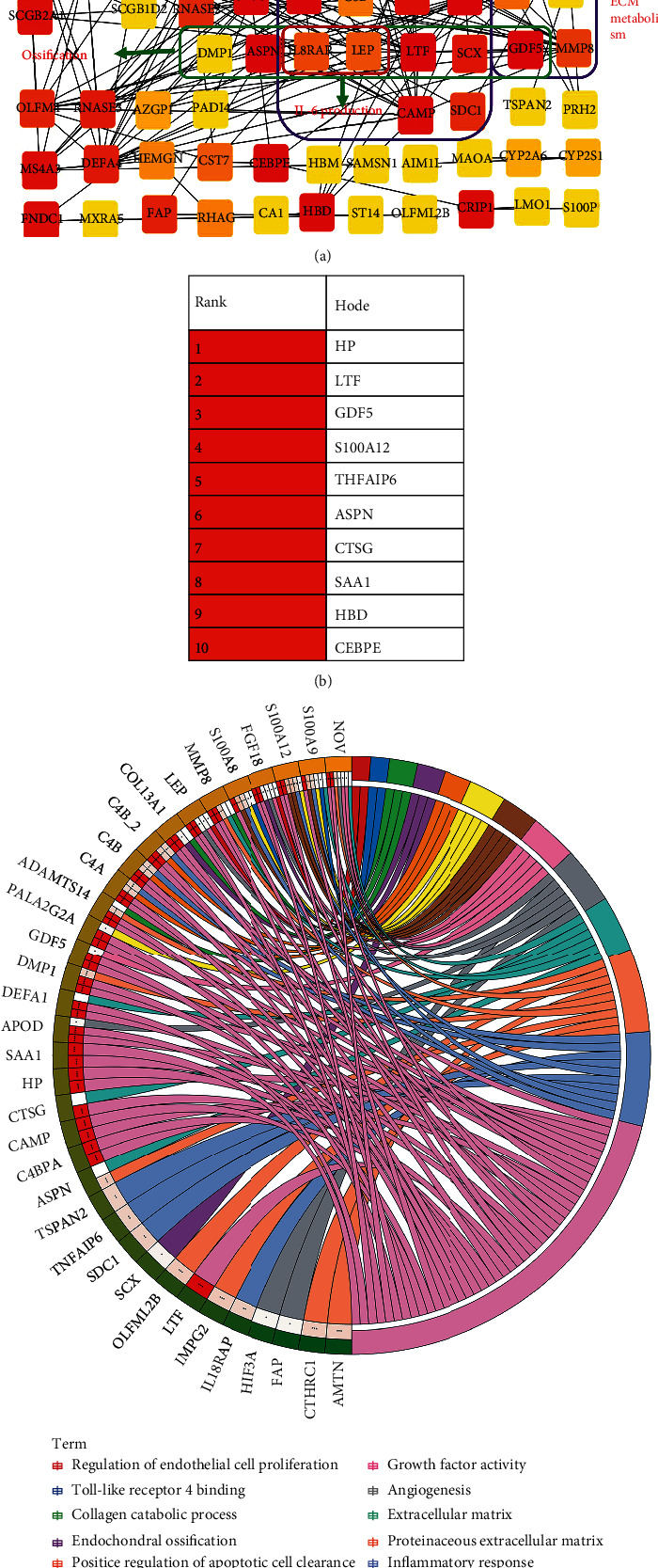
Protein-protein interaction (PPI) network analysis of DEGs. (a) PPI network was constructed and visualized with DEGs through using STRING tool and Cytoscape software. (b) Subnetwork of top 10 hub genes that extracted from (a), and the depth of the color represents the level of the betweenness score. (c) Chord plot shown the important GO enrichments analysis terms of DEGs that associated with cartilage.

PPI is an invaluable tool for studying the potential interrelationship among multiple proteins, so we mapped the 135 DEGs into STRING website to analyze the interaction among them. The PPI network stats consisted of 134 nodes and 207 edges, of which 85 nodes were found to be involved in the PPI pairs. Subsequently, these genes with interaction were ranked using betweenness method and visualized via cytoHubba plug-in of Cytoscape software (Figure 3(a)). Among them, haptoglobin (HP), scleraxis (SCX), lactoferrin (LTF), TNF*α*-stimulated gene-6 (TNFAIP6), syndecan 1(SDC1), leptin (LEP), interleukin 18 receptor accessory protein (IL18RAP), cathelicidin antimicrobial peptide (CAMP), apolipoprotein D (APOD), C4b-binding protein *α*-chain (C4BPA), S100 calcium-binding protein A8 (S100A8), S100A12, S100A9, complement C8 beta chain (C8B), phospholipase A2 group IIA (PLA2G2A), serum amyloid A1 (SAA1), complement C8 alpha chain (C4A), and C4B were associated with inflammatory response, dentin matrix protein 1 (DMP1), asporin (ASPN), growth and differentiation factor-5 (GDF5), metaloproteinase-8 (MMP8), LEP, IL18RAP, SCX, and LTF were associated with ossification, as well as GDF5, MMP8, arginase-1 (ARG1), and A disintegrin and metalloproteinase with thrombospondin motif 14 (ADAMTS14) were associated with ECM metabolism. As shown in Figure 3(b), the top 5 key hub genes were HP, LTF, GDF5, S100A12, and TNFAIP6. Furthermore, the important GO functional analysis results of all DEGs which contained genes not appearing in PPI pairs that associated with cartilage cell metabolism were displayed through chord plot (Figure 3(c)). The results indicated that collagen type III *α* 1 chain (COL3A1) is involved in “endochondral ossification,” “collagen catabolic process,” and “extracellular region,” as well as fibroblast growth factor 18 (FGF18) is involved in “endochondral ossification,” “angiogenesis,” “regulation of endothelial cell proliferation,” “extracellular region,” and “growth factor activity.”.

### 3.4. Finding the Potential Relationship between DEGs and DECs

**Figure 4:**
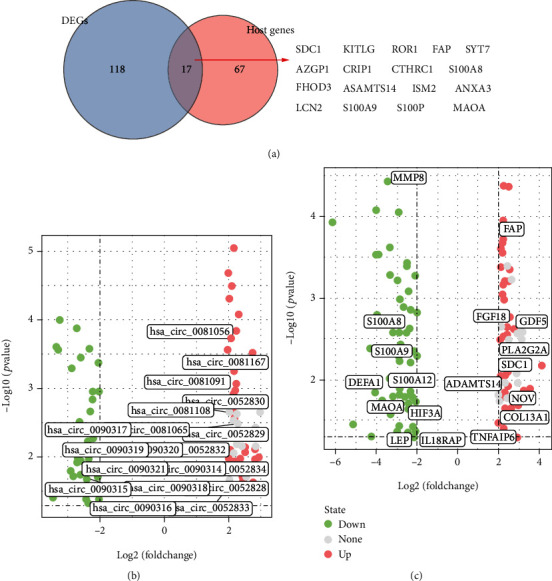
Finding the potential relationship between DEGs and DECs. (a) Venn diagram (left) shown the overlapping genes in the DEGs (green) and host genes of DECs (red), the right half displayed the specific 17 overlapping genes. (b) Volcano plot visualizing the multiple DECs that derived from one host genes. (c) Volcano plot visualizing the key DEGs that might play a role in regulating the pathological processes of CEPD.

**Figure 5:**
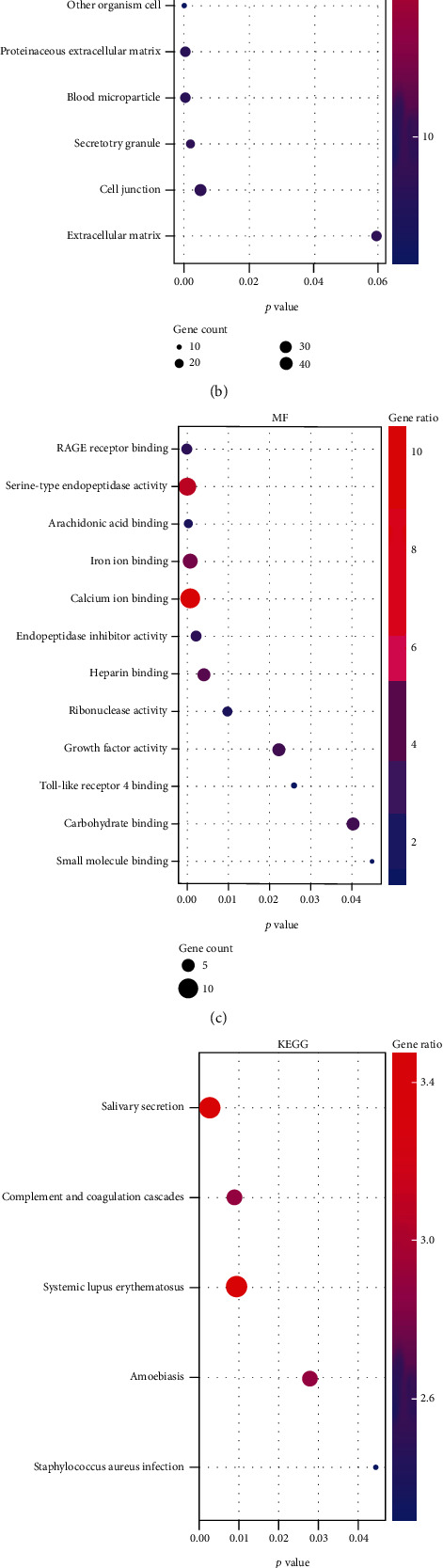
Gene Ontology (GO) and Kyoto Encyclopedia of Genes and Genomes (KEGG) enrichment analysis of host genes of the DECs. (a–c) Most enriched GO terms in biological process (BP), cellular component (CC), and molecular function (MF). The *x*-axis represents the *p* value, the left *y*-axis represents the GO terms, and the right *y*-axis represents the gene ratio (up) and gene count (down). (d) Bubble diagram exhibited the KEGG enrichment analysis results of the host genes.

Growing evidence has unveiled that circRNAs not only can regulate the expression levels of their host genes in a miRNA-dependent manner [28, 29] but also can mediate host gene alternative splicing or transcription in a miRNA-independent manner [30, 31]. For instance, circ-F-box and WD repeat domain-containing 7 (FBXW7) regulates the mRNA and protein levels of FBXW7 via sponging miR-197-3p [28]. Bai et al. [29] reported that circ-filamin-binding LIM protein 1 (FBLIM1) acts as a ceRNA to positively modulate its host gene *Fblim1* expression by repressing miR-346. A circRNA derived from *Sepallata3* gene was validated to mediate its alternative splicing through R-loop formation. Li et al. [31] demonstrated that circ-EIF3J and circ-PAIP2 can interact with U1 small nuclear ribonucleoprotein and RNA polymerase II to promote their host gene transcription. To explore whether there was a relationship between DEGs and DECs, Venn analysis was conducted to predict that there were 17 overlapping genes between the host genes of DEGs and DECs, such as SDC1 and monoamine oxidase A (MAOA) (Figure 4(a)). Intriguingly, the DECs, including hsa_circ_0052828, hsa_circ_0052829, hsa_circ_0052830, hsa_circ_0052832, hsa_circ_0052833, and hsa_circ_0052834, were all predicted upregulated and derived from the host genes SDC1, of which hsa_circ_0052830 was predicted to be the most upregulated circRNA in CEPD (Figure 5(b) and Supplementary Table 3). Additionally, the DECs predicted to be downregulated in CEPD, such as hsa_circ_0090314, hsa_circ_0090315, hsa_circ_0090316, hsa_circ_0090317, hsa_circ_0090318, hsa_circ_0090319, hsa_circ_0090320, and hsa_circ_0090321, were all derived from the host genes MAOA (Figure 4(b) and Supplementary Table 3). Moreover, 5 upregulated DECs, hsa_circ_0081056, hsa_circ_0081065, hsa_circ_0081091, hsa_circ_0081167, and hsa_circ_0081108, were all derived from COL1A2, a key component of ECM (Figure 4(b) and Supplementary Table 3).  Figure 4(c) shown the expression of key DEGs in CEPD group compared with the normal group. For example, GDF5, fibroblast growth factor (FGF18), COL13A1, and SDC1 were upregulated, whereas S100A8, S100A9, MMP8, and MAOA were downregulated in CEPD group.

### 3.5. Functional Enrichment Analysis of the Host Genes of DECs

circRNAs are derived from precursor (pre)-mRNAs and formed by back-splicing, namely, a downstream 5′ splice site is covalently binded with an upstream 3′ splice site [32]. Moreover, the abundance of circRNAs are negatively correlated with their linear host gene mRNAs because there is a competition between circRNA biogenesis and pre-mRNA splicing [33]. Thus, whether the host genes of DECs are involved in the regulation of IDD is a meaningful issue worth studying. First, the host genes of DECs were displayed in Supplementary Table 3. Subsequently, GO and KEGG pathway enrichment analyses were conducted to predict the potential biological functions of their host genes. GO function annotations included terms in the biological process (BP), molecular function (MF), and cellular component (CC) categories. The bubble diagram shown a total of 15, 12, and 11 important GO terms were significantly enriched in BP, CC, and MF, respectively (Figures 5(a)–5(c)). The BP aspect encompassed cell adhesion, extracellular matrix organization, collagen catabolic process, apoptotic process, neutrophil aggregation, chemokine production, regulation of cell growth, and leukocyte migration involved in inflammatory response (Figure 5(a)). The CC were found to be mostly enriched in the following terms: extracellular matrix, proteinaceous extracellular matrix, extracellular exosome, extracellular region, and collagen trimer (Figure 5(b)), whereas the most enriched in MF were extracellular matrix structural constituent, RAGE receptor binding, Toll-like receptor 4 binding, collagen binding, insulin-like growth factor binding, and metalloendopeptidase activity (Figure 5(c)). The results of KEGG pathway analysis uncovered the host genes of DECs were predominantly associated with 5 different signaling pathways, such as ECM-receptor interaction, focal adhesion, PI3K-Akt signaling pathway, protein digestion and absorption, amoebiasis, and platelet activation (Figure 5(d)). Taken together, these results suggested that the host genes of DECs possibly regulate ECM metabolism, chondrocytes apoptosis, and chondrocytes growth.

## 4. Discussion

The pathomechanism of CEPD is the results of multifactorial dysregulation, of which chondrocytes apoptosis, CEP calcification, and inflammatory response are the main factors [8, 9, 11]. Increasing evidence indicated that CEPD is the predominant cause of triggering IDD [34–37]. Recently, experimental research displayed that CEPD significantly aggravated IDD progression by promoting inflammatory cytokine expression or apoptosis in NP cells [34, 36]. Luo et al. [36] also observed that the recurrence rate of patients is remarkably higher with IDD with CEP inflammation after surgery. Liu et al. [37] validated that CEP matrix stiffness can promote CEP calcification which had positively correlated with the degree of IDD. Berg-Johansen et al. [35] measured CEP thickness through ultrashort echo-time MRI and reported that CEP thickness variation is associated with IDD. In this study, we displayed the landscape of RNAs in human cervical CEPD and performed an analysis of circRNA, miRNA, and gene expression profiles to identify new RNAs that may exert crucial role in modulating CEPD.

The dysregulation of RNA expression levels is a hallmark pathological characteristic of CEPD. Abundant evidence has uncovered the DEMs exert protective or destructive role in the occurrence and progression of CEPD. As previously verified by Chen and colleagues [38], miR-34a enhanced CEP chondrocyte apoptosis by directly repressing Bcl-2. miR-20a overexpression was demonstrated to enhance CEP calcification by targeting ankylosis progressive homolog [35]. These data suggest that miR-34a and miR-20a positively regulate CEPD. Among the 33 DEMs, several of them had been demonstrated to play a pivotal role in IDD. MiR-675, predicted to be the most upregulated miRNA in our study, was demonstrated to inhibit human chondrocytes apoptosis and ECM degradation [39], implying that its might protect against CEPD. Niu et al. [40] found hyperbaric oxygen treatment can elevate miR-107 to inhibit MMP3/9/13 expression in degenerated NP cells. Yu et al. [41] verified miR-10b promote NP cell proliferation by targeting the HOXD10-RhoC-Akt signaling pathway, and its levels in IDD were correlated with IDD grade. As reported by Wang et al. [42] miR-223 overexpression could repress NF-*κ*B signaling and inflammation response in NP cells by repressinging IRAK1. Additionally, miR-431, a direct downsteam target of circSEMA4B, could regulate NP cells proliferation, senescence, and ECM degradation via mediating Wnt signaling pathway [43]. Given that the pathogenesis of CEPD is similar to IDD, we guess that these DEMs or other miRNAs whose functions have not been identified might exert important role in mediating CEPD.

To the best of our knowledge, the potential role and mechanism of circRNAs in CEPD have not been identified; our current research was the first to identify the circRNA expression profile in CEPD. Zheng et al. [44] found *Hipk3* gene could produce multiple circRNA isoforms, whereas circHIPK3 is the predominant circRNA isoform and could regulate cell growth. In this study, we found *Sdc1*, *Maoa*, and *Col2a1* gene could produce 6, 8, and 5 DECs, respectively. There was an important feature that *Sdc1* and *Col2a1* gene-produced DECs are all upregulated, while the DECs derived from *Maoa* are all downregulated in CEPD. Furthermore, hsa_circ_0052830 derived from *Sdc1* is predicted to be the most upregulated DEC in CEPD. Regarding the multiple DECs derived from the same host gene, it is interesting to know which DEC is the most important isoform, or what is the difference between the functions of these DECs, and what role each DEC plays, which need future experiments to elucidate.

This study also identified a series of DEGs that exert crucial roles in regulating CEPD and IDD. LTF was observed to suppress IL-1*β*-induced apoptosis by activating cAMP responsive element-binding protein 1 in human chondrocytes [45]. FGF18 acts as a chondrocyte protective factor and can stimulate chondrogenesis and cartilage repair, which is regulated by circPDE4D-miR-103a-3p pathway [46, 47]. IL18RAP encodes a proinflammatory cytokine interleukin-18 (IL18) protein; IL18 can activate macrophages to secrete TNF-*α* and IL-1*β*, which further increased ECM degradation via upregulating MMPs during IDD [48]. Dolor et al. [49] validated that MMP8 treatment can enhance the transport properties of the human CEP to improve intervertebral disc nutrition. Zhu et al. [50] demonstrated that GDF5 can attenuate IDD in a rat model via promoting ECM synthesis.

Interestingly, SDC1 and MAOA were not only DEGs but also host genes of DECs. Literature reported SDC1 can repress inflammatory cytokine expression in epithelial [51] and epithelial apoptosis [52]. MAOA was also reported to promote tumor cell proliferation [53]. Furthermore, functional enrichment analysis of DEGs and host genes of DECs unveiled there are overlapping terms, such as inflammatory response, regulation of cell proliferation, collagen catabolic process, extracellular matrix, extracellular region, Toll-like receptor 4 binding, and RAGE receptor binding. The above phenomenon suggested that certain DECs might regulate their host gene expression, splicing, or transcription to mediate CEP chondrocytes apoptosis and proliferation as well as ECM metabolism and inflammatory response. Additionally, there is a DECs-DEMs-DEGs ceRNA regulatory network in CEPD, which needs further investigation.

However, several limitations existed in this study. Firstly, we did not conduct RNA sequencing. Secondly, we did not collect normal and degenerated NP tissues for qRT-PCR verification. At last, more molecular experiments, including qRT-PCR, Western blot, luciferase reporter assays, and RNA immunoprecipitation, as well as animal experiments, such as in situ tissue immunohistochemical tests, will be conducted to elucidate the role of key CEPD-related DECs, DEMs, and DEGs in the future.

## 5. Conclusion

Collectively, we successfully identified CEPD-related DECs, DEMs, and DEGs as well as predicted their potential biofunctions by bioinformatics analysis of the RNA expression profile. These results provide evidence that DECs, DEMs, and DEGs might have an important role in regulating the pathological process of CEPD. In fact, this is the first paper studying the DECs in CEPD, which contributes to us have a new understanding of the pathogenesis of CEPD. In the future, these information might be useful for guiding further experimental studies and provide novel therapeutic targets for CEPD-related diseases.

## Figures and Tables

**Table 1:** Basic information of the GSE153761 dataset from GEO database.

Data source	Platform	Sample size (D/N)	RNA type	Criteria	First author	Year
GSE153761	GPL22120	3/3	CircRNA gene	∣log_2_ FC | >2, *p* value < 0.05	Yuan J	2020
GSE153761	GPL22120	3/3	miRNA	∣log_2_ FC | >1, *p* value < 0.05	Yuan J	2020

GEO: Gene Expression Omnibus; D: degeneration; N: normal; circRNA: circular RNA; miRNA: microRNA; FC: fold change.

## Data Availability

The datasets supporting the conclusions of this article are included within the article.

## Supplementary Materials

Supplementary 1 Supplementary Table 1 Total circRNAs, miRNAs, and genes were identified in GSE153761 dataset.

Supplementary 2 Supplementary Table 2 The DECs, DEMs, and DEGs were identified in GSE153761 dataset.

Supplementary 3 Supplementary Table 3 The host genes of DECs.

## References

[1] Risbud M. V., Shapiro I. M. (2014). Role of cytokines in intervertebral disc degeneration: pain and disc content. *Nature Reviews Rheumatology*.

[2] Foster N. E., Anema J. R., Cherkin D. (2018). Prevention and treatment of low back pain: evidence, challenges, and promising directions. *Lancet*.

[3] Dieleman J. L., Cao J., Chapin A. (2020). US health care spending by payer and health condition, 1996-2016. *JAMA*.

[4] Bergknut N., Smolders L. A., Grinwis G. C. (2013). Intervertebral disc degeneration in the dog. Part 1: anatomy and physiology of the intervertebral disc and characteristics of intervertebral disc degeneration. *The Veterinary Journal*.

[5] Humzah M. D., Soames R. W. (1988). Human intervertebral disc: structure and function. *The Anatomical Record*.

[6] Urban J. P., Smith S., Fairbank J. C. (2004). Nutrition of the intervertebral disc. *Spine (Phila Pa 1976)*.

[7] Zhu Q., Gao X., Levene H. B., Brown M. D., Gu W. (2016). Influences of nutrition supply and pathways on the degenerative patterns in human intervertebral disc. *Spine (Phila Pa 1976)*.

[8] Li Y., Liu S., Pan D. (2021). The potential role and trend of HIF-1*α* in intervertebral disc degeneration: friend or foe? (review). *Molecular Medicine Reports*.

[9] Xie L., Chen Z., Liu M. (2020). MSC-derived exosomes protect vertebral endplate chondrocytes against apoptosis and calcification via the miR-31-5p/ATF6 Axis. *Molecular Therapy--Nucleic Acids*.

[10] Ma K., Chen S., Li Z. (2019). Mechanisms of endogenous repair failure during intervertebral disc degeneration. *Osteoarthritis and Cartilage*.

[11] Zhang J. F., Wang G. L., Zhou Z. J., Fang X. Q., Chen S., Fan S. W. (2018). Expression of matrix metalloproteinases, tissue inhibitors of metalloproteinases, and interleukins in vertebral cartilage endplate. *Orthopaedic Surgery*.

[12] Li Y., Zhou S., Peng P. (2020). Emerging role of circular RNA in intervertebral disc degeneration: knowns and unknowns (review). *Molecular Medicine Reports*.

[13] Li Y., Pan D., Liu S. (2021). Identification of circ-FAM169A sponges miR-583 involved in the regulation of intervertebral disc degeneration. *J Orthop Translat*.

[14] Chen Z., Zhang W., Deng M., Li Y., Zhou Y. (2020). CircGLCE alleviates intervertebral disc degeneration by regulating apoptosis and matrix degradation through the targeting of miR-587/STAP1. *Aging (Albany NY)*.

[15] Song J., Chen Z. H., Zheng C. J. (2020). Exosome-transported circRNA_0000253 competitively adsorbs microRNA-141-5p and increases IDD. *Molecular Therapy--Nucleic Acids*.

[16] Cheng X., Zhang L., Zhang K. (2018). Circular RNA VMA21 protects against intervertebral disc degeneration through targeting miR-200c and X linked inhibitor-of-apoptosis protein. *Annals of the Rheumatic Diseases*.

[17] Kou J., Liu G., Liu X. (2020). Profiling and bioinformatics analysis of differentially expressed circRNAs in spinal ligament tissues of patients with ankylosing spondylitis. *BioMed Research International*.

[18] Liu D., Liang Y. H., Yang Y. T. (2021). Circular RNA in osteoarthritis: an updated insight into the pathophysiology and therapeutics. *American Journal of Translational Research*.

[19] Yang Y., Shen P., Yao T. (2021). Novel role of circRSU1 in the progression of osteoarthritis by adjusting oxidative stress. *Theranostics*.

[20] Zhi L., Liang J., Huang W., Ma J., Qing Z., Wang X. (2021). Circ_AFF2 facilitates proliferation and inflammatory response of fibroblast-like synoviocytes in rheumatoid arthritis via the miR-375/TAB2 axis. *Experimental and Molecular Pathology*.

[21] Lewis B. P., Burge C. B., Bartel D. P. (2005). Conserved seed pairing, often flanked by adenosines, indicates that thousands of human genes are microRNA targets. *Cell*.

[22] Clough E., Barrett T. (2016). The gene expression omnibus database. *Methods in Molecular Biology*.

[23] Ritchie M. E., Phipson B., Wu D. (2015). limma powers differential expression analyses for RNA-sequencing and microarray studies. *Nucleic Acids Research*.

[24] Huang D. W., Sherman B. T., Lempicki R. A. (2009). Systematic and integrative analysis of large gene lists using DAVID bioinformatics resources. *Nature Protocols*.

[25] Szklarczyk D., Gable A. L., Lyon D. (2019). STRING v11: protein-protein association networks with increased coverage, supporting functional discovery in genome-wide experimental datasets. *Nucleic Acids Research*.

[26] Otasek D., Morris J. H., Bouças J., Pico A. R., Demchak B. (2019). Cytoscape automation: empowering workflow-based network analysis. *Genome Biology*.

[27] Chin C. H., Chen S. H., Wu H. H., Ho C. W., Ko M. T., Lin C. Y. (2014). cytoHubba: identifying hub objects and sub-networks from complex interactome. *BMC Systems Biology*.

[28] Ye F., Gao G., Zou Y. (2019). circFBXW7 inhibits malignant progression by sponging miR-197-3p and encoding a 185-aa protein in triple-negative breast cancer. *Molecular Therapy--Nucleic Acids*.

[29] Bai N., Peng E., Qiu X. (2018). circFBLIM1 act as a ceRNA to promote hepatocellular cancer progression by sponging miR-346. *Journal of Experimental & Clinical Cancer Research*.

[30] Conn V. M., Hugouvieux V., Nayak A. (2017). A circRNA from _SEPALLATA3_ regulates splicing of its cognate mRNA through R-loop formation. *Nat Plants*.

[31] Li Z., Huang C., Bao C. (2015). Exon-intron circular RNAs regulate transcription in the nucleus. *Nature Structural & Molecular Biology*.

[32] Kristensen L. S., Andersen M. S., Stagsted L. V. W., Ebbesen K. K., Hansen T. B., Kjems J. (2019). The biogenesis, biology and characterization of circular RNAs. *Nature Reviews. Genetics*.

[33] Ashwal-Fluss R., Meyer M., Pamudurti N. R. (2014). circRNA biogenesis competes with pre-mRNA splicing. *Molecular Cell*.

[34] Luo L., Jian X., Sun H. (2021). Cartilage endplate stem cells inhibit intervertebral disc degeneration by releasing exosomes to nucleus pulposus cells to activate Akt/autophagy. *Stem Cells*.

[35] Qiu X., Zhuang M., Lu Z. (2019). RIPK1 suppresses apoptosis mediated by TNF and caspase-3 in intervertebral discs. *Journal of Translational Medicine*.

[36] Liu M. H., Sun C., Yao Y. (2016). Matrix stiffness promotes cartilage endplate chondrocyte calcification in disc degeneration via miR-20a targeting ANKH expression. *Scientific Reports*.

[37] Berg-Johansen B., Han M., Fields A. J. (2018). Cartilage endplate thickness variation measured by ultrashort echo-time MRI is associated with adjacent disc degeneration. *Spine (Phila Pa 1976)*.

[38] Chen H., Wang J., Hu B. (2015). MiR-34a promotes Fas-mediated cartilage endplate chondrocyte apoptosis by targeting Bcl-2. *Molecular and Cellular Biochemistry*.

[39] Shen X. F., Cheng Y., Dong Q. R., Zheng M. Q. (2020). MicroRNA-675-3p regulates IL-1*β*-stimulated human chondrocyte apoptosis and cartilage degradation by targeting GNG5. *Biochemical and Biophysical Research Communications*.

[40] Niu C. C., Lin S. S., Yuan L. J. (2019). Upregulation of miR-107 expression following hyperbaric oxygen treatment suppresses HMGB1/RAGE signaling in degenerated human nucleus pulposus cells. *Arthritis Research & Therapy*.

[41] Yu X., Li Z., Shen J. (2013). MicroRNA-10b promotes nucleus pulposus cell proliferation through RhoC-Akt pathway by targeting HOXD10 in intervetebral disc degeneration. *PLoS One*.

[42] Wang H., Hao P., Zhang H., Xu C., Zhao J. (2018). MicroRNA-223 inhibits lipopolysaccharide-induced inflammatory response by directly targeting Irak1 in the nucleus pulposus cells of intervertebral disc. *IUBMB Life*.

[43] Wang X., Wang B., Zou M. (2018). CircSEMA4B targets miR-431 modulating IL-1*β*-induced degradative changes in nucleus pulposus cells in intervertebral disc degeneration via Wnt pathway. *Biochimica et Biophysica Acta - Molecular Basis of Disease*.

[44] Zheng Q., Bao C., Guo W. (2016). Circular RNA profiling reveals an abundant circHIPK3 that regulates cell growth by sponging multiple miRNAs. *Nature Communications*.

[45] Xue H., Tu Y., Ma T. (2015). Lactoferrin inhibits IL-1*β*-induced chondrocyte apoptosis through AKT1-induced CREB1 activation. *Cellular Physiology and Biochemistry*.

[46] Wu Y., Hong Z., Xu W. (2021). Circular RNA circPDE4D protects against osteoarthritis by binding to miR-103a-3p and regulating FGF18. *Molecular Therapy*.

[47] Moore E. E., Bendele A. M., Thompson D. L. (2005). Fibroblast growth factor-18 stimulates chondrogenesis and cartilage repair in a rat model of injury-induced osteoarthritis. *Osteoarthritis and Cartilage*.

[48] Dolor A., Sampson S. L., Lazar A. A., Lotz J. C., Szoka F. C., Fields A. J. (2019). Matrix modification for enhancing the transport properties of the human cartilage endplate to improve disc nutrition. *PLoS One*.

[49] Zhu J., Xia K., Yu W. (2019). Sustained release of GDF5 from a designed coacervate attenuates disc degeneration in a rat model. *Acta Biomaterialia*.

[50] Voyvodic P. L., Min D., Liu R. (2014). Loss of syndecan-1 induces a pro-inflammatory phenotype in endothelial cells with a dysregulated response to atheroprotective flow. *The Journal of Biological Chemistry*.

[51] Brauer R., Ge L., Schlesinger S. Y. (2016). Syndecan-1 attenuates lung injury during influenza infection by potentiating c-met signaling to suppress epithelial apoptosis. *American Journal of Respiratory and Critical Care Medicine*.

[52] Li J., Pu T., Yin L., Li Q., Liao C. P., Wu B. J. (2020). MAOA-mediated reprogramming of stromal fibroblasts promotes prostate tumorigenesis and cancer stemness. *Oncogene*.

