# Supplementary material for: Identification of Differentially Expressed circRNAs, miRNAs, and Genes in Patients Associated with Cartilaginous Endplate Degeneration
Source: Biomed Res Int. 2021 May 18;2021:2545459. doi: 10.1155/2021/2545459 (PMC8158415; doi:10.1155/2021/2545459)
Supplement: Supplementary 2 — Supplementary Table 2 The DECs, DEMs, and DEGs were identified in GSE153761 dataset. [file 2545459.f2.pdf]

DECs	logFC	P.Value
hsa_circ_(	3.0112461	0.0021577
hsa_circ_(	2.9322695	0.0101198
hsa_circ_(	2.9320618	0.0014888
hsa_circ_(	2.8542286	0.0037945
hsa_circ_(	2.8421766	0.0110668
hsa_circ_(	2.8323702	0.0074383
hsa_circ_(	2.7626934	0.0245654
hsa_circ_(	2.7375548	0.000301
hsa_circ_(	2.546448	0.0141502
hsa_circ_(	2.5247796	0.0074788
hsa_circ_(	2.5019093	0.0196445
hsa_circ_(	2.4988487	0.0093718
hsa_circ_(	2.4929199	0.0021279
hsa_circ_(	2.4772553	0.0017355
hsa_circ_(	2.455193	0.0228561
hsa_circ_(	2.448742	0.0085814
hsa_circ_(	2.4441484	0.0223211
hsa_circ_(	2.4319246	0.0184036
hsa_circ_(	2.4238805	0.0070902
hsa_circ_(	2.3750901	0.0101019
hsa_circ_(	2.3310092	0.000592
hsa_circ_(	2.319166	0.0110524
hsa_circ_(	2.2997468	8.10E-05
hsa_circ_(	2.299345	0.0277691
hsa_circ_(	2.2847809	0.0031945
hsa_circ_(	2.2810977	0.0047685
hsa_circ_(	2.2793838	0.0126079
hsa_circ_(	2.2515075	0.0061124
hsa_circ_(	2.2430183	0.0001503
hsa_circ_(	2.2319103	0.0008881
hsa_circ_(	2.2172669	0.0020797
hsa_circ_(	2.213023	0.001087
hsa_circ_(	2.2080801	0.0025483
hsa_circ_(	2.2062012	0.0005576
hsa_circ_(	2.1870533	0.000507
hsa_circ_(	2.1826204	0.0014904
hsa_circ_(	2.1804249	0.0012267
hsa_circ_(	2.1746017	0.0013841
hsa_circ_(	2.1733995	0.011816
hsa_circ_(	2.1708167	8.79E-06
hsa_circ_(	2.1707345	0.0028434
hsa_circ_(	2.1575691	0.0008484
hsa_circ_(	2.1554639	0.00067
hsa_circ_(	2.1502075	0.0085231
hsa_circ_(	2.1462412	0.0280209
hsa_circ_(	2.1413955	0.0018356

hsa_circ_(	2.1286627	0.0049049
hsa_circ_(	2.1155902	0.0019104
hsa_circ_(	2.103539	0.0022765
hsa_circ_(	2.1030627	0.014608
hsa_circ_(	2.0972165	3.13E-05
hsa_circ_(	2.0970978	0.0018759
hsa_circ_(	2.0940368	0.0152268
hsa_circ_(	2.0893204	0.0005467
hsa_circ_(	2.0773955	0.0134596
hsa_circ_(	2.0755325	0.0145145
hsa_circ_(	2.0505072	0.0222559
hsa_circ_(	2.0471347	0.0004748
hsa_circ_(	2.0450496	0.0047372
hsa_circ_(	2.0386032	0.0001816
hsa_circ_(	2.0356841	0.0075042
hsa_circ_(	2.0342428	4.77E-05
hsa_circ_(	2.0334149	0.0048146
hsa_circ_(	2.0306033	0.0001274
hsa_circ_(	2.0176071	0.0017403
hsa_circ_(	2.0158912	2.09E-05
hsa_circ_(	2.0156479	0.0102074
hsa_circ_(	2.014496	0.0048171
hsa_circ_(	2.0144585	0.0281394
hsa_circ_(	2.0052793	0.0092471
hsa_circ_(	2.0046892	0.0310569
hsa_circ_(	2.0031781	0.0147515
hsa_circ_(	2.002746	0.0078287
hsa_circ_(	2.0017462	0.0002749

DECs	logFC	P.Value
hsa_circ_0041063	-2.009610088	0.026183322
hsa_circ_0042061	-2.009899346	0.041814142
hsa_circ_0000262	-2.018145062	0.03644676
hsa_circ_0043606	-2.031428266	0.007523991
hsa_circ_0075839	-2.041175749	0.017654683
hsa_circ_0002456	-2.048504304	0.024889475
hsa_circ_0062226	-2.051943269	0.000422273
hsa_circ_0045418	-2.067212833	0.042569011
hsa_circ_0015383	-2.068338215	0.00109176
hsa_circ_0027510	-2.142966457	0.004221048
hsa_circ_0025135	-2.194550795	0.027919643
hsa_circ_0070174	-2.197022941	0.005210178
hsa_circ_0088733	-2.22344215	0.001089906
hsa_circ_0082796	-2.223727234	0.001430998
hsa_circ_0027511	-2.225560312	0.005470893
hsa_circ_0036498	-2.237857421	2.06E-05
hsa_circ_0029409	-2.249404787	0.00508612
hsa_circ_0073629	-2.279568152	0.002172502
hsa_circ_0091734	-2.295357301	0.005465298
hsa_circ_0022340	-2.309431758	0.009722134
hsa_circ_0079215	-2.321434544	0.00434551
hsa_circ_0088731	-2.33075338	0.000264584
hsa_circ_0090316	-2.333303782	0.047739853
hsa_circ_0020467	-2.342703781	0.029167287
hsa_circ_0090317	-2.362144119	0.006178614
hsa_circ_0090318	-2.365552297	0.020757404
hsa_circ_0014221	-2.366231272	0.010590796
hsa_circ_0045516	-2.368965531	0.024926804
hsa_circ_0031282	-2.391092034	0.003824483
hsa_circ_0050872	-2.394405235	0.039154551
hsa_circ_0014220	-2.485020545	0.004613074
hsa_circ_0014222	-2.54512011	0.003511746
hsa_circ_0090314	-2.54899377	0.018523654
hsa_circ_0070186	-2.586228303	0.000352518
hsa_circ_0090320	-2.595100007	0.011224203
hsa_circ_0015856	-2.597219563	0.017856892
hsa_circ_0022102	-2.623067448	0.003035594
hsa_circ_0090319	-2.685889987	0.009722743
hsa_circ_0069094	-2.686205595	0.011184252
hsa_circ_0070187	-2.695466675	0.000129729
hsa_circ_0081375	-2.759582184	0.019420585
hsa_circ_0090321	-2.816309376	0.019227685
hsa_circ_0088732	-2.845468917	0.000491314
hsa_circ_0003808	-2.917301681	0.015736114
hsa_circ_0090315	-3.1899664	0.027983329
hsa_circ_0075949	-3.233479938	9.82E-05

hsa_circ_0000577	-3.272610034	0.000272688
hsa_circ_0000576	-3.318806673	0.000258288
hsa_circ_0029995	-3.379995366	0.033594444
hsa_circ_0024595	-3.450884239	0.036652717
hsa_circ_0074821	2.09721653	3.13E-05
hsa_circ_0074776	2.097097778	0.001875902
hsa_circ_0000082	2.09403682	0.015226776
hsa_circ_0032363	2.08932038	0.000546708
hsa_circ_0028195	2.077395505	0.013459638
hsa_circ_0001234	2.075532501	0.014514461
hsa_circ_0077670	2.050507206	0.022255947
hsa_circ_0027700	2.0471347	0.000474772
hsa_circ_0074771	2.045049613	0.004737184
hsa_circ_0010407	2.038603187	0.000181643
hsa_circ_0020206	2.035684125	0.007504163
hsa_circ_0031927	2.03424282	4.77E-05
hsa_circ_0073242	2.033414935	0.004814588
hsa_circ_0002329	2.030603272	0.00012738
hsa_circ_0042445	2.017607126	0.001740307
hsa_circ_0089445	2.015891191	2.09E-05
hsa_circ_0089269	2.015647875	0.010207353
hsa_circ_0015069	2.014495994	0.004817069
hsa_circ_0060167	2.014458489	0.028139362
hsa_circ_0052834	2.005279253	0.009247055
hsa_circ_0001708	2.004689196	0.031056879
hsa_circ_0020788	2.003178133	0.014751473
hsa_circ_0066668	2.002745991	0.007828726
hsa_circ_0076614	2.001746241	0.000274906

MiRNAs	logFC	P.Value
MIR1202	1.0358214	0.007767091
MIR4253	1.0457501	0.000784534
MIR3194	1.0517678	0.013763622
MIR548I2	1.0643297	0.015275434
MIR5004	1.0736202	0.007977429
MIR4515	1.1269813	0.048599927
MIR6773	1.1755099	0.006942459
MIR8058	1.2017716	0.011300163
MIR4753	1.3066851	0.040283037
MIR1272	1.3439073	0.011599463
MIR3909	1.3972507	0.018754519
MIR7157	1.438892	0.001891271
MIR5093	1.5771862	0.000747085
MIR5739	1.7334128	0.04565587
MIR6843	1.7978172	0.048917716
MIR675	1.8371084	0.01718786
MIR1245A	2.0820138	0.000488588

CONTROL
CONTROL
CONTROL
CONTROL
CONTROL
CONTROL
CONTROL
CONTROL
CONTROL
CONTROL
CONTROL
CONTROL
CONTROL
CONTROL
CONTROL

CONTROL
CONTROL
CONTROL
CONTROL
CONTROL
CONTROL
CONTROL
CONTROL
CONTROL
CONTROL
CONTROL
CONTROL
CONTROL
CONTROL
CONTROL
CONTROL
CONTROL
CONTROL
CONTROL
CONTROL
CONTROL
CONTROL
CONTROL

MiRNAs	logFC	P.Value
MIR3660	-1.833726	0.0419792
MIR4740	-1.673483	0.0349963
MIR492	-1.611437	0.0094709
MIR4659B	-1.51848	0.0481186
MIR4524B	-1.441064	0.0274178
MIR3615	-1.322923	0.0148092
MIR4692	-1.304571	0.0114549
MIR4304	-1.193742	0.0409451
MIR4759	-1.165829	0.0147415
MIR6082	-1.150018	0.0089029
MIR19B1	-1.135637	0.0074442
MIR10B	-1.134829	0.0010732
MIR431	-1.111581	0.0111784
MIR4460	-1.042149	0.0249697
MIR223	-1.036135	0.0205919
MIR107	-1.030152	0.0114288

DEGs	logFC	P.Value
GALR1	2.007129	0.034731
AFF3	2.012458	0.014644
CRIP1	2.020168	0.01267
FNDC1	2.036316	0.003412
FGF18	2.06868	0.002242
C10orf105	2.069054	0.006848
TSPAN2	2.069507	0.018502
RASL12	2.085478	0.009394
CTHRC1	2.102234	0.000249
SCX	2.114418	0.03076
TNFAIP6	2.116339	0.033909
C4B_2	2.127701	0.005131
GSC	2.143845	0.000875
ZIC5	2.154683	0.000411
C4A	2.157368	0.003243
PIEZO2	2.160463	0.003038
ISM2	2.165033	0.001524
CPLX1	2.167074	0.000975
CLEC18A	2.186898	0.040414
SOX11	2.207394	0.013614
CALHM3	2.210152	0.000284
FHOD3	2.21079	0.0161
LRRC4B	2.212143	0.01639
C4B	2.227066	0.003861
FAP	2.228486	0.000193
CDHR4	2.229039	0.000625
PLEKHS1	2.231771	4.16E-05
BTBD16	2.238766	0.000113
RPA1	2.250446	0.000183
KITLG	2.267965	0.000597
GABRD	2.268903	0.009051
RIMS2	2.278716	0.036681
COL13A1	2.316527	0.021392
NOV	2.337401	0.012824
IMPG2	2.343098	0.009534
ADAMTS14	2.355761	0.010495
SVOP	2.411987	0.002175
C4BPA	2.414203	0.000134
HOXD3	2.421662	0.006477
IFITM5	2.424692	0.002727
ILDR2	2.435513	4.17E-05
ROR1	2.448575	0.008233
CHST13	2.451595	0.023224
LMO1	2.463083	0.001654
FAT3	2.467073	0.016515
SDC1	2.46912	0.008198

OLFML2B	2.486981	0.000423
DMP1	2.489372	0.012151
C8B	2.514905	0.032105
CBLN4	2.543834	0.003415
ISM1	2.566189	0.000593
MEOX1	2.62779	0.002354
GALNT16	2.679188	0.023162
GSG1L	2.697212	0.020894
PRSS35	2.831378	0.018665
GDF5	2.849716	0.002605
ADCY1	2.894792	0.008137
SAMD11	2.895394	0.048933
GPR20	2.961529	0.011052
TNFSF15	2.988296	0.020796
BIRC7	3.042691	0.005898
ASPN	3.043034	0.020632
PLA2G2A	3.154312	0.00312
GINS3	3.287875	0.002578
AMTN	3.41208	0.013722
LY6D	3.428054	0.027119
MXRA5	3.545868	0.014173
R3HDML	4.090977	0.006698

DEGs	logFC	P.Value
SCGB2A2	-6.03984	0.00012
SCGB1D2	-5.12768	0.035869
SCGB2A1	-4.34501	0.011335
DEFA4	-4.32121	0.004203
DEFA3	-4.31009	0.010157
SAA1	-4.21457	0.049247
RNASE3	-4.0262	8.42E-05
DEFA1	-4.01091	0.014403
SYT7	-3.99833	0.000289
HBD	-3.93811	0.001656
KLK11	-3.78016	0.000287
PRB2	-3.67285	0.018113
MMP8	-3.42569	3.64E-05
RNASE2	-3.36963	0.000241
CEACAM8	-3.36409	0.000517
PRB1	-3.32555	0.025576
MCEMP1	-3.28913	0.009689
S100A8	-3.17636	0.002611
MAOA	-3.09686	0.015942
CLC	-3.04814	0.004771
APOD	-2.9727	0.023012
HP	-2.94126	0.000603
CAMP	-2.92904	0.043596
PRH2	-2.91582	0.004736
CEBPE	-2.88063	8.57E-05
CCNA1	-2.86983	0.001031
CA1	-2.86764	0.002624
LTF	-2.83654	0.00541
ALOX5AP	-2.83055	0.034745
MS4A3	-2.82284	0.003918
S100P	-2.80534	0.012532
ABCA9	-2.8043	0.020043
S100A9	-2.79633	0.005957
AZGP1	-2.74932	0.015827
S100A12	-2.72929	0.013601
LCN2	-2.69741	0.00124
CYP2A6	-2.56574	0.049009
ANXA3	-2.52006	0.002581
RHAG	-2.51196	0.000378
JCHAIN	-2.50766	0.049074
TRH	-2.46723	0.000363
SCN4B	-2.46213	0.044511
XKRX	-2.45027	0.000817
HEMGN	-2.42396	0.002437
PADI4	-2.41951	0.019969
TPPP2	-2.41527	0.014849

ARG1	-2.39574	0.02274
ST8SIA1	-2.38006	0.006242
HBM	-2.36352	0.004886
ACMSD	-2.32255	0.045328
DUSP2	-2.31716	0.013748
AIM1L	-2.30672	0.015408
CYP2S1	-2.29805	0.00139
ST14	-2.23251	0.047873
FAM196A	-2.21998	0.024251
NSUN7	-2.2107	0.020086
FCGR1A	-2.1784	0.015163
LEP	-2.16939	0.03732
SAMSN1	-2.13844	0.044347
ARHGAP20	-2.07743	0.000523
CTSG	-2.06658	0.004801
ALOX15B	-2.0516	0.047825
IL18RAP	-2.0506	0.041584
HIF3A	-2.04844	0.018844
CST7	-2.04143	0.005081
IGFBP6	-2.03236	0.048458
OLFM4	-2.01052	0.001466
