# Supplementary material for: Identification of Differentially Expressed circRNAs, miRNAs, and Genes in Patients Associated with Cartilaginous Endplate Degeneration
Source: Biomed Res Int. 2021 May 18;2021:2545459. doi: 10.1155/2021/2545459 (PMC8158415; doi:10.1155/2021/2545459)
Supplement: Supplementary 3 — Supplementary Table 3 The host genes of DECs. [file 2545459.f3.pdf]

DECs	Host genes
hsa_circ_0052830	SDC1
hsa_circ_0034645	CHAC1
hsa_circ_0054651	EFEMP1
hsa_circ_0052829	SDC1
hsa_circ_0083941	GPR124
hsa_circ_0021747	PAMR1
hsa_circ_0041793	C17orf81
hsa_circ_0056886	FAP
hsa_circ_0020787	TCONS_00063837_H19
hsa_circ_0052833	SDC1
hsa_circ_0047460	FHOD3
hsa_circ_0052828	SDC1
hsa_circ_0056885	FAP
hsa_circ_0027699	KITLG
hsa_circ_0041796	C17orf81
hsa_circ_0033629	CRIP1
hsa_circ_0018671	ADAMTS14
hsa_circ_0037034	IGF1R
hsa_circ_0052832	SDC1
hsa_circ_0076855	DST
hsa_circ_0081167	COL1A2
hsa_circ_0012841	ROR1
hsa_circ_0011501	CSMD2
hsa_circ_0025106	VWF
hsa_circ_0081065	COL1A2
hsa_circ_0002803	KITLG
hsa_circ_0074899	SLIT3
hsa_circ_0056884	FAP
hsa_circ_0081056	COL1A2
hsa_circ_0023830	ODZ4
hsa_circ_0056883	FAP
hsa_circ_0020269	HTRA1
hsa_circ_0079022	LOC100293534
hsa_circ_0085289	CTHRC1
hsa_circ_0014111	TMOD4
hsa_circ_0020274	HTRA1
hsa_circ_0032359	SMOC1
hsa_circ_0073236	VCAN
hsa_circ_0024303	NCAM1
hsa_circ_0073580	EPB41L4A
hsa_circ_0078945	None
hsa_circ_0020271	HTRA1
hsa_circ_0081091	COL1A2
hsa_circ_0077682	LAMA4
hsa_circ_0079560	STEAP1B
hsa_circ_0024583	MCAM

hsa_circ_0047798	ALPK2
hsa_circ_0032784	ISM2
hsa_circ_0081108	COL1A2
hsa_circ_0057461	COL5A2
hsa_circ_0074821	EBF1
hsa_circ_0074776	CYFIP2
hsa_circ_0000082	ROR1
hsa_circ_0032363	SMOC1
hsa_circ_0028195	ANAPC7
hsa_circ_0001234	L3MBTL2
hsa_circ_0077670	LAMA4
hsa_circ_0027700	KITLG
hsa_circ_0074771	CYFIP2
hsa_circ_0010407	C1orf151-NBL1
hsa_circ_0020206	PPAPDC1A
hsa_circ_0031927	NID2
hsa_circ_0073242	VCAN
hsa_circ_0002329	KITLG
hsa_circ_0042445	TCONS_l2_00011537
hsa_circ_0089445	COL5A1
hsa_circ_0089269	SETX
hsa_circ_0015069	PBX1
hsa_circ_0060167	EPB41L1
hsa_circ_0052834	SDC1
hsa_circ_0001708	IKZF1
hsa_circ_0020788	TCONS_00063837_H19
hsa_circ_0066668	ABI3BP
hsa_circ_0076614	TMEM63B
hsa_circ_0041063	FANCA
hsa_circ_0042061	NTN1
hsa_circ_0000262	GRK5
hsa_circ_0043606	KRT19
hsa_circ_0075839	GPLD1
hsa_circ_0002456	DOCK1
hsa_circ_0062226	CLTCL1
hsa_circ_0045418	PITPNC1
hsa_circ_0015383	PAPPA2
hsa_circ_0027510	LYZ
hsa_circ_0025135	SCNN1A
hsa_circ_0070174	FRAS1
hsa_circ_0088733	LCN2
hsa_circ_0082796	MGAM
hsa_circ_0027511	LYZ
hsa_circ_0036498	CTSH
hsa_circ_0029409	GLT1D1
hsa_circ_0073629	KCNN2
hsa_circ_0091734	ZNF185

hsa_circ_0022340	SYT7
hsa_circ_0079215	SDK1
hsa_circ_0088731	LCN2
hsa_circ_0090316	MAOA
hsa_circ_0020467	DOCK1
hsa_circ_0090317	MAOA
hsa_circ_0090318	MAOA
hsa_circ_0014221	S100A9
hsa_circ_0045516	ABCA6
hsa_circ_0031282	SLC7A8
hsa_circ_0050872	MAP4K1
hsa_circ_0014220	S100A9
hsa_circ_0014222	S100A8
hsa_circ_0090314	MAOA
hsa_circ_0070186	ANXA3
hsa_circ_0090320	MAOA
hsa_circ_0015856	C1orf106
hsa_circ_0022102	PTPRJ
hsa_circ_0090319	MAOA
hsa_circ_0069094	S100P
hsa_circ_0070187	ANXA3
hsa_circ_0081375	AZGP1
hsa_circ_0090321	MAOA
hsa_circ_0088732	LCN2
hsa_circ_0003808	GFPT1
hsa_circ_0090315	MAOA
hsa_circ_0075949	None
hsa_circ_0000577	None
hsa_circ_0000576	None
hsa_circ_0029995	DCLK1
hsa_circ_0024595	THY1
