# Supplementary material for: Identification of Differentially Expressed circRNAs, miRNAs, and Genes in Patients Associated with Cartilaginous Endplate Degeneration
Source: Biomed Res Int. 2021 May 18;2021:2545459. doi: 10.1155/2021/2545459 (PMC8158415; doi:10.1155/2021/2545459)
Supplement: Supplementary 1 — Supplementary Table 1 Total circRNAs, miRNAs, and genes were identified in GSE153761 dataset. [file 2545459.f1.pdf]

CircRNAs	logFC	P.Value
hsa_circ_	2.170817	8.79E-06
hsa_circ_	-1.49355	1.53E-05
hsa_circ_	-1.68052	1.86E-05
hsa_circ_	-2.23786	2.06E-05
hsa_circ_	2.015891	2.09E-05
hsa_circ_	1.938425	2.74E-05
hsa_circ_	1.90079	2.95E-05
hsa_circ_	2.097217	3.13E-05
hsa_circ_	-1.49585	3.32E-05
hsa_circ_	1.582149	4.44E-05
hsa_circ_	1.906861	4.75E-05
hsa_circ_	2.034243	4.77E-05
hsa_circ_	1.357003	5.11E-05
hsa_circ_	1.21636	6.00E-05
hsa_circ_	-1.43064	6.32E-05
hsa_circ_	1.601038	6.75E-05
hsa_circ_	1.162142	6.91E-05
hsa_circ_	1.605477	6.94E-05
hsa_circ_	1.215104	7.16E-05
hsa_circ_	1.386639	7.31E-05
hsa_circ_	2.299747	8.10E-05
hsa_circ_	1.211893	8.18E-05
hsa_circ_	1.568603	8.28E-05
hsa_circ_	1.636367	8.33E-05
hsa_circ_	-1.40513	8.42E-05
hsa_circ_	1.234488	9.02E-05
hsa_circ_	1.065821	9.06E-05
hsa_circ_	1.240149	9.06E-05
hsa_circ_	-1.29415	9.49E-05
hsa_circ_	-1.26311	9.55E-05
hsa_circ_	-1.15254	9.75E-05
hsa_circ_	-3.23348	9.82E-05
hsa_circ_	1.738842	9.96E-05
hsa_circ_	1.055953	0.000106
hsa_circ_	1.32609	0.000106
hsa_circ_	1.57024	0.000108
hsa_circ_	1.398121	0.000108
hsa_circ_	1.573095	0.000108
hsa_circ_	-1.85248	0.000116
hsa_circ_	1.102967	0.000117
hsa_circ_	1.605459	0.000117
hsa_circ_	-1.51596	0.000119
hsa_circ_	1.657194	0.000119
hsa_circ_	1.130621	0.00012
hsa_circ_	1.023531	0.000123
hsa_circ_	2.030603	0.000127
hsa_circ_	1.585459	0.000129
hsa_circ_	-2.69547	0.00013
hsa_circ_	1.118902	0.000135
hsa_circ_	-1.27372	0.000139
hsa_circ_	-1.3841	0.000141
hsa_circ_	1.329936	0.000142
hsa_circ_	1.574534	0.000147

hsa_circ_	2.243018	0.00015
hsa_circ_	-1.35803	0.000151
hsa_circ_	1.865254	0.000154
hsa_circ_	1.513912	0.000155
hsa_circ_	-1.26125	0.000157
hsa_circ_	1.007843	0.000165
hsa_circ_	1.625767	0.000168
hsa_circ_	-1.08189	0.000169
hsa_circ_	1.579494	0.00017
hsa_circ_	1.939122	0.000176
hsa_circ_	0.994073	0.000177
hsa_circ_	2.038603	0.000182
hsa_circ_	-1.64932	0.000187
hsa_circ_	1.531999	0.000188
hsa_circ_	1.274973	0.000192
hsa_circ_	1.387413	0.000199
hsa_circ_	1.713192	0.000204
hsa_circ_	1.191518	0.000204
hsa_circ_	1.487774	0.000208
hsa_circ_	-1.22588	0.000213
hsa_circ_	1.694325	0.000215
hsa_circ_	-1.55199	0.000216
hsa_circ_	1.690836	0.000217
hsa_circ_	1.344548	0.000217
hsa_circ_	-1.00998	0.000218
hsa_circ_	-0.99867	0.000221
hsa_circ_	1.726793	0.000221
hsa_circ_	1.072936	0.000222
hsa_circ_	1.133128	0.000224
hsa_circ_	-0.94623	0.000227
hsa_circ_	-0.92258	0.000233
hsa_circ_	-1.20113	0.000242
hsa_circ_	1.350329	0.000244
hsa_circ_	1.735944	0.000245
hsa_circ_	1.441037	0.000248
hsa_circ_	1.290182	0.00025
hsa_circ_	1.168878	0.00025
hsa_circ_	1.313106	0.000252
hsa_circ_	1.588524	0.000252
hsa_circ_	1.216411	0.000253
hsa_circ_	1.563534	0.000256
hsa_circ_	-3.31881	0.000258
hsa_circ_	0.971932	0.000262
hsa_circ_	1.389245	0.000264
hsa_circ_	-2.33075	0.000265
hsa_circ_	1.542465	0.000272
hsa_circ_	-3.27261	0.000273
hsa_circ_	1.15376	0.000273
hsa_circ_	1.535336	0.000274
hsa_circ_	1.560069	0.000275
hsa_circ_	2.001746	0.000275
hsa_circ_	-0.98067	0.00028
hsa_circ_	1.114567	0.000284
hsa_circ_	-0.98958	0.000287

hsa_circ_	-1.11624	0.000288
hsa_circ_	-1.02869	0.000288
hsa_circ_	-1.11207	0.000299
hsa_circ_	1.542419	0.000301
hsa_circ_	2.737555	0.000301
hsa_circ_	-1.18602	0.000303
hsa_circ_	1.016573	0.000303
hsa_circ_	-1.71491	0.000304
hsa_circ_	1.237318	0.000305
hsa_circ_	-1.05984	0.000308
hsa_circ_	-1.55442	0.00031
hsa_circ_	1.782253	0.000313
hsa_circ_	-1.30078	0.000322
hsa_circ_	-1.98086	0.000329
hsa_circ_	1.36597	0.00033
hsa_circ_	1.047877	0.000333
hsa_circ_	1.011137	0.000338
hsa_circ_	-0.98851	0.000348
hsa_circ_	1.148038	0.00035
hsa_circ_	1.334441	0.000351
hsa_circ_	-1.17919	0.000351
hsa_circ_	-0.99478	0.000351
hsa_circ_	-2.58623	0.000353
hsa_circ_	1.07674	0.000357
hsa_circ_	1.419874	0.000359
hsa_circ_	1.001018	0.00036
hsa_circ_	1.482133	0.000362
hsa_circ_	1.093062	0.000362
hsa_circ_	1.009472	0.000364
hsa_circ_	1.365971	0.000366
hsa_circ_	1.175419	0.000367
hsa_circ_	-1.6544	0.000368
hsa_circ_	0.973606	0.00037
hsa_circ_	1.457797	0.000372
hsa_circ_	1.484061	0.000372
hsa_circ_	1.006883	0.000373
hsa_circ_	1.67136	0.000375
hsa_circ_	-1.37741	0.000375
hsa_circ_	1.767862	0.000379
hsa_circ_	1.264711	0.000379
hsa_circ_	1.446142	0.000379
hsa_circ_	1.757763	0.00038
hsa_circ_	-1.42556	0.000381
hsa_circ_	1.354834	0.000381
hsa_circ_	0.980784	0.000385
hsa_circ_	1.140974	0.000388
hsa_circ_	1.061522	0.000389
hsa_circ_	1.919506	0.000395
hsa_circ_	1.231913	0.000397
hsa_circ_	1.361513	0.000403
hsa_circ_	-1.06076	0.000404
hsa_circ_	1.711838	0.000408
hsa_circ_	1.527058	0.00041
hsa_circ_	-1.05183	0.000411

hsa_circ_	1.549813	0.000411
hsa_circ_	0.922541	0.000411
hsa_circ_	-1.68485	0.000421
hsa_circ_	-2.05194	0.000422
hsa_circ_	-0.96266	0.000424
hsa_circ_	-0.95265	0.000427
hsa_circ_	-0.95804	0.000433
hsa_circ_	1.562724	0.000434
hsa_circ_	-1.41162	0.000438
hsa_circ_	1.696881	0.000439
hsa_circ_	1.177875	0.00044
hsa_circ_	-1.58582	0.00044
hsa_circ_	-1.14586	0.000444
hsa_circ_	0.928802	0.000446
hsa_circ_	-1.32028	0.000448
hsa_circ_	1.29293	0.00045
hsa_circ_	1.359932	0.000452
hsa_circ_	0.859163	0.000452
hsa_circ_	-1.01959	0.000453
hsa_circ_	-0.88941	0.000457
hsa_circ_	1.340984	0.000466
hsa_circ_	1.080721	0.000468
hsa_circ_	-0.85192	0.000469
hsa_circ_	2.047135	0.000475
hsa_circ_	1.978203	0.000478
hsa_circ_	0.814289	0.00048
hsa_circ_	-0.82752	0.000481
hsa_circ_	1.887344	0.000482
hsa_circ_	-0.91149	0.000482
hsa_circ_	1.097816	0.000484
hsa_circ_	1.304606	0.000487
hsa_circ_	0.842513	0.000488
hsa_circ_	-2.84547	0.000491
hsa_circ_	1.51148	0.000499
hsa_circ_	0.851893	0.000501
hsa_circ_	1.419059	0.000502
hsa_circ_	1.205065	0.000502
hsa_circ_	2.187053	0.000507
hsa_circ_	-1.19281	0.000507
hsa_circ_	1.044505	0.000508
hsa_circ_	1.529867	0.000509
hsa_circ_	1.128694	0.00051
hsa_circ_	1.476846	0.000514
hsa_circ_	1.185587	0.000521
hsa_circ_	1.239679	0.000524
hsa_circ_	1.26087	0.000529
hsa_circ_	1.377038	0.00053
hsa_circ_	-1.78544	0.000533
hsa_circ_	1.052529	0.000534
hsa_circ_	0.828172	0.000535
hsa_circ_	0.816648	0.000539
hsa_circ_	1.287284	0.00054
hsa_circ_	2.08932	0.000547
hsa_circ_	-1.16639	0.000548

hsa_circ_	-1.35938	0.000549
hsa_circ_	1.083522	0.000552
hsa_circ_	1.214829	0.000554
hsa_circ_	0.868274	0.000557
hsa_circ_	2.206201	0.000558
hsa_circ_	1.758071	0.000561
hsa_circ_	-1.13422	0.000564
hsa_circ_	0.805887	0.000565
hsa_circ_	1.442258	0.000567
hsa_circ_	1.734785	0.000568
hsa_circ_	-1.17597	0.00057
hsa_circ_	1.565103	0.000572
hsa_circ_	1.465162	0.000573
hsa_circ_	1.728111	0.000573
hsa_circ_	0.780281	0.000576
hsa_circ_	1.132391	0.000576
hsa_circ_	0.879019	0.000578
hsa_circ_	1.768596	0.00058
hsa_circ_	1.010835	0.00058
hsa_circ_	1.230315	0.000581
hsa_circ_	-0.92521	0.000589
hsa_circ_	1.306282	0.000591
hsa_circ_	-1.73015	0.000592
hsa_circ_	2.331009	0.000592
hsa_circ_	0.900182	0.000594
hsa_circ_	-1.64521	0.000614
hsa_circ_	0.801751	0.000615
hsa_circ_	1.146667	0.000617
hsa_circ_	1.431659	0.000617
hsa_circ_	1.054645	0.000621
hsa_circ_	0.751162	0.000621
hsa_circ_	0.85667	0.000622
hsa_circ_	-1.5561	0.000623
hsa_circ_	1.278803	0.000625
hsa_circ_	-1.66598	0.000627
hsa_circ_	-1.02453	0.000629
hsa_circ_	-0.8639	0.000632
hsa_circ_	-1.47777	0.000632
hsa_circ_	-1.09091	0.000634
hsa_circ_	1.949248	0.000634
hsa_circ_	1.336198	0.000636
hsa_circ_	-1.21774	0.000637
hsa_circ_	1.643658	0.000643
hsa_circ_	-0.87574	0.000644
hsa_circ_	-0.76446	0.000644
hsa_circ_	-1.33353	0.000646
hsa_circ_	1.761689	0.000653
hsa_circ_	-0.97863	0.000658
hsa_circ_	-1.94424	0.000658
hsa_circ_	1.234384	0.000661
hsa_circ_	-1.59519	0.000662
hsa_circ_	1.012578	0.000664
hsa_circ_	1.418032	0.000665
hsa_circ_	2.155464	0.00067

hsa_circ_	0.834675	0.000677
hsa_circ_	-1.70404	0.000679
hsa_circ_	-1.11166	0.00068
hsa_circ_	1.34264	0.000682
hsa_circ_	1.031807	0.00069
hsa_circ_	1.543917	0.000695
hsa_circ_	-1.65759	0.000696
hsa_circ_	1.035009	0.000696
hsa_circ_	-1.60367	0.000703
hsa_circ_	1.135169	0.000704
hsa_circ_	1.086667	0.000704
hsa_circ_	0.876166	0.000704
hsa_circ_	1.476429	0.000705
hsa_circ_	1.318106	0.00071
hsa_circ_	1.329815	0.000713
hsa_circ_	1.428918	0.000714
hsa_circ_	1.296025	0.000716
hsa_circ_	1.685686	0.000716
hsa_circ_	1.594131	0.000717
hsa_circ_	1.327107	0.000717
hsa_circ_	-0.82687	0.000721
hsa_circ_	0.891546	0.000723
hsa_circ_	1.107501	0.000723
hsa_circ_	1.110288	0.000723
hsa_circ_	-1.05775	0.000731
hsa_circ_	-0.88768	0.000732
hsa_circ_	0.971185	0.000732
hsa_circ_	0.989726	0.000733
hsa_circ_	-0.83155	0.000734
hsa_circ_	-1.73059	0.000737
hsa_circ_	1.633887	0.000739
hsa_circ_	1.370147	0.00074
hsa_circ_	1.030079	0.000744
hsa_circ_	0.865778	0.000746
hsa_circ_	-0.88336	0.00075
hsa_circ_	1.250596	0.000753
hsa_circ_	-0.82338	0.000756
hsa_circ_	1.249577	0.000767
hsa_circ_	1.045863	0.00077
hsa_circ_	1.1339	0.00077
hsa_circ_	0.888386	0.000784
hsa_circ_	1.009138	0.000786
hsa_circ_	-1.5268	0.000788
hsa_circ_	0.863014	0.000788
hsa_circ_	-1.61688	0.000789
hsa_circ_	1.363257	0.00079
hsa_circ_	1.793773	0.000791
hsa_circ_	1.489671	0.000793
hsa_circ_	-0.94937	0.000797
hsa_circ_	1.125071	0.0008
hsa_circ_	-1.1181	0.000803
hsa_circ_	1.817214	0.000804
hsa_circ_	-1.53221	0.000806
hsa_circ_	1.437589	0.00081

hsa_circ_	-1.50867	0.00081
hsa_circ_	1.145037	0.000812
hsa_circ_	-1.04365	0.000815
hsa_circ_	1.204297	0.000817
hsa_circ_	-1.11347	0.000818
hsa_circ_	0.946562	0.000821
hsa_circ_	0.805721	0.000824
hsa_circ_	0.847266	0.000828
hsa_circ_	-0.95339	0.000829
hsa_circ_	1.588222	0.000829
hsa_circ_	-0.82283	0.000833
hsa_circ_	1.352448	0.000833
hsa_circ_	1.01595	0.000836
hsa_circ_	1.417644	0.000837
hsa_circ_	-0.79391	0.000837
hsa_circ_	-0.89055	0.000837
hsa_circ_	0.942999	0.000843
hsa_circ_	2.157569	0.000848
hsa_circ_	1.437837	0.000851
hsa_circ_	-1.70829	0.000854
hsa_circ_	1.284463	0.000856
hsa_circ_	-1.23617	0.000857
hsa_circ_	1.784519	0.000861
hsa_circ_	1.516615	0.000877
hsa_circ_	-1.07165	0.000877
hsa_circ_	0.961986	0.000879
hsa_circ_	1.103487	0.000884
hsa_circ_	1.12566	0.000885
hsa_circ_	1.396624	0.000885
hsa_circ_	1.505334	0.000885
hsa_circ_	-1.22099	0.000886
hsa_circ_	2.23191	0.000888
hsa_circ_	1.501625	0.000891
hsa_circ_	-0.77011	0.000892
hsa_circ_	1.03829	0.000892
hsa_circ_	1.404459	0.000893
hsa_circ_	-1.14155	0.000894
hsa_circ_	1.052261	0.000894
hsa_circ_	-1.32117	0.000896
hsa_circ_	1.497543	0.000897
hsa_circ_	-0.76419	0.0009
hsa_circ_	1.247845	0.000902
hsa_circ_	1.325101	0.000905
hsa_circ_	1.475551	0.000906
hsa_circ_	1.115724	0.000907
hsa_circ_	0.75118	0.000908
hsa_circ_	-0.77839	0.000909
hsa_circ_	-0.97945	0.000915
hsa_circ_	0.949479	0.000915
hsa_circ_	1.372091	0.000925
hsa_circ_	1.129466	0.000939
hsa_circ_	-0.85861	0.00094
hsa_circ_	1.891106	0.000949
hsa_circ_	-1.20174	0.000952

hsa_circ_	1.102443	0.000958
hsa_circ_	1.512721	0.000959
hsa_circ_	1.167261	0.000964
hsa_circ_	0.789332	0.000966
hsa_circ_	-0.71229	0.000967
hsa_circ_	-1.54524	0.000967
hsa_circ_	-1.2998	0.000973
hsa_circ_	-1.26528	0.000976
hsa_circ_	-0.81908	0.000977
hsa_circ_	1.869296	0.000978
hsa_circ_	-1.18685	0.000979
hsa_circ_	1.401499	0.000979
hsa_circ_	-0.94948	0.000983
hsa_circ_	-0.79142	0.00099
hsa_circ_	1.919328	0.00099
hsa_circ_	-0.80586	0.000991
hsa_circ_	-0.93063	0.000991
hsa_circ_	-1.53662	0.000996
hsa_circ_	0.777375	0.000999
hsa_circ_	1.173235	0.000999
hsa_circ_	0.938744	0.001
hsa_circ_	1.549011	0.001003
hsa_circ_	-0.70436	0.001006
hsa_circ_	0.938608	0.001007
hsa_circ_	-0.89379	0.001008
hsa_circ_	-1.12387	0.001009
hsa_circ_	-0.87096	0.00101
hsa_circ_	0.913423	0.001011
hsa_circ_	1.505775	0.001013
hsa_circ_	-0.75359	0.001026
hsa_circ_	1.615565	0.001031
hsa_circ_	1.342031	0.001037
hsa_circ_	-1.70908	0.001038
hsa_circ_	1.494938	0.001038
hsa_circ_	1.11959	0.00104
hsa_circ_	-1.51584	0.001043
hsa_circ_	-1.06531	0.001051
hsa_circ_	-0.83685	0.001058
hsa_circ_	1.333349	0.001064
hsa_circ_	1.387655	0.001066
hsa_circ_	0.932802	0.001067
hsa_circ_	-1.67236	0.00107
hsa_circ_	-1.67743	0.001072
hsa_circ_	-1.4621	0.001077
hsa_circ_	1.296649	0.001082
hsa_circ_	1.102982	0.001084
hsa_circ_	0.884802	0.001085
hsa_circ_	1.187428	0.001086
hsa_circ_	2.213023	0.001087
hsa_circ_	1.315725	0.001089
hsa_circ_	0.854756	0.001089
hsa_circ_	-2.22344	0.00109
hsa_circ_	-2.06834	0.001092
hsa_circ_	0.724622	0.001093

hsa_circ_	1.234453	0.001093
hsa_circ_	0.78047	0.001094
hsa_circ_	-1.44542	0.001108
hsa_circ_	0.750153	0.001113
hsa_circ_	1.370059	0.001115
hsa_circ_	1.316932	0.001127
hsa_circ_	1.370151	0.001128
hsa_circ_	1.782937	0.001129
hsa_circ_	-0.79569	0.001131
hsa_circ_	1.216175	0.001132
hsa_circ_	1.304383	0.001137
hsa_circ_	1.197151	0.001138
hsa_circ_	-0.94921	0.001142
hsa_circ_	1.100341	0.001146
hsa_circ_	-0.94575	0.001157
hsa_circ_	-1.85468	0.001164
hsa_circ_	1.208094	0.001167
hsa_circ_	0.850664	0.001172
hsa_circ_	1.731232	0.00118
hsa_circ_	1.729697	0.001182
hsa_circ_	-0.88061	0.001185
hsa_circ_	0.881348	0.001186
hsa_circ_	1.800083	0.001192
hsa_circ_	0.824237	0.001192
hsa_circ_	1.008195	0.001193
hsa_circ_	0.902216	0.001197
hsa_circ_	-1.2392	0.001201
hsa_circ_	1.03406	0.001207
hsa_circ_	1.060452	0.00121
hsa_circ_	-1.58404	0.001213
hsa_circ_	0.713991	0.001213
hsa_circ_	1.016422	0.001214
hsa_circ_	-1.08921	0.001214
hsa_circ_	0.904513	0.001217
hsa_circ_	1.560223	0.001219
hsa_circ_	2.180425	0.001227
hsa_circ_	0.820305	0.001228
hsa_circ_	-0.68272	0.001235
hsa_circ_	1.384207	0.001236
hsa_circ_	-1.12423	0.001239
hsa_circ_	-1.07063	0.001243
hsa_circ_	1.339522	0.00125
hsa_circ_	1.584427	0.00125
hsa_circ_	1.113949	0.001251
hsa_circ_	-0.74202	0.001252
hsa_circ_	0.762974	0.001253
hsa_circ_	-0.72144	0.001257
hsa_circ_	1.271861	0.001262
hsa_circ_	1.700316	0.001266
hsa_circ_	1.323038	0.001268
hsa_circ_	-0.81059	0.00127
hsa_circ_	0.771288	0.001271
hsa_circ_	0.750041	0.001271
hsa_circ_	-0.8128	0.001273

hsa_circ_	1.161728	0.001273
hsa_circ_	1.242141	0.001281
hsa_circ_	1.413778	0.001282
hsa_circ_	-0.80303	0.00129
hsa_circ_	0.725299	0.001296
hsa_circ_	-0.94934	0.001297
hsa_circ_	-1.36522	0.001297
hsa_circ_	-0.80651	0.001298
hsa_circ_	-0.78542	0.001298
hsa_circ_	-1.62915	0.001298
hsa_circ_	1.095346	0.001302
hsa_circ_	1.372688	0.001305
hsa_circ_	-0.79553	0.001307
hsa_circ_	0.736031	0.001314
hsa_circ_	1.490928	0.001318
hsa_circ_	1.024749	0.001323
hsa_circ_	-0.68668	0.001324
hsa_circ_	-1.11915	0.00133
hsa_circ_	0.770691	0.001339
hsa_circ_	0.852057	0.001344
hsa_circ_	-1.05956	0.001351
hsa_circ_	-1.01974	0.001351
hsa_circ_	1.220948	0.001356
hsa_circ_	1.277057	0.001358
hsa_circ_	-0.7727	0.001358
hsa_circ_	1.264313	0.001359
hsa_circ_	-0.88556	0.00136
hsa_circ_	1.060062	0.001365
hsa_circ_	0.884753	0.001367
hsa_circ_	0.858519	0.001378
hsa_circ_	0.700773	0.001383
hsa_circ_	1.468662	0.001383
hsa_circ_	-1.3552	0.001383
hsa_circ_	2.174602	0.001384
hsa_circ_	0.839566	0.001384
hsa_circ_	-0.85522	0.001388
hsa_circ_	0.939758	0.001405
hsa_circ_	1.54841	0.001407
hsa_circ_	-0.65838	0.00141
hsa_circ_	0.755853	0.001416
hsa_circ_	-1.55682	0.001416
hsa_circ_	0.705518	0.00142
hsa_circ_	1.542126	0.00142
hsa_circ_	1.62519	0.00142
hsa_circ_	-0.8134	0.001421
hsa_circ_	1.983064	0.001423
hsa_circ_	1.721458	0.001428
hsa_circ_	-2.22373	0.001431
hsa_circ_	0.886732	0.001433
hsa_circ_	0.747571	0.001434
hsa_circ_	0.805019	0.001434
hsa_circ_	1.000077	0.001436
hsa_circ_	0.795855	0.001439
hsa_circ_	-0.80986	0.001448

hsa_circ_	1.048277	0.001452
hsa_circ_	-1.20697	0.001454
hsa_circ_	0.712405	0.001459
hsa_circ_	-0.90178	0.001465
hsa_circ_	0.723439	0.001465
hsa_circ_	1.536153	0.001466
hsa_circ_	1.117642	0.001467
hsa_circ_	1.963024	0.001468
hsa_circ_	0.929787	0.001471
hsa_circ_	-1.62016	0.001476
hsa_circ_	1.019924	0.001478
hsa_circ_	0.982496	0.001479
hsa_circ_	0.800341	0.001484
hsa_circ_	-0.7011	0.001485
hsa_circ_	1.554484	0.001485
hsa_circ_	2.932062	0.001489
hsa_circ_	2.18262	0.00149
hsa_circ_	1.860899	0.001491
hsa_circ_	-0.79991	0.001493
hsa_circ_	-1.5494	0.001498
hsa_circ_	-1.09514	0.001499
hsa_circ_	-0.77035	0.001499
hsa_circ_	1.080659	0.001504
hsa_circ_	1.446709	0.001504
hsa_circ_	0.690508	0.001512
hsa_circ_	-1.23924	0.001517
hsa_circ_	1.200249	0.001526
hsa_circ_	-1.58111	0.001532
hsa_circ_	0.881968	0.001534
hsa_circ_	-0.82959	0.001539
hsa_circ_	1.463041	0.001543
hsa_circ_	-0.83684	0.001546
hsa_circ_	1.474841	0.001548
hsa_circ_	-1.27791	0.00155
hsa_circ_	1.578085	0.001551
hsa_circ_	-0.99047	0.001556
hsa_circ_	1.056114	0.001557
hsa_circ_	-0.86974	0.001559
hsa_circ_	1.527684	0.001564
hsa_circ_	0.883183	0.00157
hsa_circ_	-0.76661	0.001571
hsa_circ_	1.476557	0.001575
hsa_circ_	0.972853	0.001577
hsa_circ_	1.639474	0.001583
hsa_circ_	-1.10103	0.001586
hsa_circ_	1.24188	0.001588
hsa_circ_	-1.47974	0.001589
hsa_circ_	1.442457	0.001593
hsa_circ_	1.141551	0.0016
hsa_circ_	0.7568	0.001601
hsa_circ_	-1.3557	0.001601
hsa_circ_	-0.94532	0.001605
hsa_circ_	1.189745	0.001613
hsa_circ_	0.859951	0.001614

hsa_circ_	-0.93362	0.001626
hsa_circ_	1.145136	0.001627
hsa_circ_	0.932804	0.001631
hsa_circ_	1.065999	0.001631
hsa_circ_	-0.91821	0.001633
hsa_circ_	1.385421	0.001635
hsa_circ_	0.743039	0.001639
hsa_circ_	1.303713	0.001648
hsa_circ_	-0.81863	0.001648
hsa_circ_	1.082528	0.001657
hsa_circ_	-1.08477	0.001662
hsa_circ_	0.940519	0.001672
hsa_circ_	0.788108	0.001675
hsa_circ_	1.362901	0.001676
hsa_circ_	-1.00442	0.00168
hsa_circ_	0.65614	0.001686
hsa_circ_	1.463391	0.001687
hsa_circ_	-1.41272	0.001688
hsa_circ_	1.401218	0.00169
hsa_circ_	1.909769	0.001693
hsa_circ_	0.692658	0.001693
hsa_circ_	-1.02271	0.001695
hsa_circ_	0.960938	0.001696
hsa_circ_	0.79676	0.001698
hsa_circ_	0.732982	0.001699
hsa_circ_	1.488974	0.001701
hsa_circ_	0.95376	0.001703
hsa_circ_	-0.713	0.001706
hsa_circ_	1.504481	0.001708
hsa_circ_	0.921828	0.00171
hsa_circ_	1.987589	0.001711
hsa_circ_	-1.83725	0.001713
hsa_circ_	1.39975	0.001721
hsa_circ_	0.989199	0.001727
hsa_circ_	-0.74418	0.001732
hsa_circ_	2.477255	0.001736
hsa_circ_	2.017607	0.00174
hsa_circ_	-0.73867	0.001741
hsa_circ_	-1.18286	0.001744
hsa_circ_	1.288152	0.001748
hsa_circ_	0.858084	0.001758
hsa_circ_	1.397947	0.001761
hsa_circ_	1.344388	0.001764
hsa_circ_	1.382511	0.001773
hsa_circ_	0.977214	0.001773
hsa_circ_	1.896001	0.001774
hsa_circ_	0.686392	0.001779
hsa_circ_	-1.74158	0.001779
hsa_circ_	1.018397	0.001779
hsa_circ_	0.836337	0.001782
hsa_circ_	-0.703	0.001784
hsa_circ_	-0.80841	0.00179
hsa_circ_	-0.74754	0.001791
hsa_circ_	-0.78316	0.001793

hsa_circ_	1.916037	0.001794
hsa_circ_	-1.01647	0.001796
hsa_circ_	0.643816	0.001796
hsa_circ_	-1.05497	0.001796
hsa_circ_	0.824603	0.001799
hsa_circ_	-1.42193	0.001803
hsa_circ_	-0.88415	0.001809
hsa_circ_	1.229004	0.001811
hsa_circ_	-1.01029	0.001816
hsa_circ_	0.712173	0.001817
hsa_circ_	1.382794	0.00182
hsa_circ_	1.386119	0.001825
hsa_circ_	-0.91809	0.001833
hsa_circ_	2.141396	0.001836
hsa_circ_	1.29058	0.001838
hsa_circ_	1.269543	0.00184
hsa_circ_	0.803402	0.001843
hsa_circ_	-0.8279	0.001853
hsa_circ_	0.904909	0.001856
hsa_circ_	-1.48819	0.001862
hsa_circ_	0.809097	0.001873
hsa_circ_	2.097098	0.001876
hsa_circ_	-0.8023	0.001885
hsa_circ_	1.16145	0.001892
hsa_circ_	0.668073	0.001896
hsa_circ_	-1.24404	0.001902
hsa_circ_	0.761839	0.001903
hsa_circ_	0.891848	0.001905
hsa_circ_	-1.16992	0.001908
hsa_circ_	2.11559	0.00191
hsa_circ_	1.457109	0.001912
hsa_circ_	-1.43952	0.001917
hsa_circ_	1.153642	0.001919
hsa_circ_	-0.7662	0.001919
hsa_circ_	1.082825	0.001922
hsa_circ_	1.22241	0.001923
hsa_circ_	1.076908	0.001936
hsa_circ_	1.328187	0.001939
hsa_circ_	1.484756	0.001941
hsa_circ_	-1.4541	0.001946
hsa_circ_	0.695546	0.001955
hsa_circ_	1.767006	0.001959
hsa_circ_	0.932192	0.001965
hsa_circ_	0.709891	0.00197
hsa_circ_	0.746071	0.00197
hsa_circ_	0.798254	0.001976
hsa_circ_	1.88241	0.001985
hsa_circ_	-0.97677	0.001985
hsa_circ_	1.383217	0.001989
hsa_circ_	1.346389	0.001991
hsa_circ_	1.312723	0.001992
hsa_circ_	0.720267	0.001992
hsa_circ_	0.912472	0.001993
hsa_circ_	-0.74981	0.001994

hsa_circ_	-0.995	0.001994
hsa_circ_	0.642412	0.001996
hsa_circ_	-0.64227	0.001998
hsa_circ_	1.755767	0.001999
hsa_circ_	1.196421	0.002004
hsa_circ_	-1.03226	0.002009
hsa_circ_	1.278928	0.00201
hsa_circ_	-0.63027	0.002025
hsa_circ_	1.356452	0.002025
hsa_circ_	1.508241	0.002034
hsa_circ_	-1.05763	0.002045
hsa_circ_	-0.78103	0.002046
hsa_circ_	0.9791	0.002053
hsa_circ_	1.013976	0.002053
hsa_circ_	-1.81977	0.002056
hsa_circ_	-1.16211	0.002061
hsa_circ_	0.748803	0.002062
hsa_circ_	-1.29796	0.002065
hsa_circ_	-0.95218	0.002071
hsa_circ_	-0.68598	0.002072
hsa_circ_	0.872279	0.002079
hsa_circ_	2.217267	0.00208
hsa_circ_	0.618057	0.002084
hsa_circ_	0.980958	0.0021
hsa_circ_	-0.78517	0.002102
hsa_circ_	-1.0203	0.002108
hsa_circ_	1.152138	0.002112
hsa_circ_	0.987934	0.002119
hsa_circ_	1.249974	0.002121
hsa_circ_	-1.08457	0.002124
hsa_circ_	1.288308	0.002126
hsa_circ_	1.603137	0.002127
hsa_circ_	2.49292	0.002128
hsa_circ_	1.066076	0.002135
hsa_circ_	1.390374	0.002138
hsa_circ_	1.182186	0.002138
hsa_circ_	0.948715	0.00214
hsa_circ_	-0.73237	0.00214
hsa_circ_	0.809536	0.002148
hsa_circ_	0.757988	0.002154
hsa_circ_	1.150124	0.002156
hsa_circ_	3.011246	0.002158
hsa_circ_	0.796051	0.002161
hsa_circ_	-1.81298	0.002161
hsa_circ_	0.772896	0.002162
hsa_circ_	0.73083	0.002163
hsa_circ_	1.318722	0.002167
hsa_circ_	0.945568	0.002168
hsa_circ_	1.145005	0.002169
hsa_circ_	-2.27957	0.002173
hsa_circ_	1.745786	0.002173
hsa_circ_	-1.22923	0.002178
hsa_circ_	1.705106	0.002179
hsa_circ_	-0.94841	0.002181

hsa_circ_	-1.01926	0.002181
hsa_circ_	0.851435	0.002194
hsa_circ_	-0.97598	0.002194
hsa_circ_	1.801322	0.002202
hsa_circ_	1.005871	0.002212
hsa_circ_	1.33033	0.002214
hsa_circ_	-0.9279	0.002215
hsa_circ_	0.939098	0.002218
hsa_circ_	1.769725	0.002221
hsa_circ_	1.550943	0.002231
hsa_circ_	0.888411	0.002234
hsa_circ_	1.288633	0.002237
hsa_circ_	1.373783	0.002238
hsa_circ_	0.997961	0.002239
hsa_circ_	-0.69051	0.00225
hsa_circ_	-1.09244	0.002262
hsa_circ_	1.188801	0.002273
hsa_circ_	-0.75301	0.002274
hsa_circ_	2.103539	0.002276
hsa_circ_	1.380754	0.002279
hsa_circ_	1.577572	0.00228
hsa_circ_	1.292502	0.00228
hsa_circ_	0.778777	0.002285
hsa_circ_	1.44016	0.002286
hsa_circ_	1.169673	0.002289
hsa_circ_	1.306347	0.00229
hsa_circ_	0.730706	0.002296
hsa_circ_	-0.85654	0.002299
hsa_circ_	1.354626	0.002301
hsa_circ_	-0.91448	0.002304
hsa_circ_	-1.29283	0.002308
hsa_circ_	-1.13138	0.002308
hsa_circ_	0.724461	0.002316
hsa_circ_	0.744531	0.002319
hsa_circ_	1.029892	0.002323
hsa_circ_	0.998902	0.002325
hsa_circ_	0.741943	0.002325
hsa_circ_	-0.7933	0.002334
hsa_circ_	1.350677	0.002335
hsa_circ_	-0.62866	0.002339
hsa_circ_	0.630926	0.002346
hsa_circ_	-1.29441	0.002348
hsa_circ_	1.589401	0.002349
hsa_circ_	-1.3227	0.002352
hsa_circ_	1.260113	0.00236
hsa_circ_	1.933973	0.002362
hsa_circ_	0.864556	0.002368
hsa_circ_	0.804966	0.002373
hsa_circ_	-0.94357	0.002377
hsa_circ_	1.351855	0.002378
hsa_circ_	0.705837	0.002379
hsa_circ_	1.550734	0.002384
hsa_circ_	1.355705	0.002384
hsa_circ_	-1.94659	0.002395

hsa_circ_	-0.91897	0.002397
hsa_circ_	1.458474	0.002398
hsa_circ_	-1.96403	0.002401
hsa_circ_	1.230001	0.002401
hsa_circ_	1.8614	0.002405
hsa_circ_	0.742113	0.002407
hsa_circ_	-1.11207	0.00241
hsa_circ_	-1.01562	0.002416
hsa_circ_	1.245972	0.002416
hsa_circ_	0.789849	0.002416
hsa_circ_	-1.46188	0.002421
hsa_circ_	0.67466	0.002422
hsa_circ_	0.997842	0.002426
hsa_circ_	1.407747	0.002434
hsa_circ_	0.786401	0.002434
hsa_circ_	0.965366	0.002441
hsa_circ_	-1.31327	0.002451
hsa_circ_	0.595511	0.002451
hsa_circ_	-0.86273	0.002455
hsa_circ_	1.312478	0.002462
hsa_circ_	1.027091	0.002466
hsa_circ_	-1.06568	0.002473
hsa_circ_	-1.24624	0.002475
hsa_circ_	-1.29627	0.002482
hsa_circ_	-0.64597	0.002483
hsa_circ_	0.796814	0.002483
hsa_circ_	1.512366	0.002484
hsa_circ_	-0.63835	0.002488
hsa_circ_	-0.71683	0.002502
hsa_circ_	-0.84427	0.002503
hsa_circ_	-0.64374	0.002504
hsa_circ_	0.643367	0.002505
hsa_circ_	1.61955	0.002505
hsa_circ_	1.383166	0.002516
hsa_circ_	0.924342	0.002521
hsa_circ_	-0.87553	0.002526
hsa_circ_	0.959272	0.00253
hsa_circ_	0.836413	0.002534
hsa_circ_	1.296643	0.002535
hsa_circ_	-0.76392	0.002536
hsa_circ_	1.249229	0.002538
hsa_circ_	1.664241	0.002541
hsa_circ_	1.301749	0.002541
hsa_circ_	-0.67128	0.002544
hsa_circ_	2.20808	0.002548
hsa_circ_	0.907813	0.002548
hsa_circ_	1.13515	0.002557
hsa_circ_	1.331933	0.002557
hsa_circ_	-1.03645	0.002561
hsa_circ_	1.210011	0.002566
hsa_circ_	1.153697	0.002567
hsa_circ_	-0.85362	0.002567
hsa_circ_	1.053678	0.002568
hsa_circ_	0.912335	0.002577

hsa_circ_	-1.30731	0.002584
hsa_circ_	0.954545	0.002585
hsa_circ_	0.944159	0.002585
hsa_circ_	-1.31691	0.002586
hsa_circ_	1.615473	0.002587
hsa_circ_	0.823476	0.002588
hsa_circ_	1.216097	0.002588
hsa_circ_	1.080557	0.002589
hsa_circ_	0.851054	0.00259
hsa_circ_	-1.08925	0.002591
hsa_circ_	0.890492	0.002594
hsa_circ_	-0.67734	0.002596
hsa_circ_	1.004983	0.002596
hsa_circ_	1.506578	0.002597
hsa_circ_	1.878938	0.0026
hsa_circ_	1.57897	0.0026
hsa_circ_	1.160562	0.0026
hsa_circ_	0.63148	0.002607
hsa_circ_	1.664916	0.002609
hsa_circ_	0.649054	0.002609
hsa_circ_	-0.75207	0.002618
hsa_circ_	0.980783	0.002624
hsa_circ_	0.947325	0.002624
hsa_circ_	1.149917	0.00263
hsa_circ_	-0.98853	0.002631
hsa_circ_	0.579259	0.002642
hsa_circ_	1.404073	0.002658
hsa_circ_	0.73101	0.002659
hsa_circ_	1.263527	0.002665
hsa_circ_	-0.62635	0.002669
hsa_circ_	-0.89905	0.002669
hsa_circ_	0.632955	0.002681
hsa_circ_	0.987286	0.002684
hsa_circ_	0.911738	0.002686
hsa_circ_	1.527943	0.00269
hsa_circ_	0.724168	0.00269
hsa_circ_	1.278913	0.002696
hsa_circ_	1.24538	0.002704
hsa_circ_	-0.97022	0.00271
hsa_circ_	-0.67518	0.00272
hsa_circ_	-0.85079	0.002725
hsa_circ_	0.99956	0.002729
hsa_circ_	-1.11295	0.00273
hsa_circ_	0.844088	0.002731
hsa_circ_	0.852704	0.002736
hsa_circ_	0.938696	0.002736
hsa_circ_	0.735309	0.002739
hsa_circ_	1.326452	0.00274
hsa_circ_	0.78014	0.002741
hsa_circ_	1.051714	0.002744
hsa_circ_	-1.06791	0.002745
hsa_circ_	1.960774	0.002747
hsa_circ_	0.765174	0.002749
hsa_circ_	0.740439	0.00275

hsa_circ_	-0.77124	0.002757
hsa_circ_	1.34045	0.002758
hsa_circ_	1.146816	0.002761
hsa_circ_	1.287615	0.002764
hsa_circ_	1.224083	0.002771
hsa_circ_	0.681711	0.002775
hsa_circ_	0.884069	0.00278
hsa_circ_	-1.16528	0.002783
hsa_circ_	0.772655	0.002785
hsa_circ_	-0.85288	0.002787
hsa_circ_	1.582151	0.002788
hsa_circ_	-0.59077	0.002795
hsa_circ_	0.616125	0.002803
hsa_circ_	-0.61608	0.002803
hsa_circ_	-1.88478	0.002804
hsa_circ_	-0.95046	0.002812
hsa_circ_	0.653065	0.002812
hsa_circ_	-0.59123	0.00282
hsa_circ_	1.426756	0.002824
hsa_circ_	0.659866	0.002825
hsa_circ_	0.894114	0.002827
hsa_circ_	1.590795	0.002827
hsa_circ_	-0.6669	0.002828
hsa_circ_	0.727976	0.002833
hsa_circ_	1.058049	0.002835
hsa_circ_	0.802051	0.002836
hsa_circ_	0.696587	0.002839
hsa_circ_	2.170734	0.002843
hsa_circ_	-0.86364	0.002845
hsa_circ_	-0.76025	0.002846
hsa_circ_	0.905365	0.002858
hsa_circ_	-0.93947	0.002864
hsa_circ_	-1.35298	0.002864
hsa_circ_	0.591074	0.002868
hsa_circ_	0.690107	0.002871
hsa_circ_	0.569287	0.002874
hsa_circ_	-0.71558	0.002876
hsa_circ_	0.610428	0.002877
hsa_circ_	1.142133	0.00288
hsa_circ_	1.383308	0.00288
hsa_circ_	-1.03471	0.002881
hsa_circ_	0.610403	0.002884
hsa_circ_	0.795357	0.002891
hsa_circ_	-0.74063	0.002899
hsa_circ_	-0.781	0.002906
hsa_circ_	-0.94129	0.002914
hsa_circ_	-1.26543	0.002914
hsa_circ_	0.862905	0.002917
hsa_circ_	0.951431	0.002926
hsa_circ_	0.84561	0.002933
hsa_circ_	0.664778	0.002934
hsa_circ_	1.443631	0.00294
hsa_circ_	0.838026	0.002943
hsa_circ_	0.598864	0.002946

hsa_circ_	1.158815	0.002948
hsa_circ_	0.974527	0.002949
hsa_circ_	-0.69375	0.002956
hsa_circ_	1.779644	0.002958
hsa_circ_	1.214142	0.002962
hsa_circ_	0.670306	0.00297
hsa_circ_	0.767116	0.002971
hsa_circ_	1.366525	0.002972
hsa_circ_	1.20779	0.002974
hsa_circ_	1.241776	0.002976
hsa_circ_	0.797206	0.002979
hsa_circ_	-0.71746	0.002982
hsa_circ_	0.909559	0.002982
hsa_circ_	1.049429	0.002985
hsa_circ_	-0.68219	0.002988
hsa_circ_	1.555579	0.002993
hsa_circ_	-1.74999	0.002995
hsa_circ_	-1.30991	0.002999
hsa_circ_	1.377067	0.003001
hsa_circ_	-0.79781	0.00302
hsa_circ_	0.735159	0.003028
hsa_circ_	1.469272	0.003031
hsa_circ_	-1.38141	0.003034
hsa_circ_	1.363688	0.003035
hsa_circ_	-0.63269	0.003035
hsa_circ_	-2.62307	0.003036
hsa_circ_	0.769126	0.003038
hsa_circ_	0.720058	0.003039
hsa_circ_	0.756987	0.003042
hsa_circ_	0.748068	0.003043
hsa_circ_	0.737676	0.003045
hsa_circ_	1.810472	0.003051
hsa_circ_	0.905219	0.003053
hsa_circ_	1.923284	0.00306
hsa_circ_	1.174112	0.003064
hsa_circ_	1.036415	0.003072
hsa_circ_	1.248086	0.003075
hsa_circ_	-1.14712	0.003075
hsa_circ_	-0.62226	0.00308
hsa_circ_	0.628249	0.003083
hsa_circ_	1.264044	0.003087
hsa_circ_	0.675952	0.003089
hsa_circ_	0.79485	0.003092
hsa_circ_	-0.82159	0.003093
hsa_circ_	1.336258	0.0031
hsa_circ_	0.742327	0.0031
hsa_circ_	1.167598	0.003101
hsa_circ_	0.650067	0.003102
hsa_circ_	0.804832	0.00311
hsa_circ_	1.170437	0.003114
hsa_circ_	-0.62541	0.003125
hsa_circ_	1.156153	0.003129
hsa_circ_	-1.00366	0.00313
hsa_circ_	-1.32124	0.003135

hsa_circ_	1.155031	0.003137
hsa_circ_	-0.87992	0.003148
hsa_circ_	-1.35243	0.003156
hsa_circ_	1.04921	0.003156
hsa_circ_	-1.04379	0.003161
hsa_circ_	-0.85591	0.003161
hsa_circ_	-0.95072	0.003168
hsa_circ_	-0.7713	0.003172
hsa_circ_	1.147837	0.003176
hsa_circ_	-1.21687	0.003176
hsa_circ_	-0.98901	0.003177
hsa_circ_	0.820756	0.00318
hsa_circ_	1.144362	0.00319
hsa_circ_	0.834175	0.003191
hsa_circ_	-0.98428	0.003194
hsa_circ_	2.284781	0.003195
hsa_circ_	0.626555	0.003197
hsa_circ_	0.855307	0.003209
hsa_circ_	0.603569	0.003212
hsa_circ_	-1.01521	0.003212
hsa_circ_	1.229748	0.003216
hsa_circ_	-0.63817	0.003216
hsa_circ_	-1.22622	0.003218
hsa_circ_	1.627747	0.003229
hsa_circ_	0.915985	0.00323
hsa_circ_	-0.89076	0.003231
hsa_circ_	1.053167	0.003232
hsa_circ_	-0.91442	0.003234
hsa_circ_	1.462287	0.003243
hsa_circ_	-0.92163	0.003245
hsa_circ_	-1.11627	0.00325
hsa_circ_	0.645443	0.003257
hsa_circ_	0.612563	0.00326
hsa_circ_	1.732674	0.003262
hsa_circ_	0.654684	0.003271
hsa_circ_	-0.82512	0.003276
hsa_circ_	1.406858	0.003282
hsa_circ_	-1.07803	0.003282
hsa_circ_	0.715326	0.003287
hsa_circ_	1.412617	0.003287
hsa_circ_	-0.77879	0.003298
hsa_circ_	-0.95601	0.003299
hsa_circ_	-0.99617	0.0033
hsa_circ_	-0.67552	0.003302
hsa_circ_	-0.69657	0.003305
hsa_circ_	1.120143	0.003308
hsa_circ_	-1.21421	0.003314
hsa_circ_	-0.62485	0.003316
hsa_circ_	1.955167	0.003319
hsa_circ_	-0.75588	0.003321
hsa_circ_	-0.58872	0.003322
hsa_circ_	1.463325	0.003323
hsa_circ_	1.055451	0.003329
hsa_circ_	-0.93726	0.003337

hsa_circ_	0.949702	0.003337
hsa_circ_	-0.61875	0.00334
hsa_circ_	0.566978	0.003341
hsa_circ_	1.298968	0.003348
hsa_circ_	0.628272	0.003357
hsa_circ_	-0.67225	0.00336
hsa_circ_	0.642767	0.003363
hsa_circ_	-0.61588	0.003368
hsa_circ_	0.639386	0.00337
hsa_circ_	-0.72082	0.003371
hsa_circ_	-0.72942	0.003375
hsa_circ_	-0.72578	0.003378
hsa_circ_	-0.82396	0.003386
hsa_circ_	-1.08115	0.003387
hsa_circ_	-1.06011	0.003387
hsa_circ_	-0.55702	0.003393
hsa_circ_	-0.79028	0.003398
hsa_circ_	1.147742	0.003403
hsa_circ_	-0.64665	0.003407
hsa_circ_	-1.08092	0.003407
hsa_circ_	0.83565	0.003409
hsa_circ_	-0.66148	0.003411
hsa_circ_	0.584072	0.003414
hsa_circ_	-1.01538	0.003419
hsa_circ_	-1.20463	0.003424
hsa_circ_	-0.55134	0.003428
hsa_circ_	0.831999	0.003438
hsa_circ_	-0.85077	0.003444
hsa_circ_	0.918724	0.003446
hsa_circ_	0.659296	0.003451
hsa_circ_	-0.6722	0.003452
hsa_circ_	-0.62303	0.003453
hsa_circ_	1.264655	0.003457
hsa_circ_	-1.06547	0.003457
hsa_circ_	0.606469	0.003458
hsa_circ_	0.97185	0.00346
hsa_circ_	-1.12526	0.003464
hsa_circ_	0.783871	0.003468
hsa_circ_	0.849866	0.00347
hsa_circ_	0.67437	0.003477
hsa_circ_	0.839553	0.003478
hsa_circ_	-0.77499	0.00348
hsa_circ_	0.631616	0.003481
hsa_circ_	0.919561	0.003483
hsa_circ_	0.596988	0.003491
hsa_circ_	0.839806	0.003492
hsa_circ_	0.954784	0.003501
hsa_circ_	1.609616	0.003502
hsa_circ_	1.085239	0.003504
hsa_circ_	1.013697	0.003504
hsa_circ_	1.776753	0.003504
hsa_circ_	1.287769	0.003512
hsa_circ_	-2.54512	0.003512
hsa_circ_	-0.76396	0.003513

hsa_circ_	-1.08008	0.003515
hsa_circ_	-0.64702	0.00352
hsa_circ_	0.655994	0.003521
hsa_circ_	-1.88452	0.003524
hsa_circ_	1.00491	0.003528
hsa_circ_	-0.61798	0.003531
hsa_circ_	0.737911	0.003531
hsa_circ_	-1.28054	0.00354
hsa_circ_	-1.07964	0.003545
hsa_circ_	-0.6921	0.003549
hsa_circ_	-0.92938	0.003555
hsa_circ_	-0.86515	0.003559
hsa_circ_	0.60576	0.003561
hsa_circ_	1.240339	0.003566
hsa_circ_	0.931383	0.003571
hsa_circ_	-0.71172	0.003588
hsa_circ_	1.162925	0.003589
hsa_circ_	-0.59267	0.00359
hsa_circ_	-0.90456	0.003595
hsa_circ_	-1.60008	0.003597
hsa_circ_	0.787748	0.0036
hsa_circ_	0.815914	0.003601
hsa_circ_	-0.7983	0.003607
hsa_circ_	1.050162	0.003609
hsa_circ_	-0.97994	0.003612
hsa_circ_	-1.07212	0.003615
hsa_circ_	-0.7436	0.003626
hsa_circ_	0.707605	0.003634
hsa_circ_	0.691424	0.003643
hsa_circ_	1.617272	0.003652
hsa_circ_	-0.69327	0.003656
hsa_circ_	-0.66181	0.003658
hsa_circ_	0.965909	0.00366
hsa_circ_	1.491206	0.003661
hsa_circ_	0.651939	0.003661
hsa_circ_	1.324972	0.003665
hsa_circ_	1.037168	0.003666
hsa_circ_	1.17775	0.003666
hsa_circ_	-0.72678	0.003671
hsa_circ_	-0.98485	0.00368
hsa_circ_	-0.7101	0.00369
hsa_circ_	0.762252	0.003694
hsa_circ_	-0.59647	0.003696
hsa_circ_	-0.83755	0.003699
hsa_circ_	-0.8537	0.003703
hsa_circ_	1.027865	0.003704
hsa_circ_	0.919678	0.003705
hsa_circ_	0.691291	0.003708
hsa_circ_	-1.23382	0.003715
hsa_circ_	1.137118	0.003716
hsa_circ_	0.691775	0.003719
hsa_circ_	0.814686	0.003741
hsa_circ_	-0.59996	0.003746
hsa_circ_	-1.06371	0.003746

hsa_circ_	-0.97098	0.003753
hsa_circ_	-1.02925	0.003753
hsa_circ_	0.782453	0.003754
hsa_circ_	-0.92283	0.003756
hsa_circ_	-0.86501	0.003758
hsa_circ_	1.031514	0.00376
hsa_circ_	0.926728	0.003761
hsa_circ_	1.37762	0.003773
hsa_circ_	1.237923	0.003781
hsa_circ_	-1.04407	0.003787
hsa_circ_	0.689106	0.00379
hsa_circ_	0.881175	0.003793
hsa_circ_	2.854229	0.003795
hsa_circ_	1.842508	0.003806
hsa_circ_	-0.68919	0.003806
hsa_circ_	1.084787	0.003809
hsa_circ_	-0.67073	0.003815
hsa_circ_	0.890297	0.003821
hsa_circ_	-1.16379	0.003821
hsa_circ_	-0.98199	0.003823
hsa_circ_	-2.39109	0.003824
hsa_circ_	1.358545	0.003831
hsa_circ_	-0.66203	0.003839
hsa_circ_	1.869641	0.003849
hsa_circ_	-1.19457	0.00385
hsa_circ_	-0.83994	0.003852
hsa_circ_	0.903516	0.003853
hsa_circ_	-0.65637	0.003855
hsa_circ_	-0.62573	0.003865
hsa_circ_	-0.93039	0.003869
hsa_circ_	0.834401	0.00387
hsa_circ_	-1.21818	0.003872
hsa_circ_	1.102647	0.003873
hsa_circ_	1.502888	0.003873
hsa_circ_	0.909245	0.003883
hsa_circ_	0.654638	0.003885
hsa_circ_	-1.04264	0.003887
hsa_circ_	1.177225	0.003889
hsa_circ_	1.347761	0.003895
hsa_circ_	0.611079	0.003901
hsa_circ_	1.771443	0.003902
hsa_circ_	1.378304	0.003904
hsa_circ_	-0.82473	0.00391
hsa_circ_	0.876872	0.003912
hsa_circ_	1.240678	0.003913
hsa_circ_	-1.06752	0.003917
hsa_circ_	0.81184	0.003922
hsa_circ_	-0.64007	0.003922
hsa_circ_	1.133068	0.003923
hsa_circ_	0.785322	0.003936
hsa_circ_	0.896861	0.003946
hsa_circ_	1.128692	0.003947
hsa_circ_	-1.32208	0.00395
hsa_circ_	-1.1265	0.003955

hsa_circ_	0.567773	0.003956
hsa_circ_	-0.55602	0.003957
hsa_circ_	-1.2048	0.003957
hsa_circ_	-0.66878	0.003959
hsa_circ_	-0.97521	0.003971
hsa_circ_	-1.06609	0.003976
hsa_circ_	1.642197	0.003978
hsa_circ_	0.894486	0.003987
hsa_circ_	0.634668	0.00399
hsa_circ_	1.260325	0.00399
hsa_circ_	0.625817	0.003997
hsa_circ_	1.262669	0.003997
hsa_circ_	0.812632	0.003999
hsa_circ_	0.887459	0.004
hsa_circ_	-1.48936	0.004005
hsa_circ_	1.034036	0.004007
hsa_circ_	-1.09638	0.004008
hsa_circ_	0.794735	0.004008
hsa_circ_	0.997173	0.004011
hsa_circ_	-0.64903	0.004021
hsa_circ_	1.211407	0.004026
hsa_circ_	1.122872	0.004029
hsa_circ_	0.767761	0.004031
hsa_circ_	-0.95292	0.004033
hsa_circ_	-0.63774	0.004035
hsa_circ_	0.560997	0.004036
hsa_circ_	0.576054	0.004039
hsa_circ_	-0.69231	0.004042
hsa_circ_	-0.72221	0.004042
hsa_circ_	0.76534	0.004043
hsa_circ_	-0.83454	0.004043
hsa_circ_	-0.99825	0.004045
hsa_circ_	-1.03601	0.004048
hsa_circ_	0.764519	0.004049
hsa_circ_	-0.68062	0.004051
hsa_circ_	-1.06312	0.004054
hsa_circ_	-0.59792	0.004064
hsa_circ_	0.700265	0.004068
hsa_circ_	0.881318	0.004072
hsa_circ_	0.569933	0.004073
hsa_circ_	-0.6541	0.004074
hsa_circ_	1.23964	0.004077
hsa_circ_	0.937111	0.004077
hsa_circ_	0.700039	0.004079
hsa_circ_	-0.62337	0.00408
hsa_circ_	0.590599	0.004081
hsa_circ_	-0.77617	0.00409
hsa_circ_	1.675114	0.0041
hsa_circ_	-0.63515	0.004108
hsa_circ_	0.542956	0.004118
hsa_circ_	1.133801	0.00412
hsa_circ_	0.683384	0.004123
hsa_circ_	-0.95879	0.004125
hsa_circ_	-1.05395	0.00414

hsa_circ_	-0.79753	0.004145
hsa_circ_	-0.91857	0.004153
hsa_circ_	0.65488	0.00416
hsa_circ_	1.225883	0.00416
hsa_circ_	-1.81037	0.004163
hsa_circ_	0.902479	0.004169
hsa_circ_	-0.65729	0.004176
hsa_circ_	-0.65464	0.004182
hsa_circ_	-1.0594	0.004185
hsa_circ_	1.469051	0.004185
hsa_circ_	0.649588	0.004186
hsa_circ_	0.908759	0.004188
hsa_circ_	1.621669	0.004188
hsa_circ_	1.245508	0.004195
hsa_circ_	1.296572	0.004204
hsa_circ_	0.540172	0.004206
hsa_circ_	-1.15244	0.004211
hsa_circ_	0.857331	0.004217
hsa_circ_	-2.14297	0.004221
hsa_circ_	1.059633	0.004226
hsa_circ_	-1.12373	0.00423
hsa_circ_	0.740454	0.004231
hsa_circ_	-1.03872	0.004231
hsa_circ_	1.538693	0.004232
hsa_circ_	1.319752	0.004234
hsa_circ_	-0.55527	0.004237
hsa_circ_	1.291673	0.00424
hsa_circ_	0.938111	0.004242
hsa_circ_	-0.92711	0.004243
hsa_circ_	0.633762	0.004245
hsa_circ_	-1.13711	0.004247
hsa_circ_	0.677258	0.004249
hsa_circ_	-0.65351	0.00425
hsa_circ_	1.514344	0.00425
hsa_circ_	1.118066	0.004254
hsa_circ_	0.749047	0.004254
hsa_circ_	-0.69258	0.004263
hsa_circ_	0.637542	0.004264
hsa_circ_	0.889741	0.004265
hsa_circ_	0.640247	0.004269
hsa_circ_	-0.66616	0.004273
hsa_circ_	-0.66047	0.004277
hsa_circ_	-0.9753	0.004284
hsa_circ_	1.052725	0.004285
hsa_circ_	1.26009	0.00429
hsa_circ_	-1.00908	0.00429
hsa_circ_	0.899007	0.004293
hsa_circ_	1.333301	0.004294
hsa_circ_	-1.4597	0.004295
hsa_circ_	1.639603	0.004297
hsa_circ_	0.687246	0.004303
hsa_circ_	-0.56706	0.004311
hsa_circ_	1.544302	0.004312
hsa_circ_	-1.11999	0.004313

hsa_circ_	0.906212	0.004318
hsa_circ_	0.782818	0.004318
hsa_circ_	0.531748	0.004324
hsa_circ_	-1.32849	0.004325
hsa_circ_	0.934323	0.004329
hsa_circ_	1.33297	0.004329
hsa_circ_	1.257402	0.004331
hsa_circ_	-1.01122	0.004341
hsa_circ_	-0.84333	0.004342
hsa_circ_	0.842467	0.004342
hsa_circ_	-0.6033	0.004342
hsa_circ_	1.011177	0.004345
hsa_circ_	1.518343	0.004345
hsa_circ_	-2.32143	0.004346
hsa_circ_	-0.9507	0.004346
hsa_circ_	0.611427	0.004351
hsa_circ_	-1.22282	0.004352
hsa_circ_	1.482765	0.004353
hsa_circ_	-0.78819	0.004356
hsa_circ_	0.617147	0.004366
hsa_circ_	0.737575	0.004371
hsa_circ_	0.684597	0.00438
hsa_circ_	-1.04955	0.004381
hsa_circ_	-0.89042	0.004381
hsa_circ_	0.583911	0.004382
hsa_circ_	0.878596	0.004383
hsa_circ_	0.941136	0.004398
hsa_circ_	0.796245	0.004399
hsa_circ_	0.601514	0.0044
hsa_circ_	-0.90143	0.004404
hsa_circ_	0.571482	0.004413
hsa_circ_	-1.0442	0.004414
hsa_circ_	-1.04124	0.004415
hsa_circ_	0.606926	0.004417
hsa_circ_	0.688528	0.004418
hsa_circ_	-0.63061	0.004419
hsa_circ_	1.518976	0.004421
hsa_circ_	0.676321	0.004421
hsa_circ_	-0.57942	0.004421
hsa_circ_	-0.67344	0.004422
hsa_circ_	-0.87378	0.004427
hsa_circ_	0.677497	0.004431
hsa_circ_	0.788316	0.004432
hsa_circ_	0.851122	0.004438
hsa_circ_	0.781043	0.00444
hsa_circ_	1.203282	0.004443
hsa_circ_	-0.68819	0.004444
hsa_circ_	0.655391	0.004445
hsa_circ_	0.816598	0.004448
hsa_circ_	0.842392	0.00445
hsa_circ_	-0.73813	0.004452
hsa_circ_	0.69895	0.004455
hsa_circ_	1.221171	0.004461
hsa_circ_	0.578554	0.00447

hsa_circ_	0.526263	0.004475
hsa_circ_	0.747491	0.004476
hsa_circ_	1.674297	0.004478
hsa_circ_	-0.9	0.004481
hsa_circ_	-1.0353	0.004484
hsa_circ_	0.647256	0.004487
hsa_circ_	-0.69172	0.004487
hsa_circ_	1.075925	0.004489
hsa_circ_	0.894955	0.004492
hsa_circ_	-1.01217	0.004501
hsa_circ_	-0.8584	0.004503
hsa_circ_	-0.74275	0.004522
hsa_circ_	1.000318	0.004523
hsa_circ_	-1.63973	0.004527
hsa_circ_	-1.03804	0.004529
hsa_circ_	-0.88763	0.004536
hsa_circ_	1.342908	0.004538
hsa_circ_	1.864168	0.004544
hsa_circ_	0.781605	0.004555
hsa_circ_	0.573309	0.004559
hsa_circ_	0.703632	0.00456
hsa_circ_	0.580042	0.004568
hsa_circ_	0.775199	0.004578
hsa_circ_	0.578077	0.004578
hsa_circ_	1.203001	0.004579
hsa_circ_	-0.94056	0.004584
hsa_circ_	-0.73104	0.004587
hsa_circ_	1.113801	0.004588
hsa_circ_	0.848161	0.004589
hsa_circ_	0.816098	0.004591
hsa_circ_	-1.79054	0.004594
hsa_circ_	-1.41572	0.004599
hsa_circ_	0.576282	0.0046
hsa_circ_	-0.66852	0.0046
hsa_circ_	1.528272	0.0046
hsa_circ_	-0.59257	0.004606
hsa_circ_	0.582523	0.004608
hsa_circ_	-2.48502	0.004613
hsa_circ_	-1.10626	0.004615
hsa_circ_	-0.71378	0.004615
hsa_circ_	0.892253	0.004618
hsa_circ_	-0.65089	0.004628
hsa_circ_	0.5459	0.004636
hsa_circ_	0.549047	0.004637
hsa_circ_	-1.00294	0.004638
hsa_circ_	-1.11722	0.004638
hsa_circ_	-1.05661	0.004639
hsa_circ_	-0.62093	0.004645
hsa_circ_	0.917523	0.004646
hsa_circ_	-1.67834	0.004646
hsa_circ_	1.810827	0.00465
hsa_circ_	0.641675	0.00466
hsa_circ_	0.681114	0.004662
hsa_circ_	0.763283	0.004672

hsa_circ_	-1.36054	0.004685
hsa_circ_	0.819585	0.004687
hsa_circ_	1.250265	0.004688
hsa_circ_	0.97007	0.004689
hsa_circ_	1.305322	0.004695
hsa_circ_	-1.04377	0.004695
hsa_circ_	1.053394	0.004696
hsa_circ_	-0.5381	0.004697
hsa_circ_	1.002643	0.004697
hsa_circ_	-1.30207	0.004697
hsa_circ_	1.720037	0.0047
hsa_circ_	0.974945	0.004705
hsa_circ_	0.665702	0.004706
hsa_circ_	-1.04271	0.00471
hsa_circ_	-0.68029	0.004712
hsa_circ_	0.82304	0.004715
hsa_circ_	-1.13599	0.004717
hsa_circ_	1.017895	0.004729
hsa_circ_	1.811161	0.004731
hsa_circ_	-1.00022	0.004734
hsa_circ_	0.684164	0.004735
hsa_circ_	2.04505	0.004737
hsa_circ_	-0.8303	0.004739
hsa_circ_	-1.0648	0.004739
hsa_circ_	-0.90579	0.004753
hsa_circ_	-1.32958	0.004753
hsa_circ_	-0.88036	0.004758
hsa_circ_	0.877897	0.004762
hsa_circ_	-0.94336	0.004765
hsa_circ_	-1.37974	0.004768
hsa_circ_	2.281098	0.004769
hsa_circ_	1.316754	0.004774
hsa_circ_	-0.71151	0.004776
hsa_circ_	-0.7605	0.004782
hsa_circ_	-0.5665	0.004785
hsa_circ_	-0.86536	0.004792
hsa_circ_	-1.36391	0.004796
hsa_circ_	0.787456	0.004799
hsa_circ_	-1.08175	0.004801
hsa_circ_	0.721117	0.004805
hsa_circ_	2.033415	0.004815
hsa_circ_	-1.09277	0.004816
hsa_circ_	2.014496	0.004817
hsa_circ_	-0.62326	0.004825
hsa_circ_	0.750847	0.004832
hsa_circ_	-0.69369	0.004832
hsa_circ_	1.150138	0.004834
hsa_circ_	-0.59375	0.004843
hsa_circ_	0.590525	0.004851
hsa_circ_	-0.68482	0.004855
hsa_circ_	-0.6584	0.004857
hsa_circ_	0.764827	0.00487
hsa_circ_	-1.01397	0.004872
hsa_circ_	0.818707	0.004873

hsa_circ_	-1.08059	0.004875
hsa_circ_	-0.63009	0.004881
hsa_circ_	0.687375	0.004885
hsa_circ_	0.74945	0.004894
hsa_circ_	0.948253	0.004894
hsa_circ_	2.128663	0.004905
hsa_circ_	0.633182	0.004905
hsa_circ_	-1.08012	0.004907
hsa_circ_	-0.9738	0.004912
hsa_circ_	-1.05943	0.004913
hsa_circ_	-1.03578	0.004917
hsa_circ_	0.562656	0.004918
hsa_circ_	-1.39857	0.004922
hsa_circ_	-1.49202	0.004924
hsa_circ_	-1.07371	0.004924
hsa_circ_	-0.5259	0.004934
hsa_circ_	1.883949	0.004935
hsa_circ_	-0.64756	0.004936
hsa_circ_	0.546865	0.004938
hsa_circ_	0.703796	0.004946
hsa_circ_	0.796652	0.004948
hsa_circ_	1.101939	0.00495
hsa_circ_	0.705897	0.004952
hsa_circ_	1.371641	0.004955
hsa_circ_	-0.57195	0.004957
hsa_circ_	-1.85599	0.004962
hsa_circ_	-1.26648	0.004964
hsa_circ_	-1.09918	0.004967
hsa_circ_	1.160541	0.004976
hsa_circ_	1.099683	0.004977
hsa_circ_	1.057202	0.004982
hsa_circ_	0.641202	0.004986
hsa_circ_	1.332345	0.004987
hsa_circ_	-0.78559	0.004988
hsa_circ_	-1.03134	0.004999
hsa_circ_	-1.017	0.004999
hsa_circ_	0.623568	0.005003
hsa_circ_	0.896168	0.005003
hsa_circ_	-1.06117	0.005006
hsa_circ_	0.671026	0.005007
hsa_circ_	-1.01958	0.005007
hsa_circ_	0.716304	0.005008
hsa_circ_	0.754345	0.005011
hsa_circ_	0.627619	0.005018
hsa_circ_	-1.01095	0.005021
hsa_circ_	-1.07785	0.005023
hsa_circ_	-0.6829	0.005024
hsa_circ_	0.687281	0.005026
hsa_circ_	-0.65943	0.005032
hsa_circ_	1.117296	0.005035
hsa_circ_	-0.67317	0.005039
hsa_circ_	-1.0362	0.005043
hsa_circ_	-1.04473	0.005047
hsa_circ_	1.182529	0.005047

hsa_circ_	0.858589	0.00507
hsa_circ_	-1.03298	0.005072
hsa_circ_	-1.03831	0.005073
hsa_circ_	0.637945	0.005078
hsa_circ_	-0.7677	0.005079
hsa_circ_	1.837232	0.005082
hsa_circ_	-2.2494	0.005086
hsa_circ_	0.81948	0.005089
hsa_circ_	0.919376	0.00509
hsa_circ_	0.847043	0.005091
hsa_circ_	-0.56304	0.005099
hsa_circ_	-0.54859	0.005102
hsa_circ_	1.134177	0.005102
hsa_circ_	-0.90544	0.005104
hsa_circ_	-1.69353	0.005108
hsa_circ_	1.162727	0.005108
hsa_circ_	0.83937	0.005112
hsa_circ_	-1.53475	0.005114
hsa_circ_	1.066356	0.005116
hsa_circ_	0.934137	0.005121
hsa_circ_	1.035788	0.005122
hsa_circ_	-0.55812	0.005128
hsa_circ_	-1.0142	0.005129
hsa_circ_	-0.5676	0.005131
hsa_circ_	1.103678	0.005132
hsa_circ_	-1.02845	0.005134
hsa_circ_	0.651793	0.005138
hsa_circ_	1.179733	0.005143
hsa_circ_	-1.04123	0.005145
hsa_circ_	0.677577	0.005146
hsa_circ_	-0.86101	0.00515
hsa_circ_	0.759797	0.005152
hsa_circ_	-0.81761	0.005153
hsa_circ_	1.446028	0.005158
hsa_circ_	-1.83042	0.005159
hsa_circ_	0.771383	0.005164
hsa_circ_	0.818655	0.00517
hsa_circ_	0.669112	0.00517
hsa_circ_	1.366774	0.005171
hsa_circ_	0.994413	0.005173
hsa_circ_	1.353038	0.00518
hsa_circ_	1.431532	0.005181
hsa_circ_	0.98646	0.005188
hsa_circ_	1.057917	0.005195
hsa_circ_	0.781553	0.005197
hsa_circ_	-0.82887	0.0052
hsa_circ_	-1.06654	0.005202
hsa_circ_	-1.0236	0.005203
hsa_circ_	-0.76553	0.005209
hsa_circ_	0.852212	0.005209
hsa_circ_	-1.02194	0.005209
hsa_circ_	-1.01678	0.005209
hsa_circ_	-2.19702	0.00521
hsa_circ_	1.482359	0.005217

hsa_circ_	0.667887	0.005218
hsa_circ_	-1.03689	0.005223
hsa_circ_	-0.72926	0.005228
hsa_circ_	-0.99358	0.005232
hsa_circ_	0.595124	0.005233
hsa_circ_	0.941933	0.005236
hsa_circ_	1.642776	0.005237
hsa_circ_	-0.80148	0.005241
hsa_circ_	0.725235	0.005246
hsa_circ_	0.86209	0.005247
hsa_circ_	1.661584	0.005251
hsa_circ_	-0.53774	0.005255
hsa_circ_	-0.62516	0.005256
hsa_circ_	-1.02071	0.005256
hsa_circ_	-0.53135	0.00526
hsa_circ_	0.564036	0.00526
hsa_circ_	0.551398	0.005261
hsa_circ_	-0.82387	0.005261
hsa_circ_	0.879619	0.005264
hsa_circ_	-0.57748	0.005267
hsa_circ_	-1.15509	0.005273
hsa_circ_	0.522337	0.005275
hsa_circ_	0.654161	0.005276
hsa_circ_	-1.28165	0.005277
hsa_circ_	0.753626	0.005278
hsa_circ_	-1.0086	0.005283
hsa_circ_	0.722469	0.005288
hsa_circ_	1.401017	0.005292
hsa_circ_	1.014864	0.005295
hsa_circ_	0.715289	0.0053
hsa_circ_	0.572548	0.005302
hsa_circ_	-1.0257	0.005307
hsa_circ_	-0.74914	0.005308
hsa_circ_	0.903354	0.005312
hsa_circ_	-0.69082	0.005314
hsa_circ_	-1.03146	0.00532
hsa_circ_	0.843325	0.005321
hsa_circ_	-0.57582	0.005329
hsa_circ_	0.843636	0.00533
hsa_circ_	0.589784	0.005334
hsa_circ_	0.959807	0.005336
hsa_circ_	-1.04264	0.005336
hsa_circ_	0.665099	0.005353
hsa_circ_	-0.98632	0.005356
hsa_circ_	-1.05114	0.005357
hsa_circ_	-0.58877	0.005358
hsa_circ_	-0.8443	0.005367
hsa_circ_	1.386685	0.005368
hsa_circ_	1.291347	0.005369
hsa_circ_	-0.57483	0.005383
hsa_circ_	-0.54714	0.00539
hsa_circ_	-0.94238	0.005391
hsa_circ_	-1.01106	0.005393
hsa_circ_	-0.60878	0.005395

hsa_circ_	0.716341	0.005403
hsa_circ_	-1.58376	0.005408
hsa_circ_	1.07254	0.005412
hsa_circ_	-0.66361	0.005415
hsa_circ_	-0.94117	0.005416
hsa_circ_	0.892851	0.005416
hsa_circ_	1.129557	0.005419
hsa_circ_	0.583042	0.005423
hsa_circ_	-1.01497	0.005423
hsa_circ_	-0.77495	0.005424
hsa_circ_	-0.70273	0.005426
hsa_circ_	-0.74128	0.005437
hsa_circ_	-0.7358	0.005438
hsa_circ_	-0.53975	0.005448
hsa_circ_	1.44652	0.005453
hsa_circ_	0.961254	0.005454
hsa_circ_	0.816449	0.005459
hsa_circ_	0.884653	0.005465
hsa_circ_	-2.29536	0.005465
hsa_circ_	1.360404	0.00547
hsa_circ_	-2.22556	0.005471
hsa_circ_	0.655397	0.005486
hsa_circ_	-1.13877	0.005494
hsa_circ_	0.654574	0.005499
hsa_circ_	-0.54727	0.005502
hsa_circ_	1.247701	0.005506
hsa_circ_	-0.67971	0.005511
hsa_circ_	-0.68693	0.005513
hsa_circ_	0.566311	0.005513
hsa_circ_	-0.91815	0.005515
hsa_circ_	-1.36418	0.005518
hsa_circ_	-0.73801	0.005523
hsa_circ_	0.598188	0.005525
hsa_circ_	-0.84273	0.005533
hsa_circ_	-0.74586	0.005534
hsa_circ_	1.172172	0.00554
hsa_circ_	-1.24582	0.005547
hsa_circ_	0.50975	0.005549
hsa_circ_	0.935268	0.005557
hsa_circ_	-1.01745	0.005557
hsa_circ_	0.980822	0.005562
hsa_circ_	1.19316	0.005563
hsa_circ_	-0.67982	0.005567
hsa_circ_	0.765881	0.005567
hsa_circ_	-1.00983	0.005568
hsa_circ_	-1.11493	0.005572
hsa_circ_	1.242496	0.005577
hsa_circ_	-1.00952	0.00558
hsa_circ_	0.704343	0.005582
hsa_circ_	-1.28039	0.005594
hsa_circ_	0.872177	0.005599
hsa_circ_	-1.01175	0.005604
hsa_circ_	-0.98926	0.005604
hsa_circ_	0.565036	0.005607

hsa_circ_	0.586093	0.005616
hsa_circ_	1.495679	0.005617
hsa_circ_	1.374186	0.005618
hsa_circ_	0.558499	0.005619
hsa_circ_	1.132003	0.005629
hsa_circ_	-1.11747	0.005629
hsa_circ_	0.605171	0.00563
hsa_circ_	-1.06031	0.00564
hsa_circ_	0.898153	0.00564
hsa_circ_	-0.6962	0.005641
hsa_circ_	1.659067	0.005642
hsa_circ_	0.88475	0.005646
hsa_circ_	1.077777	0.005648
hsa_circ_	-0.58277	0.005649
hsa_circ_	-0.99654	0.00565
hsa_circ_	-0.66327	0.005656
hsa_circ_	0.574981	0.005668
hsa_circ_	-1.24995	0.005668
hsa_circ_	0.887975	0.005669
hsa_circ_	0.819842	0.005674
hsa_circ_	-0.68649	0.005675
hsa_circ_	0.539452	0.005678
hsa_circ_	0.63443	0.005686
hsa_circ_	0.980582	0.005689
hsa_circ_	1.61353	0.005694
hsa_circ_	-0.695	0.005697
hsa_circ_	1.377551	0.005697
hsa_circ_	-0.58749	0.005703
hsa_circ_	0.668786	0.005708
hsa_circ_	-0.57754	0.005711
hsa_circ_	1.083414	0.005713
hsa_circ_	0.60194	0.005718
hsa_circ_	1.342361	0.005725
hsa_circ_	-0.73138	0.005728
hsa_circ_	0.881507	0.005728
hsa_circ_	-0.98151	0.005729
hsa_circ_	1.255613	0.005736
hsa_circ_	-0.98443	0.005738
hsa_circ_	-0.68237	0.005739
hsa_circ_	1.134673	0.005739
hsa_circ_	-0.98307	0.005747
hsa_circ_	-0.61068	0.005751
hsa_circ_	-1.03749	0.005763
hsa_circ_	-0.91614	0.005764
hsa_circ_	1.122379	0.005764
hsa_circ_	0.552034	0.00577
hsa_circ_	1.032007	0.005773
hsa_circ_	0.893918	0.005777
hsa_circ_	0.621821	0.005778
hsa_circ_	0.777583	0.005784
hsa_circ_	-1.19215	0.005787
hsa_circ_	0.861328	0.005788
hsa_circ_	0.784358	0.005789
hsa_circ_	0.936157	0.005795

hsa_circ_	1.311548	0.005804
hsa_circ_	0.998436	0.005808
hsa_circ_	0.566503	0.005811
hsa_circ_	-0.99	0.005811
hsa_circ_	-1.00944	0.005815
hsa_circ_	1.025129	0.005816
hsa_circ_	0.89119	0.00582
hsa_circ_	1.145086	0.005828
hsa_circ_	0.95025	0.00583
hsa_circ_	-1.03424	0.00584
hsa_circ_	1.04782	0.005842
hsa_circ_	1.312168	0.005844
hsa_circ_	1.765966	0.005847
hsa_circ_	0.698839	0.005854
hsa_circ_	-0.66258	0.005862
hsa_circ_	0.647996	0.005866
hsa_circ_	0.786071	0.005868
hsa_circ_	0.563013	0.00587
hsa_circ_	-0.59505	0.005872
hsa_circ_	0.648386	0.005873
hsa_circ_	1.364121	0.005874
hsa_circ_	-0.67061	0.005875
hsa_circ_	1.587847	0.005879
hsa_circ_	-0.52782	0.00588
hsa_circ_	1.097373	0.005881
hsa_circ_	-1.35097	0.005894
hsa_circ_	1.698741	0.005899
hsa_circ_	1.042525	0.005902
hsa_circ_	1.15775	0.005905
hsa_circ_	0.790334	0.00591
hsa_circ_	0.965193	0.005911
hsa_circ_	0.755217	0.005911
hsa_circ_	-1.09873	0.005913
hsa_circ_	-0.53412	0.005916
hsa_circ_	-1.4402	0.005919
hsa_circ_	-0.67764	0.005928
hsa_circ_	0.994423	0.005931
hsa_circ_	1.120601	0.005932
hsa_circ_	0.674245	0.005934
hsa_circ_	0.601419	0.005936
hsa_circ_	-0.90039	0.005942
hsa_circ_	1.274067	0.005943
hsa_circ_	0.622231	0.005944
hsa_circ_	0.526494	0.005948
hsa_circ_	-0.81744	0.005951
hsa_circ_	-0.59842	0.005954
hsa_circ_	1.20177	0.005956
hsa_circ_	-1.40958	0.005966
hsa_circ_	0.778452	0.005967
hsa_circ_	0.718287	0.005971
hsa_circ_	1.474351	0.005972
hsa_circ_	0.753889	0.005977
hsa_circ_	-0.58432	0.005979
hsa_circ_	0.982117	0.005982

hsa_circ_	-0.54781	0.005983
hsa_circ_	-0.95282	0.005984
hsa_circ_	-0.64045	0.00599
hsa_circ_	1.067032	0.005995
hsa_circ_	1.363235	0.005998
hsa_circ_	0.911927	0.005999
hsa_circ_	0.917932	0.006001
hsa_circ_	-0.62319	0.006008
hsa_circ_	0.657624	0.00601
hsa_circ_	-1.00335	0.006018
hsa_circ_	-1.04953	0.006018
hsa_circ_	0.603061	0.006019
hsa_circ_	0.71815	0.006022
hsa_circ_	-0.62699	0.006022
hsa_circ_	1.412081	0.006023
hsa_circ_	-0.80072	0.006025
hsa_circ_	-0.92333	0.006025
hsa_circ_	-0.55603	0.006027
hsa_circ_	-0.98073	0.006029
hsa_circ_	-0.60775	0.006031
hsa_circ_	-0.74516	0.006041
hsa_circ_	0.613889	0.006043
hsa_circ_	0.881938	0.006051
hsa_circ_	0.672162	0.006052
hsa_circ_	1.311347	0.006055
hsa_circ_	1.245566	0.006079
hsa_circ_	1.551471	0.006082
hsa_circ_	1.323902	0.006084
hsa_circ_	1.066162	0.006089
hsa_circ_	-0.518	0.006091
hsa_circ_	-1.03728	0.006093
hsa_circ_	-0.76266	0.006094
hsa_circ_	-0.6022	0.006097
hsa_circ_	-0.88918	0.006098
hsa_circ_	-0.60274	0.006099
hsa_circ_	-1.09064	0.006105
hsa_circ_	2.251508	0.006112
hsa_circ_	1.388186	0.006118
hsa_circ_	0.802081	0.006122
hsa_circ_	-1.26921	0.006126
hsa_circ_	-1.07856	0.006127
hsa_circ_	0.668608	0.00613
hsa_circ_	0.658454	0.00613
hsa_circ_	-1.26554	0.006132
hsa_circ_	-1.14643	0.006133
hsa_circ_	-0.52194	0.006134
hsa_circ_	-0.61192	0.006138
hsa_circ_	-0.55474	0.006138
hsa_circ_	1.361169	0.006138
hsa_circ_	-1.16346	0.006139
hsa_circ_	0.8556	0.00614
hsa_circ_	0.586939	0.006144
hsa_circ_	-0.62185	0.006147
hsa_circ_	0.878299	0.006151

hsa_circ_	0.590274	0.006153
hsa_circ_	-0.99854	0.006158
hsa_circ_	1.101337	0.006162
hsa_circ_	-0.80321	0.006166
hsa_circ_	-1.73472	0.00617
hsa_circ_	-0.83459	0.006171
hsa_circ_	1.545397	0.006172
hsa_circ_	1.982344	0.006174
hsa_circ_	0.897181	0.006178
hsa_circ_	1.006846	0.006178
hsa_circ_	-2.36214	0.006179
hsa_circ_	0.984942	0.006185
hsa_circ_	-0.92371	0.006188
hsa_circ_	-0.93261	0.006189
hsa_circ_	-0.86492	0.00619
hsa_circ_	0.681507	0.00619
hsa_circ_	0.986391	0.006191
hsa_circ_	-1.29377	0.006195
hsa_circ_	0.52038	0.006196
hsa_circ_	0.664257	0.006197
hsa_circ_	-1.08401	0.006204
hsa_circ_	1.988708	0.006207
hsa_circ_	0.523825	0.006217
hsa_circ_	1.065086	0.006225
hsa_circ_	0.763471	0.006237
hsa_circ_	-0.63123	0.006244
hsa_circ_	-1.29903	0.006245
hsa_circ_	0.818361	0.006247
hsa_circ_	-1.20731	0.006255
hsa_circ_	-1.76681	0.00626
hsa_circ_	-1.63398	0.006262
hsa_circ_	0.773359	0.006263
hsa_circ_	-0.50808	0.006266
hsa_circ_	0.908507	0.006267
hsa_circ_	0.514239	0.006274
hsa_circ_	-1.34919	0.006279
hsa_circ_	-1.61011	0.006295
hsa_circ_	-0.99248	0.006298
hsa_circ_	-0.7415	0.0063
hsa_circ_	-0.99723	0.006307
hsa_circ_	0.844391	0.006308
hsa_circ_	1.303178	0.006318
hsa_circ_	0.935941	0.006321
hsa_circ_	0.576973	0.006326
hsa_circ_	0.808386	0.006331
hsa_circ_	-0.80891	0.006331
hsa_circ_	-0.54541	0.006332
hsa_circ_	1.221294	0.006338
hsa_circ_	0.541077	0.006339
hsa_circ_	-0.59585	0.006339
hsa_circ_	-1.17749	0.006347
hsa_circ_	1.738471	0.006364
hsa_circ_	-0.93093	0.006364
hsa_circ_	-0.96985	0.006366

hsa_circ_	-0.98096	0.006371
hsa_circ_	1.116404	0.006377
hsa_circ_	-0.63208	0.006383
hsa_circ_	0.578539	0.006384
hsa_circ_	-1.34073	0.006387
hsa_circ_	-0.78429	0.006389
hsa_circ_	1.487444	0.006397
hsa_circ_	-0.63188	0.006397
hsa_circ_	-0.89583	0.006397
hsa_circ_	-0.87034	0.0064
hsa_circ_	0.574211	0.0064
hsa_circ_	1.759235	0.006404
hsa_circ_	0.897393	0.006406
hsa_circ_	1.694729	0.006409
hsa_circ_	-1.11482	0.006411
hsa_circ_	0.578681	0.006412
hsa_circ_	-1.01502	0.006413
hsa_circ_	0.945127	0.006413
hsa_circ_	-1.00848	0.00642
hsa_circ_	0.520732	0.006426
hsa_circ_	1.203707	0.006431
hsa_circ_	0.605634	0.006436
hsa_circ_	-0.97115	0.006438
hsa_circ_	-0.9218	0.006441
hsa_circ_	-0.50899	0.006442
hsa_circ_	0.54989	0.006453
hsa_circ_	0.758398	0.006455
hsa_circ_	-0.97466	0.006459
hsa_circ_	0.727639	0.006475
hsa_circ_	1.07356	0.006476
hsa_circ_	1.743552	0.006487
hsa_circ_	1.161258	0.006495
hsa_circ_	0.504099	0.0065
hsa_circ_	0.612374	0.006502
hsa_circ_	1.574995	0.006507
hsa_circ_	0.972732	0.006508
hsa_circ_	1.055747	0.006512
hsa_circ_	1.088103	0.006516
hsa_circ_	-0.5613	0.006516
hsa_circ_	-0.74955	0.006519
hsa_circ_	0.625332	0.006519
hsa_circ_	1.074801	0.00652
hsa_circ_	0.853471	0.006523
hsa_circ_	-1.08114	0.006524
hsa_circ_	-0.71391	0.006536
hsa_circ_	0.513239	0.006539
hsa_circ_	-0.9797	0.006539
hsa_circ_	1.103792	0.00654
hsa_circ_	-0.5865	0.006548
hsa_circ_	0.682127	0.006549
hsa_circ_	1.420914	0.006551
hsa_circ_	-0.79645	0.006552
hsa_circ_	-0.53902	0.006556
hsa_circ_	0.562717	0.006557

hsa_circ_	1.050215	0.006561
hsa_circ_	-1.01909	0.006566
hsa_circ_	-0.62826	0.006566
hsa_circ_	-0.75129	0.006568
hsa_circ_	0.803337	0.006577
hsa_circ_	-1.06362	0.006577
hsa_circ_	1.438641	0.006582
hsa_circ_	-1.3301	0.006582
hsa_circ_	-0.51022	0.006585
hsa_circ_	-0.84397	0.006591
hsa_circ_	-0.63949	0.006593
hsa_circ_	-0.77401	0.006594
hsa_circ_	-1.02582	0.006603
hsa_circ_	0.646303	0.006604
hsa_circ_	0.526254	0.006605
hsa_circ_	0.519345	0.006606
hsa_circ_	0.626289	0.006609
hsa_circ_	-1.05789	0.006621
hsa_circ_	-0.6263	0.006623
hsa_circ_	-1.3449	0.006624
hsa_circ_	1.717806	0.006627
hsa_circ_	-0.57442	0.006629
hsa_circ_	-1.01207	0.006633
hsa_circ_	-0.71951	0.006633
hsa_circ_	1.253458	0.006635
hsa_circ_	0.663692	0.006638
hsa_circ_	-1.34122	0.006639
hsa_circ_	0.676824	0.006654
hsa_circ_	-1.02416	0.006656
hsa_circ_	-0.59601	0.006663
hsa_circ_	-1.04563	0.006664
hsa_circ_	-0.91901	0.006664
hsa_circ_	1.810814	0.006667
hsa_circ_	-1.07487	0.006676
hsa_circ_	0.848674	0.006678
hsa_circ_	-0.98085	0.006681
hsa_circ_	0.900168	0.006682
hsa_circ_	0.571587	0.006683
hsa_circ_	1.780872	0.006689
hsa_circ_	0.939364	0.006698
hsa_circ_	-0.51533	0.006698
hsa_circ_	0.796524	0.006701
hsa_circ_	1.240422	0.006702
hsa_circ_	0.638921	0.006703
hsa_circ_	1.342555	0.006717
hsa_circ_	0.649327	0.00672
hsa_circ_	1.263083	0.00672
hsa_circ_	0.52019	0.006724
hsa_circ_	-0.68957	0.006725
hsa_circ_	-0.95842	0.006731
hsa_circ_	-1.11042	0.006731
hsa_circ_	0.669736	0.006735
hsa_circ_	1.133238	0.006738
hsa_circ_	-1.07691	0.00674

hsa_circ_	-0.73012	0.006744
hsa_circ_	-1.58846	0.006747
hsa_circ_	1.435619	0.006748
hsa_circ_	-0.96557	0.006749
hsa_circ_	0.501509	0.006751
hsa_circ_	0.615248	0.006759
hsa_circ_	-0.97644	0.006762
hsa_circ_	0.550626	0.006766
hsa_circ_	-0.53546	0.006767
hsa_circ_	-0.63296	0.006769
hsa_circ_	-0.76136	0.00677
hsa_circ_	1.821165	0.006777
hsa_circ_	1.611561	0.006778
hsa_circ_	0.686613	0.006778
hsa_circ_	0.831315	0.006782
hsa_circ_	-0.99448	0.00679
hsa_circ_	0.798252	0.0068
hsa_circ_	-0.70602	0.006811
hsa_circ_	-0.68451	0.006811
hsa_circ_	0.778305	0.006815
hsa_circ_	-1.07933	0.006815
hsa_circ_	-0.86493	0.006818
hsa_circ_	-0.9609	0.006818
hsa_circ_	-0.98342	0.006822
hsa_circ_	-0.78316	0.006823
hsa_circ_	-0.50473	0.006825
hsa_circ_	0.930354	0.006826
hsa_circ_	-1.17391	0.006828
hsa_circ_	0.664446	0.006828
hsa_circ_	0.636378	0.006828
hsa_circ_	0.851792	0.006828
hsa_circ_	1.039081	0.006829
hsa_circ_	0.582622	0.006831
hsa_circ_	1.364376	0.006832
hsa_circ_	0.508227	0.006837
hsa_circ_	1.538405	0.00684
hsa_circ_	-1.50097	0.006844
hsa_circ_	0.623206	0.006844
hsa_circ_	0.524492	0.006846
hsa_circ_	0.880478	0.006851
hsa_circ_	0.657068	0.006861
hsa_circ_	-0.82936	0.006863
hsa_circ_	-0.55058	0.006865
hsa_circ_	-0.76046	0.006873
hsa_circ_	0.537299	0.006881
hsa_circ_	-1.22451	0.006881
hsa_circ_	1.743472	0.006886
hsa_circ_	-1.05355	0.00689
hsa_circ_	-0.9846	0.00689
hsa_circ_	1.355083	0.006892
hsa_circ_	-1.25326	0.006893
hsa_circ_	-0.82334	0.006895
hsa_circ_	0.99072	0.0069
hsa_circ_	-0.69144	0.006902

hsa_circ_	0.705376	0.006911
hsa_circ_	-0.53596	0.006912
hsa_circ_	-0.76065	0.006921
hsa_circ_	-0.70466	0.006923
hsa_circ_	0.728038	0.006924
hsa_circ_	1.743446	0.006927
hsa_circ_	0.722215	0.006928
hsa_circ_	-0.54516	0.006932
hsa_circ_	-0.59131	0.006935
hsa_circ_	0.605301	0.006936
hsa_circ_	-0.99809	0.006937
hsa_circ_	0.883118	0.006943
hsa_circ_	1.056946	0.006947
hsa_circ_	0.602284	0.00695
hsa_circ_	-0.81968	0.006951
hsa_circ_	0.636361	0.006951
hsa_circ_	-1.26252	0.006954
hsa_circ_	0.502851	0.006957
hsa_circ_	1.585997	0.00696
hsa_circ_	-0.55764	0.00696
hsa_circ_	0.809883	0.006963
hsa_circ_	-0.98422	0.006964
hsa_circ_	-0.89896	0.006966
hsa_circ_	-0.95325	0.006966
hsa_circ_	1.037132	0.006968
hsa_circ_	1.523016	0.006989
hsa_circ_	0.502945	0.006989
hsa_circ_	0.871433	0.00699
hsa_circ_	0.876176	0.006993
hsa_circ_	-0.96834	0.006994
hsa_circ_	0.751698	0.006998
hsa_circ_	-0.50805	0.007001
hsa_circ_	0.557371	0.007005
hsa_circ_	-0.95555	0.007006
hsa_circ_	-0.83774	0.007022
hsa_circ_	-0.58635	0.007023
hsa_circ_	1.07471	0.007025
hsa_circ_	-0.95736	0.007026
hsa_circ_	0.741262	0.007033
hsa_circ_	1.48371	0.007033
hsa_circ_	-0.60069	0.007039
hsa_circ_	0.839542	0.007047
hsa_circ_	-0.8425	0.007051
hsa_circ_	1.247252	0.007051
hsa_circ_	0.950336	0.007056
hsa_circ_	-0.52902	0.007064
hsa_circ_	0.746983	0.007068
hsa_circ_	0.499769	0.007071
hsa_circ_	-0.73684	0.007073
hsa_circ_	-1.01172	0.007078
hsa_circ_	-0.91865	0.007088
hsa_circ_	2.42388	0.00709
hsa_circ_	0.977052	0.007091
hsa_circ_	-0.87606	0.007096

hsa_circ_	-0.70834	0.007097
hsa_circ_	-0.69599	0.007098
hsa_circ_	-0.68527	0.0071
hsa_circ_	-0.96921	0.007105
hsa_circ_	1.403266	0.007106
hsa_circ_	0.855135	0.007109
hsa_circ_	-1.10236	0.00711
hsa_circ_	-0.46704	0.007113
hsa_circ_	-0.87386	0.007125
hsa_circ_	-0.52324	0.007127
hsa_circ_	1.122134	0.007137
hsa_circ_	-0.54958	0.00714
hsa_circ_	1.148881	0.007141
hsa_circ_	0.846535	0.00715
hsa_circ_	0.571853	0.007156
hsa_circ_	-1.07273	0.007156
hsa_circ_	-0.56237	0.007161
hsa_circ_	-1.0541	0.007176
hsa_circ_	-1.23339	0.007177
hsa_circ_	0.877808	0.00719
hsa_circ_	-0.95836	0.007203
hsa_circ_	-0.81652	0.007207
hsa_circ_	-1.06026	0.007219
hsa_circ_	-1.0643	0.007219
hsa_circ_	-1.14759	0.007223
hsa_circ_	-1.20162	0.007234
hsa_circ_	0.632205	0.007236
hsa_circ_	-0.51095	0.007243
hsa_circ_	1.199871	0.007254
hsa_circ_	-0.93808	0.00726
hsa_circ_	0.804814	0.007276
hsa_circ_	-0.72726	0.007279
hsa_circ_	0.877794	0.00728
hsa_circ_	0.782538	0.007291
hsa_circ_	0.664894	0.007292
hsa_circ_	0.791716	0.007293
hsa_circ_	0.70084	0.007296
hsa_circ_	0.623342	0.007298
hsa_circ_	-1.06606	0.007301
hsa_circ_	0.568891	0.007303
hsa_circ_	0.683991	0.007305
hsa_circ_	0.544816	0.007305
hsa_circ_	1.649199	0.007308
hsa_circ_	-0.50487	0.007318
hsa_circ_	0.752613	0.007318
hsa_circ_	-1.21929	0.007322
hsa_circ_	-0.53447	0.007323
hsa_circ_	0.710496	0.007324
hsa_circ_	-0.95342	0.007325
hsa_circ_	0.729094	0.007326
hsa_circ_	0.823113	0.007331
hsa_circ_	-0.64793	0.007335
hsa_circ_	-0.74139	0.007348
hsa_circ_	0.640847	0.00735

hsa_circ_	1.106408	0.007351
hsa_circ_	-1.25047	0.007356
hsa_circ_	0.696304	0.00736
hsa_circ_	-0.88551	0.007361
hsa_circ_	1.6636	0.007361
hsa_circ_	-0.99526	0.007365
hsa_circ_	-0.63975	0.007366
hsa_circ_	-0.68468	0.007369
hsa_circ_	-0.48079	0.007369
hsa_circ_	0.889434	0.007371
hsa_circ_	-1.10758	0.007379
hsa_circ_	0.874965	0.007385
hsa_circ_	0.657837	0.007386
hsa_circ_	0.540112	0.007386
hsa_circ_	-1.21478	0.007388
hsa_circ_	-0.7803	0.007397
hsa_circ_	-1.0661	0.007402
hsa_circ_	0.947553	0.007403
hsa_circ_	-0.64598	0.007409
hsa_circ_	-1.34917	0.007424
hsa_circ_	-0.95083	0.007426
hsa_circ_	0.789172	0.007433
hsa_circ_	0.990276	0.007435
hsa_circ_	2.83237	0.007438
hsa_circ_	0.777575	0.00744
hsa_circ_	0.736013	0.007441
hsa_circ_	0.609601	0.007445
hsa_circ_	0.713296	0.007446
hsa_circ_	0.526322	0.007449
hsa_circ_	1.88341	0.007455
hsa_circ_	0.724567	0.007457
hsa_circ_	-0.95	0.007463
hsa_circ_	0.824703	0.007468
hsa_circ_	0.705959	0.007474
hsa_circ_	1.583115	0.007474
hsa_circ_	2.52478	0.007479
hsa_circ_	0.884128	0.00748
hsa_circ_	0.923545	0.007481
hsa_circ_	-1.0273	0.007484
hsa_circ_	0.957908	0.007487
hsa_circ_	1.318564	0.007487
hsa_circ_	0.688183	0.007488
hsa_circ_	0.775149	0.007492
hsa_circ_	0.962111	0.007497
hsa_circ_	2.035684	0.007504
hsa_circ_	0.606855	0.007506
hsa_circ_	-0.96058	0.00751
hsa_circ_	1.187568	0.007513
hsa_circ_	0.512117	0.007513
hsa_circ_	-1.38815	0.00752
hsa_circ_	0.597126	0.007521
hsa_circ_	-2.03143	0.007524
hsa_circ_	-1.19649	0.007535
hsa_circ_	1.79114	0.007536

hsa_circ_	0.576717	0.007538
hsa_circ_	1.607256	0.007539
hsa_circ_	-0.97669	0.007543
hsa_circ_	1.115231	0.007545
hsa_circ_	0.906391	0.007547
hsa_circ_	-0.83242	0.007558
hsa_circ_	-1.05888	0.00756
hsa_circ_	-1.39658	0.007564
hsa_circ_	0.972303	0.007566
hsa_circ_	1.345297	0.007567
hsa_circ_	-1.32552	0.007586
hsa_circ_	0.93674	0.007606
hsa_circ_	1.208297	0.007607
hsa_circ_	0.568314	0.00761
hsa_circ_	-0.8584	0.007618
hsa_circ_	-0.58696	0.007618
hsa_circ_	-0.63068	0.007619
hsa_circ_	-0.97113	0.00762
hsa_circ_	-0.65232	0.007622
hsa_circ_	-0.71732	0.007624
hsa_circ_	-0.85826	0.007629
hsa_circ_	-0.97564	0.007643
hsa_circ_	-1.02619	0.007647
hsa_circ_	-0.57466	0.00766
hsa_circ_	0.9731	0.00766
hsa_circ_	-0.93894	0.007661
hsa_circ_	1.041527	0.007678
hsa_circ_	0.584169	0.007688
hsa_circ_	0.478815	0.007688
hsa_circ_	0.796881	0.007689
hsa_circ_	-0.72366	0.007689
hsa_circ_	-0.71165	0.007689
hsa_circ_	-0.83987	0.007692
hsa_circ_	1.841724	0.007695
hsa_circ_	-1.1985	0.007696
hsa_circ_	0.578317	0.007697
hsa_circ_	-0.75823	0.007698
hsa_circ_	0.738019	0.007708
hsa_circ_	0.944933	0.00771
hsa_circ_	-0.56693	0.007712
hsa_circ_	-1.11091	0.007717
hsa_circ_	-0.74738	0.007721
hsa_circ_	-0.80393	0.007723
hsa_circ_	0.624178	0.007728
hsa_circ_	-0.54456	0.007743
hsa_circ_	-0.63881	0.007747
hsa_circ_	1.416994	0.007748
hsa_circ_	0.920448	0.007749
hsa_circ_	-1.03244	0.007751
hsa_circ_	0.688763	0.00776
hsa_circ_	1.720792	0.007765
hsa_circ_	-0.96541	0.007767
hsa_circ_	-0.52355	0.007768
hsa_circ_	0.944512	0.007769

hsa_circ_	-0.84981	0.007779
hsa_circ_	-1.14127	0.00778
hsa_circ_	1.11948	0.00778
hsa_circ_	1.274952	0.007781
hsa_circ_	-0.92837	0.007781
hsa_circ_	-0.60459	0.007783
hsa_circ_	-0.58071	0.007784
hsa_circ_	0.951056	0.007792
hsa_circ_	-1.06017	0.007793
hsa_circ_	-1.02979	0.007798
hsa_circ_	0.550851	0.0078
hsa_circ_	0.740059	0.007801
hsa_circ_	0.523393	0.007806
hsa_circ_	-0.69061	0.007814
hsa_circ_	-0.58457	0.007816
hsa_circ_	-0.54515	0.007818
hsa_circ_	0.474529	0.007819
hsa_circ_	0.608426	0.007819
hsa_circ_	-0.48814	0.007819
hsa_circ_	-0.7045	0.007826
hsa_circ_	2.002746	0.007829
hsa_circ_	1.112148	0.00783
hsa_circ_	-0.56008	0.007836
hsa_circ_	-0.71354	0.007836
hsa_circ_	0.570859	0.007844
hsa_circ_	0.681798	0.00785
hsa_circ_	-0.94823	0.007851
hsa_circ_	-0.60611	0.007855
hsa_circ_	1.40495	0.007861
hsa_circ_	-0.5802	0.007875
hsa_circ_	-0.64553	0.007876
hsa_circ_	1.215017	0.007882
hsa_circ_	-1.01512	0.007893
hsa_circ_	-1.95638	0.007894
hsa_circ_	0.754177	0.007902
hsa_circ_	-0.76726	0.007904
hsa_circ_	-0.808	0.007915
hsa_circ_	-0.94849	0.00792
hsa_circ_	-0.76497	0.007921
hsa_circ_	-0.67196	0.007923
hsa_circ_	0.96377	0.007923
hsa_circ_	1.002928	0.007932
hsa_circ_	1.316539	0.007933
hsa_circ_	1.210708	0.007938
hsa_circ_	0.621486	0.007946
hsa_circ_	0.719636	0.007955
hsa_circ_	-0.61306	0.007956
hsa_circ_	-0.53062	0.007968
hsa_circ_	-1.0021	0.007984
hsa_circ_	-0.99944	0.007984
hsa_circ_	-1.21691	0.007988
hsa_circ_	0.626856	0.008
hsa_circ_	-0.58792	0.008011
hsa_circ_	-0.60566	0.008026

hsa_circ_	0.534124	0.008026
hsa_circ_	-0.91392	0.008035
hsa_circ_	-1.20066	0.008036
hsa_circ_	0.8047	0.008038
hsa_circ_	-0.60648	0.008044
hsa_circ_	-0.99743	0.008052
hsa_circ_	0.838658	0.008053
hsa_circ_	-1.4832	0.008058
hsa_circ_	-0.798	0.008068
hsa_circ_	0.83498	0.00807
hsa_circ_	1.399028	0.008073
hsa_circ_	0.811102	0.00808
hsa_circ_	-0.97333	0.008082
hsa_circ_	0.558539	0.008082
hsa_circ_	0.842431	0.008088
hsa_circ_	-0.51512	0.008089
hsa_circ_	-1.16606	0.00809
hsa_circ_	0.842141	0.008098
hsa_circ_	1.146892	0.008099
hsa_circ_	-0.81535	0.0081
hsa_circ_	-1.01605	0.008101
hsa_circ_	0.613982	0.008109
hsa_circ_	0.657752	0.00811
hsa_circ_	0.549687	0.008122
hsa_circ_	-0.7019	0.008129
hsa_circ_	-1.16681	0.00813
hsa_circ_	1.205055	0.00813
hsa_circ_	0.623847	0.008131
hsa_circ_	0.721423	0.008134
hsa_circ_	0.621779	0.008137
hsa_circ_	-0.86021	0.008137
hsa_circ_	-0.79539	0.008142
hsa_circ_	1.456608	0.008142
hsa_circ_	-0.49673	0.008145
hsa_circ_	0.743282	0.008147
hsa_circ_	-1.25796	0.008147
hsa_circ_	0.679198	0.008147
hsa_circ_	0.483923	0.008149
hsa_circ_	1.017963	0.008149
hsa_circ_	-0.93192	0.008154
hsa_circ_	1.219709	0.00816
hsa_circ_	-0.67006	0.008164
hsa_circ_	0.504363	0.008179
hsa_circ_	-1.25389	0.008183
hsa_circ_	0.583899	0.008184
hsa_circ_	1.798416	0.008184
hsa_circ_	-0.61988	0.008195
hsa_circ_	0.52932	0.008195
hsa_circ_	0.868907	0.008196
hsa_circ_	-0.76869	0.008208
hsa_circ_	1.012442	0.00821
hsa_circ_	-0.77937	0.008214
hsa_circ_	-0.89772	0.008214
hsa_circ_	-0.53065	0.008216

hsa_circ_	-0.91856	0.008222
hsa_circ_	1.037339	0.008228
hsa_circ_	0.583639	0.008232
hsa_circ_	0.794459	0.008235
hsa_circ_	-0.87191	0.008243
hsa_circ_	-0.51529	0.008244
hsa_circ_	0.546237	0.00825
hsa_circ_	0.885919	0.008251
hsa_circ_	0.762914	0.008256
hsa_circ_	-0.51788	0.008264
hsa_circ_	0.683313	0.008268
hsa_circ_	-0.7545	0.00827
hsa_circ_	1.154201	0.008271
hsa_circ_	1.123318	0.008275
hsa_circ_	0.679934	0.008284
hsa_circ_	-0.89658	0.008286
hsa_circ_	-0.68177	0.008293
hsa_circ_	-1.2009	0.008299
hsa_circ_	-1.12141	0.008305
hsa_circ_	-1.02936	0.00831
hsa_circ_	-0.91638	0.008321
hsa_circ_	-0.56706	0.008332
hsa_circ_	0.962749	0.008335
hsa_circ_	0.928191	0.008335
hsa_circ_	0.714763	0.008338
hsa_circ_	1.16119	0.00835
hsa_circ_	0.868437	0.008359
hsa_circ_	1.4097	0.008361
hsa_circ_	1.235663	0.008361
hsa_circ_	0.536749	0.008364
hsa_circ_	-0.84069	0.008367
hsa_circ_	-0.58506	0.00837
hsa_circ_	-0.94214	0.008377
hsa_circ_	-0.82952	0.008377
hsa_circ_	0.489835	0.008381
hsa_circ_	0.502321	0.008383
hsa_circ_	-1.0714	0.008388
hsa_circ_	-0.54663	0.008397
hsa_circ_	-0.71471	0.0084
hsa_circ_	1.023631	0.008404
hsa_circ_	-0.98466	0.008406
hsa_circ_	0.889318	0.008414
hsa_circ_	0.609443	0.008418
hsa_circ_	0.812877	0.008423
hsa_circ_	1.116577	0.008429
hsa_circ_	-1.10585	0.008432
hsa_circ_	1.004841	0.008434
hsa_circ_	0.531952	0.00844
hsa_circ_	0.683026	0.008446
hsa_circ_	0.764529	0.008446
hsa_circ_	0.78212	0.00845
hsa_circ_	0.462976	0.00845
hsa_circ_	-0.96707	0.008452
hsa_circ_	0.657448	0.008453

hsa_circ_	0.969629	0.008453
hsa_circ_	-0.61276	0.008456
hsa_circ_	0.730658	0.008466
hsa_circ_	-0.58019	0.008467
hsa_circ_	0.627616	0.008474
hsa_circ_	-0.74148	0.008475
hsa_circ_	1.466643	0.008475
hsa_circ_	0.761282	0.008476
hsa_circ_	-0.7261	0.008481
hsa_circ_	0.914725	0.008485
hsa_circ_	0.757772	0.008489
hsa_circ_	0.941153	0.008492
hsa_circ_	-0.92447	0.008497
hsa_circ_	0.495904	0.008515
hsa_circ_	-0.97916	0.008515
hsa_circ_	-0.55473	0.008515
hsa_circ_	2.150208	0.008523
hsa_circ_	0.973924	0.008538
hsa_circ_	1.430453	0.008542
hsa_circ_	0.947972	0.008545
hsa_circ_	0.920046	0.008551
hsa_circ_	-0.56958	0.008556
hsa_circ_	-1.27915	0.008558
hsa_circ_	-0.716	0.008558
hsa_circ_	-0.95391	0.008559
hsa_circ_	-0.8874	0.008561
hsa_circ_	-0.7934	0.008562
hsa_circ_	-0.50241	0.008563
hsa_circ_	-0.951	0.008573
hsa_circ_	0.839298	0.00858
hsa_circ_	0.458435	0.008581
hsa_circ_	2.448742	0.008581
hsa_circ_	1.123395	0.008582
hsa_circ_	0.552135	0.008585
hsa_circ_	-1.8178	0.008587
hsa_circ_	-0.63758	0.008589
hsa_circ_	0.521673	0.008589
hsa_circ_	-0.57891	0.008599
hsa_circ_	1.1496	0.0086
hsa_circ_	-1.03686	0.008602
hsa_circ_	-0.53811	0.008605
hsa_circ_	-1.45791	0.008608
hsa_circ_	-0.47055	0.008609
hsa_circ_	-1.28613	0.008611
hsa_circ_	-1.03895	0.008614
hsa_circ_	0.913943	0.008614
hsa_circ_	0.58826	0.008615
hsa_circ_	-0.48514	0.008616
hsa_circ_	1.25082	0.008617
hsa_circ_	0.717018	0.00862
hsa_circ_	-0.67992	0.008623
hsa_circ_	-1.0562	0.008624
hsa_circ_	-0.51108	0.008624
hsa_circ_	-0.72773	0.008627

hsa_circ_	1.109409	0.008629
hsa_circ_	1.250305	0.008631
hsa_circ_	-0.61539	0.008642
hsa_circ_	0.508855	0.008643
hsa_circ_	1.680325	0.008645
hsa_circ_	1.031248	0.008647
hsa_circ_	-0.66083	0.008651
hsa_circ_	-0.80662	0.008652
hsa_circ_	0.827229	0.008663
hsa_circ_	0.510039	0.008663
hsa_circ_	0.902577	0.008664
hsa_circ_	-0.77952	0.008664
hsa_circ_	-0.93893	0.008668
hsa_circ_	-1.18194	0.008669
hsa_circ_	-0.70498	0.008674
hsa_circ_	-0.63765	0.008677
hsa_circ_	0.942029	0.008677
hsa_circ_	0.597483	0.008678
hsa_circ_	0.858634	0.008679
hsa_circ_	-0.66767	0.00869
hsa_circ_	0.524624	0.0087
hsa_circ_	0.883334	0.008701
hsa_circ_	0.913761	0.008715
hsa_circ_	0.835894	0.008715
hsa_circ_	0.602331	0.008729
hsa_circ_	1.918385	0.008732
hsa_circ_	-0.46839	0.008741
hsa_circ_	0.62194	0.008743
hsa_circ_	1.530115	0.008743
hsa_circ_	0.575522	0.008746
hsa_circ_	-0.60771	0.008763
hsa_circ_	-0.48123	0.008765
hsa_circ_	0.657785	0.008769
hsa_circ_	1.4861	0.008769
hsa_circ_	0.67265	0.008771
hsa_circ_	-0.49948	0.008772
hsa_circ_	0.47137	0.008775
hsa_circ_	-0.71814	0.008776
hsa_circ_	0.45788	0.00878
hsa_circ_	0.897253	0.008781
hsa_circ_	0.733824	0.008789
hsa_circ_	0.652005	0.008797
hsa_circ_	0.63778	0.008798
hsa_circ_	0.506642	0.008798
hsa_circ_	-0.68305	0.008818
hsa_circ_	-0.47935	0.008821
hsa_circ_	-1.03476	0.008824
hsa_circ_	0.644359	0.008832
hsa_circ_	-0.51567	0.008833
hsa_circ_	-0.60829	0.008836
hsa_circ_	-0.76741	0.008837
hsa_circ_	-0.51712	0.00884
hsa_circ_	-0.55717	0.008841
hsa_circ_	0.734587	0.008843

hsa_circ_	-0.78745	0.008844
hsa_circ_	0.71064	0.008845
hsa_circ_	0.526646	0.008851
hsa_circ_	1.808253	0.008851
hsa_circ_	-1.24534	0.008851
hsa_circ_	-0.71301	0.008855
hsa_circ_	-1.00473	0.00886
hsa_circ_	0.706066	0.008861
hsa_circ_	0.834811	0.008866
hsa_circ_	-0.97662	0.008866
hsa_circ_	1.346322	0.008867
hsa_circ_	-0.88541	0.008874
hsa_circ_	0.629123	0.008876
hsa_circ_	0.545009	0.00889
hsa_circ_	0.74876	0.008891
hsa_circ_	-0.92509	0.008893
hsa_circ_	-0.87708	0.008897
hsa_circ_	-0.78427	0.0089
hsa_circ_	0.509925	0.008903
hsa_circ_	-1.05309	0.008905
hsa_circ_	0.717603	0.008905
hsa_circ_	-0.92869	0.008917
hsa_circ_	-0.54991	0.008925
hsa_circ_	-1.06004	0.008926
hsa_circ_	-0.49074	0.008929
hsa_circ_	0.542805	0.008932
hsa_circ_	-1.81047	0.008951
hsa_circ_	-0.56348	0.008956
hsa_circ_	1.225807	0.008963
hsa_circ_	-0.89982	0.008977
hsa_circ_	-0.55446	0.008978
hsa_circ_	0.677867	0.008987
hsa_circ_	-0.99121	0.008989
hsa_circ_	0.554346	0.008993
hsa_circ_	-0.66513	0.009001
hsa_circ_	-0.9408	0.009001
hsa_circ_	-0.93475	0.009002
hsa_circ_	0.491018	0.009003
hsa_circ_	0.624964	0.009008
hsa_circ_	1.125698	0.009016
hsa_circ_	-0.82685	0.009019
hsa_circ_	-0.50006	0.009021
hsa_circ_	0.847162	0.009021
hsa_circ_	1.289961	0.00903
hsa_circ_	0.768566	0.009035
hsa_circ_	1.088839	0.009035
hsa_circ_	0.600444	0.009042
hsa_circ_	0.569659	0.009043
hsa_circ_	0.473857	0.009045
hsa_circ_	-0.51064	0.009055
hsa_circ_	0.560578	0.009056
hsa_circ_	0.50737	0.009061
hsa_circ_	1.137793	0.009067
hsa_circ_	0.546108	0.009072

hsa_circ_	0.587091	0.009072
hsa_circ_	-1.0248	0.009077
hsa_circ_	-0.62025	0.009078
hsa_circ_	1.312483	0.009078
hsa_circ_	-0.4454	0.009081
hsa_circ_	-0.55218	0.009082
hsa_circ_	-0.84824	0.009089
hsa_circ_	1.238889	0.009091
hsa_circ_	0.545787	0.009093
hsa_circ_	0.977845	0.009095
hsa_circ_	0.851528	0.009097
hsa_circ_	0.935861	0.009098
hsa_circ_	-1.26238	0.009099
hsa_circ_	0.784399	0.009112
hsa_circ_	-0.61556	0.009116
hsa_circ_	-0.7639	0.009119
hsa_circ_	-0.70929	0.00912
hsa_circ_	0.687262	0.009127
hsa_circ_	-0.4887	0.009138
hsa_circ_	-0.52947	0.009138
hsa_circ_	-0.55333	0.009139
hsa_circ_	0.582031	0.009142
hsa_circ_	-0.87633	0.009151
hsa_circ_	1.295225	0.009152
hsa_circ_	1.353681	0.009159
hsa_circ_	0.777687	0.009166
hsa_circ_	-0.77011	0.009168
hsa_circ_	-1.08989	0.009173
hsa_circ_	-0.93258	0.009176
hsa_circ_	-0.64773	0.009178
hsa_circ_	-0.69741	0.009181
hsa_circ_	0.702556	0.009184
hsa_circ_	0.690121	0.009185
hsa_circ_	0.86892	0.009197
hsa_circ_	-0.54079	0.009203
hsa_circ_	-0.82755	0.009207
hsa_circ_	-0.89055	0.009209
hsa_circ_	-0.97466	0.00921
hsa_circ_	0.779884	0.009214
hsa_circ_	-0.5156	0.009215
hsa_circ_	0.910269	0.00923
hsa_circ_	-0.70445	0.009236
hsa_circ_	-0.95835	0.009238
hsa_circ_	1.682178	0.009242
hsa_circ_	1.05601	0.009244
hsa_circ_	-1.03389	0.009245
hsa_circ_	2.005279	0.009247
hsa_circ_	-1.24023	0.009248
hsa_circ_	0.563273	0.009253
hsa_circ_	-0.46242	0.00926
hsa_circ_	0.500846	0.009262
hsa_circ_	0.738483	0.009267
hsa_circ_	-0.63911	0.009269
hsa_circ_	-0.63194	0.009272

hsa_circ_	0.515675	0.009277
hsa_circ_	-0.87618	0.009279
hsa_circ_	0.940499	0.009281
hsa_circ_	0.476605	0.009281
hsa_circ_	0.628073	0.009285
hsa_circ_	-0.88074	0.009288
hsa_circ_	1.243906	0.009294
hsa_circ_	0.864688	0.0093
hsa_circ_	-0.50874	0.009317
hsa_circ_	0.75884	0.009332
hsa_circ_	0.738036	0.009339
hsa_circ_	-0.57512	0.009342
hsa_circ_	-0.52944	0.009351
hsa_circ_	1.204052	0.009356
hsa_circ_	-0.99696	0.009364
hsa_circ_	2.498849	0.009372
hsa_circ_	-0.86848	0.009374
hsa_circ_	-0.58781	0.009379
hsa_circ_	0.456377	0.009385
hsa_circ_	0.617215	0.009386
hsa_circ_	0.858826	0.00939
hsa_circ_	-1.04154	0.009409
hsa_circ_	0.896703	0.009412
hsa_circ_	1.773059	0.009413
hsa_circ_	0.682325	0.009414
hsa_circ_	-0.70278	0.009422
hsa_circ_	-0.51524	0.009423
hsa_circ_	-0.64701	0.00943
hsa_circ_	0.844228	0.009437
hsa_circ_	-1.17614	0.009439
hsa_circ_	1.115812	0.00944
hsa_circ_	0.776955	0.009442
hsa_circ_	0.737177	0.009444
hsa_circ_	-0.52581	0.009452
hsa_circ_	0.543689	0.009456
hsa_circ_	0.505194	0.009456
hsa_circ_	-0.54747	0.00946
hsa_circ_	1.234954	0.00946
hsa_circ_	-1.31	0.009461
hsa_circ_	0.797771	0.009473
hsa_circ_	-0.68363	0.009474
hsa_circ_	-0.60008	0.009481
hsa_circ_	-1.57519	0.009483
hsa_circ_	0.651002	0.009486
hsa_circ_	0.660534	0.009494
hsa_circ_	-0.55931	0.009504
hsa_circ_	-0.63335	0.009507
hsa_circ_	0.699379	0.00951
hsa_circ_	0.854834	0.00951
hsa_circ_	-0.56513	0.009521
hsa_circ_	0.79963	0.009523
hsa_circ_	0.913403	0.009524
hsa_circ_	1.181944	0.00953
hsa_circ_	-0.58238	0.009531

hsa_circ_	-0.535	0.009534
hsa_circ_	-0.6512	0.009541
hsa_circ_	-0.69524	0.009542
hsa_circ_	-0.5632	0.009551
hsa_circ_	0.586941	0.009572
hsa_circ_	-0.89125	0.009573
hsa_circ_	0.684532	0.009581
hsa_circ_	0.626826	0.009583
hsa_circ_	-0.77669	0.009586
hsa_circ_	-0.84489	0.009586
hsa_circ_	0.716524	0.009594
hsa_circ_	-0.58431	0.009606
hsa_circ_	0.78646	0.009613
hsa_circ_	-0.62191	0.009615
hsa_circ_	-0.58416	0.009616
hsa_circ_	0.586847	0.009619
hsa_circ_	-0.69539	0.009622
hsa_circ_	1.353801	0.009631
hsa_circ_	0.7179	0.009632
hsa_circ_	-1.80025	0.009635
hsa_circ_	1.218124	0.009635
hsa_circ_	0.807978	0.009636
hsa_circ_	0.793509	0.009638
hsa_circ_	-1.54487	0.009646
hsa_circ_	0.97044	0.00965
hsa_circ_	-0.52378	0.009665
hsa_circ_	-0.47442	0.009685
hsa_circ_	0.845091	0.009687
hsa_circ_	0.643431	0.009695
hsa_circ_	-0.7921	0.009698
hsa_circ_	1.156205	0.009699
hsa_circ_	-0.62369	0.009703
hsa_circ_	-0.58567	0.009703
hsa_circ_	1.156564	0.009705
hsa_circ_	0.535615	0.009712
hsa_circ_	0.904946	0.009715
hsa_circ_	1.304002	0.009716
hsa_circ_	-2.30943	0.009722
hsa_circ_	-2.68589	0.009723
hsa_circ_	0.583202	0.009727
hsa_circ_	1.192683	0.009728
hsa_circ_	-0.7102	0.00973
hsa_circ_	-0.7438	0.009739
hsa_circ_	-0.47562	0.009741
hsa_circ_	1.108585	0.009749
hsa_circ_	-1.11108	0.009753
hsa_circ_	0.701057	0.009757
hsa_circ_	-0.51986	0.009761
hsa_circ_	-0.6244	0.009762
hsa_circ_	-0.46921	0.00977
hsa_circ_	-0.68633	0.009776
hsa_circ_	-0.48767	0.009777
hsa_circ_	-0.98058	0.009786
hsa_circ_	-1.14522	0.009786

hsa_circ_	0.734494	0.00979
hsa_circ_	1.301097	0.009793
hsa_circ_	0.597674	0.009799
hsa_circ_	-0.48529	0.009802
hsa_circ_	0.968304	0.009802
hsa_circ_	-0.51587	0.009804
hsa_circ_	0.758769	0.00981
hsa_circ_	0.69599	0.009817
hsa_circ_	0.724025	0.009821
hsa_circ_	0.826402	0.009827
hsa_circ_	0.513602	0.009831
hsa_circ_	-0.80056	0.00984
hsa_circ_	-0.73039	0.009842
hsa_circ_	-0.52386	0.009842
hsa_circ_	-1.15009	0.009842
hsa_circ_	0.463507	0.009847
hsa_circ_	-0.57888	0.009855
hsa_circ_	-1.2414	0.009858
hsa_circ_	0.461456	0.009862
hsa_circ_	1.780788	0.009871
hsa_circ_	-1.55769	0.009876
hsa_circ_	1.36609	0.009878
hsa_circ_	-0.55803	0.009881
hsa_circ_	1.044328	0.009885
hsa_circ_	-0.65738	0.00989
hsa_circ_	0.581601	0.009899
hsa_circ_	0.549227	0.009902
hsa_circ_	-0.68958	0.009902
hsa_circ_	-0.45582	0.009905
hsa_circ_	1.132794	0.009911
hsa_circ_	1.297001	0.009913
hsa_circ_	-1.23294	0.009927
hsa_circ_	0.673771	0.009933
hsa_circ_	0.532879	0.009952
hsa_circ_	0.648773	0.009964
hsa_circ_	-1.01648	0.009964
hsa_circ_	0.614015	0.009964
hsa_circ_	0.871757	0.009974
hsa_circ_	-0.97562	0.009974
hsa_circ_	0.49962	0.009981
hsa_circ_	0.622598	0.009983
hsa_circ_	-1.497	0.010001
hsa_circ_	-0.56341	0.010002
hsa_circ_	0.962954	0.010002
hsa_circ_	0.689077	0.010003
hsa_circ_	1.040043	0.010004
hsa_circ_	0.497696	0.010007
hsa_circ_	0.834652	0.01001
hsa_circ_	-0.87949	0.010016
hsa_circ_	1.360686	0.010017
hsa_circ_	1.360673	0.010018
hsa_circ_	0.62807	0.010034
hsa_circ_	-0.5814	0.010038
hsa_circ_	0.576197	0.010038

hsa_circ_	0.941207	0.01004
hsa_circ_	1.482037	0.010043
hsa_circ_	0.655794	0.010045
hsa_circ_	0.560886	0.010052
hsa_circ_	-0.52826	0.010053
hsa_circ_	0.516486	0.010059
hsa_circ_	1.388104	0.010059
hsa_circ_	-1.47944	0.010068
hsa_circ_	0.601158	0.010072
hsa_circ_	0.805906	0.010073
hsa_circ_	0.540403	0.010074
hsa_circ_	0.971758	0.010079
hsa_circ_	-0.86192	0.010081
hsa_circ_	-0.90007	0.010082
hsa_circ_	-0.83207	0.010084
hsa_circ_	-0.79433	0.010092
hsa_circ_	-0.52393	0.010098
hsa_circ_	2.37509	0.010102
hsa_circ_	2.93227	0.01012
hsa_circ_	-0.56152	0.010125
hsa_circ_	-0.90747	0.010131
hsa_circ_	-0.45787	0.01014
hsa_circ_	-1.05237	0.010145
hsa_circ_	0.707938	0.010147
hsa_circ_	1.168108	0.010149
hsa_circ_	-1.14018	0.01015
hsa_circ_	0.846848	0.01015
hsa_circ_	0.510965	0.01015
hsa_circ_	-0.49917	0.010151
hsa_circ_	-1.47565	0.010156
hsa_circ_	-0.79895	0.010156
hsa_circ_	0.599488	0.010162
hsa_circ_	-1.12379	0.010166
hsa_circ_	-0.85012	0.010167
hsa_circ_	1.224101	0.010167
hsa_circ_	0.526991	0.010168
hsa_circ_	-0.82039	0.010168
hsa_circ_	0.651908	0.010175
hsa_circ_	0.960899	0.010178
hsa_circ_	1.066884	0.010179
hsa_circ_	-1.27885	0.010179
hsa_circ_	-1.62114	0.010181
hsa_circ_	-0.6213	0.010181
hsa_circ_	1.135928	0.010182
hsa_circ_	0.510371	0.010187
hsa_circ_	0.633011	0.010191
hsa_circ_	0.910746	0.0102
hsa_circ_	-0.88447	0.010205
hsa_circ_	2.015648	0.010207
hsa_circ_	1.2353	0.010214
hsa_circ_	-1.22971	0.010215
hsa_circ_	-0.62518	0.010221
hsa_circ_	-0.58211	0.010221
hsa_circ_	0.887654	0.010222

hsa_circ_	-1.30126	0.010225
hsa_circ_	0.602659	0.010228
hsa_circ_	-0.8093	0.01023
hsa_circ_	-1.99709	0.010232
hsa_circ_	-0.57814	0.010249
hsa_circ_	1.075624	0.010249
hsa_circ_	-0.9129	0.01025
hsa_circ_	-0.92413	0.01025
hsa_circ_	0.545076	0.010255
hsa_circ_	-0.72606	0.010258
hsa_circ_	0.742275	0.010261
hsa_circ_	-1.07098	0.010265
hsa_circ_	1.103249	0.010267
hsa_circ_	0.783936	0.010287
hsa_circ_	-0.61111	0.010287
hsa_circ_	0.649831	0.010293
hsa_circ_	-0.68496	0.010295
hsa_circ_	0.888361	0.010296
hsa_circ_	0.524978	0.010298
hsa_circ_	0.46509	0.010309
hsa_circ_	0.636722	0.01031
hsa_circ_	-0.8044	0.010318
hsa_circ_	-0.99004	0.010319
hsa_circ_	0.710005	0.010333
hsa_circ_	-0.57591	0.010333
hsa_circ_	-0.59917	0.010344
hsa_circ_	0.588039	0.010346
hsa_circ_	-0.87523	0.010358
hsa_circ_	0.74094	0.010358
hsa_circ_	0.632178	0.010358
hsa_circ_	1.431033	0.010362
hsa_circ_	-0.99109	0.010373
hsa_circ_	-0.86882	0.010374
hsa_circ_	-0.60024	0.010376
hsa_circ_	0.633619	0.010378
hsa_circ_	-0.45128	0.010379
hsa_circ_	-0.92492	0.010385
hsa_circ_	0.758447	0.01039
hsa_circ_	0.689357	0.010392
hsa_circ_	0.928347	0.0104
hsa_circ_	0.647237	0.010401
hsa_circ_	-0.57222	0.010403
hsa_circ_	-0.9333	0.010403
hsa_circ_	-0.45851	0.010406
hsa_circ_	-0.44518	0.010406
hsa_circ_	0.88589	0.010408
hsa_circ_	0.546348	0.010409
hsa_circ_	-0.66125	0.01041
hsa_circ_	0.660445	0.010414
hsa_circ_	0.844874	0.010415
hsa_circ_	0.728478	0.010415
hsa_circ_	-0.90317	0.010417
hsa_circ_	-0.85902	0.01042
hsa_circ_	0.553197	0.010421

hsa_circ_	-0.61289	0.010425
hsa_circ_	1.733998	0.010437
hsa_circ_	0.686221	0.010444
hsa_circ_	1.034377	0.010448
hsa_circ_	-0.73571	0.010454
hsa_circ_	0.902889	0.010459
hsa_circ_	-0.9216	0.010464
hsa_circ_	0.463637	0.010468
hsa_circ_	0.888112	0.010471
hsa_circ_	0.552813	0.010477
hsa_circ_	-0.87649	0.010489
hsa_circ_	0.709934	0.01049
hsa_circ_	0.462532	0.010491
hsa_circ_	0.545645	0.010499
hsa_circ_	0.629473	0.010511
hsa_circ_	1.007024	0.010512
hsa_circ_	0.656872	0.010514
hsa_circ_	-1.63493	0.01052
hsa_circ_	0.560637	0.010522
hsa_circ_	-0.84922	0.010525
hsa_circ_	-0.82852	0.010525
hsa_circ_	0.532552	0.010531
hsa_circ_	-0.5435	0.010532
hsa_circ_	0.871037	0.010534
hsa_circ_	-0.45771	0.010557
hsa_circ_	0.718417	0.010559
hsa_circ_	0.606322	0.010565
hsa_circ_	-0.45204	0.010571
hsa_circ_	0.621857	0.010574
hsa_circ_	0.658559	0.010577
hsa_circ_	-0.45507	0.010579
hsa_circ_	0.75848	0.010579
hsa_circ_	1.10474	0.01058
hsa_circ_	1.298832	0.010581
hsa_circ_	0.578208	0.010586
hsa_circ_	-0.77839	0.010589
hsa_circ_	0.482707	0.01059
hsa_circ_	-2.36623	0.010591
hsa_circ_	0.808142	0.010592
hsa_circ_	1.512542	0.010597
hsa_circ_	-0.69502	0.010597
hsa_circ_	-1.29798	0.010597
hsa_circ_	-0.88923	0.010599
hsa_circ_	-0.51564	0.010603
hsa_circ_	-0.86916	0.010614
hsa_circ_	-0.48085	0.010619
hsa_circ_	1.17276	0.010619
hsa_circ_	-0.91245	0.010633
hsa_circ_	-0.64453	0.010635
hsa_circ_	1.099634	0.010643
hsa_circ_	0.652954	0.010646
hsa_circ_	0.524126	0.010646
hsa_circ_	-0.84772	0.010657
hsa_circ_	-0.52721	0.010668

hsa_circ_	-0.70271	0.010682
hsa_circ_	-0.86653	0.010683
hsa_circ_	0.756704	0.010684
hsa_circ_	-0.8768	0.010684
hsa_circ_	-1.37853	0.010684
hsa_circ_	-0.54071	0.010684
hsa_circ_	-0.56855	0.01069
hsa_circ_	-0.78189	0.010691
hsa_circ_	0.695397	0.010692
hsa_circ_	0.481752	0.010693
hsa_circ_	-0.51428	0.010704
hsa_circ_	0.519516	0.010709
hsa_circ_	1.39636	0.010721
hsa_circ_	-1.09311	0.010725
hsa_circ_	-0.92189	0.01073
hsa_circ_	-0.61591	0.010731
hsa_circ_	0.591049	0.010735
hsa_circ_	0.489932	0.010735
hsa_circ_	0.690702	0.010736
hsa_circ_	0.653363	0.010738
hsa_circ_	-0.53851	0.01074
hsa_circ_	0.505337	0.010741
hsa_circ_	-0.54516	0.010747
hsa_circ_	-0.87407	0.010784
hsa_circ_	0.685595	0.010794
hsa_circ_	-0.85685	0.010797
hsa_circ_	-0.7561	0.010797
hsa_circ_	-0.54132	0.0108
hsa_circ_	-0.46556	0.010803
hsa_circ_	-0.50028	0.010808
hsa_circ_	0.632581	0.010812
hsa_circ_	-0.79586	0.010817
hsa_circ_	0.562704	0.010817
hsa_circ_	0.863583	0.01082
hsa_circ_	0.586265	0.010823
hsa_circ_	-0.62145	0.010827
hsa_circ_	-0.6283	0.010832
hsa_circ_	0.835503	0.010842
hsa_circ_	0.572286	0.010843
hsa_circ_	-0.55291	0.010845
hsa_circ_	-0.75133	0.010852
hsa_circ_	-1.44893	0.010854
hsa_circ_	-1.20698	0.010856
hsa_circ_	0.661163	0.010876
hsa_circ_	0.86186	0.010881
hsa_circ_	-0.65069	0.010882
hsa_circ_	-0.55761	0.010892
hsa_circ_	-0.46893	0.010893
hsa_circ_	-0.54783	0.010895
hsa_circ_	1.189572	0.010899
hsa_circ_	-0.50372	0.010899
hsa_circ_	-1.21144	0.010903
hsa_circ_	-1.04294	0.010904
hsa_circ_	0.827136	0.010908

hsa_circ_	0.762318	0.010908
hsa_circ_	0.6641	0.010912
hsa_circ_	0.674903	0.010915
hsa_circ_	-0.91607	0.010923
hsa_circ_	0.836971	0.010938
hsa_circ_	1.043468	0.010942
hsa_circ_	1.047259	0.010943
hsa_circ_	0.641701	0.010968
hsa_circ_	-0.52714	0.010969
hsa_circ_	-0.58529	0.010972
hsa_circ_	-1.24381	0.010977
hsa_circ_	-0.51196	0.010981
hsa_circ_	0.809807	0.010986
hsa_circ_	-0.77935	0.010986
hsa_circ_	0.956001	0.010993
hsa_circ_	0.473189	0.010994
hsa_circ_	-1.70351	0.010998
hsa_circ_	-0.93864	0.011
hsa_circ_	-1.10125	0.011001
hsa_circ_	-0.50402	0.011003
hsa_circ_	0.494807	0.011003
hsa_circ_	0.82677	0.01102
hsa_circ_	0.507509	0.01102
hsa_circ_	-1.10415	0.011028
hsa_circ_	-0.90555	0.011035
hsa_circ_	-0.91587	0.011037
hsa_circ_	-0.55125	0.011038
hsa_circ_	1.19222	0.011041
hsa_circ_	1.561213	0.011042
hsa_circ_	-0.45975	0.011043
hsa_circ_	1.225351	0.011048
hsa_circ_	1.242937	0.011049
hsa_circ_	1.576418	0.011052
hsa_circ_	-0.48852	0.011052
hsa_circ_	2.319166	0.011052
hsa_circ_	0.833328	0.011054
hsa_circ_	1.439067	0.011062
hsa_circ_	2.842177	0.011067
hsa_circ_	-1.19378	0.011067
hsa_circ_	-0.98802	0.011068
hsa_circ_	0.595642	0.011071
hsa_circ_	0.598267	0.011074
hsa_circ_	-0.81162	0.011075
hsa_circ_	-0.65741	0.011075
hsa_circ_	-0.9873	0.011078
hsa_circ_	-0.60939	0.011081
hsa_circ_	-0.59611	0.011085
hsa_circ_	-0.43617	0.01109
hsa_circ_	0.579025	0.011098
hsa_circ_	-0.52825	0.011099
hsa_circ_	1.699524	0.011102
hsa_circ_	0.966602	0.01111
hsa_circ_	-1.35575	0.011111
hsa_circ_	0.722565	0.011115

hsa_circ_	0.930073	0.011116
hsa_circ_	1.480923	0.011116
hsa_circ_	-1.07118	0.011117
hsa_circ_	0.821434	0.011119
hsa_circ_	-0.44671	0.011126
hsa_circ_	0.517266	0.011127
hsa_circ_	-0.43919	0.011131
hsa_circ_	1.110148	0.011133
hsa_circ_	-0.48574	0.011134
hsa_circ_	-0.48179	0.011136
hsa_circ_	-1.08234	0.011141
hsa_circ_	1.045196	0.011142
hsa_circ_	-0.93644	0.011147
hsa_circ_	1.158371	0.011165
hsa_circ_	-0.78829	0.011174
hsa_circ_	0.968346	0.011176
hsa_circ_	-0.9819	0.011182
hsa_circ_	-2.68621	0.011184
hsa_circ_	1.411649	0.011193
hsa_circ_	0.552997	0.011196
hsa_circ_	0.760388	0.011198
hsa_circ_	0.530061	0.011203
hsa_circ_	0.773091	0.011212
hsa_circ_	-0.89708	0.011212
hsa_circ_	0.807018	0.011215
hsa_circ_	0.567483	0.011219
hsa_circ_	-2.5951	0.011224
hsa_circ_	0.836152	0.011224
hsa_circ_	-0.45308	0.011231
hsa_circ_	0.647532	0.011235
hsa_circ_	0.620505	0.011238
hsa_circ_	0.648371	0.011244
hsa_circ_	0.49175	0.011253
hsa_circ_	-0.48464	0.011254
hsa_circ_	0.545101	0.011255
hsa_circ_	-0.61768	0.011255
hsa_circ_	-0.8258	0.011256
hsa_circ_	0.570289	0.011259
hsa_circ_	1.238908	0.011263
hsa_circ_	-0.47115	0.011265
hsa_circ_	-0.75099	0.011272
hsa_circ_	-0.78812	0.011274
hsa_circ_	0.646446	0.011274
hsa_circ_	-0.45692	0.011288
hsa_circ_	-0.98284	0.011292
hsa_circ_	-1.63716	0.011293
hsa_circ_	-0.90409	0.011294
hsa_circ_	0.540098	0.011294
hsa_circ_	1.859382	0.011297
hsa_circ_	1.112116	0.011298
hsa_circ_	1.386557	0.0113
hsa_circ_	-0.54329	0.011308
hsa_circ_	-0.62655	0.011309
hsa_circ_	0.529706	0.01131

hsa_circ_	-0.95662	0.011313
hsa_circ_	0.924246	0.011317
hsa_circ_	-0.44974	0.011318
hsa_circ_	-0.54485	0.011321
hsa_circ_	0.882121	0.011322
hsa_circ_	-0.55596	0.011323
hsa_circ_	1.416912	0.011336
hsa_circ_	1.469334	0.011342
hsa_circ_	0.595913	0.011348
hsa_circ_	0.551294	0.011363
hsa_circ_	1.988035	0.011365
hsa_circ_	0.462779	0.011367
hsa_circ_	-0.90742	0.011368
hsa_circ_	-0.5791	0.011377
hsa_circ_	0.544134	0.011386
hsa_circ_	0.895384	0.011395
hsa_circ_	0.505656	0.011396
hsa_circ_	0.488263	0.011399
hsa_circ_	0.524897	0.011401
hsa_circ_	0.690219	0.011403
hsa_circ_	0.888731	0.011405
hsa_circ_	-0.89722	0.011406
hsa_circ_	-0.74664	0.011416
hsa_circ_	0.6568	0.011422
hsa_circ_	-0.87204	0.011427
hsa_circ_	-0.9504	0.01143
hsa_circ_	-0.51181	0.011432
hsa_circ_	-0.83267	0.011437
hsa_circ_	0.679613	0.011438
hsa_circ_	-0.6261	0.01144
hsa_circ_	1.33518	0.011444
hsa_circ_	-1.21684	0.011446
hsa_circ_	0.758142	0.011448
hsa_circ_	0.634441	0.011458
hsa_circ_	-0.5443	0.011461
hsa_circ_	0.48233	0.011462
hsa_circ_	1.090983	0.011466
hsa_circ_	-0.52593	0.011472
hsa_circ_	-0.58007	0.011474
hsa_circ_	0.582614	0.011475
hsa_circ_	0.577888	0.011488
hsa_circ_	0.80314	0.011509
hsa_circ_	-0.46681	0.011509
hsa_circ_	-0.49192	0.011513
hsa_circ_	-0.8946	0.011517
hsa_circ_	0.81768	0.011519
hsa_circ_	-0.47173	0.011521
hsa_circ_	-0.89178	0.011531
hsa_circ_	0.579294	0.011534
hsa_circ_	-0.43062	0.011547
hsa_circ_	0.822081	0.011563
hsa_circ_	-0.51992	0.011564
hsa_circ_	-0.58476	0.011567
hsa_circ_	-0.8811	0.011572

hsa_circ_	0.86021	0.011573
hsa_circ_	0.613397	0.011576
hsa_circ_	0.802861	0.011577
hsa_circ_	0.727179	0.011585
hsa_circ_	-0.84435	0.011586
hsa_circ_	0.496702	0.011589
hsa_circ_	-0.59967	0.011598
hsa_circ_	-0.4986	0.011605
hsa_circ_	-0.62613	0.011605
hsa_circ_	-0.79841	0.011612
hsa_circ_	0.705636	0.011616
hsa_circ_	0.561271	0.011623
hsa_circ_	-1.33283	0.011625
hsa_circ_	-0.92522	0.01163
hsa_circ_	-0.82241	0.011633
hsa_circ_	0.864536	0.011645
hsa_circ_	-0.58391	0.011654
hsa_circ_	-0.83644	0.011654
hsa_circ_	-1.31725	0.011659
hsa_circ_	0.545823	0.011668
hsa_circ_	0.476876	0.011669
hsa_circ_	0.651219	0.011669
hsa_circ_	-0.98367	0.011677
hsa_circ_	-0.89393	0.011678
hsa_circ_	0.650379	0.011683
hsa_circ_	-0.57598	0.011695
hsa_circ_	-0.86401	0.011698
hsa_circ_	0.590198	0.0117
hsa_circ_	0.465429	0.011707
hsa_circ_	-0.69731	0.011723
hsa_circ_	0.751901	0.011725
hsa_circ_	1.049053	0.011735
hsa_circ_	1.532898	0.011736
hsa_circ_	0.461685	0.011745
hsa_circ_	-0.49151	0.011747
hsa_circ_	0.510743	0.011754
hsa_circ_	1.015888	0.011763
hsa_circ_	-1.53021	0.011767
hsa_circ_	-1.18519	0.011774
hsa_circ_	0.591283	0.011789
hsa_circ_	0.533302	0.011795
hsa_circ_	-0.6398	0.011798
hsa_circ_	-0.63965	0.011799
hsa_circ_	-1.40737	0.0118
hsa_circ_	-0.5636	0.011804
hsa_circ_	-0.67991	0.011809
hsa_circ_	2.1734	0.011816
hsa_circ_	0.60571	0.011819
hsa_circ_	0.674052	0.011827
hsa_circ_	0.517083	0.011829
hsa_circ_	1.010371	0.011829
hsa_circ_	0.504214	0.011832
hsa_circ_	-0.75729	0.011833
hsa_circ_	1.252129	0.011843

hsa_circ_	1.009402	0.01185
hsa_circ_	-0.75216	0.011854
hsa_circ_	0.710401	0.011854
hsa_circ_	-0.55381	0.011857
hsa_circ_	-1.1077	0.011862
hsa_circ_	0.50273	0.011863
hsa_circ_	0.744714	0.011866
hsa_circ_	0.670832	0.011867
hsa_circ_	0.645781	0.01187
hsa_circ_	-0.56895	0.01188
hsa_circ_	-0.6164	0.011882
hsa_circ_	-0.4956	0.011888
hsa_circ_	1.167568	0.011889
hsa_circ_	-0.90983	0.011891
hsa_circ_	-0.66199	0.011892
hsa_circ_	0.694347	0.011893
hsa_circ_	-0.5323	0.011894
hsa_circ_	-1.70173	0.011895
hsa_circ_	0.624533	0.011902
hsa_circ_	-0.59821	0.011904
hsa_circ_	-0.88828	0.011907
hsa_circ_	0.833111	0.011909
hsa_circ_	-1.03402	0.011919
hsa_circ_	-1.02815	0.011921
hsa_circ_	1.739252	0.011921
hsa_circ_	0.658335	0.011924
hsa_circ_	1.119489	0.01194
hsa_circ_	-0.94356	0.011941
hsa_circ_	0.886671	0.011948
hsa_circ_	-0.4676	0.011953
hsa_circ_	0.582299	0.011957
hsa_circ_	-0.69437	0.01196
hsa_circ_	-1.02821	0.011962
hsa_circ_	-0.46757	0.011968
hsa_circ_	-0.50422	0.011981
hsa_circ_	-0.47583	0.011987
hsa_circ_	-0.62784	0.011997
hsa_circ_	-0.44362	0.012005
hsa_circ_	-0.83268	0.012006
hsa_circ_	0.762851	0.01201
hsa_circ_	1.851425	0.012019
hsa_circ_	1.33565	0.012027
hsa_circ_	-0.83686	0.01203
hsa_circ_	-0.55409	0.012031
hsa_circ_	0.747588	0.012039
hsa_circ_	0.846035	0.012045
hsa_circ_	-0.74733	0.012049
hsa_circ_	-0.46901	0.012053
hsa_circ_	0.873614	0.012058
hsa_circ_	-0.72115	0.012059
hsa_circ_	0.804669	0.01207
hsa_circ_	0.603956	0.01207
hsa_circ_	0.466208	0.012073
hsa_circ_	-0.75999	0.012075

hsa_circ_	-0.56221	0.012083
hsa_circ_	-0.59647	0.012089
hsa_circ_	-0.98227	0.01209
hsa_circ_	-1.40602	0.012095
hsa_circ_	1.789851	0.012117
hsa_circ_	-0.47457	0.012121
hsa_circ_	0.578145	0.012129
hsa_circ_	0.572622	0.012134
hsa_circ_	0.512421	0.012134
hsa_circ_	-0.59205	0.012137
hsa_circ_	-0.8295	0.012137
hsa_circ_	-0.77118	0.012141
hsa_circ_	0.914251	0.012144
hsa_circ_	0.923485	0.012149
hsa_circ_	0.883418	0.012149
hsa_circ_	-0.51612	0.012154
hsa_circ_	1.283396	0.012156
hsa_circ_	-1.22835	0.012157
hsa_circ_	0.643276	0.012159
hsa_circ_	0.932255	0.012168
hsa_circ_	-0.97988	0.012172
hsa_circ_	0.630967	0.012172
hsa_circ_	0.551025	0.012176
hsa_circ_	-0.48841	0.012177
hsa_circ_	0.572054	0.01218
hsa_circ_	0.842453	0.012181
hsa_circ_	1.637749	0.012191
hsa_circ_	0.660181	0.012197
hsa_circ_	-0.84747	0.012201
hsa_circ_	-0.44458	0.012202
hsa_circ_	-0.98508	0.012204
hsa_circ_	1.587577	0.012205
hsa_circ_	0.633629	0.012213
hsa_circ_	-0.57812	0.012215
hsa_circ_	1.352307	0.012221
hsa_circ_	0.529509	0.012226
hsa_circ_	0.682399	0.012232
hsa_circ_	-0.84837	0.012244
hsa_circ_	1.418353	0.01225
hsa_circ_	-0.41341	0.012254
hsa_circ_	1.540025	0.012258
hsa_circ_	-0.57225	0.012259
hsa_circ_	-0.95304	0.01227
hsa_circ_	0.983838	0.01227
hsa_circ_	-0.59493	0.012272
hsa_circ_	-0.54339	0.012276
hsa_circ_	-0.46018	0.012277
hsa_circ_	-0.46887	0.012283
hsa_circ_	1.739822	0.012283
hsa_circ_	-0.87468	0.012286
hsa_circ_	-1.1565	0.012288
hsa_circ_	-0.47239	0.012293
hsa_circ_	-0.43623	0.012298
hsa_circ_	0.785603	0.012309

hsa_circ_	-0.4658	0.012318
hsa_circ_	-0.84893	0.012325
hsa_circ_	-1.09823	0.012332
hsa_circ_	-0.67575	0.012334
hsa_circ_	-0.94879	0.012339
hsa_circ_	1.154147	0.012343
hsa_circ_	1.093956	0.012346
hsa_circ_	0.514011	0.012347
hsa_circ_	0.537906	0.012347
hsa_circ_	0.474298	0.01235
hsa_circ_	0.566566	0.012352
hsa_circ_	0.747204	0.012377
hsa_circ_	0.942988	0.012379
hsa_circ_	1.271726	0.012388
hsa_circ_	0.557502	0.012389
hsa_circ_	-0.73575	0.012393
hsa_circ_	-0.85815	0.012396
hsa_circ_	-0.84849	0.012404
hsa_circ_	0.536384	0.012411
hsa_circ_	0.935853	0.012418
hsa_circ_	-0.96619	0.01242
hsa_circ_	0.586779	0.012421
hsa_circ_	-1.03062	0.012422
hsa_circ_	-0.79035	0.012423
hsa_circ_	0.63544	0.012424
hsa_circ_	1.911138	0.012426
hsa_circ_	0.531928	0.012427
hsa_circ_	1.862038	0.012428
hsa_circ_	-0.92969	0.012428
hsa_circ_	0.726621	0.012428
hsa_circ_	-0.88151	0.012433
hsa_circ_	-0.80266	0.012436
hsa_circ_	-0.77972	0.012437
hsa_circ_	1.00339	0.012446
hsa_circ_	-0.78017	0.012453
hsa_circ_	0.493654	0.012459
hsa_circ_	0.520918	0.012462
hsa_circ_	-0.52413	0.012468
hsa_circ_	1.063034	0.012468
hsa_circ_	0.463443	0.012469
hsa_circ_	-1.09724	0.012472
hsa_circ_	-0.87457	0.012474
hsa_circ_	0.544071	0.012476
hsa_circ_	0.439029	0.012477
hsa_circ_	0.539357	0.012479
hsa_circ_	1.189466	0.012488
hsa_circ_	-0.71201	0.012494
hsa_circ_	-0.47028	0.012498
hsa_circ_	1.059623	0.012512
hsa_circ_	0.975913	0.012518
hsa_circ_	-0.48308	0.012526
hsa_circ_	-0.78904	0.012527
hsa_circ_	-0.45049	0.012528
hsa_circ_	0.983091	0.012536

hsa_circ_	-0.44801	0.012541
hsa_circ_	-0.58193	0.012554
hsa_circ_	-0.4584	0.012555
hsa_circ_	-0.48729	0.012562
hsa_circ_	0.503512	0.012567
hsa_circ_	-0.69226	0.012568
hsa_circ_	0.785884	0.012572
hsa_circ_	-0.50659	0.012573
hsa_circ_	-0.96062	0.012577
hsa_circ_	-0.49471	0.012589
hsa_circ_	0.551049	0.012596
hsa_circ_	0.668025	0.012596
hsa_circ_	1.014845	0.012597
hsa_circ_	-0.96514	0.012597
hsa_circ_	-0.77707	0.012606
hsa_circ_	2.279384	0.012608
hsa_circ_	-0.94015	0.012616
hsa_circ_	0.805619	0.012616
hsa_circ_	-0.46832	0.012618
hsa_circ_	0.784255	0.012629
hsa_circ_	0.897767	0.012632
hsa_circ_	0.657937	0.012635
hsa_circ_	0.529775	0.012636
hsa_circ_	0.809016	0.012639
hsa_circ_	0.625981	0.01264
hsa_circ_	-0.49296	0.012644
hsa_circ_	-0.5268	0.012646
hsa_circ_	0.541477	0.012648
hsa_circ_	0.628973	0.012655
hsa_circ_	0.95776	0.012655
hsa_circ_	-0.60827	0.012665
hsa_circ_	-1.09217	0.012666
hsa_circ_	-0.69411	0.012668
hsa_circ_	-0.78908	0.012673
hsa_circ_	-1.34387	0.012696
hsa_circ_	0.447306	0.012699
hsa_circ_	1.065192	0.012702
hsa_circ_	-0.60516	0.012705
hsa_circ_	0.682643	0.012722
hsa_circ_	0.573337	0.012724
hsa_circ_	-0.57096	0.012733
hsa_circ_	-0.84819	0.012733
hsa_circ_	0.934621	0.012738
hsa_circ_	0.880979	0.012739
hsa_circ_	-0.46956	0.012742
hsa_circ_	0.567781	0.012746
hsa_circ_	0.574059	0.012749
hsa_circ_	1.502281	0.012749
hsa_circ_	0.629592	0.012758
hsa_circ_	-0.53058	0.012784
hsa_circ_	-0.55286	0.012793
hsa_circ_	0.594549	0.012799
hsa_circ_	1.624047	0.012801
hsa_circ_	-0.48354	0.012811

hsa_circ_	0.581388	0.012816
hsa_circ_	-0.51627	0.012828
hsa_circ_	-1.22824	0.01283
hsa_circ_	0.890872	0.012831
hsa_circ_	0.460967	0.012834
hsa_circ_	-0.85358	0.012835
hsa_circ_	-0.84941	0.012835
hsa_circ_	-0.85793	0.012837
hsa_circ_	1.069487	0.01284
hsa_circ_	-0.52747	0.012845
hsa_circ_	-0.43426	0.012853
hsa_circ_	-0.82084	0.012864
hsa_circ_	1.196497	0.01287
hsa_circ_	0.52937	0.012872
hsa_circ_	-0.52792	0.012877
hsa_circ_	-0.87102	0.012885
hsa_circ_	-0.50715	0.012893
hsa_circ_	0.670822	0.012904
hsa_circ_	0.687934	0.012905
hsa_circ_	1.642683	0.01291
hsa_circ_	1.010487	0.01292
hsa_circ_	-0.87656	0.012924
hsa_circ_	-0.7681	0.012925
hsa_circ_	-0.84603	0.012927
hsa_circ_	0.615422	0.012929
hsa_circ_	1.404065	0.01293
hsa_circ_	0.798447	0.012935
hsa_circ_	-0.5766	0.012937
hsa_circ_	0.641639	0.012939
hsa_circ_	0.45966	0.012946
hsa_circ_	0.677452	0.012948
hsa_circ_	-0.92491	0.01295
hsa_circ_	0.464913	0.012957
hsa_circ_	-0.93764	0.01296
hsa_circ_	1.238151	0.01296
hsa_circ_	-0.7587	0.012966
hsa_circ_	-0.4421	0.012971
hsa_circ_	-0.47541	0.012973
hsa_circ_	-0.65497	0.012978
hsa_circ_	-0.95517	0.012978
hsa_circ_	0.545871	0.01298
hsa_circ_	0.582484	0.012984
hsa_circ_	0.872124	0.012984
hsa_circ_	-0.46899	0.012985
hsa_circ_	-0.92058	0.012996
hsa_circ_	-0.80831	0.013011
hsa_circ_	0.685297	0.013012
hsa_circ_	-0.62368	0.013013
hsa_circ_	1.77086	0.013013
hsa_circ_	1.329244	0.013019
hsa_circ_	0.724375	0.013029
hsa_circ_	-1.25335	0.013037
hsa_circ_	0.952658	0.013039
hsa_circ_	-0.77068	0.013044

hsa_circ_	0.746028	0.013049
hsa_circ_	0.525286	0.013052
hsa_circ_	0.507982	0.01306
hsa_circ_	1.123832	0.01307
hsa_circ_	0.711373	0.013076
hsa_circ_	0.846152	0.013079
hsa_circ_	-0.5493	0.01308
hsa_circ_	-0.51424	0.013081
hsa_circ_	-1.03139	0.013097
hsa_circ_	0.575539	0.013099
hsa_circ_	0.510101	0.0131
hsa_circ_	0.642574	0.013101
hsa_circ_	-0.84811	0.013104
hsa_circ_	0.773846	0.013105
hsa_circ_	-0.46139	0.013112
hsa_circ_	0.537432	0.013112
hsa_circ_	0.723564	0.013115
hsa_circ_	0.718456	0.013117
hsa_circ_	0.45291	0.013118
hsa_circ_	-0.51439	0.013118
hsa_circ_	-0.47703	0.01312
hsa_circ_	-0.9198	0.013126
hsa_circ_	-0.8933	0.013138
hsa_circ_	-0.52373	0.013141
hsa_circ_	-1.09194	0.013151
hsa_circ_	0.495335	0.013153
hsa_circ_	-0.86442	0.013158
hsa_circ_	-0.9398	0.013165
hsa_circ_	-0.88527	0.013176
hsa_circ_	-0.45739	0.013177
hsa_circ_	-0.68013	0.01318
hsa_circ_	-1.81951	0.01318
hsa_circ_	0.494783	0.013188
hsa_circ_	0.677682	0.013189
hsa_circ_	0.864263	0.013192
hsa_circ_	0.652027	0.013203
hsa_circ_	0.515083	0.013216
hsa_circ_	-0.51129	0.013217
hsa_circ_	-0.89643	0.013219
hsa_circ_	0.964567	0.013222
hsa_circ_	-0.6515	0.013225
hsa_circ_	-1.21295	0.013226
hsa_circ_	-0.88091	0.013234
hsa_circ_	-0.57648	0.013236
hsa_circ_	0.899683	0.013239
hsa_circ_	-0.78654	0.013253
hsa_circ_	-0.42493	0.013255
hsa_circ_	0.835791	0.013258
hsa_circ_	-0.55647	0.013259
hsa_circ_	0.483499	0.013264
hsa_circ_	-0.83224	0.013265
hsa_circ_	0.699584	0.013271
hsa_circ_	1.650074	0.013271
hsa_circ_	-0.82607	0.013273

hsa_circ_	-0.47214	0.013284
hsa_circ_	0.422035	0.013289
hsa_circ_	-0.68586	0.013297
hsa_circ_	1.260513	0.013309
hsa_circ_	-0.50022	0.013311
hsa_circ_	-0.85787	0.013311
hsa_circ_	0.989616	0.013311
hsa_circ_	1.834443	0.013318
hsa_circ_	0.587597	0.013328
hsa_circ_	0.853951	0.013342
hsa_circ_	0.522312	0.013344
hsa_circ_	-0.67618	0.013348
hsa_circ_	0.479833	0.013364
hsa_circ_	-0.7093	0.013378
hsa_circ_	-0.61197	0.013378
hsa_circ_	-0.81397	0.01338
hsa_circ_	0.534809	0.013388
hsa_circ_	0.718669	0.013396
hsa_circ_	0.566732	0.013397
hsa_circ_	-0.93957	0.013401
hsa_circ_	0.702241	0.013404
hsa_circ_	-0.46096	0.013408
hsa_circ_	-0.47042	0.013411
hsa_circ_	-1.46093	0.013411
hsa_circ_	-0.64022	0.013412
hsa_circ_	-0.83774	0.013417
hsa_circ_	0.71659	0.013422
hsa_circ_	1.879025	0.013425
hsa_circ_	-0.89294	0.013427
hsa_circ_	0.947521	0.013433
hsa_circ_	-0.78326	0.013435
hsa_circ_	0.93167	0.013443
hsa_circ_	-0.97376	0.013453
hsa_circ_	0.441498	0.013457
hsa_circ_	2.077396	0.01346
hsa_circ_	1.036784	0.013461
hsa_circ_	0.483525	0.013477
hsa_circ_	-0.45998	0.013478
hsa_circ_	0.504787	0.013479
hsa_circ_	-1.03451	0.013479
hsa_circ_	1.14047	0.013483
hsa_circ_	-0.42527	0.013491
hsa_circ_	0.562376	0.013495
hsa_circ_	1.057717	0.013495
hsa_circ_	-0.80137	0.013505
hsa_circ_	0.937294	0.013517
hsa_circ_	-0.65542	0.013521
hsa_circ_	-0.68116	0.013521
hsa_circ_	-0.8951	0.013523
hsa_circ_	-0.48549	0.013524
hsa_circ_	1.346821	0.013538
hsa_circ_	0.515665	0.013547
hsa_circ_	-0.77919	0.013559
hsa_circ_	0.591374	0.013567

hsa_circ_	-0.77614	0.013572
hsa_circ_	0.465036	0.013572
hsa_circ_	-0.65545	0.013575
hsa_circ_	-0.85729	0.013576
hsa_circ_	0.895462	0.013579
hsa_circ_	0.688411	0.013592
hsa_circ_	-0.7601	0.013593
hsa_circ_	0.507722	0.013594
hsa_circ_	-1.45454	0.013597
hsa_circ_	1.57544	0.013597
hsa_circ_	-0.67417	0.013608
hsa_circ_	-0.48776	0.013612
hsa_circ_	-0.50687	0.013613
hsa_circ_	0.80831	0.013618
hsa_circ_	0.837264	0.013621
hsa_circ_	0.918445	0.013621
hsa_circ_	0.431544	0.013624
hsa_circ_	0.504846	0.013629
hsa_circ_	0.624475	0.013639
hsa_circ_	1.511403	0.013639
hsa_circ_	1.202138	0.013643
hsa_circ_	-0.51423	0.013651
hsa_circ_	0.588942	0.013677
hsa_circ_	-0.75529	0.01368
hsa_circ_	0.680757	0.013681
hsa_circ_	-0.83859	0.013684
hsa_circ_	-1.07867	0.0137
hsa_circ_	-0.78633	0.0137
hsa_circ_	-0.92551	0.013704
hsa_circ_	-1.44479	0.013707
hsa_circ_	0.494859	0.013707
hsa_circ_	-0.46761	0.013708
hsa_circ_	-0.72676	0.013711
hsa_circ_	0.428629	0.013713
hsa_circ_	0.557227	0.013738
hsa_circ_	-1.03402	0.013742
hsa_circ_	1.533466	0.013746
hsa_circ_	0.491653	0.01375
hsa_circ_	0.804711	0.01377
hsa_circ_	-0.5634	0.013779
hsa_circ_	-0.72021	0.013786
hsa_circ_	-0.78209	0.013791
hsa_circ_	0.847304	0.013796
hsa_circ_	-1.08275	0.013798
hsa_circ_	-0.95002	0.013805
hsa_circ_	0.437189	0.013808
hsa_circ_	0.61947	0.013823
hsa_circ_	0.618498	0.013825
hsa_circ_	1.286714	0.013829
hsa_circ_	0.972509	0.013829
hsa_circ_	0.609447	0.01383
hsa_circ_	-0.59961	0.013837
hsa_circ_	0.951251	0.013842
hsa_circ_	-0.82443	0.013849

hsa_circ_	-0.59231	0.013851
hsa_circ_	-0.74633	0.013852
hsa_circ_	0.857325	0.013854
hsa_circ_	0.574103	0.013864
hsa_circ_	-0.56203	0.013866
hsa_circ_	-0.46153	0.013874
hsa_circ_	-0.93921	0.013874
hsa_circ_	-0.54394	0.013879
hsa_circ_	-1.05747	0.01388
hsa_circ_	0.902125	0.013894
hsa_circ_	1.3493	0.013901
hsa_circ_	-0.93986	0.013905
hsa_circ_	0.536493	0.013908
hsa_circ_	1.053327	0.013918
hsa_circ_	0.455127	0.013918
hsa_circ_	-0.51608	0.013918
hsa_circ_	-1.27226	0.013922
hsa_circ_	0.545522	0.013923
hsa_circ_	0.479678	0.013923
hsa_circ_	0.727816	0.013927
hsa_circ_	-0.46008	0.013928
hsa_circ_	0.833835	0.013938
hsa_circ_	1.039493	0.013948
hsa_circ_	1.028649	0.01395
hsa_circ_	-0.70617	0.013951
hsa_circ_	-0.73766	0.013956
hsa_circ_	-1.81783	0.013971
hsa_circ_	-0.44282	0.013972
hsa_circ_	0.606196	0.013972
hsa_circ_	-0.53807	0.013974
hsa_circ_	0.491054	0.013994
hsa_circ_	-0.55203	0.013994
hsa_circ_	0.461951	0.013995
hsa_circ_	-0.68936	0.013998
hsa_circ_	-0.80739	0.014
hsa_circ_	0.833218	0.014004
hsa_circ_	0.650656	0.014004
hsa_circ_	-0.66859	0.014007
hsa_circ_	1.278284	0.014012
hsa_circ_	-0.60328	0.014013
hsa_circ_	0.493886	0.014016
hsa_circ_	0.651134	0.014017
hsa_circ_	-0.42402	0.014022
hsa_circ_	0.858002	0.014024
hsa_circ_	0.769394	0.01403
hsa_circ_	-0.81101	0.01403
hsa_circ_	-0.44426	0.014037
hsa_circ_	0.73563	0.014038
hsa_circ_	-0.79901	0.014041
hsa_circ_	-0.81151	0.014044
hsa_circ_	1.030732	0.014045
hsa_circ_	-0.67309	0.01405
hsa_circ_	-0.54039	0.014053
hsa_circ_	-0.78202	0.014071

hsa_circ_	0.644152	0.014075
hsa_circ_	-0.65036	0.014075
hsa_circ_	0.475873	0.014076
hsa_circ_	0.453306	0.014078
hsa_circ_	-0.48512	0.014079
hsa_circ_	-0.5829	0.014086
hsa_circ_	-0.74273	0.014089
hsa_circ_	1.006159	0.014089
hsa_circ_	0.650328	0.014105
hsa_circ_	-0.90137	0.01411
hsa_circ_	-1.01718	0.01411
hsa_circ_	-0.90111	0.014112
hsa_circ_	0.452337	0.014117
hsa_circ_	-0.45961	0.014128
hsa_circ_	-0.46419	0.014129
hsa_circ_	0.480804	0.014134
hsa_circ_	-0.42926	0.014144
hsa_circ_	-0.51559	0.014145
hsa_circ_	0.768338	0.014145
hsa_circ_	2.546448	0.01415
hsa_circ_	-1.87051	0.014152
hsa_circ_	0.414402	0.014153
hsa_circ_	1.352155	0.014159
hsa_circ_	1.701689	0.01416
hsa_circ_	-1.75168	0.014161
hsa_circ_	-0.75207	0.014165
hsa_circ_	1.110921	0.014167
hsa_circ_	0.440465	0.014171
hsa_circ_	-0.85017	0.014172
hsa_circ_	-1.03425	0.014173
hsa_circ_	0.482955	0.014176
hsa_circ_	0.457968	0.014186
hsa_circ_	-0.54469	0.014189
hsa_circ_	0.657761	0.014189
hsa_circ_	0.579556	0.014193
hsa_circ_	1.110973	0.014203
hsa_circ_	0.725106	0.014207
hsa_circ_	0.570489	0.014209
hsa_circ_	0.675204	0.014213
hsa_circ_	-0.7304	0.014223
hsa_circ_	-0.92041	0.014228
hsa_circ_	-0.44312	0.014232
hsa_circ_	-0.48914	0.014234
hsa_circ_	-0.57452	0.014242
hsa_circ_	-0.93707	0.014245
hsa_circ_	-0.51663	0.014251
hsa_circ_	0.84831	0.014253
hsa_circ_	0.668059	0.014257
hsa_circ_	-0.46097	0.014257
hsa_circ_	1.167265	0.014259
hsa_circ_	1.3934	0.01426
hsa_circ_	-0.48498	0.014264
hsa_circ_	-0.64951	0.014267
hsa_circ_	0.523422	0.014274

hsa_circ_	-0.45523	0.014274
hsa_circ_	-0.57636	0.014281
hsa_circ_	-0.67496	0.014284
hsa_circ_	0.619936	0.014286
hsa_circ_	-0.67309	0.014287
hsa_circ_	-0.89755	0.014287
hsa_circ_	-1.25131	0.014287
hsa_circ_	0.634908	0.014298
hsa_circ_	-0.62441	0.014299
hsa_circ_	-0.62671	0.0143
hsa_circ_	0.557985	0.014302
hsa_circ_	-0.64036	0.014306
hsa_circ_	1.304651	0.014312
hsa_circ_	-0.5742	0.014317
hsa_circ_	0.571112	0.014322
hsa_circ_	-0.54577	0.014329
hsa_circ_	-0.68345	0.01433
hsa_circ_	0.567188	0.014335
hsa_circ_	0.644852	0.014337
hsa_circ_	-0.83753	0.014337
hsa_circ_	-0.84715	0.01434
hsa_circ_	-1.12637	0.014344
hsa_circ_	-0.464	0.014344
hsa_circ_	0.805349	0.014359
hsa_circ_	-1.7389	0.01436
hsa_circ_	0.480309	0.01437
hsa_circ_	0.825942	0.014384
hsa_circ_	-0.578	0.014385
hsa_circ_	0.402394	0.014385
hsa_circ_	-1.08581	0.014386
hsa_circ_	0.43924	0.014387
hsa_circ_	-0.66113	0.014388
hsa_circ_	0.440015	0.014394
hsa_circ_	-0.61409	0.014394
hsa_circ_	-0.70135	0.014396
hsa_circ_	0.731181	0.014408
hsa_circ_	-0.65345	0.014415
hsa_circ_	0.970426	0.014432
hsa_circ_	1.27661	0.014434
hsa_circ_	0.878437	0.01444
hsa_circ_	-1.72295	0.014443
hsa_circ_	-0.59385	0.014446
hsa_circ_	-0.81327	0.014448
hsa_circ_	0.813078	0.014452
hsa_circ_	-0.43305	0.014456
hsa_circ_	1.266669	0.014463
hsa_circ_	-0.54661	0.014473
hsa_circ_	0.811001	0.014488
hsa_circ_	0.511894	0.0145
hsa_circ_	0.679249	0.014502
hsa_circ_	2.075533	0.014514
hsa_circ_	-1.14473	0.014515
hsa_circ_	-0.42855	0.014516
hsa_circ_	1.091494	0.014517

hsa_circ_	0.964218	0.01452
hsa_circ_	1.347968	0.014528
hsa_circ_	1.214239	0.014555
hsa_circ_	-0.93105	0.014558
hsa_circ_	-0.46158	0.014562
hsa_circ_	-0.62404	0.014563
hsa_circ_	-0.72332	0.014578
hsa_circ_	-1.39152	0.014581
hsa_circ_	-0.83188	0.01459
hsa_circ_	0.472962	0.014591
hsa_circ_	-0.43435	0.014593
hsa_circ_	0.409945	0.014596
hsa_circ_	0.961037	0.014597
hsa_circ_	2.103063	0.014608
hsa_circ_	-0.54647	0.01461
hsa_circ_	-1.0302	0.014613
hsa_circ_	-0.5507	0.014614
hsa_circ_	-0.43697	0.014626
hsa_circ_	-0.57116	0.014631
hsa_circ_	0.832698	0.014647
hsa_circ_	-0.57539	0.014649
hsa_circ_	0.727426	0.01465
hsa_circ_	0.612851	0.014657
hsa_circ_	-0.76151	0.014658
hsa_circ_	1.081342	0.014659
hsa_circ_	0.687463	0.014659
hsa_circ_	-0.48147	0.014662
hsa_circ_	-1.71421	0.014672
hsa_circ_	1.630849	0.014675
hsa_circ_	-0.45437	0.014677
hsa_circ_	-0.60818	0.014688
hsa_circ_	-0.5922	0.014697
hsa_circ_	0.560151	0.014713
hsa_circ_	0.71782	0.014721
hsa_circ_	0.827175	0.014731
hsa_circ_	-0.57412	0.014734
hsa_circ_	1.015246	0.014735
hsa_circ_	1.879213	0.01474
hsa_circ_	2.003178	0.014751
hsa_circ_	-0.79588	0.014756
hsa_circ_	-0.50054	0.014768
hsa_circ_	-0.43207	0.014769
hsa_circ_	-0.47903	0.01477
hsa_circ_	0.744804	0.014772
hsa_circ_	-0.83388	0.014775
hsa_circ_	-0.4468	0.014778
hsa_circ_	-0.44997	0.014781
hsa_circ_	0.701313	0.014784
hsa_circ_	-0.88343	0.014784
hsa_circ_	0.465141	0.01479
hsa_circ_	0.954407	0.014796
hsa_circ_	-0.7807	0.014801
hsa_circ_	0.477757	0.014803
hsa_circ_	0.938632	0.014806

hsa_circ_	-0.55171	0.014808
hsa_circ_	-0.51268	0.014827
hsa_circ_	1.403603	0.014833
hsa_circ_	-0.86477	0.014834
hsa_circ_	-0.41534	0.014835
hsa_circ_	-0.65506	0.014837
hsa_circ_	0.669774	0.014841
hsa_circ_	0.86736	0.014842
hsa_circ_	1.005468	0.014843
hsa_circ_	0.77697	0.014848
hsa_circ_	-0.80132	0.014856
hsa_circ_	-0.55442	0.014859
hsa_circ_	-0.55623	0.014859
hsa_circ_	0.749266	0.014862
hsa_circ_	0.55786	0.014863
hsa_circ_	1.204461	0.014863
hsa_circ_	0.436445	0.014863
hsa_circ_	0.632268	0.014872
hsa_circ_	0.770621	0.014876
hsa_circ_	-0.69174	0.01488
hsa_circ_	0.562164	0.014889
hsa_circ_	0.640908	0.014899
hsa_circ_	0.75525	0.014899
hsa_circ_	0.839763	0.014905
hsa_circ_	-0.77734	0.014906
hsa_circ_	1.031065	0.014907
hsa_circ_	-0.73965	0.01491
hsa_circ_	-0.43036	0.014924
hsa_circ_	-0.49172	0.014943
hsa_circ_	-0.81478	0.014954
hsa_circ_	-0.83895	0.014958
hsa_circ_	-0.44667	0.014964
hsa_circ_	-0.77125	0.014968
hsa_circ_	-0.98081	0.014971
hsa_circ_	0.532206	0.014972
hsa_circ_	1.050638	0.014975
hsa_circ_	-0.6914	0.014982
hsa_circ_	-0.47369	0.014982
hsa_circ_	-0.63664	0.014989
hsa_circ_	-0.48321	0.01499
hsa_circ_	-0.48087	0.01499
hsa_circ_	-0.44765	0.014991
hsa_circ_	-0.50983	0.014992
hsa_circ_	-0.6674	0.014992
hsa_circ_	0.450406	0.014992
hsa_circ_	-0.82651	0.015001
hsa_circ_	0.818074	0.015008
hsa_circ_	-0.51495	0.015014
hsa_circ_	-0.82549	0.015014
hsa_circ_	0.468368	0.015017
hsa_circ_	0.927979	0.01502
hsa_circ_	-1.0197	0.015024
hsa_circ_	0.60427	0.015031
hsa_circ_	0.483965	0.015038

hsa_circ_	-1.37397	0.015039
hsa_circ_	-0.82312	0.01504
hsa_circ_	-0.71722	0.015045
hsa_circ_	0.448297	0.015045
hsa_circ_	0.967355	0.015047
hsa_circ_	-1.61115	0.015047
hsa_circ_	0.674981	0.015051
hsa_circ_	-0.52952	0.015052
hsa_circ_	-0.49443	0.015056
hsa_circ_	0.932092	0.015057
hsa_circ_	-1.03203	0.015065
hsa_circ_	0.429889	0.015069
hsa_circ_	-0.64396	0.015074
hsa_circ_	-0.70449	0.015081
hsa_circ_	1.066739	0.01509
hsa_circ_	-0.57525	0.015092
hsa_circ_	0.554355	0.015092
hsa_circ_	0.531674	0.015105
hsa_circ_	-0.73384	0.015116
hsa_circ_	1.107144	0.015133
hsa_circ_	0.437278	0.015137
hsa_circ_	-0.96356	0.015138
hsa_circ_	-0.58068	0.015143
hsa_circ_	-1.07216	0.015145
hsa_circ_	-0.58685	0.015146
hsa_circ_	0.539291	0.015149
hsa_circ_	1.675183	0.015153
hsa_circ_	0.494096	0.015154
hsa_circ_	-0.46753	0.015163
hsa_circ_	-0.49501	0.015164
hsa_circ_	-0.84895	0.015165
hsa_circ_	-0.81385	0.015171
hsa_circ_	0.572845	0.015173
hsa_circ_	0.871624	0.015174
hsa_circ_	0.444586	0.015176
hsa_circ_	1.585638	0.015177
hsa_circ_	0.742568	0.015184
hsa_circ_	0.517567	0.015186
hsa_circ_	1.078287	0.015194
hsa_circ_	-0.76454	0.015196
hsa_circ_	0.605595	0.015203
hsa_circ_	-0.92064	0.015203
hsa_circ_	0.754882	0.015208
hsa_circ_	0.513314	0.015208
hsa_circ_	-0.96377	0.015216
hsa_circ_	0.566969	0.01522
hsa_circ_	2.094037	0.015227
hsa_circ_	-0.74661	0.015234
hsa_circ_	0.61979	0.015234
hsa_circ_	0.534497	0.015241
hsa_circ_	-0.43826	0.015246
hsa_circ_	1.031454	0.015246
hsa_circ_	-0.86408	0.015252
hsa_circ_	1.34296	0.015254

hsa_circ_	0.431205	0.015254
hsa_circ_	0.679504	0.015261
hsa_circ_	-0.48342	0.015264
hsa_circ_	1.77966	0.015264
hsa_circ_	0.662555	0.01527
hsa_circ_	0.487859	0.015271
hsa_circ_	1.524449	0.015273
hsa_circ_	1.51683	0.01528
hsa_circ_	-0.68736	0.015281
hsa_circ_	1.305856	0.015282
hsa_circ_	0.869448	0.015283
hsa_circ_	0.882841	0.015288
hsa_circ_	-1.55263	0.015289
hsa_circ_	-0.79821	0.01529
hsa_circ_	0.708012	0.015297
hsa_circ_	0.431307	0.015297
hsa_circ_	-0.81279	0.015304
hsa_circ_	0.475242	0.015321
hsa_circ_	-0.753	0.015322
hsa_circ_	0.731622	0.015324
hsa_circ_	0.67992	0.015325
hsa_circ_	0.683832	0.015331
hsa_circ_	0.794615	0.015338
hsa_circ_	0.775993	0.015339
hsa_circ_	-0.78912	0.01534
hsa_circ_	0.439905	0.015341
hsa_circ_	0.505054	0.015348
hsa_circ_	-0.86307	0.01535
hsa_circ_	0.726485	0.015359
hsa_circ_	0.626848	0.015363
hsa_circ_	-0.61395	0.015381
hsa_circ_	-0.885	0.015381
hsa_circ_	-0.69739	0.015381
hsa_circ_	-0.69558	0.015383
hsa_circ_	-0.84441	0.015386
hsa_circ_	-0.78763	0.015389
hsa_circ_	0.449013	0.015398
hsa_circ_	-0.55677	0.015403
hsa_circ_	0.500295	0.015404
hsa_circ_	1.980994	0.015407
hsa_circ_	-0.83287	0.015408
hsa_circ_	-0.92388	0.01541
hsa_circ_	-0.57023	0.015419
hsa_circ_	-0.4278	0.015421
hsa_circ_	0.612758	0.015423
hsa_circ_	0.800615	0.015426
hsa_circ_	-1.35188	0.015434
hsa_circ_	1.100416	0.01544
hsa_circ_	-0.54754	0.015442
hsa_circ_	-0.88456	0.015445
hsa_circ_	0.641785	0.015449
hsa_circ_	-0.4777	0.015459
hsa_circ_	0.428386	0.015462
hsa_circ_	1.664085	0.015469

hsa_circ_	0.445757	0.01548
hsa_circ_	-0.46408	0.01548
hsa_circ_	1.253589	0.015484
hsa_circ_	0.751727	0.015488
hsa_circ_	1.251148	0.015489
hsa_circ_	1.834007	0.015494
hsa_circ_	0.440567	0.015496
hsa_circ_	-0.89741	0.015501
hsa_circ_	1.893415	0.015507
hsa_circ_	0.427861	0.015512
hsa_circ_	0.458826	0.015514
hsa_circ_	-1.21999	0.015523
hsa_circ_	0.516977	0.015527
hsa_circ_	-0.59267	0.015543
hsa_circ_	-0.55696	0.015544
hsa_circ_	0.640066	0.015548
hsa_circ_	0.459395	0.015552
hsa_circ_	0.570428	0.015553
hsa_circ_	-0.44481	0.015558
hsa_circ_	-0.45149	0.015571
hsa_circ_	1.112561	0.015571
hsa_circ_	-0.93896	0.01558
hsa_circ_	1.3258	0.015583
hsa_circ_	0.806404	0.015591
hsa_circ_	0.749622	0.015593
hsa_circ_	0.958875	0.015594
hsa_circ_	0.995317	0.015594
hsa_circ_	-0.49499	0.015606
hsa_circ_	0.600162	0.015621
hsa_circ_	0.492402	0.015622
hsa_circ_	-0.55397	0.015624
hsa_circ_	-0.4765	0.015626
hsa_circ_	-0.83531	0.015632
hsa_circ_	-0.44791	0.015637
hsa_circ_	-0.47788	0.01564
hsa_circ_	1.565972	0.01565
hsa_circ_	0.61587	0.015659
hsa_circ_	-0.52874	0.01566
hsa_circ_	0.511611	0.015661
hsa_circ_	1.261294	0.015662
hsa_circ_	1.295875	0.015663
hsa_circ_	-0.58761	0.015663
hsa_circ_	-0.4732	0.015664
hsa_circ_	-0.94353	0.015666
hsa_circ_	0.522334	0.015668
hsa_circ_	0.433978	0.015672
hsa_circ_	-0.48004	0.015674
hsa_circ_	0.856369	0.015675
hsa_circ_	-1.49385	0.015676
hsa_circ_	0.901036	0.015681
hsa_circ_	-0.5307	0.015684
hsa_circ_	-0.42743	0.015687
hsa_circ_	-0.79624	0.015689
hsa_circ_	-1.26439	0.015709

hsa_circ_	-0.49921	0.015712
hsa_circ_	-0.45668	0.015734
hsa_circ_	-2.9173	0.015736
hsa_circ_	0.980299	0.015739
hsa_circ_	0.552597	0.01574
hsa_circ_	1.058877	0.01574
hsa_circ_	-0.42638	0.015743
hsa_circ_	0.631323	0.015751
hsa_circ_	-1.15272	0.015763
hsa_circ_	0.476543	0.015766
hsa_circ_	-0.60051	0.015771
hsa_circ_	1.031997	0.015775
hsa_circ_	0.543527	0.015776
hsa_circ_	-0.83197	0.015785
hsa_circ_	-0.5353	0.015787
hsa_circ_	0.471755	0.015795
hsa_circ_	-0.63846	0.015802
hsa_circ_	-1.30349	0.015809
hsa_circ_	-0.48487	0.01581
hsa_circ_	-0.43351	0.015813
hsa_circ_	-1.16738	0.015823
hsa_circ_	0.703764	0.015824
hsa_circ_	1.048755	0.015825
hsa_circ_	-0.55239	0.015829
hsa_circ_	0.635692	0.015845
hsa_circ_	0.688716	0.015849
hsa_circ_	0.954021	0.015853
hsa_circ_	0.614736	0.015853
hsa_circ_	-0.71001	0.015857
hsa_circ_	-0.91839	0.01586
hsa_circ_	0.507986	0.015875
hsa_circ_	0.826912	0.015884
hsa_circ_	-1.79713	0.015888
hsa_circ_	-0.92839	0.0159
hsa_circ_	0.579418	0.015907
hsa_circ_	0.703442	0.015909
hsa_circ_	0.446947	0.015915
hsa_circ_	-0.4505	0.015917
hsa_circ_	-0.66568	0.015919
hsa_circ_	-0.56058	0.015921
hsa_circ_	-1.25197	0.015924
hsa_circ_	0.671024	0.015928
hsa_circ_	0.632354	0.015928
hsa_circ_	0.493974	0.015936
hsa_circ_	-0.94541	0.015938
hsa_circ_	0.954639	0.01594
hsa_circ_	0.727303	0.015943
hsa_circ_	-0.42524	0.015948
hsa_circ_	0.678485	0.015949
hsa_circ_	-0.44966	0.015955
hsa_circ_	-0.53334	0.015961
hsa_circ_	-0.49366	0.015966
hsa_circ_	-0.61368	0.015968
hsa_circ_	1.519896	0.015971

hsa_circ_	-1.43019	0.015972
hsa_circ_	-1.40761	0.015973
hsa_circ_	-0.58469	0.015991
hsa_circ_	0.515262	0.015993
hsa_circ_	0.430667	0.015995
hsa_circ_	-0.86151	0.015996
hsa_circ_	0.609296	0.016005
hsa_circ_	0.437319	0.016009
hsa_circ_	0.79844	0.01601
hsa_circ_	-0.52564	0.016013
hsa_circ_	-0.47862	0.01602
hsa_circ_	0.409459	0.016024
hsa_circ_	0.538115	0.01603
hsa_circ_	1.021947	0.016036
hsa_circ_	-0.388	0.016041
hsa_circ_	0.727025	0.016041
hsa_circ_	-0.5956	0.016048
hsa_circ_	-0.73469	0.01605
hsa_circ_	0.595774	0.016051
hsa_circ_	0.431328	0.01606
hsa_circ_	1.241491	0.016061
hsa_circ_	0.765886	0.016063
hsa_circ_	-0.82071	0.016065
hsa_circ_	0.65577	0.016072
hsa_circ_	0.647432	0.016072
hsa_circ_	0.441087	0.01608
hsa_circ_	0.547528	0.016089
hsa_circ_	-0.52274	0.016091
hsa_circ_	-0.64971	0.016092
hsa_circ_	-0.55474	0.016101
hsa_circ_	-0.64293	0.016104
hsa_circ_	-1.22684	0.016107
hsa_circ_	-0.5628	0.016107
hsa_circ_	-0.72161	0.016109
hsa_circ_	-0.82284	0.016111
hsa_circ_	0.461484	0.01612
hsa_circ_	1.12089	0.016124
hsa_circ_	-0.8702	0.016131
hsa_circ_	0.692174	0.016132
hsa_circ_	-1.05654	0.016134
hsa_circ_	-0.87223	0.016138
hsa_circ_	0.469858	0.01614
hsa_circ_	0.514584	0.016146
hsa_circ_	-0.63015	0.016149
hsa_circ_	-0.46903	0.016152
hsa_circ_	-0.73501	0.016153
hsa_circ_	0.647553	0.016163
hsa_circ_	0.863332	0.016166
hsa_circ_	-0.77159	0.016177
hsa_circ_	-0.55026	0.016181
hsa_circ_	-0.98771	0.016187
hsa_circ_	-0.91241	0.016188
hsa_circ_	0.94773	0.016192
hsa_circ_	-0.99769	0.016194

hsa_circ_	0.701481	0.016195
hsa_circ_	1.023304	0.016197
hsa_circ_	-0.60996	0.016198
hsa_circ_	0.468427	0.0162
hsa_circ_	0.473973	0.016205
hsa_circ_	0.69959	0.016208
hsa_circ_	-1.42055	0.016209
hsa_circ_	0.77975	0.016216
hsa_circ_	1.0885	0.016217
hsa_circ_	-0.49542	0.016219
hsa_circ_	-0.77875	0.016221
hsa_circ_	0.626607	0.016222
hsa_circ_	0.419024	0.016225
hsa_circ_	-0.81844	0.016226
hsa_circ_	0.551335	0.016228
hsa_circ_	0.821532	0.016232
hsa_circ_	-0.48561	0.016233
hsa_circ_	-0.6723	0.016247
hsa_circ_	0.71389	0.016253
hsa_circ_	0.452054	0.016254
hsa_circ_	-1.16954	0.016268
hsa_circ_	0.51963	0.016272
hsa_circ_	-0.93146	0.016283
hsa_circ_	-0.68685	0.016285
hsa_circ_	0.59187	0.01629
hsa_circ_	-0.49684	0.016291
hsa_circ_	-0.67398	0.016297
hsa_circ_	0.478029	0.016297
hsa_circ_	0.750224	0.016314
hsa_circ_	1.498774	0.016317
hsa_circ_	0.562195	0.016331
hsa_circ_	-0.59424	0.016332
hsa_circ_	0.590695	0.016338
hsa_circ_	0.552473	0.016338
hsa_circ_	-0.95573	0.016343
hsa_circ_	0.573161	0.016344
hsa_circ_	-1.24025	0.016345
hsa_circ_	0.482963	0.016346
hsa_circ_	-0.96599	0.016347
hsa_circ_	-0.42891	0.016348
hsa_circ_	-0.49119	0.01635
hsa_circ_	1.39234	0.016361
hsa_circ_	-0.53612	0.016362
hsa_circ_	-0.74065	0.016363
hsa_circ_	0.75461	0.016369
hsa_circ_	-1.01765	0.016371
hsa_circ_	-0.71476	0.016372
hsa_circ_	0.660587	0.016381
hsa_circ_	0.585264	0.016382
hsa_circ_	-0.49143	0.01639
hsa_circ_	0.681634	0.016394
hsa_circ_	-0.59837	0.016395
hsa_circ_	-0.72901	0.016398
hsa_circ_	0.928474	0.016399

hsa_circ_	-0.49112	0.016409
hsa_circ_	1.447852	0.016412
hsa_circ_	-0.42639	0.016412
hsa_circ_	-0.42007	0.016415
hsa_circ_	0.718167	0.016417
hsa_circ_	0.432163	0.016418
hsa_circ_	-0.95273	0.016418
hsa_circ_	-0.43666	0.016422
hsa_circ_	0.743442	0.016423
hsa_circ_	0.57194	0.016423
hsa_circ_	0.701781	0.016424
hsa_circ_	-0.82299	0.016429
hsa_circ_	-0.97814	0.016436
hsa_circ_	-1.23711	0.016443
hsa_circ_	0.466051	0.01645
hsa_circ_	1.498595	0.016453
hsa_circ_	-0.85701	0.016459
hsa_circ_	-1.63061	0.016474
hsa_circ_	0.570789	0.016476
hsa_circ_	-0.69203	0.016477
hsa_circ_	-0.76874	0.01649
hsa_circ_	-0.44341	0.016497
hsa_circ_	0.771849	0.016501
hsa_circ_	-0.80205	0.016501
hsa_circ_	-0.45209	0.016505
hsa_circ_	-0.88607	0.016521
hsa_circ_	-0.51539	0.016525
hsa_circ_	-0.85654	0.016532
hsa_circ_	0.660489	0.016536
hsa_circ_	-1.89745	0.016537
hsa_circ_	1.127471	0.016537
hsa_circ_	-0.48835	0.01654
hsa_circ_	0.838057	0.016552
hsa_circ_	1.021167	0.016553
hsa_circ_	-0.96789	0.016558
hsa_circ_	-0.7782	0.016567
hsa_circ_	0.585798	0.016569
hsa_circ_	-0.41281	0.016571
hsa_circ_	0.57958	0.016572
hsa_circ_	-0.56989	0.016575
hsa_circ_	1.086596	0.016578
hsa_circ_	-0.89406	0.01658
hsa_circ_	0.79392	0.016582
hsa_circ_	0.77494	0.016582
hsa_circ_	-0.88984	0.016584
hsa_circ_	-0.83714	0.016586
hsa_circ_	0.400788	0.016592
hsa_circ_	-0.54104	0.016599
hsa_circ_	0.816879	0.0166
hsa_circ_	0.477724	0.016601
hsa_circ_	0.456149	0.016603
hsa_circ_	-0.48933	0.016627
hsa_circ_	-0.77109	0.016631
hsa_circ_	-0.83546	0.016632

hsa_circ_	0.471093	0.01664
hsa_circ_	-0.93153	0.016642
hsa_circ_	1.091867	0.016646
hsa_circ_	0.411705	0.016648
hsa_circ_	0.864416	0.01666
hsa_circ_	-1.11727	0.016666
hsa_circ_	0.422808	0.01667
hsa_circ_	0.515073	0.016672
hsa_circ_	0.470367	0.016676
hsa_circ_	-0.61133	0.016686
hsa_circ_	0.541854	0.016694
hsa_circ_	0.68303	0.016697
hsa_circ_	0.912082	0.016702
hsa_circ_	-0.56056	0.016703
hsa_circ_	-0.64119	0.016705
hsa_circ_	-0.75391	0.016705
hsa_circ_	1.113156	0.016712
hsa_circ_	0.474599	0.016715
hsa_circ_	-0.48219	0.016716
hsa_circ_	-0.56823	0.01672
hsa_circ_	-0.65517	0.01672
hsa_circ_	-0.77542	0.016732
hsa_circ_	0.422767	0.016737
hsa_circ_	-0.72975	0.016738
hsa_circ_	-1.38124	0.016744
hsa_circ_	0.55256	0.016744
hsa_circ_	0.623557	0.016748
hsa_circ_	-0.40549	0.016756
hsa_circ_	0.843536	0.016756
hsa_circ_	-0.77822	0.016761
hsa_circ_	1.17297	0.016764
hsa_circ_	-0.43295	0.016765
hsa_circ_	0.545264	0.016766
hsa_circ_	1.318239	0.016769
hsa_circ_	0.653887	0.01677
hsa_circ_	0.867149	0.016771
hsa_circ_	-0.79669	0.016772
hsa_circ_	-0.41184	0.016776
hsa_circ_	0.557696	0.016785
hsa_circ_	-0.833	0.016787
hsa_circ_	1.194176	0.016789
hsa_circ_	-1.0309	0.016791
hsa_circ_	0.622503	0.016806
hsa_circ_	1.013738	0.016807
hsa_circ_	-0.5627	0.016808
hsa_circ_	0.410994	0.016809
hsa_circ_	1.148184	0.016817
hsa_circ_	-0.52678	0.016819
hsa_circ_	0.503156	0.016821
hsa_circ_	0.654174	0.016823
hsa_circ_	0.929472	0.016825
hsa_circ_	1.08988	0.016828
hsa_circ_	0.618599	0.016832
hsa_circ_	0.734081	0.016834

hsa_circ_	0.69424	0.016837
hsa_circ_	0.683044	0.016839
hsa_circ_	-0.43786	0.016851
hsa_circ_	1.467075	0.016851
hsa_circ_	-0.49148	0.016857
hsa_circ_	0.558045	0.016863
hsa_circ_	1.191191	0.016864
hsa_circ_	0.574353	0.016866
hsa_circ_	-0.58535	0.01687
hsa_circ_	-0.40419	0.016872
hsa_circ_	-0.68968	0.016875
hsa_circ_	1.424079	0.016884
hsa_circ_	-0.61524	0.016905
hsa_circ_	0.914042	0.016918
hsa_circ_	0.996287	0.016923
hsa_circ_	-0.83038	0.016923
hsa_circ_	0.690312	0.016926
hsa_circ_	-0.46131	0.016928
hsa_circ_	0.528146	0.016929
hsa_circ_	1.585862	0.016929
hsa_circ_	-0.54033	0.016931
hsa_circ_	1.191344	0.016937
hsa_circ_	0.624249	0.016937
hsa_circ_	-0.67393	0.016946
hsa_circ_	0.696318	0.016948
hsa_circ_	0.912817	0.016949
hsa_circ_	0.546975	0.016949
hsa_circ_	0.536235	0.016949
hsa_circ_	-0.79134	0.016955
hsa_circ_	1.517769	0.016961
hsa_circ_	-0.57291	0.01697
hsa_circ_	-1.55625	0.016971
hsa_circ_	-0.65039	0.016971
hsa_circ_	0.425473	0.016974
hsa_circ_	-0.45319	0.016977
hsa_circ_	0.526756	0.01698
hsa_circ_	1.011194	0.016982
hsa_circ_	0.43352	0.016999
hsa_circ_	0.517733	0.017
hsa_circ_	0.592289	0.017002
hsa_circ_	0.574576	0.017017
hsa_circ_	0.537132	0.017026
hsa_circ_	0.891039	0.017028
hsa_circ_	-0.85366	0.017037
hsa_circ_	-0.53863	0.017042
hsa_circ_	0.51262	0.017052
hsa_circ_	-0.64382	0.017052
hsa_circ_	-0.72636	0.017066
hsa_circ_	-0.82364	0.017076
hsa_circ_	1.496093	0.017088
hsa_circ_	0.638558	0.017096
hsa_circ_	-0.67032	0.017107
hsa_circ_	-0.76585	0.017109
hsa_circ_	0.453505	0.017112

hsa_circ_	0.399915	0.017115
hsa_circ_	0.667792	0.017129
hsa_circ_	-0.82751	0.017133
hsa_circ_	-0.69225	0.017135
hsa_circ_	-0.44643	0.017138
hsa_circ_	1.511003	0.017138
hsa_circ_	-0.69377	0.017138
hsa_circ_	-0.83987	0.017144
hsa_circ_	0.406371	0.017145
hsa_circ_	-0.47339	0.017169
hsa_circ_	0.58858	0.017178
hsa_circ_	-0.39577	0.017182
hsa_circ_	-1.0951	0.017205
hsa_circ_	1.003785	0.017213
hsa_circ_	1.107986	0.017217
hsa_circ_	0.65354	0.017225
hsa_circ_	-0.77782	0.017226
hsa_circ_	-0.67876	0.017231
hsa_circ_	0.574138	0.017236
hsa_circ_	0.576914	0.017242
hsa_circ_	-0.47742	0.017246
hsa_circ_	-0.53356	0.017264
hsa_circ_	0.468921	0.017264
hsa_circ_	-0.89603	0.017277
hsa_circ_	0.825461	0.017278
hsa_circ_	-0.45132	0.017281
hsa_circ_	0.628672	0.017286
hsa_circ_	0.399209	0.017295
hsa_circ_	0.416322	0.017298
hsa_circ_	0.509527	0.017298
hsa_circ_	0.456855	0.017309
hsa_circ_	-0.8083	0.01731
hsa_circ_	0.823924	0.017314
hsa_circ_	-0.53024	0.017323
hsa_circ_	0.750786	0.017326
hsa_circ_	0.477822	0.017336
hsa_circ_	0.625134	0.017341
hsa_circ_	-1.06547	0.017349
hsa_circ_	-0.58938	0.017353
hsa_circ_	-0.47308	0.017358
hsa_circ_	0.914456	0.017363
hsa_circ_	1.216051	0.017371
hsa_circ_	0.515865	0.017372
hsa_circ_	-0.60594	0.017377
hsa_circ_	0.584981	0.017378
hsa_circ_	0.579389	0.017378
hsa_circ_	0.915953	0.017384
hsa_circ_	0.556095	0.017391
hsa_circ_	-0.70026	0.017398
hsa_circ_	-0.59408	0.017404
hsa_circ_	-1.1707	0.017407
hsa_circ_	-0.41157	0.017407
hsa_circ_	-0.47345	0.017414
hsa_circ_	-1.0414	0.017414

hsa_circ_	0.461026	0.017415
hsa_circ_	-0.94173	0.017418
hsa_circ_	-1.23903	0.01742
hsa_circ_	0.626783	0.017423
hsa_circ_	0.400119	0.017424
hsa_circ_	0.639994	0.017433
hsa_circ_	-1.70924	0.017436
hsa_circ_	-0.77357	0.017444
hsa_circ_	-0.5159	0.017446
hsa_circ_	-0.98549	0.017449
hsa_circ_	-0.68544	0.017464
hsa_circ_	0.501074	0.017468
hsa_circ_	0.477921	0.017471
hsa_circ_	0.56211	0.01748
hsa_circ_	0.651741	0.01748
hsa_circ_	-0.842	0.017484
hsa_circ_	-1.80853	0.017489
hsa_circ_	0.572774	0.017494
hsa_circ_	0.542208	0.017497
hsa_circ_	-0.4765	0.017498
hsa_circ_	-0.46893	0.0175
hsa_circ_	-0.70573	0.017502
hsa_circ_	0.486511	0.017516
hsa_circ_	-0.48116	0.017522
hsa_circ_	-0.83702	0.017528
hsa_circ_	0.466025	0.017529
hsa_circ_	-0.52277	0.017534
hsa_circ_	-0.56692	0.017536
hsa_circ_	0.47	0.017544
hsa_circ_	0.721599	0.017546
hsa_circ_	0.793616	0.017548
hsa_circ_	-0.86892	0.017555
hsa_circ_	0.701522	0.017556
hsa_circ_	0.642364	0.017557
hsa_circ_	0.639607	0.017559
hsa_circ_	-0.73224	0.017563
hsa_circ_	0.511366	0.017563
hsa_circ_	-0.43146	0.017564
hsa_circ_	-0.67479	0.017565
hsa_circ_	1.081687	0.017566
hsa_circ_	0.856381	0.017567
hsa_circ_	-0.42496	0.017569
hsa_circ_	-0.4962	0.01758
hsa_circ_	0.536603	0.017584
hsa_circ_	0.768521	0.01759
hsa_circ_	1.530009	0.01759
hsa_circ_	0.482859	0.0176
hsa_circ_	-0.56542	0.017613
hsa_circ_	-0.66413	0.017626
hsa_circ_	0.437969	0.017626
hsa_circ_	-0.96812	0.017628
hsa_circ_	-0.44923	0.017629
hsa_circ_	0.706254	0.017641
hsa_circ_	-0.52538	0.017644

hsa_circ_	0.590853	0.017645
hsa_circ_	0.577196	0.01765
hsa_circ_	-0.43388	0.017653
hsa_circ_	-2.04118	0.017655
hsa_circ_	1.183339	0.017661
hsa_circ_	0.897305	0.017663
hsa_circ_	-0.51102	0.017668
hsa_circ_	1.076599	0.017673
hsa_circ_	-0.71333	0.01768
hsa_circ_	-0.87718	0.017687
hsa_circ_	-0.45833	0.017689
hsa_circ_	-0.60383	0.01769
hsa_circ_	-0.74172	0.017701
hsa_circ_	0.610073	0.017708
hsa_circ_	0.450072	0.017713
hsa_circ_	-0.54855	0.017715
hsa_circ_	0.577315	0.017719
hsa_circ_	-0.76893	0.017725
hsa_circ_	0.502492	0.017727
hsa_circ_	-0.87387	0.017731
hsa_circ_	1.152682	0.017744
hsa_circ_	-0.47115	0.017747
hsa_circ_	0.428742	0.017764
hsa_circ_	0.519557	0.017765
hsa_circ_	-0.61224	0.017767
hsa_circ_	0.432133	0.017773
hsa_circ_	1.576898	0.017777
hsa_circ_	0.477778	0.017778
hsa_circ_	0.541969	0.017783
hsa_circ_	-0.78206	0.017783
hsa_circ_	0.491127	0.017786
hsa_circ_	-0.39741	0.017788
hsa_circ_	-0.64561	0.017792
hsa_circ_	0.91895	0.017795
hsa_circ_	-0.55362	0.017803
hsa_circ_	-1.374	0.017811
hsa_circ_	-0.5299	0.017818
hsa_circ_	1.394881	0.017827
hsa_circ_	-0.69203	0.017835
hsa_circ_	-0.6872	0.017838
hsa_circ_	-0.43264	0.01784
hsa_circ_	-0.50273	0.01784
hsa_circ_	0.660945	0.017846
hsa_circ_	0.540457	0.017848
hsa_circ_	-0.46427	0.01785
hsa_circ_	0.647653	0.017853
hsa_circ_	0.448185	0.017855
hsa_circ_	0.880682	0.017856
hsa_circ_	-2.59722	0.017857
hsa_circ_	-0.50657	0.017859
hsa_circ_	0.672873	0.017861
hsa_circ_	0.586795	0.017867
hsa_circ_	-0.49964	0.017874
hsa_circ_	0.576505	0.017875

hsa_circ_	0.726236	0.017876
hsa_circ_	0.516074	0.017882
hsa_circ_	-0.51491	0.017893
hsa_circ_	-0.71574	0.017893
hsa_circ_	1.290461	0.017894
hsa_circ_	1.538345	0.017898
hsa_circ_	0.63507	0.017903
hsa_circ_	0.772511	0.017904
hsa_circ_	0.49308	0.017906
hsa_circ_	-1.2058	0.017911
hsa_circ_	-0.6228	0.017911
hsa_circ_	0.557866	0.017914
hsa_circ_	0.564327	0.017916
hsa_circ_	0.69406	0.017917
hsa_circ_	-0.51133	0.017918
hsa_circ_	1.311881	0.017929
hsa_circ_	-0.43797	0.017933
hsa_circ_	-0.41325	0.017936
hsa_circ_	1.010033	0.017953
hsa_circ_	0.425146	0.017957
hsa_circ_	-0.77385	0.017959
hsa_circ_	0.551174	0.01796
hsa_circ_	-0.72832	0.01797
hsa_circ_	-0.4214	0.017971
hsa_circ_	-0.59568	0.017977
hsa_circ_	0.661376	0.017979
hsa_circ_	-0.61027	0.017983
hsa_circ_	0.551407	0.017989
hsa_circ_	0.855631	0.01799
hsa_circ_	-1.12183	0.01799
hsa_circ_	1.13029	0.017999
hsa_circ_	1.051216	0.018003
hsa_circ_	0.66623	0.018014
hsa_circ_	-0.53231	0.018031
hsa_circ_	0.553677	0.018039
hsa_circ_	0.492319	0.018043
hsa_circ_	0.481442	0.018048
hsa_circ_	-1.71434	0.018055
hsa_circ_	-0.50264	0.01806
hsa_circ_	-0.70514	0.018074
hsa_circ_	-0.54059	0.018074
hsa_circ_	-1.03264	0.01808
hsa_circ_	0.645671	0.018084
hsa_circ_	0.480754	0.018086
hsa_circ_	-0.46454	0.018092
hsa_circ_	1.237384	0.018096
hsa_circ_	0.501683	0.018104
hsa_circ_	-0.88113	0.018125
hsa_circ_	1.522035	0.018132
hsa_circ_	1.105766	0.018138
hsa_circ_	1.206537	0.01814
hsa_circ_	0.46601	0.018141
hsa_circ_	-0.52469	0.018142
hsa_circ_	0.493121	0.018145

hsa_circ_	-0.56751	0.01815
hsa_circ_	-0.55865	0.018158
hsa_circ_	0.743985	0.018158
hsa_circ_	0.451565	0.018162
hsa_circ_	0.903949	0.018175
hsa_circ_	0.470254	0.018177
hsa_circ_	-1.27364	0.018177
hsa_circ_	0.719066	0.018178
hsa_circ_	0.652287	0.018182
hsa_circ_	0.825396	0.018183
hsa_circ_	-0.45518	0.018187
hsa_circ_	-1.38805	0.018199
hsa_circ_	0.723181	0.018211
hsa_circ_	0.513329	0.018232
hsa_circ_	0.475871	0.018232
hsa_circ_	0.618066	0.018233
hsa_circ_	-0.77421	0.018237
hsa_circ_	-0.47083	0.01824
hsa_circ_	-1.43007	0.018248
hsa_circ_	-0.75986	0.018248
hsa_circ_	1.139401	0.018253
hsa_circ_	0.449433	0.018258
hsa_circ_	0.669064	0.018269
hsa_circ_	0.408616	0.018277
hsa_circ_	1.312463	0.018281
hsa_circ_	-0.45802	0.018288
hsa_circ_	-0.38633	0.01829
hsa_circ_	0.666094	0.018293
hsa_circ_	-0.52654	0.018297
hsa_circ_	0.494319	0.018298
hsa_circ_	-0.98187	0.0183
hsa_circ_	0.829435	0.018301
hsa_circ_	-0.55566	0.018304
hsa_circ_	-1.24079	0.018307
hsa_circ_	0.708783	0.018317
hsa_circ_	0.552924	0.018319
hsa_circ_	0.414986	0.018321
hsa_circ_	0.518729	0.018326
hsa_circ_	-0.44633	0.018334
hsa_circ_	0.737585	0.018345
hsa_circ_	-1.20822	0.018345
hsa_circ_	-0.99703	0.018345
hsa_circ_	-0.42076	0.018345
hsa_circ_	0.581316	0.018351
hsa_circ_	-0.74257	0.018354
hsa_circ_	0.448931	0.018365
hsa_circ_	0.481551	0.01837
hsa_circ_	-0.45805	0.018372
hsa_circ_	-0.50367	0.018373
hsa_circ_	0.432541	0.018379
hsa_circ_	0.7999	0.018387
hsa_circ_	0.470744	0.018402
hsa_circ_	1.140517	0.018402
hsa_circ_	2.431925	0.018404

hsa_circ_	0.46803	0.018404
hsa_circ_	-0.79401	0.01841
hsa_circ_	0.483615	0.018412
hsa_circ_	0.779526	0.018412
hsa_circ_	0.478232	0.018412
hsa_circ_	-0.48139	0.018419
hsa_circ_	1.043393	0.018419
hsa_circ_	1.014849	0.018434
hsa_circ_	-0.44462	0.018448
hsa_circ_	0.422985	0.018457
hsa_circ_	0.571794	0.018458
hsa_circ_	1.40683	0.018468
hsa_circ_	0.406403	0.018472
hsa_circ_	0.415752	0.01848
hsa_circ_	0.659279	0.018481
hsa_circ_	0.858744	0.018483
hsa_circ_	0.48424	0.018488
hsa_circ_	0.402671	0.01849
hsa_circ_	0.598406	0.018493
hsa_circ_	1.785298	0.018507
hsa_circ_	0.403031	0.01851
hsa_circ_	-0.56956	0.018511
hsa_circ_	0.64003	0.018522
hsa_circ_	-2.54899	0.018524
hsa_circ_	-0.83282	0.018528
hsa_circ_	-0.72683	0.018531
hsa_circ_	0.590611	0.018531
hsa_circ_	-0.62699	0.018534
hsa_circ_	1.18507	0.018536
hsa_circ_	-0.47822	0.018546
hsa_circ_	-0.39024	0.018553
hsa_circ_	-0.64598	0.018557
hsa_circ_	0.816027	0.018562
hsa_circ_	0.505436	0.018564
hsa_circ_	-0.50702	0.018568
hsa_circ_	-0.54046	0.018574
hsa_circ_	-0.41657	0.018575
hsa_circ_	-0.95704	0.018576
hsa_circ_	-0.62516	0.018579
hsa_circ_	-1.35723	0.01858
hsa_circ_	-0.50668	0.018582
hsa_circ_	-0.53775	0.018587
hsa_circ_	-1.17475	0.018605
hsa_circ_	0.45509	0.018605
hsa_circ_	0.780171	0.018614
hsa_circ_	0.602139	0.018619
hsa_circ_	-0.65828	0.018629
hsa_circ_	0.488616	0.018629
hsa_circ_	0.434877	0.018629
hsa_circ_	-0.38674	0.018633
hsa_circ_	0.38084	0.018649
hsa_circ_	1.115881	0.018654
hsa_circ_	-0.72154	0.018661
hsa_circ_	0.481207	0.018667

hsa_circ_	0.590679	0.018673
hsa_circ_	0.9836	0.018679
hsa_circ_	-0.68517	0.018681
hsa_circ_	1.497932	0.018689
hsa_circ_	-0.50526	0.018696
hsa_circ_	0.505535	0.018708
hsa_circ_	0.568548	0.01871
hsa_circ_	0.735197	0.018711
hsa_circ_	0.725436	0.018714
hsa_circ_	0.614386	0.018714
hsa_circ_	-0.52502	0.018715
hsa_circ_	0.620331	0.018715
hsa_circ_	-0.82054	0.018718
hsa_circ_	0.744799	0.018719
hsa_circ_	0.452988	0.01872
hsa_circ_	0.559566	0.018722
hsa_circ_	-0.81085	0.018739
hsa_circ_	-0.58113	0.018752
hsa_circ_	0.576398	0.01876
hsa_circ_	-0.64932	0.018764
hsa_circ_	0.432718	0.018769
hsa_circ_	-0.44818	0.018771
hsa_circ_	-0.513	0.018772
hsa_circ_	0.657372	0.018776
hsa_circ_	-1.68562	0.018777
hsa_circ_	0.401458	0.018788
hsa_circ_	-0.72435	0.018793
hsa_circ_	-0.6778	0.018794
hsa_circ_	-0.54499	0.018799
hsa_circ_	-0.59353	0.018802
hsa_circ_	-0.88162	0.018802
hsa_circ_	0.767075	0.018821
hsa_circ_	1.018438	0.018824
hsa_circ_	-0.74302	0.018828
hsa_circ_	0.894295	0.018829
hsa_circ_	-0.71747	0.018834
hsa_circ_	-0.44334	0.018839
hsa_circ_	-0.61115	0.018842
hsa_circ_	0.521969	0.018846
hsa_circ_	1.207669	0.01885
hsa_circ_	-1.04675	0.01886
hsa_circ_	1.098895	0.018863
hsa_circ_	0.600785	0.018865
hsa_circ_	-0.77796	0.018873
hsa_circ_	0.772095	0.018874
hsa_circ_	0.567543	0.018874
hsa_circ_	-0.45234	0.018887
hsa_circ_	-0.8305	0.018888
hsa_circ_	-0.50028	0.018891
hsa_circ_	-0.59526	0.018898
hsa_circ_	-0.63246	0.018917
hsa_circ_	-0.61198	0.018922
hsa_circ_	-0.68476	0.018924
hsa_circ_	-0.59619	0.01893

hsa_circ_	-0.49346	0.018935
hsa_circ_	0.608344	0.01894
hsa_circ_	0.546977	0.018941
hsa_circ_	-0.42474	0.018949
hsa_circ_	0.539903	0.018955
hsa_circ_	0.443121	0.018958
hsa_circ_	-0.56981	0.018959
hsa_circ_	-1.16318	0.018972
hsa_circ_	-1.01158	0.018975
hsa_circ_	0.57905	0.018976
hsa_circ_	-0.64995	0.01898
hsa_circ_	-0.75787	0.018988
hsa_circ_	1.201853	0.018991
hsa_circ_	-0.47217	0.018995
hsa_circ_	-0.40612	0.018997
hsa_circ_	-0.54558	0.019002
hsa_circ_	0.45554	0.019006
hsa_circ_	0.463283	0.01901
hsa_circ_	0.918163	0.019012
hsa_circ_	-0.5558	0.019015
hsa_circ_	0.691561	0.019021
hsa_circ_	-0.87407	0.019022
hsa_circ_	-0.82409	0.019026
hsa_circ_	0.515385	0.019027
hsa_circ_	0.720957	0.019041
hsa_circ_	0.569631	0.019047
hsa_circ_	0.677036	0.019047
hsa_circ_	-0.55041	0.019047
hsa_circ_	0.890351	0.019048
hsa_circ_	-0.89194	0.019048
hsa_circ_	-0.60891	0.019049
hsa_circ_	1.028828	0.019051
hsa_circ_	-0.44103	0.019056
hsa_circ_	-0.91537	0.019061
hsa_circ_	-0.70771	0.019067
hsa_circ_	0.682566	0.019069
hsa_circ_	-0.84091	0.019073
hsa_circ_	0.507605	0.019084
hsa_circ_	1.365358	0.019088
hsa_circ_	-1.17707	0.019092
hsa_circ_	0.647523	0.019094
hsa_circ_	-0.74794	0.019095
hsa_circ_	-0.39863	0.019098
hsa_circ_	-0.38527	0.019102
hsa_circ_	-1.3148	0.019103
hsa_circ_	0.753118	0.019104
hsa_circ_	0.635991	0.019105
hsa_circ_	-0.7738	0.019109
hsa_circ_	-0.48126	0.01911
hsa_circ_	-0.82247	0.019111
hsa_circ_	-0.48717	0.019111
hsa_circ_	0.521377	0.019116
hsa_circ_	0.878517	0.01915
hsa_circ_	-0.85423	0.019156

hsa_circ_	0.698018	0.019164
hsa_circ_	0.516364	0.019172
hsa_circ_	-0.49345	0.019177
hsa_circ_	-0.43206	0.019179
hsa_circ_	0.536073	0.019181
hsa_circ_	-0.91991	0.019183
hsa_circ_	1.223908	0.019194
hsa_circ_	-0.51198	0.019201
hsa_circ_	-1.91344	0.019202
hsa_circ_	1.260681	0.019203
hsa_circ_	-1.28194	0.019211
hsa_circ_	-0.76865	0.019215
hsa_circ_	1.100752	0.01922
hsa_circ_	1.965929	0.019225
hsa_circ_	-2.81631	0.019228
hsa_circ_	-0.78091	0.019229
hsa_circ_	-0.70215	0.01923
hsa_circ_	-0.65685	0.01924
hsa_circ_	0.630733	0.019244
hsa_circ_	-0.7505	0.01925
hsa_circ_	0.685109	0.019251
hsa_circ_	-0.71612	0.019258
hsa_circ_	-0.88306	0.019258
hsa_circ_	-0.66977	0.019268
hsa_circ_	0.866316	0.019272
hsa_circ_	-0.83601	0.019274
hsa_circ_	0.451762	0.019276
hsa_circ_	0.667741	0.019279
hsa_circ_	1.845764	0.019285
hsa_circ_	-0.72458	0.019286
hsa_circ_	-0.4462	0.019294
hsa_circ_	0.50705	0.019301
hsa_circ_	0.758967	0.019305
hsa_circ_	0.726128	0.019312
hsa_circ_	-0.64085	0.019322
hsa_circ_	-0.81622	0.019327
hsa_circ_	0.704266	0.019332
hsa_circ_	0.440185	0.019335
hsa_circ_	0.929199	0.019338
hsa_circ_	0.733696	0.019339
hsa_circ_	0.409689	0.019341
hsa_circ_	-0.52267	0.019352
hsa_circ_	0.551264	0.019355
hsa_circ_	1.178134	0.019356
hsa_circ_	1.47902	0.019358
hsa_circ_	-0.71084	0.019364
hsa_circ_	-0.42917	0.019378
hsa_circ_	-0.41476	0.019379
hsa_circ_	-0.74533	0.019384
hsa_circ_	0.616978	0.019387
hsa_circ_	-1.48372	0.019394
hsa_circ_	0.905962	0.019397
hsa_circ_	-0.62744	0.019398
hsa_circ_	-0.87214	0.019414

hsa_circ_	1.020611	0.019416
hsa_circ_	0.980016	0.01942
hsa_circ_	-2.75958	0.019421
hsa_circ_	0.531799	0.019426
hsa_circ_	-0.7222	0.019428
hsa_circ_	-1.02229	0.019431
hsa_circ_	0.732137	0.01945
hsa_circ_	0.875274	0.019454
hsa_circ_	-0.79846	0.019454
hsa_circ_	-0.49725	0.019472
hsa_circ_	0.884709	0.019478
hsa_circ_	-0.57124	0.019478
hsa_circ_	0.67774	0.019479
hsa_circ_	0.408096	0.019482
hsa_circ_	-0.46316	0.019483
hsa_circ_	0.528941	0.019488
hsa_circ_	-0.46316	0.019489
hsa_circ_	-0.39104	0.019505
hsa_circ_	-0.58893	0.019507
hsa_circ_	-0.97088	0.019507
hsa_circ_	0.736394	0.019521
hsa_circ_	0.383938	0.019527
hsa_circ_	0.59452	0.019528
hsa_circ_	-0.50502	0.019531
hsa_circ_	-0.75293	0.019532
hsa_circ_	0.637391	0.019542
hsa_circ_	-0.60537	0.019546
hsa_circ_	0.48831	0.019552
hsa_circ_	1.991368	0.019554
hsa_circ_	-1.0498	0.019557
hsa_circ_	0.559878	0.019561
hsa_circ_	-0.63455	0.019566
hsa_circ_	0.461944	0.019581
hsa_circ_	0.554603	0.019581
hsa_circ_	-0.54631	0.019587
hsa_circ_	0.973479	0.019589
hsa_circ_	-0.45619	0.019592
hsa_circ_	0.748429	0.019598
hsa_circ_	0.594041	0.019606
hsa_circ_	-1.04764	0.019632
hsa_circ_	-0.47597	0.019637
hsa_circ_	0.490022	0.019642
hsa_circ_	2.501909	0.019645
hsa_circ_	-1.14487	0.019646
hsa_circ_	-0.89153	0.01965
hsa_circ_	0.510396	0.019651
hsa_circ_	-1.06354	0.019658
hsa_circ_	-0.73993	0.01966
hsa_circ_	0.896355	0.01966
hsa_circ_	0.606444	0.019665
hsa_circ_	-0.70095	0.019669
hsa_circ_	0.516788	0.019674
hsa_circ_	0.685145	0.019674
hsa_circ_	-0.44477	0.019677

hsa_circ_	0.524503	0.019683
hsa_circ_	-0.75782	0.019684
hsa_circ_	0.430912	0.019686
hsa_circ_	-0.50556	0.019692
hsa_circ_	0.822173	0.019695
hsa_circ_	-0.45225	0.019699
hsa_circ_	-0.44039	0.019702
hsa_circ_	-0.52089	0.019709
hsa_circ_	0.486904	0.019719
hsa_circ_	0.518807	0.019724
hsa_circ_	1.324722	0.019742
hsa_circ_	-0.62721	0.019747
hsa_circ_	-1.5323	0.019747
hsa_circ_	-0.39418	0.019749
hsa_circ_	0.560109	0.019752
hsa_circ_	-0.44475	0.019776
hsa_circ_	0.908907	0.019779
hsa_circ_	0.549022	0.019792
hsa_circ_	-1.01893	0.019793
hsa_circ_	-0.4008	0.019794
hsa_circ_	-1.67557	0.019799
hsa_circ_	-0.46928	0.019804
hsa_circ_	-0.4281	0.019807
hsa_circ_	-0.45799	0.019808
hsa_circ_	0.629705	0.019809
hsa_circ_	1.034481	0.019811
hsa_circ_	-0.80538	0.019812
hsa_circ_	-0.5089	0.01982
hsa_circ_	0.594902	0.019829
hsa_circ_	-0.43542	0.019833
hsa_circ_	0.710043	0.019836
hsa_circ_	0.486427	0.019837
hsa_circ_	-0.78351	0.019839
hsa_circ_	0.632946	0.019839
hsa_circ_	-0.39891	0.019844
hsa_circ_	-0.69875	0.019848
hsa_circ_	-1.18436	0.019858
hsa_circ_	0.384537	0.01986
hsa_circ_	-0.66337	0.019861
hsa_circ_	-0.62022	0.019888
hsa_circ_	0.647392	0.019896
hsa_circ_	0.790901	0.019906
hsa_circ_	-0.80473	0.019906
hsa_circ_	0.432014	0.019907
hsa_circ_	-0.94814	0.019919
hsa_circ_	0.701524	0.01992
hsa_circ_	-0.47907	0.01992
hsa_circ_	-1.1373	0.019922
hsa_circ_	0.679982	0.019923
hsa_circ_	0.619901	0.019934
hsa_circ_	-1.07848	0.019934
hsa_circ_	0.588067	0.019937
hsa_circ_	0.545596	0.019938
hsa_circ_	1.005174	0.019944

hsa_circ_	0.759772	0.019959
hsa_circ_	-0.46994	0.019968
hsa_circ_	-0.54679	0.019969
hsa_circ_	-0.41521	0.01997
hsa_circ_	0.435449	0.019979
hsa_circ_	-0.64597	0.019983
hsa_circ_	-1.28268	0.019989
hsa_circ_	0.596763	0.019996
hsa_circ_	-0.38648	0.019998
hsa_circ_	-0.79516	0.020005
hsa_circ_	-1.64643	0.020007
hsa_circ_	-1.41589	0.02001
hsa_circ_	0.509561	0.020012
hsa_circ_	-0.56479	0.020012
hsa_circ_	1.623288	0.020015
hsa_circ_	-0.61561	0.020019
hsa_circ_	-0.41646	0.020022
hsa_circ_	0.781983	0.020024
hsa_circ_	0.399568	0.020026
hsa_circ_	0.900806	0.020032
hsa_circ_	-1.08951	0.020035
hsa_circ_	-0.44464	0.020039
hsa_circ_	0.701357	0.020041
hsa_circ_	0.473154	0.020044
hsa_circ_	-0.76294	0.020045
hsa_circ_	1.203257	0.020047
hsa_circ_	-0.42387	0.020049
hsa_circ_	1.071329	0.020054
hsa_circ_	-0.75881	0.020055
hsa_circ_	-0.94568	0.020055
hsa_circ_	-0.88587	0.020058
hsa_circ_	1.47012	0.020063
hsa_circ_	0.51419	0.020082
hsa_circ_	-0.55975	0.020085
hsa_circ_	-0.7382	0.020091
hsa_circ_	-0.90217	0.020096
hsa_circ_	0.539574	0.020097
hsa_circ_	-0.58773	0.020099
hsa_circ_	-0.46157	0.020108
hsa_circ_	-0.73176	0.020109
hsa_circ_	0.920002	0.020109
hsa_circ_	-0.51488	0.020122
hsa_circ_	0.727083	0.020124
hsa_circ_	1.863971	0.020132
hsa_circ_	0.946568	0.020134
hsa_circ_	-0.46769	0.020141
hsa_circ_	-0.52242	0.020149
hsa_circ_	-0.49337	0.02015
hsa_circ_	-0.96492	0.020151
hsa_circ_	1.081609	0.020168
hsa_circ_	0.461152	0.020168
hsa_circ_	-0.70316	0.020169
hsa_circ_	-0.43089	0.020171
hsa_circ_	0.578171	0.020175

hsa_circ_	-0.42067	0.020181
hsa_circ_	0.713796	0.020181
hsa_circ_	0.373603	0.020183
hsa_circ_	-0.46736	0.020185
hsa_circ_	-0.57608	0.020185
hsa_circ_	0.845762	0.020187
hsa_circ_	-0.64411	0.02019
hsa_circ_	0.702612	0.020194
hsa_circ_	-0.5161	0.020195
hsa_circ_	0.986865	0.0202
hsa_circ_	-0.55298	0.020201
hsa_circ_	-0.47903	0.020202
hsa_circ_	0.511827	0.020204
hsa_circ_	0.69754	0.020205
hsa_circ_	0.545488	0.020206
hsa_circ_	1.288023	0.020212
hsa_circ_	-1.24454	0.020222
hsa_circ_	-0.49805	0.020225
hsa_circ_	0.624117	0.020226
hsa_circ_	-0.40972	0.020229
hsa_circ_	0.412873	0.020245
hsa_circ_	-1.15858	0.020252
hsa_circ_	-0.87427	0.020257
hsa_circ_	-0.76602	0.020264
hsa_circ_	0.390825	0.020264
hsa_circ_	-0.68673	0.020271
hsa_circ_	-0.66363	0.020274
hsa_circ_	0.622346	0.020275
hsa_circ_	0.937312	0.020279
hsa_circ_	1.131265	0.020286
hsa_circ_	0.558272	0.020287
hsa_circ_	-0.48504	0.020299
hsa_circ_	-0.49793	0.020302
hsa_circ_	-0.7635	0.020305
hsa_circ_	0.73008	0.020307
hsa_circ_	-0.48752	0.020318
hsa_circ_	0.705146	0.020323
hsa_circ_	-0.70716	0.020325
hsa_circ_	0.700773	0.02033
hsa_circ_	0.656501	0.020333
hsa_circ_	-0.6033	0.020336
hsa_circ_	0.394521	0.020336
hsa_circ_	0.51176	0.020343
hsa_circ_	0.563283	0.020344
hsa_circ_	-0.9349	0.020347
hsa_circ_	-0.91352	0.020352
hsa_circ_	-0.5105	0.020368
hsa_circ_	0.541988	0.020368
hsa_circ_	-0.39776	0.020369
hsa_circ_	-0.40707	0.020373
hsa_circ_	0.476412	0.020376
hsa_circ_	-0.46541	0.020379
hsa_circ_	0.517057	0.02038
hsa_circ_	-0.88045	0.02038

hsa_circ_	-0.51667	0.020384
hsa_circ_	0.511269	0.020386
hsa_circ_	1.516212	0.020392
hsa_circ_	-0.46928	0.020395
hsa_circ_	-0.48291	0.020407
hsa_circ_	-0.36748	0.020412
hsa_circ_	0.40237	0.020425
hsa_circ_	-0.52274	0.020428
hsa_circ_	-0.73982	0.020434
hsa_circ_	-0.71918	0.020434
hsa_circ_	-0.43876	0.020435
hsa_circ_	1.191185	0.020438
hsa_circ_	0.384315	0.020442
hsa_circ_	-1.64588	0.020449
hsa_circ_	-0.80997	0.020467
hsa_circ_	-0.38721	0.020467
hsa_circ_	-0.51279	0.020468
hsa_circ_	-0.39202	0.020476
hsa_circ_	0.386876	0.020483
hsa_circ_	-0.80539	0.020484
hsa_circ_	-0.58568	0.020491
hsa_circ_	-0.43388	0.020496
hsa_circ_	0.495069	0.020496
hsa_circ_	-0.60189	0.0205
hsa_circ_	-1.21571	0.0205
hsa_circ_	0.723967	0.020502
hsa_circ_	-0.49718	0.020503
hsa_circ_	0.4415	0.020511
hsa_circ_	0.769886	0.020518
hsa_circ_	0.549461	0.020521
hsa_circ_	-0.50268	0.020521
hsa_circ_	0.409422	0.020534
hsa_circ_	-0.9661	0.020536
hsa_circ_	-0.87253	0.020548
hsa_circ_	-0.56241	0.020549
hsa_circ_	0.464843	0.020549
hsa_circ_	-0.62467	0.020555
hsa_circ_	0.53531	0.020555
hsa_circ_	0.505664	0.020557
hsa_circ_	-0.81864	0.020572
hsa_circ_	0.415073	0.020574
hsa_circ_	-0.40767	0.020578
hsa_circ_	0.874528	0.020579
hsa_circ_	-0.99997	0.020581
hsa_circ_	-0.79604	0.020589
hsa_circ_	-1.05236	0.020591
hsa_circ_	-0.69472	0.020593
hsa_circ_	-0.52795	0.020596
hsa_circ_	0.574763	0.020601
hsa_circ_	-0.43203	0.020627
hsa_circ_	-0.64341	0.020629
hsa_circ_	-0.69293	0.020632
hsa_circ_	-0.43033	0.020632
hsa_circ_	0.486619	0.020634

hsa_circ_	0.426207	0.020638
hsa_circ_	0.854584	0.020639
hsa_circ_	-0.64111	0.020641
hsa_circ_	0.825906	0.020649
hsa_circ_	0.432972	0.020656
hsa_circ_	-0.38058	0.020658
hsa_circ_	-0.40414	0.020695
hsa_circ_	0.863083	0.0207
hsa_circ_	-0.62401	0.020704
hsa_circ_	0.380476	0.020708
hsa_circ_	0.943712	0.020715
hsa_circ_	-0.81171	0.020719
hsa_circ_	0.828838	0.020727
hsa_circ_	0.767896	0.020733
hsa_circ_	-0.45404	0.020734
hsa_circ_	-0.53191	0.020744
hsa_circ_	-0.6431	0.020757
hsa_circ_	-2.36555	0.020757
hsa_circ_	1.524691	0.02076
hsa_circ_	0.500886	0.020766
hsa_circ_	0.830755	0.020769
hsa_circ_	-0.47391	0.020785
hsa_circ_	-0.56066	0.020795
hsa_circ_	0.493456	0.020797
hsa_circ_	0.511565	0.020797
hsa_circ_	-0.99013	0.020804
hsa_circ_	0.418158	0.020809
hsa_circ_	0.555017	0.020811
hsa_circ_	0.494702	0.020815
hsa_circ_	0.711776	0.020816
hsa_circ_	0.509538	0.020821
hsa_circ_	0.694839	0.020834
hsa_circ_	-0.43892	0.020836
hsa_circ_	-0.51518	0.020837
hsa_circ_	0.519959	0.020837
hsa_circ_	0.427645	0.020839
hsa_circ_	-0.56426	0.020845
hsa_circ_	-0.51302	0.020845
hsa_circ_	1.032714	0.020847
hsa_circ_	-0.64566	0.020851
hsa_circ_	1.219179	0.02086
hsa_circ_	-0.47178	0.020863
hsa_circ_	0.80511	0.020863
hsa_circ_	0.945687	0.020873
hsa_circ_	1.030023	0.020877
hsa_circ_	-0.50297	0.020878
hsa_circ_	0.430371	0.020881
hsa_circ_	0.824423	0.020882
hsa_circ_	0.719825	0.020884
hsa_circ_	-0.65135	0.020888
hsa_circ_	0.802276	0.020892
hsa_circ_	0.563331	0.020894
hsa_circ_	-1.24963	0.020896
hsa_circ_	-0.64733	0.020897

hsa_circ_	-1.23902	0.020901
hsa_circ_	0.609243	0.020904
hsa_circ_	0.875149	0.02091
hsa_circ_	-0.50834	0.020913
hsa_circ_	0.417717	0.020917
hsa_circ_	0.564293	0.020917
hsa_circ_	-0.55731	0.020919
hsa_circ_	-1.51213	0.020922
hsa_circ_	0.649886	0.020927
hsa_circ_	0.785736	0.020937
hsa_circ_	-0.76477	0.02094
hsa_circ_	0.87387	0.020942
hsa_circ_	0.649555	0.020943
hsa_circ_	0.890365	0.020953
hsa_circ_	0.489188	0.020955
hsa_circ_	0.373601	0.02096
hsa_circ_	-1.17821	0.020965
hsa_circ_	1.257428	0.020982
hsa_circ_	-0.91562	0.020986
hsa_circ_	0.821302	0.02099
hsa_circ_	0.596015	0.020993
hsa_circ_	0.557763	0.020996
hsa_circ_	-1.30334	0.020996
hsa_circ_	-0.44146	0.020998
hsa_circ_	0.966316	0.021002
hsa_circ_	1.121632	0.021005
hsa_circ_	0.973729	0.021005
hsa_circ_	0.687878	0.021016
hsa_circ_	0.636702	0.021024
hsa_circ_	-0.85816	0.021032
hsa_circ_	0.493772	0.021038
hsa_circ_	0.969005	0.021043
hsa_circ_	-0.6212	0.021062
hsa_circ_	-0.37994	0.021066
hsa_circ_	-1.22648	0.02107
hsa_circ_	-0.40522	0.021073
hsa_circ_	-1.39122	0.02108
hsa_circ_	-0.8361	0.021089
hsa_circ_	-0.86448	0.021097
hsa_circ_	-0.72064	0.0211
hsa_circ_	-1.26113	0.021104
hsa_circ_	0.798165	0.021105
hsa_circ_	-0.56349	0.021107
hsa_circ_	-0.3677	0.021111
hsa_circ_	0.491634	0.021112
hsa_circ_	0.593058	0.021115
hsa_circ_	0.523534	0.021116
hsa_circ_	-0.69885	0.021134
hsa_circ_	0.526045	0.021135
hsa_circ_	0.482907	0.021144
hsa_circ_	-0.48289	0.021144
hsa_circ_	-0.75593	0.021151
hsa_circ_	0.446695	0.021151
hsa_circ_	0.702249	0.021158

hsa_circ_	0.935855	0.021165
hsa_circ_	0.557043	0.021173
hsa_circ_	0.469157	0.02118
hsa_circ_	0.429558	0.021186
hsa_circ_	0.485351	0.021199
hsa_circ_	0.551163	0.021206
hsa_circ_	1.404809	0.021239
hsa_circ_	0.420144	0.021242
hsa_circ_	-0.423	0.021243
hsa_circ_	0.67775	0.021257
hsa_circ_	0.636783	0.021258
hsa_circ_	0.408697	0.021258
hsa_circ_	-0.71923	0.021263
hsa_circ_	1.047335	0.02127
hsa_circ_	0.659888	0.021271
hsa_circ_	0.563296	0.021274
hsa_circ_	0.574349	0.021276
hsa_circ_	1.265717	0.021282
hsa_circ_	-0.49172	0.021285
hsa_circ_	0.435435	0.021291
hsa_circ_	0.526188	0.021296
hsa_circ_	0.519135	0.021301
hsa_circ_	0.72287	0.021307
hsa_circ_	0.530581	0.021314
hsa_circ_	-0.87179	0.021319
hsa_circ_	-0.55577	0.02132
hsa_circ_	0.545999	0.021324
hsa_circ_	0.749286	0.021325
hsa_circ_	-0.82146	0.021326
hsa_circ_	-0.3839	0.021327
hsa_circ_	-0.39117	0.021328
hsa_circ_	1.77237	0.02133
hsa_circ_	-0.49522	0.021331
hsa_circ_	0.861223	0.021335
hsa_circ_	-0.40253	0.021347
hsa_circ_	0.825717	0.021351
hsa_circ_	-0.53922	0.021358
hsa_circ_	-0.6256	0.021363
hsa_circ_	0.567889	0.021365
hsa_circ_	1.285302	0.021377
hsa_circ_	-0.63664	0.021381
hsa_circ_	-0.40771	0.021387
hsa_circ_	0.645212	0.021393
hsa_circ_	0.535095	0.0214
hsa_circ_	-0.42491	0.0214
hsa_circ_	-0.64017	0.021402
hsa_circ_	0.376174	0.021405
hsa_circ_	0.968519	0.021434
hsa_circ_	-0.49078	0.021438
hsa_circ_	-0.75308	0.02144
hsa_circ_	-0.66537	0.02144
hsa_circ_	0.620864	0.021455
hsa_circ_	0.45581	0.021461
hsa_circ_	0.458079	0.021467

hsa_circ_	0.626535	0.021471
hsa_circ_	-0.59727	0.021472
hsa_circ_	0.429625	0.021476
hsa_circ_	0.542374	0.021476
hsa_circ_	0.720371	0.021485
hsa_circ_	-0.73365	0.021488
hsa_circ_	0.417506	0.021496
hsa_circ_	0.575297	0.021497
hsa_circ_	0.585841	0.021498
hsa_circ_	-0.67896	0.021498
hsa_circ_	0.448821	0.021503
hsa_circ_	1.413562	0.021506
hsa_circ_	0.73588	0.021507
hsa_circ_	-0.53472	0.02151
hsa_circ_	0.511648	0.021512
hsa_circ_	0.415922	0.021515
hsa_circ_	-0.47256	0.02152
hsa_circ_	0.515206	0.021531
hsa_circ_	-1.55909	0.021533
hsa_circ_	-0.81198	0.021535
hsa_circ_	-0.69834	0.021545
hsa_circ_	0.820604	0.021555
hsa_circ_	1.151779	0.021558
hsa_circ_	-0.62732	0.02156
hsa_circ_	1.67983	0.021566
hsa_circ_	0.625708	0.021569
hsa_circ_	-0.65739	0.021569
hsa_circ_	0.474367	0.021576
hsa_circ_	-0.47323	0.021578
hsa_circ_	0.521669	0.021587
hsa_circ_	-1.95984	0.02159
hsa_circ_	-0.65652	0.021592
hsa_circ_	-0.45967	0.021597
hsa_circ_	-0.51957	0.0216
hsa_circ_	1.163557	0.021601
hsa_circ_	1.720716	0.021607
hsa_circ_	0.428793	0.021609
hsa_circ_	-0.5938	0.02161
hsa_circ_	-0.71644	0.021615
hsa_circ_	0.571686	0.021618
hsa_circ_	0.696291	0.021619
hsa_circ_	-0.36148	0.021619
hsa_circ_	0.62295	0.021621
hsa_circ_	-0.59779	0.021627
hsa_circ_	0.93556	0.021629
hsa_circ_	0.519802	0.021637
hsa_circ_	0.480006	0.021642
hsa_circ_	-0.85971	0.021643
hsa_circ_	-0.39981	0.021656
hsa_circ_	0.430548	0.021657
hsa_circ_	-0.81581	0.02166
hsa_circ_	1.202911	0.021663
hsa_circ_	-1.04297	0.021664
hsa_circ_	-0.44896	0.021671

hsa_circ_	-0.46403	0.021675
hsa_circ_	-0.42387	0.021676
hsa_circ_	0.425703	0.021685
hsa_circ_	0.737288	0.021688
hsa_circ_	0.905099	0.02169
hsa_circ_	-0.69516	0.02169
hsa_circ_	0.679037	0.021699
hsa_circ_	0.471176	0.021703
hsa_circ_	0.768891	0.021703
hsa_circ_	-0.37717	0.021705
hsa_circ_	-0.51283	0.021707
hsa_circ_	0.730223	0.02171
hsa_circ_	-1.48076	0.021715
hsa_circ_	1.112892	0.021719
hsa_circ_	-0.41017	0.021721
hsa_circ_	0.460972	0.021727
hsa_circ_	1.93283	0.02173
hsa_circ_	0.884829	0.021734
hsa_circ_	-0.46241	0.021737
hsa_circ_	0.403695	0.02174
hsa_circ_	-0.47388	0.021747
hsa_circ_	0.593972	0.021749
hsa_circ_	0.44507	0.021751
hsa_circ_	-0.38225	0.021753
hsa_circ_	-0.46314	0.021757
hsa_circ_	-0.84558	0.021763
hsa_circ_	0.434389	0.021773
hsa_circ_	-0.66747	0.021774
hsa_circ_	0.538649	0.021778
hsa_circ_	-0.59944	0.021784
hsa_circ_	0.50957	0.021792
hsa_circ_	-0.41554	0.021797
hsa_circ_	-0.76955	0.0218
hsa_circ_	1.230304	0.021809
hsa_circ_	0.520871	0.021821
hsa_circ_	-0.42622	0.021825
hsa_circ_	-0.85659	0.021829
hsa_circ_	-0.57228	0.021836
hsa_circ_	0.928955	0.021839
hsa_circ_	0.552085	0.021839
hsa_circ_	-0.71588	0.021855
hsa_circ_	0.490768	0.021855
hsa_circ_	-0.50391	0.021857
hsa_circ_	0.844167	0.021861
hsa_circ_	0.607214	0.021863
hsa_circ_	-0.53338	0.021868
hsa_circ_	-0.5327	0.021874
hsa_circ_	-0.47364	0.021877
hsa_circ_	-0.52351	0.021884
hsa_circ_	0.398261	0.021885
hsa_circ_	0.800829	0.021889
hsa_circ_	0.613012	0.021889
hsa_circ_	0.63907	0.021901
hsa_circ_	-0.80034	0.021922

hsa_circ_	-0.53482	0.021924
hsa_circ_	-0.59584	0.021925
hsa_circ_	-0.39524	0.02193
hsa_circ_	-0.83167	0.021939
hsa_circ_	-0.64903	0.021959
hsa_circ_	-0.55483	0.02196
hsa_circ_	0.480556	0.021962
hsa_circ_	-1.17216	0.021962
hsa_circ_	-0.53338	0.021967
hsa_circ_	0.749526	0.02197
hsa_circ_	-0.41949	0.021976
hsa_circ_	-0.48331	0.021978
hsa_circ_	-0.44478	0.021983
hsa_circ_	-0.39047	0.021988
hsa_circ_	0.758086	0.021999
hsa_circ_	-0.43974	0.022
hsa_circ_	-0.50067	0.022
hsa_circ_	-0.66602	0.022001
hsa_circ_	0.407304	0.022002
hsa_circ_	-0.44892	0.022005
hsa_circ_	0.649137	0.022017
hsa_circ_	0.937988	0.022024
hsa_circ_	1.04016	0.022038
hsa_circ_	-1.25545	0.022039
hsa_circ_	0.458532	0.022048
hsa_circ_	-0.52949	0.022049
hsa_circ_	1.804669	0.022054
hsa_circ_	-1.44637	0.022067
hsa_circ_	-0.5062	0.022069
hsa_circ_	0.921585	0.022076
hsa_circ_	-0.59369	0.022079
hsa_circ_	-0.62829	0.02208
hsa_circ_	-0.92087	0.022083
hsa_circ_	0.793434	0.022087
hsa_circ_	1.542157	0.022088
hsa_circ_	0.606311	0.022088
hsa_circ_	0.633418	0.02209
hsa_circ_	-0.93112	0.022106
hsa_circ_	-0.62468	0.022124
hsa_circ_	0.738713	0.022126
hsa_circ_	-0.8469	0.022151
hsa_circ_	-0.91443	0.022151
hsa_circ_	-0.85038	0.022158
hsa_circ_	0.593217	0.022164
hsa_circ_	0.495889	0.022175
hsa_circ_	0.394773	0.022177
hsa_circ_	0.499677	0.022178
hsa_circ_	-1.19155	0.022197
hsa_circ_	-0.81106	0.022207
hsa_circ_	0.495445	0.022209
hsa_circ_	0.379702	0.022212
hsa_circ_	0.594437	0.022217
hsa_circ_	0.441791	0.022222
hsa_circ_	1.791303	0.022226

hsa_circ_	0.587613	0.022233
hsa_circ_	1.134971	0.022233
hsa_circ_	1.089397	0.022247
hsa_circ_	0.516258	0.022249
hsa_circ_	2.050507	0.022256
hsa_circ_	-0.74189	0.022263
hsa_circ_	0.67891	0.022263
hsa_circ_	0.448992	0.022273
hsa_circ_	-0.54618	0.022274
hsa_circ_	0.871971	0.022279
hsa_circ_	1.031255	0.022281
hsa_circ_	-0.44802	0.022298
hsa_circ_	0.5527	0.022304
hsa_circ_	1.239962	0.022305
hsa_circ_	0.603256	0.022305
hsa_circ_	-0.48418	0.022308
hsa_circ_	0.511351	0.022311
hsa_circ_	2.444148	0.022321
hsa_circ_	-0.41545	0.022331
hsa_circ_	-0.73873	0.022333
hsa_circ_	0.488593	0.022336
hsa_circ_	-0.52896	0.022343
hsa_circ_	-0.86529	0.022346
hsa_circ_	-0.45262	0.022346
hsa_circ_	1.035996	0.02235
hsa_circ_	-0.48883	0.022351
hsa_circ_	0.599785	0.022358
hsa_circ_	0.984329	0.022359
hsa_circ_	0.422198	0.022363
hsa_circ_	0.48026	0.022368
hsa_circ_	0.396183	0.02237
hsa_circ_	-0.8245	0.022373
hsa_circ_	-0.7755	0.022375
hsa_circ_	-0.41967	0.022383
hsa_circ_	0.432783	0.022386
hsa_circ_	0.76207	0.022389
hsa_circ_	-0.4536	0.022389
hsa_circ_	-0.54343	0.022395
hsa_circ_	-0.37266	0.022399
hsa_circ_	0.707063	0.022407
hsa_circ_	0.559969	0.022423
hsa_circ_	0.56682	0.022435
hsa_circ_	-0.61399	0.022447
hsa_circ_	-0.70071	0.022453
hsa_circ_	-0.51348	0.02246
hsa_circ_	-0.47553	0.022471
hsa_circ_	-0.65848	0.022473
hsa_circ_	-0.50409	0.022476
hsa_circ_	1.291273	0.022488
hsa_circ_	-0.4053	0.022488
hsa_circ_	-0.83102	0.02249
hsa_circ_	-0.83665	0.022495
hsa_circ_	0.417765	0.022495
hsa_circ_	0.520185	0.022504

hsa_circ_	-0.627	0.022507
hsa_circ_	-0.48441	0.022519
hsa_circ_	-0.70827	0.022529
hsa_circ_	0.453146	0.022537
hsa_circ_	-0.88754	0.022537
hsa_circ_	-0.80095	0.022538
hsa_circ_	1.060412	0.022539
hsa_circ_	0.579528	0.022541
hsa_circ_	1.171663	0.022546
hsa_circ_	0.960373	0.022549
hsa_circ_	0.477216	0.02255
hsa_circ_	-0.46294	0.022557
hsa_circ_	0.512441	0.022559
hsa_circ_	0.962148	0.022559
hsa_circ_	-0.52619	0.022559
hsa_circ_	0.894015	0.022567
hsa_circ_	0.503748	0.022567
hsa_circ_	-0.49866	0.022578
hsa_circ_	0.50448	0.02258
hsa_circ_	0.439954	0.022581
hsa_circ_	-0.56124	0.022584
hsa_circ_	0.753991	0.022584
hsa_circ_	-0.73802	0.022584
hsa_circ_	-0.55907	0.022586
hsa_circ_	-1.32809	0.02259
hsa_circ_	-0.5171	0.022606
hsa_circ_	0.597299	0.022621
hsa_circ_	-0.51255	0.022625
hsa_circ_	-0.53994	0.022629
hsa_circ_	1.47624	0.022631
hsa_circ_	-0.44189	0.022631
hsa_circ_	-0.44983	0.022632
hsa_circ_	0.953343	0.022633
hsa_circ_	-0.38102	0.022633
hsa_circ_	-0.69065	0.022642
hsa_circ_	0.645542	0.022644
hsa_circ_	0.706202	0.022646
hsa_circ_	-0.77868	0.022648
hsa_circ_	-0.67612	0.022649
hsa_circ_	0.580746	0.022653
hsa_circ_	-0.56618	0.022658
hsa_circ_	1.667605	0.022661
hsa_circ_	0.769081	0.022661
hsa_circ_	0.595729	0.022669
hsa_circ_	-0.42799	0.02267
hsa_circ_	-0.69282	0.022673
hsa_circ_	-0.5505	0.022682
hsa_circ_	1.724408	0.022684
hsa_circ_	-0.50678	0.022687
hsa_circ_	-0.72363	0.022693
hsa_circ_	-1.23474	0.022696
hsa_circ_	-0.43366	0.022703
hsa_circ_	1.654689	0.022707
hsa_circ_	-0.46374	0.022712

hsa_circ_	-0.48653	0.022714
hsa_circ_	0.537551	0.022719
hsa_circ_	-0.63316	0.022728
hsa_circ_	-0.78328	0.022732
hsa_circ_	-0.835	0.022737
hsa_circ_	1.210156	0.022744
hsa_circ_	-1.21517	0.022745
hsa_circ_	0.653113	0.022762
hsa_circ_	1.106999	0.022766
hsa_circ_	0.50933	0.022766
hsa_circ_	1.359052	0.022774
hsa_circ_	-0.40904	0.022774
hsa_circ_	0.687241	0.022777
hsa_circ_	0.777276	0.022777
hsa_circ_	-0.44369	0.022777
hsa_circ_	0.434687	0.022779
hsa_circ_	-0.4145	0.022779
hsa_circ_	0.465546	0.022786
hsa_circ_	0.627595	0.022791
hsa_circ_	0.796546	0.022794
hsa_circ_	0.497139	0.022797
hsa_circ_	-0.39068	0.022806
hsa_circ_	0.661668	0.022811
hsa_circ_	0.420771	0.022815
hsa_circ_	-0.43263	0.02282
hsa_circ_	-0.78861	0.02282
hsa_circ_	-0.74335	0.022823
hsa_circ_	0.535028	0.022823
hsa_circ_	0.790197	0.022823
hsa_circ_	0.711189	0.022825
hsa_circ_	-0.55919	0.02283
hsa_circ_	0.450953	0.022846
hsa_circ_	-0.94731	0.022849
hsa_circ_	-0.56975	0.02285
hsa_circ_	0.50904	0.022856
hsa_circ_	2.455193	0.022856
hsa_circ_	0.39599	0.022903
hsa_circ_	0.702555	0.022908
hsa_circ_	-0.78485	0.022919
hsa_circ_	0.596369	0.02292
hsa_circ_	0.704275	0.022923
hsa_circ_	1.271183	0.022923
hsa_circ_	0.593722	0.022938
hsa_circ_	-0.43419	0.022938
hsa_circ_	0.810463	0.022946
hsa_circ_	0.906656	0.022951
hsa_circ_	-0.44697	0.022952
hsa_circ_	0.729981	0.022955
hsa_circ_	-0.52278	0.022957
hsa_circ_	-0.58084	0.02296
hsa_circ_	-0.61653	0.022961
hsa_circ_	-0.67973	0.022961
hsa_circ_	-1.18685	0.022963
hsa_circ_	0.534858	0.022972

hsa_circ_	-0.75284	0.022974
hsa_circ_	-0.39024	0.022974
hsa_circ_	-0.99291	0.022977
hsa_circ_	0.495048	0.022977
hsa_circ_	-1.108	0.022977
hsa_circ_	-0.39326	0.022978
hsa_circ_	-0.72982	0.022984
hsa_circ_	1.131023	0.022989
hsa_circ_	-0.61509	0.022999
hsa_circ_	1.596988	0.023002
hsa_circ_	-0.82991	0.023006
hsa_circ_	0.365804	0.023011
hsa_circ_	-0.95416	0.023012
hsa_circ_	0.541283	0.023013
hsa_circ_	0.717595	0.023025
hsa_circ_	-0.37974	0.023026
hsa_circ_	0.398824	0.02303
hsa_circ_	-0.8099	0.023033
hsa_circ_	-1.28193	0.023048
hsa_circ_	0.406065	0.02305
hsa_circ_	-0.54747	0.023055
hsa_circ_	-0.79445	0.023057
hsa_circ_	-0.49221	0.023058
hsa_circ_	0.440284	0.023059
hsa_circ_	-0.72262	0.023065
hsa_circ_	-0.45655	0.023069
hsa_circ_	0.391297	0.02307
hsa_circ_	-0.56059	0.023072
hsa_circ_	0.591608	0.023075
hsa_circ_	1.277872	0.023075
hsa_circ_	1.540769	0.023076
hsa_circ_	-0.44776	0.023076
hsa_circ_	-0.85662	0.023077
hsa_circ_	-0.49519	0.023078
hsa_circ_	0.451934	0.023091
hsa_circ_	1.423234	0.023095
hsa_circ_	-0.37008	0.023099
hsa_circ_	0.591391	0.023103
hsa_circ_	-0.68921	0.023118
hsa_circ_	0.645217	0.023122
hsa_circ_	0.607908	0.023124
hsa_circ_	-1.20005	0.023126
hsa_circ_	1.310198	0.023127
hsa_circ_	-0.45386	0.023128
hsa_circ_	0.484357	0.02313
hsa_circ_	0.711148	0.023149
hsa_circ_	-0.44419	0.023173
hsa_circ_	-0.62591	0.023174
hsa_circ_	0.475035	0.023184
hsa_circ_	-0.43686	0.023186
hsa_circ_	-0.39421	0.023202
hsa_circ_	0.780545	0.023207
hsa_circ_	0.833223	0.02321
hsa_circ_	-1.16726	0.023227

hsa_circ_	0.631384	0.023232
hsa_circ_	0.464942	0.023234
hsa_circ_	0.589108	0.023239
hsa_circ_	0.474915	0.023243
hsa_circ_	0.40177	0.023255
hsa_circ_	-0.52911	0.023271
hsa_circ_	-0.73387	0.023284
hsa_circ_	0.5637	0.023285
hsa_circ_	0.54369	0.023288
hsa_circ_	-0.48135	0.023298
hsa_circ_	0.563478	0.023308
hsa_circ_	-0.44885	0.023315
hsa_circ_	0.855848	0.023317
hsa_circ_	0.680448	0.02332
hsa_circ_	-0.56864	0.023327
hsa_circ_	0.428121	0.023328
hsa_circ_	1.021626	0.023346
hsa_circ_	0.74796	0.023349
hsa_circ_	-0.76325	0.023354
hsa_circ_	0.552689	0.023366
hsa_circ_	-0.82021	0.023369
hsa_circ_	0.600757	0.023374
hsa_circ_	0.707915	0.023374
hsa_circ_	-1.53072	0.023376
hsa_circ_	-0.64686	0.023399
hsa_circ_	0.483337	0.023401
hsa_circ_	0.424883	0.023407
hsa_circ_	-0.73455	0.023408
hsa_circ_	-0.39109	0.02341
hsa_circ_	-0.6705	0.023415
hsa_circ_	-0.39034	0.023416
hsa_circ_	0.795899	0.023421
hsa_circ_	0.500484	0.023421
hsa_circ_	-0.47873	0.023431
hsa_circ_	0.723699	0.023442
hsa_circ_	-0.77656	0.023444
hsa_circ_	-0.37385	0.023453
hsa_circ_	-0.52908	0.023453
hsa_circ_	0.415511	0.023454
hsa_circ_	-0.39465	0.023455
hsa_circ_	-0.63451	0.02346
hsa_circ_	-1.48231	0.02346
hsa_circ_	-0.54696	0.023462
hsa_circ_	0.565586	0.023472
hsa_circ_	1.298013	0.023473
hsa_circ_	1.64356	0.023482
hsa_circ_	0.634234	0.023484
hsa_circ_	-0.93278	0.023484
hsa_circ_	0.831353	0.023486
hsa_circ_	-0.71621	0.023487
hsa_circ_	-0.409	0.023487
hsa_circ_	0.496407	0.023506
hsa_circ_	1.627108	0.023509
hsa_circ_	0.481294	0.023509

hsa_circ_	-0.48578	0.023516
hsa_circ_	-0.54839	0.023521
hsa_circ_	-1.22732	0.023527
hsa_circ_	0.96327	0.023528
hsa_circ_	0.471468	0.02353
hsa_circ_	0.537973	0.023533
hsa_circ_	-0.90252	0.023537
hsa_circ_	0.647592	0.023543
hsa_circ_	-0.62706	0.023552
hsa_circ_	-0.53057	0.023553
hsa_circ_	0.52405	0.023558
hsa_circ_	0.453464	0.023562
hsa_circ_	0.548411	0.023563
hsa_circ_	0.856384	0.023566
hsa_circ_	-0.78476	0.023569
hsa_circ_	-0.40053	0.02357
hsa_circ_	0.505946	0.023581
hsa_circ_	-1.01515	0.023593
hsa_circ_	0.668839	0.023593
hsa_circ_	0.702618	0.023594
hsa_circ_	1.040188	0.023595
hsa_circ_	0.563461	0.023595
hsa_circ_	0.648795	0.023605
hsa_circ_	0.81001	0.023605
hsa_circ_	0.958488	0.023609
hsa_circ_	-0.70094	0.023609
hsa_circ_	-0.62267	0.023624
hsa_circ_	0.51913	0.023635
hsa_circ_	0.448409	0.023638
hsa_circ_	-0.48708	0.02364
hsa_circ_	0.493274	0.023645
hsa_circ_	-1.19065	0.023667
hsa_circ_	0.558392	0.023671
hsa_circ_	-0.82828	0.023672
hsa_circ_	0.44161	0.023679
hsa_circ_	-0.42506	0.023679
hsa_circ_	1.026868	0.023685
hsa_circ_	-0.57375	0.023695
hsa_circ_	-0.45929	0.023706
hsa_circ_	-0.75321	0.023706
hsa_circ_	0.431971	0.023708
hsa_circ_	1.156984	0.023709
hsa_circ_	0.714762	0.02371
hsa_circ_	1.265564	0.023715
hsa_circ_	0.809318	0.023715
hsa_circ_	0.726112	0.023721
hsa_circ_	0.622698	0.02373
hsa_circ_	0.432929	0.023734
hsa_circ_	0.528191	0.023735
hsa_circ_	-0.51341	0.02374
hsa_circ_	0.576316	0.02375
hsa_circ_	0.391438	0.023751
hsa_circ_	0.500481	0.023754
hsa_circ_	-0.78417	0.023755

hsa_circ_	1.421605	0.023759
hsa_circ_	0.895268	0.023761
hsa_circ_	0.563402	0.023766
hsa_circ_	1.040269	0.023769
hsa_circ_	-1.0546	0.023771
hsa_circ_	0.68948	0.023774
hsa_circ_	-0.93331	0.023784
hsa_circ_	0.552528	0.023792
hsa_circ_	-0.8225	0.023807
hsa_circ_	0.608522	0.023813
hsa_circ_	0.52187	0.02382
hsa_circ_	1.423355	0.023825
hsa_circ_	-1.05185	0.023832
hsa_circ_	0.416769	0.023833
hsa_circ_	0.670621	0.023839
hsa_circ_	0.951273	0.023842
hsa_circ_	-0.74869	0.023846
hsa_circ_	-0.4562	0.023849
hsa_circ_	0.536605	0.023851
hsa_circ_	0.69553	0.023856
hsa_circ_	1.237767	0.023857
hsa_circ_	0.759857	0.023866
hsa_circ_	1.102231	0.023869
hsa_circ_	-1.01376	0.023872
hsa_circ_	-0.48926	0.023872
hsa_circ_	-0.4999	0.023879
hsa_circ_	-0.44871	0.023885
hsa_circ_	-0.47908	0.023892
hsa_circ_	-0.62235	0.023896
hsa_circ_	0.576465	0.023897
hsa_circ_	0.430811	0.02391
hsa_circ_	-0.82232	0.023916
hsa_circ_	-1.04727	0.023916
hsa_circ_	0.908494	0.023916
hsa_circ_	-0.39493	0.023923
hsa_circ_	-0.49949	0.023925
hsa_circ_	-0.51784	0.023928
hsa_circ_	0.650516	0.023929
hsa_circ_	0.424022	0.023931
hsa_circ_	0.506645	0.02395
hsa_circ_	0.680906	0.023959
hsa_circ_	0.90104	0.02397
hsa_circ_	0.49984	0.023974
hsa_circ_	-0.63433	0.023982
hsa_circ_	1.125636	0.023983
hsa_circ_	0.896008	0.023984
hsa_circ_	-0.4419	0.02399
hsa_circ_	-0.37808	0.023992
hsa_circ_	-0.4537	0.024007
hsa_circ_	0.535474	0.024008
hsa_circ_	-0.45342	0.02401
hsa_circ_	0.390171	0.024013
hsa_circ_	-0.35429	0.024018
hsa_circ_	-0.90211	0.024033

hsa_circ_	-0.92454	0.024034
hsa_circ_	0.953835	0.024035
hsa_circ_	-0.51907	0.024036
hsa_circ_	-0.51207	0.024037
hsa_circ_	-0.39055	0.024037
hsa_circ_	0.521145	0.024041
hsa_circ_	0.91136	0.024043
hsa_circ_	-0.68435	0.024045
hsa_circ_	-1.35246	0.024048
hsa_circ_	0.45447	0.024051
hsa_circ_	0.742223	0.024052
hsa_circ_	0.946462	0.024063
hsa_circ_	-0.82195	0.024063
hsa_circ_	0.394453	0.024064
hsa_circ_	0.632901	0.024065
hsa_circ_	-0.57607	0.024065
hsa_circ_	1.137878	0.024067
hsa_circ_	0.679511	0.024068
hsa_circ_	-0.4165	0.024083
hsa_circ_	0.595705	0.02409
hsa_circ_	0.496382	0.024097
hsa_circ_	-0.4417	0.024105
hsa_circ_	-0.35663	0.024116
hsa_circ_	-0.49594	0.024128
hsa_circ_	0.44314	0.02413
hsa_circ_	0.435319	0.024132
hsa_circ_	1.176807	0.024133
hsa_circ_	-0.68424	0.024137
hsa_circ_	-0.38956	0.024145
hsa_circ_	-1.46604	0.024151
hsa_circ_	0.551964	0.024161
hsa_circ_	0.548949	0.024163
hsa_circ_	1.054916	0.024164
hsa_circ_	1.025234	0.024165
hsa_circ_	0.451097	0.024172
hsa_circ_	-0.43734	0.024175
hsa_circ_	0.460759	0.024182
hsa_circ_	-0.7376	0.024184
hsa_circ_	-0.40661	0.024191
hsa_circ_	0.834034	0.024192
hsa_circ_	0.396564	0.024194
hsa_circ_	0.857773	0.024194
hsa_circ_	-0.40335	0.024199
hsa_circ_	0.437245	0.024201
hsa_circ_	0.820199	0.024201
hsa_circ_	-0.70954	0.024202
hsa_circ_	-0.54141	0.024203
hsa_circ_	0.557292	0.024203
hsa_circ_	0.536071	0.024208
hsa_circ_	-0.92892	0.024213
hsa_circ_	0.695096	0.024217
hsa_circ_	0.61437	0.024223
hsa_circ_	-0.39272	0.024227
hsa_circ_	-0.37986	0.024246

hsa_circ_	-0.60794	0.024248
hsa_circ_	1.02296	0.024251
hsa_circ_	1.212581	0.024262
hsa_circ_	-0.39151	0.024263
hsa_circ_	-0.80901	0.024264
hsa_circ_	1.259928	0.02427
hsa_circ_	0.64795	0.024275
hsa_circ_	0.548317	0.024275
hsa_circ_	-0.48088	0.024278
hsa_circ_	-0.84208	0.024279
hsa_circ_	1.017379	0.02428
hsa_circ_	0.647108	0.024285
hsa_circ_	-0.78415	0.02429
hsa_circ_	0.56988	0.024297
hsa_circ_	-0.59336	0.024298
hsa_circ_	0.406564	0.024299
hsa_circ_	-0.9662	0.024301
hsa_circ_	-0.4186	0.024303
hsa_circ_	0.662678	0.024309
hsa_circ_	0.434399	0.02431
hsa_circ_	-0.4715	0.024316
hsa_circ_	-0.60122	0.024317
hsa_circ_	-1.09332	0.02432
hsa_circ_	0.63223	0.024322
hsa_circ_	-0.81257	0.024325
hsa_circ_	0.53936	0.02433
hsa_circ_	0.575005	0.024334
hsa_circ_	1.12307	0.024336
hsa_circ_	0.390869	0.024336
hsa_circ_	0.420792	0.024339
hsa_circ_	-0.65839	0.024352
hsa_circ_	0.4147	0.024356
hsa_circ_	0.448843	0.024356
hsa_circ_	-0.44708	0.024359
hsa_circ_	-0.39702	0.024359
hsa_circ_	0.5533	0.02436
hsa_circ_	0.439276	0.02437
hsa_circ_	0.958784	0.024378
hsa_circ_	-0.38229	0.024383
hsa_circ_	-1.61398	0.024387
hsa_circ_	-0.83977	0.02439
hsa_circ_	-0.57088	0.024402
hsa_circ_	0.771135	0.024405
hsa_circ_	0.638393	0.02441
hsa_circ_	0.607064	0.024417
hsa_circ_	0.840915	0.024418
hsa_circ_	-0.42606	0.024425
hsa_circ_	0.532384	0.02443
hsa_circ_	0.804376	0.024432
hsa_circ_	0.723985	0.024438
hsa_circ_	-0.47735	0.024443
hsa_circ_	-0.84747	0.024447
hsa_circ_	-0.36424	0.024447
hsa_circ_	0.825699	0.024449

hsa_circ_	0.437294	0.024453
hsa_circ_	0.407798	0.024464
hsa_circ_	0.480319	0.024468
hsa_circ_	-1.27764	0.024476
hsa_circ_	0.529757	0.024477
hsa_circ_	0.87513	0.024485
hsa_circ_	0.55336	0.02449
hsa_circ_	-0.94649	0.024497
hsa_circ_	-0.77852	0.024502
hsa_circ_	0.505496	0.024507
hsa_circ_	0.5791	0.024507
hsa_circ_	0.461328	0.024511
hsa_circ_	-0.52976	0.024511
hsa_circ_	0.530531	0.024515
hsa_circ_	-0.74623	0.024519
hsa_circ_	-1.20165	0.024524
hsa_circ_	-0.61338	0.024535
hsa_circ_	0.561873	0.024537
hsa_circ_	-1.37491	0.024539
hsa_circ_	0.883756	0.024548
hsa_circ_	-0.65772	0.024549
hsa_circ_	-0.60984	0.024551
hsa_circ_	0.381703	0.024561
hsa_circ_	-1.28348	0.024565
hsa_circ_	2.762693	0.024565
hsa_circ_	1.007398	0.024567
hsa_circ_	0.974463	0.024578
hsa_circ_	0.463527	0.024581
hsa_circ_	-0.55526	0.024581
hsa_circ_	0.812056	0.024585
hsa_circ_	-0.89048	0.024587
hsa_circ_	-0.82068	0.02459
hsa_circ_	-0.38683	0.024591
hsa_circ_	1.189132	0.024593
hsa_circ_	0.787233	0.024599
hsa_circ_	-1.61616	0.024602
hsa_circ_	0.675755	0.024603
hsa_circ_	1.365796	0.024604
hsa_circ_	-0.49073	0.024614
hsa_circ_	-0.87939	0.024638
hsa_circ_	0.687784	0.024639
hsa_circ_	1.135335	0.024642
hsa_circ_	0.511367	0.024644
hsa_circ_	1.113317	0.024646
hsa_circ_	0.636057	0.024659
hsa_circ_	1.172536	0.024677
hsa_circ_	1.131952	0.024683
hsa_circ_	0.571362	0.024685
hsa_circ_	-0.86601	0.024694
hsa_circ_	0.636693	0.024699
hsa_circ_	-0.41044	0.024702
hsa_circ_	0.608504	0.024706
hsa_circ_	-0.69265	0.024707
hsa_circ_	0.379395	0.024716

hsa_circ_	-0.91143	0.024717
hsa_circ_	0.414094	0.024721
hsa_circ_	0.434209	0.024724
hsa_circ_	-0.91127	0.024733
hsa_circ_	0.789455	0.02474
hsa_circ_	-0.59769	0.024743
hsa_circ_	0.559299	0.024745
hsa_circ_	-0.68775	0.024776
hsa_circ_	0.509781	0.024779
hsa_circ_	0.547332	0.024781
hsa_circ_	0.63182	0.024791
hsa_circ_	-0.54442	0.024797
hsa_circ_	-0.6914	0.024801
hsa_circ_	-0.42308	0.024804
hsa_circ_	0.403691	0.024808
hsa_circ_	-0.58908	0.024811
hsa_circ_	0.395183	0.024813
hsa_circ_	-1.52157	0.024815
hsa_circ_	-0.96484	0.024818
hsa_circ_	0.741404	0.024828
hsa_circ_	-0.57483	0.024841
hsa_circ_	-1.18802	0.024841
hsa_circ_	-0.68486	0.02486
hsa_circ_	1.077819	0.024879
hsa_circ_	-0.75227	0.024887
hsa_circ_	0.523614	0.024889
hsa_circ_	-2.0485	0.024889
hsa_circ_	-0.4103	0.02489
hsa_circ_	0.546245	0.024891
hsa_circ_	0.504558	0.024891
hsa_circ_	0.715942	0.024918
hsa_circ_	-1.11904	0.024919
hsa_circ_	-0.41871	0.024924
hsa_circ_	0.420375	0.024924
hsa_circ_	-2.36897	0.024927
hsa_circ_	-0.4991	0.02493
hsa_circ_	0.888188	0.024931
hsa_circ_	-0.50802	0.024941
hsa_circ_	-0.55046	0.024942
hsa_circ_	0.460911	0.024947
hsa_circ_	-0.50779	0.024954
hsa_circ_	-0.69688	0.024958
hsa_circ_	1.012967	0.024958
hsa_circ_	0.745974	0.024963
hsa_circ_	-0.41059	0.024966
hsa_circ_	-0.71714	0.024967
hsa_circ_	-0.3876	0.024968
hsa_circ_	-0.53144	0.024971
hsa_circ_	-0.64111	0.024973
hsa_circ_	0.694945	0.024973
hsa_circ_	0.9395	0.024975
hsa_circ_	0.762379	0.024982
hsa_circ_	1.51854	0.024984
hsa_circ_	-0.40276	0.024987

hsa_circ_	0.423042	0.024992
hsa_circ_	0.596757	0.024993
hsa_circ_	0.454844	0.02501
hsa_circ_	0.645386	0.025014
hsa_circ_	0.802807	0.025015
hsa_circ_	1.556624	0.025028
hsa_circ_	0.506547	0.025029
hsa_circ_	0.888269	0.025031
hsa_circ_	-0.43393	0.025031
hsa_circ_	0.913946	0.025033
hsa_circ_	-0.56174	0.025042
hsa_circ_	0.487409	0.025064
hsa_circ_	-0.74784	0.025069
hsa_circ_	-0.60676	0.025081
hsa_circ_	0.808964	0.025082
hsa_circ_	-0.66757	0.025083
hsa_circ_	0.542752	0.025084
hsa_circ_	-0.58664	0.025092
hsa_circ_	0.661511	0.025107
hsa_circ_	0.7111	0.025109
hsa_circ_	0.497237	0.025112
hsa_circ_	-0.45601	0.025126
hsa_circ_	0.3884	0.025127
hsa_circ_	0.518166	0.02513
hsa_circ_	-0.50175	0.02513
hsa_circ_	-0.67715	0.025132
hsa_circ_	-0.54546	0.025133
hsa_circ_	0.753031	0.025135
hsa_circ_	-0.57211	0.025135
hsa_circ_	-1.22642	0.025151
hsa_circ_	-0.60457	0.025163
hsa_circ_	1.032959	0.025169
hsa_circ_	0.51468	0.025186
hsa_circ_	0.593652	0.025188
hsa_circ_	0.379694	0.025193
hsa_circ_	0.580476	0.025193
hsa_circ_	0.525263	0.025197
hsa_circ_	-1.33512	0.0252
hsa_circ_	-0.3732	0.025208
hsa_circ_	-0.50524	0.025208
hsa_circ_	0.384163	0.025222
hsa_circ_	0.695903	0.025223
hsa_circ_	-0.68427	0.025234
hsa_circ_	-0.65998	0.025238
hsa_circ_	-1.88342	0.02524
hsa_circ_	0.689559	0.02524
hsa_circ_	0.518381	0.025244
hsa_circ_	-0.97566	0.025246
hsa_circ_	-0.37741	0.025251
hsa_circ_	0.622899	0.025303
hsa_circ_	-1.18132	0.025309
hsa_circ_	0.47143	0.025312
hsa_circ_	1.64125	0.025319
hsa_circ_	-0.73528	0.025326

hsa_circ_	0.524842	0.025326
hsa_circ_	-0.43598	0.025327
hsa_circ_	-0.71305	0.025327
hsa_circ_	-1.23253	0.02533
hsa_circ_	0.887151	0.02533
hsa_circ_	-0.99761	0.025333
hsa_circ_	0.419045	0.025333
hsa_circ_	0.661044	0.025336
hsa_circ_	0.432713	0.025344
hsa_circ_	-0.80332	0.025351
hsa_circ_	-0.43705	0.025352
hsa_circ_	0.43766	0.025356
hsa_circ_	-0.84297	0.025358
hsa_circ_	-0.6969	0.025358
hsa_circ_	0.784594	0.025366
hsa_circ_	-0.43931	0.025369
hsa_circ_	0.450848	0.025382
hsa_circ_	-0.49423	0.025392
hsa_circ_	-0.62947	0.025398
hsa_circ_	0.435136	0.025403
hsa_circ_	-1.05226	0.025408
hsa_circ_	-1.59847	0.025409
hsa_circ_	0.65354	0.02541
hsa_circ_	-0.42092	0.025411
hsa_circ_	-0.4425	0.025413
hsa_circ_	0.405149	0.02542
hsa_circ_	-0.39574	0.025424
hsa_circ_	-0.43833	0.025427
hsa_circ_	-0.51736	0.025431
hsa_circ_	0.735342	0.025441
hsa_circ_	-0.37238	0.025441
hsa_circ_	-0.57863	0.025443
hsa_circ_	0.447155	0.025449
hsa_circ_	-0.72688	0.025459
hsa_circ_	0.714032	0.02546
hsa_circ_	0.506012	0.025464
hsa_circ_	-0.45026	0.025465
hsa_circ_	-0.40001	0.02547
hsa_circ_	0.39773	0.025473
hsa_circ_	-0.74951	0.025475
hsa_circ_	1.081066	0.025477
hsa_circ_	-1.53695	0.025482
hsa_circ_	0.381803	0.025486
hsa_circ_	0.988938	0.025489
hsa_circ_	1.453513	0.025492
hsa_circ_	0.541718	0.025492
hsa_circ_	0.710711	0.025506
hsa_circ_	-0.4441	0.025515
hsa_circ_	-0.52953	0.025525
hsa_circ_	-0.58863	0.02553
hsa_circ_	-1.2755	0.025541
hsa_circ_	-0.46818	0.025551
hsa_circ_	0.586464	0.025577
hsa_circ_	-0.45562	0.025584

hsa_circ_	-0.54537	0.025588
hsa_circ_	0.625633	0.025592
hsa_circ_	-0.52174	0.025607
hsa_circ_	-0.58578	0.025613
hsa_circ_	-0.46423	0.025616
hsa_circ_	0.829266	0.025619
hsa_circ_	0.514902	0.025622
hsa_circ_	0.537914	0.025622
hsa_circ_	0.43361	0.025623
hsa_circ_	0.734285	0.025624
hsa_circ_	0.875302	0.025624
hsa_circ_	-0.87798	0.025627
hsa_circ_	-0.62768	0.025634
hsa_circ_	0.666603	0.025634
hsa_circ_	0.477846	0.025638
hsa_circ_	0.842873	0.02564
hsa_circ_	0.605766	0.02564
hsa_circ_	-0.58775	0.025641
hsa_circ_	-0.53372	0.025655
hsa_circ_	-0.60752	0.025671
hsa_circ_	0.519684	0.025672
hsa_circ_	0.441932	0.025676
hsa_circ_	-0.46446	0.025682
hsa_circ_	-0.55573	0.025692
hsa_circ_	0.473983	0.025693
hsa_circ_	-0.3844	0.025695
hsa_circ_	0.619419	0.025711
hsa_circ_	1.040715	0.025731
hsa_circ_	0.537965	0.025732
hsa_circ_	0.793833	0.025741
hsa_circ_	-0.57094	0.025741
hsa_circ_	0.779615	0.025757
hsa_circ_	0.691542	0.025763
hsa_circ_	0.386887	0.025779
hsa_circ_	-0.3889	0.025781
hsa_circ_	-0.96174	0.025783
hsa_circ_	0.661815	0.025787
hsa_circ_	-0.5405	0.02579
hsa_circ_	0.398883	0.025791
hsa_circ_	-1.11498	0.025793
hsa_circ_	-0.74154	0.025802
hsa_circ_	0.548251	0.025803
hsa_circ_	0.536471	0.025812
hsa_circ_	0.815043	0.025819
hsa_circ_	0.862593	0.025826
hsa_circ_	0.710426	0.025834
hsa_circ_	-0.45403	0.025841
hsa_circ_	-0.53093	0.025845
hsa_circ_	-0.60275	0.025848
hsa_circ_	-1.0793	0.025849
hsa_circ_	0.519152	0.025853
hsa_circ_	0.908675	0.025864
hsa_circ_	-1.74088	0.025866
hsa_circ_	-0.6757	0.025868

hsa_circ_	-0.57216	0.025869
hsa_circ_	-0.70376	0.025872
hsa_circ_	0.621612	0.025873
hsa_circ_	-0.72035	0.025896
hsa_circ_	0.714314	0.025897
hsa_circ_	0.5412	0.025898
hsa_circ_	-0.35278	0.025904
hsa_circ_	1.020367	0.025904
hsa_circ_	-0.38489	0.025911
hsa_circ_	0.610067	0.025914
hsa_circ_	-0.5087	0.025914
hsa_circ_	1.413891	0.025934
hsa_circ_	1.580985	0.025938
hsa_circ_	0.709634	0.025941
hsa_circ_	-0.49157	0.025958
hsa_circ_	-0.77225	0.025971
hsa_circ_	1.386382	0.025976
hsa_circ_	-0.38815	0.025979
hsa_circ_	0.879546	0.025984
hsa_circ_	1.317234	0.025985
hsa_circ_	-0.79055	0.02599
hsa_circ_	-0.93574	0.025993
hsa_circ_	1.061139	0.025995
hsa_circ_	-0.39234	0.026001
hsa_circ_	-0.37078	0.026003
hsa_circ_	0.646312	0.026004
hsa_circ_	1.583688	0.026009
hsa_circ_	-0.87217	0.026011
hsa_circ_	0.696852	0.026016
hsa_circ_	0.861704	0.026017
hsa_circ_	-0.39448	0.026023
hsa_circ_	0.461777	0.026028
hsa_circ_	0.465559	0.026029
hsa_circ_	0.999872	0.026029
hsa_circ_	-0.77918	0.026033
hsa_circ_	0.651429	0.026038
hsa_circ_	0.896709	0.02604
hsa_circ_	0.525003	0.026055
hsa_circ_	0.414413	0.02606
hsa_circ_	1.027437	0.026066
hsa_circ_	0.433507	0.026072
hsa_circ_	-0.55881	0.026075
hsa_circ_	0.702496	0.026088
hsa_circ_	-0.44652	0.026105
hsa_circ_	-0.52021	0.026112
hsa_circ_	1.007396	0.02612
hsa_circ_	0.685864	0.02612
hsa_circ_	-0.66626	0.026124
hsa_circ_	-0.38587	0.026125
hsa_circ_	0.47133	0.026125
hsa_circ_	0.659138	0.026126
hsa_circ_	0.389865	0.026129
hsa_circ_	0.474938	0.02613
hsa_circ_	0.425533	0.026144

hsa_circ_	0.466275	0.026148
hsa_circ_	0.776541	0.026157
hsa_circ_	-0.92088	0.02616
hsa_circ_	-0.57284	0.026172
hsa_circ_	-0.48025	0.026172
hsa_circ_	1.001346	0.026178
hsa_circ_	0.988264	0.026178
hsa_circ_	-0.78671	0.026182
hsa_circ_	-2.00961	0.026183
hsa_circ_	-1.72007	0.026186
hsa_circ_	-1.09504	0.026191
hsa_circ_	-1.49082	0.026194
hsa_circ_	0.386723	0.0262
hsa_circ_	-0.82121	0.026208
hsa_circ_	-0.40678	0.026213
hsa_circ_	0.525105	0.026213
hsa_circ_	-0.50996	0.026221
hsa_circ_	-1.05327	0.026239
hsa_circ_	-0.48436	0.026241
hsa_circ_	-0.40095	0.026246
hsa_circ_	0.912906	0.026259
hsa_circ_	0.494467	0.026268
hsa_circ_	-0.44518	0.02627
hsa_circ_	-0.99493	0.026281
hsa_circ_	0.59179	0.026286
hsa_circ_	0.703932	0.026318
hsa_circ_	-0.44208	0.026328
hsa_circ_	-0.6878	0.026328
hsa_circ_	-0.5808	0.026331
hsa_circ_	0.561026	0.026332
hsa_circ_	-0.43836	0.026334
hsa_circ_	-0.48392	0.026338
hsa_circ_	-1.16661	0.026339
hsa_circ_	-0.6108	0.026339
hsa_circ_	0.345602	0.026343
hsa_circ_	-0.83574	0.026347
hsa_circ_	-0.36361	0.026353
hsa_circ_	-0.46005	0.026363
hsa_circ_	-0.4957	0.026369
hsa_circ_	0.370856	0.02637
hsa_circ_	1.43288	0.026381
hsa_circ_	-0.84233	0.026382
hsa_circ_	-0.60262	0.026382
hsa_circ_	1.114907	0.026383
hsa_circ_	-0.45972	0.026383
hsa_circ_	-0.4303	0.026391
hsa_circ_	0.751786	0.026392
hsa_circ_	0.550517	0.026392
hsa_circ_	-0.48591	0.026393
hsa_circ_	0.766295	0.026413
hsa_circ_	0.400247	0.026416
hsa_circ_	-0.68944	0.026416
hsa_circ_	0.476117	0.02642
hsa_circ_	-1.03883	0.026422

hsa_circ_	-0.81432	0.026425
hsa_circ_	-0.93021	0.026431
hsa_circ_	-0.43132	0.026442
hsa_circ_	0.976792	0.026445
hsa_circ_	-0.55112	0.026451
hsa_circ_	-0.72258	0.026461
hsa_circ_	-0.36013	0.026461
hsa_circ_	-0.51072	0.026465
hsa_circ_	-0.41649	0.026469
hsa_circ_	-0.439	0.026472
hsa_circ_	0.603379	0.026473
hsa_circ_	1.210639	0.02648
hsa_circ_	0.422037	0.026481
hsa_circ_	0.867116	0.026483
hsa_circ_	-0.38757	0.026502
hsa_circ_	-0.44838	0.026517
hsa_circ_	-0.60669	0.02652
hsa_circ_	-0.50349	0.026521
hsa_circ_	-0.47481	0.026523
hsa_circ_	0.360641	0.026524
hsa_circ_	0.442589	0.026525
hsa_circ_	-1.55541	0.026537
hsa_circ_	-0.43157	0.026557
hsa_circ_	-1.1267	0.026568
hsa_circ_	-0.52652	0.026574
hsa_circ_	-0.92526	0.026581
hsa_circ_	-0.48726	0.02659
hsa_circ_	0.598215	0.026591
hsa_circ_	0.933684	0.026596
hsa_circ_	0.969875	0.026597
hsa_circ_	-0.88872	0.026601
hsa_circ_	0.548257	0.026603
hsa_circ_	1.113592	0.026606
hsa_circ_	-0.50454	0.026606
hsa_circ_	0.611527	0.026606
hsa_circ_	0.718989	0.02661
hsa_circ_	-0.39755	0.026614
hsa_circ_	0.603819	0.026618
hsa_circ_	-1.05473	0.026636
hsa_circ_	-0.59403	0.026644
hsa_circ_	1.879698	0.026646
hsa_circ_	0.563573	0.026646
hsa_circ_	-0.79689	0.026647
hsa_circ_	-0.45032	0.02665
hsa_circ_	-0.37528	0.026654
hsa_circ_	-0.51248	0.026655
hsa_circ_	0.600249	0.026659
hsa_circ_	-1.60759	0.026668
hsa_circ_	-0.73291	0.026672
hsa_circ_	0.789494	0.026672
hsa_circ_	-0.71825	0.026686
hsa_circ_	0.393092	0.026686
hsa_circ_	-0.44466	0.026688
hsa_circ_	0.995976	0.026694

hsa_circ_	-0.43099	0.026697
hsa_circ_	-1.07529	0.026698
hsa_circ_	-0.5002	0.026704
hsa_circ_	0.565457	0.026713
hsa_circ_	-0.36838	0.026717
hsa_circ_	0.472282	0.026719
hsa_circ_	0.426506	0.026721
hsa_circ_	-0.35699	0.02673
hsa_circ_	-0.53302	0.02673
hsa_circ_	0.523333	0.026739
hsa_circ_	0.525251	0.026739
hsa_circ_	-0.37236	0.026742
hsa_circ_	0.71378	0.026753
hsa_circ_	0.564621	0.026757
hsa_circ_	1.039424	0.026778
hsa_circ_	-0.40803	0.026778
hsa_circ_	-0.92672	0.026788
hsa_circ_	-0.39026	0.026805
hsa_circ_	-0.70033	0.026809
hsa_circ_	1.609547	0.026811
hsa_circ_	-0.39069	0.026822
hsa_circ_	-0.34469	0.026832
hsa_circ_	-0.56074	0.026834
hsa_circ_	0.418082	0.026843
hsa_circ_	-0.38992	0.026846
hsa_circ_	-0.44894	0.026848
hsa_circ_	-0.45719	0.026855
hsa_circ_	-0.35282	0.026858
hsa_circ_	0.545058	0.026859
hsa_circ_	0.693072	0.026874
hsa_circ_	1.136486	0.026875
hsa_circ_	-0.43327	0.026882
hsa_circ_	0.357447	0.026884
hsa_circ_	-0.5088	0.026887
hsa_circ_	0.55297	0.026893
hsa_circ_	-0.54504	0.026897
hsa_circ_	-0.38177	0.026906
hsa_circ_	-0.50918	0.026909
hsa_circ_	1.207346	0.026926
hsa_circ_	0.484131	0.026934
hsa_circ_	-0.49413	0.026946
hsa_circ_	-0.4257	0.026956
hsa_circ_	-1.23793	0.026957
hsa_circ_	-0.38063	0.026961
hsa_circ_	0.458383	0.026964
hsa_circ_	-0.61784	0.026968
hsa_circ_	0.483054	0.026975
hsa_circ_	-0.43907	0.026975
hsa_circ_	0.900516	0.02699
hsa_circ_	1.347692	0.02699
hsa_circ_	0.568076	0.02699
hsa_circ_	0.353903	0.027015
hsa_circ_	0.657593	0.027016
hsa_circ_	0.664205	0.02702

hsa_circ_	-0.98586	0.027025
hsa_circ_	0.641847	0.02703
hsa_circ_	0.514371	0.027032
hsa_circ_	0.62593	0.027046
hsa_circ_	0.485551	0.027063
hsa_circ_	-0.49215	0.027068
hsa_circ_	0.470126	0.027072
hsa_circ_	-0.55892	0.027073
hsa_circ_	-0.47949	0.027078
hsa_circ_	-0.4228	0.027085
hsa_circ_	-0.42755	0.027087
hsa_circ_	0.691249	0.027094
hsa_circ_	-0.35779	0.027098
hsa_circ_	0.931283	0.027101
hsa_circ_	-0.5767	0.027102
hsa_circ_	0.793957	0.027105
hsa_circ_	0.402752	0.027105
hsa_circ_	-0.36681	0.027106
hsa_circ_	0.877309	0.027109
hsa_circ_	-0.49455	0.027111
hsa_circ_	-0.75197	0.027112
hsa_circ_	0.7898	0.027126
hsa_circ_	-1.35803	0.027128
hsa_circ_	-0.4237	0.027138
hsa_circ_	0.528028	0.027148
hsa_circ_	0.437972	0.027154
hsa_circ_	0.368758	0.027161
hsa_circ_	-0.35227	0.027172
hsa_circ_	-0.3886	0.027177
hsa_circ_	0.490255	0.027177
hsa_circ_	0.637243	0.027178
hsa_circ_	-0.94685	0.027185
hsa_circ_	-0.35641	0.027187
hsa_circ_	1.61323	0.0272
hsa_circ_	1.120782	0.027219
hsa_circ_	0.719461	0.027222
hsa_circ_	1.28111	0.027226
hsa_circ_	0.382441	0.027226
hsa_circ_	-0.49292	0.02723
hsa_circ_	0.713515	0.027238
hsa_circ_	0.391627	0.027243
hsa_circ_	-0.86247	0.027251
hsa_circ_	0.619516	0.027252
hsa_circ_	-0.71869	0.027257
hsa_circ_	1.465672	0.027264
hsa_circ_	0.594371	0.02727
hsa_circ_	0.447025	0.027277
hsa_circ_	1.137808	0.027281
hsa_circ_	0.487657	0.027281
hsa_circ_	-0.80815	0.027293
hsa_circ_	1.128368	0.027299
hsa_circ_	0.548752	0.027303
hsa_circ_	-1.23049	0.02731
hsa_circ_	-1.60375	0.027313

hsa_circ_	0.398931	0.027313
hsa_circ_	-1.1149	0.027322
hsa_circ_	-0.42938	0.027328
hsa_circ_	0.588884	0.027329
hsa_circ_	0.637131	0.027338
hsa_circ_	0.440994	0.027354
hsa_circ_	0.53608	0.027358
hsa_circ_	-0.69395	0.027368
hsa_circ_	-1.04673	0.02737
hsa_circ_	-0.51325	0.027379
hsa_circ_	-0.66479	0.027381
hsa_circ_	-0.4021	0.027383
hsa_circ_	1.098389	0.027388
hsa_circ_	-0.67363	0.0274
hsa_circ_	0.934951	0.027403
hsa_circ_	1.670732	0.027424
hsa_circ_	-1.0653	0.027426
hsa_circ_	0.717437	0.027428
hsa_circ_	-0.73511	0.027429
hsa_circ_	-0.67633	0.027437
hsa_circ_	-0.5134	0.02744
hsa_circ_	0.416291	0.027443
hsa_circ_	0.545674	0.027451
hsa_circ_	0.584134	0.027453
hsa_circ_	-0.42318	0.027454
hsa_circ_	-0.39573	0.027455
hsa_circ_	0.588782	0.027457
hsa_circ_	0.515639	0.027462
hsa_circ_	0.464595	0.027467
hsa_circ_	0.599199	0.027487
hsa_circ_	0.408411	0.027493
hsa_circ_	-0.40507	0.027497
hsa_circ_	0.700185	0.0275
hsa_circ_	-1.09638	0.027502
hsa_circ_	-0.34574	0.027509
hsa_circ_	0.660095	0.02752
hsa_circ_	-0.97208	0.02752
hsa_circ_	1.374794	0.027523
hsa_circ_	-0.49137	0.027525
hsa_circ_	-0.71104	0.027528
hsa_circ_	-1.23026	0.027532
hsa_circ_	0.438467	0.027533
hsa_circ_	0.456192	0.02754
hsa_circ_	-1.05556	0.027555
hsa_circ_	1.064782	0.027555
hsa_circ_	-0.41015	0.027561
hsa_circ_	-0.80858	0.027561
hsa_circ_	0.848343	0.027567
hsa_circ_	-0.50448	0.027569
hsa_circ_	0.754222	0.027569
hsa_circ_	0.947157	0.02757
hsa_circ_	0.737689	0.027571
hsa_circ_	0.589471	0.027585
hsa_circ_	1.027256	0.027591

hsa_circ_	-0.53813	0.027593
hsa_circ_	0.428983	0.027606
hsa_circ_	-0.40563	0.027606
hsa_circ_	-0.5059	0.027613
hsa_circ_	-1.75005	0.027618
hsa_circ_	0.393707	0.027621
hsa_circ_	0.52219	0.02763
hsa_circ_	-0.46141	0.02763
hsa_circ_	0.833842	0.027647
hsa_circ_	0.438318	0.027654
hsa_circ_	0.725218	0.027671
hsa_circ_	-0.75941	0.027673
hsa_circ_	0.400212	0.027674
hsa_circ_	-1.59683	0.027674
hsa_circ_	0.766701	0.027678
hsa_circ_	0.652364	0.027682
hsa_circ_	0.451351	0.027688
hsa_circ_	0.412059	0.027689
hsa_circ_	-0.59181	0.027692
hsa_circ_	0.403948	0.027694
hsa_circ_	-0.6848	0.027705
hsa_circ_	-0.40533	0.027713
hsa_circ_	0.378792	0.027719
hsa_circ_	0.486533	0.02773
hsa_circ_	-0.535	0.027742
hsa_circ_	0.46604	0.027748
hsa_circ_	-0.76258	0.027748
hsa_circ_	-0.67655	0.027752
hsa_circ_	0.885627	0.027753
hsa_circ_	-1.19475	0.027757
hsa_circ_	0.81253	0.027757
hsa_circ_	-0.54349	0.027763
hsa_circ_	-0.75385	0.027768
hsa_circ_	0.709209	0.027768
hsa_circ_	-0.46446	0.027769
hsa_circ_	2.299345	0.027769
hsa_circ_	-0.85556	0.027784
hsa_circ_	0.429575	0.027795
hsa_circ_	0.834363	0.027798
hsa_circ_	0.394788	0.027803
hsa_circ_	-0.39014	0.027816
hsa_circ_	0.653774	0.027822
hsa_circ_	-0.42399	0.027827
hsa_circ_	-0.53239	0.027834
hsa_circ_	1.164425	0.027844
hsa_circ_	0.374658	0.027847
hsa_circ_	-0.62606	0.027849
hsa_circ_	-0.60985	0.02785
hsa_circ_	0.698888	0.027858
hsa_circ_	0.588865	0.027859
hsa_circ_	-0.40464	0.027861
hsa_circ_	0.546557	0.027872
hsa_circ_	-0.39503	0.027873
hsa_circ_	1.056746	0.027873

hsa_circ_	-1.16164	0.027884
hsa_circ_	-1.41068	0.027902
hsa_circ_	0.646111	0.027905
hsa_circ_	-2.19455	0.02792
hsa_circ_	0.513699	0.027922
hsa_circ_	-0.49803	0.027932
hsa_circ_	0.353423	0.027946
hsa_circ_	0.593946	0.02796
hsa_circ_	0.406688	0.027963
hsa_circ_	0.380818	0.027982
hsa_circ_	-0.48629	0.027983
hsa_circ_	-3.18997	0.027983
hsa_circ_	-1.13986	0.02799
hsa_circ_	1.662766	0.028002
hsa_circ_	-1.02978	0.028002
hsa_circ_	-0.49741	0.028007
hsa_circ_	0.50882	0.028008
hsa_circ_	2.146241	0.028021
hsa_circ_	0.925805	0.028027
hsa_circ_	-0.4696	0.02803
hsa_circ_	-0.41501	0.028041
hsa_circ_	0.603829	0.028058
hsa_circ_	-0.46611	0.028067
hsa_circ_	0.368809	0.028067
hsa_circ_	-0.42121	0.028069
hsa_circ_	-0.50398	0.028075
hsa_circ_	0.450738	0.02808
hsa_circ_	-0.59016	0.028081
hsa_circ_	0.589313	0.028083
hsa_circ_	-0.76218	0.028096
hsa_circ_	0.669812	0.028096
hsa_circ_	-0.60925	0.028101
hsa_circ_	-0.42681	0.028102
hsa_circ_	1.154355	0.028107
hsa_circ_	-0.80189	0.028109
hsa_circ_	-0.55822	0.028109
hsa_circ_	0.379578	0.028112
hsa_circ_	-0.97717	0.028113
hsa_circ_	-1.23517	0.028115
hsa_circ_	-0.56092	0.02812
hsa_circ_	-1.28565	0.028123
hsa_circ_	2.014458	0.028139
hsa_circ_	-0.44408	0.028141
hsa_circ_	1.06863	0.028147
hsa_circ_	0.608496	0.028155
hsa_circ_	-0.65426	0.028159
hsa_circ_	-0.44649	0.02816
hsa_circ_	-1.2684	0.028164
hsa_circ_	0.468815	0.02817
hsa_circ_	0.88111	0.028171
hsa_circ_	-0.90401	0.028171
hsa_circ_	-0.38122	0.028174
hsa_circ_	1.363647	0.028175
hsa_circ_	0.750394	0.028177

hsa_circ_	-0.70199	0.028179
hsa_circ_	0.85095	0.028194
hsa_circ_	0.52816	0.028197
hsa_circ_	-0.45607	0.028205
hsa_circ_	0.808378	0.028208
hsa_circ_	-0.75031	0.028208
hsa_circ_	0.47872	0.028215
hsa_circ_	0.447574	0.028217
hsa_circ_	0.491874	0.028226
hsa_circ_	-0.57688	0.028234
hsa_circ_	-0.46154	0.028237
hsa_circ_	-0.43425	0.028237
hsa_circ_	0.580596	0.028238
hsa_circ_	0.398953	0.028246
hsa_circ_	-0.39369	0.02825
hsa_circ_	-0.64284	0.028252
hsa_circ_	-0.57404	0.028255
hsa_circ_	-0.37467	0.028262
hsa_circ_	0.828414	0.028266
hsa_circ_	0.436889	0.028266
hsa_circ_	-0.46588	0.028267
hsa_circ_	0.582836	0.028279
hsa_circ_	0.726163	0.028279
hsa_circ_	0.449248	0.028282
hsa_circ_	0.544054	0.0283
hsa_circ_	0.678096	0.028316
hsa_circ_	-0.45484	0.028316
hsa_circ_	-0.70951	0.028318
hsa_circ_	-1.27331	0.028322
hsa_circ_	-0.44861	0.028325
hsa_circ_	-0.43308	0.028331
hsa_circ_	-0.63521	0.028336
hsa_circ_	-0.50915	0.028337
hsa_circ_	-0.53053	0.028341
hsa_circ_	-1.80786	0.028342
hsa_circ_	-0.56497	0.028342
hsa_circ_	0.595446	0.02835
hsa_circ_	0.467137	0.028355
hsa_circ_	0.461618	0.028361
hsa_circ_	1.354332	0.028366
hsa_circ_	0.446582	0.028373
hsa_circ_	-0.49089	0.028379
hsa_circ_	0.497689	0.028406
hsa_circ_	1.099127	0.028408
hsa_circ_	0.761865	0.028412
hsa_circ_	-0.55601	0.02843
hsa_circ_	-0.58981	0.02843
hsa_circ_	-0.97521	0.028431
hsa_circ_	-0.67752	0.028435
hsa_circ_	-0.877	0.028443
hsa_circ_	0.649344	0.028443
hsa_circ_	-0.5283	0.028447
hsa_circ_	-0.42545	0.028448
hsa_circ_	-0.44553	0.028452

hsa_circ_	-0.62572	0.028452
hsa_circ_	0.633376	0.028454
hsa_circ_	1.786552	0.028457
hsa_circ_	0.771263	0.028459
hsa_circ_	-0.83967	0.028475
hsa_circ_	0.627747	0.02848
hsa_circ_	1.001285	0.02849
hsa_circ_	0.505169	0.028496
hsa_circ_	-0.73558	0.028503
hsa_circ_	-1.03581	0.028524
hsa_circ_	-0.81528	0.028535
hsa_circ_	0.669465	0.02854
hsa_circ_	-1.07383	0.028543
hsa_circ_	0.67848	0.028555
hsa_circ_	-0.73749	0.028559
hsa_circ_	0.352968	0.028563
hsa_circ_	0.390997	0.028575
hsa_circ_	-0.86349	0.028583
hsa_circ_	0.61552	0.028589
hsa_circ_	0.640327	0.028591
hsa_circ_	0.471411	0.028598
hsa_circ_	0.552507	0.028599
hsa_circ_	-0.43825	0.028617
hsa_circ_	-0.8745	0.028618
hsa_circ_	0.420102	0.028632
hsa_circ_	0.7193	0.028636
hsa_circ_	-0.63558	0.028637
hsa_circ_	-0.94311	0.028642
hsa_circ_	-0.60232	0.028642
hsa_circ_	0.65938	0.028653
hsa_circ_	-0.59385	0.028663
hsa_circ_	0.522168	0.028663
hsa_circ_	0.370295	0.028669
hsa_circ_	0.942411	0.02867
hsa_circ_	0.709186	0.028672
hsa_circ_	-0.65654	0.028675
hsa_circ_	-0.73997	0.028676
hsa_circ_	-0.44921	0.028681
hsa_circ_	-0.40724	0.028685
hsa_circ_	0.682667	0.028685
hsa_circ_	-0.84062	0.028693
hsa_circ_	-0.91345	0.028696
hsa_circ_	0.919339	0.028697
hsa_circ_	0.509717	0.028699
hsa_circ_	0.450201	0.0287
hsa_circ_	-0.89728	0.0287
hsa_circ_	-0.47126	0.028712
hsa_circ_	-0.47134	0.028712
hsa_circ_	-0.42645	0.028715
hsa_circ_	0.687226	0.028733
hsa_circ_	-0.69395	0.028741
hsa_circ_	-0.38829	0.028761
hsa_circ_	0.43028	0.028762
hsa_circ_	0.979449	0.028762

hsa_circ_	-0.48093	0.028767
hsa_circ_	-0.48301	0.028767
hsa_circ_	0.555082	0.028769
hsa_circ_	-0.36018	0.02877
hsa_circ_	1.10283	0.028776
hsa_circ_	1.078619	0.028778
hsa_circ_	0.345098	0.028779
hsa_circ_	-0.38656	0.028782
hsa_circ_	0.389269	0.028788
hsa_circ_	-0.94871	0.028807
hsa_circ_	-0.42181	0.028809
hsa_circ_	1.002544	0.02881
hsa_circ_	-0.99841	0.028826
hsa_circ_	0.391446	0.028831
hsa_circ_	0.491499	0.028832
hsa_circ_	0.985604	0.028833
hsa_circ_	1.184983	0.028838
hsa_circ_	0.599388	0.028839
hsa_circ_	0.424467	0.028846
hsa_circ_	0.416997	0.028849
hsa_circ_	0.746324	0.02885
hsa_circ_	0.543259	0.028869
hsa_circ_	0.350467	0.028884
hsa_circ_	0.566323	0.028888
hsa_circ_	0.865359	0.028891
hsa_circ_	-0.38006	0.028894
hsa_circ_	-1.19584	0.028895
hsa_circ_	0.405645	0.028903
hsa_circ_	-0.38575	0.028903
hsa_circ_	-0.78383	0.028906
hsa_circ_	-0.5806	0.028914
hsa_circ_	-0.38077	0.028916
hsa_circ_	-0.54157	0.028917
hsa_circ_	0.681716	0.028921
hsa_circ_	0.546836	0.028922
hsa_circ_	0.759651	0.028926
hsa_circ_	0.475584	0.028926
hsa_circ_	-0.62163	0.028934
hsa_circ_	-0.40876	0.028938
hsa_circ_	0.484156	0.028938
hsa_circ_	0.686923	0.02894
hsa_circ_	0.35821	0.028941
hsa_circ_	-0.43204	0.028943
hsa_circ_	-0.60321	0.02895
hsa_circ_	0.788346	0.028953
hsa_circ_	0.576545	0.028956
hsa_circ_	-0.74159	0.02896
hsa_circ_	-1.00396	0.028978
hsa_circ_	-1.80257	0.028981
hsa_circ_	-0.48241	0.028983
hsa_circ_	-0.4199	0.028994
hsa_circ_	-0.45229	0.028998
hsa_circ_	0.369489	0.029004
hsa_circ_	-0.521	0.029005

hsa_circ_	-0.58026	0.029006
hsa_circ_	-0.60164	0.02901
hsa_circ_	-1.23121	0.029014
hsa_circ_	0.429532	0.029016
hsa_circ_	-0.44773	0.02902
hsa_circ_	-0.64505	0.029038
hsa_circ_	1.06272	0.029049
hsa_circ_	0.598529	0.029049
hsa_circ_	1.282419	0.029053
hsa_circ_	-0.79385	0.029057
hsa_circ_	-1.14573	0.029064
hsa_circ_	0.425557	0.029067
hsa_circ_	0.888456	0.029078
hsa_circ_	-0.58849	0.029085
hsa_circ_	-0.4766	0.029085
hsa_circ_	-0.57018	0.029087
hsa_circ_	0.598366	0.029093
hsa_circ_	0.906847	0.029098
hsa_circ_	-0.82014	0.029113
hsa_circ_	1.249128	0.029125
hsa_circ_	0.68054	0.029125
hsa_circ_	-1.28343	0.029132
hsa_circ_	-0.87316	0.029134
hsa_circ_	0.528894	0.029135
hsa_circ_	-0.57585	0.029139
hsa_circ_	-1.04854	0.029144
hsa_circ_	0.652515	0.029151
hsa_circ_	-0.46112	0.029157
hsa_circ_	-0.76002	0.029158
hsa_circ_	-0.82489	0.029167
hsa_circ_	-2.3427	0.029167
hsa_circ_	0.417736	0.029176
hsa_circ_	-0.40237	0.029188
hsa_circ_	0.381144	0.029189
hsa_circ_	0.492334	0.029191
hsa_circ_	0.696802	0.029193
hsa_circ_	-0.45203	0.029193
hsa_circ_	-1.35059	0.029197
hsa_circ_	0.784586	0.029207
hsa_circ_	-0.90677	0.02921
hsa_circ_	0.701738	0.029212
hsa_circ_	-0.69966	0.029213
hsa_circ_	0.48857	0.029224
hsa_circ_	0.428765	0.029228
hsa_circ_	-0.47957	0.029243
hsa_circ_	-0.74466	0.029243
hsa_circ_	0.41033	0.029243
hsa_circ_	-0.55437	0.029245
hsa_circ_	0.773458	0.029247
hsa_circ_	-0.72836	0.029257
hsa_circ_	-0.7445	0.029262
hsa_circ_	0.615554	0.029281
hsa_circ_	0.354774	0.029298
hsa_circ_	0.748092	0.029298

hsa_circ_	0.426067	0.029304
hsa_circ_	0.805104	0.029306
hsa_circ_	0.736339	0.02931
hsa_circ_	1.235474	0.029318
hsa_circ_	0.666309	0.02933
hsa_circ_	0.898063	0.029336
hsa_circ_	0.458671	0.029342
hsa_circ_	-0.46365	0.029342
hsa_circ_	0.752161	0.029345
hsa_circ_	0.742043	0.029346
hsa_circ_	0.791411	0.029352
hsa_circ_	-0.40721	0.029355
hsa_circ_	-0.50054	0.029355
hsa_circ_	-0.42228	0.02936
hsa_circ_	0.704906	0.029364
hsa_circ_	0.532058	0.029367
hsa_circ_	-0.43209	0.02937
hsa_circ_	-0.47836	0.029374
hsa_circ_	1.154368	0.029375
hsa_circ_	0.52318	0.029375
hsa_circ_	0.59457	0.029383
hsa_circ_	-0.76552	0.029396
hsa_circ_	0.568396	0.029399
hsa_circ_	0.466844	0.029401
hsa_circ_	-0.49511	0.029402
hsa_circ_	-0.48857	0.029415
hsa_circ_	-0.51415	0.029416
hsa_circ_	0.563611	0.029418
hsa_circ_	-0.58864	0.02942
hsa_circ_	0.494624	0.029422
hsa_circ_	0.551993	0.029429
hsa_circ_	-1.38978	0.029431
hsa_circ_	0.979419	0.029434
hsa_circ_	-1.18852	0.029439
hsa_circ_	-1.13993	0.02944
hsa_circ_	0.37628	0.029441
hsa_circ_	0.42964	0.029446
hsa_circ_	-0.37841	0.02945
hsa_circ_	-0.39394	0.02945
hsa_circ_	0.677079	0.029451
hsa_circ_	0.586392	0.029455
hsa_circ_	1.057927	0.029465
hsa_circ_	-0.65888	0.029467
hsa_circ_	-0.38729	0.029467
hsa_circ_	0.562843	0.029475
hsa_circ_	1.027375	0.02949
hsa_circ_	-0.73959	0.029506
hsa_circ_	-0.37651	0.029515
hsa_circ_	0.349518	0.029517
hsa_circ_	0.613649	0.029523
hsa_circ_	-0.53612	0.029525
hsa_circ_	0.483756	0.029526
hsa_circ_	-0.47237	0.029529
hsa_circ_	-0.42341	0.029553

hsa_circ_	-0.49391	0.029554
hsa_circ_	0.384284	0.029559
hsa_circ_	-0.50734	0.029563
hsa_circ_	-0.43762	0.02957
hsa_circ_	0.417115	0.029581
hsa_circ_	0.740088	0.029584
hsa_circ_	0.530364	0.02959
hsa_circ_	-0.52539	0.029594
hsa_circ_	0.390154	0.029609
hsa_circ_	0.649519	0.029612
hsa_circ_	0.368612	0.029621
hsa_circ_	1.080125	0.029626
hsa_circ_	0.72803	0.029632
hsa_circ_	0.738193	0.029646
hsa_circ_	-0.5345	0.029653
hsa_circ_	0.606243	0.02966
hsa_circ_	0.650346	0.02966
hsa_circ_	-0.63634	0.029661
hsa_circ_	-0.94322	0.029667
hsa_circ_	0.472922	0.029668
hsa_circ_	-0.37997	0.029671
hsa_circ_	0.551592	0.029676
hsa_circ_	0.536396	0.029679
hsa_circ_	-0.85865	0.029698
hsa_circ_	-0.52974	0.029702
hsa_circ_	0.400667	0.029705
hsa_circ_	0.41501	0.029708
hsa_circ_	-0.65988	0.029714
hsa_circ_	0.447907	0.029718
hsa_circ_	-0.44642	0.029725
hsa_circ_	-0.86311	0.029731
hsa_circ_	0.673268	0.029735
hsa_circ_	0.602654	0.029736
hsa_circ_	0.49201	0.029737
hsa_circ_	-0.41586	0.029737
hsa_circ_	0.549266	0.029743
hsa_circ_	-0.51162	0.029758
hsa_circ_	0.623014	0.029764
hsa_circ_	-0.95217	0.029767
hsa_circ_	-0.50945	0.029767
hsa_circ_	0.758092	0.029767
hsa_circ_	1.083013	0.029771
hsa_circ_	-0.39267	0.029776
hsa_circ_	1.062533	0.029776
hsa_circ_	0.412193	0.029778
hsa_circ_	-0.42211	0.029784
hsa_circ_	-0.55157	0.029785
hsa_circ_	0.410591	0.029788
hsa_circ_	0.404762	0.029791
hsa_circ_	0.464434	0.029792
hsa_circ_	-0.51529	0.029794
hsa_circ_	-0.81634	0.029799
hsa_circ_	-0.91821	0.029801
hsa_circ_	0.816515	0.029801

hsa_circ_	-0.52076	0.029802
hsa_circ_	-0.73664	0.029807
hsa_circ_	-0.53301	0.029809
hsa_circ_	-0.39291	0.02981
hsa_circ_	-0.53306	0.029826
hsa_circ_	0.49037	0.029836
hsa_circ_	-0.42361	0.02984
hsa_circ_	0.423577	0.029855
hsa_circ_	0.677934	0.029857
hsa_circ_	0.788715	0.029868
hsa_circ_	0.414547	0.029871
hsa_circ_	0.642651	0.029878
hsa_circ_	-0.39699	0.029881
hsa_circ_	-0.35943	0.029886
hsa_circ_	-0.50481	0.029886
hsa_circ_	-0.79814	0.0299
hsa_circ_	0.389882	0.029905
hsa_circ_	0.508994	0.029905
hsa_circ_	0.600423	0.029905
hsa_circ_	-0.5342	0.029905
hsa_circ_	0.406319	0.029909
hsa_circ_	-0.35487	0.029913
hsa_circ_	0.393292	0.029913
hsa_circ_	-0.494	0.029925
hsa_circ_	0.944174	0.029927
hsa_circ_	0.514359	0.029929
hsa_circ_	-0.69009	0.029929
hsa_circ_	0.466401	0.029932
hsa_circ_	0.96134	0.029943
hsa_circ_	-0.85292	0.029944
hsa_circ_	0.571995	0.02995
hsa_circ_	-0.55354	0.029951
hsa_circ_	-0.50052	0.029951
hsa_circ_	0.496923	0.029955
hsa_circ_	-1.02849	0.029957
hsa_circ_	-0.81278	0.029959
hsa_circ_	-0.69089	0.029984
hsa_circ_	0.619249	0.029986
hsa_circ_	-0.92039	0.029989
hsa_circ_	0.40679	0.02999
hsa_circ_	-0.3929	0.030007
hsa_circ_	0.804189	0.030012
hsa_circ_	-1.82049	0.030017
hsa_circ_	-0.61583	0.030025
hsa_circ_	0.526785	0.030027
hsa_circ_	-0.7022	0.030032
hsa_circ_	-0.38877	0.030036
hsa_circ_	0.418042	0.030043
hsa_circ_	0.613939	0.030051
hsa_circ_	0.367373	0.030052
hsa_circ_	0.498464	0.030064
hsa_circ_	0.523932	0.030065
hsa_circ_	-0.60328	0.030066
hsa_circ_	-0.43306	0.030083

hsa_circ_	-0.43661	0.030084
hsa_circ_	-0.41849	0.03009
hsa_circ_	-0.44777	0.03009
hsa_circ_	-0.51288	0.030107
hsa_circ_	-0.96007	0.030112
hsa_circ_	-0.40246	0.030114
hsa_circ_	0.536392	0.030115
hsa_circ_	0.419799	0.030135
hsa_circ_	-0.55869	0.030138
hsa_circ_	-0.40406	0.030149
hsa_circ_	0.865461	0.030151
hsa_circ_	-0.74949	0.030151
hsa_circ_	-0.62567	0.030155
hsa_circ_	0.646746	0.030158
hsa_circ_	-0.34785	0.030172
hsa_circ_	-0.87468	0.030175
hsa_circ_	-0.43179	0.030178
hsa_circ_	-0.38612	0.030181
hsa_circ_	0.737696	0.030188
hsa_circ_	-0.5824	0.030191
hsa_circ_	0.998091	0.030194
hsa_circ_	-0.4804	0.030209
hsa_circ_	0.480919	0.030219
hsa_circ_	0.453368	0.030225
hsa_circ_	-0.54489	0.030233
hsa_circ_	-0.37731	0.030234
hsa_circ_	-0.5509	0.030247
hsa_circ_	-0.38396	0.030263
hsa_circ_	-0.35675	0.03027
hsa_circ_	-0.50115	0.030282
hsa_circ_	-0.7679	0.030295
hsa_circ_	-0.56726	0.030295
hsa_circ_	-0.46739	0.0303
hsa_circ_	-0.78053	0.0303
hsa_circ_	0.519919	0.030314
hsa_circ_	-0.73003	0.030322
hsa_circ_	-1.19758	0.030323
hsa_circ_	0.477834	0.03033
hsa_circ_	-0.90969	0.030338
hsa_circ_	0.480117	0.030342
hsa_circ_	0.638306	0.030344
hsa_circ_	-0.70482	0.03036
hsa_circ_	-1.80118	0.030371
hsa_circ_	0.440113	0.030371
hsa_circ_	0.412833	0.030382
hsa_circ_	0.917645	0.030386
hsa_circ_	0.485035	0.030388
hsa_circ_	-0.51847	0.030391
hsa_circ_	-0.4012	0.030393
hsa_circ_	-0.36552	0.030406
hsa_circ_	0.418858	0.03041
hsa_circ_	0.800024	0.03042
hsa_circ_	0.459145	0.030424
hsa_circ_	0.860141	0.030437

hsa_circ_	-0.3824	0.030437
hsa_circ_	1.277839	0.030443
hsa_circ_	0.506573	0.030451
hsa_circ_	-0.79851	0.030453
hsa_circ_	-0.46856	0.030455
hsa_circ_	0.428628	0.030465
hsa_circ_	-0.41646	0.030474
hsa_circ_	-0.64314	0.03048
hsa_circ_	-0.58804	0.030487
hsa_circ_	0.913717	0.030489
hsa_circ_	1.373869	0.0305
hsa_circ_	-0.42707	0.030501
hsa_circ_	-0.36696	0.030511
hsa_circ_	1.017883	0.030512
hsa_circ_	-0.50581	0.030512
hsa_circ_	-0.37217	0.030513
hsa_circ_	0.618096	0.030522
hsa_circ_	0.656077	0.030547
hsa_circ_	0.368559	0.030551
hsa_circ_	-1.69592	0.030558
hsa_circ_	-0.84436	0.03056
hsa_circ_	0.425266	0.030564
hsa_circ_	1.428338	0.030564
hsa_circ_	0.386785	0.030568
hsa_circ_	0.657142	0.030572
hsa_circ_	0.421403	0.030575
hsa_circ_	-1.01358	0.03058
hsa_circ_	-0.41702	0.030584
hsa_circ_	-0.52247	0.030584
hsa_circ_	0.535221	0.030587
hsa_circ_	0.698582	0.030589
hsa_circ_	0.414277	0.03059
hsa_circ_	-0.34404	0.030594
hsa_circ_	0.367843	0.030599
hsa_circ_	-0.46964	0.030602
hsa_circ_	0.831153	0.030612
hsa_circ_	0.522014	0.030617
hsa_circ_	-0.54248	0.030623
hsa_circ_	0.470106	0.030623
hsa_circ_	0.529354	0.030629
hsa_circ_	0.874944	0.030637
hsa_circ_	0.570198	0.030639
hsa_circ_	-0.48444	0.030648
hsa_circ_	-0.46791	0.030652
hsa_circ_	-0.52234	0.030656
hsa_circ_	-0.36789	0.030663
hsa_circ_	0.445107	0.030667
hsa_circ_	-0.44213	0.030682
hsa_circ_	-0.43841	0.030683
hsa_circ_	-0.37991	0.030698
hsa_circ_	-0.42426	0.030701
hsa_circ_	-0.55621	0.030702
hsa_circ_	0.645677	0.03071
hsa_circ_	1.150093	0.030712

hsa_circ_	-0.51626	0.030719
hsa_circ_	-0.3662	0.030726
hsa_circ_	-0.93977	0.030727
hsa_circ_	-0.50888	0.030727
hsa_circ_	-0.63937	0.030732
hsa_circ_	-0.76763	0.030742
hsa_circ_	0.423472	0.030743
hsa_circ_	0.645397	0.030746
hsa_circ_	-0.4731	0.030756
hsa_circ_	0.416158	0.030758
hsa_circ_	0.454886	0.030763
hsa_circ_	0.656942	0.030765
hsa_circ_	-0.39657	0.030765
hsa_circ_	-1.04967	0.030772
hsa_circ_	-0.56895	0.030792
hsa_circ_	-0.39049	0.030797
hsa_circ_	0.513217	0.030797
hsa_circ_	0.772104	0.030804
hsa_circ_	0.458674	0.030815
hsa_circ_	-0.44588	0.030821
hsa_circ_	0.520156	0.03083
hsa_circ_	-0.42711	0.030831
hsa_circ_	0.692517	0.030842
hsa_circ_	0.432553	0.030843
hsa_circ_	-0.35962	0.030843
hsa_circ_	-0.9237	0.030844
hsa_circ_	0.544377	0.030849
hsa_circ_	-0.54756	0.030851
hsa_circ_	0.492078	0.030857
hsa_circ_	-0.45504	0.030858
hsa_circ_	-0.826	0.030861
hsa_circ_	0.942154	0.030862
hsa_circ_	0.469316	0.030877
hsa_circ_	0.762381	0.030882
hsa_circ_	1.244722	0.030889
hsa_circ_	-0.52117	0.030889
hsa_circ_	-0.63916	0.03089
hsa_circ_	0.918511	0.030894
hsa_circ_	0.535973	0.030904
hsa_circ_	-0.35003	0.030904
hsa_circ_	-0.42755	0.030909
hsa_circ_	-1.07675	0.03091
hsa_circ_	-0.54638	0.030928
hsa_circ_	-0.40692	0.030931
hsa_circ_	-0.39925	0.030938
hsa_circ_	-0.46619	0.030944
hsa_circ_	-0.98643	0.030946
hsa_circ_	-0.7044	0.030949
hsa_circ_	0.381124	0.03095
hsa_circ_	0.673779	0.030963
hsa_circ_	0.497282	0.030968
hsa_circ_	0.543295	0.03098
hsa_circ_	0.589744	0.030985
hsa_circ_	0.706183	0.030986

hsa_circ_	0.689188	0.031001
hsa_circ_	-0.71685	0.031014
hsa_circ_	0.997859	0.031022
hsa_circ_	-0.43864	0.031022
hsa_circ_	0.627564	0.031023
hsa_circ_	-0.42817	0.031042
hsa_circ_	-0.4451	0.031049
hsa_circ_	-0.74168	0.031049
hsa_circ_	-0.53442	0.031054
hsa_circ_	2.004689	0.031057
hsa_circ_	-0.60269	0.03106
hsa_circ_	0.725591	0.031063
hsa_circ_	-0.52646	0.031066
hsa_circ_	-0.70232	0.031068
hsa_circ_	0.73538	0.031069
hsa_circ_	-0.57091	0.031077
hsa_circ_	0.458673	0.031084
hsa_circ_	0.434079	0.0311
hsa_circ_	-0.50379	0.031109
hsa_circ_	1.149855	0.031116
hsa_circ_	-0.90622	0.031118
hsa_circ_	0.7392	0.031119
hsa_circ_	-0.69645	0.031128
hsa_circ_	0.656289	0.031136
hsa_circ_	-0.38599	0.031147
hsa_circ_	0.40924	0.031151
hsa_circ_	1.183071	0.031155
hsa_circ_	-1.38943	0.031157
hsa_circ_	-0.48021	0.031158
hsa_circ_	-0.81051	0.031172
hsa_circ_	0.744008	0.031173
hsa_circ_	0.404191	0.031174
hsa_circ_	0.506013	0.031178
hsa_circ_	0.382787	0.031178
hsa_circ_	0.344793	0.031181
hsa_circ_	-1.34208	0.031183
hsa_circ_	-0.61279	0.031187
hsa_circ_	-0.66179	0.031189
hsa_circ_	-0.41122	0.03119
hsa_circ_	-0.38288	0.031197
hsa_circ_	-0.49299	0.031199
hsa_circ_	0.501699	0.031202
hsa_circ_	0.367976	0.031203
hsa_circ_	1.0136	0.031205
hsa_circ_	0.561952	0.031208
hsa_circ_	-0.6335	0.031208
hsa_circ_	0.763424	0.031211
hsa_circ_	-0.62219	0.031213
hsa_circ_	-0.35121	0.031216
hsa_circ_	-0.39705	0.031229
hsa_circ_	-0.7641	0.03123
hsa_circ_	1.132263	0.031232
hsa_circ_	0.600663	0.03124
hsa_circ_	0.699459	0.03124

hsa_circ_	0.540852	0.03124
hsa_circ_	1.09887	0.031249
hsa_circ_	-0.68357	0.031255
hsa_circ_	0.407605	0.03126
hsa_circ_	-0.426	0.031265
hsa_circ_	0.482604	0.031276
hsa_circ_	1.636231	0.031281
hsa_circ_	1.409708	0.031288
hsa_circ_	1.270508	0.031292
hsa_circ_	-0.33663	0.031296
hsa_circ_	-1.02817	0.031304
hsa_circ_	-0.46688	0.031309
hsa_circ_	-0.39414	0.031319
hsa_circ_	0.901034	0.031331
hsa_circ_	-0.39805	0.031335
hsa_circ_	-0.81444	0.03134
hsa_circ_	0.414989	0.031349
hsa_circ_	0.55434	0.031351
hsa_circ_	0.555085	0.031354
hsa_circ_	0.439023	0.031356
hsa_circ_	-0.39863	0.031358
hsa_circ_	0.633079	0.031362
hsa_circ_	-0.53076	0.031363
hsa_circ_	-0.4727	0.031363
hsa_circ_	0.622603	0.031372
hsa_circ_	-0.43615	0.031377
hsa_circ_	-0.60159	0.031384
hsa_circ_	-1.03165	0.031384
hsa_circ_	0.532592	0.031393
hsa_circ_	0.84255	0.031394
hsa_circ_	-0.55747	0.031395
hsa_circ_	-0.37169	0.0314
hsa_circ_	1.548914	0.031405
hsa_circ_	0.606223	0.031409
hsa_circ_	-0.39193	0.031412
hsa_circ_	0.497843	0.031412
hsa_circ_	0.448461	0.031424
hsa_circ_	1.220912	0.031424
hsa_circ_	0.626528	0.031426
hsa_circ_	0.422038	0.031434
hsa_circ_	0.358388	0.031437
hsa_circ_	0.691815	0.031441
hsa_circ_	-0.71884	0.031447
hsa_circ_	-0.35934	0.031449
hsa_circ_	-0.60391	0.031454
hsa_circ_	0.373762	0.031454
hsa_circ_	-0.35023	0.031463
hsa_circ_	0.516832	0.031463
hsa_circ_	-1.05099	0.031465
hsa_circ_	0.632674	0.03148
hsa_circ_	-0.43136	0.03148
hsa_circ_	-0.39014	0.03149
hsa_circ_	-0.50292	0.031493
hsa_circ_	-0.39529	0.031494

hsa_circ_	-0.79615	0.031497
hsa_circ_	0.362277	0.031504
hsa_circ_	0.492608	0.031508
hsa_circ_	0.581691	0.031511
hsa_circ_	0.760574	0.031517
hsa_circ_	0.426973	0.031523
hsa_circ_	0.443147	0.031529
hsa_circ_	-0.50183	0.031539
hsa_circ_	0.650577	0.03154
hsa_circ_	1.423707	0.031541
hsa_circ_	0.41171	0.031548
hsa_circ_	0.508203	0.031553
hsa_circ_	-0.70888	0.031559
hsa_circ_	-1.77894	0.031569
hsa_circ_	-0.57644	0.031581
hsa_circ_	0.343891	0.031583
hsa_circ_	0.929811	0.031598
hsa_circ_	0.913987	0.031603
hsa_circ_	1.32627	0.031621
hsa_circ_	0.53074	0.03163
hsa_circ_	-0.45382	0.031633
hsa_circ_	-0.69789	0.031639
hsa_circ_	-0.68914	0.03164
hsa_circ_	-0.92693	0.031641
hsa_circ_	-0.34658	0.031657
hsa_circ_	0.478956	0.031664
hsa_circ_	-0.74431	0.031665
hsa_circ_	-0.5726	0.031666
hsa_circ_	-0.53632	0.031669
hsa_circ_	-0.393	0.031673
hsa_circ_	-1.5525	0.031675
hsa_circ_	-0.62149	0.031688
hsa_circ_	-0.36525	0.031695
hsa_circ_	0.365518	0.031698
hsa_circ_	0.817895	0.031699
hsa_circ_	-0.43642	0.031704
hsa_circ_	0.477976	0.03171
hsa_circ_	1.337007	0.031711
hsa_circ_	0.593971	0.031711
hsa_circ_	0.533408	0.031712
hsa_circ_	-0.87759	0.031716
hsa_circ_	-0.36122	0.031721
hsa_circ_	0.375864	0.031724
hsa_circ_	0.814217	0.031727
hsa_circ_	-0.44104	0.03173
hsa_circ_	-0.46607	0.031732
hsa_circ_	-0.67154	0.031733
hsa_circ_	1.094955	0.031747
hsa_circ_	0.386327	0.031751
hsa_circ_	-0.60792	0.031753
hsa_circ_	-0.62405	0.031774
hsa_circ_	0.500714	0.031789
hsa_circ_	-0.40025	0.031789
hsa_circ_	-0.41313	0.031807

hsa_circ_	-0.38873	0.031812
hsa_circ_	0.441708	0.031819
hsa_circ_	-0.37556	0.031822
hsa_circ_	0.862578	0.031833
hsa_circ_	-0.61074	0.031833
hsa_circ_	-0.88674	0.031835
hsa_circ_	1.420912	0.031836
hsa_circ_	0.714894	0.031839
hsa_circ_	-0.7135	0.031844
hsa_circ_	-1.0799	0.031859
hsa_circ_	-0.6968	0.031867
hsa_circ_	-0.79505	0.031887
hsa_circ_	0.951652	0.031889
hsa_circ_	0.451686	0.03189
hsa_circ_	0.995226	0.031896
hsa_circ_	0.848669	0.031904
hsa_circ_	0.517907	0.031906
hsa_circ_	-0.64893	0.031911
hsa_circ_	0.759961	0.031924
hsa_circ_	-0.58945	0.031928
hsa_circ_	0.536369	0.03193
hsa_circ_	0.45467	0.03193
hsa_circ_	-0.42925	0.031936
hsa_circ_	-1.23555	0.031936
hsa_circ_	0.473341	0.031942
hsa_circ_	1.47347	0.031944
hsa_circ_	-0.51156	0.031946
hsa_circ_	1.550223	0.031947
hsa_circ_	-0.4208	0.03195
hsa_circ_	-0.54404	0.03196
hsa_circ_	0.444401	0.031962
hsa_circ_	-0.41259	0.031963
hsa_circ_	-0.65803	0.031963
hsa_circ_	0.385538	0.031965
hsa_circ_	-1.33294	0.031967
hsa_circ_	0.45709	0.03197
hsa_circ_	0.597685	0.031979
hsa_circ_	0.747825	0.031984
hsa_circ_	-1.77844	0.031989
hsa_circ_	-0.58646	0.031991
hsa_circ_	0.476017	0.031996
hsa_circ_	0.550757	0.031998
hsa_circ_	-0.91748	0.032012
hsa_circ_	0.394819	0.032027
hsa_circ_	0.466099	0.032029
hsa_circ_	0.531974	0.032036
hsa_circ_	0.460327	0.032039
hsa_circ_	0.583716	0.032042
hsa_circ_	0.845916	0.032054
hsa_circ_	0.754705	0.032063
hsa_circ_	-0.59039	0.032073
hsa_circ_	0.693887	0.03208
hsa_circ_	0.855458	0.032083
hsa_circ_	-0.84956	0.032085

hsa_circ_	0.48425	0.032085
hsa_circ_	0.584385	0.032086
hsa_circ_	0.394551	0.03209
hsa_circ_	-0.43692	0.03209
hsa_circ_	0.381874	0.032092
hsa_circ_	-1.42413	0.032092
hsa_circ_	-0.36306	0.032095
hsa_circ_	0.575086	0.032108
hsa_circ_	0.621891	0.032114
hsa_circ_	0.33889	0.032133
hsa_circ_	-0.47746	0.032135
hsa_circ_	0.706319	0.032148
hsa_circ_	0.377377	0.032153
hsa_circ_	-0.40059	0.032153
hsa_circ_	-0.42241	0.032155
hsa_circ_	-0.37672	0.032164
hsa_circ_	-0.50265	0.032167
hsa_circ_	-0.96581	0.032169
hsa_circ_	0.777657	0.032172
hsa_circ_	-0.7638	0.03218
hsa_circ_	-0.84449	0.032182
hsa_circ_	0.66091	0.032184
hsa_circ_	0.663324	0.032188
hsa_circ_	1.030816	0.03219
hsa_circ_	0.677571	0.032198
hsa_circ_	0.964624	0.032199
hsa_circ_	0.347451	0.032205
hsa_circ_	-0.81031	0.032228
hsa_circ_	0.412822	0.032232
hsa_circ_	-0.68246	0.032239
hsa_circ_	0.488675	0.032241
hsa_circ_	-0.46249	0.032245
hsa_circ_	0.513129	0.03225
hsa_circ_	-0.34708	0.032269
hsa_circ_	0.939951	0.032281
hsa_circ_	0.394814	0.032302
hsa_circ_	0.534486	0.032302
hsa_circ_	-0.45202	0.032308
hsa_circ_	-0.35314	0.032318
hsa_circ_	-0.93965	0.032329
hsa_circ_	-0.33279	0.032337
hsa_circ_	0.456095	0.032344
hsa_circ_	-0.67478	0.032348
hsa_circ_	-1.87482	0.032356
hsa_circ_	1.715953	0.032361
hsa_circ_	0.537353	0.032362
hsa_circ_	-0.37392	0.03237
hsa_circ_	0.577326	0.032371
hsa_circ_	1.137013	0.032372
hsa_circ_	1.417966	0.032392
hsa_circ_	-0.66595	0.032395
hsa_circ_	-0.64405	0.032395
hsa_circ_	-1.016	0.032398
hsa_circ_	0.448492	0.032407

hsa_circ_	1.732935	0.032407
hsa_circ_	-0.42198	0.032422
hsa_circ_	-0.73313	0.032427
hsa_circ_	0.351664	0.032428
hsa_circ_	0.74968	0.032432
hsa_circ_	-0.50185	0.032443
hsa_circ_	0.516776	0.032444
hsa_circ_	-0.7956	0.03245
hsa_circ_	-0.54722	0.032452
hsa_circ_	-1.32759	0.032464
hsa_circ_	-0.57762	0.032466
hsa_circ_	-0.421	0.03247
hsa_circ_	-0.54341	0.03247
hsa_circ_	0.403119	0.03247
hsa_circ_	-0.55337	0.032471
hsa_circ_	0.684027	0.032502
hsa_circ_	-0.39637	0.032504
hsa_circ_	0.520912	0.032507
hsa_circ_	-0.77223	0.032508
hsa_circ_	0.449414	0.032508
hsa_circ_	0.408566	0.032514
hsa_circ_	-0.46939	0.032519
hsa_circ_	0.522775	0.032527
hsa_circ_	0.557479	0.032531
hsa_circ_	-0.61056	0.032532
hsa_circ_	1.031514	0.032544
hsa_circ_	0.630322	0.032548
hsa_circ_	0.435003	0.032549
hsa_circ_	0.457061	0.032549
hsa_circ_	0.396721	0.032551
hsa_circ_	0.673366	0.032554
hsa_circ_	0.711368	0.032554
hsa_circ_	0.662298	0.032556
hsa_circ_	-0.39671	0.03256
hsa_circ_	-0.73703	0.032563
hsa_circ_	0.380571	0.032573
hsa_circ_	0.474142	0.032574
hsa_circ_	-0.43664	0.032575
hsa_circ_	-0.35987	0.032579
hsa_circ_	0.368985	0.032582
hsa_circ_	-1.67747	0.032584
hsa_circ_	-0.64652	0.032587
hsa_circ_	-1.21906	0.032588
hsa_circ_	-0.57026	0.032589
hsa_circ_	-0.49485	0.032613
hsa_circ_	-0.82322	0.032615
hsa_circ_	0.525706	0.032617
hsa_circ_	0.524178	0.032621
hsa_circ_	-0.79842	0.032629
hsa_circ_	-0.75493	0.032644
hsa_circ_	-0.43737	0.032655
hsa_circ_	0.401761	0.032663
hsa_circ_	-0.85201	0.032667
hsa_circ_	-0.37014	0.032668

hsa_circ_	1.256809	0.032671
hsa_circ_	-0.56489	0.032676
hsa_circ_	0.442477	0.032688
hsa_circ_	-0.80884	0.032698
hsa_circ_	0.465937	0.032716
hsa_circ_	1.702132	0.032719
hsa_circ_	0.451699	0.032723
hsa_circ_	0.968443	0.032727
hsa_circ_	0.53508	0.032727
hsa_circ_	0.925611	0.032737
hsa_circ_	-0.38952	0.032743
hsa_circ_	-0.98555	0.032749
hsa_circ_	-0.73442	0.03275
hsa_circ_	-0.38617	0.032751
hsa_circ_	-0.36008	0.032753
hsa_circ_	-0.53295	0.032772
hsa_circ_	0.380706	0.032772
hsa_circ_	1.988657	0.032778
hsa_circ_	0.623911	0.03278
hsa_circ_	0.450303	0.032795
hsa_circ_	0.508698	0.032795
hsa_circ_	-0.68749	0.0328
hsa_circ_	-0.52443	0.032802
hsa_circ_	0.457052	0.032806
hsa_circ_	-0.64166	0.032815
hsa_circ_	0.642735	0.032823
hsa_circ_	0.647276	0.032837
hsa_circ_	0.63777	0.032848
hsa_circ_	-0.38773	0.032854
hsa_circ_	-0.50544	0.032856
hsa_circ_	0.616625	0.032857
hsa_circ_	-0.40204	0.03286
hsa_circ_	0.733436	0.032872
hsa_circ_	-0.45666	0.032878
hsa_circ_	-0.47023	0.032879
hsa_circ_	-0.42037	0.032879
hsa_circ_	0.563526	0.032886
hsa_circ_	0.332833	0.032889
hsa_circ_	0.676514	0.032894
hsa_circ_	-0.59545	0.032896
hsa_circ_	0.467935	0.032899
hsa_circ_	0.565696	0.032901
hsa_circ_	-0.36509	0.03291
hsa_circ_	-0.63506	0.03291
hsa_circ_	0.416529	0.032919
hsa_circ_	0.611422	0.032919
hsa_circ_	-0.36087	0.032921
hsa_circ_	-0.60796	0.032923
hsa_circ_	0.613197	0.032929
hsa_circ_	1.130804	0.032931
hsa_circ_	0.611909	0.032937
hsa_circ_	1.212007	0.032937
hsa_circ_	-1.21157	0.032941
hsa_circ_	-0.63897	0.032944

hsa_circ_	0.858022	0.032947
hsa_circ_	-0.41594	0.03295
hsa_circ_	0.418109	0.032957
hsa_circ_	1.074443	0.032961
hsa_circ_	0.841799	0.032972
hsa_circ_	0.65687	0.032999
hsa_circ_	-0.98003	0.033001
hsa_circ_	0.957462	0.033002
hsa_circ_	0.447736	0.033008
hsa_circ_	0.413845	0.033017
hsa_circ_	0.811154	0.033029
hsa_circ_	-0.82259	0.033034
hsa_circ_	0.405635	0.033035
hsa_circ_	0.411951	0.033039
hsa_circ_	0.925511	0.03304
hsa_circ_	-0.45746	0.033048
hsa_circ_	0.413159	0.03305
hsa_circ_	-0.75923	0.033066
hsa_circ_	-1.5104	0.03307
hsa_circ_	0.372461	0.033072
hsa_circ_	-0.58389	0.033075
hsa_circ_	-0.80393	0.033086
hsa_circ_	-0.82461	0.03309
hsa_circ_	0.988133	0.033091
hsa_circ_	0.510638	0.033098
hsa_circ_	0.927021	0.033108
hsa_circ_	-0.37415	0.033113
hsa_circ_	0.784406	0.033122
hsa_circ_	0.389818	0.033126
hsa_circ_	-0.33852	0.033127
hsa_circ_	-1.75261	0.03313
hsa_circ_	0.680805	0.033131
hsa_circ_	0.913825	0.033136
hsa_circ_	0.619868	0.033137
hsa_circ_	0.697152	0.033155
hsa_circ_	0.673705	0.033161
hsa_circ_	0.423344	0.033169
hsa_circ_	0.627238	0.03317
hsa_circ_	-0.58729	0.033171
hsa_circ_	1.041331	0.033178
hsa_circ_	-0.42492	0.033186
hsa_circ_	-0.62685	0.033187
hsa_circ_	-0.5867	0.033188
hsa_circ_	-0.61098	0.033191
hsa_circ_	0.507502	0.033196
hsa_circ_	0.599893	0.033198
hsa_circ_	-0.56162	0.033201
hsa_circ_	-0.60582	0.033208
hsa_circ_	-0.43888	0.033208
hsa_circ_	0.717463	0.033212
hsa_circ_	-0.43911	0.033214
hsa_circ_	0.406657	0.033219
hsa_circ_	-0.36984	0.033231
hsa_circ_	0.437551	0.033232

hsa_circ_	-0.36418	0.033235
hsa_circ_	1.026411	0.033241
hsa_circ_	0.357993	0.033256
hsa_circ_	0.457245	0.03326
hsa_circ_	-0.48044	0.033261
hsa_circ_	-0.89263	0.033269
hsa_circ_	-0.34673	0.033273
hsa_circ_	-0.36701	0.03328
hsa_circ_	0.438246	0.033287
hsa_circ_	0.549723	0.033296
hsa_circ_	-0.72375	0.033298
hsa_circ_	0.495309	0.0333
hsa_circ_	0.771756	0.033303
hsa_circ_	-0.46303	0.033304
hsa_circ_	-0.46853	0.033306
hsa_circ_	0.530721	0.033307
hsa_circ_	0.539448	0.033319
hsa_circ_	-0.52752	0.03332
hsa_circ_	-0.48105	0.033322
hsa_circ_	-0.3292	0.033322
hsa_circ_	-0.53111	0.033326
hsa_circ_	0.470503	0.033326
hsa_circ_	0.579607	0.033327
hsa_circ_	-0.45353	0.033333
hsa_circ_	-0.35143	0.033348
hsa_circ_	-0.53118	0.033362
hsa_circ_	-0.32479	0.033365
hsa_circ_	0.408113	0.033377
hsa_circ_	0.503207	0.033381
hsa_circ_	0.46074	0.033385
hsa_circ_	-0.33763	0.033397
hsa_circ_	-0.72539	0.033416
hsa_circ_	-0.43828	0.033422
hsa_circ_	-0.49032	0.033424
hsa_circ_	0.749994	0.033427
hsa_circ_	-0.38222	0.033439
hsa_circ_	0.446294	0.03344
hsa_circ_	0.449376	0.033443
hsa_circ_	-0.38456	0.033444
hsa_circ_	0.399183	0.033446
hsa_circ_	1.407646	0.033448
hsa_circ_	0.493964	0.033448
hsa_circ_	0.439381	0.033458
hsa_circ_	0.501164	0.033464
hsa_circ_	0.501762	0.033465
hsa_circ_	-0.54185	0.033473
hsa_circ_	0.651307	0.033475
hsa_circ_	1.711869	0.033481
hsa_circ_	-0.35428	0.033507
hsa_circ_	0.441216	0.033524
hsa_circ_	-0.37233	0.033527
hsa_circ_	0.826979	0.033529
hsa_circ_	0.445758	0.03353
hsa_circ_	0.56046	0.033536

hsa_circ_	-0.41892	0.03354
hsa_circ_	0.44116	0.033545
hsa_circ_	0.459102	0.033547
hsa_circ_	-0.67838	0.033549
hsa_circ_	0.672615	0.033556
hsa_circ_	-0.39784	0.033557
hsa_circ_	-0.3976	0.033587
hsa_circ_	-1.17054	0.033591
hsa_circ_	-3.38	0.033594
hsa_circ_	-0.35208	0.033597
hsa_circ_	-1.06338	0.033614
hsa_circ_	0.467636	0.033616
hsa_circ_	0.623726	0.033634
hsa_circ_	-0.32731	0.033637
hsa_circ_	0.528917	0.033641
hsa_circ_	-0.90671	0.033641
hsa_circ_	-0.39914	0.033642
hsa_circ_	0.394779	0.033643
hsa_circ_	-0.60571	0.033648
hsa_circ_	-0.6261	0.03365
hsa_circ_	-0.38	0.033662
hsa_circ_	-0.54342	0.033668
hsa_circ_	0.769603	0.033671
hsa_circ_	-0.36511	0.033684
hsa_circ_	-0.37423	0.033697
hsa_circ_	-0.88333	0.033714
hsa_circ_	-0.48731	0.033723
hsa_circ_	0.479394	0.033728
hsa_circ_	-0.54601	0.033734
hsa_circ_	-0.39516	0.033737
hsa_circ_	-0.58547	0.033746
hsa_circ_	-0.47692	0.033754
hsa_circ_	0.623345	0.03377
hsa_circ_	-0.46584	0.033776
hsa_circ_	0.515925	0.033776
hsa_circ_	0.559191	0.033789
hsa_circ_	1.176812	0.033799
hsa_circ_	0.666264	0.033818
hsa_circ_	-0.72862	0.033828
hsa_circ_	-0.71634	0.033843
hsa_circ_	0.374764	0.033843
hsa_circ_	0.547601	0.033849
hsa_circ_	0.439873	0.033852
hsa_circ_	-0.65796	0.033853
hsa_circ_	-0.4016	0.033861
hsa_circ_	-0.60223	0.033865
hsa_circ_	0.758409	0.033868
hsa_circ_	-1.20027	0.033881
hsa_circ_	-0.37089	0.033883
hsa_circ_	-0.45517	0.033883
hsa_circ_	-0.42484	0.033904
hsa_circ_	1.290271	0.033911
hsa_circ_	0.405007	0.03392
hsa_circ_	-1.24818	0.033926

hsa_circ_	-0.4794	0.033926
hsa_circ_	-0.43582	0.033932
hsa_circ_	0.367228	0.033936
hsa_circ_	0.372265	0.033938
hsa_circ_	-0.48002	0.033941
hsa_circ_	-0.35043	0.033949
hsa_circ_	-0.59288	0.033971
hsa_circ_	0.592707	0.033973
hsa_circ_	0.891661	0.033976
hsa_circ_	-0.38394	0.033981
hsa_circ_	-0.44237	0.033986
hsa_circ_	-0.41769	0.033999
hsa_circ_	-0.74336	0.033999
hsa_circ_	-1.39606	0.034003
hsa_circ_	0.383821	0.034007
hsa_circ_	-0.43446	0.034007
hsa_circ_	-0.45638	0.034012
hsa_circ_	0.569811	0.034016
hsa_circ_	-0.61931	0.034022
hsa_circ_	0.657876	0.034028
hsa_circ_	-0.50436	0.034036
hsa_circ_	-0.37739	0.034044
hsa_circ_	-0.59516	0.034048
hsa_circ_	1.5474	0.034049
hsa_circ_	-0.4313	0.034061
hsa_circ_	-0.59527	0.034076
hsa_circ_	0.416675	0.034077
hsa_circ_	1.386685	0.034087
hsa_circ_	-0.37517	0.034093
hsa_circ_	0.663757	0.034107
hsa_circ_	-0.51378	0.034108
hsa_circ_	-0.37614	0.034114
hsa_circ_	-0.41588	0.034115
hsa_circ_	-0.35637	0.034123
hsa_circ_	0.731261	0.034123
hsa_circ_	0.434951	0.034128
hsa_circ_	-0.63789	0.034129
hsa_circ_	-1.48829	0.034135
hsa_circ_	0.747021	0.034148
hsa_circ_	1.729587	0.034152
hsa_circ_	0.497546	0.034158
hsa_circ_	-0.35039	0.034172
hsa_circ_	0.908611	0.034172
hsa_circ_	-0.4711	0.034174
hsa_circ_	-0.64183	0.034174
hsa_circ_	0.466843	0.034186
hsa_circ_	0.526559	0.034193
hsa_circ_	0.636813	0.034201
hsa_circ_	0.799377	0.034201
hsa_circ_	-0.3517	0.034203
hsa_circ_	0.461835	0.034203
hsa_circ_	0.550733	0.034209
hsa_circ_	-0.39745	0.034212
hsa_circ_	0.427183	0.03422

hsa_circ_	-0.35494	0.034222
hsa_circ_	1.017394	0.034226
hsa_circ_	1.701971	0.034235
hsa_circ_	0.777043	0.034247
hsa_circ_	0.436791	0.03425
hsa_circ_	0.385815	0.034272
hsa_circ_	0.818318	0.034278
hsa_circ_	1.0667	0.034279
hsa_circ_	-1.41694	0.034285
hsa_circ_	-0.34062	0.034285
hsa_circ_	1.108278	0.034286
hsa_circ_	-0.98575	0.034288
hsa_circ_	-0.56671	0.034288
hsa_circ_	0.346637	0.03429
hsa_circ_	-1.03719	0.034301
hsa_circ_	0.379233	0.034311
hsa_circ_	-0.65293	0.034313
hsa_circ_	0.554034	0.034317
hsa_circ_	0.793102	0.034318
hsa_circ_	0.475163	0.034322
hsa_circ_	0.372917	0.034323
hsa_circ_	0.592706	0.034325
hsa_circ_	1.039065	0.034326
hsa_circ_	1.156199	0.03433
hsa_circ_	1.446566	0.03434
hsa_circ_	1.422842	0.03435
hsa_circ_	-0.47157	0.034351
hsa_circ_	-0.47067	0.034369
hsa_circ_	-0.81123	0.034372
hsa_circ_	-0.45572	0.034376
hsa_circ_	1.231412	0.034377
hsa_circ_	-0.60993	0.034398
hsa_circ_	-0.4843	0.034402
hsa_circ_	0.456607	0.034403
hsa_circ_	0.643003	0.034412
hsa_circ_	0.826248	0.034415
hsa_circ_	0.588905	0.034419
hsa_circ_	0.37879	0.034426
hsa_circ_	0.625029	0.03443
hsa_circ_	-0.45051	0.034432
hsa_circ_	0.680855	0.034438
hsa_circ_	-0.62144	0.03444
hsa_circ_	-0.7915	0.034441
hsa_circ_	0.375621	0.034445
hsa_circ_	0.653671	0.034449
hsa_circ_	0.426518	0.034458
hsa_circ_	-0.59986	0.034461
hsa_circ_	-0.40134	0.034463
hsa_circ_	0.509047	0.034464
hsa_circ_	0.368608	0.034473
hsa_circ_	-0.36503	0.034478
hsa_circ_	-0.73629	0.034482
hsa_circ_	-0.47333	0.034486
hsa_circ_	0.543694	0.034489

hsa_circ_	0.610319	0.034493
hsa_circ_	-0.43572	0.034496
hsa_circ_	0.591804	0.034502
hsa_circ_	-0.51253	0.034506
hsa_circ_	1.009523	0.034507
hsa_circ_	-0.55927	0.034509
hsa_circ_	0.796374	0.034513
hsa_circ_	0.619201	0.034513
hsa_circ_	0.721903	0.034517
hsa_circ_	-0.56687	0.034523
hsa_circ_	0.370256	0.034536
hsa_circ_	0.475918	0.034537
hsa_circ_	-0.53463	0.034547
hsa_circ_	-0.44248	0.03455
hsa_circ_	-0.39039	0.034556
hsa_circ_	0.597387	0.034558
hsa_circ_	-0.86729	0.034572
hsa_circ_	-0.3247	0.034577
hsa_circ_	0.342478	0.034585
hsa_circ_	0.368184	0.034589
hsa_circ_	-0.59996	0.034597
hsa_circ_	-0.87951	0.034602
hsa_circ_	0.76911	0.034612
hsa_circ_	0.455662	0.034616
hsa_circ_	-0.33794	0.034619
hsa_circ_	-0.40075	0.034622
hsa_circ_	0.517277	0.034625
hsa_circ_	-0.3254	0.034643
hsa_circ_	-0.69913	0.034646
hsa_circ_	0.432573	0.034649
hsa_circ_	0.70972	0.034658
hsa_circ_	1.383965	0.034667
hsa_circ_	-0.47416	0.03467
hsa_circ_	-0.52376	0.034675
hsa_circ_	0.518523	0.034683
hsa_circ_	-0.70594	0.034684
hsa_circ_	-0.33316	0.034686
hsa_circ_	-1.16138	0.034687
hsa_circ_	-0.34369	0.034688
hsa_circ_	-1.2797	0.034691
hsa_circ_	-0.34524	0.034692
hsa_circ_	0.908673	0.034708
hsa_circ_	-0.41941	0.034713
hsa_circ_	-0.42066	0.034722
hsa_circ_	0.43998	0.03473
hsa_circ_	-0.95901	0.034736
hsa_circ_	1.378644	0.034739
hsa_circ_	-0.63417	0.03474
hsa_circ_	-0.43489	0.034752
hsa_circ_	0.436072	0.03476
hsa_circ_	0.368687	0.034768
hsa_circ_	0.572094	0.03477
hsa_circ_	0.347311	0.034772
hsa_circ_	0.584374	0.034786

hsa_circ_	0.750753	0.034789
hsa_circ_	-0.73827	0.034791
hsa_circ_	-0.82883	0.034794
hsa_circ_	0.439392	0.034797
hsa_circ_	0.568915	0.034802
hsa_circ_	0.541952	0.034816
hsa_circ_	0.611957	0.034819
hsa_circ_	-0.51781	0.034824
hsa_circ_	0.413907	0.034833
hsa_circ_	1.024854	0.034845
hsa_circ_	-0.39032	0.034852
hsa_circ_	-0.64423	0.034852
hsa_circ_	0.62949	0.034853
hsa_circ_	0.634782	0.034862
hsa_circ_	-0.99474	0.034877
hsa_circ_	0.496705	0.034888
hsa_circ_	0.324653	0.034891
hsa_circ_	-0.61928	0.034896
hsa_circ_	0.600081	0.034896
hsa_circ_	-0.87147	0.034896
hsa_circ_	-0.3665	0.034901
hsa_circ_	-0.37804	0.034906
hsa_circ_	-0.33761	0.034911
hsa_circ_	1.237015	0.034915
hsa_circ_	-0.42811	0.034923
hsa_circ_	0.447505	0.034924
hsa_circ_	-0.49769	0.034951
hsa_circ_	-0.40568	0.034957
hsa_circ_	-0.44018	0.034967
hsa_circ_	1.591461	0.034968
hsa_circ_	0.518194	0.034971
hsa_circ_	0.685587	0.034983
hsa_circ_	0.570042	0.034983
hsa_circ_	-0.51889	0.03499
hsa_circ_	-0.77908	0.034993
hsa_circ_	-0.45794	0.034994
hsa_circ_	0.586576	0.034995
hsa_circ_	0.409594	0.035003
hsa_circ_	0.381241	0.035007
hsa_circ_	-0.43162	0.035009
hsa_circ_	-1.18777	0.03502
hsa_circ_	-0.94084	0.035031
hsa_circ_	-1.97114	0.035058
hsa_circ_	-0.41301	0.035065
hsa_circ_	-0.49769	0.03507
hsa_circ_	-0.48045	0.035073
hsa_circ_	-0.40805	0.035075
hsa_circ_	-0.51046	0.035087
hsa_circ_	-0.37952	0.035089
hsa_circ_	-0.60128	0.035089
hsa_circ_	0.623734	0.035095
hsa_circ_	0.48811	0.035102
hsa_circ_	0.529575	0.035104
hsa_circ_	-0.45017	0.035107

hsa_circ_	0.599806	0.035111
hsa_circ_	-0.40973	0.035118
hsa_circ_	0.908767	0.035119
hsa_circ_	0.322196	0.03513
hsa_circ_	-0.48127	0.035131
hsa_circ_	0.477381	0.035145
hsa_circ_	0.35562	0.035162
hsa_circ_	1.123397	0.03517
hsa_circ_	-0.45697	0.03517
hsa_circ_	0.519054	0.035177
hsa_circ_	-0.53475	0.035184
hsa_circ_	0.389137	0.035192
hsa_circ_	-0.4233	0.035198
hsa_circ_	-0.58241	0.035205
hsa_circ_	-0.96264	0.035206
hsa_circ_	-0.80874	0.035206
hsa_circ_	0.497145	0.035208
hsa_circ_	-0.601	0.035216
hsa_circ_	-0.58098	0.03522
hsa_circ_	0.344038	0.035224
hsa_circ_	-0.33007	0.035239
hsa_circ_	0.771555	0.035241
hsa_circ_	-0.32647	0.035254
hsa_circ_	-0.96481	0.035259
hsa_circ_	0.722642	0.035269
hsa_circ_	0.57954	0.035277
hsa_circ_	0.592353	0.035307
hsa_circ_	0.491165	0.03531
hsa_circ_	0.411601	0.035315
hsa_circ_	0.757748	0.03533
hsa_circ_	-0.96743	0.035331
hsa_circ_	-0.47261	0.035335
hsa_circ_	-0.40107	0.035336
hsa_circ_	-0.49173	0.035352
hsa_circ_	0.392808	0.035368
hsa_circ_	0.641603	0.035374
hsa_circ_	-0.33223	0.035385
hsa_circ_	-0.90208	0.035385
hsa_circ_	0.523676	0.035391
hsa_circ_	-0.66179	0.035393
hsa_circ_	-0.38486	0.0354
hsa_circ_	-0.50184	0.0354
hsa_circ_	-0.35443	0.035405
hsa_circ_	0.527699	0.035406
hsa_circ_	0.59202	0.035409
hsa_circ_	-0.34554	0.035416
hsa_circ_	0.375894	0.035421
hsa_circ_	-0.64278	0.035423
hsa_circ_	0.858424	0.035426
hsa_circ_	0.469498	0.035427
hsa_circ_	0.67335	0.035437
hsa_circ_	-0.91298	0.03544
hsa_circ_	-0.84721	0.035446
hsa_circ_	0.45684	0.035448

hsa_circ_	0.356189	0.03545
hsa_circ_	0.391043	0.035451
hsa_circ_	-1.44194	0.035457
hsa_circ_	1.425067	0.035458
hsa_circ_	0.784184	0.035462
hsa_circ_	0.595557	0.035497
hsa_circ_	1.030816	0.035498
hsa_circ_	0.536223	0.035513
hsa_circ_	-0.90774	0.035518
hsa_circ_	-1.18727	0.035521
hsa_circ_	0.881046	0.035526
hsa_circ_	0.449852	0.035532
hsa_circ_	-0.43058	0.035539
hsa_circ_	-0.41721	0.03554
hsa_circ_	-0.44372	0.035549
hsa_circ_	0.331086	0.035561
hsa_circ_	-0.45117	0.035563
hsa_circ_	0.408141	0.035565
hsa_circ_	-0.50262	0.035568
hsa_circ_	-0.57132	0.03557
hsa_circ_	-0.39142	0.035572
hsa_circ_	0.846303	0.035572
hsa_circ_	-0.3572	0.035579
hsa_circ_	0.51705	0.035588
hsa_circ_	0.428494	0.035592
hsa_circ_	0.367784	0.035594
hsa_circ_	-0.46116	0.035644
hsa_circ_	0.724589	0.035657
hsa_circ_	-0.35173	0.035665
hsa_circ_	0.456855	0.035669
hsa_circ_	-0.34139	0.035674
hsa_circ_	-0.5694	0.035685
hsa_circ_	0.468905	0.035686
hsa_circ_	0.489399	0.035687
hsa_circ_	1.078309	0.035689
hsa_circ_	-0.53018	0.03569
hsa_circ_	-0.57679	0.0357
hsa_circ_	0.479153	0.035701
hsa_circ_	-0.36182	0.035706
hsa_circ_	-0.47756	0.035709
hsa_circ_	0.766141	0.03571
hsa_circ_	-0.48594	0.035713
hsa_circ_	0.429337	0.035722
hsa_circ_	-0.52725	0.035723
hsa_circ_	0.403469	0.035724
hsa_circ_	-0.39065	0.035732
hsa_circ_	-0.45103	0.035736
hsa_circ_	0.451857	0.035746
hsa_circ_	0.679215	0.035748
hsa_circ_	-0.44073	0.035759
hsa_circ_	0.498077	0.035772
hsa_circ_	-0.59867	0.035776
hsa_circ_	-0.38248	0.035777
hsa_circ_	-0.44269	0.035781

hsa_circ_	-1.20262	0.035786
hsa_circ_	0.729793	0.035787
hsa_circ_	0.371646	0.035789
hsa_circ_	-0.80994	0.035794
hsa_circ_	-0.46148	0.035797
hsa_circ_	-0.9614	0.035798
hsa_circ_	0.438159	0.035806
hsa_circ_	-1.32313	0.035806
hsa_circ_	0.940753	0.035813
hsa_circ_	-0.59326	0.035813
hsa_circ_	0.656966	0.035815
hsa_circ_	-0.4606	0.035817
hsa_circ_	-0.76655	0.035818
hsa_circ_	1.232105	0.035819
hsa_circ_	-0.48248	0.035828
hsa_circ_	-0.69343	0.03583
hsa_circ_	-0.37568	0.035833
hsa_circ_	-0.66514	0.035845
hsa_circ_	-0.70762	0.035848
hsa_circ_	0.385402	0.035851
hsa_circ_	-0.49663	0.03586
hsa_circ_	0.501925	0.035869
hsa_circ_	0.44773	0.035876
hsa_circ_	0.423369	0.035884
hsa_circ_	-0.74157	0.035885
hsa_circ_	-1.43208	0.035889
hsa_circ_	0.556527	0.03589
hsa_circ_	-0.35999	0.035896
hsa_circ_	-0.45611	0.035897
hsa_circ_	-0.62314	0.035899
hsa_circ_	0.794664	0.035914
hsa_circ_	0.502799	0.035918
hsa_circ_	0.423597	0.03592
hsa_circ_	0.565861	0.035929
hsa_circ_	0.588315	0.03594
hsa_circ_	1.876607	0.035943
hsa_circ_	0.515024	0.03595
hsa_circ_	-0.517	0.035955
hsa_circ_	-0.34971	0.035958
hsa_circ_	0.597061	0.035961
hsa_circ_	-0.43101	0.035966
hsa_circ_	-1.06974	0.035968
hsa_circ_	-0.5102	0.035976
hsa_circ_	-0.58746	0.035982
hsa_circ_	-0.64578	0.036001
hsa_circ_	0.464401	0.036015
hsa_circ_	0.534872	0.036022
hsa_circ_	-0.38092	0.036022
hsa_circ_	-0.386	0.036038
hsa_circ_	-0.57123	0.03604
hsa_circ_	-0.40172	0.036047
hsa_circ_	-0.88473	0.036062
hsa_circ_	0.560646	0.036069
hsa_circ_	-0.67017	0.036083

hsa_circ_	0.796471	0.036086
hsa_circ_	-0.64478	0.036086
hsa_circ_	0.387777	0.0361
hsa_circ_	0.617535	0.036117
hsa_circ_	-0.36298	0.036131
hsa_circ_	0.743285	0.036135
hsa_circ_	0.654608	0.036135
hsa_circ_	-0.39031	0.036136
hsa_circ_	-0.42506	0.036139
hsa_circ_	-0.66403	0.036144
hsa_circ_	-0.48785	0.036149
hsa_circ_	-0.70723	0.036173
hsa_circ_	0.53563	0.036175
hsa_circ_	-0.49762	0.036185
hsa_circ_	0.403777	0.036185
hsa_circ_	0.421236	0.036185
hsa_circ_	-0.56624	0.036199
hsa_circ_	-0.33423	0.036199
hsa_circ_	-0.38143	0.036206
hsa_circ_	0.543298	0.036211
hsa_circ_	0.594114	0.036224
hsa_circ_	0.760185	0.036232
hsa_circ_	-0.32439	0.036239
hsa_circ_	0.853525	0.036241
hsa_circ_	0.404242	0.036244
hsa_circ_	0.99675	0.036248
hsa_circ_	-0.42003	0.036254
hsa_circ_	-0.61411	0.036261
hsa_circ_	0.643142	0.036266
hsa_circ_	-0.47808	0.036276
hsa_circ_	-0.55165	0.036276
hsa_circ_	0.922773	0.03628
hsa_circ_	0.485859	0.036285
hsa_circ_	0.511135	0.036294
hsa_circ_	0.749911	0.036294
hsa_circ_	-0.70208	0.036295
hsa_circ_	0.958611	0.03631
hsa_circ_	0.387084	0.036317
hsa_circ_	-0.66687	0.036322
hsa_circ_	0.39905	0.036327
hsa_circ_	-0.70569	0.036344
hsa_circ_	0.363553	0.036375
hsa_circ_	1.062956	0.036377
hsa_circ_	0.548123	0.036388
hsa_circ_	1.473334	0.03639
hsa_circ_	-0.48201	0.036398
hsa_circ_	-0.69714	0.036404
hsa_circ_	-0.49071	0.036405
hsa_circ_	0.677098	0.036415
hsa_circ_	0.85168	0.036416
hsa_circ_	1.256023	0.036417
hsa_circ_	0.793165	0.036417
hsa_circ_	0.394896	0.036431
hsa_circ_	-1.48808	0.036438

hsa_circ_	-0.44304	0.036442
hsa_circ_	-0.42644	0.036444
hsa_circ_	0.572982	0.036444
hsa_circ_	-2.01815	0.036447
hsa_circ_	-0.43957	0.036448
hsa_circ_	-0.38175	0.03645
hsa_circ_	0.783757	0.036452
hsa_circ_	-0.5652	0.036455
hsa_circ_	-0.5771	0.036459
hsa_circ_	-0.59727	0.036461
hsa_circ_	-0.56096	0.036464
hsa_circ_	-0.35031	0.036465
hsa_circ_	0.914735	0.036468
hsa_circ_	0.470863	0.03647
hsa_circ_	0.39095	0.036472
hsa_circ_	-0.47819	0.036472
hsa_circ_	-1.55877	0.036486
hsa_circ_	-0.45237	0.036488
hsa_circ_	1.29845	0.036495
hsa_circ_	-0.52162	0.036505
hsa_circ_	-0.34936	0.036509
hsa_circ_	-0.42727	0.03651
hsa_circ_	0.428397	0.036512
hsa_circ_	0.617667	0.036516
hsa_circ_	0.433348	0.036522
hsa_circ_	-1.12636	0.036526
hsa_circ_	0.456751	0.036553
hsa_circ_	-0.4949	0.03656
hsa_circ_	-0.60831	0.036569
hsa_circ_	-0.53785	0.036576
hsa_circ_	0.454793	0.036579
hsa_circ_	0.946876	0.03658
hsa_circ_	-0.52032	0.036582
hsa_circ_	-0.40923	0.036582
hsa_circ_	-0.56301	0.036585
hsa_circ_	0.849487	0.036588
hsa_circ_	-0.86274	0.036589
hsa_circ_	0.914284	0.036599
hsa_circ_	0.435237	0.036629
hsa_circ_	-0.71474	0.03663
hsa_circ_	-0.35964	0.036631
hsa_circ_	0.532511	0.036639
hsa_circ_	0.875231	0.036652
hsa_circ_	-3.45088	0.036653
hsa_circ_	0.485776	0.036654
hsa_circ_	-0.44091	0.036659
hsa_circ_	-0.71456	0.03666
hsa_circ_	0.509431	0.036664
hsa_circ_	0.575171	0.036666
hsa_circ_	-0.60898	0.036667
hsa_circ_	0.477952	0.036669
hsa_circ_	0.325069	0.03667
hsa_circ_	-0.58843	0.03668
hsa_circ_	-0.58827	0.036687

hsa_circ_	0.550753	0.036702
hsa_circ_	-0.33403	0.036702
hsa_circ_	-0.40526	0.036705
hsa_circ_	0.391572	0.036706
hsa_circ_	-0.57647	0.036713
hsa_circ_	0.3574	0.036721
hsa_circ_	-0.38231	0.036723
hsa_circ_	-0.67987	0.036731
hsa_circ_	-0.50325	0.036732
hsa_circ_	0.410107	0.036734
hsa_circ_	-0.60518	0.036753
hsa_circ_	-0.64168	0.036756
hsa_circ_	1.255559	0.036757
hsa_circ_	-0.37278	0.036759
hsa_circ_	0.594997	0.036761
hsa_circ_	-0.81001	0.036765
hsa_circ_	0.498769	0.036768
hsa_circ_	-0.73861	0.036773
hsa_circ_	-0.38474	0.036775
hsa_circ_	-0.49275	0.036778
hsa_circ_	0.879238	0.03678
hsa_circ_	-0.33659	0.036784
hsa_circ_	0.347013	0.036785
hsa_circ_	-0.54803	0.036785
hsa_circ_	0.736572	0.036792
hsa_circ_	-0.59741	0.036798
hsa_circ_	0.487128	0.036799
hsa_circ_	-0.69199	0.0368
hsa_circ_	-0.55919	0.036801
hsa_circ_	0.478725	0.036807
hsa_circ_	1.063853	0.036817
hsa_circ_	-0.66207	0.036822
hsa_circ_	-0.36461	0.036824
hsa_circ_	-0.72659	0.036827
hsa_circ_	-0.38098	0.036835
hsa_circ_	0.329512	0.036845
hsa_circ_	0.418431	0.036854
hsa_circ_	0.354841	0.036854
hsa_circ_	-0.57528	0.036858
hsa_circ_	-0.43133	0.036865
hsa_circ_	-0.53111	0.036869
hsa_circ_	-0.87641	0.036872
hsa_circ_	0.576655	0.036885
hsa_circ_	-0.84433	0.036905
hsa_circ_	-0.72669	0.036908
hsa_circ_	1.066155	0.036926
hsa_circ_	0.383359	0.036932
hsa_circ_	-0.3873	0.036937
hsa_circ_	0.90616	0.036943
hsa_circ_	-0.56689	0.036958
hsa_circ_	-0.99609	0.036964
hsa_circ_	0.808347	0.036966
hsa_circ_	-0.40021	0.036968
hsa_circ_	0.923127	0.036974

hsa_circ_	-0.41056	0.03698
hsa_circ_	-0.39542	0.036994
hsa_circ_	-0.41955	0.037
hsa_circ_	0.347571	0.037009
hsa_circ_	0.418293	0.037009
hsa_circ_	-0.41654	0.037014
hsa_circ_	0.406526	0.037015
hsa_circ_	-0.56311	0.03702
hsa_circ_	0.415388	0.037021
hsa_circ_	-0.39197	0.037022
hsa_circ_	-0.83435	0.037025
hsa_circ_	0.365528	0.037031
hsa_circ_	0.493988	0.037033
hsa_circ_	0.553466	0.037033
hsa_circ_	-0.69713	0.037051
hsa_circ_	-0.45267	0.037054
hsa_circ_	0.575061	0.037061
hsa_circ_	0.333158	0.037066
hsa_circ_	-0.35404	0.03707
hsa_circ_	-0.46954	0.03707
hsa_circ_	-0.57378	0.03707
hsa_circ_	0.51697	0.037078
hsa_circ_	-0.39277	0.03708
hsa_circ_	-0.44423	0.037084
hsa_circ_	0.47175	0.037088
hsa_circ_	0.502167	0.037091
hsa_circ_	0.777117	0.03712
hsa_circ_	0.380275	0.037125
hsa_circ_	-0.43172	0.037134
hsa_circ_	-0.69908	0.03714
hsa_circ_	-0.63075	0.037142
hsa_circ_	0.671463	0.037146
hsa_circ_	-0.84786	0.037147
hsa_circ_	0.420758	0.037152
hsa_circ_	-0.5891	0.037153
hsa_circ_	-0.45755	0.037153
hsa_circ_	0.340225	0.037162
hsa_circ_	0.51579	0.037166
hsa_circ_	-0.66183	0.037167
hsa_circ_	0.468892	0.037174
hsa_circ_	-0.4193	0.03719
hsa_circ_	-0.52383	0.03719
hsa_circ_	0.365331	0.037194
hsa_circ_	-0.50426	0.037196
hsa_circ_	-0.48833	0.037209
hsa_circ_	-0.63131	0.037209
hsa_circ_	-0.34701	0.037214
hsa_circ_	-0.81629	0.037215
hsa_circ_	0.907978	0.03722
hsa_circ_	-0.40936	0.037232
hsa_circ_	0.44131	0.037242
hsa_circ_	-0.31769	0.037245
hsa_circ_	-0.36456	0.037253
hsa_circ_	1.390776	0.037254

hsa_circ_	-0.36827	0.037258
hsa_circ_	-0.35723	0.037262
hsa_circ_	-0.36126	0.037278
hsa_circ_	-0.64782	0.037281
hsa_circ_	0.438308	0.037281
hsa_circ_	0.824625	0.037286
hsa_circ_	-0.90845	0.03729
hsa_circ_	-0.59132	0.037304
hsa_circ_	-0.61521	0.037319
hsa_circ_	0.389528	0.037321
hsa_circ_	0.900341	0.037332
hsa_circ_	0.513669	0.037359
hsa_circ_	-1.79553	0.037364
hsa_circ_	0.504183	0.037366
hsa_circ_	-0.83644	0.037367
hsa_circ_	-0.48949	0.037373
hsa_circ_	-0.40618	0.03738
hsa_circ_	-0.62474	0.037386
hsa_circ_	-0.36842	0.037394
hsa_circ_	-0.48605	0.037396
hsa_circ_	0.482648	0.037401
hsa_circ_	-0.8481	0.037405
hsa_circ_	-0.78185	0.037417
hsa_circ_	0.380021	0.037419
hsa_circ_	-0.89757	0.03742
hsa_circ_	-0.49981	0.037435
hsa_circ_	-0.87815	0.037438
hsa_circ_	0.476468	0.037446
hsa_circ_	-0.55803	0.037448
hsa_circ_	-0.62787	0.037468
hsa_circ_	-0.56155	0.037474
hsa_circ_	-0.60907	0.037481
hsa_circ_	-0.36516	0.037481
hsa_circ_	0.588969	0.037484
hsa_circ_	0.75398	0.037488
hsa_circ_	0.428797	0.037491
hsa_circ_	1.396239	0.037494
hsa_circ_	0.735237	0.037502
hsa_circ_	0.398021	0.037503
hsa_circ_	0.513099	0.037507
hsa_circ_	0.41643	0.037511
hsa_circ_	-0.3897	0.037513
hsa_circ_	0.531071	0.037514
hsa_circ_	-1.3251	0.037515
hsa_circ_	-0.41947	0.037516
hsa_circ_	0.893001	0.037524
hsa_circ_	0.365728	0.037539
hsa_circ_	-0.34716	0.037556
hsa_circ_	-0.37668	0.037565
hsa_circ_	-0.59581	0.037568
hsa_circ_	-0.37088	0.037591
hsa_circ_	0.531425	0.037596
hsa_circ_	-0.851	0.0376
hsa_circ_	0.395706	0.037602

hsa_circ_	0.663578	0.037604
hsa_circ_	-0.50407	0.037605
hsa_circ_	0.335474	0.037605
hsa_circ_	-0.56104	0.037608
hsa_circ_	-1.06012	0.037614
hsa_circ_	0.477615	0.037617
hsa_circ_	-0.62763	0.037624
hsa_circ_	0.503341	0.037624
hsa_circ_	-0.83955	0.037628
hsa_circ_	1.043882	0.037629
hsa_circ_	0.521911	0.037633
hsa_circ_	-0.56117	0.037633
hsa_circ_	0.552921	0.037638
hsa_circ_	-0.62732	0.03764
hsa_circ_	0.316296	0.037641
hsa_circ_	-0.43892	0.037651
hsa_circ_	-1.78601	0.037662
hsa_circ_	-0.92253	0.037667
hsa_circ_	-1.68884	0.037672
hsa_circ_	0.804847	0.037675
hsa_circ_	-0.60464	0.037684
hsa_circ_	-0.43744	0.037688
hsa_circ_	-0.70613	0.037689
hsa_circ_	-0.53405	0.037697
hsa_circ_	-0.5811	0.037699
hsa_circ_	0.3581	0.037702
hsa_circ_	-0.68784	0.037703
hsa_circ_	0.473405	0.037705
hsa_circ_	0.373492	0.037707
hsa_circ_	0.451361	0.037708
hsa_circ_	0.373628	0.037711
hsa_circ_	0.480154	0.037714
hsa_circ_	-0.9415	0.037721
hsa_circ_	-1.13319	0.037723
hsa_circ_	0.635975	0.037726
hsa_circ_	-0.46842	0.03773
hsa_circ_	-0.4397	0.037751
hsa_circ_	-0.35394	0.037759
hsa_circ_	0.36941	0.037761
hsa_circ_	-0.32777	0.037761
hsa_circ_	-0.76696	0.037764
hsa_circ_	0.498494	0.037765
hsa_circ_	0.407372	0.037771
hsa_circ_	-0.40653	0.037772
hsa_circ_	0.317403	0.037786
hsa_circ_	-1.06268	0.037791
hsa_circ_	-0.41084	0.037793
hsa_circ_	0.537584	0.037797
hsa_circ_	0.86163	0.037806
hsa_circ_	0.447912	0.037806
hsa_circ_	-0.59393	0.037807
hsa_circ_	0.520624	0.037821
hsa_circ_	0.456111	0.037826
hsa_circ_	-1.21265	0.037828

hsa_circ_	-0.51491	0.037833
hsa_circ_	0.447774	0.037836
hsa_circ_	0.435421	0.03784
hsa_circ_	-1.20637	0.037853
hsa_circ_	-0.45931	0.037853
hsa_circ_	-0.40865	0.037854
hsa_circ_	-0.35463	0.037856
hsa_circ_	-0.32978	0.037869
hsa_circ_	-0.96657	0.037869
hsa_circ_	0.490478	0.037875
hsa_circ_	-0.58611	0.037876
hsa_circ_	-0.37021	0.037883
hsa_circ_	-0.43839	0.037885
hsa_circ_	0.668827	0.037908
hsa_circ_	0.846216	0.037911
hsa_circ_	0.421393	0.037913
hsa_circ_	-0.41171	0.037914
hsa_circ_	-0.61353	0.037921
hsa_circ_	-0.41148	0.037925
hsa_circ_	-0.83156	0.037954
hsa_circ_	-0.49567	0.037959
hsa_circ_	-0.43663	0.037965
hsa_circ_	0.436329	0.037967
hsa_circ_	0.88547	0.03797
hsa_circ_	0.648644	0.037977
hsa_circ_	-0.57494	0.037982
hsa_circ_	0.440593	0.037982
hsa_circ_	0.455762	0.037994
hsa_circ_	-1.09032	0.037995
hsa_circ_	0.411017	0.038003
hsa_circ_	-0.52079	0.03801
hsa_circ_	0.874853	0.038015
hsa_circ_	0.907209	0.038015
hsa_circ_	0.433678	0.038016
hsa_circ_	-0.3541	0.038018
hsa_circ_	0.406652	0.038025
hsa_circ_	-0.36459	0.038026
hsa_circ_	0.551227	0.038034
hsa_circ_	-0.65308	0.038035
hsa_circ_	0.370953	0.038038
hsa_circ_	0.524833	0.038039
hsa_circ_	1.178922	0.03804
hsa_circ_	0.48157	0.038043
hsa_circ_	-1.1309	0.038051
hsa_circ_	-1.47524	0.038058
hsa_circ_	0.367895	0.038076
hsa_circ_	0.405377	0.038076
hsa_circ_	0.546395	0.0381
hsa_circ_	1.240742	0.038101
hsa_circ_	0.632285	0.038103
hsa_circ_	-0.84841	0.038106
hsa_circ_	-0.33865	0.038106
hsa_circ_	-0.78654	0.038107
hsa_circ_	-1.0987	0.038114

hsa_circ_	-0.45506	0.038136
hsa_circ_	-0.46978	0.038151
hsa_circ_	0.740367	0.038155
hsa_circ_	1.155559	0.038156
hsa_circ_	0.48749	0.038158
hsa_circ_	0.682556	0.038161
hsa_circ_	-0.57276	0.038162
hsa_circ_	0.497468	0.038167
hsa_circ_	1.295594	0.038168
hsa_circ_	0.423978	0.038169
hsa_circ_	-0.46934	0.038174
hsa_circ_	-0.59089	0.038186
hsa_circ_	-0.46133	0.038193
hsa_circ_	-0.36087	0.038197
hsa_circ_	-0.58824	0.038198
hsa_circ_	-0.41313	0.038201
hsa_circ_	0.568804	0.038202
hsa_circ_	-0.88948	0.038211
hsa_circ_	-0.34376	0.038213
hsa_circ_	1.293167	0.038217
hsa_circ_	-0.32685	0.038224
hsa_circ_	0.592069	0.038228
hsa_circ_	-0.83643	0.038231
hsa_circ_	0.553912	0.038232
hsa_circ_	0.798522	0.038237
hsa_circ_	1.054441	0.038245
hsa_circ_	-0.42314	0.038262
hsa_circ_	-0.44847	0.038264
hsa_circ_	0.386996	0.03828
hsa_circ_	0.512536	0.038287
hsa_circ_	0.490572	0.038309
hsa_circ_	-0.63777	0.038312
hsa_circ_	-0.57089	0.038313
hsa_circ_	0.544993	0.038319
hsa_circ_	-0.47609	0.038322
hsa_circ_	0.63025	0.038324
hsa_circ_	0.419262	0.038324
hsa_circ_	-0.37197	0.038327
hsa_circ_	0.524795	0.038335
hsa_circ_	0.733134	0.038341
hsa_circ_	1.105172	0.038343
hsa_circ_	1.456805	0.038348
hsa_circ_	-0.34797	0.038349
hsa_circ_	0.640655	0.038351
hsa_circ_	-0.50449	0.038366
hsa_circ_	-0.68428	0.038383
hsa_circ_	0.602652	0.038384
hsa_circ_	-0.54648	0.038401
hsa_circ_	-0.54828	0.038402
hsa_circ_	0.460709	0.038406
hsa_circ_	-0.34831	0.038408
hsa_circ_	0.807671	0.038414
hsa_circ_	0.58366	0.038414
hsa_circ_	0.366908	0.038423

hsa_circ_	0.6721	0.038425
hsa_circ_	-0.37929	0.038428
hsa_circ_	0.40116	0.038437
hsa_circ_	0.341159	0.038445
hsa_circ_	0.552069	0.038446
hsa_circ_	0.390475	0.038449
hsa_circ_	-0.35292	0.03845
hsa_circ_	-0.38539	0.03846
hsa_circ_	0.937773	0.038469
hsa_circ_	0.619905	0.03847
hsa_circ_	-0.39331	0.038472
hsa_circ_	0.442115	0.038489
hsa_circ_	0.405128	0.038495
hsa_circ_	-0.48079	0.038495
hsa_circ_	-0.43189	0.038513
hsa_circ_	-1.0332	0.038516
hsa_circ_	0.351648	0.038519
hsa_circ_	0.694961	0.038529
hsa_circ_	0.826926	0.038531
hsa_circ_	-0.69777	0.038533
hsa_circ_	-0.49656	0.038573
hsa_circ_	0.54354	0.038575
hsa_circ_	0.409134	0.038576
hsa_circ_	0.417905	0.038583
hsa_circ_	-0.35012	0.038584
hsa_circ_	0.370684	0.038591
hsa_circ_	-0.3991	0.038607
hsa_circ_	0.685858	0.038609
hsa_circ_	-0.4675	0.03861
hsa_circ_	-0.36752	0.03861
hsa_circ_	-0.3237	0.038612
hsa_circ_	-0.94789	0.038615
hsa_circ_	0.901232	0.038659
hsa_circ_	0.416888	0.03866
hsa_circ_	-0.36358	0.03866
hsa_circ_	-0.34837	0.038662
hsa_circ_	0.503052	0.038662
hsa_circ_	-0.42398	0.038668
hsa_circ_	-0.572	0.038676
hsa_circ_	-0.43383	0.038684
hsa_circ_	0.479485	0.038688
hsa_circ_	-0.379	0.038691
hsa_circ_	-0.41798	0.038699
hsa_circ_	-0.5387	0.038702
hsa_circ_	0.414768	0.038714
hsa_circ_	0.493187	0.038728
hsa_circ_	0.623321	0.038736
hsa_circ_	-0.46783	0.038741
hsa_circ_	-0.42357	0.038741
hsa_circ_	-0.63986	0.038762
hsa_circ_	-0.71399	0.038768
hsa_circ_	-0.31072	0.038768
hsa_circ_	0.873771	0.038775
hsa_circ_	-1.01618	0.038779

hsa_circ_	-0.45102	0.038791
hsa_circ_	-0.45867	0.038791
hsa_circ_	0.513358	0.038797
hsa_circ_	-0.69193	0.038798
hsa_circ_	-0.6176	0.038807
hsa_circ_	0.800248	0.038808
hsa_circ_	-0.44736	0.038813
hsa_circ_	0.468834	0.038817
hsa_circ_	-0.35193	0.038817
hsa_circ_	0.912649	0.038824
hsa_circ_	-0.46649	0.038828
hsa_circ_	0.929731	0.038851
hsa_circ_	-0.41037	0.038862
hsa_circ_	-0.44211	0.038864
hsa_circ_	-0.48189	0.038872
hsa_circ_	0.850459	0.038875
hsa_circ_	0.399673	0.038887
hsa_circ_	-0.66493	0.038889
hsa_circ_	0.504632	0.038891
hsa_circ_	-0.58784	0.038891
hsa_circ_	0.538587	0.0389
hsa_circ_	-0.32781	0.038908
hsa_circ_	-0.48891	0.038913
hsa_circ_	-0.43319	0.038923
hsa_circ_	-0.41584	0.038925
hsa_circ_	-0.4484	0.038925
hsa_circ_	-0.47512	0.038942
hsa_circ_	-0.48463	0.038946
hsa_circ_	-0.53614	0.038956
hsa_circ_	-0.56536	0.038958
hsa_circ_	1.03212	0.038967
hsa_circ_	-0.31623	0.038968
hsa_circ_	0.562508	0.038969
hsa_circ_	0.489076	0.038971
hsa_circ_	0.429146	0.038977
hsa_circ_	-0.77213	0.038982
hsa_circ_	0.450282	0.038983
hsa_circ_	-0.42471	0.038986
hsa_circ_	-0.49142	0.039007
hsa_circ_	-0.40679	0.039014
hsa_circ_	0.631852	0.039014
hsa_circ_	0.438755	0.039015
hsa_circ_	-1.11803	0.039022
hsa_circ_	0.566278	0.039026
hsa_circ_	1.405698	0.039026
hsa_circ_	0.452024	0.039031
hsa_circ_	0.820192	0.039036
hsa_circ_	-0.55506	0.039036
hsa_circ_	0.560888	0.039041
hsa_circ_	-0.58043	0.039042
hsa_circ_	0.653951	0.039058
hsa_circ_	-0.68906	0.039067
hsa_circ_	0.580735	0.039069
hsa_circ_	-0.82047	0.039073

hsa_circ_	-0.56761	0.039081
hsa_circ_	-0.37598	0.039092
hsa_circ_	0.479187	0.039092
hsa_circ_	0.806201	0.039093
hsa_circ_	0.521769	0.039105
hsa_circ_	0.547721	0.03912
hsa_circ_	-1.56273	0.039122
hsa_circ_	0.443139	0.039128
hsa_circ_	0.466399	0.039138
hsa_circ_	0.468464	0.03914
hsa_circ_	0.394335	0.039142
hsa_circ_	0.426146	0.039151
hsa_circ_	-2.39441	0.039155
hsa_circ_	0.981647	0.039156
hsa_circ_	-0.58123	0.039158
hsa_circ_	0.693841	0.039164
hsa_circ_	0.425304	0.039167
hsa_circ_	1.293756	0.039168
hsa_circ_	0.610839	0.039184
hsa_circ_	0.426372	0.039188
hsa_circ_	-0.46959	0.039189
hsa_circ_	0.521783	0.039194
hsa_circ_	-0.41409	0.039201
hsa_circ_	-0.50393	0.039202
hsa_circ_	-0.75415	0.039212
hsa_circ_	0.624528	0.039226
hsa_circ_	-0.74734	0.039235
hsa_circ_	0.581986	0.039236
hsa_circ_	0.465505	0.039248
hsa_circ_	0.568417	0.03925
hsa_circ_	-0.35683	0.039257
hsa_circ_	-0.54292	0.039278
hsa_circ_	-0.71334	0.039283
hsa_circ_	-1.15786	0.039291
hsa_circ_	0.440386	0.039292
hsa_circ_	0.779476	0.039298
hsa_circ_	0.325623	0.039314
hsa_circ_	-0.36689	0.03932
hsa_circ_	-0.54629	0.039323
hsa_circ_	0.772768	0.03933
hsa_circ_	0.616842	0.039333
hsa_circ_	-0.56333	0.039334
hsa_circ_	0.718689	0.039346
hsa_circ_	0.668043	0.039354
hsa_circ_	-0.64001	0.039363
hsa_circ_	-0.53274	0.039366
hsa_circ_	0.624426	0.039368
hsa_circ_	0.42371	0.039369
hsa_circ_	0.429825	0.039369
hsa_circ_	-0.40236	0.039376
hsa_circ_	0.708522	0.039379
hsa_circ_	0.590112	0.039379
hsa_circ_	0.447254	0.039386
hsa_circ_	1.232904	0.039388

hsa_circ_	-0.59447	0.039397
hsa_circ_	-0.43938	0.039401
hsa_circ_	-0.43359	0.039403
hsa_circ_	0.506712	0.039404
hsa_circ_	0.845273	0.039415
hsa_circ_	1.172322	0.039417
hsa_circ_	-1.06378	0.03942
hsa_circ_	-0.35653	0.039421
hsa_circ_	0.461228	0.039423
hsa_circ_	-0.52819	0.039428
hsa_circ_	-0.82946	0.039434
hsa_circ_	-0.33334	0.039446
hsa_circ_	-1.48614	0.039447
hsa_circ_	0.678356	0.039462
hsa_circ_	0.388011	0.039468
hsa_circ_	0.445798	0.039479
hsa_circ_	0.474285	0.039484
hsa_circ_	-0.36087	0.039484
hsa_circ_	-1.48801	0.039485
hsa_circ_	-0.36243	0.039489
hsa_circ_	0.712619	0.039489
hsa_circ_	0.421632	0.039505
hsa_circ_	-0.32546	0.039512
hsa_circ_	-1.43467	0.039517
hsa_circ_	1.001486	0.039532
hsa_circ_	0.643283	0.039534
hsa_circ_	-0.84512	0.039536
hsa_circ_	-0.34528	0.03954
hsa_circ_	-0.38399	0.039553
hsa_circ_	-0.59359	0.039565
hsa_circ_	-0.46279	0.039566
hsa_circ_	-0.38068	0.039569
hsa_circ_	0.717247	0.039579
hsa_circ_	0.433984	0.039579
hsa_circ_	-0.81164	0.039581
hsa_circ_	-0.49188	0.039583
hsa_circ_	0.591483	0.03959
hsa_circ_	-0.314	0.039597
hsa_circ_	-0.59523	0.0396
hsa_circ_	0.472204	0.039605
hsa_circ_	-0.34384	0.039608
hsa_circ_	0.415829	0.039614
hsa_circ_	0.364532	0.039615
hsa_circ_	-0.40523	0.039616
hsa_circ_	0.38617	0.03962
hsa_circ_	0.468957	0.039624
hsa_circ_	0.76241	0.039638
hsa_circ_	-0.59649	0.039647
hsa_circ_	-0.4437	0.039652
hsa_circ_	0.385418	0.039652
hsa_circ_	0.819969	0.039655
hsa_circ_	-0.51158	0.039662
hsa_circ_	-0.43846	0.039678
hsa_circ_	-0.46562	0.039679

hsa_circ_	0.577038	0.039684
hsa_circ_	-0.55503	0.039692
hsa_circ_	-0.39066	0.039697
hsa_circ_	-0.59096	0.039709
hsa_circ_	-0.77959	0.03971
hsa_circ_	0.96311	0.039711
hsa_circ_	0.610996	0.03972
hsa_circ_	1.255237	0.039728
hsa_circ_	-0.59773	0.039735
hsa_circ_	0.653277	0.039737
hsa_circ_	-0.40797	0.039739
hsa_circ_	-1.73388	0.039741
hsa_circ_	0.56297	0.039746
hsa_circ_	0.405319	0.039747
hsa_circ_	0.349092	0.039756
hsa_circ_	0.406815	0.039772
hsa_circ_	-0.81338	0.039773
hsa_circ_	0.423945	0.039783
hsa_circ_	1.291707	0.039783
hsa_circ_	1.076506	0.039785
hsa_circ_	-0.37606	0.039786
hsa_circ_	-0.36815	0.039786
hsa_circ_	-0.80322	0.039796
hsa_circ_	-0.46455	0.039798
hsa_circ_	1.218101	0.039799
hsa_circ_	-0.33757	0.039819
hsa_circ_	-0.33974	0.039827
hsa_circ_	-0.40563	0.039835
hsa_circ_	-0.5028	0.039837
hsa_circ_	-0.36818	0.039845
hsa_circ_	0.667672	0.039859
hsa_circ_	0.435614	0.03986
hsa_circ_	-0.32003	0.039866
hsa_circ_	-0.4166	0.039867
hsa_circ_	-1.14725	0.039878
hsa_circ_	-0.40086	0.03988
hsa_circ_	-0.48737	0.039881
hsa_circ_	0.726745	0.039894
hsa_circ_	0.460584	0.0399
hsa_circ_	-0.37633	0.039907
hsa_circ_	0.416666	0.03991
hsa_circ_	-1.00159	0.039911
hsa_circ_	0.66393	0.039911
hsa_circ_	-1.21264	0.039917
hsa_circ_	-0.57227	0.039918
hsa_circ_	0.487496	0.039919
hsa_circ_	-0.37608	0.039921
hsa_circ_	-0.66339	0.039935
hsa_circ_	0.978316	0.039935
hsa_circ_	0.427568	0.039935
hsa_circ_	-0.49182	0.039941
hsa_circ_	0.502557	0.039945
hsa_circ_	-0.44941	0.039947
hsa_circ_	0.407089	0.039966

hsa_circ_	0.416075	0.039973
hsa_circ_	-0.48267	0.039976
hsa_circ_	-1.084	0.039988
hsa_circ_	-1.1027	0.039994
hsa_circ_	-0.46783	0.039997
hsa_circ_	0.411651	0.039997
hsa_circ_	-0.33914	0.039998
hsa_circ_	-0.53479	0.039999
hsa_circ_	0.936618	0.040005
hsa_circ_	-0.38337	0.040007
hsa_circ_	-0.66485	0.040014
hsa_circ_	0.594538	0.040017
hsa_circ_	-0.31608	0.040018
hsa_circ_	0.602999	0.040025
hsa_circ_	0.61106	0.040035
hsa_circ_	-0.56324	0.040037
hsa_circ_	-0.54513	0.040038
hsa_circ_	-0.44073	0.04004
hsa_circ_	0.539983	0.040041
hsa_circ_	0.63793	0.040052
hsa_circ_	1.579355	0.040056
hsa_circ_	0.37374	0.040069
hsa_circ_	0.453989	0.040073
hsa_circ_	0.881385	0.040079
hsa_circ_	0.863169	0.040079
hsa_circ_	-1.0996	0.040109
hsa_circ_	-0.7572	0.040109
hsa_circ_	-0.37919	0.04011
hsa_circ_	-0.97926	0.040116
hsa_circ_	-0.46611	0.040128
hsa_circ_	-0.40496	0.04013
hsa_circ_	-0.56446	0.040136
hsa_circ_	-1.02968	0.040147
hsa_circ_	1.026559	0.040147
hsa_circ_	-0.33079	0.040148
hsa_circ_	-0.47097	0.040153
hsa_circ_	0.624987	0.040155
hsa_circ_	0.685665	0.040165
hsa_circ_	0.368535	0.040166
hsa_circ_	-0.43711	0.040177
hsa_circ_	-1.24847	0.040204
hsa_circ_	-0.32114	0.04021
hsa_circ_	0.456955	0.04021
hsa_circ_	-0.69861	0.040212
hsa_circ_	-0.32496	0.040216
hsa_circ_	-0.48295	0.040217
hsa_circ_	0.516221	0.040219
hsa_circ_	-0.45929	0.040238
hsa_circ_	0.408272	0.040243
hsa_circ_	0.39541	0.040249
hsa_circ_	-0.52584	0.040263
hsa_circ_	0.762374	0.040263
hsa_circ_	-0.47312	0.040276
hsa_circ_	-0.444	0.040277

hsa_circ_	1.083617	0.040284
hsa_circ_	0.633868	0.040295
hsa_circ_	-0.33831	0.040298
hsa_circ_	0.659837	0.040301
hsa_circ_	0.614728	0.040316
hsa_circ_	0.516074	0.040323
hsa_circ_	0.426348	0.040331
hsa_circ_	-0.39082	0.040334
hsa_circ_	0.412627	0.040335
hsa_circ_	-0.47344	0.040339
hsa_circ_	-0.71225	0.040341
hsa_circ_	0.326344	0.040353
hsa_circ_	0.454327	0.040359
hsa_circ_	0.967453	0.040369
hsa_circ_	0.342609	0.040369
hsa_circ_	-0.3104	0.04037
hsa_circ_	0.37607	0.040375
hsa_circ_	-0.9138	0.040377
hsa_circ_	0.444064	0.040386
hsa_circ_	-0.61055	0.040396
hsa_circ_	-0.97555	0.040398
hsa_circ_	-0.48504	0.040401
hsa_circ_	-0.43033	0.040412
hsa_circ_	0.692714	0.040425
hsa_circ_	-0.53492	0.040433
hsa_circ_	-1.31571	0.040438
hsa_circ_	-0.56895	0.04044
hsa_circ_	-0.33656	0.040443
hsa_circ_	-0.41653	0.040444
hsa_circ_	0.424337	0.040444
hsa_circ_	0.785195	0.040446
hsa_circ_	0.542495	0.040448
hsa_circ_	-1.03814	0.040448
hsa_circ_	-0.68468	0.040454
hsa_circ_	-0.45584	0.040458
hsa_circ_	0.470734	0.040464
hsa_circ_	-0.43675	0.040465
hsa_circ_	-0.48959	0.040467
hsa_circ_	-1.40972	0.040468
hsa_circ_	0.39572	0.04047
hsa_circ_	-0.49931	0.04047
hsa_circ_	0.9494	0.040473
hsa_circ_	-0.95802	0.040474
hsa_circ_	0.374458	0.040478
hsa_circ_	-0.40724	0.040478
hsa_circ_	0.430477	0.040482
hsa_circ_	0.660734	0.040487
hsa_circ_	0.469472	0.040488
hsa_circ_	-0.47981	0.040489
hsa_circ_	0.493673	0.040499
hsa_circ_	0.39798	0.040505
hsa_circ_	0.51323	0.040507
hsa_circ_	-0.32887	0.040507
hsa_circ_	0.83699	0.040511

hsa_circ_	-0.45724	0.040512
hsa_circ_	-0.83896	0.040515
hsa_circ_	0.948712	0.040515
hsa_circ_	-1.22874	0.040526
hsa_circ_	0.44317	0.040527
hsa_circ_	0.46925	0.040532
hsa_circ_	0.439991	0.040536
hsa_circ_	-0.6774	0.040538
hsa_circ_	0.471375	0.040542
hsa_circ_	0.768511	0.040544
hsa_circ_	0.339676	0.040549
hsa_circ_	0.342229	0.040551
hsa_circ_	-0.94984	0.040567
hsa_circ_	-0.41222	0.040579
hsa_circ_	-0.44023	0.040585
hsa_circ_	0.436027	0.040606
hsa_circ_	0.465026	0.040629
hsa_circ_	-0.33673	0.040632
hsa_circ_	0.439966	0.040634
hsa_circ_	1.021918	0.040639
hsa_circ_	0.573967	0.04065
hsa_circ_	0.471326	0.040655
hsa_circ_	-0.65379	0.040668
hsa_circ_	0.64374	0.040669
hsa_circ_	0.758289	0.040674
hsa_circ_	0.733196	0.040676
hsa_circ_	-0.56854	0.040678
hsa_circ_	0.44567	0.040691
hsa_circ_	0.5235	0.040699
hsa_circ_	-0.56219	0.040701
hsa_circ_	-0.40847	0.040709
hsa_circ_	-0.46372	0.04072
hsa_circ_	1.114449	0.040725
hsa_circ_	-0.34052	0.040753
hsa_circ_	0.497582	0.040756
hsa_circ_	0.372388	0.040759
hsa_circ_	0.468745	0.040762
hsa_circ_	0.42538	0.040778
hsa_circ_	0.902849	0.04078
hsa_circ_	-0.62302	0.040789
hsa_circ_	-0.43318	0.040792
hsa_circ_	0.351187	0.040803
hsa_circ_	-0.37191	0.040821
hsa_circ_	0.599488	0.040824
hsa_circ_	-0.43022	0.040827
hsa_circ_	0.336185	0.04083
hsa_circ_	-0.35785	0.040838
hsa_circ_	-0.47762	0.040839
hsa_circ_	-0.52367	0.040839
hsa_circ_	0.643648	0.040839
hsa_circ_	0.486068	0.040864
hsa_circ_	-0.75295	0.040865
hsa_circ_	0.436168	0.040866
hsa_circ_	0.47575	0.040867

hsa_circ_	0.916999	0.040869
hsa_circ_	-0.48786	0.040874
hsa_circ_	0.415566	0.040883
hsa_circ_	-0.37952	0.040883
hsa_circ_	-1.04283	0.040886
hsa_circ_	-0.4575	0.040889
hsa_circ_	0.398829	0.04089
hsa_circ_	-0.43568	0.040906
hsa_circ_	-0.968	0.040923
hsa_circ_	-0.31634	0.040923
hsa_circ_	-1.31571	0.040923
hsa_circ_	0.521216	0.040923
hsa_circ_	0.63849	0.040926
hsa_circ_	0.363436	0.040927
hsa_circ_	0.384097	0.040928
hsa_circ_	-0.38103	0.040929
hsa_circ_	0.412666	0.04094
hsa_circ_	0.383714	0.040945
hsa_circ_	0.386963	0.040949
hsa_circ_	0.595856	0.040953
hsa_circ_	0.32711	0.040955
hsa_circ_	0.65632	0.040961
hsa_circ_	0.407489	0.040983
hsa_circ_	-0.50628	0.040983
hsa_circ_	0.360289	0.040986
hsa_circ_	0.713075	0.040988
hsa_circ_	0.47068	0.040993
hsa_circ_	0.458693	0.040996
hsa_circ_	-0.37419	0.040997
hsa_circ_	-0.30669	0.040998
hsa_circ_	-1.06714	0.040999
hsa_circ_	0.563235	0.041004
hsa_circ_	0.416009	0.041006
hsa_circ_	0.576896	0.041023
hsa_circ_	0.871074	0.041033
hsa_circ_	0.757218	0.041034
hsa_circ_	-0.34935	0.041038
hsa_circ_	0.509441	0.041047
hsa_circ_	-0.56098	0.041051
hsa_circ_	0.758762	0.041054
hsa_circ_	1.286881	0.041054
hsa_circ_	-0.64955	0.04106
hsa_circ_	0.493827	0.041061
hsa_circ_	0.414158	0.041065
hsa_circ_	-0.4666	0.041067
hsa_circ_	-0.67925	0.041069
hsa_circ_	-0.86723	0.041069
hsa_circ_	-0.7769	0.041071
hsa_circ_	0.673783	0.041079
hsa_circ_	0.480658	0.041083
hsa_circ_	1.397047	0.041089
hsa_circ_	-0.36775	0.041094
hsa_circ_	0.548692	0.041098
hsa_circ_	0.77807	0.041099

hsa_circ_	0.737296	0.041107
hsa_circ_	-0.41929	0.04111
hsa_circ_	-0.55352	0.041111
hsa_circ_	0.42711	0.041111
hsa_circ_	-0.39396	0.041111
hsa_circ_	0.341266	0.041113
hsa_circ_	-0.41883	0.041116
hsa_circ_	0.810809	0.04112
hsa_circ_	0.494316	0.04113
hsa_circ_	0.43869	0.041135
hsa_circ_	-0.59439	0.04114
hsa_circ_	0.622308	0.041144
hsa_circ_	-0.64157	0.041153
hsa_circ_	0.350666	0.041154
hsa_circ_	1.288184	0.041169
hsa_circ_	-0.4702	0.041171
hsa_circ_	-1.37082	0.041175
hsa_circ_	0.930295	0.04118
hsa_circ_	0.714415	0.041196
hsa_circ_	0.402906	0.041202
hsa_circ_	0.84811	0.041208
hsa_circ_	-0.34703	0.041209
hsa_circ_	-0.54786	0.04121
hsa_circ_	-0.75431	0.041211
hsa_circ_	-0.77808	0.041211
hsa_circ_	-0.31722	0.041215
hsa_circ_	-0.5669	0.04122
hsa_circ_	-0.61729	0.041229
hsa_circ_	0.500126	0.041231
hsa_circ_	-0.32537	0.041232
hsa_circ_	-0.4702	0.041235
hsa_circ_	0.485708	0.041249
hsa_circ_	0.576931	0.041262
hsa_circ_	-0.41367	0.041264
hsa_circ_	-0.48541	0.041266
hsa_circ_	0.494305	0.041282
hsa_circ_	0.471977	0.041288
hsa_circ_	-0.42479	0.04129
hsa_circ_	-0.37899	0.04129
hsa_circ_	-0.33847	0.041292
hsa_circ_	-0.39858	0.041298
hsa_circ_	-0.62666	0.041305
hsa_circ_	-0.43824	0.041323
hsa_circ_	-0.44463	0.041343
hsa_circ_	-0.49681	0.041347
hsa_circ_	-0.38651	0.041354
hsa_circ_	-0.73574	0.041354
hsa_circ_	0.360131	0.041355
hsa_circ_	0.637036	0.041357
hsa_circ_	0.924778	0.041359
hsa_circ_	0.460953	0.041361
hsa_circ_	-0.61639	0.041402
hsa_circ_	-0.39415	0.041402
hsa_circ_	0.772079	0.04141

hsa_circ_	0.805932	0.041414
hsa_circ_	-0.48637	0.041415
hsa_circ_	0.462992	0.041417
hsa_circ_	-0.57278	0.041418
hsa_circ_	0.608854	0.041418
hsa_circ_	0.769229	0.041422
hsa_circ_	0.603714	0.041431
hsa_circ_	-0.44399	0.041431
hsa_circ_	-0.36399	0.04144
hsa_circ_	-0.52594	0.04144
hsa_circ_	0.498015	0.041447
hsa_circ_	0.996869	0.041452
hsa_circ_	0.397537	0.041458
hsa_circ_	-0.38788	0.04146
hsa_circ_	0.356776	0.041464
hsa_circ_	0.493177	0.041468
hsa_circ_	0.395898	0.041471
hsa_circ_	-0.96826	0.041473
hsa_circ_	-0.39041	0.041485
hsa_circ_	0.822503	0.041488
hsa_circ_	-0.52344	0.041488
hsa_circ_	0.517342	0.041488
hsa_circ_	-0.93966	0.041511
hsa_circ_	0.843492	0.041535
hsa_circ_	-0.35761	0.041536
hsa_circ_	0.351352	0.041555
hsa_circ_	0.672581	0.041562
hsa_circ_	-0.52002	0.041562
hsa_circ_	-0.46326	0.041568
hsa_circ_	0.410521	0.041568
hsa_circ_	-0.37921	0.041579
hsa_circ_	-0.49094	0.041584
hsa_circ_	-0.60721	0.041586
hsa_circ_	-0.49891	0.04159
hsa_circ_	-0.33429	0.041595
hsa_circ_	0.492961	0.041603
hsa_circ_	-1.12747	0.041606
hsa_circ_	-0.3978	0.041617
hsa_circ_	-0.75548	0.04162
hsa_circ_	0.63019	0.04163
hsa_circ_	0.797396	0.041648
hsa_circ_	-1.43335	0.041649
hsa_circ_	0.740366	0.041649
hsa_circ_	-0.52243	0.04165
hsa_circ_	-0.62005	0.041652
hsa_circ_	-0.79701	0.041656
hsa_circ_	1.203936	0.041656
hsa_circ_	0.520915	0.041666
hsa_circ_	-0.36187	0.041668
hsa_circ_	0.502376	0.041678
hsa_circ_	-0.39489	0.04168
hsa_circ_	-0.48706	0.041682
hsa_circ_	-0.57796	0.041682
hsa_circ_	-0.38975	0.041683

hsa_circ_	-0.44535	0.041684
hsa_circ_	-0.63161	0.041689
hsa_circ_	-1.46071	0.0417
hsa_circ_	0.363211	0.04171
hsa_circ_	-0.52171	0.041718
hsa_circ_	-0.49648	0.041722
hsa_circ_	0.503087	0.041726
hsa_circ_	-0.34778	0.04176
hsa_circ_	0.424131	0.041767
hsa_circ_	-1.52368	0.041796
hsa_circ_	-0.41046	0.041807
hsa_circ_	-0.32996	0.04181
hsa_circ_	-0.52108	0.041814
hsa_circ_	-2.0099	0.041814
hsa_circ_	-0.3493	0.041815
hsa_circ_	0.972586	0.041816
hsa_circ_	-0.36937	0.041816
hsa_circ_	-0.3852	0.041816
hsa_circ_	-0.44568	0.04183
hsa_circ_	-0.79572	0.041831
hsa_circ_	-0.36383	0.041831
hsa_circ_	-0.4327	0.041838
hsa_circ_	0.481396	0.04185
hsa_circ_	-0.32343	0.041851
hsa_circ_	-0.98836	0.041867
hsa_circ_	0.501594	0.041876
hsa_circ_	-0.45164	0.041886
hsa_circ_	-0.47658	0.041886
hsa_circ_	-0.58836	0.041887
hsa_circ_	0.405869	0.04189
hsa_circ_	-0.35059	0.041893
hsa_circ_	0.781587	0.0419
hsa_circ_	0.513764	0.041907
hsa_circ_	1.050042	0.041911
hsa_circ_	-0.36375	0.041916
hsa_circ_	0.395462	0.04193
hsa_circ_	-0.71055	0.041935
hsa_circ_	-0.35471	0.041941
hsa_circ_	-0.59326	0.041941
hsa_circ_	0.34123	0.041946
hsa_circ_	0.687715	0.041951
hsa_circ_	0.538692	0.041952
hsa_circ_	0.324509	0.041953
hsa_circ_	0.38868	0.041955
hsa_circ_	0.65182	0.041956
hsa_circ_	-0.65702	0.041961
hsa_circ_	-0.69629	0.041971
hsa_circ_	0.477701	0.041992
hsa_circ_	0.43025	0.042006
hsa_circ_	-0.43332	0.042006
hsa_circ_	-0.50826	0.042011
hsa_circ_	-0.37933	0.042011
hsa_circ_	0.587007	0.042013
hsa_circ_	-0.41072	0.042023

hsa_circ_	-0.32421	0.04204
hsa_circ_	-1.66223	0.042075
hsa_circ_	0.345812	0.042081
hsa_circ_	-0.44098	0.042088
hsa_circ_	-0.37321	0.042094
hsa_circ_	0.422878	0.042097
hsa_circ_	-0.37376	0.0421
hsa_circ_	0.394534	0.042112
hsa_circ_	0.477327	0.042115
hsa_circ_	-0.35541	0.042124
hsa_circ_	-0.52849	0.042126
hsa_circ_	0.380835	0.042126
hsa_circ_	-0.53858	0.042135
hsa_circ_	0.570074	0.042136
hsa_circ_	-0.69351	0.042142
hsa_circ_	-0.40378	0.042144
hsa_circ_	-0.37877	0.042149
hsa_circ_	-0.45398	0.042161
hsa_circ_	0.477187	0.042171
hsa_circ_	0.692543	0.042175
hsa_circ_	0.591664	0.042176
hsa_circ_	0.485062	0.042178
hsa_circ_	-0.46282	0.042186
hsa_circ_	-0.3586	0.042211
hsa_circ_	-0.33084	0.042214
hsa_circ_	-1.20747	0.042231
hsa_circ_	0.998438	0.042231
hsa_circ_	-0.54087	0.042235
hsa_circ_	0.578566	0.042242
hsa_circ_	-0.42431	0.042246
hsa_circ_	0.736183	0.042248
hsa_circ_	0.398517	0.04225
hsa_circ_	-0.53031	0.042257
hsa_circ_	0.475201	0.042263
hsa_circ_	-0.42199	0.042263
hsa_circ_	0.55575	0.042288
hsa_circ_	0.764195	0.042289
hsa_circ_	-0.91942	0.042294
hsa_circ_	0.417493	0.042296
hsa_circ_	1.075404	0.0423
hsa_circ_	-0.38024	0.0423
hsa_circ_	0.574359	0.042312
hsa_circ_	-0.31743	0.042325
hsa_circ_	0.594804	0.042331
hsa_circ_	-0.48819	0.04234
hsa_circ_	-1.74771	0.042344
hsa_circ_	-0.63351	0.042347
hsa_circ_	0.327469	0.042356
hsa_circ_	-0.4358	0.042367
hsa_circ_	0.323117	0.042372
hsa_circ_	-0.482	0.042379
hsa_circ_	0.44366	0.042392
hsa_circ_	-0.54456	0.042393
hsa_circ_	-0.63866	0.042394

hsa_circ_	0.810257	0.042395
hsa_circ_	-0.33632	0.0424
hsa_circ_	-0.69346	0.04241
hsa_circ_	0.432817	0.042414
hsa_circ_	0.877146	0.042421
hsa_circ_	-0.52039	0.042429
hsa_circ_	0.469665	0.042435
hsa_circ_	-0.37474	0.042442
hsa_circ_	1.21976	0.042444
hsa_circ_	0.535462	0.042453
hsa_circ_	-0.39471	0.042475
hsa_circ_	-1.0728	0.042478
hsa_circ_	0.571156	0.042495
hsa_circ_	0.359479	0.042497
hsa_circ_	-0.37845	0.0425
hsa_circ_	0.668952	0.042503
hsa_circ_	-0.44718	0.042505
hsa_circ_	-0.42346	0.04251
hsa_circ_	0.678546	0.042511
hsa_circ_	0.467263	0.042516
hsa_circ_	0.435729	0.042519
hsa_circ_	0.34471	0.042522
hsa_circ_	1.037566	0.042525
hsa_circ_	0.561113	0.042539
hsa_circ_	0.362007	0.042542
hsa_circ_	0.575063	0.042543
hsa_circ_	0.501183	0.042546
hsa_circ_	0.57333	0.042552
hsa_circ_	-0.45453	0.042563
hsa_circ_	0.73661	0.042567
hsa_circ_	-2.06721	0.042569
hsa_circ_	-1.04028	0.042571
hsa_circ_	-0.32589	0.042573
hsa_circ_	-0.87657	0.042576
hsa_circ_	0.433783	0.042582
hsa_circ_	0.482708	0.042592
hsa_circ_	-0.5619	0.042597
hsa_circ_	-0.3864	0.042621
hsa_circ_	-0.36387	0.042628
hsa_circ_	-0.39403	0.04263
hsa_circ_	1.088928	0.042631
hsa_circ_	0.43037	0.042632
hsa_circ_	-0.34462	0.042635
hsa_circ_	0.325627	0.042649
hsa_circ_	1.310604	0.042651
hsa_circ_	0.885182	0.042657
hsa_circ_	0.384008	0.042659
hsa_circ_	0.49433	0.04268
hsa_circ_	1.395232	0.042681
hsa_circ_	0.651795	0.042685
hsa_circ_	-0.46476	0.042685
hsa_circ_	0.439064	0.042688
hsa_circ_	-0.3862	0.042695
hsa_circ_	0.658441	0.0427

hsa_circ_	0.710058	0.042701
hsa_circ_	0.512881	0.042713
hsa_circ_	-0.34763	0.042716
hsa_circ_	-0.32523	0.042723
hsa_circ_	0.399629	0.042727
hsa_circ_	-0.34879	0.042738
hsa_circ_	-0.98179	0.042745
hsa_circ_	0.416336	0.042757
hsa_circ_	-0.50481	0.042758
hsa_circ_	-0.47944	0.04276
hsa_circ_	-0.32295	0.04277
hsa_circ_	-0.57912	0.042785
hsa_circ_	-0.69628	0.042796
hsa_circ_	0.421065	0.042802
hsa_circ_	-0.3304	0.042804
hsa_circ_	-0.40323	0.042804
hsa_circ_	0.715953	0.042817
hsa_circ_	-1.09372	0.04283
hsa_circ_	-0.31906	0.042834
hsa_circ_	-0.36781	0.042836
hsa_circ_	-0.30209	0.042844
hsa_circ_	0.734714	0.042845
hsa_circ_	-0.7077	0.042864
hsa_circ_	-0.36268	0.042871
hsa_circ_	-0.30922	0.042874
hsa_circ_	-1.08805	0.042878
hsa_circ_	-0.41773	0.04289
hsa_circ_	-0.3539	0.042896
hsa_circ_	-0.32922	0.042911
hsa_circ_	-0.45436	0.042921
hsa_circ_	-0.32359	0.042922
hsa_circ_	-0.52568	0.042929
hsa_circ_	0.347768	0.042944
hsa_circ_	0.65217	0.042952
hsa_circ_	-0.3717	0.042957
hsa_circ_	-0.36204	0.042964
hsa_circ_	0.51204	0.042969
hsa_circ_	-0.67992	0.042973
hsa_circ_	0.369883	0.042975
hsa_circ_	0.326881	0.04299
hsa_circ_	0.463329	0.042993
hsa_circ_	0.479786	0.043018
hsa_circ_	0.42682	0.043021
hsa_circ_	-0.3631	0.043022
hsa_circ_	0.620199	0.043024
hsa_circ_	0.363892	0.043025
hsa_circ_	-0.38669	0.043029
hsa_circ_	-0.70306	0.043029
hsa_circ_	-0.37201	0.043031
hsa_circ_	0.507344	0.043035
hsa_circ_	0.554759	0.043039
hsa_circ_	0.568351	0.043045
hsa_circ_	1.533979	0.043047
hsa_circ_	-0.53202	0.043051

hsa_circ_	-1.83834	0.043052
hsa_circ_	0.759011	0.043055
hsa_circ_	-0.8047	0.043058
hsa_circ_	-0.34583	0.043059
hsa_circ_	0.566857	0.04307
hsa_circ_	-0.62447	0.04308
hsa_circ_	-1.01668	0.043082
hsa_circ_	0.812087	0.043098
hsa_circ_	0.527358	0.043103
hsa_circ_	-0.37699	0.043106
hsa_circ_	-0.47369	0.043113
hsa_circ_	0.496609	0.043119
hsa_circ_	-0.35571	0.04312
hsa_circ_	-0.31434	0.043121
hsa_circ_	0.833029	0.043121
hsa_circ_	0.419464	0.043132
hsa_circ_	0.428819	0.043141
hsa_circ_	-0.40886	0.043145
hsa_circ_	-0.33478	0.043147
hsa_circ_	0.584853	0.043154
hsa_circ_	0.666936	0.043156
hsa_circ_	-0.37101	0.043159
hsa_circ_	-0.39866	0.043169
hsa_circ_	0.671953	0.043171
hsa_circ_	-0.48409	0.043173
hsa_circ_	-0.58084	0.043174
hsa_circ_	0.400174	0.043176
hsa_circ_	1.218776	0.043187
hsa_circ_	0.771602	0.043188
hsa_circ_	0.447717	0.043208
hsa_circ_	0.513985	0.043208
hsa_circ_	0.506644	0.043216
hsa_circ_	-1.12667	0.043219
hsa_circ_	-1.25518	0.043234
hsa_circ_	-0.34978	0.043238
hsa_circ_	0.359757	0.043238
hsa_circ_	-0.79127	0.043241
hsa_circ_	-0.53228	0.043251
hsa_circ_	0.787127	0.043259
hsa_circ_	-0.33977	0.04326
hsa_circ_	-0.46953	0.043263
hsa_circ_	-0.72983	0.043267
hsa_circ_	1.194621	0.043268
hsa_circ_	0.571116	0.043276
hsa_circ_	-0.30426	0.043285
hsa_circ_	-0.36669	0.043286
hsa_circ_	-0.30112	0.043287
hsa_circ_	-0.73704	0.04329
hsa_circ_	0.731151	0.043292
hsa_circ_	-0.38724	0.043301
hsa_circ_	0.786437	0.043306
hsa_circ_	0.403189	0.043315
hsa_circ_	0.990406	0.043315
hsa_circ_	0.361126	0.043317

hsa_circ_	-0.93186	0.043318
hsa_circ_	-0.67077	0.043322
hsa_circ_	0.618504	0.043324
hsa_circ_	0.429243	0.043336
hsa_circ_	-0.39498	0.043338
hsa_circ_	-0.35984	0.04335
hsa_circ_	-1.03958	0.043362
hsa_circ_	-0.38556	0.043369
hsa_circ_	-0.46608	0.043374
hsa_circ_	-0.31811	0.043396
hsa_circ_	-0.33539	0.043399
hsa_circ_	1.003749	0.043404
hsa_circ_	-0.31478	0.043407
hsa_circ_	-0.8045	0.043409
hsa_circ_	0.461759	0.043418
hsa_circ_	-0.94212	0.043427
hsa_circ_	0.459319	0.043436
hsa_circ_	0.834267	0.043446
hsa_circ_	-0.34037	0.043464
hsa_circ_	0.80747	0.043466
hsa_circ_	-0.32609	0.043471
hsa_circ_	0.488699	0.043488
hsa_circ_	0.435136	0.043493
hsa_circ_	0.928507	0.043496
hsa_circ_	-0.37037	0.043499
hsa_circ_	-0.65546	0.04351
hsa_circ_	0.958813	0.043516
hsa_circ_	-0.51795	0.043523
hsa_circ_	0.412128	0.043529
hsa_circ_	0.800088	0.043533
hsa_circ_	0.688733	0.043539
hsa_circ_	-0.46002	0.043553
hsa_circ_	0.867349	0.043579
hsa_circ_	-0.45704	0.043582
hsa_circ_	0.714956	0.043591
hsa_circ_	0.770337	0.0436
hsa_circ_	0.991185	0.043604
hsa_circ_	0.711349	0.043614
hsa_circ_	0.378492	0.043614
hsa_circ_	-0.35624	0.043619
hsa_circ_	0.467078	0.043624
hsa_circ_	0.783419	0.043626
hsa_circ_	0.623029	0.043629
hsa_circ_	0.383088	0.043633
hsa_circ_	-0.48684	0.043636
hsa_circ_	0.337467	0.043644
hsa_circ_	0.782612	0.043645
hsa_circ_	-0.51588	0.043647
hsa_circ_	0.956316	0.043654
hsa_circ_	0.550284	0.043683
hsa_circ_	-0.39042	0.043695
hsa_circ_	-1.16903	0.043699
hsa_circ_	-0.54316	0.043699
hsa_circ_	-0.62519	0.043705

hsa_circ_	0.387731	0.043714
hsa_circ_	0.379267	0.04372
hsa_circ_	-0.32439	0.043727
hsa_circ_	-0.39271	0.043741
hsa_circ_	0.527104	0.043741
hsa_circ_	0.3848	0.043742
hsa_circ_	1.684428	0.043751
hsa_circ_	-0.48153	0.043752
hsa_circ_	0.44025	0.043754
hsa_circ_	-0.8419	0.043756
hsa_circ_	0.355246	0.043758
hsa_circ_	-0.51165	0.04376
hsa_circ_	-0.62865	0.04376
hsa_circ_	-1.03279	0.043762
hsa_circ_	-0.59321	0.043766
hsa_circ_	0.473347	0.043782
hsa_circ_	-0.43584	0.043785
hsa_circ_	0.327303	0.043788
hsa_circ_	1.225916	0.043794
hsa_circ_	0.730042	0.043794
hsa_circ_	0.382413	0.043806
hsa_circ_	0.417757	0.043809
hsa_circ_	-0.44806	0.043809
hsa_circ_	-0.42525	0.043809
hsa_circ_	0.960225	0.043813
hsa_circ_	-0.43805	0.043816
hsa_circ_	-0.37009	0.043819
hsa_circ_	0.474292	0.04382
hsa_circ_	-0.3926	0.043823
hsa_circ_	-0.90022	0.043827
hsa_circ_	-0.41085	0.043828
hsa_circ_	-1.269	0.043832
hsa_circ_	-0.43262	0.043832
hsa_circ_	-1.68827	0.043833
hsa_circ_	-0.78757	0.043842
hsa_circ_	0.310299	0.043844
hsa_circ_	-0.43943	0.04385
hsa_circ_	0.331838	0.043852
hsa_circ_	-0.48269	0.043873
hsa_circ_	0.603947	0.043874
hsa_circ_	0.334989	0.043877
hsa_circ_	-0.44723	0.043883
hsa_circ_	0.61548	0.043897
hsa_circ_	0.857188	0.0439
hsa_circ_	1.086904	0.043911
hsa_circ_	0.560164	0.043912
hsa_circ_	0.76777	0.043918
hsa_circ_	0.361237	0.043933
hsa_circ_	-0.37444	0.043935
hsa_circ_	-0.73791	0.043954
hsa_circ_	0.395674	0.043955
hsa_circ_	0.822371	0.043958
hsa_circ_	0.349499	0.043965
hsa_circ_	-0.71109	0.043975

hsa_circ_	-0.39968	0.043979
hsa_circ_	1.092345	0.043981
hsa_circ_	0.637639	0.043984
hsa_circ_	-0.48071	0.043994
hsa_circ_	0.575496	0.043996
hsa_circ_	0.342255	0.043999
hsa_circ_	0.45748	0.044005
hsa_circ_	0.54765	0.044023
hsa_circ_	-0.72012	0.044031
hsa_circ_	1.595042	0.044036
hsa_circ_	-0.7634	0.044037
hsa_circ_	-0.38382	0.044046
hsa_circ_	-0.82813	0.044053
hsa_circ_	-0.30638	0.04406
hsa_circ_	-0.78642	0.04408
hsa_circ_	-0.45852	0.044082
hsa_circ_	-1.19105	0.044083
hsa_circ_	-0.67018	0.044085
hsa_circ_	-0.55505	0.04409
hsa_circ_	-0.60182	0.04411
hsa_circ_	-0.47153	0.044132
hsa_circ_	-0.44397	0.044141
hsa_circ_	0.665755	0.044151
hsa_circ_	-0.55299	0.044157
hsa_circ_	0.719632	0.044172
hsa_circ_	0.725529	0.044174
hsa_circ_	-0.42393	0.044178
hsa_circ_	0.455532	0.044181
hsa_circ_	0.42319	0.044197
hsa_circ_	-0.36329	0.044215
hsa_circ_	-0.69828	0.044217
hsa_circ_	-1.30478	0.044219
hsa_circ_	-0.46654	0.044223
hsa_circ_	-0.47737	0.044224
hsa_circ_	-0.45761	0.044238
hsa_circ_	-1.6741	0.04424
hsa_circ_	1.093265	0.044251
hsa_circ_	-0.63494	0.044253
hsa_circ_	-0.68061	0.044258
hsa_circ_	0.609588	0.044259
hsa_circ_	0.527472	0.044265
hsa_circ_	0.783	0.044289
hsa_circ_	0.440853	0.044295
hsa_circ_	0.422596	0.044297
hsa_circ_	-0.59058	0.044298
hsa_circ_	-0.49441	0.044307
hsa_circ_	0.369106	0.044317
hsa_circ_	0.488172	0.044324
hsa_circ_	-0.6855	0.044331
hsa_circ_	0.474449	0.044358
hsa_circ_	1.039919	0.04436
hsa_circ_	0.657545	0.044363
hsa_circ_	0.719268	0.04438
hsa_circ_	0.651371	0.044381

hsa_circ_	1.125771	0.044402
hsa_circ_	0.54528	0.044405
hsa_circ_	-0.91221	0.044408
hsa_circ_	0.564942	0.044417
hsa_circ_	0.459986	0.044428
hsa_circ_	0.433702	0.04443
hsa_circ_	-0.44037	0.044433
hsa_circ_	0.422215	0.044436
hsa_circ_	-0.38386	0.04444
hsa_circ_	0.354222	0.044448
hsa_circ_	0.375366	0.044454
hsa_circ_	0.656186	0.044455
hsa_circ_	-0.35915	0.044457
hsa_circ_	-0.67761	0.04446
hsa_circ_	-0.45998	0.044474
hsa_circ_	0.473986	0.044474
hsa_circ_	0.435933	0.044479
hsa_circ_	-0.44755	0.04448
hsa_circ_	-0.34361	0.044482
hsa_circ_	-0.60115	0.044483
hsa_circ_	0.362179	0.044485
hsa_circ_	0.465326	0.044488
hsa_circ_	-0.43092	0.044498
hsa_circ_	-0.78183	0.044513
hsa_circ_	0.441072	0.044524
hsa_circ_	-1.32893	0.044526
hsa_circ_	0.413797	0.044526
hsa_circ_	-0.36633	0.044526
hsa_circ_	-0.66015	0.044532
hsa_circ_	0.722691	0.044535
hsa_circ_	-0.3322	0.044539
hsa_circ_	-0.63473	0.044542
hsa_circ_	0.392744	0.044546
hsa_circ_	0.407848	0.044569
hsa_circ_	-0.34153	0.04457
hsa_circ_	0.371166	0.044572
hsa_circ_	-0.61526	0.044572
hsa_circ_	-0.58895	0.044573
hsa_circ_	-0.37676	0.044575
hsa_circ_	1.383349	0.044579
hsa_circ_	-0.33033	0.044582
hsa_circ_	-0.39692	0.044597
hsa_circ_	-0.45951	0.044599
hsa_circ_	0.951767	0.044601
hsa_circ_	1.335574	0.044602
hsa_circ_	-0.57194	0.044605
hsa_circ_	-0.4912	0.044606
hsa_circ_	-1.11431	0.044608
hsa_circ_	0.699957	0.044609
hsa_circ_	1.283235	0.044618
hsa_circ_	-0.32708	0.044618
hsa_circ_	-0.43556	0.044625
hsa_circ_	0.437474	0.044635
hsa_circ_	-0.40944	0.044636

hsa_circ_	0.640735	0.044642
hsa_circ_	0.526666	0.044647
hsa_circ_	-0.77821	0.044648
hsa_circ_	0.929256	0.044654
hsa_circ_	0.891373	0.044662
hsa_circ_	1.068634	0.04467
hsa_circ_	1.007692	0.044675
hsa_circ_	0.378938	0.044689
hsa_circ_	-0.33569	0.044705
hsa_circ_	-0.92961	0.044706
hsa_circ_	-0.49201	0.044711
hsa_circ_	0.416959	0.044713
hsa_circ_	1.028558	0.04472
hsa_circ_	-0.5693	0.04472
hsa_circ_	0.356056	0.044724
hsa_circ_	-0.34257	0.044728
hsa_circ_	0.501147	0.044732
hsa_circ_	-0.47086	0.044735
hsa_circ_	-0.65137	0.044745
hsa_circ_	-0.6675	0.044749
hsa_circ_	0.47275	0.044765
hsa_circ_	0.424245	0.04477
hsa_circ_	0.829539	0.044776
hsa_circ_	-1.37549	0.044778
hsa_circ_	-0.51432	0.044785
hsa_circ_	0.551189	0.044786
hsa_circ_	-0.32091	0.04479
hsa_circ_	-0.35017	0.04479
hsa_circ_	-0.39135	0.044792
hsa_circ_	0.38104	0.044801
hsa_circ_	-0.478	0.044807
hsa_circ_	0.389557	0.04481
hsa_circ_	0.531116	0.044815
hsa_circ_	0.600961	0.044833
hsa_circ_	-0.36501	0.044834
hsa_circ_	-0.47217	0.044839
hsa_circ_	-0.64848	0.044844
hsa_circ_	0.600846	0.04485
hsa_circ_	-1.00249	0.044854
hsa_circ_	-0.52456	0.044865
hsa_circ_	0.541565	0.044872
hsa_circ_	0.448037	0.044876
hsa_circ_	-0.63581	0.044884
hsa_circ_	0.415516	0.044886
hsa_circ_	-0.71519	0.044892
hsa_circ_	0.651248	0.044895
hsa_circ_	-0.5651	0.044896
hsa_circ_	0.356703	0.044911
hsa_circ_	0.580657	0.044912
hsa_circ_	-0.36867	0.044916
hsa_circ_	0.42419	0.044916
hsa_circ_	0.524676	0.044924
hsa_circ_	0.977582	0.04493
hsa_circ_	-0.58341	0.044935

hsa_circ_	-1.29786	0.044954
hsa_circ_	0.502052	0.044954
hsa_circ_	0.619514	0.044956
hsa_circ_	0.648276	0.044959
hsa_circ_	-0.93113	0.044961
hsa_circ_	-0.3379	0.044967
hsa_circ_	-0.35066	0.044974
hsa_circ_	0.714675	0.044977
hsa_circ_	-0.46336	0.044977
hsa_circ_	-0.52405	0.044992
hsa_circ_	-0.41125	0.044995
hsa_circ_	0.410339	0.044995
hsa_circ_	-0.91575	0.044997
hsa_circ_	-0.36209	0.045008
hsa_circ_	0.307583	0.045015
hsa_circ_	-0.33302	0.045034
hsa_circ_	-0.48558	0.045034
hsa_circ_	-0.32736	0.04504
hsa_circ_	-0.39402	0.045041
hsa_circ_	0.440501	0.045044
hsa_circ_	0.400293	0.045051
hsa_circ_	-0.3673	0.045055
hsa_circ_	-0.59425	0.045071
hsa_circ_	-0.62024	0.045076
hsa_circ_	0.40434	0.045077
hsa_circ_	-0.38585	0.045079
hsa_circ_	-0.63007	0.04508
hsa_circ_	-0.37748	0.045084
hsa_circ_	-0.4112	0.045088
hsa_circ_	-0.35035	0.045102
hsa_circ_	0.634074	0.045129
hsa_circ_	0.420396	0.045133
hsa_circ_	0.39269	0.045134
hsa_circ_	-1.02038	0.045136
hsa_circ_	-0.33387	0.045136
hsa_circ_	-0.30484	0.045137
hsa_circ_	-0.61935	0.045142
hsa_circ_	1.201893	0.045154
hsa_circ_	-0.38514	0.045157
hsa_circ_	0.304831	0.045166
hsa_circ_	0.532027	0.045172
hsa_circ_	0.340616	0.045182
hsa_circ_	-0.61009	0.045205
hsa_circ_	-0.36457	0.045216
hsa_circ_	0.549869	0.045222
hsa_circ_	0.621128	0.045245
hsa_circ_	-0.98429	0.045246
hsa_circ_	-0.31142	0.045247
hsa_circ_	-0.83731	0.045254
hsa_circ_	0.36732	0.045262
hsa_circ_	0.881022	0.045268
hsa_circ_	0.804071	0.045287
hsa_circ_	0.345448	0.045288
hsa_circ_	0.993097	0.045295

hsa_circ_	-0.54644	0.045303
hsa_circ_	-0.33017	0.045318
hsa_circ_	0.417819	0.04533
hsa_circ_	-0.39059	0.045331
hsa_circ_	-0.68276	0.045332
hsa_circ_	0.794062	0.045346
hsa_circ_	0.323377	0.045348
hsa_circ_	-0.43128	0.045349
hsa_circ_	0.845784	0.045358
hsa_circ_	0.509067	0.045358
hsa_circ_	0.413208	0.045361
hsa_circ_	0.357523	0.045389
hsa_circ_	0.423685	0.045394
hsa_circ_	0.404231	0.045408
hsa_circ_	-0.4416	0.045422
hsa_circ_	-0.7465	0.045427
hsa_circ_	-0.44398	0.045427
hsa_circ_	-0.37515	0.045427
hsa_circ_	-0.55104	0.045428
hsa_circ_	-0.59135	0.045431
hsa_circ_	-0.35492	0.045431
hsa_circ_	-0.34194	0.045435
hsa_circ_	-0.36229	0.045447
hsa_circ_	0.707427	0.045461
hsa_circ_	-0.49388	0.045461
hsa_circ_	1.458315	0.045465
hsa_circ_	-0.35562	0.045475
hsa_circ_	-0.31233	0.045477
hsa_circ_	0.54561	0.045479
hsa_circ_	0.510173	0.045488
hsa_circ_	0.516893	0.045502
hsa_circ_	-0.34196	0.045512
hsa_circ_	-0.44948	0.045514
hsa_circ_	-0.39475	0.045514
hsa_circ_	-0.35592	0.045516
hsa_circ_	-0.47589	0.045519
hsa_circ_	0.623632	0.045522
hsa_circ_	-0.34822	0.045524
hsa_circ_	0.90348	0.045537
hsa_circ_	-0.45609	0.045541
hsa_circ_	-0.40656	0.045547
hsa_circ_	0.373298	0.045547
hsa_circ_	0.504829	0.04556
hsa_circ_	0.593042	0.045561
hsa_circ_	0.473151	0.045567
hsa_circ_	0.440808	0.04557
hsa_circ_	0.445854	0.045571
hsa_circ_	0.384105	0.045589
hsa_circ_	-0.92617	0.045593
hsa_circ_	0.507297	0.04562
hsa_circ_	0.327621	0.045624
hsa_circ_	0.336881	0.045627
hsa_circ_	-0.70262	0.045633
hsa_circ_	-0.41683	0.045639

hsa_circ_	-0.47725	0.045673
hsa_circ_	-0.55436	0.045679
hsa_circ_	0.400266	0.045685
hsa_circ_	-0.45335	0.045687
hsa_circ_	0.562451	0.045688
hsa_circ_	-0.4492	0.045693
hsa_circ_	-0.43836	0.045724
hsa_circ_	-0.44723	0.04573
hsa_circ_	0.521707	0.045738
hsa_circ_	0.369221	0.045742
hsa_circ_	0.42338	0.045748
hsa_circ_	0.581014	0.045748
hsa_circ_	-0.38497	0.045749
hsa_circ_	1.315337	0.045772
hsa_circ_	-0.42895	0.045772
hsa_circ_	-0.6126	0.045774
hsa_circ_	0.336536	0.045774
hsa_circ_	-0.45116	0.045776
hsa_circ_	0.395846	0.045777
hsa_circ_	0.400273	0.045779
hsa_circ_	-1.57729	0.045791
hsa_circ_	-0.51206	0.045792
hsa_circ_	-0.49114	0.045819
hsa_circ_	1.185047	0.045832
hsa_circ_	0.563022	0.045833
hsa_circ_	-0.89148	0.045846
hsa_circ_	0.797346	0.045849
hsa_circ_	-0.35495	0.045853
hsa_circ_	0.412636	0.045865
hsa_circ_	-0.33979	0.045867
hsa_circ_	0.305515	0.045873
hsa_circ_	0.901663	0.045876
hsa_circ_	-0.45742	0.045893
hsa_circ_	0.722171	0.045901
hsa_circ_	-0.32668	0.045906
hsa_circ_	0.502851	0.045914
hsa_circ_	0.5218	0.045935
hsa_circ_	-0.41436	0.045936
hsa_circ_	0.375267	0.045942
hsa_circ_	-0.53005	0.045945
hsa_circ_	-0.41207	0.045954
hsa_circ_	0.958088	0.045959
hsa_circ_	0.470104	0.045976
hsa_circ_	0.425935	0.04599
hsa_circ_	0.461587	0.045995
hsa_circ_	0.381018	0.046007
hsa_circ_	-0.69566	0.046021
hsa_circ_	-0.66709	0.046036
hsa_circ_	0.392385	0.04604
hsa_circ_	0.45744	0.046041
hsa_circ_	0.496175	0.046044
hsa_circ_	-0.75699	0.046051
hsa_circ_	-0.56147	0.046057
hsa_circ_	0.701089	0.046058

hsa_circ_	-0.36849	0.046069
hsa_circ_	0.332908	0.046077
hsa_circ_	-0.51639	0.04608
hsa_circ_	-0.36518	0.046089
hsa_circ_	0.723249	0.046101
hsa_circ_	-0.55568	0.046105
hsa_circ_	0.381605	0.046107
hsa_circ_	0.492996	0.046108
hsa_circ_	0.704477	0.04611
hsa_circ_	0.352198	0.04612
hsa_circ_	0.569632	0.04612
hsa_circ_	0.429378	0.046128
hsa_circ_	0.700454	0.046136
hsa_circ_	-0.62892	0.046141
hsa_circ_	-0.50623	0.046146
hsa_circ_	-0.35838	0.046148
hsa_circ_	-0.40722	0.046155
hsa_circ_	0.322722	0.046158
hsa_circ_	0.479406	0.046161
hsa_circ_	-0.30423	0.046168
hsa_circ_	-0.33498	0.046193
hsa_circ_	-0.47569	0.046198
hsa_circ_	-0.51098	0.046205
hsa_circ_	-0.3597	0.046207
hsa_circ_	0.98947	0.046213
hsa_circ_	0.813108	0.046217
hsa_circ_	-0.46802	0.046218
hsa_circ_	1.185457	0.046218
hsa_circ_	0.550813	0.046218
hsa_circ_	-0.45442	0.046221
hsa_circ_	0.788675	0.046222
hsa_circ_	0.530292	0.046224
hsa_circ_	0.688784	0.046229
hsa_circ_	0.453087	0.046239
hsa_circ_	0.768375	0.046241
hsa_circ_	1.024451	0.046244
hsa_circ_	0.403609	0.046245
hsa_circ_	0.551864	0.046253
hsa_circ_	-0.51636	0.046262
hsa_circ_	0.884257	0.046263
hsa_circ_	-0.4422	0.046267
hsa_circ_	0.596293	0.046269
hsa_circ_	-0.81992	0.04627
hsa_circ_	0.345778	0.046275
hsa_circ_	0.420183	0.046276
hsa_circ_	0.61295	0.046276
hsa_circ_	0.369997	0.046282
hsa_circ_	0.498081	0.046291
hsa_circ_	0.611252	0.046294
hsa_circ_	0.665716	0.0463
hsa_circ_	0.343656	0.046302
hsa_circ_	-0.34493	0.046305
hsa_circ_	-0.33927	0.04631
hsa_circ_	-0.52925	0.046318

hsa_circ_	0.902772	0.04632
hsa_circ_	-0.39782	0.046324
hsa_circ_	0.520728	0.046331
hsa_circ_	0.507624	0.046332
hsa_circ_	-0.43189	0.046334
hsa_circ_	0.549237	0.04634
hsa_circ_	0.409766	0.046348
hsa_circ_	0.833477	0.046351
hsa_circ_	-0.42676	0.046359
hsa_circ_	-1.76633	0.04636
hsa_circ_	-0.64989	0.046365
hsa_circ_	-0.50634	0.046368
hsa_circ_	0.45365	0.046376
hsa_circ_	0.892765	0.046387
hsa_circ_	-1.10906	0.046389
hsa_circ_	-0.60077	0.046397
hsa_circ_	0.452859	0.046404
hsa_circ_	-1.15887	0.046417
hsa_circ_	0.786599	0.046419
hsa_circ_	-0.44419	0.046429
hsa_circ_	-0.6272	0.046433
hsa_circ_	-0.30295	0.046435
hsa_circ_	0.726956	0.046436
hsa_circ_	-0.35377	0.046444
hsa_circ_	-0.49409	0.046448
hsa_circ_	0.401898	0.046454
hsa_circ_	0.38481	0.046454
hsa_circ_	-0.57327	0.046473
hsa_circ_	0.47896	0.046479
hsa_circ_	0.688934	0.046485
hsa_circ_	-0.33568	0.04649
hsa_circ_	0.474557	0.046499
hsa_circ_	-0.36899	0.046513
hsa_circ_	0.418481	0.046521
hsa_circ_	-0.36491	0.046528
hsa_circ_	0.351144	0.046529
hsa_circ_	0.841673	0.046536
hsa_circ_	-0.39466	0.046538
hsa_circ_	-0.4054	0.046557
hsa_circ_	0.736428	0.046564
hsa_circ_	0.715727	0.046567
hsa_circ_	0.979535	0.046568
hsa_circ_	0.637018	0.046568
hsa_circ_	-1.64034	0.046568
hsa_circ_	1.065582	0.046573
hsa_circ_	-0.42984	0.046575
hsa_circ_	0.507631	0.046586
hsa_circ_	-0.82552	0.046606
hsa_circ_	0.815671	0.046607
hsa_circ_	-0.53239	0.046608
hsa_circ_	-0.48908	0.046612
hsa_circ_	-0.63144	0.04664
hsa_circ_	1.405381	0.046643
hsa_circ_	-0.50693	0.046644

hsa_circ_	-1.13523	0.046648
hsa_circ_	0.485544	0.046651
hsa_circ_	-0.44675	0.046657
hsa_circ_	0.379754	0.046657
hsa_circ_	-0.45825	0.04666
hsa_circ_	0.562204	0.04666
hsa_circ_	-1.20697	0.046662
hsa_circ_	1.093736	0.046663
hsa_circ_	-0.30465	0.046675
hsa_circ_	0.582659	0.046681
hsa_circ_	0.383801	0.046688
hsa_circ_	-0.50465	0.046688
hsa_circ_	-0.74201	0.046698
hsa_circ_	0.401411	0.046705
hsa_circ_	0.571853	0.046712
hsa_circ_	1.027028	0.046716
hsa_circ_	-0.88565	0.046719
hsa_circ_	0.375381	0.04672
hsa_circ_	-0.5612	0.046724
hsa_circ_	-0.33928	0.04674
hsa_circ_	-0.37751	0.046742
hsa_circ_	-0.3202	0.046745
hsa_circ_	1.700081	0.046745
hsa_circ_	-1.20044	0.046745
hsa_circ_	0.307053	0.046757
hsa_circ_	0.300476	0.046763
hsa_circ_	0.801452	0.046765
hsa_circ_	-0.4199	0.046773
hsa_circ_	-0.37362	0.046774
hsa_circ_	-0.71626	0.0468
hsa_circ_	0.444226	0.046801
hsa_circ_	0.362038	0.046807
hsa_circ_	0.496396	0.046809
hsa_circ_	0.383375	0.046809
hsa_circ_	0.38991	0.04681
hsa_circ_	0.331124	0.046814
hsa_circ_	0.374063	0.046819
hsa_circ_	-0.72632	0.04682
hsa_circ_	-0.65753	0.04682
hsa_circ_	-0.70281	0.046842
hsa_circ_	-0.29881	0.046844
hsa_circ_	-0.70744	0.04685
hsa_circ_	-0.54858	0.046852
hsa_circ_	1.249195	0.046867
hsa_circ_	-0.51923	0.046867
hsa_circ_	0.671305	0.046871
hsa_circ_	-0.44178	0.046872
hsa_circ_	0.35329	0.046889
hsa_circ_	-0.29896	0.046895
hsa_circ_	0.417005	0.046898
hsa_circ_	-0.5344	0.046904
hsa_circ_	-0.70604	0.046906
hsa_circ_	0.333183	0.046908
hsa_circ_	0.504487	0.046911

hsa_circ_	-0.37991	0.046913
hsa_circ_	0.535121	0.046913
hsa_circ_	-0.65008	0.046914
hsa_circ_	0.478136	0.046918
hsa_circ_	-0.45609	0.046918
hsa_circ_	0.792583	0.046924
hsa_circ_	0.850174	0.046934
hsa_circ_	0.438549	0.046937
hsa_circ_	0.434526	0.046943
hsa_circ_	-0.57944	0.046944
hsa_circ_	0.848896	0.046948
hsa_circ_	0.804954	0.046949
hsa_circ_	-0.65477	0.046954
hsa_circ_	0.300037	0.046959
hsa_circ_	-0.59597	0.046973
hsa_circ_	-0.71512	0.046985
hsa_circ_	0.487316	0.046986
hsa_circ_	-0.55455	0.047005
hsa_circ_	0.628815	0.047007
hsa_circ_	-0.99153	0.047011
hsa_circ_	0.372369	0.047021
hsa_circ_	0.575162	0.047021
hsa_circ_	0.936863	0.047024
hsa_circ_	-0.37205	0.047028
hsa_circ_	0.453631	0.047033
hsa_circ_	0.556839	0.047035
hsa_circ_	0.479615	0.047043
hsa_circ_	0.437192	0.047046
hsa_circ_	-0.35788	0.04705
hsa_circ_	0.438655	0.047054
hsa_circ_	-0.82669	0.047054
hsa_circ_	0.636495	0.047084
hsa_circ_	-0.50017	0.04709
hsa_circ_	-0.35865	0.04709
hsa_circ_	0.415205	0.047091
hsa_circ_	1.169842	0.047093
hsa_circ_	-0.42975	0.047095
hsa_circ_	0.44936	0.0471
hsa_circ_	0.481252	0.0471
hsa_circ_	-0.80862	0.047101
hsa_circ_	1.266165	0.047101
hsa_circ_	-0.39208	0.047138
hsa_circ_	-0.88717	0.047139
hsa_circ_	-0.36078	0.047141
hsa_circ_	0.6827	0.047158
hsa_circ_	-0.46512	0.047158
hsa_circ_	-0.74788	0.047158
hsa_circ_	-0.38692	0.047167
hsa_circ_	0.821037	0.047173
hsa_circ_	0.383521	0.047176
hsa_circ_	0.321328	0.047188
hsa_circ_	0.325355	0.047205
hsa_circ_	0.600121	0.047207
hsa_circ_	-0.30255	0.047225

hsa_circ_	0.57295	0.047233
hsa_circ_	-1.13146	0.047238
hsa_circ_	0.454463	0.047245
hsa_circ_	-0.91421	0.047265
hsa_circ_	0.52043	0.047267
hsa_circ_	0.484298	0.047269
hsa_circ_	0.751355	0.047281
hsa_circ_	-0.62564	0.047299
hsa_circ_	0.50218	0.047301
hsa_circ_	-0.30043	0.047307
hsa_circ_	-0.31532	0.047309
hsa_circ_	-0.60381	0.047313
hsa_circ_	0.324267	0.047322
hsa_circ_	0.659199	0.047331
hsa_circ_	0.447279	0.047339
hsa_circ_	0.492656	0.047366
hsa_circ_	-0.79095	0.04737
hsa_circ_	-0.43445	0.047378
hsa_circ_	0.566298	0.047384
hsa_circ_	-0.56172	0.047385
hsa_circ_	0.555318	0.047391
hsa_circ_	0.456363	0.047391
hsa_circ_	-0.3412	0.047391
hsa_circ_	0.633219	0.047401
hsa_circ_	0.872284	0.047404
hsa_circ_	1.096424	0.047412
hsa_circ_	0.529818	0.047426
hsa_circ_	0.730037	0.047448
hsa_circ_	0.506047	0.047449
hsa_circ_	0.344191	0.047464
hsa_circ_	0.401369	0.047466
hsa_circ_	-0.52941	0.047478
hsa_circ_	0.788725	0.047481
hsa_circ_	-0.96782	0.047492
hsa_circ_	-0.35406	0.047496
hsa_circ_	-0.41427	0.0475
hsa_circ_	0.666325	0.047502
hsa_circ_	0.445805	0.047503
hsa_circ_	-0.30151	0.047504
hsa_circ_	0.563352	0.047514
hsa_circ_	0.572577	0.047518
hsa_circ_	-0.42086	0.047527
hsa_circ_	-0.35112	0.047528
hsa_circ_	0.347434	0.047535
hsa_circ_	-0.56979	0.047542
hsa_circ_	0.456963	0.047566
hsa_circ_	-0.46026	0.047567
hsa_circ_	-0.32875	0.047586
hsa_circ_	0.400166	0.047598
hsa_circ_	-0.29519	0.047608
hsa_circ_	-0.67289	0.047608
hsa_circ_	0.610614	0.047615
hsa_circ_	-0.58279	0.047616
hsa_circ_	0.373077	0.047619

hsa_circ_	-0.32613	0.047619
hsa_circ_	0.658419	0.047631
hsa_circ_	-0.40842	0.047637
hsa_circ_	-1.42698	0.04764
hsa_circ_	-0.32498	0.047646
hsa_circ_	0.403547	0.047655
hsa_circ_	0.595229	0.047659
hsa_circ_	-0.86955	0.047667
hsa_circ_	0.433039	0.047671
hsa_circ_	-0.48443	0.047673
hsa_circ_	0.449353	0.047684
hsa_circ_	-0.34801	0.047688
hsa_circ_	0.363488	0.04769
hsa_circ_	-1.1371	0.04769
hsa_circ_	1.121321	0.047691
hsa_circ_	0.47885	0.047696
hsa_circ_	0.311004	0.047699
hsa_circ_	-0.3968	0.047718
hsa_circ_	-0.79079	0.047718
hsa_circ_	-0.37359	0.047721
hsa_circ_	0.410475	0.047723
hsa_circ_	-0.44839	0.047723
hsa_circ_	0.537188	0.047726
hsa_circ_	-0.44569	0.047737
hsa_circ_	-2.3333	0.04774
hsa_circ_	1.190242	0.047746
hsa_circ_	0.346203	0.047748
hsa_circ_	0.502966	0.047749
hsa_circ_	-0.41442	0.047754
hsa_circ_	-0.52004	0.047756
hsa_circ_	-0.36063	0.047764
hsa_circ_	-0.36497	0.04778
hsa_circ_	1.363035	0.047786
hsa_circ_	0.326705	0.047789
hsa_circ_	0.360702	0.047793
hsa_circ_	0.449645	0.047801
hsa_circ_	-0.59181	0.047803
hsa_circ_	1.207603	0.047803
hsa_circ_	-0.74988	0.047812
hsa_circ_	1.177481	0.047818
hsa_circ_	-0.49449	0.04782
hsa_circ_	0.591657	0.047847
hsa_circ_	0.898373	0.047855
hsa_circ_	1.137146	0.047856
hsa_circ_	-0.59373	0.047864
hsa_circ_	-0.34441	0.047871
hsa_circ_	-0.52942	0.047876
hsa_circ_	-0.32295	0.047882
hsa_circ_	-0.64927	0.047885
hsa_circ_	0.812205	0.047886
hsa_circ_	0.93486	0.047889
hsa_circ_	-0.44001	0.047895
hsa_circ_	-0.38569	0.047896
hsa_circ_	-0.44476	0.047897

hsa_circ_	-0.88138	0.047905
hsa_circ_	-1.5937	0.047909
hsa_circ_	0.347231	0.047913
hsa_circ_	0.466515	0.047931
hsa_circ_	-0.63601	0.047938
hsa_circ_	0.553253	0.047952
hsa_circ_	1.512547	0.047954
hsa_circ_	-0.47967	0.047967
hsa_circ_	-0.52597	0.047977
hsa_circ_	0.341348	0.047992
hsa_circ_	-0.31177	0.048001
hsa_circ_	-0.70112	0.048013
hsa_circ_	0.634554	0.048015
hsa_circ_	0.483753	0.048019
hsa_circ_	-0.36572	0.048028
hsa_circ_	-0.49611	0.048034
hsa_circ_	0.566777	0.048036
hsa_circ_	0.540322	0.048041
hsa_circ_	-0.57143	0.048052
hsa_circ_	-0.35261	0.048054
hsa_circ_	0.435955	0.048055
hsa_circ_	1.052122	0.048057
hsa_circ_	-0.51289	0.048058
hsa_circ_	-0.52167	0.048063
hsa_circ_	0.866519	0.048064
hsa_circ_	0.675122	0.048066
hsa_circ_	-0.3273	0.04807
hsa_circ_	-0.35949	0.048076
hsa_circ_	0.373395	0.048079
hsa_circ_	0.366871	0.048089
hsa_circ_	0.499329	0.04809
hsa_circ_	0.443909	0.0481
hsa_circ_	0.455711	0.048102
hsa_circ_	0.425277	0.048131
hsa_circ_	0.81609	0.048133
hsa_circ_	0.678929	0.048135
hsa_circ_	-0.34334	0.048152
hsa_circ_	-0.40953	0.048159
hsa_circ_	1.485513	0.04817
hsa_circ_	0.486229	0.048172
hsa_circ_	0.359422	0.048176
hsa_circ_	-0.41899	0.048181
hsa_circ_	1.188163	0.048187
hsa_circ_	0.406085	0.048191
hsa_circ_	1.30371	0.048197
hsa_circ_	0.544586	0.048199
hsa_circ_	1.178447	0.048202
hsa_circ_	-0.76054	0.048205
hsa_circ_	1.572438	0.048206
hsa_circ_	-0.33887	0.048209
hsa_circ_	-1.08889	0.04821
hsa_circ_	0.54569	0.048212
hsa_circ_	0.363484	0.048213
hsa_circ_	0.723143	0.048221

hsa_circ_	0.501346	0.048227
hsa_circ_	0.451985	0.048227
hsa_circ_	0.85974	0.04823
hsa_circ_	-0.56956	0.048238
hsa_circ_	1.098245	0.048239
hsa_circ_	-0.55786	0.048254
hsa_circ_	-0.52127	0.048263
hsa_circ_	-0.86817	0.048268
hsa_circ_	-0.43183	0.048276
hsa_circ_	-0.47866	0.048287
hsa_circ_	0.430428	0.04829
hsa_circ_	-0.54436	0.048307
hsa_circ_	-0.35034	0.04831
hsa_circ_	-0.33277	0.048313
hsa_circ_	-0.3441	0.048319
hsa_circ_	-0.33797	0.048327
hsa_circ_	-0.56961	0.048329
hsa_circ_	-0.35281	0.048347
hsa_circ_	1.057907	0.048348
hsa_circ_	-0.81058	0.048357
hsa_circ_	-0.56193	0.048363
hsa_circ_	-0.41137	0.048363
hsa_circ_	-0.34823	0.048369
hsa_circ_	0.66901	0.048372
hsa_circ_	0.54913	0.048375
hsa_circ_	-0.45037	0.048376
hsa_circ_	-0.41037	0.048377
hsa_circ_	-0.42902	0.048383
hsa_circ_	-0.60693	0.048388
hsa_circ_	-0.4624	0.04839
hsa_circ_	-0.36992	0.048396
hsa_circ_	-0.40932	0.048398
hsa_circ_	-0.37106	0.048402
hsa_circ_	0.423412	0.048405
hsa_circ_	-0.55623	0.048406
hsa_circ_	0.799892	0.048413
hsa_circ_	0.609682	0.048415
hsa_circ_	-0.35083	0.048421
hsa_circ_	0.494057	0.048422
hsa_circ_	-0.36699	0.048433
hsa_circ_	1.053033	0.048433
hsa_circ_	-0.43611	0.048434
hsa_circ_	-0.52287	0.048447
hsa_circ_	0.452	0.048447
hsa_circ_	-0.3014	0.048453
hsa_circ_	-0.43106	0.048455
hsa_circ_	-0.34145	0.048463
hsa_circ_	-0.37515	0.048466
hsa_circ_	0.720907	0.048491
hsa_circ_	0.489614	0.048498
hsa_circ_	0.474624	0.048502
hsa_circ_	-0.46732	0.048503
hsa_circ_	-0.60188	0.048508
hsa_circ_	0.340062	0.04851

hsa_circ_	0.977934	0.048528
hsa_circ_	0.556191	0.048529
hsa_circ_	0.548559	0.04854
hsa_circ_	0.649083	0.04854
hsa_circ_	-0.42629	0.04854
hsa_circ_	-0.64123	0.048541
hsa_circ_	-0.37785	0.048553
hsa_circ_	-0.39305	0.048554
hsa_circ_	0.389241	0.048556
hsa_circ_	-0.51854	0.048558
hsa_circ_	-1.82843	0.048559
hsa_circ_	-0.40864	0.048566
hsa_circ_	0.670684	0.048569
hsa_circ_	-0.31705	0.048571
hsa_circ_	-0.36676	0.048573
hsa_circ_	0.864106	0.048573
hsa_circ_	0.591596	0.048582
hsa_circ_	0.943077	0.048584
hsa_circ_	0.396806	0.048585
hsa_circ_	0.854062	0.048586
hsa_circ_	1.008607	0.048591
hsa_circ_	-0.35505	0.048597
hsa_circ_	0.449429	0.048611
hsa_circ_	-0.40343	0.048613
hsa_circ_	-0.88392	0.048619
hsa_circ_	-0.39605	0.048624
hsa_circ_	-0.37049	0.04863
hsa_circ_	-0.32125	0.048647
hsa_circ_	-0.73127	0.04867
hsa_circ_	0.399591	0.048677
hsa_circ_	-0.30767	0.048678
hsa_circ_	1.061954	0.048679
hsa_circ_	0.498086	0.048681
hsa_circ_	0.471938	0.048682
hsa_circ_	0.649673	0.048687
hsa_circ_	0.523191	0.04869
hsa_circ_	-0.3151	0.048692
hsa_circ_	0.465231	0.048693
hsa_circ_	-0.35778	0.048697
hsa_circ_	1.180595	0.048701
hsa_circ_	0.327733	0.048715
hsa_circ_	0.460574	0.048728
hsa_circ_	0.676379	0.04873
hsa_circ_	0.456386	0.048736
hsa_circ_	-0.34809	0.048746
hsa_circ_	-0.65208	0.048763
hsa_circ_	-1.72854	0.048771
hsa_circ_	0.50714	0.048775
hsa_circ_	-0.75863	0.048785
hsa_circ_	0.443281	0.04879
hsa_circ_	0.943149	0.048796
hsa_circ_	0.951374	0.048818
hsa_circ_	-0.37316	0.048829
hsa_circ_	0.342051	0.048838

hsa_circ_	-0.61204	0.048852
hsa_circ_	0.960442	0.048856
hsa_circ_	1.112527	0.048878
hsa_circ_	-0.3323	0.04889
hsa_circ_	0.441149	0.048893
hsa_circ_	0.388832	0.048902
hsa_circ_	-0.30614	0.048903
hsa_circ_	0.443402	0.048916
hsa_circ_	-0.42369	0.048937
hsa_circ_	0.438887	0.04894
hsa_circ_	0.909943	0.04894
hsa_circ_	-0.84163	0.048944
hsa_circ_	0.671493	0.048951
hsa_circ_	-0.54628	0.048958
hsa_circ_	0.570408	0.048963
hsa_circ_	-0.98441	0.048995
hsa_circ_	0.715883	0.049002
hsa_circ_	1.308276	0.049004
hsa_circ_	-0.52295	0.049011
hsa_circ_	0.370381	0.049012
hsa_circ_	-0.39903	0.049012
hsa_circ_	-0.54665	0.049015
hsa_circ_	-1.33338	0.049018
hsa_circ_	0.788249	0.049026
hsa_circ_	-0.37846	0.049027
hsa_circ_	-0.43719	0.049031
hsa_circ_	-0.30358	0.04905
hsa_circ_	-0.68301	0.04905
hsa_circ_	-0.34816	0.049055
hsa_circ_	0.666075	0.049055
hsa_circ_	-0.39319	0.049061
hsa_circ_	0.721062	0.049065
hsa_circ_	-1.31318	0.049079
hsa_circ_	0.705239	0.049079
hsa_circ_	0.349027	0.04908
hsa_circ_	-0.38916	0.049083
hsa_circ_	-0.90082	0.049104
hsa_circ_	-0.32057	0.049114
hsa_circ_	0.622865	0.049118
hsa_circ_	0.800953	0.049123
hsa_circ_	0.352974	0.049123
hsa_circ_	0.934381	0.049133
hsa_circ_	0.412669	0.049133
hsa_circ_	0.624459	0.04914
hsa_circ_	0.507129	0.049149
hsa_circ_	-0.43043	0.049154
hsa_circ_	0.374207	0.049155
hsa_circ_	0.331881	0.049158
hsa_circ_	-0.31636	0.049158
hsa_circ_	-0.44438	0.049159
hsa_circ_	0.513584	0.049166
hsa_circ_	-0.32085	0.049171
hsa_circ_	1.128648	0.049174
hsa_circ_	0.38876	0.049175

hsa_circ_	1.261402	0.049175
hsa_circ_	-0.49456	0.04918
hsa_circ_	-0.46857	0.04918
hsa_circ_	-0.89245	0.049188
hsa_circ_	0.421259	0.04919
hsa_circ_	0.353431	0.049197
hsa_circ_	-1.41942	0.049211
hsa_circ_	0.61066	0.049212
hsa_circ_	0.427587	0.049214
hsa_circ_	0.760647	0.049214
hsa_circ_	-0.33865	0.049219
hsa_circ_	0.804493	0.04922
hsa_circ_	0.652535	0.049224
hsa_circ_	0.597537	0.049227
hsa_circ_	-0.56433	0.04923
hsa_circ_	-0.50031	0.049236
hsa_circ_	0.432779	0.049245
hsa_circ_	-0.33396	0.049253
hsa_circ_	-0.41051	0.049263
hsa_circ_	0.353655	0.049266
hsa_circ_	-0.47957	0.049277
hsa_circ_	-0.79296	0.049285
hsa_circ_	-0.45461	0.049286
hsa_circ_	-0.44137	0.04929
hsa_circ_	-0.42791	0.049296
hsa_circ_	0.440361	0.049304
hsa_circ_	-0.4876	0.049307
hsa_circ_	0.704681	0.049307
hsa_circ_	1.117461	0.04931
hsa_circ_	0.545878	0.049313
hsa_circ_	-0.33352	0.049318
hsa_circ_	0.627992	0.049331
hsa_circ_	-0.76612	0.049332
hsa_circ_	0.603935	0.049335
hsa_circ_	0.690857	0.049336
hsa_circ_	-0.48194	0.049338
hsa_circ_	-0.66521	0.049353
hsa_circ_	-0.44983	0.049372
hsa_circ_	-0.67247	0.049375
hsa_circ_	1.155743	0.049379
hsa_circ_	0.962408	0.049392
hsa_circ_	-0.36725	0.049398
hsa_circ_	0.675446	0.049405
hsa_circ_	0.662686	0.049406
hsa_circ_	0.306048	0.049409
hsa_circ_	0.397622	0.049424
hsa_circ_	0.349548	0.049424
hsa_circ_	0.720786	0.04943
hsa_circ_	0.405984	0.04943
hsa_circ_	-0.39267	0.049436
hsa_circ_	-1.00071	0.049439
hsa_circ_	0.481602	0.049442
hsa_circ_	-0.45937	0.049451
hsa_circ_	0.363718	0.049454

hsa_circ_	0.381677	0.049456
hsa_circ_	-0.49495	0.04947
hsa_circ_	0.343492	0.049479
hsa_circ_	-0.36602	0.049484
hsa_circ_	0.687754	0.049484
hsa_circ_	-0.83482	0.049487
hsa_circ_	0.95941	0.049498
hsa_circ_	-0.57917	0.049507
hsa_circ_	0.458974	0.049508
hsa_circ_	-0.61188	0.049512
hsa_circ_	-0.76074	0.049513
hsa_circ_	0.505126	0.049516
hsa_circ_	0.413635	0.049518
hsa_circ_	-0.36459	0.04952
hsa_circ_	-0.3787	0.049521
hsa_circ_	-0.99622	0.049524
hsa_circ_	-0.66603	0.04953
hsa_circ_	0.401557	0.049531
hsa_circ_	-0.61426	0.049535
hsa_circ_	-0.32748	0.049553
hsa_circ_	1.064694	0.049581
hsa_circ_	-1.14902	0.049589
hsa_circ_	0.538925	0.049591
hsa_circ_	0.32618	0.049591
hsa_circ_	0.736117	0.049592
hsa_circ_	0.31568	0.049603
hsa_circ_	0.856034	0.049604
hsa_circ_	-0.35308	0.04961
hsa_circ_	-0.3301	0.049617
hsa_circ_	-0.50993	0.049625
hsa_circ_	0.423431	0.049628
hsa_circ_	-0.32716	0.049632
hsa_circ_	-0.51205	0.049633
hsa_circ_	-0.72225	0.049636
hsa_circ_	-1.1163	0.049636
hsa_circ_	-0.32269	0.049649
hsa_circ_	-0.39138	0.049652
hsa_circ_	0.552677	0.049654
hsa_circ_	-0.89384	0.049655
hsa_circ_	0.40811	0.049665
hsa_circ_	-1.58249	0.049666
hsa_circ_	-0.54786	0.049672
hsa_circ_	1.135827	0.049674
hsa_circ_	-0.64804	0.049682
hsa_circ_	-0.58351	0.049687
hsa_circ_	-0.59725	0.049704
hsa_circ_	0.327178	0.049716
hsa_circ_	-0.53005	0.049746
hsa_circ_	-0.51758	0.049747
hsa_circ_	-0.40477	0.049749
hsa_circ_	0.435346	0.049758
hsa_circ_	0.721041	0.04976
hsa_circ_	0.48583	0.049769
hsa_circ_	-0.48199	0.049773

hsa_circ_	-0.9263	0.049774
hsa_circ_	0.607314	0.049783
hsa_circ_	0.452367	0.049795
hsa_circ_	0.465629	0.04981
hsa_circ_	-0.44679	0.049825
hsa_circ_	-0.48833	0.049828
hsa_circ_	0.555579	0.049835
hsa_circ_	-0.96676	0.049835
hsa_circ_	0.349903	0.049841
hsa_circ_	0.414534	0.049849
hsa_circ_	0.68037	0.049852
hsa_circ_	-0.45471	0.049854
hsa_circ_	0.448439	0.049875
hsa_circ_	-0.53164	0.049875
hsa_circ_	0.454449	0.049878
hsa_circ_	0.519733	0.04988
hsa_circ_	0.385981	0.049892
hsa_circ_	0.383149	0.049892
hsa_circ_	-0.50822	0.049896
hsa_circ_	0.403349	0.049906
hsa_circ_	0.621673	0.049906
hsa_circ_	0.426416	0.049915
hsa_circ_	-0.47584	0.049934
hsa_circ_	0.502441	0.049936
hsa_circ_	0.610586	0.049937
hsa_circ_	0.950559	0.04994
hsa_circ_	-0.85092	0.049943
hsa_circ_	-1.38582	0.049951
hsa_circ_	-0.54324	0.049962
hsa_circ_	-0.44017	0.049963
hsa_circ_	-0.32827	0.049964
hsa_circ_	0.383012	0.049965
hsa_circ_	-0.35592	0.049986
hsa_circ_	0.352916	0.04999
hsa_circ_	-0.44273	0.049997
hsa_circ_	-0.42063	0.049998
hsa_circ_	-0.33055	0.050005
hsa_circ_	0.412064	0.050009
hsa_circ_	0.509703	0.050028
hsa_circ_	-0.342	0.050041
hsa_circ_	0.341314	0.050046
hsa_circ_	-0.85167	0.05005
hsa_circ_	0.480852	0.05005
hsa_circ_	0.374803	0.050065
hsa_circ_	-0.5591	0.050068
hsa_circ_	-1.68991	0.050069
hsa_circ_	-0.57124	0.050071
hsa_circ_	-0.38333	0.050073
hsa_circ_	-0.64293	0.050076
hsa_circ_	0.730909	0.050079
hsa_circ_	-0.3878	0.050086
hsa_circ_	0.365809	0.050099
hsa_circ_	0.885063	0.050102
hsa_circ_	1.153925	0.050102

hsa_circ_	0.370845	0.050103
hsa_circ_	0.490369	0.050104
hsa_circ_	-0.53008	0.050111
hsa_circ_	-0.44256	0.050111
hsa_circ_	-0.32914	0.050113
hsa_circ_	0.422589	0.050115
hsa_circ_	0.534619	0.050131
hsa_circ_	-1.14124	0.050137
hsa_circ_	-0.35112	0.050141
hsa_circ_	0.707929	0.050144
hsa_circ_	-0.47973	0.050147
hsa_circ_	-0.82269	0.05015
hsa_circ_	0.398466	0.050152
hsa_circ_	1.884659	0.05016
hsa_circ_	-0.64027	0.050163
hsa_circ_	0.300985	0.050168
hsa_circ_	-0.45861	0.050178
hsa_circ_	-0.60253	0.050181
hsa_circ_	0.710624	0.050182
hsa_circ_	-0.43545	0.050193
hsa_circ_	0.346718	0.050204
hsa_circ_	0.420637	0.050206
hsa_circ_	-0.41473	0.05021
hsa_circ_	1.223017	0.050212
hsa_circ_	-0.53165	0.050223
hsa_circ_	-0.9069	0.050225
hsa_circ_	-0.43226	0.050228
hsa_circ_	-0.45483	0.050237
hsa_circ_	0.506097	0.050244
hsa_circ_	-0.80482	0.050249
hsa_circ_	-0.43004	0.050258
hsa_circ_	0.456897	0.050259
hsa_circ_	-0.48906	0.050263
hsa_circ_	0.721948	0.050268
hsa_circ_	0.731732	0.050279
hsa_circ_	0.331851	0.050283
hsa_circ_	0.365771	0.050284
hsa_circ_	0.814522	0.050296
hsa_circ_	-0.33604	0.050311
hsa_circ_	-0.43009	0.050317
hsa_circ_	-0.68212	0.050325
hsa_circ_	-0.47833	0.050328
hsa_circ_	0.33985	0.050337
hsa_circ_	-0.41948	0.05034
hsa_circ_	-0.33329	0.050341
hsa_circ_	-0.43334	0.050347
hsa_circ_	0.585411	0.050354
hsa_circ_	-0.54619	0.05036
hsa_circ_	0.457622	0.05037
hsa_circ_	0.386949	0.050373
hsa_circ_	-0.47304	0.050385
hsa_circ_	-0.40124	0.050395
hsa_circ_	-0.48484	0.050395
hsa_circ_	0.548408	0.050397

hsa_circ_	0.380516	0.050402
hsa_circ_	-1.42496	0.050402
hsa_circ_	-0.37349	0.050417
hsa_circ_	-0.93378	0.050421
hsa_circ_	0.448365	0.050425
hsa_circ_	0.346424	0.050436
hsa_circ_	-0.39784	0.050441
hsa_circ_	-0.69361	0.050444
hsa_circ_	0.430284	0.050474
hsa_circ_	0.566895	0.050475
hsa_circ_	0.468559	0.050487
hsa_circ_	0.702311	0.050494
hsa_circ_	-0.38935	0.050502
hsa_circ_	-0.55643	0.050516
hsa_circ_	-0.37826	0.050526
hsa_circ_	-1.62422	0.05053
hsa_circ_	-0.29309	0.050531
hsa_circ_	0.538866	0.050537
hsa_circ_	-0.81992	0.05054
hsa_circ_	0.439602	0.050541
hsa_circ_	-0.3506	0.050548
hsa_circ_	0.560392	0.050551
hsa_circ_	0.538478	0.050564
hsa_circ_	1.451739	0.050568
hsa_circ_	0.584044	0.050585
hsa_circ_	-1.03918	0.050587
hsa_circ_	0.575354	0.05059
hsa_circ_	-0.59313	0.050594
hsa_circ_	-0.89357	0.050595
hsa_circ_	-0.43873	0.050597
hsa_circ_	-0.35787	0.050607
hsa_circ_	-0.53307	0.050608
hsa_circ_	0.430488	0.05061
hsa_circ_	0.46578	0.050611
hsa_circ_	-0.30178	0.050612
hsa_circ_	0.672333	0.05063
hsa_circ_	-0.46746	0.050637
hsa_circ_	-0.68398	0.05065
hsa_circ_	-0.8956	0.050654
hsa_circ_	1.478643	0.05066
hsa_circ_	0.9657	0.050673
hsa_circ_	0.526906	0.050678
hsa_circ_	-0.4067	0.050708
hsa_circ_	0.476172	0.050712
hsa_circ_	0.960042	0.050712
hsa_circ_	0.508911	0.050721
hsa_circ_	-0.85406	0.050725
hsa_circ_	0.359315	0.050726
hsa_circ_	0.605089	0.050728
hsa_circ_	0.485901	0.050728
hsa_circ_	-0.33597	0.050732
hsa_circ_	-0.35878	0.050739
hsa_circ_	-0.30806	0.050741
hsa_circ_	-0.39992	0.050742

hsa_circ_	-0.42228	0.050744
hsa_circ_	0.761881	0.050752
hsa_circ_	-0.33523	0.050756
hsa_circ_	0.386357	0.050761
hsa_circ_	0.34686	0.050762
hsa_circ_	0.686168	0.050765
hsa_circ_	0.420849	0.050766
hsa_circ_	0.539814	0.050767
hsa_circ_	0.352646	0.050781
hsa_circ_	-0.30878	0.050783
hsa_circ_	-0.31335	0.050789
hsa_circ_	1.152125	0.050792
hsa_circ_	0.504249	0.05082
hsa_circ_	0.438289	0.050833
hsa_circ_	-0.84658	0.050833
hsa_circ_	-0.41759	0.050833
hsa_circ_	0.469682	0.050851
hsa_circ_	0.337658	0.050861
hsa_circ_	0.589114	0.050867
hsa_circ_	-1.6312	0.050869
hsa_circ_	-1.43195	0.05087
hsa_circ_	1.481344	0.050873
hsa_circ_	-0.41226	0.050873
hsa_circ_	-0.43116	0.050874
hsa_circ_	0.801592	0.050878
hsa_circ_	-1.89955	0.050891
hsa_circ_	0.406442	0.050896
hsa_circ_	0.326332	0.050906
hsa_circ_	0.428071	0.050923
hsa_circ_	-0.30558	0.05093
hsa_circ_	-0.54805	0.050941
hsa_circ_	0.429597	0.050963
hsa_circ_	0.484779	0.050964
hsa_circ_	-0.34088	0.050965
hsa_circ_	0.454754	0.050972
hsa_circ_	-1.65717	0.050979
hsa_circ_	0.525559	0.050981
hsa_circ_	0.434098	0.050985
hsa_circ_	0.374663	0.050993
hsa_circ_	1.262116	0.050996
hsa_circ_	-0.83064	0.050997
hsa_circ_	-0.32973	0.051006
hsa_circ_	-0.44077	0.051008
hsa_circ_	-0.36617	0.05101
hsa_circ_	-0.77798	0.051022
hsa_circ_	-0.33307	0.051024
hsa_circ_	0.773533	0.05103
hsa_circ_	0.538931	0.051037
hsa_circ_	-0.40878	0.051053
hsa_circ_	0.338055	0.051054
hsa_circ_	-0.54549	0.051057
hsa_circ_	0.817095	0.051062
hsa_circ_	-0.45893	0.051062
hsa_circ_	0.456057	0.051062

hsa_circ_	0.536195	0.051063
hsa_circ_	0.500285	0.051069
hsa_circ_	1.29659	0.05107
hsa_circ_	-0.8773	0.051079
hsa_circ_	-0.39953	0.051089
hsa_circ_	1.442899	0.051094
hsa_circ_	0.478843	0.051105
hsa_circ_	0.870541	0.051111
hsa_circ_	-0.52512	0.051123
hsa_circ_	-0.39025	0.051131
hsa_circ_	0.379232	0.051141
hsa_circ_	-0.57642	0.051143
hsa_circ_	-0.83417	0.051145
hsa_circ_	-0.30708	0.051146
hsa_circ_	-0.4912	0.051149
hsa_circ_	0.944436	0.05115
hsa_circ_	0.465688	0.051154
hsa_circ_	-0.50533	0.051157
hsa_circ_	-0.34113	0.051165
hsa_circ_	0.352336	0.051176
hsa_circ_	-0.97671	0.051184
hsa_circ_	-0.47835	0.051185
hsa_circ_	0.855126	0.0512
hsa_circ_	0.547528	0.051202
hsa_circ_	0.597946	0.051204
hsa_circ_	0.395236	0.051216
hsa_circ_	-0.79459	0.051217
hsa_circ_	0.358619	0.051218
hsa_circ_	0.506996	0.051235
hsa_circ_	-0.43189	0.051243
hsa_circ_	0.478515	0.051244
hsa_circ_	0.377244	0.051245
hsa_circ_	0.457923	0.051251
hsa_circ_	1.365491	0.051251
hsa_circ_	-1.40653	0.051252
hsa_circ_	-0.36907	0.051253
hsa_circ_	0.745563	0.051256
hsa_circ_	-1.4321	0.051263
hsa_circ_	0.433451	0.05127
hsa_circ_	0.977149	0.051271
hsa_circ_	-0.32336	0.051272
hsa_circ_	0.499917	0.051279
hsa_circ_	0.648998	0.051285
hsa_circ_	-1.8505	0.051285
hsa_circ_	0.838533	0.051291
hsa_circ_	-0.35368	0.051292
hsa_circ_	-0.38178	0.051312
hsa_circ_	-0.46802	0.051313
hsa_circ_	-0.31526	0.051313
hsa_circ_	-0.94966	0.051316
hsa_circ_	-0.37664	0.051319
hsa_circ_	-0.39083	0.05132
hsa_circ_	-1.11683	0.051321
hsa_circ_	-0.40356	0.051335

hsa_circ_	-0.48125	0.051342
hsa_circ_	0.346624	0.051346
hsa_circ_	0.532471	0.051352
hsa_circ_	0.505435	0.051353
hsa_circ_	0.309912	0.05136
hsa_circ_	0.893657	0.05136
hsa_circ_	0.359424	0.051367
hsa_circ_	0.731105	0.051369
hsa_circ_	0.725999	0.051372
hsa_circ_	-1.52759	0.051373
hsa_circ_	-1.2381	0.051379
hsa_circ_	-1.06219	0.051382
hsa_circ_	0.430222	0.051397
hsa_circ_	0.586366	0.051405
hsa_circ_	0.39003	0.051432
hsa_circ_	-0.36362	0.051449
hsa_circ_	0.464654	0.051452
hsa_circ_	-0.35939	0.051458
hsa_circ_	-0.9536	0.051478
hsa_circ_	-0.40973	0.051485
hsa_circ_	0.532966	0.051489
hsa_circ_	0.325633	0.05149
hsa_circ_	0.300948	0.051491
hsa_circ_	-1.25425	0.051503
hsa_circ_	0.352326	0.051536
hsa_circ_	-0.69404	0.051536
hsa_circ_	-0.38225	0.051541
hsa_circ_	-0.38839	0.051544
hsa_circ_	0.334668	0.051547
hsa_circ_	-0.34889	0.051548
hsa_circ_	0.557231	0.051548
hsa_circ_	-0.90879	0.051552
hsa_circ_	0.471841	0.051563
hsa_circ_	0.454615	0.051568
hsa_circ_	0.69696	0.051573
hsa_circ_	1.01888	0.051574
hsa_circ_	0.492247	0.051576
hsa_circ_	0.438054	0.051578
hsa_circ_	-0.35917	0.051582
hsa_circ_	0.374491	0.051589
hsa_circ_	0.879034	0.051593
hsa_circ_	0.999236	0.051596
hsa_circ_	0.574504	0.051602
hsa_circ_	-0.57796	0.051622
hsa_circ_	-0.36865	0.051625
hsa_circ_	-0.34171	0.051628
hsa_circ_	0.790107	0.051631
hsa_circ_	-0.36177	0.051637
hsa_circ_	-0.40797	0.051647
hsa_circ_	0.752567	0.051653
hsa_circ_	-0.39263	0.05166
hsa_circ_	-0.30877	0.051673
hsa_circ_	-0.60202	0.0517
hsa_circ_	0.504338	0.05171

hsa_circ_	0.342186	0.051711
hsa_circ_	-1.06301	0.051712
hsa_circ_	0.336722	0.051714
hsa_circ_	-0.45614	0.051719
hsa_circ_	0.650614	0.051726
hsa_circ_	0.316289	0.051734
hsa_circ_	-0.52629	0.051739
hsa_circ_	-0.70822	0.051751
hsa_circ_	-0.51486	0.051753
hsa_circ_	-0.49017	0.051754
hsa_circ_	-0.62075	0.051762
hsa_circ_	0.39082	0.051766
hsa_circ_	-1.50412	0.051773
hsa_circ_	0.548232	0.051776
hsa_circ_	-0.32484	0.051777
hsa_circ_	1.82034	0.051785
hsa_circ_	0.489714	0.051787
hsa_circ_	0.496454	0.05179
hsa_circ_	-0.78073	0.05179
hsa_circ_	-1.11353	0.051793
hsa_circ_	-0.83361	0.051794
hsa_circ_	0.480759	0.051801
hsa_circ_	0.324393	0.051804
hsa_circ_	-0.46485	0.051814
hsa_circ_	-0.3344	0.051816
hsa_circ_	-0.36255	0.051817
hsa_circ_	-0.84455	0.051825
hsa_circ_	-0.41738	0.05183
hsa_circ_	-0.45867	0.051833
hsa_circ_	0.560383	0.051839
hsa_circ_	-0.54815	0.051843
hsa_circ_	-0.57768	0.051849
hsa_circ_	0.597825	0.05185
hsa_circ_	-0.50503	0.051861
hsa_circ_	0.420217	0.051863
hsa_circ_	-0.29297	0.05187
hsa_circ_	0.556673	0.051874
hsa_circ_	0.479315	0.051876
hsa_circ_	0.389852	0.051876
hsa_circ_	-0.39476	0.051877
hsa_circ_	-0.29438	0.05188
hsa_circ_	-0.4915	0.051888
hsa_circ_	-0.59978	0.051891
hsa_circ_	-0.45879	0.051905
hsa_circ_	-0.39857	0.051908
hsa_circ_	-1.43919	0.05191
hsa_circ_	0.675929	0.051916
hsa_circ_	-0.58413	0.051926
hsa_circ_	-0.78628	0.051928
hsa_circ_	-0.34975	0.051935
hsa_circ_	-0.34676	0.051937
hsa_circ_	-0.61299	0.051941
hsa_circ_	-0.35647	0.051947
hsa_circ_	0.589533	0.051947

hsa_circ_	-0.53596	0.051953
hsa_circ_	-1.38479	0.051956
hsa_circ_	1.277005	0.051956
hsa_circ_	-0.53727	0.051959
hsa_circ_	-0.95656	0.051963
hsa_circ_	0.796622	0.051964
hsa_circ_	-0.46942	0.051966
hsa_circ_	-0.60822	0.05197
hsa_circ_	-0.38528	0.051974
hsa_circ_	0.61935	0.051981
hsa_circ_	-0.325	0.051983
hsa_circ_	0.513311	0.051985
hsa_circ_	0.519	0.051986
hsa_circ_	0.411573	0.051995
hsa_circ_	1.48985	0.051996
hsa_circ_	-0.32191	0.052004
hsa_circ_	-0.94585	0.052005
hsa_circ_	0.447206	0.052007
hsa_circ_	0.421851	0.052015
hsa_circ_	-0.37363	0.052023
hsa_circ_	0.897506	0.052025
hsa_circ_	0.664191	0.052035
hsa_circ_	-0.64713	0.052046
hsa_circ_	-0.46364	0.052046
hsa_circ_	0.355093	0.052047
hsa_circ_	0.447203	0.052049
hsa_circ_	0.331559	0.05205
hsa_circ_	0.715589	0.052061
hsa_circ_	0.520905	0.052068
hsa_circ_	-0.53393	0.052082
hsa_circ_	0.29891	0.052096
hsa_circ_	-0.49703	0.052099
hsa_circ_	-0.32562	0.052112
hsa_circ_	0.465916	0.052119
hsa_circ_	-0.33066	0.05213
hsa_circ_	-0.43864	0.052136
hsa_circ_	0.510505	0.052137
hsa_circ_	0.528056	0.05214
hsa_circ_	0.348097	0.052142
hsa_circ_	-0.40779	0.052159
hsa_circ_	-0.85859	0.052179
hsa_circ_	0.343202	0.0522
hsa_circ_	0.448481	0.052203
hsa_circ_	-0.77297	0.05221
hsa_circ_	-0.39737	0.052214
hsa_circ_	0.311486	0.052214
hsa_circ_	0.576376	0.052215
hsa_circ_	-0.36774	0.052216
hsa_circ_	0.542285	0.052247
hsa_circ_	-0.48005	0.052248
hsa_circ_	-0.4957	0.052254
hsa_circ_	-0.34705	0.052268
hsa_circ_	0.576638	0.052274
hsa_circ_	0.389569	0.052276

hsa_circ_	-0.45033	0.052283
hsa_circ_	0.800035	0.052286
hsa_circ_	-0.47232	0.052295
hsa_circ_	-0.34505	0.052295
hsa_circ_	0.609397	0.052298
hsa_circ_	-0.28535	0.052306
hsa_circ_	0.293925	0.052307
hsa_circ_	0.451367	0.052307
hsa_circ_	-0.53098	0.05231
hsa_circ_	0.306249	0.052313
hsa_circ_	-1.38685	0.052316
hsa_circ_	0.551862	0.05232
hsa_circ_	0.684521	0.052326
hsa_circ_	-0.37342	0.052329
hsa_circ_	-0.28998	0.052338
hsa_circ_	-0.4703	0.052354
hsa_circ_	0.398524	0.052354
hsa_circ_	-0.55862	0.052357
hsa_circ_	-0.37162	0.052381
hsa_circ_	-0.40416	0.052382
hsa_circ_	-0.5787	0.052384
hsa_circ_	0.851013	0.05239
hsa_circ_	-0.33182	0.052391
hsa_circ_	-0.57976	0.052401
hsa_circ_	0.435991	0.052413
hsa_circ_	-0.6869	0.052415
hsa_circ_	0.376332	0.052442
hsa_circ_	0.384086	0.052458
hsa_circ_	-1.02497	0.052467
hsa_circ_	0.546359	0.052475
hsa_circ_	-0.43095	0.052494
hsa_circ_	0.350106	0.052499
hsa_circ_	0.507881	0.052502
hsa_circ_	-0.42603	0.052508
hsa_circ_	0.423166	0.052519
hsa_circ_	0.755461	0.05252
hsa_circ_	-0.36045	0.052521
hsa_circ_	0.6483	0.052521
hsa_circ_	-0.32292	0.052522
hsa_circ_	0.390715	0.052536
hsa_circ_	-0.31613	0.052543
hsa_circ_	0.829205	0.052543
hsa_circ_	0.828847	0.05255
hsa_circ_	-0.67316	0.052552
hsa_circ_	0.401203	0.052554
hsa_circ_	0.617828	0.052556
hsa_circ_	-0.91113	0.052556
hsa_circ_	1.281919	0.052562
hsa_circ_	0.610785	0.052564
hsa_circ_	-1.0653	0.052572
hsa_circ_	0.861686	0.052578
hsa_circ_	0.548162	0.052586
hsa_circ_	0.396516	0.052597
hsa_circ_	0.698956	0.0526

hsa_circ_	-0.63732	0.052601
hsa_circ_	0.449497	0.052604
hsa_circ_	0.59094	0.05261
hsa_circ_	-0.39925	0.052616
hsa_circ_	0.491137	0.052629
hsa_circ_	-0.75123	0.052631
hsa_circ_	0.745949	0.052637
hsa_circ_	-0.57686	0.052637
hsa_circ_	-0.38636	0.052639
hsa_circ_	0.632865	0.052642
hsa_circ_	0.405051	0.052652
hsa_circ_	1.277131	0.052653
hsa_circ_	0.416058	0.052667
hsa_circ_	-0.35244	0.052672
hsa_circ_	0.879259	0.052673
hsa_circ_	-0.45358	0.052679
hsa_circ_	-0.66037	0.052682
hsa_circ_	-2.08419	0.052683
hsa_circ_	-0.40837	0.052683
hsa_circ_	0.761773	0.052685
hsa_circ_	-0.55825	0.052686
hsa_circ_	0.532908	0.052693
hsa_circ_	-0.29198	0.052695
hsa_circ_	-0.62896	0.052704
hsa_circ_	-1.17517	0.052709
hsa_circ_	-0.51406	0.052723
hsa_circ_	0.450064	0.052732
hsa_circ_	0.499997	0.052745
hsa_circ_	0.765536	0.052745
hsa_circ_	1.587042	0.052751
hsa_circ_	1.228882	0.052761
hsa_circ_	0.312751	0.052763
hsa_circ_	-0.35468	0.052765
hsa_circ_	0.44883	0.052775
hsa_circ_	1.028424	0.052779
hsa_circ_	-0.29761	0.052782
hsa_circ_	0.460172	0.05279
hsa_circ_	0.576974	0.052793
hsa_circ_	-0.43278	0.052802
hsa_circ_	-0.42727	0.052802
hsa_circ_	0.491982	0.052809
hsa_circ_	0.420693	0.052824
hsa_circ_	-0.7014	0.052824
hsa_circ_	-0.44526	0.05283
hsa_circ_	-0.37435	0.052832
hsa_circ_	2.115549	0.052834
hsa_circ_	-0.42061	0.052844
hsa_circ_	-0.47003	0.052855
hsa_circ_	-0.72915	0.052858
hsa_circ_	-0.3589	0.052863
hsa_circ_	0.344644	0.052874
hsa_circ_	0.427151	0.052874
hsa_circ_	0.947495	0.052876
hsa_circ_	-0.50877	0.052885

hsa_circ_	-0.3681	0.052898
hsa_circ_	-0.36896	0.0529
hsa_circ_	-0.29904	0.052901
hsa_circ_	-0.75604	0.052908
hsa_circ_	0.439802	0.05291
hsa_circ_	-2.865	0.052912
hsa_circ_	0.511371	0.052921
hsa_circ_	-0.39618	0.052923
hsa_circ_	-0.42659	0.052923
hsa_circ_	0.46022	0.052926
hsa_circ_	0.492979	0.052926
hsa_circ_	0.320915	0.05294
hsa_circ_	-0.46361	0.05294
hsa_circ_	-0.33439	0.052942
hsa_circ_	0.868941	0.052942
hsa_circ_	1.131613	0.052943
hsa_circ_	-0.49695	0.052947
hsa_circ_	0.473332	0.052954
hsa_circ_	-0.82975	0.052955
hsa_circ_	1.811863	0.052955
hsa_circ_	-0.83504	0.052963
hsa_circ_	0.625179	0.052974
hsa_circ_	0.368093	0.052977
hsa_circ_	-1.10207	0.052978
hsa_circ_	-0.45599	0.05298
hsa_circ_	-0.47147	0.052988
hsa_circ_	0.335394	0.052999
hsa_circ_	-0.37394	0.053008
hsa_circ_	0.391839	0.05301
hsa_circ_	-0.46697	0.053034
hsa_circ_	0.521602	0.053043
hsa_circ_	0.802937	0.053045
hsa_circ_	-0.66121	0.053058
hsa_circ_	-0.44665	0.053059
hsa_circ_	-1.06788	0.053063
hsa_circ_	0.409664	0.053069
hsa_circ_	-0.52351	0.053081
hsa_circ_	-0.55644	0.053082
hsa_circ_	-1.28352	0.053085
hsa_circ_	0.454582	0.053086
hsa_circ_	-0.63838	0.053102
hsa_circ_	-0.49983	0.053114
hsa_circ_	0.379386	0.053117
hsa_circ_	-0.41986	0.053119
hsa_circ_	0.977499	0.053122
hsa_circ_	-0.36017	0.053125
hsa_circ_	-0.66946	0.053129
hsa_circ_	-0.34305	0.053135
hsa_circ_	0.307908	0.053145
hsa_circ_	0.484716	0.053146
hsa_circ_	-0.54099	0.053149
hsa_circ_	-0.39396	0.053167
hsa_circ_	-0.78205	0.053177
hsa_circ_	0.86051	0.05318

hsa_circ_	0.562323	0.053182
hsa_circ_	-0.35975	0.0532
hsa_circ_	-0.56505	0.053205
hsa_circ_	-0.32078	0.053208
hsa_circ_	-0.52557	0.053208
hsa_circ_	-1.24002	0.053214
hsa_circ_	0.633979	0.05323
hsa_circ_	0.762015	0.053232
hsa_circ_	-0.62671	0.053233
hsa_circ_	0.450421	0.053235
hsa_circ_	-0.85507	0.053243
hsa_circ_	0.602226	0.053246
hsa_circ_	0.840668	0.053247
hsa_circ_	-0.30184	0.053254
hsa_circ_	-0.81508	0.053257
hsa_circ_	0.505995	0.053257
hsa_circ_	-0.37543	0.053264
hsa_circ_	-1.55575	0.053275
hsa_circ_	-0.34396	0.05329
hsa_circ_	-0.3565	0.053296
hsa_circ_	-0.67659	0.053302
hsa_circ_	-1.01127	0.053309
hsa_circ_	-0.38514	0.053313
hsa_circ_	-0.31186	0.053322
hsa_circ_	-0.33662	0.053328
hsa_circ_	-0.30732	0.053329
hsa_circ_	0.32347	0.05333
hsa_circ_	-0.55432	0.053333
hsa_circ_	-0.53046	0.053334
hsa_circ_	-0.74354	0.053348
hsa_circ_	-0.64173	0.053351
hsa_circ_	0.371084	0.053352
hsa_circ_	-0.74072	0.053353
hsa_circ_	0.683437	0.053359
hsa_circ_	-0.59786	0.053359
hsa_circ_	0.380717	0.053367
hsa_circ_	0.515189	0.053383
hsa_circ_	0.558228	0.053394
hsa_circ_	0.37153	0.053403
hsa_circ_	-0.30586	0.053404
hsa_circ_	0.788772	0.053415
hsa_circ_	-0.37335	0.053425
hsa_circ_	-0.42789	0.053428
hsa_circ_	-0.51347	0.053431
hsa_circ_	-0.45461	0.053432
hsa_circ_	0.918131	0.053438
hsa_circ_	0.38742	0.05344
hsa_circ_	-0.34058	0.053446
hsa_circ_	-0.89798	0.053447
hsa_circ_	-0.38408	0.053455
hsa_circ_	-0.62519	0.053457
hsa_circ_	0.584562	0.053459
hsa_circ_	-0.36271	0.053459
hsa_circ_	-0.46751	0.053462

hsa_circ_	0.630161	0.053463
hsa_circ_	-0.62203	0.053464
hsa_circ_	0.303968	0.053468
hsa_circ_	-0.40785	0.053475
hsa_circ_	-0.36602	0.053477
hsa_circ_	-0.87709	0.053479
hsa_circ_	0.687996	0.053484
hsa_circ_	-0.79075	0.053487
hsa_circ_	-0.3188	0.053489
hsa_circ_	-0.642	0.053491
hsa_circ_	-0.31696	0.053494
hsa_circ_	0.40707	0.053498
hsa_circ_	0.415006	0.053501
hsa_circ_	-0.57035	0.053509
hsa_circ_	-0.49952	0.053514
hsa_circ_	-0.58736	0.053537
hsa_circ_	-0.76975	0.053537
hsa_circ_	0.539419	0.053544
hsa_circ_	-0.37663	0.053546
hsa_circ_	-0.52372	0.053548
hsa_circ_	0.424404	0.053548
hsa_circ_	0.782356	0.053549
hsa_circ_	0.326763	0.053552
hsa_circ_	0.517101	0.053554
hsa_circ_	0.432649	0.05356
hsa_circ_	0.348827	0.053562
hsa_circ_	1.206539	0.053567
hsa_circ_	-0.76699	0.053589
hsa_circ_	0.881662	0.0536
hsa_circ_	0.544699	0.053614
hsa_circ_	-0.7995	0.053615
hsa_circ_	-0.39427	0.053615
hsa_circ_	0.595083	0.053619
hsa_circ_	-0.78904	0.053621
hsa_circ_	-0.36761	0.053624
hsa_circ_	1.369949	0.053633
hsa_circ_	-0.40914	0.053641
hsa_circ_	0.560285	0.053645
hsa_circ_	0.352561	0.053645
hsa_circ_	-0.53259	0.053648
hsa_circ_	-0.37895	0.053649
hsa_circ_	-0.35186	0.05365
hsa_circ_	0.696185	0.053657
hsa_circ_	-0.3761	0.05366
hsa_circ_	-0.32431	0.053661
hsa_circ_	0.418626	0.053667
hsa_circ_	-0.45362	0.053674
hsa_circ_	-0.34647	0.053675
hsa_circ_	-0.34946	0.053675
hsa_circ_	1.181287	0.053684
hsa_circ_	0.640432	0.05369
hsa_circ_	-0.35944	0.053691
hsa_circ_	-0.29626	0.053702
hsa_circ_	0.454748	0.053703

hsa_circ_	0.517354	0.053713
hsa_circ_	0.382451	0.053724
hsa_circ_	0.390735	0.053738
hsa_circ_	1.009207	0.053748
hsa_circ_	0.354517	0.053751
hsa_circ_	-0.29698	0.053752
hsa_circ_	0.458185	0.053756
hsa_circ_	0.404549	0.053756
hsa_circ_	1.190121	0.053779
hsa_circ_	0.326685	0.053801
hsa_circ_	-0.41702	0.053805
hsa_circ_	-0.53199	0.053806
hsa_circ_	-0.33336	0.053813
hsa_circ_	0.434142	0.053815
hsa_circ_	0.776493	0.053816
hsa_circ_	0.579745	0.053822
hsa_circ_	0.883331	0.053838
hsa_circ_	-0.33014	0.05384
hsa_circ_	0.407463	0.053846
hsa_circ_	-0.74556	0.053847
hsa_circ_	0.644454	0.053849
hsa_circ_	-0.47415	0.053852
hsa_circ_	0.670695	0.053852
hsa_circ_	-0.94405	0.053855
hsa_circ_	-0.48405	0.053856
hsa_circ_	0.366292	0.053862
hsa_circ_	0.48247	0.053864
hsa_circ_	-0.3602	0.053866
hsa_circ_	-1.53719	0.05387
hsa_circ_	-0.59485	0.053873
hsa_circ_	-0.38729	0.053873
hsa_circ_	-0.89836	0.053876
hsa_circ_	0.602052	0.053887
hsa_circ_	-0.3392	0.05389
hsa_circ_	-0.52594	0.05389
hsa_circ_	-0.38286	0.053891
hsa_circ_	0.317294	0.053895
hsa_circ_	0.657612	0.053911
hsa_circ_	-0.55896	0.053917
hsa_circ_	-0.55026	0.053918
hsa_circ_	0.521231	0.053921
hsa_circ_	-0.64852	0.053929
hsa_circ_	-0.37444	0.05393
hsa_circ_	0.369007	0.053936
hsa_circ_	-0.35427	0.053951
hsa_circ_	-0.99987	0.053951
hsa_circ_	0.950796	0.053951
hsa_circ_	0.516253	0.053951
hsa_circ_	1.163874	0.053952
hsa_circ_	-0.40087	0.053964
hsa_circ_	0.628786	0.053965
hsa_circ_	-0.3021	0.053969
hsa_circ_	-0.37172	0.053979
hsa_circ_	-0.51169	0.053985

hsa_circ_	0.915775	0.053991
hsa_circ_	-0.43415	0.053996
hsa_circ_	0.675993	0.054007
hsa_circ_	0.873039	0.05401
hsa_circ_	-0.32848	0.054013
hsa_circ_	-0.56416	0.054013
hsa_circ_	0.601069	0.054028
hsa_circ_	0.772968	0.05403
hsa_circ_	-1.27188	0.05403
hsa_circ_	0.334663	0.054038
hsa_circ_	-0.483	0.05404
hsa_circ_	-0.4678	0.054047
hsa_circ_	-0.45998	0.054068
hsa_circ_	-0.34046	0.054092
hsa_circ_	0.349837	0.054097
hsa_circ_	-0.39821	0.054099
hsa_circ_	0.741028	0.054105
hsa_circ_	0.862457	0.054106
hsa_circ_	-0.55391	0.05411
hsa_circ_	-0.29263	0.054113
hsa_circ_	0.356658	0.054121
hsa_circ_	0.401529	0.054145
hsa_circ_	-0.86474	0.054147
hsa_circ_	-0.84961	0.054149
hsa_circ_	0.420189	0.054152
hsa_circ_	-0.87645	0.054167
hsa_circ_	0.372592	0.054175
hsa_circ_	0.339392	0.054179
hsa_circ_	0.455024	0.054184
hsa_circ_	0.361118	0.054184
hsa_circ_	0.659164	0.054189
hsa_circ_	-0.33017	0.054191
hsa_circ_	-0.455	0.054193
hsa_circ_	-0.96161	0.054194
hsa_circ_	-0.56257	0.054203
hsa_circ_	-0.44056	0.054206
hsa_circ_	0.844875	0.054222
hsa_circ_	-0.3615	0.054225
hsa_circ_	-0.48111	0.05423
hsa_circ_	-0.29649	0.054233
hsa_circ_	0.306848	0.054246
hsa_circ_	-2.73463	0.054249
hsa_circ_	0.464494	0.054251
hsa_circ_	0.787541	0.05426
hsa_circ_	-0.30545	0.054271
hsa_circ_	-0.69404	0.054275
hsa_circ_	-0.87531	0.054282
hsa_circ_	-0.36235	0.054284
hsa_circ_	0.397383	0.054292
hsa_circ_	0.606793	0.054298
hsa_circ_	-0.37238	0.054301
hsa_circ_	0.483188	0.054302
hsa_circ_	-0.75886	0.054303
hsa_circ_	0.388639	0.054307

hsa_circ_	0.386611	0.054314
hsa_circ_	-0.38279	0.054321
hsa_circ_	1.208603	0.054327
hsa_circ_	0.374619	0.05433
hsa_circ_	1.605682	0.054337
hsa_circ_	-0.49653	0.054339
hsa_circ_	0.423582	0.054343
hsa_circ_	1.102228	0.054346
hsa_circ_	-0.43947	0.054359
hsa_circ_	0.626859	0.054361
hsa_circ_	-0.53528	0.054366
hsa_circ_	-0.36779	0.054379
hsa_circ_	-0.53316	0.054382
hsa_circ_	-0.79742	0.054383
hsa_circ_	0.56766	0.054385
hsa_circ_	0.531604	0.054387
hsa_circ_	0.470394	0.054392
hsa_circ_	0.355995	0.054393
hsa_circ_	-0.97684	0.054401
hsa_circ_	0.495202	0.054404
hsa_circ_	-0.88552	0.05441
hsa_circ_	0.390026	0.054418
hsa_circ_	-0.40337	0.054421
hsa_circ_	-0.53209	0.054427
hsa_circ_	-0.81919	0.054427
hsa_circ_	-0.52755	0.054432
hsa_circ_	0.618711	0.054455
hsa_circ_	-0.30979	0.054456
hsa_circ_	-0.80155	0.054458
hsa_circ_	0.539795	0.054461
hsa_circ_	0.386173	0.054465
hsa_circ_	-0.44437	0.054465
hsa_circ_	0.427193	0.05449
hsa_circ_	0.623061	0.05449
hsa_circ_	-0.41103	0.054502
hsa_circ_	-0.65234	0.054509
hsa_circ_	-0.30966	0.054518
hsa_circ_	0.916917	0.05452
hsa_circ_	0.443632	0.054521
hsa_circ_	-1.44376	0.054527
hsa_circ_	-0.56105	0.054549
hsa_circ_	-0.40197	0.054553
hsa_circ_	0.454895	0.054553
hsa_circ_	-1.39766	0.054553
hsa_circ_	0.298993	0.054557
hsa_circ_	-0.54513	0.054575
hsa_circ_	-0.66408	0.054576
hsa_circ_	0.585198	0.054581
hsa_circ_	-0.32348	0.054583
hsa_circ_	-0.68189	0.05459
hsa_circ_	0.688221	0.05459
hsa_circ_	-0.92334	0.054602
hsa_circ_	1.0719	0.054617
hsa_circ_	-0.32417	0.05462

hsa_circ_	-0.37111	0.054631
hsa_circ_	0.504184	0.054647
hsa_circ_	0.436035	0.054651
hsa_circ_	0.564589	0.054666
hsa_circ_	0.360373	0.054669
hsa_circ_	0.664064	0.054676
hsa_circ_	-0.32566	0.05468
hsa_circ_	-0.6211	0.054682
hsa_circ_	0.391847	0.054689
hsa_circ_	0.652436	0.054705
hsa_circ_	-0.82601	0.054714
hsa_circ_	0.607079	0.054722
hsa_circ_	-0.85971	0.054723
hsa_circ_	0.683782	0.054724
hsa_circ_	0.382747	0.054731
hsa_circ_	-0.51705	0.054735
hsa_circ_	-0.52251	0.054742
hsa_circ_	0.662642	0.054754
hsa_circ_	-0.83093	0.054761
hsa_circ_	-0.36638	0.054764
hsa_circ_	0.47165	0.054781
hsa_circ_	-0.4107	0.054782
hsa_circ_	-0.70599	0.054784
hsa_circ_	-0.46836	0.054795
hsa_circ_	0.444395	0.054796
hsa_circ_	-0.65681	0.054799
hsa_circ_	-0.35037	0.054803
hsa_circ_	-0.53283	0.05482
hsa_circ_	-0.49056	0.054823
hsa_circ_	0.282262	0.054832
hsa_circ_	-0.43838	0.05484
hsa_circ_	-0.67511	0.054842
hsa_circ_	-0.53632	0.054848
hsa_circ_	-0.41366	0.054853
hsa_circ_	-1.11563	0.054859
hsa_circ_	0.366547	0.054864
hsa_circ_	-0.3954	0.054874
hsa_circ_	0.775633	0.054889
hsa_circ_	0.535944	0.054896
hsa_circ_	0.302795	0.054914
hsa_circ_	-0.29013	0.054927
hsa_circ_	-0.53625	0.054929
hsa_circ_	-0.39655	0.05493
hsa_circ_	0.466505	0.054959
hsa_circ_	-0.29391	0.05496
hsa_circ_	-0.50708	0.054964
hsa_circ_	0.3078	0.054966
hsa_circ_	0.429991	0.055004
hsa_circ_	0.644706	0.055006
hsa_circ_	-0.35941	0.05501
hsa_circ_	0.571062	0.055011
hsa_circ_	0.80633	0.055012
hsa_circ_	0.843639	0.055012
hsa_circ_	0.741261	0.05502

hsa_circ_	-0.79504	0.055022
hsa_circ_	0.498896	0.055027
hsa_circ_	-0.53525	0.055031
hsa_circ_	-1.02546	0.055033
hsa_circ_	0.439552	0.055034
hsa_circ_	-0.33595	0.055036
hsa_circ_	0.322017	0.055041
hsa_circ_	0.588513	0.055041
hsa_circ_	-0.33864	0.055046
hsa_circ_	-0.67842	0.055055
hsa_circ_	-0.84747	0.055063
hsa_circ_	0.393103	0.055064
hsa_circ_	0.476386	0.055072
hsa_circ_	-0.38068	0.055079
hsa_circ_	1.134577	0.05508
hsa_circ_	-0.55312	0.055087
hsa_circ_	0.540859	0.055087
hsa_circ_	0.526598	0.055126
hsa_circ_	-0.7725	0.055132
hsa_circ_	-0.31709	0.055137
hsa_circ_	-0.35541	0.055142
hsa_circ_	1.08574	0.055145
hsa_circ_	-0.40201	0.055149
hsa_circ_	-0.40108	0.055149
hsa_circ_	-0.29096	0.055155
hsa_circ_	-0.3571	0.05516
hsa_circ_	0.635616	0.055161
hsa_circ_	-0.31108	0.055162
hsa_circ_	0.696402	0.05517
hsa_circ_	0.535346	0.055171
hsa_circ_	-0.50348	0.055188
hsa_circ_	0.503114	0.055188
hsa_circ_	-0.44473	0.05519
hsa_circ_	-0.60149	0.055194
hsa_circ_	0.331415	0.055196
hsa_circ_	-1.68451	0.055196
hsa_circ_	-0.53883	0.055197
hsa_circ_	-0.53608	0.055198
hsa_circ_	-0.51145	0.0552
hsa_circ_	0.431499	0.055204
hsa_circ_	0.399096	0.05522
hsa_circ_	-0.55727	0.055233
hsa_circ_	-0.45411	0.055238
hsa_circ_	-0.33154	0.055242
hsa_circ_	0.367915	0.055252
hsa_circ_	0.328794	0.055252
hsa_circ_	0.634401	0.055256
hsa_circ_	0.414026	0.055265
hsa_circ_	-0.41364	0.055266
hsa_circ_	0.490763	0.055271
hsa_circ_	-0.44033	0.055272
hsa_circ_	-0.34056	0.055273
hsa_circ_	-1.01657	0.055275
hsa_circ_	0.482658	0.055295

hsa_circ_	-0.47946	0.055309
hsa_circ_	0.922293	0.055314
hsa_circ_	0.410606	0.055321
hsa_circ_	-0.99119	0.055331
hsa_circ_	-0.65012	0.055338
hsa_circ_	-0.36107	0.055339
hsa_circ_	0.330123	0.055345
hsa_circ_	0.444389	0.055355
hsa_circ_	-0.54453	0.055358
hsa_circ_	1.222083	0.055363
hsa_circ_	-0.97894	0.055371
hsa_circ_	0.617321	0.055379
hsa_circ_	-0.30288	0.05538
hsa_circ_	-0.36885	0.055382
hsa_circ_	-0.61512	0.055383
hsa_circ_	0.33297	0.055389
hsa_circ_	-0.51186	0.055394
hsa_circ_	-0.91027	0.055401
hsa_circ_	0.345471	0.055401
hsa_circ_	1.149772	0.055401
hsa_circ_	0.498856	0.055404
hsa_circ_	-0.3873	0.055405
hsa_circ_	0.394147	0.055406
hsa_circ_	-0.76536	0.055407
hsa_circ_	-0.38552	0.055416
hsa_circ_	0.373913	0.055418
hsa_circ_	-0.33864	0.055426
hsa_circ_	-0.33965	0.055437
hsa_circ_	-0.41556	0.055457
hsa_circ_	-0.32149	0.055465
hsa_circ_	-0.30507	0.055466
hsa_circ_	0.570497	0.055466
hsa_circ_	-0.31444	0.055477
hsa_circ_	-0.87179	0.055478
hsa_circ_	0.367346	0.05549
hsa_circ_	-0.35376	0.055492
hsa_circ_	-0.37488	0.055512
hsa_circ_	-0.37559	0.055515
hsa_circ_	-0.36375	0.05554
hsa_circ_	-0.92638	0.055555
hsa_circ_	0.407601	0.055558
hsa_circ_	-0.36913	0.055558
hsa_circ_	0.414524	0.055559
hsa_circ_	-0.49635	0.055574
hsa_circ_	0.526632	0.055579
hsa_circ_	-0.38849	0.055585
hsa_circ_	-0.30137	0.055599
hsa_circ_	0.613366	0.055601
hsa_circ_	0.550762	0.055602
hsa_circ_	0.3617	0.055606
hsa_circ_	-0.62855	0.055606
hsa_circ_	0.336455	0.055627
hsa_circ_	-0.44095	0.055636
hsa_circ_	0.677253	0.055642

hsa_circ_	0.30095	0.055645
hsa_circ_	0.290152	0.055647
hsa_circ_	-0.64388	0.055654
hsa_circ_	-0.66191	0.055659
hsa_circ_	1.128076	0.05566
hsa_circ_	-0.51858	0.055662
hsa_circ_	0.310412	0.055674
hsa_circ_	-0.65316	0.055687
hsa_circ_	0.300573	0.055689
hsa_circ_	-0.53702	0.055697
hsa_circ_	-0.37288	0.0557
hsa_circ_	-0.35439	0.055704
hsa_circ_	0.296151	0.055705
hsa_circ_	0.744571	0.055707
hsa_circ_	-0.49925	0.055738
hsa_circ_	-1.06564	0.055769
hsa_circ_	0.533497	0.055776
hsa_circ_	-0.41257	0.055781
hsa_circ_	-0.56119	0.055783
hsa_circ_	-0.65401	0.055798
hsa_circ_	0.330648	0.055802
hsa_circ_	0.398517	0.055803
hsa_circ_	0.670524	0.055813
hsa_circ_	0.33776	0.055836
hsa_circ_	0.370402	0.055844
hsa_circ_	-0.30373	0.055849
hsa_circ_	0.42507	0.055849
hsa_circ_	-1.13609	0.055853
hsa_circ_	0.443521	0.055864
hsa_circ_	0.47372	0.055878
hsa_circ_	-0.33941	0.055888
hsa_circ_	0.326144	0.05589
hsa_circ_	-0.53803	0.055901
hsa_circ_	-0.49231	0.055903
hsa_circ_	-0.45564	0.055913
hsa_circ_	0.811222	0.055917
hsa_circ_	-1.29788	0.055918
hsa_circ_	0.335327	0.055921
hsa_circ_	0.969079	0.055949
hsa_circ_	-0.39954	0.055962
hsa_circ_	0.44041	0.055966
hsa_circ_	0.55817	0.055976
hsa_circ_	-0.33646	0.055982
hsa_circ_	0.650455	0.055993
hsa_circ_	-0.28518	0.055997
hsa_circ_	-0.40141	0.056
hsa_circ_	0.794229	0.056005
hsa_circ_	1.336367	0.056006
hsa_circ_	0.516725	0.056009
hsa_circ_	-0.7847	0.056012
hsa_circ_	-0.85463	0.056019
hsa_circ_	0.301755	0.05602
hsa_circ_	0.424032	0.056031
hsa_circ_	-0.98919	0.056032

hsa_circ_	-1.2047	0.056048
hsa_circ_	0.545345	0.05605
hsa_circ_	0.517614	0.056054
hsa_circ_	-0.99568	0.056056
hsa_circ_	-0.52511	0.056057
hsa_circ_	-0.48565	0.056061
hsa_circ_	-0.3939	0.056062
hsa_circ_	0.307849	0.056078
hsa_circ_	-0.66764	0.056084
hsa_circ_	-0.39958	0.056116
hsa_circ_	-0.91058	0.056123
hsa_circ_	0.660605	0.056173
hsa_circ_	-0.30815	0.056179
hsa_circ_	0.513806	0.056184
hsa_circ_	-0.35605	0.056186
hsa_circ_	-0.64658	0.056187
hsa_circ_	-0.38035	0.056192
hsa_circ_	0.582555	0.056194
hsa_circ_	0.41923	0.056201
hsa_circ_	0.612342	0.056201
hsa_circ_	0.481676	0.056205
hsa_circ_	-1.30038	0.05621
hsa_circ_	0.570182	0.056218
hsa_circ_	0.577059	0.056227
hsa_circ_	0.404497	0.056235
hsa_circ_	0.37678	0.056239
hsa_circ_	0.372647	0.056261
hsa_circ_	0.769922	0.056273
hsa_circ_	-0.30745	0.056276
hsa_circ_	-0.32777	0.056278
hsa_circ_	0.338999	0.056287
hsa_circ_	0.387437	0.056288
hsa_circ_	-0.36138	0.056298
hsa_circ_	-0.54574	0.056302
hsa_circ_	-0.31012	0.056303
hsa_circ_	0.941864	0.056322
hsa_circ_	-0.48357	0.056333
hsa_circ_	-0.66996	0.05634
hsa_circ_	-0.71431	0.056341
hsa_circ_	-0.58224	0.056345
hsa_circ_	-0.43018	0.056355
hsa_circ_	-0.34816	0.056358
hsa_circ_	-0.41175	0.056362
hsa_circ_	-0.42062	0.056363
hsa_circ_	0.404169	0.056367
hsa_circ_	-0.43715	0.056382
hsa_circ_	0.30594	0.056383
hsa_circ_	0.439136	0.056384
hsa_circ_	-0.46041	0.056384
hsa_circ_	-0.57995	0.056386
hsa_circ_	0.49265	0.056389
hsa_circ_	-2.00525	0.056394
hsa_circ_	-0.56164	0.056395
hsa_circ_	0.31173	0.056414

hsa_circ_	-0.62299	0.056419
hsa_circ_	0.570872	0.056425
hsa_circ_	0.519682	0.056427
hsa_circ_	0.473537	0.056428
hsa_circ_	0.418905	0.056437
hsa_circ_	1.368992	0.05646
hsa_circ_	0.44501	0.056464
hsa_circ_	0.387984	0.056488
hsa_circ_	-0.35069	0.056488
hsa_circ_	1.143471	0.056492
hsa_circ_	0.458958	0.056509
hsa_circ_	1.179994	0.056516
hsa_circ_	0.961304	0.056523
hsa_circ_	0.873335	0.056524
hsa_circ_	-0.37654	0.056554
hsa_circ_	-0.58966	0.056559
hsa_circ_	-1.62376	0.056561
hsa_circ_	0.743746	0.056568
hsa_circ_	0.380363	0.056573
hsa_circ_	0.676381	0.056573
hsa_circ_	-0.4827	0.056601
hsa_circ_	0.564663	0.056614
hsa_circ_	1.270419	0.056615
hsa_circ_	1.09493	0.056625
hsa_circ_	-0.32338	0.056627
hsa_circ_	-0.60753	0.056628
hsa_circ_	-0.31334	0.056639
hsa_circ_	0.704186	0.056649
hsa_circ_	0.335676	0.056651
hsa_circ_	0.406213	0.056653
hsa_circ_	-0.51379	0.056661
hsa_circ_	0.657375	0.056667
hsa_circ_	0.447639	0.056668
hsa_circ_	0.518837	0.056669
hsa_circ_	0.39852	0.056676
hsa_circ_	-0.54376	0.056677
hsa_circ_	0.870795	0.056686
hsa_circ_	-0.32408	0.056697
hsa_circ_	-0.41003	0.056703
hsa_circ_	0.350254	0.056711
hsa_circ_	-0.32774	0.056714
hsa_circ_	0.657595	0.056714
hsa_circ_	-0.31721	0.056715
hsa_circ_	0.396547	0.056721
hsa_circ_	-0.41145	0.056725
hsa_circ_	-0.42135	0.056731
hsa_circ_	0.399125	0.056731
hsa_circ_	-1.83266	0.056734
hsa_circ_	-0.31037	0.056736
hsa_circ_	-0.4641	0.056746
hsa_circ_	0.350021	0.056748
hsa_circ_	0.756168	0.056751
hsa_circ_	0.362201	0.056758
hsa_circ_	0.639265	0.056768

hsa_circ_	1.297265	0.056772
hsa_circ_	-0.40413	0.056776
hsa_circ_	0.338695	0.056788
hsa_circ_	-0.41799	0.056791
hsa_circ_	-0.29885	0.056793
hsa_circ_	-0.31208	0.056801
hsa_circ_	1.29679	0.056812
hsa_circ_	0.452495	0.056814
hsa_circ_	-0.38468	0.056815
hsa_circ_	0.539602	0.056816
hsa_circ_	0.844407	0.056819
hsa_circ_	-0.59055	0.056819
hsa_circ_	-0.65723	0.056821
hsa_circ_	0.54581	0.056827
hsa_circ_	-0.45562	0.056837
hsa_circ_	0.350636	0.056838
hsa_circ_	-1.04558	0.056847
hsa_circ_	-0.36156	0.056851
hsa_circ_	-0.74668	0.056871
hsa_circ_	0.309072	0.056886
hsa_circ_	-0.31094	0.056891
hsa_circ_	-0.3925	0.056893
hsa_circ_	-1.10873	0.056893
hsa_circ_	1.504494	0.056895
hsa_circ_	-0.46811	0.056904
hsa_circ_	0.450721	0.056904
hsa_circ_	-0.4857	0.056909
hsa_circ_	0.304293	0.05693
hsa_circ_	0.587757	0.056935
hsa_circ_	-0.52903	0.056939
hsa_circ_	0.560046	0.056945
hsa_circ_	-0.76988	0.056958
hsa_circ_	-0.41076	0.056975
hsa_circ_	0.299198	0.056978
hsa_circ_	-0.38123	0.056984
hsa_circ_	-0.9115	0.057004
hsa_circ_	-0.79314	0.057006
hsa_circ_	-0.32624	0.057009
hsa_circ_	-0.36508	0.057011
hsa_circ_	0.452317	0.057034
hsa_circ_	1.239785	0.057039
hsa_circ_	-0.36014	0.057055
hsa_circ_	0.425386	0.057068
hsa_circ_	-0.35239	0.057068
hsa_circ_	0.517853	0.057084
hsa_circ_	-0.29639	0.057098
hsa_circ_	0.372316	0.057099
hsa_circ_	0.467833	0.057099
hsa_circ_	0.508991	0.057104
hsa_circ_	-0.6835	0.057108
hsa_circ_	-0.45852	0.057109
hsa_circ_	0.31771	0.057109
hsa_circ_	0.357594	0.057113
hsa_circ_	-0.42319	0.057121

hsa_circ_	0.327182	0.057128
hsa_circ_	-0.37615	0.05713
hsa_circ_	0.934554	0.057132
hsa_circ_	-0.50045	0.057136
hsa_circ_	-0.44053	0.057148
hsa_circ_	-0.54047	0.057151
hsa_circ_	-0.41821	0.057153
hsa_circ_	0.381992	0.057158
hsa_circ_	1.019994	0.05717
hsa_circ_	-0.44103	0.057176
hsa_circ_	-0.69937	0.057177
hsa_circ_	0.524771	0.057181
hsa_circ_	-0.52223	0.057199
hsa_circ_	0.47185	0.057201
hsa_circ_	-0.3158	0.057204
hsa_circ_	0.462812	0.057208
hsa_circ_	-0.36747	0.057219
hsa_circ_	0.565471	0.057229
hsa_circ_	-0.89379	0.057231
hsa_circ_	-0.29835	0.057233
hsa_circ_	0.389445	0.057251
hsa_circ_	0.951497	0.057251
hsa_circ_	-0.6687	0.057257
hsa_circ_	0.406487	0.057258
hsa_circ_	-0.32207	0.057264
hsa_circ_	1.277067	0.057268
hsa_circ_	-0.32968	0.057272
hsa_circ_	0.660223	0.057281
hsa_circ_	-0.50431	0.057284
hsa_circ_	0.520329	0.057295
hsa_circ_	-0.43281	0.057301
hsa_circ_	-0.29771	0.057305
hsa_circ_	0.946234	0.057308
hsa_circ_	0.401265	0.05731
hsa_circ_	-0.47705	0.057312
hsa_circ_	-0.72237	0.057312
hsa_circ_	-0.89918	0.057317
hsa_circ_	0.308097	0.057319
hsa_circ_	-0.37046	0.05733
hsa_circ_	0.348391	0.057337
hsa_circ_	-0.41551	0.057348
hsa_circ_	-0.31586	0.057353
hsa_circ_	-0.63483	0.057355
hsa_circ_	-0.33074	0.05736
hsa_circ_	-0.5465	0.057366
hsa_circ_	-0.44819	0.057384
hsa_circ_	0.332893	0.057397
hsa_circ_	0.417969	0.0574
hsa_circ_	0.44606	0.057401
hsa_circ_	-0.37688	0.057403
hsa_circ_	0.298203	0.05741
hsa_circ_	-0.38478	0.057413
hsa_circ_	1.088676	0.057414
hsa_circ_	0.470694	0.057415

hsa_circ_	0.606237	0.057416
hsa_circ_	-0.31927	0.05742
hsa_circ_	-0.54514	0.057425
hsa_circ_	0.561824	0.057431
hsa_circ_	0.362756	0.057431
hsa_circ_	0.441758	0.057432
hsa_circ_	0.493645	0.057433
hsa_circ_	0.502738	0.057435
hsa_circ_	-0.36924	0.057438
hsa_circ_	-0.91126	0.057441
hsa_circ_	-0.80402	0.057447
hsa_circ_	0.693724	0.057455
hsa_circ_	-0.39137	0.057462
hsa_circ_	0.300579	0.057462
hsa_circ_	-1.56503	0.057476
hsa_circ_	0.314322	0.057477
hsa_circ_	0.305217	0.057483
hsa_circ_	0.376628	0.057489
hsa_circ_	-0.32239	0.05749
hsa_circ_	-0.75571	0.0575
hsa_circ_	-0.5219	0.057501
hsa_circ_	-0.52158	0.057507
hsa_circ_	-0.44913	0.05751
hsa_circ_	0.448751	0.057523
hsa_circ_	0.405344	0.057543
hsa_circ_	0.9176	0.057545
hsa_circ_	-0.5618	0.057546
hsa_circ_	-0.44463	0.057546
hsa_circ_	-0.71269	0.057552
hsa_circ_	-0.5031	0.057557
hsa_circ_	-0.39979	0.057558
hsa_circ_	0.789612	0.057563
hsa_circ_	-0.4915	0.057574
hsa_circ_	-0.40228	0.057576
hsa_circ_	0.337902	0.057578
hsa_circ_	-0.48125	0.057578
hsa_circ_	0.619332	0.057581
hsa_circ_	-0.48453	0.057594
hsa_circ_	0.905864	0.057596
hsa_circ_	-0.33288	0.057601
hsa_circ_	0.377526	0.057607
hsa_circ_	-1.05325	0.057608
hsa_circ_	-0.70958	0.057615
hsa_circ_	0.444157	0.057623
hsa_circ_	-0.61498	0.057641
hsa_circ_	0.512333	0.057646
hsa_circ_	0.573977	0.057676
hsa_circ_	-0.36662	0.057695
hsa_circ_	0.365835	0.057705
hsa_circ_	0.349056	0.057709
hsa_circ_	0.300654	0.05771
hsa_circ_	-0.88061	0.057711
hsa_circ_	0.459448	0.057716
hsa_circ_	1.343992	0.057716

hsa_circ_	0.655999	0.05772
hsa_circ_	0.294919	0.057733
hsa_circ_	-0.98246	0.057737
hsa_circ_	0.922894	0.057745
hsa_circ_	0.583294	0.057749
hsa_circ_	-0.45827	0.057752
hsa_circ_	-0.60327	0.057771
hsa_circ_	-0.55963	0.057777
hsa_circ_	0.424754	0.057778
hsa_circ_	0.534954	0.057783
hsa_circ_	-0.36526	0.057785
hsa_circ_	-0.31861	0.057793
hsa_circ_	-0.51935	0.057794
hsa_circ_	-0.41397	0.057794
hsa_circ_	0.2968	0.057799
hsa_circ_	-0.77464	0.057799
hsa_circ_	0.865149	0.057817
hsa_circ_	0.3063	0.057821
hsa_circ_	-0.39972	0.057829
hsa_circ_	0.525576	0.05783
hsa_circ_	-0.30998	0.057836
hsa_circ_	-0.31716	0.057841
hsa_circ_	0.365307	0.057847
hsa_circ_	0.638462	0.057849
hsa_circ_	-1.11022	0.057857
hsa_circ_	-0.3385	0.057858
hsa_circ_	-0.95385	0.05787
hsa_circ_	0.745527	0.057872
hsa_circ_	0.358061	0.057873
hsa_circ_	0.427462	0.057881
hsa_circ_	-0.67232	0.057885
hsa_circ_	-0.44747	0.057885
hsa_circ_	0.353549	0.057893
hsa_circ_	-0.4045	0.057897
hsa_circ_	-0.36582	0.057919
hsa_circ_	0.430789	0.057922
hsa_circ_	-0.5837	0.057928
hsa_circ_	0.616879	0.057932
hsa_circ_	-0.32618	0.057932
hsa_circ_	0.985505	0.057947
hsa_circ_	0.345494	0.057949
hsa_circ_	-0.32427	0.057952
hsa_circ_	-0.36007	0.057954
hsa_circ_	-0.28645	0.057961
hsa_circ_	-0.55685	0.05798
hsa_circ_	0.42892	0.057983
hsa_circ_	-0.33252	0.057995
hsa_circ_	0.835584	0.058004
hsa_circ_	-0.34929	0.058006
hsa_circ_	0.427241	0.058006
hsa_circ_	0.65432	0.058019
hsa_circ_	-0.55949	0.058026
hsa_circ_	-0.32451	0.058029
hsa_circ_	-0.3003	0.058034

hsa_circ_	0.534977	0.058038
hsa_circ_	-0.29727	0.058039
hsa_circ_	0.897224	0.058039
hsa_circ_	-0.50341	0.058044
hsa_circ_	0.357449	0.058058
hsa_circ_	0.516767	0.058062
hsa_circ_	0.523796	0.058076
hsa_circ_	-1.1338	0.058078
hsa_circ_	-0.29294	0.058079
hsa_circ_	0.58082	0.058087
hsa_circ_	0.36098	0.058091
hsa_circ_	0.35819	0.058095
hsa_circ_	0.560329	0.058106
hsa_circ_	1.269187	0.058109
hsa_circ_	-0.39573	0.058112
hsa_circ_	0.483185	0.058125
hsa_circ_	1.097156	0.058126
hsa_circ_	0.519787	0.058126
hsa_circ_	-0.40765	0.058127
hsa_circ_	0.487281	0.058132
hsa_circ_	-0.36544	0.058132
hsa_circ_	-0.37141	0.058136
hsa_circ_	0.570699	0.058143
hsa_circ_	0.863596	0.058146
hsa_circ_	-0.69842	0.05815
hsa_circ_	0.576442	0.058156
hsa_circ_	0.333738	0.058197
hsa_circ_	0.490845	0.0582
hsa_circ_	0.607869	0.058224
hsa_circ_	-0.91473	0.058238
hsa_circ_	-0.44778	0.058239
hsa_circ_	0.708656	0.05824
hsa_circ_	0.330231	0.058243
hsa_circ_	0.602431	0.058268
hsa_circ_	0.570725	0.058279
hsa_circ_	-0.41985	0.058288
hsa_circ_	-0.54742	0.058289
hsa_circ_	0.485027	0.058289
hsa_circ_	-0.63894	0.058291
hsa_circ_	0.330668	0.058303
hsa_circ_	0.503071	0.058306
hsa_circ_	0.637676	0.058314
hsa_circ_	0.494257	0.058319
hsa_circ_	0.729929	0.058319
hsa_circ_	0.329717	0.058325
hsa_circ_	0.720697	0.058328
hsa_circ_	-0.36624	0.058329
hsa_circ_	-0.51524	0.05834
hsa_circ_	0.424207	0.058341
hsa_circ_	-0.50982	0.058351
hsa_circ_	0.449641	0.058364
hsa_circ_	-0.55615	0.058365
hsa_circ_	0.601919	0.058375
hsa_circ_	-0.68047	0.058406

hsa_circ_	0.52227	0.05841
hsa_circ_	0.91307	0.058414
hsa_circ_	0.60618	0.058423
hsa_circ_	-0.44974	0.058429
hsa_circ_	-0.64464	0.058433
hsa_circ_	-0.64471	0.058438
hsa_circ_	0.658093	0.058439
hsa_circ_	2.118644	0.058449
hsa_circ_	-0.81614	0.05847
hsa_circ_	-1.33721	0.058472
hsa_circ_	0.464618	0.058474
hsa_circ_	0.449863	0.058475
hsa_circ_	0.331094	0.058479
hsa_circ_	-1.30957	0.058486
hsa_circ_	-0.40346	0.058492
hsa_circ_	-0.3573	0.058499
hsa_circ_	0.312298	0.058508
hsa_circ_	-0.45743	0.058513
hsa_circ_	-0.62368	0.058519
hsa_circ_	0.418556	0.058527
hsa_circ_	-0.47935	0.05855
hsa_circ_	0.313245	0.058558
hsa_circ_	-0.49128	0.058565
hsa_circ_	0.547408	0.058567
hsa_circ_	-0.38399	0.058569
hsa_circ_	-0.5191	0.058569
hsa_circ_	0.364893	0.05857
hsa_circ_	-0.34788	0.058571
hsa_circ_	0.366986	0.058576
hsa_circ_	0.413596	0.05858
hsa_circ_	0.374805	0.058586
hsa_circ_	-0.41026	0.058587
hsa_circ_	-0.4308	0.058595
hsa_circ_	-0.48629	0.058596
hsa_circ_	-0.29208	0.058599
hsa_circ_	0.348909	0.058608
hsa_circ_	1.231789	0.058612
hsa_circ_	-0.46652	0.058622
hsa_circ_	0.384475	0.058666
hsa_circ_	-0.38094	0.058694
hsa_circ_	0.888933	0.058697
hsa_circ_	-0.68158	0.058699
hsa_circ_	0.485507	0.058703
hsa_circ_	-0.35539	0.058709
hsa_circ_	-0.41187	0.058711
hsa_circ_	-1.47647	0.058715
hsa_circ_	0.713442	0.05872
hsa_circ_	0.394499	0.058723
hsa_circ_	0.719103	0.058723
hsa_circ_	0.365468	0.058738
hsa_circ_	0.550932	0.058742
hsa_circ_	-0.66122	0.058743
hsa_circ_	0.417992	0.05875
hsa_circ_	0.592761	0.058762

hsa_circ_	0.431617	0.058764
hsa_circ_	-0.60127	0.058785
hsa_circ_	-0.52945	0.058789
hsa_circ_	0.508556	0.058792
hsa_circ_	0.579922	0.058794
hsa_circ_	0.386273	0.058804
hsa_circ_	0.465081	0.05881
hsa_circ_	0.480722	0.058814
hsa_circ_	0.32716	0.058815
hsa_circ_	-0.52003	0.05882
hsa_circ_	-0.62485	0.058825
hsa_circ_	-0.61595	0.058828
hsa_circ_	-0.30992	0.058828
hsa_circ_	0.50922	0.05886
hsa_circ_	-0.42987	0.058869
hsa_circ_	0.397067	0.058877
hsa_circ_	-0.64739	0.058878
hsa_circ_	-0.92267	0.058885
hsa_circ_	-0.33194	0.058888
hsa_circ_	-0.77321	0.058912
hsa_circ_	-0.31677	0.058915
hsa_circ_	1.046993	0.058921
hsa_circ_	0.54678	0.058925
hsa_circ_	-0.33808	0.058931
hsa_circ_	-0.27772	0.058934
hsa_circ_	-0.3154	0.058958
hsa_circ_	1.098008	0.05896
hsa_circ_	-0.28116	0.05896
hsa_circ_	-0.42797	0.058973
hsa_circ_	-0.32112	0.058978
hsa_circ_	-1.30018	0.05898
hsa_circ_	0.567812	0.058997
hsa_circ_	-0.35605	0.058998
hsa_circ_	0.511258	0.059008
hsa_circ_	0.407985	0.059009
hsa_circ_	0.572559	0.059019
hsa_circ_	-0.50646	0.05902
hsa_circ_	-0.32315	0.059021
hsa_circ_	-0.39908	0.059022
hsa_circ_	0.678274	0.05904
hsa_circ_	-0.34419	0.059044
hsa_circ_	-0.32851	0.059045
hsa_circ_	-0.42255	0.059046
hsa_circ_	-0.50849	0.059066
hsa_circ_	1.023754	0.059066
hsa_circ_	-0.33977	0.059073
hsa_circ_	0.761584	0.059081
hsa_circ_	-0.8607	0.059094
hsa_circ_	1.291903	0.0591
hsa_circ_	0.461211	0.059107
hsa_circ_	-0.31548	0.059122
hsa_circ_	0.571077	0.059122
hsa_circ_	0.679274	0.059124
hsa_circ_	0.366994	0.059126

hsa_circ_	0.403482	0.059132
hsa_circ_	-0.29716	0.059133
hsa_circ_	-0.38922	0.059136
hsa_circ_	-0.3051	0.059138
hsa_circ_	0.353491	0.059141
hsa_circ_	-0.74843	0.059148
hsa_circ_	0.358425	0.059157
hsa_circ_	0.404607	0.059157
hsa_circ_	-0.29992	0.059162
hsa_circ_	0.420204	0.059175
hsa_circ_	-0.32449	0.05918
hsa_circ_	-0.99809	0.059185
hsa_circ_	-0.65774	0.059185
hsa_circ_	0.514219	0.059203
hsa_circ_	-0.41825	0.059217
hsa_circ_	0.305084	0.05922
hsa_circ_	-0.37246	0.059222
hsa_circ_	-0.31764	0.059224
hsa_circ_	-0.43144	0.059228
hsa_circ_	0.462351	0.059232
hsa_circ_	0.372975	0.059247
hsa_circ_	-0.29377	0.059251
hsa_circ_	0.33613	0.059258
hsa_circ_	-0.57079	0.059261
hsa_circ_	0.509288	0.059274
hsa_circ_	0.45256	0.059275
hsa_circ_	-0.98551	0.059279
hsa_circ_	0.310467	0.059282
hsa_circ_	0.435054	0.059287
hsa_circ_	-0.7529	0.05929
hsa_circ_	0.520608	0.05929
hsa_circ_	1.29831	0.059293
hsa_circ_	-0.31505	0.059294
hsa_circ_	-0.72811	0.059298
hsa_circ_	0.375937	0.059307
hsa_circ_	1.129215	0.059326
hsa_circ_	0.635105	0.059332
hsa_circ_	0.412242	0.059338
hsa_circ_	0.521082	0.059342
hsa_circ_	-0.37234	0.059363
hsa_circ_	-0.46484	0.059365
hsa_circ_	0.435645	0.059372
hsa_circ_	-0.50338	0.059384
hsa_circ_	-0.32543	0.059394
hsa_circ_	-0.30946	0.059395
hsa_circ_	0.465148	0.059409
hsa_circ_	0.739824	0.059419
hsa_circ_	0.940041	0.05942
hsa_circ_	-0.87608	0.059421
hsa_circ_	-0.84693	0.059443
hsa_circ_	-0.46068	0.059451
hsa_circ_	0.344068	0.059451
hsa_circ_	1.159557	0.059453
hsa_circ_	0.357368	0.059455

hsa_circ_	0.44062	0.059457
hsa_circ_	-0.39817	0.059464
hsa_circ_	0.407349	0.05947
hsa_circ_	0.627275	0.059482
hsa_circ_	-0.36233	0.059482
hsa_circ_	-0.61169	0.059495
hsa_circ_	0.470235	0.059516
hsa_circ_	-0.3172	0.059517
hsa_circ_	-0.70007	0.059519
hsa_circ_	-0.59102	0.059522
hsa_circ_	-0.37145	0.059537
hsa_circ_	-0.45933	0.059555
hsa_circ_	0.398236	0.059555
hsa_circ_	0.273839	0.059564
hsa_circ_	-0.97702	0.059566
hsa_circ_	-0.38231	0.059579
hsa_circ_	0.498072	0.059589
hsa_circ_	0.355173	0.059594
hsa_circ_	-0.92523	0.059595
hsa_circ_	0.295083	0.059607
hsa_circ_	-1.25544	0.059613
hsa_circ_	0.481536	0.05962
hsa_circ_	-1.26695	0.059626
hsa_circ_	0.464666	0.059627
hsa_circ_	-0.29183	0.059632
hsa_circ_	-0.53262	0.059636
hsa_circ_	0.714161	0.059642
hsa_circ_	-0.48606	0.059646
hsa_circ_	-0.62432	0.059647
hsa_circ_	-0.44792	0.059651
hsa_circ_	-0.77033	0.059652
hsa_circ_	-0.41045	0.059656
hsa_circ_	0.367959	0.059659
hsa_circ_	-0.3266	0.05966
hsa_circ_	0.416055	0.059663
hsa_circ_	-0.43002	0.059669
hsa_circ_	0.617468	0.059676
hsa_circ_	0.311043	0.059676
hsa_circ_	0.346595	0.059686
hsa_circ_	0.434432	0.059688
hsa_circ_	0.373387	0.059711
hsa_circ_	0.382318	0.059723
hsa_circ_	-0.60071	0.059733
hsa_circ_	0.321813	0.059737
hsa_circ_	1.015364	0.059747
hsa_circ_	-0.42376	0.059747
hsa_circ_	-1.12794	0.059752
hsa_circ_	0.73616	0.059758
hsa_circ_	0.818157	0.059763
hsa_circ_	-0.52047	0.059763
hsa_circ_	0.301588	0.059764
hsa_circ_	-0.38996	0.059765
hsa_circ_	-0.27414	0.059766
hsa_circ_	0.373973	0.059776

hsa_circ_	-0.38643	0.059778
hsa_circ_	0.322615	0.059781
hsa_circ_	-0.34712	0.059784
hsa_circ_	-0.31649	0.059785
hsa_circ_	0.351535	0.059794
hsa_circ_	0.610601	0.059799
hsa_circ_	0.644985	0.059808
hsa_circ_	-0.33343	0.059809
hsa_circ_	0.626296	0.059812
hsa_circ_	0.347456	0.059816
hsa_circ_	0.383826	0.059821
hsa_circ_	-0.33982	0.059827
hsa_circ_	-0.41143	0.05983
hsa_circ_	-0.27358	0.059836
hsa_circ_	0.750314	0.059843
hsa_circ_	-0.9322	0.059844
hsa_circ_	0.477039	0.059848
hsa_circ_	-0.46866	0.059853
hsa_circ_	0.2755	0.059854
hsa_circ_	-0.51527	0.059861
hsa_circ_	-0.3351	0.05987
hsa_circ_	0.327656	0.05987
hsa_circ_	0.370233	0.059874
hsa_circ_	0.466704	0.059887
hsa_circ_	0.432231	0.059892
hsa_circ_	0.322219	0.059896
hsa_circ_	1.024057	0.059919
hsa_circ_	-0.31292	0.059922
hsa_circ_	-0.3783	0.059927
hsa_circ_	-0.54952	0.059954
hsa_circ_	0.905516	0.059956
hsa_circ_	-0.41211	0.059956
hsa_circ_	-0.45722	0.059965
hsa_circ_	0.298697	0.059969
hsa_circ_	0.437898	0.059981
hsa_circ_	-0.63598	0.059982
hsa_circ_	0.469233	0.059983
hsa_circ_	0.333254	0.059983
hsa_circ_	0.525269	0.059984
hsa_circ_	-0.36552	0.059985
hsa_circ_	-0.30375	0.059993
hsa_circ_	0.77769	0.059998
hsa_circ_	0.610586	0.06001
hsa_circ_	0.475415	0.060021
hsa_circ_	0.696375	0.060022
hsa_circ_	-0.80784	0.060022
hsa_circ_	-0.42779	0.060023
hsa_circ_	0.527441	0.06003
hsa_circ_	0.419891	0.060035
hsa_circ_	-1.05314	0.06004
hsa_circ_	-0.40511	0.060044
hsa_circ_	0.285064	0.060046
hsa_circ_	-0.82831	0.060062
hsa_circ_	-0.31155	0.060067

hsa_circ_	0.75601	0.06007
hsa_circ_	0.327041	0.060084
hsa_circ_	-0.34842	0.060094
hsa_circ_	0.335103	0.060095
hsa_circ_	0.369049	0.060099
hsa_circ_	-0.57773	0.060099
hsa_circ_	-0.46516	0.060105
hsa_circ_	0.363897	0.060107
hsa_circ_	-0.57547	0.06012
hsa_circ_	-0.33963	0.060123
hsa_circ_	-0.55446	0.060127
hsa_circ_	0.537683	0.060129
hsa_circ_	-0.29783	0.060129
hsa_circ_	0.447284	0.060133
hsa_circ_	-1.13812	0.060134
hsa_circ_	0.354186	0.060135
hsa_circ_	0.37901	0.060135
hsa_circ_	-0.39636	0.060161
hsa_circ_	-1.66549	0.060165
hsa_circ_	-0.31432	0.060166
hsa_circ_	0.35087	0.060169
hsa_circ_	-0.36108	0.060174
hsa_circ_	0.519259	0.060187
hsa_circ_	-0.33473	0.060191
hsa_circ_	0.396601	0.060203
hsa_circ_	-1.1556	0.060209
hsa_circ_	0.742852	0.060213
hsa_circ_	-0.77611	0.060216
hsa_circ_	-0.48035	0.060218
hsa_circ_	-0.95816	0.060221
hsa_circ_	-0.55243	0.060228
hsa_circ_	-1.36317	0.060251
hsa_circ_	-0.71737	0.060258
hsa_circ_	-0.32685	0.060266
hsa_circ_	-0.38063	0.060277
hsa_circ_	-0.59859	0.06028
hsa_circ_	-0.44276	0.06028
hsa_circ_	1.322784	0.060307
hsa_circ_	0.298469	0.060307
hsa_circ_	-0.74258	0.060314
hsa_circ_	0.294868	0.060315
hsa_circ_	0.421922	0.060315
hsa_circ_	0.461843	0.060318
hsa_circ_	0.454394	0.060323
hsa_circ_	-1.03228	0.060327
hsa_circ_	-0.61941	0.06033
hsa_circ_	-0.32268	0.060332
hsa_circ_	-0.46503	0.060337
hsa_circ_	0.575489	0.060346
hsa_circ_	-0.29436	0.060353
hsa_circ_	-0.32884	0.060354
hsa_circ_	0.564744	0.060364
hsa_circ_	-0.72736	0.060371
hsa_circ_	-0.79212	0.060372

hsa_circ_	0.318134	0.0604
hsa_circ_	-0.33065	0.060403
hsa_circ_	-1.04267	0.060405
hsa_circ_	0.423994	0.060407
hsa_circ_	-0.78841	0.060423
hsa_circ_	0.547071	0.060424
hsa_circ_	0.621994	0.060425
hsa_circ_	0.414331	0.060425
hsa_circ_	0.292344	0.060429
hsa_circ_	0.347867	0.060434
hsa_circ_	-0.31477	0.060437
hsa_circ_	0.884728	0.060438
hsa_circ_	1.405476	0.060439
hsa_circ_	0.410199	0.060455
hsa_circ_	0.319079	0.060457
hsa_circ_	-0.50447	0.060462
hsa_circ_	-0.40299	0.060465
hsa_circ_	0.336784	0.060471
hsa_circ_	0.539143	0.060471
hsa_circ_	0.444026	0.060477
hsa_circ_	-0.32088	0.060477
hsa_circ_	0.784857	0.060483
hsa_circ_	-0.50564	0.060492
hsa_circ_	0.455649	0.060495
hsa_circ_	-0.29491	0.0605
hsa_circ_	0.727631	0.060501
hsa_circ_	0.469861	0.060501
hsa_circ_	0.527631	0.060515
hsa_circ_	-0.33271	0.060521
hsa_circ_	-0.47573	0.060538
hsa_circ_	0.346454	0.06054
hsa_circ_	0.507258	0.060545
hsa_circ_	-0.79759	0.060549
hsa_circ_	0.406112	0.06055
hsa_circ_	-0.68874	0.060553
hsa_circ_	0.363479	0.060554
hsa_circ_	-0.30428	0.060555
hsa_circ_	0.463682	0.060563
hsa_circ_	0.352988	0.060566
hsa_circ_	-0.55578	0.060577
hsa_circ_	1.05196	0.060577
hsa_circ_	0.307213	0.06058
hsa_circ_	-0.34901	0.060598
hsa_circ_	0.981002	0.060624
hsa_circ_	-0.37788	0.060633
hsa_circ_	0.958046	0.06066
hsa_circ_	0.776893	0.060662
hsa_circ_	-0.39535	0.060668
hsa_circ_	-0.34135	0.060683
hsa_circ_	-0.4374	0.060688
hsa_circ_	-0.84114	0.060693
hsa_circ_	-0.32356	0.060697
hsa_circ_	-0.45068	0.060697
hsa_circ_	-0.56183	0.060699

hsa_circ_	0.398215	0.060705
hsa_circ_	-0.37696	0.060706
hsa_circ_	0.414161	0.060707
hsa_circ_	-0.52251	0.060711
hsa_circ_	-0.33468	0.06072
hsa_circ_	-0.51184	0.060729
hsa_circ_	0.697854	0.060732
hsa_circ_	1.207008	0.060734
hsa_circ_	-0.30851	0.060741
hsa_circ_	-0.48473	0.06075
hsa_circ_	-0.69889	0.060761
hsa_circ_	-1.85651	0.060765
hsa_circ_	-0.32932	0.060767
hsa_circ_	-0.81376	0.06077
hsa_circ_	0.498487	0.060772
hsa_circ_	-1.32657	0.06078
hsa_circ_	-0.45853	0.06079
hsa_circ_	-0.4286	0.060792
hsa_circ_	0.552366	0.060793
hsa_circ_	0.345669	0.060799
hsa_circ_	-0.57342	0.060803
hsa_circ_	-0.57875	0.060816
hsa_circ_	-0.50918	0.060822
hsa_circ_	-0.38309	0.060833
hsa_circ_	-0.34234	0.060837
hsa_circ_	0.393195	0.06085
hsa_circ_	-0.61309	0.060856
hsa_circ_	0.324719	0.060862
hsa_circ_	0.279634	0.060867
hsa_circ_	0.542495	0.060868
hsa_circ_	-0.42698	0.060879
hsa_circ_	0.528641	0.060885
hsa_circ_	0.432308	0.060888
hsa_circ_	-0.3075	0.060894
hsa_circ_	0.273331	0.060896
hsa_circ_	0.407319	0.060904
hsa_circ_	-0.64796	0.060908
hsa_circ_	-0.58626	0.060923
hsa_circ_	-1.20799	0.060924
hsa_circ_	0.341834	0.060932
hsa_circ_	0.431233	0.060935
hsa_circ_	-0.4686	0.060962
hsa_circ_	-0.66717	0.060966
hsa_circ_	-0.45481	0.060968
hsa_circ_	0.34949	0.060969
hsa_circ_	-0.45528	0.060972
hsa_circ_	-0.40021	0.060989
hsa_circ_	-0.30621	0.060997
hsa_circ_	-0.30694	0.061
hsa_circ_	0.390556	0.061
hsa_circ_	0.504505	0.061003
hsa_circ_	0.481239	0.061009
hsa_circ_	0.537684	0.061013
hsa_circ_	0.512255	0.061019

hsa_circ_	-0.50178	0.061024
hsa_circ_	0.292829	0.061027
hsa_circ_	-0.77538	0.061039
hsa_circ_	-0.71567	0.061042
hsa_circ_	-0.34796	0.061048
hsa_circ_	-0.5439	0.06105
hsa_circ_	-0.53117	0.061052
hsa_circ_	-0.42855	0.061067
hsa_circ_	0.526974	0.06107
hsa_circ_	-0.29941	0.061073
hsa_circ_	-1.03112	0.061077
hsa_circ_	-0.41623	0.061081
hsa_circ_	0.374225	0.061081
hsa_circ_	-0.3993	0.061082
hsa_circ_	0.696573	0.061092
hsa_circ_	-0.51853	0.061097
hsa_circ_	0.420062	0.061106
hsa_circ_	0.339356	0.061108
hsa_circ_	-0.32966	0.061111
hsa_circ_	-0.54638	0.061111
hsa_circ_	-0.40892	0.061121
hsa_circ_	-0.31121	0.061126
hsa_circ_	0.548063	0.061128
hsa_circ_	-0.45283	0.061132
hsa_circ_	0.911018	0.061135
hsa_circ_	1.098855	0.061139
hsa_circ_	0.477881	0.061145
hsa_circ_	0.394575	0.061153
hsa_circ_	-0.33432	0.061153
hsa_circ_	0.972818	0.061157
hsa_circ_	0.564703	0.061179
hsa_circ_	0.810694	0.061189
hsa_circ_	-0.4244	0.061192
hsa_circ_	0.319132	0.061197
hsa_circ_	-0.2998	0.0612
hsa_circ_	0.693117	0.061204
hsa_circ_	-0.42986	0.061227
hsa_circ_	0.348758	0.061228
hsa_circ_	-0.52388	0.061232
hsa_circ_	0.361931	0.061234
hsa_circ_	-0.3251	0.061248
hsa_circ_	0.62079	0.061252
hsa_circ_	-0.47498	0.061259
hsa_circ_	-0.43223	0.061261
hsa_circ_	-0.47528	0.061265
hsa_circ_	-0.29657	0.061266
hsa_circ_	0.290779	0.061269
hsa_circ_	0.470924	0.061269
hsa_circ_	-0.30418	0.061277
hsa_circ_	-0.37197	0.061282
hsa_circ_	-0.49104	0.061312
hsa_circ_	-0.45049	0.061313
hsa_circ_	0.679965	0.061317
hsa_circ_	-0.32745	0.061318

hsa_circ_	0.666726	0.061328
hsa_circ_	-0.32471	0.06133
hsa_circ_	-0.33063	0.061337
hsa_circ_	-0.77689	0.061339
hsa_circ_	-0.33145	0.061341
hsa_circ_	1.186654	0.061342
hsa_circ_	0.377039	0.061347
hsa_circ_	0.523684	0.06136
hsa_circ_	-0.57561	0.061369
hsa_circ_	0.307004	0.061383
hsa_circ_	0.297025	0.061389
hsa_circ_	-1.04232	0.061391
hsa_circ_	-0.85179	0.061399
hsa_circ_	0.420869	0.061412
hsa_circ_	0.563789	0.06142
hsa_circ_	0.361136	0.061433
hsa_circ_	-0.52577	0.06144
hsa_circ_	-0.3605	0.061452
hsa_circ_	-0.37116	0.061453
hsa_circ_	0.394171	0.061464
hsa_circ_	-0.881	0.061468
hsa_circ_	0.497147	0.06147
hsa_circ_	0.437021	0.06147
hsa_circ_	-0.93948	0.061479
hsa_circ_	-0.98086	0.061481
hsa_circ_	0.597883	0.061487
hsa_circ_	0.555681	0.061489
hsa_circ_	0.404961	0.061513
hsa_circ_	-1.29663	0.061522
hsa_circ_	0.471474	0.061528
hsa_circ_	-0.6032	0.061531
hsa_circ_	0.661341	0.061538
hsa_circ_	0.300316	0.06154
hsa_circ_	0.36295	0.061552
hsa_circ_	-0.34717	0.061575
hsa_circ_	-0.39454	0.061582
hsa_circ_	-0.55459	0.061583
hsa_circ_	-0.35939	0.061587
hsa_circ_	1.196909	0.061595
hsa_circ_	-0.73041	0.061599
hsa_circ_	0.657764	0.061608
hsa_circ_	-0.33444	0.061611
hsa_circ_	-0.29417	0.061611
hsa_circ_	0.532776	0.061612
hsa_circ_	0.806315	0.061617
hsa_circ_	0.724025	0.061619
hsa_circ_	-0.52973	0.061625
hsa_circ_	-0.43582	0.061625
hsa_circ_	0.355424	0.061627
hsa_circ_	-0.35203	0.061633
hsa_circ_	0.346912	0.061634
hsa_circ_	-0.37426	0.061636
hsa_circ_	0.361755	0.061641
hsa_circ_	-0.52689	0.061647

hsa_circ_	0.328949	0.061655
hsa_circ_	1.98477	0.061665
hsa_circ_	-0.90534	0.061667
hsa_circ_	0.445962	0.061669
hsa_circ_	-0.3974	0.061674
hsa_circ_	0.680556	0.061674
hsa_circ_	-0.40534	0.061675
hsa_circ_	0.386534	0.061679
hsa_circ_	0.328788	0.061681
hsa_circ_	0.397374	0.061695
hsa_circ_	-0.40163	0.061696
hsa_circ_	0.410021	0.061704
hsa_circ_	0.831913	0.061706
hsa_circ_	0.912294	0.06172
hsa_circ_	-0.41021	0.061734
hsa_circ_	0.403547	0.061736
hsa_circ_	1.094737	0.061746
hsa_circ_	-0.40049	0.061748
hsa_circ_	0.33226	0.061748
hsa_circ_	-0.37463	0.061748
hsa_circ_	-0.61732	0.061752
hsa_circ_	-0.81553	0.061768
hsa_circ_	-0.3581	0.061774
hsa_circ_	1.074001	0.061776
hsa_circ_	0.652185	0.061789
hsa_circ_	-0.31296	0.061789
hsa_circ_	-0.57933	0.061791
hsa_circ_	-0.71443	0.061797
hsa_circ_	-0.49206	0.061805
hsa_circ_	-0.48128	0.06181
hsa_circ_	-0.32506	0.061821
hsa_circ_	-0.53843	0.061858
hsa_circ_	-0.51243	0.061866
hsa_circ_	-0.34075	0.061867
hsa_circ_	0.592421	0.061874
hsa_circ_	-0.52407	0.061874
hsa_circ_	0.459242	0.061886
hsa_circ_	-0.40411	0.0619
hsa_circ_	-0.3647	0.061905
hsa_circ_	0.299064	0.061906
hsa_circ_	-0.66198	0.061928
hsa_circ_	-0.40336	0.061934
hsa_circ_	-0.47781	0.06194
hsa_circ_	0.53609	0.061952
hsa_circ_	-0.36633	0.061956
hsa_circ_	0.339139	0.061956
hsa_circ_	0.329894	0.061957
hsa_circ_	-0.33846	0.061959
hsa_circ_	0.341178	0.061962
hsa_circ_	0.361612	0.061968
hsa_circ_	0.376497	0.061975
hsa_circ_	-0.35156	0.061976
hsa_circ_	0.516559	0.061978
hsa_circ_	0.359804	0.061983

hsa_circ_	-0.32583	0.061986
hsa_circ_	0.483559	0.061986
hsa_circ_	-0.50713	0.062
hsa_circ_	-0.38696	0.062002
hsa_circ_	-0.8359	0.062009
hsa_circ_	0.927768	0.062014
hsa_circ_	1.585516	0.062018
hsa_circ_	0.322313	0.06202
hsa_circ_	-1.01835	0.062026
hsa_circ_	0.473341	0.062039
hsa_circ_	0.406862	0.062039
hsa_circ_	-0.3801	0.062043
hsa_circ_	-0.54497	0.062045
hsa_circ_	-0.42205	0.06206
hsa_circ_	-0.79848	0.062068
hsa_circ_	-0.65704	0.062078
hsa_circ_	0.981126	0.062079
hsa_circ_	0.314145	0.06208
hsa_circ_	-0.3735	0.062091
hsa_circ_	0.983919	0.062098
hsa_circ_	-0.6923	0.062104
hsa_circ_	-0.44732	0.062125
hsa_circ_	-0.45091	0.062128
hsa_circ_	-0.3089	0.062134
hsa_circ_	-0.59519	0.062154
hsa_circ_	-0.35621	0.062157
hsa_circ_	-1.41802	0.062163
hsa_circ_	-0.7652	0.062165
hsa_circ_	0.303624	0.062167
hsa_circ_	-0.66986	0.062177
hsa_circ_	-0.42814	0.062184
hsa_circ_	-0.54469	0.062185
hsa_circ_	-0.86708	0.062186
hsa_circ_	0.916557	0.062186
hsa_circ_	0.292004	0.062189
hsa_circ_	1.61846	0.062189
hsa_circ_	0.397416	0.062198
hsa_circ_	-0.45619	0.062198
hsa_circ_	0.401818	0.06221
hsa_circ_	-0.9722	0.062212
hsa_circ_	0.424331	0.062217
hsa_circ_	-0.92442	0.062223
hsa_circ_	-1.18963	0.06223
hsa_circ_	0.336143	0.062232
hsa_circ_	-0.32155	0.062232
hsa_circ_	-0.31828	0.062234
hsa_circ_	-0.54511	0.062244
hsa_circ_	-0.49312	0.062251
hsa_circ_	-0.37415	0.062254
hsa_circ_	0.664317	0.062254
hsa_circ_	-0.30644	0.062256
hsa_circ_	0.364548	0.062261
hsa_circ_	-0.32731	0.062287
hsa_circ_	0.317784	0.062296

hsa_circ_	0.454736	0.062308
hsa_circ_	0.311808	0.062322
hsa_circ_	0.30999	0.062325
hsa_circ_	0.388851	0.062327
hsa_circ_	-0.31226	0.062328
hsa_circ_	-0.39337	0.06233
hsa_circ_	-0.35099	0.06233
hsa_circ_	-0.36599	0.062331
hsa_circ_	1.255764	0.062344
hsa_circ_	-0.95152	0.062345
hsa_circ_	-0.33655	0.062345
hsa_circ_	-0.35543	0.062349
hsa_circ_	-0.51916	0.062353
hsa_circ_	0.503464	0.062356
hsa_circ_	0.405327	0.062357
hsa_circ_	0.389508	0.062368
hsa_circ_	0.561892	0.062369
hsa_circ_	-0.30598	0.062377
hsa_circ_	0.75882	0.062383
hsa_circ_	-0.54301	0.062385
hsa_circ_	-0.6124	0.062386
hsa_circ_	-0.36124	0.062394
hsa_circ_	0.415817	0.062396
hsa_circ_	0.594265	0.062403
hsa_circ_	0.594829	0.062404
hsa_circ_	-0.49744	0.06241
hsa_circ_	0.859795	0.06241
hsa_circ_	0.297469	0.062423
hsa_circ_	-0.30332	0.062427
hsa_circ_	0.471083	0.062433
hsa_circ_	-0.80924	0.062436
hsa_circ_	0.391746	0.062438
hsa_circ_	0.537451	0.06244
hsa_circ_	-0.75892	0.062441
hsa_circ_	-0.82349	0.062446
hsa_circ_	-0.34829	0.06245
hsa_circ_	0.319693	0.06245
hsa_circ_	-0.29307	0.062455
hsa_circ_	-0.41913	0.062456
hsa_circ_	-0.34732	0.062475
hsa_circ_	0.297962	0.062479
hsa_circ_	-0.60966	0.062491
hsa_circ_	0.284409	0.062503
hsa_circ_	-0.42751	0.062507
hsa_circ_	0.349551	0.062514
hsa_circ_	-0.34036	0.062522
hsa_circ_	-0.37657	0.062523
hsa_circ_	-0.83969	0.062528
hsa_circ_	0.615215	0.062528
hsa_circ_	-0.85892	0.062536
hsa_circ_	-0.37496	0.062541
hsa_circ_	0.396988	0.062549
hsa_circ_	1.309768	0.062553
hsa_circ_	-0.48994	0.062554

hsa_circ_	-0.31172	0.062554
hsa_circ_	-0.48372	0.062559
hsa_circ_	-0.50535	0.062562
hsa_circ_	0.353885	0.062562
hsa_circ_	0.31351	0.062564
hsa_circ_	-0.57006	0.062578
hsa_circ_	-0.46327	0.062579
hsa_circ_	0.495529	0.06258
hsa_circ_	-0.39562	0.062592
hsa_circ_	-0.30927	0.062599
hsa_circ_	0.375948	0.062603
hsa_circ_	0.330333	0.062603
hsa_circ_	0.634379	0.062605
hsa_circ_	-0.27834	0.062612
hsa_circ_	-0.27453	0.062621
hsa_circ_	0.383216	0.062624
hsa_circ_	0.322553	0.062628
hsa_circ_	-0.28339	0.062638
hsa_circ_	0.491627	0.062645
hsa_circ_	0.359041	0.062649
hsa_circ_	0.577029	0.062652
hsa_circ_	-0.41901	0.062657
hsa_circ_	1.548163	0.062659
hsa_circ_	-0.37193	0.062661
hsa_circ_	-1.21819	0.062671
hsa_circ_	-0.3424	0.06268
hsa_circ_	0.35511	0.062694
hsa_circ_	0.467144	0.062701
hsa_circ_	0.352805	0.062703
hsa_circ_	0.291768	0.062721
hsa_circ_	0.445851	0.062722
hsa_circ_	-0.3862	0.062729
hsa_circ_	-0.37768	0.062729
hsa_circ_	0.427498	0.062744
hsa_circ_	-0.41053	0.062755
hsa_circ_	-0.69519	0.062757
hsa_circ_	0.518204	0.062769
hsa_circ_	-0.30418	0.062782
hsa_circ_	-2.15781	0.062792
hsa_circ_	-0.53787	0.062829
hsa_circ_	-0.30212	0.062831
hsa_circ_	-1.13349	0.062836
hsa_circ_	0.793161	0.062841
hsa_circ_	-0.70328	0.062842
hsa_circ_	0.325494	0.062844
hsa_circ_	1.120644	0.062852
hsa_circ_	0.348777	0.062867
hsa_circ_	-0.33466	0.062868
hsa_circ_	-0.47091	0.062871
hsa_circ_	0.723691	0.062892
hsa_circ_	-1.098	0.062898
hsa_circ_	-0.33737	0.062915
hsa_circ_	1.323547	0.062919
hsa_circ_	-0.37715	0.062922

hsa_circ_	-0.43202	0.062924
hsa_circ_	-0.30752	0.062938
hsa_circ_	0.740207	0.062941
hsa_circ_	0.515181	0.062941
hsa_circ_	-0.43786	0.062943
hsa_circ_	-1.00794	0.06295
hsa_circ_	-0.52835	0.062952
hsa_circ_	0.476659	0.062958
hsa_circ_	0.327736	0.062969
hsa_circ_	0.681995	0.062984
hsa_circ_	0.980224	0.062992
hsa_circ_	0.382529	0.062996
hsa_circ_	-0.66504	0.063007
hsa_circ_	0.602555	0.063008
hsa_circ_	0.270249	0.063008
hsa_circ_	-0.46012	0.063016
hsa_circ_	-0.30018	0.063018
hsa_circ_	0.56116	0.063034
hsa_circ_	-0.34408	0.063034
hsa_circ_	0.569491	0.063038
hsa_circ_	-0.4379	0.063039
hsa_circ_	-0.65398	0.063053
hsa_circ_	-0.28299	0.063065
hsa_circ_	0.341394	0.063073
hsa_circ_	-0.32103	0.063073
hsa_circ_	-0.30449	0.063087
hsa_circ_	0.685117	0.063094
hsa_circ_	0.404862	0.0631
hsa_circ_	-0.76645	0.063112
hsa_circ_	-0.3664	0.06312
hsa_circ_	0.400792	0.063121
hsa_circ_	0.764008	0.063124
hsa_circ_	0.690315	0.063135
hsa_circ_	-0.49736	0.063137
hsa_circ_	-0.48774	0.063139
hsa_circ_	0.543548	0.063144
hsa_circ_	0.34688	0.063144
hsa_circ_	0.350462	0.06315
hsa_circ_	0.397943	0.06315
hsa_circ_	-0.53402	0.063157
hsa_circ_	0.342248	0.06316
hsa_circ_	-0.30312	0.063167
hsa_circ_	-0.28585	0.063171
hsa_circ_	0.523424	0.063175
hsa_circ_	0.43483	0.063179
hsa_circ_	-0.40341	0.063189
hsa_circ_	-0.41411	0.063197
hsa_circ_	-0.44687	0.063199
hsa_circ_	0.977372	0.0632
hsa_circ_	-0.53643	0.063209
hsa_circ_	-0.31995	0.063216
hsa_circ_	0.383951	0.063216
hsa_circ_	-0.29405	0.063229
hsa_circ_	0.40103	0.06323

hsa_circ_	-0.37747	0.063233
hsa_circ_	-0.44409	0.063241
hsa_circ_	-0.5334	0.063249
hsa_circ_	0.315274	0.063252
hsa_circ_	-0.75406	0.063253
hsa_circ_	-0.72145	0.063257
hsa_circ_	-0.44053	0.063273
hsa_circ_	-0.32156	0.063281
hsa_circ_	0.346928	0.0633
hsa_circ_	0.477835	0.063301
hsa_circ_	-0.41884	0.063304
hsa_circ_	0.556352	0.063305
hsa_circ_	1.248558	0.06332
hsa_circ_	0.347971	0.063323
hsa_circ_	-0.36743	0.063325
hsa_circ_	0.709806	0.063328
hsa_circ_	-1.17286	0.063338
hsa_circ_	0.407162	0.063339
hsa_circ_	0.404757	0.063353
hsa_circ_	-0.41963	0.063363
hsa_circ_	-0.50218	0.06337
hsa_circ_	-0.2849	0.063377
hsa_circ_	0.308135	0.06339
hsa_circ_	0.444902	0.063406
hsa_circ_	-0.38939	0.063407
hsa_circ_	-0.50691	0.063414
hsa_circ_	-0.32738	0.063415
hsa_circ_	0.74225	0.063416
hsa_circ_	0.652806	0.063428
hsa_circ_	-0.28944	0.063434
hsa_circ_	-0.51423	0.063435
hsa_circ_	0.508429	0.063436
hsa_circ_	0.373071	0.063442
hsa_circ_	-0.75084	0.063443
hsa_circ_	-1.14547	0.06346
hsa_circ_	1.199802	0.063468
hsa_circ_	-0.59472	0.06347
hsa_circ_	-1.0185	0.063471
hsa_circ_	0.35411	0.063482
hsa_circ_	-0.3886	0.063488
hsa_circ_	0.624495	0.063488
hsa_circ_	0.883938	0.063496
hsa_circ_	-0.64954	0.063509
hsa_circ_	0.704292	0.063511
hsa_circ_	0.385935	0.063526
hsa_circ_	-0.32407	0.063535
hsa_circ_	0.331355	0.06354
hsa_circ_	-0.46417	0.063541
hsa_circ_	1.367645	0.063551
hsa_circ_	0.93925	0.063552
hsa_circ_	-2.01849	0.063555
hsa_circ_	-0.5883	0.063565
hsa_circ_	-0.54304	0.063578
hsa_circ_	-1.07487	0.063581

hsa_circ_	0.468579	0.063594
hsa_circ_	0.372188	0.06361
hsa_circ_	0.31572	0.06361
hsa_circ_	-0.29946	0.063628
hsa_circ_	0.539305	0.063631
hsa_circ_	0.556603	0.063635
hsa_circ_	-0.34604	0.063639
hsa_circ_	0.782045	0.063642
hsa_circ_	0.468491	0.063649
hsa_circ_	-0.32421	0.063654
hsa_circ_	0.378759	0.063655
hsa_circ_	0.542707	0.063661
hsa_circ_	-0.4445	0.063666
hsa_circ_	-0.43667	0.063673
hsa_circ_	0.492852	0.06369
hsa_circ_	-1.53223	0.063692
hsa_circ_	-0.31272	0.063696
hsa_circ_	0.347036	0.0637
hsa_circ_	0.387005	0.063707
hsa_circ_	0.729621	0.063709
hsa_circ_	0.332596	0.06371
hsa_circ_	0.49482	0.063724
hsa_circ_	-0.39782	0.06375
hsa_circ_	0.92595	0.063752
hsa_circ_	-0.5361	0.063764
hsa_circ_	0.673615	0.063764
hsa_circ_	0.565867	0.063765
hsa_circ_	-0.3782	0.063765
hsa_circ_	0.613441	0.063768
hsa_circ_	-0.42754	0.063768
hsa_circ_	-0.86061	0.063772
hsa_circ_	0.649728	0.063773
hsa_circ_	0.77994	0.063782
hsa_circ_	-0.66096	0.063797
hsa_circ_	0.362646	0.063802
hsa_circ_	-0.29623	0.063804
hsa_circ_	0.389025	0.063813
hsa_circ_	0.322756	0.063815
hsa_circ_	0.364664	0.063819
hsa_circ_	-1.32264	0.063819
hsa_circ_	-0.62214	0.063823
hsa_circ_	-0.36218	0.063823
hsa_circ_	-0.32039	0.063828
hsa_circ_	-0.51737	0.063838
hsa_circ_	0.401833	0.063848
hsa_circ_	0.334811	0.06386
hsa_circ_	-0.70646	0.063864
hsa_circ_	-0.2969	0.063877
hsa_circ_	0.502609	0.063887
hsa_circ_	-0.42982	0.063891
hsa_circ_	-1.21107	0.063894
hsa_circ_	-0.35188	0.063895
hsa_circ_	-0.29714	0.063903
hsa_circ_	0.419109	0.063907

hsa_circ_	0.530587	0.063912
hsa_circ_	0.442595	0.063913
hsa_circ_	0.412018	0.063935
hsa_circ_	0.461359	0.06395
hsa_circ_	0.803615	0.063951
hsa_circ_	-0.57909	0.063957
hsa_circ_	1.261557	0.063972
hsa_circ_	0.960047	0.063982
hsa_circ_	0.989516	0.063985
hsa_circ_	0.618007	0.063999
hsa_circ_	0.344703	0.064018
hsa_circ_	-0.3469	0.064023
hsa_circ_	0.590134	0.064024
hsa_circ_	-0.52479	0.064029
hsa_circ_	-0.31199	0.064041
hsa_circ_	0.369857	0.064042
hsa_circ_	0.658791	0.064046
hsa_circ_	0.675422	0.064046
hsa_circ_	-0.33165	0.064049
hsa_circ_	0.4333	0.064053
hsa_circ_	-0.58669	0.064054
hsa_circ_	-0.61643	0.064054
hsa_circ_	-0.29011	0.064056
hsa_circ_	0.604217	0.064059
hsa_circ_	-0.38808	0.06406
hsa_circ_	0.497336	0.064061
hsa_circ_	-0.94625	0.064086
hsa_circ_	0.485406	0.064095
hsa_circ_	0.447296	0.0641
hsa_circ_	0.36338	0.06412
hsa_circ_	-0.37924	0.064123
hsa_circ_	0.385238	0.064129
hsa_circ_	-0.32071	0.06413
hsa_circ_	-0.30407	0.064136
hsa_circ_	-0.47376	0.06414
hsa_circ_	-0.48226	0.064144
hsa_circ_	-0.38035	0.064153
hsa_circ_	-0.53398	0.064153
hsa_circ_	0.523271	0.064163
hsa_circ_	0.581994	0.064172
hsa_circ_	1.158848	0.064173
hsa_circ_	0.500871	0.064186
hsa_circ_	-0.5923	0.064191
hsa_circ_	0.268353	0.06421
hsa_circ_	0.312528	0.064211
hsa_circ_	0.667841	0.064219
hsa_circ_	-0.39768	0.064223
hsa_circ_	1.264314	0.064226
hsa_circ_	0.366418	0.064233
hsa_circ_	-0.84075	0.064234
hsa_circ_	0.729121	0.064235
hsa_circ_	0.302434	0.064244
hsa_circ_	0.492158	0.06426
hsa_circ_	0.361146	0.06427

hsa_circ_	-0.35789	0.064276
hsa_circ_	0.400373	0.064289
hsa_circ_	0.375895	0.064294
hsa_circ_	0.418196	0.064295
hsa_circ_	-0.43419	0.064308
hsa_circ_	-0.44833	0.064309
hsa_circ_	0.513858	0.064329
hsa_circ_	0.760166	0.064334
hsa_circ_	-0.60632	0.064338
hsa_circ_	-0.47963	0.064338
hsa_circ_	-0.68777	0.064356
hsa_circ_	-0.9866	0.064356
hsa_circ_	-0.52946	0.064358
hsa_circ_	-0.3097	0.064359
hsa_circ_	0.36418	0.064374
hsa_circ_	-1.5755	0.064377
hsa_circ_	0.290501	0.064385
hsa_circ_	-0.36509	0.064386
hsa_circ_	-0.44604	0.064394
hsa_circ_	0.469327	0.064394
hsa_circ_	0.690827	0.064394
hsa_circ_	-0.3158	0.064396
hsa_circ_	-0.3583	0.064405
hsa_circ_	0.424671	0.064407
hsa_circ_	-0.28853	0.064422
hsa_circ_	-0.48769	0.064424
hsa_circ_	0.378484	0.064433
hsa_circ_	-0.68132	0.064434
hsa_circ_	0.483351	0.064438
hsa_circ_	-0.46892	0.06444
hsa_circ_	0.340459	0.064462
hsa_circ_	-0.37575	0.064463
hsa_circ_	0.861862	0.064463
hsa_circ_	0.659371	0.064468
hsa_circ_	0.425209	0.064475
hsa_circ_	-0.33268	0.064487
hsa_circ_	-0.45712	0.064493
hsa_circ_	0.438498	0.064518
hsa_circ_	-0.50723	0.064548
hsa_circ_	-0.52586	0.064553
hsa_circ_	-0.3086	0.064556
hsa_circ_	0.564946	0.064558
hsa_circ_	0.422593	0.064561
hsa_circ_	-0.41018	0.064568
hsa_circ_	-0.47277	0.064578
hsa_circ_	0.578454	0.064581
hsa_circ_	-0.78884	0.064585
hsa_circ_	0.483138	0.064588
hsa_circ_	0.719765	0.06459
hsa_circ_	0.486723	0.064599
hsa_circ_	-0.43525	0.064604
hsa_circ_	-0.37759	0.064615
hsa_circ_	-0.30012	0.064616
hsa_circ_	-0.27464	0.064617

hsa_circ_	-0.84712	0.064633
hsa_circ_	-0.72289	0.064639
hsa_circ_	-0.99876	0.064661
hsa_circ_	0.691754	0.06467
hsa_circ_	0.497928	0.064677
hsa_circ_	0.983188	0.064684
hsa_circ_	1.245145	0.064695
hsa_circ_	-0.55707	0.0647
hsa_circ_	0.860751	0.064706
hsa_circ_	0.541296	0.064708
hsa_circ_	-0.65239	0.06471
hsa_circ_	-0.54785	0.064711
hsa_circ_	0.64929	0.064728
hsa_circ_	-0.32119	0.064733
hsa_circ_	-0.45358	0.064741
hsa_circ_	0.646494	0.064742
hsa_circ_	-0.36265	0.064746
hsa_circ_	-0.68631	0.064748
hsa_circ_	0.364885	0.06475
hsa_circ_	0.364507	0.064754
hsa_circ_	-0.3184	0.064778
hsa_circ_	0.509746	0.064784
hsa_circ_	0.439603	0.064784
hsa_circ_	0.416011	0.064803
hsa_circ_	0.419866	0.064805
hsa_circ_	0.333022	0.064827
hsa_circ_	0.344187	0.064828
hsa_circ_	0.340367	0.064829
hsa_circ_	-0.40449	0.064852
hsa_circ_	0.578282	0.064855
hsa_circ_	0.53834	0.064868
hsa_circ_	0.312976	0.06487
hsa_circ_	0.962604	0.064884
hsa_circ_	-0.78953	0.064888
hsa_circ_	0.671936	0.06489
hsa_circ_	0.273195	0.064893
hsa_circ_	-0.67763	0.064893
hsa_circ_	-0.74299	0.064896
hsa_circ_	0.304282	0.064925
hsa_circ_	0.390708	0.064936
hsa_circ_	-0.48904	0.064947
hsa_circ_	0.307469	0.064947
hsa_circ_	-0.30705	0.064948
hsa_circ_	0.335415	0.06496
hsa_circ_	-0.31292	0.064963
hsa_circ_	0.752841	0.064963
hsa_circ_	0.391584	0.064967
hsa_circ_	-0.31007	0.06497
hsa_circ_	0.335177	0.064986
hsa_circ_	-0.42198	0.064995
hsa_circ_	-0.99737	0.064996
hsa_circ_	-0.39891	0.065007
hsa_circ_	0.356544	0.065024
hsa_circ_	-0.7352	0.06504

hsa_circ_	-0.33692	0.065054
hsa_circ_	-0.31033	0.065057
hsa_circ_	0.37313	0.065062
hsa_circ_	0.964022	0.065065
hsa_circ_	-0.32865	0.065088
hsa_circ_	-0.85075	0.06509
hsa_circ_	0.474031	0.065096
hsa_circ_	-0.46366	0.065109
hsa_circ_	0.299027	0.065112
hsa_circ_	0.567241	0.065118
hsa_circ_	-0.63596	0.065118
hsa_circ_	0.393735	0.065135
hsa_circ_	0.586152	0.065142
hsa_circ_	-0.39239	0.065144
hsa_circ_	0.294234	0.065144
hsa_circ_	0.884466	0.065146
hsa_circ_	-0.88147	0.065149
hsa_circ_	0.57923	0.065155
hsa_circ_	-0.47236	0.065169
hsa_circ_	-0.52853	0.065172
hsa_circ_	0.694976	0.065185
hsa_circ_	-0.91673	0.065198
hsa_circ_	0.41106	0.065213
hsa_circ_	-0.36023	0.065215
hsa_circ_	-0.44303	0.065217
hsa_circ_	0.770626	0.065224
hsa_circ_	-0.50911	0.065225
hsa_circ_	-0.43163	0.065229
hsa_circ_	-0.7132	0.065231
hsa_circ_	-0.29047	0.065232
hsa_circ_	-0.41067	0.065251
hsa_circ_	-0.42376	0.065269
hsa_circ_	1.049269	0.065275
hsa_circ_	0.605062	0.065283
hsa_circ_	0.468058	0.065284
hsa_circ_	0.420152	0.065287
hsa_circ_	0.496072	0.065295
hsa_circ_	-0.36307	0.065295
hsa_circ_	0.721222	0.065299
hsa_circ_	-0.72168	0.065309
hsa_circ_	-1.34974	0.065314
hsa_circ_	-0.33252	0.06532
hsa_circ_	0.338843	0.065321
hsa_circ_	0.337647	0.065336
hsa_circ_	0.322879	0.065343
hsa_circ_	0.544695	0.065354
hsa_circ_	-0.37625	0.065367
hsa_circ_	-0.29791	0.06537
hsa_circ_	-2.05862	0.065384
hsa_circ_	0.428454	0.065393
hsa_circ_	-0.49535	0.065394
hsa_circ_	-0.53226	0.0654
hsa_circ_	-0.56795	0.065406
hsa_circ_	0.45376	0.065411

hsa_circ_	0.521882	0.065425
hsa_circ_	0.642753	0.065425
hsa_circ_	-0.28874	0.06543
hsa_circ_	0.490093	0.065445
hsa_circ_	0.31838	0.065452
hsa_circ_	-0.41054	0.065463
hsa_circ_	-0.38834	0.065464
hsa_circ_	-0.68038	0.065465
hsa_circ_	-0.55197	0.065468
hsa_circ_	0.347993	0.065468
hsa_circ_	0.475538	0.065476
hsa_circ_	0.354265	0.065483
hsa_circ_	0.293546	0.065501
hsa_circ_	-1.13634	0.065507
hsa_circ_	-0.50089	0.065524
hsa_circ_	-0.47866	0.065526
hsa_circ_	-0.48507	0.065529
hsa_circ_	0.28419	0.06553
hsa_circ_	0.267158	0.065535
hsa_circ_	0.366959	0.065557
hsa_circ_	-0.53817	0.065558
hsa_circ_	0.35807	0.065572
hsa_circ_	0.418589	0.065575
hsa_circ_	-0.34046	0.065578
hsa_circ_	-0.32835	0.065582
hsa_circ_	0.294857	0.065591
hsa_circ_	-0.39787	0.065598
hsa_circ_	-1.13793	0.065601
hsa_circ_	-0.66572	0.065612
hsa_circ_	-0.44767	0.065615
hsa_circ_	-0.32549	0.06562
hsa_circ_	0.527907	0.065629
hsa_circ_	0.697115	0.065634
hsa_circ_	0.657229	0.065634
hsa_circ_	0.610651	0.065643
hsa_circ_	-0.3931	0.065646
hsa_circ_	-0.94233	0.065683
hsa_circ_	-0.52928	0.065689
hsa_circ_	0.312102	0.065695
hsa_circ_	-0.32551	0.0657
hsa_circ_	0.294738	0.065701
hsa_circ_	0.316374	0.065702
hsa_circ_	0.38029	0.06571
hsa_circ_	-0.37335	0.065719
hsa_circ_	-0.29836	0.065721
hsa_circ_	0.310576	0.06573
hsa_circ_	-0.58718	0.06574
hsa_circ_	-0.38367	0.065741
hsa_circ_	-0.46221	0.065744
hsa_circ_	0.385178	0.065769
hsa_circ_	-0.53759	0.065769
hsa_circ_	-0.45407	0.065772
hsa_circ_	-0.32427	0.065777
hsa_circ_	0.603589	0.065782

hsa_circ_	-0.60364	0.06579
hsa_circ_	-0.66119	0.065797
hsa_circ_	0.393656	0.065798
hsa_circ_	-0.8177	0.065798
hsa_circ_	-0.48887	0.065808
hsa_circ_	-0.28311	0.06581
hsa_circ_	-0.36202	0.06581
hsa_circ_	0.439227	0.065814
hsa_circ_	-0.46802	0.065827
hsa_circ_	0.538872	0.065828
hsa_circ_	0.363815	0.06583
hsa_circ_	0.739853	0.065832
hsa_circ_	-0.4534	0.065838
hsa_circ_	0.369763	0.065838
hsa_circ_	-0.33239	0.065844
hsa_circ_	-0.45094	0.06585
hsa_circ_	-0.40049	0.065851
hsa_circ_	0.769788	0.065863
hsa_circ_	0.313339	0.065869
hsa_circ_	-0.34838	0.065873
hsa_circ_	-0.29565	0.065879
hsa_circ_	0.487712	0.065882
hsa_circ_	0.816666	0.065896
hsa_circ_	0.496127	0.0659
hsa_circ_	-0.94643	0.065913
hsa_circ_	-0.30202	0.065921
hsa_circ_	0.359969	0.065922
hsa_circ_	-0.50929	0.065922
hsa_circ_	-0.90678	0.065925
hsa_circ_	-0.27323	0.065927
hsa_circ_	-0.41004	0.065937
hsa_circ_	-0.3198	0.065961
hsa_circ_	0.535153	0.065963
hsa_circ_	0.638666	0.065971
hsa_circ_	0.390218	0.065984
hsa_circ_	-0.5837	0.065989
hsa_circ_	0.518379	0.06599
hsa_circ_	-0.41033	0.065995
hsa_circ_	0.441727	0.066019
hsa_circ_	-0.36511	0.066021
hsa_circ_	-0.29679	0.066032
hsa_circ_	-0.4157	0.066041
hsa_circ_	0.51083	0.066053
hsa_circ_	0.737746	0.066054
hsa_circ_	-0.36795	0.066058
hsa_circ_	0.336731	0.066059
hsa_circ_	-0.35097	0.066064
hsa_circ_	0.30564	0.066065
hsa_circ_	0.307824	0.06607
hsa_circ_	-0.30576	0.066103
hsa_circ_	0.306156	0.066107
hsa_circ_	0.325199	0.066108
hsa_circ_	0.538785	0.066116
hsa_circ_	-0.75206	0.066119

hsa_circ_	0.521174	0.06612
hsa_circ_	-0.40726	0.066131
hsa_circ_	0.451905	0.066132
hsa_circ_	-0.29536	0.066141
hsa_circ_	0.835571	0.066145
hsa_circ_	-0.36332	0.06616
hsa_circ_	-0.30179	0.06616
hsa_circ_	-0.57036	0.066162
hsa_circ_	-0.46921	0.066178
hsa_circ_	-0.76463	0.066192
hsa_circ_	-1.08156	0.066194
hsa_circ_	0.635001	0.066214
hsa_circ_	-0.76265	0.066229
hsa_circ_	-0.47183	0.06623
hsa_circ_	-0.39728	0.066233
hsa_circ_	0.571856	0.066236
hsa_circ_	0.457003	0.066246
hsa_circ_	-0.93934	0.066254
hsa_circ_	-0.2965	0.066262
hsa_circ_	-0.3915	0.066264
hsa_circ_	-1.3715	0.06627
hsa_circ_	0.43285	0.066272
hsa_circ_	-0.30663	0.066274
hsa_circ_	0.307654	0.066278
hsa_circ_	0.631832	0.066283
hsa_circ_	-0.43342	0.066284
hsa_circ_	1.560303	0.066294
hsa_circ_	0.803861	0.066309
hsa_circ_	-0.89448	0.066312
hsa_circ_	-0.40374	0.066318
hsa_circ_	-0.30813	0.066319
hsa_circ_	-0.37905	0.066319
hsa_circ_	-0.38535	0.06632
hsa_circ_	0.328362	0.066324
hsa_circ_	-0.29666	0.066328
hsa_circ_	0.812992	0.06633
hsa_circ_	-0.92475	0.066334
hsa_circ_	-0.34712	0.066334
hsa_circ_	-0.43741	0.06634
hsa_circ_	-0.5829	0.066344
hsa_circ_	-0.29023	0.066345
hsa_circ_	0.541134	0.066365
hsa_circ_	-1.12335	0.066368
hsa_circ_	-1.26816	0.066378
hsa_circ_	-0.41539	0.066383
hsa_circ_	0.338915	0.066386
hsa_circ_	0.442785	0.06639
hsa_circ_	0.937318	0.066395
hsa_circ_	1.324222	0.066407
hsa_circ_	-0.31468	0.066408
hsa_circ_	0.322304	0.066414
hsa_circ_	0.358567	0.066417
hsa_circ_	-0.53003	0.066419
hsa_circ_	-0.27527	0.066421

hsa_circ_	0.506442	0.066422
hsa_circ_	-0.36695	0.066424
hsa_circ_	0.353288	0.066427
hsa_circ_	-0.31307	0.06644
hsa_circ_	0.397041	0.066441
hsa_circ_	0.456831	0.066443
hsa_circ_	-0.315	0.066444
hsa_circ_	-0.27667	0.066457
hsa_circ_	-0.47631	0.066464
hsa_circ_	-0.3289	0.06649
hsa_circ_	-0.46298	0.066497
hsa_circ_	-0.58659	0.0665
hsa_circ_	-0.41513	0.066501
hsa_circ_	0.443934	0.066514
hsa_circ_	-1.56282	0.066518
hsa_circ_	-0.37696	0.066521
hsa_circ_	0.428692	0.066535
hsa_circ_	-0.39248	0.066537
hsa_circ_	0.435277	0.066537
hsa_circ_	-0.38825	0.066545
hsa_circ_	0.6837	0.066565
hsa_circ_	-0.49773	0.066566
hsa_circ_	0.41283	0.06657
hsa_circ_	0.77949	0.066582
hsa_circ_	-0.6436	0.066594
hsa_circ_	0.463536	0.066595
hsa_circ_	-0.35136	0.06662
hsa_circ_	0.961875	0.066623
hsa_circ_	-1.0083	0.066623
hsa_circ_	-0.34685	0.066639
hsa_circ_	-0.46163	0.066641
hsa_circ_	-1.32127	0.066652
hsa_circ_	0.558693	0.066654
hsa_circ_	0.547191	0.066656
hsa_circ_	-0.47037	0.06666
hsa_circ_	-0.80715	0.066674
hsa_circ_	0.297312	0.066676
hsa_circ_	1.523727	0.066681
hsa_circ_	-0.31878	0.066699
hsa_circ_	-0.39424	0.066707
hsa_circ_	-0.37407	0.066709
hsa_circ_	0.406344	0.066717
hsa_circ_	0.266535	0.066722
hsa_circ_	0.749572	0.066728
hsa_circ_	0.728479	0.06673
hsa_circ_	-0.3264	0.066732
hsa_circ_	0.421883	0.066736
hsa_circ_	0.536695	0.066736
hsa_circ_	-0.2827	0.066744
hsa_circ_	-0.71202	0.066745
hsa_circ_	-0.41056	0.066751
hsa_circ_	0.459589	0.06677
hsa_circ_	0.277955	0.066773
hsa_circ_	0.376404	0.066774

hsa_circ_	0.347085	0.066779
hsa_circ_	-1.08136	0.066793
hsa_circ_	0.348733	0.066797
hsa_circ_	-0.86448	0.066808
hsa_circ_	0.732512	0.066811
hsa_circ_	0.643786	0.066813
hsa_circ_	0.515599	0.066828
hsa_circ_	-0.42001	0.066829
hsa_circ_	1.393577	0.066835
hsa_circ_	0.334556	0.066838
hsa_circ_	0.346308	0.066845
hsa_circ_	0.823741	0.06686
hsa_circ_	-0.28497	0.066878
hsa_circ_	0.732202	0.066884
hsa_circ_	-0.41132	0.066893
hsa_circ_	-0.2826	0.066893
hsa_circ_	0.427706	0.066894
hsa_circ_	-0.50189	0.066901
hsa_circ_	0.511679	0.066901
hsa_circ_	0.423672	0.066902
hsa_circ_	-0.38752	0.066903
hsa_circ_	0.873798	0.066903
hsa_circ_	-0.4364	0.066916
hsa_circ_	-1.05376	0.066928
hsa_circ_	-0.94328	0.066932
hsa_circ_	0.954983	0.066934
hsa_circ_	-0.82816	0.066949
hsa_circ_	-0.39039	0.066952
hsa_circ_	0.350298	0.066954
hsa_circ_	0.529352	0.066958
hsa_circ_	0.330081	0.066963
hsa_circ_	1.11802	0.066973
hsa_circ_	-0.73467	0.066974
hsa_circ_	-0.43457	0.066974
hsa_circ_	-0.45246	0.066978
hsa_circ_	-1.07898	0.066984
hsa_circ_	0.423104	0.066996
hsa_circ_	0.761098	0.067
hsa_circ_	0.300692	0.067015
hsa_circ_	-0.42741	0.067018
hsa_circ_	0.720483	0.067032
hsa_circ_	0.471936	0.067033
hsa_circ_	-0.41245	0.067043
hsa_circ_	0.481623	0.067047
hsa_circ_	0.350135	0.067053
hsa_circ_	0.426086	0.067053
hsa_circ_	-0.34264	0.067055
hsa_circ_	-0.30332	0.067058
hsa_circ_	0.360273	0.067059
hsa_circ_	0.515816	0.067063
hsa_circ_	-0.66422	0.067071
hsa_circ_	0.94168	0.067087
hsa_circ_	0.473445	0.06709
hsa_circ_	-0.35294	0.067095

hsa_circ_	-0.29237	0.067099
hsa_circ_	0.283995	0.067099
hsa_circ_	-0.40408	0.0671
hsa_circ_	0.314359	0.067103
hsa_circ_	-1.10901	0.067108
hsa_circ_	0.585241	0.067113
hsa_circ_	-0.45684	0.067124
hsa_circ_	0.642714	0.067132
hsa_circ_	-0.26497	0.067135
hsa_circ_	0.501755	0.067136
hsa_circ_	0.591817	0.067162
hsa_circ_	0.880678	0.067163
hsa_circ_	-0.30727	0.067169
hsa_circ_	-0.31462	0.067177
hsa_circ_	-0.5325	0.067189
hsa_circ_	-0.33367	0.067198
hsa_circ_	0.457852	0.06721
hsa_circ_	-0.43794	0.067216
hsa_circ_	0.387828	0.067225
hsa_circ_	0.371363	0.067227
hsa_circ_	0.372993	0.067232
hsa_circ_	-0.38593	0.067262
hsa_circ_	0.361298	0.067267
hsa_circ_	0.264393	0.067277
hsa_circ_	-0.44473	0.067285
hsa_circ_	-0.28108	0.067286
hsa_circ_	0.897566	0.067288
hsa_circ_	-0.43358	0.067288
hsa_circ_	0.791648	0.067294
hsa_circ_	-0.84186	0.067307
hsa_circ_	0.629922	0.067311
hsa_circ_	-0.39765	0.067322
hsa_circ_	-0.48249	0.067331
hsa_circ_	-0.6002	0.067333
hsa_circ_	0.60218	0.067338
hsa_circ_	0.331561	0.067354
hsa_circ_	-0.28995	0.067357
hsa_circ_	0.570373	0.067361
hsa_circ_	0.437448	0.067363
hsa_circ_	0.389502	0.067369
hsa_circ_	-0.4012	0.067382
hsa_circ_	0.627798	0.067385
hsa_circ_	-0.33514	0.067393
hsa_circ_	-0.65358	0.067401
hsa_circ_	0.48982	0.067404
hsa_circ_	-0.60391	0.067406
hsa_circ_	0.735521	0.067409
hsa_circ_	-0.69364	0.067413
hsa_circ_	0.477234	0.06742
hsa_circ_	-0.2695	0.067443
hsa_circ_	-0.29377	0.06746
hsa_circ_	-0.4364	0.067462
hsa_circ_	0.317079	0.067465
hsa_circ_	0.439292	0.067465

hsa_circ_	0.55459	0.067465
hsa_circ_	0.847404	0.067472
hsa_circ_	0.306996	0.067481
hsa_circ_	-0.4805	0.067492
hsa_circ_	0.515694	0.067501
hsa_circ_	-0.44604	0.067505
hsa_circ_	0.302639	0.067506
hsa_circ_	0.434133	0.067522
hsa_circ_	-0.53568	0.067526
hsa_circ_	0.603326	0.067534
hsa_circ_	-0.47077	0.067549
hsa_circ_	1.322122	0.067554
hsa_circ_	-0.38148	0.067554
hsa_circ_	0.414477	0.067557
hsa_circ_	-0.40755	0.067566
hsa_circ_	-0.4314	0.067571
hsa_circ_	-0.29366	0.067572
hsa_circ_	0.6133	0.067575
hsa_circ_	0.317666	0.067581
hsa_circ_	0.630456	0.067584
hsa_circ_	0.401709	0.067585
hsa_circ_	-0.32078	0.067609
hsa_circ_	-0.39074	0.06761
hsa_circ_	0.336195	0.067613
hsa_circ_	0.327819	0.067614
hsa_circ_	-0.36003	0.067622
hsa_circ_	0.664486	0.067625
hsa_circ_	0.641021	0.067639
hsa_circ_	-0.45684	0.067647
hsa_circ_	0.523128	0.067679
hsa_circ_	-0.62614	0.06769
hsa_circ_	-0.34597	0.067693
hsa_circ_	-0.64257	0.067695
hsa_circ_	0.48966	0.067705
hsa_circ_	0.432762	0.067707
hsa_circ_	-0.31965	0.067721
hsa_circ_	-0.4044	0.067722
hsa_circ_	-0.48466	0.067745
hsa_circ_	-0.44432	0.067769
hsa_circ_	-0.29715	0.067773
hsa_circ_	0.301693	0.067774
hsa_circ_	-0.26801	0.067785
hsa_circ_	0.326649	0.067806
hsa_circ_	-0.48645	0.067808
hsa_circ_	0.431962	0.067817
hsa_circ_	0.407082	0.067818
hsa_circ_	-0.28437	0.067823
hsa_circ_	-0.68255	0.067827
hsa_circ_	-0.30716	0.067831
hsa_circ_	-0.97997	0.067838
hsa_circ_	0.296905	0.067844
hsa_circ_	0.366643	0.067847
hsa_circ_	-0.30143	0.067854
hsa_circ_	0.600299	0.067866

hsa_circ_	0.415007	0.067866
hsa_circ_	-0.65667	0.067884
hsa_circ_	0.71579	0.06789
hsa_circ_	-0.48184	0.067895
hsa_circ_	-0.28041	0.067902
hsa_circ_	-0.48258	0.067904
hsa_circ_	0.472231	0.067904
hsa_circ_	-0.6609	0.067906
hsa_circ_	0.449856	0.067917
hsa_circ_	0.494021	0.067926
hsa_circ_	0.488292	0.067929
hsa_circ_	0.407219	0.067929
hsa_circ_	-1.35648	0.067931
hsa_circ_	0.420403	0.067943
hsa_circ_	-0.40604	0.067945
hsa_circ_	0.682409	0.067948
hsa_circ_	-0.40179	0.067968
hsa_circ_	-0.57246	0.067971
hsa_circ_	0.362321	0.067973
hsa_circ_	-0.56328	0.067975
hsa_circ_	0.492123	0.067984
hsa_circ_	-0.31499	0.06799
hsa_circ_	0.442514	0.067991
hsa_circ_	1.174083	0.068004
hsa_circ_	0.33224	0.068008
hsa_circ_	0.969528	0.068031
hsa_circ_	0.928198	0.068031
hsa_circ_	-0.47746	0.068043
hsa_circ_	0.339202	0.068064
hsa_circ_	0.45025	0.06807
hsa_circ_	-0.51587	0.068081
hsa_circ_	-0.31356	0.068085
hsa_circ_	0.460184	0.06809
hsa_circ_	0.411015	0.068091
hsa_circ_	0.999117	0.068105
hsa_circ_	-0.42017	0.068135
hsa_circ_	0.586571	0.068149
hsa_circ_	-0.66474	0.068154
hsa_circ_	-0.30979	0.06816
hsa_circ_	-0.37933	0.068163
hsa_circ_	-0.30855	0.068165
hsa_circ_	0.481944	0.068179
hsa_circ_	0.31654	0.068181
hsa_circ_	-1.03283	0.06819
hsa_circ_	-0.28711	0.068194
hsa_circ_	0.440312	0.068205
hsa_circ_	-0.70333	0.068206
hsa_circ_	0.385477	0.06822
hsa_circ_	-0.28944	0.068221
hsa_circ_	0.344649	0.068235
hsa_circ_	-0.32132	0.068238
hsa_circ_	-0.43167	0.068243
hsa_circ_	0.481061	0.068247
hsa_circ_	-0.73403	0.068247

hsa_circ_	-0.29322	0.068255
hsa_circ_	0.524747	0.068264
hsa_circ_	-0.30044	0.068267
hsa_circ_	-0.30357	0.068269
hsa_circ_	-0.70055	0.068282
hsa_circ_	0.407171	0.068284
hsa_circ_	0.67617	0.068287
hsa_circ_	-0.43125	0.068294
hsa_circ_	0.393521	0.068296
hsa_circ_	-0.33432	0.068308
hsa_circ_	-0.63723	0.068319
hsa_circ_	0.356541	0.068323
hsa_circ_	0.451323	0.068323
hsa_circ_	0.39468	0.068324
hsa_circ_	0.277284	0.068343
hsa_circ_	0.458673	0.068346
hsa_circ_	1.494804	0.068349
hsa_circ_	-0.28192	0.068359
hsa_circ_	-0.32686	0.068368
hsa_circ_	0.688489	0.06838
hsa_circ_	0.372392	0.068397
hsa_circ_	-0.3059	0.068398
hsa_circ_	-0.79852	0.068403
hsa_circ_	-0.34083	0.068415
hsa_circ_	0.358994	0.068426
hsa_circ_	0.268087	0.068437
hsa_circ_	0.392921	0.068438
hsa_circ_	-0.55813	0.068448
hsa_circ_	0.336699	0.068449
hsa_circ_	-0.48366	0.068457
hsa_circ_	0.413167	0.068466
hsa_circ_	-0.39729	0.068475
hsa_circ_	-0.55021	0.068477
hsa_circ_	-0.3206	0.068481
hsa_circ_	-0.35955	0.068483
hsa_circ_	-0.48295	0.068492
hsa_circ_	0.941651	0.068496
hsa_circ_	-0.49904	0.068505
hsa_circ_	0.410624	0.068506
hsa_circ_	0.277508	0.068524
hsa_circ_	0.565087	0.068528
hsa_circ_	-0.8964	0.068533
hsa_circ_	-0.33174	0.068537
hsa_circ_	0.297425	0.068546
hsa_circ_	0.535298	0.068564
hsa_circ_	0.3678	0.068567
hsa_circ_	0.549443	0.068568
hsa_circ_	-0.57846	0.068568
hsa_circ_	-0.63734	0.068577
hsa_circ_	0.38256	0.068578
hsa_circ_	0.534168	0.068582
hsa_circ_	0.433132	0.068582
hsa_circ_	0.473197	0.068586
hsa_circ_	-1.65211	0.068588

hsa_circ_	0.524795	0.068589
hsa_circ_	0.456917	0.068591
hsa_circ_	-0.41068	0.068597
hsa_circ_	-0.34741	0.068598
hsa_circ_	-0.53534	0.068603
hsa_circ_	0.370386	0.068604
hsa_circ_	-0.54795	0.068607
hsa_circ_	0.438753	0.06861
hsa_circ_	-0.7971	0.068612
hsa_circ_	-0.39043	0.068638
hsa_circ_	-0.32823	0.068638
hsa_circ_	-0.32188	0.06864
hsa_circ_	-0.64857	0.06864
hsa_circ_	-0.44611	0.068641
hsa_circ_	-0.39191	0.068645
hsa_circ_	-0.29043	0.068647
hsa_circ_	-0.51106	0.068652
hsa_circ_	0.309945	0.068656
hsa_circ_	-0.32854	0.068669
hsa_circ_	-1.08406	0.068677
hsa_circ_	0.674807	0.068689
hsa_circ_	0.357101	0.068701
hsa_circ_	0.339066	0.068706
hsa_circ_	-0.42722	0.068712
hsa_circ_	-0.731	0.06872
hsa_circ_	-0.8619	0.068726
hsa_circ_	0.409079	0.068759
hsa_circ_	0.397294	0.068761
hsa_circ_	0.613055	0.068763
hsa_circ_	0.52383	0.068767
hsa_circ_	-0.62361	0.068773
hsa_circ_	0.416577	0.068784
hsa_circ_	-0.38528	0.068787
hsa_circ_	0.796185	0.06879
hsa_circ_	-0.3824	0.068796
hsa_circ_	-0.38347	0.068804
hsa_circ_	-0.85693	0.068823
hsa_circ_	0.390071	0.068827
hsa_circ_	-0.54392	0.068834
hsa_circ_	0.342371	0.068835
hsa_circ_	-0.38336	0.068838
hsa_circ_	0.613394	0.068839
hsa_circ_	-0.92789	0.06884
hsa_circ_	-1.28257	0.068841
hsa_circ_	-0.29939	0.068852
hsa_circ_	0.912292	0.068865
hsa_circ_	-0.54798	0.068869
hsa_circ_	-1.51247	0.068873
hsa_circ_	-0.49015	0.068876
hsa_circ_	0.269369	0.068882
hsa_circ_	0.492115	0.068887
hsa_circ_	0.317545	0.068888
hsa_circ_	-0.42017	0.068897
hsa_circ_	-0.38802	0.068908

hsa_circ_	-0.37414	0.06891
hsa_circ_	0.593169	0.068912
hsa_circ_	-0.63828	0.068914
hsa_circ_	0.340821	0.068916
hsa_circ_	-0.40729	0.068926
hsa_circ_	-0.2867	0.068929
hsa_circ_	0.439073	0.06893
hsa_circ_	0.516005	0.068949
hsa_circ_	0.555951	0.068967
hsa_circ_	0.346685	0.068983
hsa_circ_	0.285019	0.068991
hsa_circ_	-1.3706	0.069
hsa_circ_	0.472716	0.069013
hsa_circ_	-0.32258	0.069017
hsa_circ_	-0.33108	0.069022
hsa_circ_	-0.28096	0.069023
hsa_circ_	0.448259	0.06903
hsa_circ_	-0.83311	0.069033
hsa_circ_	-0.3774	0.069037
hsa_circ_	-0.53913	0.069045
hsa_circ_	-0.32113	0.069045
hsa_circ_	-0.31274	0.069063
hsa_circ_	-0.37814	0.069073
hsa_circ_	0.337528	0.069075
hsa_circ_	-0.3975	0.06909
hsa_circ_	-0.32011	0.06909
hsa_circ_	1.155387	0.069093
hsa_circ_	-0.39622	0.069115
hsa_circ_	0.546861	0.069123
hsa_circ_	0.803159	0.069136
hsa_circ_	0.411369	0.069145
hsa_circ_	0.718707	0.069152
hsa_circ_	0.443233	0.069156
hsa_circ_	-0.34339	0.069157
hsa_circ_	-0.2691	0.069166
hsa_circ_	-0.45373	0.069184
hsa_circ_	0.62577	0.069199
hsa_circ_	-0.52739	0.069206
hsa_circ_	-1.04551	0.069208
hsa_circ_	-0.83789	0.069221
hsa_circ_	-0.28499	0.069222
hsa_circ_	0.63199	0.069231
hsa_circ_	0.692587	0.069234
hsa_circ_	1.272903	0.069254
hsa_circ_	-0.33303	0.069256
hsa_circ_	1.066713	0.069263
hsa_circ_	-0.64653	0.069273
hsa_circ_	0.401479	0.069284
hsa_circ_	0.528604	0.069285
hsa_circ_	-0.45171	0.069288
hsa_circ_	0.369457	0.069289
hsa_circ_	1.484971	0.069294
hsa_circ_	0.428693	0.069298
hsa_circ_	-0.54654	0.069312

hsa_circ_	-0.3041	0.069314
hsa_circ_	-0.46576	0.069336
hsa_circ_	0.490981	0.06934
hsa_circ_	0.370502	0.06935
hsa_circ_	0.424489	0.069353
hsa_circ_	0.442628	0.069356
hsa_circ_	1.6276	0.069357
hsa_circ_	-0.37033	0.06936
hsa_circ_	0.43318	0.069379
hsa_circ_	0.287636	0.069379
hsa_circ_	0.50274	0.069385
hsa_circ_	0.710489	0.069403
hsa_circ_	0.290814	0.069406
hsa_circ_	-0.43586	0.069409
hsa_circ_	0.649916	0.06941
hsa_circ_	-0.43852	0.069423
hsa_circ_	-0.74024	0.069433
hsa_circ_	-0.39239	0.069436
hsa_circ_	0.553778	0.069449
hsa_circ_	0.81855	0.06945
hsa_circ_	0.468384	0.069455
hsa_circ_	-0.31831	0.069468
hsa_circ_	-0.3594	0.069469
hsa_circ_	-0.50467	0.069469
hsa_circ_	-0.29671	0.069489
hsa_circ_	-0.72828	0.069489
hsa_circ_	-0.46588	0.069491
hsa_circ_	-0.41943	0.069491
hsa_circ_	-0.26137	0.0695
hsa_circ_	-0.35418	0.069521
hsa_circ_	-0.31587	0.069525
hsa_circ_	-0.424	0.069553
hsa_circ_	0.803418	0.069557
hsa_circ_	0.344245	0.06956
hsa_circ_	0.391938	0.069573
hsa_circ_	-0.51088	0.069574
hsa_circ_	-0.36287	0.069574
hsa_circ_	-0.46095	0.069574
hsa_circ_	-0.5515	0.069577
hsa_circ_	-0.30354	0.069579
hsa_circ_	0.547639	0.069584
hsa_circ_	-0.39385	0.069585
hsa_circ_	-0.30107	0.069587
hsa_circ_	-0.64824	0.069589
hsa_circ_	0.381327	0.069594
hsa_circ_	1.163246	0.069602
hsa_circ_	-0.48906	0.069606
hsa_circ_	-0.36625	0.069608
hsa_circ_	-0.4731	0.06962
hsa_circ_	-0.65927	0.069621
hsa_circ_	-0.61552	0.069626
hsa_circ_	0.275454	0.069629
hsa_circ_	-0.41718	0.069636
hsa_circ_	-0.37139	0.069641

hsa_circ_	0.412214	0.069645
hsa_circ_	0.341094	0.069646
hsa_circ_	-0.57718	0.069655
hsa_circ_	-0.32635	0.06967
hsa_circ_	-0.50274	0.069671
hsa_circ_	0.453285	0.06968
hsa_circ_	0.491963	0.069682
hsa_circ_	0.80609	0.069689
hsa_circ_	-0.33398	0.069705
hsa_circ_	-0.49012	0.069709
hsa_circ_	0.299373	0.069721
hsa_circ_	-0.76771	0.069721
hsa_circ_	0.707712	0.069724
hsa_circ_	0.810706	0.069732
hsa_circ_	-0.46687	0.069735
hsa_circ_	-3.27543	0.069745
hsa_circ_	-1.75607	0.069748
hsa_circ_	-0.38099	0.069758
hsa_circ_	0.395468	0.069776
hsa_circ_	-0.81156	0.069778
hsa_circ_	0.277332	0.069783
hsa_circ_	-0.69625	0.069784
hsa_circ_	-0.3805	0.069786
hsa_circ_	-0.34031	0.069792
hsa_circ_	-0.91034	0.069796
hsa_circ_	0.513976	0.069805
hsa_circ_	-0.39597	0.06981
hsa_circ_	0.522884	0.069834
hsa_circ_	0.674041	0.069835
hsa_circ_	0.312391	0.069835
hsa_circ_	0.761446	0.069836
hsa_circ_	-0.38789	0.069838
hsa_circ_	0.393889	0.069846
hsa_circ_	-0.59772	0.069846
hsa_circ_	0.495769	0.069849
hsa_circ_	-0.31009	0.069849
hsa_circ_	0.887258	0.06985
hsa_circ_	-1.1401	0.069858
hsa_circ_	-0.3764	0.069865
hsa_circ_	0.33136	0.06987
hsa_circ_	0.320315	0.069874
hsa_circ_	-0.34173	0.069908
hsa_circ_	0.754324	0.069922
hsa_circ_	-0.44308	0.069929
hsa_circ_	0.41018	0.069934
hsa_circ_	0.297091	0.069951
hsa_circ_	-0.35541	0.069951
hsa_circ_	0.696623	0.06996
hsa_circ_	0.514429	0.069963
hsa_circ_	0.903876	0.069963
hsa_circ_	0.558974	0.069966
hsa_circ_	-0.72455	0.069966
hsa_circ_	0.424838	0.06997
hsa_circ_	-0.39045	0.069977

hsa_circ_	-0.47415	0.069979
hsa_circ_	2.1501	0.070004
hsa_circ_	-0.45545	0.070008
hsa_circ_	-0.53151	0.070009
hsa_circ_	-0.95685	0.070013
hsa_circ_	0.672741	0.070017
hsa_circ_	0.299936	0.070024
hsa_circ_	0.313074	0.070028
hsa_circ_	-0.42834	0.070039
hsa_circ_	-0.28248	0.070041
hsa_circ_	-0.48725	0.070044
hsa_circ_	-0.34991	0.070056
hsa_circ_	-0.28017	0.070061
hsa_circ_	-0.50748	0.070067
hsa_circ_	-0.51902	0.070068
hsa_circ_	0.418152	0.070069
hsa_circ_	0.323127	0.070069
hsa_circ_	0.539327	0.070071
hsa_circ_	1.183896	0.070074
hsa_circ_	0.417813	0.070083
hsa_circ_	-0.42174	0.070091
hsa_circ_	0.480744	0.070097
hsa_circ_	0.939418	0.070102
hsa_circ_	-0.29232	0.070115
hsa_circ_	0.519196	0.070118
hsa_circ_	1.060952	0.070124
hsa_circ_	-0.37104	0.070127
hsa_circ_	-0.31826	0.070134
hsa_circ_	-0.42065	0.07014
hsa_circ_	0.872586	0.070148
hsa_circ_	-0.45656	0.07015
hsa_circ_	0.402305	0.07015
hsa_circ_	0.352838	0.070153
hsa_circ_	0.521468	0.070154
hsa_circ_	0.613862	0.070154
hsa_circ_	-0.44153	0.070159
hsa_circ_	-0.64455	0.070163
hsa_circ_	-0.7583	0.070163
hsa_circ_	0.788154	0.070168
hsa_circ_	-0.7449	0.07017
hsa_circ_	-0.34908	0.070192
hsa_circ_	-0.52144	0.070192
hsa_circ_	-0.32279	0.070205
hsa_circ_	0.472507	0.070208
hsa_circ_	0.434292	0.070219
hsa_circ_	0.429437	0.07022
hsa_circ_	0.880702	0.070238
hsa_circ_	0.496358	0.070239
hsa_circ_	-0.94404	0.070245
hsa_circ_	-0.44749	0.070264
hsa_circ_	-0.3445	0.07027
hsa_circ_	0.560681	0.070293
hsa_circ_	-0.47954	0.070296
hsa_circ_	-0.36483	0.070303

hsa_circ_	0.306452	0.070313
hsa_circ_	0.411306	0.070329
hsa_circ_	-0.33665	0.070339
hsa_circ_	0.478771	0.070346
hsa_circ_	0.390725	0.070378
hsa_circ_	0.26182	0.070385
hsa_circ_	0.455785	0.070386
hsa_circ_	-0.43271	0.070391
hsa_circ_	-1.02999	0.070404
hsa_circ_	0.606696	0.070406
hsa_circ_	0.594456	0.070408
hsa_circ_	-0.68336	0.070422
hsa_circ_	0.697552	0.070429
hsa_circ_	-0.76428	0.070438
hsa_circ_	0.327657	0.070446
hsa_circ_	0.301261	0.070451
hsa_circ_	0.349979	0.070461
hsa_circ_	-1.05667	0.070495
hsa_circ_	-0.35287	0.070502
hsa_circ_	-0.44836	0.07051
hsa_circ_	-0.50699	0.070513
hsa_circ_	0.757946	0.070518
hsa_circ_	-0.47343	0.070552
hsa_circ_	-0.26196	0.070562
hsa_circ_	-1.09167	0.070562
hsa_circ_	-0.38765	0.070587
hsa_circ_	-0.31174	0.070588
hsa_circ_	0.580792	0.070592
hsa_circ_	-0.33077	0.070606
hsa_circ_	-0.6097	0.070614
hsa_circ_	-0.31196	0.070641
hsa_circ_	-0.34545	0.070641
hsa_circ_	0.479018	0.070643
hsa_circ_	-0.39339	0.07065
hsa_circ_	-0.84738	0.070651
hsa_circ_	-0.36339	0.070666
hsa_circ_	-0.2897	0.070681
hsa_circ_	-0.31819	0.070686
hsa_circ_	-1.08451	0.070693
hsa_circ_	0.675541	0.070699
hsa_circ_	0.717552	0.070701
hsa_circ_	0.44609	0.070702
hsa_circ_	-0.68278	0.070708
hsa_circ_	-0.30339	0.070715
hsa_circ_	0.474824	0.070716
hsa_circ_	0.342706	0.070726
hsa_circ_	-0.38586	0.070738
hsa_circ_	-0.45126	0.07075
hsa_circ_	-0.3286	0.070755
hsa_circ_	-0.67413	0.070771
hsa_circ_	-1.1191	0.070775
hsa_circ_	0.305484	0.070776
hsa_circ_	0.417018	0.070783
hsa_circ_	-1.0659	0.070791

hsa_circ_	-0.26858	0.070799
hsa_circ_	0.344358	0.070808
hsa_circ_	-0.51592	0.070809
hsa_circ_	0.29329	0.07081
hsa_circ_	0.498582	0.070817
hsa_circ_	-0.41837	0.070829
hsa_circ_	0.303822	0.070838
hsa_circ_	-0.29721	0.070839
hsa_circ_	0.303892	0.07084
hsa_circ_	0.959903	0.070847
hsa_circ_	0.325441	0.070854
hsa_circ_	0.484629	0.070868
hsa_circ_	-0.80957	0.070884
hsa_circ_	-0.51132	0.070898
hsa_circ_	-0.5926	0.070906
hsa_circ_	0.518462	0.07091
hsa_circ_	-0.40476	0.070917
hsa_circ_	-0.67363	0.070918
hsa_circ_	-0.37037	0.070919
hsa_circ_	0.419231	0.070932
hsa_circ_	0.323273	0.070935
hsa_circ_	-0.3649	0.070938
hsa_circ_	0.522019	0.070947
hsa_circ_	0.418725	0.070947
hsa_circ_	-0.29655	0.070952
hsa_circ_	-0.69863	0.070956
hsa_circ_	0.330319	0.070959
hsa_circ_	-0.8074	0.070962
hsa_circ_	-0.37641	0.070966
hsa_circ_	-0.4469	0.070972
hsa_circ_	0.830756	0.070979
hsa_circ_	0.476992	0.07098
hsa_circ_	0.586504	0.070984
hsa_circ_	0.49883	0.070987
hsa_circ_	0.318523	0.070987
hsa_circ_	-0.38233	0.071031
hsa_circ_	-0.38793	0.071037
hsa_circ_	0.504016	0.071052
hsa_circ_	-0.35235	0.071052
hsa_circ_	0.558955	0.071064
hsa_circ_	-1.61688	0.071069
hsa_circ_	1.072989	0.071071
hsa_circ_	-1.12534	0.071081
hsa_circ_	-0.51492	0.071082
hsa_circ_	0.30317	0.071082
hsa_circ_	1.120582	0.071088
hsa_circ_	0.301446	0.071091
hsa_circ_	0.395739	0.071096
hsa_circ_	-0.59677	0.071098
hsa_circ_	-0.339	0.071098
hsa_circ_	0.418068	0.07114
hsa_circ_	0.307328	0.071141
hsa_circ_	-0.9586	0.071144
hsa_circ_	-0.35595	0.071149

hsa_circ_	0.423168	0.071159
hsa_circ_	-0.30195	0.071162
hsa_circ_	-0.30856	0.071166
hsa_circ_	0.382981	0.071168
hsa_circ_	1.011128	0.07117
hsa_circ_	-0.37071	0.071175
hsa_circ_	0.870294	0.07118
hsa_circ_	0.565897	0.07118
hsa_circ_	0.339035	0.071183
hsa_circ_	-0.53195	0.071192
hsa_circ_	0.86435	0.071199
hsa_circ_	-0.31496	0.0712
hsa_circ_	0.786592	0.071205
hsa_circ_	0.356274	0.071205
hsa_circ_	0.45526	0.071206
hsa_circ_	0.488844	0.071207
hsa_circ_	0.590996	0.071212
hsa_circ_	0.849561	0.071232
hsa_circ_	0.33493	0.071237
hsa_circ_	-0.51138	0.071262
hsa_circ_	-0.28332	0.071289
hsa_circ_	-0.74774	0.071291
hsa_circ_	-1.0622	0.071295
hsa_circ_	-0.42698	0.071298
hsa_circ_	0.312582	0.071302
hsa_circ_	0.385903	0.071302
hsa_circ_	-0.34515	0.071327
hsa_circ_	-1.2795	0.071328
hsa_circ_	-0.68168	0.071328
hsa_circ_	-0.29501	0.071328
hsa_circ_	0.282016	0.071329
hsa_circ_	-0.33325	0.071336
hsa_circ_	-1.28226	0.071339
hsa_circ_	-0.86369	0.071345
hsa_circ_	-0.27119	0.071353
hsa_circ_	-0.28916	0.071355
hsa_circ_	0.320784	0.071359
hsa_circ_	-0.2893	0.071365
hsa_circ_	-0.27502	0.071376
hsa_circ_	0.633046	0.071383
hsa_circ_	0.313609	0.071392
hsa_circ_	-0.56212	0.07141
hsa_circ_	0.27926	0.071422
hsa_circ_	0.499704	0.071423
hsa_circ_	-0.71423	0.071437
hsa_circ_	0.359251	0.071454
hsa_circ_	0.321217	0.071462
hsa_circ_	0.958396	0.071463
hsa_circ_	-0.41222	0.071471
hsa_circ_	0.355388	0.071471
hsa_circ_	-0.43204	0.071473
hsa_circ_	0.280078	0.071494
hsa_circ_	0.499686	0.071505
hsa_circ_	0.45752	0.071509

hsa_circ_	0.293269	0.071513
hsa_circ_	-0.37539	0.071521
hsa_circ_	-0.31489	0.071529
hsa_circ_	-0.78008	0.071535
hsa_circ_	0.7005	0.071536
hsa_circ_	0.284024	0.071541
hsa_circ_	-0.3331	0.071555
hsa_circ_	-0.46498	0.071556
hsa_circ_	0.476397	0.071556
hsa_circ_	0.790049	0.071563
hsa_circ_	-1.59939	0.071566
hsa_circ_	-0.7391	0.071577
hsa_circ_	0.277011	0.071589
hsa_circ_	-0.66718	0.071589
hsa_circ_	-0.40204	0.0716
hsa_circ_	-0.47688	0.071606
hsa_circ_	1.055184	0.071613
hsa_circ_	0.680053	0.071616
hsa_circ_	0.316145	0.071621
hsa_circ_	0.575612	0.071631
hsa_circ_	-0.37169	0.071641
hsa_circ_	-0.5212	0.071659
hsa_circ_	0.9169	0.071663
hsa_circ_	-0.48524	0.071666
hsa_circ_	0.439727	0.071668
hsa_circ_	-0.31874	0.071672
hsa_circ_	0.57347	0.071678
hsa_circ_	-0.36614	0.071682
hsa_circ_	0.493484	0.071684
hsa_circ_	0.370366	0.071686
hsa_circ_	-0.7757	0.071688
hsa_circ_	0.376383	0.071699
hsa_circ_	-0.32996	0.071727
hsa_circ_	-0.29564	0.071727
hsa_circ_	-0.67426	0.071728
hsa_circ_	0.985482	0.071738
hsa_circ_	0.526131	0.07174
hsa_circ_	-0.59722	0.07174
hsa_circ_	-0.83019	0.071745
hsa_circ_	-0.31579	0.071753
hsa_circ_	-0.40197	0.071759
hsa_circ_	-0.29588	0.071764
hsa_circ_	-0.48847	0.071773
hsa_circ_	0.46939	0.071786
hsa_circ_	-0.37947	0.071801
hsa_circ_	-0.28843	0.071808
hsa_circ_	-0.32318	0.071814
hsa_circ_	0.560093	0.071837
hsa_circ_	0.423436	0.071848
hsa_circ_	-0.27037	0.071853
hsa_circ_	-0.46989	0.071862
hsa_circ_	-0.32126	0.071868
hsa_circ_	-0.40782	0.071872
hsa_circ_	-0.65469	0.071884

hsa_circ_	0.351228	0.071884
hsa_circ_	0.529659	0.071885
hsa_circ_	0.283473	0.071887
hsa_circ_	0.301085	0.071889
hsa_circ_	0.346308	0.07189
hsa_circ_	-0.34987	0.071894
hsa_circ_	0.340426	0.071896
hsa_circ_	-0.66906	0.071898
hsa_circ_	-0.62934	0.071917
hsa_circ_	-0.77763	0.071931
hsa_circ_	0.629859	0.071933
hsa_circ_	-0.31536	0.071944
hsa_circ_	-0.4521	0.071954
hsa_circ_	0.806073	0.071962
hsa_circ_	-0.41309	0.071968
hsa_circ_	-0.71141	0.071972
hsa_circ_	-0.70717	0.071989
hsa_circ_	-0.29557	0.071998
hsa_circ_	0.358123	0.072018
hsa_circ_	-0.35673	0.072028
hsa_circ_	-0.33769	0.072031
hsa_circ_	0.3078	0.072032
hsa_circ_	-0.30552	0.072044
hsa_circ_	0.322287	0.072051
hsa_circ_	-0.39406	0.072064
hsa_circ_	0.465086	0.072084
hsa_circ_	-0.43641	0.072088
hsa_circ_	-0.38444	0.072104
hsa_circ_	0.444115	0.072105
hsa_circ_	-0.5505	0.072109
hsa_circ_	-0.34595	0.072109
hsa_circ_	0.799099	0.072111
hsa_circ_	0.586862	0.072111
hsa_circ_	0.615372	0.072111
hsa_circ_	-0.52618	0.072113
hsa_circ_	1.034438	0.07212
hsa_circ_	0.426489	0.072121
hsa_circ_	-0.83801	0.072126
hsa_circ_	-0.5685	0.072131
hsa_circ_	0.352502	0.072136
hsa_circ_	-0.44401	0.072141
hsa_circ_	0.392154	0.072142
hsa_circ_	-0.55335	0.072144
hsa_circ_	-0.33263	0.072148
hsa_circ_	1.351957	0.072151
hsa_circ_	-0.40404	0.072151
hsa_circ_	0.300639	0.072155
hsa_circ_	-0.44085	0.072168
hsa_circ_	0.452986	0.072173
hsa_circ_	0.398745	0.072174
hsa_circ_	0.768637	0.072174
hsa_circ_	-1.12604	0.072182
hsa_circ_	-0.3029	0.072183
hsa_circ_	0.74227	0.072191

hsa_circ_	0.598915	0.072194
hsa_circ_	0.324585	0.072204
hsa_circ_	0.457537	0.072219
hsa_circ_	-0.28067	0.072235
hsa_circ_	-0.72332	0.072236
hsa_circ_	0.78718	0.072244
hsa_circ_	0.469448	0.072251
hsa_circ_	0.489498	0.072252
hsa_circ_	0.336831	0.072256
hsa_circ_	0.444192	0.072258
hsa_circ_	0.348332	0.072262
hsa_circ_	-0.4267	0.072267
hsa_circ_	0.469056	0.072276
hsa_circ_	1.131974	0.072277
hsa_circ_	-0.4654	0.072277
hsa_circ_	-0.55921	0.072282
hsa_circ_	-0.75514	0.072289
hsa_circ_	0.283874	0.072304
hsa_circ_	-0.32407	0.072305
hsa_circ_	-0.44653	0.072315
hsa_circ_	0.528971	0.07233
hsa_circ_	-0.46834	0.072331
hsa_circ_	0.299696	0.072335
hsa_circ_	0.366757	0.072346
hsa_circ_	-0.56866	0.072349
hsa_circ_	-0.28371	0.072353
hsa_circ_	0.336367	0.072356
hsa_circ_	0.425944	0.072363
hsa_circ_	0.335939	0.072363
hsa_circ_	-0.28131	0.072364
hsa_circ_	-0.27988	0.072368
hsa_circ_	0.330484	0.072375
hsa_circ_	-0.45109	0.072378
hsa_circ_	1.146488	0.072381
hsa_circ_	0.751519	0.072383
hsa_circ_	0.286608	0.072384
hsa_circ_	-1.23531	0.072386
hsa_circ_	-0.41242	0.072391
hsa_circ_	-0.28348	0.072396
hsa_circ_	1.106385	0.072399
hsa_circ_	-0.39766	0.072402
hsa_circ_	-0.3249	0.072406
hsa_circ_	0.361988	0.07242
hsa_circ_	0.354131	0.072427
hsa_circ_	0.502405	0.072439
hsa_circ_	0.408615	0.072442
hsa_circ_	0.482616	0.072442
hsa_circ_	-0.29105	0.072456
hsa_circ_	-0.30825	0.072456
hsa_circ_	0.466846	0.072483
hsa_circ_	0.441151	0.072487
hsa_circ_	-0.27795	0.072487
hsa_circ_	0.564624	0.072492
hsa_circ_	0.527571	0.072504

hsa_circ_	0.477937	0.072507
hsa_circ_	-0.73108	0.072514
hsa_circ_	0.317138	0.072519
hsa_circ_	-0.6831	0.072524
hsa_circ_	-0.82201	0.07254
hsa_circ_	-0.50549	0.072552
hsa_circ_	0.559647	0.072564
hsa_circ_	-0.3942	0.072587
hsa_circ_	-0.45531	0.072587
hsa_circ_	0.300394	0.072588
hsa_circ_	0.428329	0.072594
hsa_circ_	0.366492	0.072598
hsa_circ_	0.318953	0.072618
hsa_circ_	0.328284	0.072618
hsa_circ_	-0.71862	0.072619
hsa_circ_	1.073997	0.072628
hsa_circ_	-0.34925	0.07263
hsa_circ_	-0.493	0.072638
hsa_circ_	0.467395	0.072648
hsa_circ_	-0.39191	0.072653
hsa_circ_	0.953175	0.072666
hsa_circ_	0.754363	0.072684
hsa_circ_	0.45836	0.072687
hsa_circ_	-0.82681	0.07271
hsa_circ_	0.396681	0.072734
hsa_circ_	-0.37959	0.072739
hsa_circ_	0.719326	0.072745
hsa_circ_	-0.52697	0.072761
hsa_circ_	0.582787	0.072766
hsa_circ_	0.403615	0.072786
hsa_circ_	-0.35173	0.072788
hsa_circ_	0.30126	0.07279
hsa_circ_	-1.12607	0.072793
hsa_circ_	0.411024	0.0728
hsa_circ_	-0.27563	0.072804
hsa_circ_	0.384723	0.072805
hsa_circ_	0.424843	0.07281
hsa_circ_	0.422861	0.072811
hsa_circ_	0.486401	0.072819
hsa_circ_	-0.55927	0.07282
hsa_circ_	0.344923	0.07283
hsa_circ_	0.549772	0.072839
hsa_circ_	-0.26973	0.07284
hsa_circ_	0.454645	0.072849
hsa_circ_	0.392344	0.072862
hsa_circ_	-0.3209	0.072862
hsa_circ_	-0.41809	0.072865
hsa_circ_	0.348947	0.072866
hsa_circ_	-0.30366	0.072866
hsa_circ_	0.335922	0.072868
hsa_circ_	0.313104	0.072874
hsa_circ_	0.356203	0.072882
hsa_circ_	0.400637	0.072899
hsa_circ_	-0.519	0.072902

hsa_circ_	0.835231	0.072917
hsa_circ_	0.38449	0.072922
hsa_circ_	-0.32898	0.072923
hsa_circ_	0.594443	0.072928
hsa_circ_	0.455144	0.072941
hsa_circ_	0.325697	0.072945
hsa_circ_	0.340527	0.072946
hsa_circ_	0.350528	0.072952
hsa_circ_	0.481214	0.07297
hsa_circ_	0.344042	0.072985
hsa_circ_	0.392726	0.073002
hsa_circ_	0.387813	0.073007
hsa_circ_	0.582074	0.07302
hsa_circ_	-0.93284	0.073026
hsa_circ_	0.728932	0.073034
hsa_circ_	-0.27502	0.073034
hsa_circ_	0.597977	0.073044
hsa_circ_	-0.2993	0.073055
hsa_circ_	0.282274	0.073064
hsa_circ_	1.469953	0.073065
hsa_circ_	-0.32884	0.073065
hsa_circ_	-0.34431	0.073078
hsa_circ_	-0.77661	0.073078
hsa_circ_	-0.36964	0.073082
hsa_circ_	-0.5273	0.073088
hsa_circ_	-0.74777	0.073093
hsa_circ_	-0.2978	0.073102
hsa_circ_	-0.47107	0.073104
hsa_circ_	0.915075	0.073107
hsa_circ_	-0.32095	0.073108
hsa_circ_	-1.11536	0.073123
hsa_circ_	0.448142	0.07314
hsa_circ_	-0.49481	0.073144
hsa_circ_	-0.58471	0.073155
hsa_circ_	0.404054	0.073161
hsa_circ_	0.421416	0.073169
hsa_circ_	-1.55302	0.073189
hsa_circ_	0.639171	0.073192
hsa_circ_	-1.40449	0.073203
hsa_circ_	-1.31894	0.073204
hsa_circ_	-0.38589	0.073215
hsa_circ_	0.820223	0.073219
hsa_circ_	0.713282	0.07322
hsa_circ_	0.821178	0.073261
hsa_circ_	-0.9279	0.073267
hsa_circ_	1.069438	0.073269
hsa_circ_	-0.46457	0.073295
hsa_circ_	-0.36843	0.073295
hsa_circ_	-0.33807	0.073306
hsa_circ_	-0.3303	0.073307
hsa_circ_	0.40451	0.073312
hsa_circ_	-0.39442	0.073325
hsa_circ_	-0.32355	0.073327
hsa_circ_	1.042043	0.073328

hsa_circ_	0.814363	0.073332
hsa_circ_	0.441432	0.073333
hsa_circ_	-0.35776	0.073337
hsa_circ_	0.406146	0.073346
hsa_circ_	-0.39719	0.073352
hsa_circ_	-0.74398	0.073369
hsa_circ_	0.547066	0.073379
hsa_circ_	0.968625	0.073405
hsa_circ_	0.440556	0.07341
hsa_circ_	-0.30893	0.073416
hsa_circ_	0.599822	0.073439
hsa_circ_	-0.29119	0.073443
hsa_circ_	-0.63106	0.073445
hsa_circ_	0.30614	0.07345
hsa_circ_	0.443822	0.073459
hsa_circ_	0.385769	0.073459
hsa_circ_	-0.36661	0.073464
hsa_circ_	0.561179	0.073479
hsa_circ_	-0.52214	0.073484
hsa_circ_	-0.57156	0.073501
hsa_circ_	0.277507	0.073501
hsa_circ_	-0.30845	0.073502
hsa_circ_	-0.91584	0.073514
hsa_circ_	-0.46147	0.073514
hsa_circ_	-1.07536	0.073517
hsa_circ_	-0.43839	0.073529
hsa_circ_	0.54993	0.073545
hsa_circ_	0.305478	0.073551
hsa_circ_	0.271399	0.073553
hsa_circ_	-0.28691	0.073556
hsa_circ_	0.61841	0.073561
hsa_circ_	-0.48834	0.073563
hsa_circ_	0.765452	0.073565
hsa_circ_	0.297622	0.073572
hsa_circ_	-0.30521	0.073572
hsa_circ_	-0.30103	0.073575
hsa_circ_	-0.33118	0.073588
hsa_circ_	1.0901	0.073591
hsa_circ_	0.286796	0.073612
hsa_circ_	0.469906	0.073619
hsa_circ_	0.50302	0.073621
hsa_circ_	0.546624	0.073624
hsa_circ_	0.318229	0.073625
hsa_circ_	0.655331	0.073627
hsa_circ_	0.274448	0.073629
hsa_circ_	0.29804	0.073637
hsa_circ_	0.468298	0.073648
hsa_circ_	-0.77808	0.073654
hsa_circ_	0.81002	0.073658
hsa_circ_	1.170649	0.073658
hsa_circ_	0.610327	0.073662
hsa_circ_	-0.33844	0.073663
hsa_circ_	-0.49331	0.073677
hsa_circ_	0.492864	0.073678

hsa_circ_	0.454249	0.073681
hsa_circ_	-0.49282	0.073681
hsa_circ_	-0.53844	0.073692
hsa_circ_	0.907131	0.073694
hsa_circ_	0.811053	0.073696
hsa_circ_	-0.34902	0.073714
hsa_circ_	0.398283	0.073717
hsa_circ_	-0.43969	0.073723
hsa_circ_	-0.28201	0.073723
hsa_circ_	-0.27354	0.073736
hsa_circ_	-0.42058	0.073743
hsa_circ_	0.507881	0.073745
hsa_circ_	0.752595	0.073755
hsa_circ_	-0.51175	0.073755
hsa_circ_	-0.69704	0.073761
hsa_circ_	1.021648	0.073769
hsa_circ_	0.455075	0.073773
hsa_circ_	0.568885	0.073788
hsa_circ_	-0.8693	0.073789
hsa_circ_	0.435257	0.073789
hsa_circ_	-0.55878	0.07379
hsa_circ_	-0.49113	0.073814
hsa_circ_	1.007661	0.073825
hsa_circ_	-0.42583	0.073826
hsa_circ_	-1.22075	0.073831
hsa_circ_	0.574518	0.073831
hsa_circ_	0.501286	0.073836
hsa_circ_	-0.39462	0.073839
hsa_circ_	-0.32766	0.073843
hsa_circ_	-0.27769	0.073844
hsa_circ_	0.416748	0.073847
hsa_circ_	0.423744	0.073857
hsa_circ_	-1.16769	0.073867
hsa_circ_	0.785467	0.073871
hsa_circ_	-0.48161	0.073871
hsa_circ_	-0.61896	0.073896
hsa_circ_	1.018914	0.073898
hsa_circ_	-0.32952	0.07392
hsa_circ_	0.869901	0.073931
hsa_circ_	1.018137	0.073936
hsa_circ_	-0.47868	0.073939
hsa_circ_	-0.33915	0.073939
hsa_circ_	-0.48189	0.073945
hsa_circ_	0.404528	0.073949
hsa_circ_	-0.39941	0.073958
hsa_circ_	-0.42752	0.073958
hsa_circ_	0.63927	0.073959
hsa_circ_	-0.3095	0.07397
hsa_circ_	-0.58591	0.073986
hsa_circ_	0.444868	0.07399
hsa_circ_	0.590228	0.073991
hsa_circ_	-0.70588	0.073995
hsa_circ_	-0.32768	0.073996
hsa_circ_	0.430103	0.073997

hsa_circ_	0.48282	0.074011
hsa_circ_	0.409483	0.07405
hsa_circ_	1.21901	0.074055
hsa_circ_	-0.32745	0.074069
hsa_circ_	-0.26828	0.074073
hsa_circ_	0.304267	0.074074
hsa_circ_	0.381262	0.074077
hsa_circ_	1.254628	0.074083
hsa_circ_	-0.62179	0.074086
hsa_circ_	-0.35612	0.074093
hsa_circ_	0.313678	0.0741
hsa_circ_	-0.30648	0.074102
hsa_circ_	0.466826	0.074106
hsa_circ_	-0.48312	0.074108
hsa_circ_	-0.52684	0.074109
hsa_circ_	0.556398	0.074126
hsa_circ_	0.625244	0.074128
hsa_circ_	-0.29479	0.074133
hsa_circ_	0.531903	0.074144
hsa_circ_	-0.48632	0.074145
hsa_circ_	-0.42301	0.074148
hsa_circ_	-0.82046	0.074166
hsa_circ_	0.435456	0.074169
hsa_circ_	-0.32441	0.074173
hsa_circ_	-0.53769	0.074186
hsa_circ_	0.437622	0.074194
hsa_circ_	-0.2885	0.074199
hsa_circ_	0.457704	0.074202
hsa_circ_	-0.31341	0.074205
hsa_circ_	-0.3764	0.074206
hsa_circ_	0.358478	0.074225
hsa_circ_	-0.3155	0.074229
hsa_circ_	0.289795	0.074233
hsa_circ_	0.673709	0.074236
hsa_circ_	-0.31428	0.074247
hsa_circ_	1.145995	0.074263
hsa_circ_	0.817099	0.074264
hsa_circ_	-0.43117	0.074268
hsa_circ_	-0.38706	0.074276
hsa_circ_	-0.53182	0.074286
hsa_circ_	0.924198	0.074294
hsa_circ_	0.321581	0.074299
hsa_circ_	-0.62475	0.0743
hsa_circ_	0.29336	0.074303
hsa_circ_	0.389515	0.074304
hsa_circ_	-0.41721	0.074326
hsa_circ_	1.04402	0.074334
hsa_circ_	0.353598	0.074334
hsa_circ_	-1.12646	0.07434
hsa_circ_	-0.3491	0.07434
hsa_circ_	-0.2848	0.074341
hsa_circ_	0.511729	0.074349
hsa_circ_	-0.43324	0.074351
hsa_circ_	-0.68708	0.074367

hsa_circ_	-0.61557	0.07437
hsa_circ_	0.322794	0.07437
hsa_circ_	-0.36975	0.074373
hsa_circ_	0.369482	0.074377
hsa_circ_	0.614357	0.074381
hsa_circ_	-0.28074	0.074392
hsa_circ_	0.478104	0.074395
hsa_circ_	-0.56975	0.074402
hsa_circ_	0.656162	0.074408
hsa_circ_	-0.42915	0.074417
hsa_circ_	-0.44476	0.074419
hsa_circ_	-0.34817	0.074421
hsa_circ_	0.290259	0.074422
hsa_circ_	0.382757	0.074425
hsa_circ_	-0.36604	0.074437
hsa_circ_	0.572957	0.074451
hsa_circ_	-0.33916	0.074453
hsa_circ_	-0.28822	0.074453
hsa_circ_	-0.51494	0.074457
hsa_circ_	0.542507	0.074465
hsa_circ_	-0.34332	0.074473
hsa_circ_	0.25738	0.074497
hsa_circ_	0.487619	0.0745
hsa_circ_	0.744292	0.0745
hsa_circ_	-0.42979	0.074505
hsa_circ_	-0.38958	0.074514
hsa_circ_	0.358449	0.074523
hsa_circ_	0.422797	0.074526
hsa_circ_	-0.41769	0.074532
hsa_circ_	-1.19776	0.074539
hsa_circ_	0.596946	0.07454
hsa_circ_	-1.27044	0.074549
hsa_circ_	-0.51736	0.074556
hsa_circ_	0.373212	0.074561
hsa_circ_	0.442712	0.074561
hsa_circ_	-0.42237	0.07457
hsa_circ_	-0.66719	0.074572
hsa_circ_	0.415684	0.074585
hsa_circ_	0.349315	0.074586
hsa_circ_	-0.26493	0.074591
hsa_circ_	-0.42341	0.074596
hsa_circ_	0.310197	0.074605
hsa_circ_	0.306346	0.074617
hsa_circ_	-0.27114	0.074622
hsa_circ_	-0.27857	0.074639
hsa_circ_	0.666698	0.074644
hsa_circ_	0.345803	0.074646
hsa_circ_	-1.00171	0.07466
hsa_circ_	-1.30391	0.07466
hsa_circ_	-0.3127	0.074667
hsa_circ_	-0.53026	0.074669
hsa_circ_	1.158909	0.07467
hsa_circ_	-0.59201	0.074676
hsa_circ_	-0.33721	0.074677

hsa_circ_	-0.39222	0.074685
hsa_circ_	-0.29247	0.074695
hsa_circ_	0.378637	0.074695
hsa_circ_	0.72659	0.074708
hsa_circ_	0.714367	0.074731
hsa_circ_	-0.91004	0.074731
hsa_circ_	-0.27193	0.074734
hsa_circ_	-1.46659	0.074735
hsa_circ_	0.558313	0.074737
hsa_circ_	-0.36833	0.074743
hsa_circ_	-0.37392	0.074744
hsa_circ_	0.479653	0.074744
hsa_circ_	0.609824	0.074747
hsa_circ_	0.330069	0.074758
hsa_circ_	-1.54934	0.074762
hsa_circ_	-0.82518	0.074777
hsa_circ_	-0.31045	0.074781
hsa_circ_	-0.3649	0.074786
hsa_circ_	0.38316	0.074802
hsa_circ_	-0.28869	0.074805
hsa_circ_	-0.30075	0.074808
hsa_circ_	-0.3504	0.07481
hsa_circ_	-0.29026	0.074813
hsa_circ_	-0.30898	0.074824
hsa_circ_	0.859444	0.074834
hsa_circ_	0.487582	0.074862
hsa_circ_	0.509743	0.074865
hsa_circ_	-0.43542	0.074877
hsa_circ_	-0.37428	0.074878
hsa_circ_	-0.56899	0.074886
hsa_circ_	0.306811	0.074886
hsa_circ_	-0.72825	0.074886
hsa_circ_	0.43803	0.074904
hsa_circ_	0.269496	0.074912
hsa_circ_	0.630649	0.074913
hsa_circ_	-0.31594	0.074919
hsa_circ_	-0.51026	0.074945
hsa_circ_	0.493056	0.074946
hsa_circ_	0.450589	0.074953
hsa_circ_	0.447046	0.074956
hsa_circ_	0.47365	0.074966
hsa_circ_	0.364894	0.074967
hsa_circ_	-0.79527	0.074974
hsa_circ_	-0.30059	0.074974
hsa_circ_	0.36314	0.07501
hsa_circ_	-0.32427	0.075015
hsa_circ_	0.354044	0.075016
hsa_circ_	0.411333	0.075016
hsa_circ_	0.321527	0.075019
hsa_circ_	0.955299	0.075025
hsa_circ_	-0.26977	0.075041
hsa_circ_	0.346318	0.075073
hsa_circ_	0.362688	0.075073
hsa_circ_	0.340235	0.075075

hsa_circ_	-0.34336	0.075081
hsa_circ_	1.239114	0.075089
hsa_circ_	0.306772	0.075104
hsa_circ_	1.14402	0.075111
hsa_circ_	0.472147	0.075111
hsa_circ_	0.930647	0.075127
hsa_circ_	0.280005	0.075128
hsa_circ_	-0.39267	0.075131
hsa_circ_	-0.5799	0.075142
hsa_circ_	-0.30442	0.07515
hsa_circ_	0.298811	0.075154
hsa_circ_	0.352666	0.075166
hsa_circ_	0.833649	0.075169
hsa_circ_	-0.41354	0.075171
hsa_circ_	-0.2883	0.075174
hsa_circ_	-0.59691	0.075178
hsa_circ_	-0.57951	0.075181
hsa_circ_	-0.32648	0.075181
hsa_circ_	-0.96282	0.075199
hsa_circ_	0.30129	0.075217
hsa_circ_	0.303052	0.075221
hsa_circ_	-1.00392	0.075221
hsa_circ_	-0.38279	0.075228
hsa_circ_	0.483645	0.075233
hsa_circ_	0.882434	0.075245
hsa_circ_	-0.74954	0.075257
hsa_circ_	0.296653	0.075259
hsa_circ_	0.637801	0.075276
hsa_circ_	-0.33737	0.075293
hsa_circ_	-0.40207	0.075294
hsa_circ_	0.325037	0.075294
hsa_circ_	0.462084	0.075296
hsa_circ_	0.412591	0.075297
hsa_circ_	-1.30625	0.075302
hsa_circ_	-0.46543	0.075308
hsa_circ_	-0.44761	0.075322
hsa_circ_	0.494814	0.07533
hsa_circ_	0.421976	0.075338
hsa_circ_	0.397292	0.075338
hsa_circ_	-0.51246	0.075339
hsa_circ_	0.469854	0.075352
hsa_circ_	1.137578	0.075359
hsa_circ_	-0.31158	0.07536
hsa_circ_	-0.6919	0.075361
hsa_circ_	0.263583	0.075386
hsa_circ_	0.991352	0.075395
hsa_circ_	-0.31124	0.075396
hsa_circ_	-0.35613	0.075401
hsa_circ_	0.428048	0.075416
hsa_circ_	0.35411	0.075419
hsa_circ_	-0.31577	0.075428
hsa_circ_	-0.67084	0.07544
hsa_circ_	-0.65867	0.075446
hsa_circ_	-0.28384	0.075454

hsa_circ_	-1.25683	0.075461
hsa_circ_	0.591273	0.075466
hsa_circ_	-0.46413	0.075466
hsa_circ_	-0.53435	0.075469
hsa_circ_	-0.33635	0.075473
hsa_circ_	-0.33339	0.075478
hsa_circ_	-0.5887	0.075491
hsa_circ_	-1.24239	0.075493
hsa_circ_	0.500655	0.075494
hsa_circ_	0.329065	0.075497
hsa_circ_	-0.44032	0.075498
hsa_circ_	-0.44191	0.075503
hsa_circ_	-0.37993	0.075512
hsa_circ_	-0.62791	0.075517
hsa_circ_	0.307308	0.075518
hsa_circ_	0.357476	0.07552
hsa_circ_	-0.81844	0.075527
hsa_circ_	0.316539	0.07553
hsa_circ_	-0.70267	0.075533
hsa_circ_	-0.77092	0.075537
hsa_circ_	-0.60365	0.075543
hsa_circ_	0.539501	0.075546
hsa_circ_	0.958079	0.07555
hsa_circ_	0.496975	0.075558
hsa_circ_	-0.28252	0.075562
hsa_circ_	-0.4327	0.075565
hsa_circ_	-0.41323	0.07557
hsa_circ_	-0.36647	0.075575
hsa_circ_	0.362553	0.07558
hsa_circ_	-0.42483	0.075584
hsa_circ_	0.378527	0.075611
hsa_circ_	-0.36014	0.075618
hsa_circ_	-0.36157	0.075621
hsa_circ_	-0.37328	0.075635
hsa_circ_	-0.35314	0.07564
hsa_circ_	-0.64966	0.075652
hsa_circ_	-0.52695	0.075652
hsa_circ_	-0.42581	0.075659
hsa_circ_	0.643105	0.075663
hsa_circ_	-0.5128	0.075665
hsa_circ_	-1.7509	0.075665
hsa_circ_	0.844984	0.075675
hsa_circ_	0.363101	0.075682
hsa_circ_	0.373229	0.075687
hsa_circ_	0.442082	0.075691
hsa_circ_	-1.03	0.075695
hsa_circ_	0.905836	0.075696
hsa_circ_	-0.82447	0.075704
hsa_circ_	-0.42553	0.075714
hsa_circ_	-0.50882	0.075722
hsa_circ_	0.536792	0.075729
hsa_circ_	0.49495	0.075743
hsa_circ_	-0.75479	0.075744
hsa_circ_	0.732586	0.075746

hsa_circ_	0.725405	0.075748
hsa_circ_	-0.93523	0.075751
hsa_circ_	0.635022	0.075757
hsa_circ_	-0.29694	0.07578
hsa_circ_	-0.31645	0.075781
hsa_circ_	0.664564	0.075782
hsa_circ_	0.435694	0.075793
hsa_circ_	-0.28538	0.075795
hsa_circ_	0.5861	0.075796
hsa_circ_	0.77166	0.075806
hsa_circ_	0.382542	0.075823
hsa_circ_	-0.50036	0.075825
hsa_circ_	0.621242	0.075826
hsa_circ_	0.349605	0.075837
hsa_circ_	-0.30641	0.075841
hsa_circ_	-0.43789	0.075846
hsa_circ_	-0.38774	0.075847
hsa_circ_	-0.32599	0.075858
hsa_circ_	-0.55387	0.075862
hsa_circ_	0.359954	0.075882
hsa_circ_	0.431573	0.075882
hsa_circ_	0.555663	0.075889
hsa_circ_	0.3369	0.075902
hsa_circ_	-0.33409	0.075915
hsa_circ_	-0.51823	0.075929
hsa_circ_	-0.26774	0.07593
hsa_circ_	-0.42521	0.075932
hsa_circ_	0.787597	0.07594
hsa_circ_	-0.47752	0.075943
hsa_circ_	0.418563	0.07595
hsa_circ_	0.402212	0.075957
hsa_circ_	0.476913	0.075959
hsa_circ_	-0.40684	0.075966
hsa_circ_	-0.43539	0.075978
hsa_circ_	-0.5557	0.075981
hsa_circ_	-0.45267	0.075983
hsa_circ_	-0.30571	0.075985
hsa_circ_	0.425581	0.075989
hsa_circ_	-0.29115	0.075998
hsa_circ_	0.44565	0.076003
hsa_circ_	0.834792	0.076004
hsa_circ_	-0.27888	0.076005
hsa_circ_	-0.32838	0.076045
hsa_circ_	0.45961	0.076046
hsa_circ_	0.405446	0.07606
hsa_circ_	-0.36308	0.076063
hsa_circ_	-0.27321	0.076072
hsa_circ_	0.380088	0.076077
hsa_circ_	-0.54075	0.076087
hsa_circ_	0.294914	0.076107
hsa_circ_	-0.40653	0.076109
hsa_circ_	-0.42198	0.076121
hsa_circ_	0.293733	0.076123
hsa_circ_	0.270796	0.076137

hsa_circ_	-0.31614	0.076147
hsa_circ_	-0.26635	0.076148
hsa_circ_	0.312884	0.076153
hsa_circ_	-0.35809	0.076167
hsa_circ_	-0.27946	0.076172
hsa_circ_	0.704996	0.076174
hsa_circ_	-0.37359	0.076185
hsa_circ_	-0.33671	0.076196
hsa_circ_	0.359934	0.0762
hsa_circ_	1.89019	0.076224
hsa_circ_	0.348503	0.076225
hsa_circ_	-0.34541	0.076228
hsa_circ_	-1.42447	0.07623
hsa_circ_	0.42818	0.076233
hsa_circ_	0.405829	0.076234
hsa_circ_	0.655087	0.076238
hsa_circ_	0.438506	0.076252
hsa_circ_	0.713968	0.076255
hsa_circ_	-0.29384	0.076272
hsa_circ_	-0.42219	0.076287
hsa_circ_	-0.42683	0.076301
hsa_circ_	-0.46434	0.076305
hsa_circ_	0.630188	0.076306
hsa_circ_	0.262836	0.076306
hsa_circ_	0.449567	0.076313
hsa_circ_	-0.48425	0.076315
hsa_circ_	0.540825	0.076315
hsa_circ_	0.317227	0.076337
hsa_circ_	-0.30325	0.076354
hsa_circ_	0.374391	0.076357
hsa_circ_	0.729737	0.076358
hsa_circ_	0.511699	0.076379
hsa_circ_	-0.29977	0.076404
hsa_circ_	0.570592	0.076413
hsa_circ_	0.437773	0.07644
hsa_circ_	-0.69268	0.076447
hsa_circ_	0.681613	0.07645
hsa_circ_	-0.50919	0.07645
hsa_circ_	0.89959	0.07645
hsa_circ_	0.419431	0.076454
hsa_circ_	0.416071	0.076456
hsa_circ_	-0.58328	0.076464
hsa_circ_	0.30758	0.076464
hsa_circ_	0.462256	0.076464
hsa_circ_	-0.3137	0.076468
hsa_circ_	-0.26604	0.076471
hsa_circ_	-0.89961	0.076471
hsa_circ_	0.785844	0.076471
hsa_circ_	-0.34626	0.076472
hsa_circ_	0.265497	0.076482
hsa_circ_	0.315751	0.076488
hsa_circ_	0.399723	0.076489
hsa_circ_	0.278475	0.076509
hsa_circ_	0.457638	0.07651

hsa_circ_	-0.4505	0.076528
hsa_circ_	0.73924	0.076536
hsa_circ_	0.320606	0.076538
hsa_circ_	-0.64938	0.076545
hsa_circ_	0.3374	0.076551
hsa_circ_	-0.83759	0.076555
hsa_circ_	-0.29943	0.076558
hsa_circ_	0.432271	0.07656
hsa_circ_	0.387777	0.076574
hsa_circ_	-0.53797	0.076581
hsa_circ_	0.637815	0.076597
hsa_circ_	0.364529	0.076607
hsa_circ_	-0.30971	0.076609
hsa_circ_	-1.54949	0.076612
hsa_circ_	-0.32004	0.076616
hsa_circ_	-0.40361	0.076618
hsa_circ_	-0.4136	0.07663
hsa_circ_	-0.4193	0.076633
hsa_circ_	-0.43942	0.076635
hsa_circ_	-0.53479	0.076638
hsa_circ_	0.632476	0.076638
hsa_circ_	0.423156	0.076649
hsa_circ_	-0.26007	0.076657
hsa_circ_	-0.56737	0.076658
hsa_circ_	-0.49652	0.076667
hsa_circ_	0.592137	0.076672
hsa_circ_	-0.43417	0.076673
hsa_circ_	0.558857	0.076674
hsa_circ_	0.406956	0.076689
hsa_circ_	0.87723	0.076695
hsa_circ_	-0.44041	0.076703
hsa_circ_	0.337799	0.076713
hsa_circ_	-0.62168	0.076715
hsa_circ_	0.620711	0.076717
hsa_circ_	0.3516	0.076718
hsa_circ_	0.489503	0.076722
hsa_circ_	-0.2612	0.076725
hsa_circ_	0.33447	0.076733
hsa_circ_	0.311579	0.076747
hsa_circ_	0.535082	0.076749
hsa_circ_	-0.3751	0.076753
hsa_circ_	0.60718	0.076759
hsa_circ_	-0.68915	0.076766
hsa_circ_	-0.28385	0.076778
hsa_circ_	0.819951	0.076791
hsa_circ_	-0.76018	0.076794
hsa_circ_	0.585952	0.076796
hsa_circ_	-0.30225	0.076804
hsa_circ_	-0.6069	0.076805
hsa_circ_	0.547436	0.076805
hsa_circ_	0.433902	0.07683
hsa_circ_	0.646966	0.076836
hsa_circ_	-0.36774	0.076838
hsa_circ_	-0.40117	0.076845

hsa_circ_	0.525709	0.076856
hsa_circ_	-1.39342	0.076861
hsa_circ_	-0.40478	0.076861
hsa_circ_	-0.47577	0.076867
hsa_circ_	0.328435	0.076894
hsa_circ_	-1.23027	0.076901
hsa_circ_	0.285045	0.076928
hsa_circ_	-0.32739	0.07693
hsa_circ_	0.46753	0.076937
hsa_circ_	-0.61668	0.076937
hsa_circ_	-0.51469	0.076951
hsa_circ_	0.421768	0.076963
hsa_circ_	-0.49071	0.076964
hsa_circ_	-0.26523	0.076972
hsa_circ_	1.097644	0.076974
hsa_circ_	-0.30704	0.07698
hsa_circ_	0.879218	0.076982
hsa_circ_	-0.33535	0.077002
hsa_circ_	-0.29318	0.077007
hsa_circ_	0.332218	0.077008
hsa_circ_	0.492309	0.077014
hsa_circ_	-0.56994	0.077015
hsa_circ_	0.766783	0.077017
hsa_circ_	-0.28131	0.077019
hsa_circ_	0.265659	0.07702
hsa_circ_	0.657332	0.07702
hsa_circ_	-0.28708	0.077031
hsa_circ_	-0.32944	0.077063
hsa_circ_	-0.32868	0.077079
hsa_circ_	-0.31632	0.077088
hsa_circ_	-0.66136	0.077092
hsa_circ_	-0.68587	0.077102
hsa_circ_	-0.64479	0.077106
hsa_circ_	-0.5687	0.077113
hsa_circ_	0.588591	0.077115
hsa_circ_	-0.37823	0.077134
hsa_circ_	-0.42826	0.077149
hsa_circ_	0.268373	0.077159
hsa_circ_	0.393063	0.077173
hsa_circ_	0.305724	0.077184
hsa_circ_	0.37362	0.077196
hsa_circ_	-0.44911	0.077197
hsa_circ_	0.353653	0.077238
hsa_circ_	-0.37166	0.077242
hsa_circ_	0.341661	0.077244
hsa_circ_	-0.31708	0.077246
hsa_circ_	-0.64561	0.077249
hsa_circ_	-1.30504	0.077261
hsa_circ_	-0.32048	0.077266
hsa_circ_	-0.2769	0.077282
hsa_circ_	0.428589	0.0773
hsa_circ_	0.871671	0.077309
hsa_circ_	-0.27088	0.077314
hsa_circ_	-0.26913	0.077315

hsa_circ_	0.29343	0.077316
hsa_circ_	0.276766	0.077319
hsa_circ_	-0.47807	0.077319
hsa_circ_	0.358586	0.077326
hsa_circ_	0.48663	0.077339
hsa_circ_	0.382099	0.077342
hsa_circ_	0.821944	0.077343
hsa_circ_	0.513484	0.077355
hsa_circ_	0.418018	0.077355
hsa_circ_	0.357399	0.077357
hsa_circ_	-0.43225	0.077369
hsa_circ_	0.300865	0.077374
hsa_circ_	-0.26227	0.077377
hsa_circ_	0.799576	0.077386
hsa_circ_	0.936793	0.077389
hsa_circ_	-0.31307	0.077409
hsa_circ_	0.873792	0.077412
hsa_circ_	0.409942	0.07742
hsa_circ_	0.321148	0.077424
hsa_circ_	-0.31996	0.077427
hsa_circ_	-0.30283	0.077433
hsa_circ_	0.659637	0.077435
hsa_circ_	-0.57114	0.077438
hsa_circ_	-0.34677	0.077439
hsa_circ_	-0.32885	0.077443
hsa_circ_	-0.27917	0.077444
hsa_circ_	-0.40683	0.077445
hsa_circ_	0.858491	0.077448
hsa_circ_	-0.36005	0.077451
hsa_circ_	-0.77904	0.077454
hsa_circ_	-0.33382	0.077474
hsa_circ_	0.408203	0.07749
hsa_circ_	0.537325	0.077494
hsa_circ_	0.286575	0.077518
hsa_circ_	0.72616	0.077522
hsa_circ_	0.705823	0.077524
hsa_circ_	0.283495	0.077525
hsa_circ_	0.332895	0.077528
hsa_circ_	-0.41062	0.077539
hsa_circ_	0.43971	0.077543
hsa_circ_	-0.4198	0.077544
hsa_circ_	0.532736	0.077546
hsa_circ_	0.273472	0.077551
hsa_circ_	-1.05482	0.077563
hsa_circ_	0.279076	0.077591
hsa_circ_	-0.50529	0.077598
hsa_circ_	-0.36698	0.077599
hsa_circ_	0.38688	0.0776
hsa_circ_	0.498414	0.077619
hsa_circ_	-0.56586	0.07762
hsa_circ_	-0.27628	0.077628
hsa_circ_	0.342196	0.077632
hsa_circ_	0.369996	0.077644
hsa_circ_	-0.49444	0.077652

hsa_circ_	0.399921	0.077678
hsa_circ_	0.280306	0.077681
hsa_circ_	0.351304	0.07769
hsa_circ_	0.672818	0.0777
hsa_circ_	-0.32305	0.077713
hsa_circ_	0.643771	0.077714
hsa_circ_	-0.33628	0.07772
hsa_circ_	-0.92049	0.077734
hsa_circ_	-1.08138	0.077743
hsa_circ_	0.727548	0.07776
hsa_circ_	-0.27862	0.077777
hsa_circ_	0.700275	0.07778
hsa_circ_	-0.27604	0.077782
hsa_circ_	0.519949	0.077788
hsa_circ_	-0.28865	0.077791
hsa_circ_	-0.9736	0.077793
hsa_circ_	-0.38722	0.077797
hsa_circ_	-0.99146	0.077803
hsa_circ_	0.410064	0.077811
hsa_circ_	-0.30071	0.077821
hsa_circ_	-0.48041	0.077827
hsa_circ_	-0.41218	0.077835
hsa_circ_	0.294619	0.077837
hsa_circ_	-0.5455	0.07784
hsa_circ_	-0.29819	0.077851
hsa_circ_	-0.3363	0.077872
hsa_circ_	0.417696	0.077884
hsa_circ_	-0.36334	0.077888
hsa_circ_	0.584751	0.077902
hsa_circ_	-0.32693	0.077907
hsa_circ_	-0.50397	0.077914
hsa_circ_	-0.26504	0.077921
hsa_circ_	-0.45667	0.077933
hsa_circ_	-0.31329	0.077945
hsa_circ_	-0.39836	0.077948
hsa_circ_	0.286402	0.077956
hsa_circ_	-0.78459	0.077957
hsa_circ_	0.575237	0.077964
hsa_circ_	0.28467	0.077984
hsa_circ_	0.307521	0.077985
hsa_circ_	0.468539	0.077987
hsa_circ_	-0.44881	0.077987
hsa_circ_	0.463876	0.077991
hsa_circ_	0.292764	0.077993
hsa_circ_	-0.53485	0.077998
hsa_circ_	-0.30303	0.078003
hsa_circ_	0.518457	0.078015
hsa_circ_	-0.46563	0.078017
hsa_circ_	-0.38053	0.078022
hsa_circ_	0.421083	0.078038
hsa_circ_	0.552656	0.078041
hsa_circ_	-0.69207	0.078057
hsa_circ_	-0.66536	0.078076
hsa_circ_	1.089908	0.078076

hsa_circ_	-0.30131	0.078078
hsa_circ_	0.566945	0.078082
hsa_circ_	0.553349	0.078086
hsa_circ_	-0.9645	0.078086
hsa_circ_	-0.32787	0.078091
hsa_circ_	0.552622	0.0781
hsa_circ_	-0.30077	0.078105
hsa_circ_	0.326003	0.078109
hsa_circ_	-0.42123	0.078117
hsa_circ_	-0.30495	0.07812
hsa_circ_	-0.31319	0.078122
hsa_circ_	0.511482	0.078135
hsa_circ_	-1.2104	0.078139
hsa_circ_	0.398166	0.078156
hsa_circ_	-0.34484	0.078157
hsa_circ_	0.454394	0.078159
hsa_circ_	-0.81995	0.078167
hsa_circ_	-0.53781	0.07817
hsa_circ_	0.359857	0.078175
hsa_circ_	0.609546	0.07819
hsa_circ_	0.486715	0.078219
hsa_circ_	-0.65542	0.07825
hsa_circ_	-0.62563	0.078258
hsa_circ_	-0.34861	0.078259
hsa_circ_	-0.53973	0.078264
hsa_circ_	0.400564	0.078266
hsa_circ_	-0.29457	0.078268
hsa_circ_	0.345821	0.078269
hsa_circ_	-0.30157	0.078271
hsa_circ_	0.637825	0.078275
hsa_circ_	-1.04363	0.078286
hsa_circ_	0.487464	0.078297
hsa_circ_	0.538861	0.078299
hsa_circ_	0.65516	0.078304
hsa_circ_	-0.70246	0.078325
hsa_circ_	-2.40581	0.078331
hsa_circ_	0.257101	0.078333
hsa_circ_	-0.57172	0.078336
hsa_circ_	1.073379	0.078343
hsa_circ_	0.59303	0.078344
hsa_circ_	0.336753	0.078349
hsa_circ_	0.845992	0.078349
hsa_circ_	-0.33625	0.078354
hsa_circ_	-0.72227	0.078361
hsa_circ_	-0.39216	0.078365
hsa_circ_	-0.30246	0.078366
hsa_circ_	-1.02754	0.078368
hsa_circ_	-0.49336	0.078372
hsa_circ_	0.278698	0.078382
hsa_circ_	-0.57043	0.078387
hsa_circ_	0.432429	0.07839
hsa_circ_	0.299203	0.078394
hsa_circ_	-0.80298	0.078404
hsa_circ_	0.319982	0.078405

hsa_circ_	-0.42222	0.078409
hsa_circ_	-0.59636	0.078424
hsa_circ_	-0.77025	0.078427
hsa_circ_	0.359951	0.078432
hsa_circ_	-0.3683	0.078432
hsa_circ_	-0.31249	0.078432
hsa_circ_	0.364632	0.078438
hsa_circ_	0.460576	0.078444
hsa_circ_	-0.31576	0.078447
hsa_circ_	-0.27439	0.078458
hsa_circ_	0.303053	0.078461
hsa_circ_	-0.54359	0.078468
hsa_circ_	0.311046	0.078471
hsa_circ_	-0.26772	0.078473
hsa_circ_	-0.37488	0.078477
hsa_circ_	0.778566	0.078484
hsa_circ_	-0.28666	0.078486
hsa_circ_	0.438408	0.078492
hsa_circ_	1.443043	0.078497
hsa_circ_	-0.39107	0.078504
hsa_circ_	0.310236	0.078508
hsa_circ_	0.582662	0.078517
hsa_circ_	0.33049	0.078518
hsa_circ_	0.566676	0.07852
hsa_circ_	-0.84466	0.078529
hsa_circ_	0.339596	0.078531
hsa_circ_	-0.43558	0.078543
hsa_circ_	-0.82856	0.078546
hsa_circ_	0.358974	0.078558
hsa_circ_	0.319133	0.078571
hsa_circ_	0.371965	0.078577
hsa_circ_	-0.82578	0.078591
hsa_circ_	-0.39754	0.078607
hsa_circ_	-0.31353	0.078611
hsa_circ_	-0.31551	0.078617
hsa_circ_	0.307037	0.078619
hsa_circ_	0.395488	0.07862
hsa_circ_	0.401304	0.078628
hsa_circ_	-0.36447	0.078634
hsa_circ_	-0.32362	0.078645
hsa_circ_	0.29746	0.078647
hsa_circ_	0.713032	0.078654
hsa_circ_	-0.68101	0.078657
hsa_circ_	0.440498	0.07867
hsa_circ_	-1.38707	0.078684
hsa_circ_	-0.74707	0.07869
hsa_circ_	-0.78325	0.078693
hsa_circ_	0.630861	0.0787
hsa_circ_	-0.28839	0.078705
hsa_circ_	0.414535	0.078706
hsa_circ_	-0.78345	0.078708
hsa_circ_	0.431771	0.07871
hsa_circ_	0.337112	0.078717
hsa_circ_	-0.26457	0.078722

hsa_circ_	0.535342	0.07873
hsa_circ_	-1.22592	0.07875
hsa_circ_	-0.25869	0.078752
hsa_circ_	-0.93684	0.078756
hsa_circ_	-0.30418	0.078759
hsa_circ_	0.488627	0.078769
hsa_circ_	0.953055	0.078771
hsa_circ_	0.315971	0.078771
hsa_circ_	-0.50746	0.078773
hsa_circ_	-0.2575	0.078782
hsa_circ_	-0.27537	0.078782
hsa_circ_	-0.38465	0.078785
hsa_circ_	-0.5277	0.07879
hsa_circ_	0.321518	0.078792
hsa_circ_	0.353307	0.078797
hsa_circ_	0.61982	0.078798
hsa_circ_	-0.26705	0.078799
hsa_circ_	0.305845	0.078805
hsa_circ_	-1.06752	0.078806
hsa_circ_	0.544415	0.078807
hsa_circ_	1.083046	0.07881
hsa_circ_	-0.32464	0.078819
hsa_circ_	0.314103	0.07885
hsa_circ_	-0.31642	0.078857
hsa_circ_	0.528716	0.078866
hsa_circ_	0.530996	0.078866
hsa_circ_	-0.90094	0.078893
hsa_circ_	-0.28946	0.078895
hsa_circ_	-0.42715	0.078897
hsa_circ_	1.019079	0.078898
hsa_circ_	-0.40205	0.078902
hsa_circ_	-0.4613	0.078916
hsa_circ_	-0.27801	0.078921
hsa_circ_	0.527674	0.078952
hsa_circ_	-0.65796	0.078957
hsa_circ_	-0.41476	0.078963
hsa_circ_	-0.3095	0.078964
hsa_circ_	0.402077	0.07897
hsa_circ_	-0.41225	0.07898
hsa_circ_	0.992142	0.078992
hsa_circ_	-0.28244	0.078992
hsa_circ_	0.682574	0.078992
hsa_circ_	-0.3121	0.078999
hsa_circ_	-0.45429	0.079007
hsa_circ_	0.602129	0.079011
hsa_circ_	-0.32318	0.079022
hsa_circ_	0.281593	0.079022
hsa_circ_	-0.29712	0.079028
hsa_circ_	-0.30446	0.079032
hsa_circ_	0.328145	0.07906
hsa_circ_	-0.45353	0.079065
hsa_circ_	0.358461	0.079067
hsa_circ_	-0.32609	0.07907
hsa_circ_	1.031298	0.079088

hsa_circ_	0.37983	0.079089
hsa_circ_	-0.34377	0.079098
hsa_circ_	-0.31717	0.079117
hsa_circ_	-0.37627	0.079125
hsa_circ_	0.5676	0.079131
hsa_circ_	0.398995	0.079147
hsa_circ_	-0.33582	0.07915
hsa_circ_	-0.45345	0.079153
hsa_circ_	0.753394	0.079158
hsa_circ_	0.378962	0.079158
hsa_circ_	0.321664	0.079163
hsa_circ_	-0.26624	0.079169
hsa_circ_	0.346079	0.079171
hsa_circ_	-0.50538	0.079176
hsa_circ_	-0.31519	0.079183
hsa_circ_	0.752229	0.079186
hsa_circ_	-0.358	0.07919
hsa_circ_	0.773826	0.079224
hsa_circ_	-0.30053	0.079233
hsa_circ_	0.551934	0.079237
hsa_circ_	-0.31677	0.079261
hsa_circ_	-0.78705	0.079268
hsa_circ_	0.936867	0.079275
hsa_circ_	-0.66359	0.079276
hsa_circ_	-0.65157	0.079287
hsa_circ_	-0.56572	0.07929
hsa_circ_	-0.31222	0.079295
hsa_circ_	0.70366	0.079297
hsa_circ_	0.612917	0.079306
hsa_circ_	-0.26645	0.079309
hsa_circ_	0.654463	0.079316
hsa_circ_	-0.37432	0.079322
hsa_circ_	-0.36344	0.079327
hsa_circ_	-0.33625	0.079332
hsa_circ_	-0.40691	0.079339
hsa_circ_	0.415049	0.07934
hsa_circ_	-0.59077	0.079351
hsa_circ_	-0.28582	0.079352
hsa_circ_	0.351925	0.079359
hsa_circ_	0.3556	0.079366
hsa_circ_	0.618976	0.079367
hsa_circ_	0.573258	0.079382
hsa_circ_	-0.41953	0.079387
hsa_circ_	0.853894	0.079392
hsa_circ_	0.280256	0.0794
hsa_circ_	-0.35313	0.079401
hsa_circ_	0.879567	0.079411
hsa_circ_	-0.33954	0.079411
hsa_circ_	-0.72513	0.079425
hsa_circ_	0.312125	0.079429
hsa_circ_	-0.58544	0.07945
hsa_circ_	-1.2186	0.07945
hsa_circ_	0.598565	0.079471
hsa_circ_	-0.35421	0.079479

hsa_circ_	0.871272	0.079481
hsa_circ_	-0.46425	0.079485
hsa_circ_	-0.36968	0.079491
hsa_circ_	-1.27128	0.079504
hsa_circ_	0.388663	0.079508
hsa_circ_	0.550679	0.079514
hsa_circ_	0.384651	0.079526
hsa_circ_	-0.3944	0.07954
hsa_circ_	-1.00419	0.079541
hsa_circ_	-0.73164	0.079562
hsa_circ_	0.540123	0.079562
hsa_circ_	-0.91625	0.079569
hsa_circ_	0.338811	0.079576
hsa_circ_	-0.4565	0.07958
hsa_circ_	-0.31114	0.079582
hsa_circ_	0.369247	0.079587
hsa_circ_	0.331138	0.079607
hsa_circ_	-0.5409	0.079611
hsa_circ_	1.180078	0.079611
hsa_circ_	-0.43272	0.07962
hsa_circ_	0.443695	0.079624
hsa_circ_	-1.64747	0.07964
hsa_circ_	-0.25711	0.079648
hsa_circ_	-0.25485	0.079653
hsa_circ_	-1.31484	0.079653
hsa_circ_	-0.82168	0.079655
hsa_circ_	-0.25143	0.079664
hsa_circ_	-0.49672	0.079667
hsa_circ_	-0.57824	0.079673
hsa_circ_	-0.40904	0.079682
hsa_circ_	-0.76386	0.079685
hsa_circ_	-0.28077	0.079689
hsa_circ_	-0.28146	0.0797
hsa_circ_	0.624733	0.0797
hsa_circ_	-0.96855	0.079705
hsa_circ_	0.49448	0.079714
hsa_circ_	0.305094	0.079719
hsa_circ_	-0.51017	0.07973
hsa_circ_	-0.29309	0.079736
hsa_circ_	-0.50698	0.079739
hsa_circ_	0.38918	0.079753
hsa_circ_	0.420089	0.07977
hsa_circ_	0.27877	0.07977
hsa_circ_	-0.57005	0.079776
hsa_circ_	-0.2739	0.079784
hsa_circ_	-0.35852	0.079786
hsa_circ_	-0.37459	0.079796
hsa_circ_	0.38498	0.0798
hsa_circ_	0.39747	0.07984
hsa_circ_	0.390664	0.079847
hsa_circ_	0.357651	0.07985
hsa_circ_	-0.49792	0.079853
hsa_circ_	0.927438	0.079857
hsa_circ_	0.397397	0.079859

hsa_circ_	0.909985	0.079868
hsa_circ_	-0.30146	0.079872
hsa_circ_	0.353834	0.079876
hsa_circ_	1.353747	0.079901
hsa_circ_	0.266653	0.079904
hsa_circ_	-0.4205	0.079913
hsa_circ_	-0.2775	0.079928
hsa_circ_	-0.44038	0.079937
hsa_circ_	-0.88085	0.079939
hsa_circ_	0.482288	0.07995
hsa_circ_	-0.30156	0.079975
hsa_circ_	0.269979	0.079978
hsa_circ_	-0.29277	0.07998
hsa_circ_	-0.39783	0.079983
hsa_circ_	-0.3101	0.079991
hsa_circ_	0.575827	0.079991
hsa_circ_	-0.28579	0.079993
hsa_circ_	-0.37063	0.080013
hsa_circ_	0.910012	0.080021
hsa_circ_	-0.61162	0.080038
hsa_circ_	1.443572	0.080039
hsa_circ_	-0.61423	0.080039
hsa_circ_	0.693606	0.080072
hsa_circ_	-0.30414	0.08008
hsa_circ_	-0.31855	0.080084
hsa_circ_	0.284204	0.080085
hsa_circ_	-0.28977	0.080088
hsa_circ_	-0.31403	0.080128
hsa_circ_	0.30173	0.080147
hsa_circ_	-0.91792	0.080158
hsa_circ_	-0.32578	0.080166
hsa_circ_	0.491071	0.080168
hsa_circ_	0.301495	0.080169
hsa_circ_	-0.73042	0.080172
hsa_circ_	0.439765	0.080179
hsa_circ_	0.414384	0.080181
hsa_circ_	-0.92657	0.080184
hsa_circ_	0.313409	0.080186
hsa_circ_	-0.26452	0.080195
hsa_circ_	-0.28473	0.080197
hsa_circ_	0.304473	0.080198
hsa_circ_	-0.28687	0.080203
hsa_circ_	0.34343	0.080244
hsa_circ_	-0.45089	0.080246
hsa_circ_	-0.31205	0.080253
hsa_circ_	0.479482	0.080255
hsa_circ_	0.386609	0.080256
hsa_circ_	0.555715	0.080259
hsa_circ_	0.320383	0.08026
hsa_circ_	-0.38611	0.080265
hsa_circ_	-0.41887	0.080275
hsa_circ_	0.346613	0.080276
hsa_circ_	-1.06639	0.080292
hsa_circ_	-0.36026	0.080296

hsa_circ_	-0.46904	0.080302
hsa_circ_	-0.28743	0.080336
hsa_circ_	-0.67341	0.080336
hsa_circ_	-0.51716	0.080343
hsa_circ_	0.570821	0.080346
hsa_circ_	0.329246	0.080352
hsa_circ_	0.41378	0.080365
hsa_circ_	-0.99151	0.08037
hsa_circ_	-0.31769	0.080378
hsa_circ_	0.375798	0.080386
hsa_circ_	-0.36524	0.08042
hsa_circ_	0.342894	0.080427
hsa_circ_	-0.3613	0.080434
hsa_circ_	0.743207	0.080437
hsa_circ_	0.41782	0.080438
hsa_circ_	0.293522	0.080447
hsa_circ_	1.018349	0.080452
hsa_circ_	-0.59592	0.080454
hsa_circ_	0.535372	0.080477
hsa_circ_	0.387602	0.080482
hsa_circ_	0.594223	0.080493
hsa_circ_	-0.32307	0.080495
hsa_circ_	-0.34146	0.080516
hsa_circ_	-0.45428	0.080516
hsa_circ_	0.487484	0.080516
hsa_circ_	0.591585	0.080521
hsa_circ_	-0.35428	0.080524
hsa_circ_	0.334758	0.080528
hsa_circ_	0.983129	0.080528
hsa_circ_	-0.40988	0.080542
hsa_circ_	0.52098	0.080544
hsa_circ_	-0.4099	0.080555
hsa_circ_	0.360459	0.080556
hsa_circ_	0.429875	0.080561
hsa_circ_	-0.75201	0.080566
hsa_circ_	0.452487	0.080571
hsa_circ_	0.439165	0.080575
hsa_circ_	-1.13356	0.080579
hsa_circ_	0.524257	0.080582
hsa_circ_	-0.32662	0.080598
hsa_circ_	-0.5049	0.0806
hsa_circ_	-0.42177	0.080606
hsa_circ_	-0.29471	0.080617
hsa_circ_	-1.36953	0.080619
hsa_circ_	0.30522	0.080625
hsa_circ_	0.64156	0.080642
hsa_circ_	0.468773	0.08065
hsa_circ_	-0.2881	0.080657
hsa_circ_	0.288057	0.080657
hsa_circ_	-0.296	0.080658
hsa_circ_	-0.74055	0.080666
hsa_circ_	0.468573	0.080684
hsa_circ_	-0.71925	0.080686
hsa_circ_	0.343047	0.080687

hsa_circ_	0.411153	0.080688
hsa_circ_	0.43095	0.080688
hsa_circ_	-0.44752	0.080689
hsa_circ_	1.388385	0.080691
hsa_circ_	-0.31319	0.080692
hsa_circ_	0.48391	0.080697
hsa_circ_	0.330417	0.080699
hsa_circ_	0.343485	0.080701
hsa_circ_	0.52902	0.080706
hsa_circ_	0.350034	0.080706
hsa_circ_	-0.65833	0.08071
hsa_circ_	0.326751	0.080724
hsa_circ_	-0.72188	0.080729
hsa_circ_	-0.38158	0.08073
hsa_circ_	-0.36814	0.080733
hsa_circ_	0.273882	0.080734
hsa_circ_	0.508115	0.080768
hsa_circ_	-0.33976	0.080815
hsa_circ_	-0.74773	0.080828
hsa_circ_	-0.58849	0.08084
hsa_circ_	0.440098	0.080848
hsa_circ_	-0.43783	0.080881
hsa_circ_	-0.71377	0.08089
hsa_circ_	0.330412	0.080896
hsa_circ_	0.574294	0.080902
hsa_circ_	0.297516	0.080905
hsa_circ_	-0.36308	0.080914
hsa_circ_	-0.33459	0.080924
hsa_circ_	0.392761	0.080926
hsa_circ_	0.503234	0.080935
hsa_circ_	-0.38028	0.080951
hsa_circ_	0.297388	0.080951
hsa_circ_	-0.26941	0.080956
hsa_circ_	0.418072	0.080974
hsa_circ_	0.436192	0.080978
hsa_circ_	-0.32483	0.080981
hsa_circ_	-0.57921	0.080982
hsa_circ_	0.512957	0.080985
hsa_circ_	0.359664	0.080989
hsa_circ_	-0.34665	0.080992
hsa_circ_	0.307376	0.080994
hsa_circ_	0.648825	0.081003
hsa_circ_	-1.80973	0.081004
hsa_circ_	-0.29239	0.08101
hsa_circ_	-0.77046	0.081044
hsa_circ_	0.398031	0.081059
hsa_circ_	-0.37335	0.081069
hsa_circ_	0.303339	0.081073
hsa_circ_	-0.3775	0.081074
hsa_circ_	0.489567	0.081077
hsa_circ_	-0.41621	0.081083
hsa_circ_	-0.61872	0.081094
hsa_circ_	-0.30849	0.081095
hsa_circ_	0.358857	0.081103

hsa_circ_	0.410579	0.08111
hsa_circ_	0.409174	0.081112
hsa_circ_	-0.59825	0.081112
hsa_circ_	-0.7034	0.081117
hsa_circ_	-1.10207	0.081119
hsa_circ_	-0.55906	0.08112
hsa_circ_	-0.3238	0.081123
hsa_circ_	-0.90045	0.081124
hsa_circ_	-0.36806	0.08113
hsa_circ_	0.543475	0.081147
hsa_circ_	0.355014	0.081153
hsa_circ_	0.516327	0.081158
hsa_circ_	0.303322	0.081171
hsa_circ_	-0.37015	0.081202
hsa_circ_	-0.36462	0.081211
hsa_circ_	-0.34333	0.081213
hsa_circ_	-0.58952	0.081215
hsa_circ_	1.087641	0.081229
hsa_circ_	0.75374	0.081234
hsa_circ_	0.499393	0.081236
hsa_circ_	-0.25765	0.081241
hsa_circ_	0.371108	0.081244
hsa_circ_	-0.32599	0.081251
hsa_circ_	-0.26761	0.081269
hsa_circ_	0.262357	0.081274
hsa_circ_	1.480551	0.081278
hsa_circ_	0.260768	0.081298
hsa_circ_	0.462352	0.081304
hsa_circ_	0.708067	0.081305
hsa_circ_	0.698439	0.081308
hsa_circ_	0.274272	0.081308
hsa_circ_	-0.57322	0.081315
hsa_circ_	-0.56664	0.081317
hsa_circ_	0.775863	0.081318
hsa_circ_	0.941987	0.081323
hsa_circ_	0.309841	0.081344
hsa_circ_	0.771025	0.08135
hsa_circ_	-0.55466	0.081365
hsa_circ_	0.714132	0.08137
hsa_circ_	-0.35085	0.081378
hsa_circ_	-0.31278	0.081378
hsa_circ_	-1.09164	0.081381
hsa_circ_	-0.3285	0.081389
hsa_circ_	0.45009	0.0814
hsa_circ_	0.616497	0.08141
hsa_circ_	0.565829	0.081414
hsa_circ_	-0.33747	0.081417
hsa_circ_	-0.32849	0.081422
hsa_circ_	0.509242	0.081427
hsa_circ_	-0.47573	0.081428
hsa_circ_	-0.33613	0.081431
hsa_circ_	0.723661	0.081432
hsa_circ_	0.704124	0.081443
hsa_circ_	0.374915	0.081445

hsa_circ_	0.547514	0.08145
hsa_circ_	-0.77306	0.081457
hsa_circ_	-0.3571	0.081457
hsa_circ_	-0.36372	0.081458
hsa_circ_	-0.39518	0.081458
hsa_circ_	-0.50647	0.081471
hsa_circ_	0.375285	0.081472
hsa_circ_	0.760109	0.081478
hsa_circ_	0.397889	0.081479
hsa_circ_	-0.30686	0.081496
hsa_circ_	-0.33483	0.08151
hsa_circ_	-0.38599	0.081516
hsa_circ_	0.53834	0.081519
hsa_circ_	-0.44941	0.081529
hsa_circ_	0.27155	0.081531
hsa_circ_	-2.01893	0.081537
hsa_circ_	0.40944	0.081539
hsa_circ_	0.811489	0.081543
hsa_circ_	0.315702	0.081549
hsa_circ_	0.525392	0.081554
hsa_circ_	0.557457	0.081555
hsa_circ_	-0.44549	0.081557
hsa_circ_	-0.4022	0.081571
hsa_circ_	-0.28146	0.081577
hsa_circ_	0.516186	0.081581
hsa_circ_	-0.3444	0.081586
hsa_circ_	-0.36704	0.081601
hsa_circ_	0.608477	0.081605
hsa_circ_	0.331611	0.081612
hsa_circ_	0.468429	0.081621
hsa_circ_	1.192873	0.081637
hsa_circ_	-0.72512	0.081647
hsa_circ_	-0.49241	0.081661
hsa_circ_	-0.56177	0.081675
hsa_circ_	-0.65681	0.081688
hsa_circ_	-0.55833	0.081711
hsa_circ_	-0.8661	0.081718
hsa_circ_	0.607916	0.081732
hsa_circ_	-0.43323	0.081741
hsa_circ_	-0.45632	0.081745
hsa_circ_	0.461627	0.081763
hsa_circ_	0.360186	0.081773
hsa_circ_	0.303427	0.081776
hsa_circ_	0.532061	0.08178
hsa_circ_	-0.29781	0.081787
hsa_circ_	-0.34098	0.081788
hsa_circ_	-1.43211	0.081789
hsa_circ_	0.50212	0.081794
hsa_circ_	1.496312	0.081795
hsa_circ_	-0.49926	0.081797
hsa_circ_	-0.53471	0.081808
hsa_circ_	-0.3679	0.081826
hsa_circ_	-0.92163	0.081826
hsa_circ_	-0.46749	0.08184

hsa_circ_	0.518425	0.081848
hsa_circ_	0.482298	0.081859
hsa_circ_	-0.31221	0.081891
hsa_circ_	0.720801	0.081896
hsa_circ_	0.394901	0.081914
hsa_circ_	-0.278	0.081917
hsa_circ_	-0.31231	0.08192
hsa_circ_	0.297388	0.081922
hsa_circ_	0.373098	0.081927
hsa_circ_	0.333419	0.081931
hsa_circ_	0.355003	0.081935
hsa_circ_	0.294978	0.081937
hsa_circ_	0.802947	0.081937
hsa_circ_	0.606118	0.081938
hsa_circ_	-0.29842	0.081939
hsa_circ_	-0.33402	0.08194
hsa_circ_	-0.31165	0.081944
hsa_circ_	0.74409	0.081945
hsa_circ_	-0.36458	0.081953
hsa_circ_	-0.66109	0.081954
hsa_circ_	-0.72319	0.081955
hsa_circ_	0.342452	0.081958
hsa_circ_	-0.44441	0.081962
hsa_circ_	0.41458	0.081966
hsa_circ_	-0.59636	0.08197
hsa_circ_	1.632882	0.081973
hsa_circ_	-0.33628	0.081973
hsa_circ_	0.415768	0.081984
hsa_circ_	-0.36472	0.081988
hsa_circ_	-0.49153	0.081993
hsa_circ_	-0.31563	0.081998
hsa_circ_	-0.37244	0.082019
hsa_circ_	0.418548	0.082021
hsa_circ_	0.409889	0.082023
hsa_circ_	0.672767	0.082023
hsa_circ_	0.296549	0.082027
hsa_circ_	0.537097	0.082031
hsa_circ_	-0.51897	0.08204
hsa_circ_	-0.42367	0.082057
hsa_circ_	-0.59319	0.082061
hsa_circ_	-0.33425	0.08209
hsa_circ_	-0.28875	0.082093
hsa_circ_	-0.58065	0.082097
hsa_circ_	-0.3313	0.082104
hsa_circ_	0.365992	0.08211
hsa_circ_	0.510976	0.082113
hsa_circ_	0.443493	0.08212
hsa_circ_	0.274238	0.082123
hsa_circ_	0.586768	0.082124
hsa_circ_	0.46454	0.082125
hsa_circ_	-0.45048	0.082136
hsa_circ_	0.593055	0.082141
hsa_circ_	-0.44671	0.082147
hsa_circ_	-0.84053	0.082157

hsa_circ_	-0.55	0.08216
hsa_circ_	0.553366	0.08217
hsa_circ_	-0.30275	0.082183
hsa_circ_	-0.41328	0.082186
hsa_circ_	0.606514	0.082189
hsa_circ_	0.556896	0.082202
hsa_circ_	0.77353	0.082203
hsa_circ_	-0.4346	0.082203
hsa_circ_	-0.43764	0.08221
hsa_circ_	-0.3577	0.082218
hsa_circ_	0.356193	0.08222
hsa_circ_	-0.74114	0.082221
hsa_circ_	0.261911	0.082228
hsa_circ_	0.262147	0.082232
hsa_circ_	-0.51126	0.082235
hsa_circ_	0.712061	0.082242
hsa_circ_	-0.30596	0.082249
hsa_circ_	-0.3006	0.082261
hsa_circ_	-1.01423	0.082261
hsa_circ_	0.301533	0.082265
hsa_circ_	-0.28853	0.082267
hsa_circ_	-0.57863	0.082268
hsa_circ_	-0.36723	0.082274
hsa_circ_	-0.44172	0.082276
hsa_circ_	-0.29626	0.082276
hsa_circ_	-0.51825	0.08228
hsa_circ_	0.322221	0.082284
hsa_circ_	0.579515	0.082298
hsa_circ_	-0.42345	0.082299
hsa_circ_	-1.08674	0.0823
hsa_circ_	0.426997	0.082303
hsa_circ_	0.565647	0.082306
hsa_circ_	-0.84955	0.08231
hsa_circ_	-0.64508	0.082316
hsa_circ_	0.477957	0.082319
hsa_circ_	0.317712	0.082341
hsa_circ_	-0.56076	0.082342
hsa_circ_	-0.37552	0.082345
hsa_circ_	0.458349	0.082352
hsa_circ_	-0.43078	0.082354
hsa_circ_	0.361119	0.082357
hsa_circ_	0.537325	0.082363
hsa_circ_	0.367578	0.082368
hsa_circ_	-0.27195	0.082377
hsa_circ_	-0.7437	0.082384
hsa_circ_	-0.37665	0.082387
hsa_circ_	0.732759	0.082392
hsa_circ_	0.652799	0.082395
hsa_circ_	-0.70283	0.082399
hsa_circ_	0.477089	0.0824
hsa_circ_	0.3242	0.082401
hsa_circ_	-0.31463	0.082404
hsa_circ_	0.297016	0.082412
hsa_circ_	0.28796	0.082413

hsa_circ_	-0.85442	0.082416
hsa_circ_	0.317975	0.082422
hsa_circ_	0.301396	0.082423
hsa_circ_	-0.47568	0.08243
hsa_circ_	-0.96902	0.082432
hsa_circ_	0.543376	0.082444
hsa_circ_	0.74452	0.082447
hsa_circ_	0.280944	0.08245
hsa_circ_	-0.26811	0.082453
hsa_circ_	0.421748	0.082455
hsa_circ_	-0.249	0.082466
hsa_circ_	0.43667	0.082469
hsa_circ_	-0.33129	0.082473
hsa_circ_	0.369262	0.082491
hsa_circ_	-0.31462	0.082495
hsa_circ_	-0.28602	0.082513
hsa_circ_	-1.48529	0.082518
hsa_circ_	-0.31492	0.082527
hsa_circ_	0.595486	0.08253
hsa_circ_	-0.29854	0.082531
hsa_circ_	0.775092	0.082532
hsa_circ_	-0.77218	0.082538
hsa_circ_	-0.57991	0.082542
hsa_circ_	0.40739	0.082543
hsa_circ_	0.478533	0.08255
hsa_circ_	-0.36699	0.082553
hsa_circ_	-0.35496	0.082554
hsa_circ_	0.289085	0.082563
hsa_circ_	0.33697	0.082563
hsa_circ_	0.421464	0.082568
hsa_circ_	0.314795	0.082569
hsa_circ_	0.253206	0.08257
hsa_circ_	0.310314	0.082576
hsa_circ_	-0.43756	0.08258
hsa_circ_	0.390301	0.082583
hsa_circ_	0.321116	0.0826
hsa_circ_	0.670765	0.082608
hsa_circ_	-0.43659	0.082623
hsa_circ_	0.715782	0.082626
hsa_circ_	0.660359	0.082634
hsa_circ_	0.782114	0.082635
hsa_circ_	-0.6171	0.08264
hsa_circ_	0.48438	0.082651
hsa_circ_	0.593491	0.082656
hsa_circ_	-0.25593	0.082657
hsa_circ_	-0.30118	0.082665
hsa_circ_	0.304117	0.08267
hsa_circ_	0.46215	0.082671
hsa_circ_	-0.27894	0.082671
hsa_circ_	0.442683	0.082693
hsa_circ_	-0.59662	0.082693
hsa_circ_	-0.62364	0.082695
hsa_circ_	0.38822	0.082699
hsa_circ_	-0.25307	0.082701

hsa_circ_	-0.29987	0.082707
hsa_circ_	0.447802	0.082709
hsa_circ_	0.326717	0.082721
hsa_circ_	0.525535	0.082725
hsa_circ_	-0.7475	0.082727
hsa_circ_	0.814899	0.082729
hsa_circ_	0.697875	0.082742
hsa_circ_	0.328294	0.082742
hsa_circ_	0.56421	0.082744
hsa_circ_	-0.30674	0.082745
hsa_circ_	-0.29368	0.082749
hsa_circ_	-0.93981	0.082752
hsa_circ_	0.761643	0.082756
hsa_circ_	-0.25682	0.082758
hsa_circ_	-0.48027	0.08278
hsa_circ_	0.454818	0.082782
hsa_circ_	-0.40576	0.082788
hsa_circ_	-0.49653	0.082793
hsa_circ_	-0.52006	0.082794
hsa_circ_	-0.70658	0.082799
hsa_circ_	0.577822	0.082815
hsa_circ_	0.503361	0.08282
hsa_circ_	1.29964	0.082834
hsa_circ_	0.630378	0.08284
hsa_circ_	0.340585	0.082845
hsa_circ_	0.326649	0.082849
hsa_circ_	-0.30774	0.082856
hsa_circ_	-1.14783	0.082868
hsa_circ_	-0.32206	0.082872
hsa_circ_	0.644449	0.082873
hsa_circ_	0.694425	0.082874
hsa_circ_	0.350467	0.082876
hsa_circ_	0.394153	0.082878
hsa_circ_	0.490367	0.082905
hsa_circ_	-0.3654	0.08291
hsa_circ_	-0.56655	0.082916
hsa_circ_	0.452076	0.082919
hsa_circ_	-0.31807	0.082939
hsa_circ_	0.584688	0.08294
hsa_circ_	-0.60249	0.082943
hsa_circ_	0.518941	0.082957
hsa_circ_	-0.4136	0.082967
hsa_circ_	0.888417	0.082968
hsa_circ_	-0.44532	0.082976
hsa_circ_	-0.30458	0.082978
hsa_circ_	0.467195	0.082985
hsa_circ_	0.383905	0.082988
hsa_circ_	-0.63355	0.082991
hsa_circ_	0.467473	0.082994
hsa_circ_	-0.25494	0.083018
hsa_circ_	0.660374	0.083038
hsa_circ_	0.269802	0.083038
hsa_circ_	-0.50121	0.083048
hsa_circ_	0.451753	0.08305

hsa_circ_	-0.30658	0.083051
hsa_circ_	0.475368	0.083053
hsa_circ_	0.421916	0.083056
hsa_circ_	-0.36667	0.08307
hsa_circ_	0.451502	0.083075
hsa_circ_	0.4815	0.083079
hsa_circ_	-0.4003	0.083079
hsa_circ_	-0.37948	0.083082
hsa_circ_	-0.76545	0.083087
hsa_circ_	0.327242	0.08309
hsa_circ_	-0.34173	0.083098
hsa_circ_	-0.31511	0.083103
hsa_circ_	0.369411	0.08311
hsa_circ_	-0.6009	0.083113
hsa_circ_	-0.92649	0.08312
hsa_circ_	0.689953	0.08312
hsa_circ_	0.407924	0.083128
hsa_circ_	-0.41213	0.083133
hsa_circ_	0.411454	0.083149
hsa_circ_	-0.33487	0.083153
hsa_circ_	-0.469	0.083156
hsa_circ_	-0.35887	0.083156
hsa_circ_	-0.33856	0.083166
hsa_circ_	-1.12521	0.083173
hsa_circ_	1.300477	0.083176
hsa_circ_	-0.64675	0.083182
hsa_circ_	0.932845	0.083185
hsa_circ_	-0.28205	0.083203
hsa_circ_	-0.78618	0.083213
hsa_circ_	-0.4242	0.083216
hsa_circ_	-0.44801	0.083232
hsa_circ_	-1.5165	0.083253
hsa_circ_	0.343886	0.083264
hsa_circ_	0.641351	0.08327
hsa_circ_	-0.31661	0.083271
hsa_circ_	-0.29911	0.083276
hsa_circ_	-0.40505	0.083282
hsa_circ_	-0.39599	0.083284
hsa_circ_	-0.37576	0.083294
hsa_circ_	-0.48914	0.083295
hsa_circ_	-0.42017	0.083303
hsa_circ_	-0.61798	0.083307
hsa_circ_	0.53842	0.083323
hsa_circ_	-0.40602	0.083323
hsa_circ_	0.373323	0.083324
hsa_circ_	0.426113	0.083327
hsa_circ_	0.384331	0.08333
hsa_circ_	-0.33573	0.083356
hsa_circ_	0.669196	0.083364
hsa_circ_	-0.46563	0.083371
hsa_circ_	0.646777	0.083373
hsa_circ_	-0.40135	0.083384
hsa_circ_	-0.5026	0.083388
hsa_circ_	-0.55583	0.083393

hsa_circ_	-0.77979	0.083395
hsa_circ_	-0.2792	0.083395
hsa_circ_	0.378835	0.083406
hsa_circ_	0.695936	0.083415
hsa_circ_	-0.31336	0.083423
hsa_circ_	-0.44931	0.083442
hsa_circ_	-0.32265	0.083443
hsa_circ_	-0.52011	0.083445
hsa_circ_	0.721747	0.083452
hsa_circ_	-0.28254	0.083465
hsa_circ_	-0.30213	0.083469
hsa_circ_	0.44281	0.083476
hsa_circ_	0.459577	0.083477
hsa_circ_	-0.90264	0.083477
hsa_circ_	-0.48764	0.083479
hsa_circ_	-0.26555	0.083484
hsa_circ_	0.559203	0.083484
hsa_circ_	-1.14072	0.08349
hsa_circ_	-0.28897	0.083497
hsa_circ_	-1.00002	0.083502
hsa_circ_	-0.32984	0.083517
hsa_circ_	0.716497	0.083518
hsa_circ_	0.282912	0.083522
hsa_circ_	-0.26651	0.083525
hsa_circ_	-0.57416	0.083529
hsa_circ_	0.776223	0.083531
hsa_circ_	0.469873	0.083535
hsa_circ_	0.415651	0.083548
hsa_circ_	-0.29957	0.083557
hsa_circ_	-0.4061	0.083557
hsa_circ_	0.517391	0.083558
hsa_circ_	0.452806	0.083559
hsa_circ_	-0.40122	0.083564
hsa_circ_	-0.3911	0.083571
hsa_circ_	-0.46175	0.083576
hsa_circ_	0.713873	0.083583
hsa_circ_	0.376919	0.083588
hsa_circ_	0.553222	0.083592
hsa_circ_	-0.26585	0.083609
hsa_circ_	-0.45143	0.083609
hsa_circ_	-0.46207	0.08361
hsa_circ_	0.333886	0.083614
hsa_circ_	-0.52029	0.083622
hsa_circ_	0.592811	0.083622
hsa_circ_	-0.30803	0.083623
hsa_circ_	-0.75648	0.083625
hsa_circ_	0.294579	0.083628
hsa_circ_	-0.71666	0.083629
hsa_circ_	-0.54533	0.083634
hsa_circ_	-0.44676	0.083637
hsa_circ_	0.531974	0.083652
hsa_circ_	-0.37705	0.083653
hsa_circ_	-0.32226	0.083658
hsa_circ_	-0.62879	0.08366

hsa_circ_	-0.25852	0.083663
hsa_circ_	1.158194	0.083665
hsa_circ_	0.279238	0.08367
hsa_circ_	0.390228	0.083672
hsa_circ_	-0.91779	0.083684
hsa_circ_	-0.36118	0.083707
hsa_circ_	-0.44782	0.083711
hsa_circ_	-0.35435	0.08372
hsa_circ_	-0.57922	0.083723
hsa_circ_	0.401371	0.083743
hsa_circ_	1.044288	0.08377
hsa_circ_	0.96927	0.08377
hsa_circ_	0.538128	0.083772
hsa_circ_	0.603253	0.083789
hsa_circ_	0.375191	0.08379
hsa_circ_	-0.30014	0.083791
hsa_circ_	-0.6216	0.083795
hsa_circ_	0.488214	0.083825
hsa_circ_	0.588124	0.083825
hsa_circ_	-0.33399	0.083831
hsa_circ_	-0.41963	0.083831
hsa_circ_	-0.3467	0.083844
hsa_circ_	0.65883	0.08386
hsa_circ_	-0.30042	0.083864
hsa_circ_	-0.34281	0.083868
hsa_circ_	0.700255	0.083878
hsa_circ_	0.74489	0.083886
hsa_circ_	-0.99453	0.083888
hsa_circ_	0.944162	0.083897
hsa_circ_	-0.73308	0.083948
hsa_circ_	-1.0301	0.083954
hsa_circ_	-0.45912	0.083957
hsa_circ_	-0.32936	0.083966
hsa_circ_	-0.43858	0.083972
hsa_circ_	-0.94811	0.083977
hsa_circ_	-0.52172	0.083992
hsa_circ_	0.55738	0.083998
hsa_circ_	-0.60539	0.084007
hsa_circ_	-0.53115	0.084028
hsa_circ_	-0.57104	0.084029
hsa_circ_	-0.46112	0.08403
hsa_circ_	0.488009	0.084055
hsa_circ_	-0.73871	0.084081
hsa_circ_	-1.04976	0.084084
hsa_circ_	0.397228	0.084097
hsa_circ_	0.68217	0.08411
hsa_circ_	-0.39462	0.084114
hsa_circ_	-0.29712	0.084125
hsa_circ_	-0.68301	0.08414
hsa_circ_	0.727623	0.084145
hsa_circ_	0.392926	0.084153
hsa_circ_	-0.35409	0.084154
hsa_circ_	-0.48096	0.084159
hsa_circ_	0.617372	0.084183

hsa_circ_	-0.4394	0.084193
hsa_circ_	0.385147	0.084197
hsa_circ_	0.412732	0.0842
hsa_circ_	-0.29355	0.084203
hsa_circ_	0.894236	0.084203
hsa_circ_	-0.47909	0.084206
hsa_circ_	-0.57516	0.084221
hsa_circ_	0.585272	0.084221
hsa_circ_	0.39496	0.084236
hsa_circ_	-0.35783	0.084239
hsa_circ_	0.346674	0.084239
hsa_circ_	0.28873	0.084258
hsa_circ_	0.407486	0.084266
hsa_circ_	0.366229	0.084296
hsa_circ_	-0.31365	0.084314
hsa_circ_	-0.46808	0.084318
hsa_circ_	-0.50324	0.08432
hsa_circ_	-0.38286	0.084321
hsa_circ_	-0.56354	0.084322
hsa_circ_	0.505539	0.084331
hsa_circ_	0.314689	0.084342
hsa_circ_	0.453565	0.084357
hsa_circ_	0.722812	0.084359
hsa_circ_	-0.67971	0.084362
hsa_circ_	0.363508	0.084366
hsa_circ_	0.691827	0.084366
hsa_circ_	-0.25712	0.084369
hsa_circ_	-0.38773	0.084376
hsa_circ_	0.297101	0.08438
hsa_circ_	0.292658	0.084394
hsa_circ_	0.61167	0.084399
hsa_circ_	-0.33438	0.084405
hsa_circ_	0.412173	0.084405
hsa_circ_	-0.65632	0.084412
hsa_circ_	-0.38067	0.084415
hsa_circ_	-0.42501	0.084422
hsa_circ_	-0.3433	0.084425
hsa_circ_	-0.29312	0.084437
hsa_circ_	-0.66993	0.084472
hsa_circ_	0.262867	0.084487
hsa_circ_	-0.43515	0.084493
hsa_circ_	-0.6883	0.084497
hsa_circ_	-0.63355	0.084505
hsa_circ_	-0.36502	0.084508
hsa_circ_	-0.31534	0.08451
hsa_circ_	0.578546	0.084545
hsa_circ_	-0.36701	0.084546
hsa_circ_	0.288336	0.084558
hsa_circ_	-0.28478	0.084582
hsa_circ_	-0.30818	0.084586
hsa_circ_	0.816059	0.084589
hsa_circ_	-0.37407	0.084594
hsa_circ_	-0.66199	0.084625
hsa_circ_	-0.36765	0.084627

hsa_circ_	0.444455	0.084642
hsa_circ_	1.775263	0.084642
hsa_circ_	-0.64444	0.084667
hsa_circ_	0.381735	0.08467
hsa_circ_	-0.42715	0.084682
hsa_circ_	-0.65071	0.084683
hsa_circ_	0.48277	0.084692
hsa_circ_	0.960149	0.084719
hsa_circ_	-0.53729	0.084721
hsa_circ_	0.554481	0.084726
hsa_circ_	-0.30526	0.08473
hsa_circ_	-0.39952	0.084731
hsa_circ_	0.481871	0.084744
hsa_circ_	-0.68044	0.084745
hsa_circ_	-0.2736	0.084762
hsa_circ_	-0.25222	0.084773
hsa_circ_	0.484171	0.084801
hsa_circ_	-0.49532	0.084803
hsa_circ_	0.280398	0.084818
hsa_circ_	1.707665	0.084819
hsa_circ_	0.405543	0.08483
hsa_circ_	0.325476	0.084838
hsa_circ_	-0.57706	0.08484
hsa_circ_	-0.7391	0.084848
hsa_circ_	-0.48235	0.084849
hsa_circ_	-0.59926	0.084852
hsa_circ_	0.394155	0.084854
hsa_circ_	-0.4455	0.084862
hsa_circ_	-0.29925	0.084869
hsa_circ_	-0.44038	0.084879
hsa_circ_	-0.33199	0.084882
hsa_circ_	-0.25491	0.084884
hsa_circ_	0.356689	0.084888
hsa_circ_	-0.79136	0.084893
hsa_circ_	0.2841	0.084902
hsa_circ_	0.348144	0.084911
hsa_circ_	-0.27782	0.084914
hsa_circ_	-0.47955	0.084914
hsa_circ_	-0.42185	0.084924
hsa_circ_	-0.32468	0.084939
hsa_circ_	-0.39389	0.08494
hsa_circ_	-0.30759	0.084944
hsa_circ_	-0.24996	0.084956
hsa_circ_	-0.29086	0.084964
hsa_circ_	-0.38227	0.084971
hsa_circ_	-0.49764	0.08498
hsa_circ_	0.343308	0.084992
hsa_circ_	0.446805	0.084998
hsa_circ_	0.661708	0.085002
hsa_circ_	0.255216	0.085018
hsa_circ_	-0.31399	0.085023
hsa_circ_	-0.60307	0.085026
hsa_circ_	-0.31349	0.085032
hsa_circ_	0.597121	0.085035

hsa_circ_	-0.45324	0.085039
hsa_circ_	-0.38913	0.085044
hsa_circ_	0.523803	0.085045
hsa_circ_	-0.42853	0.085061
hsa_circ_	-0.29169	0.085067
hsa_circ_	0.444187	0.085069
hsa_circ_	0.387361	0.085075
hsa_circ_	-0.41171	0.08508
hsa_circ_	0.642622	0.085092
hsa_circ_	-0.46222	0.085095
hsa_circ_	-0.42022	0.085097
hsa_circ_	0.420233	0.085101
hsa_circ_	0.560316	0.085113
hsa_circ_	-0.24824	0.085114
hsa_circ_	0.37196	0.085116
hsa_circ_	-0.29022	0.085124
hsa_circ_	-0.35471	0.085126
hsa_circ_	-0.37172	0.085133
hsa_circ_	-0.35132	0.085146
hsa_circ_	0.403381	0.085164
hsa_circ_	0.728715	0.085165
hsa_circ_	0.564497	0.085175
hsa_circ_	-0.6245	0.085176
hsa_circ_	0.357177	0.085211
hsa_circ_	0.292512	0.085219
hsa_circ_	-0.48886	0.085222
hsa_circ_	1.023304	0.085227
hsa_circ_	0.522369	0.085238
hsa_circ_	0.619115	0.085256
hsa_circ_	-0.40051	0.085259
hsa_circ_	-0.33549	0.08526
hsa_circ_	-0.60118	0.085265
hsa_circ_	0.387256	0.085265
hsa_circ_	0.275746	0.085266
hsa_circ_	0.47641	0.085274
hsa_circ_	-0.39662	0.085278
hsa_circ_	-0.81125	0.08528
hsa_circ_	-0.38791	0.085287
hsa_circ_	2.081109	0.085303
hsa_circ_	0.388354	0.085313
hsa_circ_	0.342881	0.085314
hsa_circ_	-0.57115	0.085316
hsa_circ_	-0.4797	0.085319
hsa_circ_	0.523931	0.085321
hsa_circ_	0.317753	0.085324
hsa_circ_	-0.36776	0.085329
hsa_circ_	-0.37692	0.085336
hsa_circ_	-0.34439	0.085345
hsa_circ_	1.03678	0.08536
hsa_circ_	-0.7176	0.085364
hsa_circ_	-0.67659	0.085366
hsa_circ_	0.42453	0.085367
hsa_circ_	-0.45641	0.085373
hsa_circ_	-0.89734	0.085384

hsa_circ_	0.298898	0.085393
hsa_circ_	-0.29014	0.085397
hsa_circ_	0.368283	0.085407
hsa_circ_	-0.48951	0.085412
hsa_circ_	-0.28709	0.085415
hsa_circ_	-0.38331	0.08542
hsa_circ_	-0.54921	0.085423
hsa_circ_	0.495425	0.085427
hsa_circ_	-0.31599	0.085461
hsa_circ_	-0.4232	0.08547
hsa_circ_	-0.32367	0.085477
hsa_circ_	-0.2992	0.085486
hsa_circ_	0.419764	0.085492
hsa_circ_	0.346801	0.085506
hsa_circ_	-0.37503	0.085513
hsa_circ_	-0.29998	0.085513
hsa_circ_	-0.46496	0.08552
hsa_circ_	-0.30095	0.085525
hsa_circ_	-0.61356	0.085525
hsa_circ_	-0.51203	0.085551
hsa_circ_	0.262059	0.085552
hsa_circ_	-0.4325	0.085556
hsa_circ_	-0.91349	0.085557
hsa_circ_	0.272816	0.085568
hsa_circ_	0.292183	0.08557
hsa_circ_	0.380307	0.085574
hsa_circ_	-0.31075	0.08559
hsa_circ_	-0.54527	0.085593
hsa_circ_	0.550834	0.085603
hsa_circ_	-0.31797	0.085604
hsa_circ_	-0.61554	0.085607
hsa_circ_	-0.32752	0.08563
hsa_circ_	-0.38368	0.085632
hsa_circ_	-0.39359	0.085646
hsa_circ_	-0.48654	0.085658
hsa_circ_	0.472847	0.085668
hsa_circ_	0.399453	0.085686
hsa_circ_	0.495505	0.085689
hsa_circ_	0.319646	0.085692
hsa_circ_	-0.27984	0.085701
hsa_circ_	-0.38275	0.085707
hsa_circ_	0.442576	0.08571
hsa_circ_	-0.65368	0.085721
hsa_circ_	-0.56264	0.085721
hsa_circ_	0.462069	0.085724
hsa_circ_	0.571848	0.085729
hsa_circ_	1.31659	0.08573
hsa_circ_	-0.51811	0.08575
hsa_circ_	0.654691	0.085753
hsa_circ_	-0.62498	0.085755
hsa_circ_	0.299455	0.085758
hsa_circ_	0.485965	0.085766
hsa_circ_	-0.953	0.085789
hsa_circ_	0.418803	0.085795

hsa_circ_	-0.47823	0.0858
hsa_circ_	0.459598	0.085802
hsa_circ_	0.619855	0.085806
hsa_circ_	-0.44252	0.085807
hsa_circ_	0.278193	0.085808
hsa_circ_	0.35882	0.085809
hsa_circ_	0.342977	0.085812
hsa_circ_	-1.09572	0.085815
hsa_circ_	0.756579	0.085842
hsa_circ_	-0.67908	0.085844
hsa_circ_	0.336723	0.085845
hsa_circ_	-0.38561	0.085845
hsa_circ_	0.278754	0.085846
hsa_circ_	0.803173	0.085847
hsa_circ_	0.475291	0.085855
hsa_circ_	0.434183	0.085869
hsa_circ_	-0.64404	0.085876
hsa_circ_	0.435744	0.085889
hsa_circ_	0.87961	0.08589
hsa_circ_	-0.97517	0.085892
hsa_circ_	-0.26886	0.085895
hsa_circ_	-0.27554	0.085911
hsa_circ_	0.780212	0.085911
hsa_circ_	-0.41048	0.085916
hsa_circ_	-0.50797	0.085924
hsa_circ_	-0.47887	0.085926
hsa_circ_	-0.28722	0.085932
hsa_circ_	0.350222	0.085943
hsa_circ_	0.291555	0.085946
hsa_circ_	-0.71809	0.085957
hsa_circ_	-0.26726	0.08596
hsa_circ_	-0.30691	0.085978
hsa_circ_	-0.29902	0.085983
hsa_circ_	0.759963	0.085987
hsa_circ_	-0.51316	0.08599
hsa_circ_	0.275208	0.085994
hsa_circ_	-0.28757	0.085995
hsa_circ_	1.247974	0.086002
hsa_circ_	-0.9434	0.086003
hsa_circ_	0.342201	0.086018
hsa_circ_	0.64501	0.08602
hsa_circ_	0.361666	0.08603
hsa_circ_	-0.40036	0.086031
hsa_circ_	0.375833	0.086044
hsa_circ_	-0.41937	0.086049
hsa_circ_	0.414932	0.086055
hsa_circ_	0.687033	0.086058
hsa_circ_	0.344655	0.08606
hsa_circ_	-0.42361	0.08606
hsa_circ_	-0.31541	0.086064
hsa_circ_	0.382092	0.08607
hsa_circ_	0.610341	0.08607
hsa_circ_	0.290245	0.086073
hsa_circ_	-0.50092	0.086075

hsa_circ_	-0.71862	0.086076
hsa_circ_	0.63099	0.086076
hsa_circ_	-0.32553	0.086078
hsa_circ_	-0.26236	0.086084
hsa_circ_	-0.3006	0.086104
hsa_circ_	-0.28855	0.086106
hsa_circ_	0.369598	0.086114
hsa_circ_	-0.50175	0.086123
hsa_circ_	-0.89047	0.086139
hsa_circ_	-0.29337	0.086142
hsa_circ_	0.350906	0.086142
hsa_circ_	0.430519	0.086147
hsa_circ_	-1.09763	0.086158
hsa_circ_	0.337767	0.086164
hsa_circ_	0.278028	0.086171
hsa_circ_	0.392752	0.086174
hsa_circ_	-0.25548	0.086175
hsa_circ_	0.393987	0.086184
hsa_circ_	-0.66824	0.086184
hsa_circ_	0.313694	0.086186
hsa_circ_	-0.32774	0.086186
hsa_circ_	0.419207	0.086187
hsa_circ_	0.390978	0.086188
hsa_circ_	-0.28905	0.086195
hsa_circ_	0.367296	0.086195
hsa_circ_	-0.34211	0.086206
hsa_circ_	-0.31348	0.086226
hsa_circ_	-0.59982	0.086232
hsa_circ_	-0.435	0.086233
hsa_circ_	0.539986	0.086238
hsa_circ_	0.476956	0.086238
hsa_circ_	-0.30241	0.086245
hsa_circ_	0.327321	0.08625
hsa_circ_	-0.39318	0.086251
hsa_circ_	-0.78784	0.086281
hsa_circ_	-0.70331	0.086289
hsa_circ_	0.354396	0.086289
hsa_circ_	-0.32073	0.086295
hsa_circ_	-0.34805	0.086303
hsa_circ_	0.272583	0.086308
hsa_circ_	-1.26851	0.086309
hsa_circ_	-0.50094	0.086313
hsa_circ_	-0.47551	0.086317
hsa_circ_	-0.42188	0.086325
hsa_circ_	0.567469	0.086328
hsa_circ_	0.248903	0.086348
hsa_circ_	-0.56769	0.086361
hsa_circ_	-0.34333	0.086367
hsa_circ_	0.474213	0.086384
hsa_circ_	-0.55811	0.086408
hsa_circ_	0.755382	0.08641
hsa_circ_	0.49052	0.086411
hsa_circ_	-0.28476	0.086415
hsa_circ_	0.362465	0.086424

hsa_circ_	-0.36417	0.086427
hsa_circ_	-0.30179	0.086427
hsa_circ_	0.290996	0.086429
hsa_circ_	0.271871	0.08644
hsa_circ_	-0.45596	0.086442
hsa_circ_	-0.42259	0.086454
hsa_circ_	-0.27221	0.086461
hsa_circ_	-0.27763	0.086473
hsa_circ_	0.46483	0.086473
hsa_circ_	0.859991	0.086474
hsa_circ_	-0.63514	0.086478
hsa_circ_	-0.26005	0.086479
hsa_circ_	-0.28073	0.086484
hsa_circ_	-0.3579	0.086487
hsa_circ_	-0.38618	0.086497
hsa_circ_	0.68189	0.086503
hsa_circ_	0.484238	0.086511
hsa_circ_	-0.85513	0.08652
hsa_circ_	-0.58351	0.086522
hsa_circ_	-0.47379	0.086526
hsa_circ_	0.521607	0.086534
hsa_circ_	0.466756	0.08654
hsa_circ_	-0.55272	0.08655
hsa_circ_	1.13011	0.086551
hsa_circ_	0.673191	0.086558
hsa_circ_	0.937825	0.086559
hsa_circ_	-1.42077	0.08656
hsa_circ_	0.917076	0.086563
hsa_circ_	0.315014	0.08658
hsa_circ_	-0.63677	0.086582
hsa_circ_	0.521995	0.086596
hsa_circ_	-0.26865	0.086598
hsa_circ_	0.545308	0.086599
hsa_circ_	-0.3494	0.086623
hsa_circ_	0.601375	0.086625
hsa_circ_	-0.42626	0.086626
hsa_circ_	-0.51202	0.086627
hsa_circ_	0.759928	0.086633
hsa_circ_	0.529188	0.086633
hsa_circ_	-0.56588	0.086655
hsa_circ_	0.335822	0.086658
hsa_circ_	1.154552	0.086667
hsa_circ_	-0.28776	0.086684
hsa_circ_	0.316326	0.086686
hsa_circ_	0.514688	0.086687
hsa_circ_	-0.26197	0.086692
hsa_circ_	0.648331	0.086693
hsa_circ_	0.707712	0.086693
hsa_circ_	-0.27064	0.086703
hsa_circ_	-0.32357	0.086715
hsa_circ_	-0.65158	0.086716
hsa_circ_	0.347463	0.086719
hsa_circ_	-0.75109	0.086724
hsa_circ_	0.244014	0.086745

hsa_circ_	-0.73507	0.086746
hsa_circ_	0.389486	0.086752
hsa_circ_	-0.84876	0.086753
hsa_circ_	0.561771	0.086754
hsa_circ_	0.459929	0.086773
hsa_circ_	0.832543	0.086785
hsa_circ_	-2.37333	0.086787
hsa_circ_	0.821347	0.086801
hsa_circ_	-0.42804	0.086806
hsa_circ_	0.846857	0.086807
hsa_circ_	-0.42164	0.086821
hsa_circ_	-0.2995	0.08683
hsa_circ_	0.557898	0.086839
hsa_circ_	0.774523	0.086846
hsa_circ_	-0.45034	0.086846
hsa_circ_	0.452331	0.086862
hsa_circ_	-0.41473	0.086867
hsa_circ_	0.286421	0.086868
hsa_circ_	0.956746	0.086876
hsa_circ_	0.331209	0.086899
hsa_circ_	0.438981	0.086899
hsa_circ_	0.437451	0.0869
hsa_circ_	0.615964	0.0869
hsa_circ_	1.258875	0.086901
hsa_circ_	0.499278	0.086904
hsa_circ_	-0.48734	0.086914
hsa_circ_	-0.29717	0.086922
hsa_circ_	0.35692	0.086931
hsa_circ_	0.670504	0.086932
hsa_circ_	-0.64992	0.086935
hsa_circ_	0.313175	0.086946
hsa_circ_	-0.25201	0.08696
hsa_circ_	-0.26101	0.086961
hsa_circ_	-0.35483	0.086976
hsa_circ_	-0.28366	0.086979
hsa_circ_	0.315534	0.086982
hsa_circ_	-0.36096	0.086995
hsa_circ_	-0.25614	0.086995
hsa_circ_	0.377319	0.087004
hsa_circ_	0.299374	0.087027
hsa_circ_	-0.30244	0.087029
hsa_circ_	0.61989	0.087042
hsa_circ_	0.432147	0.087047
hsa_circ_	0.411176	0.087063
hsa_circ_	0.325653	0.087071
hsa_circ_	0.541624	0.087071
hsa_circ_	0.72983	0.087071
hsa_circ_	0.329506	0.087072
hsa_circ_	-1.01409	0.087076
hsa_circ_	0.334501	0.087086
hsa_circ_	1.112936	0.087103
hsa_circ_	-0.67152	0.087103
hsa_circ_	0.461693	0.087109
hsa_circ_	-0.30053	0.087114

hsa_circ_	0.541103	0.087127
hsa_circ_	-0.2637	0.087133
hsa_circ_	0.39273	0.087139
hsa_circ_	0.706867	0.087142
hsa_circ_	-0.3942	0.087157
hsa_circ_	0.443065	0.087169
hsa_circ_	-0.28168	0.087171
hsa_circ_	-0.25933	0.087172
hsa_circ_	-0.39459	0.087172
hsa_circ_	0.293181	0.087176
hsa_circ_	-0.57637	0.08718
hsa_circ_	-0.84968	0.08718
hsa_circ_	0.522658	0.087184
hsa_circ_	-0.36529	0.087185
hsa_circ_	-0.81996	0.087191
hsa_circ_	0.276646	0.087197
hsa_circ_	-0.29146	0.087206
hsa_circ_	0.644694	0.087208
hsa_circ_	0.759197	0.08721
hsa_circ_	0.310264	0.087217
hsa_circ_	0.971224	0.08722
hsa_circ_	0.646249	0.087224
hsa_circ_	-0.68181	0.087235
hsa_circ_	-0.43815	0.087238
hsa_circ_	0.315982	0.087247
hsa_circ_	0.368643	0.08725
hsa_circ_	-0.49735	0.087267
hsa_circ_	1.051439	0.087274
hsa_circ_	0.49026	0.087277
hsa_circ_	0.371406	0.087278
hsa_circ_	-0.80398	0.087283
hsa_circ_	0.427656	0.08729
hsa_circ_	0.317694	0.087292
hsa_circ_	0.255056	0.087295
hsa_circ_	0.471647	0.087295
hsa_circ_	0.415351	0.087298
hsa_circ_	-0.29551	0.087302
hsa_circ_	-0.29827	0.087303
hsa_circ_	-0.38416	0.08731
hsa_circ_	-0.31009	0.087321
hsa_circ_	-0.60848	0.087324
hsa_circ_	0.282405	0.087332
hsa_circ_	-0.46196	0.087353
hsa_circ_	0.699051	0.087355
hsa_circ_	-0.35949	0.087368
hsa_circ_	0.387867	0.087383
hsa_circ_	0.431249	0.087387
hsa_circ_	-0.28875	0.087396
hsa_circ_	-0.28529	0.087397
hsa_circ_	0.95579	0.087403
hsa_circ_	0.284094	0.087442
hsa_circ_	0.388405	0.08746
hsa_circ_	0.287206	0.08746
hsa_circ_	0.323617	0.087484

hsa_circ_	0.580596	0.087486
hsa_circ_	0.284654	0.087488
hsa_circ_	-0.33369	0.087499
hsa_circ_	-0.50405	0.087499
hsa_circ_	0.422963	0.087516
hsa_circ_	-0.39437	0.087516
hsa_circ_	-0.25668	0.087528
hsa_circ_	-0.33125	0.087547
hsa_circ_	-0.41291	0.087564
hsa_circ_	-0.30815	0.087567
hsa_circ_	-0.50768	0.087573
hsa_circ_	-0.57301	0.087577
hsa_circ_	-0.46912	0.087584
hsa_circ_	-0.86049	0.087594
hsa_circ_	1.197654	0.087613
hsa_circ_	-0.3467	0.087622
hsa_circ_	0.418486	0.087629
hsa_circ_	-0.28734	0.087653
hsa_circ_	-0.4133	0.087664
hsa_circ_	0.429203	0.08768
hsa_circ_	0.604163	0.087682
hsa_circ_	0.41287	0.087687
hsa_circ_	0.599126	0.087687
hsa_circ_	-0.46119	0.087693
hsa_circ_	-0.30383	0.087707
hsa_circ_	-0.31586	0.087711
hsa_circ_	0.519086	0.08772
hsa_circ_	-0.33195	0.087726
hsa_circ_	-0.31876	0.087727
hsa_circ_	0.359648	0.087746
hsa_circ_	-0.32408	0.087749
hsa_circ_	0.406601	0.087749
hsa_circ_	0.477268	0.087751
hsa_circ_	-0.66728	0.087761
hsa_circ_	0.382784	0.087788
hsa_circ_	-0.30871	0.08779
hsa_circ_	0.261256	0.087791
hsa_circ_	-0.26483	0.087794
hsa_circ_	0.307492	0.087807
hsa_circ_	0.314246	0.087808
hsa_circ_	-0.28413	0.087813
hsa_circ_	0.334455	0.087822
hsa_circ_	1.154433	0.087829
hsa_circ_	0.257149	0.087833
hsa_circ_	-0.45482	0.087853
hsa_circ_	0.371067	0.087856
hsa_circ_	0.296718	0.087866
hsa_circ_	-0.40591	0.087866
hsa_circ_	-0.37254	0.087869
hsa_circ_	0.912274	0.087877
hsa_circ_	-0.29315	0.08788
hsa_circ_	0.35041	0.087881
hsa_circ_	-1.35147	0.087904
hsa_circ_	-0.27561	0.087912

hsa_circ_	-0.95922	0.087912
hsa_circ_	-0.35136	0.087914
hsa_circ_	0.541499	0.087916
hsa_circ_	0.648727	0.087918
hsa_circ_	-0.7546	0.087918
hsa_circ_	-0.80588	0.087922
hsa_circ_	-0.39912	0.087923
hsa_circ_	-0.58474	0.087933
hsa_circ_	0.301558	0.087951
hsa_circ_	0.319999	0.087954
hsa_circ_	1.059815	0.087963
hsa_circ_	-0.96752	0.087972
hsa_circ_	-1.43007	0.087973
hsa_circ_	0.823749	0.087986
hsa_circ_	0.965617	0.087986
hsa_circ_	-0.38546	0.087993
hsa_circ_	-0.4412	0.087995
hsa_circ_	0.462293	0.088011
hsa_circ_	0.632541	0.088018
hsa_circ_	0.286602	0.088024
hsa_circ_	-0.33617	0.088042
hsa_circ_	-0.47685	0.088053
hsa_circ_	0.567686	0.088056
hsa_circ_	0.812337	0.088057
hsa_circ_	-0.32302	0.088058
hsa_circ_	0.565917	0.088067
hsa_circ_	-0.44789	0.088068
hsa_circ_	0.397687	0.08807
hsa_circ_	-0.31354	0.088071
hsa_circ_	0.893941	0.088079
hsa_circ_	1.09502	0.088086
hsa_circ_	0.251868	0.088103
hsa_circ_	-0.41428	0.08811
hsa_circ_	-0.46388	0.088121
hsa_circ_	-0.26951	0.088121
hsa_circ_	-0.71958	0.088138
hsa_circ_	-0.36789	0.088146
hsa_circ_	-0.85636	0.08815
hsa_circ_	0.40456	0.088152
hsa_circ_	0.818897	0.088161
hsa_circ_	-0.33257	0.088162
hsa_circ_	-0.46388	0.088199
hsa_circ_	0.474535	0.0882
hsa_circ_	0.250666	0.088203
hsa_circ_	-0.54805	0.088207
hsa_circ_	-0.44707	0.088216
hsa_circ_	0.324421	0.088229
hsa_circ_	0.435465	0.088232
hsa_circ_	0.446571	0.088239
hsa_circ_	0.397732	0.088266
hsa_circ_	0.402605	0.088267
hsa_circ_	0.501746	0.088267
hsa_circ_	1.07341	0.088268
hsa_circ_	0.270154	0.088275

hsa_circ_	0.249267	0.088286
hsa_circ_	0.298796	0.088294
hsa_circ_	-0.25853	0.088297
hsa_circ_	0.263654	0.088299
hsa_circ_	-0.53659	0.088321
hsa_circ_	0.607206	0.088331
hsa_circ_	-0.2994	0.088346
hsa_circ_	-0.34604	0.088346
hsa_circ_	0.260771	0.088366
hsa_circ_	0.330471	0.088372
hsa_circ_	0.254201	0.088373
hsa_circ_	-0.41118	0.088374
hsa_circ_	-0.77234	0.088375
hsa_circ_	-0.96649	0.088388
hsa_circ_	0.968259	0.088393
hsa_circ_	-2.69396	0.088394
hsa_circ_	0.497753	0.088406
hsa_circ_	-0.38557	0.08841
hsa_circ_	0.376454	0.088413
hsa_circ_	-0.35231	0.088432
hsa_circ_	0.573664	0.088432
hsa_circ_	0.344078	0.088447
hsa_circ_	1.754007	0.088449
hsa_circ_	-0.38526	0.088453
hsa_circ_	-0.30751	0.088466
hsa_circ_	0.350348	0.088486
hsa_circ_	0.376418	0.088486
hsa_circ_	-0.26737	0.088486
hsa_circ_	0.520031	0.088502
hsa_circ_	-0.46679	0.088511
hsa_circ_	-0.27716	0.088512
hsa_circ_	-3.29545	0.088528
hsa_circ_	-0.26898	0.08853
hsa_circ_	-0.26704	0.088538
hsa_circ_	-0.31676	0.088539
hsa_circ_	0.501162	0.088541
hsa_circ_	0.772352	0.088546
hsa_circ_	1.272182	0.088551
hsa_circ_	0.262701	0.088553
hsa_circ_	-0.45752	0.088556
hsa_circ_	-0.32195	0.088587
hsa_circ_	0.405812	0.088602
hsa_circ_	0.953545	0.088603
hsa_circ_	-0.29004	0.088614
hsa_circ_	-0.27691	0.08862
hsa_circ_	0.323151	0.088621
hsa_circ_	-0.4354	0.088622
hsa_circ_	0.345062	0.088634
hsa_circ_	-0.47319	0.088634
hsa_circ_	-0.5735	0.088651
hsa_circ_	-0.5092	0.088655
hsa_circ_	0.318105	0.088656
hsa_circ_	-0.59472	0.088656
hsa_circ_	-0.66981	0.088669

hsa_circ_	0.673043	0.088673
hsa_circ_	-0.45195	0.088675
hsa_circ_	-0.30329	0.088676
hsa_circ_	0.316889	0.088681
hsa_circ_	-0.27589	0.088681
hsa_circ_	-0.44454	0.088689
hsa_circ_	-1.3213	0.08869
hsa_circ_	0.364856	0.088699
hsa_circ_	0.278223	0.088704
hsa_circ_	-1.05214	0.088705
hsa_circ_	0.399247	0.088711
hsa_circ_	0.328577	0.088714
hsa_circ_	-1.26751	0.08873
hsa_circ_	-0.52356	0.088738
hsa_circ_	-0.40989	0.088742
hsa_circ_	-0.68487	0.088758
hsa_circ_	0.418388	0.088763
hsa_circ_	-0.77403	0.088772
hsa_circ_	0.360134	0.088784
hsa_circ_	-0.34322	0.088795
hsa_circ_	-0.30711	0.08881
hsa_circ_	2.032784	0.088821
hsa_circ_	0.478171	0.088824
hsa_circ_	-0.52417	0.088838
hsa_circ_	0.369364	0.088843
hsa_circ_	-0.56276	0.088849
hsa_circ_	-0.32768	0.08885
hsa_circ_	-0.33356	0.088865
hsa_circ_	-0.28876	0.088867
hsa_circ_	0.933507	0.088875
hsa_circ_	-0.42177	0.088883
hsa_circ_	-0.51001	0.088894
hsa_circ_	0.705694	0.088904
hsa_circ_	-0.46892	0.088905
hsa_circ_	0.431483	0.088905
hsa_circ_	0.388891	0.088907
hsa_circ_	-0.47581	0.088909
hsa_circ_	-0.4147	0.088911
hsa_circ_	0.81899	0.088929
hsa_circ_	-0.5566	0.088931
hsa_circ_	-0.31449	0.088938
hsa_circ_	-0.44556	0.088939
hsa_circ_	0.590458	0.088942
hsa_circ_	0.352518	0.088954
hsa_circ_	0.416189	0.088961
hsa_circ_	0.827383	0.088975
hsa_circ_	-0.43397	0.088983
hsa_circ_	-0.31804	0.088985
hsa_circ_	0.771315	0.088986
hsa_circ_	0.427814	0.088987
hsa_circ_	-0.26762	0.088989
hsa_circ_	0.393716	0.089011
hsa_circ_	-0.36138	0.089013
hsa_circ_	0.4444	0.089018

hsa_circ_	0.250077	0.089024
hsa_circ_	-0.63071	0.089025
hsa_circ_	-0.71542	0.08903
hsa_circ_	-0.67918	0.089032
hsa_circ_	-0.26279	0.089039
hsa_circ_	-0.60538	0.08904
hsa_circ_	0.982882	0.089058
hsa_circ_	0.357688	0.089068
hsa_circ_	-0.27935	0.089079
hsa_circ_	0.535713	0.089091
hsa_circ_	0.36303	0.089097
hsa_circ_	-0.29664	0.089098
hsa_circ_	-0.42407	0.089111
hsa_circ_	-0.78364	0.089151
hsa_circ_	-0.63495	0.089152
hsa_circ_	0.271388	0.089156
hsa_circ_	0.354401	0.089168
hsa_circ_	0.39047	0.089171
hsa_circ_	-0.4134	0.089173
hsa_circ_	0.423479	0.089188
hsa_circ_	0.440384	0.089197
hsa_circ_	-0.3734	0.089201
hsa_circ_	1.296362	0.089203
hsa_circ_	-0.51246	0.089204
hsa_circ_	0.309083	0.089214
hsa_circ_	-0.26967	0.089221
hsa_circ_	-0.46488	0.089248
hsa_circ_	-0.38559	0.089257
hsa_circ_	0.337666	0.089261
hsa_circ_	0.679375	0.089265
hsa_circ_	0.299144	0.089276
hsa_circ_	0.250181	0.089279
hsa_circ_	-0.62352	0.089281
hsa_circ_	-0.38649	0.089288
hsa_circ_	-0.24977	0.089289
hsa_circ_	-0.58463	0.089297
hsa_circ_	0.389896	0.089299
hsa_circ_	-0.30183	0.089302
hsa_circ_	-0.7133	0.089306
hsa_circ_	-0.288	0.089308
hsa_circ_	0.315334	0.089318
hsa_circ_	-0.35656	0.089337
hsa_circ_	0.435106	0.089342
hsa_circ_	-0.77449	0.089348
hsa_circ_	0.373968	0.089354
hsa_circ_	-0.39626	0.089356
hsa_circ_	-0.90358	0.089366
hsa_circ_	-0.5139	0.089371
hsa_circ_	0.373335	0.08938
hsa_circ_	-0.66048	0.089383
hsa_circ_	-0.29559	0.089392
hsa_circ_	-0.3396	0.089393
hsa_circ_	-0.38448	0.089394
hsa_circ_	-0.29762	0.0894

hsa_circ_	-0.28021	0.089405
hsa_circ_	-0.38315	0.089421
hsa_circ_	0.585199	0.089423
hsa_circ_	0.336192	0.089456
hsa_circ_	-0.35384	0.089462
hsa_circ_	-0.2715	0.08947
hsa_circ_	0.616175	0.089487
hsa_circ_	-0.70528	0.089489
hsa_circ_	0.308049	0.08949
hsa_circ_	-1.40758	0.089501
hsa_circ_	-0.38289	0.089517
hsa_circ_	-1.24337	0.089527
hsa_circ_	0.684612	0.089529
hsa_circ_	0.415098	0.089534
hsa_circ_	0.445186	0.08954
hsa_circ_	-0.43278	0.089548
hsa_circ_	-0.35864	0.089551
hsa_circ_	-0.4571	0.089561
hsa_circ_	-0.3702	0.089564
hsa_circ_	-0.29478	0.08958
hsa_circ_	-0.93006	0.089588
hsa_circ_	0.282057	0.089593
hsa_circ_	0.810624	0.089597
hsa_circ_	-0.27082	0.089606
hsa_circ_	-0.24894	0.089609
hsa_circ_	0.631018	0.089623
hsa_circ_	0.331126	0.089629
hsa_circ_	-0.36529	0.08963
hsa_circ_	-0.55054	0.08963
hsa_circ_	-0.39286	0.089633
hsa_circ_	-0.63625	0.089633
hsa_circ_	0.343262	0.089639
hsa_circ_	0.485919	0.089642
hsa_circ_	0.330114	0.089655
hsa_circ_	0.412993	0.089658
hsa_circ_	-0.52906	0.089659
hsa_circ_	0.411032	0.089661
hsa_circ_	-0.34517	0.089666
hsa_circ_	-0.26383	0.089682
hsa_circ_	-0.3133	0.089686
hsa_circ_	-0.5423	0.089689
hsa_circ_	0.370011	0.089703
hsa_circ_	0.928874	0.089704
hsa_circ_	-0.41994	0.089718
hsa_circ_	0.452716	0.089719
hsa_circ_	-0.57351	0.089744
hsa_circ_	-0.26294	0.089748
hsa_circ_	1.532287	0.089751
hsa_circ_	0.38952	0.089755
hsa_circ_	-0.51335	0.089765
hsa_circ_	-0.65316	0.08977
hsa_circ_	-0.50281	0.089771
hsa_circ_	-0.49286	0.089772
hsa_circ_	0.597559	0.089781

hsa_circ_	0.384901	0.089788
hsa_circ_	-0.3057	0.089806
hsa_circ_	-0.41847	0.08981
hsa_circ_	-0.80036	0.089827
hsa_circ_	0.268132	0.089832
hsa_circ_	0.51387	0.089835
hsa_circ_	0.397636	0.089842
hsa_circ_	0.329209	0.089856
hsa_circ_	0.297907	0.089859
hsa_circ_	-0.29547	0.089866
hsa_circ_	-0.28322	0.089902
hsa_circ_	-0.57281	0.089903
hsa_circ_	0.261655	0.089903
hsa_circ_	0.337684	0.089918
hsa_circ_	-0.56099	0.089919
hsa_circ_	0.404933	0.089924
hsa_circ_	-0.47468	0.089934
hsa_circ_	0.296201	0.089937
hsa_circ_	1.003878	0.089937
hsa_circ_	0.410775	0.089938
hsa_circ_	-0.48317	0.089942
hsa_circ_	0.318651	0.089947
hsa_circ_	-0.32766	0.089949
hsa_circ_	0.37689	0.089984
hsa_circ_	-0.42716	0.089988
hsa_circ_	0.436781	0.09
hsa_circ_	0.439051	0.090018
hsa_circ_	0.320509	0.090021
hsa_circ_	-0.64694	0.090038
hsa_circ_	-0.32061	0.090039
hsa_circ_	-0.24785	0.090047
hsa_circ_	-0.74198	0.090076
hsa_circ_	-0.80187	0.090093
hsa_circ_	0.418336	0.090096
hsa_circ_	0.600628	0.090104
hsa_circ_	-0.25911	0.090105
hsa_circ_	0.346251	0.090106
hsa_circ_	0.430038	0.090118
hsa_circ_	-0.27375	0.090121
hsa_circ_	-0.28236	0.09013
hsa_circ_	-0.2947	0.090138
hsa_circ_	0.259713	0.090148
hsa_circ_	0.277107	0.090148
hsa_circ_	0.529582	0.090168
hsa_circ_	-0.27907	0.090176
hsa_circ_	0.669609	0.09018
hsa_circ_	-0.29348	0.090182
hsa_circ_	-0.27745	0.090187
hsa_circ_	0.758287	0.090194
hsa_circ_	-0.31917	0.090207
hsa_circ_	0.340476	0.090208
hsa_circ_	-0.43524	0.090217
hsa_circ_	-0.53977	0.090227
hsa_circ_	-0.71968	0.090242

hsa_circ_	-0.86816	0.090243
hsa_circ_	-0.27209	0.090244
hsa_circ_	0.755465	0.090256
hsa_circ_	0.251454	0.090258
hsa_circ_	-0.30778	0.090273
hsa_circ_	-0.4247	0.090279
hsa_circ_	-0.33177	0.090284
hsa_circ_	-0.33214	0.090295
hsa_circ_	0.358143	0.090304
hsa_circ_	-0.67188	0.090304
hsa_circ_	0.558291	0.090308
hsa_circ_	0.345046	0.090313
hsa_circ_	-0.34379	0.090316
hsa_circ_	-0.84314	0.090322
hsa_circ_	0.498105	0.090341
hsa_circ_	-0.37302	0.090346
hsa_circ_	0.425438	0.090351
hsa_circ_	0.305229	0.090356
hsa_circ_	-0.24665	0.09037
hsa_circ_	-0.30294	0.090389
hsa_circ_	0.385594	0.090389
hsa_circ_	0.412283	0.09039
hsa_circ_	-0.31234	0.090394
hsa_circ_	0.618681	0.090399
hsa_circ_	-0.33676	0.090412
hsa_circ_	0.5265	0.090422
hsa_circ_	-0.512	0.090425
hsa_circ_	-0.39399	0.090435
hsa_circ_	-0.3095	0.090436
hsa_circ_	-0.28682	0.090448
hsa_circ_	0.364055	0.090456
hsa_circ_	0.856931	0.090467
hsa_circ_	0.499519	0.090472
hsa_circ_	-1.16725	0.090477
hsa_circ_	-0.29316	0.09048
hsa_circ_	-0.76414	0.090481
hsa_circ_	-0.28203	0.090482
hsa_circ_	-0.34124	0.090486
hsa_circ_	-0.71628	0.09049
hsa_circ_	0.657456	0.090501
hsa_circ_	0.546574	0.090503
hsa_circ_	0.526664	0.090511
hsa_circ_	0.518075	0.090523
hsa_circ_	0.298547	0.09053
hsa_circ_	-0.52411	0.090532
hsa_circ_	0.262065	0.090545
hsa_circ_	0.406403	0.090551
hsa_circ_	0.467383	0.090555
hsa_circ_	-0.25737	0.090559
hsa_circ_	-0.49449	0.090571
hsa_circ_	-0.42101	0.09058
hsa_circ_	-0.27481	0.0906
hsa_circ_	-0.50637	0.090616
hsa_circ_	-0.3725	0.090617

hsa_circ_	-0.55572	0.090624
hsa_circ_	0.825344	0.090635
hsa_circ_	0.454169	0.090642
hsa_circ_	0.306621	0.090642
hsa_circ_	-0.28844	0.090652
hsa_circ_	-0.38423	0.090653
hsa_circ_	-0.26071	0.090659
hsa_circ_	-0.36701	0.09067
hsa_circ_	-0.4331	0.090674
hsa_circ_	0.45032	0.09068
hsa_circ_	0.590971	0.090688
hsa_circ_	-0.60684	0.090689
hsa_circ_	-0.33447	0.090702
hsa_circ_	-0.60893	0.09073
hsa_circ_	0.295776	0.090737
hsa_circ_	0.452429	0.090738
hsa_circ_	-0.37765	0.090745
hsa_circ_	-0.40857	0.090749
hsa_circ_	-0.94003	0.090755
hsa_circ_	-0.56671	0.09078
hsa_circ_	0.773417	0.090782
hsa_circ_	-0.33778	0.090789
hsa_circ_	0.433605	0.090791
hsa_circ_	0.693601	0.090802
hsa_circ_	-0.43979	0.090821
hsa_circ_	-0.3748	0.090848
hsa_circ_	1.643231	0.090859
hsa_circ_	0.444381	0.090862
hsa_circ_	-0.41917	0.090866
hsa_circ_	0.40059	0.09088
hsa_circ_	-0.46288	0.090881
hsa_circ_	0.633786	0.090891
hsa_circ_	0.769953	0.090897
hsa_circ_	0.317502	0.090897
hsa_circ_	0.495784	0.090907
hsa_circ_	0.326725	0.090914
hsa_circ_	0.448313	0.090924
hsa_circ_	-0.31748	0.090931
hsa_circ_	0.438374	0.090935
hsa_circ_	0.34724	0.090939
hsa_circ_	0.476928	0.090944
hsa_circ_	0.637662	0.090949
hsa_circ_	0.737779	0.090961
hsa_circ_	0.923604	0.090965
hsa_circ_	0.525564	0.09097
hsa_circ_	-0.42002	0.090982
hsa_circ_	-0.44641	0.090984
hsa_circ_	-0.76206	0.090992
hsa_circ_	0.770916	0.091008
hsa_circ_	-0.35895	0.091012
hsa_circ_	-0.29312	0.091015
hsa_circ_	0.398157	0.091024
hsa_circ_	-0.55041	0.091036
hsa_circ_	-0.35227	0.091037

hsa_circ_	0.590586	0.091042
hsa_circ_	0.520136	0.091044
hsa_circ_	-0.44259	0.091048
hsa_circ_	0.305082	0.091049
hsa_circ_	-0.3315	0.091049
hsa_circ_	0.340436	0.091055
hsa_circ_	-0.5642	0.091055
hsa_circ_	-0.32871	0.091061
hsa_circ_	-0.34869	0.091069
hsa_circ_	-0.29488	0.091073
hsa_circ_	0.50357	0.091082
hsa_circ_	-0.41141	0.091083
hsa_circ_	0.580011	0.091089
hsa_circ_	-0.32768	0.091093
hsa_circ_	-0.99422	0.091097
hsa_circ_	0.399711	0.091117
hsa_circ_	-0.24065	0.091121
hsa_circ_	0.580738	0.091128
hsa_circ_	-0.35598	0.091132
hsa_circ_	0.359903	0.091139
hsa_circ_	0.309637	0.091145
hsa_circ_	0.284722	0.091162
hsa_circ_	0.857422	0.091174
hsa_circ_	1.366698	0.091176
hsa_circ_	0.302999	0.091183
hsa_circ_	0.587851	0.091196
hsa_circ_	-0.42866	0.091205
hsa_circ_	0.358366	0.091209
hsa_circ_	0.585924	0.091215
hsa_circ_	0.387269	0.091216
hsa_circ_	-0.72254	0.091231
hsa_circ_	1.52339	0.09124
hsa_circ_	-0.91151	0.09124
hsa_circ_	-0.43216	0.091245
hsa_circ_	0.357983	0.091248
hsa_circ_	-0.5499	0.09126
hsa_circ_	-0.67904	0.091262
hsa_circ_	-0.30286	0.091274
hsa_circ_	-0.55214	0.091277
hsa_circ_	-1.94895	0.091308
hsa_circ_	-0.2543	0.091312
hsa_circ_	0.639845	0.091319
hsa_circ_	-0.32686	0.091329
hsa_circ_	0.462797	0.091333
hsa_circ_	0.73623	0.091341
hsa_circ_	-1.07263	0.091341
hsa_circ_	0.511472	0.091347
hsa_circ_	0.307472	0.09137
hsa_circ_	-0.95551	0.091374
hsa_circ_	0.832018	0.091376
hsa_circ_	-0.25489	0.091383
hsa_circ_	-0.42818	0.091383
hsa_circ_	-0.39742	0.091385
hsa_circ_	-0.44249	0.091394

hsa_circ_	0.599478	0.091408
hsa_circ_	0.360083	0.091412
hsa_circ_	-0.35272	0.091416
hsa_circ_	-0.40892	0.091417
hsa_circ_	-1.04595	0.091426
hsa_circ_	-1.08479	0.091426
hsa_circ_	-0.26403	0.091427
hsa_circ_	0.382991	0.091428
hsa_circ_	-0.44617	0.091444
hsa_circ_	0.426911	0.091444
hsa_circ_	-0.54406	0.091455
hsa_circ_	-0.25659	0.091459
hsa_circ_	0.677901	0.091463
hsa_circ_	-0.26516	0.091463
hsa_circ_	-0.64693	0.091465
hsa_circ_	0.304668	0.091476
hsa_circ_	-0.48846	0.091498
hsa_circ_	0.698585	0.0915
hsa_circ_	-0.38427	0.091501
hsa_circ_	0.580915	0.091501
hsa_circ_	0.518114	0.091519
hsa_circ_	-0.29101	0.09153
hsa_circ_	-0.28285	0.091531
hsa_circ_	-0.26554	0.091531
hsa_circ_	0.665051	0.091548
hsa_circ_	0.32102	0.091549
hsa_circ_	0.535136	0.091553
hsa_circ_	0.298066	0.091562
hsa_circ_	0.275522	0.091563
hsa_circ_	-0.28672	0.091565
hsa_circ_	0.282915	0.091572
hsa_circ_	-0.39902	0.091584
hsa_circ_	-0.48467	0.091616
hsa_circ_	0.436824	0.091618
hsa_circ_	-0.27257	0.091631
hsa_circ_	-0.30594	0.091634
hsa_circ_	0.247409	0.091639
hsa_circ_	-0.37205	0.091655
hsa_circ_	-0.52251	0.091656
hsa_circ_	-0.34423	0.091657
hsa_circ_	0.682999	0.09166
hsa_circ_	0.474014	0.091661
hsa_circ_	0.459255	0.091663
hsa_circ_	0.737658	0.09167
hsa_circ_	-0.41704	0.091681
hsa_circ_	-0.40382	0.091684
hsa_circ_	-0.58326	0.091687
hsa_circ_	-0.40323	0.091692
hsa_circ_	0.8978	0.091702
hsa_circ_	0.371129	0.091711
hsa_circ_	0.725635	0.091723
hsa_circ_	0.652603	0.091734
hsa_circ_	-0.30451	0.09174
hsa_circ_	0.357213	0.09174

hsa_circ_	0.280406	0.091745
hsa_circ_	-0.342	0.091745
hsa_circ_	-0.38692	0.091753
hsa_circ_	0.310549	0.091763
hsa_circ_	0.629706	0.091764
hsa_circ_	0.869164	0.091766
hsa_circ_	0.464913	0.091781
hsa_circ_	0.959677	0.091783
hsa_circ_	1.159266	0.091788
hsa_circ_	-0.87396	0.091793
hsa_circ_	-0.85864	0.091795
hsa_circ_	0.353006	0.091832
hsa_circ_	-0.32921	0.091833
hsa_circ_	0.276956	0.091834
hsa_circ_	-0.40933	0.091852
hsa_circ_	-0.26335	0.091853
hsa_circ_	-0.47098	0.091855
hsa_circ_	0.377236	0.091858
hsa_circ_	-0.2604	0.091859
hsa_circ_	-0.4676	0.09186
hsa_circ_	-0.38462	0.091868
hsa_circ_	-0.45449	0.091896
hsa_circ_	0.620445	0.091898
hsa_circ_	-0.42263	0.091903
hsa_circ_	0.442375	0.091906
hsa_circ_	0.54064	0.091907
hsa_circ_	-0.41643	0.091922
hsa_circ_	0.299196	0.091925
hsa_circ_	-0.41191	0.091932
hsa_circ_	-0.24316	0.09194
hsa_circ_	-0.30197	0.091948
hsa_circ_	0.416607	0.091956
hsa_circ_	-0.98964	0.09196
hsa_circ_	0.449386	0.091965
hsa_circ_	-0.32284	0.091966
hsa_circ_	1.004643	0.091989
hsa_circ_	-0.6294	0.091995
hsa_circ_	-1.92437	0.09201
hsa_circ_	0.251759	0.09202
hsa_circ_	1.096965	0.092023
hsa_circ_	0.283086	0.092039
hsa_circ_	-0.54442	0.092039
hsa_circ_	-0.32032	0.092054
hsa_circ_	0.746368	0.092071
hsa_circ_	0.557688	0.092073
hsa_circ_	0.851145	0.092079
hsa_circ_	-0.39799	0.092117
hsa_circ_	-0.27956	0.092137
hsa_circ_	0.381102	0.09214
hsa_circ_	-0.40663	0.092147
hsa_circ_	0.396635	0.092149
hsa_circ_	0.415654	0.092152
hsa_circ_	-0.43662	0.092155
hsa_circ_	0.593529	0.09216

hsa_circ_	0.310177	0.092182
hsa_circ_	0.383559	0.092184
hsa_circ_	-0.33268	0.092187
hsa_circ_	0.299268	0.092188
hsa_circ_	1.262327	0.092194
hsa_circ_	-0.32995	0.092196
hsa_circ_	0.449414	0.092201
hsa_circ_	0.373024	0.092208
hsa_circ_	-0.40167	0.092211
hsa_circ_	0.375552	0.09222
hsa_circ_	0.367301	0.092227
hsa_circ_	-0.44102	0.092233
hsa_circ_	0.728927	0.092234
hsa_circ_	-0.61766	0.092235
hsa_circ_	-0.25654	0.092248
hsa_circ_	0.258181	0.092252
hsa_circ_	-0.28336	0.092256
hsa_circ_	0.716335	0.092258
hsa_circ_	-0.35464	0.09226
hsa_circ_	0.716554	0.09226
hsa_circ_	-0.30873	0.092262
hsa_circ_	-0.50581	0.092268
hsa_circ_	-0.62008	0.092285
hsa_circ_	-0.58369	0.092288
hsa_circ_	0.673591	0.092289
hsa_circ_	-0.41677	0.092296
hsa_circ_	0.45614	0.092321
hsa_circ_	-0.40879	0.092329
hsa_circ_	0.303693	0.092332
hsa_circ_	0.536173	0.092332
hsa_circ_	0.295584	0.092335
hsa_circ_	0.914674	0.092343
hsa_circ_	0.736163	0.092346
hsa_circ_	-0.37701	0.092358
hsa_circ_	-0.61124	0.092374
hsa_circ_	-0.3819	0.092376
hsa_circ_	-0.32753	0.092381
hsa_circ_	-0.32481	0.092391
hsa_circ_	0.813414	0.092403
hsa_circ_	0.339575	0.092412
hsa_circ_	0.350176	0.092412
hsa_circ_	0.545966	0.092416
hsa_circ_	-0.31078	0.092447
hsa_circ_	0.346967	0.092453
hsa_circ_	0.432242	0.092453
hsa_circ_	0.433819	0.092454
hsa_circ_	0.275553	0.092461
hsa_circ_	-0.30358	0.09247
hsa_circ_	-0.27361	0.092476
hsa_circ_	0.53905	0.09248
hsa_circ_	0.25108	0.092484
hsa_circ_	-0.26425	0.092485
hsa_circ_	-0.34878	0.092491
hsa_circ_	0.24867	0.0925

hsa_circ_	-0.85421	0.092517
hsa_circ_	-0.2688	0.092524
hsa_circ_	0.533408	0.092526
hsa_circ_	-0.91725	0.092526
hsa_circ_	-0.31783	0.092528
hsa_circ_	0.424269	0.09253
hsa_circ_	-0.65978	0.092534
hsa_circ_	0.47423	0.092536
hsa_circ_	-0.53978	0.092541
hsa_circ_	0.407964	0.092543
hsa_circ_	-0.36145	0.092554
hsa_circ_	-0.5483	0.092564
hsa_circ_	0.418469	0.092569
hsa_circ_	0.57489	0.092584
hsa_circ_	-0.27989	0.092593
hsa_circ_	0.329142	0.092601
hsa_circ_	0.431025	0.092609
hsa_circ_	-0.31218	0.092611
hsa_circ_	0.544641	0.092632
hsa_circ_	0.373487	0.092632
hsa_circ_	1.061552	0.092638
hsa_circ_	0.250956	0.092639
hsa_circ_	0.436058	0.09264
hsa_circ_	0.388716	0.09264
hsa_circ_	0.39978	0.092647
hsa_circ_	-0.56778	0.092649
hsa_circ_	-0.28253	0.092654
hsa_circ_	-0.80594	0.09268
hsa_circ_	0.300726	0.092685
hsa_circ_	0.536659	0.092693
hsa_circ_	-0.43171	0.092697
hsa_circ_	0.796643	0.092697
hsa_circ_	-0.49617	0.0927
hsa_circ_	-0.58519	0.092701
hsa_circ_	0.533402	0.092705
hsa_circ_	-0.2682	0.092715
hsa_circ_	0.34785	0.092716
hsa_circ_	0.397252	0.092716
hsa_circ_	-0.62398	0.092729
hsa_circ_	0.346218	0.092755
hsa_circ_	-0.36755	0.092757
hsa_circ_	0.374334	0.092765
hsa_circ_	1.077775	0.092774
hsa_circ_	0.250812	0.092774
hsa_circ_	0.957762	0.092775
hsa_circ_	0.420643	0.09279
hsa_circ_	-0.34343	0.092795
hsa_circ_	-0.26932	0.092795
hsa_circ_	0.764435	0.09281
hsa_circ_	0.486996	0.092816
hsa_circ_	-0.49756	0.092831
hsa_circ_	0.832042	0.092845
hsa_circ_	-0.31813	0.092846
hsa_circ_	0.346262	0.092847

hsa_circ_	0.341643	0.092851
hsa_circ_	-0.43985	0.092853
hsa_circ_	0.389965	0.092861
hsa_circ_	0.370913	0.092864
hsa_circ_	0.464275	0.092877
hsa_circ_	0.514336	0.092892
hsa_circ_	-0.4884	0.092899
hsa_circ_	0.325347	0.092905
hsa_circ_	0.278213	0.092905
hsa_circ_	1.841605	0.092913
hsa_circ_	0.437383	0.092917
hsa_circ_	-0.42499	0.092922
hsa_circ_	0.899931	0.092924
hsa_circ_	0.294433	0.092937
hsa_circ_	-0.79823	0.09294
hsa_circ_	-0.56427	0.092941
hsa_circ_	-0.31056	0.092952
hsa_circ_	-0.5223	0.092953
hsa_circ_	-0.31479	0.092954
hsa_circ_	-0.50611	0.092967
hsa_circ_	-1.27632	0.092981
hsa_circ_	-0.89908	0.092982
hsa_circ_	-0.30055	0.092985
hsa_circ_	0.330899	0.092989
hsa_circ_	-0.99203	0.093001
hsa_circ_	-0.90302	0.093007
hsa_circ_	-0.27173	0.093007
hsa_circ_	-0.3604	0.093026
hsa_circ_	0.523496	0.093026
hsa_circ_	0.368401	0.093029
hsa_circ_	1.246239	0.093036
hsa_circ_	1.075883	0.093052
hsa_circ_	-0.65599	0.093058
hsa_circ_	-0.42741	0.093064
hsa_circ_	-0.32492	0.093064
hsa_circ_	0.533622	0.093073
hsa_circ_	-0.31108	0.093078
hsa_circ_	-0.28026	0.093087
hsa_circ_	-0.35322	0.093096
hsa_circ_	-0.75564	0.093104
hsa_circ_	0.374071	0.093108
hsa_circ_	0.258093	0.09311
hsa_circ_	-0.29557	0.093127
hsa_circ_	0.569148	0.093132
hsa_circ_	0.433905	0.093142
hsa_circ_	0.340571	0.093143
hsa_circ_	-0.32897	0.093144
hsa_circ_	0.352371	0.093154
hsa_circ_	-0.25625	0.093158
hsa_circ_	-0.34734	0.093171
hsa_circ_	0.716783	0.09318
hsa_circ_	0.878418	0.09319
hsa_circ_	-0.41489	0.093192
hsa_circ_	0.491826	0.093204

hsa_circ_	-0.32565	0.093214
hsa_circ_	-0.42905	0.093215
hsa_circ_	0.261585	0.093217
hsa_circ_	-0.67717	0.093223
hsa_circ_	-1.53602	0.093223
hsa_circ_	0.27265	0.093227
hsa_circ_	-0.35445	0.093246
hsa_circ_	-0.35723	0.093253
hsa_circ_	0.584323	0.093253
hsa_circ_	-0.31245	0.093264
hsa_circ_	-0.50801	0.093271
hsa_circ_	0.434727	0.093275
hsa_circ_	0.489706	0.093275
hsa_circ_	-0.52996	0.093302
hsa_circ_	0.652153	0.093306
hsa_circ_	-0.47986	0.093337
hsa_circ_	0.77968	0.093346
hsa_circ_	0.407894	0.09335
hsa_circ_	-0.25212	0.093377
hsa_circ_	0.419448	0.093398
hsa_circ_	0.253385	0.093399
hsa_circ_	0.371512	0.093411
hsa_circ_	-0.28268	0.093421
hsa_circ_	-0.25271	0.093432
hsa_circ_	-0.27537	0.093441
hsa_circ_	-0.47193	0.093445
hsa_circ_	-0.50052	0.093449
hsa_circ_	0.647712	0.093449
hsa_circ_	-0.694	0.093454
hsa_circ_	0.244605	0.093457
hsa_circ_	0.321949	0.093463
hsa_circ_	-0.2722	0.09347
hsa_circ_	0.492545	0.093471
hsa_circ_	0.343209	0.093472
hsa_circ_	0.349001	0.093478
hsa_circ_	-0.30094	0.093485
hsa_circ_	-0.3529	0.093486
hsa_circ_	-0.44248	0.093506
hsa_circ_	0.316401	0.093506
hsa_circ_	0.77638	0.093511
hsa_circ_	0.370922	0.093514
hsa_circ_	0.41345	0.093515
hsa_circ_	0.536197	0.093521
hsa_circ_	0.368997	0.093526
hsa_circ_	-0.93193	0.093527
hsa_circ_	-0.27807	0.093538
hsa_circ_	0.313134	0.093538
hsa_circ_	0.587688	0.093543
hsa_circ_	-0.38035	0.093546
hsa_circ_	-0.69	0.093554
hsa_circ_	0.338822	0.093559
hsa_circ_	0.282218	0.093567
hsa_circ_	0.362839	0.09357
hsa_circ_	-0.75708	0.093594

hsa_circ_	-0.37196	0.093633
hsa_circ_	0.315477	0.093646
hsa_circ_	-0.25195	0.09365
hsa_circ_	0.298158	0.093653
hsa_circ_	-0.39235	0.09366
hsa_circ_	-0.45712	0.093664
hsa_circ_	-0.54595	0.093669
hsa_circ_	-0.44642	0.093674
hsa_circ_	0.58024	0.093681
hsa_circ_	-1.57272	0.093683
hsa_circ_	0.399748	0.093688
hsa_circ_	0.262103	0.093691
hsa_circ_	0.374219	0.093696
hsa_circ_	-0.52795	0.093698
hsa_circ_	0.309447	0.093706
hsa_circ_	0.562323	0.093709
hsa_circ_	-1.03507	0.093725
hsa_circ_	0.294322	0.093729
hsa_circ_	0.330696	0.093739
hsa_circ_	0.604987	0.093745
hsa_circ_	0.308938	0.093764
hsa_circ_	0.299787	0.093774
hsa_circ_	0.360207	0.093784
hsa_circ_	-0.29535	0.093784
hsa_circ_	-1.01463	0.093791
hsa_circ_	-0.41312	0.093814
hsa_circ_	-0.38091	0.093816
hsa_circ_	0.444147	0.093821
hsa_circ_	0.709837	0.093832
hsa_circ_	0.571972	0.093838
hsa_circ_	-0.30287	0.093846
hsa_circ_	0.43932	0.093864
hsa_circ_	-1.01654	0.093868
hsa_circ_	0.586922	0.093874
hsa_circ_	0.634526	0.093909
hsa_circ_	0.526052	0.093914
hsa_circ_	0.664332	0.093915
hsa_circ_	0.384935	0.093918
hsa_circ_	0.337229	0.093922
hsa_circ_	0.839277	0.093922
hsa_circ_	0.878135	0.093927
hsa_circ_	0.427087	0.093935
hsa_circ_	0.322523	0.093941
hsa_circ_	-0.60291	0.093953
hsa_circ_	-0.37862	0.093956
hsa_circ_	-0.33188	0.093974
hsa_circ_	0.32423	0.093978
hsa_circ_	-0.49999	0.093983
hsa_circ_	-0.31194	0.094008
hsa_circ_	-0.49587	0.094011
hsa_circ_	0.30826	0.09402
hsa_circ_	-0.29371	0.094031
hsa_circ_	-0.34975	0.094036
hsa_circ_	-0.33945	0.094039

hsa_circ_	0.448493	0.094046
hsa_circ_	0.372935	0.094046
hsa_circ_	0.5172	0.094052
hsa_circ_	0.607815	0.094054
hsa_circ_	0.307069	0.094062
hsa_circ_	0.278699	0.09409
hsa_circ_	0.370848	0.094097
hsa_circ_	-0.27914	0.094105
hsa_circ_	-1.29541	0.094114
hsa_circ_	-0.49709	0.094137
hsa_circ_	-0.33239	0.094149
hsa_circ_	-0.36308	0.094161
hsa_circ_	0.244605	0.094177
hsa_circ_	-0.40015	0.094179
hsa_circ_	-0.40496	0.094192
hsa_circ_	-0.37946	0.094214
hsa_circ_	0.3735	0.094216
hsa_circ_	0.616455	0.094221
hsa_circ_	0.49795	0.094221
hsa_circ_	-0.43165	0.094222
hsa_circ_	0.283458	0.094243
hsa_circ_	-0.74654	0.094243
hsa_circ_	0.51499	0.094253
hsa_circ_	0.884414	0.094281
hsa_circ_	-0.38171	0.094283
hsa_circ_	0.43234	0.094285
hsa_circ_	0.533524	0.094295
hsa_circ_	0.375112	0.094296
hsa_circ_	-0.47437	0.094322
hsa_circ_	0.347139	0.094339
hsa_circ_	-0.58011	0.094343
hsa_circ_	-0.31436	0.094343
hsa_circ_	-0.40163	0.094348
hsa_circ_	0.310241	0.094357
hsa_circ_	-0.39365	0.094363
hsa_circ_	-0.3597	0.094365
hsa_circ_	0.305764	0.094373
hsa_circ_	-0.29021	0.094373
hsa_circ_	0.421884	0.094378
hsa_circ_	0.362904	0.094378
hsa_circ_	0.433668	0.09438
hsa_circ_	-0.56573	0.094381
hsa_circ_	0.272362	0.094394
hsa_circ_	-0.74878	0.094403
hsa_circ_	-0.46315	0.094408
hsa_circ_	-1.04339	0.094412
hsa_circ_	-0.45149	0.094415
hsa_circ_	1.247979	0.094416
hsa_circ_	0.260754	0.094417
hsa_circ_	-0.57728	0.09442
hsa_circ_	0.413565	0.094431
hsa_circ_	-0.90569	0.094433
hsa_circ_	0.533536	0.09444
hsa_circ_	-0.24752	0.09445

hsa_circ_	-0.28901	0.094453
hsa_circ_	0.326389	0.094462
hsa_circ_	0.433359	0.094465
hsa_circ_	-0.47367	0.094468
hsa_circ_	-0.45428	0.094476
hsa_circ_	0.408414	0.094485
hsa_circ_	0.289177	0.09449
hsa_circ_	-0.25648	0.094491
hsa_circ_	-0.25392	0.094492
hsa_circ_	0.4649	0.094497
hsa_circ_	0.429467	0.094519
hsa_circ_	-0.67754	0.094519
hsa_circ_	-0.32665	0.094527
hsa_circ_	1.257391	0.094528
hsa_circ_	-0.30532	0.094536
hsa_circ_	0.449656	0.094541
hsa_circ_	0.475931	0.094546
hsa_circ_	-0.37163	0.094552
hsa_circ_	-1.65732	0.094556
hsa_circ_	-0.29569	0.09456
hsa_circ_	-2.86743	0.094561
hsa_circ_	-0.84687	0.094575
hsa_circ_	-0.28126	0.094592
hsa_circ_	0.279759	0.094596
hsa_circ_	0.294333	0.094611
hsa_circ_	0.568291	0.094631
hsa_circ_	-0.74986	0.094649
hsa_circ_	0.291905	0.094672
hsa_circ_	0.264798	0.094674
hsa_circ_	-0.30899	0.094688
hsa_circ_	0.413986	0.09469
hsa_circ_	-0.80276	0.094696
hsa_circ_	-0.43467	0.094699
hsa_circ_	0.50652	0.094702
hsa_circ_	0.301185	0.094713
hsa_circ_	0.671031	0.094721
hsa_circ_	-0.32739	0.094724
hsa_circ_	0.354942	0.094741
hsa_circ_	-0.55906	0.094742
hsa_circ_	-0.33743	0.094747
hsa_circ_	0.331478	0.094748
hsa_circ_	0.445703	0.09476
hsa_circ_	-0.36341	0.094762
hsa_circ_	-0.46662	0.094763
hsa_circ_	1.046703	0.094769
hsa_circ_	-0.35591	0.094781
hsa_circ_	0.347238	0.094809
hsa_circ_	-0.40207	0.094831
hsa_circ_	0.61525	0.094839
hsa_circ_	0.323804	0.094852
hsa_circ_	-0.66187	0.094856
hsa_circ_	-0.41321	0.094864
hsa_circ_	-0.30644	0.094865
hsa_circ_	-0.29045	0.094872

hsa_circ_	0.664972	0.094894
hsa_circ_	1.139927	0.094917
hsa_circ_	0.627454	0.094923
hsa_circ_	0.417346	0.094925
hsa_circ_	-0.25652	0.094933
hsa_circ_	1.15205	0.094944
hsa_circ_	-0.34432	0.094956
hsa_circ_	-0.24335	0.094959
hsa_circ_	0.358676	0.094974
hsa_circ_	0.302169	0.094985
hsa_circ_	0.403837	0.095
hsa_circ_	-0.47591	0.095002
hsa_circ_	-0.2616	0.095004
hsa_circ_	0.30207	0.095004
hsa_circ_	-0.27932	0.095015
hsa_circ_	0.907772	0.095017
hsa_circ_	0.303333	0.095026
hsa_circ_	-0.38656	0.095044
hsa_circ_	0.956963	0.09506
hsa_circ_	-0.3338	0.095069
hsa_circ_	0.312375	0.095072
hsa_circ_	0.307605	0.095074
hsa_circ_	-0.34743	0.095076
hsa_circ_	0.482659	0.095077
hsa_circ_	-0.27265	0.095099
hsa_circ_	-0.77976	0.095103
hsa_circ_	0.518903	0.095108
hsa_circ_	-0.39791	0.095112
hsa_circ_	0.327527	0.095113
hsa_circ_	0.516436	0.095141
hsa_circ_	0.35051	0.095142
hsa_circ_	0.264845	0.095159
hsa_circ_	0.4532	0.095163
hsa_circ_	0.263757	0.095165
hsa_circ_	-0.38487	0.095167
hsa_circ_	0.329093	0.095168
hsa_circ_	-1.18935	0.09517
hsa_circ_	0.982346	0.095187
hsa_circ_	0.353334	0.095188
hsa_circ_	-0.33325	0.095191
hsa_circ_	0.625385	0.095199
hsa_circ_	-0.35476	0.095214
hsa_circ_	0.286605	0.09522
hsa_circ_	0.620747	0.095231
hsa_circ_	-0.24709	0.095231
hsa_circ_	0.300719	0.095237
hsa_circ_	-0.44457	0.09524
hsa_circ_	0.395066	0.095256
hsa_circ_	0.920321	0.095264
hsa_circ_	-0.30442	0.095267
hsa_circ_	0.633535	0.095269
hsa_circ_	-0.47634	0.095291
hsa_circ_	-0.2779	0.095294
hsa_circ_	-0.97622	0.0953

hsa_circ_	-0.29976	0.095304
hsa_circ_	-0.31528	0.095305
hsa_circ_	0.896457	0.095305
hsa_circ_	0.609744	0.095324
hsa_circ_	-0.32334	0.095334
hsa_circ_	-0.55649	0.095339
hsa_circ_	0.649912	0.09535
hsa_circ_	-0.39599	0.095363
hsa_circ_	-0.28763	0.095381
hsa_circ_	0.251173	0.09539
hsa_circ_	-0.35967	0.095392
hsa_circ_	-0.28011	0.095394
hsa_circ_	0.50534	0.0954
hsa_circ_	-0.24942	0.095404
hsa_circ_	0.566692	0.095404
hsa_circ_	-0.28203	0.09541
hsa_circ_	-0.42517	0.095411
hsa_circ_	-0.56901	0.095416
hsa_circ_	-0.95814	0.095423
hsa_circ_	0.400279	0.095427
hsa_circ_	0.453203	0.095434
hsa_circ_	-0.33338	0.095455
hsa_circ_	-0.72977	0.095457
hsa_circ_	0.373023	0.095476
hsa_circ_	0.349365	0.095476
hsa_circ_	0.421834	0.095479
hsa_circ_	0.384286	0.095482
hsa_circ_	0.499052	0.095483
hsa_circ_	0.511437	0.095487
hsa_circ_	0.372032	0.095503
hsa_circ_	0.374167	0.095508
hsa_circ_	-0.96358	0.09551
hsa_circ_	0.317395	0.095518
hsa_circ_	0.35845	0.09552
hsa_circ_	0.880057	0.095521
hsa_circ_	0.396503	0.09553
hsa_circ_	-0.31719	0.095531
hsa_circ_	0.500286	0.09554
hsa_circ_	0.550572	0.095548
hsa_circ_	-0.34203	0.095553
hsa_circ_	0.70243	0.095554
hsa_circ_	-0.48159	0.095573
hsa_circ_	-0.35	0.095574
hsa_circ_	0.364224	0.095595
hsa_circ_	-0.72366	0.095597
hsa_circ_	-0.29173	0.095606
hsa_circ_	0.456848	0.095617
hsa_circ_	0.393143	0.095629
hsa_circ_	0.54941	0.095641
hsa_circ_	-0.45833	0.095656
hsa_circ_	0.300315	0.095694
hsa_circ_	-1.49833	0.095697
hsa_circ_	0.339793	0.095702
hsa_circ_	-0.25761	0.095732

hsa_circ_	-0.24047	0.095737
hsa_circ_	-0.4212	0.095737
hsa_circ_	-0.34049	0.095757
hsa_circ_	0.366535	0.09576
hsa_circ_	-0.33464	0.095765
hsa_circ_	-0.99378	0.095772
hsa_circ_	0.438665	0.095776
hsa_circ_	-0.28903	0.095786
hsa_circ_	-0.73862	0.095787
hsa_circ_	0.322466	0.095791
hsa_circ_	-0.28326	0.095792
hsa_circ_	0.301891	0.095832
hsa_circ_	0.587243	0.095845
hsa_circ_	-0.28081	0.095848
hsa_circ_	-0.27684	0.09586
hsa_circ_	-0.53115	0.095863
hsa_circ_	-0.27221	0.095875
hsa_circ_	-0.52238	0.095888
hsa_circ_	-0.46637	0.095893
hsa_circ_	0.295068	0.095928
hsa_circ_	-0.67352	0.095928
hsa_circ_	0.444252	0.095929
hsa_circ_	-0.81726	0.09593
hsa_circ_	-0.2599	0.095931
hsa_circ_	0.317934	0.095954
hsa_circ_	0.307302	0.095962
hsa_circ_	-1.063	0.095974
hsa_circ_	-0.59104	0.095975
hsa_circ_	-0.8146	0.095983
hsa_circ_	-0.32632	0.095984
hsa_circ_	0.713164	0.095988
hsa_circ_	-0.44595	0.095991
hsa_circ_	-0.34564	0.096005
hsa_circ_	-0.35528	0.096016
hsa_circ_	0.300467	0.096029
hsa_circ_	-0.5727	0.096034
hsa_circ_	0.355002	0.096038
hsa_circ_	-0.4224	0.096043
hsa_circ_	-0.32393	0.096048
hsa_circ_	0.279579	0.096049
hsa_circ_	0.277644	0.096062
hsa_circ_	0.335356	0.096063
hsa_circ_	0.488066	0.096063
hsa_circ_	-1.06097	0.096064
hsa_circ_	-0.26418	0.096069
hsa_circ_	0.463723	0.096072
hsa_circ_	0.388248	0.096074
hsa_circ_	-0.36308	0.09609
hsa_circ_	-0.42384	0.096116
hsa_circ_	0.393633	0.096122
hsa_circ_	-0.69217	0.096132
hsa_circ_	-0.82117	0.096133
hsa_circ_	0.377492	0.096142
hsa_circ_	0.240943	0.09615

hsa_circ_	0.608883	0.096159
hsa_circ_	-1.01513	0.096166
hsa_circ_	0.338307	0.096184
hsa_circ_	0.27118	0.096187
hsa_circ_	-0.37614	0.09619
hsa_circ_	0.587936	0.096191
hsa_circ_	-0.8095	0.096196
hsa_circ_	0.425688	0.096206
hsa_circ_	0.386412	0.096208
hsa_circ_	0.692764	0.096225
hsa_circ_	0.315753	0.096231
hsa_circ_	-0.29463	0.096233
hsa_circ_	0.322609	0.096242
hsa_circ_	-0.51776	0.09626
hsa_circ_	-0.81841	0.096273
hsa_circ_	0.246099	0.096274
hsa_circ_	0.300201	0.096281
hsa_circ_	0.428825	0.09631
hsa_circ_	-0.6858	0.09631
hsa_circ_	-0.76835	0.096317
hsa_circ_	-0.29794	0.09632
hsa_circ_	0.337517	0.096322
hsa_circ_	0.357541	0.096324
hsa_circ_	-0.66781	0.096335
hsa_circ_	0.466831	0.096335
hsa_circ_	-0.32055	0.096336
hsa_circ_	0.375749	0.096338
hsa_circ_	0.301136	0.096339
hsa_circ_	1.444369	0.096342
hsa_circ_	0.304886	0.096384
hsa_circ_	0.272125	0.0964
hsa_circ_	-0.32434	0.096401
hsa_circ_	-0.4189	0.096413
hsa_circ_	0.246088	0.096425
hsa_circ_	0.337825	0.096439
hsa_circ_	-0.30163	0.096452
hsa_circ_	-0.33433	0.096455
hsa_circ_	0.774248	0.096456
hsa_circ_	0.355642	0.096472
hsa_circ_	-0.2514	0.096473
hsa_circ_	0.346496	0.096476
hsa_circ_	0.321348	0.096487
hsa_circ_	-0.39616	0.096488
hsa_circ_	-0.28577	0.096494
hsa_circ_	0.796081	0.096496
hsa_circ_	-0.42793	0.096497
hsa_circ_	-0.67018	0.096511
hsa_circ_	0.443622	0.096511
hsa_circ_	-0.32835	0.096515
hsa_circ_	-0.35204	0.096518
hsa_circ_	-0.47541	0.096522
hsa_circ_	0.458397	0.096526
hsa_circ_	0.645833	0.096537
hsa_circ_	0.264126	0.096542

hsa_circ_	0.300733	0.096547
hsa_circ_	0.423257	0.096551
hsa_circ_	-0.66529	0.096571
hsa_circ_	-0.43681	0.096571
hsa_circ_	0.55002	0.096572
hsa_circ_	-0.56743	0.096593
hsa_circ_	-1.14752	0.096601
hsa_circ_	-0.32833	0.096611
hsa_circ_	-0.43256	0.096617
hsa_circ_	-0.45006	0.09663
hsa_circ_	-0.29374	0.09664
hsa_circ_	0.348652	0.096641
hsa_circ_	1.436713	0.096664
hsa_circ_	-0.37292	0.096671
hsa_circ_	0.695404	0.096677
hsa_circ_	-0.37852	0.096697
hsa_circ_	-0.73227	0.096707
hsa_circ_	-0.27833	0.096711
hsa_circ_	-0.28932	0.096713
hsa_circ_	-0.35828	0.096723
hsa_circ_	0.437887	0.096724
hsa_circ_	-0.25197	0.096725
hsa_circ_	0.379277	0.096729
hsa_circ_	-1.76258	0.096733
hsa_circ_	0.420847	0.096755
hsa_circ_	1.389782	0.096762
hsa_circ_	-0.74167	0.096781
hsa_circ_	-0.89918	0.096786
hsa_circ_	-0.32252	0.096788
hsa_circ_	-0.77248	0.096802
hsa_circ_	0.360181	0.096809
hsa_circ_	-0.37454	0.096814
hsa_circ_	-0.28098	0.096824
hsa_circ_	0.2976	0.096824
hsa_circ_	-0.35798	0.096844
hsa_circ_	-0.31454	0.096846
hsa_circ_	-0.28889	0.096851
hsa_circ_	-0.38811	0.096857
hsa_circ_	-0.32018	0.096864
hsa_circ_	-0.25432	0.096871
hsa_circ_	1.576528	0.096896
hsa_circ_	1.377891	0.096902
hsa_circ_	0.72888	0.096928
hsa_circ_	-0.25503	0.096932
hsa_circ_	-0.27577	0.096935
hsa_circ_	-0.29833	0.096941
hsa_circ_	-0.31805	0.096972
hsa_circ_	0.393989	0.096978
hsa_circ_	-0.52621	0.096999
hsa_circ_	0.387281	0.097015
hsa_circ_	0.284808	0.097015
hsa_circ_	-0.29058	0.097021
hsa_circ_	-0.38442	0.097022
hsa_circ_	0.249718	0.097022

hsa_circ_	-0.32715	0.09703
hsa_circ_	-0.27681	0.097045
hsa_circ_	0.470094	0.097048
hsa_circ_	0.502553	0.097056
hsa_circ_	0.709189	0.09706
hsa_circ_	0.865976	0.097073
hsa_circ_	0.662696	0.097075
hsa_circ_	-0.2905	0.09708
hsa_circ_	-0.2901	0.097092
hsa_circ_	0.435692	0.097095
hsa_circ_	-0.73742	0.097106
hsa_circ_	0.290637	0.097107
hsa_circ_	0.311468	0.097111
hsa_circ_	0.286293	0.097115
hsa_circ_	-0.32925	0.097115
hsa_circ_	0.370453	0.09712
hsa_circ_	-0.55076	0.097126
hsa_circ_	-0.73986	0.097129
hsa_circ_	0.451414	0.09713
hsa_circ_	0.295138	0.09713
hsa_circ_	0.463268	0.097133
hsa_circ_	-0.43417	0.097149
hsa_circ_	-0.31831	0.097154
hsa_circ_	-0.39972	0.097156
hsa_circ_	0.254997	0.097158
hsa_circ_	-0.45675	0.097163
hsa_circ_	0.492325	0.097205
hsa_circ_	0.620434	0.097211
hsa_circ_	0.499068	0.097211
hsa_circ_	1.152519	0.097216
hsa_circ_	-0.42021	0.097224
hsa_circ_	0.261694	0.097224
hsa_circ_	-0.93882	0.097224
hsa_circ_	0.261876	0.097224
hsa_circ_	0.333205	0.097226
hsa_circ_	-0.24227	0.097237
hsa_circ_	0.573976	0.097238
hsa_circ_	0.486876	0.097238
hsa_circ_	-0.51732	0.097239
hsa_circ_	0.460577	0.097252
hsa_circ_	-0.87634	0.097257
hsa_circ_	-2.9124	0.097257
hsa_circ_	0.307597	0.097259
hsa_circ_	0.324571	0.097272
hsa_circ_	0.361649	0.097285
hsa_circ_	0.35446	0.097287
hsa_circ_	-0.35491	0.097291
hsa_circ_	-1.07125	0.097298
hsa_circ_	0.281193	0.09731
hsa_circ_	-0.71781	0.09732
hsa_circ_	0.432298	0.097323
hsa_circ_	0.270946	0.097328
hsa_circ_	-0.49105	0.097336
hsa_circ_	-0.38345	0.097361

hsa_circ_	0.438585	0.097361
hsa_circ_	-0.25848	0.097377
hsa_circ_	-0.23807	0.097382
hsa_circ_	-0.41199	0.097396
hsa_circ_	-0.30563	0.097404
hsa_circ_	0.409674	0.097406
hsa_circ_	-0.30395	0.097407
hsa_circ_	-0.36867	0.097419
hsa_circ_	0.322921	0.097419
hsa_circ_	0.453285	0.097421
hsa_circ_	1.130644	0.097422
hsa_circ_	-1.18171	0.097424
hsa_circ_	0.453428	0.097438
hsa_circ_	-0.25209	0.097442
hsa_circ_	0.344915	0.097459
hsa_circ_	-0.3126	0.09746
hsa_circ_	-1.10051	0.097468
hsa_circ_	0.480312	0.097473
hsa_circ_	0.302342	0.097473
hsa_circ_	-0.32392	0.097473
hsa_circ_	-0.48244	0.097489
hsa_circ_	-0.4125	0.097492
hsa_circ_	0.863722	0.097507
hsa_circ_	0.386692	0.097512
hsa_circ_	-0.28278	0.097521
hsa_circ_	-0.42229	0.097527
hsa_circ_	-0.59396	0.097532
hsa_circ_	0.361963	0.097536
hsa_circ_	-0.69525	0.09754
hsa_circ_	0.598345	0.097546
hsa_circ_	-0.26157	0.097565
hsa_circ_	-0.77822	0.097566
hsa_circ_	-0.88254	0.097571
hsa_circ_	0.280318	0.097579
hsa_circ_	-0.3026	0.097596
hsa_circ_	-0.28956	0.097605
hsa_circ_	0.754558	0.097612
hsa_circ_	0.278031	0.097618
hsa_circ_	-0.29921	0.097618
hsa_circ_	-0.25444	0.097622
hsa_circ_	0.586162	0.097622
hsa_circ_	-0.26684	0.097624
hsa_circ_	0.409849	0.09763
hsa_circ_	-0.5943	0.09763
hsa_circ_	-0.32417	0.097633
hsa_circ_	-0.30554	0.097634
hsa_circ_	0.603539	0.097636
hsa_circ_	-0.35739	0.097638
hsa_circ_	0.441304	0.097644
hsa_circ_	0.293822	0.097644
hsa_circ_	0.320351	0.097667
hsa_circ_	0.333965	0.097673
hsa_circ_	-0.42142	0.097676
hsa_circ_	0.332538	0.097678

hsa_circ_	-0.38104	0.097681
hsa_circ_	-0.2814	0.097696
hsa_circ_	0.292547	0.097699
hsa_circ_	-0.23401	0.097701
hsa_circ_	-0.29062	0.097716
hsa_circ_	0.315993	0.097725
hsa_circ_	-0.6915	0.097731
hsa_circ_	0.373593	0.097738
hsa_circ_	0.51159	0.097747
hsa_circ_	-1.00528	0.097775
hsa_circ_	-0.2867	0.097779
hsa_circ_	-0.79346	0.097805
hsa_circ_	0.288847	0.097805
hsa_circ_	0.338685	0.097808
hsa_circ_	-0.61761	0.097813
hsa_circ_	-0.39396	0.097816
hsa_circ_	0.281042	0.097825
hsa_circ_	0.370052	0.097829
hsa_circ_	-0.52348	0.097831
hsa_circ_	1.103108	0.097832
hsa_circ_	0.563703	0.097836
hsa_circ_	0.51104	0.097845
hsa_circ_	0.307688	0.097854
hsa_circ_	-0.40608	0.097873
hsa_circ_	-0.23464	0.097876
hsa_circ_	0.612388	0.097914
hsa_circ_	0.654101	0.097915
hsa_circ_	-0.37338	0.09792
hsa_circ_	-0.44449	0.097922
hsa_circ_	-0.38413	0.097925
hsa_circ_	0.303123	0.097926
hsa_circ_	0.537845	0.097941
hsa_circ_	0.298275	0.097948
hsa_circ_	0.402649	0.097953
hsa_circ_	-0.31503	0.097957
hsa_circ_	0.582879	0.097972
hsa_circ_	-0.45189	0.097977
hsa_circ_	-0.54881	0.09799
hsa_circ_	0.348443	0.097996
hsa_circ_	-0.30521	0.09801
hsa_circ_	0.260093	0.098013
hsa_circ_	-0.31226	0.098021
hsa_circ_	0.387582	0.098022
hsa_circ_	0.292107	0.09803
hsa_circ_	0.287684	0.098034
hsa_circ_	-0.29638	0.098041
hsa_circ_	-0.26893	0.098048
hsa_circ_	0.477111	0.098052
hsa_circ_	-0.34138	0.098062
hsa_circ_	-1.00621	0.098063
hsa_circ_	1.35322	0.098066
hsa_circ_	-0.39826	0.098071
hsa_circ_	-0.26309	0.098076
hsa_circ_	-0.3622	0.098081

hsa_circ_	0.906817	0.098081
hsa_circ_	0.370711	0.098086
hsa_circ_	0.549744	0.098091
hsa_circ_	0.360225	0.098091
hsa_circ_	-0.32876	0.098094
hsa_circ_	0.340695	0.098099
hsa_circ_	-0.5982	0.098107
hsa_circ_	0.367372	0.098109
hsa_circ_	0.384321	0.098121
hsa_circ_	0.373413	0.098122
hsa_circ_	-0.31478	0.098136
hsa_circ_	0.369569	0.098143
hsa_circ_	0.311332	0.098145
hsa_circ_	0.316511	0.09815
hsa_circ_	0.34455	0.098152
hsa_circ_	0.330117	0.098158
hsa_circ_	0.48864	0.098158
hsa_circ_	0.317519	0.098163
hsa_circ_	-0.36968	0.098176
hsa_circ_	-0.35254	0.098179
hsa_circ_	-0.37386	0.098183
hsa_circ_	-0.37662	0.098185
hsa_circ_	-0.35438	0.098193
hsa_circ_	-0.29417	0.098195
hsa_circ_	-0.28523	0.098205
hsa_circ_	-0.26171	0.098217
hsa_circ_	0.346916	0.098223
hsa_circ_	-0.2953	0.098238
hsa_circ_	0.420118	0.098255
hsa_circ_	0.440328	0.098257
hsa_circ_	-0.26525	0.098269
hsa_circ_	-0.81922	0.098278
hsa_circ_	0.432264	0.098278
hsa_circ_	-0.23681	0.098283
hsa_circ_	-0.38468	0.098284
hsa_circ_	1.048124	0.098291
hsa_circ_	0.252837	0.098299
hsa_circ_	-0.36525	0.098311
hsa_circ_	-0.28813	0.098322
hsa_circ_	0.451929	0.098324
hsa_circ_	0.763253	0.098335
hsa_circ_	0.279438	0.098339
hsa_circ_	-0.5343	0.098344
hsa_circ_	-0.25322	0.098349
hsa_circ_	0.28105	0.098349
hsa_circ_	-0.26979	0.098349
hsa_circ_	0.368427	0.098357
hsa_circ_	0.976688	0.098364
hsa_circ_	-0.29175	0.098381
hsa_circ_	-0.32036	0.098384
hsa_circ_	0.268512	0.098405
hsa_circ_	-0.5653	0.098406
hsa_circ_	-0.68907	0.098412
hsa_circ_	-0.2549	0.098418

hsa_circ_	-0.66867	0.098429
hsa_circ_	0.414812	0.098437
hsa_circ_	0.378848	0.098438
hsa_circ_	-1.12175	0.098442
hsa_circ_	-1.06947	0.098465
hsa_circ_	1.465412	0.098474
hsa_circ_	0.40671	0.098482
hsa_circ_	-0.7494	0.098494
hsa_circ_	-0.45442	0.098495
hsa_circ_	0.651473	0.098508
hsa_circ_	0.446459	0.098511
hsa_circ_	0.253232	0.098511
hsa_circ_	0.737867	0.098516
hsa_circ_	0.330244	0.098547
hsa_circ_	0.379722	0.098549
hsa_circ_	-0.26259	0.098567
hsa_circ_	-0.4038	0.098571
hsa_circ_	-0.48936	0.098582
hsa_circ_	0.560513	0.098598
hsa_circ_	0.348292	0.098599
hsa_circ_	-0.29298	0.098603
hsa_circ_	-0.28618	0.098605
hsa_circ_	0.691495	0.098612
hsa_circ_	-0.87394	0.098613
hsa_circ_	0.423371	0.098615
hsa_circ_	0.468778	0.098615
hsa_circ_	-0.29707	0.098619
hsa_circ_	-0.32549	0.09862
hsa_circ_	-0.84812	0.098623
hsa_circ_	0.327377	0.098628
hsa_circ_	-1.43337	0.098646
hsa_circ_	-0.99403	0.098652
hsa_circ_	-0.56364	0.098657
hsa_circ_	-0.30531	0.09867
hsa_circ_	-0.28404	0.098684
hsa_circ_	0.392951	0.098713
hsa_circ_	-0.40348	0.098718
hsa_circ_	0.268484	0.098738
hsa_circ_	-0.49981	0.09874
hsa_circ_	-0.28617	0.098754
hsa_circ_	-0.68245	0.098765
hsa_circ_	-0.26934	0.098782
hsa_circ_	0.267154	0.098782
hsa_circ_	-0.58018	0.098786
hsa_circ_	-0.98164	0.098791
hsa_circ_	0.38867	0.0988
hsa_circ_	-0.43413	0.098824
hsa_circ_	0.562713	0.098849
hsa_circ_	-0.41231	0.09885
hsa_circ_	-0.5503	0.098856
hsa_circ_	-0.3349	0.09886
hsa_circ_	-0.52132	0.09887
hsa_circ_	0.452058	0.098871
hsa_circ_	0.457329	0.098872

hsa_circ_	0.474164	0.098875
hsa_circ_	1.233139	0.098876
hsa_circ_	0.418162	0.098876
hsa_circ_	-0.37631	0.098885
hsa_circ_	0.284885	0.098893
hsa_circ_	-1.04852	0.098895
hsa_circ_	0.544987	0.098897
hsa_circ_	0.310299	0.098909
hsa_circ_	0.305915	0.098909
hsa_circ_	0.300033	0.09892
hsa_circ_	0.308349	0.098922
hsa_circ_	-0.31032	0.098927
hsa_circ_	-0.26069	0.098929
hsa_circ_	0.387428	0.098968
hsa_circ_	-0.38137	0.09897
hsa_circ_	0.552599	0.098975
hsa_circ_	0.914685	0.098978
hsa_circ_	0.38493	0.098979
hsa_circ_	0.326942	0.099
hsa_circ_	0.645915	0.099007
hsa_circ_	0.336577	0.099013
hsa_circ_	-0.39274	0.099015
hsa_circ_	0.275845	0.099028
hsa_circ_	-0.37762	0.099028
hsa_circ_	-0.2769	0.099045
hsa_circ_	0.765536	0.099063
hsa_circ_	0.470792	0.099076
hsa_circ_	0.328331	0.099088
hsa_circ_	-0.30815	0.099101
hsa_circ_	-0.26566	0.099109
hsa_circ_	0.315003	0.099117
hsa_circ_	-0.39407	0.09912
hsa_circ_	-0.44526	0.099129
hsa_circ_	0.593752	0.09913
hsa_circ_	0.76559	0.099134
hsa_circ_	0.64404	0.099135
hsa_circ_	-0.37321	0.099136
hsa_circ_	0.401563	0.099136
hsa_circ_	0.331995	0.099144
hsa_circ_	-0.62263	0.099151
hsa_circ_	-0.30188	0.099156
hsa_circ_	-0.24993	0.099157
hsa_circ_	0.439031	0.099164
hsa_circ_	-0.47999	0.09917
hsa_circ_	0.7822	0.099183
hsa_circ_	1.207023	0.099192
hsa_circ_	0.256082	0.099199
hsa_circ_	0.328256	0.099213
hsa_circ_	-0.56141	0.099222
hsa_circ_	0.404715	0.099224
hsa_circ_	-0.55161	0.099245
hsa_circ_	-0.9268	0.099252
hsa_circ_	-0.28849	0.099256
hsa_circ_	-0.31896	0.09926

hsa_circ_	-0.3209	0.099264
hsa_circ_	-0.42068	0.099269
hsa_circ_	0.433244	0.099316
hsa_circ_	0.46957	0.099319
hsa_circ_	0.329599	0.099325
hsa_circ_	0.30178	0.099335
hsa_circ_	-0.42244	0.099347
hsa_circ_	0.366976	0.099348
hsa_circ_	-0.62869	0.099352
hsa_circ_	-1.10078	0.099374
hsa_circ_	0.28486	0.099376
hsa_circ_	0.380525	0.099387
hsa_circ_	-0.75886	0.099388
hsa_circ_	-0.89378	0.099389
hsa_circ_	0.283684	0.099391
hsa_circ_	-0.2547	0.099401
hsa_circ_	-0.58154	0.099407
hsa_circ_	1.388803	0.099416
hsa_circ_	0.406741	0.099418
hsa_circ_	-0.25689	0.099421
hsa_circ_	0.308553	0.099426
hsa_circ_	-0.55772	0.099439
hsa_circ_	0.27441	0.099453
hsa_circ_	-0.33685	0.099463
hsa_circ_	-0.46371	0.099464
hsa_circ_	0.445821	0.09949
hsa_circ_	-0.42978	0.099498
hsa_circ_	0.373451	0.099524
hsa_circ_	-0.32116	0.099541
hsa_circ_	0.309263	0.099545
hsa_circ_	-0.49039	0.099547
hsa_circ_	0.358973	0.0996
hsa_circ_	0.303616	0.0996
hsa_circ_	0.4959	0.099606
hsa_circ_	-0.39009	0.099615
hsa_circ_	0.308334	0.099618
hsa_circ_	0.361325	0.099623
hsa_circ_	0.381024	0.099648
hsa_circ_	0.734442	0.099659
hsa_circ_	0.890932	0.099665
hsa_circ_	-0.31562	0.099671
hsa_circ_	-0.45997	0.099673
hsa_circ_	-0.2907	0.099676
hsa_circ_	-0.26876	0.099677
hsa_circ_	0.281281	0.099677
hsa_circ_	-0.27785	0.099678
hsa_circ_	-0.39941	0.09969
hsa_circ_	-0.33736	0.099696
hsa_circ_	0.79028	0.099702
hsa_circ_	-0.30111	0.099716
hsa_circ_	-0.58096	0.099742
hsa_circ_	0.254195	0.099748
hsa_circ_	-0.76582	0.099753
hsa_circ_	0.343408	0.099759

hsa_circ_	0.467461	0.099765
hsa_circ_	-0.43305	0.099767
hsa_circ_	0.936177	0.099769
hsa_circ_	0.657304	0.099784
hsa_circ_	-0.44983	0.099787
hsa_circ_	-0.3309	0.099788
hsa_circ_	0.34782	0.099789
hsa_circ_	-0.24305	0.099791
hsa_circ_	-0.66476	0.099796
hsa_circ_	0.314316	0.099798
hsa_circ_	0.364204	0.099799
hsa_circ_	-0.30755	0.0998
hsa_circ_	0.358532	0.099804
hsa_circ_	-0.27614	0.099837
hsa_circ_	-0.36839	0.099838
hsa_circ_	-0.33129	0.099858
hsa_circ_	-0.27046	0.099864
hsa_circ_	0.29024	0.09988
hsa_circ_	-0.83828	0.099883
hsa_circ_	-0.4041	0.099894
hsa_circ_	-0.25411	0.099901
hsa_circ_	-0.48081	0.099909
hsa_circ_	-1.07603	0.099926
hsa_circ_	0.451466	0.099927
hsa_circ_	-0.403	0.099928
hsa_circ_	-0.54943	0.099931
hsa_circ_	-0.28245	0.099934
hsa_circ_	-0.2827	0.099934
hsa_circ_	-0.56254	0.099937
hsa_circ_	-1.00152	0.099939
hsa_circ_	0.267029	0.099955
hsa_circ_	0.60853	0.099975
hsa_circ_	0.30718	0.099979
hsa_circ_	0.377687	0.099979
hsa_circ_	-0.23813	0.099981
hsa_circ_	0.509254	0.099984
hsa_circ_	0.87074	0.099984
hsa_circ_	-0.28524	0.099989
hsa_circ_	-0.58564	0.100008
hsa_circ_	-0.25295	0.100008
hsa_circ_	0.258631	0.100012
hsa_circ_	-0.46549	0.100023
hsa_circ_	0.28345	0.100027
hsa_circ_	0.703192	0.100028
hsa_circ_	-0.25257	0.100034
hsa_circ_	-0.69519	0.100036
hsa_circ_	-0.28776	0.100042
hsa_circ_	0.874225	0.100046
hsa_circ_	0.465032	0.100047
hsa_circ_	-0.57815	0.100051
hsa_circ_	0.421123	0.100056
hsa_circ_	1.302289	0.100058
hsa_circ_	0.444167	0.100082
hsa_circ_	-0.48778	0.100094

hsa_circ_	0.336494	0.100097
hsa_circ_	-0.44135	0.100103
hsa_circ_	-0.51142	0.100156
hsa_circ_	0.419662	0.100156
hsa_circ_	0.414484	0.100168
hsa_circ_	-0.41338	0.10019
hsa_circ_	-0.54956	0.100205
hsa_circ_	0.376778	0.100214
hsa_circ_	0.41435	0.100233
hsa_circ_	-0.42851	0.100234
hsa_circ_	-0.90212	0.100238
hsa_circ_	-0.47809	0.100249
hsa_circ_	-0.31076	0.100259
hsa_circ_	0.552478	0.100275
hsa_circ_	-1.17244	0.100281
hsa_circ_	-0.55135	0.100289
hsa_circ_	-1.09947	0.100289
hsa_circ_	-0.88026	0.100292
hsa_circ_	-0.33143	0.100296
hsa_circ_	0.296169	0.100298
hsa_circ_	0.857076	0.100299
hsa_circ_	1.078215	0.100309
hsa_circ_	-0.49306	0.100309
hsa_circ_	-0.45834	0.100332
hsa_circ_	-0.31517	0.100333
hsa_circ_	-0.46316	0.100337
hsa_circ_	-0.29974	0.100347
hsa_circ_	-0.87974	0.100347
hsa_circ_	0.271488	0.100352
hsa_circ_	-0.23722	0.10036
hsa_circ_	-0.30284	0.10038
hsa_circ_	0.456767	0.100383
hsa_circ_	-0.2311	0.100386
hsa_circ_	-0.52877	0.100387
hsa_circ_	0.478627	0.100387
hsa_circ_	-0.52393	0.100394
hsa_circ_	-0.29748	0.100398
hsa_circ_	0.430176	0.100399
hsa_circ_	-0.27921	0.100403
hsa_circ_	0.285852	0.100412
hsa_circ_	-0.23238	0.100419
hsa_circ_	-0.36457	0.100433
hsa_circ_	-1.33924	0.100439
hsa_circ_	0.677994	0.100441
hsa_circ_	-0.28425	0.100454
hsa_circ_	-0.47847	0.100457
hsa_circ_	-0.3219	0.100464
hsa_circ_	0.362523	0.100464
hsa_circ_	-0.40023	0.100468
hsa_circ_	-0.40553	0.100468
hsa_circ_	0.304121	0.100469
hsa_circ_	-0.5981	0.100472
hsa_circ_	0.300112	0.100474
hsa_circ_	0.714886	0.100474

hsa_circ_	-0.42305	0.100476
hsa_circ_	-0.2427	0.100478
hsa_circ_	-0.4429	0.100484
hsa_circ_	-0.53552	0.100485
hsa_circ_	-0.67958	0.100496
hsa_circ_	-0.30356	0.100504
hsa_circ_	-0.36031	0.100504
hsa_circ_	-0.71051	0.100508
hsa_circ_	-0.37387	0.100518
hsa_circ_	-0.55748	0.10052
hsa_circ_	0.390387	0.10052
hsa_circ_	0.42274	0.100521
hsa_circ_	-0.41609	0.100522
hsa_circ_	-0.7414	0.100522
hsa_circ_	-0.23734	0.100527
hsa_circ_	-0.38722	0.100533
hsa_circ_	-0.29669	0.100536
hsa_circ_	0.320772	0.100546
hsa_circ_	-0.43889	0.100548
hsa_circ_	0.264735	0.100562
hsa_circ_	-0.38432	0.100575
hsa_circ_	0.541457	0.100578
hsa_circ_	0.422452	0.100579
hsa_circ_	-0.41317	0.100601
hsa_circ_	-0.37308	0.100605
hsa_circ_	0.304908	0.100606
hsa_circ_	-0.24562	0.100608
hsa_circ_	0.512342	0.100618
hsa_circ_	-0.5693	0.100632
hsa_circ_	0.427518	0.100634
hsa_circ_	-0.3566	0.100636
hsa_circ_	-0.6731	0.10064
hsa_circ_	-0.81812	0.100642
hsa_circ_	0.299006	0.100647
hsa_circ_	0.436744	0.100651
hsa_circ_	0.746224	0.100666
hsa_circ_	-0.5434	0.100667
hsa_circ_	-0.97282	0.100681
hsa_circ_	0.329948	0.100685
hsa_circ_	0.297449	0.100687
hsa_circ_	0.477674	0.100692
hsa_circ_	0.471633	0.100699
hsa_circ_	0.29165	0.1007
hsa_circ_	0.37216	0.100705
hsa_circ_	-0.62159	0.100711
hsa_circ_	0.384577	0.100718
hsa_circ_	0.447239	0.100724
hsa_circ_	-0.34631	0.100734
hsa_circ_	-0.29391	0.100734
hsa_circ_	-0.28579	0.100735
hsa_circ_	-0.27674	0.100747
hsa_circ_	-0.32733	0.100747
hsa_circ_	-0.28061	0.100772
hsa_circ_	-0.43407	0.100788

hsa_circ_	0.244219	0.100788
hsa_circ_	0.277418	0.100791
hsa_circ_	0.34916	0.100799
hsa_circ_	-2.27017	0.100808
hsa_circ_	-0.29286	0.100808
hsa_circ_	0.593154	0.100808
hsa_circ_	0.40348	0.100817
hsa_circ_	-0.35376	0.100822
hsa_circ_	-1.31351	0.100823
hsa_circ_	0.378363	0.10083
hsa_circ_	0.919244	0.100832
hsa_circ_	0.351849	0.100843
hsa_circ_	-0.34524	0.100844
hsa_circ_	-0.48385	0.100849
hsa_circ_	0.241159	0.100851
hsa_circ_	0.386366	0.100852
hsa_circ_	0.420368	0.100856
hsa_circ_	-0.93311	0.100861
hsa_circ_	-0.31757	0.100862
hsa_circ_	-0.37452	0.100863
hsa_circ_	-0.30744	0.100863
hsa_circ_	-0.30081	0.100876
hsa_circ_	0.315996	0.100894
hsa_circ_	-0.47687	0.100902
hsa_circ_	-0.43782	0.100904
hsa_circ_	0.237455	0.100906
hsa_circ_	0.730189	0.100907
hsa_circ_	-0.36607	0.100911
hsa_circ_	1.099337	0.100912
hsa_circ_	-0.39875	0.100932
hsa_circ_	0.626976	0.100943
hsa_circ_	-0.81572	0.100949
hsa_circ_	0.302083	0.100952
hsa_circ_	-0.28267	0.100955
hsa_circ_	-0.34695	0.10096
hsa_circ_	-0.29092	0.100966
hsa_circ_	-0.2855	0.100978
hsa_circ_	0.687596	0.100978
hsa_circ_	-0.26093	0.100984
hsa_circ_	-0.71383	0.101008
hsa_circ_	0.299311	0.101009
hsa_circ_	-0.40515	0.101017
hsa_circ_	-0.324	0.101024
hsa_circ_	-0.30013	0.101036
hsa_circ_	0.232891	0.101047
hsa_circ_	-0.98951	0.101053
hsa_circ_	0.558452	0.101054
hsa_circ_	0.662502	0.101058
hsa_circ_	0.252992	0.101061
hsa_circ_	-0.25922	0.101068
hsa_circ_	-0.33473	0.101073
hsa_circ_	0.359944	0.101083
hsa_circ_	-0.42346	0.101093
hsa_circ_	0.26779	0.101094

hsa_circ_	-0.25622	0.101103
hsa_circ_	-0.29652	0.101106
hsa_circ_	-0.31719	0.101108
hsa_circ_	-0.36731	0.101115
hsa_circ_	0.531639	0.101138
hsa_circ_	0.441385	0.101161
hsa_circ_	0.598557	0.101167
hsa_circ_	-0.82892	0.101183
hsa_circ_	0.264268	0.101185
hsa_circ_	0.410503	0.101196
hsa_circ_	-1.20063	0.1012
hsa_circ_	0.419785	0.101202
hsa_circ_	0.410501	0.10121
hsa_circ_	-0.57541	0.101247
hsa_circ_	0.251981	0.101251
hsa_circ_	-0.32371	0.101255
hsa_circ_	0.37391	0.101258
hsa_circ_	0.32286	0.101262
hsa_circ_	0.422488	0.101266
hsa_circ_	0.265202	0.101267
hsa_circ_	-0.48889	0.101267
hsa_circ_	0.423489	0.10127
hsa_circ_	-0.25036	0.101276
hsa_circ_	0.486739	0.101292
hsa_circ_	0.314444	0.1013
hsa_circ_	0.481548	0.101316
hsa_circ_	0.287934	0.101328
hsa_circ_	1.18189	0.101332
hsa_circ_	0.256379	0.101346
hsa_circ_	-0.25275	0.101352
hsa_circ_	-0.35364	0.101367
hsa_circ_	-0.56431	0.101375
hsa_circ_	0.444843	0.101385
hsa_circ_	-0.30085	0.101387
hsa_circ_	0.548563	0.101388
hsa_circ_	0.340924	0.10139
hsa_circ_	-0.75258	0.101402
hsa_circ_	-0.45558	0.101407
hsa_circ_	0.900986	0.101421
hsa_circ_	-0.3089	0.101462
hsa_circ_	0.275346	0.101463
hsa_circ_	0.371167	0.101469
hsa_circ_	-0.32532	0.10147
hsa_circ_	-0.32977	0.101471
hsa_circ_	-0.51626	0.101472
hsa_circ_	-0.77764	0.10148
hsa_circ_	-0.2899	0.101489
hsa_circ_	0.320665	0.101496
hsa_circ_	-0.2886	0.101499
hsa_circ_	-0.43439	0.101507
hsa_circ_	0.3035	0.101507
hsa_circ_	0.362853	0.10152
hsa_circ_	-0.28003	0.101531
hsa_circ_	-0.31223	0.101532

hsa_circ_	-0.40402	0.101534
hsa_circ_	-0.41956	0.101537
hsa_circ_	0.236237	0.101538
hsa_circ_	-0.27139	0.101547
hsa_circ_	-0.36128	0.101564
hsa_circ_	-0.68774	0.101565
hsa_circ_	0.261566	0.101567
hsa_circ_	-0.30043	0.101582
hsa_circ_	-0.36082	0.101582
hsa_circ_	0.286067	0.101585
hsa_circ_	-0.56237	0.10159
hsa_circ_	0.534566	0.101592
hsa_circ_	0.559054	0.101593
hsa_circ_	-0.3812	0.101594
hsa_circ_	0.259537	0.1016
hsa_circ_	0.627744	0.101611
hsa_circ_	-0.31067	0.101612
hsa_circ_	-0.31608	0.101631
hsa_circ_	-0.43802	0.101651
hsa_circ_	-0.30553	0.101656
hsa_circ_	-0.3915	0.101661
hsa_circ_	-0.47004	0.101662
hsa_circ_	0.374315	0.101662
hsa_circ_	-0.66135	0.101673
hsa_circ_	0.35862	0.101676
hsa_circ_	0.666858	0.101681
hsa_circ_	0.355083	0.101689
hsa_circ_	0.2999	0.10169
hsa_circ_	-1.36069	0.10169
hsa_circ_	-0.35275	0.10171
hsa_circ_	0.611737	0.101712
hsa_circ_	-0.30221	0.101747
hsa_circ_	-0.33556	0.101755
hsa_circ_	-0.48768	0.101768
hsa_circ_	-0.33455	0.101781
hsa_circ_	0.450822	0.10179
hsa_circ_	-0.50435	0.101791
hsa_circ_	0.633898	0.101795
hsa_circ_	0.48062	0.101803
hsa_circ_	-0.37225	0.101805
hsa_circ_	-0.57559	0.101807
hsa_circ_	0.586105	0.101812
hsa_circ_	0.507554	0.101821
hsa_circ_	-0.54356	0.101822
hsa_circ_	0.429075	0.101828
hsa_circ_	1.351149	0.101831
hsa_circ_	-0.95748	0.101831
hsa_circ_	-0.26534	0.101834
hsa_circ_	-0.42425	0.101836
hsa_circ_	0.326394	0.101843
hsa_circ_	-0.31344	0.101848
hsa_circ_	0.433505	0.10185
hsa_circ_	-0.25383	0.101863
hsa_circ_	0.314101	0.101864

hsa_circ_	0.358792	0.101867
hsa_circ_	0.833193	0.10189
hsa_circ_	-0.25426	0.101896
hsa_circ_	-1.09898	0.101901
hsa_circ_	1.016459	0.101942
hsa_circ_	0.333271	0.101943
hsa_circ_	0.70251	0.101947
hsa_circ_	-0.26497	0.101965
hsa_circ_	0.303563	0.101968
hsa_circ_	-0.24979	0.101978
hsa_circ_	-0.30099	0.101986
hsa_circ_	0.517522	0.10199
hsa_circ_	-0.34507	0.101997
hsa_circ_	0.36145	0.101997
hsa_circ_	0.839519	0.102004
hsa_circ_	-0.6579	0.102011
hsa_circ_	-0.42981	0.102042
hsa_circ_	-0.51261	0.102061
hsa_circ_	0.444453	0.102074
hsa_circ_	-0.36234	0.102075
hsa_circ_	-0.40495	0.102101
hsa_circ_	-0.27439	0.102101
hsa_circ_	0.312391	0.102102
hsa_circ_	1.008247	0.102106
hsa_circ_	-0.94784	0.102116
hsa_circ_	-0.36493	0.102134
hsa_circ_	-0.31095	0.102139
hsa_circ_	0.274962	0.102143
hsa_circ_	-0.75555	0.102145
hsa_circ_	0.332603	0.102147
hsa_circ_	0.740944	0.102149
hsa_circ_	-0.32802	0.10215
hsa_circ_	-0.36007	0.102163
hsa_circ_	0.372792	0.102172
hsa_circ_	0.577463	0.102185
hsa_circ_	1.052049	0.102195
hsa_circ_	1.50551	0.102202
hsa_circ_	-0.2962	0.102218
hsa_circ_	0.681324	0.10223
hsa_circ_	-0.30565	0.102245
hsa_circ_	0.82668	0.102254
hsa_circ_	0.349216	0.10227
hsa_circ_	0.729918	0.102277
hsa_circ_	0.615418	0.102278
hsa_circ_	0.588967	0.102283
hsa_circ_	0.260207	0.102283
hsa_circ_	0.568542	0.102284
hsa_circ_	-0.27744	0.102285
hsa_circ_	-0.44332	0.102296
hsa_circ_	-0.25308	0.102318
hsa_circ_	0.295698	0.102325
hsa_circ_	0.388752	0.102325
hsa_circ_	1.07363	0.102327
hsa_circ_	0.263087	0.102328

hsa_circ_	0.375666	0.102336
hsa_circ_	-0.44625	0.102337
hsa_circ_	0.240136	0.102343
hsa_circ_	0.371656	0.102343
hsa_circ_	-1.02158	0.102344
hsa_circ_	-0.30665	0.102346
hsa_circ_	0.385849	0.102347
hsa_circ_	-0.32316	0.102353
hsa_circ_	-0.76578	0.102353
hsa_circ_	-0.26025	0.102359
hsa_circ_	0.381838	0.10236
hsa_circ_	-0.29612	0.102364
hsa_circ_	-0.55926	0.102367
hsa_circ_	0.435335	0.102368
hsa_circ_	-0.35529	0.102371
hsa_circ_	0.357405	0.102376
hsa_circ_	0.666062	0.102387
hsa_circ_	-0.49006	0.102387
hsa_circ_	-0.39242	0.102393
hsa_circ_	-1.15191	0.102398
hsa_circ_	-0.39281	0.102399
hsa_circ_	-0.3348	0.102425
hsa_circ_	-0.28741	0.102428
hsa_circ_	0.355024	0.102434
hsa_circ_	-0.25918	0.102444
hsa_circ_	-0.25726	0.102447
hsa_circ_	-0.34612	0.102448
hsa_circ_	-0.33086	0.10245
hsa_circ_	-0.35305	0.102477
hsa_circ_	0.469751	0.102478
hsa_circ_	1.25193	0.102483
hsa_circ_	-0.42231	0.102491
hsa_circ_	0.259498	0.102493
hsa_circ_	-0.29046	0.102495
hsa_circ_	0.413409	0.102504
hsa_circ_	0.40145	0.102508
hsa_circ_	-0.51694	0.102509
hsa_circ_	-0.32076	0.102519
hsa_circ_	-0.29523	0.10252
hsa_circ_	-0.96949	0.102526
hsa_circ_	0.371945	0.102529
hsa_circ_	0.361382	0.102533
hsa_circ_	0.965211	0.102537
hsa_circ_	-0.28585	0.102538
hsa_circ_	0.62218	0.102555
hsa_circ_	-0.23385	0.102555
hsa_circ_	-0.5871	0.102584
hsa_circ_	0.302037	0.102627
hsa_circ_	-0.59189	0.102636
hsa_circ_	-0.38827	0.102637
hsa_circ_	-0.2832	0.10264
hsa_circ_	-0.3497	0.102647
hsa_circ_	0.291957	0.10265
hsa_circ_	-0.5551	0.102654

hsa_circ_	-0.37793	0.102661
hsa_circ_	0.534922	0.102686
hsa_circ_	0.301452	0.102711
hsa_circ_	-0.25041	0.102714
hsa_circ_	0.278512	0.102717
hsa_circ_	1.098917	0.102738
hsa_circ_	0.371496	0.102752
hsa_circ_	1.008591	0.102755
hsa_circ_	-0.27346	0.102761
hsa_circ_	1.014702	0.102773
hsa_circ_	0.264403	0.102783
hsa_circ_	-0.26525	0.102797
hsa_circ_	-0.38004	0.102805
hsa_circ_	0.4303	0.102842
hsa_circ_	-0.46362	0.102844
hsa_circ_	0.37771	0.102855
hsa_circ_	0.345824	0.102859
hsa_circ_	0.296563	0.102864
hsa_circ_	-0.33771	0.102867
hsa_circ_	-0.32808	0.102873
hsa_circ_	0.489809	0.102876
hsa_circ_	-0.64055	0.102881
hsa_circ_	-0.36708	0.102884
hsa_circ_	0.336139	0.102885
hsa_circ_	-0.28286	0.102892
hsa_circ_	-0.946	0.102892
hsa_circ_	-1.05812	0.102896
hsa_circ_	0.316605	0.102902
hsa_circ_	0.866325	0.10291
hsa_circ_	-0.30038	0.102926
hsa_circ_	0.402801	0.102927
hsa_circ_	-0.23722	0.102946
hsa_circ_	-0.25711	0.102964
hsa_circ_	0.928455	0.102965
hsa_circ_	-0.27767	0.102966
hsa_circ_	0.318402	0.102984
hsa_circ_	0.689366	0.102989
hsa_circ_	0.256693	0.102994
hsa_circ_	-0.41252	0.103002
hsa_circ_	-0.24655	0.103003
hsa_circ_	0.300316	0.103005
hsa_circ_	0.31846	0.103006
hsa_circ_	-0.30798	0.103011
hsa_circ_	-0.2687	0.103021
hsa_circ_	-1.13335	0.103028
hsa_circ_	0.553607	0.103031
hsa_circ_	-0.28357	0.103037
hsa_circ_	0.340393	0.103051
hsa_circ_	0.321852	0.103064
hsa_circ_	-0.42201	0.103074
hsa_circ_	-0.29339	0.10308
hsa_circ_	-0.58203	0.103084
hsa_circ_	-0.33217	0.103105
hsa_circ_	-0.28618	0.103107

hsa_circ_	-0.33496	0.103113
hsa_circ_	0.574069	0.103132
hsa_circ_	-0.26575	0.103141
hsa_circ_	0.912798	0.103142
hsa_circ_	-0.56913	0.103151
hsa_circ_	-0.77932	0.103167
hsa_circ_	0.469675	0.10317
hsa_circ_	0.302792	0.10317
hsa_circ_	-0.481	0.103178
hsa_circ_	0.251125	0.103178
hsa_circ_	1.022231	0.103184
hsa_circ_	0.623487	0.103188
hsa_circ_	-0.37192	0.10319
hsa_circ_	-0.28167	0.103199
hsa_circ_	-0.4158	0.103213
hsa_circ_	0.537909	0.103217
hsa_circ_	-0.30891	0.103229
hsa_circ_	-0.42265	0.103231
hsa_circ_	0.368272	0.103236
hsa_circ_	0.530757	0.103237
hsa_circ_	-0.28007	0.103246
hsa_circ_	-0.23364	0.103261
hsa_circ_	0.306454	0.103264
hsa_circ_	0.345569	0.103266
hsa_circ_	0.271862	0.10327
hsa_circ_	-0.30823	0.103287
hsa_circ_	0.346174	0.10329
hsa_circ_	-0.75137	0.103294
hsa_circ_	-0.40378	0.103301
hsa_circ_	0.861922	0.103304
hsa_circ_	0.259318	0.103314
hsa_circ_	0.484159	0.103316
hsa_circ_	-0.36096	0.103319
hsa_circ_	0.358036	0.10334
hsa_circ_	-0.49427	0.103346
hsa_circ_	-0.48127	0.103347
hsa_circ_	0.335574	0.103348
hsa_circ_	0.502165	0.103367
hsa_circ_	1.220185	0.103379
hsa_circ_	-0.30661	0.103379
hsa_circ_	-0.41486	0.103387
hsa_circ_	-0.30313	0.103414
hsa_circ_	-0.43246	0.103415
hsa_circ_	-0.35706	0.103426
hsa_circ_	0.874153	0.103441
hsa_circ_	0.81482	0.10345
hsa_circ_	-0.33045	0.103452
hsa_circ_	-0.31678	0.103454
hsa_circ_	-0.35963	0.103454
hsa_circ_	0.344046	0.103494
hsa_circ_	1.17706	0.103495
hsa_circ_	-0.38095	0.1035
hsa_circ_	0.264771	0.1035
hsa_circ_	0.548724	0.103502

hsa_circ_	-0.29004	0.103512
hsa_circ_	0.584811	0.103521
hsa_circ_	-0.30669	0.103543
hsa_circ_	-0.46041	0.103545
hsa_circ_	0.374624	0.103548
hsa_circ_	-0.45601	0.103552
hsa_circ_	-0.4004	0.103553
hsa_circ_	0.76029	0.103554
hsa_circ_	-0.60453	0.103555
hsa_circ_	0.313462	0.103565
hsa_circ_	0.583647	0.103565
hsa_circ_	0.368824	0.103569
hsa_circ_	-0.86905	0.103574
hsa_circ_	0.321648	0.10358
hsa_circ_	0.293299	0.103587
hsa_circ_	-0.24964	0.103594
hsa_circ_	0.423057	0.103595
hsa_circ_	0.407778	0.103598
hsa_circ_	-0.4677	0.103604
hsa_circ_	0.441918	0.103628
hsa_circ_	0.360835	0.103629
hsa_circ_	-0.39572	0.103632
hsa_circ_	0.422944	0.103636
hsa_circ_	-0.25709	0.103636
hsa_circ_	0.455263	0.103641
hsa_circ_	-0.31942	0.103652
hsa_circ_	-0.55309	0.103661
hsa_circ_	0.310037	0.103678
hsa_circ_	1.190349	0.103686
hsa_circ_	-0.46608	0.103709
hsa_circ_	0.560851	0.103714
hsa_circ_	0.596193	0.103721
hsa_circ_	-0.31383	0.103732
hsa_circ_	0.4334	0.103734
hsa_circ_	-0.40709	0.103738
hsa_circ_	0.714175	0.103746
hsa_circ_	-0.29012	0.103751
hsa_circ_	-0.29039	0.103753
hsa_circ_	-0.71458	0.103764
hsa_circ_	0.417189	0.103783
hsa_circ_	0.375951	0.103794
hsa_circ_	0.376057	0.103797
hsa_circ_	-0.48376	0.103806
hsa_circ_	-0.51531	0.103808
hsa_circ_	0.449531	0.103813
hsa_circ_	-0.3936	0.103814
hsa_circ_	0.737715	0.103823
hsa_circ_	-0.95327	0.103844
hsa_circ_	-0.76755	0.103847
hsa_circ_	0.371781	0.103848
hsa_circ_	0.866137	0.103848
hsa_circ_	0.607015	0.103874
hsa_circ_	-0.52854	0.103875
hsa_circ_	-0.36399	0.103883

hsa_circ_	-0.33073	0.103889
hsa_circ_	-0.4703	0.103895
hsa_circ_	0.402743	0.103903
hsa_circ_	-0.34641	0.10392
hsa_circ_	0.434438	0.103924
hsa_circ_	0.624987	0.103929
hsa_circ_	0.274916	0.103942
hsa_circ_	-0.2378	0.103947
hsa_circ_	0.474658	0.103956
hsa_circ_	0.839501	0.103958
hsa_circ_	-0.30136	0.103972
hsa_circ_	-0.32834	0.103975
hsa_circ_	-0.56947	0.103988
hsa_circ_	0.295739	0.103992
hsa_circ_	-0.39496	0.103993
hsa_circ_	-0.27303	0.103994
hsa_circ_	-0.24439	0.104001
hsa_circ_	0.442705	0.104003
hsa_circ_	-0.66224	0.104005
hsa_circ_	-1.51063	0.104006
hsa_circ_	0.453084	0.104014
hsa_circ_	-0.27436	0.104019
hsa_circ_	0.682213	0.104034
hsa_circ_	0.489229	0.104035
hsa_circ_	-1.41298	0.104039
hsa_circ_	-0.47782	0.104048
hsa_circ_	0.350614	0.104051
hsa_circ_	0.340214	0.104073
hsa_circ_	-0.22814	0.104081
hsa_circ_	-0.24751	0.104087
hsa_circ_	1.266269	0.104091
hsa_circ_	-0.23761	0.104096
hsa_circ_	-0.36054	0.104096
hsa_circ_	0.383095	0.104106
hsa_circ_	1.07305	0.104107
hsa_circ_	-0.39957	0.104109
hsa_circ_	-0.33335	0.104125
hsa_circ_	-0.68227	0.104127
hsa_circ_	0.253241	0.10414
hsa_circ_	-0.27378	0.104142
hsa_circ_	0.251998	0.104144
hsa_circ_	-0.42763	0.104147
hsa_circ_	-1.26972	0.104152
hsa_circ_	0.294392	0.10416
hsa_circ_	-0.31397	0.104161
hsa_circ_	-0.37097	0.104168
hsa_circ_	0.43711	0.104174
hsa_circ_	0.369862	0.104179
hsa_circ_	0.33057	0.10418
hsa_circ_	0.590065	0.104181
hsa_circ_	-0.41133	0.104184
hsa_circ_	-0.28123	0.104184
hsa_circ_	-0.30544	0.1042
hsa_circ_	-0.55338	0.1042

hsa_circ_	-0.23455	0.104208
hsa_circ_	-0.28698	0.10421
hsa_circ_	-0.31479	0.104211
hsa_circ_	0.335707	0.104211
hsa_circ_	-0.28257	0.104218
hsa_circ_	-0.31509	0.104223
hsa_circ_	0.540952	0.104225
hsa_circ_	-0.39149	0.104237
hsa_circ_	-0.28793	0.104245
hsa_circ_	-0.66154	0.104249
hsa_circ_	0.250944	0.104251
hsa_circ_	0.510433	0.104259
hsa_circ_	-0.28876	0.104269
hsa_circ_	0.307289	0.104276
hsa_circ_	0.42373	0.104276
hsa_circ_	-0.44024	0.104312
hsa_circ_	-0.8074	0.104319
hsa_circ_	-0.37748	0.104331
hsa_circ_	-0.24667	0.104352
hsa_circ_	0.931149	0.104354
hsa_circ_	0.290259	0.104388
hsa_circ_	-0.35967	0.104393
hsa_circ_	-0.57688	0.104402
hsa_circ_	0.866595	0.104411
hsa_circ_	-0.47409	0.104413
hsa_circ_	-0.33088	0.104414
hsa_circ_	0.448612	0.104414
hsa_circ_	-0.3887	0.104422
hsa_circ_	0.291823	0.104425
hsa_circ_	0.348307	0.104444
hsa_circ_	-0.26732	0.104445
hsa_circ_	0.377406	0.104467
hsa_circ_	-0.35846	0.104471
hsa_circ_	0.54119	0.104474
hsa_circ_	-0.29125	0.104482
hsa_circ_	-0.58074	0.104489
hsa_circ_	0.25559	0.104491
hsa_circ_	-2.55749	0.104495
hsa_circ_	0.294408	0.104499
hsa_circ_	0.297433	0.104507
hsa_circ_	0.586448	0.104525
hsa_circ_	-0.28269	0.104525
hsa_circ_	0.307856	0.104528
hsa_circ_	0.270948	0.104566
hsa_circ_	-0.33701	0.104571
hsa_circ_	-0.9351	0.104579
hsa_circ_	-0.30592	0.10458
hsa_circ_	-0.26024	0.104581
hsa_circ_	-0.5105	0.104589
hsa_circ_	0.431775	0.104594
hsa_circ_	0.453793	0.104602
hsa_circ_	0.38879	0.104612
hsa_circ_	1.377431	0.104619
hsa_circ_	-0.29914	0.10462

hsa_circ_	-0.37943	0.10462
hsa_circ_	0.323656	0.104624
hsa_circ_	-0.25924	0.104632
hsa_circ_	0.479516	0.104644
hsa_circ_	-0.34559	0.104649
hsa_circ_	0.485614	0.104654
hsa_circ_	1.010545	0.104667
hsa_circ_	-0.23453	0.104679
hsa_circ_	0.411192	0.104688
hsa_circ_	-0.23368	0.104689
hsa_circ_	-0.23835	0.1047
hsa_circ_	0.417491	0.104702
hsa_circ_	-0.33413	0.104711
hsa_circ_	0.533329	0.104728
hsa_circ_	0.362256	0.104754
hsa_circ_	0.798544	0.104757
hsa_circ_	0.610432	0.104761
hsa_circ_	0.316574	0.104767
hsa_circ_	-0.99148	0.104778
hsa_circ_	0.62586	0.104788
hsa_circ_	1.163474	0.104802
hsa_circ_	-0.31012	0.104806
hsa_circ_	0.383843	0.104807
hsa_circ_	0.849673	0.104816
hsa_circ_	0.369173	0.10482
hsa_circ_	0.291731	0.104829
hsa_circ_	-0.45222	0.104833
hsa_circ_	-0.25139	0.104843
hsa_circ_	1.093194	0.104851
hsa_circ_	0.418185	0.104854
hsa_circ_	-0.47745	0.104861
hsa_circ_	0.288324	0.104873
hsa_circ_	-0.27395	0.104874
hsa_circ_	0.712363	0.104878
hsa_circ_	0.45858	0.104882
hsa_circ_	0.545738	0.104895
hsa_circ_	-0.44541	0.104908
hsa_circ_	-0.27626	0.104912
hsa_circ_	0.356394	0.104922
hsa_circ_	-0.24724	0.104925
hsa_circ_	-0.29197	0.104926
hsa_circ_	0.347828	0.104933
hsa_circ_	-0.2812	0.104939
hsa_circ_	-0.28359	0.104943
hsa_circ_	-0.76355	0.104944
hsa_circ_	0.353605	0.104945
hsa_circ_	-0.30528	0.104946
hsa_circ_	-0.52224	0.104959
hsa_circ_	0.595036	0.104963
hsa_circ_	0.26036	0.104968
hsa_circ_	0.24525	0.104972
hsa_circ_	0.379582	0.104981
hsa_circ_	0.37908	0.104983
hsa_circ_	0.347635	0.104994

hsa_circ_	-0.24821	0.104994
hsa_circ_	-0.25643	0.104997
hsa_circ_	0.273755	0.105025
hsa_circ_	0.775544	0.105045
hsa_circ_	-0.28469	0.105066
hsa_circ_	-0.98817	0.105069
hsa_circ_	0.567759	0.105072
hsa_circ_	-0.53714	0.10509
hsa_circ_	-0.38707	0.105114
hsa_circ_	0.99503	0.105118
hsa_circ_	0.289864	0.105131
hsa_circ_	0.654299	0.105135
hsa_circ_	0.321448	0.105138
hsa_circ_	-0.62142	0.105151
hsa_circ_	-0.2402	0.105154
hsa_circ_	0.458805	0.105174
hsa_circ_	0.489283	0.105177
hsa_circ_	0.409705	0.10518
hsa_circ_	-0.31134	0.105203
hsa_circ_	-0.3416	0.105207
hsa_circ_	-0.39942	0.105211
hsa_circ_	-0.28052	0.105211
hsa_circ_	0.578657	0.105217
hsa_circ_	0.276384	0.105247
hsa_circ_	-0.53448	0.105258
hsa_circ_	0.538014	0.105268
hsa_circ_	0.641111	0.105277
hsa_circ_	0.423398	0.105302
hsa_circ_	-0.45075	0.105306
hsa_circ_	0.421821	0.105311
hsa_circ_	0.393592	0.105316
hsa_circ_	0.40752	0.105318
hsa_circ_	0.60082	0.105329
hsa_circ_	0.33212	0.105344
hsa_circ_	-0.24462	0.10537
hsa_circ_	0.228083	0.105371
hsa_circ_	-0.33961	0.105373
hsa_circ_	0.260716	0.105383
hsa_circ_	-0.60433	0.105387
hsa_circ_	0.338206	0.105392
hsa_circ_	-0.36688	0.105395
hsa_circ_	0.552812	0.105397
hsa_circ_	0.239339	0.105406
hsa_circ_	-0.3321	0.105431
hsa_circ_	-0.35087	0.105434
hsa_circ_	0.513114	0.105436
hsa_circ_	-0.65192	0.105453
hsa_circ_	0.29788	0.105454
hsa_circ_	0.270746	0.105476
hsa_circ_	-0.40594	0.105487
hsa_circ_	-0.78743	0.105489
hsa_circ_	-0.2793	0.1055
hsa_circ_	-0.33021	0.105528
hsa_circ_	-0.32716	0.105536

hsa_circ_	-0.46119	0.105538
hsa_circ_	-0.42188	0.105555
hsa_circ_	-0.25754	0.105558
hsa_circ_	-0.38521	0.105561
hsa_circ_	0.477233	0.105564
hsa_circ_	0.409003	0.105571
hsa_circ_	-0.23384	0.105575
hsa_circ_	0.364442	0.105587
hsa_circ_	-0.36661	0.1056
hsa_circ_	-0.32134	0.1056
hsa_circ_	0.960521	0.105606
hsa_circ_	0.57748	0.105613
hsa_circ_	0.226332	0.105617
hsa_circ_	0.625372	0.105627
hsa_circ_	0.267884	0.105662
hsa_circ_	0.391485	0.105666
hsa_circ_	-0.63927	0.105678
hsa_circ_	0.435817	0.105685
hsa_circ_	0.295995	0.105701
hsa_circ_	-0.38561	0.105724
hsa_circ_	0.335601	0.10573
hsa_circ_	-0.54198	0.105733
hsa_circ_	-0.24027	0.105733
hsa_circ_	0.320183	0.105734
hsa_circ_	-0.2443	0.105735
hsa_circ_	0.825104	0.105747
hsa_circ_	-0.22871	0.105752
hsa_circ_	0.274091	0.105755
hsa_circ_	1.080359	0.105758
hsa_circ_	0.311437	0.105763
hsa_circ_	-0.40959	0.105764
hsa_circ_	0.433406	0.105764
hsa_circ_	0.46729	0.105766
hsa_circ_	-0.25835	0.105767
hsa_circ_	-0.38878	0.105775
hsa_circ_	0.438956	0.105781
hsa_circ_	0.385698	0.105794
hsa_circ_	-0.3215	0.105801
hsa_circ_	-1.17156	0.105814
hsa_circ_	-0.56263	0.105817
hsa_circ_	0.320316	0.105818
hsa_circ_	-0.48959	0.105819
hsa_circ_	-0.7301	0.105821
hsa_circ_	0.662305	0.105823
hsa_circ_	-0.55139	0.105832
hsa_circ_	-0.52325	0.105852
hsa_circ_	0.420669	0.105862
hsa_circ_	0.33426	0.105889
hsa_circ_	0.303889	0.105891
hsa_circ_	-0.3355	0.105893
hsa_circ_	-0.64255	0.105918
hsa_circ_	-0.24927	0.105927
hsa_circ_	0.582826	0.105933
hsa_circ_	0.293924	0.105936

hsa_circ_	0.446866	0.105944
hsa_circ_	-0.46032	0.105952
hsa_circ_	-0.28171	0.105962
hsa_circ_	0.350266	0.105963
hsa_circ_	-0.27014	0.105967
hsa_circ_	0.42035	0.10597
hsa_circ_	-0.50498	0.105993
hsa_circ_	1.080106	0.106008
hsa_circ_	0.342223	0.106039
hsa_circ_	0.357742	0.106056
hsa_circ_	0.304381	0.106065
hsa_circ_	0.322179	0.106073
hsa_circ_	0.340226	0.106073
hsa_circ_	0.299472	0.106074
hsa_circ_	-0.64223	0.106078
hsa_circ_	0.807335	0.106081
hsa_circ_	0.429747	0.106085
hsa_circ_	-0.45784	0.106101
hsa_circ_	-0.30502	0.106128
hsa_circ_	0.379955	0.10613
hsa_circ_	0.247515	0.106131
hsa_circ_	0.486621	0.106135
hsa_circ_	0.240813	0.106135
hsa_circ_	0.419014	0.106136
hsa_circ_	-0.58936	0.106139
hsa_circ_	-0.59557	0.106141
hsa_circ_	-0.22886	0.106143
hsa_circ_	0.340243	0.106151
hsa_circ_	1.195692	0.106157
hsa_circ_	0.282372	0.106162
hsa_circ_	1.066615	0.106163
hsa_circ_	1.165037	0.106167
hsa_circ_	0.414077	0.106174
hsa_circ_	0.362866	0.106185
hsa_circ_	-0.33278	0.106192
hsa_circ_	0.354885	0.1062
hsa_circ_	-0.24619	0.106209
hsa_circ_	0.303234	0.10621
hsa_circ_	-0.41005	0.10624
hsa_circ_	0.445889	0.106247
hsa_circ_	-0.30935	0.106247
hsa_circ_	-0.33573	0.106248
hsa_circ_	0.802025	0.10626
hsa_circ_	0.455691	0.106267
hsa_circ_	0.58307	0.106272
hsa_circ_	1.225336	0.106272
hsa_circ_	0.574302	0.106286
hsa_circ_	0.581679	0.106296
hsa_circ_	0.24145	0.1063
hsa_circ_	-0.3903	0.106322
hsa_circ_	-1.10088	0.106326
hsa_circ_	-0.3227	0.106327
hsa_circ_	-0.26638	0.10636
hsa_circ_	0.291317	0.106362

hsa_circ_	0.279437	0.106363
hsa_circ_	-0.3651	0.106366
hsa_circ_	-0.47886	0.106377
hsa_circ_	0.359344	0.106378
hsa_circ_	0.379654	0.106378
hsa_circ_	0.280155	0.10639
hsa_circ_	0.273587	0.106408
hsa_circ_	0.488584	0.10641
hsa_circ_	-0.25364	0.106416
hsa_circ_	-0.34394	0.106423
hsa_circ_	-0.43849	0.106427
hsa_circ_	0.403203	0.106435
hsa_circ_	-0.71774	0.106439
hsa_circ_	0.304958	0.106456
hsa_circ_	-0.3919	0.106459
hsa_circ_	-1.12361	0.10647
hsa_circ_	1.205153	0.106484
hsa_circ_	0.381277	0.106495
hsa_circ_	0.649974	0.1065
hsa_circ_	0.446822	0.106504
hsa_circ_	-0.26541	0.106508
hsa_circ_	0.794304	0.10652
hsa_circ_	0.273881	0.106525
hsa_circ_	-0.29314	0.106529
hsa_circ_	-0.39697	0.106554
hsa_circ_	-0.61538	0.106558
hsa_circ_	-1.2593	0.106562
hsa_circ_	0.879733	0.106566
hsa_circ_	0.2698	0.106578
hsa_circ_	-0.36635	0.106579
hsa_circ_	-0.41348	0.106581
hsa_circ_	-0.31907	0.106582
hsa_circ_	-0.22774	0.106594
hsa_circ_	-0.29614	0.106607
hsa_circ_	-0.44297	0.106623
hsa_circ_	-0.66726	0.106623
hsa_circ_	-0.4451	0.106625
hsa_circ_	0.445742	0.106627
hsa_circ_	0.32044	0.106629
hsa_circ_	-1.39726	0.106632
hsa_circ_	-0.34149	0.106634
hsa_circ_	0.866517	0.106645
hsa_circ_	0.291813	0.106647
hsa_circ_	-0.32698	0.106653
hsa_circ_	0.292472	0.106654
hsa_circ_	0.822807	0.106659
hsa_circ_	0.662937	0.106666
hsa_circ_	-0.29469	0.106671
hsa_circ_	0.350235	0.106673
hsa_circ_	0.422651	0.106681
hsa_circ_	0.747869	0.106685
hsa_circ_	-0.55059	0.106691
hsa_circ_	-0.65981	0.106695
hsa_circ_	-0.33573	0.106704

hsa_circ_	0.343369	0.106704
hsa_circ_	-1.41456	0.106706
hsa_circ_	-0.29894	0.106709
hsa_circ_	0.496833	0.106711
hsa_circ_	0.330928	0.106711
hsa_circ_	-0.84273	0.106725
hsa_circ_	0.6101	0.106729
hsa_circ_	-0.54092	0.106732
hsa_circ_	-0.44405	0.106734
hsa_circ_	0.71365	0.106735
hsa_circ_	0.411044	0.106739
hsa_circ_	-0.46813	0.106741
hsa_circ_	0.386184	0.106742
hsa_circ_	1.083521	0.106743
hsa_circ_	-0.35853	0.106746
hsa_circ_	0.783785	0.106749
hsa_circ_	0.248269	0.106752
hsa_circ_	-0.23799	0.106753
hsa_circ_	-0.4808	0.106754
hsa_circ_	0.386692	0.106756
hsa_circ_	-0.62822	0.106764
hsa_circ_	-0.55472	0.106765
hsa_circ_	0.665725	0.106771
hsa_circ_	-0.89572	0.106783
hsa_circ_	-0.49443	0.106796
hsa_circ_	-0.24484	0.106808
hsa_circ_	1.254574	0.106822
hsa_circ_	-0.51151	0.106826
hsa_circ_	-0.3129	0.106829
hsa_circ_	0.511289	0.106832
hsa_circ_	0.235649	0.106835
hsa_circ_	0.366936	0.106855
hsa_circ_	0.46442	0.106856
hsa_circ_	-0.37422	0.106866
hsa_circ_	-0.48041	0.106866
hsa_circ_	0.434273	0.106867
hsa_circ_	0.273303	0.106867
hsa_circ_	0.771404	0.106869
hsa_circ_	-0.33416	0.10687
hsa_circ_	-0.63547	0.106876
hsa_circ_	-0.33315	0.106893
hsa_circ_	-0.2311	0.106893
hsa_circ_	0.31601	0.106893
hsa_circ_	-0.33571	0.106894
hsa_circ_	0.259521	0.106895
hsa_circ_	0.386822	0.106908
hsa_circ_	-0.24572	0.106913
hsa_circ_	0.730684	0.106942
hsa_circ_	-0.72312	0.106946
hsa_circ_	0.364532	0.106964
hsa_circ_	-0.28937	0.106972
hsa_circ_	0.2759	0.106976
hsa_circ_	0.332478	0.10698
hsa_circ_	0.282111	0.106981

hsa_circ_	0.319668	0.106982
hsa_circ_	-0.44212	0.106985
hsa_circ_	-0.5115	0.106988
hsa_circ_	1.049706	0.10699
hsa_circ_	-0.73502	0.106995
hsa_circ_	0.481243	0.107008
hsa_circ_	-0.5515	0.107008
hsa_circ_	0.228549	0.107013
hsa_circ_	-0.48857	0.107029
hsa_circ_	-0.29872	0.107029
hsa_circ_	0.448354	0.10703
hsa_circ_	0.246708	0.107045
hsa_circ_	0.275332	0.107059
hsa_circ_	-0.69231	0.107063
hsa_circ_	0.558419	0.107065
hsa_circ_	-0.29779	0.107066
hsa_circ_	-0.62836	0.107073
hsa_circ_	-0.36356	0.107084
hsa_circ_	0.277831	0.107087
hsa_circ_	0.355758	0.1071
hsa_circ_	-0.2483	0.107102
hsa_circ_	-0.63611	0.107103
hsa_circ_	-0.38355	0.107117
hsa_circ_	0.415806	0.107118
hsa_circ_	-0.30775	0.107119
hsa_circ_	0.548623	0.10712
hsa_circ_	0.304367	0.107121
hsa_circ_	-0.69897	0.107133
hsa_circ_	0.500756	0.107162
hsa_circ_	-0.29225	0.107162
hsa_circ_	-0.26636	0.107167
hsa_circ_	0.393593	0.107176
hsa_circ_	0.27636	0.10718
hsa_circ_	-0.23782	0.107191
hsa_circ_	0.296047	0.107201
hsa_circ_	0.246646	0.107203
hsa_circ_	0.336036	0.107209
hsa_circ_	0.54946	0.107209
hsa_circ_	0.467538	0.107223
hsa_circ_	0.987165	0.107225
hsa_circ_	-0.31016	0.10723
hsa_circ_	-0.40859	0.107231
hsa_circ_	0.338865	0.107252
hsa_circ_	1.069268	0.107254
hsa_circ_	0.50601	0.107261
hsa_circ_	-1.01195	0.10727
hsa_circ_	-0.41978	0.107285
hsa_circ_	0.746217	0.107292
hsa_circ_	0.306475	0.107309
hsa_circ_	-0.22782	0.107331
hsa_circ_	-0.32295	0.107338
hsa_circ_	-0.25144	0.10735
hsa_circ_	0.267467	0.107364
hsa_circ_	0.663278	0.107372

hsa_circ_	-0.43798	0.107375
hsa_circ_	-0.26296	0.107384
hsa_circ_	0.47489	0.107389
hsa_circ_	-0.71269	0.107395
hsa_circ_	-0.2682	0.107398
hsa_circ_	-0.87434	0.107402
hsa_circ_	0.425727	0.107403
hsa_circ_	-0.42234	0.107412
hsa_circ_	-0.63672	0.107421
hsa_circ_	0.263756	0.107435
hsa_circ_	0.479029	0.107445
hsa_circ_	-0.53595	0.107452
hsa_circ_	-0.4978	0.107455
hsa_circ_	0.256119	0.107456
hsa_circ_	-0.39569	0.107458
hsa_circ_	0.324066	0.107463
hsa_circ_	-0.62853	0.107464
hsa_circ_	-0.33926	0.107473
hsa_circ_	0.429848	0.107478
hsa_circ_	-0.87559	0.107506
hsa_circ_	-0.29198	0.107508
hsa_circ_	-0.23606	0.107509
hsa_circ_	-0.30278	0.107517
hsa_circ_	0.567087	0.107525
hsa_circ_	-0.41249	0.107528
hsa_circ_	-0.27851	0.107535
hsa_circ_	-0.26108	0.10756
hsa_circ_	0.369418	0.107563
hsa_circ_	-0.67312	0.107567
hsa_circ_	0.341169	0.107567
hsa_circ_	-0.35805	0.107572
hsa_circ_	1.314322	0.107574
hsa_circ_	-0.40206	0.107574
hsa_circ_	-0.34824	0.107574
hsa_circ_	-0.23997	0.107585
hsa_circ_	-0.26353	0.107588
hsa_circ_	0.715773	0.107593
hsa_circ_	-0.30941	0.107593
hsa_circ_	0.414338	0.107594
hsa_circ_	0.318332	0.1076
hsa_circ_	-0.35388	0.107614
hsa_circ_	-0.25413	0.107615
hsa_circ_	-0.80278	0.107635
hsa_circ_	0.396648	0.107638
hsa_circ_	0.414034	0.107643
hsa_circ_	-0.23517	0.107647
hsa_circ_	0.330349	0.10765
hsa_circ_	0.573566	0.107656
hsa_circ_	0.338982	0.107682
hsa_circ_	-0.27205	0.107701
hsa_circ_	0.526237	0.107708
hsa_circ_	0.34077	0.10771
hsa_circ_	-0.4497	0.107712
hsa_circ_	-0.23929	0.107735

hsa_circ_	-0.44821	0.107746
hsa_circ_	-0.33269	0.107765
hsa_circ_	0.637744	0.107771
hsa_circ_	0.405412	0.107775
hsa_circ_	-0.44749	0.107791
hsa_circ_	-0.29217	0.107819
hsa_circ_	0.265219	0.107829
hsa_circ_	-0.24413	0.107859
hsa_circ_	-0.30653	0.107864
hsa_circ_	0.489051	0.107872
hsa_circ_	-0.33754	0.107873
hsa_circ_	-0.55911	0.107909
hsa_circ_	-1.49147	0.107913
hsa_circ_	-1.40922	0.107914
hsa_circ_	-0.41555	0.107917
hsa_circ_	-0.41872	0.107919
hsa_circ_	-0.28537	0.107937
hsa_circ_	-0.27368	0.107947
hsa_circ_	0.411331	0.107947
hsa_circ_	-1.38677	0.107949
hsa_circ_	-0.62363	0.107951
hsa_circ_	-0.91681	0.107953
hsa_circ_	0.479032	0.107964
hsa_circ_	0.702269	0.107992
hsa_circ_	0.430113	0.108
hsa_circ_	-0.26809	0.108016
hsa_circ_	0.75042	0.108019
hsa_circ_	0.436073	0.108026
hsa_circ_	0.526205	0.10803
hsa_circ_	-0.31274	0.108036
hsa_circ_	0.26958	0.108041
hsa_circ_	0.503083	0.108059
hsa_circ_	0.365406	0.108068
hsa_circ_	-1.07483	0.108071
hsa_circ_	-0.55748	0.108081
hsa_circ_	0.488561	0.108094
hsa_circ_	0.300995	0.1081
hsa_circ_	0.4571	0.108101
hsa_circ_	0.695015	0.108102
hsa_circ_	-0.28785	0.108111
hsa_circ_	0.907927	0.108113
hsa_circ_	0.304424	0.108133
hsa_circ_	0.332491	0.108135
hsa_circ_	-0.25301	0.108145
hsa_circ_	0.240759	0.108147
hsa_circ_	0.405769	0.108162
hsa_circ_	0.471649	0.108172
hsa_circ_	0.483274	0.108178
hsa_circ_	0.333183	0.108188
hsa_circ_	0.321811	0.108193
hsa_circ_	-0.27634	0.108195
hsa_circ_	-0.28971	0.108215
hsa_circ_	-0.3113	0.108216
hsa_circ_	-0.41588	0.10822

hsa_circ_	-0.5515	0.108223
hsa_circ_	-0.28442	0.108226
hsa_circ_	0.288182	0.108233
hsa_circ_	0.506568	0.108257
hsa_circ_	0.460097	0.108259
hsa_circ_	-0.24599	0.108266
hsa_circ_	0.468114	0.108292
hsa_circ_	-0.86784	0.108294
hsa_circ_	-0.34154	0.108302
hsa_circ_	0.443677	0.108312
hsa_circ_	0.80887	0.108313
hsa_circ_	-0.45654	0.108314
hsa_circ_	-0.34483	0.108322
hsa_circ_	-0.24723	0.108326
hsa_circ_	-0.82124	0.108348
hsa_circ_	-0.45794	0.10836
hsa_circ_	0.64022	0.108368
hsa_circ_	-0.24343	0.108384
hsa_circ_	-0.349	0.108407
hsa_circ_	1.024745	0.108415
hsa_circ_	0.424877	0.108423
hsa_circ_	-0.26887	0.108437
hsa_circ_	0.461554	0.108443
hsa_circ_	-0.30164	0.10845
hsa_circ_	-0.30279	0.108461
hsa_circ_	0.847614	0.108464
hsa_circ_	-0.36759	0.108471
hsa_circ_	0.486594	0.108493
hsa_circ_	-0.79944	0.108496
hsa_circ_	0.428888	0.108502
hsa_circ_	-0.30962	0.108504
hsa_circ_	-0.63882	0.108506
hsa_circ_	0.746572	0.108507
hsa_circ_	-0.43654	0.108512
hsa_circ_	-0.3315	0.108515
hsa_circ_	-0.24468	0.108528
hsa_circ_	0.267904	0.108531
hsa_circ_	-0.48138	0.108548
hsa_circ_	0.242918	0.108549
hsa_circ_	-0.39506	0.108559
hsa_circ_	-1.17067	0.108564
hsa_circ_	-0.35463	0.108591
hsa_circ_	-0.26365	0.108592
hsa_circ_	-0.24597	0.108601
hsa_circ_	0.633719	0.108603
hsa_circ_	0.591453	0.108603
hsa_circ_	0.299152	0.108604
hsa_circ_	0.551349	0.108608
hsa_circ_	-0.44065	0.108616
hsa_circ_	0.276168	0.108616
hsa_circ_	-0.26847	0.108617
hsa_circ_	-0.63711	0.108635
hsa_circ_	-0.25148	0.108636
hsa_circ_	-0.41868	0.108641

hsa_circ_	-1.17872	0.108643
hsa_circ_	-0.42077	0.108669
hsa_circ_	-0.22692	0.108687
hsa_circ_	0.402079	0.108688
hsa_circ_	-0.56881	0.108689
hsa_circ_	0.547182	0.108704
hsa_circ_	0.32798	0.108704
hsa_circ_	-0.70573	0.10872
hsa_circ_	0.81967	0.108735
hsa_circ_	-0.46006	0.108743
hsa_circ_	-0.65301	0.108748
hsa_circ_	-0.27862	0.108755
hsa_circ_	0.282867	0.10876
hsa_circ_	0.566945	0.108769
hsa_circ_	0.274186	0.108774
hsa_circ_	-0.50875	0.108774
hsa_circ_	0.340565	0.108788
hsa_circ_	0.476221	0.108796
hsa_circ_	0.503217	0.108809
hsa_circ_	0.43778	0.108814
hsa_circ_	-0.44244	0.108816
hsa_circ_	-0.38066	0.108821
hsa_circ_	0.310469	0.108823
hsa_circ_	-0.33275	0.108837
hsa_circ_	-0.43988	0.108857
hsa_circ_	-0.38267	0.108868
hsa_circ_	0.845578	0.108871
hsa_circ_	-0.46185	0.108873
hsa_circ_	0.874079	0.108877
hsa_circ_	0.322569	0.108881
hsa_circ_	-0.24711	0.108897
hsa_circ_	-0.55102	0.108898
hsa_circ_	-0.29766	0.1089
hsa_circ_	-0.36261	0.108903
hsa_circ_	0.341971	0.108922
hsa_circ_	-0.50965	0.108923
hsa_circ_	0.773647	0.10893
hsa_circ_	0.750532	0.108934
hsa_circ_	-0.66827	0.108937
hsa_circ_	0.339686	0.108941
hsa_circ_	-0.30576	0.108944
hsa_circ_	0.280828	0.108945
hsa_circ_	-0.31951	0.108947
hsa_circ_	-0.29681	0.108951
hsa_circ_	-0.4505	0.108951
hsa_circ_	-0.26348	0.108952
hsa_circ_	0.362099	0.108955
hsa_circ_	-0.25206	0.108957
hsa_circ_	-0.34331	0.108962
hsa_circ_	0.232175	0.108983
hsa_circ_	0.396411	0.109
hsa_circ_	0.26332	0.109016
hsa_circ_	-0.30973	0.109016
hsa_circ_	0.460937	0.109031

hsa_circ_	-0.29858	0.109034
hsa_circ_	-1.53361	0.109062
hsa_circ_	0.320956	0.109073
hsa_circ_	0.309892	0.109082
hsa_circ_	-0.48619	0.109095
hsa_circ_	0.882429	0.109099
hsa_circ_	-0.27404	0.109127
hsa_circ_	0.410945	0.10914
hsa_circ_	-0.37264	0.109152
hsa_circ_	0.336269	0.10916
hsa_circ_	-0.31501	0.109178
hsa_circ_	0.436063	0.109184
hsa_circ_	0.706969	0.109185
hsa_circ_	0.362115	0.109186
hsa_circ_	0.258073	0.109192
hsa_circ_	-0.96234	0.109212
hsa_circ_	-0.84266	0.109214
hsa_circ_	-0.3743	0.109217
hsa_circ_	-0.28698	0.109218
hsa_circ_	0.569575	0.109237
hsa_circ_	-0.30997	0.109243
hsa_circ_	-0.62681	0.10925
hsa_circ_	-0.61018	0.109258
hsa_circ_	-0.69828	0.109266
hsa_circ_	0.34069	0.109269
hsa_circ_	0.407657	0.109272
hsa_circ_	0.229142	0.109291
hsa_circ_	0.740288	0.109297
hsa_circ_	-0.33521	0.109297
hsa_circ_	0.242304	0.109311
hsa_circ_	0.402808	0.109314
hsa_circ_	0.350245	0.109317
hsa_circ_	0.41645	0.109326
hsa_circ_	0.816179	0.109335
hsa_circ_	0.405144	0.109355
hsa_circ_	-0.24619	0.109366
hsa_circ_	-0.45365	0.109386
hsa_circ_	-0.6882	0.109387
hsa_circ_	0.875216	0.109404
hsa_circ_	0.468296	0.109405
hsa_circ_	-0.40954	0.109414
hsa_circ_	0.291773	0.109418
hsa_circ_	-0.37066	0.109419
hsa_circ_	0.233647	0.109421
hsa_circ_	-0.30307	0.109431
hsa_circ_	0.726068	0.109434
hsa_circ_	0.726284	0.109438
hsa_circ_	0.293391	0.109442
hsa_circ_	1.225472	0.109445
hsa_circ_	0.343086	0.109447
hsa_circ_	-0.30867	0.109452
hsa_circ_	0.29765	0.109466
hsa_circ_	-0.27684	0.109467
hsa_circ_	0.350795	0.109473

hsa_circ_	-0.24451	0.109488
hsa_circ_	-0.43628	0.10949
hsa_circ_	0.533	0.109504
hsa_circ_	-0.33313	0.109514
hsa_circ_	0.319351	0.109526
hsa_circ_	0.402877	0.109536
hsa_circ_	-0.365	0.109556
hsa_circ_	0.337168	0.109571
hsa_circ_	0.294624	0.109575
hsa_circ_	-0.30696	0.10958
hsa_circ_	0.313308	0.109601
hsa_circ_	0.27179	0.109617
hsa_circ_	-0.47031	0.109629
hsa_circ_	-0.36399	0.109658
hsa_circ_	-0.58576	0.109662
hsa_circ_	0.775265	0.109669
hsa_circ_	0.74492	0.109679
hsa_circ_	1.172416	0.109683
hsa_circ_	-1.28427	0.109685
hsa_circ_	-0.39912	0.109693
hsa_circ_	-0.45927	0.109695
hsa_circ_	0.515187	0.1097
hsa_circ_	-0.89895	0.109703
hsa_circ_	0.258162	0.109713
hsa_circ_	-0.26635	0.109717
hsa_circ_	-0.8104	0.109721
hsa_circ_	-0.2466	0.109729
hsa_circ_	0.30731	0.10973
hsa_circ_	-0.26098	0.109737
hsa_circ_	0.366695	0.109742
hsa_circ_	-0.24288	0.109743
hsa_circ_	-0.30464	0.109748
hsa_circ_	0.407614	0.109754
hsa_circ_	-0.74449	0.109771
hsa_circ_	-0.32235	0.109771
hsa_circ_	-0.29034	0.109784
hsa_circ_	0.720938	0.109797
hsa_circ_	-0.46586	0.109803
hsa_circ_	-0.34326	0.109806
hsa_circ_	-0.24113	0.109806
hsa_circ_	-0.24351	0.109818
hsa_circ_	0.261245	0.109824
hsa_circ_	0.356574	0.109824
hsa_circ_	-0.29554	0.109846
hsa_circ_	0.452206	0.109849
hsa_circ_	0.245842	0.109856
hsa_circ_	0.290063	0.109856
hsa_circ_	-0.35091	0.109858
hsa_circ_	-0.37908	0.109858
hsa_circ_	-0.22603	0.109863
hsa_circ_	-0.2495	0.109867
hsa_circ_	-0.50007	0.109885
hsa_circ_	-0.26829	0.109887
hsa_circ_	0.675302	0.109891

hsa_circ_	-0.30383	0.109907
hsa_circ_	0.479776	0.109912
hsa_circ_	0.570824	0.109926
hsa_circ_	-0.34523	0.109978
hsa_circ_	-0.24548	0.109981
hsa_circ_	0.310925	0.109989
hsa_circ_	0.317012	0.10999
hsa_circ_	0.289689	0.110002
hsa_circ_	0.271265	0.110004
hsa_circ_	-0.31006	0.110054
hsa_circ_	0.953161	0.110063
hsa_circ_	-0.55278	0.110064
hsa_circ_	-0.4429	0.110079
hsa_circ_	0.88167	0.11008
hsa_circ_	-1.0081	0.110086
hsa_circ_	-0.66124	0.110088
hsa_circ_	-0.30734	0.110094
hsa_circ_	-0.39193	0.1101
hsa_circ_	-0.37628	0.110101
hsa_circ_	-0.87828	0.110107
hsa_circ_	-0.36033	0.11013
hsa_circ_	0.278326	0.110133
hsa_circ_	-0.26754	0.110143
hsa_circ_	0.418864	0.110156
hsa_circ_	-0.29801	0.110157
hsa_circ_	-0.39014	0.110168
hsa_circ_	-0.411	0.110177
hsa_circ_	-0.2949	0.110185
hsa_circ_	0.742529	0.110195
hsa_circ_	-0.23753	0.110217
hsa_circ_	-0.48969	0.110218
hsa_circ_	-0.26384	0.110219
hsa_circ_	0.354739	0.110238
hsa_circ_	-0.29625	0.110246
hsa_circ_	0.411605	0.110253
hsa_circ_	-0.36731	0.110265
hsa_circ_	-0.41599	0.110267
hsa_circ_	-0.56013	0.110275
hsa_circ_	0.70124	0.110279
hsa_circ_	-0.57858	0.110285
hsa_circ_	0.286121	0.110288
hsa_circ_	0.96014	0.110289
hsa_circ_	-0.47071	0.110289
hsa_circ_	0.647434	0.110302
hsa_circ_	-0.35058	0.110302
hsa_circ_	0.46595	0.110313
hsa_circ_	0.33075	0.110338
hsa_circ_	0.613305	0.110342
hsa_circ_	-0.26969	0.110346
hsa_circ_	-0.35813	0.110352
hsa_circ_	-0.22808	0.110362
hsa_circ_	-0.58749	0.110372
hsa_circ_	-0.38999	0.110376
hsa_circ_	0.283629	0.110387

hsa_circ_	1.072456	0.110406
hsa_circ_	-0.30099	0.110413
hsa_circ_	0.249834	0.110437
hsa_circ_	-0.46206	0.11045
hsa_circ_	-0.31982	0.110454
hsa_circ_	0.279211	0.110457
hsa_circ_	-0.46903	0.110464
hsa_circ_	-0.23376	0.110466
hsa_circ_	-0.3836	0.11047
hsa_circ_	-0.31118	0.110471
hsa_circ_	-0.4264	0.11049
hsa_circ_	0.601554	0.110493
hsa_circ_	0.435584	0.110497
hsa_circ_	0.228526	0.11051
hsa_circ_	-0.29225	0.110516
hsa_circ_	-0.28343	0.110562
hsa_circ_	0.269811	0.110569
hsa_circ_	-0.25594	0.110571
hsa_circ_	-0.26815	0.110576
hsa_circ_	-0.47546	0.110585
hsa_circ_	-0.39614	0.110587
hsa_circ_	-1.0576	0.110603
hsa_circ_	0.354863	0.110611
hsa_circ_	0.24701	0.110616
hsa_circ_	-0.53189	0.110617
hsa_circ_	-0.23216	0.110622
hsa_circ_	0.290607	0.110626
hsa_circ_	0.798658	0.110638
hsa_circ_	0.292554	0.110646
hsa_circ_	-0.42562	0.110667
hsa_circ_	-0.30047	0.110668
hsa_circ_	0.351839	0.110671
hsa_circ_	0.395036	0.110693
hsa_circ_	-0.48248	0.110704
hsa_circ_	-0.76226	0.110706
hsa_circ_	-0.31315	0.110713
hsa_circ_	0.492799	0.110721
hsa_circ_	-0.42676	0.110727
hsa_circ_	0.453702	0.110749
hsa_circ_	-0.64984	0.11075
hsa_circ_	-0.28276	0.110752
hsa_circ_	0.380222	0.110756
hsa_circ_	0.884029	0.110757
hsa_circ_	0.320271	0.110757
hsa_circ_	-0.26544	0.110757
hsa_circ_	-0.35704	0.110768
hsa_circ_	0.267838	0.110768
hsa_circ_	-0.45788	0.110773
hsa_circ_	0.393168	0.110774
hsa_circ_	0.414479	0.110783
hsa_circ_	0.662213	0.110786
hsa_circ_	0.485416	0.11079
hsa_circ_	0.342422	0.110809
hsa_circ_	-0.30161	0.110818

hsa_circ_	0.528819	0.110819
hsa_circ_	0.34461	0.110829
hsa_circ_	-0.54395	0.11084
hsa_circ_	-0.29956	0.110847
hsa_circ_	-0.59575	0.110855
hsa_circ_	0.285018	0.110862
hsa_circ_	0.235218	0.110887
hsa_circ_	0.391009	0.110891
hsa_circ_	-0.72013	0.110894
hsa_circ_	-0.44641	0.110898
hsa_circ_	0.28707	0.1109
hsa_circ_	-0.27548	0.110905
hsa_circ_	0.376408	0.110905
hsa_circ_	-1.28002	0.11092
hsa_circ_	-0.27847	0.110929
hsa_circ_	-1.71172	0.110959
hsa_circ_	-0.38117	0.110964
hsa_circ_	0.367361	0.110985
hsa_circ_	-0.58481	0.110999
hsa_circ_	-0.59478	0.111039
hsa_circ_	0.260855	0.111077
hsa_circ_	0.369243	0.11108
hsa_circ_	-0.37268	0.111083
hsa_circ_	0.243506	0.111084
hsa_circ_	-0.43113	0.111111
hsa_circ_	-1.35453	0.111113
hsa_circ_	-0.72921	0.111117
hsa_circ_	0.431284	0.111148
hsa_circ_	-0.3765	0.111164
hsa_circ_	0.26283	0.111171
hsa_circ_	-0.24985	0.111189
hsa_circ_	-0.66699	0.11119
hsa_circ_	-0.33311	0.111193
hsa_circ_	0.875837	0.111195
hsa_circ_	-0.24109	0.111199
hsa_circ_	0.820152	0.111206
hsa_circ_	-0.44016	0.111219
hsa_circ_	-0.22699	0.11122
hsa_circ_	-0.64087	0.111221
hsa_circ_	0.656899	0.111231
hsa_circ_	0.328954	0.111233
hsa_circ_	-0.39176	0.111256
hsa_circ_	0.338898	0.11126
hsa_circ_	-0.51893	0.111269
hsa_circ_	-0.93963	0.111277
hsa_circ_	0.53297	0.111292
hsa_circ_	-0.25659	0.1113
hsa_circ_	-0.52589	0.111305
hsa_circ_	-0.39133	0.111316
hsa_circ_	0.375242	0.111339
hsa_circ_	2.161118	0.11134
hsa_circ_	-0.32498	0.11134
hsa_circ_	0.297289	0.111346
hsa_circ_	0.654488	0.111354

hsa_circ_	-0.34363	0.111365
hsa_circ_	-0.24553	0.111369
hsa_circ_	-0.46673	0.11137
hsa_circ_	-0.34091	0.111371
hsa_circ_	-0.49101	0.111373
hsa_circ_	0.946212	0.111374
hsa_circ_	1.13534	0.111375
hsa_circ_	-0.4356	0.111378
hsa_circ_	0.311168	0.111391
hsa_circ_	-0.68998	0.111394
hsa_circ_	-0.27237	0.111397
hsa_circ_	0.493532	0.111401
hsa_circ_	0.490984	0.11142
hsa_circ_	-0.56247	0.111425
hsa_circ_	0.341777	0.111433
hsa_circ_	0.54238	0.111435
hsa_circ_	-0.27856	0.111445
hsa_circ_	-0.30598	0.111472
hsa_circ_	-0.22919	0.111494
hsa_circ_	-0.30156	0.111515
hsa_circ_	0.269647	0.111518
hsa_circ_	-0.78824	0.111522
hsa_circ_	-0.66298	0.111536
hsa_circ_	-0.26993	0.111538
hsa_circ_	-0.34932	0.111542
hsa_circ_	0.425053	0.111543
hsa_circ_	-0.22884	0.111559
hsa_circ_	-0.26931	0.111561
hsa_circ_	-0.80294	0.111564
hsa_circ_	-0.65376	0.111581
hsa_circ_	0.411931	0.111583
hsa_circ_	0.427235	0.111587
hsa_circ_	-0.282	0.111589
hsa_circ_	-0.25869	0.11159
hsa_circ_	-0.26193	0.111594
hsa_circ_	-0.57265	0.111604
hsa_circ_	-0.38708	0.111614
hsa_circ_	-0.27036	0.111619
hsa_circ_	0.395611	0.111625
hsa_circ_	-0.40514	0.111626
hsa_circ_	0.263839	0.111627
hsa_circ_	0.383181	0.111633
hsa_circ_	0.355664	0.111671
hsa_circ_	-0.23842	0.111677
hsa_circ_	-0.2527	0.111689
hsa_circ_	-0.24121	0.111696
hsa_circ_	0.649631	0.111703
hsa_circ_	-0.28673	0.111716
hsa_circ_	0.34252	0.111727
hsa_circ_	-0.70615	0.11173
hsa_circ_	-0.22353	0.111756
hsa_circ_	0.33557	0.111757
hsa_circ_	0.634379	0.11176
hsa_circ_	0.516339	0.111773

hsa_circ_	-0.66165	0.111778
hsa_circ_	0.510784	0.111808
hsa_circ_	-0.38011	0.111816
hsa_circ_	-0.26204	0.111818
hsa_circ_	0.349147	0.111828
hsa_circ_	1.097488	0.111846
hsa_circ_	-0.31467	0.111848
hsa_circ_	0.339963	0.111852
hsa_circ_	-0.24409	0.111858
hsa_circ_	-0.33453	0.11186
hsa_circ_	0.394029	0.111862
hsa_circ_	0.403485	0.111862
hsa_circ_	1.19014	0.111863
hsa_circ_	-0.27245	0.111865
hsa_circ_	-0.24429	0.111871
hsa_circ_	-0.26131	0.111872
hsa_circ_	-0.46886	0.111872
hsa_circ_	0.380147	0.111875
hsa_circ_	-0.61697	0.111875
hsa_circ_	0.296592	0.111881
hsa_circ_	0.276977	0.11189
hsa_circ_	0.316402	0.111899
hsa_circ_	-0.3282	0.1119
hsa_circ_	1.033825	0.111901
hsa_circ_	0.324562	0.111906
hsa_circ_	-0.4337	0.111915
hsa_circ_	0.43789	0.111919
hsa_circ_	0.2912	0.111927
hsa_circ_	-0.26014	0.111928
hsa_circ_	0.36761	0.11193
hsa_circ_	-0.85162	0.111933
hsa_circ_	0.371637	0.111936
hsa_circ_	-0.51006	0.111941
hsa_circ_	0.343927	0.111949
hsa_circ_	-0.37316	0.111993
hsa_circ_	0.404904	0.112013
hsa_circ_	0.611576	0.112024
hsa_circ_	-0.26305	0.112046
hsa_circ_	-0.27021	0.112047
hsa_circ_	-0.45347	0.112048
hsa_circ_	0.376447	0.11205
hsa_circ_	0.406477	0.11206
hsa_circ_	-0.27318	0.112061
hsa_circ_	-0.27889	0.112062
hsa_circ_	0.484103	0.112077
hsa_circ_	-0.63274	0.112081
hsa_circ_	-0.41288	0.112085
hsa_circ_	0.406405	0.112093
hsa_circ_	0.819191	0.112095
hsa_circ_	0.405032	0.112101
hsa_circ_	-0.5952	0.112108
hsa_circ_	0.546389	0.11212
hsa_circ_	0.398062	0.11213
hsa_circ_	-0.25585	0.112131

hsa_circ_	0.395494	0.112155
hsa_circ_	-0.34511	0.112159
hsa_circ_	-0.70439	0.112172
hsa_circ_	0.526592	0.112175
hsa_circ_	0.360318	0.112176
hsa_circ_	-0.29737	0.112185
hsa_circ_	0.32315	0.112198
hsa_circ_	0.331864	0.112198
hsa_circ_	-0.24118	0.1122
hsa_circ_	-0.34027	0.112204
hsa_circ_	1.099631	0.112208
hsa_circ_	0.448476	0.112212
hsa_circ_	0.354522	0.11223
hsa_circ_	0.899967	0.112243
hsa_circ_	-0.36762	0.11225
hsa_circ_	0.282327	0.112252
hsa_circ_	-0.32919	0.112261
hsa_circ_	0.320494	0.112268
hsa_circ_	0.43752	0.11228
hsa_circ_	0.653934	0.112283
hsa_circ_	-0.31598	0.112285
hsa_circ_	0.605139	0.112289
hsa_circ_	-0.91152	0.112296
hsa_circ_	-0.87269	0.112299
hsa_circ_	0.660717	0.112302
hsa_circ_	0.278133	0.112303
hsa_circ_	-0.25024	0.112315
hsa_circ_	0.361586	0.112327
hsa_circ_	-0.40339	0.112334
hsa_circ_	-0.29699	0.112355
hsa_circ_	0.293135	0.112361
hsa_circ_	0.370799	0.112361
hsa_circ_	0.285329	0.112371
hsa_circ_	0.296156	0.112374
hsa_circ_	0.493503	0.112376
hsa_circ_	-0.94332	0.112385
hsa_circ_	0.820681	0.1124
hsa_circ_	-0.36294	0.112405
hsa_circ_	-0.28638	0.112412
hsa_circ_	-0.26199	0.112413
hsa_circ_	0.31292	0.112418
hsa_circ_	0.424604	0.112441
hsa_circ_	0.754231	0.112461
hsa_circ_	0.482691	0.112463
hsa_circ_	0.519631	0.112479
hsa_circ_	-2.83343	0.112484
hsa_circ_	-0.3854	0.112484
hsa_circ_	-1.29752	0.112485
hsa_circ_	-0.90287	0.112489
hsa_circ_	0.533968	0.112495
hsa_circ_	-0.62242	0.112497
hsa_circ_	-0.48698	0.112503
hsa_circ_	0.382701	0.112518
hsa_circ_	0.67913	0.112523

hsa_circ_	-0.32112	0.112533
hsa_circ_	0.362837	0.112538
hsa_circ_	0.427536	0.112542
hsa_circ_	0.392151	0.112554
hsa_circ_	-0.43943	0.112556
hsa_circ_	0.669694	0.112559
hsa_circ_	-0.27462	0.112565
hsa_circ_	0.48697	0.112578
hsa_circ_	0.543907	0.112587
hsa_circ_	0.266391	0.112588
hsa_circ_	-0.29299	0.112591
hsa_circ_	0.931498	0.112594
hsa_circ_	-0.25523	0.112604
hsa_circ_	-0.46808	0.112622
hsa_circ_	0.277346	0.112629
hsa_circ_	-0.43662	0.112639
hsa_circ_	-0.24683	0.112644
hsa_circ_	0.274764	0.112649
hsa_circ_	-0.43695	0.112652
hsa_circ_	-0.29828	0.112665
hsa_circ_	0.597406	0.112667
hsa_circ_	-0.46009	0.112667
hsa_circ_	0.344462	0.112669
hsa_circ_	-0.30605	0.112683
hsa_circ_	-0.24972	0.112695
hsa_circ_	0.29303	0.112697
hsa_circ_	0.473627	0.112707
hsa_circ_	0.964029	0.112711
hsa_circ_	0.296841	0.112718
hsa_circ_	-0.57152	0.112723
hsa_circ_	0.447654	0.112729
hsa_circ_	0.52291	0.112738
hsa_circ_	-0.3059	0.112741
hsa_circ_	-0.38327	0.112746
hsa_circ_	-0.66418	0.112762
hsa_circ_	1.035842	0.112764
hsa_circ_	-0.26992	0.112778
hsa_circ_	-0.24109	0.112797
hsa_circ_	0.300835	0.1128
hsa_circ_	-0.33785	0.112802
hsa_circ_	0.561108	0.112807
hsa_circ_	0.407893	0.11282
hsa_circ_	-0.34984	0.112825
hsa_circ_	0.35191	0.112861
hsa_circ_	-0.82947	0.112865
hsa_circ_	-0.37155	0.112871
hsa_circ_	0.317935	0.112888
hsa_circ_	-0.28329	0.11289
hsa_circ_	0.267295	0.112906
hsa_circ_	-0.44152	0.112916
hsa_circ_	0.344213	0.112937
hsa_circ_	-1.06917	0.112943
hsa_circ_	0.298622	0.112947
hsa_circ_	0.293365	0.112951

hsa_circ_	-0.32409	0.112995
hsa_circ_	0.51289	0.112998
hsa_circ_	0.454828	0.113005
hsa_circ_	-0.34516	0.113006
hsa_circ_	0.413487	0.113006
hsa_circ_	0.529945	0.113011
hsa_circ_	-0.24954	0.113015
hsa_circ_	-0.25113	0.113027
hsa_circ_	-0.31503	0.113039
hsa_circ_	-0.35893	0.113046
hsa_circ_	0.259855	0.113049
hsa_circ_	0.462977	0.113052
hsa_circ_	0.228829	0.113054
hsa_circ_	-0.38311	0.113074
hsa_circ_	0.432633	0.113104
hsa_circ_	0.544784	0.113114
hsa_circ_	0.301872	0.113122
hsa_circ_	0.810977	0.113128
hsa_circ_	0.302277	0.113149
hsa_circ_	-0.63447	0.113152
hsa_circ_	-0.34397	0.113168
hsa_circ_	-0.24657	0.113169
hsa_circ_	0.264816	0.11317
hsa_circ_	0.44087	0.113173
hsa_circ_	0.485773	0.113184
hsa_circ_	-0.56422	0.113191
hsa_circ_	-0.26342	0.113191
hsa_circ_	0.68189	0.113221
hsa_circ_	0.484707	0.11324
hsa_circ_	-0.25772	0.113245
hsa_circ_	-0.49939	0.113246
hsa_circ_	-0.38507	0.113266
hsa_circ_	-0.26452	0.113291
hsa_circ_	0.366473	0.113298
hsa_circ_	0.460026	0.113303
hsa_circ_	-0.47818	0.113325
hsa_circ_	-0.68698	0.113346
hsa_circ_	-0.416	0.11335
hsa_circ_	-0.51953	0.113351
hsa_circ_	0.304201	0.113367
hsa_circ_	0.271866	0.113369
hsa_circ_	-0.48269	0.113375
hsa_circ_	0.241083	0.113376
hsa_circ_	0.38	0.11338
hsa_circ_	0.416345	0.113386
hsa_circ_	-0.39482	0.113398
hsa_circ_	0.343717	0.113405
hsa_circ_	0.356761	0.113429
hsa_circ_	0.395388	0.113436
hsa_circ_	0.680101	0.113437
hsa_circ_	-0.67714	0.11346
hsa_circ_	0.370838	0.113461
hsa_circ_	-0.46602	0.113467
hsa_circ_	0.355954	0.113481

hsa_circ_	0.353307	0.113483
hsa_circ_	0.303791	0.113486
hsa_circ_	-0.25262	0.113487
hsa_circ_	0.416824	0.113503
hsa_circ_	0.341499	0.113511
hsa_circ_	0.235959	0.113525
hsa_circ_	-0.31361	0.113527
hsa_circ_	0.560432	0.113531
hsa_circ_	-0.29691	0.113542
hsa_circ_	0.533127	0.113551
hsa_circ_	0.248087	0.113557
hsa_circ_	-0.61753	0.113558
hsa_circ_	-0.64489	0.113561
hsa_circ_	0.507806	0.113569
hsa_circ_	1.145963	0.113569
hsa_circ_	0.332419	0.113577
hsa_circ_	-0.28448	0.113585
hsa_circ_	0.381281	0.113605
hsa_circ_	0.32243	0.113607
hsa_circ_	-0.30095	0.113622
hsa_circ_	0.462592	0.113625
hsa_circ_	0.354385	0.113627
hsa_circ_	0.267155	0.113627
hsa_circ_	-0.33737	0.11363
hsa_circ_	-0.25579	0.113646
hsa_circ_	-0.402	0.113677
hsa_circ_	0.286081	0.113687
hsa_circ_	-0.61436	0.1137
hsa_circ_	-0.50223	0.113703
hsa_circ_	0.679446	0.11371
hsa_circ_	-1.00638	0.113716
hsa_circ_	-0.32097	0.113731
hsa_circ_	0.744611	0.113733
hsa_circ_	0.457307	0.113736
hsa_circ_	-0.28584	0.113755
hsa_circ_	-0.44901	0.113755
hsa_circ_	-0.24168	0.113764
hsa_circ_	-0.55766	0.113765
hsa_circ_	-0.89877	0.11377
hsa_circ_	-0.30919	0.113775
hsa_circ_	-0.54575	0.113777
hsa_circ_	0.314422	0.113785
hsa_circ_	0.253321	0.113785
hsa_circ_	0.458502	0.113793
hsa_circ_	-0.264	0.113795
hsa_circ_	-0.25244	0.113805
hsa_circ_	-0.42254	0.113815
hsa_circ_	-0.38629	0.113825
hsa_circ_	0.291941	0.113827
hsa_circ_	-0.87337	0.113831
hsa_circ_	0.273367	0.113846
hsa_circ_	-0.47465	0.113854
hsa_circ_	0.874271	0.113859
hsa_circ_	-0.23719	0.113869

hsa_circ_	-0.25283	0.113871
hsa_circ_	0.399686	0.113872
hsa_circ_	0.403524	0.113875
hsa_circ_	-0.65502	0.113886
hsa_circ_	-0.41448	0.113924
hsa_circ_	-0.705	0.113935
hsa_circ_	0.297342	0.113937
hsa_circ_	-0.25869	0.113949
hsa_circ_	-0.67986	0.11395
hsa_circ_	0.328711	0.113959
hsa_circ_	-0.38524	0.113968
hsa_circ_	-0.77978	0.113983
hsa_circ_	0.62196	0.113994
hsa_circ_	-0.99621	0.114002
hsa_circ_	0.225699	0.114005
hsa_circ_	-0.72177	0.114025
hsa_circ_	-0.37218	0.114028
hsa_circ_	0.453423	0.11404
hsa_circ_	0.259761	0.114053
hsa_circ_	0.490736	0.114061
hsa_circ_	-0.28628	0.114071
hsa_circ_	-0.54475	0.114072
hsa_circ_	0.475991	0.114081
hsa_circ_	-1.02732	0.11409
hsa_circ_	0.326815	0.114099
hsa_circ_	-0.54814	0.114099
hsa_circ_	0.327604	0.1141
hsa_circ_	0.345387	0.114104
hsa_circ_	-0.49498	0.114113
hsa_circ_	0.31443	0.114123
hsa_circ_	0.257775	0.114125
hsa_circ_	-0.85657	0.114126
hsa_circ_	-0.23093	0.11413
hsa_circ_	0.402935	0.114155
hsa_circ_	0.403869	0.114159
hsa_circ_	0.527516	0.114161
hsa_circ_	-0.24047	0.114167
hsa_circ_	0.330623	0.11418
hsa_circ_	0.319036	0.114185
hsa_circ_	0.326924	0.114193
hsa_circ_	0.863644	0.114209
hsa_circ_	-0.25435	0.11421
hsa_circ_	-0.62027	0.114212
hsa_circ_	0.301829	0.114213
hsa_circ_	-0.2504	0.114229
hsa_circ_	0.441043	0.114229
hsa_circ_	0.580863	0.114251
hsa_circ_	0.329049	0.114251
hsa_circ_	0.287247	0.114259
hsa_circ_	-0.41768	0.114271
hsa_circ_	-0.31348	0.114278
hsa_circ_	0.29684	0.114289
hsa_circ_	-0.26093	0.114291
hsa_circ_	0.309747	0.114302

hsa_circ_	-0.24703	0.114306
hsa_circ_	0.762764	0.114309
hsa_circ_	0.232847	0.114315
hsa_circ_	0.688927	0.114347
hsa_circ_	-0.63275	0.114352
hsa_circ_	-0.25728	0.11436
hsa_circ_	0.282565	0.114367
hsa_circ_	0.383784	0.114368
hsa_circ_	0.227708	0.114378
hsa_circ_	0.357156	0.114384
hsa_circ_	0.225711	0.114397
hsa_circ_	-0.34607	0.11441
hsa_circ_	-0.31083	0.114412
hsa_circ_	-0.38686	0.114416
hsa_circ_	-0.41561	0.114436
hsa_circ_	-0.35797	0.114452
hsa_circ_	-0.27363	0.114454
hsa_circ_	-0.28086	0.114456
hsa_circ_	-1.22227	0.114478
hsa_circ_	-0.37108	0.114478
hsa_circ_	-0.75904	0.114479
hsa_circ_	-0.35383	0.114482
hsa_circ_	0.337539	0.114505
hsa_circ_	-1.39704	0.114511
hsa_circ_	0.359019	0.114516
hsa_circ_	-1.39147	0.114525
hsa_circ_	-0.35415	0.114526
hsa_circ_	-0.26572	0.114534
hsa_circ_	-0.24711	0.114536
hsa_circ_	-0.53412	0.114538
hsa_circ_	0.357291	0.114541
hsa_circ_	0.354863	0.114547
hsa_circ_	-0.27878	0.114562
hsa_circ_	0.359517	0.11457
hsa_circ_	0.614514	0.114575
hsa_circ_	-0.44742	0.114577
hsa_circ_	-0.52313	0.114588
hsa_circ_	-0.30141	0.114596
hsa_circ_	-0.25051	0.114599
hsa_circ_	-0.2662	0.114611
hsa_circ_	-1.00418	0.114613
hsa_circ_	-0.8291	0.11462
hsa_circ_	-0.24921	0.114631
hsa_circ_	0.415014	0.114637
hsa_circ_	0.446065	0.114642
hsa_circ_	-0.50598	0.114646
hsa_circ_	-0.6989	0.114649
hsa_circ_	-0.22972	0.114664
hsa_circ_	-0.27341	0.114667
hsa_circ_	0.576894	0.114674
hsa_circ_	-1.58046	0.114682
hsa_circ_	0.287686	0.114685
hsa_circ_	0.292806	0.114686
hsa_circ_	1.00223	0.114689

hsa_circ_	-0.36888	0.114694
hsa_circ_	-0.7849	0.114695
hsa_circ_	-0.24478	0.114698
hsa_circ_	-0.35598	0.1147
hsa_circ_	-0.83885	0.114706
hsa_circ_	0.361357	0.114711
hsa_circ_	-0.48205	0.114719
hsa_circ_	0.380782	0.11473
hsa_circ_	0.559048	0.114732
hsa_circ_	-0.58712	0.114734
hsa_circ_	0.352495	0.114746
hsa_circ_	0.363383	0.114747
hsa_circ_	0.370799	0.114748
hsa_circ_	0.630907	0.114769
hsa_circ_	0.85914	0.114775
hsa_circ_	0.240163	0.114775
hsa_circ_	-0.22965	0.114778
hsa_circ_	0.420326	0.11478
hsa_circ_	0.701181	0.114784
hsa_circ_	0.338204	0.114785
hsa_circ_	-0.95989	0.114796
hsa_circ_	-0.276	0.114804
hsa_circ_	-0.41072	0.114807
hsa_circ_	-0.29669	0.114817
hsa_circ_	-0.43239	0.114819
hsa_circ_	0.320612	0.11482
hsa_circ_	-0.39556	0.114822
hsa_circ_	-0.3273	0.114835
hsa_circ_	-0.41328	0.114851
hsa_circ_	-0.27582	0.114853
hsa_circ_	0.258866	0.114853
hsa_circ_	0.258846	0.114858
hsa_circ_	1.033241	0.114889
hsa_circ_	-0.33707	0.114891
hsa_circ_	-0.27091	0.114917
hsa_circ_	-0.31615	0.114925
hsa_circ_	0.445925	0.114933
hsa_circ_	0.329715	0.114946
hsa_circ_	0.308251	0.114949
hsa_circ_	0.263708	0.114949
hsa_circ_	-0.22457	0.11495
hsa_circ_	-0.94952	0.11496
hsa_circ_	0.282954	0.114972
hsa_circ_	-0.28698	0.114972
hsa_circ_	-1.17839	0.114973
hsa_circ_	-0.36225	0.114981
hsa_circ_	-0.31563	0.114982
hsa_circ_	0.457406	0.114984
hsa_circ_	-0.47085	0.114984
hsa_circ_	0.351812	0.11499
hsa_circ_	0.34772	0.114995
hsa_circ_	0.463149	0.115003
hsa_circ_	-0.30069	0.115028
hsa_circ_	-0.28697	0.11503

hsa_circ_	0.314723	0.115032
hsa_circ_	0.376484	0.115035
hsa_circ_	0.429829	0.115055
hsa_circ_	-0.37075	0.115057
hsa_circ_	-0.64263	0.115058
hsa_circ_	1.093209	0.115073
hsa_circ_	-0.35265	0.115084
hsa_circ_	0.283048	0.115094
hsa_circ_	-0.22444	0.115095
hsa_circ_	-0.39102	0.115098
hsa_circ_	-0.53938	0.1151
hsa_circ_	-0.39359	0.115103
hsa_circ_	-0.75459	0.11512
hsa_circ_	0.634679	0.115121
hsa_circ_	-0.58149	0.115122
hsa_circ_	-0.30148	0.115164
hsa_circ_	0.248684	0.115167
hsa_circ_	0.333045	0.11517
hsa_circ_	0.318563	0.115183
hsa_circ_	-0.2336	0.115189
hsa_circ_	-0.22138	0.115191
hsa_circ_	-0.36717	0.115193
hsa_circ_	0.237911	0.115218
hsa_circ_	-0.62408	0.115227
hsa_circ_	-0.39555	0.115237
hsa_circ_	-0.68721	0.115246
hsa_circ_	0.472604	0.115249
hsa_circ_	0.259638	0.115266
hsa_circ_	-0.65681	0.11527
hsa_circ_	0.258231	0.115272
hsa_circ_	-0.31251	0.115274
hsa_circ_	0.656939	0.115276
hsa_circ_	-0.27012	0.115277
hsa_circ_	0.998857	0.115278
hsa_circ_	-0.31945	0.115282
hsa_circ_	0.465869	0.115285
hsa_circ_	-0.52573	0.115296
hsa_circ_	0.387582	0.115299
hsa_circ_	-0.28306	0.115319
hsa_circ_	-0.26371	0.115321
hsa_circ_	-0.26808	0.115328
hsa_circ_	-0.27469	0.115332
hsa_circ_	-0.51448	0.115335
hsa_circ_	-0.28714	0.115335
hsa_circ_	-0.88604	0.115341
hsa_circ_	0.304922	0.115345
hsa_circ_	0.618913	0.115348
hsa_circ_	0.710796	0.115349
hsa_circ_	-0.59444	0.115352
hsa_circ_	0.352172	0.115355
hsa_circ_	0.383328	0.115365
hsa_circ_	-0.24768	0.11537
hsa_circ_	0.570018	0.115378
hsa_circ_	0.251555	0.115391

hsa_circ_	-0.29787	0.115415
hsa_circ_	0.776703	0.115427
hsa_circ_	-0.32105	0.115434
hsa_circ_	-1.93284	0.115434
hsa_circ_	-0.67171	0.115436
hsa_circ_	-0.38419	0.115438
hsa_circ_	0.572404	0.115439
hsa_circ_	0.281809	0.115449
hsa_circ_	0.531977	0.115453
hsa_circ_	0.293333	0.115457
hsa_circ_	-0.26711	0.115459
hsa_circ_	-0.28854	0.115474
hsa_circ_	-0.30401	0.11548
hsa_circ_	-0.54849	0.115483
hsa_circ_	0.669488	0.115511
hsa_circ_	0.897019	0.115519
hsa_circ_	0.422708	0.115534
hsa_circ_	-0.36299	0.115535
hsa_circ_	0.257263	0.115543
hsa_circ_	-0.38126	0.115547
hsa_circ_	0.416578	0.115577
hsa_circ_	0.39823	0.115591
hsa_circ_	-1.49529	0.115591
hsa_circ_	-0.25548	0.115596
hsa_circ_	-0.25324	0.115625
hsa_circ_	-0.28784	0.115642
hsa_circ_	1.079599	0.115644
hsa_circ_	0.42403	0.115645
hsa_circ_	-0.24812	0.115649
hsa_circ_	-0.24484	0.115651
hsa_circ_	-0.75937	0.115658
hsa_circ_	0.335934	0.115668
hsa_circ_	0.339987	0.115674
hsa_circ_	1.046471	0.115675
hsa_circ_	0.617132	0.115676
hsa_circ_	0.3068	0.115678
hsa_circ_	0.344532	0.115683
hsa_circ_	-0.41602	0.115687
hsa_circ_	-0.39445	0.115696
hsa_circ_	0.403669	0.115701
hsa_circ_	0.263844	0.11571
hsa_circ_	-0.26855	0.115711
hsa_circ_	-0.33071	0.115724
hsa_circ_	-0.26381	0.115729
hsa_circ_	-0.4295	0.115733
hsa_circ_	-0.33034	0.115744
hsa_circ_	-0.23887	0.115758
hsa_circ_	-0.54267	0.11578
hsa_circ_	-0.3995	0.115789
hsa_circ_	0.333585	0.115801
hsa_circ_	-0.31079	0.115809
hsa_circ_	-1.06111	0.115809
hsa_circ_	0.270767	0.115814
hsa_circ_	0.419815	0.115819

hsa_circ_	0.265832	0.115828
hsa_circ_	0.316795	0.11583
hsa_circ_	-0.299	0.115836
hsa_circ_	-0.3126	0.115839
hsa_circ_	-0.2809	0.115842
hsa_circ_	0.360748	0.115844
hsa_circ_	0.466159	0.115855
hsa_circ_	-2.21768	0.115871
hsa_circ_	0.335267	0.115872
hsa_circ_	-0.35061	0.115873
hsa_circ_	-0.333	0.115899
hsa_circ_	-0.27631	0.115903
hsa_circ_	-0.26816	0.115912
hsa_circ_	0.337968	0.115917
hsa_circ_	0.32869	0.115922
hsa_circ_	0.428003	0.115927
hsa_circ_	0.430957	0.115927
hsa_circ_	0.918406	0.115932
hsa_circ_	0.36525	0.115943
hsa_circ_	0.235508	0.115944
hsa_circ_	-0.26665	0.115947
hsa_circ_	-0.45368	0.115949
hsa_circ_	-0.41097	0.115953
hsa_circ_	-0.59932	0.115953
hsa_circ_	-0.40434	0.115963
hsa_circ_	0.243928	0.115968
hsa_circ_	-0.22249	0.11598
hsa_circ_	0.33169	0.115981
hsa_circ_	-0.56395	0.11599
hsa_circ_	-0.72737	0.116005
hsa_circ_	0.224067	0.116011
hsa_circ_	-0.28822	0.116056
hsa_circ_	0.47531	0.11606
hsa_circ_	-0.59738	0.116065
hsa_circ_	-0.2892	0.116091
hsa_circ_	0.367473	0.116099
hsa_circ_	-0.27392	0.116103
hsa_circ_	-0.31404	0.116122
hsa_circ_	0.241446	0.116122
hsa_circ_	-0.31472	0.116136
hsa_circ_	-0.33557	0.116144
hsa_circ_	-0.35126	0.116155
hsa_circ_	0.399468	0.11616
hsa_circ_	0.432409	0.11616
hsa_circ_	0.258421	0.116161
hsa_circ_	-0.23934	0.116165
hsa_circ_	-0.26756	0.116181
hsa_circ_	-0.79063	0.116198
hsa_circ_	-0.22266	0.116206
hsa_circ_	0.246097	0.116213
hsa_circ_	0.349598	0.116219
hsa_circ_	-0.95664	0.116239
hsa_circ_	0.346136	0.11624
hsa_circ_	0.755816	0.116248

hsa_circ_	1.298997	0.116267
hsa_circ_	0.479723	0.116274
hsa_circ_	0.599596	0.116294
hsa_circ_	0.363833	0.116294
hsa_circ_	-0.34297	0.1163
hsa_circ_	0.238599	0.116301
hsa_circ_	-0.87229	0.116302
hsa_circ_	-0.33451	0.116302
hsa_circ_	0.343026	0.116309
hsa_circ_	-0.51129	0.116321
hsa_circ_	0.569975	0.116328
hsa_circ_	-0.25186	0.11633
hsa_circ_	0.239321	0.116339
hsa_circ_	0.516441	0.116339
hsa_circ_	0.389103	0.116344
hsa_circ_	0.367144	0.116347
hsa_circ_	0.899293	0.11635
hsa_circ_	-0.26648	0.116366
hsa_circ_	-0.64676	0.116384
hsa_circ_	0.469323	0.116405
hsa_circ_	0.484089	0.11642
hsa_circ_	-0.32994	0.116424
hsa_circ_	-0.26219	0.116433
hsa_circ_	0.510016	0.116436
hsa_circ_	-0.41977	0.116439
hsa_circ_	0.862451	0.116441
hsa_circ_	0.482642	0.116446
hsa_circ_	-0.3417	0.116451
hsa_circ_	0.505008	0.116464
hsa_circ_	0.786893	0.116467
hsa_circ_	-0.75347	0.116474
hsa_circ_	-0.71425	0.116481
hsa_circ_	0.238114	0.116503
hsa_circ_	0.324497	0.116506
hsa_circ_	0.732533	0.116521
hsa_circ_	-0.36154	0.116536
hsa_circ_	-0.30483	0.116536
hsa_circ_	1.006803	0.116536
hsa_circ_	-0.31492	0.11654
hsa_circ_	0.244202	0.116554
hsa_circ_	-0.28866	0.116554
hsa_circ_	-0.73599	0.116557
hsa_circ_	-0.23036	0.116612
hsa_circ_	-0.26283	0.116616
hsa_circ_	-0.31848	0.116622
hsa_circ_	-0.33587	0.116628
hsa_circ_	0.518472	0.11664
hsa_circ_	-0.30509	0.116641
hsa_circ_	0.5218	0.11665
hsa_circ_	0.275581	0.116655
hsa_circ_	-0.41438	0.116667
hsa_circ_	-0.30379	0.11667
hsa_circ_	0.911034	0.11669
hsa_circ_	-0.3668	0.116705

hsa_circ_	0.252401	0.116706
hsa_circ_	-0.26461	0.11671
hsa_circ_	-0.24789	0.116711
hsa_circ_	0.293579	0.11672
hsa_circ_	-0.69247	0.116727
hsa_circ_	1.072594	0.116728
hsa_circ_	0.634955	0.116728
hsa_circ_	0.555694	0.116735
hsa_circ_	0.767973	0.116754
hsa_circ_	0.321366	0.116758
hsa_circ_	-0.74207	0.116774
hsa_circ_	0.488725	0.116781
hsa_circ_	-0.57177	0.116794
hsa_circ_	-0.30328	0.11681
hsa_circ_	0.332303	0.116824
hsa_circ_	-0.31992	0.116831
hsa_circ_	-0.31574	0.116842
hsa_circ_	0.433976	0.116844
hsa_circ_	-0.39361	0.116848
hsa_circ_	-0.28411	0.116849
hsa_circ_	-0.34402	0.116852
hsa_circ_	0.257039	0.116886
hsa_circ_	-0.29357	0.116893
hsa_circ_	0.307784	0.116897
hsa_circ_	0.403003	0.116917
hsa_circ_	0.35773	0.116919
hsa_circ_	-0.22459	0.11692
hsa_circ_	0.739469	0.116923
hsa_circ_	-1.2603	0.116923
hsa_circ_	0.401301	0.116926
hsa_circ_	-0.26493	0.116936
hsa_circ_	0.331371	0.116942
hsa_circ_	-0.54678	0.116964
hsa_circ_	0.403337	0.116982
hsa_circ_	-1.43454	0.116984
hsa_circ_	-0.22713	0.116985
hsa_circ_	-0.42582	0.116994
hsa_circ_	0.442772	0.116997
hsa_circ_	-0.57596	0.117004
hsa_circ_	-0.24922	0.117008
hsa_circ_	-0.40448	0.117012
hsa_circ_	-1.21211	0.117016
hsa_circ_	-0.39375	0.117022
hsa_circ_	0.490246	0.117023
hsa_circ_	0.518629	0.117027
hsa_circ_	0.266405	0.117039
hsa_circ_	-0.30393	0.117044
hsa_circ_	0.491184	0.117046
hsa_circ_	-0.38671	0.117066
hsa_circ_	0.776093	0.117078
hsa_circ_	0.411601	0.117086
hsa_circ_	-0.28422	0.117086
hsa_circ_	0.332254	0.117099
hsa_circ_	-0.29902	0.117112

hsa_circ_	0.249045	0.117113
hsa_circ_	-0.38154	0.117126
hsa_circ_	-0.73342	0.11714
hsa_circ_	-0.33693	0.117146
hsa_circ_	0.385591	0.117155
hsa_circ_	0.257486	0.117168
hsa_circ_	0.756868	0.117172
hsa_circ_	-1.00378	0.117176
hsa_circ_	-0.25461	0.117186
hsa_circ_	-0.31127	0.117186
hsa_circ_	-0.35344	0.117194
hsa_circ_	1.172358	0.117194
hsa_circ_	0.404569	0.117208
hsa_circ_	-0.96265	0.117227
hsa_circ_	0.276886	0.117233
hsa_circ_	-0.26803	0.117239
hsa_circ_	0.74189	0.117245
hsa_circ_	0.53465	0.117247
hsa_circ_	-0.31199	0.117258
hsa_circ_	-0.2982	0.117267
hsa_circ_	0.489283	0.117268
hsa_circ_	-0.33633	0.117271
hsa_circ_	-0.3054	0.117272
hsa_circ_	0.414911	0.117277
hsa_circ_	1.036905	0.117284
hsa_circ_	-0.24632	0.117288
hsa_circ_	-0.4789	0.117291
hsa_circ_	-0.31625	0.117299
hsa_circ_	0.866708	0.117304
hsa_circ_	0.290615	0.117304
hsa_circ_	0.880457	0.117305
hsa_circ_	-0.96925	0.117307
hsa_circ_	0.615216	0.117314
hsa_circ_	-0.62105	0.117327
hsa_circ_	0.256378	0.117336
hsa_circ_	0.608457	0.117338
hsa_circ_	-0.83262	0.117344
hsa_circ_	-0.75715	0.117345
hsa_circ_	-0.25875	0.117348
hsa_circ_	0.616063	0.117351
hsa_circ_	-1.02196	0.117357
hsa_circ_	-0.50724	0.117382
hsa_circ_	-0.34995	0.117386
hsa_circ_	0.407373	0.117391
hsa_circ_	0.321894	0.117408
hsa_circ_	0.942557	0.117429
hsa_circ_	0.45655	0.117434
hsa_circ_	-0.23665	0.117439
hsa_circ_	0.499393	0.117452
hsa_circ_	0.473543	0.117457
hsa_circ_	0.409715	0.117462
hsa_circ_	0.284341	0.117467
hsa_circ_	-0.50019	0.117475
hsa_circ_	-0.30658	0.117479

hsa_circ_	-0.29039	0.117479
hsa_circ_	-1.1697	0.117492
hsa_circ_	-0.31508	0.117528
hsa_circ_	0.601536	0.11753
hsa_circ_	-0.68807	0.117534
hsa_circ_	0.406955	0.117545
hsa_circ_	-0.37147	0.117555
hsa_circ_	0.543052	0.117579
hsa_circ_	0.592543	0.117584
hsa_circ_	0.361321	0.117592
hsa_circ_	0.436526	0.117597
hsa_circ_	0.352448	0.117619
hsa_circ_	0.343552	0.117625
hsa_circ_	-0.33802	0.117626
hsa_circ_	0.294527	0.11764
hsa_circ_	-0.4501	0.117649
hsa_circ_	-0.44216	0.117652
hsa_circ_	-0.23283	0.117656
hsa_circ_	-0.25071	0.117656
hsa_circ_	0.506868	0.117662
hsa_circ_	0.298197	0.117663
hsa_circ_	-0.79584	0.117665
hsa_circ_	0.610856	0.117668
hsa_circ_	0.847063	0.117668
hsa_circ_	-0.25118	0.117681
hsa_circ_	-0.25534	0.117684
hsa_circ_	-0.33142	0.117684
hsa_circ_	-0.49516	0.117695
hsa_circ_	-0.36231	0.117697
hsa_circ_	-0.37844	0.117719
hsa_circ_	-0.89535	0.117725
hsa_circ_	-0.43608	0.117734
hsa_circ_	0.329511	0.117753
hsa_circ_	-0.9745	0.117756
hsa_circ_	1.099885	0.117758
hsa_circ_	0.427665	0.117762
hsa_circ_	0.27335	0.11777
hsa_circ_	0.434097	0.117775
hsa_circ_	-0.41183	0.117791
hsa_circ_	0.431025	0.1178
hsa_circ_	-0.31069	0.117803
hsa_circ_	-0.49452	0.117818
hsa_circ_	0.306969	0.117827
hsa_circ_	0.625954	0.117829
hsa_circ_	0.498817	0.117833
hsa_circ_	-0.33427	0.117844
hsa_circ_	-0.80546	0.117848
hsa_circ_	0.231081	0.117873
hsa_circ_	-0.31709	0.117884
hsa_circ_	0.448676	0.117888
hsa_circ_	0.29586	0.117909
hsa_circ_	-0.31823	0.117916
hsa_circ_	-0.74088	0.11794
hsa_circ_	-0.2637	0.117949

hsa_circ_	0.961485	0.117959
hsa_circ_	0.484725	0.117967
hsa_circ_	0.261064	0.117987
hsa_circ_	0.356547	0.117992
hsa_circ_	-0.29713	0.118007
hsa_circ_	0.612636	0.11801
hsa_circ_	0.443164	0.118012
hsa_circ_	-0.57734	0.118017
hsa_circ_	-0.33047	0.118024
hsa_circ_	-0.24095	0.118024
hsa_circ_	0.948116	0.118029
hsa_circ_	-0.26397	0.118037
hsa_circ_	0.397835	0.118039
hsa_circ_	0.259558	0.11804
hsa_circ_	-0.96627	0.118044
hsa_circ_	0.447211	0.118046
hsa_circ_	0.31819	0.118047
hsa_circ_	-0.51999	0.118052
hsa_circ_	0.711201	0.118067
hsa_circ_	0.715082	0.118069
hsa_circ_	-0.2831	0.118077
hsa_circ_	0.471796	0.118081
hsa_circ_	-0.59236	0.118086
hsa_circ_	0.619978	0.11809
hsa_circ_	-0.26359	0.118091
hsa_circ_	-0.34746	0.118094
hsa_circ_	-0.52138	0.118102
hsa_circ_	0.451216	0.11811
hsa_circ_	-0.94321	0.118149
hsa_circ_	0.277214	0.118151
hsa_circ_	1.162076	0.118157
hsa_circ_	0.665212	0.11816
hsa_circ_	-0.5079	0.118203
hsa_circ_	0.340464	0.118209
hsa_circ_	-0.64838	0.11822
hsa_circ_	0.420228	0.118225
hsa_circ_	0.29752	0.118264
hsa_circ_	-0.41219	0.118268
hsa_circ_	0.882346	0.11827
hsa_circ_	0.271701	0.118274
hsa_circ_	0.375345	0.118287
hsa_circ_	0.711182	0.118303
hsa_circ_	-0.46556	0.118312
hsa_circ_	0.25392	0.118316
hsa_circ_	0.404469	0.118317
hsa_circ_	-0.30114	0.118323
hsa_circ_	0.286858	0.118324
hsa_circ_	-0.42732	0.118328
hsa_circ_	0.971006	0.118331
hsa_circ_	-0.75979	0.118334
hsa_circ_	0.253605	0.11835
hsa_circ_	0.41632	0.118351
hsa_circ_	0.394355	0.118351
hsa_circ_	-0.28301	0.118352

hsa_circ_	1.253109	0.118359
hsa_circ_	0.257583	0.118364
hsa_circ_	-0.23825	0.118368
hsa_circ_	-0.29461	0.118394
hsa_circ_	-0.57222	0.1184
hsa_circ_	-0.25789	0.118402
hsa_circ_	-0.27331	0.118407
hsa_circ_	-0.406	0.118423
hsa_circ_	0.342228	0.118432
hsa_circ_	0.978079	0.118436
hsa_circ_	-0.61895	0.118437
hsa_circ_	-0.24737	0.118451
hsa_circ_	0.507394	0.118462
hsa_circ_	0.291177	0.118464
hsa_circ_	-0.45159	0.118465
hsa_circ_	-0.44335	0.118468
hsa_circ_	0.326562	0.118489
hsa_circ_	0.45825	0.118507
hsa_circ_	0.222518	0.118509
hsa_circ_	0.272243	0.118513
hsa_circ_	0.720017	0.118518
hsa_circ_	-0.5769	0.118524
hsa_circ_	0.327661	0.118539
hsa_circ_	-0.28463	0.118555
hsa_circ_	0.565063	0.118563
hsa_circ_	0.386095	0.118568
hsa_circ_	-1.02635	0.118574
hsa_circ_	-0.31357	0.118586
hsa_circ_	-0.33546	0.118586
hsa_circ_	0.298255	0.118597
hsa_circ_	-1.14621	0.118601
hsa_circ_	0.475403	0.118602
hsa_circ_	-0.30323	0.118607
hsa_circ_	0.55386	0.118613
hsa_circ_	0.768009	0.118613
hsa_circ_	-0.94033	0.118622
hsa_circ_	-0.30523	0.118629
hsa_circ_	0.483873	0.118632
hsa_circ_	-0.52868	0.118633
hsa_circ_	0.276026	0.118633
hsa_circ_	-0.38464	0.118669
hsa_circ_	-1.30163	0.118704
hsa_circ_	-0.26838	0.118704
hsa_circ_	-0.25148	0.118708
hsa_circ_	-0.40259	0.118711
hsa_circ_	-0.23032	0.118723
hsa_circ_	-0.3459	0.118735
hsa_circ_	-0.2436	0.118739
hsa_circ_	-0.35513	0.118746
hsa_circ_	-0.28781	0.118747
hsa_circ_	1.031333	0.118753
hsa_circ_	0.655513	0.118754
hsa_circ_	0.30629	0.118755
hsa_circ_	0.41787	0.118774

hsa_circ_	-0.28125	0.118778
hsa_circ_	-0.33564	0.118781
hsa_circ_	-0.24693	0.118786
hsa_circ_	0.286237	0.11879
hsa_circ_	-0.72604	0.118793
hsa_circ_	-0.23841	0.118795
hsa_circ_	0.439604	0.118799
hsa_circ_	0.706206	0.1188
hsa_circ_	0.860268	0.118803
hsa_circ_	0.49637	0.118804
hsa_circ_	0.855109	0.118818
hsa_circ_	0.328043	0.118818
hsa_circ_	0.442118	0.118819
hsa_circ_	-1.59845	0.118831
hsa_circ_	0.478737	0.118835
hsa_circ_	-0.47141	0.118846
hsa_circ_	-0.52204	0.118855
hsa_circ_	1.043968	0.118857
hsa_circ_	0.876132	0.118871
hsa_circ_	0.573471	0.118876
hsa_circ_	-0.67249	0.118879
hsa_circ_	0.364812	0.11888
hsa_circ_	0.314259	0.118881
hsa_circ_	0.364582	0.118885
hsa_circ_	-0.41884	0.118902
hsa_circ_	0.799659	0.118904
hsa_circ_	-0.56942	0.118906
hsa_circ_	1.112107	0.118909
hsa_circ_	0.641574	0.118909
hsa_circ_	-0.31746	0.118928
hsa_circ_	-0.26418	0.118952
hsa_circ_	0.22179	0.118955
hsa_circ_	-0.27023	0.118959
hsa_circ_	0.462684	0.118964
hsa_circ_	-0.5871	0.118974
hsa_circ_	0.90619	0.118982
hsa_circ_	-0.80414	0.118994
hsa_circ_	-0.57399	0.118997
hsa_circ_	-0.36293	0.11901
hsa_circ_	0.302691	0.119025
hsa_circ_	0.485263	0.119026
hsa_circ_	-0.29237	0.119053
hsa_circ_	-0.8239	0.119057
hsa_circ_	-0.30571	0.119069
hsa_circ_	-0.24357	0.11907
hsa_circ_	-0.41764	0.119075
hsa_circ_	-3.12301	0.119077
hsa_circ_	0.992886	0.119084
hsa_circ_	0.27507	0.119088
hsa_circ_	0.359685	0.119091
hsa_circ_	-0.2492	0.119116
hsa_circ_	-0.29617	0.119117
hsa_circ_	-0.86434	0.119133
hsa_circ_	0.469341	0.119135

hsa_circ_	-0.29794	0.119136
hsa_circ_	0.590237	0.119154
hsa_circ_	0.262109	0.119154
hsa_circ_	-0.24961	0.119156
hsa_circ_	0.313873	0.119157
hsa_circ_	-0.35717	0.119158
hsa_circ_	0.888074	0.11916
hsa_circ_	0.388853	0.119167
hsa_circ_	-0.27009	0.119177
hsa_circ_	0.430256	0.119179
hsa_circ_	0.340535	0.119179
hsa_circ_	0.285699	0.119189
hsa_circ_	0.425523	0.119205
hsa_circ_	-0.97572	0.119215
hsa_circ_	0.289039	0.119227
hsa_circ_	-0.27597	0.119238
hsa_circ_	-0.29624	0.11924
hsa_circ_	-0.24282	0.119243
hsa_circ_	-0.24601	0.11926
hsa_circ_	0.326638	0.119267
hsa_circ_	-0.42271	0.11927
hsa_circ_	-0.82839	0.119296
hsa_circ_	-0.60665	0.119312
hsa_circ_	0.590717	0.119315
hsa_circ_	-0.46121	0.11933
hsa_circ_	-1.27161	0.119332
hsa_circ_	-0.80563	0.119345
hsa_circ_	-0.281	0.119357
hsa_circ_	-0.57653	0.119362
hsa_circ_	-0.22838	0.119362
hsa_circ_	0.301633	0.11938
hsa_circ_	-0.35948	0.119405
hsa_circ_	0.239209	0.119412
hsa_circ_	-0.4478	0.119415
hsa_circ_	-0.24609	0.119455
hsa_circ_	0.389707	0.119455
hsa_circ_	0.255075	0.11946
hsa_circ_	0.445887	0.119467
hsa_circ_	0.856116	0.119471
hsa_circ_	0.449393	0.119475
hsa_circ_	0.431813	0.119502
hsa_circ_	-0.35514	0.119507
hsa_circ_	0.319291	0.11951
hsa_circ_	-0.28727	0.119512
hsa_circ_	0.527906	0.119514
hsa_circ_	0.416178	0.119521
hsa_circ_	-1.08161	0.119549
hsa_circ_	0.273997	0.119551
hsa_circ_	-0.49737	0.119553
hsa_circ_	0.332382	0.119563
hsa_circ_	-0.38823	0.119564
hsa_circ_	0.644439	0.119564
hsa_circ_	-0.54295	0.119564
hsa_circ_	0.399856	0.119567

hsa_circ_	0.429681	0.119572
hsa_circ_	0.342354	0.119573
hsa_circ_	-0.28791	0.11958
hsa_circ_	0.294258	0.119581
hsa_circ_	0.388323	0.119582
hsa_circ_	0.423388	0.119592
hsa_circ_	-0.35049	0.119612
hsa_circ_	-0.31509	0.119614
hsa_circ_	-0.44756	0.119619
hsa_circ_	-0.40902	0.119621
hsa_circ_	-0.23345	0.119626
hsa_circ_	0.797134	0.119627
hsa_circ_	0.451643	0.119629
hsa_circ_	-0.72207	0.119629
hsa_circ_	-0.42415	0.119635
hsa_circ_	-0.49205	0.119638
hsa_circ_	0.323788	0.119643
hsa_circ_	0.250542	0.119644
hsa_circ_	0.407811	0.119655
hsa_circ_	-1.10844	0.119656
hsa_circ_	0.350817	0.11968
hsa_circ_	0.783164	0.119683
hsa_circ_	-0.3026	0.119683
hsa_circ_	0.436557	0.119711
hsa_circ_	0.497748	0.119717
hsa_circ_	0.492931	0.119728
hsa_circ_	-0.3009	0.119729
hsa_circ_	0.326553	0.11974
hsa_circ_	0.343536	0.119749
hsa_circ_	0.446652	0.119751
hsa_circ_	0.298299	0.119752
hsa_circ_	0.580761	0.119779
hsa_circ_	0.644397	0.119782
hsa_circ_	-0.28944	0.11979
hsa_circ_	0.291032	0.119795
hsa_circ_	-0.39539	0.119809
hsa_circ_	0.720666	0.119819
hsa_circ_	-0.61826	0.11984
hsa_circ_	-0.31941	0.119858
hsa_circ_	0.341509	0.119859
hsa_circ_	0.362154	0.119863
hsa_circ_	-0.56622	0.119877
hsa_circ_	0.630087	0.119878
hsa_circ_	0.2724	0.119891
hsa_circ_	0.276528	0.119892
hsa_circ_	-0.88851	0.119899
hsa_circ_	-0.9754	0.119913
hsa_circ_	-0.38929	0.119919
hsa_circ_	-0.22983	0.119923
hsa_circ_	0.287969	0.119932
hsa_circ_	-0.43885	0.119937
hsa_circ_	0.523054	0.119939
hsa_circ_	-0.38993	0.119962
hsa_circ_	0.275162	0.11998

hsa_circ_	0.528321	0.119985
hsa_circ_	0.625323	0.119994
hsa_circ_	-0.77241	0.119994
hsa_circ_	0.37171	0.120011
hsa_circ_	0.313885	0.120012
hsa_circ_	0.362825	0.120017
hsa_circ_	-0.25293	0.120023
hsa_circ_	-0.30119	0.120028
hsa_circ_	0.429958	0.120029
hsa_circ_	0.343296	0.120043
hsa_circ_	0.371927	0.120057
hsa_circ_	0.281318	0.120065
hsa_circ_	0.255536	0.120071
hsa_circ_	-0.30178	0.120072
hsa_circ_	-0.33926	0.12009
hsa_circ_	0.492376	0.12009
hsa_circ_	0.350661	0.120124
hsa_circ_	0.328396	0.12013
hsa_circ_	0.632296	0.120134
hsa_circ_	0.316626	0.120161
hsa_circ_	0.267116	0.120173
hsa_circ_	-0.25796	0.120198
hsa_circ_	-0.33265	0.120207
hsa_circ_	-0.28985	0.120214
hsa_circ_	-1.43928	0.120221
hsa_circ_	0.277144	0.120223
hsa_circ_	-0.25845	0.120235
hsa_circ_	-1.38571	0.120236
hsa_circ_	0.325802	0.120239
hsa_circ_	-1.07156	0.120247
hsa_circ_	0.306307	0.120255
hsa_circ_	-0.44469	0.120287
hsa_circ_	0.32717	0.12029
hsa_circ_	0.662581	0.120302
hsa_circ_	0.221762	0.120304
hsa_circ_	-0.28776	0.120336
hsa_circ_	-0.38131	0.120356
hsa_circ_	0.403292	0.120375
hsa_circ_	-0.23207	0.120377
hsa_circ_	-0.25441	0.12038
hsa_circ_	0.355145	0.120381
hsa_circ_	-0.41762	0.120391
hsa_circ_	-0.25402	0.120396
hsa_circ_	0.409479	0.120404
hsa_circ_	0.231083	0.120412
hsa_circ_	0.512656	0.120421
hsa_circ_	0.391843	0.120426
hsa_circ_	-0.32897	0.120426
hsa_circ_	-0.67692	0.120432
hsa_circ_	0.396171	0.120458
hsa_circ_	-0.44618	0.120462
hsa_circ_	0.465223	0.120465
hsa_circ_	0.436991	0.120475
hsa_circ_	-0.38604	0.120496

hsa_circ_	-0.28503	0.120497
hsa_circ_	0.347709	0.1205
hsa_circ_	-0.29381	0.120501
hsa_circ_	0.967072	0.120513
hsa_circ_	0.352927	0.12054
hsa_circ_	0.402051	0.12055
hsa_circ_	0.249711	0.120555
hsa_circ_	-0.29071	0.120561
hsa_circ_	0.259373	0.120565
hsa_circ_	-0.55635	0.120567
hsa_circ_	0.407776	0.120574
hsa_circ_	-0.3081	0.120578
hsa_circ_	0.49532	0.120585
hsa_circ_	-0.81823	0.120586
hsa_circ_	-0.3369	0.120587
hsa_circ_	-0.42428	0.120598
hsa_circ_	-0.26837	0.120604
hsa_circ_	0.2333	0.120621
hsa_circ_	-0.30604	0.120622
hsa_circ_	-0.31712	0.120625
hsa_circ_	1.169443	0.12063
hsa_circ_	-0.32734	0.120641
hsa_circ_	-0.26317	0.120643
hsa_circ_	-0.24797	0.120651
hsa_circ_	-0.32963	0.120674
hsa_circ_	0.540098	0.120675
hsa_circ_	0.276607	0.120681
hsa_circ_	-0.37027	0.120696
hsa_circ_	-0.23204	0.120701
hsa_circ_	1.056799	0.120714
hsa_circ_	-0.30351	0.120734
hsa_circ_	-0.51925	0.120743
hsa_circ_	-0.29811	0.120748
hsa_circ_	-0.59856	0.120756
hsa_circ_	0.351691	0.120771
hsa_circ_	0.252334	0.120775
hsa_circ_	-1.03037	0.120793
hsa_circ_	-0.29981	0.120828
hsa_circ_	-0.26078	0.120852
hsa_circ_	-0.38399	0.120852
hsa_circ_	-0.76181	0.120859
hsa_circ_	-0.43811	0.120865
hsa_circ_	-0.36598	0.120876
hsa_circ_	0.492458	0.120886
hsa_circ_	-0.37919	0.120891
hsa_circ_	-0.47092	0.120894
hsa_circ_	-0.39516	0.120899
hsa_circ_	-0.22397	0.120901
hsa_circ_	-0.53666	0.120913
hsa_circ_	0.346045	0.120921
hsa_circ_	-0.34606	0.120921
hsa_circ_	-0.24562	0.120922
hsa_circ_	-0.22948	0.120936
hsa_circ_	0.313357	0.120982

hsa_circ_	-0.68879	0.12103
hsa_circ_	-0.28007	0.121031
hsa_circ_	0.553126	0.121037
hsa_circ_	-0.69836	0.121041
hsa_circ_	0.946223	0.121043
hsa_circ_	-1.13332	0.12105
hsa_circ_	-0.63831	0.121053
hsa_circ_	-0.36031	0.121068
hsa_circ_	-0.27012	0.121075
hsa_circ_	-0.2536	0.121078
hsa_circ_	-0.30195	0.121079
hsa_circ_	-0.54248	0.121082
hsa_circ_	0.360387	0.121089
hsa_circ_	-0.26478	0.121093
hsa_circ_	0.351315	0.121098
hsa_circ_	0.284807	0.121122
hsa_circ_	0.951016	0.121128
hsa_circ_	-0.5989	0.12114
hsa_circ_	0.42309	0.121143
hsa_circ_	-0.29794	0.121154
hsa_circ_	-0.42376	0.121159
hsa_circ_	0.452931	0.121168
hsa_circ_	-0.56298	0.121173
hsa_circ_	-0.27242	0.121175
hsa_circ_	-0.43663	0.121182
hsa_circ_	-0.48919	0.121193
hsa_circ_	0.321565	0.121197
hsa_circ_	-0.35926	0.121199
hsa_circ_	-0.50251	0.121205
hsa_circ_	-0.94103	0.121206
hsa_circ_	0.797501	0.121208
hsa_circ_	0.283993	0.121212
hsa_circ_	0.375925	0.121217
hsa_circ_	-0.33551	0.121231
hsa_circ_	1.026206	0.121231
hsa_circ_	-0.87996	0.121233
hsa_circ_	0.331142	0.12124
hsa_circ_	-0.6222	0.121257
hsa_circ_	-0.70609	0.121264
hsa_circ_	0.478977	0.121264
hsa_circ_	-0.26653	0.121275
hsa_circ_	0.524849	0.121284
hsa_circ_	0.367349	0.121296
hsa_circ_	-0.90414	0.121297
hsa_circ_	0.425366	0.121303
hsa_circ_	0.418473	0.121308
hsa_circ_	0.839195	0.121323
hsa_circ_	-0.28593	0.121324
hsa_circ_	1.178798	0.121331
hsa_circ_	0.321816	0.121334
hsa_circ_	0.32991	0.121342
hsa_circ_	-0.50865	0.121346
hsa_circ_	0.401227	0.121351
hsa_circ_	-0.29218	0.121352

hsa_circ_	0.307913	0.121355
hsa_circ_	0.320156	0.121362
hsa_circ_	-0.3286	0.121371
hsa_circ_	-0.75732	0.121372
hsa_circ_	-0.22237	0.121376
hsa_circ_	0.752358	0.121377
hsa_circ_	0.326925	0.12138
hsa_circ_	0.261847	0.12139
hsa_circ_	0.965142	0.121392
hsa_circ_	0.354641	0.121396
hsa_circ_	-0.30873	0.121397
hsa_circ_	0.447869	0.121407
hsa_circ_	-0.28497	0.121408
hsa_circ_	1.157438	0.121433
hsa_circ_	-0.29796	0.121435
hsa_circ_	0.436692	0.121437
hsa_circ_	0.384915	0.121444
hsa_circ_	-0.26286	0.121444
hsa_circ_	0.227693	0.121446
hsa_circ_	0.341546	0.12146
hsa_circ_	0.436113	0.121467
hsa_circ_	0.247783	0.12147
hsa_circ_	0.483226	0.121489
hsa_circ_	-0.38946	0.121493
hsa_circ_	0.813054	0.121494
hsa_circ_	0.21278	0.121508
hsa_circ_	-0.39826	0.121513
hsa_circ_	0.244594	0.121548
hsa_circ_	0.420877	0.12156
hsa_circ_	-0.81677	0.12156
hsa_circ_	-0.23637	0.121561
hsa_circ_	0.636333	0.121562
hsa_circ_	-0.45189	0.121581
hsa_circ_	-0.36879	0.121582
hsa_circ_	0.341312	0.121588
hsa_circ_	-0.67369	0.121589
hsa_circ_	-1.30729	0.12159
hsa_circ_	-0.24833	0.121592
hsa_circ_	0.323934	0.121593
hsa_circ_	0.374803	0.121594
hsa_circ_	0.339454	0.121601
hsa_circ_	-0.55502	0.121613
hsa_circ_	0.424963	0.121624
hsa_circ_	-0.25449	0.121631
hsa_circ_	-0.4159	0.121647
hsa_circ_	-0.25457	0.121656
hsa_circ_	0.500747	0.121666
hsa_circ_	0.378858	0.121677
hsa_circ_	0.440693	0.121678
hsa_circ_	0.329418	0.121683
hsa_circ_	-0.40152	0.121688
hsa_circ_	-0.31406	0.121702
hsa_circ_	-0.41481	0.121706
hsa_circ_	0.730219	0.121735

hsa_circ_	-0.29735	0.121745
hsa_circ_	-0.45211	0.121745
hsa_circ_	0.727371	0.121752
hsa_circ_	0.273282	0.121753
hsa_circ_	0.357378	0.121754
hsa_circ_	-0.38387	0.121756
hsa_circ_	-0.24884	0.121768
hsa_circ_	0.233246	0.121779
hsa_circ_	-0.53685	0.121784
hsa_circ_	-0.69002	0.121792
hsa_circ_	-0.42733	0.121793
hsa_circ_	0.511683	0.121821
hsa_circ_	-0.78254	0.121824
hsa_circ_	0.677778	0.121824
hsa_circ_	-1.05647	0.121836
hsa_circ_	-0.26634	0.121845
hsa_circ_	-1.37099	0.121845
hsa_circ_	-0.76897	0.121869
hsa_circ_	-0.2754	0.121871
hsa_circ_	-0.47264	0.12188
hsa_circ_	1.097308	0.121886
hsa_circ_	-0.36957	0.121893
hsa_circ_	1.08965	0.121904
hsa_circ_	0.627598	0.121916
hsa_circ_	-0.25716	0.121916
hsa_circ_	0.672819	0.121932
hsa_circ_	0.349769	0.121937
hsa_circ_	0.377884	0.12194
hsa_circ_	-0.34892	0.121953
hsa_circ_	-0.34389	0.121968
hsa_circ_	-0.45917	0.122006
hsa_circ_	0.233946	0.122019
hsa_circ_	-0.33282	0.12203
hsa_circ_	-0.99358	0.122039
hsa_circ_	-0.43753	0.122042
hsa_circ_	-1.34461	0.12206
hsa_circ_	-0.26149	0.122061
hsa_circ_	-0.41388	0.122073
hsa_circ_	-0.34475	0.122081
hsa_circ_	0.482392	0.122081
hsa_circ_	-0.57838	0.12209
hsa_circ_	0.224811	0.122091
hsa_circ_	-0.38697	0.122093
hsa_circ_	0.558137	0.122096
hsa_circ_	-0.33224	0.122098
hsa_circ_	-0.95263	0.122101
hsa_circ_	-0.32824	0.122104
hsa_circ_	0.386989	0.122108
hsa_circ_	0.953473	0.122119
hsa_circ_	0.261166	0.122135
hsa_circ_	-0.3881	0.122147
hsa_circ_	0.502536	0.122151
hsa_circ_	0.312085	0.122161
hsa_circ_	0.98487	0.122173

hsa_circ_	0.474702	0.122188
hsa_circ_	0.712905	0.122198
hsa_circ_	-0.28146	0.122205
hsa_circ_	0.30638	0.122205
hsa_circ_	0.998823	0.122206
hsa_circ_	-1.13746	0.122218
hsa_circ_	-0.22812	0.122222
hsa_circ_	0.375376	0.122228
hsa_circ_	-0.36473	0.122239
hsa_circ_	-0.2831	0.122239
hsa_circ_	-0.46008	0.122244
hsa_circ_	0.22965	0.122246
hsa_circ_	-0.25322	0.122258
hsa_circ_	-0.42983	0.122274
hsa_circ_	-0.2588	0.122294
hsa_circ_	0.357047	0.1223
hsa_circ_	0.44877	0.122318
hsa_circ_	0.459532	0.122326
hsa_circ_	-0.3844	0.122332
hsa_circ_	-0.34326	0.122337
hsa_circ_	-0.37155	0.122337
hsa_circ_	-0.32646	0.122355
hsa_circ_	-0.25805	0.122368
hsa_circ_	-0.63612	0.122387
hsa_circ_	-0.4398	0.122413
hsa_circ_	0.33556	0.122419
hsa_circ_	0.376255	0.122421
hsa_circ_	-0.51363	0.122424
hsa_circ_	0.447384	0.122427
hsa_circ_	-0.26104	0.12243
hsa_circ_	0.218832	0.122444
hsa_circ_	0.432639	0.122446
hsa_circ_	0.219786	0.122448
hsa_circ_	0.306933	0.122454
hsa_circ_	-0.25614	0.122477
hsa_circ_	0.277783	0.122487
hsa_circ_	0.385716	0.122488
hsa_circ_	0.528074	0.122491
hsa_circ_	-0.32221	0.122501
hsa_circ_	0.775574	0.122505
hsa_circ_	0.259668	0.12251
hsa_circ_	-0.67833	0.122523
hsa_circ_	0.275416	0.122535
hsa_circ_	0.269931	0.12254
hsa_circ_	0.324577	0.122542
hsa_circ_	-0.29299	0.122565
hsa_circ_	0.353064	0.122568
hsa_circ_	-0.56532	0.122572
hsa_circ_	-0.27752	0.12258
hsa_circ_	0.276239	0.122584
hsa_circ_	-0.25033	0.122589
hsa_circ_	0.25249	0.122593
hsa_circ_	0.233277	0.122606
hsa_circ_	-1.54842	0.122613

hsa_circ_	-0.36716	0.122614
hsa_circ_	0.747098	0.122643
hsa_circ_	-0.36987	0.122644
hsa_circ_	0.90495	0.122644
hsa_circ_	0.325241	0.122657
hsa_circ_	-0.26375	0.122673
hsa_circ_	0.308174	0.122676
hsa_circ_	-0.34995	0.122687
hsa_circ_	0.784411	0.122687
hsa_circ_	-0.48421	0.122691
hsa_circ_	0.338222	0.122701
hsa_circ_	-0.43173	0.122715
hsa_circ_	0.501717	0.122716
hsa_circ_	0.266259	0.122716
hsa_circ_	0.969019	0.122717
hsa_circ_	-0.69273	0.122717
hsa_circ_	-0.78304	0.122723
hsa_circ_	-0.33176	0.122726
hsa_circ_	-0.67761	0.122727
hsa_circ_	-0.29938	0.122748
hsa_circ_	-0.27825	0.122753
hsa_circ_	0.410281	0.122757
hsa_circ_	-0.45749	0.122768
hsa_circ_	-0.30534	0.122773
hsa_circ_	-0.29109	0.122778
hsa_circ_	-0.43061	0.122782
hsa_circ_	-0.30477	0.122785
hsa_circ_	-0.7787	0.122797
hsa_circ_	0.338765	0.122804
hsa_circ_	0.732032	0.122816
hsa_circ_	-0.28287	0.122822
hsa_circ_	-0.2192	0.122831
hsa_circ_	-0.42449	0.122873
hsa_circ_	-0.31363	0.122876
hsa_circ_	-1.18743	0.122877
hsa_circ_	-0.44304	0.122879
hsa_circ_	-0.41786	0.122885
hsa_circ_	0.373426	0.122886
hsa_circ_	-0.63492	0.122906
hsa_circ_	0.2206	0.12291
hsa_circ_	0.779891	0.122914
hsa_circ_	0.446805	0.122918
hsa_circ_	0.412136	0.12292
hsa_circ_	-0.38118	0.122932
hsa_circ_	-0.40363	0.122956
hsa_circ_	0.362221	0.122968
hsa_circ_	-0.43705	0.12298
hsa_circ_	0.254776	0.122999
hsa_circ_	-0.95243	0.123002
hsa_circ_	-0.30476	0.123009
hsa_circ_	-0.85394	0.123011
hsa_circ_	-0.30997	0.123015
hsa_circ_	-0.57496	0.123041
hsa_circ_	-0.52853	0.123049

hsa_circ_	0.464123	0.123053
hsa_circ_	-0.29748	0.123058
hsa_circ_	0.699598	0.123059
hsa_circ_	0.246035	0.123098
hsa_circ_	1.001659	0.123106
hsa_circ_	0.240713	0.123108
hsa_circ_	-0.26116	0.123116
hsa_circ_	0.611397	0.123127
hsa_circ_	-0.2634	0.123127
hsa_circ_	-0.36275	0.123152
hsa_circ_	0.699344	0.123162
hsa_circ_	0.313531	0.123163
hsa_circ_	0.28371	0.123172
hsa_circ_	1.1473	0.123173
hsa_circ_	-0.63605	0.123178
hsa_circ_	0.404266	0.123188
hsa_circ_	-0.29827	0.123193
hsa_circ_	-0.87881	0.123199
hsa_circ_	-0.40564	0.123199
hsa_circ_	0.357167	0.123218
hsa_circ_	-0.25302	0.123237
hsa_circ_	-0.38806	0.12324
hsa_circ_	-0.33533	0.123243
hsa_circ_	-0.53109	0.123249
hsa_circ_	0.932905	0.123253
hsa_circ_	-0.33279	0.123263
hsa_circ_	0.249413	0.123266
hsa_circ_	0.383279	0.123267
hsa_circ_	-0.27614	0.123273
hsa_circ_	0.392533	0.123289
hsa_circ_	-0.3484	0.123303
hsa_circ_	-0.27966	0.123318
hsa_circ_	-0.2416	0.123319
hsa_circ_	0.431071	0.123325
hsa_circ_	0.231069	0.12333
hsa_circ_	-0.46225	0.123354
hsa_circ_	0.572586	0.123355
hsa_circ_	0.279241	0.12336
hsa_circ_	0.279347	0.123374
hsa_circ_	0.38856	0.123384
hsa_circ_	-0.53119	0.123388
hsa_circ_	-0.41416	0.123389
hsa_circ_	0.273784	0.123397
hsa_circ_	0.392708	0.123399
hsa_circ_	0.332144	0.123399
hsa_circ_	-0.34613	0.123403
hsa_circ_	0.251777	0.123405
hsa_circ_	0.322872	0.123417
hsa_circ_	0.760541	0.123423
hsa_circ_	0.287287	0.12344
hsa_circ_	0.322093	0.123441
hsa_circ_	-0.69899	0.123464
hsa_circ_	-0.47635	0.123467
hsa_circ_	-0.27413	0.123474

hsa_circ_	-0.41212	0.123491
hsa_circ_	0.402227	0.1235
hsa_circ_	0.877651	0.1235
hsa_circ_	0.388149	0.123507
hsa_circ_	-0.34725	0.12353
hsa_circ_	0.256491	0.123535
hsa_circ_	-0.24847	0.123536
hsa_circ_	0.301523	0.123537
hsa_circ_	-0.30102	0.123543
hsa_circ_	-0.2348	0.123565
hsa_circ_	1.089282	0.123573
hsa_circ_	-0.46155	0.123573
hsa_circ_	-0.28851	0.123577
hsa_circ_	0.345343	0.12358
hsa_circ_	0.286871	0.123586
hsa_circ_	0.412542	0.123588
hsa_circ_	0.332425	0.123589
hsa_circ_	0.354292	0.123599
hsa_circ_	0.70499	0.123603
hsa_circ_	-0.2619	0.123612
hsa_circ_	-0.25873	0.123635
hsa_circ_	0.492605	0.123648
hsa_circ_	0.910248	0.123701
hsa_circ_	-0.56389	0.123703
hsa_circ_	-0.23602	0.123707
hsa_circ_	-0.44412	0.123725
hsa_circ_	1.357681	0.123729
hsa_circ_	0.636387	0.123733
hsa_circ_	-0.42272	0.123734
hsa_circ_	-0.35622	0.123735
hsa_circ_	-0.47909	0.123738
hsa_circ_	0.698874	0.123749
hsa_circ_	0.509148	0.123754
hsa_circ_	0.444408	0.123756
hsa_circ_	-0.26188	0.123762
hsa_circ_	-0.80576	0.123768
hsa_circ_	-0.33226	0.123769
hsa_circ_	-0.26066	0.123771
hsa_circ_	-0.91624	0.123773
hsa_circ_	0.47883	0.123782
hsa_circ_	-0.34959	0.123788
hsa_circ_	0.300855	0.123804
hsa_circ_	-0.23356	0.123815
hsa_circ_	-0.29721	0.123818
hsa_circ_	-0.22577	0.123831
hsa_circ_	-0.26402	0.123839
hsa_circ_	0.245518	0.123849
hsa_circ_	-0.24787	0.12386
hsa_circ_	-0.45442	0.12386
hsa_circ_	0.337587	0.123881
hsa_circ_	-0.6415	0.123906
hsa_circ_	0.249862	0.12391
hsa_circ_	-0.26754	0.123914
hsa_circ_	-0.26619	0.123916

hsa_circ_	0.512024	0.123957
hsa_circ_	0.765028	0.123959
hsa_circ_	-0.30068	0.123972
hsa_circ_	0.376991	0.123977
hsa_circ_	0.436508	0.123982
hsa_circ_	0.310581	0.123993
hsa_circ_	-0.29021	0.124007
hsa_circ_	-0.41402	0.124009
hsa_circ_	-0.26975	0.124017
hsa_circ_	-0.35362	0.124018
hsa_circ_	-0.45362	0.124021
hsa_circ_	0.385183	0.124028
hsa_circ_	-0.33881	0.124045
hsa_circ_	0.287037	0.124053
hsa_circ_	-0.59098	0.124055
hsa_circ_	1.011593	0.124058
hsa_circ_	0.369903	0.124062
hsa_circ_	1.698545	0.124068
hsa_circ_	-0.22362	0.12408
hsa_circ_	0.55655	0.124096
hsa_circ_	-0.71909	0.124096
hsa_circ_	0.932794	0.124104
hsa_circ_	0.247623	0.124105
hsa_circ_	-0.27741	0.124112
hsa_circ_	-0.70403	0.124128
hsa_circ_	-0.36054	0.124137
hsa_circ_	0.540136	0.124159
hsa_circ_	-0.24588	0.124164
hsa_circ_	-0.27164	0.124166
hsa_circ_	-0.32977	0.124168
hsa_circ_	-0.44544	0.124168
hsa_circ_	-0.38917	0.124188
hsa_circ_	0.223533	0.124213
hsa_circ_	0.762597	0.124223
hsa_circ_	-0.41446	0.124228
hsa_circ_	0.34381	0.124243
hsa_circ_	-0.46533	0.124253
hsa_circ_	0.328808	0.124262
hsa_circ_	0.917988	0.124263
hsa_circ_	0.29167	0.124269
hsa_circ_	-0.74889	0.124274
hsa_circ_	0.228269	0.124279
hsa_circ_	-0.39602	0.124282
hsa_circ_	-0.29007	0.12429
hsa_circ_	0.339092	0.124294
hsa_circ_	-0.28947	0.124295
hsa_circ_	0.352981	0.124298
hsa_circ_	0.429257	0.124327
hsa_circ_	-0.2795	0.124337
hsa_circ_	-0.34429	0.124355
hsa_circ_	0.362365	0.124356
hsa_circ_	0.281779	0.124357
hsa_circ_	0.333862	0.124405
hsa_circ_	0.258572	0.124411

hsa_circ_	0.534899	0.124435
hsa_circ_	-0.52321	0.124459
hsa_circ_	-0.29161	0.12446
hsa_circ_	-0.52266	0.124469
hsa_circ_	-0.47368	0.124471
hsa_circ_	0.332212	0.124473
hsa_circ_	-0.6253	0.124479
hsa_circ_	0.401264	0.124481
hsa_circ_	0.379727	0.124496
hsa_circ_	0.453169	0.124499
hsa_circ_	-0.21977	0.124546
hsa_circ_	0.53985	0.124583
hsa_circ_	-0.71073	0.124596
hsa_circ_	-0.28127	0.124598
hsa_circ_	-0.3265	0.1246
hsa_circ_	0.387584	0.124604
hsa_circ_	0.410901	0.12462
hsa_circ_	0.667133	0.124636
hsa_circ_	-0.27029	0.124656
hsa_circ_	-0.43707	0.124669
hsa_circ_	0.29646	0.124675
hsa_circ_	0.363164	0.124683
hsa_circ_	-0.56388	0.124683
hsa_circ_	0.559143	0.124684
hsa_circ_	0.24657	0.124741
hsa_circ_	-0.44171	0.124754
hsa_circ_	-0.31844	0.124759
hsa_circ_	0.385802	0.124761
hsa_circ_	0.224899	0.124764
hsa_circ_	-0.28276	0.124782
hsa_circ_	0.325958	0.124796
hsa_circ_	-0.31494	0.124806
hsa_circ_	0.39726	0.124808
hsa_circ_	-0.24283	0.12481
hsa_circ_	0.312329	0.124821
hsa_circ_	-0.32431	0.124824
hsa_circ_	0.704411	0.124833
hsa_circ_	0.31679	0.124837
hsa_circ_	0.466874	0.124857
hsa_circ_	0.373797	0.124864
hsa_circ_	-0.68373	0.124866
hsa_circ_	0.309194	0.124881
hsa_circ_	-0.23361	0.124884
hsa_circ_	0.887456	0.124904
hsa_circ_	-0.48721	0.124905
hsa_circ_	0.44582	0.124944
hsa_circ_	0.365499	0.124948
hsa_circ_	0.449271	0.124956
hsa_circ_	-0.27806	0.124958
hsa_circ_	0.439014	0.124962
hsa_circ_	0.640614	0.124963
hsa_circ_	0.289793	0.124969
hsa_circ_	0.803971	0.124971
hsa_circ_	-0.68054	0.124972

hsa_circ_	-0.26146	0.124981
hsa_circ_	-0.52848	0.124983
hsa_circ_	-0.59569	0.124993
hsa_circ_	-1.14389	0.124998
hsa_circ_	-0.29161	0.125006
hsa_circ_	-0.63723	0.125009
hsa_circ_	0.346545	0.125015
hsa_circ_	0.315311	0.125018
hsa_circ_	-0.67904	0.125023
hsa_circ_	0.239018	0.125025
hsa_circ_	0.398563	0.125036
hsa_circ_	0.374741	0.12505
hsa_circ_	0.779061	0.125077
hsa_circ_	0.38715	0.125089
hsa_circ_	-0.54357	0.125099
hsa_circ_	0.260643	0.125109
hsa_circ_	-0.39656	0.125113
hsa_circ_	0.432797	0.125127
hsa_circ_	-0.66039	0.125132
hsa_circ_	0.345652	0.125149
hsa_circ_	-0.30313	0.12515
hsa_circ_	-0.51062	0.125162
hsa_circ_	-0.33967	0.125163
hsa_circ_	-0.35522	0.125177
hsa_circ_	-0.43236	0.125181
hsa_circ_	-0.21231	0.125182
hsa_circ_	0.496823	0.125183
hsa_circ_	-0.28184	0.125183
hsa_circ_	0.238295	0.125192
hsa_circ_	-0.24272	0.125204
hsa_circ_	-0.26344	0.125207
hsa_circ_	0.253585	0.125217
hsa_circ_	-0.30376	0.125228
hsa_circ_	0.349392	0.125236
hsa_circ_	0.795453	0.125244
hsa_circ_	-0.28545	0.125244
hsa_circ_	-0.52904	0.125249
hsa_circ_	0.381243	0.125254
hsa_circ_	0.358789	0.125266
hsa_circ_	0.482993	0.125268
hsa_circ_	-0.61835	0.125275
hsa_circ_	-0.23343	0.125277
hsa_circ_	0.349132	0.12528
hsa_circ_	-0.23992	0.125299
hsa_circ_	1.070234	0.125308
hsa_circ_	-0.34206	0.125311
hsa_circ_	-1.92062	0.125316
hsa_circ_	-0.36032	0.125322
hsa_circ_	-0.23589	0.125322
hsa_circ_	-0.25833	0.125326
hsa_circ_	0.433951	0.125349
hsa_circ_	0.253002	0.125366
hsa_circ_	0.294422	0.125377
hsa_circ_	0.302739	0.12538

hsa_circ_	0.444553	0.125383
hsa_circ_	-0.47063	0.125386
hsa_circ_	-0.63468	0.125387
hsa_circ_	-0.28081	0.125389
hsa_circ_	-0.45547	0.125414
hsa_circ_	0.264369	0.125432
hsa_circ_	0.319373	0.125445
hsa_circ_	-0.28009	0.125475
hsa_circ_	-0.3938	0.125479
hsa_circ_	-0.29112	0.125479
hsa_circ_	1.196447	0.12548
hsa_circ_	-0.29261	0.125485
hsa_circ_	-0.29802	0.125486
hsa_circ_	0.427191	0.125494
hsa_circ_	-0.30786	0.125505
hsa_circ_	0.848967	0.125511
hsa_circ_	-0.30872	0.125517
hsa_circ_	-0.41956	0.125526
hsa_circ_	0.38445	0.125529
hsa_circ_	-0.25925	0.125547
hsa_circ_	0.467454	0.125551
hsa_circ_	-0.43754	0.125557
hsa_circ_	-0.30511	0.125558
hsa_circ_	0.243504	0.125562
hsa_circ_	-0.33319	0.125565
hsa_circ_	0.385495	0.125575
hsa_circ_	-0.23658	0.125586
hsa_circ_	0.756596	0.125597
hsa_circ_	0.403776	0.125612
hsa_circ_	0.461314	0.125633
hsa_circ_	0.414821	0.125641
hsa_circ_	0.247222	0.125661
hsa_circ_	-0.29134	0.125687
hsa_circ_	-0.34631	0.125704
hsa_circ_	-0.37435	0.12572
hsa_circ_	-0.51428	0.125727
hsa_circ_	-0.36588	0.125735
hsa_circ_	-0.33413	0.125742
hsa_circ_	0.784083	0.125745
hsa_circ_	0.300801	0.125746
hsa_circ_	0.247497	0.125752
hsa_circ_	-0.28786	0.125761
hsa_circ_	0.336755	0.125792
hsa_circ_	0.491047	0.125799
hsa_circ_	-1.19455	0.125807
hsa_circ_	-0.44087	0.125808
hsa_circ_	0.326308	0.125813
hsa_circ_	-0.25242	0.125825
hsa_circ_	0.305852	0.125833
hsa_circ_	-0.23494	0.125835
hsa_circ_	0.678053	0.125839
hsa_circ_	-0.48785	0.12584
hsa_circ_	0.271301	0.125846
hsa_circ_	-0.62374	0.125856

hsa_circ_	0.240146	0.12586
hsa_circ_	-0.78446	0.125871
hsa_circ_	-0.46921	0.125874
hsa_circ_	0.479181	0.125889
hsa_circ_	0.297306	0.125909
hsa_circ_	-0.35282	0.125931
hsa_circ_	-0.56433	0.12594
hsa_circ_	-0.35458	0.125941
hsa_circ_	-0.78372	0.125947
hsa_circ_	0.251504	0.125968
hsa_circ_	-0.40383	0.125982
hsa_circ_	-0.37513	0.125988
hsa_circ_	-0.77357	0.12601
hsa_circ_	-0.29808	0.126017
hsa_circ_	0.508904	0.126022
hsa_circ_	-0.31816	0.126042
hsa_circ_	0.375461	0.126059
hsa_circ_	-0.55828	0.12606
hsa_circ_	0.400606	0.126069
hsa_circ_	0.364047	0.126073
hsa_circ_	-0.25651	0.126091
hsa_circ_	-0.44365	0.126096
hsa_circ_	-0.24946	0.126097
hsa_circ_	-0.24772	0.126109
hsa_circ_	-0.24287	0.126114
hsa_circ_	0.350765	0.126124
hsa_circ_	-0.47857	0.126131
hsa_circ_	0.441913	0.126152
hsa_circ_	-0.21601	0.126153
hsa_circ_	0.40365	0.126156
hsa_circ_	0.573091	0.126177
hsa_circ_	-0.75925	0.126178
hsa_circ_	0.409231	0.126205
hsa_circ_	-0.28632	0.126205
hsa_circ_	0.274105	0.126223
hsa_circ_	-0.42142	0.126233
hsa_circ_	0.448819	0.126239
hsa_circ_	-0.40089	0.126245
hsa_circ_	0.767733	0.126246
hsa_circ_	-0.6407	0.126263
hsa_circ_	0.26009	0.126264
hsa_circ_	-1.20154	0.126271
hsa_circ_	-0.22054	0.126279
hsa_circ_	0.407239	0.126288
hsa_circ_	-0.25547	0.126295
hsa_circ_	0.514177	0.126295
hsa_circ_	-0.58338	0.126302
hsa_circ_	0.404501	0.126303
hsa_circ_	0.418473	0.126304
hsa_circ_	-0.53043	0.126306
hsa_circ_	0.346778	0.126312
hsa_circ_	-0.24875	0.126315
hsa_circ_	-0.25665	0.126346
hsa_circ_	-0.39132	0.126347

hsa_circ_	-0.24865	0.126353
hsa_circ_	0.479255	0.126354
hsa_circ_	-0.32979	0.126373
hsa_circ_	0.396742	0.126374
hsa_circ_	-0.44153	0.126375
hsa_circ_	0.288582	0.126388
hsa_circ_	0.325137	0.126393
hsa_circ_	-0.23796	0.126395
hsa_circ_	0.686247	0.126407
hsa_circ_	-0.31181	0.126415
hsa_circ_	0.529346	0.126423
hsa_circ_	0.3954	0.126425
hsa_circ_	-0.33802	0.126438
hsa_circ_	-0.35811	0.126439
hsa_circ_	0.228272	0.126444
hsa_circ_	-0.59711	0.126445
hsa_circ_	-0.22525	0.126445
hsa_circ_	-0.24839	0.126463
hsa_circ_	-0.2871	0.126465
hsa_circ_	0.796869	0.126476
hsa_circ_	0.469308	0.126483
hsa_circ_	-0.47186	0.126483
hsa_circ_	-0.4899	0.126489
hsa_circ_	0.430442	0.12649
hsa_circ_	-0.23359	0.126509
hsa_circ_	-0.28995	0.126514
hsa_circ_	-0.31218	0.126539
hsa_circ_	-0.36026	0.126542
hsa_circ_	-0.27331	0.126554
hsa_circ_	0.282132	0.12656
hsa_circ_	-0.32603	0.12657
hsa_circ_	-0.23911	0.126573
hsa_circ_	0.382666	0.126577
hsa_circ_	0.283447	0.126581
hsa_circ_	-0.32422	0.126587
hsa_circ_	1.098063	0.126601
hsa_circ_	0.277086	0.12662
hsa_circ_	-0.81668	0.126629
hsa_circ_	0.320443	0.126635
hsa_circ_	-0.97692	0.126635
hsa_circ_	-0.31077	0.126638
hsa_circ_	-0.2489	0.126652
hsa_circ_	-0.24224	0.126652
hsa_circ_	0.338211	0.126665
hsa_circ_	0.501738	0.126678
hsa_circ_	0.518709	0.126695
hsa_circ_	0.406667	0.12671
hsa_circ_	0.590001	0.126736
hsa_circ_	0.303555	0.126741
hsa_circ_	-0.50025	0.126742
hsa_circ_	-0.59661	0.126747
hsa_circ_	0.246857	0.126749
hsa_circ_	0.711133	0.126749
hsa_circ_	-0.30886	0.126771

hsa_circ_	-0.30522	0.126781
hsa_circ_	-0.2658	0.126806
hsa_circ_	-0.24762	0.126826
hsa_circ_	-0.34958	0.126834
hsa_circ_	-1.02136	0.126841
hsa_circ_	0.338459	0.126844
hsa_circ_	-0.56025	0.126847
hsa_circ_	-0.28042	0.126853
hsa_circ_	-0.55314	0.126859
hsa_circ_	0.357889	0.12686
hsa_circ_	0.31972	0.126865
hsa_circ_	0.908081	0.126869
hsa_circ_	0.236296	0.126874
hsa_circ_	-0.31641	0.126888
hsa_circ_	-0.28246	0.1269
hsa_circ_	-0.23248	0.126901
hsa_circ_	-0.30332	0.126909
hsa_circ_	-0.57942	0.126914
hsa_circ_	-0.21381	0.126922
hsa_circ_	0.276277	0.12693
hsa_circ_	0.340451	0.126935
hsa_circ_	-0.54532	0.126943
hsa_circ_	0.356313	0.126947
hsa_circ_	-0.54967	0.12695
hsa_circ_	0.243665	0.12695
hsa_circ_	0.573127	0.12696
hsa_circ_	0.295871	0.126978
hsa_circ_	-0.54183	0.126988
hsa_circ_	-0.36227	0.126998
hsa_circ_	-0.22413	0.127001
hsa_circ_	0.570932	0.127007
hsa_circ_	0.650802	0.127028
hsa_circ_	-0.24556	0.127032
hsa_circ_	-0.70181	0.12704
hsa_circ_	0.661249	0.127043
hsa_circ_	-0.43931	0.127044
hsa_circ_	0.28659	0.127059
hsa_circ_	0.375461	0.127063
hsa_circ_	0.387885	0.127068
hsa_circ_	0.22082	0.127072
hsa_circ_	0.488175	0.127079
hsa_circ_	0.302621	0.12708
hsa_circ_	-0.30279	0.127088
hsa_circ_	0.273659	0.12709
hsa_circ_	-0.30059	0.127099
hsa_circ_	0.270036	0.127101
hsa_circ_	-0.27091	0.127112
hsa_circ_	-0.99199	0.127114
hsa_circ_	-0.24843	0.127133
hsa_circ_	-0.34306	0.127141
hsa_circ_	0.388344	0.127154
hsa_circ_	-0.28371	0.127157
hsa_circ_	0.382546	0.127171
hsa_circ_	-0.52964	0.127173

hsa_circ_	0.532938	0.127184
hsa_circ_	0.440962	0.127197
hsa_circ_	-0.29942	0.127199
hsa_circ_	-0.51	0.127202
hsa_circ_	0.284798	0.127207
hsa_circ_	-0.46216	0.127231
hsa_circ_	-0.30457	0.127235
hsa_circ_	-0.22121	0.127237
hsa_circ_	-0.53923	0.12725
hsa_circ_	0.277228	0.127254
hsa_circ_	0.395019	0.127255
hsa_circ_	0.265971	0.127264
hsa_circ_	-0.31437	0.127289
hsa_circ_	0.269073	0.127301
hsa_circ_	0.366323	0.127306
hsa_circ_	-0.45338	0.127329
hsa_circ_	0.51584	0.127344
hsa_circ_	-1.5546	0.127345
hsa_circ_	-0.73091	0.127394
hsa_circ_	-0.57539	0.127399
hsa_circ_	-0.28025	0.127401
hsa_circ_	-0.3594	0.127411
hsa_circ_	0.970617	0.127422
hsa_circ_	0.345925	0.127425
hsa_circ_	0.31355	0.127427
hsa_circ_	0.290487	0.127431
hsa_circ_	-0.25576	0.127431
hsa_circ_	0.301569	0.127441
hsa_circ_	0.268247	0.127458
hsa_circ_	0.22004	0.127462
hsa_circ_	-2.73765	0.127469
hsa_circ_	1.09361	0.127474
hsa_circ_	0.334616	0.127491
hsa_circ_	0.37733	0.127513
hsa_circ_	-0.49289	0.127515
hsa_circ_	0.269093	0.127532
hsa_circ_	-0.34035	0.12754
hsa_circ_	0.226311	0.127546
hsa_circ_	-0.62856	0.127547
hsa_circ_	-0.56025	0.127547
hsa_circ_	0.257305	0.127548
hsa_circ_	0.257634	0.127553
hsa_circ_	0.311233	0.127563
hsa_circ_	0.264057	0.127574
hsa_circ_	-0.304	0.127586
hsa_circ_	0.465095	0.127591
hsa_circ_	0.354045	0.127601
hsa_circ_	-0.45649	0.127607
hsa_circ_	0.217476	0.127628
hsa_circ_	0.366913	0.12763
hsa_circ_	0.69822	0.127641
hsa_circ_	-0.31621	0.127648
hsa_circ_	0.542726	0.12765
hsa_circ_	0.592179	0.127654

hsa_circ_	0.247334	0.127698
hsa_circ_	0.457565	0.1277
hsa_circ_	-0.8237	0.127702
hsa_circ_	0.451043	0.127712
hsa_circ_	0.857897	0.127715
hsa_circ_	0.381881	0.127731
hsa_circ_	0.56608	0.127735
hsa_circ_	0.284469	0.127757
hsa_circ_	0.341106	0.12777
hsa_circ_	-0.6557	0.127779
hsa_circ_	-0.2436	0.127779
hsa_circ_	0.261079	0.12778
hsa_circ_	-0.31124	0.127791
hsa_circ_	-0.30771	0.127801
hsa_circ_	0.526889	0.127803
hsa_circ_	0.283722	0.127804
hsa_circ_	0.285532	0.127805
hsa_circ_	-0.25949	0.127811
hsa_circ_	-0.31723	0.127811
hsa_circ_	-0.26781	0.127816
hsa_circ_	0.312485	0.127816
hsa_circ_	-0.4785	0.127838
hsa_circ_	-0.43476	0.127848
hsa_circ_	0.324258	0.127849
hsa_circ_	-0.22206	0.127852
hsa_circ_	0.257038	0.127858
hsa_circ_	-0.39428	0.127863
hsa_circ_	0.497923	0.127875
hsa_circ_	0.919687	0.127876
hsa_circ_	0.302409	0.127887
hsa_circ_	0.241087	0.127893
hsa_circ_	0.440841	0.127903
hsa_circ_	0.428247	0.127904
hsa_circ_	-0.23398	0.127919
hsa_circ_	0.332251	0.127927
hsa_circ_	-0.26349	0.127927
hsa_circ_	-0.64705	0.127936
hsa_circ_	-0.28722	0.127949
hsa_circ_	0.256972	0.127951
hsa_circ_	-0.49181	0.127964
hsa_circ_	0.499696	0.127991
hsa_circ_	-0.54753	0.127992
hsa_circ_	-0.33678	0.127994
hsa_circ_	-0.75137	0.127995
hsa_circ_	-0.33837	0.127996
hsa_circ_	-0.47306	0.128001
hsa_circ_	-0.38614	0.12801
hsa_circ_	0.239485	0.128027
hsa_circ_	-0.42482	0.128027
hsa_circ_	0.294626	0.128035
hsa_circ_	-0.28612	0.128058
hsa_circ_	-0.35657	0.128063
hsa_circ_	0.248185	0.12807
hsa_circ_	0.574462	0.12807

hsa_circ_	-0.27345	0.128087
hsa_circ_	-0.36273	0.128088
hsa_circ_	0.294878	0.128093
hsa_circ_	-0.29626	0.128095
hsa_circ_	-0.25822	0.128096
hsa_circ_	-0.22583	0.128098
hsa_circ_	-0.55019	0.128108
hsa_circ_	-0.34545	0.128118
hsa_circ_	0.246659	0.128118
hsa_circ_	0.249798	0.128122
hsa_circ_	-0.29675	0.128126
hsa_circ_	-0.22523	0.128129
hsa_circ_	-0.32594	0.128141
hsa_circ_	0.609356	0.128155
hsa_circ_	-0.25027	0.128174
hsa_circ_	-0.34524	0.128182
hsa_circ_	-0.81008	0.12819
hsa_circ_	0.463583	0.128193
hsa_circ_	0.229151	0.128194
hsa_circ_	-0.24409	0.128195
hsa_circ_	-0.58036	0.128199
hsa_circ_	0.236143	0.128203
hsa_circ_	-0.39706	0.128212
hsa_circ_	1.179127	0.128226
hsa_circ_	-0.38521	0.128247
hsa_circ_	-0.29913	0.128248
hsa_circ_	-0.21596	0.128252
hsa_circ_	-0.40003	0.128252
hsa_circ_	-0.25396	0.128277
hsa_circ_	-0.30847	0.128282
hsa_circ_	-0.32442	0.128283
hsa_circ_	-0.25088	0.128283
hsa_circ_	0.328171	0.128289
hsa_circ_	0.268567	0.128317
hsa_circ_	0.31108	0.128323
hsa_circ_	0.369532	0.128326
hsa_circ_	-1.30091	0.12834
hsa_circ_	-0.27201	0.128342
hsa_circ_	0.485494	0.128353
hsa_circ_	-0.32038	0.128359
hsa_circ_	-0.31132	0.128375
hsa_circ_	0.418246	0.12838
hsa_circ_	-0.24239	0.128381
hsa_circ_	0.340365	0.128383
hsa_circ_	-0.3059	0.128387
hsa_circ_	0.639195	0.128388
hsa_circ_	0.261426	0.128405
hsa_circ_	-0.35287	0.128407
hsa_circ_	-0.23573	0.128422
hsa_circ_	-0.35074	0.128423
hsa_circ_	0.376747	0.128427
hsa_circ_	0.806018	0.128452
hsa_circ_	0.47058	0.128456
hsa_circ_	0.266386	0.128473

hsa_circ_	0.421743	0.128489
hsa_circ_	-0.24773	0.128492
hsa_circ_	-0.27091	0.128496
hsa_circ_	0.309878	0.128513
hsa_circ_	0.287657	0.128517
hsa_circ_	-0.43359	0.128527
hsa_circ_	0.26211	0.128551
hsa_circ_	0.487206	0.128568
hsa_circ_	0.26291	0.12858
hsa_circ_	0.957669	0.128581
hsa_circ_	0.509369	0.128589
hsa_circ_	0.603782	0.128604
hsa_circ_	-0.7236	0.128606
hsa_circ_	0.449166	0.128609
hsa_circ_	0.218358	0.128616
hsa_circ_	0.462515	0.12862
hsa_circ_	0.215364	0.128632
hsa_circ_	0.441568	0.128634
hsa_circ_	0.457788	0.128645
hsa_circ_	-0.26124	0.128645
hsa_circ_	0.269626	0.128659
hsa_circ_	-0.29848	0.128678
hsa_circ_	-0.25728	0.128699
hsa_circ_	0.268216	0.128707
hsa_circ_	0.324923	0.128723
hsa_circ_	0.475213	0.128725
hsa_circ_	-0.32186	0.128739
hsa_circ_	0.295725	0.128758
hsa_circ_	0.314055	0.128777
hsa_circ_	0.55963	0.12879
hsa_circ_	0.683476	0.128792
hsa_circ_	-0.64248	0.128793
hsa_circ_	-0.26425	0.128795
hsa_circ_	0.711907	0.1288
hsa_circ_	0.339902	0.128806
hsa_circ_	0.354363	0.128813
hsa_circ_	-0.34588	0.128824
hsa_circ_	0.958804	0.128825
hsa_circ_	0.326696	0.128835
hsa_circ_	0.490633	0.128845
hsa_circ_	-0.34842	0.128854
hsa_circ_	1.49688	0.128868
hsa_circ_	0.230412	0.128882
hsa_circ_	0.271386	0.128889
hsa_circ_	0.480215	0.128895
hsa_circ_	0.628057	0.128901
hsa_circ_	-0.31533	0.128904
hsa_circ_	-0.40251	0.128905
hsa_circ_	-0.94626	0.128915
hsa_circ_	0.356479	0.128934
hsa_circ_	0.442156	0.128943
hsa_circ_	-0.40004	0.128972
hsa_circ_	0.874717	0.128973
hsa_circ_	0.335447	0.128988

hsa_circ_	-0.25361	0.128989
hsa_circ_	0.92853	0.128992
hsa_circ_	0.257845	0.128993
hsa_circ_	-0.45381	0.128994
hsa_circ_	0.36464	0.128998
hsa_circ_	-0.24335	0.129
hsa_circ_	0.264616	0.129006
hsa_circ_	-1.67939	0.129007
hsa_circ_	0.239564	0.129009
hsa_circ_	-0.34073	0.129014
hsa_circ_	0.272223	0.129021
hsa_circ_	0.240468	0.129027
hsa_circ_	0.390491	0.129034
hsa_circ_	0.568399	0.129042
hsa_circ_	-1.12906	0.129065
hsa_circ_	-0.32178	0.129073
hsa_circ_	0.428243	0.12908
hsa_circ_	-0.23509	0.129088
hsa_circ_	-0.28163	0.129097
hsa_circ_	0.502723	0.129101
hsa_circ_	0.237887	0.129128
hsa_circ_	0.518421	0.12913
hsa_circ_	-0.21324	0.129131
hsa_circ_	-0.50812	0.129136
hsa_circ_	0.459269	0.129136
hsa_circ_	0.489886	0.129139
hsa_circ_	0.361846	0.129165
hsa_circ_	-0.35553	0.129169
hsa_circ_	-0.33007	0.129185
hsa_circ_	1.096629	0.129187
hsa_circ_	-0.26068	0.129231
hsa_circ_	-0.25461	0.129237
hsa_circ_	-0.2746	0.129237
hsa_circ_	-0.29466	0.129242
hsa_circ_	0.317344	0.129257
hsa_circ_	0.724617	0.129258
hsa_circ_	-0.2342	0.129269
hsa_circ_	0.536151	0.129272
hsa_circ_	0.718833	0.129278
hsa_circ_	0.313233	0.129288
hsa_circ_	0.44686	0.12929
hsa_circ_	0.304239	0.129296
hsa_circ_	0.366198	0.129329
hsa_circ_	0.303039	0.129335
hsa_circ_	-0.92194	0.129342
hsa_circ_	0.458577	0.129355
hsa_circ_	-0.43796	0.129356
hsa_circ_	0.435654	0.129366
hsa_circ_	0.30036	0.129367
hsa_circ_	0.418685	0.129377
hsa_circ_	0.312513	0.129378
hsa_circ_	0.9455	0.129384
hsa_circ_	0.363459	0.129385
hsa_circ_	-0.43381	0.129396

hsa_circ_	0.376364	0.129398
hsa_circ_	-0.74739	0.129414
hsa_circ_	0.249722	0.129416
hsa_circ_	-0.28887	0.129421
hsa_circ_	0.378068	0.129421
hsa_circ_	0.306338	0.129422
hsa_circ_	0.441993	0.129442
hsa_circ_	-0.63026	0.129455
hsa_circ_	-0.303	0.129458
hsa_circ_	0.361136	0.129461
hsa_circ_	0.771023	0.129471
hsa_circ_	0.303724	0.129475
hsa_circ_	-0.43564	0.129477
hsa_circ_	0.549717	0.129478
hsa_circ_	-0.62576	0.12948
hsa_circ_	-0.28642	0.129491
hsa_circ_	0.296557	0.129496
hsa_circ_	0.354965	0.129498
hsa_circ_	-0.36967	0.129517
hsa_circ_	0.29948	0.129522
hsa_circ_	-0.5036	0.129525
hsa_circ_	-0.79943	0.129532
hsa_circ_	0.37763	0.129543
hsa_circ_	-0.30586	0.129546
hsa_circ_	0.820705	0.129552
hsa_circ_	-0.24193	0.129563
hsa_circ_	0.486423	0.129568
hsa_circ_	0.477999	0.129571
hsa_circ_	-0.3993	0.129594
hsa_circ_	0.341994	0.129596
hsa_circ_	-0.39128	0.129602
hsa_circ_	-1.09857	0.129611
hsa_circ_	0.24059	0.129614
hsa_circ_	1.101382	0.129626
hsa_circ_	-0.22097	0.129644
hsa_circ_	-0.63124	0.129647
hsa_circ_	-0.62158	0.129648
hsa_circ_	-0.92999	0.129652
hsa_circ_	-0.26976	0.129677
hsa_circ_	-0.29056	0.12968
hsa_circ_	0.282182	0.129686
hsa_circ_	-0.80163	0.129695
hsa_circ_	0.410902	0.129696
hsa_circ_	-0.37693	0.129709
hsa_circ_	0.253811	0.12974
hsa_circ_	0.437597	0.129743
hsa_circ_	-1.36615	0.129794
hsa_circ_	0.353154	0.129804
hsa_circ_	0.483158	0.129807
hsa_circ_	0.32846	0.12981
hsa_circ_	-0.27943	0.129817
hsa_circ_	0.305574	0.129823
hsa_circ_	0.236574	0.129829
hsa_circ_	0.342277	0.12983

hsa_circ_	-0.23809	0.12985
hsa_circ_	-0.3882	0.129851
hsa_circ_	-0.29816	0.129877
hsa_circ_	0.436888	0.129879
hsa_circ_	0.415347	0.129879
hsa_circ_	0.898341	0.12988
hsa_circ_	0.869198	0.129883
hsa_circ_	0.565133	0.129888
hsa_circ_	0.325462	0.129902
hsa_circ_	-0.3686	0.129904
hsa_circ_	-0.77897	0.129917
hsa_circ_	1.031446	0.129943
hsa_circ_	1.134678	0.129946
hsa_circ_	0.296107	0.129955
hsa_circ_	0.810996	0.129955
hsa_circ_	-1.43617	0.129968
hsa_circ_	-0.33548	0.129983
hsa_circ_	0.299682	0.129995
hsa_circ_	-0.34556	0.130008
hsa_circ_	0.284352	0.130008
hsa_circ_	0.257156	0.13001
hsa_circ_	-0.23001	0.130011
hsa_circ_	0.40608	0.130012
hsa_circ_	0.337552	0.130013
hsa_circ_	0.915095	0.130016
hsa_circ_	0.580111	0.130023
hsa_circ_	-0.27671	0.130074
hsa_circ_	-0.42177	0.130084
hsa_circ_	-0.55398	0.130087
hsa_circ_	0.380752	0.130087
hsa_circ_	-0.29252	0.130088
hsa_circ_	-0.74102	0.13009
hsa_circ_	0.483102	0.1301
hsa_circ_	-0.32111	0.130106
hsa_circ_	-0.31849	0.130116
hsa_circ_	0.367553	0.130117
hsa_circ_	-0.27943	0.130123
hsa_circ_	0.340049	0.130127
hsa_circ_	0.372317	0.130128
hsa_circ_	-0.70468	0.130129
hsa_circ_	0.675386	0.13013
hsa_circ_	0.345147	0.130134
hsa_circ_	-0.24886	0.130138
hsa_circ_	0.343657	0.130154
hsa_circ_	0.546642	0.130156
hsa_circ_	0.304326	0.130169
hsa_circ_	-0.25828	0.13017
hsa_circ_	0.260305	0.130194
hsa_circ_	0.448526	0.130199
hsa_circ_	-0.35066	0.130201
hsa_circ_	0.237037	0.130205
hsa_circ_	0.319545	0.13022
hsa_circ_	-0.28347	0.130234
hsa_circ_	0.322422	0.130234

hsa_circ_	0.378065	0.130239
hsa_circ_	0.544768	0.130239
hsa_circ_	0.228575	0.13024
hsa_circ_	0.315375	0.130246
hsa_circ_	-1.51659	0.130247
hsa_circ_	-0.86536	0.130248
hsa_circ_	0.421683	0.130251
hsa_circ_	0.412746	0.13026
hsa_circ_	-0.2143	0.13026
hsa_circ_	-0.3071	0.130263
hsa_circ_	-0.85307	0.130278
hsa_circ_	0.237664	0.130296
hsa_circ_	0.564088	0.130299
hsa_circ_	-0.52004	0.130302
hsa_circ_	-0.32883	0.130303
hsa_circ_	-0.65513	0.130303
hsa_circ_	0.267796	0.130313
hsa_circ_	-0.24131	0.130324
hsa_circ_	-0.79252	0.130328
hsa_circ_	0.302869	0.130333
hsa_circ_	0.227687	0.130334
hsa_circ_	-0.38866	0.130336
hsa_circ_	-0.38133	0.130344
hsa_circ_	0.286039	0.130344
hsa_circ_	0.349081	0.130345
hsa_circ_	-0.91961	0.130362
hsa_circ_	-0.26107	0.130379
hsa_circ_	-0.68748	0.130388
hsa_circ_	-0.29994	0.130389
hsa_circ_	-0.5598	0.130403
hsa_circ_	-0.3824	0.130407
hsa_circ_	0.530027	0.130415
hsa_circ_	-0.3233	0.130429
hsa_circ_	0.306037	0.130446
hsa_circ_	-0.51181	0.13045
hsa_circ_	0.312847	0.130459
hsa_circ_	-0.40517	0.130472
hsa_circ_	0.408614	0.130504
hsa_circ_	-0.46141	0.13051
hsa_circ_	0.439041	0.130527
hsa_circ_	0.420616	0.13054
hsa_circ_	-0.23689	0.130542
hsa_circ_	-0.47198	0.130553
hsa_circ_	0.277822	0.130566
hsa_circ_	0.298934	0.130578
hsa_circ_	0.3947	0.130579
hsa_circ_	-1.24662	0.130585
hsa_circ_	-0.25598	0.130588
hsa_circ_	0.27984	0.130592
hsa_circ_	0.22891	0.130627
hsa_circ_	0.332541	0.130637
hsa_circ_	-0.25327	0.130637
hsa_circ_	0.309121	0.13064
hsa_circ_	-0.69882	0.130645

hsa_circ_	0.236327	0.130653
hsa_circ_	-0.29444	0.130658
hsa_circ_	-0.40125	0.13066
hsa_circ_	-0.25616	0.13067
hsa_circ_	0.321353	0.130687
hsa_circ_	-0.32487	0.130689
hsa_circ_	-0.22663	0.130702
hsa_circ_	-0.49046	0.130712
hsa_circ_	-0.35761	0.130715
hsa_circ_	0.390438	0.130718
hsa_circ_	-0.22547	0.130722
hsa_circ_	-0.31551	0.130723
hsa_circ_	-0.33153	0.130726
hsa_circ_	0.244797	0.130729
hsa_circ_	-0.56092	0.130729
hsa_circ_	-0.61604	0.130733
hsa_circ_	-0.34735	0.130735
hsa_circ_	0.339663	0.13074
hsa_circ_	0.311817	0.130748
hsa_circ_	-0.32206	0.130754
hsa_circ_	0.24481	0.130807
hsa_circ_	-0.35746	0.130807
hsa_circ_	1.505713	0.130809
hsa_circ_	0.412269	0.130809
hsa_circ_	0.285163	0.130822
hsa_circ_	-0.29775	0.130822
hsa_circ_	-0.68044	0.130838
hsa_circ_	0.303524	0.130839
hsa_circ_	0.2745	0.130841
hsa_circ_	0.459003	0.130858
hsa_circ_	0.275312	0.130861
hsa_circ_	-0.38696	0.130864
hsa_circ_	-0.34337	0.130874
hsa_circ_	0.420072	0.130882
hsa_circ_	-0.31223	0.130883
hsa_circ_	-0.26614	0.130894
hsa_circ_	0.696017	0.130897
hsa_circ_	0.511298	0.130903
hsa_circ_	-0.39053	0.130919
hsa_circ_	-0.34215	0.130932
hsa_circ_	-0.26419	0.130937
hsa_circ_	-0.3663	0.130937
hsa_circ_	-0.32145	0.130948
hsa_circ_	0.23378	0.130956
hsa_circ_	0.241731	0.130975
hsa_circ_	-0.46526	0.130977
hsa_circ_	0.764804	0.130986
hsa_circ_	0.300543	0.130989
hsa_circ_	-0.40025	0.131004
hsa_circ_	-0.26582	0.13101
hsa_circ_	0.515827	0.131028
hsa_circ_	0.927768	0.13103
hsa_circ_	-0.41607	0.131056
hsa_circ_	0.264405	0.131057

hsa_circ_	0.56903	0.131061
hsa_circ_	-0.69927	0.131062
hsa_circ_	-0.32382	0.131078
hsa_circ_	-0.74367	0.131101
hsa_circ_	-0.2964	0.131106
hsa_circ_	0.266727	0.131115
hsa_circ_	-0.42103	0.131116
hsa_circ_	-0.23719	0.131121
hsa_circ_	-0.24213	0.131127
hsa_circ_	0.264485	0.131127
hsa_circ_	0.629712	0.131157
hsa_circ_	0.2437	0.13116
hsa_circ_	0.334778	0.131166
hsa_circ_	0.594402	0.131191
hsa_circ_	-0.47536	0.131199
hsa_circ_	0.269626	0.131219
hsa_circ_	0.602935	0.131235
hsa_circ_	0.707324	0.131239
hsa_circ_	0.346178	0.131241
hsa_circ_	0.682149	0.131265
hsa_circ_	0.224207	0.131267
hsa_circ_	0.27969	0.131283
hsa_circ_	1.226107	0.131285
hsa_circ_	0.343983	0.131286
hsa_circ_	-0.26523	0.131293
hsa_circ_	-0.62161	0.1313
hsa_circ_	0.721458	0.131303
hsa_circ_	-0.83154	0.131313
hsa_circ_	-0.23219	0.131329
hsa_circ_	-0.29266	0.131348
hsa_circ_	-0.26048	0.131359
hsa_circ_	-0.36573	0.13138
hsa_circ_	-0.218	0.131383
hsa_circ_	1.128705	0.131383
hsa_circ_	-0.2744	0.13141
hsa_circ_	-0.7281	0.131421
hsa_circ_	-0.30816	0.13144
hsa_circ_	-0.29121	0.131442
hsa_circ_	-0.32014	0.131451
hsa_circ_	0.259388	0.131468
hsa_circ_	0.543771	0.131469
hsa_circ_	0.357054	0.13149
hsa_circ_	0.394313	0.131503
hsa_circ_	-0.5222	0.131504
hsa_circ_	0.358825	0.131514
hsa_circ_	0.319937	0.131522
hsa_circ_	0.313585	0.13156
hsa_circ_	-0.28591	0.131562
hsa_circ_	-0.2343	0.131562
hsa_circ_	-0.33875	0.131562
hsa_circ_	-0.5245	0.13157
hsa_circ_	-0.30003	0.131575
hsa_circ_	0.819299	0.13158
hsa_circ_	0.423483	0.131588

hsa_circ_	-0.61267	0.131589
hsa_circ_	0.460998	0.131622
hsa_circ_	-0.50992	0.131626
hsa_circ_	0.482858	0.131628
hsa_circ_	0.431422	0.131638
hsa_circ_	-0.24875	0.131649
hsa_circ_	-0.41738	0.131655
hsa_circ_	0.790524	0.131673
hsa_circ_	-0.2704	0.131675
hsa_circ_	0.377742	0.131684
hsa_circ_	0.440511	0.131689
hsa_circ_	0.276336	0.131699
hsa_circ_	-0.38116	0.131699
hsa_circ_	0.215054	0.131701
hsa_circ_	0.279667	0.13172
hsa_circ_	-0.45527	0.131721
hsa_circ_	0.303897	0.131725
hsa_circ_	-0.32751	0.131726
hsa_circ_	0.310825	0.131734
hsa_circ_	0.483813	0.131737
hsa_circ_	0.362927	0.131739
hsa_circ_	0.252881	0.131745
hsa_circ_	0.49548	0.131749
hsa_circ_	-0.21158	0.131749
hsa_circ_	-0.25888	0.131754
hsa_circ_	0.667535	0.131761
hsa_circ_	0.333507	0.131773
hsa_circ_	0.872824	0.131774
hsa_circ_	0.571877	0.131775
hsa_circ_	0.305964	0.131784
hsa_circ_	0.785472	0.131785
hsa_circ_	0.238109	0.131786
hsa_circ_	-0.42161	0.131794
hsa_circ_	0.393806	0.131807
hsa_circ_	0.256812	0.131808
hsa_circ_	-0.26649	0.131845
hsa_circ_	-0.32953	0.131853
hsa_circ_	-0.48721	0.131858
hsa_circ_	-0.46969	0.131865
hsa_circ_	-0.70122	0.131866
hsa_circ_	-0.86729	0.131891
hsa_circ_	1.113842	0.131908
hsa_circ_	0.240892	0.13191
hsa_circ_	0.809855	0.131914
hsa_circ_	-0.41718	0.131916
hsa_circ_	-0.31316	0.131921
hsa_circ_	1.07912	0.131926
hsa_circ_	0.803969	0.131947
hsa_circ_	-0.2489	0.131955
hsa_circ_	0.260114	0.131968
hsa_circ_	0.279779	0.132003
hsa_circ_	0.234811	0.132004
hsa_circ_	0.630291	0.132018
hsa_circ_	0.396024	0.132046

hsa_circ_	-0.40857	0.132046
hsa_circ_	0.245784	0.132046
hsa_circ_	-0.26195	0.132047
hsa_circ_	0.267994	0.132069
hsa_circ_	0.603435	0.132084
hsa_circ_	0.30002	0.132092
hsa_circ_	-1.46742	0.132093
hsa_circ_	0.213171	0.132095
hsa_circ_	-0.27659	0.132114
hsa_circ_	-0.32748	0.132114
hsa_circ_	-0.22403	0.132116
hsa_circ_	-0.4455	0.132119
hsa_circ_	-0.32281	0.132121
hsa_circ_	0.404792	0.132129
hsa_circ_	0.646847	0.132141
hsa_circ_	-0.82649	0.132143
hsa_circ_	0.723461	0.132152
hsa_circ_	-0.35007	0.132152
hsa_circ_	0.43039	0.13217
hsa_circ_	-0.40179	0.132197
hsa_circ_	-0.36876	0.132199
hsa_circ_	-0.44694	0.132201
hsa_circ_	-0.51672	0.132204
hsa_circ_	0.617003	0.132214
hsa_circ_	-0.29205	0.132217
hsa_circ_	-0.3581	0.132221
hsa_circ_	0.305731	0.132223
hsa_circ_	-0.45616	0.132223
hsa_circ_	0.307169	0.13224
hsa_circ_	0.257259	0.132243
hsa_circ_	0.257985	0.132261
hsa_circ_	0.458293	0.132271
hsa_circ_	0.298616	0.132274
hsa_circ_	0.312281	0.132289
hsa_circ_	-0.29955	0.132301
hsa_circ_	-0.28667	0.132301
hsa_circ_	0.875697	0.132302
hsa_circ_	0.321951	0.132303
hsa_circ_	-0.45497	0.132313
hsa_circ_	-0.50354	0.132319
hsa_circ_	0.426363	0.13232
hsa_circ_	-0.43453	0.13232
hsa_circ_	-0.46105	0.13232
hsa_circ_	0.281746	0.132335
hsa_circ_	-0.33808	0.132337
hsa_circ_	-1.19869	0.132345
hsa_circ_	0.316901	0.132375
hsa_circ_	0.226551	0.132384
hsa_circ_	-0.23966	0.132387
hsa_circ_	0.308024	0.132389
hsa_circ_	0.247738	0.132391
hsa_circ_	-0.29979	0.132407
hsa_circ_	0.251688	0.13241
hsa_circ_	0.595846	0.132411

hsa_circ_	0.306818	0.132417
hsa_circ_	-0.43195	0.132424
hsa_circ_	0.337084	0.132424
hsa_circ_	0.346322	0.132425
hsa_circ_	0.729814	0.132439
hsa_circ_	-0.24517	0.132455
hsa_circ_	-0.3149	0.132457
hsa_circ_	0.896061	0.132468
hsa_circ_	-0.50181	0.13247
hsa_circ_	-0.26603	0.132482
hsa_circ_	0.267324	0.132491
hsa_circ_	0.347007	0.132492
hsa_circ_	-0.57675	0.132494
hsa_circ_	0.416806	0.132507
hsa_circ_	-0.37721	0.132519
hsa_circ_	1.207147	0.132521
hsa_circ_	0.80461	0.132522
hsa_circ_	0.533156	0.132528
hsa_circ_	-0.72963	0.132541
hsa_circ_	-0.3365	0.132545
hsa_circ_	-0.28166	0.132551
hsa_circ_	-0.259	0.132569
hsa_circ_	-0.36284	0.13257
hsa_circ_	-0.28424	0.132571
hsa_circ_	0.401889	0.132582
hsa_circ_	0.283745	0.132584
hsa_circ_	-0.56878	0.13259
hsa_circ_	-0.26018	0.132596
hsa_circ_	-0.33172	0.132609
hsa_circ_	0.851375	0.132611
hsa_circ_	-0.29015	0.132615
hsa_circ_	-0.22539	0.132628
hsa_circ_	0.600921	0.132637
hsa_circ_	-0.42673	0.132642
hsa_circ_	-0.24692	0.132648
hsa_circ_	0.236451	0.13265
hsa_circ_	0.211487	0.132654
hsa_circ_	-0.44813	0.132656
hsa_circ_	0.312453	0.132665
hsa_circ_	0.226369	0.13267
hsa_circ_	-0.79462	0.132678
hsa_circ_	-0.30245	0.13268
hsa_circ_	0.2819	0.132683
hsa_circ_	0.309439	0.132687
hsa_circ_	-0.22863	0.132698
hsa_circ_	0.287648	0.132712
hsa_circ_	-0.37208	0.132713
hsa_circ_	-0.41587	0.132721
hsa_circ_	0.242836	0.132734
hsa_circ_	0.306941	0.132734
hsa_circ_	-0.40844	0.132736
hsa_circ_	-0.27403	0.13275
hsa_circ_	0.28444	0.132751
hsa_circ_	0.468236	0.132755

hsa_circ_	0.322525	0.132759
hsa_circ_	0.853254	0.13276
hsa_circ_	-0.41738	0.132763
hsa_circ_	0.281713	0.132787
hsa_circ_	0.555138	0.132816
hsa_circ_	0.238982	0.13282
hsa_circ_	0.938107	0.132823
hsa_circ_	-0.26423	0.132835
hsa_circ_	0.246995	0.132836
hsa_circ_	0.496534	0.132836
hsa_circ_	-0.38035	0.132852
hsa_circ_	0.909955	0.132855
hsa_circ_	-0.25546	0.132858
hsa_circ_	-0.30606	0.132869
hsa_circ_	0.621613	0.132876
hsa_circ_	-0.47084	0.132896
hsa_circ_	-0.31223	0.132905
hsa_circ_	-0.262	0.132908
hsa_circ_	0.347243	0.132911
hsa_circ_	0.258456	0.132913
hsa_circ_	-0.30706	0.132915
hsa_circ_	-0.5904	0.132919
hsa_circ_	-0.47517	0.132923
hsa_circ_	0.783706	0.132931
hsa_circ_	-0.21506	0.132932
hsa_circ_	-0.21089	0.132981
hsa_circ_	-0.30935	0.132981
hsa_circ_	0.245231	0.132987
hsa_circ_	-0.23807	0.132995
hsa_circ_	-0.29962	0.13301
hsa_circ_	-0.28012	0.133013
hsa_circ_	-0.24448	0.133019
hsa_circ_	0.507828	0.13302
hsa_circ_	-0.47592	0.133029
hsa_circ_	0.382428	0.133044
hsa_circ_	-0.45774	0.133051
hsa_circ_	-0.65101	0.133055
hsa_circ_	0.321046	0.13306
hsa_circ_	0.396922	0.133065
hsa_circ_	-0.50243	0.133069
hsa_circ_	-0.27093	0.133072
hsa_circ_	0.400385	0.133077
hsa_circ_	0.336427	0.133079
hsa_circ_	0.311799	0.133093
hsa_circ_	0.730545	0.133101
hsa_circ_	-0.2512	0.133113
hsa_circ_	-0.46607	0.133137
hsa_circ_	-0.75051	0.133139
hsa_circ_	0.364183	0.133139
hsa_circ_	-0.57249	0.133142
hsa_circ_	0.371643	0.133147
hsa_circ_	0.347337	0.133163
hsa_circ_	0.401431	0.133174
hsa_circ_	-0.37384	0.133181

hsa_circ_	-0.37144	0.133182
hsa_circ_	0.248047	0.133183
hsa_circ_	0.276932	0.133186
hsa_circ_	0.277704	0.133186
hsa_circ_	-0.55794	0.133189
hsa_circ_	0.352794	0.133211
hsa_circ_	-0.22648	0.133224
hsa_circ_	-0.27015	0.133234
hsa_circ_	-0.27182	0.133247
hsa_circ_	0.582763	0.13326
hsa_circ_	-0.46999	0.133287
hsa_circ_	-0.36152	0.133297
hsa_circ_	0.309152	0.133301
hsa_circ_	0.550284	0.133302
hsa_circ_	-0.28161	0.133313
hsa_circ_	-0.73133	0.133333
hsa_circ_	0.2665	0.133336
hsa_circ_	-0.27624	0.133338
hsa_circ_	-0.87109	0.133345
hsa_circ_	0.330446	0.133349
hsa_circ_	1.010323	0.133356
hsa_circ_	0.585955	0.133365
hsa_circ_	-0.31164	0.133373
hsa_circ_	0.244953	0.133374
hsa_circ_	-0.23277	0.133403
hsa_circ_	0.294678	0.133404
hsa_circ_	-0.27355	0.133407
hsa_circ_	-0.32312	0.133423
hsa_circ_	0.254903	0.133425
hsa_circ_	-0.70999	0.133435
hsa_circ_	-0.28922	0.133435
hsa_circ_	-0.34455	0.133437
hsa_circ_	0.304331	0.133437
hsa_circ_	-0.45354	0.133439
hsa_circ_	-0.54366	0.13344
hsa_circ_	0.210923	0.133442
hsa_circ_	-0.23486	0.133468
hsa_circ_	0.338242	0.13347
hsa_circ_	-0.29196	0.133471
hsa_circ_	-0.22426	0.133472
hsa_circ_	-0.6819	0.133477
hsa_circ_	0.41712	0.133478
hsa_circ_	-0.27407	0.133481
hsa_circ_	0.358197	0.133482
hsa_circ_	0.400645	0.133493
hsa_circ_	-0.34157	0.133507
hsa_circ_	0.456588	0.133516
hsa_circ_	-0.642	0.133517
hsa_circ_	0.228699	0.133522
hsa_circ_	0.54899	0.133524
hsa_circ_	-0.5227	0.133525
hsa_circ_	-0.6456	0.133569
hsa_circ_	-0.24998	0.133579
hsa_circ_	-0.25481	0.133598

hsa_circ_	0.36179	0.133609
hsa_circ_	0.257703	0.133614
hsa_circ_	-0.39624	0.133621
hsa_circ_	0.271158	0.133627
hsa_circ_	0.874623	0.133627
hsa_circ_	-0.71951	0.133636
hsa_circ_	0.519832	0.133665
hsa_circ_	0.256924	0.133666
hsa_circ_	-0.70745	0.133682
hsa_circ_	-0.29269	0.133684
hsa_circ_	-0.42367	0.1337
hsa_circ_	-0.22681	0.133725
hsa_circ_	-0.34739	0.133729
hsa_circ_	0.291372	0.133731
hsa_circ_	-0.23256	0.133737
hsa_circ_	-0.86114	0.133743
hsa_circ_	0.442567	0.133744
hsa_circ_	-0.22301	0.133751
hsa_circ_	0.22987	0.133753
hsa_circ_	0.23885	0.133754
hsa_circ_	0.301375	0.13376
hsa_circ_	0.631999	0.133769
hsa_circ_	0.251357	0.133781
hsa_circ_	0.424198	0.133785
hsa_circ_	0.751581	0.133792
hsa_circ_	0.319258	0.133796
hsa_circ_	0.519296	0.133806
hsa_circ_	0.587082	0.133828
hsa_circ_	0.237505	0.133836
hsa_circ_	0.246245	0.133842
hsa_circ_	-0.27245	0.133848
hsa_circ_	0.369553	0.133848
hsa_circ_	0.352319	0.133852
hsa_circ_	-0.74655	0.133854
hsa_circ_	-0.27134	0.133876
hsa_circ_	0.887269	0.133883
hsa_circ_	0.276888	0.133888
hsa_circ_	-0.37343	0.133902
hsa_circ_	-0.54215	0.133906
hsa_circ_	0.305191	0.133912
hsa_circ_	0.245324	0.133944
hsa_circ_	-1.03495	0.13395
hsa_circ_	-0.28351	0.133957
hsa_circ_	-0.55866	0.133974
hsa_circ_	-0.37632	0.133978
hsa_circ_	-1.0824	0.133978
hsa_circ_	0.366162	0.133981
hsa_circ_	0.246313	0.133982
hsa_circ_	-0.69657	0.133992
hsa_circ_	0.479771	0.13402
hsa_circ_	0.437757	0.134021
hsa_circ_	-0.27913	0.134023
hsa_circ_	-0.27682	0.134026
hsa_circ_	-0.5804	0.134031

hsa_circ_	0.549016	0.134048
hsa_circ_	-0.27336	0.134053
hsa_circ_	0.325718	0.134073
hsa_circ_	0.315378	0.134078
hsa_circ_	0.30857	0.134088
hsa_circ_	-1.1686	0.134098
hsa_circ_	-0.72602	0.134116
hsa_circ_	-0.25198	0.134121
hsa_circ_	0.258163	0.134127
hsa_circ_	-0.87164	0.134138
hsa_circ_	0.311413	0.13414
hsa_circ_	0.427963	0.134149
hsa_circ_	-0.21393	0.134172
hsa_circ_	-0.49274	0.134177
hsa_circ_	-0.27943	0.134198
hsa_circ_	0.590379	0.134205
hsa_circ_	0.378624	0.134211
hsa_circ_	0.334631	0.134215
hsa_circ_	-0.5074	0.134234
hsa_circ_	-0.31245	0.13424
hsa_circ_	0.322744	0.134242
hsa_circ_	0.462513	0.134256
hsa_circ_	0.257154	0.134265
hsa_circ_	-0.57653	0.1343
hsa_circ_	-0.2318	0.134303
hsa_circ_	0.253703	0.134312
hsa_circ_	0.466524	0.13433
hsa_circ_	1.014777	0.134333
hsa_circ_	-0.30565	0.134335
hsa_circ_	0.209628	0.134338
hsa_circ_	0.312477	0.134341
hsa_circ_	-0.4095	0.13436
hsa_circ_	-0.23248	0.134361
hsa_circ_	-0.28979	0.134371
hsa_circ_	0.400902	0.134374
hsa_circ_	-0.48711	0.134386
hsa_circ_	-0.41974	0.134403
hsa_circ_	-0.2144	0.134407
hsa_circ_	0.304028	0.134414
hsa_circ_	-0.4939	0.134414
hsa_circ_	0.250134	0.134419
hsa_circ_	-0.22954	0.134427
hsa_circ_	-0.29467	0.134431
hsa_circ_	0.286769	0.134448
hsa_circ_	0.341929	0.134457
hsa_circ_	-0.65537	0.13446
hsa_circ_	-0.31934	0.134463
hsa_circ_	0.289285	0.134466
hsa_circ_	0.506601	0.134484
hsa_circ_	-0.26319	0.134501
hsa_circ_	0.424346	0.134517
hsa_circ_	-0.25439	0.134518
hsa_circ_	0.350969	0.134519
hsa_circ_	0.31654	0.134521

hsa_circ_	0.348463	0.134527
hsa_circ_	-0.33682	0.134538
hsa_circ_	-0.53713	0.13454
hsa_circ_	-0.58619	0.134542
hsa_circ_	0.263133	0.134545
hsa_circ_	-1.10718	0.134545
hsa_circ_	-0.21445	0.134546
hsa_circ_	-0.41911	0.134554
hsa_circ_	-0.27203	0.134598
hsa_circ_	0.416587	0.134602
hsa_circ_	-0.2566	0.13461
hsa_circ_	-0.23867	0.134614
hsa_circ_	-0.72532	0.134625
hsa_circ_	-1.30617	0.134634
hsa_circ_	0.497904	0.134639
hsa_circ_	-0.34424	0.134641
hsa_circ_	0.343607	0.134652
hsa_circ_	0.298261	0.134666
hsa_circ_	0.33967	0.134668
hsa_circ_	-0.25449	0.13467
hsa_circ_	0.511584	0.134682
hsa_circ_	0.996693	0.134682
hsa_circ_	0.352643	0.134685
hsa_circ_	0.284338	0.134687
hsa_circ_	0.50315	0.134687
hsa_circ_	-0.22032	0.134693
hsa_circ_	-0.32758	0.134694
hsa_circ_	0.265377	0.134698
hsa_circ_	-0.22517	0.134718
hsa_circ_	0.280453	0.134725
hsa_circ_	-0.53893	0.134732
hsa_circ_	1.062214	0.134752
hsa_circ_	-0.22974	0.134758
hsa_circ_	0.362297	0.13476
hsa_circ_	-0.47469	0.134794
hsa_circ_	0.244576	0.134801
hsa_circ_	0.543975	0.134808
hsa_circ_	0.330621	0.134812
hsa_circ_	-0.24455	0.13482
hsa_circ_	-0.37514	0.134847
hsa_circ_	-0.33691	0.134852
hsa_circ_	-0.35912	0.134854
hsa_circ_	0.355949	0.134874
hsa_circ_	0.24069	0.134876
hsa_circ_	-0.26787	0.134893
hsa_circ_	0.909043	0.134905
hsa_circ_	-0.33877	0.134906
hsa_circ_	-0.21456	0.13491
hsa_circ_	0.561582	0.134913
hsa_circ_	-0.39322	0.134916
hsa_circ_	-0.32821	0.134924
hsa_circ_	-0.65358	0.134929
hsa_circ_	0.265706	0.134941
hsa_circ_	-0.5006	0.134946

hsa_circ_	0.264515	0.134947
hsa_circ_	0.619846	0.134958
hsa_circ_	-0.43468	0.134973
hsa_circ_	-0.39488	0.134978
hsa_circ_	0.928414	0.134992
hsa_circ_	-0.27504	0.135
hsa_circ_	0.479613	0.135008
hsa_circ_	-0.26674	0.135009
hsa_circ_	-1.5927	0.135009
hsa_circ_	-0.24839	0.135021
hsa_circ_	-0.35076	0.135024
hsa_circ_	0.30654	0.13503
hsa_circ_	0.457884	0.135033
hsa_circ_	0.54337	0.135048
hsa_circ_	-0.23343	0.135055
hsa_circ_	-0.29226	0.135066
hsa_circ_	-0.24581	0.13507
hsa_circ_	0.240852	0.135083
hsa_circ_	-0.74489	0.13509
hsa_circ_	-0.31761	0.135103
hsa_circ_	-0.35075	0.135111
hsa_circ_	-0.22253	0.13512
hsa_circ_	-0.54105	0.135124
hsa_circ_	-0.64283	0.135148
hsa_circ_	-0.2158	0.135153
hsa_circ_	-0.21512	0.135156
hsa_circ_	-0.235	0.135164
hsa_circ_	0.387372	0.135176
hsa_circ_	0.849942	0.135176
hsa_circ_	0.30003	0.135177
hsa_circ_	0.690254	0.135189
hsa_circ_	0.255816	0.135194
hsa_circ_	-0.28818	0.135198
hsa_circ_	0.351127	0.135205
hsa_circ_	0.265847	0.135214
hsa_circ_	-0.47754	0.135214
hsa_circ_	-0.70408	0.135218
hsa_circ_	0.395506	0.135224
hsa_circ_	0.351605	0.135232
hsa_circ_	-0.58052	0.135249
hsa_circ_	-0.21598	0.135254
hsa_circ_	-0.6422	0.135259
hsa_circ_	0.286689	0.135263
hsa_circ_	0.294713	0.135268
hsa_circ_	1.131309	0.135277
hsa_circ_	0.327068	0.135294
hsa_circ_	0.468201	0.135294
hsa_circ_	-0.42599	0.135309
hsa_circ_	-0.24897	0.135311
hsa_circ_	0.253102	0.135322
hsa_circ_	0.330271	0.135323
hsa_circ_	-0.26366	0.135323
hsa_circ_	0.372381	0.135328
hsa_circ_	-0.27438	0.135329

hsa_circ_	-0.29421	0.135337
hsa_circ_	0.657077	0.135344
hsa_circ_	-0.40891	0.135355
hsa_circ_	-0.22868	0.135359
hsa_circ_	0.293969	0.135362
hsa_circ_	0.437621	0.135372
hsa_circ_	-0.23658	0.135378
hsa_circ_	-0.24274	0.135387
hsa_circ_	-0.27126	0.135392
hsa_circ_	-0.25935	0.135415
hsa_circ_	0.49455	0.135421
hsa_circ_	-0.20905	0.135422
hsa_circ_	-0.7746	0.135432
hsa_circ_	0.317631	0.135437
hsa_circ_	-0.28706	0.135442
hsa_circ_	-0.40581	0.135451
hsa_circ_	0.20787	0.135466
hsa_circ_	-0.44101	0.135469
hsa_circ_	-0.2347	0.135491
hsa_circ_	-0.61098	0.135493
hsa_circ_	0.253731	0.135503
hsa_circ_	-0.9568	0.135508
hsa_circ_	-0.44619	0.135514
hsa_circ_	0.367763	0.135525
hsa_circ_	-0.27735	0.135529
hsa_circ_	0.549341	0.135534
hsa_circ_	0.261927	0.135574
hsa_circ_	-0.3876	0.13558
hsa_circ_	0.241196	0.135582
hsa_circ_	-1.37286	0.135592
hsa_circ_	0.601859	0.135593
hsa_circ_	0.629375	0.135599
hsa_circ_	0.309069	0.135603
hsa_circ_	0.316806	0.135625
hsa_circ_	0.226743	0.135638
hsa_circ_	0.687521	0.135641
hsa_circ_	0.420193	0.135645
hsa_circ_	-0.26239	0.135652
hsa_circ_	-0.58187	0.135665
hsa_circ_	0.374585	0.135667
hsa_circ_	-0.37441	0.13568
hsa_circ_	-0.25247	0.135688
hsa_circ_	0.378869	0.135707
hsa_circ_	0.287084	0.135707
hsa_circ_	0.480457	0.135716
hsa_circ_	-0.32231	0.135718
hsa_circ_	0.26249	0.135721
hsa_circ_	0.243187	0.13573
hsa_circ_	0.946438	0.135731
hsa_circ_	0.539902	0.135733
hsa_circ_	0.513281	0.135754
hsa_circ_	-0.40477	0.13577
hsa_circ_	-0.37011	0.135772
hsa_circ_	-0.29434	0.135776

hsa_circ_	0.365383	0.135777
hsa_circ_	0.660655	0.135783
hsa_circ_	-0.23447	0.13579
hsa_circ_	-0.28758	0.135798
hsa_circ_	-0.25954	0.1358
hsa_circ_	-0.35129	0.135802
hsa_circ_	-0.41847	0.135804
hsa_circ_	-0.89672	0.135811
hsa_circ_	0.434497	0.135812
hsa_circ_	-0.24551	0.135818
hsa_circ_	0.918197	0.135824
hsa_circ_	-0.43928	0.135827
hsa_circ_	0.922489	0.135828
hsa_circ_	0.370776	0.135842
hsa_circ_	0.248795	0.135873
hsa_circ_	0.394189	0.135889
hsa_circ_	-0.48313	0.135899
hsa_circ_	-0.28072	0.135914
hsa_circ_	0.850045	0.135915
hsa_circ_	0.279919	0.135932
hsa_circ_	0.272365	0.135937
hsa_circ_	-0.43084	0.13594
hsa_circ_	-0.29101	0.135945
hsa_circ_	-0.22553	0.135973
hsa_circ_	-0.24428	0.135973
hsa_circ_	-0.25061	0.135977
hsa_circ_	0.839203	0.135979
hsa_circ_	0.320503	0.135985
hsa_circ_	0.475716	0.135988
hsa_circ_	-0.26315	0.135989
hsa_circ_	0.918893	0.135991
hsa_circ_	-0.52922	0.135996
hsa_circ_	-0.27737	0.136012
hsa_circ_	-0.30346	0.136012
hsa_circ_	-0.28817	0.136017
hsa_circ_	-0.50302	0.136018
hsa_circ_	0.528389	0.13602
hsa_circ_	0.287435	0.136028
hsa_circ_	-0.24768	0.136044
hsa_circ_	-0.32582	0.136069
hsa_circ_	0.300378	0.136078
hsa_circ_	0.320788	0.136084
hsa_circ_	0.384896	0.136093
hsa_circ_	-0.25743	0.136095
hsa_circ_	-0.39222	0.1361
hsa_circ_	0.336956	0.136121
hsa_circ_	0.32504	0.136136
hsa_circ_	-0.35891	0.13614
hsa_circ_	-0.26138	0.136149
hsa_circ_	-0.34046	0.13615
hsa_circ_	-0.33351	0.136152
hsa_circ_	-0.29515	0.136168
hsa_circ_	-0.29514	0.136171
hsa_circ_	-0.45796	0.136186

hsa_circ_	-0.28448	0.136187
hsa_circ_	-0.48887	0.136199
hsa_circ_	0.254842	0.13621
hsa_circ_	0.78228	0.136235
hsa_circ_	0.836118	0.136253
hsa_circ_	-0.22887	0.13626
hsa_circ_	-0.31273	0.136271
hsa_circ_	0.323717	0.136275
hsa_circ_	0.287992	0.136291
hsa_circ_	0.509237	0.136297
hsa_circ_	0.25606	0.136298
hsa_circ_	-0.51165	0.1363
hsa_circ_	-0.26413	0.136304
hsa_circ_	-0.31355	0.136308
hsa_circ_	0.774454	0.136309
hsa_circ_	-0.31042	0.136328
hsa_circ_	-0.38508	0.13633
hsa_circ_	0.834621	0.136341
hsa_circ_	-0.55633	0.136343
hsa_circ_	0.611548	0.136355
hsa_circ_	0.420329	0.136362
hsa_circ_	-0.33147	0.136378
hsa_circ_	-0.32401	0.136379
hsa_circ_	0.310777	0.136393
hsa_circ_	0.704695	0.1364
hsa_circ_	0.238881	0.136408
hsa_circ_	0.356809	0.136414
hsa_circ_	0.670549	0.136422
hsa_circ_	-0.30896	0.136426
hsa_circ_	0.825745	0.136433
hsa_circ_	-0.41862	0.136449
hsa_circ_	0.298503	0.136455
hsa_circ_	-0.81546	0.136461
hsa_circ_	-0.2441	0.136473
hsa_circ_	0.268149	0.136489
hsa_circ_	-0.27136	0.136491
hsa_circ_	-0.38918	0.136493
hsa_circ_	-0.697	0.136496
hsa_circ_	0.412356	0.136498
hsa_circ_	0.393203	0.136502
hsa_circ_	0.415692	0.136514
hsa_circ_	0.77306	0.136517
hsa_circ_	0.285062	0.136528
hsa_circ_	-0.41087	0.136559
hsa_circ_	0.272417	0.136563
hsa_circ_	-0.23399	0.136581
hsa_circ_	-0.32742	0.136583
hsa_circ_	0.420876	0.136585
hsa_circ_	-1.57283	0.136586
hsa_circ_	0.337525	0.136588
hsa_circ_	0.230529	0.136593
hsa_circ_	0.406449	0.136597
hsa_circ_	0.421002	0.136603
hsa_circ_	-0.76595	0.136622

hsa_circ_	-0.39433	0.136625
hsa_circ_	0.323699	0.136651
hsa_circ_	-0.23196	0.136658
hsa_circ_	-0.46855	0.136667
hsa_circ_	0.427678	0.136668
hsa_circ_	-0.20938	0.136677
hsa_circ_	-0.22582	0.136677
hsa_circ_	0.436528	0.136684
hsa_circ_	0.386809	0.136685
hsa_circ_	-0.56641	0.136686
hsa_circ_	0.748118	0.136686
hsa_circ_	0.432966	0.1367
hsa_circ_	0.313721	0.136736
hsa_circ_	0.244094	0.136741
hsa_circ_	0.361152	0.136746
hsa_circ_	-0.42777	0.136753
hsa_circ_	-0.36456	0.136761
hsa_circ_	0.274296	0.136762
hsa_circ_	-0.27757	0.136768
hsa_circ_	0.416076	0.136768
hsa_circ_	0.968471	0.136778
hsa_circ_	0.303921	0.136781
hsa_circ_	-0.46528	0.136794
hsa_circ_	0.38541	0.136799
hsa_circ_	0.571508	0.136809
hsa_circ_	0.468929	0.13681
hsa_circ_	-0.25603	0.136815
hsa_circ_	-0.2885	0.136823
hsa_circ_	0.428687	0.136824
hsa_circ_	-0.31442	0.136834
hsa_circ_	0.44702	0.136842
hsa_circ_	0.236645	0.136854
hsa_circ_	-0.50635	0.136866
hsa_circ_	-0.65511	0.136867
hsa_circ_	-0.50628	0.136874
hsa_circ_	-0.37834	0.136885
hsa_circ_	-0.22625	0.136896
hsa_circ_	-0.58392	0.136899
hsa_circ_	-0.60719	0.136902
hsa_circ_	-0.39169	0.136918
hsa_circ_	-0.42113	0.136925
hsa_circ_	-0.22238	0.136927
hsa_circ_	-0.24881	0.136942
hsa_circ_	-2.15309	0.136944
hsa_circ_	-1.1511	0.13695
hsa_circ_	-0.3007	0.13696
hsa_circ_	0.234633	0.136961
hsa_circ_	0.485048	0.136963
hsa_circ_	0.438245	0.136979
hsa_circ_	1.238993	0.136981
hsa_circ_	0.644925	0.136988
hsa_circ_	0.68859	0.13699
hsa_circ_	0.289285	0.136992
hsa_circ_	0.273496	0.136998

hsa_circ_	0.791778	0.136998
hsa_circ_	-0.21811	0.137006
hsa_circ_	-0.2489	0.137034
hsa_circ_	-0.26022	0.137053
hsa_circ_	-0.57998	0.137054
hsa_circ_	0.604813	0.137057
hsa_circ_	-0.28319	0.137064
hsa_circ_	-0.20973	0.137073
hsa_circ_	-0.49892	0.137093
hsa_circ_	0.24774	0.137094
hsa_circ_	-0.5261	0.137109
hsa_circ_	0.344386	0.137131
hsa_circ_	-0.37887	0.137133
hsa_circ_	0.360895	0.137138
hsa_circ_	0.485782	0.137142
hsa_circ_	-0.31723	0.137149
hsa_circ_	0.240325	0.137162
hsa_circ_	-0.22124	0.137164
hsa_circ_	-0.30253	0.13719
hsa_circ_	-0.33784	0.137195
hsa_circ_	0.278055	0.137196
hsa_circ_	0.252257	0.137197
hsa_circ_	0.349314	0.13721
hsa_circ_	-0.35994	0.13722
hsa_circ_	0.256924	0.137221
hsa_circ_	0.22044	0.137252
hsa_circ_	-0.34669	0.137254
hsa_circ_	-0.41462	0.137255
hsa_circ_	0.375284	0.137268
hsa_circ_	0.52684	0.137279
hsa_circ_	0.316846	0.137289
hsa_circ_	0.618206	0.137289
hsa_circ_	0.264204	0.137313
hsa_circ_	-0.30508	0.137314
hsa_circ_	0.240398	0.137322
hsa_circ_	-0.78596	0.137341
hsa_circ_	-0.32623	0.137342
hsa_circ_	-0.32878	0.137368
hsa_circ_	-0.37483	0.13738
hsa_circ_	-0.44051	0.137383
hsa_circ_	-0.38503	0.137397
hsa_circ_	-0.25798	0.137405
hsa_circ_	0.238267	0.137422
hsa_circ_	-0.22595	0.137426
hsa_circ_	-0.45738	0.137435
hsa_circ_	-0.29666	0.137443
hsa_circ_	-0.43163	0.137455
hsa_circ_	-0.22576	0.137473
hsa_circ_	-0.36796	0.137492
hsa_circ_	-0.21477	0.137519
hsa_circ_	1.090896	0.137538
hsa_circ_	-0.57404	0.137539
hsa_circ_	0.320844	0.137543
hsa_circ_	-0.23648	0.13755

hsa_circ_	-0.2389	0.137552
hsa_circ_	-0.29195	0.137571
hsa_circ_	-0.2565	0.137581
hsa_circ_	-0.60794	0.13759
hsa_circ_	-1.00669	0.137601
hsa_circ_	0.324511	0.137609
hsa_circ_	0.912895	0.137613
hsa_circ_	1.059873	0.137637
hsa_circ_	-0.28753	0.13764
hsa_circ_	0.535297	0.137643
hsa_circ_	0.391592	0.137655
hsa_circ_	0.234956	0.137656
hsa_circ_	1.120944	0.137666
hsa_circ_	-0.35877	0.137674
hsa_circ_	0.255106	0.137692
hsa_circ_	0.32174	0.137694
hsa_circ_	0.237859	0.137695
hsa_circ_	-0.24892	0.137717
hsa_circ_	0.326058	0.137729
hsa_circ_	-0.84503	0.13775
hsa_circ_	-0.33723	0.13777
hsa_circ_	-1.12407	0.137773
hsa_circ_	-0.22592	0.137778
hsa_circ_	1.550656	0.137778
hsa_circ_	0.632494	0.137787
hsa_circ_	-0.36363	0.13779
hsa_circ_	-0.23275	0.137791
hsa_circ_	0.286758	0.137792
hsa_circ_	-0.25626	0.137821
hsa_circ_	0.4926	0.137825
hsa_circ_	0.773576	0.137834
hsa_circ_	0.25289	0.137838
hsa_circ_	0.475546	0.137855
hsa_circ_	-0.21051	0.137883
hsa_circ_	-0.32026	0.137886
hsa_circ_	0.641858	0.137896
hsa_circ_	-0.32828	0.137908
hsa_circ_	0.233857	0.137925
hsa_circ_	0.536816	0.137925
hsa_circ_	0.516828	0.13793
hsa_circ_	-0.47735	0.137931
hsa_circ_	0.255868	0.137935
hsa_circ_	0.382683	0.137937
hsa_circ_	-0.2453	0.137966
hsa_circ_	-0.60458	0.137968
hsa_circ_	-0.57037	0.137972
hsa_circ_	-0.81541	0.137975
hsa_circ_	-0.28492	0.137981
hsa_circ_	0.215529	0.137986
hsa_circ_	-0.4083	0.138
hsa_circ_	1.372302	0.13802
hsa_circ_	0.30496	0.138027
hsa_circ_	-0.37779	0.138048
hsa_circ_	0.515006	0.138055

hsa_circ_	0.248459	0.138058
hsa_circ_	-0.76758	0.13806
hsa_circ_	0.916922	0.138079
hsa_circ_	0.288248	0.13808
hsa_circ_	0.312662	0.138089
hsa_circ_	-0.33161	0.138093
hsa_circ_	0.685492	0.138141
hsa_circ_	0.36394	0.138142
hsa_circ_	-0.34528	0.138144
hsa_circ_	-0.61733	0.138148
hsa_circ_	0.283455	0.13815
hsa_circ_	0.319868	0.138169
hsa_circ_	-0.25965	0.138171
hsa_circ_	0.551956	0.138172
hsa_circ_	-0.31158	0.138178
hsa_circ_	0.440739	0.138179
hsa_circ_	-0.42491	0.138187
hsa_circ_	-0.23308	0.138191
hsa_circ_	0.502171	0.138191
hsa_circ_	-0.27844	0.13821
hsa_circ_	-0.25549	0.138216
hsa_circ_	0.861981	0.138238
hsa_circ_	0.405763	0.138246
hsa_circ_	-0.21115	0.138256
hsa_circ_	0.46582	0.138258
hsa_circ_	-0.38454	0.138267
hsa_circ_	0.269911	0.138288
hsa_circ_	0.289777	0.138288
hsa_circ_	-0.23339	0.138294
hsa_circ_	0.232652	0.138297
hsa_circ_	-0.25872	0.138304
hsa_circ_	-0.47561	0.138317
hsa_circ_	-0.29004	0.138321
hsa_circ_	-0.75754	0.138322
hsa_circ_	0.810316	0.138324
hsa_circ_	-0.37289	0.138339
hsa_circ_	0.837955	0.138345
hsa_circ_	0.229032	0.138348
hsa_circ_	-0.23706	0.138348
hsa_circ_	-0.36936	0.138359
hsa_circ_	0.318775	0.138362
hsa_circ_	-0.21474	0.13837
hsa_circ_	0.282154	0.138373
hsa_circ_	-0.38642	0.138373
hsa_circ_	0.309361	0.138376
hsa_circ_	0.994637	0.138383
hsa_circ_	-2.10337	0.138388
hsa_circ_	0.246224	0.138389
hsa_circ_	0.274884	0.138405
hsa_circ_	0.224323	0.13841
hsa_circ_	-0.31909	0.138416
hsa_circ_	0.341696	0.138445
hsa_circ_	-0.26938	0.138449
hsa_circ_	0.459154	0.138462

hsa_circ_	-0.2043	0.138464
hsa_circ_	-0.33688	0.138469
hsa_circ_	0.575843	0.138473
hsa_circ_	0.243509	0.138491
hsa_circ_	-0.89392	0.138498
hsa_circ_	-0.28817	0.138528
hsa_circ_	-0.39252	0.138534
hsa_circ_	-0.29931	0.138541
hsa_circ_	0.751276	0.138552
hsa_circ_	-0.28869	0.138567
hsa_circ_	-0.87451	0.13857
hsa_circ_	-0.33745	0.138573
hsa_circ_	0.489457	0.138602
hsa_circ_	0.259928	0.138605
hsa_circ_	-0.22697	0.138619
hsa_circ_	-0.38938	0.138627
hsa_circ_	-0.35656	0.138629
hsa_circ_	0.243736	0.138641
hsa_circ_	-0.22535	0.138654
hsa_circ_	-0.66161	0.13866
hsa_circ_	0.291949	0.138662
hsa_circ_	0.396029	0.138675
hsa_circ_	-0.20743	0.138681
hsa_circ_	-0.83824	0.138683
hsa_circ_	0.264746	0.138686
hsa_circ_	-0.29274	0.138688
hsa_circ_	0.310167	0.138698
hsa_circ_	0.404443	0.138698
hsa_circ_	-0.41057	0.138728
hsa_circ_	0.295989	0.138741
hsa_circ_	0.256277	0.138745
hsa_circ_	-0.22428	0.138751
hsa_circ_	-0.43147	0.138752
hsa_circ_	-0.37192	0.138754
hsa_circ_	0.677773	0.138774
hsa_circ_	0.336239	0.138781
hsa_circ_	0.330228	0.138783
hsa_circ_	-0.2247	0.138784
hsa_circ_	-0.58795	0.138792
hsa_circ_	-0.4957	0.138801
hsa_circ_	-0.23334	0.138806
hsa_circ_	0.41088	0.138812
hsa_circ_	-0.21975	0.138818
hsa_circ_	0.995046	0.138821
hsa_circ_	-0.33666	0.138821
hsa_circ_	0.37851	0.138825
hsa_circ_	0.980363	0.138845
hsa_circ_	0.833349	0.138846
hsa_circ_	0.428996	0.138847
hsa_circ_	-0.30631	0.138848
hsa_circ_	0.286993	0.138865
hsa_circ_	0.907274	0.138901
hsa_circ_	-0.87885	0.138904
hsa_circ_	-0.25413	0.138914

hsa_circ_	-0.2341	0.138918
hsa_circ_	0.295366	0.138919
hsa_circ_	0.384951	0.138919
hsa_circ_	-0.23765	0.138926
hsa_circ_	0.534212	0.13897
hsa_circ_	0.233579	0.138994
hsa_circ_	-0.22021	0.139008
hsa_circ_	-2.85271	0.13903
hsa_circ_	0.318868	0.139039
hsa_circ_	0.245946	0.139064
hsa_circ_	1.734123	0.139068
hsa_circ_	0.317638	0.139068
hsa_circ_	-0.29466	0.139078
hsa_circ_	-0.34067	0.13909
hsa_circ_	-0.27019	0.139096
hsa_circ_	-0.29671	0.139104
hsa_circ_	0.6359	0.139114
hsa_circ_	0.463978	0.139139
hsa_circ_	-0.26053	0.139146
hsa_circ_	-0.22088	0.139149
hsa_circ_	0.392594	0.139161
hsa_circ_	-0.32093	0.139165
hsa_circ_	-0.49224	0.139168
hsa_circ_	0.486541	0.139192
hsa_circ_	0.278606	0.139196
hsa_circ_	-0.32906	0.13921
hsa_circ_	0.317536	0.139217
hsa_circ_	0.448958	0.139224
hsa_circ_	0.894577	0.139226
hsa_circ_	-0.20613	0.139228
hsa_circ_	-0.70139	0.139234
hsa_circ_	-0.64895	0.139236
hsa_circ_	0.46087	0.139241
hsa_circ_	-0.23058	0.139247
hsa_circ_	-0.53161	0.139262
hsa_circ_	0.787386	0.139264
hsa_circ_	-0.29477	0.139274
hsa_circ_	-0.30089	0.139274
hsa_circ_	0.549906	0.139277
hsa_circ_	0.308668	0.139333
hsa_circ_	0.807448	0.139334
hsa_circ_	-0.81849	0.139337
hsa_circ_	-0.28333	0.13934
hsa_circ_	-0.23655	0.139342
hsa_circ_	0.62178	0.139353
hsa_circ_	-0.51784	0.139359
hsa_circ_	0.299	0.139361
hsa_circ_	-0.61733	0.139387
hsa_circ_	0.629983	0.139397
hsa_circ_	-0.3072	0.139405
hsa_circ_	-0.61466	0.139421
hsa_circ_	0.205707	0.139424
hsa_circ_	-0.60122	0.139426
hsa_circ_	0.346351	0.139431

hsa_circ_	-0.25868	0.139441
hsa_circ_	0.290966	0.139448
hsa_circ_	-1.15878	0.13945
hsa_circ_	-0.26446	0.139451
hsa_circ_	1.007196	0.139452
hsa_circ_	-0.46226	0.139464
hsa_circ_	-0.20984	0.139472
hsa_circ_	-0.28938	0.139483
hsa_circ_	0.436784	0.13949
hsa_circ_	0.825303	0.139496
hsa_circ_	-0.25789	0.139496
hsa_circ_	-0.4377	0.139496
hsa_circ_	-1.0033	0.139504
hsa_circ_	0.306905	0.139532
hsa_circ_	-0.26314	0.139545
hsa_circ_	0.312562	0.139552
hsa_circ_	0.286437	0.13958
hsa_circ_	1.04708	0.13958
hsa_circ_	-0.43024	0.13958
hsa_circ_	0.412746	0.139594
hsa_circ_	0.389468	0.139609
hsa_circ_	-0.39853	0.139615
hsa_circ_	-0.5611	0.139619
hsa_circ_	-0.50523	0.139628
hsa_circ_	0.276863	0.139637
hsa_circ_	0.291156	0.139645
hsa_circ_	0.286167	0.139648
hsa_circ_	0.355506	0.13965
hsa_circ_	-0.46422	0.139658
hsa_circ_	0.423357	0.139677
hsa_circ_	0.281894	0.139679
hsa_circ_	-0.45206	0.139679
hsa_circ_	-0.27909	0.139681
hsa_circ_	0.515691	0.139705
hsa_circ_	0.216852	0.139707
hsa_circ_	-0.52186	0.139708
hsa_circ_	0.7404	0.139717
hsa_circ_	-0.48582	0.139721
hsa_circ_	-0.29431	0.139725
hsa_circ_	0.22667	0.139726
hsa_circ_	0.255941	0.139726
hsa_circ_	-0.75551	0.139741
hsa_circ_	0.275942	0.13976
hsa_circ_	-0.33303	0.139764
hsa_circ_	-0.4096	0.139766
hsa_circ_	-0.41649	0.139771
hsa_circ_	0.26417	0.139774
hsa_circ_	-0.24318	0.139777
hsa_circ_	-0.29075	0.139778
hsa_circ_	-0.94748	0.13978
hsa_circ_	0.253581	0.139785
hsa_circ_	-0.31437	0.139787
hsa_circ_	0.387253	0.139787
hsa_circ_	-0.24901	0.139791

hsa_circ_	-0.7253	0.139792
hsa_circ_	0.446223	0.139796
hsa_circ_	-0.27958	0.139804
hsa_circ_	-0.47251	0.139812
hsa_circ_	0.374855	0.139814
hsa_circ_	0.359675	0.139818
hsa_circ_	-0.27428	0.139824
hsa_circ_	0.345223	0.139825
hsa_circ_	1.111626	0.139856
hsa_circ_	-0.24643	0.139862
hsa_circ_	0.440153	0.139877
hsa_circ_	-0.22459	0.139901
hsa_circ_	-0.2607	0.139907
hsa_circ_	0.889323	0.139909
hsa_circ_	0.472204	0.13991
hsa_circ_	0.510359	0.139913
hsa_circ_	-0.81725	0.139915
hsa_circ_	-0.45652	0.139916
hsa_circ_	-0.29144	0.139916
hsa_circ_	0.359523	0.139926
hsa_circ_	-0.43117	0.139928
hsa_circ_	0.324429	0.139933
hsa_circ_	0.346739	0.139942
hsa_circ_	-0.24229	0.139943
hsa_circ_	0.245558	0.139959
hsa_circ_	-0.42817	0.139964
hsa_circ_	-0.51781	0.139968
hsa_circ_	-0.30979	0.139971
hsa_circ_	-0.44022	0.139975
hsa_circ_	0.266679	0.139976
hsa_circ_	-0.99412	0.13999
hsa_circ_	-0.27179	0.139997
hsa_circ_	-0.47541	0.140029
hsa_circ_	-0.64427	0.140035
hsa_circ_	-0.52186	0.140064
hsa_circ_	-0.2871	0.14007
hsa_circ_	-0.36702	0.140087
hsa_circ_	-0.6243	0.1401
hsa_circ_	-0.3828	0.140108
hsa_circ_	0.355094	0.140117
hsa_circ_	-0.58971	0.140162
hsa_circ_	-0.25412	0.140163
hsa_circ_	-0.27382	0.140169
hsa_circ_	-0.29326	0.140181
hsa_circ_	-0.28548	0.140188
hsa_circ_	-0.43149	0.140189
hsa_circ_	0.3274	0.140206
hsa_circ_	0.242079	0.140208
hsa_circ_	-0.91108	0.140212
hsa_circ_	-0.23854	0.140212
hsa_circ_	-0.24757	0.140218
hsa_circ_	-0.32652	0.140231
hsa_circ_	-0.44915	0.140247
hsa_circ_	-0.55002	0.140258

hsa_circ_	0.490035	0.14026
hsa_circ_	0.475792	0.140276
hsa_circ_	-0.2953	0.140279
hsa_circ_	-0.48732	0.140281
hsa_circ_	0.6446	0.140281
hsa_circ_	-0.22971	0.140283
hsa_circ_	0.375523	0.14029
hsa_circ_	-0.28574	0.140302
hsa_circ_	0.27409	0.140305
hsa_circ_	0.240095	0.140307
hsa_circ_	-0.49933	0.140311
hsa_circ_	-0.27455	0.14033
hsa_circ_	-0.55575	0.140334
hsa_circ_	0.281412	0.140346
hsa_circ_	1.747266	0.140353
hsa_circ_	-0.28179	0.140355
hsa_circ_	0.533429	0.140368
hsa_circ_	0.233233	0.140372
hsa_circ_	1.108386	0.140378
hsa_circ_	-0.39261	0.140378
hsa_circ_	-0.46694	0.140386
hsa_circ_	-0.34297	0.140386
hsa_circ_	-0.26598	0.140391
hsa_circ_	0.252816	0.140431
hsa_circ_	-1.12434	0.140435
hsa_circ_	-0.28047	0.140437
hsa_circ_	-0.3183	0.140441
hsa_circ_	0.887205	0.140465
hsa_circ_	0.286961	0.140476
hsa_circ_	0.38727	0.140484
hsa_circ_	-0.25435	0.14049
hsa_circ_	0.260961	0.140498
hsa_circ_	-0.22265	0.1405
hsa_circ_	0.502862	0.14052
hsa_circ_	-0.42425	0.140524
hsa_circ_	0.258226	0.140525
hsa_circ_	0.600124	0.140539
hsa_circ_	-0.28649	0.140546
hsa_circ_	-0.24745	0.140547
hsa_circ_	-0.26285	0.140549
hsa_circ_	-0.25067	0.140562
hsa_circ_	-0.26538	0.140568
hsa_circ_	0.270147	0.140571
hsa_circ_	-0.28468	0.140583
hsa_circ_	-0.23252	0.140585
hsa_circ_	-0.91786	0.140588
hsa_circ_	-0.85195	0.140591
hsa_circ_	-0.28836	0.140592
hsa_circ_	0.319724	0.140599
hsa_circ_	0.90071	0.140606
hsa_circ_	0.443503	0.140615
hsa_circ_	-0.38137	0.14064
hsa_circ_	-0.67674	0.140642
hsa_circ_	-0.21985	0.140642

hsa_circ_	0.674793	0.14065
hsa_circ_	-0.53851	0.140662
hsa_circ_	-0.36846	0.140664
hsa_circ_	0.323986	0.140667
hsa_circ_	0.253677	0.140673
hsa_circ_	0.271895	0.140687
hsa_circ_	0.496874	0.140706
hsa_circ_	0.314795	0.140717
hsa_circ_	0.366613	0.140721
hsa_circ_	-0.28382	0.140736
hsa_circ_	0.235672	0.140742
hsa_circ_	-0.34706	0.140745
hsa_circ_	-0.36205	0.140747
hsa_circ_	0.707428	0.140763
hsa_circ_	-0.67423	0.140765
hsa_circ_	-0.27761	0.140765
hsa_circ_	-0.30814	0.140778
hsa_circ_	0.502857	0.140778
hsa_circ_	0.320805	0.140788
hsa_circ_	-0.26751	0.140789
hsa_circ_	-0.23465	0.140815
hsa_circ_	-0.29825	0.140838
hsa_circ_	0.240599	0.140843
hsa_circ_	-0.34823	0.140846
hsa_circ_	-0.56865	0.140849
hsa_circ_	-0.3787	0.140849
hsa_circ_	0.488773	0.140854
hsa_circ_	-0.21416	0.140868
hsa_circ_	0.225583	0.140874
hsa_circ_	0.212328	0.140876
hsa_circ_	0.565856	0.140888
hsa_circ_	0.519697	0.140893
hsa_circ_	0.23082	0.140898
hsa_circ_	-0.41133	0.140904
hsa_circ_	0.543134	0.140905
hsa_circ_	0.206188	0.140915
hsa_circ_	0.225855	0.140931
hsa_circ_	0.511841	0.140937
hsa_circ_	-0.30177	0.14094
hsa_circ_	-0.47594	0.140976
hsa_circ_	0.256097	0.140982
hsa_circ_	1.103438	0.140987
hsa_circ_	0.439624	0.140992
hsa_circ_	-0.41229	0.141
hsa_circ_	0.765486	0.141009
hsa_circ_	0.310523	0.14101
hsa_circ_	0.32893	0.141011
hsa_circ_	0.323935	0.141021
hsa_circ_	0.283104	0.141037
hsa_circ_	0.268705	0.141037
hsa_circ_	0.258626	0.141042
hsa_circ_	-0.20187	0.141043
hsa_circ_	0.232383	0.141057
hsa_circ_	0.237565	0.141058

hsa_circ_	-0.30511	0.141063
hsa_circ_	0.267867	0.141064
hsa_circ_	-0.24853	0.141068
hsa_circ_	-0.21288	0.141069
hsa_circ_	-0.25394	0.141078
hsa_circ_	0.452865	0.141084
hsa_circ_	-0.21525	0.141099
hsa_circ_	-0.31968	0.1411
hsa_circ_	-0.23674	0.141105
hsa_circ_	0.361497	0.141107
hsa_circ_	-0.47609	0.141115
hsa_circ_	0.259625	0.141118
hsa_circ_	-0.30852	0.14112
hsa_circ_	0.305422	0.141141
hsa_circ_	-0.3176	0.14115
hsa_circ_	-0.2966	0.141152
hsa_circ_	0.43415	0.141152
hsa_circ_	-0.44309	0.141159
hsa_circ_	-0.38878	0.141167
hsa_circ_	0.351349	0.14117
hsa_circ_	0.288115	0.141184
hsa_circ_	0.2358	0.141186
hsa_circ_	-0.26809	0.141197
hsa_circ_	-0.23199	0.141211
hsa_circ_	0.386313	0.141212
hsa_circ_	0.256646	0.141272
hsa_circ_	-0.32687	0.141287
hsa_circ_	-0.22661	0.141298
hsa_circ_	0.488855	0.1413
hsa_circ_	-0.51104	0.141308
hsa_circ_	0.305227	0.141322
hsa_circ_	0.921476	0.141329
hsa_circ_	-0.4263	0.141343
hsa_circ_	-0.25337	0.141345
hsa_circ_	-0.34104	0.141348
hsa_circ_	-0.83149	0.141353
hsa_circ_	0.321755	0.141362
hsa_circ_	1.101519	0.141366
hsa_circ_	-0.35705	0.14139
hsa_circ_	-0.38906	0.141397
hsa_circ_	0.576908	0.141402
hsa_circ_	0.520698	0.14141
hsa_circ_	-0.37484	0.141412
hsa_circ_	-0.40448	0.141423
hsa_circ_	0.215192	0.141424
hsa_circ_	-0.73025	0.141426
hsa_circ_	-0.52627	0.141444
hsa_circ_	-0.34078	0.141449
hsa_circ_	0.437301	0.141455
hsa_circ_	-0.31354	0.141463
hsa_circ_	0.5157	0.141474
hsa_circ_	0.569309	0.141475
hsa_circ_	0.491949	0.141509
hsa_circ_	-0.29124	0.141522

hsa_circ_	-0.42475	0.141526
hsa_circ_	-0.22192	0.141545
hsa_circ_	-0.22008	0.14155
hsa_circ_	-0.22618	0.141561
hsa_circ_	0.340254	0.141571
hsa_circ_	-0.28197	0.141591
hsa_circ_	-1.2829	0.141592
hsa_circ_	0.425591	0.141599
hsa_circ_	0.26636	0.141621
hsa_circ_	0.280506	0.141625
hsa_circ_	0.490048	0.141626
hsa_circ_	-0.48627	0.141632
hsa_circ_	0.315668	0.141633
hsa_circ_	1.496989	0.141646
hsa_circ_	-0.57362	0.141652
hsa_circ_	-0.28487	0.141668
hsa_circ_	-0.27016	0.14167
hsa_circ_	0.239542	0.141674
hsa_circ_	0.66069	0.141693
hsa_circ_	-0.23821	0.141697
hsa_circ_	0.976149	0.141698
hsa_circ_	-0.39602	0.141749
hsa_circ_	-0.99953	0.141753
hsa_circ_	0.407279	0.14176
hsa_circ_	-0.6727	0.141768
hsa_circ_	0.318623	0.141777
hsa_circ_	0.561089	0.141786
hsa_circ_	0.251969	0.14179
hsa_circ_	-0.27267	0.141798
hsa_circ_	1.096416	0.141801
hsa_circ_	-0.34814	0.141802
hsa_circ_	-0.23608	0.141813
hsa_circ_	0.60601	0.141814
hsa_circ_	0.245545	0.141822
hsa_circ_	-0.58526	0.141823
hsa_circ_	0.239461	0.14183
hsa_circ_	-0.21418	0.141832
hsa_circ_	0.500227	0.141835
hsa_circ_	0.255674	0.141837
hsa_circ_	-0.44462	0.141862
hsa_circ_	-0.78004	0.141866
hsa_circ_	-0.31397	0.14187
hsa_circ_	0.243306	0.141874
hsa_circ_	0.496896	0.141886
hsa_circ_	0.361586	0.141887
hsa_circ_	0.447305	0.141888
hsa_circ_	-0.69887	0.141894
hsa_circ_	0.996621	0.141912
hsa_circ_	-0.22031	0.141915
hsa_circ_	-0.47277	0.141925
hsa_circ_	-0.21132	0.141936
hsa_circ_	1.036758	0.141945
hsa_circ_	0.334656	0.141948
hsa_circ_	0.476765	0.141953

hsa_circ_	0.255252	0.141962
hsa_circ_	-0.34684	0.141968
hsa_circ_	0.231743	0.141975
hsa_circ_	0.57164	0.141976
hsa_circ_	0.762737	0.141977
hsa_circ_	-0.38463	0.141979
hsa_circ_	-0.20218	0.141983
hsa_circ_	-0.73471	0.142
hsa_circ_	0.290952	0.142016
hsa_circ_	-0.23731	0.142037
hsa_circ_	-0.52411	0.142038
hsa_circ_	-0.5089	0.142039
hsa_circ_	0.26364	0.142062
hsa_circ_	-0.22225	0.142068
hsa_circ_	0.238922	0.142071
hsa_circ_	-1.14276	0.142087
hsa_circ_	0.326439	0.142093
hsa_circ_	-0.26242	0.142102
hsa_circ_	-0.28805	0.142118
hsa_circ_	0.600732	0.142119
hsa_circ_	0.314782	0.142123
hsa_circ_	-1.05247	0.142124
hsa_circ_	-0.37712	0.142125
hsa_circ_	-0.49466	0.142135
hsa_circ_	-0.21488	0.142138
hsa_circ_	-0.48183	0.14214
hsa_circ_	-0.44722	0.142151
hsa_circ_	0.318345	0.142157
hsa_circ_	0.273615	0.142186
hsa_circ_	-0.2752	0.142206
hsa_circ_	0.482967	0.14221
hsa_circ_	-0.55404	0.142216
hsa_circ_	0.274473	0.142218
hsa_circ_	0.360329	0.142223
hsa_circ_	-0.30359	0.142227
hsa_circ_	0.550839	0.142231
hsa_circ_	0.323956	0.142241
hsa_circ_	0.376606	0.142242
hsa_circ_	0.219598	0.142242
hsa_circ_	0.329174	0.142245
hsa_circ_	0.615283	0.142266
hsa_circ_	-0.30368	0.142266
hsa_circ_	-0.42329	0.142271
hsa_circ_	0.374825	0.142281
hsa_circ_	-0.28593	0.142286
hsa_circ_	0.394857	0.142291
hsa_circ_	0.271203	0.142291
hsa_circ_	0.501197	0.142302
hsa_circ_	-0.25254	0.142327
hsa_circ_	-0.3437	0.142329
hsa_circ_	-0.24817	0.142342
hsa_circ_	0.456382	0.142364
hsa_circ_	-0.61471	0.14238
hsa_circ_	0.303441	0.142389

hsa_circ_	0.380375	0.142401
hsa_circ_	0.218814	0.142404
hsa_circ_	0.600236	0.142413
hsa_circ_	-0.22492	0.142423
hsa_circ_	-0.47274	0.142425
hsa_circ_	0.474639	0.142426
hsa_circ_	-0.6053	0.142428
hsa_circ_	-0.31388	0.142437
hsa_circ_	-0.21512	0.142444
hsa_circ_	0.301774	0.142448
hsa_circ_	0.319204	0.142453
hsa_circ_	-0.30703	0.142459
hsa_circ_	0.406067	0.142463
hsa_circ_	0.373672	0.142475
hsa_circ_	0.662894	0.142479
hsa_circ_	0.250192	0.142493
hsa_circ_	-0.26557	0.142513
hsa_circ_	-0.81921	0.142515
hsa_circ_	-0.21784	0.142546
hsa_circ_	-0.28155	0.142555
hsa_circ_	0.381238	0.14256
hsa_circ_	0.484519	0.142568
hsa_circ_	0.378446	0.142581
hsa_circ_	-0.42826	0.142583
hsa_circ_	0.499899	0.142586
hsa_circ_	0.26906	0.142599
hsa_circ_	0.569039	0.1426
hsa_circ_	-0.48802	0.14262
hsa_circ_	-0.33599	0.14263
hsa_circ_	0.32525	0.142647
hsa_circ_	-0.35612	0.142648
hsa_circ_	-0.38005	0.142648
hsa_circ_	-0.26898	0.142653
hsa_circ_	0.302073	0.14266
hsa_circ_	0.296276	0.142662
hsa_circ_	-0.52581	0.142662
hsa_circ_	0.272471	0.142695
hsa_circ_	0.211514	0.1427
hsa_circ_	0.480298	0.142703
hsa_circ_	0.311433	0.142709
hsa_circ_	0.358152	0.142711
hsa_circ_	0.616655	0.142723
hsa_circ_	0.22592	0.142742
hsa_circ_	-0.41185	0.142743
hsa_circ_	0.302403	0.142743
hsa_circ_	-0.28159	0.142764
hsa_circ_	-0.79089	0.142766
hsa_circ_	-0.28734	0.142774
hsa_circ_	-0.47749	0.142775
hsa_circ_	0.280136	0.142778
hsa_circ_	-1.11048	0.142779
hsa_circ_	0.256533	0.1428
hsa_circ_	-0.31999	0.142823
hsa_circ_	0.265038	0.142823

hsa_circ_	-0.8571	0.142834
hsa_circ_	-0.61135	0.142834
hsa_circ_	0.732486	0.142841
hsa_circ_	-0.2355	0.142856
hsa_circ_	0.473092	0.142859
hsa_circ_	0.429519	0.142862
hsa_circ_	0.305916	0.142869
hsa_circ_	-0.50191	0.142871
hsa_circ_	0.974588	0.14288
hsa_circ_	0.360171	0.142898
hsa_circ_	0.258104	0.142914
hsa_circ_	-0.26678	0.142931
hsa_circ_	0.415173	0.142939
hsa_circ_	-0.64518	0.142941
hsa_circ_	-0.30212	0.142941
hsa_circ_	0.263491	0.142953
hsa_circ_	0.273566	0.14297
hsa_circ_	-0.59946	0.142983
hsa_circ_	-0.48018	0.142999
hsa_circ_	-0.36012	0.143008
hsa_circ_	-0.5847	0.143022
hsa_circ_	0.54179	0.143023
hsa_circ_	0.327172	0.14303
hsa_circ_	-0.72563	0.143031
hsa_circ_	-0.30292	0.143031
hsa_circ_	0.44507	0.143035
hsa_circ_	-0.2887	0.143037
hsa_circ_	-0.24892	0.143056
hsa_circ_	-0.22212	0.143057
hsa_circ_	-0.36144	0.143073
hsa_circ_	-0.66626	0.143074
hsa_circ_	-0.22284	0.143094
hsa_circ_	0.246395	0.143096
hsa_circ_	0.292163	0.143099
hsa_circ_	0.333661	0.1431
hsa_circ_	-0.27276	0.143108
hsa_circ_	-0.64227	0.143118
hsa_circ_	0.858136	0.14312
hsa_circ_	-0.42839	0.143121
hsa_circ_	-0.28478	0.143131
hsa_circ_	0.255645	0.143147
hsa_circ_	0.215987	0.143156
hsa_circ_	0.309315	0.14316
hsa_circ_	-0.38587	0.143171
hsa_circ_	-0.30951	0.143195
hsa_circ_	-0.67973	0.143204
hsa_circ_	0.732821	0.143212
hsa_circ_	0.261368	0.143231
hsa_circ_	1.074618	0.143241
hsa_circ_	0.635813	0.143249
hsa_circ_	0.310783	0.143257
hsa_circ_	-0.47733	0.143264
hsa_circ_	0.2131	0.143266
hsa_circ_	0.467867	0.143275

hsa_circ_	-0.53194	0.143276
hsa_circ_	0.361839	0.143292
hsa_circ_	-0.41774	0.143301
hsa_circ_	-0.64815	0.143302
hsa_circ_	-0.25874	0.143304
hsa_circ_	0.473978	0.143313
hsa_circ_	-0.29084	0.143318
hsa_circ_	-0.52046	0.143336
hsa_circ_	0.324691	0.143339
hsa_circ_	0.46368	0.14335
hsa_circ_	-0.23066	0.143351
hsa_circ_	-0.52425	0.143354
hsa_circ_	0.318234	0.143355
hsa_circ_	-0.43842	0.143363
hsa_circ_	0.722758	0.143371
hsa_circ_	0.399838	0.143375
hsa_circ_	-0.57591	0.143375
hsa_circ_	-0.2467	0.143377
hsa_circ_	0.637386	0.143383
hsa_circ_	0.266176	0.143388
hsa_circ_	0.697708	0.143407
hsa_circ_	0.374488	0.143417
hsa_circ_	0.277596	0.143418
hsa_circ_	0.411361	0.14342
hsa_circ_	-0.2956	0.143434
hsa_circ_	-0.21297	0.143438
hsa_circ_	-0.24116	0.14344
hsa_circ_	0.249097	0.143441
hsa_circ_	-0.92624	0.143452
hsa_circ_	-0.28216	0.143457
hsa_circ_	-0.23144	0.143474
hsa_circ_	-0.2966	0.143475
hsa_circ_	0.917288	0.143476
hsa_circ_	-1.09269	0.14348
hsa_circ_	-0.48277	0.143481
hsa_circ_	0.285483	0.143482
hsa_circ_	0.316755	0.143494
hsa_circ_	-0.2491	0.143495
hsa_circ_	0.308238	0.143501
hsa_circ_	-0.92318	0.143506
hsa_circ_	0.739676	0.143507
hsa_circ_	0.358305	0.143509
hsa_circ_	0.419692	0.143511
hsa_circ_	-0.21447	0.143511
hsa_circ_	0.355049	0.143516
hsa_circ_	-0.32508	0.143528
hsa_circ_	0.680521	0.143535
hsa_circ_	-0.26478	0.143537
hsa_circ_	-0.30632	0.143556
hsa_circ_	-0.22504	0.143561
hsa_circ_	0.327806	0.143586
hsa_circ_	0.377846	0.143591
hsa_circ_	0.675266	0.143599
hsa_circ_	-0.26203	0.143608

hsa_circ_	0.391192	0.143625
hsa_circ_	0.258258	0.143633
hsa_circ_	-0.42107	0.14365
hsa_circ_	0.287028	0.143655
hsa_circ_	-0.86283	0.14367
hsa_circ_	-0.29122	0.143675
hsa_circ_	-0.34937	0.143683
hsa_circ_	0.780698	0.143692
hsa_circ_	0.27191	0.143692
hsa_circ_	-0.27937	0.143718
hsa_circ_	0.407097	0.143719
hsa_circ_	0.295072	0.143722
hsa_circ_	0.347488	0.143724
hsa_circ_	-0.64323	0.143741
hsa_circ_	0.213777	0.143754
hsa_circ_	0.301333	0.143773
hsa_circ_	-0.25193	0.143781
hsa_circ_	0.362832	0.143789
hsa_circ_	-0.25456	0.143803
hsa_circ_	0.464577	0.143807
hsa_circ_	-0.41998	0.143812
hsa_circ_	1.233405	0.143813
hsa_circ_	-0.93706	0.143817
hsa_circ_	-0.57065	0.14382
hsa_circ_	-0.40724	0.143826
hsa_circ_	0.233198	0.14383
hsa_circ_	0.306854	0.143837
hsa_circ_	0.300648	0.14386
hsa_circ_	0.304636	0.143872
hsa_circ_	-0.39294	0.143877
hsa_circ_	0.233453	0.143907
hsa_circ_	0.903656	0.14391
hsa_circ_	-0.43246	0.143919
hsa_circ_	0.569382	0.14393
hsa_circ_	-0.77655	0.143946
hsa_circ_	0.680436	0.143949
hsa_circ_	-0.26093	0.14395
hsa_circ_	0.202822	0.143959
hsa_circ_	0.262482	0.143963
hsa_circ_	0.328282	0.143964
hsa_circ_	-0.34543	0.143965
hsa_circ_	0.260872	0.143981
hsa_circ_	-0.3237	0.143999
hsa_circ_	-0.53956	0.144017
hsa_circ_	0.200335	0.144026
hsa_circ_	-0.35794	0.144026
hsa_circ_	-0.31646	0.144028
hsa_circ_	-0.6332	0.144028
hsa_circ_	0.290988	0.144034
hsa_circ_	-0.26566	0.144047
hsa_circ_	-0.52233	0.144057
hsa_circ_	0.697143	0.144062
hsa_circ_	0.212276	0.144078
hsa_circ_	0.223814	0.144097

hsa_circ_	0.619034	0.144113
hsa_circ_	0.651845	0.144122
hsa_circ_	0.365872	0.144136
hsa_circ_	-0.86538	0.14414
hsa_circ_	1.063513	0.14414
hsa_circ_	-0.25961	0.144145
hsa_circ_	0.438379	0.144146
hsa_circ_	0.961948	0.14415
hsa_circ_	-0.22695	0.144152
hsa_circ_	0.288721	0.144165
hsa_circ_	-0.32889	0.144174
hsa_circ_	-0.28845	0.144176
hsa_circ_	0.278632	0.144189
hsa_circ_	-1.04616	0.144236
hsa_circ_	0.464551	0.144243
hsa_circ_	-1.09242	0.144265
hsa_circ_	-0.44834	0.144266
hsa_circ_	0.227842	0.144269
hsa_circ_	0.542986	0.144291
hsa_circ_	-0.57824	0.144296
hsa_circ_	-0.40886	0.144298
hsa_circ_	0.387682	0.14431
hsa_circ_	1.087496	0.144322
hsa_circ_	-0.45373	0.144348
hsa_circ_	0.252464	0.144358
hsa_circ_	0.277665	0.144358
hsa_circ_	0.282201	0.144377
hsa_circ_	-0.51931	0.144377
hsa_circ_	0.273467	0.14438
hsa_circ_	-0.27882	0.144387
hsa_circ_	-0.32023	0.144411
hsa_circ_	0.212459	0.144412
hsa_circ_	0.610445	0.14442
hsa_circ_	-0.92467	0.14442
hsa_circ_	-0.26883	0.144423
hsa_circ_	0.812337	0.144424
hsa_circ_	-0.31991	0.144426
hsa_circ_	0.631773	0.144439
hsa_circ_	-0.38671	0.144459
hsa_circ_	-0.58378	0.144459
hsa_circ_	-0.33223	0.144459
hsa_circ_	-0.66364	0.144465
hsa_circ_	0.310471	0.144479
hsa_circ_	-0.53787	0.14448
hsa_circ_	0.505034	0.144485
hsa_circ_	0.410236	0.144488
hsa_circ_	0.256029	0.144495
hsa_circ_	0.520607	0.144501
hsa_circ_	-0.50579	0.144509
hsa_circ_	-0.65368	0.144515
hsa_circ_	1.00991	0.144522
hsa_circ_	-0.75948	0.144535
hsa_circ_	0.251365	0.144548
hsa_circ_	-0.29017	0.144551

hsa_circ_	-0.37946	0.144553
hsa_circ_	-0.37636	0.144571
hsa_circ_	-0.92801	0.144589
hsa_circ_	-0.23524	0.14459
hsa_circ_	-0.30813	0.144601
hsa_circ_	-0.89624	0.144614
hsa_circ_	-0.62145	0.144622
hsa_circ_	0.615949	0.144628
hsa_circ_	-0.2474	0.144635
hsa_circ_	-0.25829	0.144638
hsa_circ_	0.271127	0.144652
hsa_circ_	-0.39896	0.144654
hsa_circ_	-0.33546	0.14466
hsa_circ_	0.270161	0.144664
hsa_circ_	-0.52612	0.144669
hsa_circ_	-0.23896	0.144671
hsa_circ_	0.316135	0.144676
hsa_circ_	0.43927	0.144679
hsa_circ_	-0.26169	0.144699
hsa_circ_	0.327872	0.144706
hsa_circ_	-0.37906	0.14471
hsa_circ_	-0.60731	0.144724
hsa_circ_	0.294782	0.144742
hsa_circ_	-0.83106	0.144747
hsa_circ_	-0.37723	0.144755
hsa_circ_	-0.24128	0.144762
hsa_circ_	-0.27176	0.144766
hsa_circ_	-0.23267	0.14479
hsa_circ_	-0.74881	0.144791
hsa_circ_	-0.26556	0.144805
hsa_circ_	-0.31868	0.144806
hsa_circ_	-0.41228	0.144809
hsa_circ_	0.429309	0.144809
hsa_circ_	-0.68739	0.144827
hsa_circ_	0.277582	0.14483
hsa_circ_	-0.54611	0.144833
hsa_circ_	-0.26682	0.144838
hsa_circ_	0.216953	0.144845
hsa_circ_	0.408653	0.144851
hsa_circ_	-0.34714	0.144857
hsa_circ_	-0.22966	0.144862
hsa_circ_	0.36079	0.144864
hsa_circ_	-0.36894	0.14487
hsa_circ_	0.571054	0.144876
hsa_circ_	-0.292	0.144876
hsa_circ_	0.270195	0.144883
hsa_circ_	-0.51929	0.144918
hsa_circ_	-0.84587	0.144925
hsa_circ_	0.285079	0.144932
hsa_circ_	-0.53687	0.144936
hsa_circ_	0.324691	0.144957
hsa_circ_	-0.21172	0.144963
hsa_circ_	-0.43218	0.144964
hsa_circ_	0.396179	0.144971

hsa_circ_	0.322907	0.144999
hsa_circ_	0.292076	0.145004
hsa_circ_	-0.5415	0.14502
hsa_circ_	0.457953	0.145023
hsa_circ_	0.20739	0.145043
hsa_circ_	0.26697	0.145059
hsa_circ_	-0.65304	0.145059
hsa_circ_	0.35541	0.145063
hsa_circ_	-0.23456	0.145066
hsa_circ_	-0.25705	0.14507
hsa_circ_	-0.31613	0.14507
hsa_circ_	-0.73944	0.145075
hsa_circ_	0.32819	0.145077
hsa_circ_	-0.58368	0.145082
hsa_circ_	-0.57367	0.145084
hsa_circ_	-0.32081	0.145087
hsa_circ_	-0.61543	0.145096
hsa_circ_	0.33233	0.145102
hsa_circ_	-0.22415	0.145106
hsa_circ_	0.313791	0.145116
hsa_circ_	-0.41559	0.145116
hsa_circ_	-0.23553	0.14512
hsa_circ_	-0.24125	0.145129
hsa_circ_	0.705347	0.145133
hsa_circ_	0.32706	0.14516
hsa_circ_	0.274329	0.145161
hsa_circ_	0.264087	0.145199
hsa_circ_	0.857007	0.1452
hsa_circ_	1.030847	0.145207
hsa_circ_	0.450631	0.145219
hsa_circ_	0.703446	0.145224
hsa_circ_	-0.93993	0.145224
hsa_circ_	0.712607	0.145226
hsa_circ_	-0.6824	0.145241
hsa_circ_	0.292531	0.145255
hsa_circ_	-0.59839	0.145259
hsa_circ_	-0.2651	0.145275
hsa_circ_	-0.26171	0.145292
hsa_circ_	-0.3015	0.145293
hsa_circ_	0.229017	0.145294
hsa_circ_	0.545212	0.14531
hsa_circ_	-0.27487	0.145314
hsa_circ_	0.252549	0.145333
hsa_circ_	-0.32846	0.145335
hsa_circ_	0.783957	0.145338
hsa_circ_	0.384458	0.145352
hsa_circ_	-0.69634	0.145358
hsa_circ_	0.472176	0.14537
hsa_circ_	0.368075	0.14538
hsa_circ_	0.387168	0.145391
hsa_circ_	0.461628	0.145392
hsa_circ_	0.32851	0.145396
hsa_circ_	0.786406	0.145404
hsa_circ_	-0.49411	0.145406

hsa_circ_	-0.47087	0.145408
hsa_circ_	0.243216	0.145422
hsa_circ_	-0.24988	0.145425
hsa_circ_	0.34908	0.145428
hsa_circ_	0.915304	0.14543
hsa_circ_	0.305115	0.145433
hsa_circ_	-0.537	0.14546
hsa_circ_	-0.39479	0.145474
hsa_circ_	-0.23117	0.145476
hsa_circ_	-0.2824	0.145481
hsa_circ_	0.493192	0.14549
hsa_circ_	0.296563	0.145493
hsa_circ_	-0.24369	0.145494
hsa_circ_	-0.31578	0.145517
hsa_circ_	-0.31407	0.145519
hsa_circ_	-0.29127	0.145531
hsa_circ_	0.974514	0.145535
hsa_circ_	-0.3725	0.145539
hsa_circ_	-0.23236	0.145553
hsa_circ_	-0.24153	0.145564
hsa_circ_	0.252638	0.145586
hsa_circ_	-0.34021	0.145602
hsa_circ_	-0.69115	0.145616
hsa_circ_	0.285955	0.145634
hsa_circ_	0.802521	0.145642
hsa_circ_	-0.25713	0.145653
hsa_circ_	0.653628	0.145654
hsa_circ_	-0.44556	0.145667
hsa_circ_	-0.25196	0.145668
hsa_circ_	-0.25556	0.145677
hsa_circ_	-0.31572	0.145686
hsa_circ_	0.285074	0.145705
hsa_circ_	-0.43609	0.145706
hsa_circ_	-0.3284	0.145718
hsa_circ_	0.293698	0.145721
hsa_circ_	-0.26044	0.14573
hsa_circ_	0.216877	0.145738
hsa_circ_	0.262261	0.14574
hsa_circ_	0.23735	0.145749
hsa_circ_	-1.19887	0.145768
hsa_circ_	-0.25492	0.145771
hsa_circ_	-0.44514	0.145771
hsa_circ_	-2.47015	0.145784
hsa_circ_	0.312125	0.145789
hsa_circ_	-0.35244	0.14579
hsa_circ_	-0.30214	0.145793
hsa_circ_	-0.64718	0.145794
hsa_circ_	0.225327	0.1458
hsa_circ_	0.543233	0.1458
hsa_circ_	-0.44615	0.145801
hsa_circ_	-0.56931	0.145805
hsa_circ_	0.426837	0.145812
hsa_circ_	-0.84703	0.145814
hsa_circ_	-0.24084	0.145827

hsa_circ_	0.446473	0.145832
hsa_circ_	-0.38963	0.145834
hsa_circ_	-0.24066	0.14584
hsa_circ_	0.230234	0.145841
hsa_circ_	-0.333	0.145849
hsa_circ_	-0.21528	0.145893
hsa_circ_	0.239595	0.145905
hsa_circ_	-0.30243	0.145913
hsa_circ_	0.290733	0.14593
hsa_circ_	-0.20233	0.145931
hsa_circ_	-0.97368	0.145948
hsa_circ_	-0.26483	0.145959
hsa_circ_	0.238195	0.14596
hsa_circ_	0.42146	0.145964
hsa_circ_	0.621763	0.145983
hsa_circ_	0.723502	0.145985
hsa_circ_	0.220014	0.145986
hsa_circ_	0.315366	0.145988
hsa_circ_	0.360319	0.145989
hsa_circ_	-0.2151	0.145992
hsa_circ_	0.642314	0.145994
hsa_circ_	0.290627	0.146
hsa_circ_	-0.22532	0.146009
hsa_circ_	-0.3075	0.146014
hsa_circ_	0.362948	0.146018
hsa_circ_	0.245276	0.146023
hsa_circ_	-0.24756	0.146036
hsa_circ_	0.352579	0.146036
hsa_circ_	-0.42539	0.146049
hsa_circ_	-0.29695	0.146056
hsa_circ_	0.747722	0.146071
hsa_circ_	-0.33571	0.146073
hsa_circ_	-0.26075	0.146093
hsa_circ_	-0.87103	0.146101
hsa_circ_	0.351469	0.146113
hsa_circ_	0.403687	0.146132
hsa_circ_	-0.21635	0.146138
hsa_circ_	0.603179	0.146149
hsa_circ_	0.268766	0.146153
hsa_circ_	0.306409	0.146158
hsa_circ_	0.937954	0.146159
hsa_circ_	0.359985	0.146159
hsa_circ_	0.340942	0.146168
hsa_circ_	0.237332	0.14617
hsa_circ_	-0.26663	0.146178
hsa_circ_	0.266438	0.146186
hsa_circ_	0.279638	0.146188
hsa_circ_	-0.25056	0.146192
hsa_circ_	-0.46008	0.146198
hsa_circ_	-0.35736	0.146204
hsa_circ_	0.422866	0.14621
hsa_circ_	0.308356	0.146222
hsa_circ_	-1.12758	0.146224
hsa_circ_	-0.40659	0.146225

hsa_circ_	0.686418	0.146235
hsa_circ_	0.294151	0.146235
hsa_circ_	0.25783	0.146236
hsa_circ_	-0.22016	0.146246
hsa_circ_	-0.46374	0.146271
hsa_circ_	-0.32451	0.146276
hsa_circ_	0.348943	0.14628
hsa_circ_	0.809019	0.146287
hsa_circ_	0.229719	0.146297
hsa_circ_	0.247484	0.146304
hsa_circ_	-0.23586	0.146304
hsa_circ_	0.798361	0.146315
hsa_circ_	-0.31754	0.146316
hsa_circ_	-0.23958	0.14632
hsa_circ_	0.243951	0.146327
hsa_circ_	0.479051	0.146327
hsa_circ_	-0.58762	0.146341
hsa_circ_	-0.56833	0.146342
hsa_circ_	0.396103	0.146348
hsa_circ_	-0.41286	0.146348
hsa_circ_	-0.41341	0.146359
hsa_circ_	0.212774	0.146363
hsa_circ_	0.803112	0.14639
hsa_circ_	0.579123	0.146393
hsa_circ_	0.312866	0.1464
hsa_circ_	1.261594	0.146416
hsa_circ_	0.252161	0.146436
hsa_circ_	-0.34314	0.146452
hsa_circ_	-0.31274	0.146455
hsa_circ_	0.660072	0.146457
hsa_circ_	0.366564	0.146465
hsa_circ_	0.226111	0.146474
hsa_circ_	-0.24102	0.14648
hsa_circ_	0.278918	0.146492
hsa_circ_	-0.36972	0.146517
hsa_circ_	0.329385	0.146545
hsa_circ_	-0.24669	0.146552
hsa_circ_	0.216003	0.146552
hsa_circ_	-0.22697	0.146556
hsa_circ_	0.278089	0.146559
hsa_circ_	-0.37575	0.146568
hsa_circ_	-0.23758	0.14657
hsa_circ_	-0.54007	0.146576
hsa_circ_	0.563604	0.146576
hsa_circ_	-0.23874	0.146612
hsa_circ_	0.303513	0.146615
hsa_circ_	-0.77463	0.146633
hsa_circ_	-0.25474	0.146636
hsa_circ_	0.258264	0.146657
hsa_circ_	0.395888	0.146662
hsa_circ_	-0.46894	0.146663
hsa_circ_	-0.25638	0.14667
hsa_circ_	-0.2048	0.146673
hsa_circ_	0.627848	0.146676

hsa_circ_	0.212353	0.146678
hsa_circ_	0.241709	0.14668
hsa_circ_	0.626237	0.146684
hsa_circ_	-0.36009	0.146694
hsa_circ_	0.69241	0.146698
hsa_circ_	-0.87063	0.146708
hsa_circ_	0.263086	0.146713
hsa_circ_	-0.33253	0.146715
hsa_circ_	0.450507	0.146723
hsa_circ_	-0.32951	0.146726
hsa_circ_	-0.38703	0.146728
hsa_circ_	-0.21601	0.146728
hsa_circ_	-0.25064	0.146734
hsa_circ_	-0.25061	0.146738
hsa_circ_	0.329341	0.146742
hsa_circ_	-0.26635	0.146743
hsa_circ_	0.505111	0.146745
hsa_circ_	0.446118	0.146749
hsa_circ_	-0.30505	0.146772
hsa_circ_	0.827714	0.146801
hsa_circ_	1.34514	0.146805
hsa_circ_	-0.3472	0.146825
hsa_circ_	-0.36499	0.146839
hsa_circ_	0.586576	0.14684
hsa_circ_	0.421869	0.146852
hsa_circ_	-0.24496	0.146855
hsa_circ_	0.3292	0.146862
hsa_circ_	-0.38806	0.146862
hsa_circ_	-1.13196	0.146866
hsa_circ_	0.657556	0.146868
hsa_circ_	-1.31722	0.146875
hsa_circ_	-0.48532	0.146884
hsa_circ_	0.416747	0.146885
hsa_circ_	-0.78635	0.146889
hsa_circ_	0.654685	0.146891
hsa_circ_	0.51915	0.146897
hsa_circ_	-0.32985	0.146904
hsa_circ_	0.256017	0.146909
hsa_circ_	-0.3108	0.146912
hsa_circ_	-0.25628	0.146912
hsa_circ_	0.347479	0.146915
hsa_circ_	-0.28844	0.146918
hsa_circ_	-0.35936	0.146921
hsa_circ_	-0.21489	0.146929
hsa_circ_	0.337711	0.14693
hsa_circ_	-0.23536	0.146938
hsa_circ_	-0.25708	0.146974
hsa_circ_	-0.35135	0.146974
hsa_circ_	0.356878	0.146986
hsa_circ_	0.314073	0.146992
hsa_circ_	-0.36564	0.146998
hsa_circ_	0.287716	0.147
hsa_circ_	-0.4193	0.147024
hsa_circ_	-0.25031	0.147024

hsa_circ_	0.52504	0.147033
hsa_circ_	-0.31245	0.147034
hsa_circ_	0.272323	0.147035
hsa_circ_	-0.41643	0.147078
hsa_circ_	0.264432	0.147083
hsa_circ_	-0.29832	0.147085
hsa_circ_	0.548333	0.147094
hsa_circ_	0.51342	0.147094
hsa_circ_	-0.26176	0.147094
hsa_circ_	0.392631	0.147114
hsa_circ_	-0.22768	0.147124
hsa_circ_	-0.51574	0.147127
hsa_circ_	0.394325	0.147172
hsa_circ_	0.275871	0.147174
hsa_circ_	0.313343	0.147183
hsa_circ_	0.48463	0.147192
hsa_circ_	-0.21647	0.147197
hsa_circ_	-0.23025	0.14721
hsa_circ_	-0.24497	0.147225
hsa_circ_	-0.35762	0.147229
hsa_circ_	0.41104	0.147233
hsa_circ_	0.801192	0.147239
hsa_circ_	-0.24595	0.147241
hsa_circ_	0.269072	0.147254
hsa_circ_	0.392324	0.147255
hsa_circ_	0.413164	0.147257
hsa_circ_	0.584644	0.147262
hsa_circ_	0.259502	0.147272
hsa_circ_	-0.58603	0.147304
hsa_circ_	-0.29307	0.147314
hsa_circ_	0.261413	0.147332
hsa_circ_	-0.27855	0.147338
hsa_circ_	0.234436	0.147356
hsa_circ_	0.269957	0.147363
hsa_circ_	0.444424	0.147364
hsa_circ_	0.340687	0.147364
hsa_circ_	0.271853	0.147369
hsa_circ_	-0.2197	0.14737
hsa_circ_	-0.2506	0.14737
hsa_circ_	0.342721	0.147374
hsa_circ_	-0.52111	0.147375
hsa_circ_	-0.4056	0.147387
hsa_circ_	-0.82242	0.147391
hsa_circ_	0.335594	0.147407
hsa_circ_	-0.65335	0.147408
hsa_circ_	1.155719	0.14742
hsa_circ_	-0.23981	0.147454
hsa_circ_	-1.22612	0.147464
hsa_circ_	-0.33484	0.147477
hsa_circ_	-0.39103	0.14749
hsa_circ_	-0.26978	0.147509
hsa_circ_	0.240637	0.14751
hsa_circ_	-0.45611	0.147515
hsa_circ_	-0.28036	0.14752

hsa_circ_	-0.2929	0.147522
hsa_circ_	0.524258	0.147548
hsa_circ_	-0.49089	0.147553
hsa_circ_	-0.21602	0.14757
hsa_circ_	-2.73709	0.147573
hsa_circ_	-0.36255	0.147574
hsa_circ_	0.367827	0.14761
hsa_circ_	-0.21845	0.147627
hsa_circ_	-0.33541	0.14763
hsa_circ_	-0.29455	0.147633
hsa_circ_	0.315643	0.14764
hsa_circ_	-0.2833	0.147642
hsa_circ_	-0.32714	0.147643
hsa_circ_	0.257938	0.147643
hsa_circ_	-0.29869	0.147649
hsa_circ_	-0.34991	0.147663
hsa_circ_	0.34123	0.147665
hsa_circ_	-0.38558	0.147685
hsa_circ_	0.328965	0.147689
hsa_circ_	-0.52688	0.147692
hsa_circ_	0.650367	0.147725
hsa_circ_	-0.40453	0.147732
hsa_circ_	-0.41172	0.147736
hsa_circ_	0.237331	0.147742
hsa_circ_	0.293668	0.147751
hsa_circ_	-0.35725	0.147753
hsa_circ_	-0.93149	0.147755
hsa_circ_	0.301989	0.147755
hsa_circ_	-0.22306	0.147763
hsa_circ_	0.226506	0.147769
hsa_circ_	0.391768	0.147776
hsa_circ_	0.626641	0.147778
hsa_circ_	0.415188	0.147814
hsa_circ_	-0.41406	0.147818
hsa_circ_	0.482697	0.147831
hsa_circ_	-0.46972	0.147861
hsa_circ_	0.239344	0.147867
hsa_circ_	0.364296	0.14787
hsa_circ_	-0.30292	0.14787
hsa_circ_	-0.95744	0.14787
hsa_circ_	0.37556	0.147892
hsa_circ_	-0.25515	0.147899
hsa_circ_	-0.26352	0.147907
hsa_circ_	0.289126	0.147909
hsa_circ_	-0.27534	0.14791
hsa_circ_	-0.237	0.147912
hsa_circ_	-0.30514	0.147931
hsa_circ_	0.24757	0.147963
hsa_circ_	-0.27596	0.147965
hsa_circ_	-0.31498	0.147984
hsa_circ_	-0.22838	0.147987
hsa_circ_	0.209665	0.148001
hsa_circ_	-0.30886	0.148004
hsa_circ_	-0.31893	0.148011

hsa_circ_	0.798339	0.148014
hsa_circ_	-0.24385	0.148014
hsa_circ_	0.248293	0.148016
hsa_circ_	-0.90082	0.148025
hsa_circ_	0.520553	0.148039
hsa_circ_	-0.26403	0.148045
hsa_circ_	-0.27978	0.148054
hsa_circ_	-0.42727	0.148057
hsa_circ_	0.279971	0.148067
hsa_circ_	0.333747	0.148069
hsa_circ_	-0.78611	0.148082
hsa_circ_	-0.80846	0.148094
hsa_circ_	0.499147	0.148106
hsa_circ_	-0.56567	0.148107
hsa_circ_	0.471409	0.148126
hsa_circ_	0.302051	0.148148
hsa_circ_	-0.44359	0.148157
hsa_circ_	-0.35855	0.148172
hsa_circ_	0.301254	0.148193
hsa_circ_	-1.11568	0.148193
hsa_circ_	-0.3812	0.148204
hsa_circ_	-0.2308	0.148207
hsa_circ_	-0.22771	0.14821
hsa_circ_	-0.40362	0.148216
hsa_circ_	0.22461	0.148218
hsa_circ_	-0.26362	0.148221
hsa_circ_	-0.29924	0.148242
hsa_circ_	-0.35873	0.148245
hsa_circ_	0.235586	0.148248
hsa_circ_	-0.75811	0.148259
hsa_circ_	0.263566	0.148268
hsa_circ_	-0.56005	0.148275
hsa_circ_	0.682772	0.148288
hsa_circ_	0.618701	0.148288
hsa_circ_	-0.23114	0.148289
hsa_circ_	-0.34456	0.14829
hsa_circ_	-0.47462	0.148291
hsa_circ_	0.344925	0.148295
hsa_circ_	0.435921	0.148298
hsa_circ_	0.274753	0.148303
hsa_circ_	-0.8942	0.148312
hsa_circ_	0.268382	0.148332
hsa_circ_	-0.25863	0.148351
hsa_circ_	0.212631	0.148361
hsa_circ_	-0.41042	0.148361
hsa_circ_	-0.44387	0.148378
hsa_circ_	-0.83449	0.14838
hsa_circ_	0.273806	0.148385
hsa_circ_	0.41837	0.148391
hsa_circ_	1.61507	0.148393
hsa_circ_	-0.2179	0.14841
hsa_circ_	-1.51263	0.148427
hsa_circ_	0.199215	0.148435
hsa_circ_	0.273022	0.148442

hsa_circ_	-0.59812	0.148442
hsa_circ_	0.403017	0.148448
hsa_circ_	0.220273	0.148459
hsa_circ_	-0.34932	0.148464
hsa_circ_	0.282249	0.14847
hsa_circ_	0.481521	0.148493
hsa_circ_	-0.2365	0.148498
hsa_circ_	-0.29548	0.148498
hsa_circ_	0.282806	0.148506
hsa_circ_	0.299357	0.148509
hsa_circ_	-0.40291	0.148525
hsa_circ_	-0.33236	0.148527
hsa_circ_	0.294805	0.148539
hsa_circ_	-0.26794	0.148561
hsa_circ_	-0.28803	0.148565
hsa_circ_	0.526721	0.148565
hsa_circ_	0.297861	0.148567
hsa_circ_	-0.28876	0.148568
hsa_circ_	0.269769	0.148569
hsa_circ_	-0.32985	0.14858
hsa_circ_	-0.30675	0.148587
hsa_circ_	0.607211	0.148596
hsa_circ_	-0.30454	0.148601
hsa_circ_	-1.33858	0.148603
hsa_circ_	0.331981	0.148624
hsa_circ_	-0.3517	0.14863
hsa_circ_	0.457022	0.148636
hsa_circ_	0.338793	0.148651
hsa_circ_	0.281357	0.148659
hsa_circ_	-0.98926	0.148664
hsa_circ_	0.256645	0.148665
hsa_circ_	-0.77215	0.148669
hsa_circ_	0.40424	0.148676
hsa_circ_	-1.89748	0.148677
hsa_circ_	-0.27315	0.148721
hsa_circ_	0.344511	0.148732
hsa_circ_	-0.26033	0.148743
hsa_circ_	0.860756	0.14875
hsa_circ_	0.239541	0.148758
hsa_circ_	-0.29986	0.148762
hsa_circ_	0.545749	0.148769
hsa_circ_	-0.51529	0.14877
hsa_circ_	0.323089	0.148788
hsa_circ_	-0.6326	0.14879
hsa_circ_	-0.24752	0.148808
hsa_circ_	0.584972	0.148841
hsa_circ_	0.491916	0.148855
hsa_circ_	-0.30127	0.148861
hsa_circ_	-0.4437	0.148865
hsa_circ_	-0.86469	0.148872
hsa_circ_	-0.52706	0.148874
hsa_circ_	-0.68771	0.148885
hsa_circ_	-0.40143	0.148887
hsa_circ_	0.373003	0.148887

hsa_circ_	0.475105	0.148901
hsa_circ_	-0.36813	0.14891
hsa_circ_	0.570599	0.148918
hsa_circ_	0.344622	0.14892
hsa_circ_	0.604514	0.148924
hsa_circ_	-0.27446	0.148934
hsa_circ_	0.34235	0.148939
hsa_circ_	0.300314	0.148948
hsa_circ_	0.265925	0.148953
hsa_circ_	0.281296	0.148957
hsa_circ_	-0.25836	0.148962
hsa_circ_	0.308508	0.148968
hsa_circ_	0.394882	0.148969
hsa_circ_	0.325708	0.148977
hsa_circ_	-0.79772	0.148987
hsa_circ_	0.471888	0.14899
hsa_circ_	0.406674	0.149014
hsa_circ_	0.252546	0.149031
hsa_circ_	0.338095	0.149033
hsa_circ_	0.283885	0.149036
hsa_circ_	0.235956	0.149051
hsa_circ_	0.703518	0.149062
hsa_circ_	0.464256	0.149063
hsa_circ_	0.202224	0.14909
hsa_circ_	0.836869	0.149096
hsa_circ_	-0.20604	0.149098
hsa_circ_	0.817547	0.149108
hsa_circ_	-0.26648	0.149112
hsa_circ_	0.330825	0.149119
hsa_circ_	-0.53646	0.149125
hsa_circ_	-0.45878	0.149132
hsa_circ_	-0.48774	0.149133
hsa_circ_	0.300424	0.149155
hsa_circ_	0.564266	0.149167
hsa_circ_	0.352623	0.149167
hsa_circ_	-0.39299	0.149173
hsa_circ_	-0.37302	0.14918
hsa_circ_	-0.33742	0.14919
hsa_circ_	-0.22549	0.149207
hsa_circ_	0.360683	0.149231
hsa_circ_	0.492029	0.149241
hsa_circ_	0.245356	0.149255
hsa_circ_	0.309128	0.149277
hsa_circ_	0.241635	0.149277
hsa_circ_	0.332442	0.14928
hsa_circ_	-0.36508	0.149304
hsa_circ_	-0.26389	0.14931
hsa_circ_	-0.63366	0.149313
hsa_circ_	-0.32398	0.149321
hsa_circ_	0.234125	0.149322
hsa_circ_	0.520219	0.14936
hsa_circ_	-0.42736	0.149363
hsa_circ_	-0.28744	0.149364
hsa_circ_	0.348009	0.149365

hsa_circ_	1.685782	0.149369
hsa_circ_	-0.30135	0.149389
hsa_circ_	-0.29802	0.149399
hsa_circ_	-0.3606	0.1494
hsa_circ_	0.277106	0.149417
hsa_circ_	0.714764	0.149428
hsa_circ_	-0.95774	0.14943
hsa_circ_	-0.34559	0.149434
hsa_circ_	0.38253	0.149442
hsa_circ_	0.267738	0.149444
hsa_circ_	0.322993	0.149479
hsa_circ_	-0.51966	0.149485
hsa_circ_	0.511666	0.1495
hsa_circ_	0.21517	0.149502
hsa_circ_	-0.24442	0.149507
hsa_circ_	-0.27511	0.149507
hsa_circ_	-0.36869	0.149515
hsa_circ_	-0.26151	0.149516
hsa_circ_	-0.38178	0.149522
hsa_circ_	-0.23429	0.149532
hsa_circ_	0.373277	0.149539
hsa_circ_	0.350544	0.149539
hsa_circ_	0.202781	0.149567
hsa_circ_	-0.37131	0.149573
hsa_circ_	-0.49513	0.149581
hsa_circ_	-0.29163	0.149588
hsa_circ_	0.225983	0.14959
hsa_circ_	-0.43257	0.149606
hsa_circ_	-0.74171	0.14962
hsa_circ_	0.356623	0.149636
hsa_circ_	0.463961	0.149638
hsa_circ_	0.507995	0.149639
hsa_circ_	0.23774	0.149643
hsa_circ_	0.526303	0.149647
hsa_circ_	0.580366	0.149648
hsa_circ_	-0.32234	0.149655
hsa_circ_	-0.38372	0.149673
hsa_circ_	-0.34301	0.149674
hsa_circ_	0.239702	0.149678
hsa_circ_	-0.20179	0.149681
hsa_circ_	-0.22483	0.149694
hsa_circ_	0.59835	0.149713
hsa_circ_	-0.48895	0.149715
hsa_circ_	-0.33856	0.149719
hsa_circ_	0.277419	0.149735
hsa_circ_	-0.78982	0.149736
hsa_circ_	0.262087	0.149743
hsa_circ_	0.397532	0.149754
hsa_circ_	1.888336	0.149761
hsa_circ_	0.280648	0.149762
hsa_circ_	0.294271	0.149766
hsa_circ_	0.251398	0.149767
hsa_circ_	0.327301	0.149783
hsa_circ_	0.346716	0.149795

hsa_circ_	0.273366	0.149795
hsa_circ_	0.573573	0.1498
hsa_circ_	-0.65392	0.149811
hsa_circ_	0.254137	0.149829
hsa_circ_	-0.32713	0.149831
hsa_circ_	-0.22609	0.149842
hsa_circ_	0.236792	0.149843
hsa_circ_	0.487671	0.149851
hsa_circ_	0.451144	0.149865
hsa_circ_	0.349976	0.149877
hsa_circ_	-0.20667	0.149879
hsa_circ_	-0.20957	0.149881
hsa_circ_	-0.28193	0.149884
hsa_circ_	0.622473	0.149909
hsa_circ_	-0.4312	0.149911
hsa_circ_	-0.88618	0.149922
hsa_circ_	-0.82937	0.149925
hsa_circ_	0.339719	0.149932
hsa_circ_	0.39592	0.149937
hsa_circ_	1.005591	0.149947
hsa_circ_	-0.28197	0.149954
hsa_circ_	-0.38576	0.149961
hsa_circ_	-0.46518	0.149968
hsa_circ_	0.444131	0.149988
hsa_circ_	0.247947	0.149991
hsa_circ_	-0.88482	0.150002
hsa_circ_	0.419992	0.150008
hsa_circ_	-0.43214	0.150014
hsa_circ_	0.419826	0.150015
hsa_circ_	-0.34288	0.150017
hsa_circ_	0.231467	0.150028
hsa_circ_	0.34695	0.150029
hsa_circ_	0.681267	0.150033
hsa_circ_	0.331828	0.150036
hsa_circ_	-0.71264	0.150048
hsa_circ_	-0.53486	0.150048
hsa_circ_	0.42447	0.150056
hsa_circ_	-0.29151	0.150066
hsa_circ_	0.245291	0.150072
hsa_circ_	-0.46071	0.150075
hsa_circ_	0.347034	0.150078
hsa_circ_	-0.40092	0.150079
hsa_circ_	-0.36813	0.15008
hsa_circ_	0.310381	0.150101
hsa_circ_	-0.77912	0.150127
hsa_circ_	0.239722	0.150135
hsa_circ_	-1.03163	0.150148
hsa_circ_	-0.26168	0.150185
hsa_circ_	-0.34539	0.1502
hsa_circ_	-0.34378	0.150203
hsa_circ_	-0.20479	0.150205
hsa_circ_	-0.24864	0.150217
hsa_circ_	-0.22753	0.150217
hsa_circ_	-0.50416	0.150222

hsa_circ_	0.674794	0.150236
hsa_circ_	0.359991	0.150243
hsa_circ_	0.351024	0.150248
hsa_circ_	0.31377	0.150256
hsa_circ_	-0.22232	0.150258
hsa_circ_	-0.31276	0.15026
hsa_circ_	-0.24049	0.150268
hsa_circ_	-0.52252	0.150269
hsa_circ_	-0.27208	0.150272
hsa_circ_	-0.5464	0.150287
hsa_circ_	0.28861	0.150289
hsa_circ_	-0.31309	0.15029
hsa_circ_	0.345688	0.150291
hsa_circ_	-0.27257	0.150308
hsa_circ_	0.265902	0.150308
hsa_circ_	0.299044	0.150311
hsa_circ_	1.280377	0.150314
hsa_circ_	0.460516	0.150327
hsa_circ_	-0.21972	0.150339
hsa_circ_	-0.40713	0.150359
hsa_circ_	-0.43092	0.150364
hsa_circ_	-0.80325	0.150373
hsa_circ_	-0.23983	0.150391
hsa_circ_	0.410454	0.150414
hsa_circ_	0.417569	0.15042
hsa_circ_	0.231043	0.150426
hsa_circ_	-0.64608	0.150436
hsa_circ_	0.5562	0.150456
hsa_circ_	0.20494	0.150475
hsa_circ_	0.336919	0.150481
hsa_circ_	0.293898	0.150481
hsa_circ_	0.267089	0.150484
hsa_circ_	0.432089	0.150486
hsa_circ_	0.399367	0.150491
hsa_circ_	-0.23049	0.150502
hsa_circ_	-0.60955	0.150504
hsa_circ_	0.761846	0.150511
hsa_circ_	0.686391	0.150514
hsa_circ_	0.217661	0.150516
hsa_circ_	0.241782	0.150538
hsa_circ_	0.399491	0.150566
hsa_circ_	-0.21391	0.150569
hsa_circ_	-0.2286	0.150571
hsa_circ_	1.006612	0.150579
hsa_circ_	0.601707	0.150589
hsa_circ_	-0.29113	0.15059
hsa_circ_	-0.4071	0.150592
hsa_circ_	-0.25145	0.150592
hsa_circ_	0.392939	0.150596
hsa_circ_	-0.30968	0.150606
hsa_circ_	-0.36466	0.150608
hsa_circ_	-0.43818	0.150617
hsa_circ_	-0.2565	0.150621
hsa_circ_	0.320655	0.150653

hsa_circ_	0.535169	0.15066
hsa_circ_	-0.26394	0.150664
hsa_circ_	-0.30144	0.150667
hsa_circ_	0.225051	0.150674
hsa_circ_	-0.52912	0.150691
hsa_circ_	0.254223	0.150715
hsa_circ_	-0.22931	0.150719
hsa_circ_	-0.34135	0.150732
hsa_circ_	-0.28244	0.150737
hsa_circ_	0.568693	0.150751
hsa_circ_	-1.06527	0.150761
hsa_circ_	-0.29792	0.150766
hsa_circ_	-0.5513	0.150771
hsa_circ_	0.396281	0.150772
hsa_circ_	0.424477	0.150775
hsa_circ_	0.392621	0.150788
hsa_circ_	-0.41238	0.150791
hsa_circ_	-0.26103	0.150795
hsa_circ_	0.286565	0.150796
hsa_circ_	-0.27107	0.150799
hsa_circ_	-0.33276	0.150807
hsa_circ_	-0.22117	0.150812
hsa_circ_	0.251096	0.150817
hsa_circ_	-0.20571	0.150822
hsa_circ_	-0.49778	0.150828
hsa_circ_	-0.9571	0.150835
hsa_circ_	-0.31631	0.150837
hsa_circ_	0.864497	0.150852
hsa_circ_	0.554378	0.150853
hsa_circ_	-0.27267	0.150855
hsa_circ_	0.320593	0.150872
hsa_circ_	0.370879	0.150882
hsa_circ_	-0.2631	0.150887
hsa_circ_	-0.34971	0.150889
hsa_circ_	-0.60544	0.150911
hsa_circ_	0.260779	0.150924
hsa_circ_	0.309294	0.150927
hsa_circ_	-0.28158	0.150936
hsa_circ_	-0.45818	0.150952
hsa_circ_	0.279989	0.150955
hsa_circ_	-0.47352	0.150964
hsa_circ_	-1.24962	0.150983
hsa_circ_	-0.42722	0.150986
hsa_circ_	-0.63205	0.150993
hsa_circ_	-0.26641	0.151001
hsa_circ_	-0.31549	0.151007
hsa_circ_	0.372116	0.151023
hsa_circ_	-0.20103	0.15103
hsa_circ_	-0.20953	0.151042
hsa_circ_	-0.24471	0.151051
hsa_circ_	-0.34341	0.151053
hsa_circ_	-0.50634	0.151061
hsa_circ_	-0.63137	0.151067
hsa_circ_	-0.48068	0.151068

hsa_circ_	0.330197	0.151082
hsa_circ_	0.231736	0.151112
hsa_circ_	0.514404	0.151125
hsa_circ_	0.338122	0.15113
hsa_circ_	0.700619	0.151134
hsa_circ_	0.224914	0.151141
hsa_circ_	-0.21018	0.151154
hsa_circ_	-0.54664	0.151156
hsa_circ_	0.25128	0.151159
hsa_circ_	-0.30053	0.151166
hsa_circ_	-0.62587	0.151176
hsa_circ_	0.444532	0.151179
hsa_circ_	-0.25847	0.1512
hsa_circ_	-0.25324	0.151201
hsa_circ_	-0.22701	0.151214
hsa_circ_	0.468424	0.151239
hsa_circ_	0.38637	0.151267
hsa_circ_	0.248864	0.151275
hsa_circ_	0.321273	0.151292
hsa_circ_	0.285646	0.151297
hsa_circ_	0.39441	0.151297
hsa_circ_	-0.21029	0.151298
hsa_circ_	0.671058	0.151298
hsa_circ_	-0.35886	0.151303
hsa_circ_	-0.24404	0.151308
hsa_circ_	0.311389	0.15131
hsa_circ_	0.756627	0.151329
hsa_circ_	-0.34573	0.151334
hsa_circ_	-0.30166	0.151334
hsa_circ_	0.196466	0.151334
hsa_circ_	-0.20929	0.151338
hsa_circ_	-0.29711	0.151348
hsa_circ_	-0.28072	0.151349
hsa_circ_	-0.45327	0.15137
hsa_circ_	0.379737	0.151374
hsa_circ_	-0.44503	0.151374
hsa_circ_	0.255139	0.151375
hsa_circ_	0.603988	0.151378
hsa_circ_	0.382823	0.151397
hsa_circ_	0.503669	0.151401
hsa_circ_	0.97276	0.151403
hsa_circ_	-0.29273	0.151412
hsa_circ_	-0.22131	0.151415
hsa_circ_	0.238891	0.151418
hsa_circ_	-0.52316	0.151419
hsa_circ_	-0.6261	0.151427
hsa_circ_	0.462259	0.151429
hsa_circ_	-0.87934	0.151429
hsa_circ_	-0.46852	0.15143
hsa_circ_	-0.23865	0.151442
hsa_circ_	-0.32936	0.151458
hsa_circ_	0.2299	0.151466
hsa_circ_	-0.22116	0.15148
hsa_circ_	-0.35965	0.151486

hsa_circ_	0.296787	0.151497
hsa_circ_	-0.73759	0.151503
hsa_circ_	0.25761	0.151504
hsa_circ_	0.367496	0.151511
hsa_circ_	-0.24906	0.151525
hsa_circ_	1.184136	0.151564
hsa_circ_	-0.7888	0.151571
hsa_circ_	0.295727	0.151573
hsa_circ_	-0.26157	0.151574
hsa_circ_	0.388677	0.15158
hsa_circ_	0.591698	0.151592
hsa_circ_	0.255925	0.151596
hsa_circ_	0.263806	0.151621
hsa_circ_	0.213718	0.151623
hsa_circ_	0.349674	0.151623
hsa_circ_	0.19774	0.151625
hsa_circ_	0.322549	0.15165
hsa_circ_	0.858723	0.151664
hsa_circ_	-0.77397	0.151669
hsa_circ_	0.341431	0.151673
hsa_circ_	0.26156	0.151674
hsa_circ_	-0.67504	0.151674
hsa_circ_	-0.30151	0.151679
hsa_circ_	-0.24554	0.151681
hsa_circ_	1.56275	0.151688
hsa_circ_	0.247659	0.151691
hsa_circ_	0.422068	0.151701
hsa_circ_	-0.32292	0.151702
hsa_circ_	0.414858	0.151714
hsa_circ_	-0.2355	0.151738
hsa_circ_	0.545798	0.151762
hsa_circ_	0.24488	0.151765
hsa_circ_	0.267855	0.151766
hsa_circ_	0.312485	0.151767
hsa_circ_	-0.40809	0.151767
hsa_circ_	0.305435	0.151768
hsa_circ_	-1.48589	0.151771
hsa_circ_	-0.41172	0.151771
hsa_circ_	0.449982	0.151789
hsa_circ_	-0.37546	0.15179
hsa_circ_	0.393655	0.151804
hsa_circ_	0.667593	0.151805
hsa_circ_	0.464872	0.151809
hsa_circ_	-0.40442	0.151823
hsa_circ_	-0.22019	0.151834
hsa_circ_	0.236961	0.151842
hsa_circ_	-0.37884	0.15185
hsa_circ_	0.386576	0.151854
hsa_circ_	-0.42024	0.151862
hsa_circ_	-0.26023	0.151891
hsa_circ_	-0.46198	0.151895
hsa_circ_	0.359464	0.151909
hsa_circ_	1.64982	0.151913
hsa_circ_	-0.2998	0.151914

hsa_circ_	-0.54569	0.151935
hsa_circ_	0.260726	0.151938
hsa_circ_	0.347757	0.15194
hsa_circ_	-0.25625	0.151941
hsa_circ_	0.270354	0.151944
hsa_circ_	0.5838	0.151949
hsa_circ_	-0.26229	0.151959
hsa_circ_	-1.05491	0.152002
hsa_circ_	0.30672	0.152013
hsa_circ_	0.735334	0.152014
hsa_circ_	0.417682	0.152021
hsa_circ_	-0.33903	0.152026
hsa_circ_	-0.2588	0.152038
hsa_circ_	-0.3553	0.152047
hsa_circ_	0.436915	0.152053
hsa_circ_	0.22919	0.152062
hsa_circ_	0.866165	0.152068
hsa_circ_	-0.20726	0.152092
hsa_circ_	0.24526	0.152094
hsa_circ_	-0.64241	0.152101
hsa_circ_	0.262349	0.152103
hsa_circ_	0.226292	0.152111
hsa_circ_	-1.26066	0.152118
hsa_circ_	0.30775	0.152125
hsa_circ_	0.387535	0.152126
hsa_circ_	-0.47035	0.152131
hsa_circ_	-0.28283	0.152135
hsa_circ_	0.285301	0.152137
hsa_circ_	0.456178	0.152138
hsa_circ_	0.23906	0.152144
hsa_circ_	0.997499	0.152153
hsa_circ_	-0.23308	0.152168
hsa_circ_	-0.36676	0.152169
hsa_circ_	-0.23324	0.152187
hsa_circ_	-0.31947	0.152207
hsa_circ_	-0.35521	0.15221
hsa_circ_	0.203844	0.152227
hsa_circ_	0.268692	0.152237
hsa_circ_	0.367199	0.152255
hsa_circ_	0.483528	0.152263
hsa_circ_	0.322318	0.152277
hsa_circ_	-0.40628	0.152298
hsa_circ_	-0.53427	0.152313
hsa_circ_	-0.3183	0.152322
hsa_circ_	-0.87136	0.152333
hsa_circ_	0.684833	0.152335
hsa_circ_	0.359023	0.152339
hsa_circ_	0.966056	0.152343
hsa_circ_	0.39782	0.152355
hsa_circ_	-0.38193	0.152355
hsa_circ_	0.234922	0.152357
hsa_circ_	-0.28076	0.152364
hsa_circ_	-0.40769	0.152366
hsa_circ_	0.298961	0.152391

hsa_circ_	-0.23179	0.152398
hsa_circ_	0.25916	0.152399
hsa_circ_	-0.25986	0.152418
hsa_circ_	0.320781	0.152442
hsa_circ_	0.286568	0.152449
hsa_circ_	0.291158	0.152452
hsa_circ_	0.248601	0.152454
hsa_circ_	0.553498	0.152459
hsa_circ_	-0.29717	0.152492
hsa_circ_	0.67836	0.152511
hsa_circ_	-0.32625	0.152538
hsa_circ_	0.395722	0.152546
hsa_circ_	0.250489	0.15255
hsa_circ_	0.296466	0.152559
hsa_circ_	0.242913	0.152562
hsa_circ_	-0.33567	0.152575
hsa_circ_	0.281474	0.152576
hsa_circ_	0.212914	0.152577
hsa_circ_	0.237724	0.152629
hsa_circ_	-0.27613	0.152629
hsa_circ_	-0.38404	0.152633
hsa_circ_	-0.30542	0.152635
hsa_circ_	-0.45441	0.152644
hsa_circ_	0.303711	0.152677
hsa_circ_	-0.20244	0.152689
hsa_circ_	0.257159	0.152706
hsa_circ_	-0.3228	0.152719
hsa_circ_	-0.2284	0.152723
hsa_circ_	0.392504	0.152724
hsa_circ_	0.406404	0.152727
hsa_circ_	0.220531	0.152728
hsa_circ_	0.347803	0.152729
hsa_circ_	-0.44342	0.152742
hsa_circ_	-0.70952	0.152745
hsa_circ_	-0.35882	0.152748
hsa_circ_	-0.25377	0.152759
hsa_circ_	0.593692	0.152767
hsa_circ_	0.322144	0.152776
hsa_circ_	-0.50829	0.152787
hsa_circ_	0.283725	0.152794
hsa_circ_	0.32079	0.152814
hsa_circ_	-0.28805	0.152814
hsa_circ_	0.317645	0.152818
hsa_circ_	0.49622	0.152823
hsa_circ_	0.311247	0.152823
hsa_circ_	0.347571	0.15283
hsa_circ_	0.60549	0.152831
hsa_circ_	-0.30158	0.152846
hsa_circ_	0.606972	0.152853
hsa_circ_	0.293787	0.15286
hsa_circ_	0.216148	0.152876
hsa_circ_	-0.36083	0.152884
hsa_circ_	0.521843	0.152886
hsa_circ_	-0.24496	0.15289

hsa_circ_	0.272055	0.152911
hsa_circ_	-0.4031	0.152911
hsa_circ_	0.29611	0.152914
hsa_circ_	0.398544	0.152918
hsa_circ_	0.234473	0.152933
hsa_circ_	-0.23379	0.152942
hsa_circ_	0.306858	0.152943
hsa_circ_	0.279127	0.152957
hsa_circ_	0.401489	0.152967
hsa_circ_	-0.5786	0.152975
hsa_circ_	-0.49058	0.152977
hsa_circ_	0.261619	0.152996
hsa_circ_	0.335246	0.153015
hsa_circ_	-1.03294	0.153027
hsa_circ_	-0.4728	0.153039
hsa_circ_	-0.70652	0.15306
hsa_circ_	0.269306	0.153061
hsa_circ_	-0.21569	0.153071
hsa_circ_	0.233414	0.153074
hsa_circ_	0.309639	0.153079
hsa_circ_	0.351131	0.153105
hsa_circ_	-0.43128	0.153105
hsa_circ_	-0.27506	0.153107
hsa_circ_	-0.20604	0.153107
hsa_circ_	-0.2245	0.153111
hsa_circ_	0.378154	0.153112
hsa_circ_	0.284933	0.153119
hsa_circ_	-0.25321	0.153134
hsa_circ_	0.255806	0.15314
hsa_circ_	-0.34776	0.153162
hsa_circ_	-0.4357	0.153168
hsa_circ_	-0.1958	0.153178
hsa_circ_	0.37707	0.153181
hsa_circ_	-0.6918	0.153198
hsa_circ_	-0.298	0.153203
hsa_circ_	-0.30652	0.153204
hsa_circ_	-0.79671	0.153212
hsa_circ_	0.268873	0.153233
hsa_circ_	1.037435	0.153245
hsa_circ_	-0.22644	0.153246
hsa_circ_	0.480724	0.153251
hsa_circ_	-0.20597	0.153255
hsa_circ_	0.322769	0.15326
hsa_circ_	0.250171	0.153289
hsa_circ_	-0.19619	0.153292
hsa_circ_	-0.21356	0.153296
hsa_circ_	-0.26135	0.15332
hsa_circ_	0.30948	0.153323
hsa_circ_	-0.20983	0.153339
hsa_circ_	0.395	0.153355
hsa_circ_	0.263455	0.153365
hsa_circ_	-0.60513	0.153378
hsa_circ_	0.423098	0.153384
hsa_circ_	-0.409	0.153398

hsa_circ_	-0.24064	0.153398
hsa_circ_	0.267728	0.153401
hsa_circ_	-0.36756	0.153412
hsa_circ_	-0.23361	0.15342
hsa_circ_	0.268808	0.15342
hsa_circ_	-0.34713	0.153445
hsa_circ_	-0.27149	0.153446
hsa_circ_	-0.27973	0.153452
hsa_circ_	0.277813	0.15346
hsa_circ_	-0.322	0.153462
hsa_circ_	0.29204	0.153467
hsa_circ_	0.3446	0.153474
hsa_circ_	-0.87187	0.153489
hsa_circ_	0.247942	0.15349
hsa_circ_	0.208645	0.153501
hsa_circ_	0.398822	0.153508
hsa_circ_	-0.25704	0.153514
hsa_circ_	-0.26892	0.153534
hsa_circ_	-0.32606	0.153563
hsa_circ_	-0.25309	0.153564
hsa_circ_	-0.20585	0.153568
hsa_circ_	-0.76063	0.153575
hsa_circ_	0.51403	0.15359
hsa_circ_	0.304396	0.153605
hsa_circ_	-0.30111	0.153608
hsa_circ_	-0.79243	0.153609
hsa_circ_	-0.38092	0.153614
hsa_circ_	-0.44002	0.153617
hsa_circ_	0.251818	0.15363
hsa_circ_	-0.75898	0.153643
hsa_circ_	-0.23713	0.153643
hsa_circ_	0.544966	0.153646
hsa_circ_	0.243206	0.153664
hsa_circ_	-0.3452	0.153666
hsa_circ_	-0.23578	0.153668
hsa_circ_	0.583587	0.153668
hsa_circ_	0.654878	0.153678
hsa_circ_	-0.62489	0.153691
hsa_circ_	0.239844	0.153723
hsa_circ_	-0.51535	0.153726
hsa_circ_	-0.74288	0.153729
hsa_circ_	-0.21669	0.153736
hsa_circ_	1.22758	0.153746
hsa_circ_	0.439716	0.153753
hsa_circ_	0.333828	0.153755
hsa_circ_	-1.27202	0.153757
hsa_circ_	0.261709	0.153763
hsa_circ_	-0.21676	0.153788
hsa_circ_	0.316519	0.153792
hsa_circ_	0.697959	0.1538
hsa_circ_	0.277425	0.153803
hsa_circ_	0.251559	0.153805
hsa_circ_	0.266249	0.153825
hsa_circ_	0.527121	0.153828

hsa_circ_	-0.43042	0.153831
hsa_circ_	-0.19802	0.153843
hsa_circ_	-0.48847	0.153847
hsa_circ_	0.416916	0.153859
hsa_circ_	-0.62223	0.153864
hsa_circ_	-0.59909	0.153898
hsa_circ_	0.288083	0.1539
hsa_circ_	0.782745	0.153902
hsa_circ_	0.231144	0.153903
hsa_circ_	0.39603	0.153918
hsa_circ_	-0.62604	0.153926
hsa_circ_	-0.30019	0.153942
hsa_circ_	-0.95551	0.153947
hsa_circ_	-0.25587	0.153952
hsa_circ_	0.68561	0.153965
hsa_circ_	-0.24534	0.153975
hsa_circ_	0.322349	0.153991
hsa_circ_	0.656966	0.153999
hsa_circ_	-0.4889	0.154002
hsa_circ_	0.297218	0.154002
hsa_circ_	-0.25741	0.154004
hsa_circ_	0.256739	0.154011
hsa_circ_	-0.30319	0.154015
hsa_circ_	0.325712	0.154015
hsa_circ_	0.822617	0.15402
hsa_circ_	-0.60722	0.154021
hsa_circ_	0.234272	0.154023
hsa_circ_	-0.25272	0.154027
hsa_circ_	-0.36308	0.154035
hsa_circ_	0.216091	0.154041
hsa_circ_	-0.23437	0.154059
hsa_circ_	0.843028	0.154074
hsa_circ_	0.489955	0.154076
hsa_circ_	-0.53226	0.154095
hsa_circ_	0.21411	0.154105
hsa_circ_	-0.34013	0.154109
hsa_circ_	-0.56769	0.154111
hsa_circ_	0.262525	0.154133
hsa_circ_	0.347756	0.154133
hsa_circ_	0.240083	0.154137
hsa_circ_	-0.64863	0.154145
hsa_circ_	-0.20837	0.154145
hsa_circ_	0.342102	0.154149
hsa_circ_	-0.23326	0.15416
hsa_circ_	0.300509	0.154163
hsa_circ_	-0.23319	0.154164
hsa_circ_	-0.95598	0.154166
hsa_circ_	-0.61597	0.154167
hsa_circ_	0.426707	0.154169
hsa_circ_	-0.20992	0.154169
hsa_circ_	0.667461	0.154171
hsa_circ_	0.281425	0.154173
hsa_circ_	-0.24878	0.154185
hsa_circ_	0.249164	0.154188

hsa_circ_	-0.31797	0.154189
hsa_circ_	-1.16921	0.154195
hsa_circ_	0.24081	0.15421
hsa_circ_	-0.76965	0.154221
hsa_circ_	-0.63988	0.154223
hsa_circ_	0.260036	0.154229
hsa_circ_	-0.45248	0.154244
hsa_circ_	-0.57192	0.154257
hsa_circ_	-0.67556	0.154258
hsa_circ_	-0.48512	0.154267
hsa_circ_	0.968589	0.154277
hsa_circ_	0.772704	0.154294
hsa_circ_	0.323812	0.154302
hsa_circ_	-0.6814	0.154309
hsa_circ_	1.162481	0.15431
hsa_circ_	0.212323	0.154315
hsa_circ_	-0.26434	0.15432
hsa_circ_	-0.30391	0.154327
hsa_circ_	-0.58992	0.154333
hsa_circ_	-0.74131	0.154336
hsa_circ_	-0.25601	0.154348
hsa_circ_	0.294053	0.154352
hsa_circ_	-0.27763	0.15439
hsa_circ_	-0.21617	0.15439
hsa_circ_	-0.27944	0.154399
hsa_circ_	-0.57421	0.154415
hsa_circ_	-1.23906	0.154418
hsa_circ_	0.308161	0.154432
hsa_circ_	0.367947	0.154433
hsa_circ_	-0.50247	0.154438
hsa_circ_	-0.21497	0.154453
hsa_circ_	-0.21435	0.154467
hsa_circ_	0.257032	0.154475
hsa_circ_	0.272517	0.154479
hsa_circ_	-0.83268	0.154482
hsa_circ_	-0.37947	0.154508
hsa_circ_	-0.34265	0.154512
hsa_circ_	-0.33323	0.154519
hsa_circ_	-0.26841	0.154522
hsa_circ_	0.365176	0.154534
hsa_circ_	-0.23857	0.154547
hsa_circ_	0.290286	0.154551
hsa_circ_	-0.25633	0.154551
hsa_circ_	0.854439	0.154565
hsa_circ_	-0.38624	0.15457
hsa_circ_	-0.2599	0.154577
hsa_circ_	-0.38782	0.154583
hsa_circ_	0.229336	0.154601
hsa_circ_	0.419004	0.154614
hsa_circ_	0.304495	0.154625
hsa_circ_	0.666402	0.154628
hsa_circ_	0.326915	0.154636
hsa_circ_	-0.21947	0.154654
hsa_circ_	0.203903	0.154655

hsa_circ_	-1.55192	0.154658
hsa_circ_	0.322546	0.154662
hsa_circ_	-0.39289	0.154666
hsa_circ_	0.843852	0.154673
hsa_circ_	0.364409	0.154697
hsa_circ_	-0.66162	0.154705
hsa_circ_	0.239182	0.15471
hsa_circ_	-0.21339	0.154714
hsa_circ_	0.559079	0.154735
hsa_circ_	0.687107	0.15475
hsa_circ_	0.991659	0.154758
hsa_circ_	-0.31491	0.154781
hsa_circ_	-0.22936	0.154786
hsa_circ_	0.450904	0.154799
hsa_circ_	0.290783	0.154807
hsa_circ_	0.220201	0.154811
hsa_circ_	-0.32266	0.154825
hsa_circ_	-0.40266	0.154856
hsa_circ_	-0.46405	0.154859
hsa_circ_	0.561645	0.154861
hsa_circ_	0.509396	0.154866
hsa_circ_	-0.30221	0.154889
hsa_circ_	-0.31481	0.154901
hsa_circ_	0.264036	0.154907
hsa_circ_	-0.22823	0.154907
hsa_circ_	0.287185	0.154915
hsa_circ_	-0.3829	0.154924
hsa_circ_	-0.96201	0.154928
hsa_circ_	0.291183	0.154933
hsa_circ_	-0.79522	0.154934
hsa_circ_	-0.40233	0.154964
hsa_circ_	-0.21882	0.154976
hsa_circ_	0.216791	0.154982
hsa_circ_	0.311364	0.154992
hsa_circ_	0.475671	0.154996
hsa_circ_	0.306146	0.154997
hsa_circ_	0.247717	0.155001
hsa_circ_	-0.31258	0.155004
hsa_circ_	0.3145	0.15501
hsa_circ_	-0.35574	0.155022
hsa_circ_	-0.45324	0.155024
hsa_circ_	-0.40213	0.155053
hsa_circ_	-0.33114	0.155053
hsa_circ_	-0.34619	0.155055
hsa_circ_	0.585859	0.15506
hsa_circ_	0.232957	0.155061
hsa_circ_	0.390748	0.155061
hsa_circ_	-0.20095	0.15507
hsa_circ_	-0.30832	0.155074
hsa_circ_	0.621947	0.155096
hsa_circ_	1.00464	0.155105
hsa_circ_	-0.34324	0.155107
hsa_circ_	0.217542	0.15513
hsa_circ_	-0.25899	0.155131

hsa_circ_	0.359848	0.15514
hsa_circ_	-0.3568	0.155155
hsa_circ_	0.283899	0.155178
hsa_circ_	0.26784	0.155191
hsa_circ_	-0.36225	0.155217
hsa_circ_	0.530478	0.155241
hsa_circ_	0.394943	0.15527
hsa_circ_	-0.90304	0.155294
hsa_circ_	0.364434	0.155296
hsa_circ_	0.480709	0.155299
hsa_circ_	-0.20153	0.155312
hsa_circ_	0.363289	0.155317
hsa_circ_	-0.35269	0.155321
hsa_circ_	0.299249	0.15533
hsa_circ_	-0.21637	0.155338
hsa_circ_	0.870405	0.155339
hsa_circ_	0.405998	0.155349
hsa_circ_	0.25777	0.155352
hsa_circ_	-0.32379	0.155354
hsa_circ_	0.452246	0.155356
hsa_circ_	-0.41707	0.155357
hsa_circ_	-0.35578	0.155368
hsa_circ_	0.242268	0.155368
hsa_circ_	0.322139	0.155378
hsa_circ_	0.499295	0.155382
hsa_circ_	-0.3691	0.155392
hsa_circ_	0.703126	0.155399
hsa_circ_	-0.22393	0.155409
hsa_circ_	0.370604	0.155416
hsa_circ_	0.606017	0.15542
hsa_circ_	-0.2925	0.155433
hsa_circ_	-0.91037	0.155437
hsa_circ_	0.631018	0.155445
hsa_circ_	0.214694	0.155456
hsa_circ_	-0.90339	0.155456
hsa_circ_	-0.42094	0.155463
hsa_circ_	-0.28889	0.155464
hsa_circ_	-0.30147	0.155466
hsa_circ_	-0.41352	0.155471
hsa_circ_	-0.3988	0.155482
hsa_circ_	0.284843	0.155504
hsa_circ_	-0.28434	0.155526
hsa_circ_	0.300849	0.155527
hsa_circ_	0.53626	0.155534
hsa_circ_	-0.44438	0.155547
hsa_circ_	0.316833	0.155548
hsa_circ_	0.269672	0.15556
hsa_circ_	1.033713	0.155561
hsa_circ_	0.268402	0.155583
hsa_circ_	0.301353	0.155595
hsa_circ_	-0.47707	0.155596
hsa_circ_	-0.63691	0.155597
hsa_circ_	0.248497	0.1556
hsa_circ_	-0.24462	0.155604

hsa_circ_	-0.44908	0.15561
hsa_circ_	-0.39823	0.155617
hsa_circ_	0.42355	0.155624
hsa_circ_	0.21929	0.15564
hsa_circ_	0.262283	0.15564
hsa_circ_	-0.34285	0.155643
hsa_circ_	-0.882	0.155644
hsa_circ_	0.358273	0.155649
hsa_circ_	-0.29621	0.155651
hsa_circ_	0.358974	0.155653
hsa_circ_	-0.34372	0.155666
hsa_circ_	-0.24938	0.155671
hsa_circ_	-0.25895	0.15568
hsa_circ_	0.418424	0.155685
hsa_circ_	0.514686	0.155695
hsa_circ_	0.311887	0.155697
hsa_circ_	-0.23839	0.155699
hsa_circ_	0.241911	0.155709
hsa_circ_	-0.26388	0.155721
hsa_circ_	-0.37259	0.155723
hsa_circ_	-0.19866	0.155749
hsa_circ_	-0.3813	0.155754
hsa_circ_	-0.40067	0.155756
hsa_circ_	-0.61976	0.155763
hsa_circ_	-0.42586	0.155773
hsa_circ_	0.624328	0.155779
hsa_circ_	-0.21076	0.155815
hsa_circ_	0.326837	0.155821
hsa_circ_	-0.20496	0.155829
hsa_circ_	0.202971	0.155831
hsa_circ_	-0.21772	0.155831
hsa_circ_	0.256472	0.155864
hsa_circ_	0.578944	0.155864
hsa_circ_	0.475769	0.15587
hsa_circ_	-0.81254	0.155874
hsa_circ_	0.40574	0.155877
hsa_circ_	0.764271	0.155878
hsa_circ_	0.394323	0.155883
hsa_circ_	-1.016	0.155888
hsa_circ_	-0.34817	0.155929
hsa_circ_	-0.27385	0.155939
hsa_circ_	-0.29334	0.155958
hsa_circ_	1.034818	0.155961
hsa_circ_	-0.30999	0.155966
hsa_circ_	-0.23116	0.155967
hsa_circ_	0.476114	0.15597
hsa_circ_	0.896679	0.155979
hsa_circ_	0.509794	0.15598
hsa_circ_	0.303379	0.155987
hsa_circ_	0.396346	0.155987
hsa_circ_	-0.26625	0.155992
hsa_circ_	-0.40439	0.156008
hsa_circ_	0.315217	0.156012
hsa_circ_	-0.215	0.156023

hsa_circ_	-0.27388	0.15604
hsa_circ_	0.214513	0.15604
hsa_circ_	0.362302	0.156046
hsa_circ_	-0.29884	0.156072
hsa_circ_	0.332094	0.156075
hsa_circ_	0.439366	0.156078
hsa_circ_	0.234905	0.15609
hsa_circ_	-0.33454	0.156099
hsa_circ_	-0.55384	0.156109
hsa_circ_	-0.96067	0.15612
hsa_circ_	-0.20024	0.156126
hsa_circ_	0.359913	0.15613
hsa_circ_	-0.2152	0.156137
hsa_circ_	0.219865	0.15614
hsa_circ_	0.875556	0.156144
hsa_circ_	-0.39635	0.156146
hsa_circ_	-0.27743	0.156149
hsa_circ_	0.726094	0.156201
hsa_circ_	0.402398	0.156203
hsa_circ_	0.28405	0.156205
hsa_circ_	-0.22211	0.156207
hsa_circ_	-0.2046	0.156207
hsa_circ_	0.203733	0.156208
hsa_circ_	-0.24046	0.156241
hsa_circ_	0.389497	0.156242
hsa_circ_	-0.62921	0.156244
hsa_circ_	-0.25072	0.156255
hsa_circ_	0.562841	0.156263
hsa_circ_	0.500001	0.156269
hsa_circ_	0.326601	0.156276
hsa_circ_	0.359704	0.156284
hsa_circ_	-0.41999	0.156286
hsa_circ_	0.388116	0.156297
hsa_circ_	0.607628	0.156312
hsa_circ_	-0.68189	0.156326
hsa_circ_	0.452154	0.156331
hsa_circ_	-0.45844	0.156336
hsa_circ_	-0.29649	0.156343
hsa_circ_	0.204768	0.15638
hsa_circ_	-0.62773	0.156386
hsa_circ_	0.242404	0.156389
hsa_circ_	0.264636	0.156403
hsa_circ_	0.202819	0.156406
hsa_circ_	0.222833	0.156409
hsa_circ_	0.37115	0.156415
hsa_circ_	-0.42773	0.156415
hsa_circ_	-0.22495	0.15643
hsa_circ_	0.362868	0.156442
hsa_circ_	-0.88191	0.156465
hsa_circ_	0.31113	0.156473
hsa_circ_	0.264443	0.156486
hsa_circ_	0.269972	0.156491
hsa_circ_	0.543497	0.156509
hsa_circ_	0.411607	0.156516

hsa_circ_	0.370953	0.156531
hsa_circ_	0.260354	0.156533
hsa_circ_	-0.35956	0.156537
hsa_circ_	-0.31121	0.156537
hsa_circ_	0.34365	0.156553
hsa_circ_	0.282666	0.156576
hsa_circ_	0.642137	0.156586
hsa_circ_	0.243898	0.156597
hsa_circ_	-0.33081	0.156603
hsa_circ_	-0.28497	0.156607
hsa_circ_	0.306099	0.156614
hsa_circ_	-0.2232	0.156616
hsa_circ_	-0.39449	0.156627
hsa_circ_	0.257017	0.156627
hsa_circ_	-0.58754	0.156646
hsa_circ_	0.22624	0.156656
hsa_circ_	-0.28507	0.156661
hsa_circ_	-0.37717	0.156674
hsa_circ_	0.71333	0.156682
hsa_circ_	0.382264	0.156691
hsa_circ_	-0.23227	0.156692
hsa_circ_	-0.2282	0.156694
hsa_circ_	-0.35889	0.156694
hsa_circ_	-0.20728	0.156709
hsa_circ_	0.742378	0.15672
hsa_circ_	-0.20847	0.15672
hsa_circ_	0.303558	0.156722
hsa_circ_	-0.22119	0.156731
hsa_circ_	-0.2372	0.156734
hsa_circ_	0.982537	0.15675
hsa_circ_	-0.46775	0.15678
hsa_circ_	0.479931	0.156781
hsa_circ_	-1.12583	0.156788
hsa_circ_	-0.27509	0.15679
hsa_circ_	-0.2325	0.156793
hsa_circ_	-0.23671	0.156803
hsa_circ_	-0.31778	0.156807
hsa_circ_	0.43502	0.156815
hsa_circ_	0.26466	0.15684
hsa_circ_	0.389743	0.15684
hsa_circ_	-0.27908	0.156849
hsa_circ_	-0.22712	0.156853
hsa_circ_	0.247002	0.156858
hsa_circ_	-0.2924	0.156873
hsa_circ_	0.261369	0.156873
hsa_circ_	0.30884	0.15689
hsa_circ_	-0.23588	0.156898
hsa_circ_	0.452942	0.156898
hsa_circ_	-0.71806	0.1569
hsa_circ_	0.259181	0.15692
hsa_circ_	-0.19831	0.15693
hsa_circ_	0.857531	0.156931
hsa_circ_	-0.58556	0.15694
hsa_circ_	-0.2511	0.156946

hsa_circ_	0.673506	0.156952
hsa_circ_	0.307797	0.156956
hsa_circ_	-0.40562	0.156956
hsa_circ_	0.345054	0.156981
hsa_circ_	0.270474	0.157
hsa_circ_	-0.49427	0.157019
hsa_circ_	0.216231	0.157025
hsa_circ_	-0.31959	0.157034
hsa_circ_	0.267449	0.157049
hsa_circ_	0.254516	0.15706
hsa_circ_	-0.24039	0.157067
hsa_circ_	-0.50464	0.157077
hsa_circ_	-0.33428	0.157079
hsa_circ_	-0.23649	0.15708
hsa_circ_	-0.30207	0.157092
hsa_circ_	0.354022	0.157106
hsa_circ_	0.201716	0.157134
hsa_circ_	-0.25234	0.15714
hsa_circ_	-0.24334	0.157143
hsa_circ_	-0.63408	0.157143
hsa_circ_	0.512939	0.157144
hsa_circ_	-0.56554	0.157158
hsa_circ_	-0.29124	0.157159
hsa_circ_	-0.28961	0.157159
hsa_circ_	-0.21942	0.157169
hsa_circ_	-2.01645	0.157181
hsa_circ_	0.645319	0.157202
hsa_circ_	-0.27264	0.157212
hsa_circ_	0.204147	0.157236
hsa_circ_	-0.38344	0.157239
hsa_circ_	-0.26088	0.157268
hsa_circ_	0.340891	0.157278
hsa_circ_	0.362372	0.157298
hsa_circ_	0.222231	0.157309
hsa_circ_	0.703774	0.15731
hsa_circ_	0.358031	0.15732
hsa_circ_	-0.24133	0.15732
hsa_circ_	0.368665	0.157329
hsa_circ_	-0.33473	0.157333
hsa_circ_	0.334466	0.157348
hsa_circ_	-0.27128	0.157356
hsa_circ_	0.349438	0.15736
hsa_circ_	1.040236	0.157395
hsa_circ_	0.726036	0.157408
hsa_circ_	-0.48412	0.157409
hsa_circ_	0.47529	0.157411
hsa_circ_	-0.24361	0.157416
hsa_circ_	-0.27722	0.157421
hsa_circ_	-0.20942	0.157425
hsa_circ_	-0.25692	0.157437
hsa_circ_	-0.53778	0.157441
hsa_circ_	-0.22629	0.157446
hsa_circ_	0.247589	0.157451
hsa_circ_	-0.34461	0.157459

hsa_circ_	0.526606	0.157465
hsa_circ_	-0.36114	0.157468
hsa_circ_	0.802946	0.157503
hsa_circ_	1.025949	0.157507
hsa_circ_	0.278386	0.157512
hsa_circ_	0.415401	0.157514
hsa_circ_	-0.22174	0.157519
hsa_circ_	0.32163	0.157521
hsa_circ_	0.336533	0.157532
hsa_circ_	0.337373	0.157532
hsa_circ_	-0.56454	0.157561
hsa_circ_	0.255312	0.157564
hsa_circ_	-0.74886	0.157584
hsa_circ_	0.431176	0.157586
hsa_circ_	0.347919	0.157586
hsa_circ_	-0.47526	0.157597
hsa_circ_	0.211963	0.157597
hsa_circ_	0.303276	0.157614
hsa_circ_	-0.21844	0.157617
hsa_circ_	-0.3593	0.157628
hsa_circ_	-0.27249	0.157644
hsa_circ_	-0.25855	0.157653
hsa_circ_	0.231542	0.157654
hsa_circ_	-1.02619	0.157664
hsa_circ_	0.261209	0.157675
hsa_circ_	0.293755	0.157676
hsa_circ_	0.485574	0.157679
hsa_circ_	-0.95042	0.157693
hsa_circ_	-0.65269	0.157699
hsa_circ_	-0.48055	0.157701
hsa_circ_	0.397916	0.15773
hsa_circ_	0.483318	0.157735
hsa_circ_	-0.25751	0.157775
hsa_circ_	0.207176	0.157787
hsa_circ_	-0.27328	0.157815
hsa_circ_	-0.43489	0.157831
hsa_circ_	-0.61777	0.157837
hsa_circ_	0.45416	0.157838
hsa_circ_	-0.65024	0.15784
hsa_circ_	0.355939	0.157841
hsa_circ_	0.331174	0.157848
hsa_circ_	0.292237	0.15785
hsa_circ_	0.239518	0.15786
hsa_circ_	0.343192	0.157865
hsa_circ_	-0.24867	0.157869
hsa_circ_	0.202498	0.157876
hsa_circ_	0.812584	0.157877
hsa_circ_	-0.41967	0.157882
hsa_circ_	0.228459	0.157897
hsa_circ_	-0.2657	0.157903
hsa_circ_	-0.29258	0.15791
hsa_circ_	0.473072	0.157926
hsa_circ_	0.313094	0.15793
hsa_circ_	-0.3368	0.157931

hsa_circ_	-0.31306	0.157937
hsa_circ_	-0.27236	0.157943
hsa_circ_	-0.62946	0.157944
hsa_circ_	-0.27633	0.157959
hsa_circ_	-0.55457	0.15799
hsa_circ_	-0.25481	0.157994
hsa_circ_	-0.21432	0.157995
hsa_circ_	-0.22967	0.157998
hsa_circ_	0.405606	0.158002
hsa_circ_	-0.47833	0.158008
hsa_circ_	-0.22011	0.158023
hsa_circ_	-0.23165	0.158029
hsa_circ_	0.794958	0.158029
hsa_circ_	-0.32221	0.158035
hsa_circ_	-0.24785	0.15807
hsa_circ_	0.379623	0.158072
hsa_circ_	0.198418	0.158073
hsa_circ_	0.57982	0.158077
hsa_circ_	-1.28856	0.158102
hsa_circ_	0.691115	0.158109
hsa_circ_	0.364017	0.158118
hsa_circ_	0.401411	0.158121
hsa_circ_	-0.25282	0.158127
hsa_circ_	-0.34372	0.158133
hsa_circ_	-0.36102	0.158144
hsa_circ_	-0.24851	0.158148
hsa_circ_	-0.82378	0.15816
hsa_circ_	-0.78473	0.158163
hsa_circ_	-0.2205	0.158167
hsa_circ_	0.390548	0.158187
hsa_circ_	-0.63531	0.158191
hsa_circ_	0.45985	0.158201
hsa_circ_	0.289731	0.158207
hsa_circ_	-0.27363	0.158208
hsa_circ_	0.547019	0.158218
hsa_circ_	-0.47951	0.158218
hsa_circ_	0.240356	0.15822
hsa_circ_	0.617455	0.158222
hsa_circ_	-0.22158	0.158235
hsa_circ_	0.729453	0.158239
hsa_circ_	-0.93597	0.158243
hsa_circ_	0.210413	0.158259
hsa_circ_	0.288524	0.158262
hsa_circ_	-0.36145	0.158267
hsa_circ_	0.374735	0.158286
hsa_circ_	-0.53109	0.15829
hsa_circ_	0.596355	0.158302
hsa_circ_	-0.55933	0.158315
hsa_circ_	-0.19724	0.158315
hsa_circ_	0.711382	0.158326
hsa_circ_	0.330852	0.15833
hsa_circ_	0.328052	0.158335
hsa_circ_	-0.23373	0.158352
hsa_circ_	-0.55777	0.158357

hsa_circ_	-0.35236	0.158367
hsa_circ_	-0.3264	0.158391
hsa_circ_	-0.23011	0.1584
hsa_circ_	-0.69378	0.158413
hsa_circ_	0.390083	0.158424
hsa_circ_	-0.2392	0.158425
hsa_circ_	-0.26293	0.158492
hsa_circ_	0.413395	0.158493
hsa_circ_	0.238972	0.158512
hsa_circ_	-0.35804	0.158517
hsa_circ_	0.760397	0.158525
hsa_circ_	-0.34336	0.158534
hsa_circ_	-0.67852	0.158547
hsa_circ_	0.226074	0.158559
hsa_circ_	0.973781	0.158559
hsa_circ_	-0.28987	0.15856
hsa_circ_	2.171018	0.158566
hsa_circ_	-0.25527	0.158578
hsa_circ_	-0.33213	0.158593
hsa_circ_	0.278852	0.158601
hsa_circ_	0.356053	0.158614
hsa_circ_	0.25027	0.158626
hsa_circ_	0.24857	0.158629
hsa_circ_	-0.23739	0.158638
hsa_circ_	-0.93841	0.158639
hsa_circ_	0.307962	0.158651
hsa_circ_	-0.54428	0.158656
hsa_circ_	0.555623	0.158659
hsa_circ_	0.398635	0.158676
hsa_circ_	-0.26146	0.158679
hsa_circ_	-0.29063	0.158692
hsa_circ_	-0.45479	0.158692
hsa_circ_	0.313694	0.158698
hsa_circ_	-0.19948	0.158704
hsa_circ_	0.484201	0.158707
hsa_circ_	0.244024	0.15871
hsa_circ_	0.459138	0.158724
hsa_circ_	-0.36124	0.158725
hsa_circ_	0.438144	0.158737
hsa_circ_	-0.79029	0.158752
hsa_circ_	-0.97059	0.158756
hsa_circ_	0.506863	0.158768
hsa_circ_	-0.22492	0.15877
hsa_circ_	0.435287	0.158773
hsa_circ_	0.366521	0.158792
hsa_circ_	-0.34959	0.1588
hsa_circ_	0.336847	0.158807
hsa_circ_	0.199302	0.158825
hsa_circ_	-0.30523	0.158829
hsa_circ_	-0.31723	0.158848
hsa_circ_	0.276144	0.158852
hsa_circ_	-0.62789	0.158862
hsa_circ_	0.381833	0.158862
hsa_circ_	-0.2376	0.158876

hsa_circ_	0.238273	0.158882
hsa_circ_	0.557639	0.15889
hsa_circ_	-0.52217	0.158906
hsa_circ_	0.760049	0.158915
hsa_circ_	-0.20039	0.158936
hsa_circ_	0.357759	0.158937
hsa_circ_	0.532486	0.158957
hsa_circ_	-0.38067	0.158962
hsa_circ_	-0.21883	0.15897
hsa_circ_	0.357416	0.158976
hsa_circ_	0.353843	0.158977
hsa_circ_	0.300183	0.158978
hsa_circ_	0.49522	0.158988
hsa_circ_	-0.30869	0.158993
hsa_circ_	-0.4057	0.159011
hsa_circ_	0.479184	0.159013
hsa_circ_	-0.88015	0.159033
hsa_circ_	-0.24152	0.159041
hsa_circ_	-0.46855	0.159046
hsa_circ_	-0.57622	0.159056
hsa_circ_	0.292977	0.159093
hsa_circ_	0.33689	0.1591
hsa_circ_	0.37316	0.159103
hsa_circ_	0.675933	0.159117
hsa_circ_	-0.29169	0.159117
hsa_circ_	-0.50949	0.15912
hsa_circ_	0.230883	0.159123
hsa_circ_	-0.30245	0.159136
hsa_circ_	0.341306	0.159147
hsa_circ_	-0.22428	0.15915
hsa_circ_	-0.71528	0.159152
hsa_circ_	-0.36411	0.159155
hsa_circ_	-0.3931	0.159159
hsa_circ_	-0.23268	0.159161
hsa_circ_	-0.53889	0.15917
hsa_circ_	-0.25178	0.159174
hsa_circ_	0.315295	0.159183
hsa_circ_	-0.2501	0.159208
hsa_circ_	-0.32728	0.159238
hsa_circ_	-0.27946	0.15925
hsa_circ_	-1.09711	0.15925
hsa_circ_	-0.33328	0.159253
hsa_circ_	0.384316	0.159254
hsa_circ_	-0.26131	0.159266
hsa_circ_	0.288167	0.159283
hsa_circ_	0.311229	0.159283
hsa_circ_	0.553338	0.159301
hsa_circ_	0.794016	0.159305
hsa_circ_	-0.48755	0.159311
hsa_circ_	-0.2731	0.159315
hsa_circ_	0.282828	0.159323
hsa_circ_	-0.23505	0.159331
hsa_circ_	-0.29695	0.159338
hsa_circ_	-0.44296	0.159343

hsa_circ_	0.25759	0.159353
hsa_circ_	0.416904	0.159354
hsa_circ_	0.237054	0.159355
hsa_circ_	-0.63534	0.159355
hsa_circ_	0.413409	0.159362
hsa_circ_	0.574958	0.159368
hsa_circ_	-0.24411	0.159369
hsa_circ_	-0.28734	0.159372
hsa_circ_	-0.28442	0.159376
hsa_circ_	0.221939	0.159376
hsa_circ_	0.257736	0.159382
hsa_circ_	0.818148	0.159386
hsa_circ_	0.343201	0.159397
hsa_circ_	0.26964	0.159399
hsa_circ_	0.314981	0.159406
hsa_circ_	-1.11408	0.159408
hsa_circ_	0.245544	0.159413
hsa_circ_	-0.22948	0.159428
hsa_circ_	-0.32545	0.159448
hsa_circ_	-0.58819	0.15945
hsa_circ_	-0.30387	0.159452
hsa_circ_	0.246784	0.15946
hsa_circ_	-0.72272	0.159463
hsa_circ_	-0.23806	0.159465
hsa_circ_	0.304006	0.159467
hsa_circ_	-0.35593	0.159476
hsa_circ_	0.322103	0.159497
hsa_circ_	0.404286	0.1595
hsa_circ_	0.337415	0.159517
hsa_circ_	0.645414	0.159518
hsa_circ_	1.075588	0.159526
hsa_circ_	0.219245	0.159533
hsa_circ_	-0.2452	0.159541
hsa_circ_	-0.24666	0.159545
hsa_circ_	0.304174	0.159553
hsa_circ_	-0.37289	0.159553
hsa_circ_	-0.78592	0.159553
hsa_circ_	0.254783	0.159553
hsa_circ_	-0.22825	0.159572
hsa_circ_	-0.22246	0.159576
hsa_circ_	-0.25639	0.159591
hsa_circ_	0.40713	0.159598
hsa_circ_	0.263194	0.15961
hsa_circ_	0.209343	0.15962
hsa_circ_	0.340665	0.15962
hsa_circ_	0.65625	0.159623
hsa_circ_	-0.25659	0.159627
hsa_circ_	-0.22884	0.159639
hsa_circ_	0.404533	0.159646
hsa_circ_	0.238334	0.159652
hsa_circ_	-0.36518	0.159661
hsa_circ_	0.366524	0.159674
hsa_circ_	-0.4452	0.159691
hsa_circ_	1.228261	0.159692

hsa_circ_	-0.6298	0.159695
hsa_circ_	0.253836	0.159703
hsa_circ_	-0.58518	0.159707
hsa_circ_	-0.25723	0.159711
hsa_circ_	-0.37273	0.159711
hsa_circ_	0.249712	0.159724
hsa_circ_	-0.27702	0.159734
hsa_circ_	0.375853	0.15975
hsa_circ_	-0.53895	0.159762
hsa_circ_	-0.27615	0.159776
hsa_circ_	-0.76723	0.159777
hsa_circ_	0.354809	0.159777
hsa_circ_	-0.33394	0.159803
hsa_circ_	-0.21951	0.159803
hsa_circ_	0.375124	0.159811
hsa_circ_	-0.72821	0.159818
hsa_circ_	-0.24717	0.159828
hsa_circ_	0.261595	0.159844
hsa_circ_	0.290865	0.15985
hsa_circ_	-0.59055	0.159857
hsa_circ_	-0.30401	0.159864
hsa_circ_	0.386723	0.15988
hsa_circ_	-0.49225	0.159883
hsa_circ_	-0.22679	0.159887
hsa_circ_	0.646568	0.15991
hsa_circ_	-0.33731	0.159912
hsa_circ_	0.787084	0.159916
hsa_circ_	-0.3414	0.159921
hsa_circ_	-0.24111	0.159934
hsa_circ_	-0.22077	0.159952
hsa_circ_	-0.8854	0.159956
hsa_circ_	-0.6574	0.159957
hsa_circ_	-0.39848	0.159966
hsa_circ_	-0.2546	0.159967
hsa_circ_	-0.29043	0.159973
hsa_circ_	0.279596	0.159973
hsa_circ_	-0.28057	0.159999
hsa_circ_	0.262179	0.160024
hsa_circ_	0.929311	0.160037
hsa_circ_	-0.36738	0.160042
hsa_circ_	0.674004	0.160046
hsa_circ_	0.509074	0.160062
hsa_circ_	-0.268	0.16007
hsa_circ_	-0.3562	0.160072
hsa_circ_	0.487301	0.160088
hsa_circ_	0.28164	0.160104
hsa_circ_	-0.33863	0.160107
hsa_circ_	-1.07229	0.160115
hsa_circ_	0.235127	0.160117
hsa_circ_	-0.78683	0.160124
hsa_circ_	-0.7454	0.160126
hsa_circ_	-1.45082	0.160127
hsa_circ_	0.401334	0.160148
hsa_circ_	0.824994	0.160159

hsa_circ_	0.473491	0.160171
hsa_circ_	0.331687	0.160181
hsa_circ_	-0.52227	0.160188
hsa_circ_	0.251373	0.160196
hsa_circ_	0.320949	0.160197
hsa_circ_	0.245636	0.160217
hsa_circ_	0.337433	0.16022
hsa_circ_	0.328051	0.160221
hsa_circ_	-0.24463	0.160225
hsa_circ_	-0.36599	0.160231
hsa_circ_	0.728874	0.16024
hsa_circ_	0.409414	0.160249
hsa_circ_	0.381631	0.160263
hsa_circ_	0.192781	0.160277
hsa_circ_	-0.2006	0.16028
hsa_circ_	0.617004	0.160282
hsa_circ_	-0.36602	0.160283
hsa_circ_	-0.37511	0.160285
hsa_circ_	0.46051	0.160287
hsa_circ_	-0.33569	0.160315
hsa_circ_	-0.68793	0.160318
hsa_circ_	-0.51416	0.160322
hsa_circ_	0.242831	0.160369
hsa_circ_	-0.28662	0.160373
hsa_circ_	-0.23395	0.160396
hsa_circ_	-0.2797	0.160405
hsa_circ_	0.949794	0.160406
hsa_circ_	0.353191	0.160411
hsa_circ_	-0.29081	0.160417
hsa_circ_	-0.34596	0.16042
hsa_circ_	-0.2142	0.160423
hsa_circ_	0.260649	0.160425
hsa_circ_	-0.24936	0.160436
hsa_circ_	-0.32664	0.160467
hsa_circ_	-0.91944	0.160487
hsa_circ_	-0.48881	0.160514
hsa_circ_	0.406249	0.160531
hsa_circ_	0.467747	0.160544
hsa_circ_	-0.21181	0.160545
hsa_circ_	-0.23343	0.160551
hsa_circ_	0.256973	0.160556
hsa_circ_	-0.25706	0.160564
hsa_circ_	0.35016	0.160565
hsa_circ_	0.219248	0.160577
hsa_circ_	-0.42335	0.160578
hsa_circ_	0.294653	0.160579
hsa_circ_	0.238669	0.160589
hsa_circ_	-0.69153	0.160616
hsa_circ_	-0.56128	0.160619
hsa_circ_	0.335522	0.16062
hsa_circ_	-0.30495	0.160632
hsa_circ_	1.311009	0.160675
hsa_circ_	-0.2169	0.160676
hsa_circ_	-0.22193	0.160684

hsa_circ_	-0.27695	0.160691
hsa_circ_	-0.71342	0.160705
hsa_circ_	0.74918	0.160714
hsa_circ_	1.031711	0.160715
hsa_circ_	0.879527	0.16072
hsa_circ_	-0.40068	0.160721
hsa_circ_	-0.24403	0.160739
hsa_circ_	0.366053	0.160739
hsa_circ_	-0.22634	0.160748
hsa_circ_	0.296406	0.160751
hsa_circ_	-0.31931	0.16076
hsa_circ_	-0.27428	0.16076
hsa_circ_	-0.67391	0.160761
hsa_circ_	-0.20973	0.160797
hsa_circ_	0.262782	0.160805
hsa_circ_	-0.21997	0.160808
hsa_circ_	0.277878	0.160811
hsa_circ_	0.466507	0.160814
hsa_circ_	0.337666	0.160824
hsa_circ_	0.218257	0.160825
hsa_circ_	-0.28611	0.160841
hsa_circ_	-0.23269	0.160847
hsa_circ_	-0.64065	0.160857
hsa_circ_	-0.33321	0.160859
hsa_circ_	0.467632	0.160875
hsa_circ_	-0.30235	0.160878
hsa_circ_	0.224652	0.160883
hsa_circ_	0.324081	0.160909
hsa_circ_	-0.54487	0.160911
hsa_circ_	-0.48269	0.160914
hsa_circ_	0.303541	0.160921
hsa_circ_	0.64158	0.160926
hsa_circ_	0.387338	0.160933
hsa_circ_	0.218297	0.160935
hsa_circ_	0.374869	0.160939
hsa_circ_	-0.61538	0.160949
hsa_circ_	0.786544	0.160951
hsa_circ_	0.204738	0.160975
hsa_circ_	-1.25519	0.160991
hsa_circ_	0.250943	0.161008
hsa_circ_	-0.59173	0.161022
hsa_circ_	-0.32044	0.161026
hsa_circ_	0.297419	0.161027
hsa_circ_	-0.24354	0.161034
hsa_circ_	0.420302	0.161034
hsa_circ_	-0.22956	0.161043
hsa_circ_	0.214293	0.161054
hsa_circ_	0.307211	0.161072
hsa_circ_	0.253552	0.161081
hsa_circ_	-0.33717	0.161083
hsa_circ_	-0.48325	0.161088
hsa_circ_	0.473989	0.161089
hsa_circ_	0.257761	0.161095
hsa_circ_	-0.25928	0.161097

hsa_circ_	-0.23822	0.161098
hsa_circ_	0.443086	0.161108
hsa_circ_	-0.22179	0.161111
hsa_circ_	-0.28696	0.161129
hsa_circ_	0.245219	0.161136
hsa_circ_	-0.23056	0.161143
hsa_circ_	0.511814	0.161148
hsa_circ_	0.395542	0.161165
hsa_circ_	-0.52637	0.16117
hsa_circ_	-0.26223	0.161216
hsa_circ_	0.525858	0.161236
hsa_circ_	0.329064	0.161242
hsa_circ_	-0.38497	0.161247
hsa_circ_	0.207901	0.161253
hsa_circ_	0.548754	0.161273
hsa_circ_	-0.42331	0.161283
hsa_circ_	0.555329	0.1613
hsa_circ_	0.4229	0.161304
hsa_circ_	-0.23688	0.161305
hsa_circ_	-0.38404	0.161306
hsa_circ_	0.341703	0.16131
hsa_circ_	0.329351	0.161325
hsa_circ_	-0.70055	0.161334
hsa_circ_	0.374711	0.161339
hsa_circ_	0.310495	0.161365
hsa_circ_	-0.4288	0.161377
hsa_circ_	-0.53539	0.161401
hsa_circ_	0.421057	0.161401
hsa_circ_	0.390277	0.161408
hsa_circ_	-0.31712	0.161412
hsa_circ_	0.63596	0.161433
hsa_circ_	0.244529	0.16144
hsa_circ_	-0.43646	0.161474
hsa_circ_	0.632096	0.161475
hsa_circ_	-0.85271	0.161477
hsa_circ_	0.234644	0.161493
hsa_circ_	0.361585	0.161512
hsa_circ_	0.439967	0.161533
hsa_circ_	-0.2306	0.161533
hsa_circ_	0.545211	0.161537
hsa_circ_	-0.24465	0.161543
hsa_circ_	0.387241	0.161558
hsa_circ_	-0.27468	0.161566
hsa_circ_	0.769209	0.161567
hsa_circ_	0.634913	0.161569
hsa_circ_	0.417356	0.161577
hsa_circ_	-0.23662	0.161589
hsa_circ_	0.3341	0.161594
hsa_circ_	0.263329	0.161608
hsa_circ_	-0.33655	0.161611
hsa_circ_	0.50843	0.16162
hsa_circ_	0.371243	0.161634
hsa_circ_	0.267424	0.161634
hsa_circ_	0.27622	0.161641

hsa_circ_	-0.23694	0.161657
hsa_circ_	0.35861	0.161659
hsa_circ_	0.918309	0.161659
hsa_circ_	-0.33376	0.16167
hsa_circ_	-0.27208	0.161672
hsa_circ_	-0.23567	0.161678
hsa_circ_	0.339132	0.16168
hsa_circ_	0.342573	0.161686
hsa_circ_	-0.3578	0.161725
hsa_circ_	-0.2759	0.161736
hsa_circ_	0.277864	0.161743
hsa_circ_	-0.2701	0.161747
hsa_circ_	-0.61689	0.161761
hsa_circ_	0.276323	0.161764
hsa_circ_	0.229821	0.161767
hsa_circ_	0.24477	0.161772
hsa_circ_	0.214125	0.161782
hsa_circ_	0.298738	0.161817
hsa_circ_	0.395042	0.161836
hsa_circ_	0.438598	0.161857
hsa_circ_	-0.32614	0.161868
hsa_circ_	0.416329	0.161882
hsa_circ_	-0.35396	0.1619
hsa_circ_	-0.36881	0.161904
hsa_circ_	0.319476	0.161915
hsa_circ_	0.429184	0.161915
hsa_circ_	-0.23933	0.16192
hsa_circ_	-0.77742	0.161921
hsa_circ_	-0.33933	0.161923
hsa_circ_	0.335858	0.161936
hsa_circ_	-0.21645	0.161956
hsa_circ_	-0.22493	0.16196
hsa_circ_	-0.27208	0.161966
hsa_circ_	0.582185	0.16197
hsa_circ_	0.359376	0.161974
hsa_circ_	-0.32169	0.161977
hsa_circ_	0.426715	0.161986
hsa_circ_	0.225384	0.161992
hsa_circ_	0.708441	0.162001
hsa_circ_	-0.40619	0.162002
hsa_circ_	-0.25307	0.162009
hsa_circ_	-0.31414	0.16201
hsa_circ_	-0.28504	0.16202
hsa_circ_	0.443003	0.16203
hsa_circ_	0.547111	0.162048
hsa_circ_	1.028958	0.162071
hsa_circ_	-0.58494	0.162079
hsa_circ_	-0.22126	0.16208
hsa_circ_	-0.30452	0.162093
hsa_circ_	-1.63015	0.162093
hsa_circ_	-0.45895	0.162105
hsa_circ_	-0.8522	0.162109
hsa_circ_	0.8178	0.162125
hsa_circ_	-0.23369	0.162127

hsa_circ_	-0.23798	0.162148
hsa_circ_	-0.43573	0.162162
hsa_circ_	0.825987	0.162165
hsa_circ_	-0.32127	0.162181
hsa_circ_	-0.80737	0.162193
hsa_circ_	-0.34201	0.162205
hsa_circ_	0.412289	0.162207
hsa_circ_	0.273793	0.162214
hsa_circ_	-0.43146	0.162214
hsa_circ_	1.262283	0.162221
hsa_circ_	-0.36782	0.162224
hsa_circ_	-0.37089	0.162232
hsa_circ_	-0.33356	0.162234
hsa_circ_	-0.67436	0.162236
hsa_circ_	-0.28043	0.162236
hsa_circ_	-0.38573	0.162244
hsa_circ_	0.375673	0.162248
hsa_circ_	-0.34021	0.162258
hsa_circ_	0.340779	0.162259
hsa_circ_	-0.24309	0.162274
hsa_circ_	-0.31205	0.162275
hsa_circ_	-0.60669	0.162276
hsa_circ_	-0.45714	0.16228
hsa_circ_	-0.33587	0.16228
hsa_circ_	0.349422	0.162291
hsa_circ_	-0.28625	0.16231
hsa_circ_	-1.21322	0.162338
hsa_circ_	-0.57733	0.162342
hsa_circ_	0.357705	0.162354
hsa_circ_	0.353551	0.162367
hsa_circ_	1.540188	0.162378
hsa_circ_	0.248315	0.162406
hsa_circ_	0.379387	0.162416
hsa_circ_	0.480309	0.162421
hsa_circ_	0.352218	0.162424
hsa_circ_	-0.30936	0.162427
hsa_circ_	-0.24315	0.162429
hsa_circ_	-0.23397	0.162437
hsa_circ_	0.699917	0.162455
hsa_circ_	-0.43495	0.162466
hsa_circ_	0.272525	0.162467
hsa_circ_	-0.44613	0.162478
hsa_circ_	-0.37108	0.162485
hsa_circ_	0.270454	0.16249
hsa_circ_	-0.36313	0.162497
hsa_circ_	-0.34327	0.162501
hsa_circ_	-0.35329	0.162522
hsa_circ_	0.432621	0.162524
hsa_circ_	-0.24211	0.162524
hsa_circ_	0.233602	0.162533
hsa_circ_	-0.19394	0.162541
hsa_circ_	-0.38533	0.162545
hsa_circ_	-0.26519	0.162553
hsa_circ_	0.823861	0.162553

hsa_circ_	-0.21119	0.162556
hsa_circ_	-0.29173	0.162559
hsa_circ_	-0.74834	0.162561
hsa_circ_	-0.28228	0.162575
hsa_circ_	-0.27167	0.162577
hsa_circ_	0.682233	0.162579
hsa_circ_	0.88132	0.162583
hsa_circ_	0.45226	0.162584
hsa_circ_	0.444277	0.16259
hsa_circ_	0.346746	0.162596
hsa_circ_	0.338243	0.162639
hsa_circ_	0.247385	0.162644
hsa_circ_	0.210523	0.162666
hsa_circ_	-0.79793	0.16267
hsa_circ_	-0.23261	0.162682
hsa_circ_	0.642965	0.16269
hsa_circ_	0.397967	0.162696
hsa_circ_	0.75691	0.162703
hsa_circ_	0.235011	0.162706
hsa_circ_	0.434832	0.162766
hsa_circ_	0.949664	0.162781
hsa_circ_	0.414908	0.162793
hsa_circ_	0.815493	0.162796
hsa_circ_	-0.47266	0.162828
hsa_circ_	-0.29782	0.162833
hsa_circ_	-0.23772	0.162837
hsa_circ_	-0.26222	0.162837
hsa_circ_	0.570766	0.162867
hsa_circ_	0.209801	0.162899
hsa_circ_	0.313056	0.162953
hsa_circ_	0.236803	0.162956
hsa_circ_	0.2625	0.162956
hsa_circ_	0.635356	0.162968
hsa_circ_	0.535825	0.16298
hsa_circ_	0.230344	0.162987
hsa_circ_	-0.24394	0.162997
hsa_circ_	-0.21689	0.162998
hsa_circ_	0.2463	0.163
hsa_circ_	0.324329	0.163008
hsa_circ_	0.220199	0.163009
hsa_circ_	-0.20905	0.163011
hsa_circ_	-0.30135	0.163018
hsa_circ_	-0.24872	0.16302
hsa_circ_	-0.59486	0.163024
hsa_circ_	0.531001	0.163034
hsa_circ_	-0.28557	0.16304
hsa_circ_	-0.50137	0.163041
hsa_circ_	0.244384	0.16305
hsa_circ_	-0.45325	0.163052
hsa_circ_	-0.49303	0.163065
hsa_circ_	-0.29898	0.163069
hsa_circ_	-0.39468	0.163127
hsa_circ_	-0.27252	0.163131
hsa_circ_	0.686257	0.163146

hsa_circ_	-0.77163	0.163159
hsa_circ_	0.302428	0.163162
hsa_circ_	-0.39242	0.163166
hsa_circ_	-0.24995	0.163171
hsa_circ_	-1.71044	0.163175
hsa_circ_	0.308038	0.163181
hsa_circ_	-0.78178	0.163185
hsa_circ_	0.52812	0.163199
hsa_circ_	0.361378	0.163207
hsa_circ_	-0.45348	0.163213
hsa_circ_	0.286501	0.163219
hsa_circ_	-0.39272	0.163229
hsa_circ_	-0.27452	0.16323
hsa_circ_	-0.58612	0.163252
hsa_circ_	0.288526	0.163271
hsa_circ_	0.286069	0.163299
hsa_circ_	0.226372	0.163304
hsa_circ_	-0.18921	0.163305
hsa_circ_	-0.22885	0.163306
hsa_circ_	0.217266	0.163318
hsa_circ_	-0.27768	0.163326
hsa_circ_	-0.51758	0.163342
hsa_circ_	-0.20603	0.163346
hsa_circ_	-0.24773	0.16335
hsa_circ_	-0.75905	0.163357
hsa_circ_	-0.19431	0.163383
hsa_circ_	-0.29973	0.163394
hsa_circ_	0.263715	0.163404
hsa_circ_	-0.70954	0.163405
hsa_circ_	-0.46709	0.163412
hsa_circ_	0.285436	0.16344
hsa_circ_	-0.3311	0.163463
hsa_circ_	0.338637	0.163464
hsa_circ_	-0.25889	0.163469
hsa_circ_	0.303328	0.163474
hsa_circ_	-0.20872	0.163483
hsa_circ_	-0.23962	0.163487
hsa_circ_	0.236519	0.163496
hsa_circ_	-0.25228	0.163503
hsa_circ_	0.413397	0.163507
hsa_circ_	0.269349	0.163528
hsa_circ_	0.240911	0.163529
hsa_circ_	-0.55479	0.163531
hsa_circ_	1.722471	0.163536
hsa_circ_	-0.58639	0.163552
hsa_circ_	0.373557	0.163565
hsa_circ_	-0.29062	0.16357
hsa_circ_	-0.45078	0.163586
hsa_circ_	0.505542	0.163588
hsa_circ_	0.393234	0.163593
hsa_circ_	-0.27804	0.163594
hsa_circ_	0.822234	0.163598
hsa_circ_	0.258453	0.163604
hsa_circ_	-0.29703	0.163625

hsa_circ_	0.225005	0.163636
hsa_circ_	0.21992	0.163639
hsa_circ_	-0.23494	0.163642
hsa_circ_	0.375167	0.163663
hsa_circ_	-0.42208	0.163673
hsa_circ_	0.255539	0.163678
hsa_circ_	0.256116	0.163684
hsa_circ_	-0.56364	0.163732
hsa_circ_	-0.65529	0.163734
hsa_circ_	-0.20773	0.163735
hsa_circ_	0.290362	0.163743
hsa_circ_	-0.71243	0.163743
hsa_circ_	-0.20358	0.163744
hsa_circ_	-0.81461	0.163756
hsa_circ_	0.375146	0.163759
hsa_circ_	-0.36597	0.163762
hsa_circ_	0.679244	0.163776
hsa_circ_	0.33569	0.16378
hsa_circ_	0.895293	0.163785
hsa_circ_	1.7285	0.163795
hsa_circ_	-0.34135	0.163798
hsa_circ_	-0.5826	0.163805
hsa_circ_	-0.21931	0.163806
hsa_circ_	0.339563	0.163817
hsa_circ_	-0.30043	0.163823
hsa_circ_	0.361675	0.163835
hsa_circ_	0.506071	0.163838
hsa_circ_	-0.45132	0.163839
hsa_circ_	0.259875	0.163846
hsa_circ_	1.671545	0.163849
hsa_circ_	0.296793	0.16385
hsa_circ_	-0.29692	0.163856
hsa_circ_	0.297078	0.163864
hsa_circ_	-0.37163	0.163865
hsa_circ_	-0.22195	0.163865
hsa_circ_	0.414411	0.16388
hsa_circ_	0.217429	0.163885
hsa_circ_	-0.54498	0.163886
hsa_circ_	0.400295	0.163901
hsa_circ_	0.248585	0.163903
hsa_circ_	0.406202	0.163914
hsa_circ_	-1.06077	0.163922
hsa_circ_	-0.22358	0.163922
hsa_circ_	-0.26238	0.163928
hsa_circ_	-0.23543	0.163928
hsa_circ_	-0.35245	0.163931
hsa_circ_	-0.30527	0.163944
hsa_circ_	0.274344	0.163949
hsa_circ_	-0.23568	0.163949
hsa_circ_	-0.84571	0.16395
hsa_circ_	-0.56658	0.163957
hsa_circ_	0.291141	0.163966
hsa_circ_	-0.19958	0.163973
hsa_circ_	0.268401	0.163995

hsa_circ_	0.527675	0.163999
hsa_circ_	0.488388	0.164001
hsa_circ_	0.514123	0.164019
hsa_circ_	-0.30517	0.164019
hsa_circ_	0.505069	0.16402
hsa_circ_	0.537253	0.164021
hsa_circ_	0.362494	0.164042
hsa_circ_	0.29079	0.164062
hsa_circ_	0.316163	0.164073
hsa_circ_	0.224774	0.16408
hsa_circ_	-0.54359	0.16408
hsa_circ_	0.701773	0.164088
hsa_circ_	0.305487	0.164099
hsa_circ_	-0.65524	0.164103
hsa_circ_	-0.31888	0.164118
hsa_circ_	-0.44056	0.164122
hsa_circ_	-0.28581	0.164132
hsa_circ_	0.286098	0.164135
hsa_circ_	0.317803	0.16416
hsa_circ_	-0.44882	0.164199
hsa_circ_	-0.41426	0.164199
hsa_circ_	-0.23374	0.164207
hsa_circ_	-0.3311	0.16422
hsa_circ_	0.393457	0.164244
hsa_circ_	-0.21089	0.164244
hsa_circ_	-0.26384	0.164263
hsa_circ_	0.249014	0.164273
hsa_circ_	0.306836	0.164274
hsa_circ_	0.232303	0.164285
hsa_circ_	-0.20475	0.164305
hsa_circ_	-0.32345	0.164311
hsa_circ_	-0.34958	0.164314
hsa_circ_	0.256821	0.164318
hsa_circ_	-0.26654	0.164334
hsa_circ_	0.248708	0.164345
hsa_circ_	-0.463	0.164351
hsa_circ_	0.370606	0.164359
hsa_circ_	-0.2777	0.164367
hsa_circ_	0.468055	0.164369
hsa_circ_	0.298174	0.164379
hsa_circ_	0.468687	0.164383
hsa_circ_	-0.22292	0.164384
hsa_circ_	0.554076	0.164396
hsa_circ_	-0.20925	0.164419
hsa_circ_	0.392373	0.164431
hsa_circ_	-0.43055	0.16444
hsa_circ_	0.318992	0.164445
hsa_circ_	-0.23944	0.164452
hsa_circ_	0.322566	0.16447
hsa_circ_	-0.39848	0.16449
hsa_circ_	-0.25605	0.164501
hsa_circ_	0.295071	0.164507
hsa_circ_	0.24323	0.164511
hsa_circ_	-0.39209	0.164512

hsa_circ_	-0.56838	0.164515
hsa_circ_	1.503677	0.164528
hsa_circ_	0.253007	0.164533
hsa_circ_	0.389344	0.164551
hsa_circ_	0.232315	0.164561
hsa_circ_	0.352169	0.164569
hsa_circ_	-0.78662	0.16457
hsa_circ_	-0.28975	0.164576
hsa_circ_	0.612063	0.164589
hsa_circ_	0.350849	0.164591
hsa_circ_	0.389598	0.164592
hsa_circ_	0.331311	0.1646
hsa_circ_	0.238239	0.1646
hsa_circ_	-0.36747	0.164621
hsa_circ_	-0.26186	0.164629
hsa_circ_	0.432744	0.164631
hsa_circ_	1.303006	0.164637
hsa_circ_	-0.41867	0.164639
hsa_circ_	0.275091	0.164646
hsa_circ_	-0.44755	0.16466
hsa_circ_	0.290898	0.164689
hsa_circ_	0.432904	0.16469
hsa_circ_	0.736645	0.164694
hsa_circ_	0.233179	0.164703
hsa_circ_	-0.4554	0.164704
hsa_circ_	-0.36662	0.16471
hsa_circ_	-0.3251	0.16471
hsa_circ_	-0.21657	0.164717
hsa_circ_	0.298865	0.164719
hsa_circ_	-0.40146	0.164722
hsa_circ_	0.283708	0.164733
hsa_circ_	-0.23378	0.164739
hsa_circ_	0.24367	0.16476
hsa_circ_	-0.33888	0.164767
hsa_circ_	0.629158	0.164768
hsa_circ_	0.585106	0.164774
hsa_circ_	0.272238	0.164784
hsa_circ_	0.82114	0.164789
hsa_circ_	0.352339	0.164796
hsa_circ_	-0.38763	0.164799
hsa_circ_	-0.34118	0.164802
hsa_circ_	-0.3983	0.164805
hsa_circ_	0.216465	0.164823
hsa_circ_	0.27443	0.164832
hsa_circ_	-0.32533	0.164841
hsa_circ_	-0.3666	0.164882
hsa_circ_	0.410153	0.164888
hsa_circ_	0.383461	0.164906
hsa_circ_	-0.5097	0.164908
hsa_circ_	0.363671	0.16491
hsa_circ_	0.207337	0.164914
hsa_circ_	0.279953	0.164915
hsa_circ_	0.218138	0.164924
hsa_circ_	-0.37956	0.164926

hsa_circ_	-0.29139	0.164939
hsa_circ_	0.346988	0.164945
hsa_circ_	0.270863	0.164946
hsa_circ_	0.381039	0.164954
hsa_circ_	0.268091	0.16496
hsa_circ_	-0.29615	0.164962
hsa_circ_	0.37978	0.164965
hsa_circ_	-0.39324	0.164971
hsa_circ_	-0.21935	0.164979
hsa_circ_	-0.22254	0.164986
hsa_circ_	0.260187	0.164986
hsa_circ_	0.49375	0.165001
hsa_circ_	-1.98441	0.16501
hsa_circ_	0.360807	0.165015
hsa_circ_	-0.30508	0.165023
hsa_circ_	-0.47392	0.165028
hsa_circ_	-0.26403	0.165036
hsa_circ_	0.232283	0.165041
hsa_circ_	0.290007	0.165051
hsa_circ_	-0.3289	0.165053
hsa_circ_	0.390828	0.165056
hsa_circ_	-0.27557	0.165057
hsa_circ_	-1.12009	0.165087
hsa_circ_	-0.57132	0.165093
hsa_circ_	-0.2047	0.165107
hsa_circ_	-0.20289	0.165109
hsa_circ_	0.316303	0.165121
hsa_circ_	0.287818	0.165123
hsa_circ_	0.267484	0.165129
hsa_circ_	-0.22431	0.165131
hsa_circ_	-0.24471	0.165132
hsa_circ_	0.992413	0.165137
hsa_circ_	0.494914	0.165145
hsa_circ_	-0.57957	0.165151
hsa_circ_	0.202279	0.165153
hsa_circ_	0.53208	0.165155
hsa_circ_	-0.30094	0.165157
hsa_circ_	-0.25672	0.165158
hsa_circ_	0.207073	0.165158
hsa_circ_	0.4163	0.165166
hsa_circ_	-0.25064	0.165172
hsa_circ_	-0.24534	0.165173
hsa_circ_	-0.33045	0.165179
hsa_circ_	-0.52264	0.165188
hsa_circ_	-0.38884	0.165189
hsa_circ_	-0.31585	0.165213
hsa_circ_	-0.20022	0.165214
hsa_circ_	-0.44459	0.165216
hsa_circ_	0.216199	0.165233
hsa_circ_	-0.58656	0.165242
hsa_circ_	-0.67296	0.165251
hsa_circ_	-0.28299	0.16526
hsa_circ_	-0.22711	0.165296
hsa_circ_	-0.52498	0.165341

hsa_circ_	-0.22228	0.165347
hsa_circ_	-0.76937	0.165349
hsa_circ_	0.536655	0.165358
hsa_circ_	-0.43423	0.165359
hsa_circ_	0.312332	0.165361
hsa_circ_	-1.84059	0.165369
hsa_circ_	0.425975	0.16539
hsa_circ_	0.48443	0.165391
hsa_circ_	0.298564	0.165397
hsa_circ_	0.249771	0.165404
hsa_circ_	-0.24474	0.165416
hsa_circ_	0.23923	0.165419
hsa_circ_	-0.21956	0.165426
hsa_circ_	0.271846	0.165427
hsa_circ_	-0.39726	0.165435
hsa_circ_	-0.20245	0.165444
hsa_circ_	-0.20107	0.165449
hsa_circ_	-0.29201	0.165455
hsa_circ_	0.406832	0.16547
hsa_circ_	-0.24501	0.16548
hsa_circ_	-0.35304	0.165484
hsa_circ_	-0.24644	0.165499
hsa_circ_	0.24149	0.165509
hsa_circ_	-0.53346	0.165511
hsa_circ_	0.201206	0.165522
hsa_circ_	-0.48094	0.165529
hsa_circ_	0.281649	0.165531
hsa_circ_	-2.47855	0.165552
hsa_circ_	-0.33818	0.165552
hsa_circ_	-0.23283	0.165554
hsa_circ_	0.394463	0.165564
hsa_circ_	0.416616	0.165566
hsa_circ_	-0.29701	0.165572
hsa_circ_	-0.3186	0.165574
hsa_circ_	-0.38506	0.165579
hsa_circ_	-0.35531	0.165585
hsa_circ_	-0.28404	0.165594
hsa_circ_	0.434209	0.165596
hsa_circ_	0.257646	0.165602
hsa_circ_	-0.24835	0.165607
hsa_circ_	-0.21845	0.165607
hsa_circ_	-0.24399	0.165619
hsa_circ_	0.321494	0.165621
hsa_circ_	0.677886	0.165625
hsa_circ_	-0.30816	0.165642
hsa_circ_	0.346863	0.165644
hsa_circ_	-0.24953	0.165657
hsa_circ_	0.232832	0.165674
hsa_circ_	0.826482	0.165676
hsa_circ_	-0.42989	0.165689
hsa_circ_	0.360962	0.1657
hsa_circ_	0.334224	0.165713
hsa_circ_	0.29065	0.165714
hsa_circ_	-0.28197	0.165714

hsa_circ_	-0.32943	0.165717
hsa_circ_	0.255638	0.165735
hsa_circ_	-0.24958	0.165736
hsa_circ_	0.275966	0.165754
hsa_circ_	-0.37589	0.165755
hsa_circ_	0.465339	0.165756
hsa_circ_	-0.19942	0.165758
hsa_circ_	1.265952	0.165768
hsa_circ_	0.625593	0.165772
hsa_circ_	-0.22115	0.165773
hsa_circ_	0.365618	0.165773
hsa_circ_	-0.2016	0.165782
hsa_circ_	-0.2383	0.165793
hsa_circ_	0.260926	0.165797
hsa_circ_	-0.68828	0.1658
hsa_circ_	0.264543	0.165801
hsa_circ_	-0.23245	0.165804
hsa_circ_	0.255139	0.165808
hsa_circ_	-0.22148	0.165815
hsa_circ_	-0.36462	0.165818
hsa_circ_	0.226166	0.165822
hsa_circ_	-0.40647	0.165823
hsa_circ_	-0.21568	0.165826
hsa_circ_	-1.07344	0.165838
hsa_circ_	-0.19608	0.165865
hsa_circ_	-0.28966	0.165867
hsa_circ_	0.646887	0.165878
hsa_circ_	0.595844	0.165888
hsa_circ_	-0.55262	0.165889
hsa_circ_	0.357841	0.165893
hsa_circ_	-0.5367	0.16591
hsa_circ_	-0.33357	0.165919
hsa_circ_	0.55074	0.165934
hsa_circ_	0.359057	0.165939
hsa_circ_	-0.33831	0.16594
hsa_circ_	0.299405	0.165949
hsa_circ_	-0.37277	0.16595
hsa_circ_	0.558342	0.165952
hsa_circ_	-0.22162	0.165956
hsa_circ_	-0.26686	0.165958
hsa_circ_	0.337319	0.165964
hsa_circ_	0.462555	0.165971
hsa_circ_	-0.93091	0.165975
hsa_circ_	-0.23175	0.165978
hsa_circ_	-0.34435	0.165992
hsa_circ_	0.575539	0.165999
hsa_circ_	-0.24909	0.166
hsa_circ_	0.326496	0.166001
hsa_circ_	-0.29519	0.166009
hsa_circ_	0.264077	0.166011
hsa_circ_	-0.24735	0.166016
hsa_circ_	-0.19554	0.166019
hsa_circ_	-0.41381	0.166025
hsa_circ_	-0.45974	0.166036

hsa_circ_	0.254629	0.166039
hsa_circ_	0.285468	0.16604
hsa_circ_	0.230429	0.16604
hsa_circ_	-0.59226	0.166045
hsa_circ_	0.269826	0.166052
hsa_circ_	0.433646	0.166079
hsa_circ_	0.341656	0.166089
hsa_circ_	-0.5078	0.166096
hsa_circ_	-0.25036	0.166099
hsa_circ_	0.233345	0.166112
hsa_circ_	0.984689	0.166131
hsa_circ_	-0.20053	0.166145
hsa_circ_	0.305981	0.166164
hsa_circ_	1.285215	0.166172
hsa_circ_	0.884128	0.166186
hsa_circ_	0.812762	0.166198
hsa_circ_	0.352144	0.166201
hsa_circ_	-0.49586	0.166203
hsa_circ_	0.263189	0.166207
hsa_circ_	-0.28481	0.166219
hsa_circ_	-0.44953	0.166232
hsa_circ_	-0.30525	0.166235
hsa_circ_	0.24346	0.166237
hsa_circ_	-0.24709	0.166269
hsa_circ_	-0.2403	0.166275
hsa_circ_	-0.41021	0.166276
hsa_circ_	-0.42608	0.16629
hsa_circ_	-0.25986	0.166295
hsa_circ_	0.393772	0.166299
hsa_circ_	0.445047	0.166301
hsa_circ_	-0.22517	0.166301
hsa_circ_	-0.69798	0.166305
hsa_circ_	-0.38227	0.166315
hsa_circ_	-0.53798	0.166316
hsa_circ_	0.288336	0.166343
hsa_circ_	0.238429	0.166375
hsa_circ_	0.256763	0.166377
hsa_circ_	0.674423	0.166383
hsa_circ_	-0.58334	0.166386
hsa_circ_	0.262946	0.166404
hsa_circ_	0.488563	0.166427
hsa_circ_	-0.20692	0.166439
hsa_circ_	0.321159	0.166446
hsa_circ_	0.221231	0.166453
hsa_circ_	-0.28359	0.166462
hsa_circ_	-0.52174	0.166476
hsa_circ_	0.248152	0.16648
hsa_circ_	-0.47158	0.166509
hsa_circ_	-0.33128	0.166524
hsa_circ_	-0.44926	0.166539
hsa_circ_	-0.28028	0.166549
hsa_circ_	-0.38768	0.166549
hsa_circ_	-0.26748	0.166557
hsa_circ_	0.358759	0.166557

hsa_circ_	-0.93285	0.166582
hsa_circ_	0.956036	0.166584
hsa_circ_	-0.30576	0.1666
hsa_circ_	-0.22337	0.166605
hsa_circ_	-0.43216	0.166609
hsa_circ_	0.354373	0.166611
hsa_circ_	-0.27837	0.166614
hsa_circ_	-0.36646	0.166632
hsa_circ_	0.36789	0.166635
hsa_circ_	-0.21426	0.166659
hsa_circ_	0.398749	0.166663
hsa_circ_	-0.25149	0.166695
hsa_circ_	0.242805	0.166701
hsa_circ_	-0.31453	0.166705
hsa_circ_	-0.8608	0.166707
hsa_circ_	-0.2271	0.166714
hsa_circ_	0.377878	0.166719
hsa_circ_	0.275794	0.166738
hsa_circ_	0.411536	0.166738
hsa_circ_	0.42244	0.166739
hsa_circ_	0.231102	0.166767
hsa_circ_	0.365771	0.166769
hsa_circ_	0.21713	0.166774
hsa_circ_	-0.21638	0.166775
hsa_circ_	-0.29477	0.166775
hsa_circ_	-0.37218	0.166779
hsa_circ_	-0.93908	0.166781
hsa_circ_	0.935617	0.166784
hsa_circ_	0.386306	0.166797
hsa_circ_	-0.31209	0.1668
hsa_circ_	-1.33964	0.166818
hsa_circ_	0.462036	0.166819
hsa_circ_	1.068651	0.166829
hsa_circ_	0.581891	0.166833
hsa_circ_	0.510809	0.166865
hsa_circ_	0.280392	0.166869
hsa_circ_	0.283777	0.166874
hsa_circ_	-0.24424	0.166876
hsa_circ_	-0.23652	0.166878
hsa_circ_	-0.55748	0.166887
hsa_circ_	0.540091	0.166934
hsa_circ_	0.383316	0.16696
hsa_circ_	-0.36869	0.166969
hsa_circ_	-0.19193	0.166986
hsa_circ_	0.269015	0.166995
hsa_circ_	0.676309	0.167002
hsa_circ_	-0.28936	0.167012
hsa_circ_	-0.38476	0.167029
hsa_circ_	0.766816	0.167038
hsa_circ_	-0.22402	0.167058
hsa_circ_	-0.24457	0.167071
hsa_circ_	0.232949	0.167073
hsa_circ_	-0.27439	0.167087
hsa_circ_	-0.32248	0.167089

hsa_circ_	-0.59744	0.167089
hsa_circ_	0.335875	0.167096
hsa_circ_	-0.26832	0.167097
hsa_circ_	-0.20669	0.167107
hsa_circ_	-0.30191	0.167107
hsa_circ_	-0.40196	0.167114
hsa_circ_	0.872581	0.167134
hsa_circ_	0.374792	0.167142
hsa_circ_	-0.24745	0.167144
hsa_circ_	-0.56996	0.167147
hsa_circ_	0.208373	0.167151
hsa_circ_	0.458109	0.167159
hsa_circ_	0.334488	0.167159
hsa_circ_	0.818471	0.167159
hsa_circ_	-0.36686	0.167177
hsa_circ_	0.319896	0.167181
hsa_circ_	-0.29008	0.167195
hsa_circ_	-0.31048	0.167207
hsa_circ_	0.247625	0.167215
hsa_circ_	-0.28565	0.167222
hsa_circ_	-0.2442	0.167227
hsa_circ_	0.222181	0.167239
hsa_circ_	-0.66369	0.167273
hsa_circ_	0.206295	0.167282
hsa_circ_	0.38821	0.167285
hsa_circ_	0.350215	0.167289
hsa_circ_	-0.8971	0.167294
hsa_circ_	-0.93193	0.167313
hsa_circ_	1.248278	0.167319
hsa_circ_	-0.76866	0.16733
hsa_circ_	0.43346	0.167333
hsa_circ_	-0.37868	0.167334
hsa_circ_	-0.34557	0.167343
hsa_circ_	-0.52592	0.167344
hsa_circ_	0.28961	0.167345
hsa_circ_	0.827944	0.167359
hsa_circ_	-0.2621	0.167359
hsa_circ_	-0.36498	0.16736
hsa_circ_	-0.43468	0.167363
hsa_circ_	0.420426	0.167366
hsa_circ_	0.376146	0.167366
hsa_circ_	-0.21765	0.167373
hsa_circ_	0.238293	0.167392
hsa_circ_	-0.27925	0.167413
hsa_circ_	-0.36951	0.167413
hsa_circ_	0.230673	0.167416
hsa_circ_	0.289575	0.167423
hsa_circ_	0.283537	0.167432
hsa_circ_	0.260142	0.167434
hsa_circ_	-0.55219	0.167435
hsa_circ_	-0.3302	0.167437
hsa_circ_	0.372368	0.167441
hsa_circ_	0.264344	0.167457
hsa_circ_	-0.2614	0.16746

hsa_circ_	0.205375	0.167466
hsa_circ_	0.3138	0.167473
hsa_circ_	0.237828	0.167478
hsa_circ_	0.664084	0.167487
hsa_circ_	-0.38156	0.167491
hsa_circ_	-0.85722	0.167496
hsa_circ_	0.353934	0.167502
hsa_circ_	-0.28388	0.167504
hsa_circ_	-0.70287	0.167513
hsa_circ_	-0.22232	0.167525
hsa_circ_	0.302897	0.167532
hsa_circ_	-0.30659	0.167533
hsa_circ_	-0.25648	0.16755
hsa_circ_	-0.36297	0.167557
hsa_circ_	-0.33713	0.167573
hsa_circ_	0.546272	0.167579
hsa_circ_	-0.22996	0.167595
hsa_circ_	0.407167	0.167608
hsa_circ_	0.796646	0.167615
hsa_circ_	-0.58994	0.16762
hsa_circ_	0.30671	0.167641
hsa_circ_	-0.19199	0.167657
hsa_circ_	0.351686	0.167668
hsa_circ_	-0.79629	0.167673
hsa_circ_	0.582219	0.167678
hsa_circ_	-1.42325	0.167689
hsa_circ_	-0.23468	0.167723
hsa_circ_	0.348021	0.167728
hsa_circ_	-0.20913	0.167748
hsa_circ_	0.585009	0.167787
hsa_circ_	-0.58609	0.167789
hsa_circ_	1.859322	0.167791
hsa_circ_	0.350799	0.167806
hsa_circ_	-1.12365	0.16782
hsa_circ_	0.203149	0.167833
hsa_circ_	0.379717	0.167837
hsa_circ_	1.073883	0.167848
hsa_circ_	0.267777	0.167849
hsa_circ_	0.420493	0.167851
hsa_circ_	-0.25824	0.167859
hsa_circ_	-0.27813	0.167865
hsa_circ_	-0.27973	0.16788
hsa_circ_	0.276813	0.167881
hsa_circ_	-0.76014	0.167883
hsa_circ_	0.255546	0.167886
hsa_circ_	0.246572	0.167889
hsa_circ_	0.208622	0.167919
hsa_circ_	0.257018	0.167924
hsa_circ_	1.573283	0.167926
hsa_circ_	-0.35332	0.16794
hsa_circ_	0.36414	0.167943
hsa_circ_	-0.61655	0.167944
hsa_circ_	0.346199	0.167949
hsa_circ_	-0.29637	0.167968

hsa_circ_	-0.62889	0.167975
hsa_circ_	-0.28914	0.167976
hsa_circ_	-0.6552	0.16798
hsa_circ_	-0.38097	0.167981
hsa_circ_	-1.16356	0.167984
hsa_circ_	0.493059	0.167989
hsa_circ_	0.204088	0.167989
hsa_circ_	-0.29925	0.167989
hsa_circ_	-0.467	0.168006
hsa_circ_	-0.55751	0.16801
hsa_circ_	-0.30667	0.16803
hsa_circ_	0.575292	0.168034
hsa_circ_	-0.25997	0.168044
hsa_circ_	0.317101	0.168053
hsa_circ_	-0.22516	0.168055
hsa_circ_	-0.37685	0.168055
hsa_circ_	0.187323	0.168057
hsa_circ_	-0.36001	0.168067
hsa_circ_	-0.84115	0.168075
hsa_circ_	0.921594	0.168094
hsa_circ_	0.430598	0.168098
hsa_circ_	-0.76699	0.168105
hsa_circ_	1.086356	0.168112
hsa_circ_	-0.2704	0.168125
hsa_circ_	0.309787	0.168133
hsa_circ_	0.366211	0.16816
hsa_circ_	-0.33383	0.168172
hsa_circ_	-0.38636	0.168173
hsa_circ_	0.314509	0.168187
hsa_circ_	-0.56785	0.168193
hsa_circ_	-0.43665	0.168202
hsa_circ_	0.328954	0.168206
hsa_circ_	0.242663	0.168207
hsa_circ_	-0.21858	0.168217
hsa_circ_	-0.23055	0.168221
hsa_circ_	0.651937	0.16824
hsa_circ_	-0.18949	0.168243
hsa_circ_	-0.2471	0.168261
hsa_circ_	-0.21693	0.168286
hsa_circ_	-0.2779	0.168304
hsa_circ_	-0.26378	0.168308
hsa_circ_	0.227308	0.16831
hsa_circ_	0.546568	0.16832
hsa_circ_	-0.27371	0.168328
hsa_circ_	-0.25041	0.168329
hsa_circ_	0.880379	0.168333
hsa_circ_	-0.26375	0.168339
hsa_circ_	0.23937	0.168344
hsa_circ_	-0.56352	0.168371
hsa_circ_	0.524017	0.168392
hsa_circ_	-0.36026	0.168394
hsa_circ_	0.200864	0.168396
hsa_circ_	-0.33489	0.168412
hsa_circ_	-0.19789	0.168412

hsa_circ_	0.224919	0.16844
hsa_circ_	0.367889	0.168461
hsa_circ_	0.229811	0.168474
hsa_circ_	0.236215	0.168474
hsa_circ_	0.363169	0.168478
hsa_circ_	0.536297	0.168484
hsa_circ_	-0.37522	0.168487
hsa_circ_	-0.36163	0.168487
hsa_circ_	-0.26303	0.16849
hsa_circ_	-0.22588	0.168499
hsa_circ_	-0.20837	0.168503
hsa_circ_	0.292441	0.168529
hsa_circ_	-0.27551	0.16853
hsa_circ_	-0.24529	0.168531
hsa_circ_	-1.40567	0.168537
hsa_circ_	-0.35259	0.168539
hsa_circ_	-0.62371	0.168542
hsa_circ_	0.251384	0.168567
hsa_circ_	-0.67236	0.168595
hsa_circ_	-0.31979	0.168598
hsa_circ_	-0.26389	0.168603
hsa_circ_	-0.41298	0.168615
hsa_circ_	0.287109	0.168633
hsa_circ_	-0.25153	0.168636
hsa_circ_	-0.76375	0.168649
hsa_circ_	-0.29341	0.168649
hsa_circ_	0.365096	0.168663
hsa_circ_	-0.38876	0.168665
hsa_circ_	1.065733	0.168667
hsa_circ_	-0.24922	0.168679
hsa_circ_	0.316986	0.168697
hsa_circ_	0.575325	0.1687
hsa_circ_	0.282071	0.168707
hsa_circ_	-0.20183	0.168707
hsa_circ_	0.466583	0.168712
hsa_circ_	-1.26572	0.168724
hsa_circ_	-0.26562	0.168738
hsa_circ_	-0.51037	0.168748
hsa_circ_	-0.69067	0.168757
hsa_circ_	-0.25736	0.168767
hsa_circ_	0.259026	0.168778
hsa_circ_	-0.51445	0.168788
hsa_circ_	0.297854	0.168796
hsa_circ_	0.267445	0.168797
hsa_circ_	-0.23848	0.168802
hsa_circ_	0.191832	0.168824
hsa_circ_	0.284269	0.168835
hsa_circ_	-0.3528	0.168836
hsa_circ_	0.226531	0.168838
hsa_circ_	-0.28711	0.16884
hsa_circ_	0.678567	0.168853
hsa_circ_	0.372478	0.168868
hsa_circ_	0.778867	0.168872
hsa_circ_	0.384425	0.168894

hsa_circ_	0.314846	0.168895
hsa_circ_	0.233687	0.168906
hsa_circ_	0.405382	0.168913
hsa_circ_	0.509323	0.168924
hsa_circ_	1.116507	0.168925
hsa_circ_	0.202792	0.168933
hsa_circ_	-0.22212	0.168935
hsa_circ_	-0.52001	0.168975
hsa_circ_	0.941616	0.168984
hsa_circ_	-0.26091	0.169024
hsa_circ_	-0.50427	0.169034
hsa_circ_	0.568142	0.169063
hsa_circ_	0.253424	0.169065
hsa_circ_	0.653457	0.169076
hsa_circ_	0.206988	0.169085
hsa_circ_	0.210655	0.169085
hsa_circ_	0.366234	0.169092
hsa_circ_	-0.37031	0.169121
hsa_circ_	-0.26696	0.169122
hsa_circ_	-0.47455	0.169126
hsa_circ_	0.293473	0.169127
hsa_circ_	0.279451	0.169142
hsa_circ_	-0.3816	0.169166
hsa_circ_	-0.47304	0.16918
hsa_circ_	0.21585	0.169186
hsa_circ_	-0.48372	0.169208
hsa_circ_	-0.32507	0.169252
hsa_circ_	0.411166	0.169264
hsa_circ_	-0.36041	0.169274
hsa_circ_	-0.50014	0.169295
hsa_circ_	0.256801	0.169319
hsa_circ_	0.51491	0.169319
hsa_circ_	-0.31383	0.169327
hsa_circ_	0.344976	0.169328
hsa_circ_	-0.47224	0.169335
hsa_circ_	0.271188	0.169344
hsa_circ_	0.290344	0.16935
hsa_circ_	-0.92561	0.169365
hsa_circ_	-0.20432	0.169384
hsa_circ_	0.64474	0.16939
hsa_circ_	0.299206	0.169425
hsa_circ_	-0.38164	0.169432
hsa_circ_	-0.20635	0.169441
hsa_circ_	-0.508	0.169445
hsa_circ_	0.451684	0.169458
hsa_circ_	0.300237	0.169462
hsa_circ_	-0.30044	0.169486
hsa_circ_	0.217842	0.169493
hsa_circ_	0.301524	0.169525
hsa_circ_	0.277211	0.16953
hsa_circ_	-0.26155	0.169535
hsa_circ_	-0.18694	0.16954
hsa_circ_	0.205982	0.169543
hsa_circ_	-0.27121	0.16955

hsa_circ_	0.344591	0.169572
hsa_circ_	-0.36581	0.169581
hsa_circ_	0.189338	0.169584
hsa_circ_	0.239802	0.169605
hsa_circ_	0.333326	0.169611
hsa_circ_	-0.41576	0.16962
hsa_circ_	0.472767	0.16962
hsa_circ_	-0.32486	0.169651
hsa_circ_	-0.20244	0.16967
hsa_circ_	-0.25646	0.169679
hsa_circ_	-0.29524	0.169679
hsa_circ_	0.350123	0.169685
hsa_circ_	0.255601	0.169695
hsa_circ_	0.339695	0.1697
hsa_circ_	-0.37572	0.1697
hsa_circ_	-0.60711	0.1697
hsa_circ_	0.204224	0.169705
hsa_circ_	-1.2429	0.169705
hsa_circ_	-0.24525	0.169723
hsa_circ_	-0.75748	0.169738
hsa_circ_	-0.25947	0.16975
hsa_circ_	-0.29291	0.169752
hsa_circ_	0.34021	0.169757
hsa_circ_	-0.19216	0.169762
hsa_circ_	0.254809	0.169765
hsa_circ_	-0.32201	0.169788
hsa_circ_	0.488448	0.16979
hsa_circ_	-0.57536	0.169796
hsa_circ_	0.979496	0.169803
hsa_circ_	0.301917	0.169804
hsa_circ_	0.880405	0.169831
hsa_circ_	-0.30804	0.169848
hsa_circ_	-0.29516	0.169853
hsa_circ_	0.710721	0.169857
hsa_circ_	0.44631	0.16986
hsa_circ_	-0.84877	0.169862
hsa_circ_	0.952768	0.169864
hsa_circ_	-0.32869	0.169867
hsa_circ_	-0.32735	0.169873
hsa_circ_	-0.46566	0.169882
hsa_circ_	-0.44331	0.169882
hsa_circ_	-0.19101	0.169883
hsa_circ_	-0.36039	0.169902
hsa_circ_	0.272069	0.169905
hsa_circ_	-0.25035	0.169963
hsa_circ_	0.520516	0.17
hsa_circ_	-0.47415	0.170002
hsa_circ_	0.907509	0.170017
hsa_circ_	-0.26881	0.17002
hsa_circ_	0.585565	0.170028
hsa_circ_	-0.41155	0.170034
hsa_circ_	0.21233	0.170034
hsa_circ_	-0.22535	0.170051
hsa_circ_	0.214332	0.170057

hsa_circ_	-0.55085	0.170068
hsa_circ_	0.391792	0.170091
hsa_circ_	-0.28117	0.170093
hsa_circ_	0.21197	0.170094
hsa_circ_	-0.29641	0.170106
hsa_circ_	0.363614	0.170107
hsa_circ_	-0.20611	0.170113
hsa_circ_	-0.37581	0.170119
hsa_circ_	0.193833	0.170131
hsa_circ_	0.395508	0.170146
hsa_circ_	0.415944	0.170147
hsa_circ_	-0.40441	0.170153
hsa_circ_	-0.19072	0.170183
hsa_circ_	-0.21707	0.170191
hsa_circ_	0.815725	0.170199
hsa_circ_	0.336564	0.170207
hsa_circ_	-0.23415	0.170215
hsa_circ_	-0.49857	0.170222
hsa_circ_	0.385081	0.170241
hsa_circ_	0.315969	0.17026
hsa_circ_	-0.43739	0.170274
hsa_circ_	0.379729	0.170276
hsa_circ_	-0.35818	0.17028
hsa_circ_	0.299103	0.170281
hsa_circ_	-0.36897	0.170299
hsa_circ_	-0.45528	0.170301
hsa_circ_	-0.23455	0.170301
hsa_circ_	-0.25226	0.170307
hsa_circ_	-0.36347	0.170314
hsa_circ_	0.337952	0.170316
hsa_circ_	-1.0322	0.170322
hsa_circ_	0.544014	0.170329
hsa_circ_	-0.24067	0.170346
hsa_circ_	0.310272	0.170356
hsa_circ_	-0.2598	0.170358
hsa_circ_	-0.26226	0.170374
hsa_circ_	0.258613	0.1704
hsa_circ_	0.443696	0.170404
hsa_circ_	-0.28033	0.170405
hsa_circ_	-0.20943	0.170407
hsa_circ_	-0.30321	0.170415
hsa_circ_	-0.34351	0.170421
hsa_circ_	0.466337	0.170423
hsa_circ_	-0.18639	0.170427
hsa_circ_	0.578736	0.170433
hsa_circ_	1.004578	0.170434
hsa_circ_	-0.46206	0.170437
hsa_circ_	-0.59077	0.170451
hsa_circ_	0.21256	0.170451
hsa_circ_	-1.04003	0.170453
hsa_circ_	0.23201	0.170471
hsa_circ_	0.523754	0.170496
hsa_circ_	0.269001	0.170503
hsa_circ_	0.335383	0.170523

hsa_circ_	0.277371	0.17054
hsa_circ_	0.22918	0.170549
hsa_circ_	-0.26929	0.170556
hsa_circ_	0.271729	0.170561
hsa_circ_	-0.22085	0.170566
hsa_circ_	0.536534	0.170569
hsa_circ_	-0.2906	0.170577
hsa_circ_	-0.23971	0.170589
hsa_circ_	-0.30875	0.170594
hsa_circ_	0.775929	0.170596
hsa_circ_	-0.25227	0.170633
hsa_circ_	0.438189	0.170638
hsa_circ_	0.463992	0.170648
hsa_circ_	0.405202	0.170652
hsa_circ_	-1.33599	0.170662
hsa_circ_	0.632967	0.170664
hsa_circ_	0.580635	0.170684
hsa_circ_	1.090383	0.170688
hsa_circ_	-0.33499	0.170715
hsa_circ_	0.34641	0.170718
hsa_circ_	0.252603	0.170722
hsa_circ_	-0.22415	0.17073
hsa_circ_	0.229544	0.17073
hsa_circ_	-0.25063	0.170731
hsa_circ_	-0.26849	0.170732
hsa_circ_	0.363707	0.170733
hsa_circ_	-0.35824	0.170735
hsa_circ_	0.738017	0.170735
hsa_circ_	1.600064	0.170737
hsa_circ_	0.233312	0.170738
hsa_circ_	0.246234	0.170738
hsa_circ_	0.726422	0.170762
hsa_circ_	0.728986	0.170767
hsa_circ_	-0.57737	0.170791
hsa_circ_	-0.25353	0.170798
hsa_circ_	-0.41961	0.170803
hsa_circ_	-0.34591	0.170804
hsa_circ_	0.237824	0.170809
hsa_circ_	-0.28334	0.170826
hsa_circ_	0.234149	0.170843
hsa_circ_	0.270674	0.17085
hsa_circ_	0.400022	0.170862
hsa_circ_	-0.96261	0.170869
hsa_circ_	-0.31961	0.170872
hsa_circ_	-0.29039	0.170905
hsa_circ_	0.269858	0.170914
hsa_circ_	-0.25229	0.170915
hsa_circ_	-0.20822	0.170921
hsa_circ_	-0.396	0.170924
hsa_circ_	0.285472	0.170927
hsa_circ_	0.298516	0.170927
hsa_circ_	0.442682	0.170927
hsa_circ_	-0.3556	0.17095
hsa_circ_	-0.2367	0.170964

hsa_circ_	-0.29002	0.170973
hsa_circ_	0.21515	0.170978
hsa_circ_	-0.47168	0.17098
hsa_circ_	0.261284	0.170996
hsa_circ_	-0.30316	0.171029
hsa_circ_	-0.72983	0.171056
hsa_circ_	-0.47583	0.171063
hsa_circ_	0.249096	0.171072
hsa_circ_	0.269426	0.171084
hsa_circ_	0.315475	0.171092
hsa_circ_	0.225001	0.171093
hsa_circ_	0.277924	0.17111
hsa_circ_	-0.22341	0.171127
hsa_circ_	-0.22858	0.171137
hsa_circ_	-0.21899	0.17114
hsa_circ_	0.511756	0.171157
hsa_circ_	-0.20496	0.171162
hsa_circ_	-0.2685	0.171163
hsa_circ_	0.278823	0.171165
hsa_circ_	-0.41254	0.171171
hsa_circ_	-0.34351	0.171184
hsa_circ_	-0.3942	0.171195
hsa_circ_	0.306155	0.171212
hsa_circ_	0.290603	0.171223
hsa_circ_	0.319588	0.171232
hsa_circ_	0.346811	0.171244
hsa_circ_	-0.25009	0.171255
hsa_circ_	-0.49689	0.171266
hsa_circ_	0.222107	0.171271
hsa_circ_	-0.37885	0.171272
hsa_circ_	-0.33683	0.171283
hsa_circ_	0.47753	0.171287
hsa_circ_	0.225671	0.171299
hsa_circ_	0.240643	0.171301
hsa_circ_	-0.52475	0.171303
hsa_circ_	0.643074	0.171331
hsa_circ_	-0.27025	0.171346
hsa_circ_	-0.7692	0.171346
hsa_circ_	0.226003	0.171347
hsa_circ_	-0.35835	0.171373
hsa_circ_	-0.58163	0.171375
hsa_circ_	0.746468	0.171401
hsa_circ_	-0.66582	0.171419
hsa_circ_	-0.40755	0.17143
hsa_circ_	-0.21192	0.171433
hsa_circ_	0.236751	0.171443
hsa_circ_	0.264798	0.171445
hsa_circ_	-0.75452	0.171452
hsa_circ_	-0.62435	0.171454
hsa_circ_	-0.20507	0.171455
hsa_circ_	-0.24682	0.171463
hsa_circ_	-0.28135	0.171491
hsa_circ_	-0.45854	0.171493
hsa_circ_	-0.25831	0.171504

hsa_circ_	0.586828	0.171506
hsa_circ_	-0.2254	0.171517
hsa_circ_	-0.35016	0.171517
hsa_circ_	-0.50563	0.171519
hsa_circ_	-0.76128	0.171521
hsa_circ_	0.485185	0.171529
hsa_circ_	-0.36134	0.171537
hsa_circ_	0.345998	0.171558
hsa_circ_	0.244334	0.171576
hsa_circ_	-0.22873	0.171581
hsa_circ_	0.296021	0.171591
hsa_circ_	0.276875	0.171597
hsa_circ_	-0.18849	0.171605
hsa_circ_	0.240784	0.171631
hsa_circ_	-0.73609	0.171635
hsa_circ_	0.195172	0.171643
hsa_circ_	-0.24019	0.171658
hsa_circ_	-0.24356	0.171663
hsa_circ_	0.251373	0.171667
hsa_circ_	-0.59529	0.171671
hsa_circ_	-0.21721	0.17169
hsa_circ_	0.297318	0.171692
hsa_circ_	-0.32596	0.171695
hsa_circ_	-0.45775	0.1717
hsa_circ_	-0.57367	0.171731
hsa_circ_	-0.27095	0.171732
hsa_circ_	0.332319	0.171735
hsa_circ_	0.725698	0.171748
hsa_circ_	-0.26067	0.171773
hsa_circ_	0.341279	0.171774
hsa_circ_	-0.64164	0.171775
hsa_circ_	-0.95118	0.171789
hsa_circ_	-0.28826	0.171798
hsa_circ_	0.605127	0.1718
hsa_circ_	-0.24271	0.171805
hsa_circ_	-0.35083	0.171813
hsa_circ_	0.544979	0.171822
hsa_circ_	0.339906	0.171824
hsa_circ_	-0.24451	0.171851
hsa_circ_	-0.32436	0.171853
hsa_circ_	-0.24313	0.171868
hsa_circ_	0.290759	0.171868
hsa_circ_	0.93799	0.171885
hsa_circ_	0.823341	0.171914
hsa_circ_	0.459359	0.171922
hsa_circ_	0.246929	0.171934
hsa_circ_	-0.33083	0.17194
hsa_circ_	1.015801	0.171942
hsa_circ_	-0.54252	0.171953
hsa_circ_	0.599774	0.171959
hsa_circ_	0.35065	0.171961
hsa_circ_	1.119642	0.171975
hsa_circ_	0.318309	0.171976
hsa_circ_	-0.24846	0.171984

hsa_circ_	0.205413	0.171999
hsa_circ_	0.220408	0.172013
hsa_circ_	0.232453	0.172021
hsa_circ_	0.235545	0.172022
hsa_circ_	0.315684	0.172023
hsa_circ_	-0.20782	0.172025
hsa_circ_	0.262427	0.172034
hsa_circ_	-0.20997	0.172036
hsa_circ_	0.594113	0.172039
hsa_circ_	-0.23039	0.172051
hsa_circ_	-0.23336	0.172052
hsa_circ_	0.574776	0.172057
hsa_circ_	0.54449	0.172061
hsa_circ_	-0.79685	0.172061
hsa_circ_	-0.24926	0.172075
hsa_circ_	-0.21921	0.172075
hsa_circ_	0.471576	0.172078
hsa_circ_	0.657353	0.172085
hsa_circ_	-0.23237	0.172087
hsa_circ_	0.291507	0.1721
hsa_circ_	-0.28559	0.172102
hsa_circ_	0.317014	0.172124
hsa_circ_	0.250924	0.172139
hsa_circ_	-0.53542	0.172143
hsa_circ_	0.292066	0.172147
hsa_circ_	0.238371	0.172158
hsa_circ_	0.444982	0.172159
hsa_circ_	-0.263	0.17216
hsa_circ_	0.303615	0.172193
hsa_circ_	0.228771	0.172193
hsa_circ_	0.313856	0.172205
hsa_circ_	0.298161	0.17221
hsa_circ_	-0.3984	0.172223
hsa_circ_	0.308063	0.172247
hsa_circ_	-0.38033	0.172254
hsa_circ_	-0.40727	0.172257
hsa_circ_	0.261482	0.172282
hsa_circ_	0.377426	0.172324
hsa_circ_	0.244308	0.172333
hsa_circ_	0.559945	0.172334
hsa_circ_	-0.31876	0.172337
hsa_circ_	0.250762	0.172339
hsa_circ_	0.356977	0.172366
hsa_circ_	-0.72517	0.172372
hsa_circ_	-0.28667	0.172378
hsa_circ_	-0.27135	0.172381
hsa_circ_	0.733773	0.172384
hsa_circ_	0.313051	0.172395
hsa_circ_	-0.3566	0.172401
hsa_circ_	-0.19985	0.172419
hsa_circ_	-0.29782	0.172422
hsa_circ_	-0.25635	0.172432
hsa_circ_	0.226684	0.172438
hsa_circ_	-0.96241	0.172445

hsa_circ_	0.254856	0.172449
hsa_circ_	1.650368	0.172449
hsa_circ_	-0.34174	0.172465
hsa_circ_	-0.29894	0.172494
hsa_circ_	-1.39481	0.172498
hsa_circ_	0.266545	0.172519
hsa_circ_	0.34353	0.172526
hsa_circ_	-0.20857	0.172537
hsa_circ_	0.209716	0.172572
hsa_circ_	0.379942	0.172575
hsa_circ_	-0.34405	0.172575
hsa_circ_	-0.40949	0.172576
hsa_circ_	0.342077	0.172596
hsa_circ_	0.259789	0.172616
hsa_circ_	-0.73208	0.172618
hsa_circ_	-0.3373	0.172635
hsa_circ_	-0.23171	0.172637
hsa_circ_	0.242732	0.172646
hsa_circ_	0.224253	0.172661
hsa_circ_	0.220606	0.172672
hsa_circ_	-0.20762	0.172678
hsa_circ_	0.274811	0.172693
hsa_circ_	-0.49404	0.172696
hsa_circ_	0.891079	0.172716
hsa_circ_	0.245027	0.172718
hsa_circ_	0.462334	0.172723
hsa_circ_	0.358608	0.172725
hsa_circ_	0.531038	0.172745
hsa_circ_	-0.43694	0.172762
hsa_circ_	0.20544	0.17277
hsa_circ_	-0.36213	0.17281
hsa_circ_	-0.21078	0.172834
hsa_circ_	0.298373	0.172843
hsa_circ_	0.26633	0.172844
hsa_circ_	-0.21063	0.172848
hsa_circ_	0.354856	0.172848
hsa_circ_	-0.19066	0.172864
hsa_circ_	0.310153	0.172868
hsa_circ_	-1.05975	0.172875
hsa_circ_	-0.232	0.172883
hsa_circ_	0.327223	0.17289
hsa_circ_	0.339158	0.172892
hsa_circ_	-0.53037	0.172896
hsa_circ_	-0.3661	0.172907
hsa_circ_	0.198147	0.172916
hsa_circ_	1.094933	0.172922
hsa_circ_	-0.50496	0.172941
hsa_circ_	0.19869	0.172964
hsa_circ_	-0.86746	0.172969
hsa_circ_	0.202446	0.172982
hsa_circ_	-0.31553	0.172983
hsa_circ_	-0.20397	0.172994
hsa_circ_	-0.82673	0.173009
hsa_circ_	-0.45684	0.173011

hsa_circ_	0.306793	0.173015
hsa_circ_	0.252541	0.173026
hsa_circ_	-0.22025	0.173027
hsa_circ_	0.270372	0.173029
hsa_circ_	0.540864	0.17303
hsa_circ_	0.782846	0.17304
hsa_circ_	0.51072	0.173056
hsa_circ_	-0.25524	0.17306
hsa_circ_	0.983823	0.173063
hsa_circ_	0.695467	0.173068
hsa_circ_	-0.24679	0.173069
hsa_circ_	-0.18764	0.173072
hsa_circ_	-0.25617	0.173077
hsa_circ_	0.346426	0.173087
hsa_circ_	0.500349	0.173139
hsa_circ_	0.415669	0.173157
hsa_circ_	-0.43292	0.173168
hsa_circ_	0.290517	0.173173
hsa_circ_	0.354997	0.173176
hsa_circ_	-0.33657	0.173182
hsa_circ_	0.277072	0.173192
hsa_circ_	0.466334	0.173193
hsa_circ_	0.236191	0.173199
hsa_circ_	-0.24903	0.173205
hsa_circ_	0.196556	0.173207
hsa_circ_	-0.21089	0.173232
hsa_circ_	-0.35296	0.173241
hsa_circ_	0.315327	0.173243
hsa_circ_	-0.33394	0.173253
hsa_circ_	-0.43053	0.173257
hsa_circ_	-0.35858	0.173264
hsa_circ_	0.470106	0.173279
hsa_circ_	0.285793	0.173293
hsa_circ_	-0.63718	0.173294
hsa_circ_	0.230096	0.1733
hsa_circ_	-0.2048	0.1733
hsa_circ_	0.215224	0.173302
hsa_circ_	-0.21069	0.173333
hsa_circ_	-1.6322	0.173346
hsa_circ_	0.660434	0.173364
hsa_circ_	0.28654	0.173366
hsa_circ_	-0.7805	0.173371
hsa_circ_	-0.43027	0.173373
hsa_circ_	1.302322	0.173375
hsa_circ_	0.223728	0.173382
hsa_circ_	-0.38062	0.173386
hsa_circ_	0.216303	0.173386
hsa_circ_	0.261447	0.1734
hsa_circ_	-0.41773	0.173406
hsa_circ_	-0.25132	0.173412
hsa_circ_	0.412842	0.173419
hsa_circ_	-0.22174	0.173429
hsa_circ_	0.291666	0.17343
hsa_circ_	-0.36029	0.173431

hsa_circ_	0.373579	0.173436
hsa_circ_	-0.34642	0.173447
hsa_circ_	-0.23212	0.17345
hsa_circ_	0.323181	0.173458
hsa_circ_	-0.3136	0.173467
hsa_circ_	0.437111	0.173472
hsa_circ_	0.845613	0.173481
hsa_circ_	0.294343	0.173492
hsa_circ_	0.322707	0.173494
hsa_circ_	0.236733	0.173503
hsa_circ_	-0.34888	0.173518
hsa_circ_	0.245971	0.173542
hsa_circ_	1.00187	0.173544
hsa_circ_	0.24499	0.173545
hsa_circ_	-0.41768	0.173552
hsa_circ_	0.252401	0.173554
hsa_circ_	-0.51471	0.173562
hsa_circ_	0.254358	0.173564
hsa_circ_	-0.50911	0.173577
hsa_circ_	0.547636	0.173583
hsa_circ_	-0.22794	0.173586
hsa_circ_	0.299474	0.173595
hsa_circ_	0.344968	0.173596
hsa_circ_	0.245598	0.173598
hsa_circ_	-0.25406	0.173601
hsa_circ_	0.207345	0.173602
hsa_circ_	-0.30443	0.173608
hsa_circ_	0.418212	0.173614
hsa_circ_	-0.33657	0.17365
hsa_circ_	0.243494	0.173675
hsa_circ_	-0.20211	0.173686
hsa_circ_	0.288563	0.173693
hsa_circ_	-0.33062	0.173694
hsa_circ_	0.512103	0.173712
hsa_circ_	0.310428	0.173715
hsa_circ_	-0.92442	0.173715
hsa_circ_	0.466531	0.173719
hsa_circ_	-0.21784	0.173727
hsa_circ_	-0.23715	0.173742
hsa_circ_	0.254728	0.173743
hsa_circ_	0.23373	0.173746
hsa_circ_	-0.20014	0.173747
hsa_circ_	0.417976	0.173757
hsa_circ_	-0.26278	0.17377
hsa_circ_	0.270792	0.173772
hsa_circ_	0.940604	0.173781
hsa_circ_	0.226058	0.173791
hsa_circ_	-0.27903	0.173792
hsa_circ_	0.242829	0.173809
hsa_circ_	-1.00404	0.173822
hsa_circ_	0.275235	0.173825
hsa_circ_	-0.46232	0.173828
hsa_circ_	0.206493	0.173829
hsa_circ_	0.265197	0.173831

hsa_circ_	-0.23755	0.173859
hsa_circ_	-0.22605	0.173863
hsa_circ_	-0.19718	0.173869
hsa_circ_	-0.42805	0.173887
hsa_circ_	-0.21498	0.17389
hsa_circ_	0.239646	0.173893
hsa_circ_	0.453314	0.173908
hsa_circ_	0.224492	0.173916
hsa_circ_	-0.27869	0.173926
hsa_circ_	0.264988	0.173934
hsa_circ_	0.377358	0.173947
hsa_circ_	-0.21783	0.173953
hsa_circ_	0.710639	0.173979
hsa_circ_	-0.23208	0.17398
hsa_circ_	-0.46566	0.173981
hsa_circ_	-0.47784	0.173989
hsa_circ_	-0.33876	0.174
hsa_circ_	-0.20675	0.174004
hsa_circ_	-0.45989	0.174009
hsa_circ_	0.999607	0.174014
hsa_circ_	-0.31861	0.174026
hsa_circ_	0.215101	0.174034
hsa_circ_	-0.57056	0.174036
hsa_circ_	-0.78295	0.174038
hsa_circ_	-0.20741	0.17404
hsa_circ_	0.614356	0.174041
hsa_circ_	0.339725	0.174057
hsa_circ_	-0.90278	0.174062
hsa_circ_	-0.26181	0.174069
hsa_circ_	0.474286	0.174074
hsa_circ_	0.233212	0.174075
hsa_circ_	0.51526	0.174076
hsa_circ_	0.305794	0.174076
hsa_circ_	-0.29315	0.174083
hsa_circ_	0.300604	0.174091
hsa_circ_	0.351776	0.174092
hsa_circ_	0.565403	0.17411
hsa_circ_	-0.18919	0.174117
hsa_circ_	-0.22345	0.174125
hsa_circ_	0.357791	0.17413
hsa_circ_	-0.41091	0.174141
hsa_circ_	0.194868	0.174146
hsa_circ_	0.435246	0.17415
hsa_circ_	0.363793	0.174152
hsa_circ_	-0.24755	0.174156
hsa_circ_	0.282293	0.17416
hsa_circ_	-0.25511	0.174163
hsa_circ_	0.267143	0.174166
hsa_circ_	0.259573	0.17417
hsa_circ_	0.851277	0.174174
hsa_circ_	0.285913	0.174178
hsa_circ_	-0.23347	0.174212
hsa_circ_	0.33656	0.174215
hsa_circ_	-0.74727	0.174232

hsa_circ_	-0.21136	0.174234
hsa_circ_	-0.29124	0.174236
hsa_circ_	-0.34234	0.174237
hsa_circ_	-0.34084	0.174242
hsa_circ_	0.371658	0.174242
hsa_circ_	0.240388	0.174244
hsa_circ_	0.359983	0.174253
hsa_circ_	0.212705	0.174267
hsa_circ_	-0.4054	0.174273
hsa_circ_	-1.25107	0.174276
hsa_circ_	-0.33679	0.174281
hsa_circ_	-0.42874	0.174287
hsa_circ_	-0.25909	0.174301
hsa_circ_	-0.22801	0.17431
hsa_circ_	-0.28182	0.174312
hsa_circ_	0.231187	0.17432
hsa_circ_	0.22584	0.174321
hsa_circ_	0.331725	0.174338
hsa_circ_	-0.20968	0.17435
hsa_circ_	0.231885	0.174367
hsa_circ_	0.338479	0.174368
hsa_circ_	0.211219	0.174372
hsa_circ_	-0.26215	0.174397
hsa_circ_	-0.23214	0.174406
hsa_circ_	-0.20991	0.174423
hsa_circ_	0.278374	0.174428
hsa_circ_	-0.2127	0.174442
hsa_circ_	-0.21944	0.174449
hsa_circ_	-0.21885	0.174459
hsa_circ_	-0.94556	0.174474
hsa_circ_	0.77632	0.174483
hsa_circ_	0.811893	0.174483
hsa_circ_	0.984765	0.174489
hsa_circ_	0.350259	0.17451
hsa_circ_	-0.26295	0.174514
hsa_circ_	-0.24555	0.174514
hsa_circ_	0.241353	0.174517
hsa_circ_	-0.24268	0.174522
hsa_circ_	-0.24505	0.174526
hsa_circ_	-0.7449	0.17453
hsa_circ_	0.364804	0.17453
hsa_circ_	0.358154	0.174536
hsa_circ_	-0.38912	0.174537
hsa_circ_	0.344877	0.174562
hsa_circ_	0.266054	0.174569
hsa_circ_	0.198366	0.174571
hsa_circ_	-0.58355	0.17459
hsa_circ_	-0.39696	0.174596
hsa_circ_	0.343905	0.174601
hsa_circ_	0.267875	0.174611
hsa_circ_	-0.24869	0.174618
hsa_circ_	-0.75269	0.174619
hsa_circ_	0.445813	0.174619
hsa_circ_	0.223057	0.174625

hsa_circ_	-0.50605	0.174626
hsa_circ_	0.301907	0.17463
hsa_circ_	-0.25529	0.174634
hsa_circ_	-0.21749	0.174638
hsa_circ_	-0.23983	0.174643
hsa_circ_	0.88215	0.174662
hsa_circ_	0.242355	0.174665
hsa_circ_	0.262033	0.174669
hsa_circ_	0.34176	0.174682
hsa_circ_	-0.20999	0.174702
hsa_circ_	-0.23195	0.174704
hsa_circ_	-0.34859	0.174704
hsa_circ_	0.520159	0.17472
hsa_circ_	0.215366	0.174745
hsa_circ_	-0.31215	0.174763
hsa_circ_	0.417916	0.17477
hsa_circ_	0.449724	0.17479
hsa_circ_	-0.30224	0.174798
hsa_circ_	0.884345	0.174806
hsa_circ_	-0.29287	0.174811
hsa_circ_	0.491746	0.174821
hsa_circ_	-0.60459	0.174828
hsa_circ_	-0.35502	0.174841
hsa_circ_	0.218598	0.174846
hsa_circ_	-0.26478	0.174853
hsa_circ_	0.34811	0.174855
hsa_circ_	-0.25658	0.174857
hsa_circ_	0.444546	0.17486
hsa_circ_	0.312135	0.174861
hsa_circ_	-0.21343	0.174861
hsa_circ_	-1.43811	0.174862
hsa_circ_	0.277749	0.17487
hsa_circ_	-0.31047	0.17487
hsa_circ_	-1.52058	0.174875
hsa_circ_	1.119199	0.174892
hsa_circ_	0.257454	0.174905
hsa_circ_	0.381266	0.174906
hsa_circ_	0.273499	0.174908
hsa_circ_	0.42549	0.174909
hsa_circ_	0.240056	0.174909
hsa_circ_	-0.25209	0.174921
hsa_circ_	-0.34381	0.174923
hsa_circ_	-0.70511	0.174947
hsa_circ_	0.653544	0.174948
hsa_circ_	-0.24699	0.174951
hsa_circ_	0.862672	0.174958
hsa_circ_	-0.22906	0.174959
hsa_circ_	-1.02684	0.174968
hsa_circ_	0.954334	0.174972
hsa_circ_	0.220349	0.174997
hsa_circ_	-0.18672	0.174999
hsa_circ_	0.348816	0.175025
hsa_circ_	-0.20601	0.175025
hsa_circ_	-0.22807	0.175033

hsa_circ_	-0.27736	0.175041
hsa_circ_	0.454884	0.175052
hsa_circ_	-0.49675	0.175053
hsa_circ_	0.215089	0.175057
hsa_circ_	0.364515	0.17506
hsa_circ_	-0.30291	0.175062
hsa_circ_	0.366698	0.175072
hsa_circ_	-0.27777	0.175075
hsa_circ_	0.37915	0.175098
hsa_circ_	-0.32057	0.175099
hsa_circ_	-0.45179	0.1751
hsa_circ_	0.494141	0.175116
hsa_circ_	-0.228	0.175124
hsa_circ_	-0.25797	0.175126
hsa_circ_	-0.32691	0.175135
hsa_circ_	-0.20982	0.175153
hsa_circ_	0.291165	0.175161
hsa_circ_	0.301565	0.175167
hsa_circ_	0.352206	0.175168
hsa_circ_	-0.39711	0.175178
hsa_circ_	-0.20306	0.175184
hsa_circ_	0.508077	0.175186
hsa_circ_	0.258712	0.17519
hsa_circ_	1.128825	0.175194
hsa_circ_	-0.70025	0.175197
hsa_circ_	-0.21952	0.175222
hsa_circ_	-0.20147	0.175227
hsa_circ_	-0.2598	0.175237
hsa_circ_	-0.26482	0.175237
hsa_circ_	0.664798	0.17524
hsa_circ_	-0.22509	0.175246
hsa_circ_	-0.29659	0.175252
hsa_circ_	0.439239	0.175255
hsa_circ_	-0.38117	0.175261
hsa_circ_	0.26782	0.175284
hsa_circ_	-1.00738	0.175285
hsa_circ_	-0.26095	0.175285
hsa_circ_	0.258119	0.175292
hsa_circ_	-0.2873	0.175299
hsa_circ_	0.217079	0.175305
hsa_circ_	-0.48632	0.175306
hsa_circ_	-0.34888	0.175316
hsa_circ_	-0.5189	0.175332
hsa_circ_	0.31604	0.17534
hsa_circ_	0.310903	0.175346
hsa_circ_	-0.48931	0.175353
hsa_circ_	0.234918	0.175363
hsa_circ_	-0.18161	0.175371
hsa_circ_	-0.24514	0.175387
hsa_circ_	0.648657	0.175391
hsa_circ_	-0.21868	0.175392
hsa_circ_	0.310267	0.175401
hsa_circ_	-0.3561	0.175402
hsa_circ_	-0.85551	0.175406

hsa_circ_	0.374098	0.175413
hsa_circ_	-0.1996	0.175421
hsa_circ_	-0.41639	0.175421
hsa_circ_	-0.34248	0.175421
hsa_circ_	0.313428	0.175437
hsa_circ_	-0.36141	0.17544
hsa_circ_	0.36383	0.175444
hsa_circ_	0.584219	0.175452
hsa_circ_	-0.36313	0.175453
hsa_circ_	-0.22325	0.175459
hsa_circ_	0.788976	0.175463
hsa_circ_	0.889541	0.175469
hsa_circ_	0.493017	0.175485
hsa_circ_	-0.21936	0.175488
hsa_circ_	0.276655	0.175494
hsa_circ_	-0.19951	0.175506
hsa_circ_	-0.85856	0.175519
hsa_circ_	-0.18902	0.175525
hsa_circ_	0.221025	0.175547
hsa_circ_	0.470877	0.175582
hsa_circ_	0.549916	0.175594
hsa_circ_	-0.24085	0.175594
hsa_circ_	0.571665	0.175595
hsa_circ_	-0.24829	0.175596
hsa_circ_	-0.39087	0.175603
hsa_circ_	0.224506	0.17561
hsa_circ_	0.248027	0.175617
hsa_circ_	0.253923	0.17562
hsa_circ_	-0.26227	0.175638
hsa_circ_	-1.12793	0.175644
hsa_circ_	-0.32438	0.175656
hsa_circ_	0.295786	0.175677
hsa_circ_	0.359233	0.175686
hsa_circ_	-0.24404	0.175697
hsa_circ_	-0.18714	0.175715
hsa_circ_	-0.30416	0.175728
hsa_circ_	0.25448	0.175734
hsa_circ_	-0.41705	0.17574
hsa_circ_	-0.41731	0.175744
hsa_circ_	-0.72153	0.17575
hsa_circ_	0.329634	0.175754
hsa_circ_	-0.22847	0.175761
hsa_circ_	0.257643	0.175788
hsa_circ_	-0.26089	0.175801
hsa_circ_	-0.61531	0.175815
hsa_circ_	0.218942	0.175815
hsa_circ_	-0.49737	0.175821
hsa_circ_	0.729099	0.175823
hsa_circ_	0.25983	0.175831
hsa_circ_	-0.29855	0.175832
hsa_circ_	0.213358	0.175835
hsa_circ_	0.238936	0.175837
hsa_circ_	-0.38042	0.175855
hsa_circ_	0.295403	0.175871

hsa_circ_	-0.40626	0.175878
hsa_circ_	0.717833	0.175887
hsa_circ_	0.263996	0.175901
hsa_circ_	0.623325	0.175907
hsa_circ_	0.196669	0.175908
hsa_circ_	0.278053	0.17592
hsa_circ_	0.35537	0.175923
hsa_circ_	0.245585	0.175928
hsa_circ_	0.34339	0.175945
hsa_circ_	-0.48678	0.175945
hsa_circ_	-0.21721	0.175955
hsa_circ_	-0.33187	0.175961
hsa_circ_	-0.59619	0.17597
hsa_circ_	0.478684	0.175971
hsa_circ_	-0.45598	0.175992
hsa_circ_	-0.47206	0.176042
hsa_circ_	-0.48437	0.176044
hsa_circ_	-0.22761	0.176046
hsa_circ_	-0.25985	0.176053
hsa_circ_	0.471251	0.176053
hsa_circ_	0.209636	0.176055
hsa_circ_	-0.38655	0.176064
hsa_circ_	0.208799	0.176103
hsa_circ_	-0.36778	0.17611
hsa_circ_	-0.25098	0.176119
hsa_circ_	0.212048	0.176123
hsa_circ_	0.575882	0.176131
hsa_circ_	0.252112	0.176132
hsa_circ_	-0.30872	0.176132
hsa_circ_	-0.24619	0.176148
hsa_circ_	-0.35499	0.176156
hsa_circ_	0.336223	0.176162
hsa_circ_	0.283688	0.176182
hsa_circ_	-0.27246	0.176185
hsa_circ_	0.326887	0.176207
hsa_circ_	0.195053	0.176207
hsa_circ_	-0.29256	0.176213
hsa_circ_	0.477887	0.176242
hsa_circ_	0.403809	0.176243
hsa_circ_	0.344627	0.176243
hsa_circ_	-0.22363	0.176245
hsa_circ_	-0.2788	0.17625
hsa_circ_	-0.28359	0.176257
hsa_circ_	-0.21905	0.176274
hsa_circ_	-0.22388	0.176291
hsa_circ_	-0.19223	0.176294
hsa_circ_	-0.20483	0.176308
hsa_circ_	-0.27301	0.17631
hsa_circ_	-0.18927	0.176319
hsa_circ_	-0.23406	0.176319
hsa_circ_	0.497079	0.176331
hsa_circ_	0.206355	0.176338
hsa_circ_	0.347384	0.176342
hsa_circ_	-0.26668	0.176367

hsa_circ_	-0.28776	0.176376
hsa_circ_	-0.33731	0.17638
hsa_circ_	-0.19836	0.17638
hsa_circ_	0.288321	0.176384
hsa_circ_	-0.33846	0.176386
hsa_circ_	-0.46447	0.17639
hsa_circ_	-0.2076	0.176406
hsa_circ_	-0.38244	0.176438
hsa_circ_	-0.25899	0.176449
hsa_circ_	-0.20633	0.176456
hsa_circ_	-0.25615	0.17652
hsa_circ_	0.284914	0.176523
hsa_circ_	0.244604	0.176538
hsa_circ_	-0.21456	0.176544
hsa_circ_	-0.27895	0.176549
hsa_circ_	-0.24854	0.17656
hsa_circ_	-0.27738	0.176561
hsa_circ_	0.436288	0.176568
hsa_circ_	0.219343	0.176575
hsa_circ_	0.247792	0.176575
hsa_circ_	0.199099	0.176591
hsa_circ_	0.747216	0.176595
hsa_circ_	0.211562	0.176595
hsa_circ_	-0.20646	0.176596
hsa_circ_	0.516605	0.176603
hsa_circ_	-0.36085	0.176636
hsa_circ_	-0.32402	0.176637
hsa_circ_	-0.24234	0.176642
hsa_circ_	-0.20876	0.176672
hsa_circ_	0.368452	0.176691
hsa_circ_	-0.19959	0.176691
hsa_circ_	-0.18815	0.176706
hsa_circ_	-0.57259	0.176707
hsa_circ_	-0.34396	0.176724
hsa_circ_	0.275682	0.17673
hsa_circ_	-0.4421	0.176732
hsa_circ_	-0.94319	0.176736
hsa_circ_	-0.29836	0.176742
hsa_circ_	-0.22872	0.176765
hsa_circ_	0.267392	0.176775
hsa_circ_	0.33808	0.176777
hsa_circ_	0.356712	0.176804
hsa_circ_	0.251941	0.176809
hsa_circ_	0.198853	0.176814
hsa_circ_	0.302791	0.176818
hsa_circ_	0.272688	0.176819
hsa_circ_	-0.35505	0.176833
hsa_circ_	0.373574	0.176843
hsa_circ_	-0.22707	0.17685
hsa_circ_	0.29893	0.176852
hsa_circ_	0.197294	0.176861
hsa_circ_	0.775084	0.176869
hsa_circ_	0.648996	0.176869
hsa_circ_	0.273804	0.176873

hsa_circ_	-0.25335	0.176877
hsa_circ_	1.115244	0.1769
hsa_circ_	-0.46865	0.176913
hsa_circ_	-0.2486	0.176914
hsa_circ_	0.327763	0.17692
hsa_circ_	-0.2313	0.176921
hsa_circ_	-0.57732	0.176935
hsa_circ_	-0.20561	0.176935
hsa_circ_	0.258501	0.176936
hsa_circ_	0.274062	0.176942
hsa_circ_	-0.44568	0.176966
hsa_circ_	0.794462	0.176967
hsa_circ_	-0.36579	0.176968
hsa_circ_	-0.30073	0.176986
hsa_circ_	-0.20848	0.177
hsa_circ_	-0.69129	0.177014
hsa_circ_	0.208421	0.177018
hsa_circ_	-0.42535	0.177019
hsa_circ_	-0.31894	0.177021
hsa_circ_	-0.211	0.177027
hsa_circ_	-0.22999	0.177036
hsa_circ_	-0.62368	0.17704
hsa_circ_	-0.29815	0.177068
hsa_circ_	-0.41338	0.177089
hsa_circ_	-0.23639	0.177102
hsa_circ_	-0.34198	0.17711
hsa_circ_	-0.19767	0.177117
hsa_circ_	-0.52576	0.177121
hsa_circ_	0.737652	0.177146
hsa_circ_	-0.46542	0.177147
hsa_circ_	-0.26695	0.177164
hsa_circ_	0.819224	0.17719
hsa_circ_	-0.41272	0.177194
hsa_circ_	0.442211	0.177201
hsa_circ_	0.721271	0.177239
hsa_circ_	-0.20283	0.177241
hsa_circ_	-0.54398	0.177252
hsa_circ_	0.20349	0.177252
hsa_circ_	-0.20661	0.177258
hsa_circ_	-0.35903	0.177259
hsa_circ_	-0.32473	0.177271
hsa_circ_	-0.35733	0.177274
hsa_circ_	-0.20129	0.177277
hsa_circ_	-0.75252	0.177281
hsa_circ_	0.197029	0.177283
hsa_circ_	-0.33489	0.177297
hsa_circ_	0.216226	0.177318
hsa_circ_	0.577554	0.177321
hsa_circ_	-0.3507	0.177333
hsa_circ_	0.276996	0.177337
hsa_circ_	0.303205	0.177338
hsa_circ_	1.266148	0.177344
hsa_circ_	-0.77987	0.177345
hsa_circ_	-0.7119	0.177348

hsa_circ_	0.270233	0.177352
hsa_circ_	0.279451	0.177353
hsa_circ_	-0.58535	0.177355
hsa_circ_	-0.31815	0.17737
hsa_circ_	-0.42513	0.177372
hsa_circ_	-0.37311	0.177391
hsa_circ_	0.235404	0.177397
hsa_circ_	-0.26317	0.177399
hsa_circ_	0.310489	0.177406
hsa_circ_	0.669668	0.17741
hsa_circ_	-0.18846	0.177419
hsa_circ_	0.241137	0.177444
hsa_circ_	0.193777	0.177446
hsa_circ_	-0.40607	0.177453
hsa_circ_	0.610062	0.177472
hsa_circ_	-0.32808	0.177483
hsa_circ_	-0.23722	0.177484
hsa_circ_	-0.31169	0.1775
hsa_circ_	0.189588	0.17751
hsa_circ_	0.297861	0.177517
hsa_circ_	-0.47341	0.177538
hsa_circ_	-0.29163	0.177539
hsa_circ_	0.204039	0.177561
hsa_circ_	1.501107	0.177564
hsa_circ_	-0.6349	0.177568
hsa_circ_	0.244443	0.17757
hsa_circ_	0.292804	0.177575
hsa_circ_	0.517439	0.177577
hsa_circ_	0.300677	0.177589
hsa_circ_	-0.469	0.177596
hsa_circ_	-0.22168	0.177597
hsa_circ_	-0.61554	0.17761
hsa_circ_	-0.25867	0.177617
hsa_circ_	-0.36318	0.177622
hsa_circ_	-0.26672	0.177622
hsa_circ_	-0.40192	0.177636
hsa_circ_	-0.49177	0.177637
hsa_circ_	0.280665	0.177648
hsa_circ_	-0.5556	0.177651
hsa_circ_	0.332381	0.177655
hsa_circ_	0.381781	0.177661
hsa_circ_	0.357531	0.177669
hsa_circ_	-0.30396	0.177691
hsa_circ_	-0.49311	0.177695
hsa_circ_	0.218877	0.177701
hsa_circ_	0.25217	0.177702
hsa_circ_	0.330507	0.177724
hsa_circ_	-0.28626	0.17773
hsa_circ_	0.234676	0.177739
hsa_circ_	-0.43304	0.177747
hsa_circ_	-0.25251	0.17777
hsa_circ_	-0.2136	0.177772
hsa_circ_	0.288974	0.17778
hsa_circ_	0.267161	0.177786

hsa_circ_	-0.40824	0.177793
hsa_circ_	0.421274	0.177802
hsa_circ_	0.394392	0.177803
hsa_circ_	0.219786	0.177805
hsa_circ_	-0.30209	0.177811
hsa_circ_	-0.28079	0.177817
hsa_circ_	-0.23807	0.17782
hsa_circ_	0.236845	0.177851
hsa_circ_	1.11273	0.177879
hsa_circ_	-0.22833	0.177911
hsa_circ_	-0.68806	0.177913
hsa_circ_	0.500693	0.177925
hsa_circ_	0.394331	0.177928
hsa_circ_	-0.64192	0.177942
hsa_circ_	1.085219	0.177947
hsa_circ_	0.263502	0.17795
hsa_circ_	0.784434	0.177954
hsa_circ_	0.257019	0.177958
hsa_circ_	-0.80202	0.177962
hsa_circ_	-0.24009	0.177962
hsa_circ_	-0.27721	0.177973
hsa_circ_	-0.21939	0.178004
hsa_circ_	0.302978	0.178007
hsa_circ_	-0.29878	0.178011
hsa_circ_	0.500438	0.178012
hsa_circ_	0.414999	0.178017
hsa_circ_	0.25646	0.178019
hsa_circ_	-0.69762	0.178027
hsa_circ_	-0.32018	0.17803
hsa_circ_	0.35097	0.17803
hsa_circ_	-0.20488	0.178056
hsa_circ_	0.26942	0.178083
hsa_circ_	-0.33333	0.178095
hsa_circ_	-0.30869	0.178097
hsa_circ_	-0.69954	0.178104
hsa_circ_	0.564775	0.178106
hsa_circ_	-0.34863	0.178115
hsa_circ_	0.221839	0.178131
hsa_circ_	0.229975	0.178141
hsa_circ_	-0.61708	0.178162
hsa_circ_	0.254327	0.178173
hsa_circ_	0.401933	0.178177
hsa_circ_	-0.27953	0.17818
hsa_circ_	-0.2059	0.178181
hsa_circ_	0.675383	0.178187
hsa_circ_	0.298388	0.178188
hsa_circ_	-0.27367	0.178188
hsa_circ_	0.303116	0.178201
hsa_circ_	-0.20806	0.178201
hsa_circ_	0.347677	0.178204
hsa_circ_	0.290558	0.178205
hsa_circ_	0.373152	0.178209
hsa_circ_	-0.27647	0.178241
hsa_circ_	-0.5071	0.178245

hsa_circ_	-0.46515	0.178262
hsa_circ_	0.415678	0.178296
hsa_circ_	0.21036	0.178301
hsa_circ_	-0.74303	0.178302
hsa_circ_	0.226981	0.178306
hsa_circ_	0.207278	0.178307
hsa_circ_	-0.2392	0.178326
hsa_circ_	0.718115	0.178335
hsa_circ_	1.214697	0.178335
hsa_circ_	-0.20279	0.178367
hsa_circ_	0.19347	0.178369
hsa_circ_	0.22535	0.178372
hsa_circ_	0.206418	0.178395
hsa_circ_	0.529863	0.178428
hsa_circ_	0.949221	0.178455
hsa_circ_	0.239581	0.178472
hsa_circ_	-0.25123	0.178478
hsa_circ_	0.257692	0.178508
hsa_circ_	0.299189	0.17851
hsa_circ_	0.612508	0.17851
hsa_circ_	-0.45114	0.178523
hsa_circ_	0.406512	0.178524
hsa_circ_	-0.51385	0.178538
hsa_circ_	0.22042	0.178556
hsa_circ_	0.234934	0.178559
hsa_circ_	0.378801	0.178566
hsa_circ_	0.72171	0.178566
hsa_circ_	-0.35519	0.178567
hsa_circ_	-0.26364	0.178569
hsa_circ_	0.237808	0.178595
hsa_circ_	-0.19271	0.178596
hsa_circ_	-0.55761	0.178598
hsa_circ_	-0.29197	0.178598
hsa_circ_	0.518501	0.178607
hsa_circ_	-0.23371	0.178608
hsa_circ_	0.185874	0.178608
hsa_circ_	-0.36715	0.178613
hsa_circ_	0.367913	0.178623
hsa_circ_	0.499046	0.178625
hsa_circ_	0.393085	0.178629
hsa_circ_	0.190922	0.17863
hsa_circ_	0.42531	0.178635
hsa_circ_	0.436341	0.178638
hsa_circ_	0.21301	0.178655
hsa_circ_	-0.52199	0.178658
hsa_circ_	0.266871	0.17868
hsa_circ_	0.334069	0.17869
hsa_circ_	-0.37936	0.1787
hsa_circ_	-0.30038	0.178702
hsa_circ_	-0.20313	0.178705
hsa_circ_	-0.28323	0.178713
hsa_circ_	-0.41977	0.178714
hsa_circ_	-0.80096	0.178724
hsa_circ_	-0.28832	0.178729

hsa_circ_	0.260281	0.178738
hsa_circ_	-0.74766	0.178745
hsa_circ_	-0.22909	0.178748
hsa_circ_	0.271605	0.178751
hsa_circ_	0.562781	0.178772
hsa_circ_	0.334732	0.178776
hsa_circ_	-0.20593	0.178794
hsa_circ_	0.360865	0.178799
hsa_circ_	0.562686	0.178831
hsa_circ_	-0.26335	0.178833
hsa_circ_	0.307601	0.178836
hsa_circ_	0.22078	0.178848
hsa_circ_	0.235019	0.178854
hsa_circ_	0.343822	0.178856
hsa_circ_	-0.3557	0.178865
hsa_circ_	0.453524	0.178867
hsa_circ_	0.220694	0.178876
hsa_circ_	0.219715	0.17888
hsa_circ_	-0.25727	0.178883
hsa_circ_	0.61104	0.178888
hsa_circ_	-0.29482	0.178889
hsa_circ_	-0.18361	0.178891
hsa_circ_	-0.3521	0.178899
hsa_circ_	-0.18966	0.178905
hsa_circ_	0.575967	0.178909
hsa_circ_	0.214593	0.17891
hsa_circ_	0.255611	0.178927
hsa_circ_	-0.61836	0.178928
hsa_circ_	-0.28744	0.178935
hsa_circ_	0.701791	0.178948
hsa_circ_	-0.32816	0.178949
hsa_circ_	-0.58187	0.178956
hsa_circ_	-0.65299	0.178978
hsa_circ_	-0.93373	0.178989
hsa_circ_	-0.19014	0.179001
hsa_circ_	-0.41917	0.179006
hsa_circ_	-0.32506	0.179008
hsa_circ_	-0.39957	0.179008
hsa_circ_	-0.20635	0.179009
hsa_circ_	-0.75891	0.179012
hsa_circ_	0.459567	0.179025
hsa_circ_	-0.70729	0.179031
hsa_circ_	-0.89607	0.179044
hsa_circ_	-0.20364	0.179053
hsa_circ_	0.300704	0.179056
hsa_circ_	0.26903	0.179062
hsa_circ_	-0.5423	0.179067
hsa_circ_	-0.29419	0.179069
hsa_circ_	-0.24975	0.179081
hsa_circ_	-0.33834	0.179082
hsa_circ_	-0.2972	0.179091
hsa_circ_	0.378652	0.179098
hsa_circ_	-0.59217	0.179105
hsa_circ_	-0.25087	0.179111

hsa_circ_	-0.25623	0.17912
hsa_circ_	0.865146	0.179128
hsa_circ_	0.728812	0.179131
hsa_circ_	-0.50861	0.179137
hsa_circ_	-0.24132	0.179139
hsa_circ_	0.335201	0.179141
hsa_circ_	-0.28128	0.179143
hsa_circ_	-0.80909	0.179144
hsa_circ_	-0.31326	0.179152
hsa_circ_	-0.21282	0.179159
hsa_circ_	-0.21174	0.179166
hsa_circ_	-0.30229	0.179192
hsa_circ_	-0.363	0.179196
hsa_circ_	-0.23386	0.179197
hsa_circ_	-0.51725	0.179203
hsa_circ_	0.300786	0.179211
hsa_circ_	0.366886	0.179232
hsa_circ_	-0.39839	0.179249
hsa_circ_	0.294757	0.179263
hsa_circ_	0.447697	0.179268
hsa_circ_	-0.307	0.17927
hsa_circ_	-0.30804	0.179271
hsa_circ_	0.212645	0.179278
hsa_circ_	-0.31873	0.179279
hsa_circ_	-0.24756	0.179291
hsa_circ_	0.296307	0.179295
hsa_circ_	-0.21065	0.179296
hsa_circ_	0.228728	0.179298
hsa_circ_	-0.33862	0.179299
hsa_circ_	-0.43657	0.179304
hsa_circ_	-0.27218	0.179307
hsa_circ_	0.783945	0.179315
hsa_circ_	0.269498	0.17934
hsa_circ_	-0.19892	0.179344
hsa_circ_	-0.24062	0.179351
hsa_circ_	0.365181	0.179351
hsa_circ_	-0.83815	0.179353
hsa_circ_	-0.82173	0.179365
hsa_circ_	-0.26235	0.17937
hsa_circ_	-0.23914	0.179374
hsa_circ_	-0.80892	0.17939
hsa_circ_	0.927344	0.179395
hsa_circ_	0.544595	0.179403
hsa_circ_	-0.22947	0.179408
hsa_circ_	0.780053	0.17941
hsa_circ_	-0.44123	0.179412
hsa_circ_	-0.25699	0.179427
hsa_circ_	0.278994	0.179437
hsa_circ_	-0.90423	0.179458
hsa_circ_	0.21576	0.179463
hsa_circ_	0.272455	0.179478
hsa_circ_	0.327882	0.1795
hsa_circ_	-0.7956	0.179513
hsa_circ_	0.610408	0.179516

hsa_circ_	-0.3866	0.179519
hsa_circ_	-0.74653	0.179531
hsa_circ_	-0.22619	0.179534
hsa_circ_	-0.90988	0.179534
hsa_circ_	0.251206	0.179553
hsa_circ_	0.223425	0.179575
hsa_circ_	0.591459	0.179581
hsa_circ_	0.245623	0.179589
hsa_circ_	-0.29395	0.17959
hsa_circ_	-0.35342	0.179598
hsa_circ_	0.757015	0.179605
hsa_circ_	-0.24583	0.179625
hsa_circ_	0.207629	0.179647
hsa_circ_	0.310986	0.179665
hsa_circ_	-0.38342	0.179679
hsa_circ_	0.251392	0.17968
hsa_circ_	0.372739	0.179696
hsa_circ_	-0.55138	0.179696
hsa_circ_	-0.24799	0.179706
hsa_circ_	-0.19643	0.179708
hsa_circ_	0.322444	0.179716
hsa_circ_	-0.73823	0.179725
hsa_circ_	-0.29957	0.17973
hsa_circ_	0.256041	0.179741
hsa_circ_	0.328407	0.179744
hsa_circ_	0.28797	0.179745
hsa_circ_	0.34352	0.179759
hsa_circ_	-0.27308	0.179761
hsa_circ_	0.361708	0.179763
hsa_circ_	-0.35066	0.179769
hsa_circ_	-0.25801	0.179782
hsa_circ_	-0.62468	0.179804
hsa_circ_	0.301783	0.179816
hsa_circ_	0.360982	0.179833
hsa_circ_	-0.21336	0.179834
hsa_circ_	0.358775	0.179836
hsa_circ_	-0.41645	0.179842
hsa_circ_	-0.32389	0.179847
hsa_circ_	-0.22485	0.179847
hsa_circ_	0.657361	0.179856
hsa_circ_	-0.34946	0.179862
hsa_circ_	0.255354	0.17988
hsa_circ_	0.360645	0.179881
hsa_circ_	0.352312	0.179897
hsa_circ_	-0.19716	0.179911
hsa_circ_	0.455238	0.179933
hsa_circ_	-1.55004	0.179934
hsa_circ_	0.571169	0.179939
hsa_circ_	-0.28341	0.179945
hsa_circ_	-0.196	0.17995
hsa_circ_	-0.32745	0.179972
hsa_circ_	0.296676	0.179985
hsa_circ_	0.790786	0.179991
hsa_circ_	-0.37758	0.179996

hsa_circ_	-0.55374	0.180024
hsa_circ_	-0.28466	0.180054
hsa_circ_	0.269478	0.180054
hsa_circ_	-0.27766	0.180063
hsa_circ_	0.34342	0.180081
hsa_circ_	-0.41284	0.180086
hsa_circ_	-0.42781	0.180091
hsa_circ_	0.430443	0.180091
hsa_circ_	-0.19841	0.180096
hsa_circ_	0.202751	0.180099
hsa_circ_	-0.43318	0.180112
hsa_circ_	-0.30715	0.180114
hsa_circ_	-0.36963	0.180114
hsa_circ_	-0.24293	0.180116
hsa_circ_	1.105743	0.180121
hsa_circ_	-0.56072	0.180168
hsa_circ_	0.3309	0.180184
hsa_circ_	-0.18269	0.180189
hsa_circ_	0.268592	0.180203
hsa_circ_	-0.65126	0.180212
hsa_circ_	0.534755	0.180222
hsa_circ_	-0.3185	0.180237
hsa_circ_	-0.2273	0.180244
hsa_circ_	0.253771	0.180246
hsa_circ_	0.336909	0.180272
hsa_circ_	-0.44271	0.180288
hsa_circ_	0.220101	0.180302
hsa_circ_	0.436677	0.180312
hsa_circ_	-0.83979	0.180327
hsa_circ_	0.345739	0.180342
hsa_circ_	-0.22704	0.180362
hsa_circ_	0.314498	0.180371
hsa_circ_	-0.21262	0.180374
hsa_circ_	0.228533	0.18038
hsa_circ_	-0.27966	0.180393
hsa_circ_	-0.38892	0.180406
hsa_circ_	0.20972	0.180413
hsa_circ_	0.609065	0.180413
hsa_circ_	-0.3035	0.180422
hsa_circ_	-0.54148	0.180436
hsa_circ_	0.501484	0.180437
hsa_circ_	-0.25566	0.180445
hsa_circ_	-0.30871	0.180454
hsa_circ_	-0.25579	0.180467
hsa_circ_	0.633257	0.180485
hsa_circ_	-0.35231	0.180485
hsa_circ_	0.477042	0.180486
hsa_circ_	-0.64849	0.180496
hsa_circ_	0.358114	0.180506
hsa_circ_	0.640054	0.180531
hsa_circ_	0.29118	0.180537
hsa_circ_	0.20186	0.180544
hsa_circ_	-0.32255	0.18063
hsa_circ_	-0.68469	0.180646

hsa_circ_	0.403194	0.180647
hsa_circ_	-0.18858	0.180668
hsa_circ_	-0.21902	0.180702
hsa_circ_	0.619926	0.180703
hsa_circ_	0.273825	0.180716
hsa_circ_	-0.22164	0.18072
hsa_circ_	0.259688	0.180727
hsa_circ_	0.271043	0.180744
hsa_circ_	0.398185	0.180752
hsa_circ_	-0.59755	0.180757
hsa_circ_	0.198793	0.180764
hsa_circ_	0.386688	0.18077
hsa_circ_	0.241832	0.180772
hsa_circ_	0.243481	0.180795
hsa_circ_	-0.72595	0.180841
hsa_circ_	0.58269	0.180849
hsa_circ_	0.198136	0.180867
hsa_circ_	-0.45258	0.180874
hsa_circ_	1.065863	0.180887
hsa_circ_	0.317903	0.180896
hsa_circ_	0.370428	0.1809
hsa_circ_	-0.34915	0.180901
hsa_circ_	-0.70625	0.180905
hsa_circ_	0.421549	0.180911
hsa_circ_	-0.19259	0.180922
hsa_circ_	0.20765	0.180936
hsa_circ_	0.298453	0.180942
hsa_circ_	-0.40239	0.180944
hsa_circ_	-0.23776	0.18095
hsa_circ_	-0.22337	0.180958
hsa_circ_	0.244704	0.180965
hsa_circ_	-0.38694	0.180973
hsa_circ_	-0.22761	0.181004
hsa_circ_	0.200811	0.181006
hsa_circ_	-0.75986	0.181006
hsa_circ_	-0.23458	0.181007
hsa_circ_	0.294809	0.181015
hsa_circ_	-0.64747	0.18102
hsa_circ_	0.193996	0.181023
hsa_circ_	-0.27392	0.181035
hsa_circ_	0.304628	0.181036
hsa_circ_	0.904094	0.181037
hsa_circ_	0.448383	0.181058
hsa_circ_	0.364651	0.181061
hsa_circ_	0.353255	0.181061
hsa_circ_	1.41625	0.181067
hsa_circ_	0.329987	0.181088
hsa_circ_	0.998558	0.181098
hsa_circ_	-0.50221	0.181098
hsa_circ_	0.451963	0.1811
hsa_circ_	-0.22914	0.181103
hsa_circ_	-0.21585	0.18113
hsa_circ_	0.594682	0.181134
hsa_circ_	-0.26952	0.181139

hsa_circ_	-0.73887	0.18115
hsa_circ_	0.332594	0.181152
hsa_circ_	0.237755	0.181168
hsa_circ_	0.222898	0.18117
hsa_circ_	0.433988	0.181171
hsa_circ_	-0.25123	0.181193
hsa_circ_	-0.23811	0.181201
hsa_circ_	0.50948	0.181206
hsa_circ_	-0.24799	0.181206
hsa_circ_	0.236888	0.181211
hsa_circ_	-0.23261	0.181213
hsa_circ_	-1.88312	0.181214
hsa_circ_	1.170993	0.181216
hsa_circ_	0.94837	0.181226
hsa_circ_	-0.32439	0.181244
hsa_circ_	-0.40412	0.181262
hsa_circ_	0.340272	0.181268
hsa_circ_	0.313007	0.181306
hsa_circ_	-0.21395	0.181316
hsa_circ_	-0.21707	0.18132
hsa_circ_	-0.57779	0.181322
hsa_circ_	0.307167	0.181349
hsa_circ_	-0.23937	0.181361
hsa_circ_	-0.23218	0.181364
hsa_circ_	-0.4692	0.18137
hsa_circ_	0.382645	0.181403
hsa_circ_	0.938361	0.181419
hsa_circ_	-0.38156	0.18143
hsa_circ_	-0.23784	0.181431
hsa_circ_	1.210749	0.181431
hsa_circ_	-0.76968	0.181438
hsa_circ_	0.546904	0.181442
hsa_circ_	0.224604	0.181454
hsa_circ_	-0.22342	0.181462
hsa_circ_	-0.39789	0.18147
hsa_circ_	-0.36478	0.181473
hsa_circ_	-0.22639	0.181483
hsa_circ_	-0.21341	0.181488
hsa_circ_	0.795617	0.181529
hsa_circ_	0.217706	0.181529
hsa_circ_	-0.2305	0.181531
hsa_circ_	0.278316	0.18155
hsa_circ_	-0.38449	0.181552
hsa_circ_	-0.37164	0.181568
hsa_circ_	-0.20582	0.181575
hsa_circ_	-0.21623	0.181594
hsa_circ_	0.206724	0.181596
hsa_circ_	0.898956	0.181597
hsa_circ_	-0.77236	0.181624
hsa_circ_	-0.39599	0.181634
hsa_circ_	0.250054	0.181635
hsa_circ_	0.228665	0.181649
hsa_circ_	0.256386	0.181661
hsa_circ_	0.23068	0.181672

hsa_circ_	0.208996	0.181683
hsa_circ_	0.816152	0.181702
hsa_circ_	-0.37572	0.181709
hsa_circ_	-0.27012	0.18171
hsa_circ_	0.27446	0.181728
hsa_circ_	0.253268	0.181732
hsa_circ_	0.407109	0.181738
hsa_circ_	0.723711	0.18174
hsa_circ_	-0.21759	0.18174
hsa_circ_	0.383488	0.181742
hsa_circ_	0.19493	0.181758
hsa_circ_	-0.21076	0.181759
hsa_circ_	0.440087	0.181767
hsa_circ_	-0.80954	0.181776
hsa_circ_	-0.29096	0.181791
hsa_circ_	-0.63459	0.181793
hsa_circ_	-0.35888	0.181804
hsa_circ_	0.698334	0.18181
hsa_circ_	0.426294	0.181816
hsa_circ_	-0.36794	0.181835
hsa_circ_	0.300199	0.181837
hsa_circ_	0.504019	0.181855
hsa_circ_	-0.21956	0.181859
hsa_circ_	-0.47669	0.181885
hsa_circ_	-0.90907	0.181886
hsa_circ_	-0.66638	0.18191
hsa_circ_	0.274578	0.181929
hsa_circ_	-0.7901	0.181945
hsa_circ_	0.265672	0.181949
hsa_circ_	-0.30904	0.181957
hsa_circ_	0.28368	0.181962
hsa_circ_	-0.20857	0.181976
hsa_circ_	0.267115	0.181977
hsa_circ_	-0.39588	0.181984
hsa_circ_	-0.1977	0.181998
hsa_circ_	-0.30553	0.182008
hsa_circ_	-0.27619	0.182022
hsa_circ_	0.190673	0.182024
hsa_circ_	-0.28967	0.182034
hsa_circ_	0.311023	0.182046
hsa_circ_	0.532656	0.182059
hsa_circ_	0.204839	0.182063
hsa_circ_	0.29973	0.182064
hsa_circ_	0.543133	0.182064
hsa_circ_	-0.32281	0.18207
hsa_circ_	-0.28635	0.182073
hsa_circ_	0.520412	0.182078
hsa_circ_	0.384189	0.182088
hsa_circ_	0.537664	0.1821
hsa_circ_	-0.30968	0.182129
hsa_circ_	-0.33669	0.18218
hsa_circ_	-0.50102	0.182193
hsa_circ_	0.565778	0.182203
hsa_circ_	0.484808	0.182203

hsa_circ_	0.208017	0.182222
hsa_circ_	0.295886	0.182236
hsa_circ_	-0.28308	0.182236
hsa_circ_	0.307681	0.18224
hsa_circ_	0.247983	0.182245
hsa_circ_	-0.29315	0.182249
hsa_circ_	-0.18558	0.182256
hsa_circ_	-0.27104	0.182257
hsa_circ_	-0.20188	0.182259
hsa_circ_	-0.31741	0.182271
hsa_circ_	0.231627	0.182286
hsa_circ_	0.18993	0.182305
hsa_circ_	0.282996	0.182311
hsa_circ_	0.725731	0.182363
hsa_circ_	0.280806	0.182366
hsa_circ_	-0.2085	0.182368
hsa_circ_	-0.7289	0.18239
hsa_circ_	0.342774	0.182394
hsa_circ_	0.182311	0.182405
hsa_circ_	1.094611	0.182435
hsa_circ_	-0.20494	0.182439
hsa_circ_	0.319173	0.182454
hsa_circ_	0.983053	0.182455
hsa_circ_	0.296202	0.182459
hsa_circ_	0.317691	0.182469
hsa_circ_	-0.39525	0.182475
hsa_circ_	0.372348	0.182487
hsa_circ_	0.498683	0.182492
hsa_circ_	-0.45729	0.182515
hsa_circ_	0.26658	0.182521
hsa_circ_	-0.29821	0.182534
hsa_circ_	-0.23072	0.182564
hsa_circ_	0.31365	0.182578
hsa_circ_	-0.43985	0.18258
hsa_circ_	0.757485	0.182584
hsa_circ_	-0.24359	0.182587
hsa_circ_	0.666641	0.182594
hsa_circ_	0.259854	0.182602
hsa_circ_	0.32703	0.182606
hsa_circ_	0.303293	0.182631
hsa_circ_	-0.65595	0.182638
hsa_circ_	-0.59813	0.182647
hsa_circ_	0.276427	0.182648
hsa_circ_	0.367875	0.182685
hsa_circ_	0.223546	0.182715
hsa_circ_	0.181044	0.182718
hsa_circ_	-0.52041	0.18273
hsa_circ_	0.182777	0.182739
hsa_circ_	-0.2147	0.182741
hsa_circ_	0.1818	0.182748
hsa_circ_	0.236228	0.182753
hsa_circ_	0.261212	0.182774
hsa_circ_	-0.27912	0.182774
hsa_circ_	-0.18889	0.182784

hsa_circ_	-0.205	0.182785
hsa_circ_	-0.39372	0.182786
hsa_circ_	0.861802	0.182814
hsa_circ_	0.439467	0.182815
hsa_circ_	0.325717	0.18282
hsa_circ_	-0.65146	0.182829
hsa_circ_	-0.43771	0.18283
hsa_circ_	-0.25205	0.182839
hsa_circ_	0.251769	0.182848
hsa_circ_	-0.23137	0.182849
hsa_circ_	0.258956	0.182849
hsa_circ_	0.235503	0.182851
hsa_circ_	0.241224	0.182855
hsa_circ_	0.552764	0.182871
hsa_circ_	0.221873	0.182875
hsa_circ_	0.198801	0.182879
hsa_circ_	-0.19309	0.182884
hsa_circ_	-0.27536	0.182896
hsa_circ_	0.366181	0.182896
hsa_circ_	0.276537	0.182899
hsa_circ_	-0.29472	0.182904
hsa_circ_	0.267808	0.18292
hsa_circ_	-0.33519	0.182927
hsa_circ_	0.969248	0.182936
hsa_circ_	-0.2387	0.182955
hsa_circ_	0.224099	0.18296
hsa_circ_	0.348497	0.182964
hsa_circ_	-0.18417	0.182967
hsa_circ_	0.488493	0.182985
hsa_circ_	-0.44345	0.182986
hsa_circ_	0.180443	0.183002
hsa_circ_	-0.40058	0.183006
hsa_circ_	0.236465	0.183025
hsa_circ_	0.283956	0.183032
hsa_circ_	-0.4662	0.183071
hsa_circ_	0.223551	0.183079
hsa_circ_	-0.45797	0.183085
hsa_circ_	-0.24441	0.183085
hsa_circ_	0.425083	0.183097
hsa_circ_	-0.51496	0.183102
hsa_circ_	0.202598	0.183112
hsa_circ_	-0.26255	0.183116
hsa_circ_	-0.22572	0.183121
hsa_circ_	-0.23654	0.183125
hsa_circ_	0.230039	0.18314
hsa_circ_	-0.18775	0.183163
hsa_circ_	-0.29713	0.183167
hsa_circ_	0.376989	0.183177
hsa_circ_	0.28455	0.18319
hsa_circ_	0.712215	0.183196
hsa_circ_	0.239279	0.183204
hsa_circ_	-0.45185	0.183232
hsa_circ_	-0.2105	0.183236
hsa_circ_	-0.38016	0.183241

hsa_circ_	0.453234	0.183274
hsa_circ_	-0.22526	0.183282
hsa_circ_	0.284978	0.183301
hsa_circ_	0.227922	0.183306
hsa_circ_	0.409233	0.18332
hsa_circ_	-0.2618	0.183323
hsa_circ_	0.510187	0.183328
hsa_circ_	-0.23619	0.18333
hsa_circ_	0.246934	0.183337
hsa_circ_	-0.18635	0.183344
hsa_circ_	0.610626	0.183355
hsa_circ_	0.249201	0.183358
hsa_circ_	0.275277	0.183371
hsa_circ_	-0.29501	0.183396
hsa_circ_	0.250183	0.183407
hsa_circ_	-0.46484	0.183416
hsa_circ_	-0.6008	0.183421
hsa_circ_	0.26481	0.18344
hsa_circ_	-0.58922	0.183446
hsa_circ_	-0.7217	0.183462
hsa_circ_	1.490512	0.183473
hsa_circ_	0.191418	0.183474
hsa_circ_	0.340764	0.183478
hsa_circ_	0.343153	0.183482
hsa_circ_	0.437114	0.183517
hsa_circ_	-0.21208	0.18352
hsa_circ_	-0.43691	0.183542
hsa_circ_	0.551247	0.183556
hsa_circ_	0.221342	0.183561
hsa_circ_	-0.25792	0.183561
hsa_circ_	-0.31765	0.18357
hsa_circ_	-0.19393	0.183574
hsa_circ_	-0.24344	0.183602
hsa_circ_	0.280961	0.183616
hsa_circ_	-0.19533	0.183619
hsa_circ_	-0.24646	0.183628
hsa_circ_	-0.53191	0.183642
hsa_circ_	-0.33426	0.18368
hsa_circ_	-0.37391	0.183682
hsa_circ_	0.264996	0.183687
hsa_circ_	0.223807	0.183688
hsa_circ_	0.186683	0.1837
hsa_circ_	0.2654	0.183709
hsa_circ_	-0.21743	0.183714
hsa_circ_	-0.2546	0.183736
hsa_circ_	-0.25068	0.183745
hsa_circ_	-0.23553	0.183745
hsa_circ_	-0.32404	0.18375
hsa_circ_	-0.26728	0.183754
hsa_circ_	-0.18672	0.183769
hsa_circ_	0.365872	0.183783
hsa_circ_	0.563107	0.183794
hsa_circ_	-0.29971	0.183797
hsa_circ_	0.291476	0.183798

hsa_circ_	0.63492	0.1838
hsa_circ_	-0.2816	0.183813
hsa_circ_	0.258873	0.18382
hsa_circ_	0.830678	0.183834
hsa_circ_	-0.704	0.183842
hsa_circ_	-0.51967	0.18385
hsa_circ_	-0.78359	0.183859
hsa_circ_	0.272068	0.183868
hsa_circ_	0.337703	0.18387
hsa_circ_	0.258657	0.183878
hsa_circ_	-0.21722	0.183888
hsa_circ_	0.463846	0.183895
hsa_circ_	-0.81375	0.183898
hsa_circ_	1.033553	0.183914
hsa_circ_	0.215302	0.183926
hsa_circ_	-0.3143	0.183947
hsa_circ_	-0.21465	0.183948
hsa_circ_	0.209878	0.183952
hsa_circ_	0.244554	0.183963
hsa_circ_	-0.21085	0.183966
hsa_circ_	-0.39763	0.183966
hsa_circ_	-0.35962	0.183972
hsa_circ_	-0.25566	0.183974
hsa_circ_	-0.2013	0.183992
hsa_circ_	0.685852	0.184002
hsa_circ_	-0.28935	0.184015
hsa_circ_	0.407938	0.18402
hsa_circ_	-0.22837	0.184029
hsa_circ_	-0.57838	0.184041
hsa_circ_	0.264737	0.184051
hsa_circ_	0.229112	0.184055
hsa_circ_	0.285382	0.184056
hsa_circ_	0.414118	0.18406
hsa_circ_	0.809619	0.184093
hsa_circ_	0.316981	0.184105
hsa_circ_	-0.23318	0.184116
hsa_circ_	-0.31998	0.184144
hsa_circ_	0.583697	0.184166
hsa_circ_	0.267758	0.184171
hsa_circ_	0.311034	0.184177
hsa_circ_	-0.21337	0.184181
hsa_circ_	0.204386	0.184181
hsa_circ_	-0.2604	0.184187
hsa_circ_	0.307118	0.184195
hsa_circ_	0.30568	0.184201
hsa_circ_	0.543596	0.184203
hsa_circ_	0.299101	0.184219
hsa_circ_	-0.53307	0.184225
hsa_circ_	-0.90343	0.184227
hsa_circ_	-0.35423	0.18423
hsa_circ_	-0.75395	0.184245
hsa_circ_	0.291822	0.184269
hsa_circ_	-0.21044	0.184269
hsa_circ_	-0.36728	0.184275

hsa_circ_	-0.74959	0.184289
hsa_circ_	0.763849	0.184306
hsa_circ_	0.277827	0.184323
hsa_circ_	-1.36761	0.184331
hsa_circ_	0.320566	0.184335
hsa_circ_	0.79816	0.184357
hsa_circ_	0.215405	0.184374
hsa_circ_	-0.23387	0.184399
hsa_circ_	0.419339	0.184414
hsa_circ_	0.253568	0.184428
hsa_circ_	0.291632	0.184429
hsa_circ_	-0.45166	0.184435
hsa_circ_	-0.19173	0.184444
hsa_circ_	-0.28474	0.184453
hsa_circ_	0.284144	0.184469
hsa_circ_	-0.43796	0.184479
hsa_circ_	0.487129	0.184499
hsa_circ_	0.511483	0.184507
hsa_circ_	0.262979	0.184507
hsa_circ_	-0.21708	0.18451
hsa_circ_	-0.33623	0.184518
hsa_circ_	0.529821	0.184527
hsa_circ_	0.403018	0.184528
hsa_circ_	-0.34502	0.184543
hsa_circ_	-0.30564	0.184543
hsa_circ_	-0.23724	0.184566
hsa_circ_	0.448786	0.184585
hsa_circ_	0.266095	0.184587
hsa_circ_	0.393733	0.184597
hsa_circ_	0.316356	0.1846
hsa_circ_	-0.37027	0.184616
hsa_circ_	0.425214	0.184628
hsa_circ_	0.474634	0.184628
hsa_circ_	0.360715	0.18463
hsa_circ_	-0.38796	0.184633
hsa_circ_	-0.23995	0.184636
hsa_circ_	-0.19726	0.184648
hsa_circ_	-0.30763	0.184679
hsa_circ_	-0.29111	0.184682
hsa_circ_	-0.31517	0.184703
hsa_circ_	0.298338	0.184714
hsa_circ_	0.250301	0.184718
hsa_circ_	-0.27076	0.184736
hsa_circ_	0.93622	0.18474
hsa_circ_	-0.53187	0.18474
hsa_circ_	-0.42243	0.184743
hsa_circ_	-0.63981	0.184746
hsa_circ_	0.233866	0.184747
hsa_circ_	0.382505	0.184753
hsa_circ_	0.662688	0.18476
hsa_circ_	-0.20109	0.184774
hsa_circ_	-0.19101	0.184789
hsa_circ_	-0.26814	0.184791
hsa_circ_	0.443585	0.184799

hsa_circ_	-0.66772	0.184803
hsa_circ_	-0.40351	0.184809
hsa_circ_	0.235184	0.184826
hsa_circ_	-0.71076	0.184834
hsa_circ_	0.39734	0.184838
hsa_circ_	0.193715	0.184838
hsa_circ_	-0.62081	0.184841
hsa_circ_	0.343697	0.184866
hsa_circ_	-0.28791	0.18487
hsa_circ_	0.308731	0.184874
hsa_circ_	-0.47463	0.184877
hsa_circ_	0.24646	0.184878
hsa_circ_	-0.22001	0.184879
hsa_circ_	0.469379	0.184881
hsa_circ_	-0.42556	0.184893
hsa_circ_	0.659961	0.184897
hsa_circ_	0.223577	0.184899
hsa_circ_	-0.28682	0.184904
hsa_circ_	0.865127	0.184913
hsa_circ_	-0.41469	0.184916
hsa_circ_	0.199291	0.184937
hsa_circ_	-0.2242	0.184971
hsa_circ_	0.327751	0.184979
hsa_circ_	-0.2477	0.18498
hsa_circ_	-0.27443	0.184987
hsa_circ_	0.391534	0.185009
hsa_circ_	0.31713	0.185016
hsa_circ_	-1.3586	0.185017
hsa_circ_	-0.21579	0.185019
hsa_circ_	-0.34641	0.18502
hsa_circ_	1.542673	0.185022
hsa_circ_	-0.23199	0.185036
hsa_circ_	0.373209	0.185068
hsa_circ_	-0.38776	0.18508
hsa_circ_	-0.24432	0.185085
hsa_circ_	-0.33785	0.185088
hsa_circ_	-0.22726	0.185088
hsa_circ_	0.892161	0.185088
hsa_circ_	0.248366	0.185104
hsa_circ_	0.273582	0.18511
hsa_circ_	0.795792	0.185119
hsa_circ_	-0.56941	0.185139
hsa_circ_	0.253715	0.185166
hsa_circ_	-0.21044	0.185182
hsa_circ_	-0.33961	0.185188
hsa_circ_	0.348558	0.185216
hsa_circ_	-0.35948	0.185221
hsa_circ_	-0.33473	0.185222
hsa_circ_	-0.2583	0.185231
hsa_circ_	0.287239	0.185235
hsa_circ_	0.312006	0.185254
hsa_circ_	-0.1991	0.185257
hsa_circ_	-0.38015	0.18526
hsa_circ_	-0.17873	0.185266

hsa_circ_	-0.65403	0.185274
hsa_circ_	0.20352	0.185278
hsa_circ_	0.999976	0.185307
hsa_circ_	0.195331	0.185313
hsa_circ_	-0.19857	0.185319
hsa_circ_	0.371095	0.185337
hsa_circ_	0.267529	0.185366
hsa_circ_	-0.50079	0.185366
hsa_circ_	-0.29379	0.185377
hsa_circ_	-0.95685	0.185386
hsa_circ_	-0.56823	0.185392
hsa_circ_	-0.83767	0.185409
hsa_circ_	-0.7736	0.185417
hsa_circ_	-0.27458	0.18543
hsa_circ_	0.481687	0.185433
hsa_circ_	-0.24416	0.18544
hsa_circ_	-0.20604	0.185446
hsa_circ_	0.26038	0.185455
hsa_circ_	-0.44277	0.18546
hsa_circ_	-0.31499	0.185472
hsa_circ_	0.237548	0.185481
hsa_circ_	0.50487	0.185485
hsa_circ_	-0.19024	0.185488
hsa_circ_	-0.21544	0.185493
hsa_circ_	0.476895	0.185496
hsa_circ_	-0.30656	0.185507
hsa_circ_	0.197162	0.185511
hsa_circ_	-0.42855	0.185516
hsa_circ_	-0.28161	0.185518
hsa_circ_	-0.18448	0.185519
hsa_circ_	-1.40383	0.185522
hsa_circ_	-0.2816	0.185536
hsa_circ_	-0.26181	0.185546
hsa_circ_	0.56719	0.185549
hsa_circ_	-0.26952	0.185556
hsa_circ_	0.434244	0.185562
hsa_circ_	0.254177	0.185563
hsa_circ_	0.820887	0.185585
hsa_circ_	-0.78434	0.185586
hsa_circ_	0.702425	0.185589
hsa_circ_	-0.46638	0.185594
hsa_circ_	-0.29564	0.185604
hsa_circ_	0.416817	0.185609
hsa_circ_	-0.33358	0.18561
hsa_circ_	-0.20327	0.185612
hsa_circ_	0.277861	0.185644
hsa_circ_	0.893035	0.185654
hsa_circ_	-0.46105	0.185683
hsa_circ_	-0.42764	0.185695
hsa_circ_	-0.42536	0.1857
hsa_circ_	0.302806	0.185732
hsa_circ_	0.606885	0.185732
hsa_circ_	0.812853	0.185738
hsa_circ_	0.186807	0.185762

hsa_circ_	-0.33253	0.185778
hsa_circ_	-0.33494	0.185782
hsa_circ_	-0.32831	0.185801
hsa_circ_	0.271152	0.185809
hsa_circ_	0.258627	0.185817
hsa_circ_	0.357209	0.185824
hsa_circ_	-0.25542	0.185845
hsa_circ_	-0.41016	0.185848
hsa_circ_	0.646572	0.185851
hsa_circ_	-0.57647	0.185864
hsa_circ_	-0.2004	0.185882
hsa_circ_	0.891634	0.185896
hsa_circ_	-0.28687	0.1859
hsa_circ_	0.29293	0.185903
hsa_circ_	-0.60695	0.185909
hsa_circ_	0.505185	0.18591
hsa_circ_	0.341189	0.18593
hsa_circ_	-0.20906	0.185949
hsa_circ_	0.29882	0.185961
hsa_circ_	0.415976	0.185961
hsa_circ_	-0.58569	0.185964
hsa_circ_	0.192388	0.18597
hsa_circ_	-0.23872	0.185978
hsa_circ_	-0.60968	0.185987
hsa_circ_	0.531222	0.185993
hsa_circ_	0.279172	0.186003
hsa_circ_	0.216391	0.186006
hsa_circ_	-0.25713	0.186009
hsa_circ_	-0.21851	0.186018
hsa_circ_	0.308685	0.186022
hsa_circ_	0.231743	0.186025
hsa_circ_	-0.25158	0.186039
hsa_circ_	0.59983	0.186046
hsa_circ_	-0.63051	0.186054
hsa_circ_	0.408198	0.186071
hsa_circ_	0.533202	0.186083
hsa_circ_	-0.25852	0.186092
hsa_circ_	0.662818	0.186103
hsa_circ_	-0.30257	0.186109
hsa_circ_	-0.51883	0.186111
hsa_circ_	-0.36287	0.186118
hsa_circ_	-0.23384	0.186123
hsa_circ_	0.29331	0.186135
hsa_circ_	-0.28201	0.186146
hsa_circ_	-0.32089	0.186154
hsa_circ_	-0.25516	0.186156
hsa_circ_	0.222615	0.186164
hsa_circ_	-0.30086	0.186166
hsa_circ_	-0.20843	0.186189
hsa_circ_	0.338718	0.186192
hsa_circ_	-0.22285	0.186204
hsa_circ_	-0.19698	0.186204
hsa_circ_	-0.409	0.186214
hsa_circ_	0.245027	0.186219

hsa_circ_	-0.48398	0.186224
hsa_circ_	-0.46392	0.186225
hsa_circ_	0.57488	0.186242
hsa_circ_	-0.41835	0.186243
hsa_circ_	0.243118	0.186251
hsa_circ_	-0.4134	0.186262
hsa_circ_	-0.42	0.186275
hsa_circ_	-0.55465	0.186284
hsa_circ_	0.224499	0.186289
hsa_circ_	0.358591	0.186294
hsa_circ_	-0.3616	0.186294
hsa_circ_	-0.21157	0.186297
hsa_circ_	0.766622	0.186306
hsa_circ_	-0.26906	0.186311
hsa_circ_	0.666827	0.186318
hsa_circ_	0.253846	0.186323
hsa_circ_	0.242992	0.186328
hsa_circ_	0.571836	0.186335
hsa_circ_	0.281243	0.186352
hsa_circ_	-0.21922	0.186354
hsa_circ_	0.223251	0.18636
hsa_circ_	-0.31759	0.186364
hsa_circ_	-1.0086	0.186384
hsa_circ_	-0.27883	0.186385
hsa_circ_	0.313138	0.186388
hsa_circ_	-0.36784	0.18641
hsa_circ_	-0.21978	0.186412
hsa_circ_	0.230039	0.186422
hsa_circ_	0.182641	0.186444
hsa_circ_	-0.21649	0.18645
hsa_circ_	0.255448	0.186453
hsa_circ_	-0.22189	0.186458
hsa_circ_	0.295608	0.18647
hsa_circ_	0.465144	0.18647
hsa_circ_	-0.29259	0.186481
hsa_circ_	-0.27179	0.186518
hsa_circ_	-0.31355	0.186518
hsa_circ_	-0.24525	0.186519
hsa_circ_	0.351492	0.186521
hsa_circ_	-0.28368	0.186559
hsa_circ_	-0.23931	0.186576
hsa_circ_	0.320211	0.186584
hsa_circ_	-0.22149	0.186586
hsa_circ_	0.376518	0.186586
hsa_circ_	-0.30018	0.186623
hsa_circ_	-0.3233	0.186628
hsa_circ_	0.356586	0.186641
hsa_circ_	-0.26681	0.186666
hsa_circ_	-0.20871	0.186673
hsa_circ_	-0.36688	0.186676
hsa_circ_	0.493356	0.186685
hsa_circ_	-0.2241	0.186689
hsa_circ_	0.274441	0.186725
hsa_circ_	0.185981	0.186732

hsa_circ_	1.077019	0.186736
hsa_circ_	-0.22923	0.18674
hsa_circ_	0.272984	0.186746
hsa_circ_	-0.25231	0.186749
hsa_circ_	-0.34206	0.186763
hsa_circ_	0.2119	0.186771
hsa_circ_	-0.40313	0.186778
hsa_circ_	-0.19947	0.186796
hsa_circ_	-0.19075	0.186798
hsa_circ_	-0.22445	0.186821
hsa_circ_	-0.39169	0.186831
hsa_circ_	0.57166	0.186832
hsa_circ_	0.241425	0.186832
hsa_circ_	-0.18191	0.186838
hsa_circ_	0.304156	0.186852
hsa_circ_	0.554795	0.186853
hsa_circ_	0.219076	0.186857
hsa_circ_	0.206874	0.186862
hsa_circ_	0.372669	0.186866
hsa_circ_	0.27845	0.186881
hsa_circ_	0.239198	0.186893
hsa_circ_	-0.23635	0.186896
hsa_circ_	0.254778	0.186939
hsa_circ_	0.330671	0.18695
hsa_circ_	-0.50319	0.186953
hsa_circ_	-0.19667	0.186971
hsa_circ_	0.270438	0.186992
hsa_circ_	0.250042	0.186999
hsa_circ_	-0.25859	0.187009
hsa_circ_	-0.27701	0.187017
hsa_circ_	-0.64195	0.187022
hsa_circ_	-0.80613	0.187026
hsa_circ_	0.250523	0.187051
hsa_circ_	-0.20282	0.187057
hsa_circ_	0.276248	0.187061
hsa_circ_	-0.77131	0.187065
hsa_circ_	0.468298	0.187066
hsa_circ_	0.203839	0.187077
hsa_circ_	-0.45448	0.187086
hsa_circ_	0.322746	0.187089
hsa_circ_	0.295752	0.18709
hsa_circ_	-0.46044	0.187095
hsa_circ_	0.402669	0.187099
hsa_circ_	0.561163	0.187107
hsa_circ_	0.189999	0.187136
hsa_circ_	-0.70046	0.187138
hsa_circ_	0.229748	0.187139
hsa_circ_	-0.68448	0.187139
hsa_circ_	-0.77332	0.187146
hsa_circ_	-0.26477	0.187148
hsa_circ_	0.912775	0.187154
hsa_circ_	0.741982	0.187165
hsa_circ_	-0.24578	0.187166
hsa_circ_	-0.42726	0.187176

hsa_circ_	-0.26999	0.187183
hsa_circ_	0.240008	0.187197
hsa_circ_	0.38857	0.187197
hsa_circ_	-0.21044	0.187205
hsa_circ_	0.238079	0.187215
hsa_circ_	-0.25189	0.187223
hsa_circ_	0.30692	0.187227
hsa_circ_	-0.21848	0.187239
hsa_circ_	-0.56357	0.187244
hsa_circ_	-0.22347	0.187275
hsa_circ_	0.220092	0.187308
hsa_circ_	-0.50402	0.18731
hsa_circ_	0.312283	0.187315
hsa_circ_	0.288273	0.187315
hsa_circ_	-0.42908	0.187316
hsa_circ_	-0.96994	0.187324
hsa_circ_	0.249662	0.187332
hsa_circ_	-0.21623	0.187334
hsa_circ_	0.309591	0.187338
hsa_circ_	-0.25109	0.187338
hsa_circ_	0.236143	0.187345
hsa_circ_	-0.24419	0.187359
hsa_circ_	-0.8366	0.18736
hsa_circ_	-0.26888	0.187362
hsa_circ_	-0.65818	0.187364
hsa_circ_	0.217602	0.18739
hsa_circ_	-0.29735	0.187391
hsa_circ_	0.632633	0.187415
hsa_circ_	0.253369	0.187416
hsa_circ_	0.730932	0.187429
hsa_circ_	-0.55848	0.187431
hsa_circ_	-0.51926	0.187432
hsa_circ_	-0.55948	0.187436
hsa_circ_	0.286553	0.187449
hsa_circ_	-0.24438	0.187449
hsa_circ_	0.314028	0.18746
hsa_circ_	0.399878	0.18747
hsa_circ_	-0.27705	0.187474
hsa_circ_	0.486899	0.187484
hsa_circ_	-0.18739	0.187498
hsa_circ_	0.193725	0.187499
hsa_circ_	-0.67163	0.18751
hsa_circ_	0.236718	0.187526
hsa_circ_	0.350474	0.187532
hsa_circ_	-0.2278	0.187549
hsa_circ_	0.559332	0.187557
hsa_circ_	-0.92953	0.187562
hsa_circ_	-0.62238	0.187581
hsa_circ_	-0.30757	0.187585
hsa_circ_	-0.40989	0.187588
hsa_circ_	-0.25474	0.187593
hsa_circ_	0.276527	0.187595
hsa_circ_	0.303423	0.187595
hsa_circ_	-0.43424	0.187603

hsa_circ_	-0.39237	0.187619
hsa_circ_	-0.21821	0.187623
hsa_circ_	-0.54769	0.187631
hsa_circ_	0.402313	0.187635
hsa_circ_	-0.35674	0.187638
hsa_circ_	-0.72937	0.187648
hsa_circ_	0.356283	0.187665
hsa_circ_	-0.29477	0.187669
hsa_circ_	0.216056	0.187669
hsa_circ_	-0.27063	0.187669
hsa_circ_	0.206676	0.187677
hsa_circ_	0.216818	0.187677
hsa_circ_	-0.31104	0.187678
hsa_circ_	0.306786	0.18768
hsa_circ_	-0.23527	0.187687
hsa_circ_	-0.24736	0.187697
hsa_circ_	0.37985	0.187699
hsa_circ_	0.209112	0.187704
hsa_circ_	0.385875	0.187706
hsa_circ_	-0.8664	0.187713
hsa_circ_	-0.27025	0.187722
hsa_circ_	0.333888	0.187726
hsa_circ_	0.853115	0.187735
hsa_circ_	-0.30068	0.187746
hsa_circ_	0.324205	0.187757
hsa_circ_	-0.34859	0.187758
hsa_circ_	-0.27665	0.187762
hsa_circ_	-0.55163	0.187778
hsa_circ_	0.312762	0.187779
hsa_circ_	-0.19848	0.187783
hsa_circ_	0.390388	0.1878
hsa_circ_	-0.38496	0.187843
hsa_circ_	-0.23088	0.187847
hsa_circ_	0.316455	0.187854
hsa_circ_	-0.25565	0.187862
hsa_circ_	0.330118	0.187865
hsa_circ_	-0.26958	0.18787
hsa_circ_	-0.48289	0.187873
hsa_circ_	-0.75633	0.187878
hsa_circ_	-0.48416	0.187903
hsa_circ_	-0.23596	0.187923
hsa_circ_	0.546534	0.18793
hsa_circ_	0.423954	0.187934
hsa_circ_	0.313251	0.187944
hsa_circ_	0.341704	0.187952
hsa_circ_	-0.18769	0.187971
hsa_circ_	-0.72252	0.187973
hsa_circ_	0.270233	0.187974
hsa_circ_	-1.64725	0.18798
hsa_circ_	0.292507	0.18799
hsa_circ_	-0.50251	0.188002
hsa_circ_	-0.22343	0.188022
hsa_circ_	0.472332	0.188033
hsa_circ_	0.692245	0.188046

hsa_circ_	-0.33441	0.188046
hsa_circ_	0.229468	0.188046
hsa_circ_	0.203014	0.188053
hsa_circ_	-0.67616	0.188069
hsa_circ_	0.378387	0.188079
hsa_circ_	-0.22158	0.188092
hsa_circ_	0.227102	0.188111
hsa_circ_	0.29725	0.188116
hsa_circ_	-0.17903	0.188119
hsa_circ_	-0.31746	0.18812
hsa_circ_	-0.18158	0.188146
hsa_circ_	-0.23268	0.188161
hsa_circ_	-0.34565	0.188167
hsa_circ_	0.247484	0.188173
hsa_circ_	0.61918	0.188186
hsa_circ_	0.235718	0.188191
hsa_circ_	0.225381	0.188204
hsa_circ_	-0.36416	0.188208
hsa_circ_	-0.71709	0.188247
hsa_circ_	-0.56903	0.188247
hsa_circ_	0.799283	0.188258
hsa_circ_	-0.20457	0.188259
hsa_circ_	-0.31878	0.188264
hsa_circ_	-0.18445	0.188266
hsa_circ_	0.429375	0.188268
hsa_circ_	-0.29085	0.18827
hsa_circ_	-0.54229	0.188284
hsa_circ_	0.321831	0.188289
hsa_circ_	0.426971	0.188291
hsa_circ_	-0.26767	0.188301
hsa_circ_	0.43803	0.188314
hsa_circ_	-0.26472	0.188324
hsa_circ_	-0.28468	0.188325
hsa_circ_	0.87399	0.188326
hsa_circ_	-0.35888	0.188348
hsa_circ_	0.361011	0.188358
hsa_circ_	-0.82036	0.18836
hsa_circ_	0.592255	0.188361
hsa_circ_	0.310258	0.188361
hsa_circ_	1.068944	0.18837
hsa_circ_	0.457239	0.188376
hsa_circ_	-0.71151	0.188381
hsa_circ_	0.23241	0.188384
hsa_circ_	-0.20224	0.188385
hsa_circ_	0.285608	0.188389
hsa_circ_	-0.77959	0.18839
hsa_circ_	-0.30252	0.188423
hsa_circ_	-0.23648	0.188434
hsa_circ_	-0.94163	0.188436
hsa_circ_	0.411534	0.188438
hsa_circ_	0.433401	0.188439
hsa_circ_	-0.18676	0.188446
hsa_circ_	-0.26711	0.188461
hsa_circ_	-0.35668	0.188464

hsa_circ_	-0.21151	0.188469
hsa_circ_	0.191327	0.18847
hsa_circ_	-0.29843	0.188476
hsa_circ_	0.473805	0.188484
hsa_circ_	-0.33988	0.188491
hsa_circ_	-0.67278	0.188495
hsa_circ_	-0.19589	0.188499
hsa_circ_	-0.34731	0.188503
hsa_circ_	-0.19842	0.188511
hsa_circ_	-0.18597	0.188516
hsa_circ_	0.247394	0.188527
hsa_circ_	0.344822	0.188534
hsa_circ_	-0.36176	0.188549
hsa_circ_	-0.22105	0.188563
hsa_circ_	-0.20455	0.188565
hsa_circ_	0.23047	0.188568
hsa_circ_	-0.22126	0.188568
hsa_circ_	0.468124	0.188574
hsa_circ_	-0.28218	0.188601
hsa_circ_	-0.32057	0.188613
hsa_circ_	-0.2345	0.188617
hsa_circ_	-0.20696	0.188626
hsa_circ_	-0.43431	0.188629
hsa_circ_	-0.2837	0.188645
hsa_circ_	-0.34909	0.188655
hsa_circ_	0.264959	0.188666
hsa_circ_	0.286451	0.18868
hsa_circ_	0.504127	0.18869
hsa_circ_	0.236073	0.1887
hsa_circ_	-0.24115	0.188719
hsa_circ_	-0.31158	0.188727
hsa_circ_	-0.22901	0.18876
hsa_circ_	0.364215	0.188778
hsa_circ_	-1.10706	0.188785
hsa_circ_	-0.23986	0.188788
hsa_circ_	-0.26246	0.188803
hsa_circ_	0.186441	0.188828
hsa_circ_	-0.42315	0.188852
hsa_circ_	0.287475	0.188858
hsa_circ_	0.630199	0.188863
hsa_circ_	-0.42302	0.188864
hsa_circ_	-0.47916	0.188961
hsa_circ_	0.672171	0.188965
hsa_circ_	-0.46949	0.188999
hsa_circ_	0.272797	0.189038
hsa_circ_	1.462985	0.189047
hsa_circ_	0.302671	0.189049
hsa_circ_	0.195149	0.189052
hsa_circ_	-0.31876	0.189059
hsa_circ_	-0.57587	0.189065
hsa_circ_	-0.23927	0.189067
hsa_circ_	-0.46338	0.189098
hsa_circ_	0.264591	0.189106
hsa_circ_	-0.61484	0.189114

hsa_circ_	-0.50431	0.189115
hsa_circ_	-0.2602	0.189122
hsa_circ_	-0.34601	0.189125
hsa_circ_	0.990012	0.189128
hsa_circ_	0.241468	0.18913
hsa_circ_	-1.10137	0.189158
hsa_circ_	-0.22533	0.189161
hsa_circ_	-0.42655	0.189169
hsa_circ_	-0.20778	0.18917
hsa_circ_	-0.31578	0.189182
hsa_circ_	-0.55726	0.189197
hsa_circ_	-0.25574	0.189206
hsa_circ_	0.495581	0.18921
hsa_circ_	0.269797	0.189213
hsa_circ_	0.181865	0.189235
hsa_circ_	0.30064	0.189251
hsa_circ_	0.403696	0.189283
hsa_circ_	-0.35621	0.189286
hsa_circ_	-0.41397	0.1893
hsa_circ_	-0.20156	0.189307
hsa_circ_	0.258894	0.189317
hsa_circ_	0.28374	0.189329
hsa_circ_	0.710302	0.18933
hsa_circ_	-0.23099	0.189344
hsa_circ_	0.42713	0.189358
hsa_circ_	-0.65888	0.18938
hsa_circ_	-0.28232	0.189388
hsa_circ_	-0.4454	0.189425
hsa_circ_	-0.74716	0.189431
hsa_circ_	0.200237	0.18944
hsa_circ_	-0.22756	0.189459
hsa_circ_	-0.34032	0.189473
hsa_circ_	0.686781	0.189476
hsa_circ_	0.64102	0.189483
hsa_circ_	-0.73475	0.189488
hsa_circ_	-0.18022	0.189493
hsa_circ_	-0.19638	0.189495
hsa_circ_	-0.64908	0.189503
hsa_circ_	-0.28107	0.189519
hsa_circ_	0.264745	0.189535
hsa_circ_	0.661591	0.189556
hsa_circ_	-0.25404	0.189576
hsa_circ_	-0.26162	0.189592
hsa_circ_	-0.44777	0.189605
hsa_circ_	-0.2708	0.189606
hsa_circ_	0.488678	0.189608
hsa_circ_	-0.18578	0.189621
hsa_circ_	0.271532	0.189641
hsa_circ_	-0.27694	0.189647
hsa_circ_	-0.28218	0.189648
hsa_circ_	0.870611	0.18966
hsa_circ_	-0.61397	0.189662
hsa_circ_	0.308928	0.189666
hsa_circ_	0.280394	0.189675

hsa_circ_	0.225923	0.189678
hsa_circ_	-0.23806	0.18969
hsa_circ_	0.361734	0.189699
hsa_circ_	-0.31944	0.189714
hsa_circ_	0.24425	0.189715
hsa_circ_	-0.44741	0.189719
hsa_circ_	0.192746	0.189725
hsa_circ_	-2.43668	0.189728
hsa_circ_	-0.69944	0.189733
hsa_circ_	-0.19048	0.189739
hsa_circ_	0.288559	0.189741
hsa_circ_	-0.61564	0.189757
hsa_circ_	-0.21514	0.189758
hsa_circ_	-0.21173	0.189798
hsa_circ_	0.42055	0.189801
hsa_circ_	0.280875	0.189816
hsa_circ_	-0.92636	0.189846
hsa_circ_	0.175842	0.189854
hsa_circ_	0.191248	0.189857
hsa_circ_	-0.33241	0.189865
hsa_circ_	0.242233	0.189944
hsa_circ_	-0.26654	0.189955
hsa_circ_	-0.25164	0.189978
hsa_circ_	-0.20101	0.18998
hsa_circ_	-0.3224	0.189981
hsa_circ_	-0.27784	0.190012
hsa_circ_	-0.481	0.190023
hsa_circ_	-0.2935	0.190027
hsa_circ_	-0.26183	0.190038
hsa_circ_	-0.21427	0.190056
hsa_circ_	-0.27061	0.190059
hsa_circ_	-0.32723	0.190082
hsa_circ_	0.180819	0.190093
hsa_circ_	-0.49102	0.190103
hsa_circ_	0.263467	0.190109
hsa_circ_	-0.94896	0.190116
hsa_circ_	-0.24088	0.190118
hsa_circ_	-0.41854	0.190119
hsa_circ_	0.258904	0.190132
hsa_circ_	-0.29425	0.19016
hsa_circ_	0.287462	0.190185
hsa_circ_	0.255637	0.190196
hsa_circ_	-0.59826	0.190211
hsa_circ_	-0.80552	0.190226
hsa_circ_	-0.29328	0.190263
hsa_circ_	0.221648	0.190266
hsa_circ_	-0.36807	0.190272
hsa_circ_	-0.39335	0.190309
hsa_circ_	-0.2261	0.190312
hsa_circ_	0.370798	0.190312
hsa_circ_	0.39709	0.190319
hsa_circ_	-0.26933	0.190319
hsa_circ_	-0.20044	0.190326
hsa_circ_	0.562632	0.190329

hsa_circ_	-0.29934	0.190342
hsa_circ_	-0.24992	0.190363
hsa_circ_	-0.27503	0.190376
hsa_circ_	0.193635	0.190383
hsa_circ_	0.20229	0.190394
hsa_circ_	-0.37043	0.190395
hsa_circ_	-0.23947	0.190397
hsa_circ_	-0.42857	0.190399
hsa_circ_	0.302489	0.190415
hsa_circ_	0.287744	0.19042
hsa_circ_	-0.26489	0.190428
hsa_circ_	-0.21722	0.190434
hsa_circ_	-0.64696	0.190437
hsa_circ_	0.610471	0.190441
hsa_circ_	0.802663	0.190442
hsa_circ_	-0.32299	0.190448
hsa_circ_	-0.2811	0.19045
hsa_circ_	0.402701	0.190451
hsa_circ_	-0.3008	0.190458
hsa_circ_	0.204069	0.190463
hsa_circ_	0.229776	0.190468
hsa_circ_	0.325556	0.190479
hsa_circ_	-0.19366	0.190501
hsa_circ_	0.194321	0.190503
hsa_circ_	0.23404	0.190504
hsa_circ_	-0.27511	0.190508
hsa_circ_	-0.3672	0.19051
hsa_circ_	-0.39041	0.190523
hsa_circ_	0.342037	0.190527
hsa_circ_	-0.38637	0.190548
hsa_circ_	-1.16059	0.190567
hsa_circ_	1.025655	0.1906
hsa_circ_	-0.37646	0.19061
hsa_circ_	0.387775	0.190616
hsa_circ_	-0.34082	0.190622
hsa_circ_	0.48316	0.190632
hsa_circ_	0.430162	0.19064
hsa_circ_	0.232509	0.190664
hsa_circ_	0.45566	0.190687
hsa_circ_	0.739306	0.19069
hsa_circ_	-2.79676	0.190695
hsa_circ_	0.250012	0.190703
hsa_circ_	0.191772	0.190713
hsa_circ_	0.415646	0.190715
hsa_circ_	0.343496	0.190717
hsa_circ_	-0.32697	0.190731
hsa_circ_	-0.35439	0.190786
hsa_circ_	-0.24166	0.19079
hsa_circ_	-0.25711	0.190792
hsa_circ_	-0.41891	0.190794
hsa_circ_	0.570309	0.190805
hsa_circ_	0.201049	0.190817
hsa_circ_	-0.24148	0.190842
hsa_circ_	0.259811	0.190846

hsa_circ_	-0.20334	0.190855
hsa_circ_	-0.5479	0.190858
hsa_circ_	0.180617	0.190861
hsa_circ_	-0.50788	0.190864
hsa_circ_	-0.55554	0.190868
hsa_circ_	-0.22123	0.190876
hsa_circ_	0.716812	0.190882
hsa_circ_	-0.47941	0.190894
hsa_circ_	0.409293	0.19092
hsa_circ_	0.362418	0.190926
hsa_circ_	-0.2756	0.190955
hsa_circ_	-0.2295	0.190957
hsa_circ_	0.282982	0.190969
hsa_circ_	-0.46639	0.190993
hsa_circ_	-0.24914	0.190999
hsa_circ_	0.299476	0.191006
hsa_circ_	-0.23916	0.191017
hsa_circ_	0.36316	0.191021
hsa_circ_	0.237262	0.191032
hsa_circ_	-0.29367	0.191046
hsa_circ_	0.368572	0.191076
hsa_circ_	-0.26991	0.191077
hsa_circ_	0.323561	0.191098
hsa_circ_	-0.21574	0.191106
hsa_circ_	-0.53615	0.191117
hsa_circ_	-0.54165	0.191117
hsa_circ_	0.18859	0.191127
hsa_circ_	-0.2267	0.191154
hsa_circ_	-0.70344	0.191156
hsa_circ_	-0.20034	0.191161
hsa_circ_	-0.21445	0.191166
hsa_circ_	-0.27309	0.191168
hsa_circ_	-0.1848	0.191182
hsa_circ_	0.466313	0.191188
hsa_circ_	-0.40016	0.191223
hsa_circ_	0.265017	0.191226
hsa_circ_	-0.29711	0.191226
hsa_circ_	0.678951	0.191236
hsa_circ_	-0.34694	0.191267
hsa_circ_	0.23303	0.191276
hsa_circ_	0.255107	0.191295
hsa_circ_	-0.3317	0.191298
hsa_circ_	0.210234	0.191299
hsa_circ_	-0.29946	0.191305
hsa_circ_	0.232267	0.191338
hsa_circ_	0.315051	0.191346
hsa_circ_	-0.19339	0.191355
hsa_circ_	-0.38959	0.191357
hsa_circ_	-0.21059	0.191361
hsa_circ_	-0.25297	0.191369
hsa_circ_	-0.32059	0.191387
hsa_circ_	0.262727	0.191387
hsa_circ_	0.42616	0.191388
hsa_circ_	-0.26927	0.191419

hsa_circ_	-0.36842	0.191422
hsa_circ_	-0.5689	0.191446
hsa_circ_	-0.39355	0.191448
hsa_circ_	-0.42274	0.191459
hsa_circ_	0.289943	0.191482
hsa_circ_	-0.25703	0.191494
hsa_circ_	-0.55708	0.191501
hsa_circ_	-0.29529	0.191504
hsa_circ_	-0.97366	0.19152
hsa_circ_	-0.38722	0.191522
hsa_circ_	-0.39915	0.191524
hsa_circ_	0.248127	0.191525
hsa_circ_	0.21119	0.191534
hsa_circ_	-0.24351	0.191536
hsa_circ_	-0.45843	0.191585
hsa_circ_	0.283387	0.191592
hsa_circ_	-0.23692	0.191629
hsa_circ_	-0.30183	0.19163
hsa_circ_	-0.26648	0.191639
hsa_circ_	0.229782	0.191642
hsa_circ_	-0.81166	0.191658
hsa_circ_	-0.20803	0.191659
hsa_circ_	-0.20542	0.191668
hsa_circ_	-0.80514	0.19167
hsa_circ_	-0.32041	0.191692
hsa_circ_	0.334331	0.1917
hsa_circ_	0.213876	0.191715
hsa_circ_	-0.43783	0.191718
hsa_circ_	-0.26119	0.19172
hsa_circ_	-0.27362	0.191725
hsa_circ_	-0.59398	0.19173
hsa_circ_	-0.27991	0.19175
hsa_circ_	0.242532	0.191753
hsa_circ_	-1.13187	0.19176
hsa_circ_	-0.19029	0.191768
hsa_circ_	0.388179	0.191778
hsa_circ_	-0.46021	0.19178
hsa_circ_	0.204922	0.191784
hsa_circ_	0.275143	0.191799
hsa_circ_	0.333733	0.191806
hsa_circ_	-0.22353	0.191821
hsa_circ_	-0.75074	0.191823
hsa_circ_	0.833591	0.191831
hsa_circ_	0.242882	0.191849
hsa_circ_	0.230933	0.191856
hsa_circ_	-0.33326	0.191866
hsa_circ_	-0.19168	0.191874
hsa_circ_	-0.28782	0.19188
hsa_circ_	0.88644	0.191888
hsa_circ_	0.640206	0.191895
hsa_circ_	0.187624	0.1919
hsa_circ_	-0.61526	0.191948
hsa_circ_	-0.24167	0.191962
hsa_circ_	-0.30395	0.19197

hsa_circ_	-1.40744	0.191975
hsa_circ_	0.201359	0.191979
hsa_circ_	0.199409	0.192019
hsa_circ_	0.189308	0.192026
hsa_circ_	0.394012	0.19203
hsa_circ_	-0.23685	0.192045
hsa_circ_	-0.18088	0.19206
hsa_circ_	-0.23795	0.192066
hsa_circ_	-0.22924	0.192073
hsa_circ_	0.252638	0.19211
hsa_circ_	0.335742	0.192115
hsa_circ_	-0.219	0.192128
hsa_circ_	-0.23918	0.192168
hsa_circ_	0.466655	0.192178
hsa_circ_	-0.66751	0.192178
hsa_circ_	0.444776	0.192181
hsa_circ_	-0.69706	0.192191
hsa_circ_	-0.64919	0.192191
hsa_circ_	0.670357	0.192199
hsa_circ_	0.63587	0.192201
hsa_circ_	-0.22537	0.192211
hsa_circ_	0.76104	0.192218
hsa_circ_	0.505482	0.192223
hsa_circ_	0.35008	0.192244
hsa_circ_	-0.25769	0.192252
hsa_circ_	-0.22745	0.192255
hsa_circ_	-0.21057	0.192283
hsa_circ_	-0.19971	0.192286
hsa_circ_	-0.23905	0.192293
hsa_circ_	0.210946	0.192301
hsa_circ_	0.429233	0.192326
hsa_circ_	0.797327	0.192327
hsa_circ_	0.527756	0.192338
hsa_circ_	-0.17985	0.192345
hsa_circ_	0.350015	0.192367
hsa_circ_	0.787936	0.192374
hsa_circ_	0.296816	0.192376
hsa_circ_	-0.39098	0.192382
hsa_circ_	0.409328	0.192387
hsa_circ_	0.326198	0.192402
hsa_circ_	-0.71691	0.192407
hsa_circ_	-0.55225	0.192411
hsa_circ_	-1.33982	0.192414
hsa_circ_	0.202767	0.192414
hsa_circ_	0.251113	0.192419
hsa_circ_	-0.42252	0.192424
hsa_circ_	-0.18696	0.192426
hsa_circ_	0.267736	0.192461
hsa_circ_	-0.25529	0.192462
hsa_circ_	-0.41074	0.192466
hsa_circ_	0.320576	0.192473
hsa_circ_	-0.43653	0.192474
hsa_circ_	-0.23131	0.192493
hsa_circ_	0.373078	0.192496

hsa_circ_	0.199465	0.192509
hsa_circ_	-0.25145	0.192517
hsa_circ_	-0.2026	0.192528
hsa_circ_	0.393142	0.192531
hsa_circ_	0.389575	0.192542
hsa_circ_	0.271482	0.192559
hsa_circ_	-0.38913	0.19256
hsa_circ_	0.339852	0.19257
hsa_circ_	0.629141	0.192599
hsa_circ_	0.226691	0.19261
hsa_circ_	0.260831	0.192654
hsa_circ_	-0.24352	0.192658
hsa_circ_	-0.38054	0.192659
hsa_circ_	-1.36163	0.19266
hsa_circ_	-0.58342	0.192668
hsa_circ_	0.244313	0.192671
hsa_circ_	0.325654	0.19268
hsa_circ_	-0.19709	0.192682
hsa_circ_	0.224919	0.192686
hsa_circ_	0.250172	0.192705
hsa_circ_	-0.25428	0.192718
hsa_circ_	-0.40723	0.192722
hsa_circ_	0.199014	0.192729
hsa_circ_	0.26836	0.192731
hsa_circ_	0.50915	0.192733
hsa_circ_	0.392588	0.192733
hsa_circ_	1.135195	0.192735
hsa_circ_	-0.2016	0.192742
hsa_circ_	-0.29019	0.192749
hsa_circ_	-0.33438	0.192752
hsa_circ_	-0.28576	0.192752
hsa_circ_	-0.23296	0.192756
hsa_circ_	-0.26737	0.192762
hsa_circ_	-0.26644	0.192763
hsa_circ_	-0.31336	0.192773
hsa_circ_	-0.22074	0.192791
hsa_circ_	0.687061	0.192795
hsa_circ_	0.498256	0.192813
hsa_circ_	0.203316	0.192821
hsa_circ_	-0.51531	0.192823
hsa_circ_	0.257201	0.192838
hsa_circ_	0.35909	0.192839
hsa_circ_	0.284445	0.192855
hsa_circ_	-0.23622	0.19286
hsa_circ_	0.439259	0.192877
hsa_circ_	-0.24979	0.192886
hsa_circ_	-0.22284	0.192888
hsa_circ_	0.187053	0.192892
hsa_circ_	-0.24368	0.192901
hsa_circ_	0.556462	0.192911
hsa_circ_	-0.42032	0.192913
hsa_circ_	0.53657	0.192922
hsa_circ_	-0.1959	0.192923
hsa_circ_	-0.48772	0.192935

hsa_circ_	-0.22619	0.192951
hsa_circ_	0.455455	0.19296
hsa_circ_	0.554363	0.192966
hsa_circ_	0.81685	0.192967
hsa_circ_	-0.31879	0.192971
hsa_circ_	0.48714	0.192977
hsa_circ_	0.41364	0.19298
hsa_circ_	0.282909	0.192986
hsa_circ_	-0.2023	0.192989
hsa_circ_	-0.17926	0.193017
hsa_circ_	0.208236	0.193023
hsa_circ_	-0.22852	0.193034
hsa_circ_	0.21237	0.193034
hsa_circ_	0.238711	0.193043
hsa_circ_	0.283318	0.193075
hsa_circ_	-0.2096	0.193082
hsa_circ_	0.243913	0.193094
hsa_circ_	0.320834	0.193101
hsa_circ_	0.509738	0.193114
hsa_circ_	-0.29142	0.193122
hsa_circ_	-0.26012	0.193127
hsa_circ_	0.887024	0.193141
hsa_circ_	-0.32906	0.193145
hsa_circ_	0.844755	0.193156
hsa_circ_	-0.28469	0.193157
hsa_circ_	0.208873	0.193162
hsa_circ_	0.20309	0.193194
hsa_circ_	-0.20505	0.193205
hsa_circ_	-0.27557	0.193223
hsa_circ_	0.300139	0.193229
hsa_circ_	0.307974	0.193243
hsa_circ_	0.250685	0.193252
hsa_circ_	0.261601	0.193296
hsa_circ_	-0.22525	0.1933
hsa_circ_	-0.21946	0.193303
hsa_circ_	-0.28592	0.193303
hsa_circ_	0.256942	0.193336
hsa_circ_	0.325085	0.193338
hsa_circ_	0.257566	0.193354
hsa_circ_	-0.20227	0.193377
hsa_circ_	0.518723	0.193387
hsa_circ_	0.600453	0.193392
hsa_circ_	-0.64924	0.193404
hsa_circ_	0.447175	0.193414
hsa_circ_	0.236778	0.193415
hsa_circ_	-0.25657	0.193425
hsa_circ_	0.510047	0.193426
hsa_circ_	0.934197	0.193428
hsa_circ_	-0.22647	0.193428
hsa_circ_	0.18748	0.193429
hsa_circ_	-0.2238	0.19344
hsa_circ_	0.250274	0.193454
hsa_circ_	0.386453	0.193468
hsa_circ_	0.364705	0.193473

hsa_circ_	0.616532	0.193479
hsa_circ_	-0.38839	0.193483
hsa_circ_	-1.31457	0.193483
hsa_circ_	0.707687	0.193486
hsa_circ_	0.192482	0.193503
hsa_circ_	-0.3522	0.193504
hsa_circ_	0.271665	0.193512
hsa_circ_	0.327677	0.193514
hsa_circ_	0.271234	0.193519
hsa_circ_	0.393722	0.19355
hsa_circ_	0.314202	0.193564
hsa_circ_	0.215493	0.19358
hsa_circ_	0.194163	0.193584
hsa_circ_	0.241248	0.193585
hsa_circ_	-0.54174	0.193604
hsa_circ_	0.258069	0.193604
hsa_circ_	-0.30125	0.193615
hsa_circ_	0.220811	0.193615
hsa_circ_	-0.38654	0.193627
hsa_circ_	-0.20263	0.19363
hsa_circ_	-0.37198	0.193638
hsa_circ_	0.433479	0.19364
hsa_circ_	0.201956	0.193643
hsa_circ_	0.215468	0.193661
hsa_circ_	0.320041	0.193664
hsa_circ_	0.39257	0.193684
hsa_circ_	0.545787	0.193694
hsa_circ_	-0.54903	0.193731
hsa_circ_	-0.72679	0.193731
hsa_circ_	0.290301	0.193757
hsa_circ_	0.692721	0.193757
hsa_circ_	0.240643	0.193784
hsa_circ_	0.400566	0.193784
hsa_circ_	-0.2283	0.193785
hsa_circ_	-0.18572	0.193785
hsa_circ_	-0.23061	0.193793
hsa_circ_	-0.23502	0.193807
hsa_circ_	-0.38601	0.19381
hsa_circ_	0.327723	0.193816
hsa_circ_	-0.38526	0.193823
hsa_circ_	0.202455	0.193824
hsa_circ_	0.266519	0.193839
hsa_circ_	-0.51455	0.193843
hsa_circ_	-0.60876	0.193852
hsa_circ_	-0.28953	0.193855
hsa_circ_	-0.60728	0.19386
hsa_circ_	-0.18366	0.193913
hsa_circ_	-0.17922	0.193922
hsa_circ_	-0.53003	0.193928
hsa_circ_	0.279875	0.193929
hsa_circ_	-0.44525	0.193933
hsa_circ_	0.305236	0.193942
hsa_circ_	0.295368	0.193947
hsa_circ_	-0.25058	0.193948

hsa_circ_	-0.22262	0.193959
hsa_circ_	0.284879	0.193967
hsa_circ_	-0.27113	0.193971
hsa_circ_	0.76371	0.193989
hsa_circ_	-0.24215	0.193994
hsa_circ_	0.577878	0.194006
hsa_circ_	-0.36735	0.19402
hsa_circ_	0.208814	0.194057
hsa_circ_	0.203237	0.194074
hsa_circ_	0.371171	0.194076
hsa_circ_	-0.38937	0.194082
hsa_circ_	0.466323	0.194087
hsa_circ_	0.298704	0.194106
hsa_circ_	0.262137	0.194109
hsa_circ_	0.361287	0.194125
hsa_circ_	-0.31121	0.19413
hsa_circ_	0.1914	0.194178
hsa_circ_	0.185034	0.194188
hsa_circ_	-0.43036	0.194189
hsa_circ_	-0.21455	0.194202
hsa_circ_	-0.35276	0.194251
hsa_circ_	-0.1911	0.194254
hsa_circ_	-0.25597	0.194255
hsa_circ_	0.277558	0.194263
hsa_circ_	0.270311	0.194264
hsa_circ_	-0.39096	0.194273
hsa_circ_	0.320245	0.194282
hsa_circ_	0.369004	0.194284
hsa_circ_	-0.67895	0.194297
hsa_circ_	0.220399	0.194299
hsa_circ_	0.271838	0.194299
hsa_circ_	-0.24204	0.194303
hsa_circ_	-0.20363	0.194303
hsa_circ_	-0.23439	0.194307
hsa_circ_	-0.38952	0.194309
hsa_circ_	0.208639	0.194324
hsa_circ_	-0.22053	0.194349
hsa_circ_	0.430502	0.194358
hsa_circ_	0.292328	0.194374
hsa_circ_	-0.22144	0.194378
hsa_circ_	-0.23381	0.194384
hsa_circ_	0.355821	0.194392
hsa_circ_	0.346442	0.194395
hsa_circ_	-0.23045	0.194397
hsa_circ_	0.182881	0.194401
hsa_circ_	0.732948	0.194415
hsa_circ_	-0.46236	0.194429
hsa_circ_	0.23933	0.194434
hsa_circ_	0.205884	0.194436
hsa_circ_	0.221187	0.194437
hsa_circ_	-0.53821	0.194456
hsa_circ_	0.249083	0.19446
hsa_circ_	0.761982	0.19447
hsa_circ_	-0.36419	0.194473

hsa_circ_	0.344969	0.194473
hsa_circ_	-0.23554	0.194487
hsa_circ_	-0.37822	0.194491
hsa_circ_	-0.52111	0.194498
hsa_circ_	-0.71439	0.194505
hsa_circ_	-0.26612	0.19451
hsa_circ_	0.488706	0.194532
hsa_circ_	0.276481	0.194565
hsa_circ_	-0.19543	0.194569
hsa_circ_	0.210293	0.194584
hsa_circ_	0.795531	0.194612
hsa_circ_	-0.19913	0.194613
hsa_circ_	0.295802	0.194625
hsa_circ_	-0.54492	0.194627
hsa_circ_	0.209191	0.194629
hsa_circ_	-0.26252	0.19464
hsa_circ_	-0.45567	0.194659
hsa_circ_	0.318583	0.194662
hsa_circ_	-0.19652	0.194673
hsa_circ_	0.328334	0.194706
hsa_circ_	-0.40721	0.194714
hsa_circ_	-0.26084	0.194721
hsa_circ_	0.410742	0.194725
hsa_circ_	0.235612	0.194745
hsa_circ_	-0.37964	0.194755
hsa_circ_	0.210346	0.194767
hsa_circ_	0.432537	0.194795
hsa_circ_	-0.2434	0.19481
hsa_circ_	-0.21592	0.194825
hsa_circ_	0.306868	0.194826
hsa_circ_	0.222945	0.194832
hsa_circ_	-0.30369	0.194835
hsa_circ_	-0.2523	0.194838
hsa_circ_	-0.18713	0.194872
hsa_circ_	0.236924	0.194877
hsa_circ_	0.257855	0.194884
hsa_circ_	0.237086	0.194892
hsa_circ_	0.285067	0.194898
hsa_circ_	-0.43758	0.194918
hsa_circ_	0.205531	0.194929
hsa_circ_	-0.51427	0.194939
hsa_circ_	0.307028	0.194968
hsa_circ_	-0.19716	0.194977
hsa_circ_	-0.23867	0.194988
hsa_circ_	0.337949	0.194988
hsa_circ_	-0.23497	0.194989
hsa_circ_	0.330656	0.19501
hsa_circ_	0.593321	0.195026
hsa_circ_	-0.36395	0.195028
hsa_circ_	0.368741	0.19503
hsa_circ_	0.461754	0.195036
hsa_circ_	0.218486	0.195037
hsa_circ_	0.282819	0.195051
hsa_circ_	0.516625	0.195058

hsa_circ_	-0.3259	0.195079
hsa_circ_	0.411649	0.195088
hsa_circ_	0.300189	0.195095
hsa_circ_	0.276828	0.195096
hsa_circ_	0.521749	0.195106
hsa_circ_	-0.24849	0.195133
hsa_circ_	-0.6711	0.195134
hsa_circ_	-0.17964	0.195136
hsa_circ_	0.36896	0.195157
hsa_circ_	-0.32598	0.195164
hsa_circ_	-0.18643	0.195169
hsa_circ_	1.330984	0.195169
hsa_circ_	-0.27297	0.195173
hsa_circ_	-0.19782	0.195175
hsa_circ_	-0.24348	0.19518
hsa_circ_	-0.19768	0.195182
hsa_circ_	0.544984	0.195188
hsa_circ_	0.219586	0.19519
hsa_circ_	-0.75012	0.195193
hsa_circ_	0.234906	0.195202
hsa_circ_	0.68054	0.195206
hsa_circ_	0.833718	0.19521
hsa_circ_	0.452235	0.195231
hsa_circ_	0.290292	0.195234
hsa_circ_	0.238687	0.195237
hsa_circ_	0.308129	0.195239
hsa_circ_	-0.28454	0.195243
hsa_circ_	-0.44922	0.195243
hsa_circ_	0.262459	0.195259
hsa_circ_	0.436842	0.195261
hsa_circ_	-0.31748	0.195263
hsa_circ_	0.235644	0.195264
hsa_circ_	0.212477	0.195267
hsa_circ_	0.344119	0.195274
hsa_circ_	-0.27355	0.195279
hsa_circ_	-0.24991	0.195284
hsa_circ_	-0.5448	0.195293
hsa_circ_	0.516217	0.195306
hsa_circ_	-0.24361	0.195312
hsa_circ_	-0.23923	0.195314
hsa_circ_	0.266435	0.195317
hsa_circ_	0.86278	0.195327
hsa_circ_	0.199068	0.195347
hsa_circ_	-0.29511	0.195379
hsa_circ_	0.221048	0.195387
hsa_circ_	-0.40605	0.195387
hsa_circ_	-0.38171	0.195401
hsa_circ_	0.238571	0.195401
hsa_circ_	0.201294	0.19542
hsa_circ_	0.217239	0.195427
hsa_circ_	0.234047	0.195455
hsa_circ_	0.225782	0.19546
hsa_circ_	-0.20177	0.195476
hsa_circ_	0.345354	0.195478

hsa_circ_	0.316495	0.195503
hsa_circ_	0.249382	0.195521
hsa_circ_	-0.26973	0.195524
hsa_circ_	-0.46548	0.195532
hsa_circ_	0.226647	0.195555
hsa_circ_	-0.2455	0.195559
hsa_circ_	-0.30756	0.195578
hsa_circ_	-0.27284	0.195586
hsa_circ_	0.249814	0.195592
hsa_circ_	0.3198	0.195598
hsa_circ_	0.30985	0.195601
hsa_circ_	-0.20195	0.195604
hsa_circ_	0.482792	0.195619
hsa_circ_	-0.49019	0.19562
hsa_circ_	0.292911	0.195638
hsa_circ_	0.221914	0.19568
hsa_circ_	1.171551	0.195692
hsa_circ_	-0.71115	0.195713
hsa_circ_	-0.62016	0.195724
hsa_circ_	0.19948	0.195732
hsa_circ_	0.67955	0.195732
hsa_circ_	0.644441	0.195739
hsa_circ_	-0.19424	0.195747
hsa_circ_	-0.22659	0.195749
hsa_circ_	0.295337	0.195758
hsa_circ_	-0.28928	0.195764
hsa_circ_	0.263248	0.195764
hsa_circ_	-0.36574	0.195767
hsa_circ_	-0.62418	0.195768
hsa_circ_	0.934971	0.195779
hsa_circ_	0.253688	0.195808
hsa_circ_	-0.24384	0.195812
hsa_circ_	0.475386	0.19583
hsa_circ_	-1.8409	0.195832
hsa_circ_	-0.58796	0.195847
hsa_circ_	-0.24607	0.195857
hsa_circ_	-0.72699	0.195869
hsa_circ_	0.280172	0.195871
hsa_circ_	-0.29322	0.195874
hsa_circ_	-1.71743	0.195893
hsa_circ_	-0.22629	0.195926
hsa_circ_	-0.36569	0.195927
hsa_circ_	-0.78215	0.195928
hsa_circ_	1.473897	0.195939
hsa_circ_	0.300663	0.195956
hsa_circ_	-0.18571	0.195956
hsa_circ_	-0.79793	0.195962
hsa_circ_	-0.20175	0.195967
hsa_circ_	0.409518	0.195969
hsa_circ_	-0.49017	0.195973
hsa_circ_	0.873863	0.195977
hsa_circ_	-0.19254	0.195978
hsa_circ_	-0.44595	0.196003
hsa_circ_	-0.86194	0.196025

hsa_circ_	0.235671	0.196033
hsa_circ_	-0.21739	0.196048
hsa_circ_	-0.18823	0.196049
hsa_circ_	0.18123	0.19606
hsa_circ_	-0.38242	0.196075
hsa_circ_	0.273766	0.196079
hsa_circ_	-0.3304	0.196093
hsa_circ_	-0.35772	0.196108
hsa_circ_	-0.29581	0.19611
hsa_circ_	-0.35671	0.196111
hsa_circ_	0.404899	0.196114
hsa_circ_	0.316499	0.196115
hsa_circ_	-0.8325	0.196127
hsa_circ_	-0.67463	0.196128
hsa_circ_	0.301223	0.196159
hsa_circ_	-0.18857	0.19617
hsa_circ_	-0.35548	0.196182
hsa_circ_	-0.46699	0.196188
hsa_circ_	-0.36682	0.196217
hsa_circ_	0.625041	0.196218
hsa_circ_	0.677967	0.196222
hsa_circ_	-0.36418	0.196224
hsa_circ_	-0.19995	0.196234
hsa_circ_	-0.44711	0.196237
hsa_circ_	-0.40009	0.196248
hsa_circ_	-0.53663	0.196257
hsa_circ_	-0.30574	0.19627
hsa_circ_	0.245617	0.196276
hsa_circ_	0.338242	0.196292
hsa_circ_	-0.26118	0.196296
hsa_circ_	0.324754	0.196303
hsa_circ_	-0.26269	0.196304
hsa_circ_	0.431215	0.196316
hsa_circ_	0.271783	0.196318
hsa_circ_	-0.26068	0.196324
hsa_circ_	-0.29021	0.196325
hsa_circ_	-0.24984	0.196339
hsa_circ_	-0.46025	0.19635
hsa_circ_	0.204219	0.19635
hsa_circ_	-0.28217	0.196365
hsa_circ_	0.212402	0.196372
hsa_circ_	-0.23508	0.196385
hsa_circ_	-0.2211	0.196388
hsa_circ_	-0.20474	0.196388
hsa_circ_	0.306766	0.196391
hsa_circ_	-0.20655	0.196394
hsa_circ_	-0.29241	0.196398
hsa_circ_	0.29732	0.196409
hsa_circ_	0.36695	0.196416
hsa_circ_	-0.20337	0.196429
hsa_circ_	0.210727	0.196438
hsa_circ_	-0.22078	0.196456
hsa_circ_	0.331108	0.196472
hsa_circ_	-0.34102	0.196478

hsa_circ_	-1.01452	0.196479
hsa_circ_	0.763378	0.196486
hsa_circ_	0.448832	0.196503
hsa_circ_	0.330083	0.196508
hsa_circ_	-0.36925	0.196523
hsa_circ_	-0.30292	0.196529
hsa_circ_	-0.27589	0.196532
hsa_circ_	0.31024	0.196535
hsa_circ_	0.322821	0.196543
hsa_circ_	-0.63267	0.196544
hsa_circ_	-0.36178	0.196544
hsa_circ_	-0.30346	0.196545
hsa_circ_	-0.28166	0.19655
hsa_circ_	-1.57015	0.19655
hsa_circ_	0.312831	0.196576
hsa_circ_	0.311272	0.19659
hsa_circ_	-0.63744	0.196612
hsa_circ_	-0.27985	0.196615
hsa_circ_	-0.24232	0.196627
hsa_circ_	-0.18579	0.196648
hsa_circ_	-0.56554	0.196659
hsa_circ_	0.40028	0.196661
hsa_circ_	-0.25529	0.196665
hsa_circ_	0.893771	0.196668
hsa_circ_	0.854377	0.196674
hsa_circ_	-0.40109	0.19668
hsa_circ_	-0.23817	0.196715
hsa_circ_	-0.25487	0.19675
hsa_circ_	-0.23138	0.196752
hsa_circ_	0.452731	0.196761
hsa_circ_	0.212285	0.196765
hsa_circ_	-0.24629	0.196767
hsa_circ_	-0.31836	0.196769
hsa_circ_	0.198804	0.196779
hsa_circ_	0.451835	0.19678
hsa_circ_	-0.21005	0.196782
hsa_circ_	0.272818	0.196784
hsa_circ_	-0.38877	0.196787
hsa_circ_	0.482624	0.196792
hsa_circ_	0.238602	0.196801
hsa_circ_	0.223308	0.196818
hsa_circ_	-0.28885	0.196828
hsa_circ_	-0.35498	0.196843
hsa_circ_	-0.22467	0.19686
hsa_circ_	0.291556	0.196874
hsa_circ_	-0.19877	0.196882
hsa_circ_	-0.23487	0.196885
hsa_circ_	0.183924	0.196892
hsa_circ_	0.241913	0.196926
hsa_circ_	0.235994	0.196934
hsa_circ_	0.390216	0.196941
hsa_circ_	-0.23718	0.196954
hsa_circ_	-0.48177	0.196957
hsa_circ_	-0.24067	0.196966

hsa_circ_	0.877123	0.196983
hsa_circ_	-0.29856	0.196987
hsa_circ_	0.472358	0.196992
hsa_circ_	-0.23058	0.196994
hsa_circ_	0.281048	0.197007
hsa_circ_	-0.37724	0.197022
hsa_circ_	-0.22924	0.197028
hsa_circ_	-0.40557	0.197031
hsa_circ_	-0.3156	0.197042
hsa_circ_	0.382245	0.197042
hsa_circ_	0.365749	0.197048
hsa_circ_	0.506165	0.197055
hsa_circ_	0.24513	0.19706
hsa_circ_	-0.87688	0.197087
hsa_circ_	0.635936	0.197102
hsa_circ_	-0.25046	0.197105
hsa_circ_	0.216883	0.197112
hsa_circ_	0.215868	0.197114
hsa_circ_	0.724598	0.197135
hsa_circ_	-0.69445	0.197137
hsa_circ_	-1.06511	0.197139
hsa_circ_	0.678446	0.19714
hsa_circ_	-0.31387	0.197156
hsa_circ_	0.551214	0.197157
hsa_circ_	0.591246	0.19716
hsa_circ_	-0.29134	0.19716
hsa_circ_	-0.25698	0.197165
hsa_circ_	0.321662	0.197184
hsa_circ_	-0.2079	0.197186
hsa_circ_	0.209706	0.197227
hsa_circ_	-0.20606	0.197237
hsa_circ_	-0.21107	0.197244
hsa_circ_	-0.40472	0.197247
hsa_circ_	0.6715	0.197254
hsa_circ_	-0.40069	0.197255
hsa_circ_	-0.19238	0.197263
hsa_circ_	0.354931	0.197267
hsa_circ_	0.306099	0.197268
hsa_circ_	0.26857	0.197269
hsa_circ_	-0.4304	0.197269
hsa_circ_	-0.21522	0.197291
hsa_circ_	-0.34984	0.197291
hsa_circ_	0.248214	0.197292
hsa_circ_	-0.41844	0.1973
hsa_circ_	-0.52919	0.197306
hsa_circ_	-0.21777	0.197328
hsa_circ_	0.24943	0.197329
hsa_circ_	0.424894	0.197333
hsa_circ_	-0.26103	0.197338
hsa_circ_	0.370994	0.197358
hsa_circ_	-0.21506	0.197365
hsa_circ_	-0.67699	0.19737
hsa_circ_	-0.36898	0.19737
hsa_circ_	-0.20409	0.197371

hsa_circ_	0.178983	0.197378
hsa_circ_	-0.22188	0.197386
hsa_circ_	0.220577	0.197389
hsa_circ_	-0.19223	0.197394
hsa_circ_	0.300882	0.197394
hsa_circ_	0.443656	0.197409
hsa_circ_	0.486988	0.197416
hsa_circ_	0.23128	0.197427
hsa_circ_	-0.19993	0.197446
hsa_circ_	0.292835	0.197451
hsa_circ_	0.316476	0.197452
hsa_circ_	0.349363	0.197469
hsa_circ_	0.184537	0.197473
hsa_circ_	-0.24534	0.197492
hsa_circ_	0.292959	0.197506
hsa_circ_	-0.24112	0.197514
hsa_circ_	-0.51962	0.19753
hsa_circ_	-0.48247	0.197534
hsa_circ_	-0.18761	0.197539
hsa_circ_	0.758171	0.197541
hsa_circ_	1.11177	0.197548
hsa_circ_	-0.30577	0.197549
hsa_circ_	0.234964	0.197553
hsa_circ_	0.342228	0.197591
hsa_circ_	-0.20783	0.1976
hsa_circ_	0.922834	0.197608
hsa_circ_	0.387032	0.197618
hsa_circ_	0.271609	0.197651
hsa_circ_	-0.93363	0.197653
hsa_circ_	-0.27611	0.197665
hsa_circ_	-0.3467	0.197685
hsa_circ_	0.787452	0.197694
hsa_circ_	-0.7131	0.197715
hsa_circ_	0.223705	0.197724
hsa_circ_	0.411579	0.197728
hsa_circ_	0.285402	0.197728
hsa_circ_	0.390244	0.197768
hsa_circ_	0.31589	0.197781
hsa_circ_	0.394315	0.197789
hsa_circ_	-0.22419	0.197791
hsa_circ_	0.321908	0.197801
hsa_circ_	0.290286	0.19781
hsa_circ_	0.218282	0.197828
hsa_circ_	-0.22377	0.19783
hsa_circ_	-0.65886	0.197833
hsa_circ_	0.362933	0.19784
hsa_circ_	-0.21831	0.197877
hsa_circ_	-0.28872	0.197887
hsa_circ_	-0.43265	0.197899
hsa_circ_	-0.39654	0.197924
hsa_circ_	0.233739	0.197933
hsa_circ_	0.20604	0.197941
hsa_circ_	-0.2667	0.197942
hsa_circ_	0.271855	0.197946

hsa_circ_	0.401473	0.19796
hsa_circ_	0.352687	0.197972
hsa_circ_	0.338633	0.197977
hsa_circ_	-0.23185	0.197979
hsa_circ_	-0.65344	0.197982
hsa_circ_	0.240089	0.197991
hsa_circ_	-0.38131	0.198007
hsa_circ_	-0.34505	0.198019
hsa_circ_	-0.31656	0.19802
hsa_circ_	-0.24661	0.198025
hsa_circ_	-0.20728	0.198032
hsa_circ_	0.637204	0.198042
hsa_circ_	0.449025	0.198056
hsa_circ_	-0.56615	0.198066
hsa_circ_	-0.35994	0.198074
hsa_circ_	0.240573	0.198077
hsa_circ_	-0.31268	0.198078
hsa_circ_	0.260668	0.198081
hsa_circ_	0.350349	0.198086
hsa_circ_	-0.38611	0.198089
hsa_circ_	-0.57748	0.19809
hsa_circ_	0.326102	0.198101
hsa_circ_	0.551459	0.198118
hsa_circ_	-0.18255	0.198134
hsa_circ_	-0.20045	0.198158
hsa_circ_	0.350416	0.198165
hsa_circ_	-0.39196	0.198168
hsa_circ_	-0.44039	0.19817
hsa_circ_	-0.21862	0.198176
hsa_circ_	0.258186	0.198176
hsa_circ_	-0.2398	0.198179
hsa_circ_	-0.193	0.198181
hsa_circ_	-0.80511	0.198187
hsa_circ_	0.268005	0.198208
hsa_circ_	0.505221	0.198219
hsa_circ_	0.326751	0.198223
hsa_circ_	0.743588	0.198225
hsa_circ_	0.321361	0.198227
hsa_circ_	0.208913	0.198228
hsa_circ_	0.790847	0.198228
hsa_circ_	-0.21618	0.198229
hsa_circ_	-0.20002	0.198231
hsa_circ_	-0.25065	0.198236
hsa_circ_	0.245466	0.198251
hsa_circ_	-0.19696	0.198251
hsa_circ_	0.378508	0.198259
hsa_circ_	0.440718	0.198265
hsa_circ_	-0.38634	0.198272
hsa_circ_	-0.39111	0.198277
hsa_circ_	0.440523	0.198279
hsa_circ_	0.604415	0.198283
hsa_circ_	-0.23018	0.198283
hsa_circ_	-0.37582	0.198285
hsa_circ_	0.28724	0.198285

hsa_circ_	-0.20244	0.198293
hsa_circ_	0.331707	0.198332
hsa_circ_	0.442345	0.198341
hsa_circ_	-0.24598	0.198358
hsa_circ_	-0.26775	0.19838
hsa_circ_	-0.19656	0.198393
hsa_circ_	-0.31294	0.198396
hsa_circ_	0.372781	0.198396
hsa_circ_	0.672372	0.198401
hsa_circ_	-0.20163	0.198406
hsa_circ_	-0.20306	0.198406
hsa_circ_	0.2214	0.19841
hsa_circ_	0.747794	0.198432
hsa_circ_	-0.59347	0.198433
hsa_circ_	-0.63272	0.198434
hsa_circ_	1.471692	0.19845
hsa_circ_	-0.47533	0.198451
hsa_circ_	-0.30433	0.19846
hsa_circ_	-0.28593	0.198465
hsa_circ_	0.659903	0.198473
hsa_circ_	0.87171	0.198474
hsa_circ_	0.225436	0.198483
hsa_circ_	0.432404	0.198489
hsa_circ_	0.448858	0.198494
hsa_circ_	-0.22604	0.198496
hsa_circ_	0.228461	0.198507
hsa_circ_	0.923613	0.198562
hsa_circ_	-0.27768	0.198565
hsa_circ_	-0.18667	0.198573
hsa_circ_	0.220002	0.198583
hsa_circ_	0.659345	0.1986
hsa_circ_	-0.25206	0.198617
hsa_circ_	0.294587	0.19863
hsa_circ_	-0.48136	0.198637
hsa_circ_	-0.39312	0.198659
hsa_circ_	-0.20052	0.198689
hsa_circ_	0.301201	0.198692
hsa_circ_	-0.31332	0.198702
hsa_circ_	-0.20721	0.198746
hsa_circ_	1.00862	0.198758
hsa_circ_	-0.25298	0.19876
hsa_circ_	0.225367	0.198761
hsa_circ_	-0.85446	0.198767
hsa_circ_	-0.19245	0.198778
hsa_circ_	0.252403	0.198801
hsa_circ_	0.298259	0.198804
hsa_circ_	-0.23154	0.198808
hsa_circ_	0.247611	0.198846
hsa_circ_	-0.72281	0.198851
hsa_circ_	-0.23723	0.198853
hsa_circ_	0.278203	0.198858
hsa_circ_	0.225245	0.198861
hsa_circ_	0.372117	0.198862
hsa_circ_	0.314197	0.19888

hsa_circ_	0.190707	0.198887
hsa_circ_	0.808408	0.198892
hsa_circ_	-0.27015	0.198916
hsa_circ_	-0.55408	0.198927
hsa_circ_	0.375243	0.198942
hsa_circ_	0.295537	0.198946
hsa_circ_	0.346468	0.198964
hsa_circ_	-0.43849	0.198971
hsa_circ_	-0.30589	0.198972
hsa_circ_	0.28505	0.198976
hsa_circ_	0.754261	0.198981
hsa_circ_	-0.45591	0.198982
hsa_circ_	-0.31399	0.198983
hsa_circ_	-0.37222	0.198985
hsa_circ_	-0.71166	0.198986
hsa_circ_	-0.29615	0.198988
hsa_circ_	-0.53666	0.198996
hsa_circ_	0.430137	0.198999
hsa_circ_	-0.22951	0.199003
hsa_circ_	0.220849	0.199009
hsa_circ_	0.205874	0.199021
hsa_circ_	0.242077	0.199023
hsa_circ_	0.373384	0.199035
hsa_circ_	-0.2103	0.199041
hsa_circ_	-0.21591	0.199065
hsa_circ_	0.265082	0.199076
hsa_circ_	0.223072	0.199083
hsa_circ_	-0.80375	0.199096
hsa_circ_	0.374747	0.199098
hsa_circ_	-0.25625	0.199101
hsa_circ_	-0.20036	0.199113
hsa_circ_	-0.20934	0.199117
hsa_circ_	0.955932	0.199122
hsa_circ_	-0.66924	0.199125
hsa_circ_	0.340028	0.199129
hsa_circ_	-0.27514	0.199131
hsa_circ_	-0.84528	0.199138
hsa_circ_	0.23466	0.199154
hsa_circ_	0.367878	0.199158
hsa_circ_	-0.68049	0.199163
hsa_circ_	-0.28385	0.199165
hsa_circ_	0.241986	0.199184
hsa_circ_	0.276419	0.199184
hsa_circ_	0.211243	0.199186
hsa_circ_	-0.24618	0.199192
hsa_circ_	0.319136	0.199203
hsa_circ_	0.412615	0.199208
hsa_circ_	0.316789	0.199208
hsa_circ_	-0.18305	0.199212
hsa_circ_	0.447757	0.199218
hsa_circ_	0.261206	0.199226
hsa_circ_	-0.33508	0.199229
hsa_circ_	-0.23417	0.19924
hsa_circ_	0.807092	0.199241

hsa_circ_	-0.60244	0.199251
hsa_circ_	0.190519	0.199256
hsa_circ_	-0.28649	0.199306
hsa_circ_	0.234489	0.199311
hsa_circ_	-0.21725	0.199325
hsa_circ_	0.456576	0.199327
hsa_circ_	-0.31047	0.199328
hsa_circ_	0.20449	0.199331
hsa_circ_	-0.25302	0.199336
hsa_circ_	0.324789	0.199337
hsa_circ_	-0.82635	0.199339
hsa_circ_	0.204344	0.199342
hsa_circ_	-0.29022	0.199343
hsa_circ_	0.25185	0.199351
hsa_circ_	0.196374	0.199356
hsa_circ_	0.362894	0.19937
hsa_circ_	0.35096	0.199373
hsa_circ_	-1.64603	0.199377
hsa_circ_	-0.28414	0.199379
hsa_circ_	-0.85151	0.199381
hsa_circ_	0.203776	0.199382
hsa_circ_	0.461133	0.199394
hsa_circ_	0.20909	0.199395
hsa_circ_	0.500204	0.199402
hsa_circ_	0.277151	0.199404
hsa_circ_	0.494297	0.199415
hsa_circ_	-0.71519	0.199421
hsa_circ_	-2.17526	0.199439
hsa_circ_	0.315119	0.199444
hsa_circ_	0.225224	0.199454
hsa_circ_	-0.22756	0.19946
hsa_circ_	0.187754	0.199462
hsa_circ_	-0.43497	0.199469
hsa_circ_	-0.73385	0.199496
hsa_circ_	0.340299	0.199505
hsa_circ_	0.178601	0.19952
hsa_circ_	-0.20274	0.199526
hsa_circ_	0.211114	0.199528
hsa_circ_	-0.62786	0.199544
hsa_circ_	0.506175	0.199563
hsa_circ_	-3.9595	0.199576
hsa_circ_	-0.31985	0.199576
hsa_circ_	0.256471	0.19959
hsa_circ_	-0.41504	0.199592
hsa_circ_	0.411314	0.199624
hsa_circ_	0.291205	0.199634
hsa_circ_	-0.33113	0.199634
hsa_circ_	-0.18434	0.199639
hsa_circ_	-0.19773	0.199648
hsa_circ_	0.592775	0.199689
hsa_circ_	0.605988	0.199707
hsa_circ_	0.289508	0.199713
hsa_circ_	0.398899	0.199723
hsa_circ_	0.221642	0.199731

hsa_circ_	0.224767	0.199777
hsa_circ_	0.284983	0.199779
hsa_circ_	-0.55151	0.199783
hsa_circ_	0.203444	0.199797
hsa_circ_	0.237551	0.199811
hsa_circ_	0.291613	0.199839
hsa_circ_	-0.30021	0.19984
hsa_circ_	0.211043	0.199846
hsa_circ_	0.237114	0.199871
hsa_circ_	-0.33874	0.199879
hsa_circ_	-0.27931	0.199887
hsa_circ_	0.73668	0.199891
hsa_circ_	0.483863	0.199892
hsa_circ_	-0.20656	0.199916
hsa_circ_	-0.18203	0.199923
hsa_circ_	0.214543	0.199947
hsa_circ_	0.812364	0.199948
hsa_circ_	0.434037	0.199954
hsa_circ_	-0.27406	0.19996
hsa_circ_	-0.71555	0.199977
hsa_circ_	-0.42902	0.199982
hsa_circ_	0.270372	0.199988
hsa_circ_	-0.17741	0.200017
hsa_circ_	0.848041	0.200028
hsa_circ_	-0.22384	0.200038
hsa_circ_	-0.25241	0.20004
hsa_circ_	0.303961	0.200042
hsa_circ_	0.237721	0.200052
hsa_circ_	-0.30617	0.200053
hsa_circ_	0.47518	0.200064
hsa_circ_	-0.2255	0.200071
hsa_circ_	-0.19487	0.200079
hsa_circ_	-0.55655	0.200084
hsa_circ_	-0.18794	0.200084
hsa_circ_	0.758841	0.200098
hsa_circ_	-0.22855	0.200098
hsa_circ_	-0.63776	0.200125
hsa_circ_	-0.31466	0.200128
hsa_circ_	-0.58156	0.200139
hsa_circ_	-0.34306	0.200142
hsa_circ_	-0.24572	0.200142
hsa_circ_	-0.69433	0.200152
hsa_circ_	-0.73034	0.200163
hsa_circ_	0.213792	0.200164
hsa_circ_	-1.18501	0.200171
hsa_circ_	-0.30876	0.200174
hsa_circ_	-0.20608	0.200193
hsa_circ_	0.820798	0.200208
hsa_circ_	-0.66994	0.200213
hsa_circ_	-0.48771	0.200231
hsa_circ_	0.236317	0.200262
hsa_circ_	0.807915	0.200272
hsa_circ_	0.276518	0.200284
hsa_circ_	-0.35635	0.200285

hsa_circ_	0.268584	0.2003
hsa_circ_	-0.30089	0.200329
hsa_circ_	-0.20883	0.200331
hsa_circ_	0.334535	0.200343
hsa_circ_	0.41915	0.200344
hsa_circ_	-1.12127	0.200344
hsa_circ_	0.284189	0.20037
hsa_circ_	-0.53592	0.200376
hsa_circ_	-0.86211	0.200378
hsa_circ_	0.323512	0.200392
hsa_circ_	0.202773	0.200396
hsa_circ_	0.318434	0.200416
hsa_circ_	-0.68941	0.200418
hsa_circ_	-0.32013	0.200419
hsa_circ_	-0.28933	0.200429
hsa_circ_	-0.41495	0.200433
hsa_circ_	0.366716	0.200442
hsa_circ_	-0.21938	0.20046
hsa_circ_	-0.40575	0.20046
hsa_circ_	-0.28588	0.20049
hsa_circ_	-0.21532	0.200501
hsa_circ_	-0.48528	0.200503
hsa_circ_	-0.2777	0.200521
hsa_circ_	0.239809	0.200526
hsa_circ_	0.32836	0.200532
hsa_circ_	0.268908	0.200545
hsa_circ_	-0.37563	0.200564
hsa_circ_	0.188556	0.200568
hsa_circ_	-0.49209	0.200588
hsa_circ_	-0.91846	0.200608
hsa_circ_	-0.25151	0.200608
hsa_circ_	-0.26088	0.200642
hsa_circ_	0.248045	0.20065
hsa_circ_	0.816239	0.200662
hsa_circ_	0.238577	0.200667
hsa_circ_	-0.34381	0.200682
hsa_circ_	-0.30657	0.200691
hsa_circ_	-0.46644	0.200692
hsa_circ_	-1.10122	0.20072
hsa_circ_	-0.33342	0.20072
hsa_circ_	-0.37497	0.200726
hsa_circ_	-0.21712	0.200726
hsa_circ_	-0.27904	0.200726
hsa_circ_	0.444525	0.200728
hsa_circ_	-0.35321	0.200744
hsa_circ_	0.221066	0.200744
hsa_circ_	0.36325	0.200744
hsa_circ_	-1.64122	0.200759
hsa_circ_	0.285475	0.200764
hsa_circ_	-0.22524	0.200769
hsa_circ_	0.213896	0.200777
hsa_circ_	-0.49059	0.20079
hsa_circ_	0.707393	0.200824
hsa_circ_	-0.277	0.200833

hsa_circ_	-0.55784	0.200853
hsa_circ_	-0.27114	0.200874
hsa_circ_	-0.29418	0.200892
hsa_circ_	-0.595	0.200928
hsa_circ_	0.563108	0.200938
hsa_circ_	0.208315	0.200939
hsa_circ_	-0.2059	0.200953
hsa_circ_	0.347117	0.200955
hsa_circ_	-0.17694	0.200962
hsa_circ_	-0.74325	0.200993
hsa_circ_	-0.44145	0.200995
hsa_circ_	-0.88863	0.200999
hsa_circ_	0.819555	0.20101
hsa_circ_	0.39577	0.201012
hsa_circ_	-0.25275	0.201026
hsa_circ_	-0.23916	0.201026
hsa_circ_	0.655227	0.201035
hsa_circ_	0.482109	0.201047
hsa_circ_	-0.18347	0.201053
hsa_circ_	0.236269	0.201084
hsa_circ_	-0.27017	0.201093
hsa_circ_	-0.56066	0.201094
hsa_circ_	-0.47785	0.20112
hsa_circ_	-0.24651	0.201122
hsa_circ_	-0.23109	0.201131
hsa_circ_	-0.5942	0.201146
hsa_circ_	1.14266	0.201159
hsa_circ_	-0.44139	0.201159
hsa_circ_	-0.43468	0.201161
hsa_circ_	-0.20137	0.201165
hsa_circ_	-0.20464	0.201165
hsa_circ_	0.220392	0.201193
hsa_circ_	-0.19592	0.201199
hsa_circ_	-0.17983	0.201211
hsa_circ_	0.437945	0.201227
hsa_circ_	-0.24408	0.201229
hsa_circ_	-0.33773	0.201229
hsa_circ_	-0.1877	0.20123
hsa_circ_	-0.25567	0.201248
hsa_circ_	0.221397	0.201268
hsa_circ_	0.269399	0.201284
hsa_circ_	0.226514	0.201311
hsa_circ_	0.214864	0.201313
hsa_circ_	-0.27477	0.201315
hsa_circ_	-0.45609	0.201323
hsa_circ_	0.702305	0.201334
hsa_circ_	-0.37892	0.201336
hsa_circ_	0.742168	0.20134
hsa_circ_	-0.4152	0.201347
hsa_circ_	-0.22525	0.201354
hsa_circ_	-0.30318	0.201356
hsa_circ_	-0.20156	0.201363
hsa_circ_	-0.21165	0.201375
hsa_circ_	-0.61984	0.20139

hsa_circ_	-0.28493	0.201416
hsa_circ_	-0.37522	0.201499
hsa_circ_	-0.67257	0.201508
hsa_circ_	-0.51649	0.201511
hsa_circ_	-0.24418	0.201522
hsa_circ_	-0.18251	0.201526
hsa_circ_	0.973145	0.201526
hsa_circ_	-0.20532	0.201527
hsa_circ_	-0.22704	0.201562
hsa_circ_	-0.32114	0.201568
hsa_circ_	-0.52078	0.201569
hsa_circ_	-0.25358	0.201571
hsa_circ_	0.292319	0.201581
hsa_circ_	-0.54598	0.201583
hsa_circ_	-0.24026	0.20159
hsa_circ_	0.294638	0.201593
hsa_circ_	-0.86961	0.201596
hsa_circ_	0.280464	0.201616
hsa_circ_	-0.344	0.201634
hsa_circ_	-0.36565	0.201637
hsa_circ_	-0.52224	0.201642
hsa_circ_	0.486964	0.201647
hsa_circ_	0.604197	0.201647
hsa_circ_	0.321721	0.201663
hsa_circ_	-0.30764	0.201675
hsa_circ_	-0.37525	0.201676
hsa_circ_	0.430874	0.201702
hsa_circ_	-1.26531	0.201712
hsa_circ_	0.215465	0.20174
hsa_circ_	-0.23655	0.201741
hsa_circ_	-0.9413	0.201747
hsa_circ_	-0.38396	0.201771
hsa_circ_	-0.67879	0.201783
hsa_circ_	-0.78665	0.201787
hsa_circ_	-0.30916	0.201796
hsa_circ_	0.271472	0.201797
hsa_circ_	0.368717	0.201806
hsa_circ_	-0.34551	0.201806
hsa_circ_	0.269661	0.201809
hsa_circ_	-0.26674	0.201814
hsa_circ_	0.341381	0.201835
hsa_circ_	0.267577	0.201868
hsa_circ_	0.246667	0.201869
hsa_circ_	0.283179	0.201879
hsa_circ_	0.260356	0.201884
hsa_circ_	-0.70426	0.201894
hsa_circ_	-0.36973	0.201899
hsa_circ_	-0.56602	0.201901
hsa_circ_	0.359714	0.201903
hsa_circ_	-1.43572	0.20191
hsa_circ_	-0.55563	0.201912
hsa_circ_	-0.58116	0.201921
hsa_circ_	0.181157	0.201925
hsa_circ_	0.286028	0.201929

hsa_circ_	-0.19611	0.201932
hsa_circ_	0.183416	0.201934
hsa_circ_	-0.4662	0.201934
hsa_circ_	-0.22518	0.201943
hsa_circ_	0.177741	0.201945
hsa_circ_	0.326783	0.201949
hsa_circ_	0.696033	0.20196
hsa_circ_	0.490976	0.20197
hsa_circ_	0.494777	0.201978
hsa_circ_	0.263203	0.201984
hsa_circ_	-0.20786	0.201984
hsa_circ_	0.248742	0.202007
hsa_circ_	0.243019	0.202009
hsa_circ_	-0.72404	0.20202
hsa_circ_	-0.37423	0.202048
hsa_circ_	-0.22659	0.202057
hsa_circ_	-0.18912	0.202062
hsa_circ_	0.24695	0.202076
hsa_circ_	0.251339	0.202077
hsa_circ_	-0.2431	0.202082
hsa_circ_	-0.20171	0.202083
hsa_circ_	-0.26407	0.202086
hsa_circ_	0.212781	0.202091
hsa_circ_	-0.22829	0.202092
hsa_circ_	0.249045	0.202116
hsa_circ_	0.891442	0.202117
hsa_circ_	-0.28623	0.20213
hsa_circ_	-0.50189	0.202154
hsa_circ_	0.855228	0.202166
hsa_circ_	0.180982	0.202174
hsa_circ_	0.198931	0.202174
hsa_circ_	-0.35295	0.202175
hsa_circ_	-0.22808	0.202183
hsa_circ_	-0.17854	0.202195
hsa_circ_	-0.47133	0.202206
hsa_circ_	-0.25899	0.202206
hsa_circ_	-0.18397	0.202207
hsa_circ_	0.354025	0.20222
hsa_circ_	-0.27837	0.202227
hsa_circ_	0.289201	0.202242
hsa_circ_	-0.19857	0.202244
hsa_circ_	-0.29042	0.202245
hsa_circ_	0.240973	0.202251
hsa_circ_	-0.26323	0.20226
hsa_circ_	-0.61792	0.202277
hsa_circ_	0.83865	0.202297
hsa_circ_	-0.25284	0.202304
hsa_circ_	0.301783	0.202336
hsa_circ_	0.345152	0.202345
hsa_circ_	-0.21898	0.202355
hsa_circ_	0.514021	0.202359
hsa_circ_	-0.21788	0.20236
hsa_circ_	-0.46675	0.202379
hsa_circ_	-0.21537	0.202412

hsa_circ_	-0.19928	0.202455
hsa_circ_	0.239507	0.20246
hsa_circ_	-0.29907	0.202462
hsa_circ_	0.213466	0.202469
hsa_circ_	-0.71585	0.202478
hsa_circ_	1.02972	0.202487
hsa_circ_	0.256213	0.202487
hsa_circ_	0.888544	0.202504
hsa_circ_	-0.55532	0.202514
hsa_circ_	-0.17295	0.202514
hsa_circ_	-0.21406	0.202514
hsa_circ_	0.217738	0.202524
hsa_circ_	-0.20522	0.202533
hsa_circ_	-0.339	0.202555
hsa_circ_	0.639754	0.202555
hsa_circ_	0.358245	0.202558
hsa_circ_	0.232453	0.202562
hsa_circ_	0.410483	0.202573
hsa_circ_	0.227379	0.202596
hsa_circ_	0.272271	0.202598
hsa_circ_	0.226699	0.202606
hsa_circ_	-0.32067	0.202642
hsa_circ_	0.242405	0.202647
hsa_circ_	-0.51447	0.202649
hsa_circ_	-0.2721	0.202649
hsa_circ_	-0.21806	0.202654
hsa_circ_	-0.38403	0.202655
hsa_circ_	-0.19932	0.202661
hsa_circ_	-0.26388	0.202665
hsa_circ_	0.241691	0.202671
hsa_circ_	0.236932	0.202675
hsa_circ_	0.311849	0.202704
hsa_circ_	-0.21269	0.202719
hsa_circ_	0.422992	0.202721
hsa_circ_	-0.45024	0.202732
hsa_circ_	-0.19621	0.202742
hsa_circ_	0.196715	0.202744
hsa_circ_	0.275132	0.202744
hsa_circ_	0.608122	0.202761
hsa_circ_	0.248942	0.202769
hsa_circ_	0.289076	0.202785
hsa_circ_	0.379973	0.202793
hsa_circ_	0.264083	0.202798
hsa_circ_	-0.21661	0.202805
hsa_circ_	0.41428	0.202819
hsa_circ_	-0.19777	0.202832
hsa_circ_	0.205243	0.202835
hsa_circ_	0.378516	0.202853
hsa_circ_	-0.24511	0.202859
hsa_circ_	-0.19295	0.202862
hsa_circ_	-0.17719	0.202867
hsa_circ_	0.239508	0.20287
hsa_circ_	0.236329	0.20288
hsa_circ_	0.198109	0.202888

hsa_circ_	-0.27641	0.202909
hsa_circ_	-0.20213	0.20291
hsa_circ_	-1.06118	0.20291
hsa_circ_	0.388239	0.202926
hsa_circ_	0.82347	0.202941
hsa_circ_	-0.54616	0.202943
hsa_circ_	0.328909	0.202945
hsa_circ_	-0.24409	0.202968
hsa_circ_	0.601035	0.202969
hsa_circ_	-0.84113	0.202983
hsa_circ_	-0.49582	0.202983
hsa_circ_	-0.17806	0.20301
hsa_circ_	0.318983	0.203016
hsa_circ_	-0.37843	0.203019
hsa_circ_	-0.24184	0.203026
hsa_circ_	0.33141	0.203033
hsa_circ_	-0.45493	0.203038
hsa_circ_	0.45782	0.203038
hsa_circ_	0.371286	0.20304
hsa_circ_	-0.25297	0.203041
hsa_circ_	0.598954	0.203043
hsa_circ_	0.214819	0.203052
hsa_circ_	-0.29728	0.203083
hsa_circ_	0.238981	0.203094
hsa_circ_	-0.32252	0.203095
hsa_circ_	0.232286	0.203106
hsa_circ_	1.064849	0.203108
hsa_circ_	-0.21253	0.203121
hsa_circ_	-0.68754	0.203132
hsa_circ_	0.270772	0.203143
hsa_circ_	-0.39907	0.203148
hsa_circ_	-0.26305	0.203153
hsa_circ_	-0.17748	0.203183
hsa_circ_	-0.3405	0.203188
hsa_circ_	0.343104	0.203193
hsa_circ_	0.454901	0.203193
hsa_circ_	-0.21791	0.203202
hsa_circ_	0.225201	0.203202
hsa_circ_	-0.81704	0.203211
hsa_circ_	-0.23887	0.203228
hsa_circ_	0.289754	0.203232
hsa_circ_	-0.24803	0.203252
hsa_circ_	0.223096	0.20328
hsa_circ_	0.380963	0.203308
hsa_circ_	0.415985	0.203322
hsa_circ_	0.251755	0.203327
hsa_circ_	-0.17069	0.203367
hsa_circ_	-0.18724	0.203379
hsa_circ_	-0.19863	0.203382
hsa_circ_	-0.20751	0.203403
hsa_circ_	-0.61741	0.203405
hsa_circ_	-0.57976	0.203416
hsa_circ_	0.354014	0.203421
hsa_circ_	0.30735	0.203442

hsa_circ_	-0.30564	0.203457
hsa_circ_	0.666227	0.203461
hsa_circ_	0.385016	0.203475
hsa_circ_	0.420937	0.203484
hsa_circ_	0.388964	0.203485
hsa_circ_	0.219577	0.203488
hsa_circ_	-0.63894	0.203497
hsa_circ_	-0.16884	0.203511
hsa_circ_	-0.18745	0.203528
hsa_circ_	-0.4413	0.203541
hsa_circ_	-0.18111	0.203542
hsa_circ_	0.285634	0.203543
hsa_circ_	-0.87869	0.203553
hsa_circ_	0.344865	0.203555
hsa_circ_	0.447927	0.203579
hsa_circ_	0.318528	0.203584
hsa_circ_	-0.19371	0.203597
hsa_circ_	0.357966	0.203597
hsa_circ_	0.481336	0.203598
hsa_circ_	-0.45988	0.203601
hsa_circ_	-0.30794	0.203602
hsa_circ_	0.308614	0.203607
hsa_circ_	-0.24861	0.203608
hsa_circ_	-0.20903	0.203617
hsa_circ_	0.396028	0.203636
hsa_circ_	-0.49135	0.20365
hsa_circ_	-0.28194	0.203653
hsa_circ_	0.554445	0.203657
hsa_circ_	-0.31088	0.203664
hsa_circ_	-0.23998	0.203665
hsa_circ_	-0.2482	0.20374
hsa_circ_	-0.36713	0.203769
hsa_circ_	-0.30001	0.203783
hsa_circ_	-0.34784	0.203802
hsa_circ_	0.335546	0.203815
hsa_circ_	-0.19137	0.203818
hsa_circ_	-0.19193	0.203819
hsa_circ_	0.252721	0.203825
hsa_circ_	-0.93651	0.203832
hsa_circ_	0.974367	0.203836
hsa_circ_	-0.79635	0.203845
hsa_circ_	-0.23349	0.203854
hsa_circ_	0.328925	0.20386
hsa_circ_	0.827437	0.203901
hsa_circ_	0.304579	0.203907
hsa_circ_	0.240915	0.203925
hsa_circ_	0.253943	0.203934
hsa_circ_	-0.25886	0.203934
hsa_circ_	0.242196	0.203939
hsa_circ_	-0.31838	0.203942
hsa_circ_	-0.30337	0.203947
hsa_circ_	-0.29585	0.203958
hsa_circ_	-0.29436	0.203958
hsa_circ_	0.614862	0.203964

hsa_circ_	-0.44622	0.203964
hsa_circ_	-0.29193	0.203965
hsa_circ_	0.343348	0.203966
hsa_circ_	0.270305	0.203973
hsa_circ_	-0.18367	0.203975
hsa_circ_	0.220854	0.203988
hsa_circ_	0.195978	0.203994
hsa_circ_	-0.71575	0.204004
hsa_circ_	-0.6089	0.204022
hsa_circ_	0.181632	0.20403
hsa_circ_	-0.23525	0.204042
hsa_circ_	-0.78885	0.20405
hsa_circ_	-0.23328	0.204055
hsa_circ_	-0.26015	0.204061
hsa_circ_	0.183895	0.204071
hsa_circ_	0.19917	0.204078
hsa_circ_	-0.28023	0.204091
hsa_circ_	-0.18599	0.204097
hsa_circ_	-0.76906	0.204099
hsa_circ_	0.252237	0.204121
hsa_circ_	-0.31548	0.204131
hsa_circ_	0.243362	0.204141
hsa_circ_	-0.31928	0.204148
hsa_circ_	0.210448	0.204153
hsa_circ_	1.037861	0.204192
hsa_circ_	0.217571	0.204204
hsa_circ_	0.244202	0.204207
hsa_circ_	-0.5589	0.204208
hsa_circ_	-0.56806	0.20421
hsa_circ_	-0.37747	0.204231
hsa_circ_	-0.19766	0.20425
hsa_circ_	0.662156	0.204275
hsa_circ_	0.215765	0.20428
hsa_circ_	-0.38585	0.204296
hsa_circ_	-0.35793	0.204301
hsa_circ_	0.622566	0.204305
hsa_circ_	-0.32179	0.204317
hsa_circ_	-0.40447	0.204321
hsa_circ_	-0.28027	0.204328
hsa_circ_	0.991277	0.204347
hsa_circ_	-0.31695	0.204365
hsa_circ_	0.216167	0.20437
hsa_circ_	-0.28652	0.20437
hsa_circ_	-1.53904	0.20438
hsa_circ_	0.314372	0.20439
hsa_circ_	-0.44171	0.20442
hsa_circ_	-0.29714	0.204436
hsa_circ_	0.300903	0.204437
hsa_circ_	0.230038	0.204438
hsa_circ_	0.235082	0.204445
hsa_circ_	0.188012	0.204451
hsa_circ_	0.200572	0.204457
hsa_circ_	-0.59717	0.20449
hsa_circ_	-0.18322	0.204535

hsa_circ_	-0.3109	0.204541
hsa_circ_	-0.21518	0.204562
hsa_circ_	-0.29802	0.204564
hsa_circ_	0.441249	0.20457
hsa_circ_	0.478577	0.204573
hsa_circ_	-0.2298	0.204596
hsa_circ_	-0.25838	0.204609
hsa_circ_	-0.28296	0.20461
hsa_circ_	-0.63647	0.204656
hsa_circ_	0.242246	0.204684
hsa_circ_	-0.45152	0.204689
hsa_circ_	-0.26429	0.204707
hsa_circ_	0.801945	0.204709
hsa_circ_	0.34437	0.204722
hsa_circ_	-0.21908	0.204723
hsa_circ_	0.251929	0.204731
hsa_circ_	0.550242	0.204732
hsa_circ_	-0.27681	0.204739
hsa_circ_	0.303026	0.204767
hsa_circ_	-0.1816	0.204768
hsa_circ_	0.603128	0.204779
hsa_circ_	-0.5731	0.204786
hsa_circ_	0.197341	0.204794
hsa_circ_	0.449979	0.204802
hsa_circ_	-0.38722	0.204803
hsa_circ_	0.25559	0.204823
hsa_circ_	0.241421	0.204854
hsa_circ_	-0.62456	0.204872
hsa_circ_	-0.63951	0.204876
hsa_circ_	0.29165	0.204882
hsa_circ_	-0.66588	0.204892
hsa_circ_	-0.25628	0.204902
hsa_circ_	0.600622	0.204911
hsa_circ_	-0.2507	0.204919
hsa_circ_	0.289117	0.204922
hsa_circ_	0.254109	0.204927
hsa_circ_	-0.23163	0.204932
hsa_circ_	-0.18526	0.204956
hsa_circ_	0.287047	0.205018
hsa_circ_	-0.35903	0.205047
hsa_circ_	-0.53629	0.205056
hsa_circ_	0.273108	0.205066
hsa_circ_	-0.50392	0.20509
hsa_circ_	0.231914	0.205104
hsa_circ_	-0.25817	0.205115
hsa_circ_	0.20629	0.205117
hsa_circ_	-1.08733	0.205122
hsa_circ_	-0.50833	0.205137
hsa_circ_	0.390385	0.205155
hsa_circ_	0.771108	0.205159
hsa_circ_	-0.29587	0.205164
hsa_circ_	0.174639	0.205167
hsa_circ_	-0.50528	0.205205
hsa_circ_	-1.01685	0.20521

hsa_circ_	0.328744	0.205222
hsa_circ_	-0.27393	0.205226
hsa_circ_	-0.41229	0.205226
hsa_circ_	-0.45517	0.205231
hsa_circ_	-0.21609	0.20525
hsa_circ_	-0.37955	0.205258
hsa_circ_	0.252616	0.205278
hsa_circ_	0.291508	0.205292
hsa_circ_	-0.2056	0.205293
hsa_circ_	0.319118	0.205302
hsa_circ_	0.178049	0.205307
hsa_circ_	0.260113	0.205319
hsa_circ_	0.409614	0.205321
hsa_circ_	-1.23225	0.205322
hsa_circ_	-0.23813	0.205325
hsa_circ_	-0.17321	0.205328
hsa_circ_	0.520738	0.20533
hsa_circ_	0.308168	0.205334
hsa_circ_	-0.21793	0.205341
hsa_circ_	-0.25213	0.205358
hsa_circ_	0.336484	0.205372
hsa_circ_	0.415142	0.205388
hsa_circ_	0.359737	0.205391
hsa_circ_	0.643345	0.205402
hsa_circ_	-0.2048	0.205406
hsa_circ_	-0.42057	0.205407
hsa_circ_	-0.93069	0.205407
hsa_circ_	-0.21986	0.205408
hsa_circ_	-0.22153	0.205429
hsa_circ_	0.994273	0.20543
hsa_circ_	-0.20986	0.20545
hsa_circ_	0.234058	0.205453
hsa_circ_	-0.19724	0.205476
hsa_circ_	-0.19427	0.205492
hsa_circ_	-0.21661	0.205497
hsa_circ_	0.196242	0.205498
hsa_circ_	-0.19128	0.205519
hsa_circ_	0.586332	0.205531
hsa_circ_	-0.18774	0.20554
hsa_circ_	-0.17933	0.205543
hsa_circ_	-0.55178	0.205544
hsa_circ_	0.267383	0.20555
hsa_circ_	-0.37281	0.205567
hsa_circ_	0.378781	0.205573
hsa_circ_	0.348944	0.205585
hsa_circ_	-0.18033	0.205594
hsa_circ_	-0.23456	0.205623
hsa_circ_	-0.17463	0.20564
hsa_circ_	-0.665	0.205641
hsa_circ_	0.39058	0.205644
hsa_circ_	0.444392	0.205644
hsa_circ_	0.292829	0.20565
hsa_circ_	0.366896	0.205651
hsa_circ_	-0.31095	0.205657

hsa_circ_	0.408589	0.20566
hsa_circ_	-0.38241	0.205687
hsa_circ_	-0.2445	0.205696
hsa_circ_	0.209014	0.2057
hsa_circ_	0.39004	0.205701
hsa_circ_	0.929791	0.205701
hsa_circ_	-0.27469	0.205701
hsa_circ_	0.285605	0.205704
hsa_circ_	0.213297	0.205726
hsa_circ_	0.211761	0.205727
hsa_circ_	0.354989	0.205737
hsa_circ_	0.245232	0.205738
hsa_circ_	0.238231	0.205756
hsa_circ_	-0.29697	0.205775
hsa_circ_	-0.24682	0.205783
hsa_circ_	-0.2076	0.205798
hsa_circ_	0.547955	0.205811
hsa_circ_	-0.22501	0.205817
hsa_circ_	-0.19215	0.205821
hsa_circ_	-0.17387	0.205828
hsa_circ_	0.694304	0.205838
hsa_circ_	0.308246	0.205848
hsa_circ_	1.297641	0.205852
hsa_circ_	-0.41219	0.205855
hsa_circ_	0.714591	0.205856
hsa_circ_	-0.28719	0.205901
hsa_circ_	0.241478	0.205913
hsa_circ_	-0.67731	0.205932
hsa_circ_	0.224662	0.205948
hsa_circ_	0.344877	0.205959
hsa_circ_	-0.53263	0.206018
hsa_circ_	-0.44601	0.206031
hsa_circ_	-0.51487	0.206036
hsa_circ_	0.193982	0.20605
hsa_circ_	-0.30821	0.206051
hsa_circ_	0.254761	0.206054
hsa_circ_	-0.48088	0.206054
hsa_circ_	-0.33191	0.206063
hsa_circ_	-0.26523	0.206063
hsa_circ_	-0.19541	0.206069
hsa_circ_	-0.25387	0.206076
hsa_circ_	0.274668	0.206078
hsa_circ_	0.197517	0.20608
hsa_circ_	-0.22956	0.206118
hsa_circ_	0.320692	0.206124
hsa_circ_	0.1852	0.206126
hsa_circ_	0.40971	0.20614
hsa_circ_	-0.41269	0.206157
hsa_circ_	-0.74309	0.206194
hsa_circ_	0.178269	0.20621
hsa_circ_	0.403992	0.20621
hsa_circ_	-0.56207	0.206212
hsa_circ_	-0.27316	0.206226
hsa_circ_	0.369649	0.206238

hsa_circ_	0.311187	0.206261
hsa_circ_	-0.50649	0.206275
hsa_circ_	-0.39343	0.206282
hsa_circ_	-0.26968	0.206305
hsa_circ_	-0.39303	0.206307
hsa_circ_	0.276701	0.206308
hsa_circ_	0.183315	0.206318
hsa_circ_	0.48798	0.206337
hsa_circ_	-0.83676	0.206383
hsa_circ_	-0.20985	0.206388
hsa_circ_	-0.17786	0.2064
hsa_circ_	0.605811	0.206402
hsa_circ_	0.425392	0.206403
hsa_circ_	0.74569	0.206413
hsa_circ_	-0.19884	0.206421
hsa_circ_	-0.37481	0.206441
hsa_circ_	0.209577	0.206448
hsa_circ_	-0.19057	0.20646
hsa_circ_	-0.54051	0.206485
hsa_circ_	-0.28202	0.206492
hsa_circ_	0.324179	0.20653
hsa_circ_	-0.76757	0.206539
hsa_circ_	0.853624	0.206554
hsa_circ_	0.878452	0.206561
hsa_circ_	0.22076	0.206597
hsa_circ_	-0.19493	0.206608
hsa_circ_	-0.4425	0.206617
hsa_circ_	0.388983	0.206651
hsa_circ_	-0.33325	0.206653
hsa_circ_	-0.2312	0.206664
hsa_circ_	-0.21185	0.206665
hsa_circ_	0.530464	0.206669
hsa_circ_	0.217955	0.206677
hsa_circ_	0.892811	0.206682
hsa_circ_	-0.24226	0.206689
hsa_circ_	1.712146	0.206694
hsa_circ_	0.354017	0.206695
hsa_circ_	0.4067	0.206695
hsa_circ_	-0.19641	0.206707
hsa_circ_	0.228792	0.206717
hsa_circ_	-0.35788	0.206718
hsa_circ_	0.200547	0.206721
hsa_circ_	0.850753	0.206723
hsa_circ_	-0.25655	0.206735
hsa_circ_	0.221123	0.206744
hsa_circ_	0.263177	0.206767
hsa_circ_	-0.42352	0.206782
hsa_circ_	1.133757	0.206786
hsa_circ_	-0.21995	0.206792
hsa_circ_	-0.21719	0.206828
hsa_circ_	-0.22149	0.206838
hsa_circ_	-0.36863	0.206862
hsa_circ_	0.17323	0.206866
hsa_circ_	0.209627	0.206883

hsa_circ_	-0.23058	0.206889
hsa_circ_	0.203095	0.206921
hsa_circ_	0.271731	0.206921
hsa_circ_	-0.69104	0.206923
hsa_circ_	-0.28595	0.206926
hsa_circ_	0.554501	0.206926
hsa_circ_	-0.58886	0.206936
hsa_circ_	0.201005	0.206938
hsa_circ_	-0.39103	0.206947
hsa_circ_	-0.28699	0.206948
hsa_circ_	-0.19483	0.206961
hsa_circ_	-0.19081	0.206966
hsa_circ_	-0.27566	0.206968
hsa_circ_	-0.42045	0.20697
hsa_circ_	0.340127	0.206973
hsa_circ_	-0.27245	0.206984
hsa_circ_	0.23697	0.206991
hsa_circ_	-0.27328	0.207021
hsa_circ_	-0.24532	0.20703
hsa_circ_	0.37503	0.20704
hsa_circ_	0.185405	0.207055
hsa_circ_	-0.88289	0.207074
hsa_circ_	0.25304	0.207079
hsa_circ_	-0.2583	0.207106
hsa_circ_	-0.43363	0.207108
hsa_circ_	-0.43819	0.207124
hsa_circ_	0.227738	0.207125
hsa_circ_	-0.40381	0.207153
hsa_circ_	0.249115	0.207173
hsa_circ_	-0.26124	0.207174
hsa_circ_	0.342015	0.207209
hsa_circ_	-0.24075	0.207229
hsa_circ_	0.233394	0.207246
hsa_circ_	-0.18009	0.207249
hsa_circ_	0.235827	0.207265
hsa_circ_	0.26695	0.207266
hsa_circ_	-0.37545	0.207269
hsa_circ_	0.226315	0.207271
hsa_circ_	-0.18048	0.207278
hsa_circ_	-0.65102	0.207295
hsa_circ_	0.211671	0.207339
hsa_circ_	0.200255	0.207344
hsa_circ_	-0.18214	0.207344
hsa_circ_	-0.25854	0.207349
hsa_circ_	-0.53048	0.207365
hsa_circ_	-0.47068	0.207372
hsa_circ_	-0.1997	0.207379
hsa_circ_	0.405121	0.20739
hsa_circ_	0.207351	0.207413
hsa_circ_	0.204198	0.207441
hsa_circ_	-1.06789	0.207469
hsa_circ_	-0.30329	0.207479
hsa_circ_	0.226199	0.207482
hsa_circ_	-0.35543	0.207484

hsa_circ_	0.253905	0.207489
hsa_circ_	0.281585	0.207526
hsa_circ_	0.281328	0.207526
hsa_circ_	-1.24162	0.207541
hsa_circ_	0.205357	0.207545
hsa_circ_	0.256255	0.207562
hsa_circ_	0.394852	0.207568
hsa_circ_	0.225159	0.207577
hsa_circ_	0.396094	0.207585
hsa_circ_	0.177043	0.207587
hsa_circ_	-0.18768	0.207595
hsa_circ_	0.247567	0.2076
hsa_circ_	0.202085	0.207617
hsa_circ_	-0.18042	0.207634
hsa_circ_	-0.19213	0.207637
hsa_circ_	0.229128	0.207638
hsa_circ_	0.377152	0.207638
hsa_circ_	-0.22525	0.207646
hsa_circ_	0.461664	0.207654
hsa_circ_	0.611924	0.207656
hsa_circ_	-0.20147	0.207661
hsa_circ_	0.625502	0.207674
hsa_circ_	-0.17842	0.207676
hsa_circ_	-0.21049	0.207695
hsa_circ_	-0.22368	0.207704
hsa_circ_	0.593301	0.20771
hsa_circ_	0.419774	0.207734
hsa_circ_	-0.1857	0.207746
hsa_circ_	-0.36319	0.207767
hsa_circ_	0.25364	0.207774
hsa_circ_	-0.18542	0.207783
hsa_circ_	-0.20013	0.207801
hsa_circ_	0.477537	0.207807
hsa_circ_	0.298304	0.207811
hsa_circ_	0.254931	0.20782
hsa_circ_	-0.48772	0.207822
hsa_circ_	0.285729	0.207832
hsa_circ_	-0.35036	0.207836
hsa_circ_	-0.22078	0.207838
hsa_circ_	-0.72075	0.20785
hsa_circ_	-0.2414	0.207861
hsa_circ_	-0.28153	0.207874
hsa_circ_	0.537153	0.207881
hsa_circ_	-0.18701	0.207882
hsa_circ_	-0.24088	0.207884
hsa_circ_	-0.48572	0.207901
hsa_circ_	-0.64055	0.207902
hsa_circ_	0.321896	0.207904
hsa_circ_	-0.30975	0.207904
hsa_circ_	-0.32578	0.207909
hsa_circ_	0.280551	0.20791
hsa_circ_	-0.3173	0.207933
hsa_circ_	-0.44925	0.20795
hsa_circ_	-0.19084	0.207952

hsa_circ_	-0.19707	0.207962
hsa_circ_	-0.40816	0.208005
hsa_circ_	-0.2489	0.208012
hsa_circ_	-0.25403	0.208024
hsa_circ_	-0.22102	0.208024
hsa_circ_	-0.19529	0.208032
hsa_circ_	-0.61176	0.20804
hsa_circ_	0.274439	0.208057
hsa_circ_	-0.45216	0.208077
hsa_circ_	-0.21026	0.208077
hsa_circ_	0.320194	0.208087
hsa_circ_	-0.27227	0.208103
hsa_circ_	0.401202	0.20811
hsa_circ_	0.283545	0.208111
hsa_circ_	-0.20132	0.208133
hsa_circ_	0.226859	0.208134
hsa_circ_	0.485421	0.208138
hsa_circ_	0.567185	0.208142
hsa_circ_	0.426763	0.208143
hsa_circ_	-0.73131	0.208144
hsa_circ_	-0.22749	0.208155
hsa_circ_	-0.294	0.20816
hsa_circ_	0.315077	0.208161
hsa_circ_	0.356625	0.208183
hsa_circ_	-0.32253	0.208187
hsa_circ_	0.436939	0.208214
hsa_circ_	-0.73518	0.208217
hsa_circ_	0.248308	0.208228
hsa_circ_	-0.25114	0.20824
hsa_circ_	0.179713	0.208251
hsa_circ_	-0.21043	0.208252
hsa_circ_	-0.49235	0.208256
hsa_circ_	0.558961	0.208264
hsa_circ_	-0.17233	0.208268
hsa_circ_	0.306211	0.20827
hsa_circ_	0.202526	0.208273
hsa_circ_	0.424106	0.208274
hsa_circ_	0.270773	0.208289
hsa_circ_	-0.39226	0.208303
hsa_circ_	0.2058	0.208323
hsa_circ_	0.223876	0.208337
hsa_circ_	0.428013	0.208355
hsa_circ_	0.556548	0.208372
hsa_circ_	0.499482	0.208393
hsa_circ_	0.217615	0.208413
hsa_circ_	0.218062	0.208417
hsa_circ_	-0.24483	0.208423
hsa_circ_	-0.57128	0.208424
hsa_circ_	-0.20925	0.20843
hsa_circ_	0.381933	0.208473
hsa_circ_	-0.18955	0.208478
hsa_circ_	0.243643	0.20849
hsa_circ_	-0.37452	0.208495
hsa_circ_	-0.19305	0.208507

hsa_circ_	-0.43107	0.20852
hsa_circ_	0.31605	0.208528
hsa_circ_	-0.19164	0.208553
hsa_circ_	-0.41777	0.208553
hsa_circ_	0.537408	0.208556
hsa_circ_	0.447708	0.208561
hsa_circ_	-0.87731	0.208562
hsa_circ_	-0.26331	0.208566
hsa_circ_	0.607764	0.208572
hsa_circ_	-0.253	0.208578
hsa_circ_	0.365519	0.20859
hsa_circ_	0.180203	0.208591
hsa_circ_	0.197643	0.208594
hsa_circ_	-0.50723	0.208599
hsa_circ_	0.353866	0.208605
hsa_circ_	-0.26537	0.208612
hsa_circ_	0.298053	0.208612
hsa_circ_	-0.23602	0.208616
hsa_circ_	0.431075	0.20863
hsa_circ_	0.265692	0.20864
hsa_circ_	-0.1893	0.208641
hsa_circ_	-0.23189	0.208662
hsa_circ_	-0.24769	0.208669
hsa_circ_	0.241218	0.208686
hsa_circ_	-0.58183	0.208693
hsa_circ_	0.209188	0.208721
hsa_circ_	-0.19678	0.208735
hsa_circ_	0.365333	0.208746
hsa_circ_	-0.25674	0.208749
hsa_circ_	-0.25581	0.208753
hsa_circ_	-0.33341	0.208763
hsa_circ_	-0.21552	0.208764
hsa_circ_	0.717325	0.208791
hsa_circ_	0.20747	0.208795
hsa_circ_	0.244162	0.208816
hsa_circ_	-0.77533	0.208818
hsa_circ_	0.314578	0.208819
hsa_circ_	0.300615	0.208824
hsa_circ_	-0.20055	0.208828
hsa_circ_	-0.2109	0.208832
hsa_circ_	0.316995	0.20887
hsa_circ_	0.260589	0.20887
hsa_circ_	0.442591	0.208872
hsa_circ_	-0.21782	0.20888
hsa_circ_	1.278184	0.208884
hsa_circ_	-0.17599	0.208888
hsa_circ_	-0.45309	0.208897
hsa_circ_	-0.71981	0.208897
hsa_circ_	0.379478	0.208907
hsa_circ_	-0.21195	0.208907
hsa_circ_	0.199942	0.208912
hsa_circ_	0.226481	0.208921
hsa_circ_	0.223714	0.208933
hsa_circ_	-0.25039	0.208936

hsa_circ_	0.798562	0.208953
hsa_circ_	-0.22606	0.208961
hsa_circ_	0.73227	0.208963
hsa_circ_	-0.74574	0.20897
hsa_circ_	0.310045	0.208972
hsa_circ_	-0.30406	0.208995
hsa_circ_	0.485866	0.208999
hsa_circ_	-0.2702	0.209001
hsa_circ_	0.387308	0.209011
hsa_circ_	-0.24198	0.209043
hsa_circ_	0.404558	0.209047
hsa_circ_	-0.29348	0.209052
hsa_circ_	-0.44575	0.209072
hsa_circ_	0.326847	0.209084
hsa_circ_	-0.71611	0.209089
hsa_circ_	0.341473	0.20909
hsa_circ_	0.683839	0.209096
hsa_circ_	-0.20761	0.209105
hsa_circ_	0.318467	0.209127
hsa_circ_	0.276395	0.209139
hsa_circ_	0.444404	0.209148
hsa_circ_	0.357299	0.209148
hsa_circ_	-0.24876	0.209149
hsa_circ_	-0.22076	0.209151
hsa_circ_	-0.31452	0.209152
hsa_circ_	1.123152	0.209157
hsa_circ_	-0.79837	0.209175
hsa_circ_	0.536783	0.209193
hsa_circ_	-0.26566	0.20922
hsa_circ_	-0.3045	0.209221
hsa_circ_	0.251532	0.209237
hsa_circ_	-0.17668	0.209249
hsa_circ_	0.204644	0.20925
hsa_circ_	0.54587	0.209258
hsa_circ_	-0.43871	0.209261
hsa_circ_	-0.19557	0.209304
hsa_circ_	-0.2168	0.20931
hsa_circ_	-0.18571	0.209314
hsa_circ_	-0.68171	0.209326
hsa_circ_	0.228563	0.209351
hsa_circ_	0.475045	0.209352
hsa_circ_	0.320793	0.209353
hsa_circ_	0.232428	0.209356
hsa_circ_	-0.46205	0.209374
hsa_circ_	-0.30074	0.209374
hsa_circ_	0.184674	0.209395
hsa_circ_	-0.23676	0.209397
hsa_circ_	0.790181	0.209398
hsa_circ_	-0.35606	0.209403
hsa_circ_	0.293055	0.209422
hsa_circ_	-0.20282	0.209423
hsa_circ_	0.237974	0.209429
hsa_circ_	-0.55223	0.209442
hsa_circ_	0.501766	0.209443

hsa_circ_	0.187026	0.209449
hsa_circ_	0.449055	0.20946
hsa_circ_	0.597063	0.209461
hsa_circ_	-0.46439	0.209471
hsa_circ_	0.430713	0.209482
hsa_circ_	-0.20914	0.209487
hsa_circ_	-0.35078	0.209491
hsa_circ_	0.376917	0.209503
hsa_circ_	0.218223	0.209508
hsa_circ_	0.267982	0.209543
hsa_circ_	-0.18332	0.209549
hsa_circ_	1.308915	0.209551
hsa_circ_	-0.1876	0.20957
hsa_circ_	1.066666	0.20957
hsa_circ_	0.237754	0.209585
hsa_circ_	-0.38008	0.209586
hsa_circ_	-0.19384	0.209601
hsa_circ_	0.314384	0.209602
hsa_circ_	0.333105	0.209612
hsa_circ_	0.645557	0.209612
hsa_circ_	-0.50456	0.209624
hsa_circ_	0.204859	0.209652
hsa_circ_	-0.22896	0.209653
hsa_circ_	0.26633	0.209654
hsa_circ_	0.234917	0.209654
hsa_circ_	-0.23225	0.209666
hsa_circ_	-0.4214	0.209687
hsa_circ_	0.977809	0.20969
hsa_circ_	0.204602	0.209702
hsa_circ_	0.31376	0.209735
hsa_circ_	-0.24028	0.209744
hsa_circ_	0.882461	0.209749
hsa_circ_	-0.18765	0.209751
hsa_circ_	0.172355	0.209766
hsa_circ_	0.173956	0.209782
hsa_circ_	0.282233	0.209795
hsa_circ_	-0.23378	0.209804
hsa_circ_	-0.2616	0.209819
hsa_circ_	-0.19605	0.209828
hsa_circ_	-1.32998	0.209831
hsa_circ_	0.295303	0.209836
hsa_circ_	0.510908	0.209839
hsa_circ_	0.209119	0.209842
hsa_circ_	0.491069	0.209855
hsa_circ_	0.268322	0.209884
hsa_circ_	-0.25438	0.209886
hsa_circ_	0.349251	0.209886
hsa_circ_	0.251067	0.209899
hsa_circ_	0.349635	0.209899
hsa_circ_	-0.35	0.209959
hsa_circ_	0.183514	0.209962
hsa_circ_	-0.28889	0.209971
hsa_circ_	-0.28642	0.209978
hsa_circ_	-0.33429	0.209978

hsa_circ_	0.269109	0.210015
hsa_circ_	-0.25223	0.210017
hsa_circ_	-0.6162	0.210021
hsa_circ_	0.182934	0.210031
hsa_circ_	-0.54616	0.210061
hsa_circ_	-0.1951	0.210083
hsa_circ_	0.181083	0.210093
hsa_circ_	0.16892	0.210094
hsa_circ_	-0.25516	0.210102
hsa_circ_	-0.33149	0.210123
hsa_circ_	-0.26484	0.21013
hsa_circ_	-0.27196	0.210143
hsa_circ_	0.230042	0.210145
hsa_circ_	0.298091	0.210165
hsa_circ_	0.516869	0.210168
hsa_circ_	-0.65099	0.210198
hsa_circ_	0.236898	0.2102
hsa_circ_	-0.16871	0.210213
hsa_circ_	0.296386	0.210213
hsa_circ_	0.258283	0.210218
hsa_circ_	0.258511	0.210223
hsa_circ_	0.193126	0.210225
hsa_circ_	-0.23875	0.210237
hsa_circ_	-0.34968	0.210244
hsa_circ_	-0.54394	0.210251
hsa_circ_	0.241242	0.210254
hsa_circ_	0.411264	0.210276
hsa_circ_	-0.53043	0.210286
hsa_circ_	-0.50378	0.210286
hsa_circ_	-0.19685	0.210296
hsa_circ_	-0.49558	0.210299
hsa_circ_	-0.20694	0.210304
hsa_circ_	-0.20504	0.210319
hsa_circ_	0.189601	0.210319
hsa_circ_	0.216808	0.210329
hsa_circ_	-0.22759	0.210329
hsa_circ_	-0.22743	0.210333
hsa_circ_	0.200588	0.210354
hsa_circ_	-0.38171	0.210385
hsa_circ_	0.294304	0.210402
hsa_circ_	-0.445	0.210403
hsa_circ_	0.225364	0.210413
hsa_circ_	0.464113	0.210419
hsa_circ_	0.185512	0.210432
hsa_circ_	0.244246	0.210435
hsa_circ_	-0.27569	0.210457
hsa_circ_	0.327762	0.210463
hsa_circ_	-0.20143	0.210484
hsa_circ_	0.249107	0.210493
hsa_circ_	0.288212	0.210502
hsa_circ_	0.48098	0.210505
hsa_circ_	0.206924	0.210507
hsa_circ_	0.378297	0.21051
hsa_circ_	-0.17963	0.210517

hsa_circ_	0.264574	0.210523
hsa_circ_	-0.33935	0.210523
hsa_circ_	-0.63321	0.210532
hsa_circ_	-0.54675	0.210536
hsa_circ_	-0.35919	0.210537
hsa_circ_	-0.17256	0.210547
hsa_circ_	-0.54875	0.210547
hsa_circ_	-0.24563	0.210566
hsa_circ_	0.323378	0.210569
hsa_circ_	0.395777	0.210575
hsa_circ_	-0.27234	0.210588
hsa_circ_	-0.7837	0.210591
hsa_circ_	-0.22589	0.210628
hsa_circ_	-0.32313	0.210634
hsa_circ_	0.171488	0.210647
hsa_circ_	-0.20239	0.210662
hsa_circ_	0.210716	0.210675
hsa_circ_	-0.29596	0.21068
hsa_circ_	0.504807	0.210687
hsa_circ_	0.663351	0.210705
hsa_circ_	0.467795	0.210712
hsa_circ_	0.24673	0.210713
hsa_circ_	0.236137	0.210718
hsa_circ_	-0.22175	0.210736
hsa_circ_	0.646745	0.210755
hsa_circ_	-0.29109	0.210757
hsa_circ_	-0.38828	0.210763
hsa_circ_	0.293705	0.210764
hsa_circ_	0.223132	0.210772
hsa_circ_	-0.22205	0.210793
hsa_circ_	0.721773	0.210797
hsa_circ_	0.606569	0.210802
hsa_circ_	-0.36607	0.210814
hsa_circ_	0.183111	0.210818
hsa_circ_	-0.50066	0.21083
hsa_circ_	-0.21366	0.210841
hsa_circ_	0.739669	0.210843
hsa_circ_	-0.19377	0.210847
hsa_circ_	0.193888	0.210858
hsa_circ_	0.563991	0.210871
hsa_circ_	0.271347	0.210892
hsa_circ_	-0.42807	0.210895
hsa_circ_	-0.16963	0.210905
hsa_circ_	0.262171	0.21091
hsa_circ_	-0.43621	0.210914
hsa_circ_	0.328801	0.210914
hsa_circ_	-0.17373	0.210918
hsa_circ_	0.713522	0.210931
hsa_circ_	-0.36322	0.210934
hsa_circ_	0.234953	0.210935
hsa_circ_	-0.35721	0.210941
hsa_circ_	-0.41193	0.21095
hsa_circ_	0.670011	0.210958
hsa_circ_	0.220109	0.210963

hsa_circ_	0.429222	0.210964
hsa_circ_	-0.19774	0.210964
hsa_circ_	-0.20149	0.210968
hsa_circ_	-0.25562	0.210975
hsa_circ_	0.245024	0.211006
hsa_circ_	-0.25678	0.211014
hsa_circ_	0.21563	0.211017
hsa_circ_	0.236698	0.211031
hsa_circ_	-0.24166	0.211032
hsa_circ_	0.295961	0.211036
hsa_circ_	-0.2998	0.21104
hsa_circ_	-0.21538	0.211048
hsa_circ_	0.311137	0.211056
hsa_circ_	-0.26517	0.211057
hsa_circ_	0.328477	0.211058
hsa_circ_	0.190693	0.21106
hsa_circ_	0.499502	0.211084
hsa_circ_	0.21873	0.211087
hsa_circ_	0.533662	0.211112
hsa_circ_	-0.39957	0.211138
hsa_circ_	-0.18353	0.211142
hsa_circ_	-0.35609	0.211156
hsa_circ_	0.187125	0.211161
hsa_circ_	-0.22371	0.211163
hsa_circ_	-0.19385	0.211163
hsa_circ_	0.27596	0.211165
hsa_circ_	0.212945	0.211168
hsa_circ_	0.943808	0.211173
hsa_circ_	0.620685	0.211176
hsa_circ_	0.194315	0.21118
hsa_circ_	-0.24314	0.211187
hsa_circ_	0.3989	0.21121
hsa_circ_	-0.2629	0.21122
hsa_circ_	0.369721	0.211227
hsa_circ_	0.22405	0.211233
hsa_circ_	1.543365	0.211243
hsa_circ_	0.476446	0.211246
hsa_circ_	-0.19365	0.211249
hsa_circ_	-0.31476	0.21127
hsa_circ_	0.339028	0.21128
hsa_circ_	-0.26483	0.211281
hsa_circ_	-0.43356	0.211283
hsa_circ_	-0.35377	0.211298
hsa_circ_	-0.26398	0.211304
hsa_circ_	0.214532	0.211307
hsa_circ_	-0.2355	0.211328
hsa_circ_	0.253929	0.211331
hsa_circ_	-0.54729	0.211336
hsa_circ_	0.848147	0.211345
hsa_circ_	0.248328	0.211347
hsa_circ_	0.744996	0.211366
hsa_circ_	0.25905	0.211375
hsa_circ_	-0.26982	0.211376
hsa_circ_	-0.53181	0.211389

hsa_circ_	0.571818	0.211404
hsa_circ_	-0.64817	0.211415
hsa_circ_	0.368027	0.211417
hsa_circ_	0.186254	0.211424
hsa_circ_	0.199041	0.211431
hsa_circ_	-0.21601	0.211431
hsa_circ_	0.515092	0.211447
hsa_circ_	-0.19145	0.211454
hsa_circ_	0.238514	0.211463
hsa_circ_	-0.28299	0.211464
hsa_circ_	0.49626	0.211468
hsa_circ_	0.183456	0.211486
hsa_circ_	0.166843	0.211499
hsa_circ_	-0.2595	0.211518
hsa_circ_	0.224668	0.211525
hsa_circ_	0.277632	0.211544
hsa_circ_	0.205926	0.211552
hsa_circ_	-0.46791	0.211556
hsa_circ_	-0.17713	0.211564
hsa_circ_	0.268432	0.211591
hsa_circ_	0.28003	0.211599
hsa_circ_	0.439816	0.211618
hsa_circ_	0.278449	0.211633
hsa_circ_	-0.33611	0.211636
hsa_circ_	0.307867	0.211636
hsa_circ_	-0.25934	0.211643
hsa_circ_	0.431815	0.211644
hsa_circ_	-0.18458	0.211648
hsa_circ_	-0.21477	0.211649
hsa_circ_	0.247211	0.21166
hsa_circ_	0.185049	0.21166
hsa_circ_	0.186447	0.211689
hsa_circ_	0.253302	0.211694
hsa_circ_	-0.43379	0.211712
hsa_circ_	0.234945	0.211719
hsa_circ_	-0.60986	0.211728
hsa_circ_	-0.22736	0.211745
hsa_circ_	0.336193	0.211752
hsa_circ_	-0.24842	0.211755
hsa_circ_	-0.1935	0.211761
hsa_circ_	-0.17834	0.211769
hsa_circ_	0.784891	0.211776
hsa_circ_	0.729868	0.211779
hsa_circ_	1.067581	0.21179
hsa_circ_	-0.48306	0.211795
hsa_circ_	0.240325	0.211797
hsa_circ_	-0.31202	0.211799
hsa_circ_	-1.43338	0.211803
hsa_circ_	0.322299	0.211823
hsa_circ_	-0.19103	0.211833
hsa_circ_	0.325065	0.211848
hsa_circ_	0.222049	0.211848
hsa_circ_	-0.26149	0.211856
hsa_circ_	0.860268	0.211872

hsa_circ_	0.260684	0.211873
hsa_circ_	0.352706	0.211879
hsa_circ_	0.190398	0.21188
hsa_circ_	-0.94871	0.211883
hsa_circ_	0.203894	0.211891
hsa_circ_	-0.61669	0.2119
hsa_circ_	0.294854	0.211904
hsa_circ_	0.733474	0.211906
hsa_circ_	0.296635	0.211927
hsa_circ_	0.345041	0.211929
hsa_circ_	0.338308	0.211937
hsa_circ_	0.514375	0.211952
hsa_circ_	-0.18334	0.211968
hsa_circ_	0.328706	0.211982
hsa_circ_	-0.21737	0.212
hsa_circ_	-0.35397	0.212012
hsa_circ_	0.220891	0.212015
hsa_circ_	0.171259	0.212024
hsa_circ_	0.271069	0.212028
hsa_circ_	0.187525	0.212044
hsa_circ_	0.190593	0.212052
hsa_circ_	0.387765	0.212053
hsa_circ_	0.871534	0.212054
hsa_circ_	0.220358	0.212055
hsa_circ_	0.315516	0.212057
hsa_circ_	-0.17225	0.212058
hsa_circ_	0.366171	0.212062
hsa_circ_	0.832308	0.212072
hsa_circ_	-0.3892	0.212099
hsa_circ_	0.599634	0.212108
hsa_circ_	-0.18355	0.212122
hsa_circ_	0.337457	0.212138
hsa_circ_	-0.30306	0.212163
hsa_circ_	0.391324	0.212177
hsa_circ_	-0.26064	0.212185
hsa_circ_	0.286849	0.212188
hsa_circ_	0.223315	0.212196
hsa_circ_	0.380525	0.212217
hsa_circ_	-0.52436	0.212222
hsa_circ_	-0.19117	0.212226
hsa_circ_	-0.24715	0.212245
hsa_circ_	-0.18954	0.212245
hsa_circ_	-0.91471	0.212248
hsa_circ_	0.230506	0.212261
hsa_circ_	-0.27794	0.212262
hsa_circ_	0.20807	0.21227
hsa_circ_	-0.69479	0.212276
hsa_circ_	-0.19594	0.212284
hsa_circ_	0.231418	0.212299
hsa_circ_	0.255949	0.212304
hsa_circ_	0.235926	0.212326
hsa_circ_	-0.28704	0.212358
hsa_circ_	0.265881	0.212359
hsa_circ_	0.431357	0.212361

hsa_circ_	-0.23542	0.212378
hsa_circ_	-0.39988	0.212396
hsa_circ_	0.241309	0.212417
hsa_circ_	0.304248	0.212435
hsa_circ_	-0.42116	0.212453
hsa_circ_	0.7514	0.212462
hsa_circ_	-0.19711	0.212478
hsa_circ_	-0.20184	0.212485
hsa_circ_	-0.21458	0.212485
hsa_circ_	-0.40499	0.212494
hsa_circ_	-0.21952	0.212509
hsa_circ_	-1.12602	0.212521
hsa_circ_	-0.58302	0.212522
hsa_circ_	-0.21414	0.212541
hsa_circ_	-0.21616	0.212552
hsa_circ_	-1.07879	0.212577
hsa_circ_	-0.31653	0.212592
hsa_circ_	-0.1875	0.212592
hsa_circ_	-0.21762	0.212592
hsa_circ_	-0.18459	0.21263
hsa_circ_	0.181408	0.212633
hsa_circ_	0.314971	0.212646
hsa_circ_	-0.18087	0.212647
hsa_circ_	-0.85154	0.212683
hsa_circ_	-0.24953	0.212694
hsa_circ_	0.422485	0.212707
hsa_circ_	-0.5496	0.212719
hsa_circ_	0.802796	0.21272
hsa_circ_	0.187442	0.212721
hsa_circ_	0.432441	0.212738
hsa_circ_	-0.71068	0.212764
hsa_circ_	-0.17773	0.212766
hsa_circ_	0.179094	0.212772
hsa_circ_	-0.23854	0.212776
hsa_circ_	0.199082	0.21278
hsa_circ_	-0.54408	0.212787
hsa_circ_	-0.35677	0.212789
hsa_circ_	0.258356	0.212789
hsa_circ_	0.181666	0.212806
hsa_circ_	-0.29394	0.212811
hsa_circ_	0.649109	0.212832
hsa_circ_	-0.72195	0.212844
hsa_circ_	-0.87196	0.212867
hsa_circ_	-0.33329	0.212871
hsa_circ_	-0.18683	0.212879
hsa_circ_	0.66492	0.21288
hsa_circ_	0.558326	0.212899
hsa_circ_	-0.36962	0.2129
hsa_circ_	-0.32508	0.212932
hsa_circ_	0.344685	0.212934
hsa_circ_	-0.24439	0.212954
hsa_circ_	-0.29327	0.212963
hsa_circ_	-0.21301	0.212978
hsa_circ_	0.257218	0.212984

hsa_circ_	-0.35618	0.212985
hsa_circ_	0.448685	0.212988
hsa_circ_	-0.24934	0.212992
hsa_circ_	-0.23316	0.213001
hsa_circ_	0.185145	0.213015
hsa_circ_	-0.39586	0.213027
hsa_circ_	0.214808	0.213044
hsa_circ_	-0.29807	0.213074
hsa_circ_	-0.31707	0.213078
hsa_circ_	-2.02379	0.213094
hsa_circ_	0.869097	0.213097
hsa_circ_	0.295967	0.213139
hsa_circ_	0.479483	0.213142
hsa_circ_	0.205075	0.213145
hsa_circ_	0.308371	0.21315
hsa_circ_	-0.29933	0.213169
hsa_circ_	0.2435	0.213184
hsa_circ_	-0.38197	0.213189
hsa_circ_	-0.22769	0.213211
hsa_circ_	0.165125	0.213212
hsa_circ_	-0.83222	0.213229
hsa_circ_	0.283094	0.213231
hsa_circ_	0.226681	0.21324
hsa_circ_	-0.37361	0.213242
hsa_circ_	0.291223	0.213256
hsa_circ_	0.259581	0.213259
hsa_circ_	-0.25483	0.213276
hsa_circ_	-0.44537	0.213278
hsa_circ_	0.238621	0.213283
hsa_circ_	0.29279	0.213288
hsa_circ_	0.227052	0.213298
hsa_circ_	-0.2517	0.213299
hsa_circ_	0.30191	0.213302
hsa_circ_	0.247597	0.213307
hsa_circ_	-0.18753	0.213315
hsa_circ_	0.780987	0.213331
hsa_circ_	0.188735	0.213332
hsa_circ_	-0.31695	0.213333
hsa_circ_	-0.54015	0.213359
hsa_circ_	0.527973	0.213362
hsa_circ_	0.27764	0.213372
hsa_circ_	0.204433	0.213375
hsa_circ_	-0.23978	0.213384
hsa_circ_	-0.30893	0.21339
hsa_circ_	-0.23093	0.213394
hsa_circ_	-0.21462	0.21341
hsa_circ_	-0.21705	0.213439
hsa_circ_	0.34793	0.213444
hsa_circ_	-1.04714	0.213447
hsa_circ_	-0.5824	0.213459
hsa_circ_	0.459468	0.213461
hsa_circ_	0.3433	0.213468
hsa_circ_	-0.24888	0.21347
hsa_circ_	-0.22943	0.213475

hsa_circ_	0.196414	0.213497
hsa_circ_	-0.39909	0.213498
hsa_circ_	0.279412	0.213505
hsa_circ_	0.741425	0.213515
hsa_circ_	0.416637	0.213521
hsa_circ_	-0.34656	0.213538
hsa_circ_	-0.30773	0.21354
hsa_circ_	-0.36028	0.213541
hsa_circ_	-0.37667	0.213543
hsa_circ_	0.406787	0.213555
hsa_circ_	0.279458	0.213563
hsa_circ_	-0.19525	0.213564
hsa_circ_	0.233701	0.213581
hsa_circ_	-0.2602	0.213597
hsa_circ_	0.314424	0.213601
hsa_circ_	0.168159	0.213603
hsa_circ_	0.440584	0.21361
hsa_circ_	0.251104	0.213637
hsa_circ_	-0.26261	0.21364
hsa_circ_	-0.34975	0.213641
hsa_circ_	0.360137	0.213662
hsa_circ_	-0.20015	0.213681
hsa_circ_	0.286172	0.213693
hsa_circ_	0.391635	0.213701
hsa_circ_	-0.18591	0.213727
hsa_circ_	0.251358	0.213739
hsa_circ_	-0.19358	0.213745
hsa_circ_	0.554111	0.213749
hsa_circ_	-0.19817	0.213749
hsa_circ_	0.277796	0.213767
hsa_circ_	0.197303	0.213777
hsa_circ_	0.240267	0.213778
hsa_circ_	-0.53605	0.21381
hsa_circ_	0.297271	0.213811
hsa_circ_	0.411606	0.213812
hsa_circ_	0.382125	0.213819
hsa_circ_	0.182264	0.213822
hsa_circ_	-0.25421	0.213834
hsa_circ_	0.325067	0.213834
hsa_circ_	0.218767	0.213843
hsa_circ_	0.288405	0.213864
hsa_circ_	0.275979	0.213894
hsa_circ_	-0.29187	0.213895
hsa_circ_	0.229975	0.213897
hsa_circ_	0.3411	0.213911
hsa_circ_	0.268186	0.213917
hsa_circ_	-0.25242	0.213918
hsa_circ_	0.298048	0.213919
hsa_circ_	-0.22372	0.213928
hsa_circ_	-0.35052	0.213954
hsa_circ_	-0.44708	0.21396
hsa_circ_	-0.54413	0.213965
hsa_circ_	0.661576	0.21397
hsa_circ_	-0.22414	0.213973

hsa_circ_	0.236484	0.213978
hsa_circ_	0.288986	0.214032
hsa_circ_	-0.25563	0.214037
hsa_circ_	-0.58438	0.214045
hsa_circ_	0.285922	0.214049
hsa_circ_	-0.34568	0.21405
hsa_circ_	-0.2219	0.214052
hsa_circ_	0.253161	0.214062
hsa_circ_	0.247967	0.214064
hsa_circ_	-0.20298	0.214077
hsa_circ_	-0.21351	0.214095
hsa_circ_	0.278132	0.214113
hsa_circ_	-0.57971	0.214114
hsa_circ_	0.307567	0.214116
hsa_circ_	-0.35173	0.214118
hsa_circ_	-0.72763	0.214125
hsa_circ_	0.276492	0.214135
hsa_circ_	-0.19289	0.214145
hsa_circ_	0.190272	0.214148
hsa_circ_	-0.19657	0.214164
hsa_circ_	0.271387	0.214181
hsa_circ_	-1.04334	0.214188
hsa_circ_	0.308733	0.214196
hsa_circ_	0.236968	0.214208
hsa_circ_	-0.24639	0.214211
hsa_circ_	0.285787	0.21422
hsa_circ_	0.492492	0.214222
hsa_circ_	-0.24623	0.214226
hsa_circ_	-0.23565	0.214231
hsa_circ_	-0.19062	0.214251
hsa_circ_	0.329679	0.214255
hsa_circ_	0.217623	0.214297
hsa_circ_	-0.45287	0.214304
hsa_circ_	-0.17863	0.214324
hsa_circ_	0.260461	0.214332
hsa_circ_	-0.22328	0.214335
hsa_circ_	-0.54574	0.214338
hsa_circ_	-0.26051	0.214363
hsa_circ_	-0.20923	0.214364
hsa_circ_	0.964731	0.214372
hsa_circ_	-0.27182	0.214389
hsa_circ_	0.223679	0.214403
hsa_circ_	0.848111	0.214422
hsa_circ_	0.222849	0.214428
hsa_circ_	-0.52803	0.214432
hsa_circ_	0.19674	0.214445
hsa_circ_	-0.68878	0.214474
hsa_circ_	-0.54782	0.214497
hsa_circ_	0.323287	0.214511
hsa_circ_	-0.28311	0.21452
hsa_circ_	-1.01277	0.214522
hsa_circ_	-0.39325	0.21454
hsa_circ_	0.18019	0.214552
hsa_circ_	0.279165	0.214565

hsa_circ_	0.365112	0.214579
hsa_circ_	0.264919	0.214588
hsa_circ_	-0.19927	0.214592
hsa_circ_	0.387026	0.214592
hsa_circ_	0.489828	0.214595
hsa_circ_	-0.36773	0.214598
hsa_circ_	-0.34182	0.214613
hsa_circ_	0.587883	0.214615
hsa_circ_	-0.222	0.214627
hsa_circ_	-0.22526	0.214628
hsa_circ_	-1.30669	0.214648
hsa_circ_	-0.25403	0.214648
hsa_circ_	-0.2743	0.214663
hsa_circ_	0.355058	0.214663
hsa_circ_	0.319893	0.214681
hsa_circ_	0.176478	0.214682
hsa_circ_	0.188829	0.214703
hsa_circ_	-0.41816	0.214707
hsa_circ_	0.185366	0.214708
hsa_circ_	0.673464	0.214708
hsa_circ_	0.234548	0.214722
hsa_circ_	0.348187	0.214725
hsa_circ_	0.338496	0.214732
hsa_circ_	-0.24002	0.214743
hsa_circ_	0.345057	0.214747
hsa_circ_	0.258634	0.214761
hsa_circ_	0.459366	0.214766
hsa_circ_	-0.45964	0.214773
hsa_circ_	-0.46224	0.214788
hsa_circ_	-0.19154	0.214791
hsa_circ_	-0.65183	0.214801
hsa_circ_	0.279415	0.214802
hsa_circ_	-0.21489	0.214823
hsa_circ_	0.314742	0.21484
hsa_circ_	-0.34471	0.214845
hsa_circ_	-0.19289	0.214847
hsa_circ_	-0.19413	0.214857
hsa_circ_	-0.21474	0.214858
hsa_circ_	0.290466	0.214861
hsa_circ_	-0.20524	0.214881
hsa_circ_	-0.9855	0.214888
hsa_circ_	0.486809	0.2149
hsa_circ_	-0.27681	0.214912
hsa_circ_	0.301549	0.214925
hsa_circ_	-0.3603	0.214927
hsa_circ_	-0.21701	0.214934
hsa_circ_	0.396504	0.214941
hsa_circ_	-0.23467	0.214968
hsa_circ_	-0.57585	0.214972
hsa_circ_	0.227757	0.214975
hsa_circ_	-0.20064	0.214988
hsa_circ_	-0.38773	0.215001
hsa_circ_	-0.34767	0.215009
hsa_circ_	0.245135	0.215011

hsa_circ_	0.362325	0.215054
hsa_circ_	0.242706	0.215057
hsa_circ_	0.240979	0.215084
hsa_circ_	0.241185	0.215092
hsa_circ_	-0.29065	0.215103
hsa_circ_	0.85338	0.215132
hsa_circ_	-1.604	0.215135
hsa_circ_	-0.50942	0.215136
hsa_circ_	-0.17458	0.21514
hsa_circ_	-0.18182	0.215143
hsa_circ_	0.406331	0.215143
hsa_circ_	0.21484	0.21515
hsa_circ_	-0.43022	0.215156
hsa_circ_	-0.18124	0.215169
hsa_circ_	0.248679	0.21517
hsa_circ_	0.329221	0.215172
hsa_circ_	0.32271	0.215178
hsa_circ_	0.176526	0.215181
hsa_circ_	0.630112	0.215195
hsa_circ_	-0.39064	0.21521
hsa_circ_	-0.42435	0.215211
hsa_circ_	-0.24602	0.215216
hsa_circ_	0.278327	0.215219
hsa_circ_	0.226119	0.21522
hsa_circ_	0.989519	0.215246
hsa_circ_	-0.33187	0.215263
hsa_circ_	-0.3545	0.215263
hsa_circ_	-0.18753	0.215279
hsa_circ_	0.371883	0.215299
hsa_circ_	0.239524	0.215299
hsa_circ_	0.438786	0.215312
hsa_circ_	0.19708	0.215315
hsa_circ_	0.2826	0.21532
hsa_circ_	-0.22433	0.215331
hsa_circ_	-0.17862	0.215334
hsa_circ_	-0.18038	0.21534
hsa_circ_	-0.18864	0.215348
hsa_circ_	0.324027	0.215349
hsa_circ_	-0.45202	0.215357
hsa_circ_	-0.46621	0.215369
hsa_circ_	-0.61498	0.215379
hsa_circ_	-0.2558	0.215382
hsa_circ_	-0.55234	0.215388
hsa_circ_	-0.2883	0.215416
hsa_circ_	0.607941	0.215452
hsa_circ_	-0.17792	0.215453
hsa_circ_	-0.36	0.215459
hsa_circ_	-0.35896	0.215464
hsa_circ_	0.487531	0.21548
hsa_circ_	-0.18304	0.215505
hsa_circ_	-0.23055	0.215508
hsa_circ_	-0.35824	0.215511
hsa_circ_	0.303158	0.215512
hsa_circ_	-0.18669	0.215515

hsa_circ_	-0.2993	0.215516
hsa_circ_	-0.34401	0.21552
hsa_circ_	-0.52785	0.215556
hsa_circ_	-0.47356	0.215558
hsa_circ_	-0.24697	0.215567
hsa_circ_	-0.69799	0.215578
hsa_circ_	0.694349	0.215588
hsa_circ_	-0.18907	0.215589
hsa_circ_	0.657386	0.215599
hsa_circ_	-0.61641	0.215599
hsa_circ_	-0.26597	0.215606
hsa_circ_	-0.20002	0.215606
hsa_circ_	0.200373	0.215643
hsa_circ_	-0.22344	0.215643
hsa_circ_	-0.20681	0.215646
hsa_circ_	0.368255	0.215652
hsa_circ_	0.367386	0.215659
hsa_circ_	-0.54531	0.215661
hsa_circ_	0.210224	0.215662
hsa_circ_	-0.35979	0.215664
hsa_circ_	-0.18541	0.215697
hsa_circ_	-0.17887	0.215713
hsa_circ_	-0.68714	0.215729
hsa_circ_	0.25648	0.215731
hsa_circ_	-0.20105	0.215743
hsa_circ_	-0.43489	0.215754
hsa_circ_	0.216751	0.215773
hsa_circ_	0.566995	0.215778
hsa_circ_	-0.24072	0.215779
hsa_circ_	0.473845	0.215803
hsa_circ_	-0.32745	0.215806
hsa_circ_	0.235272	0.21582
hsa_circ_	0.457903	0.215826
hsa_circ_	-0.31842	0.215831
hsa_circ_	-0.4142	0.215835
hsa_circ_	0.399853	0.215837
hsa_circ_	0.24112	0.215844
hsa_circ_	-0.32734	0.215852
hsa_circ_	-1.07887	0.215862
hsa_circ_	-0.26911	0.215875
hsa_circ_	0.57133	0.215875
hsa_circ_	-0.31092	0.215876
hsa_circ_	-0.2559	0.215892
hsa_circ_	-0.19912	0.215892
hsa_circ_	0.220188	0.215904
hsa_circ_	0.241869	0.215908
hsa_circ_	0.32473	0.215916
hsa_circ_	-1.20238	0.215927
hsa_circ_	-0.22099	0.215941
hsa_circ_	0.342359	0.215943
hsa_circ_	-0.4188	0.215957
hsa_circ_	0.248139	0.215981
hsa_circ_	-0.27782	0.215981
hsa_circ_	-0.37937	0.215981

hsa_circ_	0.265202	0.215992
hsa_circ_	-0.56814	0.216001
hsa_circ_	0.293507	0.216011
hsa_circ_	0.387013	0.216014
hsa_circ_	0.296136	0.216017
hsa_circ_	0.234326	0.216019
hsa_circ_	0.227858	0.216021
hsa_circ_	0.2098	0.216024
hsa_circ_	-0.22147	0.216037
hsa_circ_	0.354971	0.216048
hsa_circ_	0.222427	0.21605
hsa_circ_	0.330668	0.216068
hsa_circ_	0.473145	0.216078
hsa_circ_	0.278184	0.216082
hsa_circ_	0.496127	0.216087
hsa_circ_	-0.17289	0.216087
hsa_circ_	0.678935	0.21609
hsa_circ_	-0.60333	0.216092
hsa_circ_	-0.26408	0.216103
hsa_circ_	0.87287	0.216118
hsa_circ_	-1.13146	0.216121
hsa_circ_	-0.63284	0.216134
hsa_circ_	-0.22578	0.21614
hsa_circ_	-0.35274	0.216146
hsa_circ_	-0.51163	0.216152
hsa_circ_	-0.22564	0.216173
hsa_circ_	-0.26829	0.216175
hsa_circ_	-0.54464	0.216178
hsa_circ_	0.19998	0.216211
hsa_circ_	-0.44422	0.216227
hsa_circ_	-0.23554	0.216242
hsa_circ_	-0.27775	0.216258
hsa_circ_	0.343231	0.216265
hsa_circ_	0.966056	0.216291
hsa_circ_	-0.18051	0.216292
hsa_circ_	0.622561	0.216295
hsa_circ_	0.183911	0.216323
hsa_circ_	-0.31554	0.216344
hsa_circ_	-0.5735	0.216347
hsa_circ_	-0.51746	0.216358
hsa_circ_	0.279543	0.216382
hsa_circ_	0.324026	0.216388
hsa_circ_	-0.18031	0.21639
hsa_circ_	-0.18466	0.216404
hsa_circ_	-0.60105	0.216431
hsa_circ_	-0.24729	0.216443
hsa_circ_	0.993988	0.216448
hsa_circ_	-0.36237	0.216453
hsa_circ_	-0.31353	0.216454
hsa_circ_	-0.3843	0.216485
hsa_circ_	-0.80212	0.216507
hsa_circ_	0.303498	0.216523
hsa_circ_	0.458086	0.216523
hsa_circ_	-0.33511	0.216526

hsa_circ_	-0.21124	0.216536
hsa_circ_	0.297582	0.216553
hsa_circ_	-0.26182	0.216571
hsa_circ_	0.365628	0.216574
hsa_circ_	-0.20501	0.21658
hsa_circ_	-0.34485	0.216592
hsa_circ_	0.255398	0.216607
hsa_circ_	-1.07874	0.216619
hsa_circ_	-0.16529	0.21663
hsa_circ_	-0.19403	0.216631
hsa_circ_	-0.24343	0.216645
hsa_circ_	-0.17903	0.216645
hsa_circ_	0.188514	0.216656
hsa_circ_	0.509889	0.216657
hsa_circ_	0.285144	0.216664
hsa_circ_	-0.41891	0.216669
hsa_circ_	0.241453	0.216686
hsa_circ_	-0.28324	0.21669
hsa_circ_	0.353135	0.216706
hsa_circ_	0.242849	0.216709
hsa_circ_	-0.20236	0.21672
hsa_circ_	-0.62529	0.216723
hsa_circ_	-0.20214	0.21673
hsa_circ_	-0.59589	0.21673
hsa_circ_	0.245263	0.216747
hsa_circ_	-0.25208	0.21676
hsa_circ_	0.195476	0.216765
hsa_circ_	0.613575	0.216774
hsa_circ_	-0.5327	0.216776
hsa_circ_	0.196862	0.21678
hsa_circ_	0.274494	0.216796
hsa_circ_	-0.24756	0.216796
hsa_circ_	-0.22411	0.216818
hsa_circ_	-0.25954	0.216822
hsa_circ_	-0.35648	0.216853
hsa_circ_	-0.35754	0.216867
hsa_circ_	-0.22746	0.216871
hsa_circ_	-0.51927	0.216875
hsa_circ_	0.238992	0.21688
hsa_circ_	-0.22647	0.216881
hsa_circ_	-0.25968	0.216888
hsa_circ_	-0.29903	0.216896
hsa_circ_	0.251459	0.216915
hsa_circ_	0.224245	0.216917
hsa_circ_	-0.29915	0.216937
hsa_circ_	0.209466	0.21695
hsa_circ_	0.652053	0.216951
hsa_circ_	0.364925	0.216952
hsa_circ_	0.666759	0.216969
hsa_circ_	-0.64991	0.216977
hsa_circ_	0.195122	0.21698
hsa_circ_	0.295859	0.21698
hsa_circ_	0.18123	0.217003
hsa_circ_	0.359086	0.217014

hsa_circ_	-0.2711	0.217021
hsa_circ_	-0.28462	0.217027
hsa_circ_	-0.20694	0.217027
hsa_circ_	-0.17497	0.21703
hsa_circ_	-0.29783	0.217042
hsa_circ_	0.229178	0.21705
hsa_circ_	-0.81358	0.217059
hsa_circ_	-0.3801	0.217061
hsa_circ_	-0.23392	0.217082
hsa_circ_	0.464052	0.217086
hsa_circ_	-1.06205	0.217153
hsa_circ_	0.243997	0.21716
hsa_circ_	0.185654	0.217173
hsa_circ_	-0.57637	0.217195
hsa_circ_	-0.22102	0.217206
hsa_circ_	-0.27625	0.217236
hsa_circ_	0.214598	0.217256
hsa_circ_	0.421086	0.217258
hsa_circ_	0.309341	0.217273
hsa_circ_	0.399252	0.217283
hsa_circ_	0.558396	0.217287
hsa_circ_	0.272337	0.217301
hsa_circ_	-0.30782	0.217301
hsa_circ_	-0.38304	0.217306
hsa_circ_	-0.785	0.217309
hsa_circ_	0.199254	0.21731
hsa_circ_	-0.43029	0.217321
hsa_circ_	0.51044	0.217348
hsa_circ_	-0.8333	0.217356
hsa_circ_	-0.21146	0.217387
hsa_circ_	-0.23383	0.21742
hsa_circ_	0.477016	0.217461
hsa_circ_	0.298159	0.217465
hsa_circ_	-0.28391	0.21747
hsa_circ_	-0.194	0.217477
hsa_circ_	0.259667	0.217482
hsa_circ_	-0.7138	0.217501
hsa_circ_	-0.19614	0.217502
hsa_circ_	-0.52155	0.217512
hsa_circ_	-0.26626	0.217515
hsa_circ_	-0.20203	0.217519
hsa_circ_	0.238299	0.217524
hsa_circ_	0.219683	0.217525
hsa_circ_	-0.17742	0.217527
hsa_circ_	0.216113	0.217529
hsa_circ_	-0.18939	0.217539
hsa_circ_	0.220457	0.217544
hsa_circ_	0.246472	0.21755
hsa_circ_	0.599337	0.217554
hsa_circ_	-0.34704	0.217562
hsa_circ_	0.336595	0.217565
hsa_circ_	-0.3598	0.217572
hsa_circ_	-0.61729	0.217574
hsa_circ_	-0.39223	0.217577

hsa_circ_	-0.21385	0.217583
hsa_circ_	0.23762	0.217591
hsa_circ_	0.499709	0.217606
hsa_circ_	-0.39315	0.21761
hsa_circ_	-0.20154	0.217619
hsa_circ_	-0.55206	0.217626
hsa_circ_	-0.18106	0.217631
hsa_circ_	-0.22274	0.217636
hsa_circ_	-0.17634	0.217663
hsa_circ_	0.23411	0.217671
hsa_circ_	-0.29706	0.217674
hsa_circ_	-0.34406	0.217683
hsa_circ_	0.291366	0.217684
hsa_circ_	-0.39152	0.217698
hsa_circ_	0.202706	0.217712
hsa_circ_	-0.2799	0.217733
hsa_circ_	-0.40467	0.217741
hsa_circ_	0.267902	0.217746
hsa_circ_	-0.6039	0.217753
hsa_circ_	0.200567	0.217758
hsa_circ_	-0.29455	0.217766
hsa_circ_	-0.33399	0.217769
hsa_circ_	-0.23422	0.217782
hsa_circ_	-0.30145	0.217786
hsa_circ_	-0.65984	0.217792
hsa_circ_	0.263905	0.217796
hsa_circ_	-0.34924	0.217798
hsa_circ_	0.315215	0.217805
hsa_circ_	-0.21521	0.217806
hsa_circ_	-0.47348	0.217808
hsa_circ_	-0.21369	0.217821
hsa_circ_	-0.23907	0.217823
hsa_circ_	0.209837	0.217856
hsa_circ_	0.489401	0.217857
hsa_circ_	-0.22757	0.217887
hsa_circ_	0.712893	0.217905
hsa_circ_	-0.27029	0.217907
hsa_circ_	-0.29714	0.217909
hsa_circ_	-0.31497	0.217917
hsa_circ_	-0.46039	0.217978
hsa_circ_	-0.4423	0.217984
hsa_circ_	-0.20945	0.217987
hsa_circ_	0.868339	0.217998
hsa_circ_	0.557232	0.218001
hsa_circ_	-0.17642	0.218011
hsa_circ_	0.276529	0.218013
hsa_circ_	1.272258	0.21803
hsa_circ_	-1.32431	0.218031
hsa_circ_	0.55181	0.218038
hsa_circ_	0.175709	0.218045
hsa_circ_	0.26584	0.218048
hsa_circ_	-0.19816	0.218053
hsa_circ_	0.233318	0.218063
hsa_circ_	-0.31179	0.218065

hsa_circ_	-0.19981	0.218074
hsa_circ_	0.550229	0.218081
hsa_circ_	-0.30662	0.218086
hsa_circ_	-0.19149	0.218092
hsa_circ_	0.608713	0.218097
hsa_circ_	-0.27885	0.218107
hsa_circ_	-0.20348	0.218121
hsa_circ_	-0.23989	0.218134
hsa_circ_	0.285892	0.218148
hsa_circ_	0.301874	0.218153
hsa_circ_	0.644018	0.218153
hsa_circ_	-0.39216	0.218158
hsa_circ_	0.427721	0.218172
hsa_circ_	-0.19771	0.218174
hsa_circ_	0.195587	0.218175
hsa_circ_	0.568024	0.218184
hsa_circ_	-0.65835	0.218186
hsa_circ_	-0.61524	0.218188
hsa_circ_	0.311879	0.218189
hsa_circ_	0.2084	0.218193
hsa_circ_	0.631042	0.218202
hsa_circ_	0.190699	0.218209
hsa_circ_	0.343857	0.218223
hsa_circ_	-0.18166	0.218224
hsa_circ_	0.299878	0.218231
hsa_circ_	0.201203	0.218234
hsa_circ_	-1.56589	0.218234
hsa_circ_	-0.47767	0.218239
hsa_circ_	-0.23568	0.218249
hsa_circ_	-0.28505	0.218256
hsa_circ_	0.399663	0.218257
hsa_circ_	0.373152	0.218259
hsa_circ_	-0.2742	0.218276
hsa_circ_	-0.19704	0.218277
hsa_circ_	0.512777	0.218285
hsa_circ_	0.222209	0.218297
hsa_circ_	0.44112	0.218301
hsa_circ_	-0.24502	0.218306
hsa_circ_	-0.45302	0.218306
hsa_circ_	-0.24059	0.218325
hsa_circ_	-0.64439	0.218345
hsa_circ_	-0.25829	0.218364
hsa_circ_	1.775672	0.218366
hsa_circ_	-0.20932	0.21838
hsa_circ_	0.228893	0.218384
hsa_circ_	0.183193	0.218403
hsa_circ_	-0.43459	0.218405
hsa_circ_	-0.64843	0.218414
hsa_circ_	0.311524	0.218437
hsa_circ_	0.217902	0.218455
hsa_circ_	0.380333	0.218459
hsa_circ_	-0.26416	0.21846
hsa_circ_	0.227104	0.218466
hsa_circ_	0.220636	0.218468

hsa_circ_	0.797502	0.218504
hsa_circ_	-0.18895	0.218506
hsa_circ_	0.183883	0.218511
hsa_circ_	0.205545	0.218517
hsa_circ_	0.208753	0.218522
hsa_circ_	-0.43776	0.218525
hsa_circ_	-0.32574	0.218527
hsa_circ_	-0.20007	0.218531
hsa_circ_	0.218984	0.218534
hsa_circ_	0.888887	0.218536
hsa_circ_	-0.3781	0.218537
hsa_circ_	0.278104	0.218544
hsa_circ_	-0.21321	0.218555
hsa_circ_	0.256185	0.218557
hsa_circ_	-0.20251	0.218573
hsa_circ_	0.439049	0.218582
hsa_circ_	-0.22399	0.218583
hsa_circ_	-0.40839	0.218583
hsa_circ_	-0.18649	0.218585
hsa_circ_	0.304313	0.218597
hsa_circ_	0.264926	0.218602
hsa_circ_	0.189999	0.218606
hsa_circ_	-1.18449	0.218652
hsa_circ_	-0.32438	0.218664
hsa_circ_	-0.24434	0.218665
hsa_circ_	0.396992	0.218696
hsa_circ_	0.655752	0.218701
hsa_circ_	0.410228	0.218706
hsa_circ_	-0.29175	0.21871
hsa_circ_	0.198475	0.218718
hsa_circ_	0.182283	0.218719
hsa_circ_	-0.29697	0.218727
hsa_circ_	-0.17469	0.218735
hsa_circ_	-0.47858	0.218741
hsa_circ_	0.709641	0.218744
hsa_circ_	-0.17115	0.218762
hsa_circ_	0.173748	0.218765
hsa_circ_	0.308243	0.218768
hsa_circ_	-0.31113	0.218769
hsa_circ_	-0.48711	0.218787
hsa_circ_	-0.23432	0.218788
hsa_circ_	0.243911	0.218802
hsa_circ_	0.214381	0.218803
hsa_circ_	-0.17895	0.218819
hsa_circ_	-0.26723	0.218825
hsa_circ_	0.19815	0.218829
hsa_circ_	0.249918	0.218835
hsa_circ_	-0.29235	0.218839
hsa_circ_	0.230209	0.218856
hsa_circ_	0.28111	0.218885
hsa_circ_	-1.07302	0.218897
hsa_circ_	-0.30917	0.218915
hsa_circ_	-0.37552	0.218922
hsa_circ_	0.774919	0.218928

hsa_circ_	-0.22455	0.218931
hsa_circ_	-0.19885	0.218934
hsa_circ_	-0.19445	0.218939
hsa_circ_	0.321052	0.218942
hsa_circ_	-0.26153	0.218953
hsa_circ_	0.441933	0.218953
hsa_circ_	-0.47234	0.218987
hsa_circ_	-0.29561	0.218995
hsa_circ_	-0.39693	0.218997
hsa_circ_	-0.17553	0.219006
hsa_circ_	-0.29856	0.219009
hsa_circ_	-0.75217	0.219013
hsa_circ_	-0.20348	0.219033
hsa_circ_	0.257731	0.219084
hsa_circ_	-0.78575	0.2191
hsa_circ_	0.521379	0.21911
hsa_circ_	-0.23931	0.219116
hsa_circ_	-0.1919	0.219127
hsa_circ_	0.668393	0.219132
hsa_circ_	-0.32752	0.219137
hsa_circ_	0.198347	0.21914
hsa_circ_	-0.18467	0.219154
hsa_circ_	0.957047	0.21918
hsa_circ_	0.229223	0.219224
hsa_circ_	0.430846	0.219237
hsa_circ_	-0.18437	0.219277
hsa_circ_	-0.19775	0.21929
hsa_circ_	0.311841	0.219317
hsa_circ_	-0.27601	0.219319
hsa_circ_	0.302484	0.219331
hsa_circ_	0.438519	0.219338
hsa_circ_	-0.31239	0.219342
hsa_circ_	-1.17521	0.21935
hsa_circ_	0.236144	0.21935
hsa_circ_	0.330138	0.219363
hsa_circ_	-0.21338	0.219375
hsa_circ_	-0.27886	0.219401
hsa_circ_	-0.17295	0.219404
hsa_circ_	-0.23819	0.21941
hsa_circ_	-0.42759	0.219418
hsa_circ_	0.220608	0.21944
hsa_circ_	0.21116	0.219451
hsa_circ_	1.368212	0.219468
hsa_circ_	-0.19282	0.219476
hsa_circ_	-0.22497	0.219498
hsa_circ_	-0.33268	0.219511
hsa_circ_	0.24815	0.219515
hsa_circ_	0.313769	0.219521
hsa_circ_	-0.27633	0.219524
hsa_circ_	0.242057	0.219535
hsa_circ_	-0.22301	0.219539
hsa_circ_	0.337033	0.219564
hsa_circ_	-0.49897	0.219571
hsa_circ_	0.276235	0.219584

hsa_circ_	0.205474	0.219599
hsa_circ_	-0.21155	0.219603
hsa_circ_	0.823279	0.219606
hsa_circ_	0.211987	0.219611
hsa_circ_	-0.46268	0.219624
hsa_circ_	0.288003	0.219624
hsa_circ_	-0.63577	0.219633
hsa_circ_	-0.44112	0.219667
hsa_circ_	-0.63701	0.219668
hsa_circ_	0.219576	0.219681
hsa_circ_	0.81748	0.219699
hsa_circ_	0.292852	0.21972
hsa_circ_	0.400935	0.219743
hsa_circ_	0.185865	0.219746
hsa_circ_	0.322289	0.219749
hsa_circ_	0.344781	0.21975
hsa_circ_	-0.34884	0.219752
hsa_circ_	-0.29588	0.219782
hsa_circ_	0.386372	0.219785
hsa_circ_	0.307548	0.2198
hsa_circ_	0.214801	0.219803
hsa_circ_	0.595368	0.219804
hsa_circ_	0.219342	0.219846
hsa_circ_	-0.17108	0.219847
hsa_circ_	0.378203	0.219851
hsa_circ_	0.200173	0.219852
hsa_circ_	-0.18922	0.219862
hsa_circ_	0.224991	0.219863
hsa_circ_	0.331006	0.219864
hsa_circ_	-0.24149	0.219866
hsa_circ_	-0.29095	0.219867
hsa_circ_	0.237397	0.219872
hsa_circ_	0.326719	0.219875
hsa_circ_	-0.38652	0.219885
hsa_circ_	-0.50447	0.219887
hsa_circ_	0.326258	0.219893
hsa_circ_	-0.17411	0.219909
hsa_circ_	0.459364	0.219911
hsa_circ_	-0.5012	0.219924
hsa_circ_	0.20395	0.219943
hsa_circ_	-0.2262	0.21995
hsa_circ_	-1.38436	0.21996
hsa_circ_	-0.51073	0.219963
hsa_circ_	-0.20499	0.219988
hsa_circ_	-0.19752	0.219999
hsa_circ_	0.640351	0.220026
hsa_circ_	0.319875	0.220033
hsa_circ_	0.295008	0.22004
hsa_circ_	0.481207	0.220047
hsa_circ_	0.357791	0.220057
hsa_circ_	-0.23599	0.220068
hsa_circ_	-0.52256	0.220104
hsa_circ_	-0.19854	0.220115
hsa_circ_	-0.5038	0.220117

hsa_circ_	0.424295	0.220121
hsa_circ_	-0.1988	0.220147
hsa_circ_	-0.28426	0.22015
hsa_circ_	-0.26676	0.220152
hsa_circ_	-0.18736	0.220156
hsa_circ_	0.179302	0.220173
hsa_circ_	-0.30885	0.220178
hsa_circ_	-1.275	0.220184
hsa_circ_	-0.54109	0.220205
hsa_circ_	-0.17304	0.220206
hsa_circ_	0.178849	0.220208
hsa_circ_	0.190818	0.220209
hsa_circ_	0.303462	0.220216
hsa_circ_	-0.35214	0.220229
hsa_circ_	-0.31387	0.220246
hsa_circ_	-0.2164	0.220253
hsa_circ_	0.404029	0.220278
hsa_circ_	0.225464	0.220298
hsa_circ_	-0.43525	0.220306
hsa_circ_	-0.79892	0.220309
hsa_circ_	-0.2468	0.220318
hsa_circ_	-0.28855	0.220323
hsa_circ_	-0.30857	0.22033
hsa_circ_	0.368063	0.220346
hsa_circ_	-0.29012	0.220349
hsa_circ_	0.786293	0.220367
hsa_circ_	-0.58202	0.220373
hsa_circ_	0.722486	0.220381
hsa_circ_	0.558301	0.220383
hsa_circ_	0.248705	0.220388
hsa_circ_	0.289844	0.220393
hsa_circ_	0.592353	0.220406
hsa_circ_	1.242439	0.220415
hsa_circ_	0.276001	0.22043
hsa_circ_	-0.29661	0.22043
hsa_circ_	-0.64943	0.22043
hsa_circ_	0.282941	0.220434
hsa_circ_	0.23819	0.220442
hsa_circ_	-0.26495	0.220446
hsa_circ_	0.251016	0.220447
hsa_circ_	0.342166	0.220449
hsa_circ_	-0.224	0.220472
hsa_circ_	-0.5326	0.220476
hsa_circ_	-0.55306	0.220479
hsa_circ_	-0.35527	0.220491
hsa_circ_	-0.29356	0.220496
hsa_circ_	-0.2694	0.220526
hsa_circ_	0.806602	0.220539
hsa_circ_	0.264929	0.220577
hsa_circ_	-0.20373	0.220589
hsa_circ_	0.39783	0.220622
hsa_circ_	-0.27761	0.220629
hsa_circ_	-0.43395	0.22067
hsa_circ_	-0.23911	0.220678

hsa_circ_	0.232919	0.220711
hsa_circ_	0.164461	0.220737
hsa_circ_	0.437338	0.220751
hsa_circ_	0.300098	0.22077
hsa_circ_	0.694875	0.220772
hsa_circ_	-0.58255	0.220787
hsa_circ_	0.302701	0.220788
hsa_circ_	0.731899	0.220792
hsa_circ_	-0.2183	0.220795
hsa_circ_	-0.25955	0.220796
hsa_circ_	0.24126	0.220796
hsa_circ_	-0.21512	0.220807
hsa_circ_	-0.20384	0.220816
hsa_circ_	0.189833	0.220817
hsa_circ_	-0.22963	0.220822
hsa_circ_	-0.23136	0.220823
hsa_circ_	-0.2129	0.220826
hsa_circ_	-0.61398	0.220831
hsa_circ_	-0.40865	0.220834
hsa_circ_	0.371208	0.220837
hsa_circ_	0.237235	0.22084
hsa_circ_	-0.92287	0.220845
hsa_circ_	-0.43511	0.22085
hsa_circ_	-0.34664	0.22085
hsa_circ_	0.300176	0.220851
hsa_circ_	0.263703	0.220883
hsa_circ_	-0.67805	0.220893
hsa_circ_	-0.1953	0.220907
hsa_circ_	0.621749	0.220913
hsa_circ_	-0.40092	0.220924
hsa_circ_	0.634983	0.220925
hsa_circ_	0.334484	0.220931
hsa_circ_	-0.21826	0.220952
hsa_circ_	-0.45593	0.220964
hsa_circ_	0.998397	0.220973
hsa_circ_	0.335305	0.220997
hsa_circ_	-0.29759	0.220999
hsa_circ_	-0.17826	0.221071
hsa_circ_	-0.28253	0.221081
hsa_circ_	0.312138	0.221096
hsa_circ_	-0.45145	0.221098
hsa_circ_	0.205405	0.221102
hsa_circ_	-0.26962	0.221102
hsa_circ_	0.56019	0.221112
hsa_circ_	0.370145	0.221125
hsa_circ_	0.259051	0.221137
hsa_circ_	-0.17889	0.22114
hsa_circ_	0.318107	0.221142
hsa_circ_	0.218126	0.221153
hsa_circ_	0.212193	0.221161
hsa_circ_	-0.23482	0.221171
hsa_circ_	-0.33257	0.221174
hsa_circ_	-0.31351	0.221176
hsa_circ_	0.313178	0.221176

hsa_circ_	-0.38425	0.221178
hsa_circ_	-0.60141	0.221197
hsa_circ_	-0.28672	0.221218
hsa_circ_	-0.42461	0.221219
hsa_circ_	-0.32959	0.221222
hsa_circ_	0.278601	0.221236
hsa_circ_	-0.1922	0.221242
hsa_circ_	-0.35259	0.221264
hsa_circ_	0.667328	0.221264
hsa_circ_	0.532363	0.221279
hsa_circ_	-0.30852	0.221293
hsa_circ_	0.243766	0.221297
hsa_circ_	0.849589	0.221308
hsa_circ_	-0.30437	0.221308
hsa_circ_	0.169064	0.221314
hsa_circ_	0.26487	0.221317
hsa_circ_	0.435996	0.221318
hsa_circ_	-0.25888	0.221323
hsa_circ_	-0.22071	0.221334
hsa_circ_	-0.31776	0.221342
hsa_circ_	-0.49943	0.221346
hsa_circ_	0.49006	0.221354
hsa_circ_	-0.47539	0.221362
hsa_circ_	0.556783	0.221364
hsa_circ_	-1.3172	0.22137
hsa_circ_	0.279686	0.22137
hsa_circ_	-0.23293	0.221377
hsa_circ_	-0.39566	0.221396
hsa_circ_	0.776627	0.221405
hsa_circ_	-0.42081	0.221416
hsa_circ_	1.502866	0.221419
hsa_circ_	0.243615	0.221476
hsa_circ_	-0.31089	0.221504
hsa_circ_	-0.18557	0.221517
hsa_circ_	-0.23905	0.221517
hsa_circ_	-0.40118	0.221518
hsa_circ_	-0.31282	0.221528
hsa_circ_	-0.3201	0.22153
hsa_circ_	-0.22685	0.221531
hsa_circ_	0.664834	0.221539
hsa_circ_	0.360235	0.221549
hsa_circ_	0.455037	0.221557
hsa_circ_	0.25014	0.221558
hsa_circ_	0.50876	0.221572
hsa_circ_	-0.51063	0.221614
hsa_circ_	0.181693	0.221614
hsa_circ_	0.572438	0.221623
hsa_circ_	0.324037	0.221624
hsa_circ_	0.648793	0.221633
hsa_circ_	-0.20964	0.221637
hsa_circ_	0.269723	0.221639
hsa_circ_	0.222485	0.22164
hsa_circ_	-0.38557	0.221656
hsa_circ_	0.23864	0.221663

hsa_circ_	0.2777	0.221674
hsa_circ_	-0.2654	0.221686
hsa_circ_	-0.20694	0.221693
hsa_circ_	0.233461	0.221715
hsa_circ_	0.233555	0.22173
hsa_circ_	0.594544	0.22174
hsa_circ_	0.482601	0.221747
hsa_circ_	-0.39347	0.221749
hsa_circ_	0.222422	0.221751
hsa_circ_	0.267219	0.221752
hsa_circ_	-0.28978	0.221755
hsa_circ_	0.246877	0.221758
hsa_circ_	0.190027	0.22178
hsa_circ_	0.34433	0.221783
hsa_circ_	-0.28263	0.221789
hsa_circ_	-0.52886	0.221812
hsa_circ_	-0.23067	0.221844
hsa_circ_	-0.2572	0.22186
hsa_circ_	0.353498	0.221898
hsa_circ_	-0.22423	0.221907
hsa_circ_	0.225716	0.221928
hsa_circ_	-0.26006	0.221935
hsa_circ_	-2.04759	0.221962
hsa_circ_	-0.38673	0.221969
hsa_circ_	-0.22631	0.221974
hsa_circ_	-0.21305	0.221982
hsa_circ_	0.25174	0.221985
hsa_circ_	-0.2089	0.221989
hsa_circ_	0.662135	0.222014
hsa_circ_	-0.20635	0.222016
hsa_circ_	0.207727	0.22202
hsa_circ_	-0.43596	0.222028
hsa_circ_	-0.20898	0.222032
hsa_circ_	-0.26961	0.222032
hsa_circ_	-0.22154	0.222037
hsa_circ_	0.335114	0.222053
hsa_circ_	0.225782	0.222067
hsa_circ_	-0.19391	0.222071
hsa_circ_	0.23481	0.222072
hsa_circ_	0.220934	0.22208
hsa_circ_	0.279795	0.222088
hsa_circ_	-0.18675	0.222096
hsa_circ_	0.856424	0.222107
hsa_circ_	0.648394	0.222108
hsa_circ_	0.272091	0.222118
hsa_circ_	0.212906	0.222122
hsa_circ_	0.234813	0.222143
hsa_circ_	-0.19164	0.222154
hsa_circ_	-0.18706	0.222184
hsa_circ_	0.172123	0.222187
hsa_circ_	0.676307	0.222188
hsa_circ_	-0.63299	0.222225
hsa_circ_	-0.71471	0.22224
hsa_circ_	0.656407	0.22224

hsa_circ_	0.470799	0.222243
hsa_circ_	-0.63471	0.222252
hsa_circ_	0.847516	0.222256
hsa_circ_	0.211924	0.222269
hsa_circ_	-0.19179	0.222305
hsa_circ_	-0.24088	0.222362
hsa_circ_	-0.23808	0.222363
hsa_circ_	-0.17213	0.222365
hsa_circ_	-0.30343	0.22237
hsa_circ_	0.254081	0.222372
hsa_circ_	0.19202	0.222378
hsa_circ_	-0.95992	0.222384
hsa_circ_	0.177803	0.22239
hsa_circ_	0.175911	0.222405
hsa_circ_	0.522251	0.222415
hsa_circ_	0.417018	0.222426
hsa_circ_	-0.51911	0.222436
hsa_circ_	0.480876	0.22246
hsa_circ_	-0.21212	0.222492
hsa_circ_	-0.51228	0.222495
hsa_circ_	-0.2655	0.222502
hsa_circ_	-0.35092	0.222527
hsa_circ_	-0.5886	0.222539
hsa_circ_	0.303991	0.222554
hsa_circ_	-1.03401	0.222564
hsa_circ_	0.31291	0.222586
hsa_circ_	-0.23813	0.222588
hsa_circ_	-0.36767	0.222603
hsa_circ_	-0.21642	0.222615
hsa_circ_	-0.20276	0.222622
hsa_circ_	0.248732	0.222627
hsa_circ_	-0.71226	0.222628
hsa_circ_	0.217733	0.22263
hsa_circ_	-0.17223	0.222647
hsa_circ_	-0.32114	0.222653
hsa_circ_	0.35933	0.222653
hsa_circ_	0.685336	0.222659
hsa_circ_	0.291771	0.222659
hsa_circ_	0.344605	0.222662
hsa_circ_	0.197049	0.222664
hsa_circ_	-0.5975	0.222678
hsa_circ_	0.252658	0.222696
hsa_circ_	0.214094	0.222696
hsa_circ_	-0.42308	0.222707
hsa_circ_	-0.26709	0.222712
hsa_circ_	0.234549	0.222719
hsa_circ_	-0.29225	0.22273
hsa_circ_	-0.45639	0.222736
hsa_circ_	0.34671	0.222739
hsa_circ_	0.544258	0.222743
hsa_circ_	-0.1933	0.222749
hsa_circ_	-0.31581	0.222752
hsa_circ_	0.226147	0.222763
hsa_circ_	0.361484	0.222769

hsa_circ_	0.528711	0.222779
hsa_circ_	-0.18839	0.222784
hsa_circ_	0.181636	0.222803
hsa_circ_	-0.4222	0.222806
hsa_circ_	-0.25105	0.222814
hsa_circ_	-0.20712	0.222819
hsa_circ_	-0.19554	0.222835
hsa_circ_	-0.32428	0.222839
hsa_circ_	-0.17089	0.222848
hsa_circ_	-0.61322	0.222851
hsa_circ_	0.274302	0.222854
hsa_circ_	-1.06156	0.222856
hsa_circ_	-0.37425	0.222861
hsa_circ_	0.244237	0.222868
hsa_circ_	-0.27557	0.222882
hsa_circ_	-0.37614	0.222892
hsa_circ_	0.549409	0.222917
hsa_circ_	0.200134	0.222929
hsa_circ_	0.22815	0.222931
hsa_circ_	-0.23934	0.222936
hsa_circ_	0.615227	0.22296
hsa_circ_	0.597848	0.222962
hsa_circ_	-0.3126	0.222969
hsa_circ_	-0.24773	0.222977
hsa_circ_	-0.19195	0.222994
hsa_circ_	-0.30554	0.223004
hsa_circ_	-0.28039	0.223018
hsa_circ_	0.17943	0.223028
hsa_circ_	-0.27994	0.223054
hsa_circ_	0.209288	0.223055
hsa_circ_	0.36234	0.223056
hsa_circ_	-0.27494	0.223058
hsa_circ_	-0.17569	0.223064
hsa_circ_	-0.24036	0.223065
hsa_circ_	-0.21959	0.223083
hsa_circ_	0.237887	0.223084
hsa_circ_	0.697638	0.223087
hsa_circ_	0.224554	0.223091
hsa_circ_	0.319742	0.223143
hsa_circ_	-0.16585	0.223177
hsa_circ_	-0.36509	0.223184
hsa_circ_	-0.17691	0.223187
hsa_circ_	0.184446	0.223194
hsa_circ_	-0.1838	0.223201
hsa_circ_	-0.18636	0.223206
hsa_circ_	0.341585	0.22322
hsa_circ_	0.246645	0.223247
hsa_circ_	0.513566	0.223265
hsa_circ_	-0.49717	0.22327
hsa_circ_	-0.21281	0.223278
hsa_circ_	-0.51572	0.223293
hsa_circ_	0.373071	0.22333
hsa_circ_	-0.20562	0.223335
hsa_circ_	1.01122	0.22335

hsa_circ_	-0.26575	0.22335
hsa_circ_	0.488742	0.223363
hsa_circ_	0.463727	0.223367
hsa_circ_	1.383218	0.223369
hsa_circ_	-0.16158	0.223376
hsa_circ_	0.255848	0.223383
hsa_circ_	0.385006	0.223399
hsa_circ_	0.506086	0.223402
hsa_circ_	0.270892	0.223402
hsa_circ_	-0.30983	0.223404
hsa_circ_	-0.83543	0.22341
hsa_circ_	0.231605	0.22342
hsa_circ_	-0.22954	0.223425
hsa_circ_	-0.27876	0.223435
hsa_circ_	-0.42942	0.223436
hsa_circ_	-0.19042	0.223438
hsa_circ_	0.906632	0.223455
hsa_circ_	0.303743	0.22347
hsa_circ_	-0.17156	0.223479
hsa_circ_	0.234188	0.223487
hsa_circ_	-0.20132	0.223492
hsa_circ_	0.352756	0.223501
hsa_circ_	0.249416	0.223509
hsa_circ_	0.227846	0.223514
hsa_circ_	-0.18485	0.223515
hsa_circ_	-0.19318	0.223519
hsa_circ_	0.274759	0.223526
hsa_circ_	0.30722	0.223536
hsa_circ_	0.198832	0.223556
hsa_circ_	-0.30303	0.223581
hsa_circ_	-0.6026	0.223585
hsa_circ_	0.328712	0.223585
hsa_circ_	0.256562	0.223625
hsa_circ_	0.214321	0.223627
hsa_circ_	-0.25442	0.223636
hsa_circ_	-0.68953	0.223637
hsa_circ_	-0.24093	0.223639
hsa_circ_	-0.20099	0.223643
hsa_circ_	0.257872	0.223647
hsa_circ_	-0.22806	0.223649
hsa_circ_	-0.20494	0.223651
hsa_circ_	-0.23132	0.223671
hsa_circ_	1.373673	0.223672
hsa_circ_	-0.20446	0.223678
hsa_circ_	-0.39553	0.223693
hsa_circ_	-0.23134	0.2237
hsa_circ_	0.258002	0.223734
hsa_circ_	-0.26728	0.223779
hsa_circ_	0.221214	0.223779
hsa_circ_	-0.48333	0.22379
hsa_circ_	-0.17931	0.223796
hsa_circ_	0.202352	0.223819
hsa_circ_	-0.17551	0.223825
hsa_circ_	0.504328	0.223826

hsa_circ_	-0.30303	0.223828
hsa_circ_	0.343857	0.223828
hsa_circ_	-0.288	0.223837
hsa_circ_	-0.22007	0.223844
hsa_circ_	0.531085	0.223872
hsa_circ_	0.223387	0.223873
hsa_circ_	-0.22104	0.223875
hsa_circ_	-0.27985	0.223882
hsa_circ_	-1.64179	0.223884
hsa_circ_	0.317926	0.223889
hsa_circ_	0.554995	0.22389
hsa_circ_	-0.17231	0.223899
hsa_circ_	-0.30606	0.223909
hsa_circ_	0.186136	0.223918
hsa_circ_	-0.1705	0.223957
hsa_circ_	0.388498	0.223972
hsa_circ_	-0.36291	0.223976
hsa_circ_	-0.59106	0.223976
hsa_circ_	0.525359	0.22398
hsa_circ_	0.358611	0.223984
hsa_circ_	-0.18337	0.22399
hsa_circ_	-0.18815	0.224004
hsa_circ_	-0.32702	0.224004
hsa_circ_	-0.29604	0.224008
hsa_circ_	-0.18254	0.224016
hsa_circ_	-0.60738	0.224017
hsa_circ_	0.630483	0.224018
hsa_circ_	0.39144	0.224025
hsa_circ_	0.264801	0.224051
hsa_circ_	-0.19416	0.22409
hsa_circ_	-0.54713	0.224099
hsa_circ_	0.350365	0.2241
hsa_circ_	-0.31995	0.224115
hsa_circ_	0.221659	0.224118
hsa_circ_	0.538017	0.224121
hsa_circ_	0.222426	0.224123
hsa_circ_	0.480774	0.224126
hsa_circ_	-0.43844	0.224152
hsa_circ_	0.549624	0.224152
hsa_circ_	0.356655	0.224177
hsa_circ_	-0.20732	0.224181
hsa_circ_	-0.29422	0.224203
hsa_circ_	-0.22849	0.224206
hsa_circ_	0.279694	0.22421
hsa_circ_	-0.4435	0.224214
hsa_circ_	-0.19123	0.224222
hsa_circ_	0.330872	0.224244
hsa_circ_	0.525858	0.224268
hsa_circ_	-0.26074	0.224273
hsa_circ_	0.168322	0.224278
hsa_circ_	0.334747	0.224298
hsa_circ_	0.18388	0.224301
hsa_circ_	0.307854	0.224302
hsa_circ_	0.843808	0.224307

hsa_circ_	-0.36153	0.224319
hsa_circ_	-0.26779	0.224322
hsa_circ_	0.525199	0.224342
hsa_circ_	-0.17791	0.22435
hsa_circ_	-0.18258	0.224356
hsa_circ_	-0.45119	0.224379
hsa_circ_	-0.29402	0.224381
hsa_circ_	-0.23366	0.224382
hsa_circ_	0.705448	0.224396
hsa_circ_	0.221158	0.224434
hsa_circ_	0.301395	0.224448
hsa_circ_	0.207894	0.224486
hsa_circ_	-0.23642	0.224491
hsa_circ_	0.307727	0.224508
hsa_circ_	0.307828	0.22452
hsa_circ_	0.201173	0.224544
hsa_circ_	0.665309	0.224565
hsa_circ_	-0.20256	0.224567
hsa_circ_	-0.2641	0.224583
hsa_circ_	0.252333	0.224592
hsa_circ_	-0.22399	0.224603
hsa_circ_	0.374824	0.224617
hsa_circ_	-0.50222	0.224625
hsa_circ_	0.233312	0.22464
hsa_circ_	0.286219	0.224664
hsa_circ_	-0.2778	0.224672
hsa_circ_	-0.36579	0.224681
hsa_circ_	-0.22308	0.224692
hsa_circ_	0.498346	0.224697
hsa_circ_	-0.20024	0.224703
hsa_circ_	0.179425	0.224714
hsa_circ_	-0.19871	0.224729
hsa_circ_	-0.26481	0.224752
hsa_circ_	-0.22072	0.224753
hsa_circ_	0.255974	0.224755
hsa_circ_	0.675564	0.224775
hsa_circ_	0.416665	0.224799
hsa_circ_	-0.25373	0.224802
hsa_circ_	0.88514	0.224821
hsa_circ_	0.199956	0.224826
hsa_circ_	0.226997	0.224863
hsa_circ_	-0.22854	0.224866
hsa_circ_	0.190591	0.224886
hsa_circ_	0.250151	0.224887
hsa_circ_	0.247696	0.224891
hsa_circ_	0.261439	0.224905
hsa_circ_	0.274905	0.224917
hsa_circ_	0.803369	0.224926
hsa_circ_	-0.2585	0.224939
hsa_circ_	-0.63304	0.224944
hsa_circ_	0.180582	0.22495
hsa_circ_	-0.19366	0.224957
hsa_circ_	0.262815	0.224968
hsa_circ_	0.201399	0.224973

hsa_circ_	-0.16667	0.224996
hsa_circ_	0.369869	0.225006
hsa_circ_	-0.19713	0.225029
hsa_circ_	0.341139	0.22503
hsa_circ_	-0.75442	0.225036
hsa_circ_	0.255091	0.225046
hsa_circ_	0.219741	0.225058
hsa_circ_	0.457034	0.22506
hsa_circ_	-0.18146	0.225069
hsa_circ_	0.330957	0.225094
hsa_circ_	-0.38528	0.225095
hsa_circ_	0.226841	0.225109
hsa_circ_	-0.20491	0.225113
hsa_circ_	-0.24393	0.225117
hsa_circ_	-0.23206	0.225123
hsa_circ_	-0.45309	0.225125
hsa_circ_	0.249127	0.225133
hsa_circ_	0.268008	0.225142
hsa_circ_	-0.20175	0.22516
hsa_circ_	-0.45378	0.225186
hsa_circ_	-0.79955	0.225212
hsa_circ_	-0.25219	0.225214
hsa_circ_	-0.39625	0.225217
hsa_circ_	-0.35437	0.225247
hsa_circ_	-0.17236	0.225264
hsa_circ_	-0.47722	0.225269
hsa_circ_	-0.23107	0.225269
hsa_circ_	0.345119	0.225287
hsa_circ_	0.402362	0.225295
hsa_circ_	0.246593	0.225308
hsa_circ_	0.316993	0.225316
hsa_circ_	0.240046	0.225354
hsa_circ_	-0.59533	0.225369
hsa_circ_	-0.22674	0.22537
hsa_circ_	0.204808	0.22537
hsa_circ_	-0.2017	0.225378
hsa_circ_	-1.86126	0.225392
hsa_circ_	-1.08115	0.225395
hsa_circ_	-0.31633	0.2254
hsa_circ_	-0.20801	0.225426
hsa_circ_	-0.20248	0.225432
hsa_circ_	0.466333	0.225436
hsa_circ_	-0.25755	0.22546
hsa_circ_	-0.32532	0.225461
hsa_circ_	0.237396	0.225466
hsa_circ_	0.290679	0.225468
hsa_circ_	-0.27705	0.225473
hsa_circ_	-0.33619	0.225473
hsa_circ_	-0.36545	0.225474
hsa_circ_	-0.21964	0.225489
hsa_circ_	0.238193	0.225498
hsa_circ_	-0.19625	0.225499
hsa_circ_	-0.19937	0.225502
hsa_circ_	-0.1962	0.225504

hsa_circ_	-0.5758	0.225524
hsa_circ_	1.038432	0.225541
hsa_circ_	-0.19969	0.225543
hsa_circ_	0.17393	0.22555
hsa_circ_	0.209752	0.225553
hsa_circ_	-0.3713	0.225555
hsa_circ_	0.176305	0.225556
hsa_circ_	-0.174	0.225563
hsa_circ_	-0.51517	0.22559
hsa_circ_	-0.22564	0.225596
hsa_circ_	0.185958	0.2256
hsa_circ_	0.218526	0.225606
hsa_circ_	-0.249	0.225618
hsa_circ_	-0.24641	0.225623
hsa_circ_	0.518662	0.22563
hsa_circ_	-0.18068	0.225649
hsa_circ_	-0.1778	0.225651
hsa_circ_	0.303893	0.225658
hsa_circ_	0.336428	0.225659
hsa_circ_	0.230217	0.225661
hsa_circ_	-0.27085	0.225678
hsa_circ_	-0.2329	0.225684
hsa_circ_	-0.23947	0.225709
hsa_circ_	-0.21648	0.225785
hsa_circ_	-0.226	0.225794
hsa_circ_	0.251281	0.225822
hsa_circ_	-1.30875	0.225842
hsa_circ_	-0.20838	0.225848
hsa_circ_	0.218061	0.225849
hsa_circ_	-0.36914	0.225863
hsa_circ_	0.331537	0.225871
hsa_circ_	-0.24305	0.225877
hsa_circ_	-0.2949	0.225885
hsa_circ_	-0.17925	0.225887
hsa_circ_	-0.2683	0.22589
hsa_circ_	-0.24593	0.22589
hsa_circ_	-0.27758	0.225896
hsa_circ_	0.308288	0.225919
hsa_circ_	0.281427	0.22592
hsa_circ_	0.243129	0.22593
hsa_circ_	0.248998	0.225947
hsa_circ_	-0.29475	0.22595
hsa_circ_	0.227744	0.225959
hsa_circ_	0.239519	0.225979
hsa_circ_	0.197989	0.225996
hsa_circ_	0.255249	0.225998
hsa_circ_	0.451447	0.226012
hsa_circ_	-0.29765	0.226014
hsa_circ_	0.29337	0.226015
hsa_circ_	-0.18413	0.22603
hsa_circ_	0.33836	0.226036
hsa_circ_	-0.2623	0.226046
hsa_circ_	0.198539	0.226058
hsa_circ_	-0.29823	0.226058

hsa_circ_	-0.22106	0.226063
hsa_circ_	-0.21491	0.226109
hsa_circ_	-0.61622	0.226113
hsa_circ_	0.211941	0.226114
hsa_circ_	-0.39911	0.226123
hsa_circ_	0.175837	0.226127
hsa_circ_	0.579258	0.226171
hsa_circ_	-1.65088	0.226174
hsa_circ_	-0.77987	0.226179
hsa_circ_	0.711494	0.226186
hsa_circ_	0.398709	0.226201
hsa_circ_	0.279195	0.226217
hsa_circ_	0.211396	0.226223
hsa_circ_	-0.37091	0.226233
hsa_circ_	-0.33926	0.226238
hsa_circ_	-0.40243	0.226246
hsa_circ_	0.57657	0.226275
hsa_circ_	0.300297	0.22632
hsa_circ_	-0.25474	0.226323
hsa_circ_	0.203696	0.226344
hsa_circ_	0.530975	0.226359
hsa_circ_	0.204863	0.226359
hsa_circ_	-0.26291	0.226361
hsa_circ_	-0.1849	0.226365
hsa_circ_	0.772491	0.226371
hsa_circ_	0.317483	0.226392
hsa_circ_	-0.21988	0.226401
hsa_circ_	0.231554	0.226414
hsa_circ_	0.496134	0.226414
hsa_circ_	-0.35394	0.226421
hsa_circ_	-0.31744	0.226425
hsa_circ_	0.428695	0.226438
hsa_circ_	-0.18779	0.226456
hsa_circ_	0.393996	0.226457
hsa_circ_	-0.18067	0.22646
hsa_circ_	-0.32617	0.226476
hsa_circ_	-0.60423	0.226479
hsa_circ_	-0.38581	0.226484
hsa_circ_	0.185292	0.226499
hsa_circ_	-0.22661	0.226524
hsa_circ_	0.260946	0.226535
hsa_circ_	-0.74668	0.226539
hsa_circ_	-0.21222	0.226559
hsa_circ_	-0.24452	0.226562
hsa_circ_	0.732224	0.226566
hsa_circ_	0.268028	0.226586
hsa_circ_	-0.26233	0.226635
hsa_circ_	0.608359	0.226651
hsa_circ_	0.180087	0.226652
hsa_circ_	0.218243	0.226663
hsa_circ_	-0.22834	0.226663
hsa_circ_	0.864604	0.226678
hsa_circ_	-0.20172	0.226697
hsa_circ_	-0.19001	0.226741

hsa_circ_	-0.23575	0.226741
hsa_circ_	0.469201	0.226791
hsa_circ_	0.298667	0.226802
hsa_circ_	0.20011	0.226813
hsa_circ_	-1.00952	0.226818
hsa_circ_	0.24673	0.22683
hsa_circ_	0.266635	0.226836
hsa_circ_	-0.44011	0.226838
hsa_circ_	0.861909	0.226839
hsa_circ_	-0.16513	0.226847
hsa_circ_	-0.43005	0.226852
hsa_circ_	-0.82533	0.226854
hsa_circ_	-0.43395	0.226864
hsa_circ_	-0.22444	0.226865
hsa_circ_	0.260677	0.226882
hsa_circ_	-0.24533	0.226907
hsa_circ_	-0.27723	0.226916
hsa_circ_	0.31202	0.226918
hsa_circ_	-0.39665	0.226922
hsa_circ_	0.271676	0.22693
hsa_circ_	-0.31129	0.226935
hsa_circ_	0.621472	0.226955
hsa_circ_	-0.27893	0.226958
hsa_circ_	-0.29551	0.22697
hsa_circ_	-0.32229	0.226974
hsa_circ_	0.194062	0.226986
hsa_circ_	0.208869	0.226992
hsa_circ_	-0.25959	0.227011
hsa_circ_	0.215283	0.227036
hsa_circ_	-0.28098	0.227039
hsa_circ_	0.271902	0.227039
hsa_circ_	0.381239	0.227043
hsa_circ_	-0.16792	0.227045
hsa_circ_	0.323383	0.227047
hsa_circ_	-0.2318	0.227049
hsa_circ_	0.294926	0.227056
hsa_circ_	0.282962	0.227058
hsa_circ_	-2.95095	0.227062
hsa_circ_	-0.61609	0.227063
hsa_circ_	-0.23423	0.227067
hsa_circ_	0.243563	0.227076
hsa_circ_	-0.2296	0.227086
hsa_circ_	0.20874	0.227091
hsa_circ_	0.173524	0.227107
hsa_circ_	-0.22573	0.227144
hsa_circ_	0.285521	0.227146
hsa_circ_	0.395485	0.227154
hsa_circ_	-0.44561	0.227154
hsa_circ_	-0.63464	0.227158
hsa_circ_	0.224365	0.227158
hsa_circ_	-0.45706	0.22716
hsa_circ_	0.278751	0.227174
hsa_circ_	0.312724	0.227183
hsa_circ_	0.267154	0.227183

hsa_circ_	0.548387	0.227185
hsa_circ_	-0.41161	0.22719
hsa_circ_	0.211313	0.227197
hsa_circ_	-0.26396	0.227201
hsa_circ_	-0.21225	0.22721
hsa_circ_	0.24233	0.227218
hsa_circ_	-0.17459	0.227224
hsa_circ_	-0.21661	0.227236
hsa_circ_	-0.72473	0.22726
hsa_circ_	0.335392	0.227292
hsa_circ_	0.21933	0.227295
hsa_circ_	0.676819	0.227321
hsa_circ_	-0.2796	0.227328
hsa_circ_	-0.2206	0.227342
hsa_circ_	0.850351	0.227366
hsa_circ_	-0.22714	0.227377
hsa_circ_	-0.24219	0.227381
hsa_circ_	-0.16971	0.227385
hsa_circ_	-0.62415	0.227389
hsa_circ_	0.192506	0.227389
hsa_circ_	0.213019	0.227394
hsa_circ_	0.179169	0.22743
hsa_circ_	0.588635	0.227437
hsa_circ_	-0.38108	0.22744
hsa_circ_	0.282556	0.227445
hsa_circ_	0.203105	0.227449
hsa_circ_	-0.17856	0.227461
hsa_circ_	0.298414	0.227477
hsa_circ_	0.416494	0.227482
hsa_circ_	0.258571	0.227483
hsa_circ_	-0.20224	0.227494
hsa_circ_	-0.22478	0.22751
hsa_circ_	-0.18144	0.227556
hsa_circ_	-0.42511	0.227563
hsa_circ_	-0.35628	0.227564
hsa_circ_	0.239905	0.227619
hsa_circ_	1.179243	0.227629
hsa_circ_	-0.20129	0.22763
hsa_circ_	-0.3617	0.227632
hsa_circ_	0.192579	0.227636
hsa_circ_	-0.21628	0.22764
hsa_circ_	0.217028	0.227652
hsa_circ_	-0.44038	0.227663
hsa_circ_	0.214445	0.227691
hsa_circ_	-0.17345	0.227695
hsa_circ_	-0.21387	0.227697
hsa_circ_	0.388642	0.227699
hsa_circ_	-0.22067	0.227702
hsa_circ_	0.337982	0.227704
hsa_circ_	0.297214	0.227707
hsa_circ_	0.319748	0.227709
hsa_circ_	0.439574	0.227729
hsa_circ_	0.253035	0.22773
hsa_circ_	0.255279	0.227731

hsa_circ_	-1.00281	0.227733
hsa_circ_	0.191119	0.227761
hsa_circ_	0.56851	0.227762
hsa_circ_	0.210679	0.227764
hsa_circ_	-0.2575	0.227765
hsa_circ_	0.348315	0.227789
hsa_circ_	0.304272	0.227812
hsa_circ_	-0.32463	0.227827
hsa_circ_	-0.18084	0.227827
hsa_circ_	0.447168	0.227833
hsa_circ_	-0.18306	0.227834
hsa_circ_	-0.4791	0.227845
hsa_circ_	-0.22104	0.227865
hsa_circ_	-0.36615	0.227878
hsa_circ_	1.117856	0.227884
hsa_circ_	0.436632	0.227894
hsa_circ_	-0.37952	0.227896
hsa_circ_	0.281348	0.22791
hsa_circ_	-0.22353	0.227931
hsa_circ_	0.261558	0.227937
hsa_circ_	0.519543	0.227943
hsa_circ_	-0.19738	0.227955
hsa_circ_	-0.33534	0.227967
hsa_circ_	-0.29764	0.227973
hsa_circ_	-0.33893	0.227974
hsa_circ_	-0.20207	0.227977
hsa_circ_	-0.21406	0.227979
hsa_circ_	0.351776	0.227981
hsa_circ_	0.948168	0.227981
hsa_circ_	-0.36349	0.227993
hsa_circ_	-1.55337	0.228005
hsa_circ_	1.045596	0.228021
hsa_circ_	0.933927	0.228026
hsa_circ_	0.2473	0.228048
hsa_circ_	0.248587	0.228057
hsa_circ_	-0.39896	0.228088
hsa_circ_	-0.22782	0.228091
hsa_circ_	-0.33232	0.228113
hsa_circ_	0.288761	0.228125
hsa_circ_	-0.3411	0.228162
hsa_circ_	-0.30544	0.228165
hsa_circ_	-0.18531	0.228166
hsa_circ_	0.271006	0.228181
hsa_circ_	-0.42675	0.228182
hsa_circ_	0.426973	0.228192
hsa_circ_	-0.25758	0.228212
hsa_circ_	0.847319	0.228218
hsa_circ_	-0.31852	0.22825
hsa_circ_	-0.26541	0.228257
hsa_circ_	0.191046	0.228272
hsa_circ_	0.1644	0.228309
hsa_circ_	-0.21782	0.228311
hsa_circ_	-0.34599	0.228313
hsa_circ_	-0.18045	0.228314

hsa_circ_	-0.17865	0.228335
hsa_circ_	1.748085	0.228335
hsa_circ_	0.883132	0.228339
hsa_circ_	-0.65491	0.228345
hsa_circ_	0.243044	0.228349
hsa_circ_	-0.32209	0.228353
hsa_circ_	-0.25184	0.228354
hsa_circ_	-0.66566	0.228361
hsa_circ_	-0.22402	0.228367
hsa_circ_	0.887442	0.228368
hsa_circ_	0.361176	0.228386
hsa_circ_	-0.38154	0.228388
hsa_circ_	0.252493	0.228397
hsa_circ_	0.374039	0.228405
hsa_circ_	-0.36023	0.228419
hsa_circ_	-0.16281	0.228422
hsa_circ_	0.371114	0.228434
hsa_circ_	0.258582	0.228496
hsa_circ_	0.381123	0.228497
hsa_circ_	-0.18401	0.228513
hsa_circ_	-0.22435	0.228521
hsa_circ_	-0.16779	0.228529
hsa_circ_	0.356647	0.228543
hsa_circ_	0.418677	0.228547
hsa_circ_	-0.61387	0.228554
hsa_circ_	0.671943	0.228561
hsa_circ_	0.20579	0.228563
hsa_circ_	-0.2922	0.228566
hsa_circ_	0.269778	0.228572
hsa_circ_	0.187043	0.228583
hsa_circ_	0.205784	0.228589
hsa_circ_	0.199395	0.228595
hsa_circ_	-0.56637	0.228595
hsa_circ_	-0.38472	0.22862
hsa_circ_	0.29485	0.228665
hsa_circ_	-0.19116	0.228674
hsa_circ_	-0.52461	0.228678
hsa_circ_	0.268956	0.228724
hsa_circ_	0.381958	0.228725
hsa_circ_	-0.21234	0.228725
hsa_circ_	-0.16613	0.228727
hsa_circ_	0.217323	0.228729
hsa_circ_	-0.25551	0.22874
hsa_circ_	0.406316	0.228746
hsa_circ_	-0.24033	0.228752
hsa_circ_	-0.19544	0.228755
hsa_circ_	-0.20084	0.228758
hsa_circ_	0.210364	0.228769
hsa_circ_	0.352952	0.22877
hsa_circ_	0.171948	0.228791
hsa_circ_	-0.40822	0.228792
hsa_circ_	0.382896	0.228818
hsa_circ_	-0.21279	0.228849
hsa_circ_	-0.17405	0.228862

hsa_circ_	-0.24743	0.228875
hsa_circ_	-0.17633	0.228885
hsa_circ_	0.286896	0.22889
hsa_circ_	-0.18674	0.228904
hsa_circ_	-0.31926	0.228924
hsa_circ_	0.431608	0.228935
hsa_circ_	0.201173	0.228941
hsa_circ_	-0.23199	0.228951
hsa_circ_	-0.22045	0.228972
hsa_circ_	-0.67082	0.228977
hsa_circ_	-0.2961	0.228992
hsa_circ_	0.207355	0.229003
hsa_circ_	-0.43089	0.229007
hsa_circ_	0.670457	0.22902
hsa_circ_	0.801935	0.229037
hsa_circ_	-0.61407	0.229039
hsa_circ_	0.327725	0.22904
hsa_circ_	0.417557	0.229043
hsa_circ_	0.200824	0.229067
hsa_circ_	0.163839	0.229071
hsa_circ_	0.356721	0.229081
hsa_circ_	0.275079	0.229083
hsa_circ_	0.188462	0.229092
hsa_circ_	-0.35196	0.229093
hsa_circ_	-0.16637	0.229095
hsa_circ_	-0.19899	0.2291
hsa_circ_	-0.70136	0.229104
hsa_circ_	-0.27039	0.229109
hsa_circ_	0.251827	0.229112
hsa_circ_	-0.34775	0.229114
hsa_circ_	0.168158	0.229124
hsa_circ_	-0.2381	0.229129
hsa_circ_	0.823816	0.229132
hsa_circ_	-0.16298	0.229164
hsa_circ_	-0.19711	0.229174
hsa_circ_	0.385159	0.229179
hsa_circ_	-0.40382	0.229211
hsa_circ_	0.188195	0.229217
hsa_circ_	0.198843	0.229221
hsa_circ_	0.582332	0.229243
hsa_circ_	-0.23789	0.229257
hsa_circ_	-0.83735	0.229264
hsa_circ_	0.390743	0.229266
hsa_circ_	-0.19853	0.229272
hsa_circ_	-0.67433	0.229273
hsa_circ_	0.264281	0.22928
hsa_circ_	-0.27865	0.229287
hsa_circ_	0.394773	0.229291
hsa_circ_	-0.22202	0.229308
hsa_circ_	-0.25325	0.229326
hsa_circ_	0.258625	0.229326
hsa_circ_	0.178568	0.229328
hsa_circ_	0.197766	0.229346
hsa_circ_	0.360815	0.229353

hsa_circ_	-0.32259	0.229358
hsa_circ_	-0.45659	0.229373
hsa_circ_	-0.61266	0.229379
hsa_circ_	0.453556	0.229379
hsa_circ_	-0.26118	0.229387
hsa_circ_	-0.53454	0.229387
hsa_circ_	0.291097	0.229389
hsa_circ_	-0.70455	0.229405
hsa_circ_	0.34437	0.229425
hsa_circ_	-0.17915	0.22945
hsa_circ_	0.484663	0.22947
hsa_circ_	-0.21001	0.229496
hsa_circ_	0.259054	0.229519
hsa_circ_	0.211757	0.229523
hsa_circ_	-1.20713	0.229531
hsa_circ_	-0.2163	0.229541
hsa_circ_	-0.43486	0.229592
hsa_circ_	-0.17088	0.2296
hsa_circ_	0.315403	0.229617
hsa_circ_	0.34554	0.229633
hsa_circ_	0.223879	0.229642
hsa_circ_	0.272659	0.229649
hsa_circ_	0.278459	0.229663
hsa_circ_	0.36092	0.229665
hsa_circ_	-3.91388	0.22967
hsa_circ_	-0.20922	0.229685
hsa_circ_	-0.18053	0.229711
hsa_circ_	0.268395	0.229749
hsa_circ_	-0.31497	0.229755
hsa_circ_	-0.19184	0.229758
hsa_circ_	-0.59659	0.229766
hsa_circ_	1.708604	0.229778
hsa_circ_	-1.27928	0.229778
hsa_circ_	-0.22886	0.229779
hsa_circ_	-0.66916	0.229782
hsa_circ_	0.821283	0.22979
hsa_circ_	0.264199	0.229792
hsa_circ_	0.244904	0.229794
hsa_circ_	-0.2691	0.229801
hsa_circ_	-0.20596	0.229812
hsa_circ_	0.183184	0.229826
hsa_circ_	-0.72798	0.229829
hsa_circ_	-0.16129	0.229836
hsa_circ_	-0.43522	0.229847
hsa_circ_	0.683708	0.22987
hsa_circ_	0.234887	0.229871
hsa_circ_	-0.45755	0.229877
hsa_circ_	-0.39739	0.229879
hsa_circ_	-0.19134	0.229879
hsa_circ_	-0.20848	0.229885
hsa_circ_	-0.29996	0.229895
hsa_circ_	0.227778	0.2299
hsa_circ_	0.30307	0.229903
hsa_circ_	-0.39228	0.229914

hsa_circ_	-0.20435	0.229921
hsa_circ_	-0.22817	0.229924
hsa_circ_	-0.24674	0.229943
hsa_circ_	-0.26405	0.229968
hsa_circ_	-0.63944	0.229993
hsa_circ_	0.398657	0.230007
hsa_circ_	0.41748	0.230011
hsa_circ_	0.292793	0.23002
hsa_circ_	-0.44178	0.230027
hsa_circ_	0.291431	0.230038
hsa_circ_	-0.2122	0.230041
hsa_circ_	0.881477	0.230045
hsa_circ_	-0.2275	0.23006
hsa_circ_	-0.23972	0.230062
hsa_circ_	-0.20831	0.230072
hsa_circ_	0.222265	0.230085
hsa_circ_	-0.18689	0.230088
hsa_circ_	-0.16805	0.230089
hsa_circ_	-0.22056	0.230095
hsa_circ_	0.162962	0.230096
hsa_circ_	0.343527	0.230098
hsa_circ_	-0.24518	0.230131
hsa_circ_	-0.34074	0.230133
hsa_circ_	-0.33672	0.230142
hsa_circ_	-3.02846	0.230143
hsa_circ_	0.379079	0.230144
hsa_circ_	0.255517	0.230165
hsa_circ_	-0.64289	0.230169
hsa_circ_	0.252015	0.230192
hsa_circ_	0.206157	0.230213
hsa_circ_	0.594458	0.230218
hsa_circ_	0.482473	0.230222
hsa_circ_	0.585477	0.230227
hsa_circ_	0.198443	0.230239
hsa_circ_	-0.36842	0.230244
hsa_circ_	0.675949	0.23026
hsa_circ_	0.488191	0.230268
hsa_circ_	-0.37256	0.230287
hsa_circ_	-0.20557	0.23029
hsa_circ_	0.238872	0.230299
hsa_circ_	0.353057	0.230301
hsa_circ_	-0.16616	0.230315
hsa_circ_	-0.3139	0.230317
hsa_circ_	-0.2732	0.230325
hsa_circ_	-0.75256	0.230327
hsa_circ_	0.265777	0.230332
hsa_circ_	0.167665	0.230333
hsa_circ_	0.170839	0.230359
hsa_circ_	1.069426	0.230361
hsa_circ_	0.408376	0.230396
hsa_circ_	-0.33624	0.230398
hsa_circ_	-0.73055	0.230414
hsa_circ_	0.229634	0.230416
hsa_circ_	-0.28293	0.230419

hsa_circ_	-0.17825	0.230422
hsa_circ_	-0.25154	0.230424
hsa_circ_	-0.17483	0.230428
hsa_circ_	0.233687	0.23043
hsa_circ_	0.1854	0.230431
hsa_circ_	-0.319	0.230433
hsa_circ_	0.341759	0.230445
hsa_circ_	0.210608	0.230451
hsa_circ_	-0.18358	0.23046
hsa_circ_	-0.33698	0.230463
hsa_circ_	-0.25132	0.230473
hsa_circ_	0.580799	0.230481
hsa_circ_	-0.16747	0.230494
hsa_circ_	-0.17783	0.230498
hsa_circ_	-0.32915	0.230505
hsa_circ_	-1.77297	0.230506
hsa_circ_	0.332074	0.230517
hsa_circ_	-0.2682	0.230518
hsa_circ_	0.445659	0.230538
hsa_circ_	0.257569	0.230539
hsa_circ_	0.445008	0.23055
hsa_circ_	0.264641	0.230578
hsa_circ_	-0.19511	0.230582
hsa_circ_	0.243303	0.230587
hsa_circ_	0.334939	0.230591
hsa_circ_	0.342208	0.230608
hsa_circ_	-0.5235	0.230616
hsa_circ_	0.298283	0.230619
hsa_circ_	0.35356	0.230624
hsa_circ_	-0.25324	0.230637
hsa_circ_	0.290282	0.23064
hsa_circ_	0.294572	0.230651
hsa_circ_	-0.2453	0.230652
hsa_circ_	-0.2326	0.230655
hsa_circ_	0.258109	0.230655
hsa_circ_	-0.22296	0.230674
hsa_circ_	-0.18564	0.230685
hsa_circ_	-0.46156	0.230707
hsa_circ_	-0.20398	0.230711
hsa_circ_	0.268391	0.230725
hsa_circ_	-0.23247	0.230756
hsa_circ_	-0.30092	0.230756
hsa_circ_	-0.17781	0.230786
hsa_circ_	-0.5211	0.230789
hsa_circ_	-0.28579	0.23079
hsa_circ_	-0.92798	0.230797
hsa_circ_	0.297117	0.230807
hsa_circ_	-2.25468	0.230808
hsa_circ_	0.386006	0.23081
hsa_circ_	-0.20515	0.230818
hsa_circ_	0.527363	0.230849
hsa_circ_	0.232964	0.230859
hsa_circ_	0.333104	0.230878
hsa_circ_	-0.53624	0.230889

hsa_circ_	0.578669	0.230926
hsa_circ_	0.391199	0.230954
hsa_circ_	-0.23741	0.230974
hsa_circ_	0.376974	0.230976
hsa_circ_	-0.38952	0.230982
hsa_circ_	0.277505	0.230984
hsa_circ_	-0.52916	0.231009
hsa_circ_	0.740465	0.231022
hsa_circ_	0.288589	0.231031
hsa_circ_	-0.50023	0.231051
hsa_circ_	-0.42362	0.231057
hsa_circ_	-0.34102	0.23106
hsa_circ_	-0.21935	0.231062
hsa_circ_	-0.32588	0.23107
hsa_circ_	-0.22941	0.23109
hsa_circ_	0.174059	0.231121
hsa_circ_	0.842683	0.231136
hsa_circ_	-0.18654	0.231136
hsa_circ_	0.216989	0.231158
hsa_circ_	-0.32584	0.231165
hsa_circ_	0.167901	0.231167
hsa_circ_	-0.28625	0.231171
hsa_circ_	-0.47568	0.231172
hsa_circ_	0.206773	0.231175
hsa_circ_	0.224006	0.231176
hsa_circ_	-0.17606	0.231187
hsa_circ_	-0.1782	0.231188
hsa_circ_	-0.21064	0.231205
hsa_circ_	-0.25997	0.231205
hsa_circ_	-0.21485	0.231229
hsa_circ_	-0.25809	0.231238
hsa_circ_	-0.22506	0.231239
hsa_circ_	0.320864	0.231241
hsa_circ_	-0.24966	0.231243
hsa_circ_	-0.19556	0.231251
hsa_circ_	-0.18923	0.231253
hsa_circ_	0.229056	0.231257
hsa_circ_	-0.25859	0.231265
hsa_circ_	0.230301	0.231266
hsa_circ_	0.338864	0.231267
hsa_circ_	-0.48819	0.231276
hsa_circ_	0.428917	0.231281
hsa_circ_	-0.42601	0.231296
hsa_circ_	0.232139	0.231316
hsa_circ_	0.986325	0.231335
hsa_circ_	0.724601	0.231338
hsa_circ_	-0.44808	0.23134
hsa_circ_	0.170975	0.231349
hsa_circ_	-0.24589	0.231352
hsa_circ_	-0.81022	0.231355
hsa_circ_	0.259353	0.231369
hsa_circ_	-0.54939	0.23138
hsa_circ_	0.269137	0.231397
hsa_circ_	0.210744	0.231397

hsa_circ_	0.324626	0.231399
hsa_circ_	-0.22181	0.231413
hsa_circ_	-0.19186	0.231422
hsa_circ_	-0.23181	0.231425
hsa_circ_	0.244528	0.231431
hsa_circ_	0.19761	0.231432
hsa_circ_	0.627212	0.231439
hsa_circ_	0.24688	0.231441
hsa_circ_	-0.30944	0.231487
hsa_circ_	0.162303	0.231503
hsa_circ_	0.330252	0.231513
hsa_circ_	-0.67594	0.231516
hsa_circ_	0.195854	0.231522
hsa_circ_	-1.31534	0.231531
hsa_circ_	0.281892	0.231532
hsa_circ_	0.212091	0.231542
hsa_circ_	0.25402	0.231565
hsa_circ_	0.186837	0.23157
hsa_circ_	-0.29595	0.231577
hsa_circ_	0.27984	0.23158
hsa_circ_	-0.16287	0.231622
hsa_circ_	0.382139	0.231626
hsa_circ_	0.378519	0.231631
hsa_circ_	0.171591	0.231635
hsa_circ_	0.305995	0.231674
hsa_circ_	-0.18733	0.231674
hsa_circ_	-1.35557	0.231682
hsa_circ_	-0.16931	0.231684
hsa_circ_	-0.16389	0.231695
hsa_circ_	0.177859	0.231705
hsa_circ_	0.268583	0.231707
hsa_circ_	0.702939	0.231711
hsa_circ_	-0.62698	0.231715
hsa_circ_	0.268541	0.231739
hsa_circ_	0.500009	0.231741
hsa_circ_	-0.36104	0.231761
hsa_circ_	-0.4042	0.231768
hsa_circ_	0.219133	0.231774
hsa_circ_	-0.34103	0.231784
hsa_circ_	-0.23312	0.231786
hsa_circ_	-0.23359	0.231812
hsa_circ_	-0.41256	0.231818
hsa_circ_	0.360771	0.231841
hsa_circ_	0.263728	0.231845
hsa_circ_	0.220597	0.231849
hsa_circ_	0.389825	0.231852
hsa_circ_	-0.31649	0.231859
hsa_circ_	0.231792	0.231891
hsa_circ_	-0.41179	0.231894
hsa_circ_	-0.22928	0.231896
hsa_circ_	0.274199	0.231903
hsa_circ_	-0.36087	0.231904
hsa_circ_	-0.18208	0.231917
hsa_circ_	-0.37631	0.231941

hsa_circ_	-0.19245	0.231942
hsa_circ_	0.185136	0.231942
hsa_circ_	0.323455	0.231951
hsa_circ_	-0.24656	0.231987
hsa_circ_	-0.40872	0.231993
hsa_circ_	-0.55241	0.231995
hsa_circ_	0.500418	0.231997
hsa_circ_	0.396506	0.232005
hsa_circ_	0.416868	0.232024
hsa_circ_	0.48159	0.232054
hsa_circ_	-0.28286	0.232056
hsa_circ_	-0.18706	0.232066
hsa_circ_	0.193506	0.232074
hsa_circ_	-0.2048	0.232076
hsa_circ_	-0.33942	0.232102
hsa_circ_	-0.30239	0.232108
hsa_circ_	-0.5556	0.232122
hsa_circ_	-0.23579	0.232131
hsa_circ_	-2.82001	0.232138
hsa_circ_	-0.20509	0.232146
hsa_circ_	0.4639	0.232163
hsa_circ_	0.305905	0.232165
hsa_circ_	0.368718	0.232173
hsa_circ_	0.22859	0.232197
hsa_circ_	0.444497	0.232198
hsa_circ_	0.691759	0.232224
hsa_circ_	-0.45226	0.232234
hsa_circ_	0.184077	0.232239
hsa_circ_	0.312186	0.232254
hsa_circ_	-0.34065	0.232275
hsa_circ_	0.443056	0.232301
hsa_circ_	-0.43567	0.232304
hsa_circ_	0.284686	0.232306
hsa_circ_	-0.23856	0.232322
hsa_circ_	0.164077	0.232365
hsa_circ_	0.261987	0.232366
hsa_circ_	0.269592	0.232367
hsa_circ_	-0.18789	0.23243
hsa_circ_	-0.35729	0.232431
hsa_circ_	0.282875	0.232455
hsa_circ_	0.268129	0.232456
hsa_circ_	0.287075	0.232467
hsa_circ_	-0.23729	0.232471
hsa_circ_	0.211313	0.232474
hsa_circ_	-0.39927	0.232481
hsa_circ_	-0.33689	0.232491
hsa_circ_	-0.2532	0.2325
hsa_circ_	-0.24998	0.232507
hsa_circ_	-0.33927	0.232507
hsa_circ_	0.229996	0.232533
hsa_circ_	-0.17325	0.232543
hsa_circ_	-0.28405	0.232549
hsa_circ_	-0.22093	0.232555
hsa_circ_	0.292463	0.232569

hsa_circ_	0.182589	0.232597
hsa_circ_	-0.18663	0.232598
hsa_circ_	0.221187	0.232618
hsa_circ_	-0.21572	0.232624
hsa_circ_	0.445472	0.232624
hsa_circ_	0.229101	0.232637
hsa_circ_	-0.18446	0.23265
hsa_circ_	0.300844	0.232655
hsa_circ_	-0.24746	0.232664
hsa_circ_	-0.20415	0.232664
hsa_circ_	-0.23249	0.232691
hsa_circ_	0.270526	0.232707
hsa_circ_	-0.30538	0.232711
hsa_circ_	0.250357	0.23272
hsa_circ_	-0.20838	0.23272
hsa_circ_	0.24448	0.232731
hsa_circ_	0.171765	0.232733
hsa_circ_	0.242254	0.232737
hsa_circ_	0.22482	0.23275
hsa_circ_	0.208872	0.232754
hsa_circ_	-0.69154	0.232765
hsa_circ_	-0.31493	0.232776
hsa_circ_	0.748602	0.232787
hsa_circ_	-0.25442	0.232789
hsa_circ_	0.874591	0.232795
hsa_circ_	-0.34173	0.232799
hsa_circ_	-0.17365	0.232801
hsa_circ_	-0.38765	0.232801
hsa_circ_	0.36984	0.232802
hsa_circ_	0.350131	0.232808
hsa_circ_	0.197999	0.232812
hsa_circ_	0.212014	0.232825
hsa_circ_	-0.2529	0.232826
hsa_circ_	-0.19546	0.232833
hsa_circ_	-0.45242	0.232841
hsa_circ_	-0.19283	0.232845
hsa_circ_	0.220029	0.232855
hsa_circ_	-0.1754	0.232867
hsa_circ_	-0.21693	0.232867
hsa_circ_	0.378128	0.232881
hsa_circ_	-0.3091	0.232891
hsa_circ_	-0.17295	0.232912
hsa_circ_	0.22971	0.232933
hsa_circ_	0.341228	0.232944
hsa_circ_	0.443459	0.23295
hsa_circ_	0.644038	0.232951
hsa_circ_	0.176197	0.232953
hsa_circ_	0.329461	0.232956
hsa_circ_	0.662064	0.232964
hsa_circ_	0.982041	0.232981
hsa_circ_	-0.34361	0.232987
hsa_circ_	0.188223	0.232993
hsa_circ_	-0.18559	0.232995
hsa_circ_	-0.53603	0.233014

hsa_circ_	0.732553	0.233017
hsa_circ_	-0.24212	0.233018
hsa_circ_	-0.33259	0.233028
hsa_circ_	-0.26626	0.23303
hsa_circ_	0.423292	0.233035
hsa_circ_	0.210549	0.233039
hsa_circ_	-0.19595	0.233041
hsa_circ_	-0.23356	0.233085
hsa_circ_	-0.20982	0.233086
hsa_circ_	0.428164	0.233093
hsa_circ_	0.184175	0.233108
hsa_circ_	0.227128	0.233127
hsa_circ_	-0.58229	0.23313
hsa_circ_	0.197821	0.23313
hsa_circ_	0.660745	0.233134
hsa_circ_	0.223918	0.233155
hsa_circ_	0.651315	0.233193
hsa_circ_	-0.1633	0.233213
hsa_circ_	-0.67751	0.233222
hsa_circ_	0.227849	0.233232
hsa_circ_	0.273689	0.233247
hsa_circ_	-0.19139	0.233248
hsa_circ_	-0.20585	0.233262
hsa_circ_	-0.55633	0.233271
hsa_circ_	-0.17945	0.233273
hsa_circ_	-0.40809	0.233289
hsa_circ_	-0.21636	0.233297
hsa_circ_	0.719816	0.233303
hsa_circ_	-0.17973	0.233312
hsa_circ_	-0.38448	0.233324
hsa_circ_	-1.00473	0.233332
hsa_circ_	-0.25473	0.233334
hsa_circ_	-0.21603	0.233344
hsa_circ_	0.306585	0.233352
hsa_circ_	-0.34095	0.233371
hsa_circ_	-0.21777	0.233376
hsa_circ_	-0.30149	0.233396
hsa_circ_	0.177843	0.233398
hsa_circ_	-0.18214	0.233412
hsa_circ_	0.260676	0.233426
hsa_circ_	-0.21967	0.233429
hsa_circ_	-0.17195	0.233433
hsa_circ_	0.206927	0.233436
hsa_circ_	-0.51341	0.233441
hsa_circ_	-0.38048	0.233445
hsa_circ_	0.279521	0.233445
hsa_circ_	-0.62216	0.233456
hsa_circ_	0.298525	0.233464
hsa_circ_	0.480844	0.233472
hsa_circ_	0.241299	0.233477
hsa_circ_	-0.16492	0.233487
hsa_circ_	0.22215	0.23349
hsa_circ_	-0.57531	0.233496
hsa_circ_	0.273782	0.233507

hsa_circ_	-0.21053	0.233511
hsa_circ_	-0.19631	0.233555
hsa_circ_	-0.36444	0.233571
hsa_circ_	-0.17395	0.233571
hsa_circ_	-0.17957	0.233575
hsa_circ_	-0.24107	0.233583
hsa_circ_	0.39267	0.233605
hsa_circ_	0.315205	0.233612
hsa_circ_	0.271065	0.233612
hsa_circ_	0.273454	0.233628
hsa_circ_	0.265029	0.233634
hsa_circ_	-0.30356	0.233638
hsa_circ_	0.316716	0.233639
hsa_circ_	-0.26622	0.233653
hsa_circ_	0.619003	0.233661
hsa_circ_	-0.26561	0.233663
hsa_circ_	-0.17241	0.233671
hsa_circ_	-0.52886	0.233676
hsa_circ_	0.358746	0.233679
hsa_circ_	0.931861	0.233714
hsa_circ_	-0.45257	0.233742
hsa_circ_	-0.17833	0.233743
hsa_circ_	0.283629	0.233747
hsa_circ_	0.176444	0.233779
hsa_circ_	0.305856	0.233792
hsa_circ_	0.293937	0.233806
hsa_circ_	-0.5692	0.233861
hsa_circ_	0.308522	0.233863
hsa_circ_	0.737628	0.233864
hsa_circ_	0.440708	0.233864
hsa_circ_	0.340374	0.233876
hsa_circ_	-0.2717	0.233877
hsa_circ_	0.326828	0.233879
hsa_circ_	-0.24799	0.233888
hsa_circ_	-0.23854	0.233891
hsa_circ_	-0.19219	0.233908
hsa_circ_	0.27658	0.233925
hsa_circ_	0.339524	0.233933
hsa_circ_	-0.42519	0.233944
hsa_circ_	-0.66629	0.233948
hsa_circ_	0.651188	0.233952
hsa_circ_	0.382353	0.233952
hsa_circ_	-0.24876	0.233955
hsa_circ_	0.242095	0.233963
hsa_circ_	-0.42722	0.233963
hsa_circ_	-1.07499	0.233982
hsa_circ_	0.229894	0.233983
hsa_circ_	-0.23539	0.233985
hsa_circ_	-0.36218	0.233993
hsa_circ_	-0.61267	0.233995
hsa_circ_	0.33602	0.234
hsa_circ_	-0.50297	0.234002
hsa_circ_	-0.20153	0.234007
hsa_circ_	-0.20973	0.234013

hsa_circ_	-0.23304	0.234016
hsa_circ_	-0.2503	0.234019
hsa_circ_	-0.23103	0.234026
hsa_circ_	0.515011	0.23403
hsa_circ_	0.44101	0.234042
hsa_circ_	0.720282	0.234046
hsa_circ_	-0.24583	0.234046
hsa_circ_	0.207261	0.234046
hsa_circ_	0.188865	0.234074
hsa_circ_	-0.23598	0.23408
hsa_circ_	-0.15994	0.234085
hsa_circ_	-0.52083	0.234105
hsa_circ_	0.183093	0.234119
hsa_circ_	-0.64106	0.234122
hsa_circ_	-0.63137	0.234128
hsa_circ_	0.416011	0.234154
hsa_circ_	-0.67321	0.234156
hsa_circ_	0.573802	0.234157
hsa_circ_	0.435645	0.234159
hsa_circ_	0.214147	0.234184
hsa_circ_	-0.38757	0.234195
hsa_circ_	-0.20414	0.234207
hsa_circ_	-0.69469	0.234211
hsa_circ_	0.438436	0.234242
hsa_circ_	-0.6229	0.234281
hsa_circ_	0.269157	0.234282
hsa_circ_	0.625723	0.234315
hsa_circ_	-0.2066	0.234325
hsa_circ_	0.928658	0.234339
hsa_circ_	-0.24957	0.234358
hsa_circ_	0.161277	0.234359
hsa_circ_	-0.29951	0.23436
hsa_circ_	0.618555	0.234365
hsa_circ_	-0.23113	0.234402
hsa_circ_	0.332994	0.234417
hsa_circ_	0.400188	0.234434
hsa_circ_	-0.44085	0.234451
hsa_circ_	-0.21351	0.234452
hsa_circ_	0.353782	0.234454
hsa_circ_	0.31918	0.234464
hsa_circ_	0.268356	0.234483
hsa_circ_	0.174093	0.234485
hsa_circ_	0.637067	0.234493
hsa_circ_	-0.43186	0.234509
hsa_circ_	0.278127	0.234524
hsa_circ_	0.772458	0.234536
hsa_circ_	-0.20319	0.234544
hsa_circ_	-0.48767	0.234595
hsa_circ_	0.309676	0.234612
hsa_circ_	-0.31318	0.23463
hsa_circ_	0.235907	0.234633
hsa_circ_	0.219678	0.234636
hsa_circ_	0.553632	0.234638
hsa_circ_	0.334527	0.234662

hsa_circ_	0.378322	0.234678
hsa_circ_	-0.19195	0.234702
hsa_circ_	-0.25796	0.234704
hsa_circ_	-0.31934	0.234706
hsa_circ_	-0.35597	0.23473
hsa_circ_	-0.28953	0.23474
hsa_circ_	0.174119	0.234772
hsa_circ_	0.256512	0.234814
hsa_circ_	-0.35625	0.23482
hsa_circ_	0.395297	0.234838
hsa_circ_	0.493913	0.234844
hsa_circ_	-0.27808	0.234864
hsa_circ_	0.241675	0.234866
hsa_circ_	-0.21349	0.234898
hsa_circ_	0.237847	0.234902
hsa_circ_	-0.60234	0.234938
hsa_circ_	0.339424	0.234949
hsa_circ_	0.238828	0.234954
hsa_circ_	-0.17477	0.234965
hsa_circ_	0.190961	0.234966
hsa_circ_	-0.17143	0.234973
hsa_circ_	-0.22181	0.234978
hsa_circ_	-0.34763	0.234992
hsa_circ_	0.196036	0.234995
hsa_circ_	-0.18757	0.235012
hsa_circ_	-0.30799	0.235013
hsa_circ_	0.27971	0.235025
hsa_circ_	0.19558	0.235026
hsa_circ_	-0.24933	0.235066
hsa_circ_	-0.20513	0.23508
hsa_circ_	-0.38653	0.235087
hsa_circ_	0.219951	0.235121
hsa_circ_	-0.28333	0.235123
hsa_circ_	-0.30542	0.235123
hsa_circ_	0.184461	0.235144
hsa_circ_	0.255283	0.23515
hsa_circ_	0.392877	0.235153
hsa_circ_	0.220586	0.235154
hsa_circ_	-0.30653	0.235155
hsa_circ_	0.268096	0.23516
hsa_circ_	0.44279	0.235189
hsa_circ_	-0.48426	0.235212
hsa_circ_	-0.25883	0.23523
hsa_circ_	-0.21919	0.235231
hsa_circ_	-0.18836	0.235238
hsa_circ_	-0.4787	0.235286
hsa_circ_	0.517539	0.23529
hsa_circ_	0.239664	0.235297
hsa_circ_	-0.18859	0.235307
hsa_circ_	-0.44156	0.23531
hsa_circ_	0.259659	0.235322
hsa_circ_	-0.25102	0.235325
hsa_circ_	0.173476	0.235369
hsa_circ_	-0.23822	0.235374

hsa_circ_	0.269678	0.235395
hsa_circ_	0.45099	0.235405
hsa_circ_	0.324532	0.235408
hsa_circ_	0.219908	0.235415
hsa_circ_	-0.35004	0.235424
hsa_circ_	-0.23329	0.23543
hsa_circ_	-0.25087	0.235464
hsa_circ_	-0.2845	0.235466
hsa_circ_	0.278394	0.235475
hsa_circ_	0.184554	0.235477
hsa_circ_	-0.66435	0.235479
hsa_circ_	0.194693	0.23548
hsa_circ_	-0.39775	0.235487
hsa_circ_	0.871289	0.235496
hsa_circ_	0.286346	0.235525
hsa_circ_	0.217559	0.23558
hsa_circ_	-0.19197	0.23559
hsa_circ_	0.196481	0.235617
hsa_circ_	-0.20174	0.235626
hsa_circ_	-0.26831	0.235632
hsa_circ_	-0.41566	0.235651
hsa_circ_	-0.35437	0.235653
hsa_circ_	-0.27884	0.235655
hsa_circ_	0.162466	0.235656
hsa_circ_	0.632286	0.235661
hsa_circ_	-0.88451	0.235684
hsa_circ_	-0.20596	0.235711
hsa_circ_	-0.31267	0.235741
hsa_circ_	-0.21277	0.235748
hsa_circ_	0.161377	0.235763
hsa_circ_	-0.3286	0.235788
hsa_circ_	-0.37228	0.235806
hsa_circ_	-0.21002	0.235816
hsa_circ_	-0.17714	0.235818
hsa_circ_	0.406717	0.23583
hsa_circ_	-0.23107	0.235835
hsa_circ_	-0.19317	0.235835
hsa_circ_	-0.51276	0.235843
hsa_circ_	-0.22707	0.235845
hsa_circ_	-0.43796	0.235845
hsa_circ_	0.461625	0.235856
hsa_circ_	-0.22436	0.23587
hsa_circ_	0.69337	0.235872
hsa_circ_	0.270121	0.235891
hsa_circ_	0.278213	0.235914
hsa_circ_	-0.17493	0.235923
hsa_circ_	-0.26481	0.235944
hsa_circ_	0.220318	0.235955
hsa_circ_	-0.2156	0.235969
hsa_circ_	0.487327	0.235969
hsa_circ_	-0.37651	0.235973
hsa_circ_	-0.27364	0.235978
hsa_circ_	-0.31332	0.235984
hsa_circ_	-0.32545	0.235987

hsa_circ_	-0.25216	0.235992
hsa_circ_	0.215927	0.235998
hsa_circ_	0.234603	0.236004
hsa_circ_	-0.40859	0.236022
hsa_circ_	0.209461	0.236023
hsa_circ_	0.309145	0.236035
hsa_circ_	-0.21681	0.236035
hsa_circ_	0.197441	0.236037
hsa_circ_	-0.64438	0.236044
hsa_circ_	-0.31994	0.236049
hsa_circ_	-0.24848	0.236051
hsa_circ_	-0.177	0.236052
hsa_circ_	-0.33144	0.236055
hsa_circ_	0.738087	0.236062
hsa_circ_	-0.32331	0.236063
hsa_circ_	0.214366	0.236068
hsa_circ_	-0.31134	0.236075
hsa_circ_	-0.15908	0.236094
hsa_circ_	0.452003	0.236096
hsa_circ_	0.261977	0.236107
hsa_circ_	0.26282	0.23613
hsa_circ_	-0.16335	0.236132
hsa_circ_	-0.19219	0.23614
hsa_circ_	-0.86241	0.236168
hsa_circ_	0.336994	0.236169
hsa_circ_	0.180963	0.236172
hsa_circ_	0.339334	0.236182
hsa_circ_	0.202597	0.236197
hsa_circ_	0.292638	0.236227
hsa_circ_	0.315384	0.236233
hsa_circ_	0.304205	0.236236
hsa_circ_	-0.19542	0.236259
hsa_circ_	0.357537	0.23626
hsa_circ_	-0.24248	0.236266
hsa_circ_	0.196856	0.236304
hsa_circ_	-0.67309	0.236304
hsa_circ_	0.333053	0.236316
hsa_circ_	-0.17153	0.236317
hsa_circ_	-0.4489	0.236324
hsa_circ_	-0.19244	0.236339
hsa_circ_	-0.45029	0.236347
hsa_circ_	0.195182	0.236348
hsa_circ_	0.207673	0.236351
hsa_circ_	0.633112	0.236355
hsa_circ_	0.597068	0.236356
hsa_circ_	-0.4396	0.23636
hsa_circ_	-0.45816	0.236366
hsa_circ_	0.313978	0.23638
hsa_circ_	-0.17325	0.236386
hsa_circ_	-0.58935	0.236402
hsa_circ_	0.176108	0.236412
hsa_circ_	-0.17231	0.23642
hsa_circ_	-0.18459	0.23644
hsa_circ_	0.257741	0.236448

hsa_circ_	-0.43288	0.23646
hsa_circ_	-0.60236	0.236472
hsa_circ_	-0.50882	0.236492
hsa_circ_	0.199256	0.236502
hsa_circ_	0.398454	0.236512
hsa_circ_	-0.20857	0.23652
hsa_circ_	-0.18392	0.236534
hsa_circ_	0.518022	0.236545
hsa_circ_	-0.62737	0.236551
hsa_circ_	0.850095	0.236554
hsa_circ_	0.275865	0.236557
hsa_circ_	0.549727	0.236574
hsa_circ_	0.505198	0.236587
hsa_circ_	-0.19682	0.236639
hsa_circ_	-0.4089	0.236639
hsa_circ_	-0.29434	0.236642
hsa_circ_	0.261899	0.236659
hsa_circ_	-0.23844	0.236664
hsa_circ_	-0.40443	0.236684
hsa_circ_	-0.16953	0.236701
hsa_circ_	-0.27869	0.236701
hsa_circ_	-0.37055	0.236707
hsa_circ_	-0.17523	0.236735
hsa_circ_	0.217558	0.236735
hsa_circ_	0.323802	0.236771
hsa_circ_	0.174143	0.236782
hsa_circ_	0.396742	0.236788
hsa_circ_	-0.566	0.236788
hsa_circ_	0.166374	0.236789
hsa_circ_	0.185232	0.2368
hsa_circ_	-1.61198	0.236804
hsa_circ_	0.177557	0.236818
hsa_circ_	-1.37846	0.23684
hsa_circ_	-0.20668	0.236855
hsa_circ_	-0.37673	0.236874
hsa_circ_	0.643907	0.236887
hsa_circ_	-0.275	0.236893
hsa_circ_	-0.32156	0.236906
hsa_circ_	0.319958	0.236915
hsa_circ_	0.203445	0.236917
hsa_circ_	0.297425	0.236925
hsa_circ_	0.270001	0.236932
hsa_circ_	-0.33179	0.236941
hsa_circ_	-0.36263	0.236947
hsa_circ_	-0.16609	0.236948
hsa_circ_	0.171624	0.236948
hsa_circ_	0.17282	0.236949
hsa_circ_	0.203127	0.23695
hsa_circ_	0.228625	0.236952
hsa_circ_	-0.27886	0.236952
hsa_circ_	-0.19131	0.236961
hsa_circ_	0.39556	0.236968
hsa_circ_	-0.56916	0.236987
hsa_circ_	-0.17901	0.236998

hsa_circ_	-0.65805	0.237007
hsa_circ_	0.273657	0.237017
hsa_circ_	-0.18056	0.237021
hsa_circ_	0.184191	0.237029
hsa_circ_	0.454111	0.237034
hsa_circ_	0.687812	0.237063
hsa_circ_	-0.22816	0.237071
hsa_circ_	-0.19869	0.237076
hsa_circ_	-0.48271	0.237088
hsa_circ_	0.244673	0.237102
hsa_circ_	-0.23563	0.237125
hsa_circ_	-0.34405	0.237135
hsa_circ_	-0.87103	0.237147
hsa_circ_	0.467291	0.237154
hsa_circ_	-0.76991	0.237161
hsa_circ_	0.261928	0.237173
hsa_circ_	-0.2748	0.237203
hsa_circ_	-0.20352	0.237209
hsa_circ_	0.282888	0.237211
hsa_circ_	-0.25391	0.237214
hsa_circ_	-0.18608	0.237219
hsa_circ_	-0.19811	0.237233
hsa_circ_	-0.32372	0.237241
hsa_circ_	0.393298	0.237261
hsa_circ_	-0.59643	0.237262
hsa_circ_	-0.54046	0.237272
hsa_circ_	1.119061	0.237285
hsa_circ_	-0.30706	0.237295
hsa_circ_	-0.30177	0.237297
hsa_circ_	0.792963	0.237299
hsa_circ_	0.706818	0.237304
hsa_circ_	-0.33072	0.23732
hsa_circ_	-0.49944	0.237359
hsa_circ_	-0.18399	0.237364
hsa_circ_	1.027162	0.237383
hsa_circ_	-0.25574	0.237392
hsa_circ_	-0.22115	0.237403
hsa_circ_	-0.79549	0.237411
hsa_circ_	0.975351	0.237414
hsa_circ_	0.233956	0.237418
hsa_circ_	0.202022	0.237422
hsa_circ_	-0.1795	0.237425
hsa_circ_	-0.25156	0.237431
hsa_circ_	-0.25076	0.237435
hsa_circ_	0.272588	0.237439
hsa_circ_	-0.19743	0.237445
hsa_circ_	0.212431	0.237458
hsa_circ_	-0.25509	0.237458
hsa_circ_	0.193787	0.237466
hsa_circ_	-0.32669	0.237468
hsa_circ_	0.166755	0.237469
hsa_circ_	0.234229	0.23747
hsa_circ_	-0.17765	0.237476
hsa_circ_	0.221118	0.237491

hsa_circ_	0.534638	0.237504
hsa_circ_	0.205582	0.237511
hsa_circ_	-0.77385	0.237511
hsa_circ_	-0.39617	0.237521
hsa_circ_	0.235282	0.237522
hsa_circ_	-0.21978	0.237526
hsa_circ_	-0.53313	0.23753
hsa_circ_	0.690101	0.237531
hsa_circ_	0.179339	0.23755
hsa_circ_	-0.35474	0.237577
hsa_circ_	0.346063	0.237589
hsa_circ_	0.716755	0.237603
hsa_circ_	-0.30861	0.237604
hsa_circ_	0.325879	0.237607
hsa_circ_	-0.28013	0.237614
hsa_circ_	-0.3066	0.237638
hsa_circ_	0.184452	0.237645
hsa_circ_	-0.17204	0.237646
hsa_circ_	-0.32752	0.237651
hsa_circ_	0.861585	0.237659
hsa_circ_	0.295861	0.23766
hsa_circ_	0.186595	0.237669
hsa_circ_	0.31649	0.237673
hsa_circ_	-0.22547	0.237675
hsa_circ_	0.50168	0.237684
hsa_circ_	-0.40048	0.237696
hsa_circ_	-0.17995	0.237699
hsa_circ_	0.186872	0.237702
hsa_circ_	-0.34016	0.237707
hsa_circ_	-0.48624	0.237734
hsa_circ_	-0.18044	0.237749
hsa_circ_	-0.19475	0.237764
hsa_circ_	-0.19299	0.2378
hsa_circ_	0.374046	0.237844
hsa_circ_	-0.55011	0.237846
hsa_circ_	0.269296	0.23785
hsa_circ_	0.216272	0.237851
hsa_circ_	0.477738	0.237861
hsa_circ_	-0.60714	0.237863
hsa_circ_	0.160939	0.237868
hsa_circ_	0.369554	0.237874
hsa_circ_	0.281839	0.237875
hsa_circ_	-0.47949	0.237888
hsa_circ_	-0.36381	0.237888
hsa_circ_	0.561962	0.237922
hsa_circ_	-0.31385	0.237924
hsa_circ_	0.51042	0.237934
hsa_circ_	-0.16925	0.237942
hsa_circ_	-0.17231	0.237943
hsa_circ_	0.204032	0.237948
hsa_circ_	0.409284	0.237957
hsa_circ_	-0.24135	0.237968
hsa_circ_	0.318057	0.237971
hsa_circ_	-0.39757	0.237978

hsa_circ_	0.61789	0.237983
hsa_circ_	0.530509	0.238
hsa_circ_	0.234488	0.238001
hsa_circ_	-0.15949	0.238004
hsa_circ_	-0.16939	0.238006
hsa_circ_	0.446213	0.238011
hsa_circ_	-0.21769	0.238027
hsa_circ_	-0.96402	0.238035
hsa_circ_	-0.38333	0.238053
hsa_circ_	-0.28306	0.238106
hsa_circ_	-0.2223	0.238113
hsa_circ_	-1.44457	0.238113
hsa_circ_	0.577707	0.238119
hsa_circ_	0.450408	0.238121
hsa_circ_	0.308415	0.238135
hsa_circ_	-0.16612	0.238141
hsa_circ_	0.22763	0.238169
hsa_circ_	0.442372	0.238189
hsa_circ_	0.383148	0.238221
hsa_circ_	0.585142	0.238225
hsa_circ_	0.420934	0.238239
hsa_circ_	-0.21392	0.238249
hsa_circ_	0.186799	0.238267
hsa_circ_	-0.20019	0.238289
hsa_circ_	-0.18495	0.238293
hsa_circ_	-0.48822	0.23831
hsa_circ_	0.714893	0.238319
hsa_circ_	0.274553	0.238322
hsa_circ_	0.375204	0.238349
hsa_circ_	-0.32847	0.23835
hsa_circ_	-0.23376	0.238357
hsa_circ_	-0.20366	0.238358
hsa_circ_	-0.19936	0.238364
hsa_circ_	-0.22803	0.238366
hsa_circ_	-0.19964	0.238366
hsa_circ_	0.178312	0.23837
hsa_circ_	-0.21429	0.238382
hsa_circ_	0.293852	0.238413
hsa_circ_	0.272096	0.238431
hsa_circ_	0.292477	0.238441
hsa_circ_	-0.26344	0.238442
hsa_circ_	-0.21486	0.238461
hsa_circ_	-0.26597	0.238468
hsa_circ_	0.25882	0.238471
hsa_circ_	0.464396	0.238492
hsa_circ_	-0.20801	0.2385
hsa_circ_	0.229565	0.238501
hsa_circ_	0.28037	0.238515
hsa_circ_	0.19998	0.238559
hsa_circ_	-0.29473	0.238559
hsa_circ_	-0.26177	0.238571
hsa_circ_	0.338242	0.238583
hsa_circ_	0.333182	0.238615
hsa_circ_	0.655469	0.238648

hsa_circ_	0.232907	0.238654
hsa_circ_	-0.4264	0.238672
hsa_circ_	-0.19062	0.238673
hsa_circ_	-0.33513	0.238675
hsa_circ_	0.244571	0.238678
hsa_circ_	-0.80523	0.238692
hsa_circ_	-0.19089	0.238692
hsa_circ_	0.472506	0.238694
hsa_circ_	-0.20838	0.238714
hsa_circ_	-0.22639	0.238716
hsa_circ_	0.190369	0.238727
hsa_circ_	-0.20523	0.238735
hsa_circ_	-0.18604	0.238737
hsa_circ_	-0.7616	0.238742
hsa_circ_	-0.60678	0.238751
hsa_circ_	0.326233	0.238765
hsa_circ_	0.166149	0.238788
hsa_circ_	-0.20921	0.238788
hsa_circ_	-0.38792	0.238791
hsa_circ_	-0.326	0.238818
hsa_circ_	-0.39308	0.238819
hsa_circ_	0.19774	0.238821
hsa_circ_	-0.17147	0.238822
hsa_circ_	0.879834	0.238833
hsa_circ_	0.198257	0.238834
hsa_circ_	-0.18877	0.238848
hsa_circ_	-0.582	0.238859
hsa_circ_	-0.35503	0.238888
hsa_circ_	0.498273	0.238888
hsa_circ_	-0.22573	0.238895
hsa_circ_	0.236371	0.2389
hsa_circ_	-0.33893	0.238912
hsa_circ_	0.213732	0.238914
hsa_circ_	-0.21669	0.23892
hsa_circ_	0.445345	0.238944
hsa_circ_	0.176846	0.238964
hsa_circ_	0.507742	0.238972
hsa_circ_	0.27724	0.238977
hsa_circ_	0.171244	0.238981
hsa_circ_	-0.47832	0.238999
hsa_circ_	-0.17731	0.239021
hsa_circ_	-0.22335	0.239028
hsa_circ_	-0.39321	0.239032
hsa_circ_	0.490864	0.239043
hsa_circ_	0.354716	0.239044
hsa_circ_	0.286991	0.239049
hsa_circ_	-1.09947	0.239049
hsa_circ_	0.248304	0.239064
hsa_circ_	0.486512	0.239067
hsa_circ_	0.482597	0.239076
hsa_circ_	0.214657	0.239082
hsa_circ_	0.627242	0.239088
hsa_circ_	-0.17798	0.239097
hsa_circ_	0.316608	0.239098

hsa_circ_	-0.21206	0.239107
hsa_circ_	-0.1745	0.239108
hsa_circ_	-0.18159	0.239109
hsa_circ_	-0.20439	0.239117
hsa_circ_	-0.56163	0.239142
hsa_circ_	0.242503	0.239151
hsa_circ_	-0.6922	0.239151
hsa_circ_	-0.3927	0.239152
hsa_circ_	0.36863	0.239181
hsa_circ_	0.317988	0.239188
hsa_circ_	0.33359	0.239207
hsa_circ_	0.247129	0.239213
hsa_circ_	-0.25321	0.239219
hsa_circ_	-0.67617	0.239229
hsa_circ_	0.293382	0.239236
hsa_circ_	-0.62945	0.239254
hsa_circ_	0.305415	0.239256
hsa_circ_	0.207756	0.239263
hsa_circ_	0.167395	0.239265
hsa_circ_	0.309301	0.239272
hsa_circ_	-0.20807	0.239278
hsa_circ_	0.280219	0.239282
hsa_circ_	-0.24672	0.239285
hsa_circ_	-0.2585	0.239307
hsa_circ_	-0.25638	0.239308
hsa_circ_	0.43685	0.239319
hsa_circ_	0.207329	0.239321
hsa_circ_	-0.25954	0.239326
hsa_circ_	0.312795	0.239338
hsa_circ_	-0.27586	0.239398
hsa_circ_	-0.17091	0.239412
hsa_circ_	-0.15822	0.239426
hsa_circ_	0.230194	0.239445
hsa_circ_	-0.18675	0.239455
hsa_circ_	-0.23384	0.23947
hsa_circ_	0.58909	0.239473
hsa_circ_	-0.50827	0.2395
hsa_circ_	0.197302	0.2395
hsa_circ_	-0.16986	0.239501
hsa_circ_	-0.42737	0.239527
hsa_circ_	-0.39875	0.23953
hsa_circ_	0.24551	0.239537
hsa_circ_	-0.54383	0.239537
hsa_circ_	0.239727	0.239553
hsa_circ_	-0.28608	0.239554
hsa_circ_	0.231908	0.239576
hsa_circ_	0.581374	0.239583
hsa_circ_	-0.24907	0.239583
hsa_circ_	0.230497	0.239586
hsa_circ_	-0.23828	0.239593
hsa_circ_	0.675905	0.239603
hsa_circ_	0.308613	0.239619
hsa_circ_	0.220335	0.239624
hsa_circ_	0.384979	0.239631

hsa_circ_	0.182098	0.239636
hsa_circ_	-0.21084	0.23964
hsa_circ_	0.257712	0.239643
hsa_circ_	-0.24768	0.239654
hsa_circ_	-0.16414	0.239677
hsa_circ_	0.215571	0.239681
hsa_circ_	-0.46499	0.239685
hsa_circ_	0.236907	0.23969
hsa_circ_	0.363111	0.239732
hsa_circ_	0.658738	0.239758
hsa_circ_	-0.17957	0.239764
hsa_circ_	-0.74023	0.239765
hsa_circ_	-0.24635	0.239774
hsa_circ_	-0.66445	0.239779
hsa_circ_	-0.1751	0.23978
hsa_circ_	0.293318	0.239793
hsa_circ_	-0.54156	0.239805
hsa_circ_	-0.46993	0.239807
hsa_circ_	0.568677	0.23982
hsa_circ_	0.204746	0.239825
hsa_circ_	-0.66954	0.239837
hsa_circ_	0.313125	0.239838
hsa_circ_	-0.18794	0.239862
hsa_circ_	0.243959	0.239869
hsa_circ_	-0.19041	0.239881
hsa_circ_	0.291496	0.239902
hsa_circ_	0.612355	0.239918
hsa_circ_	-0.255	0.239921
hsa_circ_	-0.44302	0.239928
hsa_circ_	-0.43889	0.239935
hsa_circ_	-0.38474	0.239936
hsa_circ_	-0.19406	0.23994
hsa_circ_	-0.17924	0.239951
hsa_circ_	0.460387	0.239956
hsa_circ_	0.178247	0.239978
hsa_circ_	0.224074	0.23999
hsa_circ_	-0.38203	0.24
hsa_circ_	-0.16324	0.240054
hsa_circ_	-0.56487	0.240079
hsa_circ_	0.756655	0.240082
hsa_circ_	-0.47723	0.240087
hsa_circ_	-0.52404	0.240093
hsa_circ_	-0.20347	0.240095
hsa_circ_	0.351426	0.240106
hsa_circ_	-0.40959	0.240106
hsa_circ_	-0.3654	0.240115
hsa_circ_	-0.33497	0.24012
hsa_circ_	0.228211	0.24015
hsa_circ_	0.25644	0.240173
hsa_circ_	0.363397	0.240184
hsa_circ_	0.227916	0.240185
hsa_circ_	-0.87789	0.240187
hsa_circ_	0.422768	0.240187
hsa_circ_	0.26681	0.240193

hsa_circ_	0.63701	0.240196
hsa_circ_	-0.23808	0.240201
hsa_circ_	0.318612	0.240204
hsa_circ_	-0.24536	0.240207
hsa_circ_	0.374181	0.240213
hsa_circ_	-2.88648	0.240229
hsa_circ_	0.197347	0.240231
hsa_circ_	-0.3035	0.240235
hsa_circ_	-0.21799	0.24024
hsa_circ_	-0.37545	0.240257
hsa_circ_	0.251445	0.240284
hsa_circ_	-0.2022	0.240289
hsa_circ_	-0.26189	0.24033
hsa_circ_	0.346238	0.24034
hsa_circ_	0.198707	0.240341
hsa_circ_	0.328167	0.240344
hsa_circ_	0.872494	0.240346
hsa_circ_	0.196667	0.240388
hsa_circ_	0.207769	0.240396
hsa_circ_	-0.27066	0.240415
hsa_circ_	-0.2453	0.240429
hsa_circ_	-0.20637	0.240439
hsa_circ_	-0.1759	0.240444
hsa_circ_	0.196054	0.240445
hsa_circ_	-0.16225	0.240486
hsa_circ_	-0.23362	0.240492
hsa_circ_	-0.27358	0.240515
hsa_circ_	0.433587	0.240517
hsa_circ_	0.501959	0.240518
hsa_circ_	-0.26383	0.24053
hsa_circ_	0.223025	0.240532
hsa_circ_	-0.19078	0.240535
hsa_circ_	0.246796	0.240538
hsa_circ_	0.384538	0.240544
hsa_circ_	0.773804	0.240544
hsa_circ_	-0.26183	0.24055
hsa_circ_	0.329869	0.240565
hsa_circ_	-0.36117	0.240568
hsa_circ_	0.43144	0.240578
hsa_circ_	-0.19232	0.240579
hsa_circ_	0.435941	0.240585
hsa_circ_	-0.33733	0.240587
hsa_circ_	-0.25203	0.240592
hsa_circ_	-0.17065	0.240598
hsa_circ_	0.372205	0.240617
hsa_circ_	0.204908	0.240621
hsa_circ_	-0.361	0.240635
hsa_circ_	0.244498	0.240638
hsa_circ_	0.20886	0.240646
hsa_circ_	-0.45312	0.240651
hsa_circ_	0.191602	0.240651
hsa_circ_	-0.25048	0.240661
hsa_circ_	0.332597	0.240702
hsa_circ_	-0.34376	0.240709

hsa_circ_	0.164446	0.240728
hsa_circ_	-0.28349	0.240748
hsa_circ_	-0.42437	0.240753
hsa_circ_	0.584469	0.240755
hsa_circ_	0.270489	0.240762
hsa_circ_	-0.31608	0.240789
hsa_circ_	-0.22551	0.240789
hsa_circ_	-3.88352	0.24079
hsa_circ_	0.20794	0.240796
hsa_circ_	-0.17216	0.240797
hsa_circ_	0.197032	0.240822
hsa_circ_	0.238115	0.240835
hsa_circ_	-0.23353	0.240846
hsa_circ_	0.240956	0.240865
hsa_circ_	-1.5207	0.240866
hsa_circ_	0.363842	0.240892
hsa_circ_	-0.19476	0.24091
hsa_circ_	0.196786	0.240913
hsa_circ_	0.178858	0.24092
hsa_circ_	0.257303	0.240934
hsa_circ_	-0.52352	0.240951
hsa_circ_	-0.30129	0.240956
hsa_circ_	0.183434	0.240957
hsa_circ_	-0.17756	0.240963
hsa_circ_	0.365328	0.240966
hsa_circ_	-0.23972	0.240968
hsa_circ_	0.387392	0.240975
hsa_circ_	-0.17803	0.240985
hsa_circ_	-0.46553	0.240986
hsa_circ_	0.316828	0.241013
hsa_circ_	-0.27282	0.241028
hsa_circ_	-0.50689	0.241037
hsa_circ_	0.250837	0.241046
hsa_circ_	-0.20144	0.241055
hsa_circ_	0.186009	0.241079
hsa_circ_	0.281799	0.2411
hsa_circ_	-0.78503	0.241116
hsa_circ_	0.20362	0.241127
hsa_circ_	-0.18868	0.241138
hsa_circ_	-0.20427	0.241186
hsa_circ_	-0.22654	0.241186
hsa_circ_	0.201708	0.241188
hsa_circ_	-0.26785	0.241188
hsa_circ_	0.191759	0.24119
hsa_circ_	0.305894	0.241198
hsa_circ_	-0.44919	0.241198
hsa_circ_	0.185314	0.241199
hsa_circ_	-0.26478	0.241214
hsa_circ_	0.201847	0.241217
hsa_circ_	-0.22311	0.241222
hsa_circ_	-0.37837	0.241223
hsa_circ_	0.219499	0.241228
hsa_circ_	-0.52447	0.241228
hsa_circ_	-0.23842	0.241233

hsa_circ_	-0.61481	0.241241
hsa_circ_	-0.26498	0.241251
hsa_circ_	-0.65658	0.241254
hsa_circ_	-0.22712	0.241261
hsa_circ_	-0.38778	0.241269
hsa_circ_	-0.23116	0.24127
hsa_circ_	-0.17271	0.241277
hsa_circ_	0.268176	0.24129
hsa_circ_	-1.12351	0.241316
hsa_circ_	0.166683	0.24132
hsa_circ_	0.657651	0.241321
hsa_circ_	-0.21852	0.241322
hsa_circ_	-0.17719	0.24133
hsa_circ_	0.804584	0.241331
hsa_circ_	0.239501	0.241333
hsa_circ_	-0.16854	0.241357
hsa_circ_	-0.1724	0.241375
hsa_circ_	-0.44887	0.241382
hsa_circ_	-0.17276	0.241398
hsa_circ_	0.176292	0.241408
hsa_circ_	-0.19263	0.241419
hsa_circ_	-0.3773	0.241436
hsa_circ_	0.692176	0.241456
hsa_circ_	-0.45689	0.241458
hsa_circ_	-0.29116	0.241464
hsa_circ_	-0.34539	0.241475
hsa_circ_	-0.17218	0.241478
hsa_circ_	-0.17421	0.241509
hsa_circ_	0.315785	0.241514
hsa_circ_	0.206608	0.241517
hsa_circ_	-0.21707	0.241522
hsa_circ_	0.20476	0.241522
hsa_circ_	-0.16461	0.241527
hsa_circ_	0.608362	0.24153
hsa_circ_	-0.59874	0.241553
hsa_circ_	0.63595	0.241555
hsa_circ_	-0.19858	0.241562
hsa_circ_	0.295349	0.241563
hsa_circ_	-0.30209	0.241583
hsa_circ_	0.483348	0.241585
hsa_circ_	0.211305	0.241587
hsa_circ_	-0.24972	0.241599
hsa_circ_	0.220398	0.241605
hsa_circ_	-0.25634	0.241606
hsa_circ_	-0.4479	0.241612
hsa_circ_	0.254807	0.241636
hsa_circ_	-0.22795	0.241636
hsa_circ_	0.587109	0.241649
hsa_circ_	0.398946	0.241652
hsa_circ_	-0.32324	0.241663
hsa_circ_	-0.18807	0.241682
hsa_circ_	0.264708	0.241682
hsa_circ_	-0.8483	0.241694
hsa_circ_	-0.92309	0.241696

hsa_circ_	0.241708	0.241705
hsa_circ_	-0.32505	0.241717
hsa_circ_	0.194319	0.241738
hsa_circ_	0.637042	0.241746
hsa_circ_	-0.26888	0.241775
hsa_circ_	0.445592	0.241798
hsa_circ_	-0.29134	0.241816
hsa_circ_	-0.1783	0.241816
hsa_circ_	0.30044	0.241835
hsa_circ_	0.215276	0.241842
hsa_circ_	0.745767	0.241845
hsa_circ_	0.460407	0.241884
hsa_circ_	-0.22194	0.241891
hsa_circ_	-0.71113	0.241896
hsa_circ_	-0.24454	0.241911
hsa_circ_	0.414234	0.241951
hsa_circ_	0.228526	0.241963
hsa_circ_	0.168247	0.241966
hsa_circ_	-0.24276	0.241972
hsa_circ_	-0.20259	0.24199
hsa_circ_	-0.4466	0.241992
hsa_circ_	-0.2108	0.242001
hsa_circ_	-0.19821	0.242015
hsa_circ_	0.735299	0.242023
hsa_circ_	0.531736	0.242026
hsa_circ_	0.191939	0.24204
hsa_circ_	0.485381	0.242041
hsa_circ_	-0.17239	0.242045
hsa_circ_	0.476749	0.242047
hsa_circ_	-0.22542	0.24206
hsa_circ_	-0.29728	0.242081
hsa_circ_	0.31684	0.242122
hsa_circ_	0.408743	0.242129
hsa_circ_	0.954947	0.24214
hsa_circ_	-0.30156	0.242144
hsa_circ_	-0.32445	0.242144
hsa_circ_	0.238537	0.242167
hsa_circ_	0.276347	0.242173
hsa_circ_	-0.16034	0.242184
hsa_circ_	-0.30348	0.242224
hsa_circ_	0.346004	0.242224
hsa_circ_	-0.3797	0.242232
hsa_circ_	0.163866	0.242234
hsa_circ_	0.184269	0.242242
hsa_circ_	-0.17505	0.242252
hsa_circ_	0.253288	0.242255
hsa_circ_	-0.18682	0.242259
hsa_circ_	-0.24839	0.242262
hsa_circ_	0.314698	0.24229
hsa_circ_	0.255451	0.24232
hsa_circ_	0.586684	0.242335
hsa_circ_	0.265023	0.242348
hsa_circ_	-0.19195	0.24235
hsa_circ_	-0.25605	0.242356

hsa_circ_	0.223269	0.242364
hsa_circ_	-0.25328	0.242368
hsa_circ_	0.22723	0.242376
hsa_circ_	-0.5313	0.242377
hsa_circ_	0.212642	0.242389
hsa_circ_	0.781337	0.242397
hsa_circ_	0.22899	0.242408
hsa_circ_	-0.36888	0.242411
hsa_circ_	-0.19369	0.242447
hsa_circ_	-0.25896	0.242449
hsa_circ_	0.360291	0.242467
hsa_circ_	-0.19583	0.242469
hsa_circ_	-0.21146	0.24247
hsa_circ_	-0.35243	0.242473
hsa_circ_	0.22583	0.242477
hsa_circ_	-0.18483	0.24248
hsa_circ_	0.399309	0.242481
hsa_circ_	0.622177	0.242486
hsa_circ_	-0.3351	0.242489
hsa_circ_	0.183916	0.242494
hsa_circ_	0.192919	0.242512
hsa_circ_	0.170149	0.242518
hsa_circ_	0.415973	0.242521
hsa_circ_	-0.6067	0.24254
hsa_circ_	0.258191	0.242555
hsa_circ_	0.244404	0.242573
hsa_circ_	-0.64305	0.242584
hsa_circ_	0.321811	0.242588
hsa_circ_	-0.23252	0.242589
hsa_circ_	-0.16763	0.242598
hsa_circ_	-0.34822	0.242602
hsa_circ_	0.239132	0.24262
hsa_circ_	0.179676	0.242622
hsa_circ_	-0.24802	0.242622
hsa_circ_	-0.63511	0.242625
hsa_circ_	-0.19099	0.242648
hsa_circ_	0.354467	0.242654
hsa_circ_	0.242371	0.242657
hsa_circ_	0.198648	0.242666
hsa_circ_	-0.31149	0.242673
hsa_circ_	0.251007	0.242679
hsa_circ_	0.956308	0.24268
hsa_circ_	0.950036	0.242686
hsa_circ_	0.183076	0.242686
hsa_circ_	-0.77784	0.242717
hsa_circ_	0.387696	0.242726
hsa_circ_	0.617574	0.242742
hsa_circ_	-0.1566	0.242742
hsa_circ_	-0.38266	0.24275
hsa_circ_	0.210436	0.242759
hsa_circ_	-0.17628	0.242785
hsa_circ_	0.290765	0.242795
hsa_circ_	0.525977	0.242806
hsa_circ_	-0.61839	0.242812

hsa_circ_	-0.18292	0.242819
hsa_circ_	0.243605	0.242821
hsa_circ_	-0.92606	0.242828
hsa_circ_	-0.21411	0.242831
hsa_circ_	-0.1869	0.242877
hsa_circ_	0.653306	0.24288
hsa_circ_	0.264449	0.242882
hsa_circ_	0.322222	0.242896
hsa_circ_	0.327519	0.242905
hsa_circ_	-0.53434	0.242913
hsa_circ_	0.263411	0.242923
hsa_circ_	-0.1904	0.242946
hsa_circ_	-0.19534	0.242957
hsa_circ_	-0.19274	0.242969
hsa_circ_	-0.25682	0.242976
hsa_circ_	0.827437	0.242978
hsa_circ_	-0.26626	0.242979
hsa_circ_	-0.37875	0.242991
hsa_circ_	-0.65615	0.242993
hsa_circ_	0.557541	0.242999
hsa_circ_	0.191558	0.243006
hsa_circ_	-0.37948	0.243047
hsa_circ_	-0.55015	0.243052
hsa_circ_	-0.32602	0.243073
hsa_circ_	0.196584	0.243082
hsa_circ_	-0.29699	0.243086
hsa_circ_	-0.17218	0.243087
hsa_circ_	0.395509	0.24309
hsa_circ_	-0.29702	0.24313
hsa_circ_	-0.18687	0.243143
hsa_circ_	-0.34226	0.24315
hsa_circ_	-0.35371	0.243154
hsa_circ_	1.322367	0.243168
hsa_circ_	-0.18841	0.243178
hsa_circ_	0.335004	0.24318
hsa_circ_	-0.30864	0.243188
hsa_circ_	-0.24037	0.243205
hsa_circ_	0.176394	0.243223
hsa_circ_	0.173847	0.243225
hsa_circ_	-0.22049	0.243225
hsa_circ_	-0.62798	0.24323
hsa_circ_	0.43975	0.243231
hsa_circ_	-0.27367	0.243234
hsa_circ_	-0.55258	0.243236
hsa_circ_	-0.17078	0.243268
hsa_circ_	0.233056	0.243269
hsa_circ_	-0.6704	0.243269
hsa_circ_	-0.22295	0.243276
hsa_circ_	-0.1863	0.243278
hsa_circ_	-0.20802	0.243281
hsa_circ_	0.172081	0.243286
hsa_circ_	0.655385	0.243292
hsa_circ_	0.222794	0.243296
hsa_circ_	-0.20879	0.243328

hsa_circ_	-0.41384	0.243337
hsa_circ_	0.397503	0.243345
hsa_circ_	0.223698	0.243367
hsa_circ_	0.658924	0.243372
hsa_circ_	0.204573	0.243381
hsa_circ_	0.36898	0.243392
hsa_circ_	-0.29378	0.243401
hsa_circ_	-0.3289	0.243421
hsa_circ_	-0.23199	0.243431
hsa_circ_	0.224433	0.243457
hsa_circ_	0.156601	0.243465
hsa_circ_	-0.18355	0.243467
hsa_circ_	-0.53536	0.243482
hsa_circ_	0.592348	0.243484
hsa_circ_	-0.20684	0.243485
hsa_circ_	-0.32862	0.243487
hsa_circ_	0.606489	0.24351
hsa_circ_	-0.19798	0.24351
hsa_circ_	-0.30234	0.243515
hsa_circ_	-0.32463	0.243521
hsa_circ_	0.257786	0.243539
hsa_circ_	-0.19614	0.24354
hsa_circ_	0.496909	0.243545
hsa_circ_	0.177304	0.243583
hsa_circ_	0.528193	0.243607
hsa_circ_	0.293778	0.243626
hsa_circ_	-0.1661	0.243648
hsa_circ_	-0.72108	0.243682
hsa_circ_	-0.20291	0.243687
hsa_circ_	-0.35138	0.243693
hsa_circ_	0.249356	0.243707
hsa_circ_	-0.93043	0.243714
hsa_circ_	-0.92387	0.243722
hsa_circ_	0.241937	0.243763
hsa_circ_	0.398751	0.243771
hsa_circ_	-0.79549	0.243776
hsa_circ_	-0.24821	0.243782
hsa_circ_	0.735703	0.243788
hsa_circ_	-0.22374	0.24379
hsa_circ_	0.215968	0.243801
hsa_circ_	0.512023	0.243814
hsa_circ_	-0.18325	0.243822
hsa_circ_	0.211395	0.243837
hsa_circ_	-0.2038	0.24385
hsa_circ_	0.595303	0.24387
hsa_circ_	0.328137	0.243882
hsa_circ_	0.220462	0.243887
hsa_circ_	0.229232	0.243894
hsa_circ_	-0.49685	0.243898
hsa_circ_	-0.21115	0.243925
hsa_circ_	-0.20591	0.243926
hsa_circ_	-0.25833	0.243935
hsa_circ_	0.288542	0.243941
hsa_circ_	-0.40045	0.243943

hsa_circ_	0.606553	0.243951
hsa_circ_	0.168724	0.243958
hsa_circ_	0.304222	0.24398
hsa_circ_	-0.61767	0.24398
hsa_circ_	0.812569	0.243981
hsa_circ_	0.238182	0.244016
hsa_circ_	0.235898	0.244019
hsa_circ_	0.40408	0.244029
hsa_circ_	-0.38382	0.244032
hsa_circ_	0.640132	0.244047
hsa_circ_	-0.28021	0.244067
hsa_circ_	-0.32671	0.244071
hsa_circ_	-0.27404	0.244114
hsa_circ_	0.233362	0.244121
hsa_circ_	-0.22018	0.244131
hsa_circ_	0.277287	0.244145
hsa_circ_	0.241057	0.244162
hsa_circ_	-0.2246	0.244174
hsa_circ_	-0.22947	0.244196
hsa_circ_	-0.17375	0.244207
hsa_circ_	0.229473	0.244235
hsa_circ_	-0.21698	0.244239
hsa_circ_	0.37563	0.244242
hsa_circ_	0.237452	0.244266
hsa_circ_	0.245322	0.244281
hsa_circ_	0.752947	0.244288
hsa_circ_	0.445649	0.244294
hsa_circ_	0.252629	0.244302
hsa_circ_	-1.31199	0.244333
hsa_circ_	0.354007	0.244355
hsa_circ_	-0.18795	0.244368
hsa_circ_	0.200525	0.244379
hsa_circ_	-0.30029	0.244382
hsa_circ_	-0.59368	0.244389
hsa_circ_	0.646767	0.244403
hsa_circ_	-0.28744	0.244404
hsa_circ_	0.305755	0.244411
hsa_circ_	0.73179	0.244412
hsa_circ_	0.391827	0.244424
hsa_circ_	-0.17312	0.244467
hsa_circ_	0.284602	0.244473
hsa_circ_	0.17806	0.244494
hsa_circ_	0.284907	0.244532
hsa_circ_	-0.55483	0.244537
hsa_circ_	-0.19907	0.244539
hsa_circ_	-0.19576	0.244555
hsa_circ_	-0.29227	0.244556
hsa_circ_	-0.55002	0.244561
hsa_circ_	-0.27407	0.244577
hsa_circ_	0.205495	0.244581
hsa_circ_	-0.26389	0.244586
hsa_circ_	-0.24744	0.244592
hsa_circ_	-0.3488	0.244593
hsa_circ_	0.276047	0.244596

hsa_circ_	0.341146	0.244597
hsa_circ_	0.265181	0.2446
hsa_circ_	0.389455	0.244606
hsa_circ_	-0.20109	0.244612
hsa_circ_	-1.81547	0.244613
hsa_circ_	0.277063	0.244633
hsa_circ_	-0.16444	0.244634
hsa_circ_	1.106216	0.244642
hsa_circ_	-0.20437	0.244657
hsa_circ_	0.20732	0.24468
hsa_circ_	0.211632	0.244693
hsa_circ_	-0.17845	0.244698
hsa_circ_	0.299835	0.244706
hsa_circ_	-0.15746	0.244715
hsa_circ_	-0.24101	0.24472
hsa_circ_	0.161072	0.244743
hsa_circ_	-0.28805	0.244761
hsa_circ_	0.179952	0.244781
hsa_circ_	0.400424	0.24479
hsa_circ_	0.327244	0.244794
hsa_circ_	-0.30752	0.2448
hsa_circ_	-0.22216	0.244808
hsa_circ_	0.256706	0.244819
hsa_circ_	-0.69347	0.244825
hsa_circ_	0.263258	0.244853
hsa_circ_	-0.18483	0.244862
hsa_circ_	-0.41493	0.244866
hsa_circ_	-0.17867	0.244913
hsa_circ_	0.326411	0.244917
hsa_circ_	-0.31608	0.244977
hsa_circ_	-0.18528	0.24501
hsa_circ_	0.232335	0.24502
hsa_circ_	0.311133	0.245026
hsa_circ_	-0.18267	0.245032
hsa_circ_	0.276004	0.245057
hsa_circ_	-0.21041	0.245103
hsa_circ_	-0.23063	0.245104
hsa_circ_	-0.16926	0.24513
hsa_circ_	-0.42201	0.245133
hsa_circ_	0.198517	0.245134
hsa_circ_	-0.41668	0.245151
hsa_circ_	-0.15688	0.245152
hsa_circ_	-0.23526	0.245154
hsa_circ_	-0.23119	0.245155
hsa_circ_	0.291067	0.245159
hsa_circ_	-0.91305	0.245178
hsa_circ_	0.264823	0.245196
hsa_circ_	-0.25073	0.245204
hsa_circ_	0.204918	0.245215
hsa_circ_	0.199173	0.245218
hsa_circ_	-0.33234	0.245223
hsa_circ_	-0.2592	0.245226
hsa_circ_	-0.29361	0.245226
hsa_circ_	-0.29653	0.245227

hsa_circ_	0.216569	0.245237
hsa_circ_	0.52104	0.245249
hsa_circ_	0.182555	0.245257
hsa_circ_	-0.51522	0.245266
hsa_circ_	0.63704	0.245267
hsa_circ_	0.185861	0.245271
hsa_circ_	0.223492	0.245284
hsa_circ_	0.191152	0.245287
hsa_circ_	-0.22225	0.245293
hsa_circ_	0.310986	0.245299
hsa_circ_	-0.34549	0.245299
hsa_circ_	0.632269	0.245309
hsa_circ_	-0.24609	0.245311
hsa_circ_	0.555233	0.245317
hsa_circ_	0.175929	0.245329
hsa_circ_	0.191538	0.245343
hsa_circ_	-0.34536	0.245355
hsa_circ_	-0.27324	0.245375
hsa_circ_	-0.16812	0.245377
hsa_circ_	-1.11383	0.245378
hsa_circ_	-0.70395	0.245384
hsa_circ_	-0.49517	0.24539
hsa_circ_	-0.25988	0.245397
hsa_circ_	0.165708	0.245398
hsa_circ_	-0.2575	0.245404
hsa_circ_	0.28415	0.245431
hsa_circ_	-0.19848	0.245457
hsa_circ_	-0.16924	0.245457
hsa_circ_	0.18233	0.245458
hsa_circ_	-0.54726	0.245458
hsa_circ_	0.711592	0.245461
hsa_circ_	0.195232	0.245478
hsa_circ_	-0.58224	0.245482
hsa_circ_	-0.35147	0.245482
hsa_circ_	-0.18702	0.245489
hsa_circ_	-0.36373	0.245514
hsa_circ_	-0.20728	0.24552
hsa_circ_	-0.46425	0.245524
hsa_circ_	-0.61322	0.245535
hsa_circ_	0.203268	0.245543
hsa_circ_	0.384766	0.245552
hsa_circ_	0.166071	0.245561
hsa_circ_	-0.59306	0.245598
hsa_circ_	-0.27335	0.245607
hsa_circ_	-0.31694	0.245611
hsa_circ_	-0.26991	0.245622
hsa_circ_	1.477398	0.245626
hsa_circ_	-0.20745	0.245627
hsa_circ_	-0.21465	0.245627
hsa_circ_	0.237503	0.245635
hsa_circ_	-0.15813	0.245638
hsa_circ_	-0.22583	0.245641
hsa_circ_	0.529539	0.245646
hsa_circ_	0.287019	0.245646

hsa_circ_	0.214412	0.245656
hsa_circ_	0.286098	0.245671
hsa_circ_	-0.37694	0.245676
hsa_circ_	0.213871	0.245676
hsa_circ_	0.343186	0.245689
hsa_circ_	0.231031	0.245694
hsa_circ_	0.329066	0.245694
hsa_circ_	-0.19518	0.245701
hsa_circ_	-0.16245	0.24571
hsa_circ_	-0.32422	0.245719
hsa_circ_	-0.28576	0.245724
hsa_circ_	-0.19275	0.245726
hsa_circ_	-0.20637	0.245749
hsa_circ_	0.404424	0.245755
hsa_circ_	-0.17022	0.245808
hsa_circ_	-0.54014	0.245811
hsa_circ_	0.406929	0.245813
hsa_circ_	0.257377	0.245824
hsa_circ_	-0.40541	0.245827
hsa_circ_	0.626164	0.245839
hsa_circ_	-0.23419	0.245866
hsa_circ_	0.238122	0.245879
hsa_circ_	-0.76517	0.245882
hsa_circ_	-0.23508	0.245892
hsa_circ_	0.671286	0.245893
hsa_circ_	-0.19215	0.245898
hsa_circ_	-0.45242	0.245902
hsa_circ_	-0.20147	0.245919
hsa_circ_	-0.18963	0.24592
hsa_circ_	-0.18151	0.245945
hsa_circ_	0.1922	0.245971
hsa_circ_	0.337147	0.245995
hsa_circ_	-0.31861	0.245996
hsa_circ_	-0.28091	0.245998
hsa_circ_	0.439965	0.24601
hsa_circ_	0.261495	0.246011
hsa_circ_	-0.47184	0.246014
hsa_circ_	0.215069	0.246023
hsa_circ_	0.196402	0.246029
hsa_circ_	0.209169	0.24606
hsa_circ_	-0.4986	0.246061
hsa_circ_	0.296407	0.2461
hsa_circ_	-0.25825	0.246122
hsa_circ_	-0.18215	0.246132
hsa_circ_	0.190652	0.246151
hsa_circ_	-0.32107	0.246167
hsa_circ_	0.225504	0.246175
hsa_circ_	0.592183	0.246201
hsa_circ_	-0.26059	0.246207
hsa_circ_	0.189917	0.24621
hsa_circ_	0.160235	0.246223
hsa_circ_	-0.20344	0.246232
hsa_circ_	-0.3528	0.246234
hsa_circ_	0.283599	0.246239

hsa_circ_	0.170024	0.246241
hsa_circ_	0.333672	0.246252
hsa_circ_	-0.82446	0.246253
hsa_circ_	0.409225	0.246262
hsa_circ_	-0.15382	0.246265
hsa_circ_	-0.18971	0.246265
hsa_circ_	0.167812	0.246267
hsa_circ_	0.658063	0.246271
hsa_circ_	-0.18551	0.246297
hsa_circ_	0.26815	0.246301
hsa_circ_	-0.6065	0.246324
hsa_circ_	-0.52326	0.246344
hsa_circ_	0.232414	0.246352
hsa_circ_	-0.15945	0.246352
hsa_circ_	0.235455	0.246354
hsa_circ_	0.209513	0.246356
hsa_circ_	0.224773	0.24636
hsa_circ_	-0.44517	0.246364
hsa_circ_	0.22593	0.246382
hsa_circ_	-0.23907	0.246383
hsa_circ_	-0.15696	0.246402
hsa_circ_	0.240755	0.246406
hsa_circ_	-0.37983	0.246407
hsa_circ_	1.167969	0.246413
hsa_circ_	0.284893	0.246422
hsa_circ_	0.384119	0.246438
hsa_circ_	0.301026	0.246442
hsa_circ_	0.173412	0.246451
hsa_circ_	0.27932	0.246453
hsa_circ_	0.209348	0.246457
hsa_circ_	0.208684	0.24647
hsa_circ_	-0.34165	0.24648
hsa_circ_	0.269302	0.24648
hsa_circ_	-0.25795	0.246507
hsa_circ_	0.533505	0.24651
hsa_circ_	0.285036	0.246517
hsa_circ_	-0.20399	0.246529
hsa_circ_	0.261111	0.24653
hsa_circ_	0.166303	0.246552
hsa_circ_	0.443722	0.246557
hsa_circ_	-0.27825	0.246569
hsa_circ_	-0.31335	0.246578
hsa_circ_	-0.35675	0.246589
hsa_circ_	-0.19243	0.246603
hsa_circ_	-0.20361	0.246625
hsa_circ_	-0.2497	0.24663
hsa_circ_	-0.16561	0.246636
hsa_circ_	0.378222	0.246665
hsa_circ_	0.841687	0.246692
hsa_circ_	0.195128	0.246701
hsa_circ_	-0.23652	0.246719
hsa_circ_	0.297451	0.246736
hsa_circ_	0.613889	0.246774
hsa_circ_	0.181883	0.246774

hsa_circ_	-0.44551	0.246775
hsa_circ_	-0.69317	0.246779
hsa_circ_	-0.23231	0.246809
hsa_circ_	0.257739	0.24682
hsa_circ_	-0.18193	0.246829
hsa_circ_	-0.38848	0.246863
hsa_circ_	0.221618	0.246864
hsa_circ_	-0.28259	0.246893
hsa_circ_	-0.46487	0.2469
hsa_circ_	0.23052	0.246904
hsa_circ_	-0.23308	0.246941
hsa_circ_	-0.22406	0.246964
hsa_circ_	0.504832	0.24697
hsa_circ_	0.24872	0.246979
hsa_circ_	-0.22632	0.246998
hsa_circ_	0.473585	0.247002
hsa_circ_	-0.22606	0.247011
hsa_circ_	-0.25806	0.247019
hsa_circ_	-0.21631	0.247021
hsa_circ_	-0.1541	0.247024
hsa_circ_	0.639988	0.247044
hsa_circ_	-0.19213	0.247045
hsa_circ_	-0.71012	0.247053
hsa_circ_	-0.86633	0.24707
hsa_circ_	-0.25299	0.247084
hsa_circ_	0.239226	0.247101
hsa_circ_	0.24078	0.24711
hsa_circ_	-0.38647	0.247115
hsa_circ_	-0.15695	0.247121
hsa_circ_	-0.19537	0.247143
hsa_circ_	0.40045	0.247152
hsa_circ_	0.268537	0.247179
hsa_circ_	-0.33714	0.247179
hsa_circ_	-0.16167	0.247193
hsa_circ_	0.166747	0.247226
hsa_circ_	-0.15968	0.24723
hsa_circ_	0.245765	0.24723
hsa_circ_	-0.57369	0.247277
hsa_circ_	-0.33836	0.247295
hsa_circ_	0.16826	0.247296
hsa_circ_	0.334668	0.247297
hsa_circ_	-0.2255	0.24731
hsa_circ_	-0.25066	0.247312
hsa_circ_	0.489171	0.247314
hsa_circ_	0.256682	0.247321
hsa_circ_	0.378041	0.247324
hsa_circ_	-0.69938	0.247353
hsa_circ_	0.194357	0.247357
hsa_circ_	-0.53645	0.247357
hsa_circ_	-0.17106	0.247361
hsa_circ_	-0.28006	0.247377
hsa_circ_	-0.16075	0.247393
hsa_circ_	-0.74255	0.247398
hsa_circ_	0.191213	0.247399

hsa_circ_	0.569676	0.247419
hsa_circ_	-0.19609	0.24742
hsa_circ_	-0.7045	0.247424
hsa_circ_	-0.19396	0.247431
hsa_circ_	0.165923	0.247442
hsa_circ_	0.182459	0.247449
hsa_circ_	0.622075	0.247458
hsa_circ_	0.966527	0.247463
hsa_circ_	-0.37504	0.247474
hsa_circ_	-0.44321	0.247492
hsa_circ_	-0.17045	0.247496
hsa_circ_	0.267708	0.247522
hsa_circ_	0.35476	0.247525
hsa_circ_	-0.16668	0.247567
hsa_circ_	-0.15213	0.247582
hsa_circ_	-0.57186	0.247646
hsa_circ_	-0.37764	0.247667
hsa_circ_	-0.32946	0.247684
hsa_circ_	0.34497	0.247687
hsa_circ_	0.199546	0.247704
hsa_circ_	0.230071	0.247738
hsa_circ_	-0.80902	0.247785
hsa_circ_	-0.23079	0.247788
hsa_circ_	0.356839	0.247795
hsa_circ_	0.236253	0.247796
hsa_circ_	-0.18793	0.247817
hsa_circ_	-0.23886	0.24782
hsa_circ_	0.178316	0.247842
hsa_circ_	-0.35033	0.247851
hsa_circ_	-0.39373	0.247874
hsa_circ_	-0.25136	0.24788
hsa_circ_	0.396899	0.247883
hsa_circ_	0.229388	0.247911
hsa_circ_	-0.2399	0.247932
hsa_circ_	-0.37312	0.247945
hsa_circ_	-0.33903	0.24796
hsa_circ_	-0.19763	0.247961
hsa_circ_	0.168456	0.247961
hsa_circ_	0.194935	0.247962
hsa_circ_	-0.48545	0.247962
hsa_circ_	0.346204	0.247966
hsa_circ_	-0.287	0.247972
hsa_circ_	-0.2096	0.24798
hsa_circ_	-0.47566	0.247982
hsa_circ_	-0.66437	0.247997
hsa_circ_	-0.2175	0.248003
hsa_circ_	0.326538	0.248022
hsa_circ_	0.347984	0.248044
hsa_circ_	0.258238	0.248049
hsa_circ_	-0.48116	0.248085
hsa_circ_	0.196043	0.248089
hsa_circ_	-0.72417	0.248114
hsa_circ_	-0.30769	0.248115
hsa_circ_	-0.26576	0.248129

hsa_circ_	-0.21475	0.248133
hsa_circ_	-1.03188	0.248134
hsa_circ_	0.315187	0.248166
hsa_circ_	-0.28188	0.248166
hsa_circ_	-0.22018	0.248175
hsa_circ_	-0.16211	0.248178
hsa_circ_	0.176688	0.248179
hsa_circ_	-0.27359	0.248183
hsa_circ_	0.200996	0.248224
hsa_circ_	-0.27661	0.248226
hsa_circ_	0.567388	0.24823
hsa_circ_	0.19015	0.248234
hsa_circ_	-0.22084	0.248243
hsa_circ_	0.259711	0.248248
hsa_circ_	0.487492	0.248255
hsa_circ_	-0.30528	0.248258
hsa_circ_	-0.17878	0.248262
hsa_circ_	-0.24346	0.248263
hsa_circ_	0.293425	0.248273
hsa_circ_	0.203109	0.248294
hsa_circ_	-0.46261	0.248295
hsa_circ_	0.604116	0.248301
hsa_circ_	-0.23031	0.248307
hsa_circ_	-0.23809	0.248309
hsa_circ_	0.18916	0.248312
hsa_circ_	-0.21956	0.24832
hsa_circ_	0.587933	0.248332
hsa_circ_	-0.81286	0.248343
hsa_circ_	0.175353	0.248354
hsa_circ_	-0.32778	0.248356
hsa_circ_	-0.17701	0.248358
hsa_circ_	-0.40662	0.248378
hsa_circ_	-0.51437	0.248405
hsa_circ_	0.420528	0.248418
hsa_circ_	0.245817	0.248457
hsa_circ_	-0.3382	0.24846
hsa_circ_	-0.44334	0.248462
hsa_circ_	0.441503	0.248462
hsa_circ_	-0.17481	0.248473
hsa_circ_	-0.58687	0.248475
hsa_circ_	0.166175	0.24848
hsa_circ_	0.272989	0.248485
hsa_circ_	-0.21105	0.248486
hsa_circ_	0.15318	0.24849
hsa_circ_	0.210114	0.248508
hsa_circ_	-0.60004	0.248523
hsa_circ_	0.606828	0.248532
hsa_circ_	0.609533	0.248539
hsa_circ_	-0.27446	0.24855
hsa_circ_	0.286257	0.248562
hsa_circ_	-0.20228	0.248569
hsa_circ_	-0.27675	0.248573
hsa_circ_	-0.49951	0.248574
hsa_circ_	-0.37244	0.2486

hsa_circ_	0.342105	0.24861
hsa_circ_	-0.29393	0.248627
hsa_circ_	0.382547	0.248637
hsa_circ_	0.501462	0.248642
hsa_circ_	-0.47875	0.248653
hsa_circ_	0.277249	0.248669
hsa_circ_	-0.31348	0.248707
hsa_circ_	0.265747	0.24871
hsa_circ_	0.183245	0.248717
hsa_circ_	-0.38504	0.248726
hsa_circ_	0.186961	0.248744
hsa_circ_	0.619776	0.248752
hsa_circ_	-0.18455	0.248769
hsa_circ_	0.290953	0.248781
hsa_circ_	0.350442	0.248783
hsa_circ_	0.300135	0.248785
hsa_circ_	0.992769	0.248789
hsa_circ_	-0.24754	0.248797
hsa_circ_	-0.75803	0.2488
hsa_circ_	0.714718	0.248817
hsa_circ_	-0.2357	0.248828
hsa_circ_	-0.28698	0.248828
hsa_circ_	0.36492	0.248833
hsa_circ_	-0.21197	0.248853
hsa_circ_	-0.2012	0.248876
hsa_circ_	0.182254	0.248888
hsa_circ_	0.508757	0.248888
hsa_circ_	-0.33725	0.248899
hsa_circ_	-0.32302	0.248912
hsa_circ_	-0.30822	0.248917
hsa_circ_	-0.17856	0.248929
hsa_circ_	0.204358	0.248933
hsa_circ_	-0.15221	0.248941
hsa_circ_	0.222639	0.248946
hsa_circ_	-0.28141	0.248957
hsa_circ_	-0.1755	0.248968
hsa_circ_	-0.24192	0.248976
hsa_circ_	0.274561	0.248978
hsa_circ_	-0.17865	0.248991
hsa_circ_	-0.33779	0.248996
hsa_circ_	0.288483	0.249009
hsa_circ_	0.289788	0.249031
hsa_circ_	-0.26072	0.249057
hsa_circ_	-0.25703	0.249087
hsa_circ_	0.222876	0.249088
hsa_circ_	-0.21821	0.249092
hsa_circ_	-0.51868	0.249098
hsa_circ_	-0.27332	0.249105
hsa_circ_	0.292717	0.249109
hsa_circ_	0.308986	0.24911
hsa_circ_	-1.08295	0.249114
hsa_circ_	-0.26046	0.249129
hsa_circ_	0.237075	0.249139
hsa_circ_	-0.22331	0.249148

hsa_circ_	-0.21365	0.249162
hsa_circ_	0.241451	0.24917
hsa_circ_	-0.19043	0.249179
hsa_circ_	0.307789	0.24918
hsa_circ_	0.273978	0.249182
hsa_circ_	0.626889	0.249184
hsa_circ_	0.359057	0.249194
hsa_circ_	-0.21054	0.249197
hsa_circ_	0.255711	0.249198
hsa_circ_	0.227892	0.249199
hsa_circ_	-0.25755	0.249208
hsa_circ_	-0.19297	0.249211
hsa_circ_	-0.71439	0.249212
hsa_circ_	-0.34349	0.24923
hsa_circ_	-0.47842	0.249238
hsa_circ_	-0.31342	0.249254
hsa_circ_	-0.40812	0.249263
hsa_circ_	-0.27972	0.249265
hsa_circ_	-0.26502	0.249274
hsa_circ_	-0.46218	0.249287
hsa_circ_	-0.65183	0.249295
hsa_circ_	-0.16151	0.249315
hsa_circ_	-0.48184	0.249318
hsa_circ_	0.267596	0.249337
hsa_circ_	-0.45947	0.249338
hsa_circ_	0.182179	0.249344
hsa_circ_	0.237432	0.249348
hsa_circ_	-0.42829	0.249371
hsa_circ_	0.312019	0.249376
hsa_circ_	0.15745	0.249382
hsa_circ_	0.272729	0.249393
hsa_circ_	0.802303	0.249396
hsa_circ_	-0.28403	0.249399
hsa_circ_	-0.55297	0.249401
hsa_circ_	-0.30158	0.249402
hsa_circ_	-0.54123	0.249408
hsa_circ_	0.232726	0.249413
hsa_circ_	0.709691	0.249419
hsa_circ_	-1.21561	0.249434
hsa_circ_	0.219625	0.249439
hsa_circ_	-0.23999	0.24944
hsa_circ_	-0.37505	0.249445
hsa_circ_	-0.49851	0.249452
hsa_circ_	0.430022	0.249463
hsa_circ_	0.162512	0.249491
hsa_circ_	-0.19882	0.249503
hsa_circ_	-0.19544	0.249507
hsa_circ_	-0.21215	0.249508
hsa_circ_	-0.1736	0.249509
hsa_circ_	0.232525	0.249509
hsa_circ_	-0.16855	0.249511
hsa_circ_	0.16321	0.249512
hsa_circ_	0.19498	0.249538
hsa_circ_	0.79964	0.249583

hsa_circ_	0.245981	0.249585
hsa_circ_	-0.17635	0.249588
hsa_circ_	-0.17018	0.249595
hsa_circ_	0.361297	0.249608
hsa_circ_	-0.52876	0.249616
hsa_circ_	-0.23859	0.249642
hsa_circ_	-0.17229	0.249647
hsa_circ_	-0.26581	0.249652
hsa_circ_	-0.21078	0.249666
hsa_circ_	-0.87936	0.249698
hsa_circ_	0.347353	0.249711
hsa_circ_	-0.18464	0.249716
hsa_circ_	-0.3694	0.249717
hsa_circ_	-0.16892	0.24973
hsa_circ_	0.523924	0.249737
hsa_circ_	0.218229	0.249743
hsa_circ_	-0.57075	0.249746
hsa_circ_	-0.16689	0.24975
hsa_circ_	-0.23083	0.249754
hsa_circ_	-0.32334	0.249795
hsa_circ_	0.705767	0.249803
hsa_circ_	0.283755	0.24981
hsa_circ_	0.178007	0.249819
hsa_circ_	0.666536	0.249841
hsa_circ_	0.181416	0.249841
hsa_circ_	-0.45014	0.249844
hsa_circ_	0.181623	0.249847
hsa_circ_	0.349589	0.249848
hsa_circ_	0.337306	0.249867
hsa_circ_	0.214667	0.249908
hsa_circ_	-0.42721	0.249924
hsa_circ_	0.197882	0.249932
hsa_circ_	0.215572	0.24996
hsa_circ_	0.171726	0.249964
hsa_circ_	0.167211	0.249993
hsa_circ_	0.210886	0.249997
hsa_circ_	-0.19887	0.249999
hsa_circ_	-0.30236	0.250012
hsa_circ_	0.205962	0.250015
hsa_circ_	-0.16085	0.250019
hsa_circ_	0.184185	0.250028
hsa_circ_	0.309952	0.25003
hsa_circ_	0.356179	0.250037
hsa_circ_	-0.37273	0.25004
hsa_circ_	0.25947	0.250046
hsa_circ_	0.181099	0.250051
hsa_circ_	-0.21745	0.250056
hsa_circ_	0.225528	0.25006
hsa_circ_	0.200362	0.250066
hsa_circ_	-0.66123	0.250074
hsa_circ_	-0.87891	0.250082
hsa_circ_	0.200602	0.250111
hsa_circ_	0.176918	0.250122
hsa_circ_	-0.53126	0.250128

hsa_circ_	0.424708	0.250131
hsa_circ_	0.322408	0.250138
hsa_circ_	0.277791	0.250139
hsa_circ_	-0.15418	0.250143
hsa_circ_	-0.44531	0.250153
hsa_circ_	0.325635	0.250159
hsa_circ_	-0.26211	0.250162
hsa_circ_	0.594313	0.250163
hsa_circ_	0.237558	0.250166
hsa_circ_	-0.21101	0.250171
hsa_circ_	-0.2451	0.250195
hsa_circ_	-0.1829	0.250197
hsa_circ_	0.193995	0.2502
hsa_circ_	0.460949	0.250212
hsa_circ_	-0.15462	0.250232
hsa_circ_	0.218491	0.250239
hsa_circ_	-0.36873	0.250245
hsa_circ_	-0.19802	0.250259
hsa_circ_	0.186985	0.250265
hsa_circ_	-0.247	0.250283
hsa_circ_	-0.20922	0.250319
hsa_circ_	-0.67671	0.250331
hsa_circ_	-0.19215	0.250331
hsa_circ_	-0.20015	0.250338
hsa_circ_	0.336165	0.250347
hsa_circ_	0.28581	0.250351
hsa_circ_	-0.17971	0.25036
hsa_circ_	0.176719	0.25042
hsa_circ_	0.262975	0.250428
hsa_circ_	-0.18325	0.250431
hsa_circ_	0.212076	0.25044
hsa_circ_	-0.16691	0.250453
hsa_circ_	0.303854	0.250458
hsa_circ_	0.623547	0.250467
hsa_circ_	0.231777	0.25047
hsa_circ_	-0.1955	0.250477
hsa_circ_	-0.28206	0.250495
hsa_circ_	0.355242	0.250498
hsa_circ_	0.286124	0.250524
hsa_circ_	0.229192	0.250528
hsa_circ_	0.156926	0.250532
hsa_circ_	-0.17054	0.250534
hsa_circ_	0.268519	0.250562
hsa_circ_	-0.17052	0.25061
hsa_circ_	-0.1737	0.250623
hsa_circ_	-0.33519	0.250631
hsa_circ_	0.521054	0.250633
hsa_circ_	-0.24238	0.250635
hsa_circ_	0.490386	0.250653
hsa_circ_	-0.16461	0.25069
hsa_circ_	-0.46551	0.250699
hsa_circ_	0.23942	0.250704
hsa_circ_	0.206844	0.250713
hsa_circ_	-0.56675	0.250724

hsa_circ_	-0.20966	0.250731
hsa_circ_	-0.21646	0.25074
hsa_circ_	-0.23214	0.250749
hsa_circ_	-0.21888	0.250754
hsa_circ_	0.407702	0.250769
hsa_circ_	-0.25466	0.250777
hsa_circ_	0.292675	0.250782
hsa_circ_	-0.47976	0.250784
hsa_circ_	-0.2149	0.250823
hsa_circ_	0.270849	0.250846
hsa_circ_	-0.43989	0.250895
hsa_circ_	-0.21232	0.250902
hsa_circ_	-0.58592	0.250918
hsa_circ_	0.203347	0.25092
hsa_circ_	-0.40149	0.250935
hsa_circ_	0.257831	0.250941
hsa_circ_	-0.20678	0.250948
hsa_circ_	0.277558	0.250954
hsa_circ_	-1.08833	0.250968
hsa_circ_	0.351965	0.250981
hsa_circ_	0.500015	0.250981
hsa_circ_	0.288344	0.250985
hsa_circ_	0.668968	0.251012
hsa_circ_	-0.18433	0.251029
hsa_circ_	-0.20332	0.251031
hsa_circ_	0.156517	0.251031
hsa_circ_	-0.28387	0.251075
hsa_circ_	-0.52314	0.251078
hsa_circ_	-0.66586	0.251103
hsa_circ_	0.18541	0.251103
hsa_circ_	-0.17113	0.251107
hsa_circ_	0.229779	0.251117
hsa_circ_	0.500389	0.251149
hsa_circ_	0.272153	0.25115
hsa_circ_	-0.18929	0.25115
hsa_circ_	0.37693	0.251157
hsa_circ_	0.15301	0.251163
hsa_circ_	-0.54807	0.251172
hsa_circ_	-0.56508	0.251175
hsa_circ_	0.257522	0.25118
hsa_circ_	-0.23217	0.251182
hsa_circ_	-0.40621	0.251195
hsa_circ_	0.214776	0.251207
hsa_circ_	0.379022	0.251227
hsa_circ_	0.292045	0.251247
hsa_circ_	0.18725	0.251259
hsa_circ_	0.282748	0.251262
hsa_circ_	0.207972	0.251263
hsa_circ_	0.367345	0.251265
hsa_circ_	-0.42636	0.251278
hsa_circ_	0.278946	0.251292
hsa_circ_	0.457584	0.251293
hsa_circ_	-0.30753	0.251293
hsa_circ_	0.288701	0.251298

hsa_circ_	0.354931	0.251304
hsa_circ_	-0.23738	0.251305
hsa_circ_	-0.20251	0.251336
hsa_circ_	-0.17526	0.251338
hsa_circ_	-0.62863	0.251344
hsa_circ_	0.24581	0.251357
hsa_circ_	-0.22036	0.25137
hsa_circ_	-0.68071	0.251372
hsa_circ_	-0.27546	0.251373
hsa_circ_	0.184198	0.251373
hsa_circ_	-0.1834	0.251392
hsa_circ_	1.156059	0.251399
hsa_circ_	0.228635	0.251414
hsa_circ_	0.392756	0.251425
hsa_circ_	0.371198	0.251431
hsa_circ_	-0.1993	0.251435
hsa_circ_	0.177309	0.251437
hsa_circ_	-0.28377	0.251441
hsa_circ_	0.228	0.251444
hsa_circ_	-0.21657	0.251477
hsa_circ_	-0.52325	0.25148
hsa_circ_	-0.27734	0.251497
hsa_circ_	-0.31593	0.2515
hsa_circ_	-0.17274	0.251507
hsa_circ_	0.166598	0.251556
hsa_circ_	-0.69178	0.251565
hsa_circ_	0.245511	0.25157
hsa_circ_	0.374323	0.251572
hsa_circ_	-0.22608	0.251578
hsa_circ_	-0.34359	0.251606
hsa_circ_	-0.84302	0.251613
hsa_circ_	-0.3886	0.251622
hsa_circ_	0.479287	0.251639
hsa_circ_	-0.25935	0.251658
hsa_circ_	-0.53486	0.251664
hsa_circ_	-0.3365	0.251696
hsa_circ_	0.192782	0.251722
hsa_circ_	0.246883	0.251729
hsa_circ_	-0.56369	0.25175
hsa_circ_	-0.22347	0.251751
hsa_circ_	0.432753	0.251754
hsa_circ_	-0.1626	0.25176
hsa_circ_	0.374587	0.251775
hsa_circ_	0.199317	0.251787
hsa_circ_	0.68484	0.251817
hsa_circ_	-0.38824	0.251826
hsa_circ_	-0.232	0.251838
hsa_circ_	0.343356	0.251859
hsa_circ_	0.267896	0.251869
hsa_circ_	0.223451	0.25187
hsa_circ_	-0.29013	0.251888
hsa_circ_	0.257205	0.251888
hsa_circ_	0.291793	0.251891
hsa_circ_	-0.23975	0.25191

hsa_circ_	-0.27052	0.251915
hsa_circ_	-0.15489	0.251923
hsa_circ_	-0.36506	0.251929
hsa_circ_	0.218639	0.251931
hsa_circ_	0.338503	0.251934
hsa_circ_	-0.34339	0.251939
hsa_circ_	0.354512	0.25194
hsa_circ_	0.197941	0.251945
hsa_circ_	-0.22841	0.251981
hsa_circ_	0.189278	0.251984
hsa_circ_	-0.56275	0.251992
hsa_circ_	0.181339	0.251997
hsa_circ_	0.192656	0.252015
hsa_circ_	0.383875	0.252028
hsa_circ_	-0.18246	0.252034
hsa_circ_	0.170621	0.252043
hsa_circ_	0.179611	0.252049
hsa_circ_	0.329864	0.252049
hsa_circ_	-0.33425	0.252053
hsa_circ_	0.255647	0.252059
hsa_circ_	-0.18482	0.252061
hsa_circ_	-0.25073	0.252063
hsa_circ_	-0.16646	0.252074
hsa_circ_	-0.1988	0.252091
hsa_circ_	-0.15826	0.252096
hsa_circ_	2.370211	0.252108
hsa_circ_	0.242715	0.252118
hsa_circ_	-0.27277	0.252119
hsa_circ_	-0.16756	0.252149
hsa_circ_	-0.22437	0.252196
hsa_circ_	-0.28902	0.252202
hsa_circ_	0.226054	0.252229
hsa_circ_	-0.78824	0.252233
hsa_circ_	-0.70878	0.252235
hsa_circ_	0.237949	0.252255
hsa_circ_	0.518296	0.252261
hsa_circ_	0.414441	0.252264
hsa_circ_	0.275527	0.252267
hsa_circ_	0.249001	0.25228
hsa_circ_	-0.25626	0.25229
hsa_circ_	0.642538	0.252299
hsa_circ_	0.299989	0.25231
hsa_circ_	0.280846	0.252323
hsa_circ_	0.602521	0.252329
hsa_circ_	0.189728	0.252333
hsa_circ_	0.203642	0.252356
hsa_circ_	-0.52224	0.252359
hsa_circ_	0.246595	0.25236
hsa_circ_	0.224083	0.25237
hsa_circ_	0.232427	0.252376
hsa_circ_	-0.31697	0.252379
hsa_circ_	0.152333	0.252402
hsa_circ_	0.63869	0.252413
hsa_circ_	0.239085	0.252438

hsa_circ_	-0.90628	0.252465
hsa_circ_	0.354655	0.252467
hsa_circ_	0.54737	0.252474
hsa_circ_	0.275894	0.252475
hsa_circ_	0.244924	0.252486
hsa_circ_	0.277309	0.252491
hsa_circ_	1.40664	0.252492
hsa_circ_	-0.37159	0.252508
hsa_circ_	0.268259	0.252541
hsa_circ_	-0.20646	0.252544
hsa_circ_	0.192688	0.252545
hsa_circ_	-0.21122	0.252547
hsa_circ_	-0.28253	0.252547
hsa_circ_	-0.29118	0.252548
hsa_circ_	-0.2264	0.252577
hsa_circ_	0.200609	0.252585
hsa_circ_	0.663911	0.252599
hsa_circ_	0.267857	0.252604
hsa_circ_	-0.50695	0.252612
hsa_circ_	0.296164	0.252614
hsa_circ_	-0.6687	0.252614
hsa_circ_	-0.786	0.252615
hsa_circ_	-0.16958	0.252619
hsa_circ_	-0.26966	0.252628
hsa_circ_	-0.28023	0.25264
hsa_circ_	0.284593	0.252646
hsa_circ_	-0.24557	0.252678
hsa_circ_	-0.42235	0.252681
hsa_circ_	0.182448	0.252691
hsa_circ_	-0.5807	0.252694
hsa_circ_	-0.307	0.252699
hsa_circ_	0.250968	0.252713
hsa_circ_	-0.17104	0.252739
hsa_circ_	-0.17426	0.25276
hsa_circ_	0.262275	0.252764
hsa_circ_	0.618984	0.252767
hsa_circ_	-0.17456	0.252779
hsa_circ_	0.256224	0.252812
hsa_circ_	-0.45997	0.252818
hsa_circ_	0.260641	0.252825
hsa_circ_	0.170787	0.252826
hsa_circ_	0.581402	0.252848
hsa_circ_	-0.6017	0.252854
hsa_circ_	0.190285	0.252857
hsa_circ_	-0.18355	0.252859
hsa_circ_	-0.40861	0.252861
hsa_circ_	-0.2136	0.252896
hsa_circ_	-0.18483	0.252912
hsa_circ_	0.299319	0.252913
hsa_circ_	0.180508	0.252918
hsa_circ_	-0.1518	0.25292
hsa_circ_	0.307801	0.252925
hsa_circ_	0.168166	0.252944
hsa_circ_	0.694158	0.252953

hsa_circ_	0.663814	0.252976
hsa_circ_	0.201011	0.252981
hsa_circ_	-0.15317	0.253006
hsa_circ_	-0.48391	0.25301
hsa_circ_	-0.6327	0.253011
hsa_circ_	-0.18778	0.253025
hsa_circ_	-0.45258	0.253029
hsa_circ_	0.197278	0.25303
hsa_circ_	0.322446	0.253031
hsa_circ_	0.390336	0.253076
hsa_circ_	0.295917	0.253137
hsa_circ_	-0.30134	0.253138
hsa_circ_	0.246917	0.253185
hsa_circ_	0.401815	0.253196
hsa_circ_	-0.23277	0.253212
hsa_circ_	-0.2423	0.253225
hsa_circ_	-0.16949	0.253228
hsa_circ_	-0.15837	0.253234
hsa_circ_	-0.22345	0.253246
hsa_circ_	0.385671	0.253254
hsa_circ_	-0.78735	0.253258
hsa_circ_	-0.18706	0.253285
hsa_circ_	0.342748	0.253291
hsa_circ_	-0.20866	0.253305
hsa_circ_	-0.60239	0.253312
hsa_circ_	0.255892	0.253326
hsa_circ_	0.629878	0.25333
hsa_circ_	-0.547	0.253333
hsa_circ_	0.507493	0.253336
hsa_circ_	0.594483	0.253337
hsa_circ_	-0.38042	0.253362
hsa_circ_	0.559625	0.253367
hsa_circ_	-0.16035	0.253371
hsa_circ_	0.2251	0.253387
hsa_circ_	0.193943	0.253403
hsa_circ_	-0.20632	0.253404
hsa_circ_	0.162015	0.253404
hsa_circ_	-0.40093	0.253419
hsa_circ_	0.240942	0.253434
hsa_circ_	-0.20227	0.253445
hsa_circ_	0.635577	0.253465
hsa_circ_	0.234244	0.253477
hsa_circ_	0.478491	0.253485
hsa_circ_	0.235017	0.253493
hsa_circ_	-0.29821	0.253544
hsa_circ_	0.18074	0.253548
hsa_circ_	-0.41365	0.25355
hsa_circ_	0.228459	0.253555
hsa_circ_	-0.18266	0.253569
hsa_circ_	-0.28042	0.25359
hsa_circ_	-1.79889	0.253594
hsa_circ_	-0.57115	0.253596
hsa_circ_	-0.65848	0.253596
hsa_circ_	-0.22522	0.253605

hsa_circ_	0.315579	0.253614
hsa_circ_	0.197635	0.25363
hsa_circ_	0.166806	0.253633
hsa_circ_	0.234348	0.253639
hsa_circ_	-0.66699	0.253654
hsa_circ_	0.440313	0.253661
hsa_circ_	0.237245	0.253662
hsa_circ_	-0.58986	0.253666
hsa_circ_	-0.28326	0.253671
hsa_circ_	-0.1877	0.253691
hsa_circ_	0.245283	0.253701
hsa_circ_	0.248294	0.253706
hsa_circ_	-1.61265	0.253708
hsa_circ_	0.203004	0.253714
hsa_circ_	-0.33416	0.253716
hsa_circ_	0.293027	0.25372
hsa_circ_	-0.25474	0.253726
hsa_circ_	0.54921	0.253729
hsa_circ_	-0.21177	0.253742
hsa_circ_	0.185836	0.253749
hsa_circ_	-0.25994	0.253751
hsa_circ_	-0.28191	0.253755
hsa_circ_	-0.3636	0.253771
hsa_circ_	-0.19256	0.253785
hsa_circ_	-0.46372	0.253793
hsa_circ_	-0.70222	0.253796
hsa_circ_	-0.25695	0.253803
hsa_circ_	0.212038	0.253825
hsa_circ_	0.252321	0.253826
hsa_circ_	0.238949	0.253833
hsa_circ_	-0.16115	0.253847
hsa_circ_	-0.20519	0.253848
hsa_circ_	0.310011	0.253853
hsa_circ_	-0.36669	0.253864
hsa_circ_	0.190519	0.253866
hsa_circ_	-0.68724	0.253868
hsa_circ_	-0.63626	0.253877
hsa_circ_	0.197321	0.253905
hsa_circ_	0.334922	0.253917
hsa_circ_	-0.90588	0.253931
hsa_circ_	0.402541	0.253947
hsa_circ_	-0.4221	0.253952
hsa_circ_	0.336352	0.253962
hsa_circ_	0.46481	0.253964
hsa_circ_	0.179724	0.253964
hsa_circ_	-0.25391	0.253968
hsa_circ_	0.18338	0.253982
hsa_circ_	0.239862	0.253986
hsa_circ_	0.192637	0.253989
hsa_circ_	-0.3322	0.254025
hsa_circ_	-0.22649	0.254031
hsa_circ_	-0.18496	0.254031
hsa_circ_	0.611706	0.254035
hsa_circ_	0.389871	0.254057

hsa_circ_	0.191101	0.254066
hsa_circ_	0.217384	0.254068
hsa_circ_	0.517983	0.254074
hsa_circ_	0.717193	0.254087
hsa_circ_	-0.18071	0.254117
hsa_circ_	-0.18957	0.254125
hsa_circ_	0.324126	0.254146
hsa_circ_	0.194553	0.254148
hsa_circ_	-0.18473	0.254151
hsa_circ_	-0.15993	0.254157
hsa_circ_	-0.30334	0.254164
hsa_circ_	0.249481	0.254165
hsa_circ_	-0.18002	0.254177
hsa_circ_	-0.43573	0.254181
hsa_circ_	-0.27847	0.254181
hsa_circ_	-0.22849	0.254198
hsa_circ_	-0.24163	0.2542
hsa_circ_	-0.22759	0.254208
hsa_circ_	0.296843	0.254209
hsa_circ_	0.347491	0.254216
hsa_circ_	0.179114	0.25424
hsa_circ_	0.27919	0.254249
hsa_circ_	-0.54224	0.254263
hsa_circ_	0.25495	0.254287
hsa_circ_	-1.26151	0.254297
hsa_circ_	-0.15625	0.254311
hsa_circ_	0.272159	0.254317
hsa_circ_	-0.23731	0.25432
hsa_circ_	0.214488	0.254334
hsa_circ_	0.163186	0.254344
hsa_circ_	-0.80666	0.254347
hsa_circ_	0.181875	0.254349
hsa_circ_	0.215031	0.254352
hsa_circ_	-0.15814	0.254383
hsa_circ_	-0.2628	0.25439
hsa_circ_	0.204932	0.254417
hsa_circ_	0.204166	0.254418
hsa_circ_	-0.55332	0.254446
hsa_circ_	-0.24584	0.254455
hsa_circ_	0.196648	0.254456
hsa_circ_	0.174614	0.254461
hsa_circ_	-0.40837	0.254466
hsa_circ_	-0.29188	0.254479
hsa_circ_	0.51154	0.25448
hsa_circ_	0.305158	0.254488
hsa_circ_	0.157297	0.254545
hsa_circ_	-0.2295	0.254549
hsa_circ_	-0.1676	0.254554
hsa_circ_	0.201581	0.254556
hsa_circ_	-0.34341	0.254576
hsa_circ_	0.298985	0.254593
hsa_circ_	0.213448	0.254595
hsa_circ_	-0.16633	0.254604
hsa_circ_	0.324356	0.254611

hsa_circ_	-0.35095	0.254622
hsa_circ_	-0.22391	0.254623
hsa_circ_	-0.27038	0.254639
hsa_circ_	-0.30835	0.254643
hsa_circ_	-0.24694	0.254668
hsa_circ_	0.351274	0.254675
hsa_circ_	-0.79535	0.254678
hsa_circ_	-1.3611	0.254717
hsa_circ_	-0.24669	0.254739
hsa_circ_	-0.66998	0.254746
hsa_circ_	0.242922	0.254752
hsa_circ_	-0.49176	0.254752
hsa_circ_	0.169402	0.254776
hsa_circ_	-0.26452	0.254781
hsa_circ_	-0.23578	0.254785
hsa_circ_	0.208688	0.254787
hsa_circ_	0.196115	0.254787
hsa_circ_	-0.26481	0.254792
hsa_circ_	-0.365	0.254812
hsa_circ_	-0.19141	0.254816
hsa_circ_	0.763422	0.254827
hsa_circ_	-0.45021	0.254827
hsa_circ_	0.597115	0.254847
hsa_circ_	-0.28698	0.254851
hsa_circ_	-0.3214	0.254873
hsa_circ_	-0.27816	0.2549
hsa_circ_	-0.15725	0.254909
hsa_circ_	-0.73978	0.25493
hsa_circ_	0.308188	0.254936
hsa_circ_	0.179741	0.254947
hsa_circ_	-0.18858	0.254981
hsa_circ_	0.429092	0.254983
hsa_circ_	-0.37138	0.254989
hsa_circ_	-0.2433	0.254994
hsa_circ_	-0.18096	0.255042
hsa_circ_	-0.33107	0.255051
hsa_circ_	0.316756	0.255086
hsa_circ_	-0.69946	0.2551
hsa_circ_	-0.37072	0.255101
hsa_circ_	0.340404	0.255127
hsa_circ_	0.211708	0.255133
hsa_circ_	0.279956	0.255135
hsa_circ_	0.588745	0.255154
hsa_circ_	-0.64904	0.255154
hsa_circ_	-0.1718	0.25516
hsa_circ_	0.345544	0.255164
hsa_circ_	-0.23545	0.255174
hsa_circ_	-0.4645	0.255203
hsa_circ_	-0.20113	0.255206
hsa_circ_	0.200204	0.25521
hsa_circ_	0.259996	0.255215
hsa_circ_	-0.28722	0.255216
hsa_circ_	0.249244	0.255235
hsa_circ_	0.388513	0.255246

hsa_circ_	-0.19602	0.255254
hsa_circ_	0.396801	0.255264
hsa_circ_	0.337452	0.255299
hsa_circ_	-0.23783	0.255319
hsa_circ_	-0.21479	0.255332
hsa_circ_	-0.36298	0.255334
hsa_circ_	0.335442	0.255342
hsa_circ_	-0.38334	0.255344
hsa_circ_	0.185929	0.255355
hsa_circ_	0.845001	0.255383
hsa_circ_	0.492573	0.255387
hsa_circ_	0.270219	0.255391
hsa_circ_	0.207026	0.2554
hsa_circ_	-0.19966	0.255401
hsa_circ_	-0.24989	0.255404
hsa_circ_	-0.25845	0.25541
hsa_circ_	-0.20164	0.255411
hsa_circ_	-0.62003	0.255414
hsa_circ_	-0.21638	0.25542
hsa_circ_	-0.21764	0.255432
hsa_circ_	0.353728	0.255448
hsa_circ_	-0.25501	0.25547
hsa_circ_	-0.34788	0.255483
hsa_circ_	0.389909	0.255483
hsa_circ_	0.178709	0.255502
hsa_circ_	-0.15638	0.25552
hsa_circ_	-0.16992	0.255558
hsa_circ_	-0.1997	0.255558
hsa_circ_	0.531502	0.25557
hsa_circ_	0.197142	0.255576
hsa_circ_	-0.2156	0.255592
hsa_circ_	0.213534	0.255598
hsa_circ_	-0.6113	0.255601
hsa_circ_	-0.28878	0.25562
hsa_circ_	-0.15886	0.255647
hsa_circ_	0.478768	0.255651
hsa_circ_	-0.28272	0.255655
hsa_circ_	0.63234	0.255659
hsa_circ_	-0.83514	0.255663
hsa_circ_	-0.14998	0.255668
hsa_circ_	0.166029	0.255668
hsa_circ_	0.284755	0.255679
hsa_circ_	-0.64048	0.255686
hsa_circ_	-0.29121	0.255695
hsa_circ_	0.305578	0.255701
hsa_circ_	0.199694	0.255712
hsa_circ_	-0.26448	0.255718
hsa_circ_	0.278704	0.25574
hsa_circ_	0.17735	0.255772
hsa_circ_	-0.2152	0.25579
hsa_circ_	0.278806	0.255806
hsa_circ_	0.357335	0.255806
hsa_circ_	-0.25249	0.255823
hsa_circ_	-0.34668	0.255825

hsa_circ_	-0.17713	0.255828
hsa_circ_	-0.39827	0.25585
hsa_circ_	-0.15727	0.255852
hsa_circ_	-0.24777	0.255868
hsa_circ_	-0.28187	0.255869
hsa_circ_	-0.32665	0.255872
hsa_circ_	0.190822	0.255902
hsa_circ_	-0.46451	0.255925
hsa_circ_	0.38312	0.255935
hsa_circ_	0.423591	0.255951
hsa_circ_	0.707251	0.255954
hsa_circ_	-0.26983	0.255974
hsa_circ_	0.207292	0.255992
hsa_circ_	-0.28821	0.255997
hsa_circ_	-0.17421	0.255998
hsa_circ_	-0.62673	0.256026
hsa_circ_	0.204422	0.256039
hsa_circ_	-0.29285	0.256042
hsa_circ_	0.276176	0.256056
hsa_circ_	0.215229	0.256082
hsa_circ_	-0.8951	0.256093
hsa_circ_	-0.27425	0.256095
hsa_circ_	-0.2809	0.256095
hsa_circ_	0.256386	0.256097
hsa_circ_	-0.37038	0.2561
hsa_circ_	0.223263	0.256101
hsa_circ_	-0.1986	0.256108
hsa_circ_	0.463174	0.256112
hsa_circ_	-0.19911	0.256119
hsa_circ_	0.24618	0.256125
hsa_circ_	0.532198	0.256128
hsa_circ_	0.667252	0.256129
hsa_circ_	-0.24699	0.256145
hsa_circ_	0.251008	0.256149
hsa_circ_	0.208576	0.256167
hsa_circ_	0.25512	0.256172
hsa_circ_	0.252783	0.256179
hsa_circ_	0.240428	0.256181
hsa_circ_	-0.19572	0.256198
hsa_circ_	0.240997	0.256213
hsa_circ_	-0.47051	0.256227
hsa_circ_	0.329197	0.25623
hsa_circ_	-0.19053	0.256243
hsa_circ_	-0.22885	0.256244
hsa_circ_	-0.50151	0.256269
hsa_circ_	-0.1634	0.256279
hsa_circ_	0.184934	0.256283
hsa_circ_	-0.18138	0.256289
hsa_circ_	0.558055	0.256302
hsa_circ_	-0.17491	0.256316
hsa_circ_	0.823104	0.256325
hsa_circ_	0.211976	0.25634
hsa_circ_	0.198757	0.256348
hsa_circ_	0.214488	0.256355

hsa_circ_	-0.17897	0.25636
hsa_circ_	0.161819	0.256364
hsa_circ_	-0.34451	0.256376
hsa_circ_	-0.38506	0.25638
hsa_circ_	-0.34671	0.25638
hsa_circ_	0.257108	0.256382
hsa_circ_	-1.10964	0.256388
hsa_circ_	-0.18209	0.25639
hsa_circ_	-0.34773	0.256405
hsa_circ_	-0.18372	0.256405
hsa_circ_	0.424382	0.256407
hsa_circ_	0.158937	0.256418
hsa_circ_	-0.42301	0.256422
hsa_circ_	-0.21768	0.256439
hsa_circ_	-0.48216	0.25645
hsa_circ_	0.252413	0.25646
hsa_circ_	0.458032	0.256463
hsa_circ_	0.61911	0.256467
hsa_circ_	-0.17048	0.256471
hsa_circ_	-0.1751	0.256477
hsa_circ_	-0.4452	0.25648
hsa_circ_	-0.15182	0.256567
hsa_circ_	-0.33874	0.25657
hsa_circ_	-0.23377	0.256585
hsa_circ_	-0.44759	0.25659
hsa_circ_	-0.20703	0.256614
hsa_circ_	0.329624	0.256614
hsa_circ_	0.220841	0.256628
hsa_circ_	0.179933	0.256644
hsa_circ_	0.267366	0.25666
hsa_circ_	0.290067	0.25667
hsa_circ_	-0.21524	0.256687
hsa_circ_	-0.21057	0.256704
hsa_circ_	0.153896	0.256717
hsa_circ_	0.216298	0.256728
hsa_circ_	0.223771	0.256762
hsa_circ_	0.218382	0.256769
hsa_circ_	-0.21266	0.25677
hsa_circ_	0.179395	0.256784
hsa_circ_	0.219782	0.256791
hsa_circ_	0.19624	0.2568
hsa_circ_	-0.32411	0.256808
hsa_circ_	-0.19972	0.256815
hsa_circ_	0.158931	0.256826
hsa_circ_	-0.32689	0.256834
hsa_circ_	0.549992	0.256834
hsa_circ_	3.118789	0.256839
hsa_circ_	-0.68582	0.256842
hsa_circ_	-0.63174	0.256843
hsa_circ_	0.807445	0.256844
hsa_circ_	-0.17737	0.256844
hsa_circ_	-0.33945	0.256852
hsa_circ_	-0.22281	0.256852
hsa_circ_	0.449119	0.256858

hsa_circ_	-0.51636	0.256872
hsa_circ_	0.251866	0.256944
hsa_circ_	-0.19499	0.256999
hsa_circ_	-0.16728	0.257004
hsa_circ_	0.47671	0.257024
hsa_circ_	0.301152	0.257031
hsa_circ_	-0.1786	0.257034
hsa_circ_	0.187212	0.257068
hsa_circ_	0.210296	0.257076
hsa_circ_	0.34054	0.257084
hsa_circ_	0.202557	0.257093
hsa_circ_	0.532046	0.257123
hsa_circ_	-0.28044	0.257124
hsa_circ_	-0.28667	0.257129
hsa_circ_	-0.19325	0.25715
hsa_circ_	0.236288	0.257151
hsa_circ_	0.353374	0.257163
hsa_circ_	0.247155	0.257166
hsa_circ_	0.219944	0.257193
hsa_circ_	0.205848	0.257194
hsa_circ_	0.313523	0.257195
hsa_circ_	0.234641	0.257196
hsa_circ_	0.342362	0.257198
hsa_circ_	0.567011	0.257237
hsa_circ_	0.171996	0.257239
hsa_circ_	0.20504	0.257241
hsa_circ_	0.285119	0.257261
hsa_circ_	-0.17015	0.257269
hsa_circ_	0.355975	0.257287
hsa_circ_	-0.34702	0.25729
hsa_circ_	0.700038	0.257295
hsa_circ_	-0.17028	0.2573
hsa_circ_	0.517068	0.257311
hsa_circ_	0.195268	0.257313
hsa_circ_	0.20402	0.257315
hsa_circ_	-0.22102	0.257322
hsa_circ_	0.237566	0.257327
hsa_circ_	-0.17337	0.257337
hsa_circ_	-0.37645	0.257341
hsa_circ_	-0.32543	0.257348
hsa_circ_	-0.32545	0.257359
hsa_circ_	-0.25707	0.257366
hsa_circ_	0.878444	0.257367
hsa_circ_	0.527944	0.257399
hsa_circ_	0.561961	0.257412
hsa_circ_	-0.21482	0.25743
hsa_circ_	-0.93442	0.257436
hsa_circ_	-0.1523	0.257446
hsa_circ_	0.233112	0.257447
hsa_circ_	-0.39564	0.257453
hsa_circ_	0.29777	0.257456
hsa_circ_	-0.27671	0.257466
hsa_circ_	0.414118	0.257478
hsa_circ_	-0.51629	0.257484

hsa_circ_	-0.21693	0.257484
hsa_circ_	-0.505	0.257487
hsa_circ_	0.213006	0.257505
hsa_circ_	-0.2996	0.257519
hsa_circ_	-0.28144	0.257524
hsa_circ_	-0.18193	0.257537
hsa_circ_	-0.55889	0.257546
hsa_circ_	-0.29252	0.257547
hsa_circ_	-0.21892	0.257557
hsa_circ_	-0.25139	0.257558
hsa_circ_	-0.48349	0.257566
hsa_circ_	-0.27789	0.257574
hsa_circ_	0.332353	0.257575
hsa_circ_	-0.75259	0.257579
hsa_circ_	0.185807	0.257612
hsa_circ_	-0.21149	0.257616
hsa_circ_	-0.16825	0.257621
hsa_circ_	-0.58768	0.25763
hsa_circ_	0.302051	0.257633
hsa_circ_	0.179763	0.257636
hsa_circ_	-0.24957	0.257645
hsa_circ_	-0.53029	0.257661
hsa_circ_	-0.22164	0.25767
hsa_circ_	-0.22094	0.257686
hsa_circ_	-0.23728	0.257718
hsa_circ_	-0.7037	0.257732
hsa_circ_	-0.1652	0.257736
hsa_circ_	-0.48718	0.257744
hsa_circ_	0.654217	0.257747
hsa_circ_	-0.19563	0.257751
hsa_circ_	0.243735	0.257771
hsa_circ_	0.456226	0.257801
hsa_circ_	0.223612	0.257815
hsa_circ_	-0.23993	0.257823
hsa_circ_	0.239427	0.257824
hsa_circ_	-0.52	0.257825
hsa_circ_	-0.38814	0.257834
hsa_circ_	0.219748	0.257838
hsa_circ_	-0.17946	0.257847
hsa_circ_	-0.17253	0.257875
hsa_circ_	0.206305	0.257938
hsa_circ_	-0.24145	0.25797
hsa_circ_	0.611771	0.25798
hsa_circ_	-0.28001	0.257982
hsa_circ_	0.162672	0.257985
hsa_circ_	0.327524	0.257985
hsa_circ_	0.596773	0.257987
hsa_circ_	-0.27067	0.257995
hsa_circ_	-0.2542	0.258013
hsa_circ_	-0.62008	0.258046
hsa_circ_	-0.23659	0.258054
hsa_circ_	0.178653	0.258063
hsa_circ_	0.177543	0.258083
hsa_circ_	0.15981	0.258108

hsa_circ_	-0.16865	0.258122
hsa_circ_	0.423763	0.25813
hsa_circ_	-0.24478	0.258134
hsa_circ_	-0.17454	0.258134
hsa_circ_	0.260033	0.258138
hsa_circ_	-0.50573	0.258148
hsa_circ_	0.403851	0.258176
hsa_circ_	-0.24302	0.258176
hsa_circ_	-0.22362	0.258186
hsa_circ_	0.239771	0.258191
hsa_circ_	-0.44481	0.258194
hsa_circ_	-0.16378	0.258206
hsa_circ_	-0.30076	0.258208
hsa_circ_	0.199499	0.258213
hsa_circ_	0.579968	0.258218
hsa_circ_	-0.28574	0.258222
hsa_circ_	-0.4434	0.258235
hsa_circ_	-0.19588	0.258239
hsa_circ_	0.198138	0.258264
hsa_circ_	-0.27547	0.258267
hsa_circ_	-0.34518	0.258287
hsa_circ_	0.230707	0.258291
hsa_circ_	-0.83408	0.258297
hsa_circ_	0.209601	0.258298
hsa_circ_	-0.18869	0.258306
hsa_circ_	-0.51576	0.258312
hsa_circ_	0.212864	0.258324
hsa_circ_	-0.16549	0.25835
hsa_circ_	0.318202	0.258351
hsa_circ_	-0.25838	0.258367
hsa_circ_	-0.19751	0.258377
hsa_circ_	-0.20632	0.258378
hsa_circ_	-0.17525	0.258404
hsa_circ_	0.247993	0.258412
hsa_circ_	0.220555	0.258443
hsa_circ_	0.201617	0.258466
hsa_circ_	0.223713	0.258469
hsa_circ_	-0.17326	0.258473
hsa_circ_	-0.20263	0.258474
hsa_circ_	-0.22015	0.25848
hsa_circ_	-0.18649	0.258481
hsa_circ_	0.226605	0.2585
hsa_circ_	-0.23924	0.258521
hsa_circ_	-0.14964	0.258522
hsa_circ_	-0.32168	0.258525
hsa_circ_	0.225981	0.258539
hsa_circ_	-0.18083	0.258545
hsa_circ_	-0.4967	0.25855
hsa_circ_	-0.19645	0.258552
hsa_circ_	0.253193	0.258581
hsa_circ_	-0.19418	0.258614
hsa_circ_	0.176573	0.258626
hsa_circ_	-0.18231	0.258637
hsa_circ_	0.169861	0.258637

hsa_circ_	-0.7672	0.258643
hsa_circ_	0.631532	0.258646
hsa_circ_	-0.20459	0.258659
hsa_circ_	0.581486	0.258663
hsa_circ_	-0.22396	0.258666
hsa_circ_	0.363336	0.258673
hsa_circ_	0.234644	0.258677
hsa_circ_	-0.235	0.258683
hsa_circ_	-0.24961	0.258686
hsa_circ_	0.358864	0.258687
hsa_circ_	0.234999	0.25869
hsa_circ_	-0.20393	0.258697
hsa_circ_	-0.15525	0.258713
hsa_circ_	-0.20176	0.258724
hsa_circ_	-0.23593	0.258737
hsa_circ_	-0.26622	0.258745
hsa_circ_	0.17138	0.25875
hsa_circ_	-0.23966	0.258755
hsa_circ_	-0.33258	0.25877
hsa_circ_	-0.18769	0.258789
hsa_circ_	-0.26216	0.258795
hsa_circ_	0.451331	0.258798
hsa_circ_	-0.23613	0.258811
hsa_circ_	0.329596	0.25882
hsa_circ_	0.181371	0.258822
hsa_circ_	-0.28909	0.258825
hsa_circ_	0.227648	0.258842
hsa_circ_	-0.17947	0.258851
hsa_circ_	0.238609	0.258853
hsa_circ_	0.34383	0.258871
hsa_circ_	0.243528	0.258873
hsa_circ_	0.710638	0.258875
hsa_circ_	-0.22117	0.258945
hsa_circ_	-0.16022	0.259009
hsa_circ_	-0.19157	0.259022
hsa_circ_	0.466059	0.259046
hsa_circ_	-0.17951	0.259052
hsa_circ_	0.171638	0.259056
hsa_circ_	-0.15091	0.259062
hsa_circ_	0.405239	0.259063
hsa_circ_	0.248677	0.25907
hsa_circ_	0.287107	0.259088
hsa_circ_	0.226955	0.259089
hsa_circ_	0.490591	0.259092
hsa_circ_	0.353968	0.259092
hsa_circ_	0.240091	0.259099
hsa_circ_	0.205217	0.259103
hsa_circ_	-0.21901	0.259116
hsa_circ_	-0.31707	0.259117
hsa_circ_	0.268211	0.25917
hsa_circ_	0.241512	0.259186
hsa_circ_	-0.21206	0.259191
hsa_circ_	0.403229	0.259193
hsa_circ_	-0.18162	0.259201

hsa_circ_	-0.22184	0.25921
hsa_circ_	-0.20011	0.259223
hsa_circ_	-0.14939	0.259225
hsa_circ_	0.203482	0.259226
hsa_circ_	0.191448	0.259242
hsa_circ_	-0.29089	0.259252
hsa_circ_	0.859142	0.259258
hsa_circ_	0.281923	0.259261
hsa_circ_	-0.5886	0.259294
hsa_circ_	-0.268	0.259315
hsa_circ_	-0.17109	0.259374
hsa_circ_	0.206414	0.259377
hsa_circ_	0.171909	0.259395
hsa_circ_	-0.15391	0.2594
hsa_circ_	0.370667	0.259409
hsa_circ_	0.321237	0.25941
hsa_circ_	-0.25308	0.259417
hsa_circ_	-0.1656	0.259422
hsa_circ_	0.203311	0.259436
hsa_circ_	-0.22026	0.259465
hsa_circ_	0.262995	0.259474
hsa_circ_	-0.24755	0.259478
hsa_circ_	-0.34861	0.2595
hsa_circ_	-0.20973	0.259535
hsa_circ_	-0.24021	0.259536
hsa_circ_	-0.26046	0.25956
hsa_circ_	0.453775	0.259561
hsa_circ_	0.404907	0.259564
hsa_circ_	0.309596	0.259567
hsa_circ_	-0.16569	0.259586
hsa_circ_	0.250993	0.259588
hsa_circ_	-0.2537	0.259595
hsa_circ_	0.247612	0.259598
hsa_circ_	-1.10247	0.259605
hsa_circ_	-0.17199	0.259615
hsa_circ_	-0.59158	0.259616
hsa_circ_	-0.34955	0.259629
hsa_circ_	-0.47907	0.259633
hsa_circ_	0.191966	0.259635
hsa_circ_	0.259014	0.259643
hsa_circ_	0.211525	0.259647
hsa_circ_	-0.18959	0.259671
hsa_circ_	-0.35328	0.259674
hsa_circ_	-0.32059	0.259675
hsa_circ_	0.202301	0.25969
hsa_circ_	-0.32269	0.259696
hsa_circ_	-0.47423	0.259703
hsa_circ_	-0.17798	0.259716
hsa_circ_	-0.2987	0.259743
hsa_circ_	0.705235	0.259747
hsa_circ_	0.463243	0.259758
hsa_circ_	-0.20683	0.25977
hsa_circ_	-0.22782	0.259777
hsa_circ_	-0.25738	0.259779

hsa_circ_	-0.18591	0.25978
hsa_circ_	-0.18303	0.259786
hsa_circ_	-0.15626	0.259791
hsa_circ_	-0.19868	0.259794
hsa_circ_	-0.192	0.259827
hsa_circ_	-0.60325	0.259853
hsa_circ_	-0.18409	0.259869
hsa_circ_	-0.22253	0.259875
hsa_circ_	0.17003	0.259875
hsa_circ_	0.652981	0.259883
hsa_circ_	0.178396	0.259894
hsa_circ_	0.201933	0.259894
hsa_circ_	0.34872	0.259899
hsa_circ_	-0.25066	0.259903
hsa_circ_	-0.37585	0.259906
hsa_circ_	0.227752	0.259909
hsa_circ_	-0.50163	0.259911
hsa_circ_	-0.19482	0.259937
hsa_circ_	-0.4937	0.259956
hsa_circ_	0.324153	0.259958
hsa_circ_	0.406829	0.25996
hsa_circ_	0.340249	0.259967
hsa_circ_	0.24396	0.259978
hsa_circ_	0.336966	0.259985
hsa_circ_	-0.53267	0.259998
hsa_circ_	-0.17658	0.260008
hsa_circ_	0.301625	0.260039
hsa_circ_	0.227527	0.260068
hsa_circ_	-0.19673	0.260083
hsa_circ_	-0.24852	0.260087
hsa_circ_	0.290529	0.260096
hsa_circ_	1.086609	0.260109
hsa_circ_	0.179245	0.260122
hsa_circ_	0.411691	0.260133
hsa_circ_	-0.22091	0.260137
hsa_circ_	0.313397	0.260138
hsa_circ_	0.186833	0.260141
hsa_circ_	-0.31842	0.260153
hsa_circ_	-0.27242	0.26017
hsa_circ_	0.354827	0.260177
hsa_circ_	0.177118	0.260192
hsa_circ_	0.458693	0.260209
hsa_circ_	-0.33819	0.260243
hsa_circ_	-0.62826	0.260245
hsa_circ_	-0.19317	0.260247
hsa_circ_	0.262173	0.260254
hsa_circ_	-0.35138	0.260275
hsa_circ_	0.32631	0.260278
hsa_circ_	0.173969	0.260284
hsa_circ_	0.216588	0.260285
hsa_circ_	0.254614	0.260294
hsa_circ_	0.401412	0.260312
hsa_circ_	0.286137	0.260324
hsa_circ_	0.288504	0.260326

hsa_circ_	-0.4289	0.26033
hsa_circ_	0.238813	0.260343
hsa_circ_	-0.28673	0.260346
hsa_circ_	-0.27021	0.26035
hsa_circ_	0.39	0.260354
hsa_circ_	0.48639	0.260361
hsa_circ_	-0.28658	0.260368
hsa_circ_	-0.21346	0.26037
hsa_circ_	0.697318	0.260374
hsa_circ_	-0.23848	0.260379
hsa_circ_	-0.30885	0.260403
hsa_circ_	-0.17035	0.260404
hsa_circ_	-0.19385	0.260416
hsa_circ_	1.003755	0.260433
hsa_circ_	0.239364	0.260438
hsa_circ_	0.238528	0.260444
hsa_circ_	0.18459	0.260445
hsa_circ_	-0.33493	0.260446
hsa_circ_	-0.19074	0.26045
hsa_circ_	-0.25777	0.26046
hsa_circ_	-0.37385	0.260471
hsa_circ_	0.407003	0.260505
hsa_circ_	0.183715	0.260511
hsa_circ_	0.473356	0.260531
hsa_circ_	0.33051	0.26055
hsa_circ_	0.195193	0.260565
hsa_circ_	-0.25509	0.260569
hsa_circ_	0.303585	0.260572
hsa_circ_	0.257893	0.260576
hsa_circ_	0.248688	0.260579
hsa_circ_	-0.16055	0.260616
hsa_circ_	0.262944	0.26062
hsa_circ_	0.15986	0.260626
hsa_circ_	0.150671	0.260636
hsa_circ_	-0.15592	0.26067
hsa_circ_	-0.19287	0.260675
hsa_circ_	-0.15331	0.260683
hsa_circ_	-0.48164	0.26069
hsa_circ_	0.163956	0.260702
hsa_circ_	-0.31391	0.260724
hsa_circ_	-1.40939	0.260733
hsa_circ_	0.241392	0.260741
hsa_circ_	-0.56929	0.260742
hsa_circ_	-0.27784	0.260742
hsa_circ_	0.178235	0.260743
hsa_circ_	0.170989	0.260744
hsa_circ_	-0.25922	0.260749
hsa_circ_	0.241597	0.260752
hsa_circ_	-0.2865	0.260763
hsa_circ_	0.159947	0.260771
hsa_circ_	-0.48255	0.260785
hsa_circ_	0.167923	0.260794
hsa_circ_	-0.35324	0.260819
hsa_circ_	-0.29195	0.260822

hsa_circ_	0.298357	0.260843
hsa_circ_	-0.42587	0.260843
hsa_circ_	-0.19731	0.260857
hsa_circ_	0.500893	0.260859
hsa_circ_	0.199198	0.260861
hsa_circ_	0.290676	0.260864
hsa_circ_	0.432206	0.260868
hsa_circ_	-0.17798	0.260871
hsa_circ_	-0.19775	0.260882
hsa_circ_	0.67579	0.2609
hsa_circ_	-0.67345	0.260905
hsa_circ_	-0.16146	0.260931
hsa_circ_	-0.17733	0.260933
hsa_circ_	-1.33366	0.260937
hsa_circ_	-0.51866	0.260941
hsa_circ_	-0.2248	0.260946
hsa_circ_	-0.70212	0.260949
hsa_circ_	0.331955	0.260953
hsa_circ_	-0.1724	0.260954
hsa_circ_	-0.30166	0.26096
hsa_circ_	-0.94327	0.260964
hsa_circ_	-0.27411	0.260966
hsa_circ_	0.630976	0.260979
hsa_circ_	-0.27046	0.26099
hsa_circ_	-0.36086	0.260994
hsa_circ_	-0.19077	0.261013
hsa_circ_	0.491212	0.261018
hsa_circ_	-0.21468	0.261018
hsa_circ_	0.239718	0.261051
hsa_circ_	-0.2483	0.261054
hsa_circ_	-0.24857	0.261072
hsa_circ_	0.161936	0.261083
hsa_circ_	0.374774	0.261089
hsa_circ_	-0.19995	0.26109
hsa_circ_	0.637026	0.261116
hsa_circ_	0.386869	0.261126
hsa_circ_	-0.18455	0.26115
hsa_circ_	-0.72224	0.261157
hsa_circ_	0.435697	0.261158
hsa_circ_	0.200923	0.26116
hsa_circ_	0.380726	0.26117
hsa_circ_	-0.22094	0.26118
hsa_circ_	-0.60254	0.261226
hsa_circ_	-0.16465	0.261239
hsa_circ_	-0.48608	0.261256
hsa_circ_	-0.40268	0.261292
hsa_circ_	-0.19716	0.261293
hsa_circ_	-0.27715	0.261301
hsa_circ_	0.16397	0.261305
hsa_circ_	0.605756	0.261312
hsa_circ_	0.366991	0.261338
hsa_circ_	0.178856	0.261339
hsa_circ_	-0.61608	0.261348
hsa_circ_	-0.25236	0.261376

hsa_circ_	-1.0156	0.261399
hsa_circ_	0.411048	0.261403
hsa_circ_	0.8594	0.261408
hsa_circ_	-0.15923	0.261409
hsa_circ_	-0.3507	0.261418
hsa_circ_	-0.58894	0.261433
hsa_circ_	0.513024	0.261476
hsa_circ_	0.414428	0.261479
hsa_circ_	-0.20815	0.261496
hsa_circ_	0.335293	0.261506
hsa_circ_	-0.37276	0.261517
hsa_circ_	0.343045	0.261538
hsa_circ_	-0.18974	0.26154
hsa_circ_	0.150849	0.261543
hsa_circ_	0.218393	0.261545
hsa_circ_	-0.34386	0.261556
hsa_circ_	-0.31321	0.26157
hsa_circ_	0.201466	0.261581
hsa_circ_	-0.18623	0.261586
hsa_circ_	0.407066	0.2616
hsa_circ_	-0.67489	0.261619
hsa_circ_	0.606397	0.261637
hsa_circ_	-0.34366	0.261645
hsa_circ_	-1.20596	0.261688
hsa_circ_	-0.20676	0.261691
hsa_circ_	-0.24587	0.261696
hsa_circ_	0.187969	0.261698
hsa_circ_	-0.36799	0.261715
hsa_circ_	0.349743	0.261726
hsa_circ_	0.279257	0.261735
hsa_circ_	0.263739	0.261738
hsa_circ_	-0.17382	0.261747
hsa_circ_	0.776727	0.261757
hsa_circ_	-0.17538	0.261766
hsa_circ_	-0.39222	0.261776
hsa_circ_	0.221005	0.261779
hsa_circ_	-0.23653	0.261799
hsa_circ_	0.201886	0.2618
hsa_circ_	0.355765	0.261804
hsa_circ_	-0.4858	0.261804
hsa_circ_	0.273404	0.261823
hsa_circ_	0.786298	0.261845
hsa_circ_	0.223951	0.261846
hsa_circ_	-0.22769	0.261856
hsa_circ_	-0.26841	0.261875
hsa_circ_	0.159253	0.261877
hsa_circ_	0.304966	0.261878
hsa_circ_	0.50626	0.261885
hsa_circ_	-0.21961	0.261906
hsa_circ_	-0.24376	0.261914
hsa_circ_	-0.15884	0.261921
hsa_circ_	-0.75827	0.261941
hsa_circ_	-0.20458	0.261943
hsa_circ_	0.430273	0.261968

hsa_circ_	0.191921	0.261974
hsa_circ_	0.656156	0.261982
hsa_circ_	0.190556	0.261992
hsa_circ_	0.237877	0.261992
hsa_circ_	0.354755	0.261995
hsa_circ_	0.164286	0.262014
hsa_circ_	-0.67765	0.262037
hsa_circ_	0.336428	0.26204
hsa_circ_	0.528801	0.262051
hsa_circ_	-0.23902	0.262052
hsa_circ_	-0.17171	0.262054
hsa_circ_	0.413588	0.262065
hsa_circ_	-0.21536	0.262067
hsa_circ_	0.320384	0.262074
hsa_circ_	0.18212	0.262106
hsa_circ_	-0.21148	0.262117
hsa_circ_	-0.19659	0.262117
hsa_circ_	0.286018	0.262124
hsa_circ_	-0.16285	0.262127
hsa_circ_	0.205003	0.262138
hsa_circ_	-0.29409	0.262149
hsa_circ_	-0.15591	0.262168
hsa_circ_	0.383433	0.262172
hsa_circ_	-0.29628	0.262178
hsa_circ_	0.201829	0.262178
hsa_circ_	-0.25417	0.262185
hsa_circ_	-0.40784	0.262189
hsa_circ_	0.221603	0.262199
hsa_circ_	0.255018	0.262206
hsa_circ_	0.238165	0.26224
hsa_circ_	0.323977	0.262276
hsa_circ_	0.401484	0.262288
hsa_circ_	0.3939	0.262314
hsa_circ_	0.280531	0.262316
hsa_circ_	-0.2939	0.262325
hsa_circ_	-0.30311	0.262332
hsa_circ_	0.17539	0.262346
hsa_circ_	-0.47558	0.262356
hsa_circ_	0.267606	0.262364
hsa_circ_	-0.17389	0.262367
hsa_circ_	-0.20643	0.262373
hsa_circ_	0.357787	0.26239
hsa_circ_	0.189283	0.26239
hsa_circ_	0.149008	0.262394
hsa_circ_	-0.25691	0.262407
hsa_circ_	0.182667	0.262415
hsa_circ_	0.29732	0.262422
hsa_circ_	-0.1507	0.262457
hsa_circ_	0.229892	0.262459
hsa_circ_	0.217444	0.262462
hsa_circ_	-0.25446	0.262468
hsa_circ_	-0.17134	0.262471
hsa_circ_	-0.43168	0.262472
hsa_circ_	-0.72459	0.26249

hsa_circ_	-0.23372	0.262493
hsa_circ_	-0.20277	0.262517
hsa_circ_	-0.21003	0.262525
hsa_circ_	-0.1638	0.262526
hsa_circ_	-0.90051	0.262542
hsa_circ_	0.314833	0.262552
hsa_circ_	0.186332	0.262554
hsa_circ_	-0.69587	0.262564
hsa_circ_	0.171708	0.26257
hsa_circ_	-0.28192	0.262571
hsa_circ_	0.316366	0.262583
hsa_circ_	-0.21055	0.262587
hsa_circ_	-0.41581	0.262594
hsa_circ_	-0.3089	0.262612
hsa_circ_	-0.29536	0.262625
hsa_circ_	0.234446	0.262626
hsa_circ_	-0.4325	0.262633
hsa_circ_	-0.19663	0.262637
hsa_circ_	-0.23608	0.262641
hsa_circ_	0.275593	0.262644
hsa_circ_	-0.19819	0.262663
hsa_circ_	-0.35713	0.262678
hsa_circ_	-0.30328	0.262682
hsa_circ_	0.188222	0.262686
hsa_circ_	-0.19028	0.262696
hsa_circ_	-0.22494	0.262704
hsa_circ_	-0.27304	0.262736
hsa_circ_	0.224315	0.26276
hsa_circ_	-0.18225	0.262761
hsa_circ_	0.518689	0.26277
hsa_circ_	0.381718	0.26279
hsa_circ_	0.231259	0.262805
hsa_circ_	-0.37163	0.262809
hsa_circ_	0.158819	0.262813
hsa_circ_	0.246229	0.262814
hsa_circ_	-0.54342	0.262856
hsa_circ_	0.158793	0.262875
hsa_circ_	0.257436	0.262915
hsa_circ_	-0.73093	0.262915
hsa_circ_	-0.18372	0.262929
hsa_circ_	0.368119	0.262934
hsa_circ_	0.169507	0.262936
hsa_circ_	-0.1962	0.262939
hsa_circ_	0.307151	0.262942
hsa_circ_	0.448891	0.262942
hsa_circ_	0.375425	0.26295
hsa_circ_	0.209606	0.262969
hsa_circ_	-0.16589	0.262996
hsa_circ_	0.752943	0.262997
hsa_circ_	0.723032	0.263
hsa_circ_	0.23884	0.263014
hsa_circ_	0.212879	0.263021
hsa_circ_	-0.19555	0.263025
hsa_circ_	0.248647	0.263063

hsa_circ_	-0.21983	0.263073
hsa_circ_	-0.24252	0.263077
hsa_circ_	-0.17982	0.263079
hsa_circ_	0.323408	0.263118
hsa_circ_	0.267337	0.263118
hsa_circ_	0.262881	0.263134
hsa_circ_	-0.98043	0.263135
hsa_circ_	0.187943	0.263139
hsa_circ_	0.285588	0.263163
hsa_circ_	0.779775	0.263175
hsa_circ_	-0.59444	0.263197
hsa_circ_	0.208944	0.263228
hsa_circ_	-0.15876	0.263242
hsa_circ_	0.320153	0.263243
hsa_circ_	0.205059	0.263251
hsa_circ_	0.804593	0.263259
hsa_circ_	-0.23737	0.26326
hsa_circ_	-0.6615	0.26327
hsa_circ_	-0.19565	0.263275
hsa_circ_	0.243598	0.263277
hsa_circ_	-0.45058	0.263279
hsa_circ_	0.239523	0.263301
hsa_circ_	-0.31485	0.263325
hsa_circ_	-0.38677	0.26335
hsa_circ_	-0.20087	0.263355
hsa_circ_	0.276692	0.263366
hsa_circ_	0.166501	0.263367
hsa_circ_	-0.30256	0.263368
hsa_circ_	-0.29274	0.263388
hsa_circ_	-0.17802	0.26339
hsa_circ_	0.452356	0.263397
hsa_circ_	-0.15842	0.263405
hsa_circ_	-0.39132	0.263432
hsa_circ_	-0.24574	0.263438
hsa_circ_	0.169553	0.263441
hsa_circ_	-0.23632	0.26347
hsa_circ_	0.254265	0.263479
hsa_circ_	-0.23722	0.263482
hsa_circ_	-0.42895	0.263488
hsa_circ_	-0.33034	0.263502
hsa_circ_	0.235548	0.263508
hsa_circ_	-0.20167	0.263512
hsa_circ_	-0.24803	0.263517
hsa_circ_	-0.46224	0.263522
hsa_circ_	-0.14967	0.263522
hsa_circ_	-0.21033	0.26356
hsa_circ_	-0.19306	0.26357
hsa_circ_	0.282847	0.26359
hsa_circ_	0.499116	0.263616
hsa_circ_	-0.59431	0.263641
hsa_circ_	0.191822	0.263645
hsa_circ_	0.307548	0.263645
hsa_circ_	-0.2468	0.263659
hsa_circ_	-0.44682	0.26366

hsa_circ_	-0.35526	0.263684
hsa_circ_	0.233665	0.263693
hsa_circ_	-0.23912	0.263708
hsa_circ_	0.225295	0.26371
hsa_circ_	-0.2416	0.263735
hsa_circ_	0.446957	0.263739
hsa_circ_	0.318306	0.263748
hsa_circ_	-0.25939	0.263752
hsa_circ_	0.46095	0.263756
hsa_circ_	0.497255	0.263762
hsa_circ_	0.59164	0.263772
hsa_circ_	0.249009	0.263775
hsa_circ_	0.450681	0.263788
hsa_circ_	0.475857	0.263802
hsa_circ_	-0.54712	0.263807
hsa_circ_	0.234106	0.263828
hsa_circ_	0.529438	0.263831
hsa_circ_	-0.17514	0.263842
hsa_circ_	0.257653	0.263846
hsa_circ_	0.257169	0.263859
hsa_circ_	0.279957	0.263871
hsa_circ_	-0.39558	0.26388
hsa_circ_	-0.43517	0.263884
hsa_circ_	0.187255	0.263893
hsa_circ_	-0.20059	0.263918
hsa_circ_	-0.16765	0.263942
hsa_circ_	0.229641	0.263945
hsa_circ_	-0.39888	0.263954
hsa_circ_	-0.2685	0.263987
hsa_circ_	-0.18134	0.264013
hsa_circ_	-0.15735	0.264017
hsa_circ_	0.206226	0.264031
hsa_circ_	0.238315	0.264037
hsa_circ_	-0.19581	0.264044
hsa_circ_	-0.48946	0.264048
hsa_circ_	0.286035	0.264049
hsa_circ_	0.253813	0.264064
hsa_circ_	0.212915	0.26408
hsa_circ_	0.282464	0.264083
hsa_circ_	-0.28006	0.264092
hsa_circ_	0.207092	0.264103
hsa_circ_	-0.59025	0.26412
hsa_circ_	-0.47003	0.26412
hsa_circ_	-0.64259	0.26414
hsa_circ_	-0.24301	0.26415
hsa_circ_	0.275803	0.264153
hsa_circ_	0.243815	0.26416
hsa_circ_	0.300281	0.264168
hsa_circ_	-0.21193	0.264168
hsa_circ_	-0.33583	0.264173
hsa_circ_	-0.48024	0.264176
hsa_circ_	-0.4715	0.264183
hsa_circ_	-0.21534	0.264198
hsa_circ_	0.176324	0.264221

hsa_circ_	-0.26703	0.264224
hsa_circ_	-0.28879	0.264231
hsa_circ_	-0.15977	0.264245
hsa_circ_	0.165533	0.264259
hsa_circ_	0.173838	0.264262
hsa_circ_	0.254488	0.264274
hsa_circ_	0.388763	0.264315
hsa_circ_	0.271147	0.264357
hsa_circ_	0.18499	0.264366
hsa_circ_	0.170888	0.264373
hsa_circ_	0.280736	0.264376
hsa_circ_	-0.31082	0.264377
hsa_circ_	0.302398	0.264377
hsa_circ_	0.52175	0.26441
hsa_circ_	0.274649	0.264413
hsa_circ_	0.167647	0.264414
hsa_circ_	-0.26743	0.264419
hsa_circ_	-0.16266	0.264421
hsa_circ_	-0.20755	0.26443
hsa_circ_	-0.18106	0.264434
hsa_circ_	0.171937	0.264455
hsa_circ_	-0.18057	0.264467
hsa_circ_	-0.2199	0.264472
hsa_circ_	0.514971	0.26448
hsa_circ_	-0.18395	0.264494
hsa_circ_	-0.24467	0.264506
hsa_circ_	-0.2347	0.264516
hsa_circ_	-0.19199	0.264517
hsa_circ_	-0.19422	0.264537
hsa_circ_	-0.30348	0.264544
hsa_circ_	0.771009	0.264563
hsa_circ_	-0.30868	0.264574
hsa_circ_	0.543882	0.26459
hsa_circ_	-0.1826	0.264597
hsa_circ_	-0.21264	0.264603
hsa_circ_	0.280781	0.264616
hsa_circ_	-0.26406	0.264616
hsa_circ_	0.213184	0.264632
hsa_circ_	0.161262	0.264638
hsa_circ_	0.23925	0.264645
hsa_circ_	0.490737	0.264671
hsa_circ_	-0.65395	0.264677
hsa_circ_	0.215747	0.264693
hsa_circ_	-0.19637	0.264697
hsa_circ_	0.344917	0.264701
hsa_circ_	-0.39198	0.264707
hsa_circ_	0.272041	0.264709
hsa_circ_	-0.16374	0.264716
hsa_circ_	-0.27602	0.264726
hsa_circ_	0.174034	0.264726
hsa_circ_	-0.17343	0.264751
hsa_circ_	0.202104	0.264756
hsa_circ_	0.305927	0.264759
hsa_circ_	0.396096	0.264765

hsa_circ_	0.279935	0.264768
hsa_circ_	0.202754	0.26478
hsa_circ_	-0.23394	0.26478
hsa_circ_	-0.16463	0.264783
hsa_circ_	-0.42154	0.264793
hsa_circ_	0.357472	0.264795
hsa_circ_	0.214451	0.264799
hsa_circ_	-0.27658	0.2648
hsa_circ_	-0.26644	0.264804
hsa_circ_	0.391847	0.264806
hsa_circ_	0.344551	0.264806
hsa_circ_	-0.67407	0.264811
hsa_circ_	-0.21415	0.264816
hsa_circ_	0.282398	0.264824
hsa_circ_	-0.15349	0.264827
hsa_circ_	0.160477	0.264829
hsa_circ_	-0.19758	0.264834
hsa_circ_	0.407109	0.264835
hsa_circ_	0.191196	0.264851
hsa_circ_	0.202738	0.264858
hsa_circ_	-0.4276	0.264874
hsa_circ_	-0.36498	0.264875
hsa_circ_	0.176531	0.264879
hsa_circ_	0.293141	0.264888
hsa_circ_	-0.15792	0.264897
hsa_circ_	-0.17274	0.264901
hsa_circ_	-0.20489	0.264952
hsa_circ_	-0.19507	0.264953
hsa_circ_	-0.22754	0.264962
hsa_circ_	0.157198	0.264963
hsa_circ_	-0.4524	0.264987
hsa_circ_	0.710439	0.264992
hsa_circ_	-0.82847	0.265003
hsa_circ_	-0.35301	0.265009
hsa_circ_	-0.22285	0.265026
hsa_circ_	0.167092	0.265045
hsa_circ_	0.166872	0.265049
hsa_circ_	-0.15887	0.265049
hsa_circ_	0.188978	0.265051
hsa_circ_	0.186964	0.265065
hsa_circ_	-0.47901	0.265072
hsa_circ_	-0.50964	0.265073
hsa_circ_	0.482483	0.265075
hsa_circ_	-0.15035	0.265081
hsa_circ_	0.605617	0.265097
hsa_circ_	0.494613	0.265099
hsa_circ_	0.157857	0.265104
hsa_circ_	-0.20851	0.265118
hsa_circ_	-0.17392	0.265148
hsa_circ_	0.45984	0.265151
hsa_circ_	-0.4692	0.26517
hsa_circ_	-0.22862	0.265188
hsa_circ_	-0.66314	0.265197
hsa_circ_	0.208056	0.265217

hsa_circ_	0.183724	0.265223
hsa_circ_	-0.15872	0.265224
hsa_circ_	0.461074	0.265228
hsa_circ_	-0.81965	0.265235
hsa_circ_	-0.58735	0.265235
hsa_circ_	0.3344	0.265236
hsa_circ_	0.579612	0.265239
hsa_circ_	0.277836	0.265263
hsa_circ_	-0.38226	0.265305
hsa_circ_	0.19952	0.265307
hsa_circ_	-0.43827	0.265313
hsa_circ_	-0.36837	0.265323
hsa_circ_	-0.25074	0.265325
hsa_circ_	0.223251	0.265347
hsa_circ_	-0.31674	0.265372
hsa_circ_	0.832659	0.265387
hsa_circ_	-0.34266	0.265397
hsa_circ_	-0.76135	0.265414
hsa_circ_	0.196407	0.265434
hsa_circ_	0.284624	0.26544
hsa_circ_	-0.59289	0.265449
hsa_circ_	-0.19053	0.265455
hsa_circ_	-0.23861	0.265455
hsa_circ_	0.182864	0.265459
hsa_circ_	0.797022	0.265469
hsa_circ_	0.223304	0.265479
hsa_circ_	-0.37364	0.265504
hsa_circ_	0.324133	0.265504
hsa_circ_	0.166992	0.265515
hsa_circ_	-0.23641	0.265518
hsa_circ_	-0.26394	0.265526
hsa_circ_	-0.23893	0.265529
hsa_circ_	-0.21742	0.265547
hsa_circ_	0.213966	0.26556
hsa_circ_	-0.25294	0.265561
hsa_circ_	0.217481	0.265575
hsa_circ_	0.182694	0.265586
hsa_circ_	0.312683	0.26559
hsa_circ_	0.449864	0.265602
hsa_circ_	0.437081	0.265606
hsa_circ_	0.156601	0.265612
hsa_circ_	-0.20215	0.265627
hsa_circ_	0.231408	0.26563
hsa_circ_	-0.26855	0.265644
hsa_circ_	-0.88578	0.265677
hsa_circ_	0.20866	0.26571
hsa_circ_	-0.2403	0.26572
hsa_circ_	-0.20229	0.265751
hsa_circ_	-0.23801	0.265753
hsa_circ_	-0.23535	0.265756
hsa_circ_	-0.55515	0.265758
hsa_circ_	-0.24698	0.265758
hsa_circ_	-0.21229	0.265768
hsa_circ_	0.288025	0.265778

hsa_circ_	-0.78274	0.265782
hsa_circ_	-0.3665	0.265784
hsa_circ_	-0.16532	0.265805
hsa_circ_	0.191898	0.265806
hsa_circ_	0.258871	0.265812
hsa_circ_	-0.2048	0.265906
hsa_circ_	-0.1665	0.265937
hsa_circ_	-0.1863	0.265941
hsa_circ_	0.302786	0.265948
hsa_circ_	0.190736	0.265949
hsa_circ_	0.200184	0.265966
hsa_circ_	0.623894	0.265971
hsa_circ_	-0.30279	0.265977
hsa_circ_	0.228816	0.265985
hsa_circ_	0.196268	0.266023
hsa_circ_	0.204722	0.266028
hsa_circ_	0.650829	0.266067
hsa_circ_	-0.77748	0.266079
hsa_circ_	-0.2123	0.266087
hsa_circ_	-0.34558	0.266089
hsa_circ_	0.242648	0.266095
hsa_circ_	0.61531	0.266097
hsa_circ_	-0.36326	0.266115
hsa_circ_	-0.22856	0.266119
hsa_circ_	-0.22075	0.266121
hsa_circ_	0.278144	0.266124
hsa_circ_	0.200573	0.266157
hsa_circ_	-0.67134	0.266163
hsa_circ_	-0.22086	0.26617
hsa_circ_	0.237448	0.266221
hsa_circ_	0.238224	0.266261
hsa_circ_	-0.22639	0.266263
hsa_circ_	-0.31711	0.266286
hsa_circ_	0.373207	0.266299
hsa_circ_	-0.15792	0.266302
hsa_circ_	0.199477	0.266308
hsa_circ_	-0.3366	0.266308
hsa_circ_	-0.43267	0.266312
hsa_circ_	0.198243	0.266314
hsa_circ_	-0.40865	0.26633
hsa_circ_	0.336446	0.266333
hsa_circ_	-0.19317	0.26639
hsa_circ_	0.359692	0.266398
hsa_circ_	-0.9307	0.266431
hsa_circ_	0.305676	0.266447
hsa_circ_	-0.38253	0.266499
hsa_circ_	-0.18743	0.266517
hsa_circ_	-0.19778	0.266553
hsa_circ_	-0.3393	0.266562
hsa_circ_	0.196376	0.266578
hsa_circ_	-0.36686	0.266582
hsa_circ_	-0.31875	0.266584
hsa_circ_	0.284403	0.266593
hsa_circ_	0.529591	0.266626

hsa_circ_	-0.18576	0.266634
hsa_circ_	0.186717	0.266636
hsa_circ_	0.163349	0.266639
hsa_circ_	0.195573	0.266666
hsa_circ_	-0.23684	0.266668
hsa_circ_	-0.2501	0.266676
hsa_circ_	0.261548	0.266686
hsa_circ_	-0.70703	0.266686
hsa_circ_	-0.29776	0.266691
hsa_circ_	-0.22976	0.266696
hsa_circ_	-0.2628	0.266703
hsa_circ_	-0.45286	0.266704
hsa_circ_	-0.2805	0.26671
hsa_circ_	-0.18852	0.26672
hsa_circ_	0.204997	0.266739
hsa_circ_	-0.3571	0.266743
hsa_circ_	-0.26226	0.266744
hsa_circ_	-0.18995	0.266747
hsa_circ_	0.354449	0.266764
hsa_circ_	0.228734	0.266776
hsa_circ_	-0.40606	0.266779
hsa_circ_	-0.56113	0.266806
hsa_circ_	0.450807	0.266808
hsa_circ_	-0.73117	0.266814
hsa_circ_	-0.25239	0.26683
hsa_circ_	0.151809	0.26684
hsa_circ_	-0.20221	0.266842
hsa_circ_	-0.15983	0.266852
hsa_circ_	0.278904	0.26686
hsa_circ_	-0.44905	0.266877
hsa_circ_	-0.22856	0.266885
hsa_circ_	1.2524	0.266889
hsa_circ_	-0.15017	0.266908
hsa_circ_	0.170485	0.26691
hsa_circ_	0.389794	0.266921
hsa_circ_	-0.27559	0.266924
hsa_circ_	0.271959	0.266928
hsa_circ_	-0.2669	0.266929
hsa_circ_	0.524755	0.266932
hsa_circ_	-0.25706	0.26695
hsa_circ_	-0.64136	0.26695
hsa_circ_	0.166608	0.26696
hsa_circ_	0.155478	0.266968
hsa_circ_	0.171009	0.266987
hsa_circ_	0.21877	0.266998
hsa_circ_	-0.15502	0.267003
hsa_circ_	-0.20671	0.267005
hsa_circ_	0.178355	0.267018
hsa_circ_	-0.18414	0.267034
hsa_circ_	0.308406	0.267039
hsa_circ_	-0.16439	0.267044
hsa_circ_	0.454188	0.267049
hsa_circ_	-0.24249	0.267055
hsa_circ_	0.42571	0.267056

hsa_circ_	-0.56982	0.267067
hsa_circ_	0.423269	0.267075
hsa_circ_	-0.26556	0.267078
hsa_circ_	0.206589	0.267124
hsa_circ_	0.289778	0.267136
hsa_circ_	-0.31435	0.267153
hsa_circ_	0.301229	0.267156
hsa_circ_	-0.29421	0.267178
hsa_circ_	-0.20721	0.267193
hsa_circ_	-0.20752	0.267196
hsa_circ_	0.197655	0.267202
hsa_circ_	-0.34537	0.267218
hsa_circ_	-0.22388	0.267218
hsa_circ_	-0.85423	0.267252
hsa_circ_	-0.19664	0.267264
hsa_circ_	0.189123	0.267265
hsa_circ_	0.771927	0.267267
hsa_circ_	-0.23641	0.267271
hsa_circ_	-0.16889	0.267275
hsa_circ_	-0.24739	0.267284
hsa_circ_	-0.17824	0.267293
hsa_circ_	-0.71035	0.267296
hsa_circ_	0.35316	0.267303
hsa_circ_	0.761941	0.267313
hsa_circ_	-0.26248	0.267331
hsa_circ_	-0.49488	0.267337
hsa_circ_	-0.65865	0.267359
hsa_circ_	-0.22191	0.267367
hsa_circ_	-0.21771	0.267382
hsa_circ_	0.194729	0.267395
hsa_circ_	-0.18327	0.267399
hsa_circ_	0.219837	0.267405
hsa_circ_	-0.25365	0.267405
hsa_circ_	-0.2197	0.267415
hsa_circ_	-0.66758	0.267416
hsa_circ_	0.397404	0.267417
hsa_circ_	-0.21084	0.267424
hsa_circ_	0.228186	0.267427
hsa_circ_	0.173821	0.267451
hsa_circ_	0.425375	0.267465
hsa_circ_	-0.20609	0.267473
hsa_circ_	-0.20121	0.267474
hsa_circ_	-0.24933	0.267512
hsa_circ_	-1.36466	0.267519
hsa_circ_	-0.26481	0.267529
hsa_circ_	-0.16966	0.267538
hsa_circ_	0.729683	0.267558
hsa_circ_	-0.51396	0.26756
hsa_circ_	-0.47936	0.267578
hsa_circ_	-0.24464	0.267589
hsa_circ_	-0.22603	0.267596
hsa_circ_	-0.18117	0.2676
hsa_circ_	0.510705	0.267605
hsa_circ_	-0.29181	0.267642

hsa_circ_	-0.14721	0.26765
hsa_circ_	-0.39548	0.267664
hsa_circ_	-0.20887	0.267678
hsa_circ_	-0.5288	0.267682
hsa_circ_	-0.77877	0.267686
hsa_circ_	0.171809	0.267693
hsa_circ_	-0.32312	0.267707
hsa_circ_	-0.17292	0.267737
hsa_circ_	-0.34358	0.267739
hsa_circ_	0.186319	0.267758
hsa_circ_	-0.20193	0.267758
hsa_circ_	-0.36715	0.267763
hsa_circ_	-0.69359	0.26779
hsa_circ_	0.361718	0.267794
hsa_circ_	-0.79877	0.267815
hsa_circ_	0.241467	0.267819
hsa_circ_	-0.18207	0.267832
hsa_circ_	-0.18096	0.267835
hsa_circ_	0.195919	0.267857
hsa_circ_	-0.40697	0.267858
hsa_circ_	0.184006	0.267876
hsa_circ_	-0.37159	0.267905
hsa_circ_	0.182864	0.267925
hsa_circ_	-0.30295	0.267939
hsa_circ_	-0.24244	0.267947
hsa_circ_	-0.16015	0.267995
hsa_circ_	0.183892	0.268013
hsa_circ_	0.24466	0.268018
hsa_circ_	-0.22148	0.268034
hsa_circ_	-0.21051	0.268044
hsa_circ_	-0.37473	0.268044
hsa_circ_	-0.47047	0.268047
hsa_circ_	-0.30635	0.268053
hsa_circ_	-0.18112	0.268062
hsa_circ_	-0.18925	0.268064
hsa_circ_	0.196487	0.268064
hsa_circ_	-0.32387	0.268082
hsa_circ_	0.441733	0.268106
hsa_circ_	0.495526	0.268125
hsa_circ_	0.252094	0.268139
hsa_circ_	-0.26747	0.268142
hsa_circ_	0.611642	0.268181
hsa_circ_	0.596592	0.268194
hsa_circ_	-0.39636	0.268196
hsa_circ_	-0.29989	0.26821
hsa_circ_	0.268149	0.268211
hsa_circ_	-0.61256	0.268213
hsa_circ_	0.15368	0.268233
hsa_circ_	-0.30955	0.268235
hsa_circ_	-0.24056	0.268258
hsa_circ_	0.313529	0.268264
hsa_circ_	0.383954	0.268268
hsa_circ_	-0.17232	0.268275
hsa_circ_	-0.53475	0.268305

hsa_circ_	-0.17483	0.268306
hsa_circ_	-0.2314	0.268323
hsa_circ_	-0.46139	0.268327
hsa_circ_	-0.18203	0.268343
hsa_circ_	-0.26816	0.268368
hsa_circ_	0.537832	0.268403
hsa_circ_	-0.23509	0.268409
hsa_circ_	0.231071	0.268417
hsa_circ_	-0.35864	0.268427
hsa_circ_	-0.16918	0.268428
hsa_circ_	-0.17312	0.268441
hsa_circ_	-1.13207	0.268443
hsa_circ_	0.197657	0.268446
hsa_circ_	-0.16579	0.268472
hsa_circ_	0.218668	0.268489
hsa_circ_	-0.30276	0.268506
hsa_circ_	1.871387	0.268519
hsa_circ_	-0.18488	0.268529
hsa_circ_	-0.1495	0.268554
hsa_circ_	0.460452	0.268555
hsa_circ_	-0.24165	0.268572
hsa_circ_	-0.7004	0.268577
hsa_circ_	0.174704	0.268584
hsa_circ_	0.209074	0.268647
hsa_circ_	-0.1554	0.268649
hsa_circ_	0.180943	0.268661
hsa_circ_	0.457404	0.268666
hsa_circ_	-0.18728	0.26868
hsa_circ_	-0.98908	0.268683
hsa_circ_	0.153377	0.268691
hsa_circ_	-0.16659	0.268719
hsa_circ_	0.398261	0.26872
hsa_circ_	-0.89066	0.268736
hsa_circ_	-0.17024	0.268742
hsa_circ_	0.255179	0.268746
hsa_circ_	0.416577	0.268759
hsa_circ_	-0.43626	0.268767
hsa_circ_	-0.24669	0.268768
hsa_circ_	0.190357	0.268769
hsa_circ_	-0.88093	0.268775
hsa_circ_	-0.25503	0.268849
hsa_circ_	0.220718	0.268853
hsa_circ_	-0.18332	0.268857
hsa_circ_	-0.27029	0.268868
hsa_circ_	0.33246	0.268879
hsa_circ_	-0.60444	0.268881
hsa_circ_	0.3041	0.268882
hsa_circ_	0.210999	0.268883
hsa_circ_	-0.26144	0.268887
hsa_circ_	0.433808	0.268924
hsa_circ_	0.211277	0.268924
hsa_circ_	0.551261	0.26893
hsa_circ_	-0.22438	0.268936
hsa_circ_	0.241568	0.268946

hsa_circ_	0.2228	0.268974
hsa_circ_	-0.47875	0.268979
hsa_circ_	-0.22479	0.26899
hsa_circ_	-0.4397	0.268997
hsa_circ_	0.213672	0.269001
hsa_circ_	-0.8772	0.269003
hsa_circ_	-1.27668	0.269008
hsa_circ_	0.443949	0.269027
hsa_circ_	0.334533	0.269029
hsa_circ_	0.350292	0.269041
hsa_circ_	0.428774	0.269043
hsa_circ_	-1.09359	0.269058
hsa_circ_	0.200877	0.269058
hsa_circ_	-0.3263	0.269082
hsa_circ_	-0.42119	0.269137
hsa_circ_	0.274769	0.269184
hsa_circ_	-0.22545	0.269199
hsa_circ_	-0.22985	0.269211
hsa_circ_	0.179398	0.269214
hsa_circ_	0.213485	0.269215
hsa_circ_	-0.2248	0.269217
hsa_circ_	-0.1931	0.269222
hsa_circ_	-0.17066	0.269227
hsa_circ_	-0.15501	0.269228
hsa_circ_	0.479923	0.269228
hsa_circ_	-0.20449	0.269254
hsa_circ_	0.154774	0.26926
hsa_circ_	-0.21873	0.269267
hsa_circ_	0.355054	0.269302
hsa_circ_	0.266361	0.269306
hsa_circ_	0.196529	0.269317
hsa_circ_	-0.20213	0.269326
hsa_circ_	0.385068	0.269335
hsa_circ_	0.369802	0.269344
hsa_circ_	0.204922	0.269352
hsa_circ_	0.278888	0.269369
hsa_circ_	-0.15952	0.269379
hsa_circ_	0.18145	0.269445
hsa_circ_	0.357572	0.269449
hsa_circ_	0.208033	0.269457
hsa_circ_	0.393379	0.269467
hsa_circ_	-0.85353	0.269482
hsa_circ_	-0.19512	0.2695
hsa_circ_	0.292402	0.269551
hsa_circ_	-0.25463	0.269572
hsa_circ_	0.218318	0.269612
hsa_circ_	0.246557	0.269614
hsa_circ_	-0.41339	0.269617
hsa_circ_	-0.23755	0.269624
hsa_circ_	-0.36723	0.269629
hsa_circ_	-0.29857	0.269632
hsa_circ_	-0.22303	0.269637
hsa_circ_	-0.21811	0.269663
hsa_circ_	-0.35564	0.269672

hsa_circ_	-0.3477	0.269673
hsa_circ_	-0.14978	0.269697
hsa_circ_	0.200629	0.269718
hsa_circ_	0.362965	0.269723
hsa_circ_	0.208804	0.269724
hsa_circ_	0.321016	0.269732
hsa_circ_	-0.27081	0.269754
hsa_circ_	-0.24355	0.269762
hsa_circ_	-0.31479	0.269775
hsa_circ_	-0.22872	0.269787
hsa_circ_	0.435667	0.269789
hsa_circ_	0.342358	0.26981
hsa_circ_	-0.47022	0.269811
hsa_circ_	-0.2348	0.269818
hsa_circ_	-0.29288	0.269834
hsa_circ_	-0.34183	0.269834
hsa_circ_	0.380738	0.269846
hsa_circ_	-0.21587	0.269857
hsa_circ_	-0.16324	0.269865
hsa_circ_	0.256996	0.269877
hsa_circ_	0.18261	0.269879
hsa_circ_	-0.32891	0.269992
hsa_circ_	-1.33662	0.270018
hsa_circ_	-0.46896	0.270028
hsa_circ_	-0.22747	0.270042
hsa_circ_	0.235246	0.270046
hsa_circ_	0.752757	0.27008
hsa_circ_	-0.21542	0.270088
hsa_circ_	-0.44706	0.270091
hsa_circ_	-0.52218	0.270094
hsa_circ_	0.174644	0.270107
hsa_circ_	0.19806	0.270117
hsa_circ_	-0.24204	0.27012
hsa_circ_	-0.1852	0.270133
hsa_circ_	0.272319	0.270137
hsa_circ_	0.230578	0.270158
hsa_circ_	-1.03248	0.270174
hsa_circ_	-0.42372	0.270174
hsa_circ_	-0.17126	0.270187
hsa_circ_	-0.22391	0.270205
hsa_circ_	0.656686	0.270215
hsa_circ_	-0.43168	0.270217
hsa_circ_	0.69439	0.270227
hsa_circ_	-0.15492	0.270238
hsa_circ_	0.629497	0.270287
hsa_circ_	0.169456	0.270287
hsa_circ_	-0.62698	0.270307
hsa_circ_	0.271758	0.270326
hsa_circ_	0.187412	0.270328
hsa_circ_	0.259875	0.270341
hsa_circ_	-0.21193	0.270347
hsa_circ_	-0.42915	0.270352
hsa_circ_	-0.43775	0.270354
hsa_circ_	-0.19129	0.27036

hsa_circ_	-0.18898	0.270362
hsa_circ_	-0.75342	0.270387
hsa_circ_	-0.17397	0.270387
hsa_circ_	0.211159	0.270392
hsa_circ_	-0.25667	0.270412
hsa_circ_	0.807901	0.270414
hsa_circ_	-1.10832	0.270417
hsa_circ_	0.235356	0.270425
hsa_circ_	-0.44855	0.270447
hsa_circ_	-0.19225	0.270449
hsa_circ_	0.274527	0.27045
hsa_circ_	0.201687	0.270475
hsa_circ_	0.222423	0.270486
hsa_circ_	-0.35788	0.270492
hsa_circ_	-0.25683	0.270493
hsa_circ_	-0.1839	0.270501
hsa_circ_	-0.21491	0.270502
hsa_circ_	0.52818	0.270516
hsa_circ_	0.75464	0.270516
hsa_circ_	-0.19236	0.270529
hsa_circ_	0.159729	0.270532
hsa_circ_	-0.60317	0.270534
hsa_circ_	-0.18541	0.270535
hsa_circ_	0.554065	0.270537
hsa_circ_	-0.21603	0.270551
hsa_circ_	-0.37341	0.270581
hsa_circ_	0.447374	0.27059
hsa_circ_	-0.56688	0.270595
hsa_circ_	0.195219	0.270595
hsa_circ_	-0.71447	0.270612
hsa_circ_	0.162374	0.270621
hsa_circ_	-0.16815	0.270633
hsa_circ_	-0.79741	0.270642
hsa_circ_	-0.21098	0.270652
hsa_circ_	0.359948	0.270657
hsa_circ_	0.335664	0.270684
hsa_circ_	-0.54065	0.270709
hsa_circ_	-0.63034	0.270712
hsa_circ_	0.257902	0.270716
hsa_circ_	-0.19818	0.270719
hsa_circ_	0.265109	0.270732
hsa_circ_	0.325285	0.270736
hsa_circ_	-0.18591	0.270739
hsa_circ_	-0.4052	0.270763
hsa_circ_	-0.16029	0.270763
hsa_circ_	0.227556	0.270768
hsa_circ_	-0.65264	0.270788
hsa_circ_	0.17421	0.270801
hsa_circ_	0.48194	0.270802
hsa_circ_	-0.25174	0.270815
hsa_circ_	-0.4901	0.270828
hsa_circ_	0.30487	0.270828
hsa_circ_	0.16998	0.270834
hsa_circ_	-0.52012	0.270846

hsa_circ_	0.440458	0.270861
hsa_circ_	0.247576	0.270881
hsa_circ_	0.168009	0.270886
hsa_circ_	-0.52171	0.270887
hsa_circ_	-0.22218	0.270891
hsa_circ_	0.540182	0.2709
hsa_circ_	-0.15981	0.270905
hsa_circ_	0.210748	0.270905
hsa_circ_	0.226581	0.270944
hsa_circ_	0.806967	0.270959
hsa_circ_	0.240578	0.270962
hsa_circ_	-0.28136	0.270963
hsa_circ_	0.535951	0.271025
hsa_circ_	0.361207	0.271034
hsa_circ_	-0.2881	0.271048
hsa_circ_	-0.34861	0.271058
hsa_circ_	0.160285	0.271074
hsa_circ_	0.328373	0.271079
hsa_circ_	-0.2223	0.271089
hsa_circ_	-0.1778	0.271094
hsa_circ_	-0.17146	0.271095
hsa_circ_	-0.26185	0.271105
hsa_circ_	-0.34828	0.271107
hsa_circ_	0.661341	0.271126
hsa_circ_	0.175655	0.271151
hsa_circ_	-2.27118	0.271154
hsa_circ_	0.333711	0.271168
hsa_circ_	0.36692	0.271177
hsa_circ_	0.144287	0.271178
hsa_circ_	0.369679	0.271179
hsa_circ_	-1.08873	0.271185
hsa_circ_	-0.28309	0.271203
hsa_circ_	-0.20929	0.271218
hsa_circ_	-0.27612	0.271235
hsa_circ_	-0.59236	0.271246
hsa_circ_	0.179189	0.271252
hsa_circ_	0.250607	0.271252
hsa_circ_	-0.21995	0.271256
hsa_circ_	-0.17677	0.271265
hsa_circ_	0.198746	0.271269
hsa_circ_	0.333048	0.271274
hsa_circ_	0.359566	0.27129
hsa_circ_	0.161942	0.2713
hsa_circ_	-0.2032	0.271305
hsa_circ_	-0.23752	0.271328
hsa_circ_	0.149501	0.271341
hsa_circ_	0.170087	0.271349
hsa_circ_	-0.82698	0.271359
hsa_circ_	-0.69941	0.271362
hsa_circ_	0.270902	0.271367
hsa_circ_	-0.18095	0.271369
hsa_circ_	0.242241	0.271374
hsa_circ_	-0.41347	0.271374
hsa_circ_	0.508016	0.271382

hsa_circ_	0.263765	0.27139
hsa_circ_	0.402857	0.271406
hsa_circ_	-0.35877	0.271409
hsa_circ_	-0.15219	0.271411
hsa_circ_	-0.50491	0.271412
hsa_circ_	-0.24831	0.271413
hsa_circ_	0.404649	0.271422
hsa_circ_	0.395416	0.271424
hsa_circ_	0.353471	0.271431
hsa_circ_	-0.25776	0.27144
hsa_circ_	-0.38	0.27144
hsa_circ_	0.22569	0.271442
hsa_circ_	0.280556	0.271443
hsa_circ_	0.318186	0.271453
hsa_circ_	-0.2624	0.271455
hsa_circ_	-0.17657	0.271464
hsa_circ_	-0.29109	0.271486
hsa_circ_	0.242056	0.271508
hsa_circ_	-0.17596	0.271526
hsa_circ_	0.186589	0.271552
hsa_circ_	-0.70761	0.271552
hsa_circ_	-1.02289	0.271563
hsa_circ_	0.270305	0.271563
hsa_circ_	-0.21014	0.271581
hsa_circ_	0.710249	0.271592
hsa_circ_	0.235679	0.271603
hsa_circ_	0.173941	0.27162
hsa_circ_	0.920558	0.271658
hsa_circ_	0.201683	0.271678
hsa_circ_	1.066383	0.271689
hsa_circ_	0.32547	0.271697
hsa_circ_	0.218302	0.271698
hsa_circ_	-0.17359	0.271729
hsa_circ_	-0.21123	0.27173
hsa_circ_	-0.21081	0.271746
hsa_circ_	-0.17648	0.271769
hsa_circ_	0.253688	0.271779
hsa_circ_	0.252298	0.271786
hsa_circ_	0.148059	0.271792
hsa_circ_	-0.37287	0.271801
hsa_circ_	-0.28138	0.271803
hsa_circ_	0.181351	0.27183
hsa_circ_	-0.23809	0.271833
hsa_circ_	0.252073	0.271836
hsa_circ_	0.208385	0.271841
hsa_circ_	-0.19282	0.271848
hsa_circ_	0.168992	0.271865
hsa_circ_	-0.36809	0.271878
hsa_circ_	0.2711	0.271881
hsa_circ_	-0.42373	0.271886
hsa_circ_	0.235472	0.271896
hsa_circ_	-0.46197	0.271905
hsa_circ_	0.393696	0.271914
hsa_circ_	-0.19483	0.271953

hsa_circ_	0.156643	0.271957
hsa_circ_	0.462583	0.271961
hsa_circ_	0.177094	0.271963
hsa_circ_	-0.15625	0.271966
hsa_circ_	-0.69566	0.272013
hsa_circ_	0.442099	0.272016
hsa_circ_	-0.15598	0.27202
hsa_circ_	0.243724	0.272045
hsa_circ_	-0.54458	0.272059
hsa_circ_	-0.19431	0.272075
hsa_circ_	0.397641	0.272082
hsa_circ_	-0.59174	0.272084
hsa_circ_	-0.17212	0.272095
hsa_circ_	-0.39944	0.272108
hsa_circ_	0.462287	0.272111
hsa_circ_	-0.17828	0.272129
hsa_circ_	-0.21104	0.272165
hsa_circ_	0.315497	0.272172
hsa_circ_	-0.26456	0.272173
hsa_circ_	-0.81527	0.272187
hsa_circ_	0.668867	0.272192
hsa_circ_	-0.16	0.272204
hsa_circ_	0.220146	0.272211
hsa_circ_	0.152863	0.272228
hsa_circ_	-0.96131	0.272244
hsa_circ_	-0.43541	0.272264
hsa_circ_	-0.19802	0.272275
hsa_circ_	-0.22634	0.272322
hsa_circ_	-0.28793	0.272323
hsa_circ_	-0.37056	0.272323
hsa_circ_	0.215902	0.272346
hsa_circ_	1.120368	0.272352
hsa_circ_	0.645844	0.272377
hsa_circ_	0.569254	0.272378
hsa_circ_	0.322292	0.272389
hsa_circ_	0.270391	0.272395
hsa_circ_	-0.15322	0.272399
hsa_circ_	0.198688	0.272427
hsa_circ_	0.22891	0.272432
hsa_circ_	-0.18589	0.272435
hsa_circ_	-0.16375	0.272452
hsa_circ_	0.269294	0.272455
hsa_circ_	0.345302	0.272464
hsa_circ_	-0.15743	0.272467
hsa_circ_	0.207463	0.272468
hsa_circ_	-0.2201	0.272515
hsa_circ_	-0.20329	0.272516
hsa_circ_	0.421114	0.272539
hsa_circ_	-0.34613	0.272544
hsa_circ_	-0.24048	0.272544
hsa_circ_	0.200476	0.272567
hsa_circ_	0.199344	0.272584
hsa_circ_	-0.19271	0.272588
hsa_circ_	0.161857	0.272591

hsa_circ_	-0.2134	0.272593
hsa_circ_	-0.50501	0.272597
hsa_circ_	-0.22261	0.272609
hsa_circ_	-0.16749	0.272621
hsa_circ_	0.246238	0.272633
hsa_circ_	0.365833	0.272668
hsa_circ_	0.190206	0.272679
hsa_circ_	0.241333	0.272682
hsa_circ_	-0.24529	0.272682
hsa_circ_	-0.49454	0.272684
hsa_circ_	-0.16357	0.272684
hsa_circ_	0.174813	0.272684
hsa_circ_	0.660656	0.272689
hsa_circ_	-0.63269	0.272704
hsa_circ_	0.366924	0.272723
hsa_circ_	-0.16138	0.272727
hsa_circ_	0.166804	0.272741
hsa_circ_	-0.4087	0.272757
hsa_circ_	0.262598	0.27276
hsa_circ_	0.205296	0.272767
hsa_circ_	0.2247	0.272803
hsa_circ_	-0.16794	0.272855
hsa_circ_	-0.25727	0.272862
hsa_circ_	-0.18721	0.27287
hsa_circ_	0.327138	0.272876
hsa_circ_	-0.17244	0.272883
hsa_circ_	-0.88098	0.272893
hsa_circ_	0.334777	0.272895
hsa_circ_	-0.86271	0.272898
hsa_circ_	0.258867	0.272912
hsa_circ_	-0.24501	0.27293
hsa_circ_	-0.21724	0.272934
hsa_circ_	-0.17058	0.272936
hsa_circ_	-0.16073	0.272941
hsa_circ_	-0.63757	0.272949
hsa_circ_	0.351985	0.272952
hsa_circ_	0.178756	0.272962
hsa_circ_	0.17945	0.272981
hsa_circ_	-0.23671	0.272986
hsa_circ_	0.336026	0.272997
hsa_circ_	0.713363	0.273001
hsa_circ_	-0.3412	0.273004
hsa_circ_	-0.48198	0.273036
hsa_circ_	-0.80068	0.273057
hsa_circ_	-0.24251	0.273085
hsa_circ_	-0.32549	0.273117
hsa_circ_	0.214932	0.273124
hsa_circ_	0.197529	0.273144
hsa_circ_	0.162083	0.273144
hsa_circ_	-0.37124	0.273149
hsa_circ_	-0.48387	0.27315
hsa_circ_	-0.29538	0.273151
hsa_circ_	0.304139	0.273162
hsa_circ_	0.404882	0.273181

hsa_circ_	-0.50712	0.273186
hsa_circ_	-0.16675	0.273192
hsa_circ_	0.304325	0.273193
hsa_circ_	0.215444	0.273224
hsa_circ_	0.243901	0.273236
hsa_circ_	0.168511	0.273236
hsa_circ_	-0.17157	0.273249
hsa_circ_	-0.21153	0.273264
hsa_circ_	-0.32205	0.273273
hsa_circ_	-0.40326	0.273294
hsa_circ_	0.60685	0.273311
hsa_circ_	-0.1903	0.273318
hsa_circ_	-0.64065	0.273329
hsa_circ_	0.329351	0.273331
hsa_circ_	0.154043	0.273347
hsa_circ_	-0.41891	0.273362
hsa_circ_	-0.59879	0.273387
hsa_circ_	-0.29028	0.273393
hsa_circ_	0.252926	0.273398
hsa_circ_	0.56147	0.273412
hsa_circ_	0.355599	0.273415
hsa_circ_	-0.1604	0.273416
hsa_circ_	0.651012	0.273436
hsa_circ_	-0.22174	0.273453
hsa_circ_	0.305042	0.273462
hsa_circ_	0.445652	0.273471
hsa_circ_	-0.33672	0.273501
hsa_circ_	-0.21894	0.273506
hsa_circ_	-0.3212	0.273508
hsa_circ_	-0.27385	0.273524
hsa_circ_	-0.23223	0.273531
hsa_circ_	-0.23208	0.273531
hsa_circ_	0.370745	0.27354
hsa_circ_	0.147139	0.273571
hsa_circ_	0.176319	0.273576
hsa_circ_	-0.15988	0.273585
hsa_circ_	0.750443	0.273597
hsa_circ_	-0.1789	0.273624
hsa_circ_	-0.22399	0.273625
hsa_circ_	0.232283	0.27364
hsa_circ_	-0.18522	0.273657
hsa_circ_	0.196195	0.273662
hsa_circ_	-0.50131	0.273669
hsa_circ_	-0.24804	0.27367
hsa_circ_	0.190711	0.273687
hsa_circ_	-0.15378	0.273699
hsa_circ_	0.425522	0.2737
hsa_circ_	0.33366	0.2737
hsa_circ_	-0.43226	0.273703
hsa_circ_	-0.1652	0.273707
hsa_circ_	0.526098	0.273709
hsa_circ_	-0.41358	0.273711
hsa_circ_	0.252838	0.273731
hsa_circ_	0.187732	0.273746

hsa_circ_	0.32197	0.273747
hsa_circ_	0.243173	0.273755
hsa_circ_	0.424399	0.273756
hsa_circ_	-0.27475	0.273762
hsa_circ_	0.377649	0.273788
hsa_circ_	-0.24758	0.273788
hsa_circ_	-0.46638	0.27379
hsa_circ_	-0.43047	0.273802
hsa_circ_	0.209771	0.273818
hsa_circ_	-0.23415	0.27382
hsa_circ_	0.191025	0.273825
hsa_circ_	0.180054	0.273834
hsa_circ_	0.315446	0.273845
hsa_circ_	-0.15911	0.273848
hsa_circ_	-0.19083	0.273849
hsa_circ_	0.230081	0.273849
hsa_circ_	-0.17979	0.273873
hsa_circ_	-0.31169	0.273874
hsa_circ_	-0.19975	0.273875
hsa_circ_	0.513575	0.273895
hsa_circ_	0.189051	0.273908
hsa_circ_	-0.44783	0.273911
hsa_circ_	-0.28851	0.273916
hsa_circ_	-0.52461	0.273954
hsa_circ_	0.721906	0.273955
hsa_circ_	0.199308	0.27398
hsa_circ_	0.575983	0.273983
hsa_circ_	0.268879	0.273989
hsa_circ_	0.208943	0.273989
hsa_circ_	0.221513	0.273991
hsa_circ_	0.161886	0.274001
hsa_circ_	-0.34517	0.274009
hsa_circ_	-0.21514	0.274031
hsa_circ_	0.275205	0.274038
hsa_circ_	-0.53047	0.274048
hsa_circ_	-0.33059	0.274055
hsa_circ_	-0.19383	0.274071
hsa_circ_	0.175971	0.274074
hsa_circ_	0.392318	0.274098
hsa_circ_	0.266123	0.274115
hsa_circ_	0.284544	0.274115
hsa_circ_	0.2135	0.274141
hsa_circ_	0.236377	0.274145
hsa_circ_	-0.21984	0.274181
hsa_circ_	0.152918	0.274186
hsa_circ_	-0.54452	0.274194
hsa_circ_	0.513262	0.274195
hsa_circ_	0.247064	0.2742
hsa_circ_	-0.27113	0.274236
hsa_circ_	-0.30261	0.274242
hsa_circ_	0.286175	0.274249
hsa_circ_	-0.33296	0.274251
hsa_circ_	-0.21317	0.274262
hsa_circ_	0.228986	0.274281

hsa_circ_	-0.21597	0.274281
hsa_circ_	-0.59324	0.274282
hsa_circ_	-0.30359	0.274326
hsa_circ_	-0.54944	0.274338
hsa_circ_	-0.23215	0.274353
hsa_circ_	-0.23181	0.274356
hsa_circ_	-0.16949	0.274358
hsa_circ_	-0.34382	0.274361
hsa_circ_	-0.70993	0.274361
hsa_circ_	0.637852	0.274386
hsa_circ_	0.465598	0.274386
hsa_circ_	-0.19897	0.274398
hsa_circ_	-0.63085	0.2744
hsa_circ_	-0.17256	0.274412
hsa_circ_	-0.1932	0.274415
hsa_circ_	-0.15321	0.274442
hsa_circ_	-0.55857	0.274457
hsa_circ_	-0.23537	0.274462
hsa_circ_	-0.33996	0.274464
hsa_circ_	0.385173	0.274469
hsa_circ_	0.846924	0.27449
hsa_circ_	-0.26649	0.274519
hsa_circ_	-0.18705	0.274527
hsa_circ_	0.687262	0.274532
hsa_circ_	0.489214	0.274536
hsa_circ_	-0.23429	0.274551
hsa_circ_	-0.27769	0.27456
hsa_circ_	-0.39411	0.274561
hsa_circ_	0.179221	0.274562
hsa_circ_	1.350311	0.274565
hsa_circ_	-0.21966	0.274571
hsa_circ_	0.195269	0.274574
hsa_circ_	0.203761	0.2746
hsa_circ_	-0.15961	0.274615
hsa_circ_	0.166151	0.274663
hsa_circ_	0.227168	0.274664
hsa_circ_	-0.29667	0.274691
hsa_circ_	0.242599	0.274699
hsa_circ_	-0.52972	0.27471
hsa_circ_	-0.56624	0.274745
hsa_circ_	0.342742	0.274747
hsa_circ_	-0.18835	0.274752
hsa_circ_	-0.21693	0.274765
hsa_circ_	-0.24116	0.274795
hsa_circ_	-0.28117	0.274824
hsa_circ_	0.204791	0.274841
hsa_circ_	-0.17426	0.274856
hsa_circ_	0.916449	0.274863
hsa_circ_	-0.59375	0.274877
hsa_circ_	-0.41134	0.274882
hsa_circ_	0.369512	0.274905
hsa_circ_	0.554886	0.274921
hsa_circ_	0.273494	0.274934
hsa_circ_	-0.34331	0.274943

hsa_circ_	-0.59921	0.274957
hsa_circ_	-0.34141	0.27496
hsa_circ_	0.332755	0.274965
hsa_circ_	0.270473	0.274971
hsa_circ_	0.291904	0.27499
hsa_circ_	0.318637	0.274991
hsa_circ_	-0.26555	0.274994
hsa_circ_	-0.61808	0.274997
hsa_circ_	0.247219	0.275004
hsa_circ_	0.345068	0.275006
hsa_circ_	-0.58947	0.27501
hsa_circ_	-0.17616	0.275031
hsa_circ_	0.627973	0.275032
hsa_circ_	-0.77101	0.275049
hsa_circ_	0.205568	0.275054
hsa_circ_	0.215199	0.27506
hsa_circ_	0.305854	0.275068
hsa_circ_	-0.46435	0.275089
hsa_circ_	-0.15999	0.275096
hsa_circ_	0.166869	0.275109
hsa_circ_	0.158885	0.27511
hsa_circ_	-0.461	0.275121
hsa_circ_	0.326561	0.275142
hsa_circ_	0.213966	0.275143
hsa_circ_	-0.59521	0.275165
hsa_circ_	0.720936	0.275173
hsa_circ_	0.240328	0.275215
hsa_circ_	-0.21087	0.275223
hsa_circ_	-0.29462	0.275224
hsa_circ_	-0.18332	0.275278
hsa_circ_	-0.53287	0.275281
hsa_circ_	0.415002	0.275289
hsa_circ_	-0.15083	0.275296
hsa_circ_	-0.17356	0.275297
hsa_circ_	0.314573	0.27531
hsa_circ_	0.324888	0.275311
hsa_circ_	-0.16169	0.275315
hsa_circ_	1.604114	0.275317
hsa_circ_	-0.15543	0.275342
hsa_circ_	-0.17542	0.275384
hsa_circ_	0.204544	0.275391
hsa_circ_	0.245564	0.275405
hsa_circ_	-0.20751	0.275433
hsa_circ_	0.234324	0.275434
hsa_circ_	0.492959	0.275441
hsa_circ_	0.198097	0.275451
hsa_circ_	-0.29246	0.275452
hsa_circ_	-0.2796	0.275454
hsa_circ_	-0.51175	0.275459
hsa_circ_	0.181681	0.275466
hsa_circ_	-0.18463	0.27547
hsa_circ_	-0.22388	0.275474
hsa_circ_	0.2485	0.275488
hsa_circ_	0.495807	0.275489

hsa_circ_	-0.45327	0.275497
hsa_circ_	0.199914	0.27551
hsa_circ_	0.182798	0.275512
hsa_circ_	-0.58005	0.275513
hsa_circ_	-0.5921	0.275517
hsa_circ_	0.203903	0.275519
hsa_circ_	-0.45099	0.275532
hsa_circ_	-0.29075	0.275537
hsa_circ_	-0.19095	0.275549
hsa_circ_	0.184639	0.275566
hsa_circ_	0.478349	0.27558
hsa_circ_	0.190397	0.275591
hsa_circ_	-0.51139	0.275599
hsa_circ_	-0.91935	0.2756
hsa_circ_	0.169926	0.275604
hsa_circ_	-0.18252	0.275604
hsa_circ_	-0.22834	0.275613
hsa_circ_	-0.60971	0.275617
hsa_circ_	0.388547	0.27562
hsa_circ_	-0.16733	0.275625
hsa_circ_	0.163528	0.275631
hsa_circ_	0.459387	0.275632
hsa_circ_	-0.1699	0.275638
hsa_circ_	0.432896	0.275656
hsa_circ_	-0.62059	0.275662
hsa_circ_	0.230189	0.275667
hsa_circ_	0.216093	0.275678
hsa_circ_	0.185626	0.275684
hsa_circ_	-0.22592	0.27569
hsa_circ_	-0.42248	0.2757
hsa_circ_	0.249169	0.2757
hsa_circ_	0.257153	0.275723
hsa_circ_	-0.15142	0.275735
hsa_circ_	-0.2502	0.275736
hsa_circ_	0.175088	0.275737
hsa_circ_	0.303734	0.275739
hsa_circ_	0.185164	0.275758
hsa_circ_	-0.28865	0.27576
hsa_circ_	0.198018	0.275762
hsa_circ_	0.265336	0.275769
hsa_circ_	-0.23885	0.275773
hsa_circ_	-0.14853	0.275775
hsa_circ_	-0.30007	0.275778
hsa_circ_	-0.3503	0.275782
hsa_circ_	-1.08146	0.275786
hsa_circ_	-0.18012	0.275805
hsa_circ_	-0.68671	0.275815
hsa_circ_	0.358367	0.275828
hsa_circ_	0.246113	0.275836
hsa_circ_	-0.14706	0.275867
hsa_circ_	0.243572	0.27588
hsa_circ_	-0.1695	0.275884
hsa_circ_	0.192304	0.275884
hsa_circ_	-0.22273	0.275921

hsa_circ_	0.300999	0.275944
hsa_circ_	-0.15867	0.275945
hsa_circ_	-0.17061	0.275963
hsa_circ_	-1.38551	0.275965
hsa_circ_	-0.14381	0.275997
hsa_circ_	0.486289	0.276019
hsa_circ_	0.649158	0.27604
hsa_circ_	-0.17881	0.276052
hsa_circ_	0.252887	0.276088
hsa_circ_	0.227913	0.276128
hsa_circ_	0.167294	0.276139
hsa_circ_	0.159454	0.27614
hsa_circ_	-0.39029	0.276144
hsa_circ_	-0.53058	0.276152
hsa_circ_	0.396082	0.276159
hsa_circ_	-0.21768	0.276221
hsa_circ_	-0.22644	0.276233
hsa_circ_	-0.3143	0.276256
hsa_circ_	-0.15052	0.27627
hsa_circ_	-0.35479	0.276273
hsa_circ_	0.183884	0.27631
hsa_circ_	0.239247	0.276314
hsa_circ_	-0.17861	0.276321
hsa_circ_	-0.28834	0.276333
hsa_circ_	-0.2216	0.276339
hsa_circ_	-0.45005	0.276343
hsa_circ_	-0.25309	0.27635
hsa_circ_	0.408127	0.276352
hsa_circ_	0.338796	0.276356
hsa_circ_	-0.27217	0.276367
hsa_circ_	0.176377	0.276383
hsa_circ_	-0.1924	0.276386
hsa_circ_	-1.21315	0.276389
hsa_circ_	-0.15263	0.276406
hsa_circ_	-0.23533	0.27642
hsa_circ_	0.241434	0.276439
hsa_circ_	0.416437	0.276442
hsa_circ_	0.20547	0.276453
hsa_circ_	0.281655	0.276458
hsa_circ_	-0.37359	0.276471
hsa_circ_	-0.27434	0.276484
hsa_circ_	-0.27323	0.276496
hsa_circ_	0.368287	0.276497
hsa_circ_	-0.20576	0.276507
hsa_circ_	-0.29281	0.276509
hsa_circ_	0.200759	0.276531
hsa_circ_	-0.19551	0.276532
hsa_circ_	-0.25478	0.276537
hsa_circ_	-0.15239	0.276568
hsa_circ_	0.31698	0.276592
hsa_circ_	-0.53992	0.276592
hsa_circ_	-0.22784	0.276601
hsa_circ_	-0.26643	0.276602
hsa_circ_	-0.15872	0.276608

hsa_circ_	0.3391	0.276609
hsa_circ_	0.344409	0.276612
hsa_circ_	-0.14366	0.276617
hsa_circ_	0.192884	0.276627
hsa_circ_	0.300483	0.276641
hsa_circ_	0.826469	0.276676
hsa_circ_	0.151357	0.276681
hsa_circ_	0.208675	0.276716
hsa_circ_	-0.37565	0.276726
hsa_circ_	-0.2087	0.276749
hsa_circ_	0.263578	0.276755
hsa_circ_	0.166993	0.276765
hsa_circ_	-0.17984	0.276778
hsa_circ_	-0.1728	0.27678
hsa_circ_	0.709485	0.276787
hsa_circ_	0.243992	0.276803
hsa_circ_	-0.24301	0.276825
hsa_circ_	0.146681	0.276829
hsa_circ_	-0.46679	0.276846
hsa_circ_	-0.44914	0.276894
hsa_circ_	0.270196	0.276899
hsa_circ_	-0.14343	0.276917
hsa_circ_	0.221595	0.276928
hsa_circ_	0.348273	0.276935
hsa_circ_	0.214254	0.276938
hsa_circ_	0.160367	0.276941
hsa_circ_	-0.19146	0.276943
hsa_circ_	-0.47693	0.276987
hsa_circ_	0.268352	0.276991
hsa_circ_	-0.18677	0.277008
hsa_circ_	0.265432	0.277011
hsa_circ_	-0.26654	0.277025
hsa_circ_	0.216279	0.27703
hsa_circ_	0.169858	0.277037
hsa_circ_	0.197287	0.277048
hsa_circ_	-0.31843	0.27705
hsa_circ_	-0.17887	0.277058
hsa_circ_	0.183884	0.27706
hsa_circ_	-0.38294	0.277078
hsa_circ_	-0.14999	0.27708
hsa_circ_	-0.34796	0.277082
hsa_circ_	-0.17621	0.27709
hsa_circ_	-0.23216	0.27709
hsa_circ_	0.242298	0.277121
hsa_circ_	-0.19374	0.277129
hsa_circ_	0.187596	0.277162
hsa_circ_	-0.29673	0.277174
hsa_circ_	-0.28751	0.277177
hsa_circ_	0.307663	0.277198
hsa_circ_	0.175544	0.277201
hsa_circ_	-0.18707	0.277209
hsa_circ_	-0.21473	0.277222
hsa_circ_	0.166911	0.277226
hsa_circ_	-0.59084	0.27723

hsa_circ_	0.33083	0.27724
hsa_circ_	0.259143	0.27724
hsa_circ_	-0.48901	0.27726
hsa_circ_	0.326316	0.277271
hsa_circ_	-0.34	0.277272
hsa_circ_	-0.23989	0.277313
hsa_circ_	0.215651	0.277323
hsa_circ_	0.300225	0.277332
hsa_circ_	0.174894	0.277342
hsa_circ_	-0.20236	0.277358
hsa_circ_	0.549662	0.277361
hsa_circ_	0.214883	0.27737
hsa_circ_	0.195308	0.277373
hsa_circ_	-0.76396	0.277381
hsa_circ_	0.252402	0.277408
hsa_circ_	-0.63371	0.277417
hsa_circ_	0.467625	0.277432
hsa_circ_	0.270955	0.277448
hsa_circ_	-0.57066	0.277452
hsa_circ_	0.229088	0.277452
hsa_circ_	-0.20567	0.277461
hsa_circ_	0.895003	0.277463
hsa_circ_	0.273316	0.277482
hsa_circ_	-0.5028	0.277526
hsa_circ_	0.286806	0.277538
hsa_circ_	0.219217	0.277538
hsa_circ_	-0.27887	0.277542
hsa_circ_	-0.30007	0.277548
hsa_circ_	-0.21394	0.277556
hsa_circ_	0.281608	0.277572
hsa_circ_	0.210273	0.277575
hsa_circ_	0.227115	0.277584
hsa_circ_	-0.28819	0.277603
hsa_circ_	0.376894	0.277606
hsa_circ_	0.214628	0.277612
hsa_circ_	-0.21139	0.277622
hsa_circ_	0.279347	0.277647
hsa_circ_	-0.23626	0.277676
hsa_circ_	-0.17755	0.277691
hsa_circ_	-0.1828	0.277695
hsa_circ_	0.424861	0.277697
hsa_circ_	0.189981	0.277711
hsa_circ_	-0.20591	0.277721
hsa_circ_	0.191143	0.277729
hsa_circ_	-0.55323	0.277733
hsa_circ_	-0.22481	0.277769
hsa_circ_	0.172606	0.277782
hsa_circ_	-0.25995	0.27779
hsa_circ_	0.326722	0.277791
hsa_circ_	-0.25602	0.277793
hsa_circ_	-0.17848	0.277802
hsa_circ_	-0.16396	0.277818
hsa_circ_	0.223772	0.277826
hsa_circ_	0.634802	0.27784

hsa_circ_	-0.33516	0.277855
hsa_circ_	-0.60743	0.277859
hsa_circ_	-0.14525	0.277878
hsa_circ_	0.41862	0.277895
hsa_circ_	0.464503	0.277897
hsa_circ_	0.532813	0.277922
hsa_circ_	-0.24655	0.277945
hsa_circ_	-0.27415	0.277948
hsa_circ_	0.395857	0.277963
hsa_circ_	0.302579	0.277978
hsa_circ_	0.321149	0.277999
hsa_circ_	-0.15732	0.277999
hsa_circ_	-0.501	0.278011
hsa_circ_	-0.29366	0.278022
hsa_circ_	-0.36103	0.278027
hsa_circ_	0.480317	0.27803
hsa_circ_	0.215653	0.278038
hsa_circ_	-0.34433	0.278046
hsa_circ_	-0.25662	0.278061
hsa_circ_	-0.20859	0.278076
hsa_circ_	-0.3318	0.278085
hsa_circ_	-0.2625	0.278105
hsa_circ_	-0.19928	0.27812
hsa_circ_	0.696926	0.278123
hsa_circ_	-0.2128	0.278143
hsa_circ_	0.282491	0.278144
hsa_circ_	0.255746	0.278149
hsa_circ_	-0.87008	0.278163
hsa_circ_	0.445436	0.278192
hsa_circ_	0.36852	0.278202
hsa_circ_	0.462329	0.278205
hsa_circ_	-0.49717	0.278207
hsa_circ_	-0.2041	0.278209
hsa_circ_	-0.15815	0.278212
hsa_circ_	0.634979	0.278223
hsa_circ_	0.280752	0.278232
hsa_circ_	-1.66074	0.278248
hsa_circ_	-0.28933	0.278256
hsa_circ_	0.65643	0.278257
hsa_circ_	0.224139	0.278281
hsa_circ_	0.801358	0.278289
hsa_circ_	-0.46851	0.278306
hsa_circ_	-0.38004	0.278325
hsa_circ_	0.302326	0.278327
hsa_circ_	-0.16482	0.278331
hsa_circ_	-0.21184	0.278332
hsa_circ_	-0.17786	0.278335
hsa_circ_	0.949563	0.278351
hsa_circ_	0.259761	0.278369
hsa_circ_	0.21082	0.278381
hsa_circ_	0.422863	0.278392
hsa_circ_	0.231991	0.278393
hsa_circ_	-0.14909	0.278397
hsa_circ_	0.790005	0.278439

hsa_circ_	0.583548	0.278467
hsa_circ_	0.514459	0.278471
hsa_circ_	0.153574	0.278505
hsa_circ_	-0.19517	0.278506
hsa_circ_	0.190382	0.278514
hsa_circ_	0.288159	0.278526
hsa_circ_	-0.23672	0.278544
hsa_circ_	0.332326	0.278551
hsa_circ_	0.207541	0.278569
hsa_circ_	-0.16613	0.278595
hsa_circ_	-0.30958	0.278596
hsa_circ_	-0.31791	0.278599
hsa_circ_	0.178005	0.278611
hsa_circ_	-0.57675	0.278618
hsa_circ_	-0.48742	0.278634
hsa_circ_	-0.43039	0.278636
hsa_circ_	0.286593	0.278641
hsa_circ_	-0.20642	0.278647
hsa_circ_	-0.37455	0.27866
hsa_circ_	0.753559	0.278669
hsa_circ_	0.179507	0.278672
hsa_circ_	-0.28247	0.278679
hsa_circ_	-0.15437	0.278682
hsa_circ_	0.301844	0.278686
hsa_circ_	-0.32358	0.278702
hsa_circ_	0.250386	0.278708
hsa_circ_	-1.32257	0.278709
hsa_circ_	0.212651	0.278714
hsa_circ_	-0.61896	0.278722
hsa_circ_	-0.49996	0.278763
hsa_circ_	0.150034	0.278795
hsa_circ_	-0.22254	0.278811
hsa_circ_	0.22586	0.278812
hsa_circ_	-0.32203	0.278813
hsa_circ_	0.233698	0.278814
hsa_circ_	0.22588	0.278821
hsa_circ_	0.167677	0.278826
hsa_circ_	-0.22667	0.278828
hsa_circ_	0.389033	0.278829
hsa_circ_	-0.16859	0.278831
hsa_circ_	-0.24737	0.278842
hsa_circ_	-0.38252	0.278859
hsa_circ_	-0.17701	0.278882
hsa_circ_	0.268448	0.278893
hsa_circ_	-0.61247	0.278911
hsa_circ_	-0.36314	0.278933
hsa_circ_	0.177652	0.278935
hsa_circ_	-0.17724	0.278943
hsa_circ_	-0.2497	0.278953
hsa_circ_	-0.21493	0.278955
hsa_circ_	-0.22584	0.27897
hsa_circ_	-0.15446	0.278977
hsa_circ_	0.235533	0.278992
hsa_circ_	-0.81219	0.279003

hsa_circ_	0.739726	0.279003
hsa_circ_	0.485479	0.279015
hsa_circ_	0.700369	0.27902
hsa_circ_	0.285756	0.279036
hsa_circ_	-0.20628	0.279056
hsa_circ_	-0.48505	0.279065
hsa_circ_	-0.15462	0.27908
hsa_circ_	-0.58163	0.279087
hsa_circ_	0.245729	0.279094
hsa_circ_	0.210302	0.2791
hsa_circ_	-0.18698	0.279108
hsa_circ_	0.324123	0.279113
hsa_circ_	-0.70179	0.279115
hsa_circ_	-0.18515	0.279116
hsa_circ_	-0.37066	0.27912
hsa_circ_	0.368656	0.279143
hsa_circ_	0.250586	0.279151
hsa_circ_	-0.20302	0.279163
hsa_circ_	-0.2028	0.279163
hsa_circ_	-0.23246	0.279164
hsa_circ_	0.27334	0.279172
hsa_circ_	0.221046	0.27919
hsa_circ_	0.293035	0.279193
hsa_circ_	-0.69085	0.279228
hsa_circ_	-0.17615	0.279231
hsa_circ_	-0.19321	0.279243
hsa_circ_	-0.30439	0.279252
hsa_circ_	0.181076	0.279256
hsa_circ_	0.342264	0.279289
hsa_circ_	0.204239	0.279293
hsa_circ_	-0.14664	0.279306
hsa_circ_	-0.61305	0.27931
hsa_circ_	0.226637	0.27932
hsa_circ_	-0.15567	0.279323
hsa_circ_	0.675341	0.279341
hsa_circ_	-0.15444	0.279358
hsa_circ_	-0.23141	0.279363
hsa_circ_	0.524697	0.279365
hsa_circ_	0.262959	0.279365
hsa_circ_	0.230971	0.279403
hsa_circ_	0.250779	0.27941
hsa_circ_	0.620251	0.279428
hsa_circ_	0.630073	0.27943
hsa_circ_	-0.18292	0.279432
hsa_circ_	0.510501	0.27945
hsa_circ_	-0.2026	0.279451
hsa_circ_	-0.18583	0.279456
hsa_circ_	-0.18589	0.279497
hsa_circ_	-0.33983	0.279506
hsa_circ_	0.155403	0.279523
hsa_circ_	-0.65789	0.279525
hsa_circ_	-1.30117	0.279568
hsa_circ_	0.34784	0.279568
hsa_circ_	-0.94188	0.279573

hsa_circ_	-0.16461	0.279578
hsa_circ_	0.260218	0.279598
hsa_circ_	0.308513	0.279598
hsa_circ_	0.710843	0.279602
hsa_circ_	-0.2658	0.279632
hsa_circ_	-0.50705	0.279652
hsa_circ_	-0.55829	0.279668
hsa_circ_	0.258123	0.279693
hsa_circ_	-0.26196	0.279706
hsa_circ_	-0.15088	0.279709
hsa_circ_	0.198448	0.279721
hsa_circ_	-0.23383	0.279727
hsa_circ_	-0.2796	0.279736
hsa_circ_	-0.28339	0.279749
hsa_circ_	-0.24395	0.279758
hsa_circ_	-0.8478	0.279799
hsa_circ_	-0.1938	0.2798
hsa_circ_	-0.6805	0.279811
hsa_circ_	-0.37308	0.279813
hsa_circ_	-0.37116	0.279829
hsa_circ_	-0.16611	0.279833
hsa_circ_	0.27113	0.279851
hsa_circ_	0.258802	0.279859
hsa_circ_	-0.40323	0.27986
hsa_circ_	0.853333	0.279877
hsa_circ_	-0.21185	0.279879
hsa_circ_	-0.22218	0.279886
hsa_circ_	0.150357	0.279893
hsa_circ_	-0.77602	0.279897
hsa_circ_	0.200187	0.279898
hsa_circ_	0.23605	0.279927
hsa_circ_	-0.67234	0.279928
hsa_circ_	0.242554	0.279933
hsa_circ_	-0.54436	0.27997
hsa_circ_	0.320174	0.279979
hsa_circ_	0.253897	0.27998
hsa_circ_	0.250254	0.279987
hsa_circ_	-0.23631	0.27999
hsa_circ_	0.36567	0.28
hsa_circ_	-0.15173	0.280005
hsa_circ_	-0.18472	0.280028
hsa_circ_	-0.28273	0.280029
hsa_circ_	-0.15588	0.280063
hsa_circ_	0.324553	0.280068
hsa_circ_	-0.24698	0.280085
hsa_circ_	0.402763	0.280085
hsa_circ_	-0.1832	0.280104
hsa_circ_	-0.49301	0.28011
hsa_circ_	0.192015	0.280117
hsa_circ_	0.234833	0.280127
hsa_circ_	0.227333	0.280139
hsa_circ_	0.50871	0.280151
hsa_circ_	-0.16497	0.280161
hsa_circ_	0.15679	0.280165

hsa_circ_	0.18831	0.280174
hsa_circ_	0.380173	0.280181
hsa_circ_	0.178563	0.280186
hsa_circ_	-0.16849	0.280191
hsa_circ_	-0.67088	0.280197
hsa_circ_	-0.15505	0.280198
hsa_circ_	-0.34348	0.280218
hsa_circ_	0.714817	0.280226
hsa_circ_	-0.15779	0.280254
hsa_circ_	0.452904	0.280264
hsa_circ_	-0.31559	0.280274
hsa_circ_	-0.30825	0.280286
hsa_circ_	-1.04171	0.280299
hsa_circ_	0.203576	0.280318
hsa_circ_	0.190081	0.280319
hsa_circ_	0.305341	0.280323
hsa_circ_	-0.16613	0.280328
hsa_circ_	-0.2951	0.280334
hsa_circ_	0.185065	0.280346
hsa_circ_	0.230716	0.280354
hsa_circ_	0.236984	0.28041
hsa_circ_	-0.18591	0.280414
hsa_circ_	0.165709	0.280424
hsa_circ_	0.167095	0.280429
hsa_circ_	0.213442	0.280431
hsa_circ_	-0.19032	0.280437
hsa_circ_	-0.2166	0.280458
hsa_circ_	-0.18384	0.280472
hsa_circ_	-0.15877	0.280474
hsa_circ_	0.214412	0.280482
hsa_circ_	-0.60777	0.280483
hsa_circ_	0.179337	0.280492
hsa_circ_	-0.15419	0.280518
hsa_circ_	0.169993	0.280527
hsa_circ_	-0.18748	0.280551
hsa_circ_	0.232141	0.280552
hsa_circ_	0.352333	0.280557
hsa_circ_	-0.19974	0.280566
hsa_circ_	-0.15957	0.280567
hsa_circ_	0.203732	0.280572
hsa_circ_	-0.2051	0.28058
hsa_circ_	0.322559	0.280584
hsa_circ_	0.215061	0.280587
hsa_circ_	-0.23418	0.280607
hsa_circ_	-0.29053	0.280612
hsa_circ_	0.354529	0.280618
hsa_circ_	-0.24816	0.280619
hsa_circ_	-0.39429	0.280621
hsa_circ_	-0.22245	0.280631
hsa_circ_	0.195382	0.280635
hsa_circ_	0.162965	0.280641
hsa_circ_	-0.71268	0.280647
hsa_circ_	0.386779	0.280655
hsa_circ_	-0.27222	0.280662

hsa_circ_	0.520834	0.280674
hsa_circ_	0.169897	0.280678
hsa_circ_	0.183312	0.280682
hsa_circ_	-0.2381	0.280732
hsa_circ_	-0.17972	0.280739
hsa_circ_	-0.17932	0.280752
hsa_circ_	0.430172	0.280759
hsa_circ_	0.334974	0.280798
hsa_circ_	0.158666	0.280805
hsa_circ_	-0.2526	0.280809
hsa_circ_	0.194926	0.28082
hsa_circ_	0.586908	0.280836
hsa_circ_	0.189568	0.280863
hsa_circ_	0.32566	0.280879
hsa_circ_	0.245713	0.280883
hsa_circ_	0.15929	0.280885
hsa_circ_	0.589146	0.280901
hsa_circ_	-0.17301	0.280906
hsa_circ_	0.150737	0.280912
hsa_circ_	0.678817	0.280916
hsa_circ_	-0.60358	0.280917
hsa_circ_	-0.19327	0.280931
hsa_circ_	-0.23205	0.280955
hsa_circ_	0.640275	0.280983
hsa_circ_	0.323425	0.280984
hsa_circ_	-0.54103	0.280998
hsa_circ_	-0.50702	0.281006
hsa_circ_	-0.54764	0.281008
hsa_circ_	0.212105	0.28102
hsa_circ_	-0.3814	0.281028
hsa_circ_	-0.24824	0.281028
hsa_circ_	-0.20029	0.281049
hsa_circ_	-0.23626	0.281056
hsa_circ_	0.245543	0.281073
hsa_circ_	-0.19066	0.28109
hsa_circ_	-0.22974	0.281093
hsa_circ_	0.285732	0.28111
hsa_circ_	-0.60634	0.28111
hsa_circ_	0.254336	0.28112
hsa_circ_	-0.42668	0.281126
hsa_circ_	-0.2485	0.28114
hsa_circ_	0.2993	0.281158
hsa_circ_	-0.20812	0.281163
hsa_circ_	-0.61069	0.281172
hsa_circ_	-0.98883	0.281173
hsa_circ_	0.168183	0.281175
hsa_circ_	-0.30137	0.281178
hsa_circ_	-0.43819	0.281205
hsa_circ_	0.286413	0.281208
hsa_circ_	0.366962	0.281231
hsa_circ_	-0.20201	0.281243
hsa_circ_	0.187353	0.281264
hsa_circ_	0.296376	0.281272
hsa_circ_	-0.30741	0.281276

hsa_circ_	0.148932	0.281284
hsa_circ_	0.224095	0.281311
hsa_circ_	-0.17496	0.281314
hsa_circ_	-0.16727	0.281315
hsa_circ_	0.251068	0.28132
hsa_circ_	-0.16977	0.281321
hsa_circ_	0.240387	0.281327
hsa_circ_	0.242649	0.281333
hsa_circ_	-0.23166	0.281338
hsa_circ_	0.272489	0.281351
hsa_circ_	0.238742	0.281364
hsa_circ_	-0.21167	0.281374
hsa_circ_	-0.36912	0.28138
hsa_circ_	0.70827	0.281386
hsa_circ_	-0.22845	0.281408
hsa_circ_	0.234627	0.281421
hsa_circ_	-0.41136	0.281441
hsa_circ_	-0.18161	0.281451
hsa_circ_	-0.63891	0.281463
hsa_circ_	-0.16881	0.28149
hsa_circ_	-0.16094	0.281492
hsa_circ_	-0.15893	0.281493
hsa_circ_	0.176685	0.281502
hsa_circ_	-0.31967	0.281504
hsa_circ_	-0.35722	0.281518
hsa_circ_	0.56232	0.28153
hsa_circ_	0.361958	0.281573
hsa_circ_	0.304026	0.281575
hsa_circ_	0.745821	0.281589
hsa_circ_	0.418327	0.281626
hsa_circ_	0.200984	0.281634
hsa_circ_	0.311483	0.281639
hsa_circ_	-0.44573	0.281644
hsa_circ_	-0.22065	0.281652
hsa_circ_	-0.21285	0.281659
hsa_circ_	-0.19198	0.281665
hsa_circ_	0.237949	0.281687
hsa_circ_	-0.65046	0.281687
hsa_circ_	0.28588	0.281691
hsa_circ_	-0.81112	0.281694
hsa_circ_	0.247737	0.28171
hsa_circ_	-0.27231	0.281718
hsa_circ_	-0.40254	0.281797
hsa_circ_	0.2196	0.281852
hsa_circ_	0.382046	0.281856
hsa_circ_	0.16629	0.281866
hsa_circ_	0.254624	0.281882
hsa_circ_	-0.21163	0.281898
hsa_circ_	0.153297	0.281898
hsa_circ_	-0.50409	0.2819
hsa_circ_	0.160676	0.281905
hsa_circ_	-0.2013	0.281912
hsa_circ_	0.304056	0.281922
hsa_circ_	0.242315	0.281926

hsa_circ_	0.168625	0.281932
hsa_circ_	-0.20336	0.281932
hsa_circ_	0.267792	0.281934
hsa_circ_	-0.56405	0.281936
hsa_circ_	0.422712	0.281942
hsa_circ_	0.277165	0.281946
hsa_circ_	0.484702	0.281947
hsa_circ_	-0.19004	0.281957
hsa_circ_	-0.35303	0.281958
hsa_circ_	0.420735	0.281961
hsa_circ_	0.150217	0.282016
hsa_circ_	0.189933	0.282018
hsa_circ_	-0.36925	0.282021
hsa_circ_	-0.2109	0.282024
hsa_circ_	0.230203	0.282026
hsa_circ_	0.306485	0.282061
hsa_circ_	0.209631	0.282063
hsa_circ_	-0.19305	0.282063
hsa_circ_	0.409116	0.282071
hsa_circ_	0.160977	0.282076
hsa_circ_	0.166576	0.282083
hsa_circ_	-0.24079	0.282086
hsa_circ_	0.172542	0.282089
hsa_circ_	0.193938	0.282096
hsa_circ_	0.430758	0.282122
hsa_circ_	0.183488	0.282122
hsa_circ_	0.451235	0.28213
hsa_circ_	-0.64119	0.282136
hsa_circ_	0.327761	0.282141
hsa_circ_	0.336217	0.282143
hsa_circ_	-0.36523	0.282144
hsa_circ_	0.449421	0.282146
hsa_circ_	0.410099	0.282153
hsa_circ_	0.175875	0.282155
hsa_circ_	-0.23538	0.28216
hsa_circ_	-0.44819	0.282168
hsa_circ_	0.253985	0.28217
hsa_circ_	-0.24641	0.282179
hsa_circ_	-0.35398	0.282219
hsa_circ_	0.353098	0.28222
hsa_circ_	-0.16668	0.282244
hsa_circ_	-0.15517	0.28232
hsa_circ_	-0.1753	0.282324
hsa_circ_	0.199636	0.282326
hsa_circ_	-0.45711	0.28233
hsa_circ_	-0.37553	0.282336
hsa_circ_	-0.21699	0.282343
hsa_circ_	-0.17369	0.282372
hsa_circ_	0.330622	0.28239
hsa_circ_	0.61341	0.28239
hsa_circ_	-0.20187	0.282443
hsa_circ_	-0.31588	0.282456
hsa_circ_	-0.18672	0.282473
hsa_circ_	0.35235	0.28249

hsa_circ_	-0.80654	0.282492
hsa_circ_	0.206569	0.282493
hsa_circ_	-0.65197	0.282494
hsa_circ_	0.286774	0.282496
hsa_circ_	-0.16517	0.282499
hsa_circ_	0.268767	0.282504
hsa_circ_	-0.25413	0.282506
hsa_circ_	0.198203	0.282508
hsa_circ_	-0.23726	0.282525
hsa_circ_	0.198676	0.282526
hsa_circ_	-0.19691	0.282545
hsa_circ_	-0.21894	0.282547
hsa_circ_	-0.28883	0.282559
hsa_circ_	0.596336	0.282578
hsa_circ_	-0.17058	0.28258
hsa_circ_	-0.21038	0.282604
hsa_circ_	-0.20488	0.28261
hsa_circ_	0.577573	0.282611
hsa_circ_	-0.28903	0.282625
hsa_circ_	-0.26923	0.28266
hsa_circ_	0.243841	0.282664
hsa_circ_	-0.77522	0.282671
hsa_circ_	0.19284	0.282696
hsa_circ_	0.163926	0.282698
hsa_circ_	-0.21982	0.282722
hsa_circ_	-0.82458	0.282732
hsa_circ_	0.64567	0.282765
hsa_circ_	0.155148	0.282771
hsa_circ_	0.390225	0.282783
hsa_circ_	-0.23615	0.282802
hsa_circ_	-0.3691	0.282808
hsa_circ_	-0.18913	0.282818
hsa_circ_	0.387736	0.282825
hsa_circ_	-0.1459	0.282832
hsa_circ_	0.179268	0.282834
hsa_circ_	-0.22006	0.282836
hsa_circ_	-0.66311	0.282858
hsa_circ_	0.419366	0.282859
hsa_circ_	0.218265	0.282869
hsa_circ_	-0.25661	0.282876
hsa_circ_	-0.35351	0.282879
hsa_circ_	-0.17718	0.282895
hsa_circ_	0.154573	0.282915
hsa_circ_	-0.24974	0.28293
hsa_circ_	-0.53284	0.282944
hsa_circ_	0.18149	0.28295
hsa_circ_	0.218358	0.282958
hsa_circ_	0.260019	0.282976
hsa_circ_	0.279698	0.283001
hsa_circ_	-0.25535	0.283001
hsa_circ_	0.234105	0.283005
hsa_circ_	-0.29285	0.283009
hsa_circ_	0.447691	0.283024
hsa_circ_	0.164787	0.283039

hsa_circ_	-0.17094	0.283061
hsa_circ_	0.185239	0.283077
hsa_circ_	0.253948	0.283078
hsa_circ_	0.211691	0.283091
hsa_circ_	0.206596	0.283104
hsa_circ_	0.226147	0.283107
hsa_circ_	0.176471	0.283156
hsa_circ_	0.199041	0.283158
hsa_circ_	0.191691	0.283175
hsa_circ_	-0.18187	0.283184
hsa_circ_	0.323522	0.283186
hsa_circ_	0.456046	0.283206
hsa_circ_	0.198771	0.283229
hsa_circ_	0.250209	0.28323
hsa_circ_	0.288706	0.283241
hsa_circ_	-1.24521	0.283249
hsa_circ_	0.732857	0.283262
hsa_circ_	0.922084	0.283271
hsa_circ_	-0.26539	0.283313
hsa_circ_	-0.14851	0.283319
hsa_circ_	-0.27258	0.283324
hsa_circ_	-0.281	0.283328
hsa_circ_	0.159148	0.283331
hsa_circ_	-0.22424	0.283333
hsa_circ_	0.160596	0.283433
hsa_circ_	0.23155	0.283433
hsa_circ_	0.369063	0.283434
hsa_circ_	-0.27273	0.28344
hsa_circ_	0.822029	0.283451
hsa_circ_	-0.68432	0.283462
hsa_circ_	0.200653	0.283479
hsa_circ_	-0.53269	0.283491
hsa_circ_	0.182663	0.283502
hsa_circ_	0.168817	0.28357
hsa_circ_	-0.34493	0.28359
hsa_circ_	0.440549	0.283613
hsa_circ_	-0.36602	0.283614
hsa_circ_	0.289641	0.283708
hsa_circ_	0.178564	0.283715
hsa_circ_	-0.14927	0.283725
hsa_circ_	0.247373	0.283731
hsa_circ_	0.218468	0.283751
hsa_circ_	0.276659	0.283763
hsa_circ_	-0.40591	0.283776
hsa_circ_	0.214062	0.283781
hsa_circ_	0.270634	0.283839
hsa_circ_	0.159059	0.283841
hsa_circ_	-0.39331	0.283842
hsa_circ_	-0.14879	0.283844
hsa_circ_	0.553332	0.283852
hsa_circ_	0.29428	0.283857
hsa_circ_	0.219589	0.283861
hsa_circ_	0.561233	0.283895
hsa_circ_	-0.42909	0.283896

hsa_circ_	-0.23724	0.283922
hsa_circ_	-0.21877	0.283927
hsa_circ_	0.184345	0.283928
hsa_circ_	-0.27601	0.283955
hsa_circ_	-0.35112	0.283956
hsa_circ_	-0.23594	0.28396
hsa_circ_	-0.24443	0.283993
hsa_circ_	0.247856	0.283999
hsa_circ_	0.354115	0.284009
hsa_circ_	-0.28153	0.284024
hsa_circ_	-0.44088	0.284033
hsa_circ_	-0.24333	0.284036
hsa_circ_	0.35025	0.284039
hsa_circ_	0.166364	0.284045
hsa_circ_	0.278222	0.28407
hsa_circ_	0.642931	0.284079
hsa_circ_	-0.14289	0.284081
hsa_circ_	-0.18001	0.284092
hsa_circ_	0.372103	0.284093
hsa_circ_	-0.42404	0.284096
hsa_circ_	-0.23674	0.284103
hsa_circ_	0.160347	0.284105
hsa_circ_	-0.22343	0.284107
hsa_circ_	0.443017	0.284132
hsa_circ_	-0.42482	0.284133
hsa_circ_	-1.36516	0.284136
hsa_circ_	-0.15895	0.284137
hsa_circ_	-0.15985	0.284142
hsa_circ_	0.181011	0.284142
hsa_circ_	-0.14944	0.284156
hsa_circ_	0.162409	0.284156
hsa_circ_	-0.19565	0.284159
hsa_circ_	0.380915	0.28417
hsa_circ_	-0.20151	0.284182
hsa_circ_	-0.57926	0.284195
hsa_circ_	-0.19205	0.284212
hsa_circ_	-0.29619	0.284232
hsa_circ_	-0.1723	0.284236
hsa_circ_	0.296169	0.28424
hsa_circ_	-0.18962	0.284274
hsa_circ_	-0.22888	0.284283
hsa_circ_	-0.17714	0.2843
hsa_circ_	-0.40347	0.284313
hsa_circ_	-0.20081	0.284322
hsa_circ_	-0.1777	0.284324
hsa_circ_	-0.20515	0.284331
hsa_circ_	0.152969	0.284331
hsa_circ_	-0.20642	0.284344
hsa_circ_	0.197001	0.28435
hsa_circ_	-0.67176	0.284358
hsa_circ_	-0.47022	0.284366
hsa_circ_	-0.18229	0.284369
hsa_circ_	0.280841	0.284373
hsa_circ_	0.165622	0.284389

hsa_circ_	0.221289	0.284391
hsa_circ_	0.21791	0.284396
hsa_circ_	-0.33117	0.284403
hsa_circ_	0.584686	0.284425
hsa_circ_	-0.27782	0.284476
hsa_circ_	0.217324	0.284499
hsa_circ_	-0.37441	0.2845
hsa_circ_	-0.35752	0.284507
hsa_circ_	0.224414	0.284507
hsa_circ_	-0.60653	0.284547
hsa_circ_	0.453927	0.28455
hsa_circ_	-0.42859	0.284557
hsa_circ_	-0.19986	0.284585
hsa_circ_	-0.35373	0.284591
hsa_circ_	0.164637	0.284607
hsa_circ_	-0.16941	0.284613
hsa_circ_	-0.15848	0.284619
hsa_circ_	-0.36214	0.284621
hsa_circ_	0.291744	0.284636
hsa_circ_	0.297224	0.284638
hsa_circ_	0.260991	0.284645
hsa_circ_	0.179682	0.284653
hsa_circ_	0.266883	0.284662
hsa_circ_	-0.27645	0.284668
hsa_circ_	-0.15967	0.284677
hsa_circ_	-0.65347	0.284691
hsa_circ_	0.553201	0.284694
hsa_circ_	0.198954	0.284719
hsa_circ_	-0.29855	0.284721
hsa_circ_	0.472315	0.28473
hsa_circ_	-0.25558	0.284765
hsa_circ_	-0.19819	0.284781
hsa_circ_	0.28845	0.284783
hsa_circ_	0.671101	0.284791
hsa_circ_	0.341781	0.284797
hsa_circ_	0.159619	0.2848
hsa_circ_	-0.66341	0.284805
hsa_circ_	-0.39642	0.284807
hsa_circ_	0.196549	0.284816
hsa_circ_	-0.19653	0.284819
hsa_circ_	-0.47551	0.284836
hsa_circ_	0.261933	0.284859
hsa_circ_	-0.80819	0.284862
hsa_circ_	0.242028	0.284895
hsa_circ_	1.040419	0.284897
hsa_circ_	0.194451	0.284902
hsa_circ_	-0.17499	0.284909
hsa_circ_	-0.15631	0.284909
hsa_circ_	-0.17062	0.284924
hsa_circ_	0.167047	0.284926
hsa_circ_	-0.20548	0.284927
hsa_circ_	0.301681	0.284935
hsa_circ_	0.632355	0.284947
hsa_circ_	-0.31482	0.284961

hsa_circ_	-0.68071	0.284972
hsa_circ_	0.239982	0.284973
hsa_circ_	-0.30739	0.284978
hsa_circ_	0.260116	0.284993
hsa_circ_	0.280462	0.285008
hsa_circ_	-0.2136	0.285016
hsa_circ_	-0.1583	0.285018
hsa_circ_	-0.16312	0.285037
hsa_circ_	0.50449	0.285041
hsa_circ_	-0.35481	0.285044
hsa_circ_	0.516556	0.285054
hsa_circ_	0.325383	0.285056
hsa_circ_	0.184626	0.285057
hsa_circ_	0.21297	0.285073
hsa_circ_	0.23261	0.285073
hsa_circ_	0.192721	0.285079
hsa_circ_	-0.17691	0.285081
hsa_circ_	0.162546	0.285116
hsa_circ_	-0.21613	0.285128
hsa_circ_	0.15523	0.285135
hsa_circ_	0.258184	0.285149
hsa_circ_	-0.36122	0.285157
hsa_circ_	-0.37763	0.285177
hsa_circ_	0.172441	0.285184
hsa_circ_	-0.64953	0.285185
hsa_circ_	-0.20157	0.285195
hsa_circ_	0.170509	0.285198
hsa_circ_	0.196947	0.285198
hsa_circ_	-0.2722	0.285214
hsa_circ_	-0.20499	0.285214
hsa_circ_	0.259786	0.285215
hsa_circ_	0.222238	0.285219
hsa_circ_	0.157286	0.285222
hsa_circ_	-0.14692	0.285238
hsa_circ_	0.325586	0.285238
hsa_circ_	0.565416	0.285243
hsa_circ_	-0.19492	0.285246
hsa_circ_	-0.18374	0.285257
hsa_circ_	-0.37985	0.285259
hsa_circ_	0.27891	0.285281
hsa_circ_	-0.20681	0.285299
hsa_circ_	-0.37798	0.285322
hsa_circ_	-0.17687	0.285334
hsa_circ_	-0.42333	0.285336
hsa_circ_	0.224216	0.285336
hsa_circ_	-0.3604	0.285391
hsa_circ_	-0.25273	0.285398
hsa_circ_	-0.46354	0.285399
hsa_circ_	0.23859	0.285432
hsa_circ_	0.258367	0.285447
hsa_circ_	-0.19176	0.28546
hsa_circ_	0.24849	0.285469
hsa_circ_	0.330533	0.285479
hsa_circ_	-0.24471	0.285484

hsa_circ_	0.348998	0.285485
hsa_circ_	-0.42715	0.285487
hsa_circ_	-0.32759	0.285491
hsa_circ_	0.478418	0.285518
hsa_circ_	0.158866	0.285552
hsa_circ_	-0.30883	0.285553
hsa_circ_	-0.21605	0.285567
hsa_circ_	-0.21434	0.285569
hsa_circ_	0.312984	0.28558
hsa_circ_	-0.46362	0.285586
hsa_circ_	0.209841	0.285597
hsa_circ_	-0.46828	0.28561
hsa_circ_	-0.20216	0.285631
hsa_circ_	-0.18356	0.285649
hsa_circ_	0.218821	0.28565
hsa_circ_	-0.42362	0.285659
hsa_circ_	0.296178	0.285661
hsa_circ_	-0.95308	0.285663
hsa_circ_	0.219378	0.285666
hsa_circ_	-0.44616	0.285719
hsa_circ_	-0.17436	0.28572
hsa_circ_	0.29586	0.285726
hsa_circ_	0.377829	0.285729
hsa_circ_	-0.25009	0.28573
hsa_circ_	-0.15136	0.285735
hsa_circ_	-0.46671	0.285737
hsa_circ_	0.156248	0.285748
hsa_circ_	0.176735	0.285775
hsa_circ_	0.181057	0.285779
hsa_circ_	0.163166	0.285789
hsa_circ_	-0.22636	0.285793
hsa_circ_	0.34528	0.285809
hsa_circ_	0.611273	0.28584
hsa_circ_	0.330778	0.285844
hsa_circ_	0.16455	0.285848
hsa_circ_	-0.26847	0.285862
hsa_circ_	-0.82502	0.285864
hsa_circ_	-0.18356	0.285867
hsa_circ_	0.183645	0.285877
hsa_circ_	-0.20038	0.285881
hsa_circ_	0.277796	0.285887
hsa_circ_	-0.41411	0.285906
hsa_circ_	-0.21416	0.285909
hsa_circ_	-0.18992	0.285912
hsa_circ_	-0.36061	0.285913
hsa_circ_	0.385723	0.285914
hsa_circ_	-0.26733	0.285915
hsa_circ_	0.219227	0.285923
hsa_circ_	-0.48284	0.285929
hsa_circ_	-0.29486	0.285934
hsa_circ_	-0.18503	0.285963
hsa_circ_	-0.17256	0.285965
hsa_circ_	-0.17949	0.285977
hsa_circ_	0.175777	0.285985

hsa_circ_	0.258977	0.286004
hsa_circ_	-0.18722	0.286004
hsa_circ_	-0.2129	0.286017
hsa_circ_	0.389421	0.28603
hsa_circ_	-0.16899	0.286053
hsa_circ_	-0.51686	0.286059
hsa_circ_	0.293193	0.286075
hsa_circ_	0.288113	0.286094
hsa_circ_	0.242496	0.286099
hsa_circ_	0.438695	0.286119
hsa_circ_	-0.1573	0.286144
hsa_circ_	0.349796	0.286152
hsa_circ_	0.159637	0.286155
hsa_circ_	0.324119	0.286159
hsa_circ_	-0.18694	0.286211
hsa_circ_	-0.27706	0.286214
hsa_circ_	0.300025	0.286215
hsa_circ_	0.406394	0.286216
hsa_circ_	0.153783	0.286221
hsa_circ_	-0.35082	0.286247
hsa_circ_	-0.28799	0.286252
hsa_circ_	0.170393	0.286263
hsa_circ_	0.226846	0.286272
hsa_circ_	0.170811	0.286275
hsa_circ_	-0.16428	0.286279
hsa_circ_	-0.26926	0.286279
hsa_circ_	-0.15419	0.286313
hsa_circ_	-0.52258	0.286319
hsa_circ_	-0.15611	0.286324
hsa_circ_	-0.19712	0.286324
hsa_circ_	0.174577	0.286341
hsa_circ_	-0.27737	0.286358
hsa_circ_	0.542251	0.286389
hsa_circ_	-0.37247	0.286392
hsa_circ_	-0.35283	0.286403
hsa_circ_	0.215613	0.286429
hsa_circ_	-0.17214	0.286435
hsa_circ_	0.30599	0.286446
hsa_circ_	1.255685	0.286448
hsa_circ_	0.571702	0.286449
hsa_circ_	-0.44628	0.286454
hsa_circ_	-0.15711	0.286462
hsa_circ_	0.202679	0.286468
hsa_circ_	-0.32129	0.286473
hsa_circ_	-0.61459	0.28648
hsa_circ_	0.174309	0.2865
hsa_circ_	0.176036	0.286504
hsa_circ_	0.158055	0.286526
hsa_circ_	-0.18707	0.286536
hsa_circ_	-0.1753	0.286548
hsa_circ_	-0.26947	0.286554
hsa_circ_	-0.29682	0.286569
hsa_circ_	-0.19518	0.286573
hsa_circ_	0.264872	0.286589

hsa_circ_	-0.48814	0.286592
hsa_circ_	0.187336	0.286653
hsa_circ_	0.210643	0.286658
hsa_circ_	0.511261	0.286658
hsa_circ_	0.270751	0.286665
hsa_circ_	-0.19611	0.286667
hsa_circ_	-0.25492	0.28667
hsa_circ_	0.359656	0.286678
hsa_circ_	0.417998	0.286682
hsa_circ_	-0.19454	0.286683
hsa_circ_	-0.21918	0.286691
hsa_circ_	0.148646	0.286703
hsa_circ_	0.26168	0.286704
hsa_circ_	-0.21913	0.286705
hsa_circ_	0.635363	0.286716
hsa_circ_	-0.8069	0.286719
hsa_circ_	0.495172	0.286727
hsa_circ_	-0.28954	0.286729
hsa_circ_	-1.43593	0.286738
hsa_circ_	0.16552	0.286751
hsa_circ_	0.154316	0.286765
hsa_circ_	0.18298	0.286794
hsa_circ_	-0.26512	0.286825
hsa_circ_	-0.19869	0.286838
hsa_circ_	0.363957	0.28685
hsa_circ_	0.238592	0.286862
hsa_circ_	0.27153	0.286867
hsa_circ_	0.246173	0.286899
hsa_circ_	-0.50481	0.286904
hsa_circ_	0.463395	0.286911
hsa_circ_	0.499435	0.286941
hsa_circ_	-0.69436	0.286954
hsa_circ_	0.159063	0.286966
hsa_circ_	-0.2015	0.286982
hsa_circ_	-0.3141	0.28703
hsa_circ_	-0.1893	0.287046
hsa_circ_	0.224676	0.287055
hsa_circ_	-0.49255	0.287074
hsa_circ_	-0.15671	0.287082
hsa_circ_	0.9263	0.287093
hsa_circ_	0.196708	0.287148
hsa_circ_	0.191367	0.287173
hsa_circ_	0.220917	0.287184
hsa_circ_	-0.17437	0.287194
hsa_circ_	0.24398	0.287211
hsa_circ_	0.174674	0.287227
hsa_circ_	-0.37591	0.287247
hsa_circ_	0.19592	0.287263
hsa_circ_	-0.14898	0.287265
hsa_circ_	-0.46981	0.287295
hsa_circ_	-0.27729	0.287312
hsa_circ_	-0.22203	0.287326
hsa_circ_	-0.27612	0.287326
hsa_circ_	-0.22003	0.287332

hsa_circ_	0.532671	0.287337
hsa_circ_	0.165753	0.287354
hsa_circ_	0.145077	0.287365
hsa_circ_	-0.15467	0.287374
hsa_circ_	0.296161	0.287399
hsa_circ_	-0.15735	0.28741
hsa_circ_	-0.34746	0.287417
hsa_circ_	-0.15539	0.287421
hsa_circ_	-0.18473	0.287432
hsa_circ_	-0.14414	0.287434
hsa_circ_	-0.34386	0.287439
hsa_circ_	0.161054	0.287455
hsa_circ_	-0.23932	0.287491
hsa_circ_	-0.18547	0.287496
hsa_circ_	0.730411	0.287521
hsa_circ_	-0.27793	0.28753
hsa_circ_	-0.3336	0.287564
hsa_circ_	0.210158	0.287572
hsa_circ_	-0.23451	0.287572
hsa_circ_	0.369159	0.28758
hsa_circ_	-0.25638	0.287587
hsa_circ_	0.182997	0.28759
hsa_circ_	-0.20224	0.2876
hsa_circ_	0.238625	0.287618
hsa_circ_	1.797702	0.28763
hsa_circ_	-0.28068	0.28763
hsa_circ_	-0.3132	0.287631
hsa_circ_	0.817325	0.287664
hsa_circ_	-0.23549	0.287685
hsa_circ_	0.352158	0.287736
hsa_circ_	0.333219	0.287743
hsa_circ_	0.429638	0.287757
hsa_circ_	-0.50186	0.287759
hsa_circ_	-0.17003	0.287759
hsa_circ_	-0.15474	0.287763
hsa_circ_	-0.62932	0.287776
hsa_circ_	-0.17314	0.287806
hsa_circ_	0.18298	0.287823
hsa_circ_	-0.16365	0.287826
hsa_circ_	0.457584	0.287841
hsa_circ_	0.145037	0.287848
hsa_circ_	-0.16841	0.287855
hsa_circ_	0.262982	0.287888
hsa_circ_	-0.27817	0.287893
hsa_circ_	-0.66916	0.287917
hsa_circ_	-0.20531	0.287934
hsa_circ_	-0.56124	0.287946
hsa_circ_	0.231764	0.287954
hsa_circ_	-0.3468	0.287967
hsa_circ_	-0.44507	0.287983
hsa_circ_	-0.56756	0.287988
hsa_circ_	-0.32505	0.287992
hsa_circ_	0.185685	0.288001
hsa_circ_	0.215374	0.288019

hsa_circ_	-0.93139	0.288019
hsa_circ_	0.148725	0.288028
hsa_circ_	0.329874	0.288033
hsa_circ_	0.35433	0.288049
hsa_circ_	-0.15737	0.288056
hsa_circ_	0.264866	0.288058
hsa_circ_	0.236316	0.288069
hsa_circ_	-0.17559	0.288083
hsa_circ_	0.253804	0.288091
hsa_circ_	-0.21474	0.288102
hsa_circ_	-0.49156	0.288111
hsa_circ_	-0.15041	0.288117
hsa_circ_	0.18821	0.288122
hsa_circ_	-0.17742	0.288139
hsa_circ_	-0.14806	0.288174
hsa_circ_	0.247391	0.28818
hsa_circ_	0.547104	0.288198
hsa_circ_	0.173619	0.288218
hsa_circ_	0.310623	0.288244
hsa_circ_	-0.22904	0.288244
hsa_circ_	0.211367	0.288249
hsa_circ_	0.35301	0.288258
hsa_circ_	0.335521	0.28827
hsa_circ_	0.315884	0.288277
hsa_circ_	-0.32952	0.288278
hsa_circ_	0.41714	0.288284
hsa_circ_	0.233714	0.288284
hsa_circ_	0.181444	0.288291
hsa_circ_	0.509242	0.288297
hsa_circ_	0.292198	0.288334
hsa_circ_	-0.66556	0.288338
hsa_circ_	0.410359	0.288366
hsa_circ_	0.18144	0.288376
hsa_circ_	0.445522	0.288376
hsa_circ_	-0.29064	0.28838
hsa_circ_	0.203437	0.288383
hsa_circ_	0.592873	0.288393
hsa_circ_	0.423564	0.288396
hsa_circ_	-0.30381	0.288411
hsa_circ_	-0.17086	0.288413
hsa_circ_	-0.19786	0.288425
hsa_circ_	0.271928	0.288431
hsa_circ_	0.290264	0.288477
hsa_circ_	-0.1732	0.288482
hsa_circ_	-0.15428	0.288503
hsa_circ_	0.848938	0.288505
hsa_circ_	-0.54346	0.288511
hsa_circ_	-0.64102	0.288524
hsa_circ_	-0.19964	0.288569
hsa_circ_	0.366165	0.288574
hsa_circ_	0.313171	0.288581
hsa_circ_	-0.29075	0.288596
hsa_circ_	0.351799	0.28861
hsa_circ_	-0.14504	0.288613

hsa_circ_	-0.3111	0.288615
hsa_circ_	-0.2512	0.288635
hsa_circ_	-0.20764	0.288636
hsa_circ_	-0.1486	0.288662
hsa_circ_	-0.17233	0.288664
hsa_circ_	-0.15421	0.288681
hsa_circ_	-0.15417	0.288684
hsa_circ_	0.18991	0.288688
hsa_circ_	-0.55283	0.288692
hsa_circ_	0.231303	0.288711
hsa_circ_	0.241531	0.288715
hsa_circ_	0.355003	0.288722
hsa_circ_	0.157236	0.288737
hsa_circ_	0.246245	0.288748
hsa_circ_	-0.14721	0.288763
hsa_circ_	0.140928	0.288772
hsa_circ_	-0.20272	0.288795
hsa_circ_	-0.21271	0.288796
hsa_circ_	-0.18316	0.288855
hsa_circ_	0.181544	0.288895
hsa_circ_	0.26778	0.288897
hsa_circ_	0.801876	0.2889
hsa_circ_	0.422915	0.288911
hsa_circ_	0.157245	0.288954
hsa_circ_	0.214251	0.288973
hsa_circ_	0.510002	0.288976
hsa_circ_	0.264478	0.288983
hsa_circ_	0.175946	0.288986
hsa_circ_	0.182876	0.288993
hsa_circ_	0.221803	0.289001
hsa_circ_	-0.15186	0.289002
hsa_circ_	0.23904	0.289014
hsa_circ_	-0.57704	0.289027
hsa_circ_	-0.29936	0.289052
hsa_circ_	-0.18416	0.289061
hsa_circ_	0.310735	0.289074
hsa_circ_	0.183987	0.28909
hsa_circ_	0.30853	0.289102
hsa_circ_	-0.19599	0.289106
hsa_circ_	-0.20808	0.28912
hsa_circ_	0.189131	0.289123
hsa_circ_	-0.22595	0.289132
hsa_circ_	0.154527	0.289132
hsa_circ_	0.223738	0.289161
hsa_circ_	-0.39013	0.289162
hsa_circ_	0.178087	0.289188
hsa_circ_	0.210252	0.289195
hsa_circ_	-0.17984	0.289199
hsa_circ_	0.199401	0.289199
hsa_circ_	0.300693	0.289208
hsa_circ_	-0.14907	0.28921
hsa_circ_	-0.15393	0.289228
hsa_circ_	0.175361	0.289233
hsa_circ_	0.306082	0.289237

hsa_circ_	-0.34529	0.289244
hsa_circ_	-0.35384	0.289247
hsa_circ_	0.26227	0.289257
hsa_circ_	0.537713	0.289258
hsa_circ_	-0.15552	0.289265
hsa_circ_	-0.17799	0.289265
hsa_circ_	-0.15378	0.28927
hsa_circ_	0.247483	0.289302
hsa_circ_	-0.181	0.289306
hsa_circ_	-0.1957	0.289314
hsa_circ_	-0.2603	0.289319
hsa_circ_	-0.29207	0.289323
hsa_circ_	-0.293	0.289332
hsa_circ_	0.240912	0.289334
hsa_circ_	-0.15264	0.289351
hsa_circ_	-0.16934	0.289369
hsa_circ_	0.308409	0.28937
hsa_circ_	-0.14884	0.28937
hsa_circ_	0.366859	0.289373
hsa_circ_	0.18185	0.289381
hsa_circ_	-0.50076	0.289386
hsa_circ_	0.423414	0.289389
hsa_circ_	-0.24959	0.289406
hsa_circ_	-0.19302	0.289407
hsa_circ_	0.274851	0.289416
hsa_circ_	0.391199	0.289418
hsa_circ_	0.312029	0.289437
hsa_circ_	-0.48925	0.289445
hsa_circ_	-0.26747	0.289459
hsa_circ_	-0.17036	0.289466
hsa_circ_	0.176169	0.289468
hsa_circ_	0.147417	0.289473
hsa_circ_	0.215709	0.289481
hsa_circ_	0.715386	0.289483
hsa_circ_	0.527456	0.289511
hsa_circ_	0.458814	0.289512
hsa_circ_	0.147255	0.289525
hsa_circ_	0.251502	0.289551
hsa_circ_	-0.16853	0.289556
hsa_circ_	-0.18243	0.289594
hsa_circ_	-0.4604	0.289616
hsa_circ_	0.218994	0.289619
hsa_circ_	-0.29556	0.289622
hsa_circ_	0.253026	0.289625
hsa_circ_	-0.35773	0.289635
hsa_circ_	0.282157	0.289639
hsa_circ_	-0.28381	0.289639
hsa_circ_	0.212238	0.289655
hsa_circ_	0.148805	0.28966
hsa_circ_	-0.15049	0.289672
hsa_circ_	0.229427	0.289679
hsa_circ_	-0.17318	0.289689
hsa_circ_	-0.68355	0.289695
hsa_circ_	-0.17514	0.289712

hsa_circ_	0.255859	0.289713
hsa_circ_	-0.56919	0.289721
hsa_circ_	-0.8837	0.28973
hsa_circ_	-0.19569	0.289743
hsa_circ_	0.260186	0.289749
hsa_circ_	-0.22744	0.289758
hsa_circ_	0.673239	0.289783
hsa_circ_	-0.15904	0.289784
hsa_circ_	-0.24141	0.289792
hsa_circ_	-0.19137	0.289805
hsa_circ_	0.185784	0.289823
hsa_circ_	0.175397	0.289825
hsa_circ_	0.224635	0.289831
hsa_circ_	-0.38732	0.289836
hsa_circ_	0.180456	0.289843
hsa_circ_	0.302536	0.289879
hsa_circ_	-0.97994	0.289886
hsa_circ_	-0.16778	0.289889
hsa_circ_	-0.23069	0.28992
hsa_circ_	0.975015	0.289924
hsa_circ_	-0.195	0.289925
hsa_circ_	-0.45802	0.28994
hsa_circ_	-0.17378	0.289948
hsa_circ_	-0.50556	0.289984
hsa_circ_	0.266166	0.290002
hsa_circ_	-0.32606	0.290013
hsa_circ_	0.305009	0.29003
hsa_circ_	0.193889	0.290039
hsa_circ_	-0.33885	0.290058
hsa_circ_	0.207635	0.290064
hsa_circ_	0.246057	0.290083
hsa_circ_	-0.20552	0.290098
hsa_circ_	0.296293	0.290106
hsa_circ_	-0.21697	0.290108
hsa_circ_	-0.19542	0.290132
hsa_circ_	-0.54258	0.290138
hsa_circ_	0.633015	0.290148
hsa_circ_	-0.57414	0.290159
hsa_circ_	-0.18524	0.290169
hsa_circ_	-0.17587	0.29017
hsa_circ_	0.506754	0.290172
hsa_circ_	-0.15844	0.290175
hsa_circ_	0.16592	0.290182
hsa_circ_	0.24517	0.290203
hsa_circ_	-0.16045	0.290235
hsa_circ_	0.226704	0.290238
hsa_circ_	-0.19137	0.290248
hsa_circ_	-0.16755	0.290258
hsa_circ_	-0.3044	0.290266
hsa_circ_	-0.35242	0.29028
hsa_circ_	0.232628	0.290287
hsa_circ_	0.213368	0.2903
hsa_circ_	-0.19266	0.290328
hsa_circ_	-0.36123	0.290357

hsa_circ_	0.582195	0.290362
hsa_circ_	-0.47889	0.290373
hsa_circ_	0.261859	0.2904
hsa_circ_	0.368433	0.290405
hsa_circ_	-0.1675	0.290428
hsa_circ_	0.148222	0.290438
hsa_circ_	0.292885	0.29046
hsa_circ_	0.386144	0.290466
hsa_circ_	-0.19571	0.290471
hsa_circ_	-0.4073	0.290483
hsa_circ_	-0.97249	0.290484
hsa_circ_	-0.34838	0.29051
hsa_circ_	-0.74918	0.290517
hsa_circ_	-0.15512	0.29052
hsa_circ_	0.256724	0.290526
hsa_circ_	-0.29993	0.290534
hsa_circ_	0.186246	0.290547
hsa_circ_	-0.18169	0.290548
hsa_circ_	0.225358	0.290585
hsa_circ_	0.23816	0.290585
hsa_circ_	-0.14249	0.290604
hsa_circ_	0.179285	0.290616
hsa_circ_	0.228108	0.290617
hsa_circ_	-0.25258	0.290634
hsa_circ_	-0.17123	0.290635
hsa_circ_	-0.20603	0.290647
hsa_circ_	0.265433	0.290651
hsa_circ_	0.382389	0.29066
hsa_circ_	-0.28475	0.290662
hsa_circ_	-0.61182	0.290662
hsa_circ_	0.150819	0.290663
hsa_circ_	0.207694	0.290666
hsa_circ_	0.365616	0.290669
hsa_circ_	0.27684	0.290688
hsa_circ_	0.224469	0.290694
hsa_circ_	0.188565	0.290696
hsa_circ_	0.576444	0.290708
hsa_circ_	-0.17177	0.29071
hsa_circ_	-0.21248	0.29071
hsa_circ_	0.612636	0.290715
hsa_circ_	-0.18935	0.290719
hsa_circ_	-0.18548	0.290759
hsa_circ_	0.189511	0.29076
hsa_circ_	0.14791	0.290783
hsa_circ_	-0.18718	0.290819
hsa_circ_	-0.19668	0.290847
hsa_circ_	0.142295	0.290853
hsa_circ_	0.242085	0.29087
hsa_circ_	0.233609	0.290872
hsa_circ_	0.152542	0.290873
hsa_circ_	0.222798	0.290883
hsa_circ_	-0.51454	0.290885
hsa_circ_	0.414119	0.290889
hsa_circ_	0.227006	0.290893

hsa_circ_	-0.27748	0.290899
hsa_circ_	1.152818	0.290908
hsa_circ_	-0.322	0.290919
hsa_circ_	-0.20877	0.290922
hsa_circ_	0.305203	0.290922
hsa_circ_	-0.18704	0.290922
hsa_circ_	0.234291	0.290932
hsa_circ_	0.164306	0.290934
hsa_circ_	-0.16319	0.290942
hsa_circ_	0.324072	0.290959
hsa_circ_	-0.3389	0.290965
hsa_circ_	-0.31395	0.290974
hsa_circ_	-0.25139	0.290979
hsa_circ_	-0.27195	0.291001
hsa_circ_	0.79086	0.291006
hsa_circ_	0.265656	0.291024
hsa_circ_	-0.16312	0.291025
hsa_circ_	0.294536	0.291046
hsa_circ_	0.28361	0.291046
hsa_circ_	-0.52261	0.291047
hsa_circ_	0.323425	0.291069
hsa_circ_	-0.27979	0.291078
hsa_circ_	0.636538	0.291081
hsa_circ_	-0.51573	0.291086
hsa_circ_	-0.17881	0.291089
hsa_circ_	0.202588	0.291091
hsa_circ_	-0.16333	0.291101
hsa_circ_	0.179957	0.291102
hsa_circ_	-0.25681	0.291102
hsa_circ_	0.170474	0.291107
hsa_circ_	-1.10856	0.291119
hsa_circ_	-0.30306	0.291147
hsa_circ_	-0.33517	0.291148
hsa_circ_	-0.25254	0.291151
hsa_circ_	-0.55424	0.291156
hsa_circ_	-0.1663	0.291163
hsa_circ_	-0.19276	0.291167
hsa_circ_	0.169031	0.291167
hsa_circ_	-1.61182	0.291198
hsa_circ_	0.611253	0.2912
hsa_circ_	0.265065	0.291208
hsa_circ_	-0.28053	0.291215
hsa_circ_	0.474572	0.291216
hsa_circ_	0.204308	0.291225
hsa_circ_	0.261826	0.29123
hsa_circ_	-0.31298	0.291233
hsa_circ_	0.322717	0.291238
hsa_circ_	-0.38552	0.291283
hsa_circ_	-0.91018	0.291303
hsa_circ_	0.581126	0.291317
hsa_circ_	-0.24861	0.291324
hsa_circ_	0.369645	0.291343
hsa_circ_	-0.58243	0.291348
hsa_circ_	0.253871	0.29135

hsa_circ_	0.59516	0.291364
hsa_circ_	-0.1667	0.291365
hsa_circ_	0.35987	0.291371
hsa_circ_	-0.40394	0.291381
hsa_circ_	0.187754	0.291386
hsa_circ_	0.179646	0.291397
hsa_circ_	-0.22021	0.291406
hsa_circ_	-0.53215	0.291411
hsa_circ_	-0.40244	0.291415
hsa_circ_	0.223921	0.291432
hsa_circ_	-0.49159	0.291442
hsa_circ_	0.354581	0.291457
hsa_circ_	0.245218	0.291466
hsa_circ_	0.857172	0.291474
hsa_circ_	-0.21086	0.291489
hsa_circ_	0.210002	0.291513
hsa_circ_	0.220965	0.291519
hsa_circ_	-0.2661	0.291522
hsa_circ_	-0.21954	0.291524
hsa_circ_	-0.2074	0.291533
hsa_circ_	0.517413	0.291546
hsa_circ_	-0.64344	0.291556
hsa_circ_	0.174601	0.29156
hsa_circ_	0.297019	0.291563
hsa_circ_	0.146292	0.29157
hsa_circ_	0.267301	0.291572
hsa_circ_	-0.13893	0.29159
hsa_circ_	-0.17959	0.291592
hsa_circ_	-0.26764	0.291598
hsa_circ_	0.40804	0.2916
hsa_circ_	0.672657	0.291603
hsa_circ_	0.161454	0.291605
hsa_circ_	0.191891	0.291612
hsa_circ_	-0.23231	0.291613
hsa_circ_	-0.17183	0.291616
hsa_circ_	-0.25673	0.291619
hsa_circ_	-0.28269	0.291619
hsa_circ_	0.17061	0.29163
hsa_circ_	0.1787	0.291643
hsa_circ_	-0.20318	0.291653
hsa_circ_	-0.18273	0.29167
hsa_circ_	-0.21229	0.29167
hsa_circ_	0.233063	0.29167
hsa_circ_	-0.47763	0.291671
hsa_circ_	-0.23622	0.291753
hsa_circ_	0.19257	0.291754
hsa_circ_	0.164737	0.291754
hsa_circ_	-0.159	0.291775
hsa_circ_	0.340871	0.291777
hsa_circ_	0.170084	0.291791
hsa_circ_	-0.16618	0.291798
hsa_circ_	-0.55367	0.291811
hsa_circ_	-0.17773	0.291827
hsa_circ_	0.483506	0.291844

hsa_circ_	0.194522	0.291867
hsa_circ_	0.190491	0.29187
hsa_circ_	0.242702	0.291876
hsa_circ_	-0.24671	0.291878
hsa_circ_	-0.14056	0.291881
hsa_circ_	-0.16293	0.291882
hsa_circ_	-0.16142	0.291892
hsa_circ_	-0.20009	0.291893
hsa_circ_	0.251447	0.291893
hsa_circ_	0.175611	0.291909
hsa_circ_	-0.21058	0.291917
hsa_circ_	-0.60977	0.291931
hsa_circ_	0.147381	0.291937
hsa_circ_	-0.68466	0.291938
hsa_circ_	-0.14852	0.29194
hsa_circ_	0.159237	0.291972
hsa_circ_	0.345739	0.291974
hsa_circ_	-0.45118	0.291981
hsa_circ_	0.286115	0.291983
hsa_circ_	-0.44455	0.291989
hsa_circ_	-0.72127	0.291998
hsa_circ_	-1.09249	0.292004
hsa_circ_	-0.28518	0.292018
hsa_circ_	0.189642	0.292021
hsa_circ_	-0.31988	0.292027
hsa_circ_	0.220353	0.292027
hsa_circ_	0.188919	0.292027
hsa_circ_	0.367007	0.292034
hsa_circ_	0.148307	0.29205
hsa_circ_	-0.16391	0.292063
hsa_circ_	-0.5224	0.292084
hsa_circ_	-0.15501	0.292089
hsa_circ_	0.212177	0.292089
hsa_circ_	0.153142	0.292094
hsa_circ_	-0.15334	0.292104
hsa_circ_	-0.25387	0.292106
hsa_circ_	-0.27571	0.292123
hsa_circ_	0.254539	0.292151
hsa_circ_	0.3009	0.29217
hsa_circ_	-0.44756	0.292199
hsa_circ_	-0.79486	0.292207
hsa_circ_	-0.1594	0.292208
hsa_circ_	0.342734	0.292211
hsa_circ_	-0.2728	0.292217
hsa_circ_	0.748331	0.29222
hsa_circ_	0.147381	0.292233
hsa_circ_	-0.20955	0.292249
hsa_circ_	-1.06174	0.292251
hsa_circ_	-0.19255	0.292252
hsa_circ_	-0.69303	0.292265
hsa_circ_	-0.19531	0.292304
hsa_circ_	-0.18626	0.292318
hsa_circ_	-0.1472	0.292328
hsa_circ_	-0.48028	0.292353

hsa_circ_	0.228318	0.292355
hsa_circ_	0.314964	0.292378
hsa_circ_	0.169637	0.292379
hsa_circ_	-0.3639	0.292379
hsa_circ_	-0.20704	0.292397
hsa_circ_	-0.49535	0.292397
hsa_circ_	-0.17491	0.292411
hsa_circ_	0.172056	0.292438
hsa_circ_	-0.21213	0.292441
hsa_circ_	-0.26791	0.292456
hsa_circ_	0.229396	0.292462
hsa_circ_	-0.55189	0.292525
hsa_circ_	-0.22588	0.292546
hsa_circ_	-1.18209	0.292579
hsa_circ_	0.23516	0.292611
hsa_circ_	-0.22577	0.292612
hsa_circ_	-0.19338	0.292633
hsa_circ_	-0.17979	0.292637
hsa_circ_	0.327361	0.292645
hsa_circ_	-0.17859	0.292648
hsa_circ_	-0.25909	0.292655
hsa_circ_	-0.16362	0.292682
hsa_circ_	0.222368	0.292694
hsa_circ_	-0.16613	0.2927
hsa_circ_	-0.14948	0.292709
hsa_circ_	-0.21931	0.292734
hsa_circ_	0.722874	0.292763
hsa_circ_	0.352197	0.292772
hsa_circ_	-0.40152	0.292779
hsa_circ_	0.211126	0.292819
hsa_circ_	0.537736	0.292838
hsa_circ_	0.410465	0.292862
hsa_circ_	0.165137	0.292889
hsa_circ_	-0.14301	0.292899
hsa_circ_	0.215246	0.292929
hsa_circ_	-0.24425	0.29293
hsa_circ_	0.330769	0.292943
hsa_circ_	-0.21451	0.292951
hsa_circ_	0.224274	0.292954
hsa_circ_	0.273004	0.292973
hsa_circ_	0.320772	0.292979
hsa_circ_	0.160244	0.292981
hsa_circ_	-0.34626	0.293014
hsa_circ_	-0.16295	0.293027
hsa_circ_	0.143822	0.293044
hsa_circ_	0.207986	0.293051
hsa_circ_	0.484988	0.293055
hsa_circ_	-0.30161	0.293086
hsa_circ_	0.141027	0.293087
hsa_circ_	0.258518	0.293098
hsa_circ_	0.156341	0.2931
hsa_circ_	-0.21174	0.293103
hsa_circ_	-0.22633	0.293109
hsa_circ_	-0.41539	0.293113

hsa_circ_	0.683756	0.293114
hsa_circ_	-0.26312	0.293155
hsa_circ_	0.27295	0.293158
hsa_circ_	-0.23	0.293162
hsa_circ_	0.533333	0.293213
hsa_circ_	-0.15385	0.29322
hsa_circ_	0.241146	0.293222
hsa_circ_	0.210284	0.293232
hsa_circ_	-0.16458	0.293237
hsa_circ_	-0.1805	0.293264
hsa_circ_	-0.1534	0.293265
hsa_circ_	0.204302	0.293269
hsa_circ_	0.423168	0.293269
hsa_circ_	-0.19493	0.293271
hsa_circ_	-0.1532	0.293276
hsa_circ_	-0.26716	0.29328
hsa_circ_	-0.14198	0.293283
hsa_circ_	-0.15379	0.293305
hsa_circ_	-0.21473	0.293307
hsa_circ_	0.202526	0.293309
hsa_circ_	0.240336	0.293316
hsa_circ_	0.309253	0.293326
hsa_circ_	-0.15352	0.293345
hsa_circ_	-0.15125	0.293345
hsa_circ_	-0.34507	0.293349
hsa_circ_	-0.37099	0.29337
hsa_circ_	0.232237	0.293409
hsa_circ_	0.28797	0.293424
hsa_circ_	0.180174	0.293429
hsa_circ_	-0.31903	0.293441
hsa_circ_	-0.31378	0.293448
hsa_circ_	0.286885	0.293456
hsa_circ_	-0.18195	0.293456
hsa_circ_	-0.39421	0.293473
hsa_circ_	-0.21415	0.293482
hsa_circ_	0.407762	0.29352
hsa_circ_	0.328261	0.293544
hsa_circ_	-0.20979	0.293545
hsa_circ_	0.184039	0.293555
hsa_circ_	0.281202	0.293593
hsa_circ_	0.208874	0.293593
hsa_circ_	0.21543	0.293599
hsa_circ_	-0.28389	0.293608
hsa_circ_	-0.19926	0.293616
hsa_circ_	0.794811	0.293631
hsa_circ_	-0.53355	0.293649
hsa_circ_	-0.1415	0.293659
hsa_circ_	0.189333	0.293662
hsa_circ_	-0.41419	0.293666
hsa_circ_	0.313664	0.293668
hsa_circ_	0.297349	0.293687
hsa_circ_	0.639382	0.293693
hsa_circ_	0.810236	0.293694
hsa_circ_	0.24006	0.293706

hsa_circ_	0.694303	0.293739
hsa_circ_	-0.15616	0.293762
hsa_circ_	0.189721	0.293776
hsa_circ_	-0.15113	0.29378
hsa_circ_	-0.34266	0.293781
hsa_circ_	0.611099	0.293781
hsa_circ_	-0.16876	0.29379
hsa_circ_	-0.15104	0.293804
hsa_circ_	-0.17509	0.293809
hsa_circ_	-0.44353	0.293834
hsa_circ_	0.175003	0.293834
hsa_circ_	0.228625	0.293852
hsa_circ_	-0.28545	0.293852
hsa_circ_	-0.22048	0.293864
hsa_circ_	-0.30414	0.293874
hsa_circ_	-0.26973	0.293891
hsa_circ_	-0.14639	0.293911
hsa_circ_	-0.21568	0.293929
hsa_circ_	-0.19355	0.293942
hsa_circ_	-0.42414	0.293943
hsa_circ_	0.185941	0.293968
hsa_circ_	0.278297	0.293968
hsa_circ_	-0.25932	0.293986
hsa_circ_	0.240262	0.293994
hsa_circ_	-0.15291	0.294008
hsa_circ_	-0.15487	0.29401
hsa_circ_	-0.20036	0.294011
hsa_circ_	0.207682	0.294048
hsa_circ_	0.432484	0.294049
hsa_circ_	0.249401	0.294067
hsa_circ_	-0.24706	0.294097
hsa_circ_	0.448842	0.294108
hsa_circ_	-0.27931	0.294114
hsa_circ_	0.18468	0.294116
hsa_circ_	0.178954	0.294121
hsa_circ_	-0.21266	0.294123
hsa_circ_	-0.1658	0.294131
hsa_circ_	0.171776	0.294133
hsa_circ_	0.200331	0.294149
hsa_circ_	0.357817	0.294157
hsa_circ_	-0.37906	0.294166
hsa_circ_	0.609493	0.29418
hsa_circ_	0.303938	0.294232
hsa_circ_	0.173285	0.294253
hsa_circ_	0.371367	0.294255
hsa_circ_	0.231919	0.294258
hsa_circ_	-0.27445	0.294265
hsa_circ_	0.328824	0.294277
hsa_circ_	-0.37921	0.294278
hsa_circ_	0.188334	0.294291
hsa_circ_	-1.11199	0.294295
hsa_circ_	0.365538	0.294303
hsa_circ_	-0.33186	0.294314
hsa_circ_	-0.24092	0.294319

hsa_circ_	0.251332	0.294339
hsa_circ_	-0.35853	0.294349
hsa_circ_	-0.24383	0.294357
hsa_circ_	0.28821	0.294358
hsa_circ_	0.184865	0.29437
hsa_circ_	0.253678	0.29438
hsa_circ_	-0.17222	0.294386
hsa_circ_	-0.41474	0.294388
hsa_circ_	-0.37897	0.294402
hsa_circ_	-0.29087	0.294403
hsa_circ_	-0.18169	0.294412
hsa_circ_	0.861807	0.294412
hsa_circ_	-0.1863	0.294419
hsa_circ_	0.348009	0.294435
hsa_circ_	0.702981	0.294437
hsa_circ_	-0.41819	0.294447
hsa_circ_	0.228352	0.294448
hsa_circ_	0.283944	0.294454
hsa_circ_	-0.1537	0.294455
hsa_circ_	-0.20115	0.294463
hsa_circ_	0.416494	0.294471
hsa_circ_	-0.20565	0.294474
hsa_circ_	0.277005	0.294475
hsa_circ_	-0.53222	0.294478
hsa_circ_	-0.52678	0.294488
hsa_circ_	-0.31001	0.294534
hsa_circ_	-0.23206	0.294546
hsa_circ_	-0.17105	0.294551
hsa_circ_	-0.18107	0.29457
hsa_circ_	-0.16427	0.294576
hsa_circ_	-0.17455	0.294616
hsa_circ_	-0.22762	0.294623
hsa_circ_	-0.22631	0.294623
hsa_circ_	0.744388	0.294642
hsa_circ_	0.15286	0.294645
hsa_circ_	-0.27684	0.294654
hsa_circ_	-0.98374	0.294655
hsa_circ_	-0.15745	0.294682
hsa_circ_	0.157948	0.294693
hsa_circ_	0.420689	0.294712
hsa_circ_	0.294643	0.29472
hsa_circ_	-0.22374	0.294723
hsa_circ_	0.170561	0.29473
hsa_circ_	0.170694	0.294754
hsa_circ_	0.18213	0.294759
hsa_circ_	-0.25754	0.29476
hsa_circ_	0.188439	0.294802
hsa_circ_	-1.13581	0.294803
hsa_circ_	0.150756	0.294806
hsa_circ_	-0.22926	0.29482
hsa_circ_	0.233734	0.294845
hsa_circ_	-0.18686	0.294856
hsa_circ_	-0.31327	0.294861
hsa_circ_	-0.14994	0.294863

hsa_circ_	0.258448	0.294871
hsa_circ_	-0.146	0.294902
hsa_circ_	0.691859	0.294903
hsa_circ_	-0.14914	0.294903
hsa_circ_	-0.2395	0.294904
hsa_circ_	-0.19259	0.294904
hsa_circ_	-0.50948	0.29491
hsa_circ_	-0.26107	0.29491
hsa_circ_	0.166563	0.294917
hsa_circ_	-0.26915	0.294943
hsa_circ_	-0.22522	0.294956
hsa_circ_	0.203999	0.294958
hsa_circ_	-0.18519	0.294964
hsa_circ_	-0.38612	0.294968
hsa_circ_	0.154608	0.295008
hsa_circ_	-0.27583	0.295027
hsa_circ_	0.154494	0.295035
hsa_circ_	0.211214	0.295037
hsa_circ_	0.329089	0.295041
hsa_circ_	0.339942	0.295064
hsa_circ_	-0.21136	0.295072
hsa_circ_	0.146663	0.29511
hsa_circ_	0.61291	0.295112
hsa_circ_	0.544868	0.295116
hsa_circ_	-0.22467	0.295121
hsa_circ_	0.151916	0.295127
hsa_circ_	0.236937	0.295135
hsa_circ_	-0.17795	0.29514
hsa_circ_	-0.47472	0.295171
hsa_circ_	0.16099	0.295174
hsa_circ_	0.173764	0.295179
hsa_circ_	-0.17485	0.295212
hsa_circ_	0.25812	0.295216
hsa_circ_	-0.1775	0.295226
hsa_circ_	0.342612	0.295242
hsa_circ_	-0.16211	0.295251
hsa_circ_	-0.44184	0.295254
hsa_circ_	-0.17565	0.295268
hsa_circ_	-0.1594	0.29528
hsa_circ_	0.716003	0.295282
hsa_circ_	0.202907	0.295288
hsa_circ_	0.278184	0.2953
hsa_circ_	-0.24002	0.295313
hsa_circ_	0.238697	0.29532
hsa_circ_	0.280344	0.295326
hsa_circ_	0.441094	0.295327
hsa_circ_	-0.15048	0.295348
hsa_circ_	-0.20838	0.295352
hsa_circ_	-0.35281	0.295368
hsa_circ_	-0.15561	0.295377
hsa_circ_	-0.23503	0.295405
hsa_circ_	-0.30414	0.295434
hsa_circ_	-0.20943	0.295435
hsa_circ_	-0.16106	0.295438

hsa_circ_	-0.57429	0.295442
hsa_circ_	0.180429	0.295457
hsa_circ_	-0.19661	0.295502
hsa_circ_	-0.35763	0.295523
hsa_circ_	-0.48257	0.295549
hsa_circ_	-0.15504	0.29556
hsa_circ_	0.215392	0.295562
hsa_circ_	0.193229	0.295571
hsa_circ_	-0.29365	0.295591
hsa_circ_	-0.18399	0.295603
hsa_circ_	0.300362	0.295636
hsa_circ_	-0.26557	0.295648
hsa_circ_	0.153781	0.295658
hsa_circ_	-0.24852	0.295662
hsa_circ_	0.15408	0.295669
hsa_circ_	-0.18218	0.295682
hsa_circ_	0.392423	0.295693
hsa_circ_	0.282654	0.295697
hsa_circ_	0.445324	0.295718
hsa_circ_	0.218073	0.295745
hsa_circ_	-0.1946	0.295764
hsa_circ_	0.228051	0.295809
hsa_circ_	-0.29442	0.295812
hsa_circ_	-0.28983	0.295833
hsa_circ_	-0.47302	0.295833
hsa_circ_	0.220617	0.295862
hsa_circ_	-0.31064	0.295863
hsa_circ_	-0.17144	0.295864
hsa_circ_	0.202659	0.295873
hsa_circ_	-0.35059	0.295878
hsa_circ_	0.23836	0.295893
hsa_circ_	-0.16488	0.295895
hsa_circ_	-0.72471	0.295908
hsa_circ_	0.227021	0.295928
hsa_circ_	-0.19383	0.295937
hsa_circ_	0.277059	0.295939
hsa_circ_	-0.20232	0.295952
hsa_circ_	0.167217	0.295953
hsa_circ_	0.21196	0.295965
hsa_circ_	0.479513	0.295968
hsa_circ_	-0.20645	0.29597
hsa_circ_	-0.35855	0.295976
hsa_circ_	-0.1549	0.295986
hsa_circ_	-0.31735	0.29599
hsa_circ_	-0.3701	0.296004
hsa_circ_	0.24639	0.296009
hsa_circ_	-0.21442	0.296011
hsa_circ_	0.322623	0.296022
hsa_circ_	-0.26594	0.296026
hsa_circ_	-0.5031	0.296068
hsa_circ_	-0.16161	0.296073
hsa_circ_	0.169783	0.296074
hsa_circ_	0.157266	0.296108
hsa_circ_	0.231349	0.296117

hsa_circ_	0.22156	0.296127
hsa_circ_	0.300751	0.296129
hsa_circ_	-0.21949	0.29614
hsa_circ_	0.224803	0.296145
hsa_circ_	0.302453	0.296171
hsa_circ_	-0.15888	0.296186
hsa_circ_	0.588924	0.296208
hsa_circ_	0.35472	0.296222
hsa_circ_	0.265987	0.296223
hsa_circ_	0.163052	0.296233
hsa_circ_	-0.21123	0.296236
hsa_circ_	-0.14052	0.296253
hsa_circ_	-0.21326	0.29628
hsa_circ_	0.179612	0.296286
hsa_circ_	-0.48722	0.296294
hsa_circ_	0.32347	0.296313
hsa_circ_	0.195126	0.296318
hsa_circ_	-0.14704	0.29632
hsa_circ_	-0.52101	0.296363
hsa_circ_	-0.28256	0.296374
hsa_circ_	0.483361	0.296388
hsa_circ_	-0.16796	0.296408
hsa_circ_	0.220242	0.29643
hsa_circ_	0.416538	0.296432
hsa_circ_	0.287306	0.296432
hsa_circ_	-0.25877	0.296443
hsa_circ_	0.218074	0.296444
hsa_circ_	0.854253	0.296485
hsa_circ_	0.852945	0.296509
hsa_circ_	0.240225	0.296519
hsa_circ_	-0.41307	0.296519
hsa_circ_	-0.14592	0.296528
hsa_circ_	-0.15748	0.296537
hsa_circ_	-0.15764	0.296563
hsa_circ_	0.242276	0.296578
hsa_circ_	0.141698	0.296598
hsa_circ_	-0.29784	0.296617
hsa_circ_	-0.14328	0.296618
hsa_circ_	-0.24022	0.29662
hsa_circ_	-0.17575	0.296671
hsa_circ_	-0.29326	0.296674
hsa_circ_	0.175503	0.29669
hsa_circ_	-0.49986	0.296695
hsa_circ_	-0.21597	0.296698
hsa_circ_	0.663161	0.296707
hsa_circ_	0.182125	0.296711
hsa_circ_	0.439953	0.296711
hsa_circ_	-0.26603	0.296723
hsa_circ_	0.293464	0.296734
hsa_circ_	-0.18824	0.296748
hsa_circ_	0.250699	0.296772
hsa_circ_	0.178117	0.296781
hsa_circ_	-0.18168	0.296807
hsa_circ_	0.297557	0.296817

hsa_circ_	-0.29926	0.296817
hsa_circ_	0.15459	0.29683
hsa_circ_	-0.15702	0.29683
hsa_circ_	-0.20198	0.29685
hsa_circ_	-0.68711	0.296851
hsa_circ_	0.566095	0.296853
hsa_circ_	0.179549	0.296857
hsa_circ_	-0.40437	0.296902
hsa_circ_	0.319813	0.296948
hsa_circ_	0.205302	0.296951
hsa_circ_	0.216585	0.296956
hsa_circ_	0.23782	0.296961
hsa_circ_	0.144848	0.296962
hsa_circ_	-0.35094	0.296964
hsa_circ_	-0.15845	0.296973
hsa_circ_	0.207761	0.296975
hsa_circ_	-0.37967	0.296987
hsa_circ_	0.458519	0.297009
hsa_circ_	0.167726	0.297013
hsa_circ_	-0.26016	0.297032
hsa_circ_	-0.20122	0.297033
hsa_circ_	0.179445	0.297035
hsa_circ_	-0.196	0.297036
hsa_circ_	0.288959	0.297062
hsa_circ_	-0.22114	0.297098
hsa_circ_	0.1832	0.297112
hsa_circ_	-0.32529	0.297151
hsa_circ_	-0.40958	0.297178
hsa_circ_	0.278294	0.297198
hsa_circ_	-0.25894	0.297203
hsa_circ_	-0.14691	0.297206
hsa_circ_	-0.69435	0.297212
hsa_circ_	0.55748	0.297213
hsa_circ_	0.20868	0.297213
hsa_circ_	-0.28296	0.297217
hsa_circ_	0.348075	0.297232
hsa_circ_	0.300487	0.297239
hsa_circ_	-0.16907	0.297245
hsa_circ_	0.388489	0.297263
hsa_circ_	0.149198	0.297269
hsa_circ_	0.277194	0.297295
hsa_circ_	-0.22107	0.2973
hsa_circ_	-0.44377	0.297324
hsa_circ_	0.317237	0.297329
hsa_circ_	0.455832	0.297334
hsa_circ_	0.152906	0.297337
hsa_circ_	-0.48311	0.297359
hsa_circ_	-0.88056	0.297363
hsa_circ_	-0.2155	0.297364
hsa_circ_	-0.19206	0.297369
hsa_circ_	0.349575	0.297395
hsa_circ_	0.451588	0.297407
hsa_circ_	0.139408	0.297408
hsa_circ_	-0.55077	0.297418

hsa_circ_	-0.70888	0.29742
hsa_circ_	0.313018	0.297443
hsa_circ_	0.162919	0.297446
hsa_circ_	-0.15615	0.297478
hsa_circ_	0.275056	0.29749
hsa_circ_	-0.178	0.297516
hsa_circ_	-0.26482	0.297532
hsa_circ_	-0.20591	0.297533
hsa_circ_	-0.24459	0.297535
hsa_circ_	-0.19634	0.297541
hsa_circ_	-0.40296	0.297544
hsa_circ_	-0.18391	0.297545
hsa_circ_	0.38057	0.297549
hsa_circ_	-0.48851	0.297552
hsa_circ_	0.439071	0.297557
hsa_circ_	0.349713	0.297559
hsa_circ_	-0.28622	0.297564
hsa_circ_	0.630487	0.297581
hsa_circ_	-0.25798	0.297582
hsa_circ_	-0.1722	0.29759
hsa_circ_	0.168298	0.297613
hsa_circ_	0.744618	0.297623
hsa_circ_	-0.15947	0.297625
hsa_circ_	0.186516	0.297632
hsa_circ_	-0.26197	0.297634
hsa_circ_	0.531553	0.297635
hsa_circ_	0.256041	0.29764
hsa_circ_	-0.23193	0.297643
hsa_circ_	-0.32322	0.297644
hsa_circ_	-0.21105	0.297687
hsa_circ_	0.217797	0.297697
hsa_circ_	0.156492	0.297701
hsa_circ_	-0.22837	0.297717
hsa_circ_	-0.4407	0.297734
hsa_circ_	0.215287	0.297738
hsa_circ_	0.177755	0.297749
hsa_circ_	-0.38434	0.29775
hsa_circ_	0.257981	0.297754
hsa_circ_	0.20192	0.297756
hsa_circ_	-0.35834	0.297798
hsa_circ_	-0.57507	0.297802
hsa_circ_	-0.19701	0.297803
hsa_circ_	0.168918	0.297805
hsa_circ_	0.190331	0.297809
hsa_circ_	0.17473	0.297825
hsa_circ_	-0.18658	0.297827
hsa_circ_	0.197971	0.297827
hsa_circ_	-0.40565	0.297846
hsa_circ_	-0.24427	0.297848
hsa_circ_	-0.24556	0.297856
hsa_circ_	-0.25709	0.297878
hsa_circ_	-0.24674	0.297882
hsa_circ_	0.309084	0.297896
hsa_circ_	-0.86183	0.297936

hsa_circ_	-0.38812	0.297953
hsa_circ_	-0.17957	0.297959
hsa_circ_	0.182272	0.297966
hsa_circ_	0.204498	0.297994
hsa_circ_	0.241688	0.298032
hsa_circ_	0.339403	0.298044
hsa_circ_	-0.18317	0.298051
hsa_circ_	0.231897	0.298064
hsa_circ_	-0.20606	0.298064
hsa_circ_	0.214715	0.298066
hsa_circ_	-0.14541	0.298074
hsa_circ_	0.362856	0.298106
hsa_circ_	-0.17545	0.29812
hsa_circ_	-0.17017	0.298128
hsa_circ_	-0.43947	0.29815
hsa_circ_	0.595876	0.298214
hsa_circ_	-0.18666	0.29828
hsa_circ_	0.299087	0.298292
hsa_circ_	-0.60848	0.298294
hsa_circ_	0.383163	0.298299
hsa_circ_	0.351811	0.298321
hsa_circ_	0.232358	0.298377
hsa_circ_	0.452352	0.298381
hsa_circ_	0.242163	0.298391
hsa_circ_	0.179273	0.298408
hsa_circ_	0.323057	0.298415
hsa_circ_	0.241511	0.298419
hsa_circ_	-0.57097	0.29842
hsa_circ_	0.224649	0.298454
hsa_circ_	-0.19003	0.298466
hsa_circ_	-0.23581	0.298486
hsa_circ_	0.177588	0.298493
hsa_circ_	0.259746	0.298501
hsa_circ_	-0.18584	0.298506
hsa_circ_	-0.14235	0.298514
hsa_circ_	0.252395	0.298515
hsa_circ_	0.476112	0.298519
hsa_circ_	0.243558	0.29853
hsa_circ_	-0.54341	0.298532
hsa_circ_	-0.42415	0.298558
hsa_circ_	-0.58194	0.298568
hsa_circ_	-0.15042	0.2986
hsa_circ_	0.805245	0.298609
hsa_circ_	-0.2425	0.298625
hsa_circ_	-0.14562	0.298634
hsa_circ_	0.698297	0.298658
hsa_circ_	-0.14995	0.298677
hsa_circ_	-0.31348	0.298679
hsa_circ_	-0.64203	0.298693
hsa_circ_	-0.17578	0.298695
hsa_circ_	-0.1737	0.298706
hsa_circ_	-0.33696	0.298722
hsa_circ_	-0.13894	0.298742
hsa_circ_	-0.2501	0.298747

hsa_circ_	-0.17862	0.29875
hsa_circ_	0.168222	0.298783
hsa_circ_	1.073164	0.298793
hsa_circ_	0.203225	0.298799
hsa_circ_	0.249323	0.298803
hsa_circ_	0.256262	0.298809
hsa_circ_	0.622447	0.298832
hsa_circ_	0.271777	0.298839
hsa_circ_	0.165536	0.29884
hsa_circ_	-0.41141	0.298868
hsa_circ_	-0.14761	0.298882
hsa_circ_	-0.29146	0.298893
hsa_circ_	0.204069	0.298902
hsa_circ_	0.638259	0.298927
hsa_circ_	0.337808	0.29893
hsa_circ_	-0.32478	0.29894
hsa_circ_	-0.58036	0.298945
hsa_circ_	-0.21335	0.298962
hsa_circ_	-0.37192	0.298968
hsa_circ_	0.505699	0.298975
hsa_circ_	0.27093	0.298979
hsa_circ_	0.292006	0.298988
hsa_circ_	-0.15007	0.29899
hsa_circ_	0.226534	0.299003
hsa_circ_	-0.17028	0.299014
hsa_circ_	0.298185	0.29904
hsa_circ_	0.213264	0.299046
hsa_circ_	-0.29801	0.299063
hsa_circ_	0.40263	0.299093
hsa_circ_	-0.3066	0.299099
hsa_circ_	-0.33507	0.299114
hsa_circ_	0.302241	0.299116
hsa_circ_	0.19173	0.299121
hsa_circ_	-0.2147	0.299137
hsa_circ_	-0.14715	0.299168
hsa_circ_	-0.1865	0.299171
hsa_circ_	-0.19684	0.299194
hsa_circ_	0.646191	0.299209
hsa_circ_	-0.19712	0.299211
hsa_circ_	-0.31209	0.299224
hsa_circ_	-0.69447	0.29924
hsa_circ_	-0.33023	0.299256
hsa_circ_	0.166035	0.299263
hsa_circ_	-0.19423	0.299309
hsa_circ_	0.183707	0.299314
hsa_circ_	0.139897	0.299319
hsa_circ_	-0.15242	0.299321
hsa_circ_	0.336407	0.299325
hsa_circ_	0.211348	0.299328
hsa_circ_	0.237258	0.299336
hsa_circ_	0.206045	0.299351
hsa_circ_	-0.15729	0.299365
hsa_circ_	-0.57783	0.29938
hsa_circ_	0.604174	0.299391

hsa_circ_	-0.28563	0.299395
hsa_circ_	-0.34221	0.299402
hsa_circ_	0.327248	0.299408
hsa_circ_	0.56816	0.299432
hsa_circ_	0.344649	0.299434
hsa_circ_	0.271206	0.299435
hsa_circ_	-0.48342	0.29944
hsa_circ_	0.217058	0.299448
hsa_circ_	-0.16107	0.299451
hsa_circ_	0.21084	0.299472
hsa_circ_	-0.15656	0.299475
hsa_circ_	-0.2947	0.299481
hsa_circ_	-0.22728	0.299504
hsa_circ_	-0.18314	0.299529
hsa_circ_	0.362898	0.299539
hsa_circ_	-0.25018	0.299555
hsa_circ_	0.146454	0.299575
hsa_circ_	0.199847	0.299584
hsa_circ_	-0.46263	0.299588
hsa_circ_	0.373821	0.299612
hsa_circ_	0.643796	0.29963
hsa_circ_	0.746493	0.299672
hsa_circ_	0.191791	0.299674
hsa_circ_	-0.24754	0.299694
hsa_circ_	-0.24414	0.29972
hsa_circ_	-0.31939	0.299749
hsa_circ_	0.553972	0.299755
hsa_circ_	-0.21923	0.299821
hsa_circ_	-0.30028	0.299823
hsa_circ_	-0.59532	0.299837
hsa_circ_	0.258092	0.299839
hsa_circ_	-0.20837	0.299859
hsa_circ_	-0.19538	0.299879
hsa_circ_	-0.38304	0.299883
hsa_circ_	-0.30553	0.299883
hsa_circ_	0.340721	0.299885
hsa_circ_	0.325929	0.2999
hsa_circ_	0.171855	0.299913
hsa_circ_	-0.21718	0.299918
hsa_circ_	0.26302	0.299925
hsa_circ_	-0.24052	0.299932
hsa_circ_	0.43518	0.299937
hsa_circ_	-0.21219	0.299942
hsa_circ_	-0.42159	0.299956
hsa_circ_	0.295483	0.299965
hsa_circ_	-0.35034	0.299975
hsa_circ_	-0.22695	0.299984
hsa_circ_	-0.18396	0.299991
hsa_circ_	-0.15511	0.299996
hsa_circ_	-0.27075	0.299998
hsa_circ_	-0.25405	0.30001
hsa_circ_	0.273837	0.300013
hsa_circ_	-0.16872	0.300023
hsa_circ_	0.284132	0.300026

hsa_circ_	-0.26773	0.300031
hsa_circ_	0.175452	0.300059
hsa_circ_	-0.14557	0.300086
hsa_circ_	-0.50307	0.300104
hsa_circ_	-0.21967	0.300123
hsa_circ_	0.326079	0.300126
hsa_circ_	-0.24625	0.300143
hsa_circ_	-0.21515	0.300143
hsa_circ_	0.455826	0.300149
hsa_circ_	0.239148	0.300166
hsa_circ_	-0.43336	0.300167
hsa_circ_	0.244004	0.300169
hsa_circ_	0.211456	0.30019
hsa_circ_	-0.22809	0.30019
hsa_circ_	0.168096	0.300197
hsa_circ_	0.3334	0.300202
hsa_circ_	0.533397	0.300214
hsa_circ_	0.380693	0.30022
hsa_circ_	0.400036	0.300228
hsa_circ_	-0.16967	0.300243
hsa_circ_	0.190193	0.300249
hsa_circ_	1.382157	0.300249
hsa_circ_	-0.23724	0.300261
hsa_circ_	-0.21649	0.300302
hsa_circ_	-0.2054	0.300333
hsa_circ_	-0.3224	0.300334
hsa_circ_	-0.20248	0.300334
hsa_circ_	0.291524	0.300343
hsa_circ_	-0.20326	0.300363
hsa_circ_	0.490617	0.300369
hsa_circ_	-0.35581	0.300375
hsa_circ_	0.166373	0.300381
hsa_circ_	0.164939	0.300392
hsa_circ_	-0.28328	0.300397
hsa_circ_	-0.38374	0.300407
hsa_circ_	-0.18609	0.300421
hsa_circ_	-0.18452	0.300436
hsa_circ_	-0.24875	0.300439
hsa_circ_	0.509973	0.300452
hsa_circ_	-0.35248	0.300452
hsa_circ_	0.17865	0.300453
hsa_circ_	-0.39695	0.300454
hsa_circ_	0.370981	0.300481
hsa_circ_	-0.28136	0.300481
hsa_circ_	-0.27249	0.300484
hsa_circ_	0.327961	0.300491
hsa_circ_	-0.2128	0.300491
hsa_circ_	-0.23207	0.300498
hsa_circ_	-0.31152	0.300498
hsa_circ_	0.180851	0.300507
hsa_circ_	-0.43342	0.300519
hsa_circ_	-0.48104	0.300523
hsa_circ_	0.359232	0.300526
hsa_circ_	-0.23168	0.300527

hsa_circ_	-0.69771	0.300533
hsa_circ_	0.276701	0.300566
hsa_circ_	-0.32525	0.300571
hsa_circ_	-0.20176	0.300586
hsa_circ_	0.274298	0.300607
hsa_circ_	0.291697	0.300609
hsa_circ_	0.341201	0.300617
hsa_circ_	0.362521	0.30062
hsa_circ_	-0.1743	0.300628
hsa_circ_	-0.17483	0.300649
hsa_circ_	-0.33549	0.300665
hsa_circ_	0.157152	0.300683
hsa_circ_	0.193076	0.300705
hsa_circ_	-0.22076	0.300719
hsa_circ_	-0.25792	0.300727
hsa_circ_	-0.2948	0.300729
hsa_circ_	0.148833	0.300751
hsa_circ_	-0.17952	0.300766
hsa_circ_	0.264802	0.300769
hsa_circ_	0.212043	0.300775
hsa_circ_	0.2935	0.300776
hsa_circ_	-0.25016	0.300781
hsa_circ_	0.280237	0.300792
hsa_circ_	-0.21097	0.300793
hsa_circ_	-0.23722	0.300811
hsa_circ_	0.195797	0.300826
hsa_circ_	0.286892	0.300834
hsa_circ_	-0.18846	0.300852
hsa_circ_	-0.14213	0.300859
hsa_circ_	-0.33057	0.300865
hsa_circ_	-0.1379	0.300866
hsa_circ_	0.166811	0.300869
hsa_circ_	0.68343	0.300875
hsa_circ_	-0.20243	0.300879
hsa_circ_	-0.24315	0.300886
hsa_circ_	-0.63161	0.300895
hsa_circ_	-0.49888	0.300898
hsa_circ_	-0.18511	0.300926
hsa_circ_	0.242269	0.300937
hsa_circ_	0.270506	0.300949
hsa_circ_	-0.17508	0.30096
hsa_circ_	-0.18575	0.300965
hsa_circ_	0.206768	0.300965
hsa_circ_	-0.25167	0.300968
hsa_circ_	-0.3911	0.300973
hsa_circ_	-0.15917	0.300974
hsa_circ_	0.164607	0.300988
hsa_circ_	-0.42575	0.300997
hsa_circ_	0.220158	0.30104
hsa_circ_	0.328085	0.30106
hsa_circ_	-0.63012	0.301067
hsa_circ_	-0.43199	0.301078
hsa_circ_	0.147515	0.301082
hsa_circ_	0.268245	0.301083

hsa_circ_	0.337906	0.301106
hsa_circ_	0.401339	0.301112
hsa_circ_	-0.31111	0.30113
hsa_circ_	-0.58481	0.301146
hsa_circ_	-0.37367	0.301187
hsa_circ_	-0.22608	0.301192
hsa_circ_	-0.18194	0.301192
hsa_circ_	-0.14731	0.301219
hsa_circ_	0.157576	0.301229
hsa_circ_	0.288675	0.301229
hsa_circ_	0.277973	0.301233
hsa_circ_	0.321302	0.301236
hsa_circ_	0.638794	0.301245
hsa_circ_	-0.22321	0.301246
hsa_circ_	-0.26305	0.301248
hsa_circ_	-0.58615	0.301248
hsa_circ_	-0.16314	0.301248
hsa_circ_	-0.40338	0.301253
hsa_circ_	-0.6227	0.301265
hsa_circ_	0.289959	0.301268
hsa_circ_	0.244507	0.30127
hsa_circ_	0.240346	0.301287
hsa_circ_	0.366257	0.301318
hsa_circ_	-0.15825	0.301324
hsa_circ_	0.330774	0.301334
hsa_circ_	0.147851	0.301378
hsa_circ_	-0.19959	0.3014
hsa_circ_	-0.2069	0.301403
hsa_circ_	-0.19962	0.301411
hsa_circ_	-0.29388	0.301413
hsa_circ_	-0.35083	0.301426
hsa_circ_	-0.28656	0.301443
hsa_circ_	0.24256	0.301479
hsa_circ_	0.325472	0.30149
hsa_circ_	0.205599	0.301492
hsa_circ_	0.400489	0.30152
hsa_circ_	-0.18837	0.30153
hsa_circ_	-0.43478	0.301531
hsa_circ_	0.175641	0.301534
hsa_circ_	0.260686	0.301554
hsa_circ_	0.192046	0.30156
hsa_circ_	-0.44342	0.301576
hsa_circ_	0.473809	0.30158
hsa_circ_	0.189691	0.30161
hsa_circ_	0.248425	0.301642
hsa_circ_	0.262399	0.301658
hsa_circ_	-0.17987	0.301659
hsa_circ_	-0.15321	0.301662
hsa_circ_	-0.19828	0.301667
hsa_circ_	-0.40372	0.301685
hsa_circ_	0.249543	0.301716
hsa_circ_	-0.2632	0.301729
hsa_circ_	0.154524	0.301733
hsa_circ_	-0.29117	0.301733

hsa_circ_	-0.46134	0.301743
hsa_circ_	-0.26855	0.301754
hsa_circ_	-0.19837	0.301769
hsa_circ_	0.183015	0.301781
hsa_circ_	-0.24928	0.301796
hsa_circ_	-0.22486	0.301805
hsa_circ_	-0.18316	0.301828
hsa_circ_	0.246901	0.30185
hsa_circ_	0.174977	0.301857
hsa_circ_	0.215788	0.301857
hsa_circ_	0.225398	0.301875
hsa_circ_	0.801459	0.301882
hsa_circ_	0.349496	0.301883
hsa_circ_	0.167537	0.301886
hsa_circ_	0.293415	0.301903
hsa_circ_	-0.25291	0.30193
hsa_circ_	-0.36246	0.301945
hsa_circ_	-0.33506	0.30195
hsa_circ_	-0.14898	0.301962
hsa_circ_	0.152567	0.301984
hsa_circ_	-0.34042	0.301988
hsa_circ_	0.197254	0.301993
hsa_circ_	-0.36692	0.30202
hsa_circ_	-0.59892	0.302025
hsa_circ_	0.197394	0.302033
hsa_circ_	-0.23851	0.302036
hsa_circ_	0.21107	0.302041
hsa_circ_	0.249373	0.30205
hsa_circ_	0.244043	0.302054
hsa_circ_	-0.58324	0.302059
hsa_circ_	-0.15745	0.302089
hsa_circ_	0.18846	0.302101
hsa_circ_	-0.18878	0.302108
hsa_circ_	-0.18274	0.302112
hsa_circ_	-0.29136	0.302117
hsa_circ_	-0.48524	0.302124
hsa_circ_	-0.17705	0.302125
hsa_circ_	-0.29915	0.302138
hsa_circ_	0.843169	0.302146
hsa_circ_	-0.42577	0.302148
hsa_circ_	0.18439	0.302148
hsa_circ_	0.53745	0.302171
hsa_circ_	0.193435	0.302174
hsa_circ_	0.280633	0.302202
hsa_circ_	0.164678	0.302215
hsa_circ_	0.307058	0.302216
hsa_circ_	-0.23629	0.302252
hsa_circ_	-0.18509	0.30226
hsa_circ_	0.143643	0.302265
hsa_circ_	-0.1607	0.302275
hsa_circ_	-0.41416	0.302291
hsa_circ_	0.164085	0.302295
hsa_circ_	-0.21186	0.30231
hsa_circ_	0.279497	0.302314

hsa_circ_	-0.28123	0.302343
hsa_circ_	-0.2669	0.302352
hsa_circ_	-0.20752	0.302368
hsa_circ_	-0.19578	0.302385
hsa_circ_	0.321281	0.302407
hsa_circ_	-0.16929	0.302432
hsa_circ_	-0.31748	0.302477
hsa_circ_	0.149761	0.302486
hsa_circ_	-0.24507	0.302495
hsa_circ_	0.148802	0.302518
hsa_circ_	-0.57712	0.302536
hsa_circ_	0.146006	0.302571
hsa_circ_	0.323993	0.30258
hsa_circ_	0.171507	0.302588
hsa_circ_	0.407365	0.30259
hsa_circ_	0.2247	0.302602
hsa_circ_	0.294968	0.302611
hsa_circ_	0.304146	0.302613
hsa_circ_	0.18759	0.302613
hsa_circ_	0.22856	0.302615
hsa_circ_	0.716789	0.302641
hsa_circ_	-0.32094	0.302643
hsa_circ_	-0.1872	0.302654
hsa_circ_	0.172308	0.30266
hsa_circ_	-0.2604	0.302672
hsa_circ_	0.365015	0.302679
hsa_circ_	-0.24455	0.30268
hsa_circ_	0.262395	0.302685
hsa_circ_	0.167666	0.302689
hsa_circ_	-0.15609	0.3027
hsa_circ_	-0.30683	0.302718
hsa_circ_	-0.15929	0.302721
hsa_circ_	0.406249	0.302724
hsa_circ_	-0.26222	0.302729
hsa_circ_	0.813996	0.302749
hsa_circ_	0.297394	0.302758
hsa_circ_	0.140202	0.302764
hsa_circ_	0.184716	0.302764
hsa_circ_	-0.13875	0.302777
hsa_circ_	-0.17544	0.302785
hsa_circ_	0.223306	0.302803
hsa_circ_	0.375617	0.302815
hsa_circ_	-0.1502	0.302817
hsa_circ_	0.320539	0.302826
hsa_circ_	-0.31626	0.302828
hsa_circ_	0.41919	0.302839
hsa_circ_	-0.3506	0.302864
hsa_circ_	-0.17644	0.302883
hsa_circ_	0.23058	0.302885
hsa_circ_	-0.23604	0.302906
hsa_circ_	-0.18445	0.302925
hsa_circ_	0.204495	0.30293
hsa_circ_	0.827867	0.302931
hsa_circ_	0.167497	0.302939

hsa_circ_	0.278757	0.302953
hsa_circ_	0.41111	0.302968
hsa_circ_	0.374506	0.302968
hsa_circ_	0.214181	0.302968
hsa_circ_	-0.43975	0.302991
hsa_circ_	-0.18293	0.302996
hsa_circ_	-0.1429	0.302997
hsa_circ_	0.325127	0.303001
hsa_circ_	0.155894	0.303002
hsa_circ_	-0.28253	0.303015
hsa_circ_	0.216164	0.303016
hsa_circ_	0.204619	0.303029
hsa_circ_	-0.1624	0.303036
hsa_circ_	0.382379	0.303062
hsa_circ_	0.728827	0.303067
hsa_circ_	0.29539	0.303069
hsa_circ_	-0.28514	0.303071
hsa_circ_	0.917497	0.303086
hsa_circ_	-0.18362	0.303095
hsa_circ_	0.156948	0.303112
hsa_circ_	-0.15103	0.303115
hsa_circ_	0.248914	0.303134
hsa_circ_	-0.19344	0.303171
hsa_circ_	-0.17382	0.303173
hsa_circ_	-0.21385	0.303201
hsa_circ_	0.151675	0.303206
hsa_circ_	-0.22054	0.303243
hsa_circ_	-0.19475	0.303266
hsa_circ_	-0.47933	0.303275
hsa_circ_	0.209378	0.303287
hsa_circ_	0.368176	0.303294
hsa_circ_	0.592868	0.303301
hsa_circ_	-0.23519	0.303319
hsa_circ_	0.442079	0.303324
hsa_circ_	-0.17289	0.303348
hsa_circ_	-1.01336	0.303352
hsa_circ_	-0.37414	0.303357
hsa_circ_	-0.13879	0.303364
hsa_circ_	-0.19791	0.303368
hsa_circ_	0.182544	0.303394
hsa_circ_	-0.41057	0.3034
hsa_circ_	-0.18074	0.303409
hsa_circ_	-0.15039	0.30341
hsa_circ_	0.411204	0.303417
hsa_circ_	0.274037	0.303417
hsa_circ_	-0.49607	0.303428
hsa_circ_	0.254698	0.303431
hsa_circ_	0.146346	0.303445
hsa_circ_	-0.42428	0.303449
hsa_circ_	-0.25572	0.303456
hsa_circ_	-0.32838	0.303462
hsa_circ_	0.155951	0.30347
hsa_circ_	-0.20774	0.303493
hsa_circ_	-0.19958	0.303496

hsa_circ_	0.220384	0.303503
hsa_circ_	-0.1736	0.303504
hsa_circ_	-0.18424	0.303517
hsa_circ_	0.183096	0.303532
hsa_circ_	0.156824	0.303535
hsa_circ_	0.499762	0.303539
hsa_circ_	0.569805	0.303543
hsa_circ_	-0.29855	0.303543
hsa_circ_	-0.1766	0.303554
hsa_circ_	0.136903	0.303567
hsa_circ_	0.207364	0.303574
hsa_circ_	0.180836	0.303612
hsa_circ_	0.187237	0.30362
hsa_circ_	0.211166	0.303623
hsa_circ_	0.154814	0.303632
hsa_circ_	-0.21059	0.303649
hsa_circ_	0.210239	0.303654
hsa_circ_	0.299776	0.303669
hsa_circ_	-0.14751	0.303687
hsa_circ_	0.51763	0.303705
hsa_circ_	0.304598	0.303723
hsa_circ_	0.558022	0.303729
hsa_circ_	0.247212	0.303736
hsa_circ_	0.226699	0.303736
hsa_circ_	0.496353	0.30374
hsa_circ_	-0.2848	0.303757
hsa_circ_	0.261653	0.303815
hsa_circ_	-0.6771	0.303815
hsa_circ_	0.226717	0.303822
hsa_circ_	-0.16027	0.303828
hsa_circ_	1.128886	0.303845
hsa_circ_	-0.46357	0.303849
hsa_circ_	-0.2187	0.303857
hsa_circ_	-0.25832	0.303878
hsa_circ_	-0.23322	0.303882
hsa_circ_	-0.2463	0.303952
hsa_circ_	-0.14419	0.303974
hsa_circ_	-0.15352	0.303975
hsa_circ_	0.273205	0.303976
hsa_circ_	-0.23931	0.30403
hsa_circ_	-0.19263	0.304038
hsa_circ_	-0.18139	0.30404
hsa_circ_	0.23158	0.304053
hsa_circ_	0.159153	0.304057
hsa_circ_	-0.21751	0.304065
hsa_circ_	-0.24449	0.304068
hsa_circ_	-0.17096	0.30408
hsa_circ_	0.227789	0.30408
hsa_circ_	-0.19798	0.304082
hsa_circ_	-0.35601	0.304082
hsa_circ_	-0.2406	0.304084
hsa_circ_	-0.31447	0.30409
hsa_circ_	0.517985	0.304109
hsa_circ_	0.197579	0.304114

hsa_circ_	0.410768	0.304115
hsa_circ_	0.379845	0.304122
hsa_circ_	-0.25405	0.304156
hsa_circ_	0.318017	0.304208
hsa_circ_	0.157654	0.304216
hsa_circ_	0.394364	0.304223
hsa_circ_	0.357913	0.30424
hsa_circ_	-0.18215	0.304247
hsa_circ_	0.207378	0.304294
hsa_circ_	-0.14528	0.304304
hsa_circ_	0.736388	0.304332
hsa_circ_	-0.44354	0.304333
hsa_circ_	0.354894	0.304338
hsa_circ_	-0.14397	0.304376
hsa_circ_	-0.18949	0.304408
hsa_circ_	0.248391	0.304417
hsa_circ_	-0.21082	0.304429
hsa_circ_	0.358943	0.304429
hsa_circ_	-0.32577	0.304432
hsa_circ_	-0.40522	0.30447
hsa_circ_	0.175169	0.304509
hsa_circ_	-0.15758	0.304516
hsa_circ_	0.224162	0.304532
hsa_circ_	-0.14236	0.304542
hsa_circ_	0.452198	0.304554
hsa_circ_	0.215492	0.304559
hsa_circ_	-0.2473	0.304561
hsa_circ_	0.276935	0.304562
hsa_circ_	-0.16077	0.304582
hsa_circ_	0.609326	0.304582
hsa_circ_	0.22217	0.304588
hsa_circ_	-0.17953	0.304599
hsa_circ_	0.475939	0.3046
hsa_circ_	-0.18328	0.304603
hsa_circ_	0.32158	0.304618
hsa_circ_	-0.19052	0.304647
hsa_circ_	0.231839	0.304655
hsa_circ_	-0.63251	0.304665
hsa_circ_	0.234515	0.30469
hsa_circ_	-0.17814	0.304694
hsa_circ_	0.169456	0.304705
hsa_circ_	-0.13433	0.304716
hsa_circ_	-0.18967	0.304735
hsa_circ_	-0.14532	0.304737
hsa_circ_	0.47054	0.304742
hsa_circ_	-0.2612	0.304752
hsa_circ_	0.313261	0.304767
hsa_circ_	0.181939	0.30478
hsa_circ_	-0.18242	0.304783
hsa_circ_	-0.16235	0.304784
hsa_circ_	-0.21007	0.304796
hsa_circ_	0.158413	0.304805
hsa_circ_	0.470171	0.304816
hsa_circ_	-0.25174	0.304818

hsa_circ_	-0.17547	0.304822
hsa_circ_	-0.20043	0.304831
hsa_circ_	0.309094	0.304836
hsa_circ_	0.400739	0.304859
hsa_circ_	-0.2128	0.30486
hsa_circ_	-0.15791	0.30487
hsa_circ_	0.153102	0.304882
hsa_circ_	-0.20477	0.304901
hsa_circ_	-0.738	0.30491
hsa_circ_	0.284944	0.304915
hsa_circ_	-0.54051	0.304917
hsa_circ_	-0.19423	0.304948
hsa_circ_	-0.22781	0.304978
hsa_circ_	-0.72326	0.304986
hsa_circ_	-0.36997	0.304992
hsa_circ_	-0.13511	0.304993
hsa_circ_	-0.46588	0.305
hsa_circ_	0.22412	0.305005
hsa_circ_	-0.29522	0.305011
hsa_circ_	-0.7889	0.305034
hsa_circ_	-0.50447	0.305045
hsa_circ_	-0.50724	0.30505
hsa_circ_	-0.15626	0.305052
hsa_circ_	-0.37032	0.305054
hsa_circ_	0.566607	0.305063
hsa_circ_	0.279444	0.305081
hsa_circ_	0.186247	0.305094
hsa_circ_	-0.18209	0.305109
hsa_circ_	0.26273	0.305197
hsa_circ_	0.146658	0.305204
hsa_circ_	-0.4342	0.305205
hsa_circ_	0.177239	0.305216
hsa_circ_	0.199673	0.305237
hsa_circ_	-0.29449	0.305243
hsa_circ_	0.362236	0.30526
hsa_circ_	0.198974	0.305274
hsa_circ_	-0.19133	0.305287
hsa_circ_	0.32024	0.305289
hsa_circ_	0.202989	0.305298
hsa_circ_	-0.21241	0.305314
hsa_circ_	0.149599	0.305322
hsa_circ_	0.300627	0.305329
hsa_circ_	-0.63065	0.305333
hsa_circ_	-0.14125	0.305342
hsa_circ_	-0.13873	0.305343
hsa_circ_	-0.25519	0.305354
hsa_circ_	-0.15898	0.305361
hsa_circ_	-0.77008	0.305395
hsa_circ_	0.139285	0.305395
hsa_circ_	-0.14967	0.305403
hsa_circ_	-0.15321	0.305405
hsa_circ_	0.146716	0.305411
hsa_circ_	0.312616	0.305446
hsa_circ_	-0.15909	0.305453

hsa_circ_	-0.32847	0.305462
hsa_circ_	0.255139	0.305467
hsa_circ_	0.257077	0.305473
hsa_circ_	-0.14138	0.30549
hsa_circ_	-0.23212	0.305492
hsa_circ_	0.549465	0.305522
hsa_circ_	0.187205	0.305524
hsa_circ_	0.330557	0.305529
hsa_circ_	0.152873	0.305531
hsa_circ_	0.194094	0.305558
hsa_circ_	-0.78845	0.305568
hsa_circ_	0.19763	0.305576
hsa_circ_	-0.13994	0.305622
hsa_circ_	0.267897	0.305661
hsa_circ_	-0.31764	0.305676
hsa_circ_	-0.26162	0.305715
hsa_circ_	-0.27018	0.305719
hsa_circ_	-0.40632	0.305725
hsa_circ_	0.296593	0.305739
hsa_circ_	-0.19494	0.305745
hsa_circ_	0.265261	0.305765
hsa_circ_	0.263875	0.305776
hsa_circ_	0.17192	0.305778
hsa_circ_	-0.21057	0.305786
hsa_circ_	-0.22912	0.305796
hsa_circ_	0.204529	0.305798
hsa_circ_	0.2565	0.305814
hsa_circ_	0.264746	0.305828
hsa_circ_	0.318986	0.305834
hsa_circ_	-0.25744	0.305837
hsa_circ_	-0.19367	0.305884
hsa_circ_	0.311506	0.30589
hsa_circ_	-1.36832	0.305891
hsa_circ_	-0.23105	0.305898
hsa_circ_	0.173335	0.305904
hsa_circ_	-0.32778	0.305914
hsa_circ_	0.19415	0.305919
hsa_circ_	-0.78578	0.30592
hsa_circ_	0.19399	0.305956
hsa_circ_	-0.15978	0.305964
hsa_circ_	-0.15312	0.306021
hsa_circ_	0.501419	0.306024
hsa_circ_	-0.46505	0.306029
hsa_circ_	-0.14575	0.306036
hsa_circ_	-0.26001	0.306045
hsa_circ_	0.137238	0.306116
hsa_circ_	-0.16858	0.306119
hsa_circ_	0.296287	0.306122
hsa_circ_	0.378805	0.306123
hsa_circ_	-0.16153	0.306129
hsa_circ_	-0.23311	0.306138
hsa_circ_	-0.16166	0.306143
hsa_circ_	0.398965	0.306171
hsa_circ_	-0.30072	0.306184

hsa_circ_	0.174414	0.306215
hsa_circ_	-0.30488	0.306228
hsa_circ_	-0.22075	0.306229
hsa_circ_	0.18258	0.30623
hsa_circ_	-0.1682	0.306244
hsa_circ_	-0.14902	0.306274
hsa_circ_	0.178137	0.306285
hsa_circ_	0.233479	0.3063
hsa_circ_	0.397291	0.306304
hsa_circ_	-0.25376	0.306313
hsa_circ_	-0.2522	0.306316
hsa_circ_	-0.20984	0.306321
hsa_circ_	0.208563	0.306323
hsa_circ_	0.281097	0.306324
hsa_circ_	-0.29123	0.306331
hsa_circ_	-0.21498	0.306344
hsa_circ_	-0.20048	0.306344
hsa_circ_	-0.16001	0.306353
hsa_circ_	0.229244	0.306356
hsa_circ_	-0.15314	0.306358
hsa_circ_	0.210501	0.306374
hsa_circ_	0.223354	0.306375
hsa_circ_	0.327295	0.306384
hsa_circ_	-0.31744	0.306405
hsa_circ_	-0.32361	0.30642
hsa_circ_	-0.15821	0.30642
hsa_circ_	0.67792	0.306425
hsa_circ_	-0.39793	0.306446
hsa_circ_	0.324945	0.306455
hsa_circ_	0.34461	0.306466
hsa_circ_	0.274199	0.306466
hsa_circ_	0.686244	0.306487
hsa_circ_	0.137601	0.306505
hsa_circ_	0.147194	0.306537
hsa_circ_	-0.21268	0.306542
hsa_circ_	0.161291	0.306574
hsa_circ_	-0.20015	0.306585
hsa_circ_	-1.06944	0.306589
hsa_circ_	0.151763	0.306591
hsa_circ_	-0.4921	0.306596
hsa_circ_	-0.23814	0.306598
hsa_circ_	-0.18389	0.306604
hsa_circ_	-0.16921	0.306604
hsa_circ_	-0.37131	0.306615
hsa_circ_	0.491571	0.306648
hsa_circ_	0.197968	0.306664
hsa_circ_	-0.32353	0.306686
hsa_circ_	-0.22519	0.306697
hsa_circ_	0.165394	0.306715
hsa_circ_	-0.48325	0.306721
hsa_circ_	-0.65753	0.306724
hsa_circ_	-0.37	0.306725
hsa_circ_	0.682467	0.306725
hsa_circ_	-0.19337	0.306736

hsa_circ_	-0.17727	0.306759
hsa_circ_	0.240444	0.306763
hsa_circ_	0.713259	0.306773
hsa_circ_	0.347101	0.306796
hsa_circ_	1.108573	0.306802
hsa_circ_	0.236009	0.306809
hsa_circ_	0.158574	0.306811
hsa_circ_	0.196524	0.306811
hsa_circ_	-0.22302	0.306838
hsa_circ_	-0.21381	0.306856
hsa_circ_	0.237219	0.306868
hsa_circ_	-0.30189	0.306889
hsa_circ_	0.243649	0.30689
hsa_circ_	-0.17503	0.306894
hsa_circ_	0.198302	0.306903
hsa_circ_	0.159807	0.30691
hsa_circ_	0.380747	0.306931
hsa_circ_	-0.37823	0.306949
hsa_circ_	-0.3025	0.306972
hsa_circ_	0.469649	0.306995
hsa_circ_	-0.27329	0.306999
hsa_circ_	0.239915	0.307002
hsa_circ_	0.385493	0.307004
hsa_circ_	-0.224	0.307015
hsa_circ_	-0.17268	0.307035
hsa_circ_	0.250519	0.307035
hsa_circ_	0.136582	0.307043
hsa_circ_	0.586799	0.307067
hsa_circ_	0.161967	0.307068
hsa_circ_	-0.45333	0.307082
hsa_circ_	-1.09812	0.307096
hsa_circ_	0.341452	0.307096
hsa_circ_	0.180593	0.307109
hsa_circ_	0.17971	0.307122
hsa_circ_	-0.18355	0.307135
hsa_circ_	0.160673	0.307148
hsa_circ_	0.17077	0.307153
hsa_circ_	-0.56658	0.307154
hsa_circ_	-0.2463	0.307188
hsa_circ_	0.192139	0.307193
hsa_circ_	-0.21414	0.307243
hsa_circ_	-0.39286	0.30726
hsa_circ_	0.185338	0.307287
hsa_circ_	0.532546	0.307307
hsa_circ_	0.550883	0.307308
hsa_circ_	0.272676	0.307316
hsa_circ_	0.612643	0.307322
hsa_circ_	0.179472	0.307343
hsa_circ_	0.200953	0.307352
hsa_circ_	0.272232	0.307358
hsa_circ_	0.356553	0.30736
hsa_circ_	-0.80448	0.307362
hsa_circ_	0.335496	0.307363
hsa_circ_	-0.37039	0.30742

hsa_circ_	0.493632	0.307429
hsa_circ_	-0.55816	0.307431
hsa_circ_	-0.1669	0.307454
hsa_circ_	0.395928	0.307458
hsa_circ_	-0.14562	0.307462
hsa_circ_	-0.13591	0.307471
hsa_circ_	0.198389	0.30748
hsa_circ_	0.166673	0.307503
hsa_circ_	0.199487	0.307512
hsa_circ_	0.215677	0.307537
hsa_circ_	0.168134	0.307562
hsa_circ_	-0.3916	0.307574
hsa_circ_	-0.20371	0.307577
hsa_circ_	-0.35368	0.307609
hsa_circ_	-0.27878	0.307615
hsa_circ_	-0.22582	0.307617
hsa_circ_	-1.1032	0.30763
hsa_circ_	-0.18426	0.307633
hsa_circ_	0.199885	0.307671
hsa_circ_	0.285627	0.307677
hsa_circ_	-0.26492	0.307684
hsa_circ_	-0.20896	0.307726
hsa_circ_	0.169321	0.307727
hsa_circ_	-0.46592	0.307727
hsa_circ_	0.179513	0.307736
hsa_circ_	-0.19022	0.307736
hsa_circ_	0.156606	0.307749
hsa_circ_	-1.13672	0.307775
hsa_circ_	-0.26343	0.307795
hsa_circ_	-0.24412	0.307797
hsa_circ_	-0.25762	0.307802
hsa_circ_	0.196132	0.307822
hsa_circ_	0.170477	0.307823
hsa_circ_	-0.6341	0.30784
hsa_circ_	-0.23802	0.307864
hsa_circ_	-0.23054	0.307872
hsa_circ_	0.142881	0.307886
hsa_circ_	0.191905	0.307894
hsa_circ_	-0.18491	0.307909
hsa_circ_	-0.24236	0.307963
hsa_circ_	-0.19874	0.307965
hsa_circ_	-0.27211	0.307976
hsa_circ_	0.178552	0.307978
hsa_circ_	0.222541	0.307983
hsa_circ_	0.533697	0.307995
hsa_circ_	-0.31812	0.307998
hsa_circ_	-0.22629	0.308009
hsa_circ_	-0.25023	0.30807
hsa_circ_	0.21322	0.308096
hsa_circ_	-0.17943	0.308124
hsa_circ_	0.238813	0.30814
hsa_circ_	-0.24135	0.308152
hsa_circ_	0.628922	0.308184
hsa_circ_	-0.14113	0.308196

hsa_circ_	0.348017	0.308214
hsa_circ_	-0.17075	0.308216
hsa_circ_	-0.29199	0.308224
hsa_circ_	-0.18377	0.308248
hsa_circ_	-0.14881	0.308269
hsa_circ_	-0.64942	0.308273
hsa_circ_	0.228282	0.308275
hsa_circ_	-0.16108	0.308302
hsa_circ_	0.246525	0.308302
hsa_circ_	-0.58672	0.308303
hsa_circ_	0.196299	0.308343
hsa_circ_	-0.39854	0.308368
hsa_circ_	0.215254	0.308382
hsa_circ_	0.18527	0.308393
hsa_circ_	-0.246	0.308398
hsa_circ_	-0.3113	0.308407
hsa_circ_	0.239403	0.308412
hsa_circ_	-0.34492	0.308414
hsa_circ_	0.208	0.308417
hsa_circ_	0.269043	0.308463
hsa_circ_	-0.26244	0.308472
hsa_circ_	0.266059	0.308495
hsa_circ_	-0.78326	0.308507
hsa_circ_	-0.48128	0.308513
hsa_circ_	-0.4671	0.308515
hsa_circ_	0.167579	0.308519
hsa_circ_	-0.22064	0.308535
hsa_circ_	-0.95337	0.308556
hsa_circ_	0.195412	0.308579
hsa_circ_	-0.1496	0.308593
hsa_circ_	-0.13973	0.308604
hsa_circ_	0.240203	0.308618
hsa_circ_	0.149243	0.308622
hsa_circ_	-0.2987	0.308623
hsa_circ_	-0.15977	0.308626
hsa_circ_	-0.18197	0.30863
hsa_circ_	-0.14267	0.30867
hsa_circ_	0.170556	0.30867
hsa_circ_	0.184466	0.308685
hsa_circ_	-0.36578	0.308691
hsa_circ_	-0.3818	0.308706
hsa_circ_	0.212378	0.308714
hsa_circ_	0.97769	0.308718
hsa_circ_	-0.20871	0.308727
hsa_circ_	-0.1614	0.30873
hsa_circ_	0.35392	0.308743
hsa_circ_	0.272666	0.308745
hsa_circ_	0.359426	0.308778
hsa_circ_	-0.18639	0.308778
hsa_circ_	0.158882	0.308793
hsa_circ_	-0.16295	0.308797
hsa_circ_	0.551642	0.308812
hsa_circ_	0.221962	0.308843
hsa_circ_	0.178676	0.308865

hsa_circ_	-0.43637	0.308868
hsa_circ_	0.558392	0.308877
hsa_circ_	-0.23941	0.308925
hsa_circ_	-0.29848	0.308926
hsa_circ_	-0.47745	0.308946
hsa_circ_	0.319934	0.308947
hsa_circ_	0.176978	0.308955
hsa_circ_	-0.2322	0.308966
hsa_circ_	0.239545	0.308985
hsa_circ_	-0.14964	0.308986
hsa_circ_	-0.27182	0.308989
hsa_circ_	-0.16109	0.309
hsa_circ_	0.21408	0.30901
hsa_circ_	-0.16042	0.309013
hsa_circ_	0.222594	0.309029
hsa_circ_	0.333735	0.309077
hsa_circ_	0.255395	0.309079
hsa_circ_	0.171726	0.309081
hsa_circ_	-0.56295	0.309091
hsa_circ_	0.331817	0.309096
hsa_circ_	0.170842	0.309098
hsa_circ_	-0.26181	0.30911
hsa_circ_	-0.20818	0.309114
hsa_circ_	0.172097	0.309114
hsa_circ_	0.226361	0.309118
hsa_circ_	-0.2167	0.309138
hsa_circ_	-0.25201	0.309147
hsa_circ_	0.636367	0.309152
hsa_circ_	-0.27533	0.309175
hsa_circ_	0.173953	0.309192
hsa_circ_	-0.24706	0.309199
hsa_circ_	0.82911	0.309213
hsa_circ_	0.22186	0.309216
hsa_circ_	-0.3807	0.30923
hsa_circ_	0.270032	0.30925
hsa_circ_	0.589765	0.309259
hsa_circ_	-0.5815	0.309263
hsa_circ_	0.247761	0.309263
hsa_circ_	0.224872	0.309277
hsa_circ_	-0.36769	0.309284
hsa_circ_	0.165226	0.309303
hsa_circ_	-0.55715	0.309304
hsa_circ_	-0.38143	0.309312
hsa_circ_	0.305736	0.309313
hsa_circ_	-0.67582	0.309325
hsa_circ_	-0.20141	0.309326
hsa_circ_	0.452137	0.309332
hsa_circ_	-0.35355	0.309332
hsa_circ_	0.244016	0.309345
hsa_circ_	0.138728	0.309354
hsa_circ_	-0.18485	0.309358
hsa_circ_	0.927069	0.309366
hsa_circ_	-0.17304	0.309367
hsa_circ_	0.250497	0.309378

hsa_circ_	0.482817	0.309384
hsa_circ_	-0.37525	0.309393
hsa_circ_	-0.38298	0.309397
hsa_circ_	0.184679	0.309417
hsa_circ_	-0.41113	0.30942
hsa_circ_	-0.1561	0.30942
hsa_circ_	-0.16229	0.309424
hsa_circ_	0.45836	0.309427
hsa_circ_	-0.15353	0.309435
hsa_circ_	-0.50421	0.309446
hsa_circ_	0.275134	0.309448
hsa_circ_	-0.19992	0.309459
hsa_circ_	-0.19347	0.30946
hsa_circ_	-0.4846	0.309466
hsa_circ_	0.13954	0.309466
hsa_circ_	0.165384	0.30948
hsa_circ_	0.179035	0.30949
hsa_circ_	0.425292	0.309491
hsa_circ_	-0.19426	0.309494
hsa_circ_	0.345457	0.309514
hsa_circ_	0.390525	0.309517
hsa_circ_	0.310877	0.309518
hsa_circ_	0.641192	0.309518
hsa_circ_	0.149497	0.30954
hsa_circ_	0.33237	0.309555
hsa_circ_	-0.13512	0.309559
hsa_circ_	-0.30583	0.30957
hsa_circ_	0.251589	0.309591
hsa_circ_	-0.17079	0.309599
hsa_circ_	-0.20384	0.309617
hsa_circ_	0.162559	0.309625
hsa_circ_	-0.14225	0.30966
hsa_circ_	-0.21586	0.309674
hsa_circ_	-0.21469	0.309678
hsa_circ_	0.2436	0.309693
hsa_circ_	-0.76375	0.309711
hsa_circ_	0.267472	0.309712
hsa_circ_	-0.19736	0.309716
hsa_circ_	0.330743	0.309745
hsa_circ_	0.566334	0.309767
hsa_circ_	-0.3106	0.309767
hsa_circ_	0.449803	0.309779
hsa_circ_	-0.20571	0.309786
hsa_circ_	-0.16463	0.30982
hsa_circ_	-0.18153	0.309835
hsa_circ_	-0.31109	0.309878
hsa_circ_	0.163172	0.309886
hsa_circ_	-0.41511	0.309892
hsa_circ_	0.160152	0.309898
hsa_circ_	0.211867	0.309909
hsa_circ_	-0.20803	0.309921
hsa_circ_	0.159108	0.309931
hsa_circ_	0.230345	0.309947
hsa_circ_	0.253141	0.309961

hsa_circ_	0.419864	0.309994
hsa_circ_	0.199231	0.310007
hsa_circ_	0.166234	0.310009
hsa_circ_	-0.57123	0.310009
hsa_circ_	-0.3303	0.310034
hsa_circ_	0.357264	0.310045
hsa_circ_	-0.27704	0.310057
hsa_circ_	0.514587	0.31007
hsa_circ_	-0.6785	0.310074
hsa_circ_	0.388721	0.310103
hsa_circ_	-0.20095	0.310145
hsa_circ_	0.147616	0.310161
hsa_circ_	-0.37827	0.310171
hsa_circ_	-0.13386	0.310185
hsa_circ_	-0.22279	0.3102
hsa_circ_	0.667744	0.310204
hsa_circ_	-0.2559	0.310206
hsa_circ_	0.365503	0.31023
hsa_circ_	0.236865	0.310235
hsa_circ_	0.65913	0.310248
hsa_circ_	0.532949	0.310259
hsa_circ_	0.496004	0.310259
hsa_circ_	0.174528	0.310273
hsa_circ_	-0.32917	0.310284
hsa_circ_	0.148017	0.310297
hsa_circ_	-0.40153	0.3103
hsa_circ_	-0.21275	0.310321
hsa_circ_	0.156921	0.310333
hsa_circ_	-0.67526	0.310345
hsa_circ_	-0.34438	0.310346
hsa_circ_	-0.21207	0.310348
hsa_circ_	0.146517	0.310388
hsa_circ_	-0.27802	0.310413
hsa_circ_	-0.20737	0.310437
hsa_circ_	0.133443	0.310442
hsa_circ_	-0.58018	0.310471
hsa_circ_	0.589976	0.310473
hsa_circ_	0.215061	0.310476
hsa_circ_	-0.25578	0.310483
hsa_circ_	-0.18272	0.310487
hsa_circ_	0.460275	0.310492
hsa_circ_	-0.21664	0.310497
hsa_circ_	0.185117	0.310498
hsa_circ_	-0.19138	0.310504
hsa_circ_	0.203556	0.310519
hsa_circ_	0.183575	0.310524
hsa_circ_	-0.24221	0.310541
hsa_circ_	-0.22275	0.310553
hsa_circ_	-0.3648	0.310555
hsa_circ_	-0.38674	0.31058
hsa_circ_	0.148244	0.310595
hsa_circ_	-0.30622	0.310598
hsa_circ_	-0.21376	0.310602
hsa_circ_	-0.23241	0.310612

hsa_circ_	0.425568	0.310632
hsa_circ_	0.214772	0.310637
hsa_circ_	0.282089	0.31064
hsa_circ_	0.202612	0.310647
hsa_circ_	-0.20999	0.310653
hsa_circ_	-0.20951	0.310657
hsa_circ_	-0.14511	0.31067
hsa_circ_	-0.45284	0.310708
hsa_circ_	-0.18438	0.310719
hsa_circ_	0.241526	0.310722
hsa_circ_	0.701319	0.310724
hsa_circ_	-0.16118	0.310739
hsa_circ_	-1.08538	0.310745
hsa_circ_	-0.19144	0.310757
hsa_circ_	-0.33652	0.310776
hsa_circ_	0.243831	0.310799
hsa_circ_	-0.56379	0.31081
hsa_circ_	-0.22181	0.310813
hsa_circ_	0.280026	0.310841
hsa_circ_	-0.2429	0.310854
hsa_circ_	-0.1839	0.310871
hsa_circ_	0.360807	0.310875
hsa_circ_	0.171506	0.310881
hsa_circ_	0.160711	0.310891
hsa_circ_	0.205431	0.310945
hsa_circ_	-1.98083	0.310951
hsa_circ_	-0.16321	0.310961
hsa_circ_	-0.47668	0.310974
hsa_circ_	0.521979	0.310995
hsa_circ_	0.312023	0.31104
hsa_circ_	-0.19911	0.311052
hsa_circ_	0.197655	0.311061
hsa_circ_	-0.17512	0.311062
hsa_circ_	0.360252	0.311067
hsa_circ_	-0.3733	0.311068
hsa_circ_	-0.20765	0.311073
hsa_circ_	-0.31125	0.311085
hsa_circ_	0.566243	0.311097
hsa_circ_	0.14421	0.311098
hsa_circ_	0.173389	0.311108
hsa_circ_	-0.16465	0.311113
hsa_circ_	-0.51161	0.311124
hsa_circ_	0.242331	0.311145
hsa_circ_	0.201513	0.31116
hsa_circ_	0.405559	0.311168
hsa_circ_	0.349052	0.311175
hsa_circ_	0.364425	0.311185
hsa_circ_	0.189853	0.311192
hsa_circ_	-0.13455	0.311198
hsa_circ_	-0.34309	0.311223
hsa_circ_	0.346326	0.31123
hsa_circ_	-0.16188	0.31123
hsa_circ_	-0.49571	0.311248
hsa_circ_	-0.2693	0.31125

hsa_circ_	-0.2755	0.311251
hsa_circ_	0.162805	0.311266
hsa_circ_	-0.38292	0.31127
hsa_circ_	0.474106	0.31128
hsa_circ_	-0.17437	0.311281
hsa_circ_	0.328254	0.311299
hsa_circ_	-0.21248	0.311301
hsa_circ_	-0.88826	0.311306
hsa_circ_	-0.79888	0.311334
hsa_circ_	-0.52055	0.311334
hsa_circ_	0.438725	0.311344
hsa_circ_	0.215941	0.31138
hsa_circ_	0.175029	0.311396
hsa_circ_	0.209661	0.311399
hsa_circ_	0.285439	0.311407
hsa_circ_	0.442443	0.311418
hsa_circ_	-0.2871	0.311436
hsa_circ_	-0.22867	0.311466
hsa_circ_	-0.22421	0.311485
hsa_circ_	-0.25135	0.31151
hsa_circ_	-0.18472	0.311512
hsa_circ_	0.16332	0.311529
hsa_circ_	-0.17065	0.311538
hsa_circ_	0.375935	0.311539
hsa_circ_	-0.26135	0.311552
hsa_circ_	0.145579	0.311554
hsa_circ_	0.292011	0.311561
hsa_circ_	0.269222	0.311564
hsa_circ_	0.234557	0.311588
hsa_circ_	-0.79457	0.311589
hsa_circ_	0.245955	0.311592
hsa_circ_	0.41928	0.311596
hsa_circ_	-0.24411	0.311597
hsa_circ_	0.337017	0.311613
hsa_circ_	0.686785	0.311625
hsa_circ_	0.273103	0.31163
hsa_circ_	-0.15574	0.311636
hsa_circ_	-0.15001	0.311644
hsa_circ_	-0.37442	0.311646
hsa_circ_	-0.50393	0.311649
hsa_circ_	0.154967	0.31165
hsa_circ_	-0.28613	0.311659
hsa_circ_	-0.21937	0.311672
hsa_circ_	0.203124	0.311675
hsa_circ_	-0.23248	0.311727
hsa_circ_	0.142183	0.311752
hsa_circ_	-0.21605	0.311757
hsa_circ_	-0.54605	0.311782
hsa_circ_	-0.2743	0.311793
hsa_circ_	-0.19686	0.311797
hsa_circ_	-0.52529	0.311798
hsa_circ_	-0.19255	0.311821
hsa_circ_	0.189239	0.311827
hsa_circ_	-0.51915	0.311836

hsa_circ_	0.250568	0.311857
hsa_circ_	-0.16901	0.311857
hsa_circ_	0.214163	0.311863
hsa_circ_	0.200009	0.311873
hsa_circ_	0.294386	0.311883
hsa_circ_	-0.43169	0.311909
hsa_circ_	0.178889	0.311921
hsa_circ_	0.293187	0.311926
hsa_circ_	-0.33303	0.311936
hsa_circ_	-0.15005	0.311944
hsa_circ_	0.178681	0.311953
hsa_circ_	0.199597	0.31196
hsa_circ_	-0.16638	0.311962
hsa_circ_	0.31455	0.311964
hsa_circ_	-0.19145	0.311986
hsa_circ_	0.167729	0.312016
hsa_circ_	-0.45233	0.312027
hsa_circ_	0.917949	0.312032
hsa_circ_	-0.34155	0.312049
hsa_circ_	-0.18482	0.312067
hsa_circ_	-0.49537	0.312085
hsa_circ_	0.146461	0.312087
hsa_circ_	-0.16356	0.312092
hsa_circ_	-0.15163	0.312099
hsa_circ_	0.408911	0.31217
hsa_circ_	0.283978	0.31218
hsa_circ_	-0.19322	0.312192
hsa_circ_	0.273914	0.31222
hsa_circ_	0.191051	0.312225
hsa_circ_	-0.16889	0.312241
hsa_circ_	-0.1605	0.312246
hsa_circ_	-0.28927	0.312263
hsa_circ_	-0.16322	0.312272
hsa_circ_	0.199535	0.312273
hsa_circ_	-0.24014	0.312281
hsa_circ_	-0.17325	0.312294
hsa_circ_	0.208244	0.312307
hsa_circ_	0.204325	0.312333
hsa_circ_	-0.18328	0.312371
hsa_circ_	0.483641	0.312375
hsa_circ_	-0.21123	0.312375
hsa_circ_	0.203928	0.312384
hsa_circ_	-0.44395	0.312395
hsa_circ_	-0.15047	0.312398
hsa_circ_	0.399795	0.312404
hsa_circ_	-0.21788	0.312409
hsa_circ_	0.200545	0.312415
hsa_circ_	0.224771	0.312432
hsa_circ_	0.391869	0.312444
hsa_circ_	-0.13534	0.312454
hsa_circ_	-0.17042	0.312458
hsa_circ_	0.218838	0.312466
hsa_circ_	-0.13977	0.312475
hsa_circ_	-0.15766	0.312485

hsa_circ_	0.170986	0.312489
hsa_circ_	0.148805	0.312505
hsa_circ_	-0.14489	0.31251
hsa_circ_	-0.18726	0.312528
hsa_circ_	-0.25117	0.312531
hsa_circ_	-0.49819	0.312553
hsa_circ_	0.217798	0.312561
hsa_circ_	0.265513	0.312578
hsa_circ_	0.538026	0.312593
hsa_circ_	0.312403	0.312607
hsa_circ_	0.175554	0.312613
hsa_circ_	0.189041	0.312632
hsa_circ_	-0.18601	0.312637
hsa_circ_	-0.17388	0.312651
hsa_circ_	-0.21614	0.312653
hsa_circ_	-0.33747	0.312662
hsa_circ_	-0.7027	0.312664
hsa_circ_	0.23461	0.312673
hsa_circ_	-2.01687	0.312674
hsa_circ_	0.30067	0.312678
hsa_circ_	0.260391	0.312687
hsa_circ_	-0.15207	0.312692
hsa_circ_	-0.17784	0.31272
hsa_circ_	0.319158	0.312724
hsa_circ_	-0.18923	0.312744
hsa_circ_	-0.1744	0.312753
hsa_circ_	0.261203	0.312773
hsa_circ_	0.180748	0.312773
hsa_circ_	-0.19774	0.312778
hsa_circ_	0.232153	0.312783
hsa_circ_	-0.17986	0.312788
hsa_circ_	0.86898	0.312788
hsa_circ_	-0.15471	0.312809
hsa_circ_	0.284656	0.312811
hsa_circ_	-0.18459	0.312811
hsa_circ_	-0.45464	0.312829
hsa_circ_	-0.17756	0.31284
hsa_circ_	0.155771	0.312843
hsa_circ_	-0.38461	0.312861
hsa_circ_	0.178563	0.312886
hsa_circ_	-0.30796	0.312907
hsa_circ_	-0.48097	0.312954
hsa_circ_	-0.26659	0.312968
hsa_circ_	0.187409	0.312988
hsa_circ_	-0.18733	0.312992
hsa_circ_	0.274844	0.313005
hsa_circ_	-0.35154	0.313012
hsa_circ_	-0.20956	0.313013
hsa_circ_	-0.22707	0.313018
hsa_circ_	-0.25036	0.313059
hsa_circ_	0.756542	0.313062
hsa_circ_	-1.21032	0.313073
hsa_circ_	-0.40269	0.313088
hsa_circ_	0.249405	0.31312

hsa_circ_	-0.41397	0.313127
hsa_circ_	0.359553	0.313142
hsa_circ_	-1.15945	0.313151
hsa_circ_	0.983111	0.313182
hsa_circ_	-0.22289	0.313188
hsa_circ_	0.399256	0.31319
hsa_circ_	-0.24602	0.313194
hsa_circ_	-0.16164	0.313201
hsa_circ_	0.148728	0.313204
hsa_circ_	-0.21661	0.313212
hsa_circ_	0.349806	0.313227
hsa_circ_	0.226198	0.313239
hsa_circ_	-0.25697	0.313242
hsa_circ_	-0.31854	0.313254
hsa_circ_	-0.49627	0.313261
hsa_circ_	0.245846	0.313262
hsa_circ_	-3.07287	0.313297
hsa_circ_	-0.23616	0.313309
hsa_circ_	0.297881	0.313311
hsa_circ_	0.333111	0.313327
hsa_circ_	-0.20627	0.31333
hsa_circ_	0.496547	0.313341
hsa_circ_	0.334063	0.313344
hsa_circ_	-0.16056	0.31337
hsa_circ_	0.765369	0.313381
hsa_circ_	0.325841	0.313402
hsa_circ_	-0.23373	0.31342
hsa_circ_	0.282437	0.313425
hsa_circ_	-0.14853	0.313427
hsa_circ_	-0.13585	0.313445
hsa_circ_	-0.17349	0.313455
hsa_circ_	-0.90846	0.313481
hsa_circ_	-0.22064	0.313488
hsa_circ_	0.566395	0.313494
hsa_circ_	-0.44081	0.31355
hsa_circ_	0.246839	0.313562
hsa_circ_	-0.2076	0.313574
hsa_circ_	-0.27712	0.313584
hsa_circ_	-0.13481	0.313599
hsa_circ_	-0.16256	0.313608
hsa_circ_	0.155718	0.313612
hsa_circ_	-0.23088	0.31362
hsa_circ_	0.150786	0.313627
hsa_circ_	0.250564	0.313643
hsa_circ_	-0.61841	0.313674
hsa_circ_	-0.46623	0.31368
hsa_circ_	0.168088	0.313689
hsa_circ_	-0.59946	0.313692
hsa_circ_	0.143366	0.313694
hsa_circ_	-0.41864	0.313703
hsa_circ_	-0.16282	0.313712
hsa_circ_	-0.14579	0.313735
hsa_circ_	0.21574	0.313743
hsa_circ_	-0.34173	0.313746

hsa_circ_	-0.14657	0.313751
hsa_circ_	-0.32467	0.313766
hsa_circ_	-0.24421	0.313773
hsa_circ_	0.280432	0.313792
hsa_circ_	0.734846	0.313802
hsa_circ_	-0.37602	0.313816
hsa_circ_	-0.20761	0.31383
hsa_circ_	-0.14918	0.313834
hsa_circ_	0.239463	0.313835
hsa_circ_	-0.15214	0.31384
hsa_circ_	-0.17645	0.313856
hsa_circ_	-0.22897	0.313864
hsa_circ_	0.219311	0.313867
hsa_circ_	0.185045	0.313872
hsa_circ_	0.24354	0.313882
hsa_circ_	-0.53769	0.313893
hsa_circ_	0.196763	0.313896
hsa_circ_	-0.29147	0.313934
hsa_circ_	-0.21901	0.31394
hsa_circ_	0.448456	0.313943
hsa_circ_	0.262188	0.313949
hsa_circ_	-0.31564	0.313961
hsa_circ_	0.272621	0.313969
hsa_circ_	-0.66897	0.313983
hsa_circ_	-0.20191	0.313984
hsa_circ_	0.316092	0.313991
hsa_circ_	0.511205	0.314001
hsa_circ_	-0.14262	0.314003
hsa_circ_	-0.45582	0.314012
hsa_circ_	-0.24264	0.314012
hsa_circ_	-0.22547	0.314017
hsa_circ_	-0.34629	0.314033
hsa_circ_	0.196905	0.314038
hsa_circ_	-0.54278	0.31404
hsa_circ_	0.450035	0.314049
hsa_circ_	0.287054	0.314053
hsa_circ_	0.267581	0.314058
hsa_circ_	0.192355	0.314063
hsa_circ_	-0.14178	0.314069
hsa_circ_	0.141274	0.314081
hsa_circ_	0.277678	0.314088
hsa_circ_	0.28748	0.314107
hsa_circ_	-0.50013	0.31411
hsa_circ_	-0.28326	0.314128
hsa_circ_	0.225787	0.314131
hsa_circ_	0.166584	0.314146
hsa_circ_	-0.16184	0.314167
hsa_circ_	-0.16995	0.314189
hsa_circ_	-0.70282	0.314202
hsa_circ_	0.658178	0.314222
hsa_circ_	-0.15023	0.314222
hsa_circ_	0.274431	0.314251
hsa_circ_	0.17509	0.314258
hsa_circ_	-0.28759	0.314268

hsa_circ_	0.640205	0.314275
hsa_circ_	0.180278	0.314277
hsa_circ_	-0.23223	0.314281
hsa_circ_	-0.14145	0.314307
hsa_circ_	-0.20939	0.314315
hsa_circ_	-0.43362	0.314331
hsa_circ_	0.352681	0.314334
hsa_circ_	0.99752	0.314335
hsa_circ_	0.24707	0.314354
hsa_circ_	0.526009	0.314369
hsa_circ_	-0.30086	0.314385
hsa_circ_	-0.13728	0.314391
hsa_circ_	0.195268	0.314394
hsa_circ_	-0.19262	0.314403
hsa_circ_	-0.2827	0.314418
hsa_circ_	-0.17704	0.314421
hsa_circ_	-0.43516	0.314426
hsa_circ_	0.187145	0.31443
hsa_circ_	0.532728	0.314431
hsa_circ_	0.351144	0.314448
hsa_circ_	-0.16409	0.314455
hsa_circ_	-0.16488	0.314461
hsa_circ_	0.195373	0.314464
hsa_circ_	0.185762	0.314464
hsa_circ_	-0.15259	0.314478
hsa_circ_	-0.15776	0.314483
hsa_circ_	-0.53252	0.314488
hsa_circ_	-0.17414	0.314499
hsa_circ_	-0.18983	0.314507
hsa_circ_	-0.15877	0.314507
hsa_circ_	0.147271	0.31451
hsa_circ_	-0.15782	0.314517
hsa_circ_	-0.18925	0.314525
hsa_circ_	0.222611	0.314526
hsa_circ_	-0.24045	0.314534
hsa_circ_	0.142591	0.314555
hsa_circ_	-0.18652	0.314558
hsa_circ_	-0.30446	0.314559
hsa_circ_	0.171391	0.314584
hsa_circ_	-0.24475	0.314584
hsa_circ_	-0.1806	0.314585
hsa_circ_	0.501408	0.314591
hsa_circ_	0.259235	0.314593
hsa_circ_	-0.24419	0.3146
hsa_circ_	0.192699	0.314611
hsa_circ_	-0.14758	0.314611
hsa_circ_	-0.57508	0.314633
hsa_circ_	0.232139	0.314644
hsa_circ_	0.736879	0.314657
hsa_circ_	0.295637	0.314678
hsa_circ_	0.228392	0.31468
hsa_circ_	-0.16776	0.314683
hsa_circ_	0.226309	0.314691
hsa_circ_	0.213556	0.314696

hsa_circ_	-0.14139	0.314701
hsa_circ_	0.277844	0.31471
hsa_circ_	0.164796	0.31472
hsa_circ_	-0.19686	0.314729
hsa_circ_	-0.1472	0.314754
hsa_circ_	0.479606	0.314757
hsa_circ_	-0.24111	0.314757
hsa_circ_	0.758277	0.314802
hsa_circ_	-0.29759	0.314806
hsa_circ_	0.233579	0.314845
hsa_circ_	0.411792	0.314857
hsa_circ_	-0.15775	0.314869
hsa_circ_	-0.18948	0.314876
hsa_circ_	-0.16197	0.314884
hsa_circ_	0.432743	0.314889
hsa_circ_	-1.03213	0.314893
hsa_circ_	0.194567	0.314902
hsa_circ_	0.487895	0.31494
hsa_circ_	-0.21106	0.314945
hsa_circ_	-0.77013	0.314949
hsa_circ_	-0.26424	0.31495
hsa_circ_	-0.2807	0.314952
hsa_circ_	-0.25435	0.314953
hsa_circ_	-0.23701	0.314955
hsa_circ_	-0.2785	0.314973
hsa_circ_	0.19323	0.314974
hsa_circ_	-0.18865	0.314995
hsa_circ_	0.196386	0.315013
hsa_circ_	0.206841	0.315051
hsa_circ_	-0.23696	0.315056
hsa_circ_	-0.57934	0.315061
hsa_circ_	0.417175	0.315063
hsa_circ_	-0.1869	0.315073
hsa_circ_	-0.16708	0.315106
hsa_circ_	0.253536	0.315107
hsa_circ_	-0.46488	0.315109
hsa_circ_	-0.22539	0.315133
hsa_circ_	-0.21872	0.315139
hsa_circ_	-0.33976	0.315142
hsa_circ_	-0.13056	0.315145
hsa_circ_	0.236532	0.31517
hsa_circ_	0.196756	0.315194
hsa_circ_	-0.24169	0.315198
hsa_circ_	-0.14306	0.315206
hsa_circ_	0.275635	0.315227
hsa_circ_	-0.37532	0.315233
hsa_circ_	-0.19725	0.315253
hsa_circ_	-0.54102	0.315259
hsa_circ_	0.298228	0.315266
hsa_circ_	-0.24655	0.31527
hsa_circ_	0.142712	0.315276
hsa_circ_	0.264331	0.315284
hsa_circ_	-0.34914	0.315285
hsa_circ_	0.192028	0.315317

hsa_circ_	0.39588	0.315328
hsa_circ_	0.148977	0.315334
hsa_circ_	-0.24223	0.315352
hsa_circ_	-0.23146	0.315353
hsa_circ_	-0.14462	0.315354
hsa_circ_	-0.26868	0.315388
hsa_circ_	0.313314	0.315402
hsa_circ_	-0.69227	0.315422
hsa_circ_	0.480458	0.315426
hsa_circ_	0.366976	0.315443
hsa_circ_	-0.26753	0.315467
hsa_circ_	-0.31899	0.315478
hsa_circ_	-0.6664	0.315509
hsa_circ_	-0.19806	0.315513
hsa_circ_	0.160051	0.315515
hsa_circ_	0.166021	0.31552
hsa_circ_	-0.21668	0.315521
hsa_circ_	-0.26584	0.315525
hsa_circ_	-0.19165	0.315546
hsa_circ_	-0.14358	0.315553
hsa_circ_	-0.3814	0.315611
hsa_circ_	-0.37234	0.315626
hsa_circ_	-0.16327	0.315661
hsa_circ_	0.197773	0.315663
hsa_circ_	-0.21047	0.31568
hsa_circ_	-0.35176	0.315699
hsa_circ_	-0.16596	0.315702
hsa_circ_	-0.22522	0.315708
hsa_circ_	-0.18824	0.315713
hsa_circ_	0.153442	0.315718
hsa_circ_	-0.12983	0.315738
hsa_circ_	0.263358	0.315742
hsa_circ_	-0.19969	0.315751
hsa_circ_	0.276019	0.315762
hsa_circ_	0.290671	0.315768
hsa_circ_	0.133029	0.315768
hsa_circ_	-0.56341	0.315786
hsa_circ_	0.166881	0.315805
hsa_circ_	0.282087	0.315808
hsa_circ_	-0.43182	0.315816
hsa_circ_	-0.1459	0.315826
hsa_circ_	-0.19227	0.315831
hsa_circ_	-0.31706	0.315834
hsa_circ_	0.244552	0.315839
hsa_circ_	-0.19577	0.315845
hsa_circ_	0.181713	0.315845
hsa_circ_	-0.19496	0.315868
hsa_circ_	-0.15504	0.31587
hsa_circ_	-0.15241	0.315899
hsa_circ_	0.143375	0.315905
hsa_circ_	-0.34647	0.315908
hsa_circ_	0.327742	0.315919
hsa_circ_	-0.16008	0.315923
hsa_circ_	0.221698	0.315932

hsa_circ_	-0.14473	0.315933
hsa_circ_	-0.19009	0.315936
hsa_circ_	-0.16355	0.31594
hsa_circ_	0.199134	0.315945
hsa_circ_	0.166065	0.315955
hsa_circ_	0.447532	0.315962
hsa_circ_	-0.31146	0.315967
hsa_circ_	-0.18647	0.315978
hsa_circ_	0.487771	0.315993
hsa_circ_	0.16341	0.315999
hsa_circ_	-0.14822	0.316005
hsa_circ_	0.155968	0.316014
hsa_circ_	-0.73662	0.316021
hsa_circ_	-0.20045	0.316056
hsa_circ_	0.14268	0.316062
hsa_circ_	0.313736	0.316062
hsa_circ_	-0.17471	0.316089
hsa_circ_	-0.21708	0.316091
hsa_circ_	0.614365	0.316104
hsa_circ_	-0.15279	0.316111
hsa_circ_	0.184894	0.316114
hsa_circ_	0.156848	0.316136
hsa_circ_	0.18432	0.31615
hsa_circ_	-0.42888	0.316175
hsa_circ_	0.305025	0.316183
hsa_circ_	0.547332	0.316186
hsa_circ_	-0.42448	0.316205
hsa_circ_	-0.48445	0.316207
hsa_circ_	0.261451	0.316217
hsa_circ_	-0.42146	0.316228
hsa_circ_	-1.21922	0.316235
hsa_circ_	-0.19212	0.31625
hsa_circ_	0.193411	0.316259
hsa_circ_	0.281089	0.316261
hsa_circ_	-0.56481	0.316263
hsa_circ_	0.160591	0.31627
hsa_circ_	-0.39944	0.316285
hsa_circ_	-0.20648	0.316285
hsa_circ_	0.166223	0.316298
hsa_circ_	-0.19057	0.31631
hsa_circ_	-0.27656	0.316328
hsa_circ_	-0.15734	0.316331
hsa_circ_	-0.14646	0.316354
hsa_circ_	-0.14718	0.316357
hsa_circ_	-0.16776	0.316364
hsa_circ_	-0.17647	0.316396
hsa_circ_	0.325115	0.316439
hsa_circ_	-0.15642	0.31645
hsa_circ_	-0.32357	0.316453
hsa_circ_	-0.70245	0.316474
hsa_circ_	0.181456	0.316492
hsa_circ_	0.397757	0.316496
hsa_circ_	-0.20786	0.316506
hsa_circ_	-0.16306	0.316517

hsa_circ_	0.408856	0.316529
hsa_circ_	0.331356	0.316531
hsa_circ_	-0.23023	0.316533
hsa_circ_	-0.18508	0.316548
hsa_circ_	-0.44347	0.316551
hsa_circ_	0.180768	0.316558
hsa_circ_	0.346212	0.316566
hsa_circ_	-0.28622	0.316582
hsa_circ_	-0.2193	0.316607
hsa_circ_	0.18029	0.316635
hsa_circ_	-0.13845	0.316636
hsa_circ_	0.208384	0.31664
hsa_circ_	-0.27033	0.316653
hsa_circ_	-0.2172	0.316661
hsa_circ_	0.269124	0.316679
hsa_circ_	-0.13867	0.316682
hsa_circ_	0.189434	0.316682
hsa_circ_	-0.16248	0.316688
hsa_circ_	0.385333	0.316708
hsa_circ_	-0.54845	0.316749
hsa_circ_	0.614345	0.316757
hsa_circ_	0.974671	0.316771
hsa_circ_	-0.2096	0.316777
hsa_circ_	0.153338	0.316778
hsa_circ_	0.181718	0.316779
hsa_circ_	-0.56306	0.316801
hsa_circ_	0.152637	0.316805
hsa_circ_	-0.3358	0.31682
hsa_circ_	0.164489	0.316828
hsa_circ_	0.466136	0.316871
hsa_circ_	-0.39727	0.316916
hsa_circ_	0.149244	0.316919
hsa_circ_	-0.18727	0.316934
hsa_circ_	-0.2254	0.316936
hsa_circ_	-0.1955	0.316938
hsa_circ_	0.39295	0.316944
hsa_circ_	0.396377	0.316957
hsa_circ_	0.177633	0.31697
hsa_circ_	0.149511	0.316986
hsa_circ_	0.505627	0.317002
hsa_circ_	0.483247	0.317011
hsa_circ_	-0.5861	0.317038
hsa_circ_	-0.41576	0.317043
hsa_circ_	-0.24129	0.317049
hsa_circ_	0.337219	0.317057
hsa_circ_	-0.38168	0.317063
hsa_circ_	0.157405	0.3171
hsa_circ_	0.141938	0.317123
hsa_circ_	-0.14137	0.317134
hsa_circ_	0.429189	0.317134
hsa_circ_	-0.30706	0.317143
hsa_circ_	0.248751	0.317167
hsa_circ_	0.175533	0.317176
hsa_circ_	0.177232	0.317234

hsa_circ_	0.16322	0.317254
hsa_circ_	-0.19574	0.317261
hsa_circ_	0.179429	0.317269
hsa_circ_	0.614496	0.317277
hsa_circ_	-0.14926	0.317286
hsa_circ_	0.529141	0.317298
hsa_circ_	0.204991	0.317304
hsa_circ_	-0.2089	0.317312
hsa_circ_	0.16104	0.317313
hsa_circ_	0.273425	0.317317
hsa_circ_	-0.20282	0.317325
hsa_circ_	0.303501	0.31733
hsa_circ_	-0.2918	0.317334
hsa_circ_	-0.13415	0.317334
hsa_circ_	0.154026	0.317358
hsa_circ_	-0.2904	0.317378
hsa_circ_	0.233747	0.317381
hsa_circ_	-0.19279	0.317386
hsa_circ_	0.24918	0.3174
hsa_circ_	-0.13515	0.317401
hsa_circ_	0.400069	0.317412
hsa_circ_	0.172874	0.317417
hsa_circ_	-0.17222	0.317431
hsa_circ_	0.264734	0.317448
hsa_circ_	0.591224	0.317453
hsa_circ_	-0.83527	0.317454
hsa_circ_	-0.38475	0.317462
hsa_circ_	-0.14357	0.317483
hsa_circ_	-0.1984	0.317494
hsa_circ_	0.266787	0.317513
hsa_circ_	0.511401	0.317532
hsa_circ_	0.225307	0.317546
hsa_circ_	0.305203	0.31756
hsa_circ_	0.282659	0.317565
hsa_circ_	0.359173	0.317576
hsa_circ_	-0.16724	0.317577
hsa_circ_	0.552412	0.317641
hsa_circ_	-0.13133	0.317655
hsa_circ_	-0.18705	0.31767
hsa_circ_	0.496496	0.317674
hsa_circ_	-0.21053	0.317687
hsa_circ_	-0.58528	0.317711
hsa_circ_	-0.29532	0.317713
hsa_circ_	0.179438	0.317748
hsa_circ_	-0.20649	0.31775
hsa_circ_	0.298729	0.31775
hsa_circ_	-0.34334	0.317776
hsa_circ_	-0.20229	0.317777
hsa_circ_	0.193568	0.317786
hsa_circ_	0.299251	0.317796
hsa_circ_	0.222613	0.317813
hsa_circ_	-0.20788	0.317819
hsa_circ_	0.159185	0.317821
hsa_circ_	-0.3952	0.317824

hsa_circ_	-0.18723	0.317827
hsa_circ_	-0.30321	0.317838
hsa_circ_	0.183996	0.317841
hsa_circ_	-0.27102	0.317874
hsa_circ_	0.437301	0.317876
hsa_circ_	0.141125	0.317876
hsa_circ_	0.279348	0.317879
hsa_circ_	0.54494	0.317881
hsa_circ_	-0.50286	0.317884
hsa_circ_	0.457579	0.317892
hsa_circ_	0.180961	0.317903
hsa_circ_	0.195006	0.317919
hsa_circ_	-0.31237	0.317925
hsa_circ_	-0.70047	0.317946
hsa_circ_	-0.16449	0.317957
hsa_circ_	0.295597	0.317976
hsa_circ_	0.1691	0.317981
hsa_circ_	-0.189	0.317997
hsa_circ_	0.440919	0.318006
hsa_circ_	-0.34236	0.318017
hsa_circ_	-0.14973	0.318025
hsa_circ_	-0.27132	0.318037
hsa_circ_	0.168922	0.318043
hsa_circ_	-0.17429	0.318047
hsa_circ_	0.252192	0.318056
hsa_circ_	0.282614	0.318065
hsa_circ_	0.47793	0.31807
hsa_circ_	-0.4472	0.318074
hsa_circ_	-0.13184	0.318085
hsa_circ_	0.20032	0.318086
hsa_circ_	0.146238	0.318091
hsa_circ_	0.153545	0.318096
hsa_circ_	0.271023	0.318119
hsa_circ_	0.148015	0.318128
hsa_circ_	-0.20807	0.31813
hsa_circ_	0.424621	0.318176
hsa_circ_	-0.19413	0.318186
hsa_circ_	-0.35694	0.318194
hsa_circ_	-0.18924	0.318215
hsa_circ_	0.209715	0.318218
hsa_circ_	0.19294	0.318226
hsa_circ_	0.292868	0.318234
hsa_circ_	0.182293	0.318258
hsa_circ_	0.315278	0.31826
hsa_circ_	-0.27205	0.31826
hsa_circ_	0.38038	0.318271
hsa_circ_	-0.24877	0.318271
hsa_circ_	0.23745	0.318281
hsa_circ_	-0.19856	0.318294
hsa_circ_	-0.17968	0.31831
hsa_circ_	-0.30783	0.31832
hsa_circ_	-0.25152	0.318337
hsa_circ_	-0.1477	0.318339
hsa_circ_	-0.31311	0.318345

hsa_circ_	-0.22878	0.31835
hsa_circ_	0.927789	0.318357
hsa_circ_	-0.21002	0.318362
hsa_circ_	-0.35165	0.318378
hsa_circ_	-0.16531	0.318392
hsa_circ_	-0.19773	0.318394
hsa_circ_	-0.28159	0.318397
hsa_circ_	0.14244	0.318401
hsa_circ_	0.177111	0.318402
hsa_circ_	-0.18218	0.318407
hsa_circ_	0.21213	0.318409
hsa_circ_	-1.38103	0.31841
hsa_circ_	-0.52157	0.31841
hsa_circ_	-0.40786	0.318422
hsa_circ_	-0.22312	0.31843
hsa_circ_	-0.15635	0.318436
hsa_circ_	0.163975	0.318439
hsa_circ_	-0.2715	0.318475
hsa_circ_	0.212839	0.318477
hsa_circ_	-0.16031	0.318479
hsa_circ_	-0.2515	0.318493
hsa_circ_	-0.16363	0.318498
hsa_circ_	-0.39317	0.318502
hsa_circ_	0.676431	0.31853
hsa_circ_	-0.21915	0.318547
hsa_circ_	-0.40544	0.318561
hsa_circ_	0.327424	0.318575
hsa_circ_	-0.39555	0.318587
hsa_circ_	0.336024	0.318601
hsa_circ_	-0.24838	0.318605
hsa_circ_	0.194802	0.318605
hsa_circ_	-0.49967	0.318615
hsa_circ_	-0.56179	0.318624
hsa_circ_	-0.19672	0.318639
hsa_circ_	0.153376	0.318644
hsa_circ_	-0.22518	0.318646
hsa_circ_	0.177412	0.318648
hsa_circ_	-0.24837	0.318658
hsa_circ_	-0.21961	0.318673
hsa_circ_	-0.18039	0.318705
hsa_circ_	0.301128	0.318718
hsa_circ_	-0.20573	0.318729
hsa_circ_	-0.20385	0.318738
hsa_circ_	0.161445	0.318739
hsa_circ_	-0.37609	0.318755
hsa_circ_	-0.16948	0.318805
hsa_circ_	0.289642	0.31883
hsa_circ_	-0.2689	0.31883
hsa_circ_	-0.23305	0.318836
hsa_circ_	0.688418	0.318839
hsa_circ_	0.335117	0.318851
hsa_circ_	-0.2934	0.318858
hsa_circ_	-0.13452	0.318862
hsa_circ_	-0.20431	0.318869

hsa_circ_	-0.20714	0.318871
hsa_circ_	-0.20003	0.318872
hsa_circ_	0.17871	0.318891
hsa_circ_	-0.16869	0.318914
hsa_circ_	0.254934	0.31893
hsa_circ_	-0.23205	0.318941
hsa_circ_	0.19472	0.318957
hsa_circ_	0.25	0.318968
hsa_circ_	-0.49027	0.318984
hsa_circ_	-0.37611	0.318993
hsa_circ_	-0.14387	0.318999
hsa_circ_	-0.18896	0.31901
hsa_circ_	-0.21334	0.319022
hsa_circ_	-0.14333	0.319037
hsa_circ_	0.366508	0.319044
hsa_circ_	-0.2676	0.319067
hsa_circ_	-0.17918	0.319077
hsa_circ_	-0.23515	0.319117
hsa_circ_	-0.17503	0.319117
hsa_circ_	-0.21232	0.319142
hsa_circ_	0.662292	0.319154
hsa_circ_	0.227698	0.319165
hsa_circ_	0.325452	0.319166
hsa_circ_	0.24252	0.319171
hsa_circ_	-0.17818	0.319174
hsa_circ_	0.160288	0.319202
hsa_circ_	-0.29546	0.319204
hsa_circ_	0.183397	0.319205
hsa_circ_	-0.43307	0.319211
hsa_circ_	0.171447	0.319243
hsa_circ_	-0.24732	0.319263
hsa_circ_	0.156233	0.319294
hsa_circ_	0.522203	0.3193
hsa_circ_	-0.19283	0.319301
hsa_circ_	0.175998	0.319302
hsa_circ_	-0.14027	0.319308
hsa_circ_	0.198243	0.31933
hsa_circ_	0.375854	0.319333
hsa_circ_	-0.14592	0.319335
hsa_circ_	-0.22726	0.319351
hsa_circ_	-0.732	0.319358
hsa_circ_	0.245319	0.31936
hsa_circ_	-0.29349	0.319386
hsa_circ_	-0.47302	0.319394
hsa_circ_	-0.22641	0.319414
hsa_circ_	0.228693	0.319419
hsa_circ_	-0.89349	0.319433
hsa_circ_	0.40513	0.319449
hsa_circ_	-0.37113	0.319465
hsa_circ_	-0.17583	0.319478
hsa_circ_	0.204356	0.319482
hsa_circ_	-0.18473	0.319489
hsa_circ_	0.295048	0.319492
hsa_circ_	0.15968	0.319496

hsa_circ_	-0.36184	0.319501
hsa_circ_	-0.27599	0.319503
hsa_circ_	-0.40078	0.319505
hsa_circ_	0.540498	0.319524
hsa_circ_	0.14567	0.319528
hsa_circ_	0.164609	0.319531
hsa_circ_	0.16006	0.319559
hsa_circ_	-0.23753	0.319565
hsa_circ_	-0.13277	0.319569
hsa_circ_	0.635946	0.319573
hsa_circ_	0.52706	0.319576
hsa_circ_	0.232002	0.319585
hsa_circ_	0.19448	0.319588
hsa_circ_	-0.46842	0.319592
hsa_circ_	-0.15434	0.319595
hsa_circ_	-0.15006	0.319603
hsa_circ_	0.235438	0.319629
hsa_circ_	0.321919	0.319656
hsa_circ_	0.181926	0.319674
hsa_circ_	0.153058	0.319677
hsa_circ_	-0.40514	0.319678
hsa_circ_	0.163422	0.319689
hsa_circ_	-0.42963	0.319704
hsa_circ_	-0.24562	0.319708
hsa_circ_	-0.1706	0.31973
hsa_circ_	0.227382	0.319764
hsa_circ_	-1.015	0.319826
hsa_circ_	-0.19817	0.319829
hsa_circ_	0.368022	0.319832
hsa_circ_	0.291523	0.319833
hsa_circ_	-0.17394	0.319843
hsa_circ_	-0.21706	0.319851
hsa_circ_	-0.38061	0.319851
hsa_circ_	0.204475	0.319854
hsa_circ_	0.275135	0.319865
hsa_circ_	-0.28542	0.319879
hsa_circ_	-0.17232	0.319897
hsa_circ_	-0.1355	0.319925
hsa_circ_	0.137269	0.319943
hsa_circ_	-0.20523	0.31998
hsa_circ_	-0.24309	0.319981
hsa_circ_	-0.2422	0.319992
hsa_circ_	-0.12902	0.319994
hsa_circ_	-0.16419	0.320056
hsa_circ_	0.246042	0.320062
hsa_circ_	0.221247	0.320071
hsa_circ_	0.556613	0.320074
hsa_circ_	-0.20959	0.320092
hsa_circ_	0.176355	0.320126
hsa_circ_	-0.14359	0.320143
hsa_circ_	0.365703	0.320144
hsa_circ_	0.191303	0.320146
hsa_circ_	0.265248	0.320157
hsa_circ_	-0.13199	0.320157

hsa_circ_	-0.23543	0.320165
hsa_circ_	0.393755	0.320169
hsa_circ_	0.220481	0.3202
hsa_circ_	-0.2037	0.320208
hsa_circ_	-0.36436	0.320209
hsa_circ_	0.370486	0.320252
hsa_circ_	-0.20654	0.320253
hsa_circ_	0.158263	0.320268
hsa_circ_	0.226458	0.320275
hsa_circ_	0.20645	0.320299
hsa_circ_	0.168226	0.320305
hsa_circ_	0.363284	0.320312
hsa_circ_	0.150562	0.320336
hsa_circ_	0.13268	0.320336
hsa_circ_	0.547986	0.320357
hsa_circ_	0.161476	0.320369
hsa_circ_	-0.28399	0.320372
hsa_circ_	-0.20235	0.320375
hsa_circ_	-0.30619	0.320385
hsa_circ_	-0.29261	0.3204
hsa_circ_	0.194395	0.32044
hsa_circ_	-0.1892	0.320448
hsa_circ_	0.272116	0.320455
hsa_circ_	0.537406	0.320487
hsa_circ_	-0.2479	0.32052
hsa_circ_	0.156448	0.320527
hsa_circ_	0.367384	0.320527
hsa_circ_	-0.22637	0.320563
hsa_circ_	0.247115	0.320564
hsa_circ_	0.515548	0.320566
hsa_circ_	0.182152	0.320569
hsa_circ_	-0.53774	0.320573
hsa_circ_	-0.24086	0.320593
hsa_circ_	0.240688	0.320632
hsa_circ_	-0.41002	0.320637
hsa_circ_	-0.18383	0.320656
hsa_circ_	-0.16946	0.320671
hsa_circ_	0.247665	0.320679
hsa_circ_	-0.3523	0.32069
hsa_circ_	-0.24551	0.320697
hsa_circ_	0.262617	0.320704
hsa_circ_	0.790181	0.320724
hsa_circ_	0.196527	0.320729
hsa_circ_	0.236703	0.320742
hsa_circ_	0.484276	0.32076
hsa_circ_	-0.24734	0.320774
hsa_circ_	0.300562	0.320794
hsa_circ_	0.16335	0.320802
hsa_circ_	0.275899	0.320822
hsa_circ_	0.554324	0.320822
hsa_circ_	-0.28268	0.320833
hsa_circ_	-0.99651	0.320854
hsa_circ_	0.393256	0.320864
hsa_circ_	-0.20069	0.320904

hsa_circ_	0.265425	0.32091
hsa_circ_	-0.16482	0.32091
hsa_circ_	-0.19274	0.320914
hsa_circ_	-0.14232	0.320941
hsa_circ_	0.194508	0.320942
hsa_circ_	0.197555	0.320942
hsa_circ_	-0.16396	0.320943
hsa_circ_	-0.15152	0.320962
hsa_circ_	-0.31516	0.320985
hsa_circ_	-0.19928	0.320993
hsa_circ_	0.340088	0.320994
hsa_circ_	-0.16625	0.320996
hsa_circ_	-0.48072	0.321017
hsa_circ_	-0.31736	0.321041
hsa_circ_	-0.25153	0.321045
hsa_circ_	0.375233	0.321054
hsa_circ_	0.227805	0.321072
hsa_circ_	0.200246	0.321079
hsa_circ_	0.136135	0.321084
hsa_circ_	-0.17683	0.321092
hsa_circ_	-0.21316	0.3211
hsa_circ_	0.162527	0.321118
hsa_circ_	0.405581	0.321134
hsa_circ_	-0.23269	0.321188
hsa_circ_	0.270217	0.321189
hsa_circ_	0.167019	0.321199
hsa_circ_	-0.27422	0.321201
hsa_circ_	0.591576	0.321217
hsa_circ_	0.176854	0.321224
hsa_circ_	-0.2637	0.321248
hsa_circ_	0.174039	0.321275
hsa_circ_	-0.26619	0.321276
hsa_circ_	-0.17962	0.321291
hsa_circ_	-0.33517	0.321292
hsa_circ_	0.285128	0.32132
hsa_circ_	-0.19214	0.321328
hsa_circ_	0.213061	0.321339
hsa_circ_	-0.15693	0.321342
hsa_circ_	0.133601	0.321355
hsa_circ_	0.14633	0.321356
hsa_circ_	-0.19183	0.321372
hsa_circ_	-0.25961	0.321378
hsa_circ_	-0.66732	0.32138
hsa_circ_	0.294849	0.321391
hsa_circ_	0.190617	0.321391
hsa_circ_	-0.16449	0.321406
hsa_circ_	0.254149	0.321408
hsa_circ_	-0.50274	0.321436
hsa_circ_	-0.26535	0.321442
hsa_circ_	0.283682	0.321444
hsa_circ_	-0.16714	0.321472
hsa_circ_	0.630388	0.321478
hsa_circ_	-0.1643	0.321482
hsa_circ_	0.257472	0.3215

hsa_circ_	0.249693	0.3215
hsa_circ_	0.269761	0.321527
hsa_circ_	0.336574	0.321527
hsa_circ_	0.535274	0.321541
hsa_circ_	-0.20132	0.321549
hsa_circ_	-0.174	0.321555
hsa_circ_	0.147637	0.321578
hsa_circ_	-0.56372	0.321615
hsa_circ_	-0.15449	0.321618
hsa_circ_	-0.18237	0.321628
hsa_circ_	-0.20046	0.32163
hsa_circ_	-0.21005	0.321631
hsa_circ_	-0.17874	0.321641
hsa_circ_	-0.33653	0.321645
hsa_circ_	0.190526	0.321683
hsa_circ_	0.186705	0.321683
hsa_circ_	0.151717	0.321697
hsa_circ_	0.384688	0.321699
hsa_circ_	-0.55017	0.321703
hsa_circ_	0.346044	0.321715
hsa_circ_	-0.27996	0.321728
hsa_circ_	0.184453	0.32178
hsa_circ_	-0.14164	0.321786
hsa_circ_	0.459951	0.321788
hsa_circ_	-0.24647	0.32179
hsa_circ_	-0.12951	0.321807
hsa_circ_	-0.41687	0.32181
hsa_circ_	0.268031	0.321811
hsa_circ_	0.203884	0.321816
hsa_circ_	0.139094	0.32183
hsa_circ_	-0.63735	0.321835
hsa_circ_	0.260709	0.321835
hsa_circ_	0.231254	0.321866
hsa_circ_	0.274691	0.321874
hsa_circ_	0.189924	0.321891
hsa_circ_	0.190191	0.321895
hsa_circ_	-0.29406	0.321902
hsa_circ_	-1.08784	0.321908
hsa_circ_	0.177724	0.321931
hsa_circ_	0.354656	0.321941
hsa_circ_	0.249174	0.321942
hsa_circ_	0.251025	0.321946
hsa_circ_	0.169958	0.32197
hsa_circ_	0.35538	0.321976
hsa_circ_	0.254443	0.321996
hsa_circ_	-0.42334	0.321999
hsa_circ_	-0.22266	0.322015
hsa_circ_	0.16456	0.322028
hsa_circ_	-0.21697	0.322031
hsa_circ_	-0.191	0.322032
hsa_circ_	0.511372	0.322041
hsa_circ_	0.178152	0.322059
hsa_circ_	0.208193	0.322061
hsa_circ_	-0.14801	0.322076

hsa_circ_	0.211998	0.322105
hsa_circ_	0.164647	0.322125
hsa_circ_	0.286722	0.322126
hsa_circ_	-0.12869	0.32215
hsa_circ_	-0.18562	0.322164
hsa_circ_	0.783903	0.322165
hsa_circ_	0.140568	0.322178
hsa_circ_	0.216874	0.322231
hsa_circ_	0.312849	0.32224
hsa_circ_	0.552893	0.322288
hsa_circ_	0.218657	0.322333
hsa_circ_	0.152635	0.322359
hsa_circ_	-0.21935	0.32237
hsa_circ_	-0.37085	0.322378
hsa_circ_	-0.18149	0.322381
hsa_circ_	-0.23267	0.322382
hsa_circ_	0.216183	0.322384
hsa_circ_	0.269367	0.322384
hsa_circ_	-0.28507	0.322387
hsa_circ_	0.546478	0.322397
hsa_circ_	-0.15986	0.322397
hsa_circ_	0.153729	0.322404
hsa_circ_	-0.1886	0.322416
hsa_circ_	0.286459	0.322424
hsa_circ_	-0.18336	0.322444
hsa_circ_	0.197911	0.322447
hsa_circ_	-0.22492	0.322461
hsa_circ_	0.378848	0.322464
hsa_circ_	0.168997	0.322499
hsa_circ_	0.134909	0.32252
hsa_circ_	0.228715	0.322522
hsa_circ_	0.697153	0.322533
hsa_circ_	0.189661	0.322536
hsa_circ_	-0.28987	0.32254
hsa_circ_	-0.19561	0.32254
hsa_circ_	0.2505	0.322545
hsa_circ_	0.174962	0.32255
hsa_circ_	-0.16787	0.322567
hsa_circ_	0.461975	0.322622
hsa_circ_	-0.19038	0.322631
hsa_circ_	-0.25477	0.322638
hsa_circ_	0.161289	0.322642
hsa_circ_	0.218308	0.322645
hsa_circ_	-0.26826	0.322652
hsa_circ_	0.426844	0.322662
hsa_circ_	-0.27312	0.322663
hsa_circ_	0.910484	0.322685
hsa_circ_	0.144673	0.322699
hsa_circ_	0.730169	0.32271
hsa_circ_	-0.26265	0.322712
hsa_circ_	-0.20121	0.322727
hsa_circ_	-0.50234	0.322728
hsa_circ_	0.212229	0.322742
hsa_circ_	0.159187	0.322743

hsa_circ_	0.267298	0.32276
hsa_circ_	0.502836	0.322771
hsa_circ_	-0.1489	0.322781
hsa_circ_	0.284912	0.322781
hsa_circ_	0.21004	0.322798
hsa_circ_	-0.30036	0.322799
hsa_circ_	0.30283	0.322844
hsa_circ_	-0.39224	0.322872
hsa_circ_	-0.19254	0.322883
hsa_circ_	0.490281	0.322906
hsa_circ_	-0.1614	0.322912
hsa_circ_	0.517773	0.322914
hsa_circ_	0.194256	0.322929
hsa_circ_	-0.51829	0.322951
hsa_circ_	-0.22303	0.322962
hsa_circ_	0.181823	0.322964
hsa_circ_	-0.36026	0.322968
hsa_circ_	0.184891	0.322991
hsa_circ_	-0.14462	0.322992
hsa_circ_	-0.34279	0.323
hsa_circ_	0.209322	0.323011
hsa_circ_	0.726972	0.323018
hsa_circ_	-0.14112	0.323022
hsa_circ_	0.156211	0.323025
hsa_circ_	0.199966	0.323032
hsa_circ_	-0.32732	0.323076
hsa_circ_	0.672564	0.323076
hsa_circ_	-0.2329	0.323098
hsa_circ_	0.272913	0.323128
hsa_circ_	0.367249	0.323145
hsa_circ_	0.147041	0.323176
hsa_circ_	-0.26796	0.323187
hsa_circ_	-0.20362	0.323207
hsa_circ_	0.158202	0.323218
hsa_circ_	-0.42143	0.323221
hsa_circ_	-0.29957	0.323227
hsa_circ_	0.197646	0.323235
hsa_circ_	0.220658	0.323239
hsa_circ_	-0.33203	0.323263
hsa_circ_	-0.23667	0.323284
hsa_circ_	-0.17599	0.323295
hsa_circ_	0.169487	0.323302
hsa_circ_	0.158281	0.323303
hsa_circ_	0.605384	0.323307
hsa_circ_	-0.14044	0.323335
hsa_circ_	0.203268	0.323339
hsa_circ_	0.322169	0.323349
hsa_circ_	-0.14791	0.323352
hsa_circ_	0.159812	0.323358
hsa_circ_	0.285359	0.323363
hsa_circ_	0.189974	0.323367
hsa_circ_	-0.76914	0.323387
hsa_circ_	0.211195	0.323404
hsa_circ_	-1.67142	0.323408

hsa_circ_	-0.28449	0.323414
hsa_circ_	-0.25873	0.323427
hsa_circ_	-0.28058	0.323431
hsa_circ_	0.394365	0.32344
hsa_circ_	-0.19148	0.323453
hsa_circ_	-0.39026	0.323456
hsa_circ_	0.261979	0.323465
hsa_circ_	0.269308	0.323465
hsa_circ_	0.321593	0.32347
hsa_circ_	-0.15177	0.323471
hsa_circ_	-0.21697	0.323481
hsa_circ_	0.429733	0.323484
hsa_circ_	-0.23081	0.3235
hsa_circ_	-0.14241	0.323503
hsa_circ_	0.209882	0.323506
hsa_circ_	-0.27575	0.323507
hsa_circ_	-0.16283	0.323508
hsa_circ_	-0.32972	0.323531
hsa_circ_	-0.2749	0.323541
hsa_circ_	0.17069	0.323545
hsa_circ_	-0.40848	0.323567
hsa_circ_	-0.1938	0.323592
hsa_circ_	-0.77056	0.323592
hsa_circ_	0.202297	0.323596
hsa_circ_	0.555992	0.323599
hsa_circ_	0.180198	0.323606
hsa_circ_	-0.18277	0.323611
hsa_circ_	-0.17382	0.323616
hsa_circ_	0.19063	0.323634
hsa_circ_	0.185294	0.323654
hsa_circ_	-0.22548	0.323655
hsa_circ_	-0.54642	0.32366
hsa_circ_	-0.372	0.323663
hsa_circ_	0.210007	0.323669
hsa_circ_	0.547478	0.32369
hsa_circ_	0.613446	0.323702
hsa_circ_	-0.1449	0.323714
hsa_circ_	0.186431	0.32374
hsa_circ_	0.18139	0.323754
hsa_circ_	0.311972	0.323755
hsa_circ_	-0.16057	0.323778
hsa_circ_	0.184193	0.323779
hsa_circ_	0.230197	0.323786
hsa_circ_	0.56045	0.32379
hsa_circ_	0.254086	0.323805
hsa_circ_	0.297696	0.32381
hsa_circ_	0.154771	0.323811
hsa_circ_	-0.15859	0.323813
hsa_circ_	-0.14446	0.323848
hsa_circ_	-0.15719	0.323855
hsa_circ_	-0.30008	0.323861
hsa_circ_	-0.55451	0.323864
hsa_circ_	-0.36187	0.323866
hsa_circ_	0.285351	0.323876

hsa_circ_	0.158089	0.323883
hsa_circ_	-0.22137	0.323886
hsa_circ_	-0.37031	0.323895
hsa_circ_	-0.72176	0.323907
hsa_circ_	0.483042	0.323908
hsa_circ_	0.153976	0.323909
hsa_circ_	0.170823	0.323911
hsa_circ_	-0.1431	0.323938
hsa_circ_	-0.24085	0.323939
hsa_circ_	0.179228	0.32394
hsa_circ_	0.260823	0.323969
hsa_circ_	-0.16901	0.323978
hsa_circ_	0.241843	0.323993
hsa_circ_	0.432807	0.323994
hsa_circ_	-0.21578	0.324014
hsa_circ_	-0.18228	0.32402
hsa_circ_	0.449435	0.324051
hsa_circ_	0.239501	0.324056
hsa_circ_	-0.20284	0.324063
hsa_circ_	-0.15248	0.324076
hsa_circ_	-0.33188	0.324112
hsa_circ_	-0.1621	0.324127
hsa_circ_	-0.13737	0.324147
hsa_circ_	0.243994	0.324149
hsa_circ_	-0.42018	0.324167
hsa_circ_	-0.29493	0.324171
hsa_circ_	-0.41023	0.324184
hsa_circ_	0.141611	0.324188
hsa_circ_	-0.13721	0.324192
hsa_circ_	-0.27144	0.324222
hsa_circ_	-0.15706	0.324274
hsa_circ_	-0.57051	0.324276
hsa_circ_	-0.2781	0.324279
hsa_circ_	-0.39453	0.324282
hsa_circ_	0.18768	0.324293
hsa_circ_	0.3478	0.324339
hsa_circ_	0.412475	0.324347
hsa_circ_	0.307506	0.324374
hsa_circ_	-0.40239	0.324379
hsa_circ_	-0.1562	0.32439
hsa_circ_	0.24178	0.324391
hsa_circ_	-0.16354	0.324398
hsa_circ_	0.154382	0.324401
hsa_circ_	0.343818	0.324415
hsa_circ_	0.191233	0.324441
hsa_circ_	-0.17307	0.324457
hsa_circ_	0.221125	0.324469
hsa_circ_	0.280581	0.324493
hsa_circ_	0.716376	0.324496
hsa_circ_	-0.50877	0.324505
hsa_circ_	0.38171	0.32452
hsa_circ_	0.485851	0.32452
hsa_circ_	-0.39923	0.324522
hsa_circ_	-0.22437	0.324546

hsa_circ_	0.229596	0.324552
hsa_circ_	-1.01163	0.324571
hsa_circ_	-0.14904	0.324602
hsa_circ_	-0.2477	0.324628
hsa_circ_	0.180767	0.324647
hsa_circ_	-0.55267	0.324685
hsa_circ_	-0.19133	0.324689
hsa_circ_	-0.21459	0.324697
hsa_circ_	0.199896	0.32471
hsa_circ_	-0.17645	0.324719
hsa_circ_	-0.475	0.324731
hsa_circ_	0.652031	0.324731
hsa_circ_	0.216607	0.324736
hsa_circ_	-0.23306	0.324746
hsa_circ_	-0.25551	0.324747
hsa_circ_	0.33207	0.324757
hsa_circ_	0.207188	0.324826
hsa_circ_	0.141534	0.324828
hsa_circ_	0.205639	0.32483
hsa_circ_	-0.15067	0.324853
hsa_circ_	0.213017	0.324856
hsa_circ_	0.44003	0.324859
hsa_circ_	0.160876	0.324878
hsa_circ_	0.171497	0.324901
hsa_circ_	0.262739	0.32492
hsa_circ_	-0.43079	0.324937
hsa_circ_	0.634499	0.324957
hsa_circ_	0.374344	0.324994
hsa_circ_	-0.15459	0.325013
hsa_circ_	0.194254	0.325079
hsa_circ_	0.82198	0.325083
hsa_circ_	0.312469	0.325092
hsa_circ_	0.518286	0.325098
hsa_circ_	0.230003	0.325099
hsa_circ_	-0.14544	0.325102
hsa_circ_	0.326223	0.325109
hsa_circ_	-0.28924	0.325113
hsa_circ_	-0.5405	0.325117
hsa_circ_	-0.17276	0.325121
hsa_circ_	-0.15025	0.325121
hsa_circ_	-0.22921	0.325131
hsa_circ_	-0.13725	0.325148
hsa_circ_	0.146825	0.325161
hsa_circ_	0.148559	0.325175
hsa_circ_	-1.09066	0.325181
hsa_circ_	-0.17332	0.325187
hsa_circ_	0.499155	0.32519
hsa_circ_	0.255729	0.325205
hsa_circ_	0.200122	0.325206
hsa_circ_	0.183125	0.325209
hsa_circ_	-0.16716	0.325227
hsa_circ_	0.216465	0.325238
hsa_circ_	0.1684	0.325238
hsa_circ_	0.427865	0.325241

hsa_circ_	0.333561	0.325249
hsa_circ_	-0.43851	0.325252
hsa_circ_	-0.21422	0.325265
hsa_circ_	0.533218	0.325271
hsa_circ_	-0.2386	0.32528
hsa_circ_	-0.66274	0.325293
hsa_circ_	-0.20019	0.325301
hsa_circ_	-0.1398	0.325304
hsa_circ_	0.233145	0.325311
hsa_circ_	-0.16575	0.325329
hsa_circ_	-0.1646	0.325346
hsa_circ_	0.194024	0.325378
hsa_circ_	-0.21421	0.325402
hsa_circ_	-0.16715	0.325427
hsa_circ_	-0.18688	0.325468
hsa_circ_	0.234328	0.325472
hsa_circ_	-0.25457	0.325474
hsa_circ_	-0.36508	0.325474
hsa_circ_	-0.30305	0.325493
hsa_circ_	-0.16283	0.325509
hsa_circ_	0.152192	0.325549
hsa_circ_	0.159708	0.325581
hsa_circ_	0.312128	0.325586
hsa_circ_	-0.21741	0.325587
hsa_circ_	-0.37582	0.325631
hsa_circ_	-0.56616	0.325639
hsa_circ_	-0.24034	0.325645
hsa_circ_	-0.18926	0.325672
hsa_circ_	0.237959	0.325677
hsa_circ_	-0.18917	0.325684
hsa_circ_	-0.13583	0.325699
hsa_circ_	-0.34457	0.325709
hsa_circ_	-0.26464	0.325715
hsa_circ_	0.249322	0.325716
hsa_circ_	-0.44151	0.325716
hsa_circ_	-0.19075	0.325725
hsa_circ_	-0.17412	0.325745
hsa_circ_	-0.20556	0.325746
hsa_circ_	0.161175	0.325756
hsa_circ_	0.135489	0.325765
hsa_circ_	-0.21025	0.325788
hsa_circ_	0.169844	0.325792
hsa_circ_	0.41716	0.325811
hsa_circ_	0.200756	0.325835
hsa_circ_	-0.21717	0.325839
hsa_circ_	0.240552	0.325876
hsa_circ_	-0.38382	0.32588
hsa_circ_	-0.17336	0.325886
hsa_circ_	0.201157	0.325896
hsa_circ_	-0.43269	0.325898
hsa_circ_	0.446024	0.3259
hsa_circ_	-0.1828	0.325912
hsa_circ_	-0.37561	0.325915
hsa_circ_	-0.13613	0.325921

hsa_circ_	0.452986	0.325939
hsa_circ_	-0.15359	0.325945
hsa_circ_	-0.31457	0.325952
hsa_circ_	0.474587	0.325956
hsa_circ_	0.367071	0.325959
hsa_circ_	0.341695	0.325969
hsa_circ_	0.176357	0.325991
hsa_circ_	0.547472	0.325991
hsa_circ_	-0.31747	0.325994
hsa_circ_	0.201976	0.326011
hsa_circ_	0.158869	0.326014
hsa_circ_	0.281437	0.326019
hsa_circ_	0.138915	0.326033
hsa_circ_	0.503272	0.326037
hsa_circ_	0.14396	0.326045
hsa_circ_	-0.40583	0.326049
hsa_circ_	-0.2166	0.326051
hsa_circ_	0.197126	0.326059
hsa_circ_	-0.13333	0.326067
hsa_circ_	-0.15765	0.326083
hsa_circ_	0.465865	0.326092
hsa_circ_	-0.24467	0.326126
hsa_circ_	-0.1869	0.326127
hsa_circ_	0.133826	0.326137
hsa_circ_	-0.25966	0.326145
hsa_circ_	-0.1356	0.326156
hsa_circ_	-1.37194	0.32616
hsa_circ_	0.231861	0.326173
hsa_circ_	0.188949	0.32622
hsa_circ_	-0.26435	0.326223
hsa_circ_	-0.21234	0.326241
hsa_circ_	-0.17148	0.326249
hsa_circ_	-0.17822	0.32626
hsa_circ_	0.138137	0.32626
hsa_circ_	0.557463	0.326283
hsa_circ_	-0.41244	0.326291
hsa_circ_	-0.19435	0.326301
hsa_circ_	-0.19155	0.326302
hsa_circ_	-0.33729	0.326309
hsa_circ_	-0.18368	0.326315
hsa_circ_	0.198711	0.326317
hsa_circ_	-0.30019	0.326322
hsa_circ_	0.188922	0.326344
hsa_circ_	0.293316	0.326347
hsa_circ_	-0.202	0.326358
hsa_circ_	-0.15649	0.326366
hsa_circ_	-0.22004	0.326409
hsa_circ_	-0.46867	0.326421
hsa_circ_	-0.23782	0.32644
hsa_circ_	-0.25493	0.326455
hsa_circ_	-0.23458	0.326462
hsa_circ_	-0.20669	0.326463
hsa_circ_	0.213419	0.326463
hsa_circ_	-0.18331	0.326466

hsa_circ_	0.1915	0.326488
hsa_circ_	0.220511	0.326509
hsa_circ_	-0.42656	0.326513
hsa_circ_	0.180746	0.32652
hsa_circ_	-0.31258	0.326524
hsa_circ_	-0.13277	0.326524
hsa_circ_	0.152659	0.326531
hsa_circ_	0.16913	0.326551
hsa_circ_	-0.1441	0.32659
hsa_circ_	-0.13079	0.326598
hsa_circ_	-0.41464	0.326611
hsa_circ_	0.173814	0.326621
hsa_circ_	0.31592	0.326623
hsa_circ_	-0.18356	0.326623
hsa_circ_	-0.18451	0.326638
hsa_circ_	0.35079	0.326696
hsa_circ_	-0.1691	0.326698
hsa_circ_	-0.3713	0.326699
hsa_circ_	-0.19965	0.326711
hsa_circ_	0.227076	0.326717
hsa_circ_	0.140657	0.326718
hsa_circ_	-0.24374	0.32672
hsa_circ_	-0.29992	0.326722
hsa_circ_	-0.16479	0.326733
hsa_circ_	0.417446	0.326749
hsa_circ_	-0.1985	0.326766
hsa_circ_	0.236816	0.326787
hsa_circ_	-0.16736	0.32683
hsa_circ_	0.230401	0.326861
hsa_circ_	-0.2449	0.326861
hsa_circ_	0.152522	0.326868
hsa_circ_	-0.13497	0.326904
hsa_circ_	0.29278	0.326906
hsa_circ_	-0.17672	0.326922
hsa_circ_	-2.12861	0.326926
hsa_circ_	-0.24261	0.326938
hsa_circ_	0.309128	0.326942
hsa_circ_	-0.29066	0.32695
hsa_circ_	0.194818	0.326955
hsa_circ_	0.24421	0.326971
hsa_circ_	-0.24896	0.32698
hsa_circ_	0.144237	0.326994
hsa_circ_	0.453317	0.327
hsa_circ_	0.173213	0.327005
hsa_circ_	0.593057	0.327022
hsa_circ_	0.417653	0.32703
hsa_circ_	-0.17716	0.327047
hsa_circ_	-1.39304	0.327053
hsa_circ_	-0.18234	0.327081
hsa_circ_	0.347088	0.32715
hsa_circ_	-0.62262	0.327152
hsa_circ_	0.18004	0.327164
hsa_circ_	0.595686	0.327164
hsa_circ_	-0.29821	0.327195

hsa_circ_	0.162235	0.3272
hsa_circ_	0.163175	0.3272
hsa_circ_	0.457688	0.327205
hsa_circ_	0.217282	0.327208
hsa_circ_	-0.18445	0.327213
hsa_circ_	-0.17675	0.327221
hsa_circ_	0.276294	0.327254
hsa_circ_	0.156772	0.327273
hsa_circ_	-0.15245	0.327276
hsa_circ_	-0.16295	0.327292
hsa_circ_	0.298087	0.327298
hsa_circ_	0.162128	0.327306
hsa_circ_	0.138554	0.327307
hsa_circ_	-0.38312	0.327309
hsa_circ_	-0.77901	0.32731
hsa_circ_	0.292352	0.327332
hsa_circ_	-0.19509	0.327336
hsa_circ_	-0.36869	0.327386
hsa_circ_	-0.17853	0.327391
hsa_circ_	0.432774	0.327396
hsa_circ_	0.179349	0.327396
hsa_circ_	-0.3313	0.327413
hsa_circ_	-0.17173	0.327458
hsa_circ_	-0.13996	0.327463
hsa_circ_	-0.14042	0.327482
hsa_circ_	0.330502	0.32749
hsa_circ_	-0.20419	0.327502
hsa_circ_	0.157321	0.327556
hsa_circ_	-0.29049	0.327562
hsa_circ_	0.253643	0.327577
hsa_circ_	-0.31528	0.327578
hsa_circ_	0.235707	0.327603
hsa_circ_	0.283465	0.327616
hsa_circ_	0.221365	0.327636
hsa_circ_	0.172207	0.327641
hsa_circ_	-0.18628	0.32765
hsa_circ_	-0.50893	0.327665
hsa_circ_	-0.20616	0.327674
hsa_circ_	-0.2017	0.327696
hsa_circ_	0.132553	0.327696
hsa_circ_	-0.36336	0.327734
hsa_circ_	-0.14799	0.327734
hsa_circ_	-0.23508	0.327743
hsa_circ_	-0.31078	0.327752
hsa_circ_	0.18378	0.327757
hsa_circ_	0.397225	0.32776
hsa_circ_	0.168243	0.327768
hsa_circ_	-0.25314	0.327768
hsa_circ_	-0.22906	0.327776
hsa_circ_	-0.1461	0.327813
hsa_circ_	-0.18054	0.32782
hsa_circ_	0.238953	0.327821
hsa_circ_	-0.39702	0.32783
hsa_circ_	-0.21751	0.32783

hsa_circ_	0.143662	0.327859
hsa_circ_	0.155095	0.32787
hsa_circ_	0.246041	0.327874
hsa_circ_	-0.18031	0.32788
hsa_circ_	0.236062	0.327881
hsa_circ_	-0.22244	0.327924
hsa_circ_	0.510786	0.327941
hsa_circ_	0.400169	0.327942
hsa_circ_	-0.14837	0.327946
hsa_circ_	0.393002	0.327959
hsa_circ_	-0.1304	0.327961
hsa_circ_	0.15076	0.327995
hsa_circ_	0.146155	0.328011
hsa_circ_	-0.3624	0.328021
hsa_circ_	0.328114	0.328062
hsa_circ_	0.216886	0.328076
hsa_circ_	-0.52944	0.328083
hsa_circ_	0.297015	0.328084
hsa_circ_	0.170476	0.328097
hsa_circ_	-0.15565	0.328102
hsa_circ_	0.285011	0.328108
hsa_circ_	-0.31422	0.328125
hsa_circ_	-0.16469	0.328148
hsa_circ_	0.633878	0.328179
hsa_circ_	-0.13125	0.328183
hsa_circ_	-0.33387	0.328185
hsa_circ_	0.152987	0.328234
hsa_circ_	0.279051	0.328251
hsa_circ_	-0.24577	0.328288
hsa_circ_	0.33484	0.328296
hsa_circ_	0.400654	0.328309
hsa_circ_	0.752384	0.328312
hsa_circ_	0.195196	0.328316
hsa_circ_	-0.20502	0.328316
hsa_circ_	-0.13068	0.328319
hsa_circ_	0.240489	0.328323
hsa_circ_	-0.36702	0.328323
hsa_circ_	0.358369	0.32834
hsa_circ_	0.231077	0.328357
hsa_circ_	-0.15109	0.32838
hsa_circ_	0.174078	0.32841
hsa_circ_	-0.23508	0.328416
hsa_circ_	-0.23156	0.328431
hsa_circ_	-0.40103	0.328435
hsa_circ_	-0.24088	0.328436
hsa_circ_	0.163381	0.328446
hsa_circ_	0.218751	0.328457
hsa_circ_	0.195934	0.328485
hsa_circ_	0.216277	0.328491
hsa_circ_	-0.25677	0.328506
hsa_circ_	0.392737	0.328516
hsa_circ_	0.596131	0.328516
hsa_circ_	-0.17874	0.328542
hsa_circ_	0.420279	0.328547

hsa_circ_	-0.36009	0.328556
hsa_circ_	0.325263	0.328558
hsa_circ_	-0.20684	0.328561
hsa_circ_	-0.52363	0.328561
hsa_circ_	-0.35539	0.328578
hsa_circ_	0.144921	0.328593
hsa_circ_	-0.19909	0.32861
hsa_circ_	0.341254	0.328625
hsa_circ_	-0.14362	0.328646
hsa_circ_	-0.29727	0.328649
hsa_circ_	-0.48239	0.328672
hsa_circ_	0.369158	0.328673
hsa_circ_	-0.56341	0.328681
hsa_circ_	0.144569	0.328717
hsa_circ_	0.223437	0.32873
hsa_circ_	0.185617	0.328734
hsa_circ_	-0.14717	0.328739
hsa_circ_	0.359997	0.32875
hsa_circ_	-0.5038	0.328752
hsa_circ_	-0.17319	0.328769
hsa_circ_	0.354802	0.328786
hsa_circ_	-0.13967	0.328813
hsa_circ_	-0.1586	0.328838
hsa_circ_	-0.3304	0.328852
hsa_circ_	0.145265	0.328864
hsa_circ_	0.303413	0.328867
hsa_circ_	0.182427	0.328869
hsa_circ_	-0.21732	0.328878
hsa_circ_	-0.33357	0.328888
hsa_circ_	-0.23863	0.328897
hsa_circ_	-0.13119	0.328906
hsa_circ_	-0.17113	0.328909
hsa_circ_	0.144812	0.328915
hsa_circ_	0.198397	0.328946
hsa_circ_	-0.21894	0.328953
hsa_circ_	0.342727	0.328961
hsa_circ_	0.625711	0.329005
hsa_circ_	-0.32303	0.329009
hsa_circ_	0.15966	0.329015
hsa_circ_	-0.47484	0.329017
hsa_circ_	-0.16485	0.329018
hsa_circ_	-0.31429	0.329059
hsa_circ_	-0.24757	0.329073
hsa_circ_	0.138338	0.329092
hsa_circ_	-0.51936	0.329109
hsa_circ_	0.289374	0.329127
hsa_circ_	0.256955	0.329148
hsa_circ_	-0.44504	0.329157
hsa_circ_	0.152533	0.329179
hsa_circ_	-0.26205	0.329213
hsa_circ_	0.193587	0.32922
hsa_circ_	-0.17476	0.329227
hsa_circ_	-0.15716	0.329246
hsa_circ_	-0.16022	0.329249

hsa_circ_	-0.21854	0.32928
hsa_circ_	0.717963	0.329281
hsa_circ_	-0.20483	0.329286
hsa_circ_	-0.23365	0.329289
hsa_circ_	0.152228	0.329293
hsa_circ_	-0.35229	0.329298
hsa_circ_	-0.1662	0.329314
hsa_circ_	-0.15232	0.329339
hsa_circ_	0.16231	0.329344
hsa_circ_	-0.3132	0.329355
hsa_circ_	-0.35247	0.329385
hsa_circ_	-0.40948	0.32939
hsa_circ_	-0.14228	0.329402
hsa_circ_	0.140979	0.329406
hsa_circ_	-0.29946	0.329419
hsa_circ_	0.183519	0.329438
hsa_circ_	-0.59841	0.329457
hsa_circ_	0.208451	0.329459
hsa_circ_	-0.18849	0.32946
hsa_circ_	-0.49127	0.329467
hsa_circ_	0.241068	0.329475
hsa_circ_	0.17859	0.329476
hsa_circ_	-0.21367	0.329484
hsa_circ_	0.231619	0.329499
hsa_circ_	-0.19167	0.329509
hsa_circ_	-0.42726	0.329518
hsa_circ_	-0.13764	0.329532
hsa_circ_	-0.35773	0.329556
hsa_circ_	-0.14471	0.329586
hsa_circ_	0.214342	0.329589
hsa_circ_	-0.18009	0.329609
hsa_circ_	0.249253	0.32962
hsa_circ_	-0.17089	0.329641
hsa_circ_	-0.18746	0.329648
hsa_circ_	0.187	0.329666
hsa_circ_	0.154758	0.329681
hsa_circ_	0.158908	0.329682
hsa_circ_	0.261717	0.329698
hsa_circ_	0.140658	0.329705
hsa_circ_	-0.25924	0.329712
hsa_circ_	-0.19302	0.329712
hsa_circ_	-0.14575	0.32972
hsa_circ_	0.248356	0.329739
hsa_circ_	-0.13176	0.329745
hsa_circ_	-0.15202	0.329758
hsa_circ_	-0.25552	0.329764
hsa_circ_	-0.24423	0.329774
hsa_circ_	0.556096	0.329798
hsa_circ_	0.575239	0.329805
hsa_circ_	0.155215	0.329809
hsa_circ_	0.213141	0.329829
hsa_circ_	-0.22543	0.329831
hsa_circ_	0.152557	0.329838
hsa_circ_	0.326162	0.329844

hsa_circ_	0.285882	0.329851
hsa_circ_	-0.13033	0.329854
hsa_circ_	-0.14427	0.32986
hsa_circ_	-0.16419	0.329872
hsa_circ_	-0.15889	0.329872
hsa_circ_	0.15437	0.329873
hsa_circ_	-0.15611	0.329876
hsa_circ_	0.281796	0.329901
hsa_circ_	0.13144	0.329914
hsa_circ_	-0.35363	0.329921
hsa_circ_	-0.2099	0.329927
hsa_circ_	-0.13702	0.32993
hsa_circ_	0.249934	0.329934
hsa_circ_	0.218797	0.329948
hsa_circ_	-0.14885	0.329953
hsa_circ_	-0.15592	0.329963
hsa_circ_	-0.54986	0.329972
hsa_circ_	0.242961	0.330014
hsa_circ_	0.956269	0.330022
hsa_circ_	0.200707	0.330022
hsa_circ_	0.132102	0.330031
hsa_circ_	-0.38895	0.330046
hsa_circ_	0.229273	0.330055
hsa_circ_	-0.15712	0.330057
hsa_circ_	-0.13937	0.330059
hsa_circ_	0.15304	0.330066
hsa_circ_	-0.13748	0.330113
hsa_circ_	-0.26028	0.330114
hsa_circ_	-0.43291	0.330137
hsa_circ_	-0.36686	0.330161
hsa_circ_	0.163765	0.330162
hsa_circ_	-0.67415	0.330167
hsa_circ_	0.196245	0.330183
hsa_circ_	-0.14516	0.33022
hsa_circ_	-0.22657	0.33023
hsa_circ_	0.286395	0.330248
hsa_circ_	-0.43905	0.330249
hsa_circ_	0.291599	0.330252
hsa_circ_	-0.13263	0.330262
hsa_circ_	-0.27743	0.330277
hsa_circ_	0.234405	0.330287
hsa_circ_	-0.36991	0.330297
hsa_circ_	-0.14606	0.330298
hsa_circ_	-0.71476	0.330328
hsa_circ_	-0.17048	0.330337
hsa_circ_	-0.1625	0.330338
hsa_circ_	-0.35684	0.330346
hsa_circ_	-0.13249	0.330372
hsa_circ_	0.169979	0.330386
hsa_circ_	0.292418	0.330397
hsa_circ_	0.18803	0.3304
hsa_circ_	0.21873	0.330415
hsa_circ_	-0.16209	0.330427
hsa_circ_	0.215147	0.330428

hsa_circ_	-0.48217	0.330431
hsa_circ_	0.336452	0.330441
hsa_circ_	-0.38257	0.330442
hsa_circ_	-0.36572	0.330442
hsa_circ_	-0.29872	0.330482
hsa_circ_	0.195512	0.330484
hsa_circ_	0.196445	0.330489
hsa_circ_	-0.25685	0.330497
hsa_circ_	0.219544	0.330502
hsa_circ_	0.215632	0.330502
hsa_circ_	0.163271	0.330507
hsa_circ_	-0.18025	0.330538
hsa_circ_	0.644626	0.330559
hsa_circ_	-0.26961	0.330564
hsa_circ_	0.266036	0.330573
hsa_circ_	0.146887	0.330577
hsa_circ_	0.19692	0.330577
hsa_circ_	-0.27066	0.330598
hsa_circ_	0.379822	0.3306
hsa_circ_	0.161194	0.330611
hsa_circ_	-0.5934	0.330617
hsa_circ_	0.297333	0.330639
hsa_circ_	-0.16016	0.33064
hsa_circ_	0.168721	0.330658
hsa_circ_	-0.1622	0.330674
hsa_circ_	0.515987	0.330683
hsa_circ_	-0.26795	0.330684
hsa_circ_	-0.1454	0.330718
hsa_circ_	-0.16721	0.330728
hsa_circ_	-0.12975	0.330734
hsa_circ_	0.247897	0.330742
hsa_circ_	0.772162	0.330745
hsa_circ_	-0.1539	0.330746
hsa_circ_	-0.33931	0.330762
hsa_circ_	-0.5038	0.330777
hsa_circ_	0.34261	0.330779
hsa_circ_	-0.27783	0.330785
hsa_circ_	-0.15596	0.330788
hsa_circ_	0.38201	0.330822
hsa_circ_	0.138362	0.330872
hsa_circ_	-0.14837	0.330882
hsa_circ_	0.20708	0.330912
hsa_circ_	-0.24181	0.330919
hsa_circ_	0.53602	0.330928
hsa_circ_	-0.2826	0.33093
hsa_circ_	0.334308	0.330936
hsa_circ_	-0.16473	0.330942
hsa_circ_	0.485212	0.330948
hsa_circ_	-0.5466	0.33095
hsa_circ_	0.255616	0.330969
hsa_circ_	0.179018	0.330987
hsa_circ_	-0.24023	0.330993
hsa_circ_	0.430573	0.331001
hsa_circ_	-0.28121	0.33101

hsa_circ_	-0.17248	0.331057
hsa_circ_	-0.22	0.331067
hsa_circ_	-0.50025	0.331069
hsa_circ_	0.44341	0.33107
hsa_circ_	-0.29184	0.331071
hsa_circ_	-0.21998	0.331074
hsa_circ_	0.235206	0.331082
hsa_circ_	0.196063	0.331092
hsa_circ_	0.16092	0.331102
hsa_circ_	0.269512	0.331111
hsa_circ_	0.478859	0.331124
hsa_circ_	0.720291	0.331132
hsa_circ_	-0.63027	0.33114
hsa_circ_	0.211998	0.331145
hsa_circ_	-0.15714	0.331153
hsa_circ_	-0.29256	0.331156
hsa_circ_	-0.18316	0.331225
hsa_circ_	0.159816	0.331254
hsa_circ_	-0.47324	0.331273
hsa_circ_	0.197792	0.331278
hsa_circ_	-0.1387	0.331297
hsa_circ_	-0.19636	0.331303
hsa_circ_	0.487252	0.331317
hsa_circ_	-0.17929	0.331317
hsa_circ_	0.754796	0.331322
hsa_circ_	-0.14715	0.331324
hsa_circ_	-0.14678	0.331356
hsa_circ_	-0.19735	0.331385
hsa_circ_	0.272962	0.331386
hsa_circ_	-0.20195	0.331403
hsa_circ_	-0.20739	0.331426
hsa_circ_	0.165502	0.331481
hsa_circ_	0.197823	0.331495
hsa_circ_	-0.29531	0.331513
hsa_circ_	0.540131	0.33153
hsa_circ_	-0.12725	0.331537
hsa_circ_	-0.1798	0.331563
hsa_circ_	-0.27841	0.331565
hsa_circ_	0.529273	0.331566
hsa_circ_	0.249642	0.331601
hsa_circ_	0.373512	0.331604
hsa_circ_	-0.26216	0.331606
hsa_circ_	-0.29207	0.331617
hsa_circ_	0.14371	0.331619
hsa_circ_	-0.17404	0.331651
hsa_circ_	-0.2198	0.331659
hsa_circ_	-0.16533	0.331663
hsa_circ_	0.157423	0.331668
hsa_circ_	0.14538	0.331675
hsa_circ_	0.156673	0.331682
hsa_circ_	0.29624	0.33169
hsa_circ_	0.179424	0.331715
hsa_circ_	-0.13351	0.331772
hsa_circ_	-0.34408	0.331775

hsa_circ_	-0.47621	0.331779
hsa_circ_	0.221298	0.331808
hsa_circ_	0.185149	0.331842
hsa_circ_	-0.21771	0.331865
hsa_circ_	-0.14492	0.331866
hsa_circ_	-0.16443	0.331877
hsa_circ_	-0.18478	0.331881
hsa_circ_	0.218612	0.331887
hsa_circ_	0.292539	0.331896
hsa_circ_	-0.17051	0.331896
hsa_circ_	-0.17718	0.331917
hsa_circ_	-0.20984	0.331942
hsa_circ_	0.259952	0.331961
hsa_circ_	-0.23657	0.331985
hsa_circ_	-0.19972	0.331987
hsa_circ_	0.238945	0.332009
hsa_circ_	0.180611	0.332036
hsa_circ_	-0.43613	0.33205
hsa_circ_	-1.00369	0.332055
hsa_circ_	-0.18483	0.332056
hsa_circ_	0.371425	0.332056
hsa_circ_	0.520458	0.332061
hsa_circ_	-0.23953	0.332065
hsa_circ_	-0.17401	0.332085
hsa_circ_	-0.17767	0.332095
hsa_circ_	-0.16204	0.332102
hsa_circ_	-0.29312	0.332136
hsa_circ_	0.150772	0.332153
hsa_circ_	0.185446	0.332168
hsa_circ_	-0.16309	0.332204
hsa_circ_	0.227683	0.332206
hsa_circ_	0.131812	0.332217
hsa_circ_	0.467008	0.332228
hsa_circ_	-0.20659	0.332253
hsa_circ_	0.449955	0.332276
hsa_circ_	0.267011	0.332308
hsa_circ_	-3.28832	0.332308
hsa_circ_	-0.49069	0.332313
hsa_circ_	-0.13944	0.332328
hsa_circ_	-0.55446	0.332333
hsa_circ_	0.641017	0.332345
hsa_circ_	0.173157	0.332348
hsa_circ_	-0.36717	0.332353
hsa_circ_	0.266371	0.332363
hsa_circ_	-0.79076	0.332376
hsa_circ_	-0.16625	0.332377
hsa_circ_	0.132909	0.332419
hsa_circ_	-0.34853	0.33243
hsa_circ_	-0.47614	0.332439
hsa_circ_	0.31855	0.332449
hsa_circ_	-0.15155	0.332461
hsa_circ_	-0.15787	0.332463
hsa_circ_	-0.24603	0.332464
hsa_circ_	0.324289	0.332465

hsa_circ_	-0.43425	0.332469
hsa_circ_	0.25838	0.332473
hsa_circ_	0.217497	0.332476
hsa_circ_	-0.23374	0.332484
hsa_circ_	-0.31513	0.332512
hsa_circ_	-0.16795	0.33252
hsa_circ_	-0.23563	0.332526
hsa_circ_	-0.2052	0.332529
hsa_circ_	-0.29674	0.33253
hsa_circ_	0.19427	0.332531
hsa_circ_	-0.26528	0.332548
hsa_circ_	-0.15309	0.332599
hsa_circ_	-0.15665	0.332617
hsa_circ_	-0.16196	0.332624
hsa_circ_	0.849487	0.332629
hsa_circ_	-0.16244	0.33263
hsa_circ_	-0.24104	0.33264
hsa_circ_	0.165718	0.332646
hsa_circ_	0.300299	0.332654
hsa_circ_	0.236151	0.332673
hsa_circ_	-0.18429	0.332685
hsa_circ_	0.295445	0.332695
hsa_circ_	-0.24053	0.332745
hsa_circ_	0.16967	0.332746
hsa_circ_	0.468344	0.332755
hsa_circ_	-0.13698	0.332761
hsa_circ_	-0.18607	0.332764
hsa_circ_	0.171271	0.33277
hsa_circ_	0.275674	0.332796
hsa_circ_	-0.12751	0.332803
hsa_circ_	0.168098	0.332828
hsa_circ_	0.195922	0.332841
hsa_circ_	0.130283	0.332844
hsa_circ_	-0.16467	0.332852
hsa_circ_	-0.40815	0.332855
hsa_circ_	0.254417	0.332857
hsa_circ_	-0.19669	0.332864
hsa_circ_	-0.24123	0.332876
hsa_circ_	-0.23221	0.332902
hsa_circ_	0.605819	0.332902
hsa_circ_	0.180178	0.332905
hsa_circ_	-0.76446	0.332909
hsa_circ_	0.159636	0.332911
hsa_circ_	-0.51061	0.332938
hsa_circ_	-0.16279	0.332945
hsa_circ_	0.212789	0.332947
hsa_circ_	0.356978	0.33295
hsa_circ_	0.199239	0.332954
hsa_circ_	-0.12813	0.332955
hsa_circ_	-0.19384	0.332965
hsa_circ_	-0.36349	0.333007
hsa_circ_	0.253081	0.333011
hsa_circ_	-0.16541	0.333031
hsa_circ_	-0.16119	0.33305

hsa_circ_	0.197449	0.333092
hsa_circ_	0.184909	0.333098
hsa_circ_	0.260981	0.333099
hsa_circ_	-0.22323	0.333105
hsa_circ_	-0.16274	0.333119
hsa_circ_	-0.1696	0.333121
hsa_circ_	0.433874	0.333152
hsa_circ_	0.711824	0.333158
hsa_circ_	0.185682	0.333171
hsa_circ_	-0.47589	0.333192
hsa_circ_	-0.3064	0.333207
hsa_circ_	-0.35394	0.333227
hsa_circ_	-0.24007	0.33323
hsa_circ_	0.189252	0.333232
hsa_circ_	0.172582	0.333234
hsa_circ_	0.6491	0.333253
hsa_circ_	-0.21301	0.333266
hsa_circ_	-0.16174	0.333269
hsa_circ_	-0.25538	0.333295
hsa_circ_	0.218636	0.333297
hsa_circ_	-0.23351	0.333297
hsa_circ_	-0.31226	0.333302
hsa_circ_	0.133007	0.333304
hsa_circ_	-0.18591	0.333318
hsa_circ_	-0.26183	0.333325
hsa_circ_	-0.26375	0.33335
hsa_circ_	0.18103	0.333369
hsa_circ_	-0.25234	0.333385
hsa_circ_	0.239646	0.333386
hsa_circ_	0.198743	0.333397
hsa_circ_	-0.35187	0.333416
hsa_circ_	-0.62462	0.333428
hsa_circ_	-0.28868	0.333431
hsa_circ_	-0.39765	0.333435
hsa_circ_	-0.17481	0.333458
hsa_circ_	0.268109	0.333473
hsa_circ_	-0.17898	0.333477
hsa_circ_	0.295086	0.33348
hsa_circ_	-0.23779	0.333503
hsa_circ_	0.176492	0.333503
hsa_circ_	0.229919	0.333506
hsa_circ_	0.205337	0.333509
hsa_circ_	0.153506	0.333523
hsa_circ_	0.133702	0.333525
hsa_circ_	-0.33704	0.333531
hsa_circ_	-0.24791	0.333532
hsa_circ_	0.334223	0.333532
hsa_circ_	0.309085	0.333534
hsa_circ_	0.188047	0.333584
hsa_circ_	-2.02594	0.333594
hsa_circ_	0.505652	0.333604
hsa_circ_	-0.22945	0.33363
hsa_circ_	0.238889	0.333644
hsa_circ_	0.260032	0.333659

hsa_circ_	-0.26099	0.333669
hsa_circ_	0.145156	0.333674
hsa_circ_	0.219504	0.333674
hsa_circ_	-0.16901	0.333679
hsa_circ_	0.203529	0.33369
hsa_circ_	0.246096	0.333698
hsa_circ_	0.446662	0.333704
hsa_circ_	0.33861	0.33371
hsa_circ_	-0.14066	0.333739
hsa_circ_	0.205828	0.333742
hsa_circ_	0.155614	0.333744
hsa_circ_	-0.29131	0.333749
hsa_circ_	-0.22492	0.333749
hsa_circ_	0.359394	0.333759
hsa_circ_	0.555357	0.333773
hsa_circ_	0.367115	0.333804
hsa_circ_	0.223169	0.333811
hsa_circ_	0.180172	0.333819
hsa_circ_	-0.14784	0.333829
hsa_circ_	-0.21683	0.33384
hsa_circ_	-0.2783	0.333848
hsa_circ_	0.188814	0.333857
hsa_circ_	-0.15459	0.333875
hsa_circ_	-0.25681	0.333911
hsa_circ_	-0.13712	0.33395
hsa_circ_	-0.27188	0.333963
hsa_circ_	-0.2146	0.333963
hsa_circ_	-0.56286	0.333964
hsa_circ_	-0.17184	0.333976
hsa_circ_	-0.17146	0.333977
hsa_circ_	-0.37087	0.333988
hsa_circ_	0.491326	0.334014
hsa_circ_	-0.19054	0.334022
hsa_circ_	0.406622	0.334024
hsa_circ_	-0.23372	0.334028
hsa_circ_	0.181056	0.33404
hsa_circ_	0.295888	0.334051
hsa_circ_	-0.16597	0.334053
hsa_circ_	0.166797	0.334059
hsa_circ_	0.196433	0.334065
hsa_circ_	-0.38767	0.334072
hsa_circ_	-0.15637	0.334073
hsa_circ_	-0.23078	0.334073
hsa_circ_	0.210633	0.334074
hsa_circ_	0.538287	0.33408
hsa_circ_	-0.21495	0.334111
hsa_circ_	0.409515	0.334112
hsa_circ_	-0.21591	0.334119
hsa_circ_	-1.52901	0.334121
hsa_circ_	-0.53078	0.334143
hsa_circ_	0.157124	0.33415
hsa_circ_	-0.29271	0.334189
hsa_circ_	0.450555	0.334189
hsa_circ_	-0.16867	0.334212

hsa_circ_	0.205198	0.334216
hsa_circ_	-0.46278	0.33422
hsa_circ_	0.470159	0.334222
hsa_circ_	-0.14215	0.33423
hsa_circ_	-0.17532	0.33424
hsa_circ_	0.136103	0.33426
hsa_circ_	0.186823	0.33426
hsa_circ_	0.206966	0.334266
hsa_circ_	-0.20042	0.334278
hsa_circ_	0.167236	0.33428
hsa_circ_	-0.18141	0.334292
hsa_circ_	0.244683	0.334296
hsa_circ_	-0.23858	0.334305
hsa_circ_	-0.16822	0.334307
hsa_circ_	0.585948	0.33436
hsa_circ_	-0.16988	0.334378
hsa_circ_	0.333265	0.334382
hsa_circ_	0.161579	0.334386
hsa_circ_	-0.30113	0.334393
hsa_circ_	0.27318	0.334398
hsa_circ_	-0.23054	0.334418
hsa_circ_	-0.28909	0.334421
hsa_circ_	-0.17825	0.334429
hsa_circ_	0.182221	0.334438
hsa_circ_	-0.31408	0.33444
hsa_circ_	0.220839	0.334452
hsa_circ_	0.284997	0.334471
hsa_circ_	-0.20777	0.334481
hsa_circ_	-0.57524	0.334493
hsa_circ_	-0.13112	0.334506
hsa_circ_	0.138438	0.334532
hsa_circ_	-0.14647	0.334556
hsa_circ_	-0.15667	0.334558
hsa_circ_	0.164457	0.334559
hsa_circ_	0.223437	0.334568
hsa_circ_	0.209329	0.334574
hsa_circ_	0.953085	0.334616
hsa_circ_	0.223297	0.334618
hsa_circ_	-0.15433	0.334619
hsa_circ_	0.18376	0.334622
hsa_circ_	0.488839	0.334637
hsa_circ_	0.171229	0.334642
hsa_circ_	0.191307	0.33465
hsa_circ_	-0.19553	0.334651
hsa_circ_	-0.16647	0.334663
hsa_circ_	-0.25218	0.334689
hsa_circ_	-0.60868	0.334694
hsa_circ_	0.140842	0.334698
hsa_circ_	0.156254	0.334738
hsa_circ_	-0.23737	0.33474
hsa_circ_	0.639601	0.334745
hsa_circ_	0.150842	0.334749
hsa_circ_	0.4117	0.334753
hsa_circ_	-0.2364	0.33476

hsa_circ_	-0.16221	0.334805
hsa_circ_	0.171168	0.334828
hsa_circ_	-0.17793	0.334861
hsa_circ_	-0.17009	0.334869
hsa_circ_	-0.51791	0.334876
hsa_circ_	-0.18499	0.334879
hsa_circ_	-0.14448	0.334881
hsa_circ_	0.196543	0.334883
hsa_circ_	0.148499	0.334928
hsa_circ_	0.283367	0.334942
hsa_circ_	-0.16436	0.334951
hsa_circ_	-0.36074	0.334954
hsa_circ_	-0.44863	0.334966
hsa_circ_	0.194331	0.334974
hsa_circ_	-0.19841	0.334986
hsa_circ_	-0.15962	0.334993
hsa_circ_	-0.29396	0.33506
hsa_circ_	0.242964	0.335071
hsa_circ_	0.516854	0.335089
hsa_circ_	-0.30568	0.335094
hsa_circ_	0.311929	0.335101
hsa_circ_	0.197252	0.335104
hsa_circ_	0.318125	0.335109
hsa_circ_	0.142586	0.335112
hsa_circ_	0.321363	0.33518
hsa_circ_	-0.16678	0.335182
hsa_circ_	-0.45829	0.335189
hsa_circ_	-0.22071	0.33519
hsa_circ_	-0.46742	0.335194
hsa_circ_	0.184162	0.335201
hsa_circ_	-0.27459	0.335205
hsa_circ_	0.191978	0.335238
hsa_circ_	0.536318	0.335241
hsa_circ_	-0.16063	0.335255
hsa_circ_	0.14783	0.335257
hsa_circ_	-0.1407	0.335263
hsa_circ_	-0.13412	0.335277
hsa_circ_	0.304549	0.335281
hsa_circ_	-0.50423	0.335282
hsa_circ_	-0.14699	0.335293
hsa_circ_	-0.59175	0.335296
hsa_circ_	1.114299	0.3353
hsa_circ_	0.417505	0.335318
hsa_circ_	-0.16115	0.335322
hsa_circ_	-0.25322	0.335324
hsa_circ_	0.128513	0.335325
hsa_circ_	-0.33133	0.335345
hsa_circ_	0.617218	0.335369
hsa_circ_	-0.13271	0.335371
hsa_circ_	0.207501	0.335382
hsa_circ_	0.430188	0.33539
hsa_circ_	0.302454	0.335394
hsa_circ_	0.229442	0.335402
hsa_circ_	-0.24268	0.335412

hsa_circ_	0.227897	0.335419
hsa_circ_	0.201557	0.335419
hsa_circ_	-0.1808	0.335428
hsa_circ_	-0.14152	0.33544
hsa_circ_	-0.51739	0.335474
hsa_circ_	-0.67165	0.335477
hsa_circ_	-0.51457	0.33549
hsa_circ_	0.191762	0.335504
hsa_circ_	0.17222	0.335516
hsa_circ_	0.192217	0.335518
hsa_circ_	-0.18426	0.335519
hsa_circ_	0.651107	0.335534
hsa_circ_	-0.14936	0.335541
hsa_circ_	-0.14136	0.335546
hsa_circ_	0.273651	0.335558
hsa_circ_	-0.20221	0.335619
hsa_circ_	0.158861	0.335627
hsa_circ_	0.155603	0.335648
hsa_circ_	-0.36638	0.335652
hsa_circ_	-0.20995	0.335674
hsa_circ_	0.192027	0.335682
hsa_circ_	-0.19147	0.335704
hsa_circ_	-0.15838	0.33573
hsa_circ_	-0.39833	0.335737
hsa_circ_	-0.37784	0.335756
hsa_circ_	0.176372	0.335778
hsa_circ_	0.156039	0.335793
hsa_circ_	-0.26646	0.335798
hsa_circ_	0.162875	0.335818
hsa_circ_	0.241691	0.335819
hsa_circ_	-0.18504	0.335833
hsa_circ_	-0.19676	0.335835
hsa_circ_	-0.20797	0.335836
hsa_circ_	-0.18021	0.335847
hsa_circ_	-0.18444	0.335875
hsa_circ_	-0.25036	0.335891
hsa_circ_	-0.19275	0.3359
hsa_circ_	-0.42736	0.335931
hsa_circ_	0.224694	0.335933
hsa_circ_	0.325032	0.335936
hsa_circ_	-0.34188	0.335951
hsa_circ_	0.314941	0.335954
hsa_circ_	-0.14547	0.335961
hsa_circ_	0.178132	0.335984
hsa_circ_	-0.3341	0.336033
hsa_circ_	-0.16156	0.336062
hsa_circ_	-0.13967	0.336071
hsa_circ_	0.208018	0.33608
hsa_circ_	0.484587	0.336091
hsa_circ_	0.132336	0.336091
hsa_circ_	0.240573	0.336116
hsa_circ_	0.184561	0.336119
hsa_circ_	-0.34098	0.336122
hsa_circ_	0.185724	0.336126

hsa_circ_	-0.139	0.336148
hsa_circ_	-0.23002	0.336148
hsa_circ_	-0.1919	0.336151
hsa_circ_	-0.5043	0.336152
hsa_circ_	-0.15508	0.336155
hsa_circ_	-0.20489	0.336228
hsa_circ_	-0.1474	0.336242
hsa_circ_	0.184775	0.336262
hsa_circ_	0.150596	0.336294
hsa_circ_	0.20598	0.336311
hsa_circ_	-0.28759	0.336349
hsa_circ_	0.148748	0.336356
hsa_circ_	-0.23589	0.336366
hsa_circ_	0.453633	0.336371
hsa_circ_	0.313636	0.336376
hsa_circ_	-0.46205	0.33638
hsa_circ_	-0.18818	0.336407
hsa_circ_	0.402705	0.336408
hsa_circ_	-0.17978	0.336425
hsa_circ_	-0.32358	0.336452
hsa_circ_	0.564103	0.336457
hsa_circ_	-0.14319	0.336461
hsa_circ_	0.176451	0.336467
hsa_circ_	0.181566	0.336474
hsa_circ_	-0.15827	0.336505
hsa_circ_	-0.23123	0.336506
hsa_circ_	0.169965	0.336509
hsa_circ_	-0.34811	0.336512
hsa_circ_	0.174206	0.336527
hsa_circ_	-0.52197	0.336531
hsa_circ_	0.406506	0.336533
hsa_circ_	-0.13196	0.336561
hsa_circ_	-0.20956	0.336564
hsa_circ_	-0.26978	0.336565
hsa_circ_	0.345534	0.336566
hsa_circ_	-0.35215	0.336568
hsa_circ_	-0.21768	0.33657
hsa_circ_	-0.29302	0.336572
hsa_circ_	-0.18261	0.336581
hsa_circ_	-0.5207	0.336586
hsa_circ_	-2.60351	0.336598
hsa_circ_	-0.16073	0.336612
hsa_circ_	0.243823	0.336612
hsa_circ_	-0.16474	0.336614
hsa_circ_	0.338609	0.336623
hsa_circ_	0.166799	0.336629
hsa_circ_	-0.20691	0.33663
hsa_circ_	-0.24735	0.33663
hsa_circ_	0.154654	0.336631
hsa_circ_	-0.42527	0.336641
hsa_circ_	-0.26331	0.336723
hsa_circ_	0.497135	0.336736
hsa_circ_	-0.32755	0.336744
hsa_circ_	-0.19404	0.336749

hsa_circ_	0.184945	0.336759
hsa_circ_	0.191325	0.33676
hsa_circ_	-0.28063	0.336767
hsa_circ_	0.541043	0.336771
hsa_circ_	0.304901	0.336779
hsa_circ_	-0.19472	0.336793
hsa_circ_	-0.52164	0.336806
hsa_circ_	0.276901	0.336807
hsa_circ_	-0.22089	0.33683
hsa_circ_	0.210744	0.336835
hsa_circ_	-0.19891	0.336837
hsa_circ_	0.980354	0.336843
hsa_circ_	0.212389	0.336862
hsa_circ_	0.319592	0.336871
hsa_circ_	0.231509	0.336876
hsa_circ_	0.318854	0.336888
hsa_circ_	0.365687	0.336898
hsa_circ_	0.267708	0.336901
hsa_circ_	0.510585	0.336907
hsa_circ_	-0.32756	0.336911
hsa_circ_	0.222083	0.336938
hsa_circ_	-0.18895	0.336944
hsa_circ_	-0.21954	0.336954
hsa_circ_	0.134761	0.336968
hsa_circ_	0.394503	0.336968
hsa_circ_	0.151001	0.336982
hsa_circ_	0.341471	0.336994
hsa_circ_	-0.54144	0.336995
hsa_circ_	-0.19962	0.337
hsa_circ_	0.167117	0.337014
hsa_circ_	-0.42657	0.337016
hsa_circ_	0.499363	0.337017
hsa_circ_	0.222111	0.337052
hsa_circ_	0.324228	0.337055
hsa_circ_	-0.2244	0.337065
hsa_circ_	0.167939	0.337068
hsa_circ_	-0.13118	0.337076
hsa_circ_	0.204892	0.337076
hsa_circ_	0.22094	0.337085
hsa_circ_	-1.09115	0.337086
hsa_circ_	0.215227	0.337104
hsa_circ_	-0.16716	0.337107
hsa_circ_	-0.56885	0.337108
hsa_circ_	-0.18203	0.33711
hsa_circ_	-0.20985	0.337114
hsa_circ_	0.198353	0.337136
hsa_circ_	-0.46384	0.337139
hsa_circ_	0.575957	0.337148
hsa_circ_	-0.71051	0.337164
hsa_circ_	0.255588	0.337172
hsa_circ_	-0.18018	0.337172
hsa_circ_	-0.20252	0.337217
hsa_circ_	-0.24843	0.337217
hsa_circ_	-0.20168	0.33724

hsa_circ_	-0.30137	0.337256
hsa_circ_	0.214761	0.337256
hsa_circ_	0.159998	0.337262
hsa_circ_	0.369903	0.337262
hsa_circ_	-0.20297	0.337264
hsa_circ_	-0.15697	0.337278
hsa_circ_	-0.53384	0.337287
hsa_circ_	-0.18215	0.337288
hsa_circ_	-0.21819	0.337291
hsa_circ_	0.204597	0.337297
hsa_circ_	-0.16615	0.337319
hsa_circ_	-0.51328	0.337323
hsa_circ_	-0.25758	0.337333
hsa_circ_	-0.72039	0.337363
hsa_circ_	0.21184	0.337368
hsa_circ_	-0.1852	0.337374
hsa_circ_	0.200543	0.337381
hsa_circ_	-0.14447	0.33739
hsa_circ_	-0.35519	0.337404
hsa_circ_	0.207606	0.337416
hsa_circ_	0.254856	0.337417
hsa_circ_	0.204355	0.337425
hsa_circ_	-0.31829	0.337437
hsa_circ_	0.689575	0.337456
hsa_circ_	-0.13945	0.337457
hsa_circ_	-0.14266	0.337482
hsa_circ_	0.160338	0.337494
hsa_circ_	0.166856	0.3375
hsa_circ_	-0.30175	0.337523
hsa_circ_	0.166786	0.337558
hsa_circ_	-0.144	0.337562
hsa_circ_	-0.16449	0.337566
hsa_circ_	0.270612	0.337569
hsa_circ_	-0.17074	0.337579
hsa_circ_	-0.19906	0.337589
hsa_circ_	-0.16353	0.33759
hsa_circ_	-0.14769	0.337593
hsa_circ_	-0.57127	0.337603
hsa_circ_	-0.49829	0.337604
hsa_circ_	0.302865	0.337605
hsa_circ_	-0.2517	0.337606
hsa_circ_	0.560743	0.33761
hsa_circ_	0.233883	0.337612
hsa_circ_	0.323803	0.33762
hsa_circ_	0.561975	0.337625
hsa_circ_	-0.12807	0.33764
hsa_circ_	-0.53432	0.337668
hsa_circ_	-0.15246	0.337693
hsa_circ_	-0.291	0.337698
hsa_circ_	-0.46945	0.337706
hsa_circ_	-0.37801	0.337729
hsa_circ_	-0.15914	0.337733
hsa_circ_	-0.42397	0.337754
hsa_circ_	-0.51035	0.33777

hsa_circ_	-0.93012	0.33777
hsa_circ_	-0.48544	0.337773
hsa_circ_	-0.20447	0.337803
hsa_circ_	-0.16474	0.337804
hsa_circ_	0.137126	0.337815
hsa_circ_	0.228071	0.337818
hsa_circ_	-0.17139	0.337832
hsa_circ_	-0.37284	0.337845
hsa_circ_	0.606478	0.337881
hsa_circ_	0.269851	0.337886
hsa_circ_	-0.79028	0.337893
hsa_circ_	0.149274	0.337949
hsa_circ_	-0.13235	0.337956
hsa_circ_	-0.96295	0.337961
hsa_circ_	-0.67489	0.337966
hsa_circ_	0.217496	0.337978
hsa_circ_	0.460632	0.33802
hsa_circ_	0.380303	0.338035
hsa_circ_	-0.39823	0.338041
hsa_circ_	0.283261	0.338076
hsa_circ_	0.24859	0.338081
hsa_circ_	-0.28366	0.3381
hsa_circ_	-0.25545	0.338118
hsa_circ_	-0.30472	0.338137
hsa_circ_	-0.19743	0.338147
hsa_circ_	-0.17447	0.338173
hsa_circ_	0.274931	0.338189
hsa_circ_	-0.30971	0.338197
hsa_circ_	0.137349	0.338208
hsa_circ_	0.414599	0.338243
hsa_circ_	0.614886	0.338244
hsa_circ_	-0.4303	0.338256
hsa_circ_	0.511213	0.338258
hsa_circ_	0.195964	0.338316
hsa_circ_	-0.3597	0.338327
hsa_circ_	0.27563	0.338332
hsa_circ_	0.152741	0.338359
hsa_circ_	-0.241	0.338373
hsa_circ_	-0.63648	0.338376
hsa_circ_	0.293777	0.33841
hsa_circ_	0.203735	0.338421
hsa_circ_	0.153611	0.338425
hsa_circ_	-0.23759	0.338456
hsa_circ_	0.152886	0.33847
hsa_circ_	-0.16325	0.338483
hsa_circ_	0.149961	0.338487
hsa_circ_	-0.21551	0.338537
hsa_circ_	0.19477	0.338548
hsa_circ_	0.146449	0.338552
hsa_circ_	-0.16179	0.338565
hsa_circ_	-0.30702	0.338566
hsa_circ_	0.198237	0.338591
hsa_circ_	0.19837	0.338597
hsa_circ_	-0.16689	0.338615

hsa_circ_	-0.21999	0.338626
hsa_circ_	-0.19329	0.338638
hsa_circ_	0.18278	0.338642
hsa_circ_	-0.35574	0.338645
hsa_circ_	0.175539	0.338715
hsa_circ_	-0.37924	0.338716
hsa_circ_	-0.12938	0.338728
hsa_circ_	0.184091	0.338735
hsa_circ_	-0.17934	0.338737
hsa_circ_	-0.45497	0.338741
hsa_circ_	-0.13632	0.338742
hsa_circ_	-0.16847	0.338753
hsa_circ_	0.221174	0.338782
hsa_circ_	0.13777	0.33879
hsa_circ_	0.210177	0.338849
hsa_circ_	0.157108	0.33885
hsa_circ_	0.147101	0.338857
hsa_circ_	-0.29811	0.33886
hsa_circ_	0.215932	0.33886
hsa_circ_	-0.17834	0.338875
hsa_circ_	0.170027	0.338951
hsa_circ_	0.230172	0.338997
hsa_circ_	0.365803	0.339002
hsa_circ_	-0.31293	0.339023
hsa_circ_	-0.18724	0.339023
hsa_circ_	0.223851	0.339069
hsa_circ_	0.125144	0.339078
hsa_circ_	-0.18925	0.339101
hsa_circ_	0.1538	0.339116
hsa_circ_	-0.13514	0.339117
hsa_circ_	0.165714	0.339118
hsa_circ_	-0.18964	0.339131
hsa_circ_	-0.30348	0.339169
hsa_circ_	-0.35099	0.339171
hsa_circ_	0.197264	0.339173
hsa_circ_	0.128246	0.339174
hsa_circ_	-0.29502	0.339187
hsa_circ_	0.213019	0.339199
hsa_circ_	0.67798	0.339212
hsa_circ_	0.21095	0.339217
hsa_circ_	-0.22732	0.339232
hsa_circ_	-0.1871	0.339234
hsa_circ_	0.178285	0.339236
hsa_circ_	-0.4252	0.339262
hsa_circ_	-0.28317	0.339294
hsa_circ_	0.241296	0.339294
hsa_circ_	-0.13867	0.339295
hsa_circ_	0.307804	0.339296
hsa_circ_	0.260772	0.339298
hsa_circ_	0.38095	0.339319
hsa_circ_	-0.38445	0.339326
hsa_circ_	-0.15031	0.339327
hsa_circ_	0.284629	0.339342
hsa_circ_	0.137267	0.339342

hsa_circ_	-0.15366	0.339349
hsa_circ_	0.204044	0.33936
hsa_circ_	-0.15911	0.339375
hsa_circ_	-0.28178	0.33938
hsa_circ_	0.27967	0.339381
hsa_circ_	-0.19832	0.339383
hsa_circ_	-0.42945	0.339386
hsa_circ_	0.576244	0.339392
hsa_circ_	0.128823	0.339397
hsa_circ_	0.447359	0.339401
hsa_circ_	-0.15473	0.339419
hsa_circ_	0.781791	0.339446
hsa_circ_	0.167314	0.339446
hsa_circ_	-0.21351	0.339449
hsa_circ_	-0.69299	0.339451
hsa_circ_	0.195837	0.339461
hsa_circ_	-0.21235	0.339474
hsa_circ_	-0.17263	0.339477
hsa_circ_	0.132452	0.339478
hsa_circ_	0.754987	0.339505
hsa_circ_	-0.21498	0.339507
hsa_circ_	0.186543	0.339514
hsa_circ_	0.13467	0.339526
hsa_circ_	-0.19369	0.339546
hsa_circ_	-0.20908	0.339556
hsa_circ_	-0.28558	0.339565
hsa_circ_	0.197187	0.339571
hsa_circ_	0.19961	0.339579
hsa_circ_	0.165471	0.339581
hsa_circ_	-0.25243	0.339599
hsa_circ_	-0.25883	0.339603
hsa_circ_	-0.29515	0.339619
hsa_circ_	-0.14356	0.339636
hsa_circ_	-0.59857	0.339639
hsa_circ_	0.448586	0.339651
hsa_circ_	-0.3588	0.339667
hsa_circ_	-0.35041	0.33967
hsa_circ_	-0.26374	0.339672
hsa_circ_	-0.1641	0.339685
hsa_circ_	0.336444	0.33969
hsa_circ_	0.339727	0.339733
hsa_circ_	0.272806	0.339744
hsa_circ_	-0.40198	0.339752
hsa_circ_	0.170163	0.339755
hsa_circ_	0.296166	0.339759
hsa_circ_	0.873608	0.33977
hsa_circ_	-0.13034	0.339786
hsa_circ_	0.193761	0.33979
hsa_circ_	0.308253	0.339799
hsa_circ_	-0.16426	0.3398
hsa_circ_	-0.14856	0.339813
hsa_circ_	0.234492	0.339826
hsa_circ_	0.158409	0.339826
hsa_circ_	-0.1547	0.339831

hsa_circ_	0.30705	0.339839
hsa_circ_	-0.9117	0.339857
hsa_circ_	-0.3896	0.339875
hsa_circ_	0.212458	0.339875
hsa_circ_	-0.4193	0.339889
hsa_circ_	-1.01131	0.339922
hsa_circ_	0.26986	0.339924
hsa_circ_	-0.33141	0.339953
hsa_circ_	-0.39524	0.339958
hsa_circ_	-0.30091	0.339971
hsa_circ_	0.147773	0.34
hsa_circ_	-0.24807	0.340001
hsa_circ_	-0.38122	0.340016
hsa_circ_	-0.38681	0.34004
hsa_circ_	0.336513	0.340044
hsa_circ_	0.285506	0.340049
hsa_circ_	-0.2558	0.340064
hsa_circ_	0.274022	0.340073
hsa_circ_	-0.18841	0.340086
hsa_circ_	-0.18717	0.340088
hsa_circ_	0.199419	0.340099
hsa_circ_	-0.30049	0.340113
hsa_circ_	0.262897	0.340115
hsa_circ_	-0.39911	0.340118
hsa_circ_	0.183539	0.340124
hsa_circ_	-0.63221	0.340136
hsa_circ_	-0.37576	0.340141
hsa_circ_	-0.18184	0.340146
hsa_circ_	-0.15641	0.340161
hsa_circ_	-0.22245	0.340177
hsa_circ_	-0.22669	0.340182
hsa_circ_	-0.47388	0.340182
hsa_circ_	-0.25359	0.340186
hsa_circ_	-0.14727	0.340206
hsa_circ_	0.182131	0.340221
hsa_circ_	-0.16301	0.340229
hsa_circ_	0.147003	0.340231
hsa_circ_	-0.19695	0.340243
hsa_circ_	-0.16544	0.340247
hsa_circ_	-0.666	0.340267
hsa_circ_	-0.39138	0.340291
hsa_circ_	0.381953	0.3403
hsa_circ_	-0.18248	0.340302
hsa_circ_	-1.24643	0.340304
hsa_circ_	0.242321	0.340304
hsa_circ_	0.405306	0.340308
hsa_circ_	0.228338	0.340313
hsa_circ_	0.152758	0.340325
hsa_circ_	-0.28709	0.340327
hsa_circ_	-0.13232	0.340356
hsa_circ_	-0.15855	0.340364
hsa_circ_	-0.20791	0.340409
hsa_circ_	0.171447	0.34041
hsa_circ_	-0.35659	0.340425

hsa_circ_	0.252976	0.340444
hsa_circ_	0.301747	0.340475
hsa_circ_	0.182413	0.340477
hsa_circ_	-0.49497	0.340477
hsa_circ_	-0.25859	0.340477
hsa_circ_	-0.64647	0.340497
hsa_circ_	-0.57497	0.340499
hsa_circ_	-0.177	0.340517
hsa_circ_	0.778688	0.340561
hsa_circ_	0.197591	0.340577
hsa_circ_	0.22233	0.340586
hsa_circ_	-0.33273	0.340693
hsa_circ_	-0.27663	0.340694
hsa_circ_	0.23376	0.340696
hsa_circ_	0.323835	0.340703
hsa_circ_	0.175913	0.340713
hsa_circ_	-0.40062	0.340724
hsa_circ_	0.203455	0.340729
hsa_circ_	0.264001	0.340734
hsa_circ_	0.1515	0.340749
hsa_circ_	-0.15244	0.340777
hsa_circ_	0.309927	0.340778
hsa_circ_	-0.19923	0.340786
hsa_circ_	-0.18441	0.340809
hsa_circ_	-0.32938	0.340824
hsa_circ_	0.323374	0.340825
hsa_circ_	0.243776	0.340828
hsa_circ_	0.210995	0.340846
hsa_circ_	0.160145	0.340861
hsa_circ_	0.268261	0.34087
hsa_circ_	0.30549	0.340877
hsa_circ_	0.239563	0.340878
hsa_circ_	0.562338	0.340884
hsa_circ_	-0.49239	0.340887
hsa_circ_	0.199557	0.340891
hsa_circ_	0.530342	0.340899
hsa_circ_	0.165521	0.340907
hsa_circ_	0.137757	0.340917
hsa_circ_	0.327925	0.340918
hsa_circ_	-0.23808	0.340975
hsa_circ_	0.244013	0.340975
hsa_circ_	0.273355	0.340986
hsa_circ_	-0.17515	0.340989
hsa_circ_	0.141436	0.34102
hsa_circ_	0.287699	0.341028
hsa_circ_	0.663635	0.341048
hsa_circ_	-0.21803	0.341056
hsa_circ_	-0.19001	0.341077
hsa_circ_	0.349218	0.341087
hsa_circ_	0.195592	0.341124
hsa_circ_	-0.31513	0.341166
hsa_circ_	0.243557	0.341186
hsa_circ_	-0.20301	0.341196
hsa_circ_	0.661212	0.341196

hsa_circ_	-1.72526	0.341208
hsa_circ_	-0.16534	0.341211
hsa_circ_	-0.45661	0.341231
hsa_circ_	-0.17174	0.341257
hsa_circ_	-0.20358	0.341263
hsa_circ_	0.153229	0.341275
hsa_circ_	0.140058	0.341287
hsa_circ_	-0.28542	0.341298
hsa_circ_	0.18617	0.341303
hsa_circ_	0.140243	0.341303
hsa_circ_	0.590423	0.341303
hsa_circ_	-0.31396	0.341311
hsa_circ_	0.201831	0.341322
hsa_circ_	0.166804	0.341339
hsa_circ_	-0.14711	0.341351
hsa_circ_	-0.13763	0.341352
hsa_circ_	0.244157	0.341354
hsa_circ_	0.144444	0.341382
hsa_circ_	0.549989	0.341382
hsa_circ_	-0.22901	0.341393
hsa_circ_	-0.15349	0.341395
hsa_circ_	-0.13183	0.3414
hsa_circ_	-0.18339	0.341409
hsa_circ_	0.238127	0.341419
hsa_circ_	-0.1369	0.341433
hsa_circ_	0.208624	0.341438
hsa_circ_	-0.31366	0.341464
hsa_circ_	0.153267	0.341469
hsa_circ_	0.138912	0.341471
hsa_circ_	-0.30022	0.341505
hsa_circ_	0.150465	0.341514
hsa_circ_	-0.19601	0.341535
hsa_circ_	-0.82322	0.341538
hsa_circ_	-0.18563	0.341549
hsa_circ_	0.249398	0.341559
hsa_circ_	-0.29702	0.341568
hsa_circ_	-0.13667	0.341638
hsa_circ_	0.219867	0.341643
hsa_circ_	0.625719	0.341659
hsa_circ_	0.152713	0.341686
hsa_circ_	-0.26924	0.341717
hsa_circ_	-0.15659	0.341719
hsa_circ_	0.454859	0.341727
hsa_circ_	0.131792	0.341737
hsa_circ_	-0.19716	0.341749
hsa_circ_	-0.29788	0.341769
hsa_circ_	0.307193	0.341773
hsa_circ_	0.259617	0.341796
hsa_circ_	0.131879	0.341803
hsa_circ_	-0.19106	0.341823
hsa_circ_	-0.16514	0.341845
hsa_circ_	-0.83612	0.341862
hsa_circ_	0.468661	0.341868
hsa_circ_	0.210423	0.341869

hsa_circ_	-0.1599	0.341871
hsa_circ_	0.130597	0.341872
hsa_circ_	0.151513	0.341881
hsa_circ_	0.161331	0.341893
hsa_circ_	0.152857	0.341901
hsa_circ_	0.656799	0.341907
hsa_circ_	-0.32873	0.341946
hsa_circ_	0.289073	0.341988
hsa_circ_	-0.17758	0.341996
hsa_circ_	0.315274	0.342002
hsa_circ_	-0.14892	0.342016
hsa_circ_	0.191604	0.342027
hsa_circ_	0.400498	0.342036
hsa_circ_	-0.14652	0.342065
hsa_circ_	0.159253	0.342074
hsa_circ_	-0.16424	0.34208
hsa_circ_	0.19206	0.342097
hsa_circ_	-0.14454	0.342102
hsa_circ_	-0.18671	0.342103
hsa_circ_	0.127674	0.342105
hsa_circ_	0.300735	0.342108
hsa_circ_	0.262956	0.342116
hsa_circ_	0.471111	0.342117
hsa_circ_	0.148916	0.34215
hsa_circ_	0.227089	0.342158
hsa_circ_	-0.2247	0.342167
hsa_circ_	0.155238	0.342173
hsa_circ_	0.311079	0.342189
hsa_circ_	0.207031	0.34219
hsa_circ_	-0.32729	0.342193
hsa_circ_	0.158283	0.342201
hsa_circ_	-0.15608	0.342202
hsa_circ_	-0.27898	0.342208
hsa_circ_	0.423003	0.342219
hsa_circ_	-0.19343	0.342219
hsa_circ_	0.319383	0.34222
hsa_circ_	-0.22337	0.342225
hsa_circ_	-0.16415	0.342242
hsa_circ_	0.278318	0.342272
hsa_circ_	0.227447	0.342282
hsa_circ_	0.639984	0.342289
hsa_circ_	-0.26221	0.342298
hsa_circ_	0.129185	0.34231
hsa_circ_	0.478859	0.342351
hsa_circ_	-0.16455	0.34237
hsa_circ_	0.208126	0.342397
hsa_circ_	0.204661	0.34241
hsa_circ_	0.164992	0.34241
hsa_circ_	-0.96053	0.342432
hsa_circ_	0.413912	0.342464
hsa_circ_	-0.56605	0.342472
hsa_circ_	0.137154	0.342488
hsa_circ_	-0.24931	0.342496
hsa_circ_	0.243056	0.342507

hsa_circ_	0.174662	0.342509
hsa_circ_	-0.22762	0.342511
hsa_circ_	0.415488	0.342516
hsa_circ_	0.146816	0.342528
hsa_circ_	0.236865	0.34254
hsa_circ_	0.190422	0.342543
hsa_circ_	0.222222	0.34258
hsa_circ_	0.134245	0.342589
hsa_circ_	0.336044	0.342644
hsa_circ_	0.137608	0.342657
hsa_circ_	0.236823	0.342664
hsa_circ_	-0.13484	0.342671
hsa_circ_	0.433105	0.342675
hsa_circ_	-0.40202	0.34268
hsa_circ_	0.266567	0.342683
hsa_circ_	-0.44086	0.342703
hsa_circ_	-0.1499	0.342708
hsa_circ_	0.205154	0.342709
hsa_circ_	-0.20833	0.34272
hsa_circ_	0.192666	0.342728
hsa_circ_	0.511858	0.342736
hsa_circ_	0.210149	0.342739
hsa_circ_	0.134778	0.342746
hsa_circ_	-0.14417	0.342753
hsa_circ_	-0.21444	0.342774
hsa_circ_	0.190311	0.342775
hsa_circ_	-0.28838	0.34278
hsa_circ_	-0.17421	0.342806
hsa_circ_	0.221663	0.342832
hsa_circ_	0.13957	0.342838
hsa_circ_	-0.42649	0.34284
hsa_circ_	-0.1358	0.342843
hsa_circ_	0.290833	0.342844
hsa_circ_	-0.16117	0.342849
hsa_circ_	0.202169	0.342866
hsa_circ_	0.233984	0.342872
hsa_circ_	-0.15508	0.342895
hsa_circ_	0.350025	0.342898
hsa_circ_	0.213098	0.342899
hsa_circ_	0.78954	0.342901
hsa_circ_	0.288919	0.342931
hsa_circ_	0.261988	0.342939
hsa_circ_	0.19354	0.342945
hsa_circ_	-0.2863	0.342949
hsa_circ_	-0.14297	0.342962
hsa_circ_	0.172333	0.342964
hsa_circ_	-0.15628	0.342964
hsa_circ_	-0.14042	0.342997
hsa_circ_	0.436475	0.343009
hsa_circ_	-0.19121	0.343045
hsa_circ_	-0.31319	0.343056
hsa_circ_	0.279699	0.343056
hsa_circ_	0.208393	0.343058
hsa_circ_	0.319214	0.343106

hsa_circ_	0.756504	0.343118
hsa_circ_	-0.33175	0.343141
hsa_circ_	-0.21545	0.343159
hsa_circ_	-0.47419	0.343161
hsa_circ_	-0.15138	0.343162
hsa_circ_	-0.13075	0.343163
hsa_circ_	0.16981	0.343163
hsa_circ_	-0.27919	0.343166
hsa_circ_	0.146126	0.34317
hsa_circ_	0.268564	0.343175
hsa_circ_	0.181973	0.343186
hsa_circ_	-0.15544	0.343204
hsa_circ_	0.159955	0.343213
hsa_circ_	0.476321	0.343216
hsa_circ_	-0.2232	0.343221
hsa_circ_	-0.1976	0.343223
hsa_circ_	0.167629	0.343233
hsa_circ_	0.577831	0.343243
hsa_circ_	0.640982	0.34325
hsa_circ_	0.25412	0.343256
hsa_circ_	-0.15592	0.343258
hsa_circ_	0.321364	0.343264
hsa_circ_	-0.27935	0.343272
hsa_circ_	-0.15477	0.343317
hsa_circ_	0.177258	0.34334
hsa_circ_	-0.16663	0.343343
hsa_circ_	-0.1959	0.343346
hsa_circ_	0.211439	0.343356
hsa_circ_	0.139767	0.343366
hsa_circ_	0.34864	0.343368
hsa_circ_	-0.20714	0.343372
hsa_circ_	0.428636	0.343384
hsa_circ_	0.246404	0.343404
hsa_circ_	0.143792	0.343414
hsa_circ_	-0.33167	0.343431
hsa_circ_	-0.29746	0.343441
hsa_circ_	0.209143	0.343451
hsa_circ_	0.310211	0.343468
hsa_circ_	0.180001	0.343476
hsa_circ_	0.160437	0.343497
hsa_circ_	0.147001	0.343531
hsa_circ_	0.166065	0.34357
hsa_circ_	-0.79979	0.343585
hsa_circ_	-0.16671	0.343626
hsa_circ_	-0.23585	0.343627
hsa_circ_	-0.23584	0.343636
hsa_circ_	0.337671	0.343642
hsa_circ_	-0.25522	0.343646
hsa_circ_	0.140522	0.343648
hsa_circ_	-0.19435	0.34365
hsa_circ_	-0.32002	0.343658
hsa_circ_	0.38256	0.34368
hsa_circ_	-0.22668	0.343682
hsa_circ_	-0.17231	0.343708

hsa_circ_	0.217105	0.343721
hsa_circ_	0.715448	0.343749
hsa_circ_	-0.15538	0.343754
hsa_circ_	-0.42749	0.34376
hsa_circ_	-0.12959	0.343803
hsa_circ_	-0.47179	0.343805
hsa_circ_	0.33136	0.343812
hsa_circ_	-0.48319	0.343814
hsa_circ_	0.225987	0.343879
hsa_circ_	-0.2228	0.343901
hsa_circ_	0.277634	0.343902
hsa_circ_	-0.13009	0.343911
hsa_circ_	-0.17486	0.343919
hsa_circ_	-0.15875	0.343922
hsa_circ_	0.439281	0.343927
hsa_circ_	-0.2265	0.343969
hsa_circ_	0.332001	0.343976
hsa_circ_	-0.16364	0.343998
hsa_circ_	0.215858	0.344009
hsa_circ_	0.457625	0.344018
hsa_circ_	0.135087	0.344039
hsa_circ_	-0.13281	0.344056
hsa_circ_	-0.22192	0.344063
hsa_circ_	-0.49391	0.344067
hsa_circ_	-0.33989	0.344076
hsa_circ_	0.270949	0.344092
hsa_circ_	-0.33937	0.344092
hsa_circ_	-0.27188	0.344096
hsa_circ_	-0.1589	0.344102
hsa_circ_	-0.12255	0.344104
hsa_circ_	0.313503	0.344127
hsa_circ_	0.152129	0.344139
hsa_circ_	-0.26833	0.34414
hsa_circ_	0.172559	0.344142
hsa_circ_	-0.61784	0.344145
hsa_circ_	-0.13772	0.344155
hsa_circ_	0.692779	0.34416
hsa_circ_	0.202119	0.344162
hsa_circ_	0.182129	0.344218
hsa_circ_	-0.13975	0.344241
hsa_circ_	0.451373	0.344245
hsa_circ_	0.274797	0.344255
hsa_circ_	-0.38576	0.344255
hsa_circ_	0.397675	0.34427
hsa_circ_	-0.14485	0.344279
hsa_circ_	-0.38781	0.34429
hsa_circ_	0.215704	0.344297
hsa_circ_	0.321162	0.344322
hsa_circ_	-0.14848	0.344331
hsa_circ_	0.202104	0.344333
hsa_circ_	-0.66184	0.344334
hsa_circ_	-0.1935	0.344338
hsa_circ_	-0.34562	0.344344
hsa_circ_	-0.95394	0.344346

hsa_circ_	0.139483	0.344361
hsa_circ_	-0.14196	0.344362
hsa_circ_	0.466694	0.344362
hsa_circ_	-0.27163	0.344368
hsa_circ_	0.382497	0.344399
hsa_circ_	-0.34576	0.344407
hsa_circ_	0.320709	0.344434
hsa_circ_	0.301654	0.344444
hsa_circ_	-0.23666	0.34445
hsa_circ_	0.169637	0.34446
hsa_circ_	-0.58307	0.344509
hsa_circ_	-0.20205	0.344521
hsa_circ_	-0.28369	0.344528
hsa_circ_	0.175714	0.344529
hsa_circ_	0.248603	0.344552
hsa_circ_	0.150852	0.344557
hsa_circ_	0.139272	0.344584
hsa_circ_	-0.51745	0.344601
hsa_circ_	0.198492	0.344626
hsa_circ_	0.250499	0.344634
hsa_circ_	-0.16133	0.344641
hsa_circ_	0.363251	0.344681
hsa_circ_	-0.20329	0.344697
hsa_circ_	0.222079	0.344715
hsa_circ_	0.243853	0.344737
hsa_circ_	-0.17547	0.34474
hsa_circ_	0.500106	0.344741
hsa_circ_	0.266111	0.344769
hsa_circ_	0.193593	0.344781
hsa_circ_	-0.13847	0.344808
hsa_circ_	0.264144	0.344809
hsa_circ_	-0.21492	0.344822
hsa_circ_	0.162183	0.344838
hsa_circ_	0.150203	0.34485
hsa_circ_	-0.1809	0.344854
hsa_circ_	0.591429	0.344859
hsa_circ_	-0.40103	0.34487
hsa_circ_	0.253682	0.344916
hsa_circ_	0.42432	0.344931
hsa_circ_	-0.23699	0.344931
hsa_circ_	0.235955	0.34495
hsa_circ_	-0.26537	0.344955
hsa_circ_	-0.16867	0.344957
hsa_circ_	-0.16458	0.344966
hsa_circ_	-0.14303	0.344987
hsa_circ_	-0.45032	0.344998
hsa_circ_	0.237793	0.345007
hsa_circ_	0.204123	0.345019
hsa_circ_	-0.14608	0.345029
hsa_circ_	0.388564	0.345041
hsa_circ_	0.151176	0.345041
hsa_circ_	0.186715	0.345058
hsa_circ_	-0.36253	0.345075
hsa_circ_	-0.28195	0.345092

hsa_circ_	-0.36053	0.34511
hsa_circ_	-0.12341	0.345121
hsa_circ_	0.124411	0.345142
hsa_circ_	-0.22111	0.345147
hsa_circ_	0.202072	0.345149
hsa_circ_	-0.41109	0.345153
hsa_circ_	-0.20611	0.345159
hsa_circ_	-0.58743	0.345162
hsa_circ_	-0.39893	0.345176
hsa_circ_	0.242956	0.345199
hsa_circ_	-0.14336	0.345201
hsa_circ_	-0.28877	0.345208
hsa_circ_	-0.24228	0.345208
hsa_circ_	0.24324	0.34521
hsa_circ_	0.266611	0.345222
hsa_circ_	-0.15952	0.345224
hsa_circ_	-0.16017	0.345263
hsa_circ_	-0.18235	0.345267
hsa_circ_	-0.22167	0.345269
hsa_circ_	0.297978	0.345269
hsa_circ_	0.485002	0.345285
hsa_circ_	-0.31389	0.345289
hsa_circ_	0.140695	0.345297
hsa_circ_	0.161238	0.345331
hsa_circ_	-0.20373	0.345332
hsa_circ_	0.159761	0.345344
hsa_circ_	-0.27867	0.345364
hsa_circ_	-0.31345	0.345365
hsa_circ_	0.196253	0.345373
hsa_circ_	-0.15164	0.345385
hsa_circ_	0.202242	0.34539
hsa_circ_	-0.3974	0.34543
hsa_circ_	0.184	0.345438
hsa_circ_	-0.1693	0.345465
hsa_circ_	-0.1232	0.345472
hsa_circ_	-0.15893	0.345487
hsa_circ_	-0.17018	0.345488
hsa_circ_	-0.23533	0.345499
hsa_circ_	0.3071	0.345517
hsa_circ_	0.224234	0.345556
hsa_circ_	-0.25029	0.345561
hsa_circ_	0.190554	0.345571
hsa_circ_	-0.16346	0.345573
hsa_circ_	0.27005	0.345586
hsa_circ_	-0.58065	0.345629
hsa_circ_	0.227618	0.345642
hsa_circ_	-0.5402	0.34565
hsa_circ_	-0.23086	0.345685
hsa_circ_	0.157549	0.345688
hsa_circ_	0.179816	0.345693
hsa_circ_	0.27132	0.345722
hsa_circ_	-0.139	0.34573
hsa_circ_	-0.15875	0.345733
hsa_circ_	0.264819	0.345778

hsa_circ_	0.780295	0.345797
hsa_circ_	0.25627	0.345806
hsa_circ_	0.166671	0.345811
hsa_circ_	-0.19616	0.345815
hsa_circ_	0.13621	0.345818
hsa_circ_	-0.4102	0.345825
hsa_circ_	0.176295	0.345828
hsa_circ_	0.124762	0.345832
hsa_circ_	0.153817	0.345832
hsa_circ_	0.207141	0.345862
hsa_circ_	0.185729	0.345885
hsa_circ_	0.183442	0.345913
hsa_circ_	-0.15291	0.345929
hsa_circ_	0.208226	0.34594
hsa_circ_	-0.13871	0.345959
hsa_circ_	-0.16432	0.345969
hsa_circ_	-0.22164	0.345982
hsa_circ_	-0.21772	0.346014
hsa_circ_	-0.17192	0.346017
hsa_circ_	-0.2498	0.346018
hsa_circ_	-0.24475	0.346028
hsa_circ_	0.322983	0.346058
hsa_circ_	-0.20561	0.346059
hsa_circ_	-0.28426	0.34606
hsa_circ_	0.228283	0.346079
hsa_circ_	-0.18951	0.346085
hsa_circ_	0.248254	0.346098
hsa_circ_	-0.20273	0.3461
hsa_circ_	-0.22497	0.346105
hsa_circ_	-0.24484	0.346111
hsa_circ_	0.166662	0.346115
hsa_circ_	-0.20332	0.346131
hsa_circ_	0.256869	0.34615
hsa_circ_	0.145235	0.346177
hsa_circ_	-0.15241	0.34619
hsa_circ_	-0.27403	0.346199
hsa_circ_	-0.18136	0.34621
hsa_circ_	0.362166	0.346215
hsa_circ_	0.170137	0.346221
hsa_circ_	0.208208	0.346233
hsa_circ_	0.154143	0.346257
hsa_circ_	-0.14729	0.346272
hsa_circ_	-0.14877	0.346272
hsa_circ_	-0.18568	0.346273
hsa_circ_	-0.76057	0.346276
hsa_circ_	-0.16353	0.346283
hsa_circ_	0.494618	0.346294
hsa_circ_	-0.19288	0.346307
hsa_circ_	-0.33659	0.34632
hsa_circ_	-0.58813	0.346327
hsa_circ_	-0.5161	0.34636
hsa_circ_	-0.28933	0.346371
hsa_circ_	-0.14143	0.34639
hsa_circ_	-0.13978	0.346412

hsa_circ_	-0.31086	0.346415
hsa_circ_	0.332351	0.346422
hsa_circ_	0.338652	0.346429
hsa_circ_	-0.14485	0.346433
hsa_circ_	0.22982	0.346437
hsa_circ_	0.803208	0.346448
hsa_circ_	-0.18895	0.346449
hsa_circ_	-0.26789	0.346482
hsa_circ_	-0.16962	0.346492
hsa_circ_	-0.28027	0.346515
hsa_circ_	0.519414	0.346517
hsa_circ_	0.195536	0.346542
hsa_circ_	0.159968	0.346547
hsa_circ_	0.499189	0.346566
hsa_circ_	0.224534	0.346577
hsa_circ_	0.227567	0.346605
hsa_circ_	-0.16449	0.346607
hsa_circ_	-0.16992	0.346614
hsa_circ_	-0.2023	0.346614
hsa_circ_	-0.47747	0.346625
hsa_circ_	0.568997	0.34663
hsa_circ_	-0.15324	0.346652
hsa_circ_	0.161023	0.346658
hsa_circ_	-0.23251	0.346663
hsa_circ_	0.223314	0.346665
hsa_circ_	-0.21247	0.346668
hsa_circ_	0.172093	0.346681
hsa_circ_	-0.45717	0.346698
hsa_circ_	-0.28405	0.346699
hsa_circ_	-0.1268	0.346711
hsa_circ_	0.133224	0.346725
hsa_circ_	0.337596	0.346745
hsa_circ_	-0.22144	0.346762
hsa_circ_	-0.12772	0.346764
hsa_circ_	0.140179	0.34677
hsa_circ_	-0.14364	0.346785
hsa_circ_	-0.26177	0.346791
hsa_circ_	-0.42834	0.346795
hsa_circ_	0.162296	0.346798
hsa_circ_	-0.17428	0.346817
hsa_circ_	0.214754	0.346822
hsa_circ_	0.423818	0.346827
hsa_circ_	0.410344	0.346834
hsa_circ_	0.497639	0.346845
hsa_circ_	0.196009	0.346851
hsa_circ_	0.165671	0.346861
hsa_circ_	-0.43713	0.346867
hsa_circ_	-0.13117	0.346883
hsa_circ_	-0.16246	0.346902
hsa_circ_	-0.19884	0.346907
hsa_circ_	-0.64553	0.346933
hsa_circ_	0.216307	0.346939
hsa_circ_	0.382417	0.346942
hsa_circ_	-0.13634	0.34695

hsa_circ_	-0.48684	0.346964
hsa_circ_	0.160922	0.34698
hsa_circ_	-0.15013	0.346985
hsa_circ_	0.148639	0.346986
hsa_circ_	-0.24735	0.346987
hsa_circ_	-0.12837	0.347015
hsa_circ_	0.168108	0.347019
hsa_circ_	0.194293	0.347021
hsa_circ_	-0.53062	0.34704
hsa_circ_	0.490769	0.347072
hsa_circ_	-0.41714	0.347084
hsa_circ_	0.175043	0.347094
hsa_circ_	-0.56727	0.347099
hsa_circ_	0.188075	0.3471
hsa_circ_	-0.22327	0.347117
hsa_circ_	0.186455	0.347118
hsa_circ_	-0.15845	0.34714
hsa_circ_	-0.12414	0.347193
hsa_circ_	-0.27597	0.347225
hsa_circ_	-0.17019	0.347238
hsa_circ_	-0.29824	0.347243
hsa_circ_	0.143843	0.347259
hsa_circ_	0.142729	0.347266
hsa_circ_	-0.15163	0.347285
hsa_circ_	-0.20514	0.347321
hsa_circ_	-0.25091	0.347322
hsa_circ_	-0.16247	0.347344
hsa_circ_	0.14191	0.347362
hsa_circ_	0.156245	0.347368
hsa_circ_	-0.13378	0.347379
hsa_circ_	0.170169	0.347384
hsa_circ_	0.199727	0.347389
hsa_circ_	0.201778	0.347408
hsa_circ_	0.210424	0.347413
hsa_circ_	-0.15935	0.34742
hsa_circ_	-0.68217	0.347428
hsa_circ_	-0.14541	0.347429
hsa_circ_	0.228291	0.347446
hsa_circ_	0.380025	0.347447
hsa_circ_	0.42532	0.347475
hsa_circ_	-0.18972	0.347482
hsa_circ_	-0.14401	0.347499
hsa_circ_	0.130421	0.347503
hsa_circ_	-0.47179	0.347524
hsa_circ_	0.244729	0.347532
hsa_circ_	-0.24684	0.347539
hsa_circ_	-0.18352	0.347542
hsa_circ_	0.165792	0.347552
hsa_circ_	-0.14641	0.347554
hsa_circ_	-0.13573	0.347556
hsa_circ_	0.196844	0.347561
hsa_circ_	0.405409	0.347562
hsa_circ_	-0.12404	0.347563
hsa_circ_	-0.18221	0.347572

hsa_circ_	0.13975	0.347574
hsa_circ_	-0.19899	0.347586
hsa_circ_	-0.3482	0.347609
hsa_circ_	-0.18139	0.34762
hsa_circ_	0.232083	0.347623
hsa_circ_	-0.17785	0.347625
hsa_circ_	0.16884	0.347628
hsa_circ_	0.179028	0.347629
hsa_circ_	-0.20289	0.347639
hsa_circ_	0.347219	0.347645
hsa_circ_	0.257426	0.347662
hsa_circ_	-0.56946	0.347666
hsa_circ_	-0.13758	0.347669
hsa_circ_	0.337863	0.347669
hsa_circ_	-0.22423	0.347706
hsa_circ_	0.225133	0.347716
hsa_circ_	-0.26505	0.347724
hsa_circ_	0.147042	0.347758
hsa_circ_	0.18475	0.347803
hsa_circ_	-0.12187	0.347843
hsa_circ_	0.170679	0.347852
hsa_circ_	0.311304	0.347867
hsa_circ_	-0.21478	0.347877
hsa_circ_	-0.14886	0.347883
hsa_circ_	-0.38266	0.347888
hsa_circ_	0.369546	0.347892
hsa_circ_	0.15184	0.347921
hsa_circ_	0.181766	0.347934
hsa_circ_	0.184671	0.347943
hsa_circ_	-0.23143	0.347944
hsa_circ_	0.693914	0.347954
hsa_circ_	0.22028	0.347967
hsa_circ_	-1.59165	0.347973
hsa_circ_	0.260469	0.347994
hsa_circ_	-0.84305	0.347997
hsa_circ_	0.445418	0.348011
hsa_circ_	-0.13432	0.348022
hsa_circ_	0.129617	0.348108
hsa_circ_	-1.92247	0.348159
hsa_circ_	-0.21361	0.348159
hsa_circ_	-0.1717	0.348173
hsa_circ_	0.274634	0.348184
hsa_circ_	0.220835	0.348193
hsa_circ_	-0.12797	0.348194
hsa_circ_	-0.2058	0.348222
hsa_circ_	-0.14188	0.348253
hsa_circ_	0.151484	0.348257
hsa_circ_	0.148644	0.348267
hsa_circ_	-0.35576	0.348278
hsa_circ_	0.488125	0.348285
hsa_circ_	-0.64088	0.348305
hsa_circ_	0.213525	0.348306
hsa_circ_	-0.18138	0.348319
hsa_circ_	-0.19897	0.348325

hsa_circ_	-0.19424	0.348328
hsa_circ_	0.137673	0.348329
hsa_circ_	-0.14831	0.348336
hsa_circ_	-0.16499	0.34834
hsa_circ_	0.236478	0.348342
hsa_circ_	-0.27283	0.348345
hsa_circ_	-0.28441	0.348345
hsa_circ_	0.291706	0.348346
hsa_circ_	0.378794	0.348351
hsa_circ_	0.270198	0.348353
hsa_circ_	0.304265	0.348408
hsa_circ_	-0.42977	0.348431
hsa_circ_	-0.19933	0.348435
hsa_circ_	-0.23965	0.348435
hsa_circ_	0.173452	0.348439
hsa_circ_	0.222126	0.348465
hsa_circ_	0.454808	0.34847
hsa_circ_	-0.122	0.348475
hsa_circ_	-0.25964	0.348485
hsa_circ_	-0.33053	0.34849
hsa_circ_	0.202499	0.348497
hsa_circ_	-0.69818	0.348499
hsa_circ_	-0.19748	0.348516
hsa_circ_	0.233982	0.348526
hsa_circ_	3.324157	0.348563
hsa_circ_	0.358127	0.34857
hsa_circ_	-0.1683	0.348577
hsa_circ_	-0.32113	0.348578
hsa_circ_	-0.48102	0.348597
hsa_circ_	0.211514	0.348626
hsa_circ_	-0.13805	0.34865
hsa_circ_	-0.20457	0.348664
hsa_circ_	0.580912	0.348672
hsa_circ_	0.251904	0.34868
hsa_circ_	0.194002	0.348684
hsa_circ_	-0.1493	0.348693
hsa_circ_	0.390301	0.348693
hsa_circ_	0.349724	0.348695
hsa_circ_	-0.13593	0.348701
hsa_circ_	-0.14079	0.348707
hsa_circ_	-0.26532	0.348714
hsa_circ_	0.269083	0.348766
hsa_circ_	-0.31246	0.348766
hsa_circ_	-0.40365	0.348769
hsa_circ_	-0.14777	0.348769
hsa_circ_	0.136674	0.348805
hsa_circ_	-0.17243	0.348809
hsa_circ_	-0.23622	0.348821
hsa_circ_	0.173691	0.348837
hsa_circ_	0.151504	0.348872
hsa_circ_	-0.31946	0.348886
hsa_circ_	-0.2388	0.348901
hsa_circ_	0.215848	0.348934
hsa_circ_	0.606569	0.348943

hsa_circ_	-0.19414	0.348944
hsa_circ_	-0.2466	0.348961
hsa_circ_	0.227931	0.34898
hsa_circ_	-0.20872	0.349009
hsa_circ_	0.176086	0.34901
hsa_circ_	-0.82808	0.349014
hsa_circ_	0.175636	0.349038
hsa_circ_	0.569264	0.349045
hsa_circ_	-0.24529	0.349053
hsa_circ_	0.190122	0.349055
hsa_circ_	-0.18395	0.349059
hsa_circ_	0.265686	0.349076
hsa_circ_	0.519176	0.349079
hsa_circ_	0.146604	0.349083
hsa_circ_	0.605449	0.34909
hsa_circ_	-0.35868	0.349109
hsa_circ_	0.134203	0.349115
hsa_circ_	-0.13789	0.349128
hsa_circ_	0.191212	0.34916
hsa_circ_	-0.38493	0.349169
hsa_circ_	-0.16561	0.34917
hsa_circ_	-0.28815	0.349185
hsa_circ_	-0.19464	0.34919
hsa_circ_	-0.18591	0.349209
hsa_circ_	0.22243	0.349213
hsa_circ_	-0.1967	0.349221
hsa_circ_	-0.17305	0.349224
hsa_circ_	0.137747	0.349251
hsa_circ_	0.234212	0.349267
hsa_circ_	-0.29208	0.349288
hsa_circ_	-0.36623	0.349307
hsa_circ_	-0.25129	0.349325
hsa_circ_	0.442804	0.349347
hsa_circ_	0.15246	0.349365
hsa_circ_	-0.1888	0.349369
hsa_circ_	-0.12788	0.349388
hsa_circ_	-0.42708	0.349401
hsa_circ_	0.329339	0.349409
hsa_circ_	-0.12112	0.349409
hsa_circ_	-0.23346	0.349424
hsa_circ_	-0.13996	0.349434
hsa_circ_	0.2484	0.349436
hsa_circ_	0.188915	0.349438
hsa_circ_	0.663008	0.349443
hsa_circ_	0.148602	0.349456
hsa_circ_	0.254885	0.349457
hsa_circ_	0.174135	0.349465
hsa_circ_	-0.5532	0.349473
hsa_circ_	-0.22678	0.349477
hsa_circ_	-0.14938	0.349477
hsa_circ_	-0.36782	0.349501
hsa_circ_	-0.15364	0.349518
hsa_circ_	-0.37848	0.349523
hsa_circ_	0.452189	0.349579

hsa_circ_	-0.48391	0.349594
hsa_circ_	0.210988	0.349628
hsa_circ_	-0.16463	0.349638
hsa_circ_	-0.20949	0.349654
hsa_circ_	-0.52005	0.349664
hsa_circ_	0.239643	0.349668
hsa_circ_	-0.1861	0.349684
hsa_circ_	-0.19377	0.349688
hsa_circ_	0.427757	0.349699
hsa_circ_	-0.162	0.349707
hsa_circ_	-0.17233	0.349711
hsa_circ_	-0.13364	0.349726
hsa_circ_	-0.15695	0.349728
hsa_circ_	-0.16498	0.349738
hsa_circ_	0.167973	0.349739
hsa_circ_	-0.13105	0.349752
hsa_circ_	0.124461	0.349752
hsa_circ_	0.403748	0.349785
hsa_circ_	-0.13867	0.349787
hsa_circ_	-0.50239	0.349789
hsa_circ_	-0.53664	0.349835
hsa_circ_	-0.22042	0.349839
hsa_circ_	0.377487	0.349847
hsa_circ_	0.14902	0.349849
hsa_circ_	-0.1903	0.349865
hsa_circ_	0.143472	0.349889
hsa_circ_	0.129425	0.349894
hsa_circ_	0.642035	0.349895
hsa_circ_	0.161314	0.349899
hsa_circ_	-0.40712	0.3499
hsa_circ_	0.174526	0.349904
hsa_circ_	0.547249	0.349918
hsa_circ_	0.502767	0.34992
hsa_circ_	-0.1247	0.349923
hsa_circ_	-0.16555	0.349928
hsa_circ_	-0.23837	0.349937
hsa_circ_	0.266984	0.349937
hsa_circ_	0.323124	0.349938
hsa_circ_	0.166705	0.349961
hsa_circ_	0.159883	0.349983
hsa_circ_	-0.16284	0.349992
hsa_circ_	0.196221	0.350003
hsa_circ_	0.180139	0.350006
hsa_circ_	0.715519	0.350008
hsa_circ_	0.265382	0.35001
hsa_circ_	-0.36235	0.350049
hsa_circ_	-0.1526	0.350077
hsa_circ_	0.131675	0.350083
hsa_circ_	-0.41582	0.350088
hsa_circ_	0.13896	0.350105
hsa_circ_	-0.17403	0.350108
hsa_circ_	-0.12969	0.350115
hsa_circ_	-0.14654	0.35014
hsa_circ_	-0.20275	0.350143

hsa_circ_	-0.33382	0.35015
hsa_circ_	-0.30868	0.350158
hsa_circ_	0.210208	0.350175
hsa_circ_	-0.22617	0.350175
hsa_circ_	-0.15493	0.35022
hsa_circ_	-0.15863	0.350224
hsa_circ_	0.594699	0.350235
hsa_circ_	0.324949	0.350237
hsa_circ_	0.165577	0.350245
hsa_circ_	0.183568	0.350272
hsa_circ_	0.352838	0.350282
hsa_circ_	0.185738	0.350316
hsa_circ_	-0.39544	0.350328
hsa_circ_	-0.14379	0.350333
hsa_circ_	-0.43281	0.350353
hsa_circ_	0.175008	0.350367
hsa_circ_	0.169511	0.350379
hsa_circ_	-0.28751	0.350394
hsa_circ_	-0.21706	0.350423
hsa_circ_	0.521261	0.350447
hsa_circ_	0.194964	0.350448
hsa_circ_	0.23141	0.35045
hsa_circ_	0.210945	0.350453
hsa_circ_	0.198147	0.350455
hsa_circ_	-0.46638	0.350475
hsa_circ_	0.212337	0.350483
hsa_circ_	0.569138	0.350495
hsa_circ_	-0.14063	0.350508
hsa_circ_	0.16525	0.350531
hsa_circ_	-0.23472	0.350538
hsa_circ_	0.369299	0.350546
hsa_circ_	-0.18596	0.350565
hsa_circ_	-0.56074	0.35057
hsa_circ_	0.28307	0.350571
hsa_circ_	0.180498	0.350589
hsa_circ_	0.275819	0.350598
hsa_circ_	0.3264	0.350603
hsa_circ_	0.210759	0.350614
hsa_circ_	-0.15568	0.350617
hsa_circ_	0.188114	0.350622
hsa_circ_	0.24634	0.350632
hsa_circ_	0.25053	0.350637
hsa_circ_	0.137207	0.350677
hsa_circ_	-0.22157	0.350678
hsa_circ_	-0.24435	0.350698
hsa_circ_	0.168515	0.350701
hsa_circ_	0.34737	0.350701
hsa_circ_	0.249355	0.350707
hsa_circ_	-1.6625	0.350712
hsa_circ_	0.225984	0.350727
hsa_circ_	-0.20783	0.350735
hsa_circ_	0.145486	0.350759
hsa_circ_	0.128124	0.350761
hsa_circ_	0.207029	0.350784

hsa_circ_	0.194411	0.350793
hsa_circ_	0.310971	0.350839
hsa_circ_	0.273819	0.350845
hsa_circ_	-0.16567	0.350847
hsa_circ_	-0.17375	0.35086
hsa_circ_	-0.2211	0.350865
hsa_circ_	-0.16179	0.350875
hsa_circ_	-0.19993	0.350877
hsa_circ_	-0.27722	0.350898
hsa_circ_	-0.17214	0.350898
hsa_circ_	0.152367	0.350914
hsa_circ_	0.15376	0.350949
hsa_circ_	-0.18806	0.350976
hsa_circ_	0.513417	0.350982
hsa_circ_	-0.47472	0.35101
hsa_circ_	-0.2446	0.351046
hsa_circ_	0.228424	0.351055
hsa_circ_	-0.12838	0.35108
hsa_circ_	0.451187	0.351081
hsa_circ_	-0.15386	0.351095
hsa_circ_	0.204887	0.351099
hsa_circ_	-0.25344	0.351146
hsa_circ_	-0.2416	0.351155
hsa_circ_	0.189614	0.351172
hsa_circ_	-0.27384	0.351173
hsa_circ_	-0.20407	0.351181
hsa_circ_	-0.26456	0.351193
hsa_circ_	0.223167	0.351199
hsa_circ_	0.193285	0.351206
hsa_circ_	0.253064	0.351234
hsa_circ_	1.237552	0.351242
hsa_circ_	0.128018	0.351242
hsa_circ_	0.336819	0.351245
hsa_circ_	0.373162	0.351251
hsa_circ_	0.13997	0.35126
hsa_circ_	-0.16697	0.351264
hsa_circ_	-0.14382	0.351264
hsa_circ_	0.15022	0.351272
hsa_circ_	0.169496	0.351276
hsa_circ_	-0.34414	0.351285
hsa_circ_	-0.18468	0.351289
hsa_circ_	-0.33909	0.351298
hsa_circ_	0.148381	0.351302
hsa_circ_	-0.12962	0.351304
hsa_circ_	0.241695	0.351329
hsa_circ_	-0.41342	0.351331
hsa_circ_	0.120187	0.351353
hsa_circ_	0.520539	0.3514
hsa_circ_	-0.16084	0.351431
hsa_circ_	-0.18237	0.351432
hsa_circ_	0.154898	0.351438
hsa_circ_	-0.22076	0.351442
hsa_circ_	0.256656	0.35145
hsa_circ_	-0.15987	0.351466

hsa_circ_	-0.18686	0.351477
hsa_circ_	0.285157	0.351478
hsa_circ_	-0.28023	0.351507
hsa_circ_	0.271843	0.351526
hsa_circ_	-0.65197	0.351527
hsa_circ_	-0.20503	0.351542
hsa_circ_	0.238667	0.35155
hsa_circ_	0.148034	0.351557
hsa_circ_	0.139933	0.351558
hsa_circ_	-0.23708	0.351562
hsa_circ_	0.693804	0.351564
hsa_circ_	0.344291	0.351578
hsa_circ_	0.198264	0.351583
hsa_circ_	-0.13902	0.351585
hsa_circ_	0.47832	0.351592
hsa_circ_	0.181021	0.35161
hsa_circ_	0.13856	0.351612
hsa_circ_	-0.22209	0.351618
hsa_circ_	0.155776	0.35164
hsa_circ_	0.327532	0.351683
hsa_circ_	0.273027	0.351692
hsa_circ_	0.22538	0.351701
hsa_circ_	-1.39383	0.351703
hsa_circ_	-0.26298	0.351704
hsa_circ_	-0.26362	0.351733
hsa_circ_	-0.78522	0.351738
hsa_circ_	-0.19659	0.351741
hsa_circ_	-0.13492	0.351747
hsa_circ_	-0.37835	0.35177
hsa_circ_	0.378582	0.351772
hsa_circ_	-0.35009	0.35178
hsa_circ_	-0.33642	0.351789
hsa_circ_	-0.41652	0.351801
hsa_circ_	-0.29055	0.351824
hsa_circ_	0.510703	0.35183
hsa_circ_	-0.44008	0.351874
hsa_circ_	-0.20148	0.3519
hsa_circ_	0.226731	0.351902
hsa_circ_	0.16002	0.351921
hsa_circ_	-0.27551	0.351922
hsa_circ_	-0.18755	0.351923
hsa_circ_	0.749542	0.351927
hsa_circ_	-0.4255	0.351995
hsa_circ_	0.199307	0.351999
hsa_circ_	-0.27894	0.352005
hsa_circ_	-0.16387	0.352022
hsa_circ_	-0.17297	0.352023
hsa_circ_	0.413576	0.352025
hsa_circ_	-0.15612	0.35205
hsa_circ_	0.204398	0.352067
hsa_circ_	-0.41898	0.352083
hsa_circ_	0.178541	0.352107
hsa_circ_	-0.37087	0.352114
hsa_circ_	0.128743	0.352116

hsa_circ_	0.293595	0.35212
hsa_circ_	0.145828	0.352139
hsa_circ_	-0.18438	0.352157
hsa_circ_	0.13501	0.352157
hsa_circ_	0.30308	0.352188
hsa_circ_	-0.19762	0.352219
hsa_circ_	-0.13738	0.352231
hsa_circ_	-0.38084	0.352231
hsa_circ_	0.196994	0.352242
hsa_circ_	-0.3755	0.352251
hsa_circ_	-0.18865	0.352258
hsa_circ_	-0.15503	0.352261
hsa_circ_	0.176374	0.352282
hsa_circ_	0.272027	0.352284
hsa_circ_	0.158964	0.352293
hsa_circ_	-0.19281	0.352298
hsa_circ_	-0.29413	0.352346
hsa_circ_	-0.15882	0.352357
hsa_circ_	-0.18747	0.35236
hsa_circ_	-1.4688	0.352403
hsa_circ_	-0.26362	0.352413
hsa_circ_	-0.16762	0.352419
hsa_circ_	0.328132	0.35242
hsa_circ_	0.223478	0.352433
hsa_circ_	-0.12199	0.352452
hsa_circ_	-0.24311	0.352469
hsa_circ_	-0.28293	0.35247
hsa_circ_	0.203313	0.352475
hsa_circ_	-0.14526	0.352486
hsa_circ_	0.189456	0.352486
hsa_circ_	-0.26906	0.352504
hsa_circ_	-0.13951	0.352545
hsa_circ_	-0.55976	0.35259
hsa_circ_	-0.11983	0.352594
hsa_circ_	0.984873	0.352598
hsa_circ_	-0.45976	0.352599
hsa_circ_	-0.30804	0.352612
hsa_circ_	-0.21075	0.352636
hsa_circ_	-0.1854	0.352646
hsa_circ_	0.484124	0.352658
hsa_circ_	0.451063	0.352662
hsa_circ_	-0.16047	0.352665
hsa_circ_	0.598852	0.352682
hsa_circ_	-0.20026	0.352684
hsa_circ_	-0.18096	0.35269
hsa_circ_	0.409943	0.35272
hsa_circ_	-1.08988	0.352727
hsa_circ_	0.24103	0.352727
hsa_circ_	-0.18926	0.352737
hsa_circ_	0.336544	0.352749
hsa_circ_	0.625254	0.35275
hsa_circ_	-0.2119	0.352751
hsa_circ_	0.201979	0.352771
hsa_circ_	0.129377	0.352802

hsa_circ_	-0.25502	0.352823
hsa_circ_	-0.13738	0.352842
hsa_circ_	-0.42829	0.352846
hsa_circ_	0.123533	0.352849
hsa_circ_	-2.5346	0.352851
hsa_circ_	0.358939	0.35287
hsa_circ_	0.418616	0.352871
hsa_circ_	0.148833	0.352893
hsa_circ_	-0.51123	0.35291
hsa_circ_	-0.27492	0.352912
hsa_circ_	-0.23808	0.352966
hsa_circ_	-0.45877	0.35297
hsa_circ_	0.194379	0.352973
hsa_circ_	-0.35717	0.352976
hsa_circ_	-0.14504	0.352977
hsa_circ_	-0.22586	0.35298
hsa_circ_	-0.15525	0.352996
hsa_circ_	0.450919	0.353021
hsa_circ_	0.354426	0.353038
hsa_circ_	0.196159	0.353042
hsa_circ_	0.343435	0.353072
hsa_circ_	-0.33505	0.353089
hsa_circ_	0.362992	0.3531
hsa_circ_	0.163958	0.353102
hsa_circ_	-0.15764	0.353103
hsa_circ_	0.149381	0.353109
hsa_circ_	-0.18386	0.35313
hsa_circ_	0.309705	0.353135
hsa_circ_	-0.20629	0.35314
hsa_circ_	-0.23412	0.353153
hsa_circ_	-0.20658	0.353153
hsa_circ_	0.183145	0.353154
hsa_circ_	0.347922	0.353227
hsa_circ_	-0.15423	0.353232
hsa_circ_	0.253007	0.353243
hsa_circ_	-0.3598	0.353267
hsa_circ_	-0.17974	0.353275
hsa_circ_	-0.31271	0.353277
hsa_circ_	-0.60187	0.353285
hsa_circ_	0.134451	0.353298
hsa_circ_	-0.12881	0.353299
hsa_circ_	0.209681	0.353315
hsa_circ_	-0.23935	0.353332
hsa_circ_	0.160623	0.353333
hsa_circ_	-0.16483	0.353344
hsa_circ_	0.197245	0.353356
hsa_circ_	-0.14985	0.353375
hsa_circ_	-0.1659	0.353399
hsa_circ_	-0.14944	0.353412
hsa_circ_	-0.1566	0.353427
hsa_circ_	-0.43729	0.353429
hsa_circ_	-0.18031	0.353431
hsa_circ_	-0.73105	0.353432
hsa_circ_	0.312917	0.353449

hsa_circ_	0.508055	0.353458
hsa_circ_	-0.25222	0.353478
hsa_circ_	-0.31034	0.353529
hsa_circ_	0.327171	0.353582
hsa_circ_	0.24485	0.353598
hsa_circ_	-0.14327	0.353606
hsa_circ_	0.165241	0.353618
hsa_circ_	-0.19345	0.353632
hsa_circ_	0.143346	0.353649
hsa_circ_	-0.13829	0.35365
hsa_circ_	-0.30569	0.353652
hsa_circ_	-0.15534	0.353672
hsa_circ_	0.289486	0.353677
hsa_circ_	0.152808	0.353722
hsa_circ_	0.190413	0.353732
hsa_circ_	0.546385	0.353748
hsa_circ_	0.238711	0.353772
hsa_circ_	0.421786	0.353777
hsa_circ_	-0.17059	0.353789
hsa_circ_	0.232665	0.353799
hsa_circ_	0.296913	0.353803
hsa_circ_	-0.23722	0.353811
hsa_circ_	0.436087	0.353822
hsa_circ_	-0.1396	0.353832
hsa_circ_	0.146289	0.353837
hsa_circ_	-0.32594	0.35385
hsa_circ_	-0.19867	0.353858
hsa_circ_	-0.32196	0.353859
hsa_circ_	0.133357	0.353874
hsa_circ_	0.486599	0.353883
hsa_circ_	0.162905	0.353898
hsa_circ_	-0.15693	0.353902
hsa_circ_	-0.21686	0.353914
hsa_circ_	-0.17976	0.35393
hsa_circ_	0.197289	0.35395
hsa_circ_	-0.46746	0.353952
hsa_circ_	0.168957	0.353962
hsa_circ_	0.136632	0.353969
hsa_circ_	-0.25471	0.35399
hsa_circ_	0.253362	0.354005
hsa_circ_	-0.20898	0.354006
hsa_circ_	0.25685	0.354039
hsa_circ_	-0.14568	0.354048
hsa_circ_	-0.15564	0.354057
hsa_circ_	0.16717	0.35406
hsa_circ_	-0.1903	0.354075
hsa_circ_	0.150487	0.354102
hsa_circ_	0.174262	0.354119
hsa_circ_	-0.33666	0.354122
hsa_circ_	0.153017	0.35413
hsa_circ_	0.181683	0.354134
hsa_circ_	-0.15375	0.354142
hsa_circ_	-0.20133	0.354145
hsa_circ_	-0.20597	0.354153

hsa_circ_	0.374261	0.354161
hsa_circ_	0.209817	0.354177
hsa_circ_	0.142874	0.354187
hsa_circ_	0.338537	0.354194
hsa_circ_	0.150461	0.354207
hsa_circ_	-0.14676	0.354216
hsa_circ_	-0.34155	0.354232
hsa_circ_	-0.29751	0.354244
hsa_circ_	0.190796	0.354267
hsa_circ_	-0.18503	0.354271
hsa_circ_	0.126633	0.354278
hsa_circ_	0.419534	0.354278
hsa_circ_	-0.24849	0.354283
hsa_circ_	-0.14616	0.35429
hsa_circ_	0.412504	0.354293
hsa_circ_	-0.19981	0.354303
hsa_circ_	-0.1854	0.354339
hsa_circ_	0.315341	0.354341
hsa_circ_	0.98617	0.354354
hsa_circ_	0.155681	0.35436
hsa_circ_	0.574187	0.354365
hsa_circ_	-0.73335	0.354394
hsa_circ_	-0.16864	0.354399
hsa_circ_	-0.1518	0.354404
hsa_circ_	-0.1468	0.354436
hsa_circ_	0.267721	0.354441
hsa_circ_	-0.18245	0.354447
hsa_circ_	-0.13857	0.354452
hsa_circ_	0.684316	0.354477
hsa_circ_	-0.1349	0.354482
hsa_circ_	-0.13596	0.354487
hsa_circ_	0.534027	0.354488
hsa_circ_	-0.13305	0.354494
hsa_circ_	0.162603	0.354517
hsa_circ_	0.219655	0.354521
hsa_circ_	-0.41119	0.35454
hsa_circ_	-0.13899	0.354578
hsa_circ_	-0.16968	0.354596
hsa_circ_	0.477529	0.35464
hsa_circ_	-1.03807	0.354641
hsa_circ_	0.459611	0.354645
hsa_circ_	-0.22086	0.354653
hsa_circ_	-0.2543	0.354666
hsa_circ_	0.127216	0.354692
hsa_circ_	-0.13188	0.354696
hsa_circ_	-0.24902	0.354722
hsa_circ_	0.152296	0.354725
hsa_circ_	-0.36066	0.354735
hsa_circ_	0.152246	0.354757
hsa_circ_	-0.15678	0.354761
hsa_circ_	-0.25962	0.354767
hsa_circ_	0.160513	0.35478
hsa_circ_	0.22148	0.354808
hsa_circ_	0.174264	0.354811

hsa_circ_	-0.66902	0.354811
hsa_circ_	-0.19391	0.354822
hsa_circ_	0.188232	0.354832
hsa_circ_	-0.25475	0.354866
hsa_circ_	0.355744	0.354883
hsa_circ_	-0.18433	0.35489
hsa_circ_	-0.17848	0.354894
hsa_circ_	-0.64986	0.354897
hsa_circ_	0.192075	0.354902
hsa_circ_	0.365922	0.354909
hsa_circ_	-0.48766	0.35491
hsa_circ_	0.144964	0.354924
hsa_circ_	0.213629	0.354955
hsa_circ_	0.151637	0.354969
hsa_circ_	-0.15617	0.354986
hsa_circ_	0.330537	0.355006
hsa_circ_	0.284148	0.35501
hsa_circ_	-0.15769	0.355029
hsa_circ_	-0.22925	0.355052
hsa_circ_	-0.24653	0.355054
hsa_circ_	-0.17815	0.355066
hsa_circ_	0.309346	0.355068
hsa_circ_	-0.13911	0.355088
hsa_circ_	-0.22895	0.355101
hsa_circ_	0.165863	0.355108
hsa_circ_	-0.13005	0.355134
hsa_circ_	-0.33066	0.35514
hsa_circ_	-0.14154	0.355141
hsa_circ_	0.201315	0.355143
hsa_circ_	0.317273	0.355143
hsa_circ_	-0.19393	0.355149
hsa_circ_	-0.42816	0.355198
hsa_circ_	0.20198	0.355224
hsa_circ_	-0.42692	0.355231
hsa_circ_	0.673716	0.355234
hsa_circ_	0.137637	0.355247
hsa_circ_	0.238348	0.355257
hsa_circ_	-0.43661	0.355285
hsa_circ_	0.15366	0.355312
hsa_circ_	0.154789	0.355331
hsa_circ_	-0.21362	0.355344
hsa_circ_	0.182992	0.355378
hsa_circ_	-0.13863	0.355389
hsa_circ_	0.141833	0.355404
hsa_circ_	-0.90435	0.355434
hsa_circ_	-0.37748	0.355438
hsa_circ_	0.32876	0.35544
hsa_circ_	-0.40187	0.35545
hsa_circ_	-0.15768	0.355473
hsa_circ_	-0.27361	0.355475
hsa_circ_	0.446874	0.355483
hsa_circ_	-0.28514	0.355496
hsa_circ_	-0.50263	0.3555
hsa_circ_	-0.22232	0.355508

hsa_circ_	0.169937	0.355522
hsa_circ_	0.201509	0.355523
hsa_circ_	0.303394	0.355529
hsa_circ_	-0.5817	0.355535
hsa_circ_	0.188176	0.355542
hsa_circ_	0.372525	0.355543
hsa_circ_	-0.81384	0.355562
hsa_circ_	0.234068	0.355582
hsa_circ_	-0.12507	0.355599
hsa_circ_	-0.5829	0.355599
hsa_circ_	0.188181	0.355632
hsa_circ_	-0.21486	0.355636
hsa_circ_	-0.16233	0.355645
hsa_circ_	0.199843	0.35565
hsa_circ_	-0.14981	0.355651
hsa_circ_	-0.27876	0.355652
hsa_circ_	0.286065	0.355659
hsa_circ_	-0.33149	0.355686
hsa_circ_	0.241315	0.355688
hsa_circ_	0.194007	0.355691
hsa_circ_	-0.13978	0.355692
hsa_circ_	0.198308	0.355694
hsa_circ_	-0.26015	0.355701
hsa_circ_	-3.02	0.355704
hsa_circ_	0.227534	0.355728
hsa_circ_	-0.32013	0.355737
hsa_circ_	0.562807	0.355743
hsa_circ_	-0.20387	0.355743
hsa_circ_	0.534357	0.355753
hsa_circ_	-0.21736	0.355756
hsa_circ_	-0.16273	0.355761
hsa_circ_	-0.14996	0.355767
hsa_circ_	-0.15868	0.355785
hsa_circ_	-0.16458	0.355793
hsa_circ_	0.201836	0.355806
hsa_circ_	-0.18853	0.355844
hsa_circ_	-0.23835	0.355848
hsa_circ_	0.150869	0.355881
hsa_circ_	-0.44754	0.355882
hsa_circ_	0.384488	0.355888
hsa_circ_	-0.16579	0.3559
hsa_circ_	0.164042	0.355915
hsa_circ_	-0.14544	0.355936
hsa_circ_	0.2528	0.355954
hsa_circ_	0.15532	0.355975
hsa_circ_	0.209539	0.355987
hsa_circ_	0.150578	0.355989
hsa_circ_	-0.30681	0.355997
hsa_circ_	0.161344	0.35602
hsa_circ_	0.299873	0.356045
hsa_circ_	-0.2833	0.356066
hsa_circ_	-0.13653	0.356114
hsa_circ_	0.215696	0.356114
hsa_circ_	-0.17208	0.356136

hsa_circ_	0.160956	0.356143
hsa_circ_	-0.26547	0.356166
hsa_circ_	0.160324	0.356171
hsa_circ_	-0.1737	0.356187
hsa_circ_	-0.15621	0.356223
hsa_circ_	-0.26021	0.356247
hsa_circ_	0.499766	0.356252
hsa_circ_	0.231672	0.356259
hsa_circ_	0.16692	0.356261
hsa_circ_	0.206602	0.356262
hsa_circ_	-0.22441	0.356264
hsa_circ_	0.218137	0.356267
hsa_circ_	0.159347	0.356269
hsa_circ_	-0.29112	0.356276
hsa_circ_	0.153301	0.3563
hsa_circ_	0.391578	0.356301
hsa_circ_	0.579804	0.356302
hsa_circ_	0.141092	0.356336
hsa_circ_	0.260717	0.356398
hsa_circ_	-0.14936	0.356404
hsa_circ_	0.332756	0.356407
hsa_circ_	-0.18117	0.356408
hsa_circ_	-0.31503	0.35641
hsa_circ_	0.129863	0.356433
hsa_circ_	-0.28162	0.356492
hsa_circ_	0.252171	0.356522
hsa_circ_	-0.19127	0.356524
hsa_circ_	-0.20055	0.356552
hsa_circ_	0.293985	0.356553
hsa_circ_	-0.1289	0.356565
hsa_circ_	-0.27157	0.356573
hsa_circ_	0.174164	0.356578
hsa_circ_	0.542339	0.356579
hsa_circ_	0.285827	0.356593
hsa_circ_	0.228876	0.356598
hsa_circ_	0.209773	0.356603
hsa_circ_	0.176393	0.356626
hsa_circ_	0.190195	0.356635
hsa_circ_	-0.39199	0.356642
hsa_circ_	-0.18398	0.356657
hsa_circ_	-0.17338	0.356671
hsa_circ_	0.34992	0.356684
hsa_circ_	0.558386	0.356708
hsa_circ_	0.477214	0.356732
hsa_circ_	0.126413	0.356753
hsa_circ_	0.335295	0.356754
hsa_circ_	-0.33371	0.356759
hsa_circ_	0.314067	0.356777
hsa_circ_	0.764764	0.356777
hsa_circ_	0.199452	0.356794
hsa_circ_	-0.37935	0.356819
hsa_circ_	-0.39372	0.356842
hsa_circ_	-0.48026	0.356863
hsa_circ_	0.532	0.356905

hsa_circ_	-1.96379	0.35692
hsa_circ_	0.459013	0.356943
hsa_circ_	-0.1232	0.356948
hsa_circ_	0.146545	0.356954
hsa_circ_	0.348879	0.356955
hsa_circ_	0.44328	0.356961
hsa_circ_	-0.20481	0.356967
hsa_circ_	0.137194	0.356975
hsa_circ_	-0.32101	0.356982
hsa_circ_	0.283567	0.35699
hsa_circ_	-0.15372	0.356993
hsa_circ_	0.382838	0.356996
hsa_circ_	-0.41501	0.356997
hsa_circ_	-0.22008	0.357
hsa_circ_	0.16506	0.357004
hsa_circ_	0.219483	0.357013
hsa_circ_	-0.37921	0.357015
hsa_circ_	0.217329	0.357025
hsa_circ_	-0.19765	0.35703
hsa_circ_	-0.14719	0.357031
hsa_circ_	-0.41397	0.35704
hsa_circ_	-0.15797	0.357062
hsa_circ_	-0.25177	0.357095
hsa_circ_	-0.11844	0.357107
hsa_circ_	0.23314	0.357145
hsa_circ_	0.167802	0.357148
hsa_circ_	0.280477	0.357153
hsa_circ_	0.199129	0.357191
hsa_circ_	0.31765	0.357206
hsa_circ_	-0.28608	0.357218
hsa_circ_	-0.2211	0.357225
hsa_circ_	-0.17787	0.357228
hsa_circ_	-0.28257	0.357233
hsa_circ_	0.25137	0.357244
hsa_circ_	0.138871	0.35725
hsa_circ_	-0.18542	0.357259
hsa_circ_	0.203841	0.357261
hsa_circ_	-0.35061	0.357262
hsa_circ_	-0.37139	0.357273
hsa_circ_	-0.29865	0.357282
hsa_circ_	0.161924	0.357293
hsa_circ_	0.149902	0.35731
hsa_circ_	-0.13861	0.357311
hsa_circ_	0.387769	0.357338
hsa_circ_	-0.18255	0.35735
hsa_circ_	-0.40096	0.357377
hsa_circ_	0.179737	0.357378
hsa_circ_	-0.45971	0.357388
hsa_circ_	-0.14242	0.357402
hsa_circ_	0.772832	0.357417
hsa_circ_	0.48033	0.35743
hsa_circ_	0.212496	0.357453
hsa_circ_	-0.1536	0.357462
hsa_circ_	-0.24684	0.357466

hsa_circ_	-0.54689	0.357469
hsa_circ_	0.242416	0.357493
hsa_circ_	-0.14003	0.3575
hsa_circ_	0.137237	0.357503
hsa_circ_	0.273585	0.357544
hsa_circ_	0.180285	0.357573
hsa_circ_	-0.5593	0.357581
hsa_circ_	0.235059	0.3576
hsa_circ_	0.160895	0.357608
hsa_circ_	0.234838	0.357652
hsa_circ_	-0.16222	0.357706
hsa_circ_	-0.42754	0.357708
hsa_circ_	-0.19086	0.357725
hsa_circ_	0.204361	0.357751
hsa_circ_	0.251458	0.357777
hsa_circ_	-0.83662	0.357778
hsa_circ_	0.133568	0.357785
hsa_circ_	0.167459	0.357801
hsa_circ_	-0.1721	0.357827
hsa_circ_	0.347185	0.35783
hsa_circ_	-0.15602	0.357834
hsa_circ_	0.202746	0.35785
hsa_circ_	0.16659	0.357858
hsa_circ_	-0.15842	0.357874
hsa_circ_	-0.13644	0.357928
hsa_circ_	0.163373	0.357929
hsa_circ_	-0.20871	0.357955
hsa_circ_	0.401242	0.357959
hsa_circ_	0.209301	0.35797
hsa_circ_	-0.14388	0.357973
hsa_circ_	0.149015	0.357978
hsa_circ_	-0.22854	0.357979
hsa_circ_	-0.14064	0.357988
hsa_circ_	-0.55808	0.358005
hsa_circ_	0.151619	0.358024
hsa_circ_	0.172521	0.358025
hsa_circ_	0.167119	0.358045
hsa_circ_	0.14913	0.358049
hsa_circ_	-0.13889	0.358055
hsa_circ_	-0.12699	0.358061
hsa_circ_	-0.20226	0.358073
hsa_circ_	0.216456	0.358073
hsa_circ_	-0.40064	0.358097
hsa_circ_	-0.24745	0.358099
hsa_circ_	0.286625	0.358117
hsa_circ_	0.138918	0.358133
hsa_circ_	0.194947	0.358136
hsa_circ_	-0.14216	0.358144
hsa_circ_	-0.21814	0.358174
hsa_circ_	-0.13154	0.358184
hsa_circ_	-0.41473	0.3582
hsa_circ_	0.272338	0.358212
hsa_circ_	0.162939	0.358257
hsa_circ_	-0.17992	0.358273

hsa_circ_	0.278695	0.35829
hsa_circ_	0.237637	0.358302
hsa_circ_	0.137393	0.358315
hsa_circ_	0.266273	0.358346
hsa_circ_	-0.12035	0.358353
hsa_circ_	-0.49844	0.358355
hsa_circ_	0.133288	0.35837
hsa_circ_	-0.17922	0.358391
hsa_circ_	-0.15938	0.358391
hsa_circ_	0.156099	0.358406
hsa_circ_	0.226399	0.35841
hsa_circ_	-0.16034	0.358419
hsa_circ_	0.212095	0.35846
hsa_circ_	0.45466	0.358464
hsa_circ_	-0.20856	0.358465
hsa_circ_	0.295778	0.358487
hsa_circ_	-0.14753	0.358502
hsa_circ_	-0.41507	0.358513
hsa_circ_	-0.16122	0.358539
hsa_circ_	0.317781	0.358547
hsa_circ_	-0.28456	0.35856
hsa_circ_	0.268155	0.358581
hsa_circ_	0.464856	0.35859
hsa_circ_	0.175489	0.358599
hsa_circ_	-0.44987	0.358599
hsa_circ_	0.226747	0.358601
hsa_circ_	-0.21838	0.358619
hsa_circ_	-0.12424	0.358621
hsa_circ_	-0.26668	0.358623
hsa_circ_	0.590036	0.35863
hsa_circ_	-0.26	0.358637
hsa_circ_	-0.49325	0.358658
hsa_circ_	-0.46378	0.358661
hsa_circ_	0.376646	0.358673
hsa_circ_	-0.16529	0.358674
hsa_circ_	-0.23649	0.358675
hsa_circ_	0.161043	0.358678
hsa_circ_	0.145713	0.358679
hsa_circ_	-0.51483	0.358709
hsa_circ_	-0.46989	0.358741
hsa_circ_	0.461472	0.358748
hsa_circ_	-0.48548	0.35875
hsa_circ_	-0.1715	0.358756
hsa_circ_	-0.79683	0.358782
hsa_circ_	-0.14671	0.358789
hsa_circ_	-0.21433	0.35881
hsa_circ_	-0.15364	0.358818
hsa_circ_	-0.1552	0.358837
hsa_circ_	0.125339	0.358841
hsa_circ_	0.211862	0.358899
hsa_circ_	-0.18193	0.358903
hsa_circ_	-0.17247	0.358912
hsa_circ_	-0.19903	0.35893
hsa_circ_	-0.14605	0.358934

hsa_circ_	0.652062	0.358944
hsa_circ_	-0.44583	0.358986
hsa_circ_	-0.31546	0.358989
hsa_circ_	-0.16454	0.358991
hsa_circ_	0.125534	0.358998
hsa_circ_	-0.15833	0.359032
hsa_circ_	0.148165	0.359053
hsa_circ_	-0.5172	0.359056
hsa_circ_	1.601212	0.359076
hsa_circ_	0.212641	0.359099
hsa_circ_	0.339466	0.359104
hsa_circ_	-0.18185	0.359106
hsa_circ_	0.199657	0.359128
hsa_circ_	-0.5278	0.359134
hsa_circ_	-0.15796	0.359152
hsa_circ_	0.536966	0.359157
hsa_circ_	0.453703	0.359172
hsa_circ_	-0.35003	0.359181
hsa_circ_	-0.18085	0.359184
hsa_circ_	-0.32083	0.359185
hsa_circ_	0.483786	0.359206
hsa_circ_	0.1533	0.359211
hsa_circ_	-0.13159	0.359231
hsa_circ_	0.225179	0.359249
hsa_circ_	0.424158	0.359249
hsa_circ_	-1.18179	0.359251
hsa_circ_	-0.23108	0.359252
hsa_circ_	0.234892	0.359253
hsa_circ_	-0.11879	0.359275
hsa_circ_	-0.37101	0.359291
hsa_circ_	-0.14409	0.359299
hsa_circ_	-0.13205	0.359322
hsa_circ_	-0.13755	0.35935
hsa_circ_	-0.14149	0.359377
hsa_circ_	0.200662	0.359391
hsa_circ_	0.498495	0.359428
hsa_circ_	0.196891	0.359429
hsa_circ_	-0.29864	0.359445
hsa_circ_	0.220105	0.35946
hsa_circ_	0.15145	0.359466
hsa_circ_	0.428292	0.359475
hsa_circ_	-0.15531	0.359498
hsa_circ_	0.197352	0.35955
hsa_circ_	-0.37212	0.35959
hsa_circ_	-0.26343	0.359602
hsa_circ_	-0.32586	0.359605
hsa_circ_	-0.15924	0.359608
hsa_circ_	-0.36244	0.359616
hsa_circ_	-0.16584	0.359641
hsa_circ_	0.155043	0.359646
hsa_circ_	0.140398	0.359652
hsa_circ_	-0.24122	0.359664
hsa_circ_	-0.34391	0.359689
hsa_circ_	-0.632	0.359704

hsa_circ_	-0.18797	0.359721
hsa_circ_	-0.16815	0.359726
hsa_circ_	0.125751	0.359751
hsa_circ_	0.134819	0.359753
hsa_circ_	-0.16486	0.359758
hsa_circ_	-0.79662	0.359764
hsa_circ_	0.334294	0.359772
hsa_circ_	0.741737	0.359778
hsa_circ_	0.151207	0.359789
hsa_circ_	0.215819	0.35979
hsa_circ_	-0.3739	0.359799
hsa_circ_	0.130824	0.359816
hsa_circ_	0.449244	0.359827
hsa_circ_	0.269519	0.359848
hsa_circ_	0.158516	0.359851
hsa_circ_	-0.37454	0.359852
hsa_circ_	-0.26713	0.359867
hsa_circ_	0.128216	0.359868
hsa_circ_	-0.17895	0.359874
hsa_circ_	0.306975	0.359879
hsa_circ_	0.140547	0.359883
hsa_circ_	0.144073	0.359892
hsa_circ_	0.820057	0.359901
hsa_circ_	0.234651	0.359908
hsa_circ_	0.151458	0.359916
hsa_circ_	-0.24587	0.359935
hsa_circ_	0.156076	0.359943
hsa_circ_	-0.14404	0.359978
hsa_circ_	0.243611	0.359997
hsa_circ_	0.199116	0.360002
hsa_circ_	0.337108	0.360006
hsa_circ_	-0.32433	0.360017
hsa_circ_	0.300268	0.360028
hsa_circ_	-1.0722	0.360037
hsa_circ_	-0.59435	0.360084
hsa_circ_	0.219242	0.360085
hsa_circ_	-1.71011	0.360088
hsa_circ_	-0.30069	0.360095
hsa_circ_	-0.13884	0.360102
hsa_circ_	-0.40546	0.360108
hsa_circ_	-0.3829	0.360119
hsa_circ_	-0.60073	0.360142
hsa_circ_	-0.18072	0.360143
hsa_circ_	0.266652	0.360165
hsa_circ_	0.147946	0.360166
hsa_circ_	0.165189	0.360167
hsa_circ_	0.348072	0.360223
hsa_circ_	0.21069	0.360234
hsa_circ_	-0.30574	0.360258
hsa_circ_	0.217974	0.36026
hsa_circ_	0.305628	0.360277
hsa_circ_	-0.16887	0.360299
hsa_circ_	0.145184	0.360317
hsa_circ_	0.165794	0.360318

hsa_circ_	-0.48012	0.360318
hsa_circ_	-0.20785	0.360321
hsa_circ_	-0.18869	0.360327
hsa_circ_	-0.19116	0.36034
hsa_circ_	-0.30235	0.360352
hsa_circ_	-0.13806	0.36037
hsa_circ_	-0.19266	0.360382
hsa_circ_	-0.34635	0.360387
hsa_circ_	0.292701	0.360398
hsa_circ_	0.329983	0.3604
hsa_circ_	0.399444	0.360402
hsa_circ_	0.148854	0.360407
hsa_circ_	0.281911	0.360417
hsa_circ_	-0.16375	0.36042
hsa_circ_	-0.13377	0.36043
hsa_circ_	-0.11826	0.360434
hsa_circ_	0.164188	0.360442
hsa_circ_	-0.15668	0.360445
hsa_circ_	-0.23068	0.360448
hsa_circ_	0.251666	0.360451
hsa_circ_	0.234126	0.360465
hsa_circ_	0.187305	0.360475
hsa_circ_	0.283558	0.360497
hsa_circ_	-0.27168	0.360532
hsa_circ_	0.229571	0.360539
hsa_circ_	0.294329	0.36054
hsa_circ_	-0.19924	0.360543
hsa_circ_	-0.16985	0.360544
hsa_circ_	-0.32027	0.360554
hsa_circ_	0.16737	0.360561
hsa_circ_	0.474549	0.360561
hsa_circ_	0.357549	0.360567
hsa_circ_	0.277354	0.360568
hsa_circ_	-0.15392	0.360577
hsa_circ_	0.476884	0.360591
hsa_circ_	-0.26932	0.360597
hsa_circ_	-0.16624	0.36065
hsa_circ_	-0.18221	0.360669
hsa_circ_	-0.16221	0.36067
hsa_circ_	-0.27871	0.360673
hsa_circ_	-0.14758	0.360682
hsa_circ_	0.132908	0.360696
hsa_circ_	0.217348	0.360698
hsa_circ_	-0.15909	0.360706
hsa_circ_	0.557161	0.360752
hsa_circ_	0.217348	0.360776
hsa_circ_	-0.24321	0.360819
hsa_circ_	-0.17257	0.36082
hsa_circ_	-0.52225	0.360843
hsa_circ_	0.230366	0.360849
hsa_circ_	0.231093	0.36086
hsa_circ_	0.206714	0.360868
hsa_circ_	0.258404	0.360871
hsa_circ_	-0.16359	0.360873

hsa_circ_	-0.16467	0.360902
hsa_circ_	-0.42531	0.360912
hsa_circ_	-0.12674	0.360931
hsa_circ_	-0.43251	0.360946
hsa_circ_	0.153549	0.360947
hsa_circ_	-0.16326	0.360984
hsa_circ_	-0.40454	0.360992
hsa_circ_	0.311052	0.360998
hsa_circ_	-0.13856	0.361013
hsa_circ_	-0.33211	0.361016
hsa_circ_	0.139167	0.361059
hsa_circ_	0.161012	0.361063
hsa_circ_	0.206542	0.361066
hsa_circ_	0.181671	0.361075
hsa_circ_	0.531685	0.361087
hsa_circ_	1.082141	0.361107
hsa_circ_	0.148743	0.361109
hsa_circ_	0.170614	0.361113
hsa_circ_	0.140227	0.361116
hsa_circ_	-0.20356	0.361163
hsa_circ_	0.275518	0.361175
hsa_circ_	-0.12375	0.361178
hsa_circ_	0.176723	0.361182
hsa_circ_	-0.18153	0.361185
hsa_circ_	0.130095	0.361192
hsa_circ_	-0.18022	0.361204
hsa_circ_	-0.19741	0.361219
hsa_circ_	-0.25377	0.361221
hsa_circ_	-0.21889	0.361255
hsa_circ_	-0.40635	0.361258
hsa_circ_	-0.12873	0.361284
hsa_circ_	-0.1256	0.361293
hsa_circ_	-0.15789	0.361294
hsa_circ_	0.648521	0.361299
hsa_circ_	-0.17065	0.361316
hsa_circ_	-0.14222	0.361325
hsa_circ_	-0.21558	0.361331
hsa_circ_	-0.38171	0.361361
hsa_circ_	-0.5206	0.361383
hsa_circ_	0.175152	0.361391
hsa_circ_	0.296299	0.361394
hsa_circ_	0.211802	0.361407
hsa_circ_	-0.12641	0.36141
hsa_circ_	-0.12056	0.361412
hsa_circ_	0.206017	0.361443
hsa_circ_	-0.23802	0.361473
hsa_circ_	0.199596	0.361488
hsa_circ_	-0.1661	0.36149
hsa_circ_	-0.30166	0.361493
hsa_circ_	0.230026	0.361508
hsa_circ_	0.303057	0.361528
hsa_circ_	-0.25692	0.361543
hsa_circ_	-0.22465	0.361562
hsa_circ_	-0.14946	0.361565

hsa_circ_	-0.16099	0.361595
hsa_circ_	0.129672	0.361596
hsa_circ_	-0.20954	0.361598
hsa_circ_	0.194422	0.361602
hsa_circ_	0.122211	0.361604
hsa_circ_	0.137919	0.361622
hsa_circ_	0.271316	0.361638
hsa_circ_	-0.1664	0.36164
hsa_circ_	0.293046	0.36165
hsa_circ_	0.17697	0.36168
hsa_circ_	0.180031	0.361683
hsa_circ_	-0.50902	0.361685
hsa_circ_	-0.25626	0.361695
hsa_circ_	0.155338	0.3617
hsa_circ_	0.288558	0.361701
hsa_circ_	0.23302	0.361704
hsa_circ_	-0.36019	0.361718
hsa_circ_	0.166939	0.361723
hsa_circ_	-0.44374	0.36174
hsa_circ_	-0.12251	0.361751
hsa_circ_	-0.43425	0.361752
hsa_circ_	0.151972	0.361756
hsa_circ_	-0.40389	0.36176
hsa_circ_	0.167191	0.36177
hsa_circ_	-0.16995	0.361771
hsa_circ_	-0.1609	0.361772
hsa_circ_	-0.25527	0.361775
hsa_circ_	0.169078	0.361788
hsa_circ_	-0.14582	0.361838
hsa_circ_	-0.17888	0.361841
hsa_circ_	-0.20046	0.361857
hsa_circ_	0.121816	0.361864
hsa_circ_	-0.26757	0.36187
hsa_circ_	-0.22992	0.361885
hsa_circ_	0.166125	0.361886
hsa_circ_	0.161349	0.361907
hsa_circ_	0.140492	0.361919
hsa_circ_	0.314976	0.361922
hsa_circ_	-0.44852	0.361927
hsa_circ_	0.167186	0.361944
hsa_circ_	0.199896	0.361972
hsa_circ_	-0.21308	0.36198
hsa_circ_	-0.24696	0.36198
hsa_circ_	0.134708	0.361993
hsa_circ_	-0.14419	0.362019
hsa_circ_	-0.28264	0.362023
hsa_circ_	-0.82045	0.362055
hsa_circ_	-0.21566	0.362062
hsa_circ_	0.237966	0.362069
hsa_circ_	0.184587	0.362089
hsa_circ_	-0.18636	0.362111
hsa_circ_	0.170722	0.362117
hsa_circ_	-0.59399	0.362124
hsa_circ_	0.226211	0.362131

hsa_circ_	0.352855	0.362158
hsa_circ_	-0.25057	0.362159
hsa_circ_	0.124929	0.362168
hsa_circ_	-0.287	0.362186
hsa_circ_	0.705738	0.362204
hsa_circ_	-0.21177	0.362211
hsa_circ_	-0.1563	0.362217
hsa_circ_	-0.12042	0.362227
hsa_circ_	0.210037	0.362236
hsa_circ_	0.454769	0.362247
hsa_circ_	0.262099	0.362274
hsa_circ_	0.224744	0.36228
hsa_circ_	0.217442	0.362328
hsa_circ_	0.332617	0.362331
hsa_circ_	-0.1581	0.362332
hsa_circ_	-0.16539	0.36238
hsa_circ_	0.161847	0.362389
hsa_circ_	0.245059	0.362395
hsa_circ_	0.181784	0.362403
hsa_circ_	-0.15605	0.362413
hsa_circ_	-0.1255	0.362419
hsa_circ_	0.319645	0.362434
hsa_circ_	-0.15779	0.362442
hsa_circ_	0.164982	0.362451
hsa_circ_	0.943373	0.362453
hsa_circ_	-0.56424	0.362465
hsa_circ_	-0.15011	0.362494
hsa_circ_	0.153886	0.36251
hsa_circ_	0.175993	0.36251
hsa_circ_	0.157506	0.362519
hsa_circ_	-0.19694	0.362534
hsa_circ_	0.151929	0.362549
hsa_circ_	-0.11782	0.362553
hsa_circ_	-0.36668	0.362554
hsa_circ_	-0.16545	0.362577
hsa_circ_	0.384942	0.362585
hsa_circ_	0.203766	0.362596
hsa_circ_	-0.24675	0.36263
hsa_circ_	-0.13072	0.362632
hsa_circ_	-0.4704	0.362656
hsa_circ_	0.63125	0.362664
hsa_circ_	0.137012	0.362672
hsa_circ_	-0.3814	0.362688
hsa_circ_	0.385668	0.362698
hsa_circ_	0.243368	0.362715
hsa_circ_	0.220491	0.362716
hsa_circ_	0.155022	0.362726
hsa_circ_	0.340473	0.362747
hsa_circ_	-0.12746	0.362769
hsa_circ_	0.580618	0.36278
hsa_circ_	-0.44941	0.362785
hsa_circ_	0.267052	0.362823
hsa_circ_	0.208928	0.362827
hsa_circ_	-0.15378	0.36284

hsa_circ_	0.212395	0.36286
hsa_circ_	-0.27001	0.362882
hsa_circ_	-0.13243	0.362911
hsa_circ_	-0.26502	0.362928
hsa_circ_	0.150333	0.362933
hsa_circ_	-0.17349	0.362955
hsa_circ_	-0.20913	0.362957
hsa_circ_	0.248793	0.362978
hsa_circ_	-0.1923	0.362988
hsa_circ_	-0.12593	0.362996
hsa_circ_	0.190405	0.362998
hsa_circ_	-0.28793	0.363022
hsa_circ_	0.155005	0.363027
hsa_circ_	-0.21565	0.363032
hsa_circ_	-0.14098	0.363041
hsa_circ_	-0.27347	0.363042
hsa_circ_	0.34887	0.36305
hsa_circ_	-0.14044	0.363068
hsa_circ_	-0.15814	0.363092
hsa_circ_	0.275803	0.363094
hsa_circ_	0.303133	0.363101
hsa_circ_	-0.13627	0.363104
hsa_circ_	0.170976	0.363133
hsa_circ_	0.162317	0.36314
hsa_circ_	-0.13504	0.363169
hsa_circ_	-0.13617	0.36317
hsa_circ_	0.204971	0.363196
hsa_circ_	-0.17178	0.363196
hsa_circ_	0.221092	0.363201
hsa_circ_	0.166898	0.363203
hsa_circ_	0.25847	0.36322
hsa_circ_	-0.12984	0.363239
hsa_circ_	0.181854	0.363245
hsa_circ_	0.233541	0.363253
hsa_circ_	-0.12988	0.363273
hsa_circ_	-0.22636	0.363317
hsa_circ_	0.154823	0.363335
hsa_circ_	-0.13749	0.36334
hsa_circ_	-0.24041	0.363357
hsa_circ_	-0.46838	0.363361
hsa_circ_	0.342456	0.363367
hsa_circ_	-0.17751	0.363393
hsa_circ_	-0.13329	0.36346
hsa_circ_	-0.19327	0.363474
hsa_circ_	-0.14423	0.363484
hsa_circ_	-0.30091	0.363492
hsa_circ_	-0.12983	0.363503
hsa_circ_	-0.21298	0.363507
hsa_circ_	0.197033	0.36352
hsa_circ_	0.241341	0.363522
hsa_circ_	-0.12481	0.363542
hsa_circ_	0.496909	0.363576
hsa_circ_	0.231901	0.363578
hsa_circ_	0.273109	0.36359

hsa_circ_	-0.36099	0.363617
hsa_circ_	-0.1779	0.363623
hsa_circ_	0.245229	0.363631
hsa_circ_	-0.2016	0.363631
hsa_circ_	-0.12547	0.363643
hsa_circ_	-0.38513	0.363666
hsa_circ_	-0.6591	0.363698
hsa_circ_	0.460405	0.363715
hsa_circ_	0.372766	0.363717
hsa_circ_	-0.33018	0.363733
hsa_circ_	0.277342	0.363733
hsa_circ_	0.278516	0.363768
hsa_circ_	0.222057	0.363773
hsa_circ_	-0.17025	0.363808
hsa_circ_	-0.62196	0.363812
hsa_circ_	-0.12723	0.363812
hsa_circ_	0.232505	0.363813
hsa_circ_	-0.27613	0.363816
hsa_circ_	-0.1685	0.363822
hsa_circ_	-1.85468	0.363832
hsa_circ_	-0.21802	0.363845
hsa_circ_	0.133699	0.363845
hsa_circ_	-0.22104	0.363847
hsa_circ_	0.286267	0.36386
hsa_circ_	-0.17025	0.36386
hsa_circ_	0.119217	0.363867
hsa_circ_	0.378562	0.363889
hsa_circ_	-0.35533	0.363891
hsa_circ_	-0.37641	0.363897
hsa_circ_	0.279574	0.363899
hsa_circ_	0.206505	0.363899
hsa_circ_	0.124888	0.363905
hsa_circ_	0.121874	0.363942
hsa_circ_	0.16658	0.363975
hsa_circ_	-0.22807	0.363976
hsa_circ_	-0.182	0.364003
hsa_circ_	-0.13811	0.364056
hsa_circ_	-0.21442	0.36406
hsa_circ_	-0.14536	0.364061
hsa_circ_	-0.13907	0.364084
hsa_circ_	0.208785	0.364096
hsa_circ_	0.3703	0.364101
hsa_circ_	0.315991	0.364106
hsa_circ_	0.168615	0.36411
hsa_circ_	0.125293	0.36412
hsa_circ_	-0.15405	0.364122
hsa_circ_	-0.33035	0.364124
hsa_circ_	0.491356	0.364154
hsa_circ_	-0.16448	0.364157
hsa_circ_	0.33486	0.364161
hsa_circ_	0.27985	0.364165
hsa_circ_	-0.19773	0.364174
hsa_circ_	-0.19753	0.364178
hsa_circ_	-0.60374	0.364187

hsa_circ_	-0.26293	0.364196
hsa_circ_	-0.18882	0.3642
hsa_circ_	0.201101	0.364209
hsa_circ_	-0.31871	0.364222
hsa_circ_	-0.35498	0.364266
hsa_circ_	-0.24432	0.364292
hsa_circ_	0.224437	0.364328
hsa_circ_	-0.37653	0.364333
hsa_circ_	-0.19305	0.364333
hsa_circ_	-0.19126	0.364335
hsa_circ_	0.175136	0.364346
hsa_circ_	-0.12674	0.364358
hsa_circ_	0.194592	0.364368
hsa_circ_	0.187897	0.364378
hsa_circ_	-0.14577	0.364384
hsa_circ_	-0.57135	0.36439
hsa_circ_	-0.13836	0.364393
hsa_circ_	-0.13131	0.3644
hsa_circ_	0.274396	0.364404
hsa_circ_	-0.1413	0.36443
hsa_circ_	-0.1971	0.364457
hsa_circ_	-0.12023	0.36446
hsa_circ_	0.50167	0.364463
hsa_circ_	0.136387	0.36452
hsa_circ_	0.151507	0.364554
hsa_circ_	0.15648	0.364558
hsa_circ_	-0.32471	0.364572
hsa_circ_	0.281669	0.364581
hsa_circ_	-0.12643	0.364589
hsa_circ_	0.268844	0.364592
hsa_circ_	-0.35944	0.364601
hsa_circ_	-0.18317	0.364617
hsa_circ_	-0.58829	0.364625
hsa_circ_	0.151216	0.364627
hsa_circ_	-0.13555	0.364631
hsa_circ_	0.129139	0.364635
hsa_circ_	0.157924	0.364637
hsa_circ_	0.141621	0.364645
hsa_circ_	-0.14917	0.364665
hsa_circ_	-0.14103	0.364678
hsa_circ_	-0.18803	0.36473
hsa_circ_	-0.36445	0.364744
hsa_circ_	0.295777	0.364775
hsa_circ_	-0.1891	0.364792
hsa_circ_	0.174192	0.364794
hsa_circ_	-0.1649	0.364817
hsa_circ_	-0.3781	0.364833
hsa_circ_	0.152525	0.364843
hsa_circ_	-0.39785	0.364864
hsa_circ_	-0.42905	0.364866
hsa_circ_	-0.21441	0.364881
hsa_circ_	-0.12567	0.364896
hsa_circ_	-0.15662	0.364926
hsa_circ_	0.287888	0.364927

hsa_circ_	-0.75412	0.364938
hsa_circ_	-0.2592	0.364951
hsa_circ_	0.719233	0.364952
hsa_circ_	0.155275	0.364969
hsa_circ_	0.189522	0.364997
hsa_circ_	-0.13798	0.365021
hsa_circ_	0.165574	0.365068
hsa_circ_	-0.46491	0.365133
hsa_circ_	-0.27054	0.365134
hsa_circ_	0.17584	0.365138
hsa_circ_	-0.12549	0.365149
hsa_circ_	0.166722	0.365156
hsa_circ_	-0.5381	0.365166
hsa_circ_	0.222489	0.365167
hsa_circ_	0.250679	0.365177
hsa_circ_	0.200729	0.365191
hsa_circ_	0.330227	0.365208
hsa_circ_	0.202129	0.365244
hsa_circ_	0.421611	0.365252
hsa_circ_	0.151595	0.365254
hsa_circ_	-0.40031	0.365268
hsa_circ_	0.253114	0.365296
hsa_circ_	0.301784	0.365308
hsa_circ_	-0.49065	0.365324
hsa_circ_	0.160582	0.365336
hsa_circ_	-0.29461	0.365363
hsa_circ_	-0.52557	0.365386
hsa_circ_	0.186045	0.365386
hsa_circ_	-0.17652	0.365393
hsa_circ_	-0.21158	0.365401
hsa_circ_	-0.16719	0.365404
hsa_circ_	0.135279	0.365409
hsa_circ_	0.172215	0.365415
hsa_circ_	0.332443	0.36544
hsa_circ_	0.2445	0.365446
hsa_circ_	0.494294	0.365455
hsa_circ_	-0.51175	0.365456
hsa_circ_	-0.17202	0.365467
hsa_circ_	-0.24397	0.365472
hsa_circ_	-0.24797	0.365493
hsa_circ_	-0.1773	0.365496
hsa_circ_	-0.16892	0.365497
hsa_circ_	0.646728	0.3655
hsa_circ_	0.402285	0.365516
hsa_circ_	0.354517	0.365522
hsa_circ_	0.196222	0.365541
hsa_circ_	-0.23135	0.365544
hsa_circ_	0.152944	0.365548
hsa_circ_	-0.17838	0.365556
hsa_circ_	-0.14724	0.365584
hsa_circ_	0.200365	0.36559
hsa_circ_	-0.27911	0.3656
hsa_circ_	0.143163	0.365618
hsa_circ_	-0.56122	0.365652

hsa_circ_	0.333423	0.365656
hsa_circ_	-0.32146	0.365657
hsa_circ_	0.255615	0.365662
hsa_circ_	0.174886	0.365676
hsa_circ_	0.16843	0.365679
hsa_circ_	0.158428	0.36568
hsa_circ_	-0.17863	0.365687
hsa_circ_	0.140453	0.36569
hsa_circ_	-0.2837	0.365742
hsa_circ_	0.157888	0.365785
hsa_circ_	0.32863	0.365785
hsa_circ_	-0.23638	0.365807
hsa_circ_	-0.13135	0.365825
hsa_circ_	0.156657	0.365835
hsa_circ_	-0.38479	0.365836
hsa_circ_	-0.29873	0.365844
hsa_circ_	0.177379	0.365848
hsa_circ_	-0.23104	0.365864
hsa_circ_	-0.39872	0.365873
hsa_circ_	0.401856	0.365878
hsa_circ_	0.139383	0.365893
hsa_circ_	-0.21926	0.365917
hsa_circ_	0.274627	0.365923
hsa_circ_	-0.56893	0.365956
hsa_circ_	-0.35828	0.365957
hsa_circ_	0.166273	0.365968
hsa_circ_	0.175435	0.365988
hsa_circ_	0.163235	0.366011
hsa_circ_	-0.20505	0.366016
hsa_circ_	-0.14808	0.366024
hsa_circ_	-0.23396	0.366025
hsa_circ_	-0.3067	0.36603
hsa_circ_	-0.32389	0.366031
hsa_circ_	-0.40322	0.366043
hsa_circ_	0.262817	0.366058
hsa_circ_	0.146896	0.366067
hsa_circ_	-0.19096	0.366072
hsa_circ_	0.180292	0.366095
hsa_circ_	-0.14884	0.366121
hsa_circ_	-0.19745	0.366145
hsa_circ_	-0.26304	0.366159
hsa_circ_	0.163482	0.366174
hsa_circ_	-0.28273	0.366179
hsa_circ_	-0.24444	0.366184
hsa_circ_	-0.53634	0.366197
hsa_circ_	0.194027	0.366204
hsa_circ_	0.216838	0.366207
hsa_circ_	-0.19237	0.366213
hsa_circ_	0.230655	0.366215
hsa_circ_	0.389638	0.366215
hsa_circ_	-0.34503	0.36622
hsa_circ_	0.233295	0.366234
hsa_circ_	-0.16509	0.366274
hsa_circ_	-0.36188	0.366287

hsa_circ_	0.219241	0.366301
hsa_circ_	-0.61016	0.366327
hsa_circ_	1.099076	0.366333
hsa_circ_	0.133156	0.366374
hsa_circ_	0.325643	0.366396
hsa_circ_	0.404943	0.366397
hsa_circ_	0.312886	0.366398
hsa_circ_	0.163315	0.366413
hsa_circ_	-0.20418	0.366427
hsa_circ_	0.196175	0.366446
hsa_circ_	-0.21615	0.366462
hsa_circ_	0.340087	0.36648
hsa_circ_	0.165703	0.366536
hsa_circ_	-0.24404	0.36654
hsa_circ_	0.241134	0.366587
hsa_circ_	0.221015	0.366587
hsa_circ_	-0.19639	0.366589
hsa_circ_	-0.27688	0.366605
hsa_circ_	-0.25456	0.366608
hsa_circ_	0.22105	0.366614
hsa_circ_	-0.12699	0.366633
hsa_circ_	0.137953	0.36665
hsa_circ_	0.190126	0.366656
hsa_circ_	-0.1542	0.366656
hsa_circ_	-0.50814	0.366678
hsa_circ_	0.123407	0.366679
hsa_circ_	0.191542	0.36668
hsa_circ_	0.432679	0.366687
hsa_circ_	0.439694	0.366693
hsa_circ_	-0.62325	0.366697
hsa_circ_	0.121783	0.366701
hsa_circ_	-0.74853	0.36671
hsa_circ_	-0.15651	0.366715
hsa_circ_	0.211752	0.366716
hsa_circ_	-0.32138	0.366745
hsa_circ_	-0.23791	0.366821
hsa_circ_	0.217434	0.366825
hsa_circ_	-0.23474	0.366855
hsa_circ_	0.222423	0.366873
hsa_circ_	-0.1563	0.36688
hsa_circ_	-0.16504	0.36692
hsa_circ_	-0.23646	0.366922
hsa_circ_	-0.31317	0.366939
hsa_circ_	-0.30315	0.366943
hsa_circ_	-0.17764	0.366946
hsa_circ_	-0.37135	0.366966
hsa_circ_	0.128957	0.366989
hsa_circ_	-0.1638	0.36699
hsa_circ_	0.159658	0.366996
hsa_circ_	-0.27356	0.367035
hsa_circ_	-0.15849	0.367035
hsa_circ_	0.310892	0.367087
hsa_circ_	-0.15516	0.367096
hsa_circ_	0.197344	0.367106

hsa_circ_	-0.16184	0.367115
hsa_circ_	0.431365	0.367141
hsa_circ_	0.224302	0.367163
hsa_circ_	-0.17549	0.367173
hsa_circ_	0.177063	0.36718
hsa_circ_	-0.21505	0.367184
hsa_circ_	-0.14351	0.367188
hsa_circ_	-0.17631	0.36721
hsa_circ_	-0.22189	0.367224
hsa_circ_	0.364179	0.367225
hsa_circ_	0.387963	0.36724
hsa_circ_	0.389793	0.367264
hsa_circ_	-0.17456	0.367266
hsa_circ_	0.237086	0.367276
hsa_circ_	-0.20512	0.367296
hsa_circ_	-0.1432	0.367299
hsa_circ_	-0.18549	0.367302
hsa_circ_	-0.50848	0.367302
hsa_circ_	-0.51358	0.367321
hsa_circ_	-0.13513	0.367324
hsa_circ_	0.143336	0.367356
hsa_circ_	0.399163	0.36737
hsa_circ_	-0.13893	0.367371
hsa_circ_	0.134158	0.367376
hsa_circ_	-0.15542	0.367404
hsa_circ_	-0.32164	0.367404
hsa_circ_	-0.14972	0.367411
hsa_circ_	-0.14086	0.367428
hsa_circ_	-0.47709	0.367437
hsa_circ_	-0.25329	0.367459
hsa_circ_	-0.14906	0.367471
hsa_circ_	0.324803	0.367514
hsa_circ_	-0.19908	0.36753
hsa_circ_	-0.28439	0.367539
hsa_circ_	0.157531	0.367548
hsa_circ_	-0.37486	0.367563
hsa_circ_	-0.15104	0.367565
hsa_circ_	-0.18137	0.367566
hsa_circ_	-0.18469	0.36757
hsa_circ_	-0.16597	0.367588
hsa_circ_	-0.42599	0.367591
hsa_circ_	-0.12956	0.367596
hsa_circ_	0.172106	0.367601
hsa_circ_	-0.17805	0.367629
hsa_circ_	0.136514	0.367632
hsa_circ_	0.535432	0.367635
hsa_circ_	0.301854	0.367661
hsa_circ_	-0.13554	0.367662
hsa_circ_	-0.14423	0.367672
hsa_circ_	-0.14352	0.367676
hsa_circ_	0.353753	0.367689
hsa_circ_	0.280777	0.367706
hsa_circ_	-0.22366	0.367721
hsa_circ_	0.216272	0.367726

hsa_circ_	0.272159	0.367731
hsa_circ_	-0.23101	0.367736
hsa_circ_	-0.45644	0.367749
hsa_circ_	0.491919	0.367755
hsa_circ_	0.194588	0.367782
hsa_circ_	-0.1204	0.367785
hsa_circ_	0.126491	0.367796
hsa_circ_	0.471508	0.367797
hsa_circ_	0.15874	0.367811
hsa_circ_	-0.18126	0.367829
hsa_circ_	-0.20131	0.367834
hsa_circ_	-0.16722	0.367839
hsa_circ_	-0.22255	0.36784
hsa_circ_	-0.23333	0.367851
hsa_circ_	-0.12548	0.367853
hsa_circ_	0.125151	0.367872
hsa_circ_	-0.13407	0.367881
hsa_circ_	-0.32254	0.367885
hsa_circ_	-0.18012	0.367897
hsa_circ_	-0.42226	0.367913
hsa_circ_	0.1928	0.367916
hsa_circ_	-0.22091	0.367972
hsa_circ_	-0.33702	0.367994
hsa_circ_	-0.14137	0.368
hsa_circ_	0.46448	0.36805
hsa_circ_	-0.3918	0.368061
hsa_circ_	-0.3306	0.368075
hsa_circ_	0.180103	0.368115
hsa_circ_	0.158606	0.368136
hsa_circ_	-0.23968	0.368139
hsa_circ_	-0.15307	0.368147
hsa_circ_	-0.12077	0.368177
hsa_circ_	-0.34242	0.368199
hsa_circ_	0.834781	0.368207
hsa_circ_	-0.62121	0.36822
hsa_circ_	0.273932	0.368226
hsa_circ_	-0.55978	0.368235
hsa_circ_	0.236948	0.368236
hsa_circ_	-0.1482	0.36825
hsa_circ_	0.256361	0.368274
hsa_circ_	0.265002	0.368297
hsa_circ_	-0.15167	0.368304
hsa_circ_	0.267945	0.368318
hsa_circ_	0.145519	0.368324
hsa_circ_	-0.2124	0.368324
hsa_circ_	0.296095	0.368362
hsa_circ_	0.209631	0.368366
hsa_circ_	0.184701	0.36838
hsa_circ_	-0.17594	0.368396
hsa_circ_	-0.1473	0.36841
hsa_circ_	0.185506	0.368418
hsa_circ_	0.131381	0.368421
hsa_circ_	-0.19974	0.368436
hsa_circ_	-0.37893	0.368438

hsa_circ_	-0.20105	0.368441
hsa_circ_	-0.12064	0.368444
hsa_circ_	-0.14844	0.36845
hsa_circ_	-0.13778	0.368451
hsa_circ_	0.153536	0.368451
hsa_circ_	-0.14422	0.368467
hsa_circ_	-0.37742	0.368478
hsa_circ_	-0.67502	0.368488
hsa_circ_	-0.24424	0.368489
hsa_circ_	0.977945	0.36849
hsa_circ_	0.147713	0.368492
hsa_circ_	-0.80669	0.368522
hsa_circ_	-0.26622	0.368531
hsa_circ_	0.309979	0.368549
hsa_circ_	-0.17631	0.368583
hsa_circ_	-0.14813	0.368584
hsa_circ_	-0.2014	0.368605
hsa_circ_	-0.20993	0.368623
hsa_circ_	0.130823	0.368626
hsa_circ_	0.252417	0.368646
hsa_circ_	0.215299	0.368658
hsa_circ_	0.160082	0.368684
hsa_circ_	-2.06833	0.36871
hsa_circ_	0.2232	0.368719
hsa_circ_	0.233848	0.368725
hsa_circ_	-0.15601	0.368744
hsa_circ_	-0.21116	0.368775
hsa_circ_	-0.44995	0.368781
hsa_circ_	0.136257	0.3688
hsa_circ_	0.24467	0.368812
hsa_circ_	0.171752	0.368829
hsa_circ_	-0.17014	0.368832
hsa_circ_	0.153956	0.368833
hsa_circ_	0.241358	0.368838
hsa_circ_	0.218907	0.368871
hsa_circ_	0.261129	0.368885
hsa_circ_	0.156336	0.368885
hsa_circ_	-0.38304	0.368896
hsa_circ_	-0.36617	0.368918
hsa_circ_	0.260824	0.368941
hsa_circ_	-0.14917	0.36895
hsa_circ_	0.21586	0.36898
hsa_circ_	-0.24658	0.369093
hsa_circ_	0.457945	0.369116
hsa_circ_	-0.22314	0.36912
hsa_circ_	-0.52667	0.36915
hsa_circ_	-0.39991	0.369152
hsa_circ_	-0.16588	0.369154
hsa_circ_	-0.22076	0.369154
hsa_circ_	-0.14227	0.369173
hsa_circ_	-0.34868	0.369182
hsa_circ_	-2.62786	0.369189
hsa_circ_	-0.1869	0.369194
hsa_circ_	-0.14761	0.369199

hsa_circ_	0.125417	0.369207
hsa_circ_	-0.24997	0.369211
hsa_circ_	-0.19442	0.369219
hsa_circ_	-0.59349	0.369226
hsa_circ_	-0.14246	0.369232
hsa_circ_	-0.12789	0.369238
hsa_circ_	-0.21571	0.36925
hsa_circ_	-0.20788	0.369274
hsa_circ_	-0.1632	0.369275
hsa_circ_	-0.16022	0.369288
hsa_circ_	-0.11735	0.369288
hsa_circ_	0.198991	0.369297
hsa_circ_	-0.38008	0.369299
hsa_circ_	-0.13847	0.369312
hsa_circ_	-0.46377	0.369318
hsa_circ_	-0.19344	0.369326
hsa_circ_	0.263502	0.369329
hsa_circ_	-0.13603	0.36933
hsa_circ_	0.167661	0.369333
hsa_circ_	-0.13558	0.369334
hsa_circ_	0.288661	0.369346
hsa_circ_	0.399197	0.369351
hsa_circ_	-0.14282	0.369358
hsa_circ_	0.842252	0.369371
hsa_circ_	0.174017	0.369419
hsa_circ_	-0.26812	0.369431
hsa_circ_	0.171875	0.369445
hsa_circ_	0.246131	0.369447
hsa_circ_	-0.17113	0.369451
hsa_circ_	0.273776	0.369458
hsa_circ_	0.311754	0.369464
hsa_circ_	0.168305	0.369474
hsa_circ_	0.159739	0.369477
hsa_circ_	-0.19655	0.369496
hsa_circ_	-0.16074	0.369528
hsa_circ_	0.204193	0.369536
hsa_circ_	0.167764	0.369549
hsa_circ_	-0.30218	0.369551
hsa_circ_	0.329958	0.369554
hsa_circ_	-0.1856	0.369556
hsa_circ_	0.557285	0.36957
hsa_circ_	0.188423	0.36958
hsa_circ_	-0.18005	0.369582
hsa_circ_	0.184479	0.369587
hsa_circ_	-0.14532	0.369588
hsa_circ_	-0.14077	0.369592
hsa_circ_	-0.44093	0.369599
hsa_circ_	0.188406	0.369616
hsa_circ_	0.180664	0.369618
hsa_circ_	0.464232	0.369622
hsa_circ_	-0.20033	0.369656
hsa_circ_	-0.16647	0.369659
hsa_circ_	-0.16372	0.369661
hsa_circ_	0.320893	0.36967

hsa_circ_	-0.13554	0.369686
hsa_circ_	0.123417	0.369691
hsa_circ_	0.161223	0.369703
hsa_circ_	-0.21241	0.369716
hsa_circ_	-0.2125	0.369753
hsa_circ_	-0.15776	0.36976
hsa_circ_	-0.55707	0.369785
hsa_circ_	0.158821	0.369789
hsa_circ_	-0.24216	0.369794
hsa_circ_	-0.23552	0.369796
hsa_circ_	0.391765	0.369809
hsa_circ_	0.361765	0.369825
hsa_circ_	0.171505	0.36983
hsa_circ_	0.224325	0.369831
hsa_circ_	-0.18429	0.369853
hsa_circ_	0.295053	0.369854
hsa_circ_	-0.18192	0.36986
hsa_circ_	-0.18031	0.369869
hsa_circ_	-0.13458	0.369876
hsa_circ_	-0.14844	0.369878
hsa_circ_	-0.31045	0.369891
hsa_circ_	0.180111	0.369906
hsa_circ_	-0.57104	0.369916
hsa_circ_	0.24421	0.369916
hsa_circ_	-0.15838	0.369957
hsa_circ_	-0.28957	0.369959
hsa_circ_	-0.22423	0.370012
hsa_circ_	-0.23432	0.370017
hsa_circ_	-0.13047	0.370018
hsa_circ_	0.248776	0.370031
hsa_circ_	0.259314	0.370045
hsa_circ_	0.240813	0.370046
hsa_circ_	-0.17085	0.37007
hsa_circ_	-0.19072	0.370074
hsa_circ_	0.193407	0.370099
hsa_circ_	-0.16177	0.370106
hsa_circ_	0.209212	0.37012
hsa_circ_	0.221994	0.370123
hsa_circ_	-0.16029	0.370153
hsa_circ_	0.274787	0.370166
hsa_circ_	-0.12355	0.370187
hsa_circ_	-0.28837	0.370199
hsa_circ_	0.151903	0.370219
hsa_circ_	-0.14047	0.370221
hsa_circ_	0.204864	0.370226
hsa_circ_	0.15643	0.370237
hsa_circ_	0.576983	0.370239
hsa_circ_	0.218322	0.370241
hsa_circ_	-0.19754	0.370244
hsa_circ_	0.126959	0.370258
hsa_circ_	0.176082	0.370259
hsa_circ_	0.188178	0.37026
hsa_circ_	0.150013	0.370267
hsa_circ_	0.183418	0.370273

hsa_circ_	-0.20473	0.370285
hsa_circ_	-0.14484	0.370291
hsa_circ_	-0.54188	0.370316
hsa_circ_	-0.19589	0.370331
hsa_circ_	0.645737	0.370332
hsa_circ_	-0.13626	0.370354
hsa_circ_	0.258328	0.370366
hsa_circ_	0.311149	0.370366
hsa_circ_	-0.32035	0.370374
hsa_circ_	-0.17374	0.370382
hsa_circ_	-0.16722	0.370389
hsa_circ_	-0.17721	0.370395
hsa_circ_	-0.30554	0.370403
hsa_circ_	-0.32623	0.370444
hsa_circ_	0.276724	0.370458
hsa_circ_	-0.16403	0.370474
hsa_circ_	0.406434	0.370484
hsa_circ_	-0.20049	0.370486
hsa_circ_	0.24107	0.370522
hsa_circ_	-0.13289	0.370553
hsa_circ_	0.131735	0.37058
hsa_circ_	-0.25388	0.370584
hsa_circ_	0.673149	0.370587
hsa_circ_	0.273345	0.370598
hsa_circ_	-0.27353	0.370643
hsa_circ_	-0.46835	0.370674
hsa_circ_	0.166733	0.370679
hsa_circ_	-0.23414	0.370681
hsa_circ_	-0.6579	0.37069
hsa_circ_	-0.36116	0.370694
hsa_circ_	0.432549	0.370706
hsa_circ_	-0.16997	0.370711
hsa_circ_	-0.22046	0.370719
hsa_circ_	-0.18957	0.370722
hsa_circ_	-0.19689	0.370726
hsa_circ_	-0.76543	0.370758
hsa_circ_	0.119817	0.37076
hsa_circ_	-0.35363	0.370773
hsa_circ_	0.171946	0.370775
hsa_circ_	-0.1978	0.370793
hsa_circ_	0.317812	0.3708
hsa_circ_	-0.16405	0.370826
hsa_circ_	-0.1744	0.37083
hsa_circ_	-0.46583	0.370831
hsa_circ_	0.706416	0.370853
hsa_circ_	-0.15586	0.370855
hsa_circ_	0.141967	0.370867
hsa_circ_	-0.18515	0.370876
hsa_circ_	0.18238	0.370883
hsa_circ_	-0.13393	0.370897
hsa_circ_	0.1772	0.370901
hsa_circ_	0.13284	0.370903
hsa_circ_	-0.24109	0.370903
hsa_circ_	-0.12994	0.370907

hsa_circ_	0.206207	0.370921
hsa_circ_	-0.30118	0.370924
hsa_circ_	-0.18103	0.370929
hsa_circ_	-0.24258	0.370944
hsa_circ_	0.180274	0.370965
hsa_circ_	0.214689	0.371007
hsa_circ_	0.200939	0.371021
hsa_circ_	0.168171	0.371046
hsa_circ_	0.121491	0.371057
hsa_circ_	-0.12918	0.371062
hsa_circ_	-0.53945	0.371078
hsa_circ_	-0.17511	0.371101
hsa_circ_	-0.3177	0.371103
hsa_circ_	-0.24241	0.371109
hsa_circ_	-0.3492	0.371112
hsa_circ_	0.162389	0.371114
hsa_circ_	-0.15302	0.371128
hsa_circ_	-0.16445	0.371129
hsa_circ_	0.395219	0.371139
hsa_circ_	-0.41684	0.371142
hsa_circ_	-0.22346	0.371144
hsa_circ_	-0.20333	0.371153
hsa_circ_	0.154983	0.371156
hsa_circ_	-0.35736	0.371172
hsa_circ_	0.179225	0.371176
hsa_circ_	0.283213	0.371178
hsa_circ_	0.139625	0.371186
hsa_circ_	-0.19316	0.371194
hsa_circ_	0.58695	0.371196
hsa_circ_	-0.25905	0.371199
hsa_circ_	-0.21062	0.371199
hsa_circ_	-0.15382	0.371205
hsa_circ_	-0.17887	0.371219
hsa_circ_	-0.1872	0.371229
hsa_circ_	-0.1483	0.371244
hsa_circ_	0.255532	0.371268
hsa_circ_	-0.49763	0.371273
hsa_circ_	-0.25106	0.371281
hsa_circ_	0.284863	0.371281
hsa_circ_	-0.19784	0.371292
hsa_circ_	-0.20406	0.371303
hsa_circ_	0.304999	0.371341
hsa_circ_	0.300151	0.37135
hsa_circ_	-0.1698	0.371352
hsa_circ_	-0.37324	0.371357
hsa_circ_	0.155434	0.371363
hsa_circ_	0.28557	0.371372
hsa_circ_	0.180977	0.371374
hsa_circ_	0.20578	0.371376
hsa_circ_	0.143912	0.371381
hsa_circ_	0.222711	0.371397
hsa_circ_	-0.24417	0.371402
hsa_circ_	0.193218	0.371414
hsa_circ_	0.167634	0.371425

hsa_circ_	-0.27102	0.371436
hsa_circ_	0.213548	0.371438
hsa_circ_	-0.17817	0.371449
hsa_circ_	-0.43217	0.371457
hsa_circ_	0.190882	0.371474
hsa_circ_	0.228348	0.371491
hsa_circ_	-0.45242	0.371506
hsa_circ_	0.195827	0.37151
hsa_circ_	0.177197	0.371522
hsa_circ_	0.181742	0.371526
hsa_circ_	0.176823	0.371526
hsa_circ_	0.203756	0.371526
hsa_circ_	0.212094	0.371526
hsa_circ_	-0.88896	0.371539
hsa_circ_	0.243697	0.371542
hsa_circ_	-0.13912	0.371579
hsa_circ_	-0.17471	0.371582
hsa_circ_	0.184298	0.371606
hsa_circ_	-0.21231	0.371614
hsa_circ_	0.159324	0.371617
hsa_circ_	0.573494	0.37162
hsa_circ_	-0.45668	0.371637
hsa_circ_	-0.22325	0.371638
hsa_circ_	0.335097	0.371642
hsa_circ_	-0.16014	0.371649
hsa_circ_	-0.11991	0.371655
hsa_circ_	-0.13715	0.371692
hsa_circ_	0.192416	0.371699
hsa_circ_	0.257079	0.371719
hsa_circ_	-0.1882	0.371725
hsa_circ_	-0.18326	0.371749
hsa_circ_	-0.13286	0.37176
hsa_circ_	-0.5442	0.37177
hsa_circ_	0.31756	0.371798
hsa_circ_	0.216196	0.371809
hsa_circ_	-0.15571	0.371812
hsa_circ_	0.130792	0.371822
hsa_circ_	-0.16374	0.371834
hsa_circ_	-0.17321	0.371862
hsa_circ_	0.183536	0.371876
hsa_circ_	-0.23122	0.371891
hsa_circ_	-0.22421	0.371894
hsa_circ_	-0.15814	0.37193
hsa_circ_	0.268651	0.371931
hsa_circ_	-0.16278	0.371958
hsa_circ_	0.34468	0.371977
hsa_circ_	0.258425	0.372022
hsa_circ_	-0.25595	0.372038
hsa_circ_	-0.1374	0.372039
hsa_circ_	0.210215	0.372061
hsa_circ_	-0.18613	0.372064
hsa_circ_	-0.12593	0.372075
hsa_circ_	-0.26154	0.372076
hsa_circ_	-0.14434	0.372085

hsa_circ_	0.23203	0.372124
hsa_circ_	0.150759	0.372151
hsa_circ_	0.226167	0.372159
hsa_circ_	0.207401	0.372212
hsa_circ_	0.268962	0.372219
hsa_circ_	0.280665	0.37222
hsa_circ_	0.146399	0.372225
hsa_circ_	0.254957	0.372227
hsa_circ_	-0.15263	0.372237
hsa_circ_	-0.50897	0.372239
hsa_circ_	-0.3583	0.372255
hsa_circ_	-0.32961	0.372267
hsa_circ_	0.152419	0.372286
hsa_circ_	0.529467	0.372328
hsa_circ_	0.154757	0.372334
hsa_circ_	-0.26522	0.372359
hsa_circ_	0.145217	0.37239
hsa_circ_	0.192372	0.372414
hsa_circ_	-0.22852	0.372419
hsa_circ_	0.161072	0.372463
hsa_circ_	0.191708	0.372469
hsa_circ_	-0.12782	0.372502
hsa_circ_	-0.14167	0.37251
hsa_circ_	0.562183	0.372568
hsa_circ_	0.322682	0.372576
hsa_circ_	0.18241	0.37259
hsa_circ_	0.497402	0.372639
hsa_circ_	-0.46569	0.372652
hsa_circ_	-0.16453	0.372658
hsa_circ_	-0.36925	0.372676
hsa_circ_	-0.26858	0.372677
hsa_circ_	0.241313	0.372687
hsa_circ_	0.224826	0.372693
hsa_circ_	0.666262	0.372705
hsa_circ_	-0.32684	0.372721
hsa_circ_	0.239125	0.372739
hsa_circ_	0.216689	0.372771
hsa_circ_	-0.15552	0.372778
hsa_circ_	-0.16928	0.372779
hsa_circ_	-0.19424	0.372794
hsa_circ_	0.167283	0.372794
hsa_circ_	0.153279	0.3728
hsa_circ_	0.132358	0.372808
hsa_circ_	-0.15791	0.372814
hsa_circ_	-0.52383	0.372839
hsa_circ_	-0.30975	0.372846
hsa_circ_	0.427591	0.37287
hsa_circ_	0.236364	0.372889
hsa_circ_	-0.1713	0.37289
hsa_circ_	-0.15024	0.372904
hsa_circ_	0.215787	0.372922
hsa_circ_	0.11956	0.372934
hsa_circ_	0.131251	0.372953
hsa_circ_	-0.44018	0.372984

hsa_circ_	0.165408	0.373015
hsa_circ_	0.201932	0.373019
hsa_circ_	-0.14515	0.373033
hsa_circ_	-0.24229	0.373065
hsa_circ_	0.324509	0.373078
hsa_circ_	0.134984	0.373078
hsa_circ_	-0.23118	0.373083
hsa_circ_	-0.19707	0.373091
hsa_circ_	0.144464	0.373114
hsa_circ_	0.148221	0.373147
hsa_circ_	-0.47094	0.373173
hsa_circ_	-0.1448	0.373176
hsa_circ_	-0.17628	0.373186
hsa_circ_	-0.13432	0.373187
hsa_circ_	0.139042	0.373192
hsa_circ_	-0.23047	0.373227
hsa_circ_	-0.15939	0.373233
hsa_circ_	-0.30099	0.37324
hsa_circ_	0.164865	0.37325
hsa_circ_	-0.13562	0.373256
hsa_circ_	-0.1802	0.373269
hsa_circ_	-0.19039	0.37328
hsa_circ_	-0.22138	0.373289
hsa_circ_	-0.12785	0.373298
hsa_circ_	0.251165	0.373303
hsa_circ_	0.320818	0.373311
hsa_circ_	0.149679	0.373324
hsa_circ_	-0.12074	0.373329
hsa_circ_	-0.15368	0.373335
hsa_circ_	-0.18611	0.373337
hsa_circ_	0.149998	0.37334
hsa_circ_	-0.15574	0.373347
hsa_circ_	-0.13421	0.373355
hsa_circ_	0.147229	0.373357
hsa_circ_	0.146349	0.373369
hsa_circ_	-0.17426	0.373373
hsa_circ_	0.150455	0.37338
hsa_circ_	0.159789	0.373403
hsa_circ_	-0.233	0.373433
hsa_circ_	0.500091	0.373435
hsa_circ_	-0.5289	0.373445
hsa_circ_	-0.2558	0.373448
hsa_circ_	0.348286	0.373457
hsa_circ_	-0.49161	0.373465
hsa_circ_	0.176739	0.373471
hsa_circ_	-0.43694	0.373474
hsa_circ_	0.202	0.37348
hsa_circ_	0.142605	0.373481
hsa_circ_	0.161059	0.373486
hsa_circ_	-0.28321	0.373491
hsa_circ_	-0.22594	0.373503
hsa_circ_	-0.23118	0.373517
hsa_circ_	-0.12604	0.373535
hsa_circ_	0.180841	0.37354

hsa_circ_	-0.33877	0.373542
hsa_circ_	-0.13764	0.373557
hsa_circ_	-0.48632	0.373574
hsa_circ_	-0.16959	0.373581
hsa_circ_	-0.14096	0.373618
hsa_circ_	-0.13147	0.373626
hsa_circ_	0.188761	0.373655
hsa_circ_	-0.12254	0.373673
hsa_circ_	-0.22433	0.373676
hsa_circ_	0.298635	0.373685
hsa_circ_	-0.14658	0.373691
hsa_circ_	0.260697	0.373693
hsa_circ_	-0.36335	0.373711
hsa_circ_	-0.2191	0.373714
hsa_circ_	0.159666	0.373716
hsa_circ_	0.139735	0.373737
hsa_circ_	-0.13886	0.373748
hsa_circ_	-1.32719	0.373762
hsa_circ_	-0.57289	0.373818
hsa_circ_	-0.30097	0.373823
hsa_circ_	0.240278	0.373826
hsa_circ_	0.153871	0.373857
hsa_circ_	0.142264	0.373879
hsa_circ_	-0.2983	0.3739
hsa_circ_	-0.22274	0.373905
hsa_circ_	0.161715	0.373921
hsa_circ_	-0.41447	0.373924
hsa_circ_	-0.22727	0.373925
hsa_circ_	-0.2294	0.37393
hsa_circ_	0.218571	0.37395
hsa_circ_	-0.13173	0.373959
hsa_circ_	0.483708	0.373968
hsa_circ_	-1.31373	0.373992
hsa_circ_	-0.15823	0.373993
hsa_circ_	-1.03553	0.374009
hsa_circ_	-0.18284	0.374018
hsa_circ_	-0.12007	0.374031
hsa_circ_	0.164145	0.374032
hsa_circ_	0.65272	0.374032
hsa_circ_	0.321148	0.374098
hsa_circ_	-0.18501	0.37411
hsa_circ_	-0.16643	0.374123
hsa_circ_	-0.14235	0.374137
hsa_circ_	-0.39249	0.374147
hsa_circ_	0.176202	0.374162
hsa_circ_	0.144295	0.374181
hsa_circ_	-0.18589	0.374199
hsa_circ_	-0.3279	0.374229
hsa_circ_	-0.13453	0.374244
hsa_circ_	-0.16709	0.374253
hsa_circ_	-0.45157	0.374268
hsa_circ_	-0.36069	0.374287
hsa_circ_	-0.15507	0.374289
hsa_circ_	-0.16482	0.374294

hsa_circ_	-0.30063	0.374299
hsa_circ_	0.118218	0.374306
hsa_circ_	-0.51266	0.374311
hsa_circ_	-0.38802	0.374311
hsa_circ_	0.116782	0.374363
hsa_circ_	-0.17002	0.374398
hsa_circ_	0.270401	0.374414
hsa_circ_	-0.13067	0.37442
hsa_circ_	-0.12919	0.374425
hsa_circ_	0.213974	0.37443
hsa_circ_	-0.18834	0.374433
hsa_circ_	-0.14329	0.374436
hsa_circ_	-0.12935	0.374443
hsa_circ_	0.158202	0.374461
hsa_circ_	-0.40644	0.374483
hsa_circ_	-0.18697	0.374484
hsa_circ_	-0.20275	0.374485
hsa_circ_	0.361212	0.374488
hsa_circ_	0.292243	0.3745
hsa_circ_	-0.15361	0.374501
hsa_circ_	0.193619	0.374506
hsa_circ_	0.135357	0.374507
hsa_circ_	0.164796	0.37451
hsa_circ_	0.120285	0.374527
hsa_circ_	-0.45017	0.374528
hsa_circ_	0.150263	0.374531
hsa_circ_	-0.23275	0.374533
hsa_circ_	0.27457	0.374538
hsa_circ_	0.202278	0.374559
hsa_circ_	0.327228	0.374561
hsa_circ_	0.300231	0.374562
hsa_circ_	0.235874	0.374574
hsa_circ_	0.16195	0.374594
hsa_circ_	-0.30783	0.374602
hsa_circ_	-0.25017	0.374606
hsa_circ_	0.127887	0.374606
hsa_circ_	0.142718	0.374618
hsa_circ_	0.18818	0.374635
hsa_circ_	0.243214	0.374662
hsa_circ_	0.148002	0.374664
hsa_circ_	0.1729	0.374672
hsa_circ_	-0.20497	0.374675
hsa_circ_	-0.22841	0.374709
hsa_circ_	0.168408	0.374711
hsa_circ_	0.399501	0.374712
hsa_circ_	-0.14156	0.374713
hsa_circ_	-0.16752	0.374731
hsa_circ_	-0.27903	0.374742
hsa_circ_	-0.3158	0.374759
hsa_circ_	-0.28768	0.374761
hsa_circ_	-0.15272	0.37477
hsa_circ_	-0.6675	0.374791
hsa_circ_	0.592095	0.374805
hsa_circ_	0.121703	0.374811

hsa_circ_	-0.15663	0.374816
hsa_circ_	0.23646	0.374851
hsa_circ_	0.174135	0.374858
hsa_circ_	0.138579	0.374864
hsa_circ_	-0.18881	0.374867
hsa_circ_	-0.12816	0.374881
hsa_circ_	-0.17274	0.374885
hsa_circ_	0.459584	0.374886
hsa_circ_	0.31496	0.374888
hsa_circ_	0.160941	0.374888
hsa_circ_	0.166754	0.374901
hsa_circ_	0.499992	0.374905
hsa_circ_	-0.38634	0.374917
hsa_circ_	-0.11554	0.374924
hsa_circ_	0.128925	0.374929
hsa_circ_	-0.25844	0.374941
hsa_circ_	0.168232	0.374988
hsa_circ_	0.170672	0.375012
hsa_circ_	0.5804	0.37504
hsa_circ_	-0.12125	0.375059
hsa_circ_	0.198885	0.375108
hsa_circ_	0.470311	0.375121
hsa_circ_	0.300842	0.375131
hsa_circ_	-0.18394	0.375135
hsa_circ_	-0.13845	0.375172
hsa_circ_	-0.15834	0.375176
hsa_circ_	0.416429	0.375199
hsa_circ_	0.277163	0.375205
hsa_circ_	0.192354	0.375227
hsa_circ_	-0.15391	0.375246
hsa_circ_	-0.23377	0.375273
hsa_circ_	0.530081	0.375279
hsa_circ_	0.193846	0.375289
hsa_circ_	-0.19331	0.375293
hsa_circ_	-0.20716	0.375322
hsa_circ_	0.38456	0.375335
hsa_circ_	0.238635	0.375337
hsa_circ_	0.557099	0.375338
hsa_circ_	-0.36857	0.375358
hsa_circ_	-0.18603	0.375363
hsa_circ_	0.468898	0.375376
hsa_circ_	0.192759	0.37541
hsa_circ_	0.266238	0.375412
hsa_circ_	0.570077	0.375468
hsa_circ_	-0.14475	0.375473
hsa_circ_	-0.2927	0.3755
hsa_circ_	-0.15482	0.3755
hsa_circ_	0.301585	0.375508
hsa_circ_	0.196836	0.375509
hsa_circ_	0.128691	0.375512
hsa_circ_	-0.33619	0.37553
hsa_circ_	-0.3821	0.37553
hsa_circ_	-0.13763	0.375538
hsa_circ_	0.175715	0.375585

hsa_circ_	-0.12975	0.375586
hsa_circ_	-0.1617	0.375596
hsa_circ_	0.159243	0.375607
hsa_circ_	-0.16811	0.375608
hsa_circ_	-0.35981	0.375626
hsa_circ_	0.165146	0.375657
hsa_circ_	0.131314	0.375688
hsa_circ_	0.181935	0.3757
hsa_circ_	-0.16285	0.375712
hsa_circ_	-0.37345	0.37572
hsa_circ_	-0.23798	0.375723
hsa_circ_	0.145881	0.375734
hsa_circ_	-0.18398	0.375769
hsa_circ_	0.178277	0.375777
hsa_circ_	-0.17467	0.3758
hsa_circ_	0.163029	0.375805
hsa_circ_	-0.13963	0.375807
hsa_circ_	-0.26997	0.375831
hsa_circ_	0.154753	0.375857
hsa_circ_	-0.49777	0.375878
hsa_circ_	-0.18176	0.375891
hsa_circ_	0.682593	0.375898
hsa_circ_	0.193292	0.375903
hsa_circ_	-0.47543	0.375911
hsa_circ_	0.501153	0.375945
hsa_circ_	-0.12548	0.375948
hsa_circ_	-0.15749	0.375977
hsa_circ_	-0.18069	0.376027
hsa_circ_	-0.16433	0.376055
hsa_circ_	-0.63277	0.376057
hsa_circ_	0.118798	0.376066
hsa_circ_	0.603042	0.376075
hsa_circ_	-0.35197	0.376076
hsa_circ_	-0.26255	0.376103
hsa_circ_	-0.13666	0.376104
hsa_circ_	0.136253	0.376134
hsa_circ_	-0.15772	0.376154
hsa_circ_	0.187629	0.376155
hsa_circ_	0.207146	0.376175
hsa_circ_	0.154063	0.376197
hsa_circ_	-0.31849	0.376208
hsa_circ_	-0.15543	0.376218
hsa_circ_	-0.87799	0.376226
hsa_circ_	-0.16435	0.37624
hsa_circ_	0.241908	0.376245
hsa_circ_	-0.24276	0.376247
hsa_circ_	0.28998	0.376263
hsa_circ_	-0.30375	0.376266
hsa_circ_	-0.21924	0.376276
hsa_circ_	-0.17172	0.376287
hsa_circ_	0.160149	0.376292
hsa_circ_	0.12599	0.376296
hsa_circ_	-0.11402	0.376311
hsa_circ_	0.357061	0.376319

hsa_circ_	0.253257	0.376334
hsa_circ_	-0.25176	0.376337
hsa_circ_	-0.12334	0.376367
hsa_circ_	0.146537	0.376392
hsa_circ_	0.268237	0.376397
hsa_circ_	0.48741	0.376403
hsa_circ_	0.194756	0.376416
hsa_circ_	-0.13692	0.376416
hsa_circ_	-0.12218	0.376436
hsa_circ_	0.398939	0.376442
hsa_circ_	0.174551	0.376444
hsa_circ_	0.124264	0.376454
hsa_circ_	-0.1282	0.376485
hsa_circ_	-0.32369	0.376493
hsa_circ_	0.305836	0.376494
hsa_circ_	0.313014	0.376498
hsa_circ_	-0.15408	0.376518
hsa_circ_	0.223231	0.376554
hsa_circ_	0.447284	0.376557
hsa_circ_	-0.27631	0.376595
hsa_circ_	-0.17937	0.3766
hsa_circ_	0.180564	0.376624
hsa_circ_	0.186723	0.376638
hsa_circ_	-0.12549	0.376677
hsa_circ_	0.410137	0.37668
hsa_circ_	-0.29484	0.376716
hsa_circ_	0.383208	0.376723
hsa_circ_	-0.11643	0.376725
hsa_circ_	-0.13201	0.376733
hsa_circ_	0.252468	0.37675
hsa_circ_	0.502802	0.376753
hsa_circ_	-0.36124	0.376771
hsa_circ_	-0.24462	0.376779
hsa_circ_	0.179429	0.376796
hsa_circ_	0.273975	0.376805
hsa_circ_	0.147363	0.376807
hsa_circ_	0.43952	0.376814
hsa_circ_	-0.27287	0.37682
hsa_circ_	-0.16462	0.376828
hsa_circ_	-0.11791	0.376852
hsa_circ_	-0.20865	0.376871
hsa_circ_	0.179427	0.376871
hsa_circ_	0.256391	0.376887
hsa_circ_	0.18447	0.376901
hsa_circ_	-0.56587	0.376937
hsa_circ_	0.376013	0.376959
hsa_circ_	0.202224	0.376972
hsa_circ_	-0.39982	0.376973
hsa_circ_	0.412291	0.376973
hsa_circ_	-0.20233	0.376975
hsa_circ_	0.311962	0.376987
hsa_circ_	0.24022	0.37699
hsa_circ_	0.165571	0.376996
hsa_circ_	-0.2197	0.37702

hsa_circ_	-0.16254	0.377061
hsa_circ_	0.361196	0.377076
hsa_circ_	0.152147	0.377101
hsa_circ_	-0.12549	0.377105
hsa_circ_	-0.30441	0.377139
hsa_circ_	-0.12951	0.37714
hsa_circ_	-0.32845	0.37714
hsa_circ_	0.135491	0.37716
hsa_circ_	0.221895	0.377181
hsa_circ_	0.20245	0.377181
hsa_circ_	-0.25283	0.377182
hsa_circ_	-0.6975	0.377231
hsa_circ_	0.773874	0.377242
hsa_circ_	-0.5339	0.377262
hsa_circ_	0.165064	0.377266
hsa_circ_	-0.57202	0.377277
hsa_circ_	-0.14404	0.377292
hsa_circ_	0.197559	0.377298
hsa_circ_	0.380105	0.37731
hsa_circ_	-0.42567	0.377312
hsa_circ_	-0.17724	0.377318
hsa_circ_	-0.32692	0.377325
hsa_circ_	0.140337	0.377347
hsa_circ_	0.133155	0.377353
hsa_circ_	-0.31665	0.377363
hsa_circ_	-0.39463	0.377402
hsa_circ_	0.626642	0.377403
hsa_circ_	0.152911	0.377414
hsa_circ_	-0.25972	0.377419
hsa_circ_	-0.15866	0.37745
hsa_circ_	-0.35743	0.377467
hsa_circ_	-0.2186	0.377493
hsa_circ_	-0.42606	0.377506
hsa_circ_	0.198335	0.377531
hsa_circ_	0.299327	0.377547
hsa_circ_	0.271932	0.377553
hsa_circ_	0.241001	0.377582
hsa_circ_	-0.18954	0.377599
hsa_circ_	0.134335	0.37762
hsa_circ_	-0.26522	0.377632
hsa_circ_	-0.13841	0.377645
hsa_circ_	0.328078	0.377657
hsa_circ_	-0.17487	0.37766
hsa_circ_	-0.16953	0.377663
hsa_circ_	-0.22108	0.377666
hsa_circ_	-0.134	0.377668
hsa_circ_	-0.186	0.37768
hsa_circ_	-0.2824	0.377713
hsa_circ_	0.598684	0.377718
hsa_circ_	0.35215	0.377728
hsa_circ_	0.200448	0.377736
hsa_circ_	-0.2685	0.377743
hsa_circ_	-0.20092	0.377766
hsa_circ_	-0.57566	0.37777

hsa_circ_	0.144501	0.377771
hsa_circ_	0.132259	0.377773
hsa_circ_	-0.13582	0.377775
hsa_circ_	0.505138	0.377782
hsa_circ_	-1.21133	0.377787
hsa_circ_	-0.15366	0.377793
hsa_circ_	-0.67979	0.377797
hsa_circ_	0.139946	0.377847
hsa_circ_	0.37904	0.377859
hsa_circ_	0.19453	0.377865
hsa_circ_	-0.25365	0.377893
hsa_circ_	-0.32987	0.377895
hsa_circ_	-0.39427	0.377942
hsa_circ_	-0.17492	0.377962
hsa_circ_	0.147602	0.377971
hsa_circ_	-0.12879	0.377981
hsa_circ_	-0.22274	0.377989
hsa_circ_	-0.55279	0.378002
hsa_circ_	0.244182	0.37801
hsa_circ_	-0.2325	0.378022
hsa_circ_	-0.37733	0.378023
hsa_circ_	0.136057	0.378036
hsa_circ_	0.1493	0.378051
hsa_circ_	-0.12458	0.378114
hsa_circ_	-0.14064	0.378154
hsa_circ_	-0.12364	0.378215
hsa_circ_	-0.26818	0.378233
hsa_circ_	-0.69811	0.378258
hsa_circ_	-0.14445	0.378259
hsa_circ_	-0.31812	0.378274
hsa_circ_	0.122344	0.37829
hsa_circ_	0.420497	0.378291
hsa_circ_	0.181305	0.378309
hsa_circ_	-0.16828	0.378317
hsa_circ_	-0.33935	0.378322
hsa_circ_	-0.38796	0.378325
hsa_circ_	-0.32606	0.378328
hsa_circ_	-0.51914	0.378331
hsa_circ_	-0.92237	0.378337
hsa_circ_	0.165299	0.378342
hsa_circ_	-0.18145	0.378344
hsa_circ_	0.192533	0.378345
hsa_circ_	-0.33506	0.378361
hsa_circ_	-0.31335	0.378404
hsa_circ_	0.159746	0.378428
hsa_circ_	0.136882	0.37845
hsa_circ_	-0.4063	0.378464
hsa_circ_	-0.17721	0.378467
hsa_circ_	0.132485	0.378487
hsa_circ_	0.251222	0.378509
hsa_circ_	-0.20636	0.378511
hsa_circ_	-0.35735	0.378514
hsa_circ_	-0.31359	0.378515
hsa_circ_	-0.91781	0.37852

hsa_circ_	0.145914	0.378541
hsa_circ_	-0.11434	0.37857
hsa_circ_	-0.16335	0.378583
hsa_circ_	0.157077	0.378606
hsa_circ_	-0.24845	0.378618
hsa_circ_	-0.24574	0.378635
hsa_circ_	-0.48618	0.378646
hsa_circ_	0.129777	0.378672
hsa_circ_	0.286907	0.378673
hsa_circ_	0.290113	0.378686
hsa_circ_	-0.12586	0.378697
hsa_circ_	-0.17667	0.378701
hsa_circ_	-0.15495	0.378722
hsa_circ_	-0.16867	0.37873
hsa_circ_	-0.13317	0.378732
hsa_circ_	0.140487	0.378736
hsa_circ_	0.139368	0.378741
hsa_circ_	-0.23916	0.378742
hsa_circ_	0.123785	0.378746
hsa_circ_	0.206236	0.378751
hsa_circ_	0.548631	0.378809
hsa_circ_	-0.24207	0.378816
hsa_circ_	-0.1562	0.378827
hsa_circ_	0.132815	0.378827
hsa_circ_	0.329037	0.378913
hsa_circ_	-0.15245	0.378915
hsa_circ_	0.138818	0.378946
hsa_circ_	-0.42807	0.378965
hsa_circ_	-0.1568	0.378965
hsa_circ_	-0.20304	0.378967
hsa_circ_	-0.12721	0.378967
hsa_circ_	-0.24162	0.378971
hsa_circ_	-0.21881	0.37898
hsa_circ_	0.141916	0.378981
hsa_circ_	0.569848	0.378985
hsa_circ_	-0.19292	0.378986
hsa_circ_	-0.1399	0.378998
hsa_circ_	-0.14965	0.379009
hsa_circ_	-0.26078	0.379016
hsa_circ_	0.264621	0.379021
hsa_circ_	0.394269	0.379032
hsa_circ_	-0.29958	0.379042
hsa_circ_	0.569212	0.379057
hsa_circ_	0.185427	0.379069
hsa_circ_	-0.16638	0.379089
hsa_circ_	0.367685	0.379092
hsa_circ_	-0.78575	0.379107
hsa_circ_	-0.15479	0.379118
hsa_circ_	-0.26781	0.379121
hsa_circ_	0.198325	0.379124
hsa_circ_	-0.17348	0.379141
hsa_circ_	-0.21725	0.379159
hsa_circ_	0.192234	0.379161
hsa_circ_	-0.49616	0.37918

hsa_circ_	-0.48707	0.379181
hsa_circ_	0.121713	0.379184
hsa_circ_	-0.37724	0.379218
hsa_circ_	-0.14779	0.379227
hsa_circ_	-0.50804	0.379236
hsa_circ_	0.200554	0.379241
hsa_circ_	0.142335	0.379279
hsa_circ_	-0.21086	0.379283
hsa_circ_	-0.16712	0.379304
hsa_circ_	-0.16204	0.379318
hsa_circ_	-0.17023	0.379319
hsa_circ_	0.35575	0.379328
hsa_circ_	0.231301	0.379331
hsa_circ_	-0.13441	0.379335
hsa_circ_	0.135733	0.379338
hsa_circ_	0.210938	0.379343
hsa_circ_	-0.33894	0.379347
hsa_circ_	-0.2483	0.379355
hsa_circ_	-0.17789	0.379356
hsa_circ_	-0.30278	0.37937
hsa_circ_	-0.15281	0.379392
hsa_circ_	-0.128	0.379398
hsa_circ_	-0.1425	0.379405
hsa_circ_	-0.19414	0.379422
hsa_circ_	-0.12247	0.379446
hsa_circ_	0.485355	0.379472
hsa_circ_	-0.62523	0.379475
hsa_circ_	-0.17547	0.379486
hsa_circ_	-0.24989	0.379524
hsa_circ_	-0.49034	0.379533
hsa_circ_	0.126536	0.379545
hsa_circ_	-0.30777	0.379548
hsa_circ_	0.318256	0.379569
hsa_circ_	-0.26461	0.379577
hsa_circ_	0.302009	0.379584
hsa_circ_	0.227514	0.379599
hsa_circ_	-0.17359	0.379632
hsa_circ_	0.326496	0.379633
hsa_circ_	-0.51728	0.379641
hsa_circ_	0.141131	0.379647
hsa_circ_	0.14139	0.379648
hsa_circ_	-0.12405	0.379651
hsa_circ_	0.2212	0.379681
hsa_circ_	-0.48128	0.379682
hsa_circ_	0.238307	0.379682
hsa_circ_	-1.11211	0.379686
hsa_circ_	0.232304	0.379695
hsa_circ_	0.15957	0.379695
hsa_circ_	-0.28703	0.379711
hsa_circ_	-0.14288	0.379718
hsa_circ_	-0.13759	0.379719
hsa_circ_	-0.41707	0.37972
hsa_circ_	0.182756	0.379737
hsa_circ_	-0.20488	0.37974

hsa_circ_	-0.14509	0.379791
hsa_circ_	-0.1951	0.379804
hsa_circ_	-0.78211	0.379807
hsa_circ_	-0.12835	0.379807
hsa_circ_	0.12159	0.379841
hsa_circ_	-0.47757	0.379847
hsa_circ_	-0.18531	0.379857
hsa_circ_	-0.1369	0.379877
hsa_circ_	0.512362	0.379882
hsa_circ_	-0.1979	0.379885
hsa_circ_	-0.20137	0.379885
hsa_circ_	-0.12041	0.379909
hsa_circ_	0.210223	0.379912
hsa_circ_	0.172343	0.379916
hsa_circ_	-0.11503	0.37992
hsa_circ_	-0.20625	0.379926
hsa_circ_	-0.1802	0.379942
hsa_circ_	-0.17497	0.379965
hsa_circ_	-0.16496	0.379969
hsa_circ_	0.218362	0.379982
hsa_circ_	-0.38155	0.379983
hsa_circ_	0.126589	0.380011
hsa_circ_	0.154182	0.380018
hsa_circ_	-0.1295	0.380033
hsa_circ_	0.686014	0.380042
hsa_circ_	-0.12226	0.380053
hsa_circ_	-0.12469	0.380061
hsa_circ_	0.262298	0.380065
hsa_circ_	0.291586	0.380085
hsa_circ_	0.135124	0.380098
hsa_circ_	0.18892	0.380107
hsa_circ_	0.557317	0.380134
hsa_circ_	-0.22334	0.380134
hsa_circ_	0.1754	0.380137
hsa_circ_	0.137689	0.380141
hsa_circ_	0.141831	0.380161
hsa_circ_	-1.74007	0.380166
hsa_circ_	0.426263	0.380176
hsa_circ_	0.209904	0.38018
hsa_circ_	-0.27437	0.380198
hsa_circ_	-0.15019	0.380213
hsa_circ_	-0.21496	0.380238
hsa_circ_	0.341017	0.380264
hsa_circ_	0.160744	0.380277
hsa_circ_	-0.13542	0.38028
hsa_circ_	-0.18044	0.380288
hsa_circ_	0.155409	0.380296
hsa_circ_	0.196025	0.380311
hsa_circ_	-0.13466	0.380313
hsa_circ_	-0.11903	0.380329
hsa_circ_	0.614592	0.38033
hsa_circ_	-0.24652	0.380374
hsa_circ_	0.273919	0.380385
hsa_circ_	0.616932	0.380395

hsa_circ_	0.156835	0.3804
hsa_circ_	-0.24837	0.380407
hsa_circ_	-0.17826	0.380424
hsa_circ_	0.219265	0.380428
hsa_circ_	-0.53821	0.380429
hsa_circ_	-0.15658	0.38044
hsa_circ_	-0.7929	0.380461
hsa_circ_	-0.18926	0.380558
hsa_circ_	-0.32163	0.380606
hsa_circ_	0.195642	0.380608
hsa_circ_	0.140437	0.380632
hsa_circ_	0.116898	0.38064
hsa_circ_	-0.53621	0.380642
hsa_circ_	-0.24975	0.380647
hsa_circ_	-0.43208	0.380652
hsa_circ_	0.320885	0.380686
hsa_circ_	0.14277	0.380711
hsa_circ_	-0.20592	0.380715
hsa_circ_	-0.34608	0.380718
hsa_circ_	-0.18828	0.380733
hsa_circ_	-0.15547	0.380775
hsa_circ_	-0.22743	0.380784
hsa_circ_	0.236371	0.380791
hsa_circ_	0.266649	0.380804
hsa_circ_	-0.25896	0.380809
hsa_circ_	0.116159	0.380824
hsa_circ_	-0.40862	0.380833
hsa_circ_	-0.11861	0.38084
hsa_circ_	-0.35211	0.380847
hsa_circ_	-0.30382	0.380879
hsa_circ_	0.53738	0.380918
hsa_circ_	0.148656	0.38092
hsa_circ_	0.158414	0.38093
hsa_circ_	0.202148	0.380944
hsa_circ_	-0.26793	0.380949
hsa_circ_	-0.80864	0.380957
hsa_circ_	0.305232	0.380995
hsa_circ_	-0.12813	0.381021
hsa_circ_	-0.48167	0.38103
hsa_circ_	-0.21754	0.38105
hsa_circ_	-0.15624	0.381064
hsa_circ_	-0.1361	0.381087
hsa_circ_	-0.14199	0.381097
hsa_circ_	-0.44845	0.381117
hsa_circ_	-0.2143	0.381123
hsa_circ_	0.155268	0.381125
hsa_circ_	-0.14874	0.381125
hsa_circ_	-0.19799	0.381126
hsa_circ_	-1.51155	0.38114
hsa_circ_	-0.80012	0.381166
hsa_circ_	-0.1454	0.381203
hsa_circ_	-0.36823	0.381206
hsa_circ_	0.143416	0.381251
hsa_circ_	-0.17755	0.381271

hsa_circ_	0.213632	0.381291
hsa_circ_	0.146156	0.381303
hsa_circ_	-0.21254	0.381309
hsa_circ_	-0.31816	0.38131
hsa_circ_	0.371055	0.381326
hsa_circ_	0.158584	0.381326
hsa_circ_	-0.2276	0.381331
hsa_circ_	0.122985	0.381337
hsa_circ_	-0.13477	0.381343
hsa_circ_	0.224244	0.381352
hsa_circ_	0.13261	0.381362
hsa_circ_	-0.12121	0.381364
hsa_circ_	0.281093	0.381377
hsa_circ_	0.146728	0.381379
hsa_circ_	0.315112	0.381397
hsa_circ_	-0.14173	0.381408
hsa_circ_	0.4125	0.381409
hsa_circ_	-0.55015	0.381445
hsa_circ_	-0.12689	0.381454
hsa_circ_	-0.17783	0.381465
hsa_circ_	-0.16178	0.381509
hsa_circ_	0.217507	0.381518
hsa_circ_	0.562437	0.381522
hsa_circ_	-0.46758	0.381539
hsa_circ_	0.241488	0.381586
hsa_circ_	-0.50363	0.381597
hsa_circ_	-0.14626	0.381614
hsa_circ_	0.254742	0.381625
hsa_circ_	-0.11516	0.381645
hsa_circ_	-0.14343	0.381646
hsa_circ_	0.138383	0.381647
hsa_circ_	-0.45911	0.381647
hsa_circ_	-0.27864	0.381672
hsa_circ_	-0.20323	0.381673
hsa_circ_	-0.276	0.381695
hsa_circ_	-0.12114	0.381702
hsa_circ_	0.731222	0.381706
hsa_circ_	-0.4317	0.381726
hsa_circ_	-0.20185	0.381729
hsa_circ_	0.157034	0.381731
hsa_circ_	0.141046	0.381736
hsa_circ_	-0.81532	0.381763
hsa_circ_	0.301799	0.381771
hsa_circ_	0.194278	0.381791
hsa_circ_	0.165216	0.381801
hsa_circ_	-0.15208	0.381805
hsa_circ_	-0.31971	0.381809
hsa_circ_	0.565457	0.381834
hsa_circ_	0.2989	0.381845
hsa_circ_	-0.1983	0.381861
hsa_circ_	-0.32312	0.381877
hsa_circ_	-0.16039	0.381896
hsa_circ_	-0.15556	0.381917
hsa_circ_	-0.47012	0.381928

hsa_circ_	-0.28243	0.381937
hsa_circ_	0.260279	0.381945
hsa_circ_	-0.50617	0.381954
hsa_circ_	-0.27155	0.381978
hsa_circ_	-0.27688	0.38198
hsa_circ_	0.12296	0.381987
hsa_circ_	-0.23171	0.381993
hsa_circ_	0.289817	0.382004
hsa_circ_	0.223582	0.382019
hsa_circ_	0.143536	0.382022
hsa_circ_	-0.31887	0.382029
hsa_circ_	-0.13848	0.382069
hsa_circ_	0.429168	0.382079
hsa_circ_	-0.16105	0.382093
hsa_circ_	0.3412	0.382098
hsa_circ_	0.581902	0.382106
hsa_circ_	-0.12159	0.382106
hsa_circ_	-0.17409	0.38212
hsa_circ_	-0.14985	0.382139
hsa_circ_	0.149911	0.382145
hsa_circ_	0.155831	0.382155
hsa_circ_	-0.15452	0.38216
hsa_circ_	-0.12924	0.382174
hsa_circ_	0.944083	0.382194
hsa_circ_	0.200908	0.382217
hsa_circ_	0.208951	0.382285
hsa_circ_	-0.1818	0.382286
hsa_circ_	-0.15513	0.38229
hsa_circ_	-0.11853	0.382294
hsa_circ_	-0.14899	0.382315
hsa_circ_	-0.14059	0.382319
hsa_circ_	0.198791	0.38233
hsa_circ_	-0.15879	0.382334
hsa_circ_	-0.22949	0.382345
hsa_circ_	0.268823	0.382366
hsa_circ_	-0.16215	0.382388
hsa_circ_	0.319577	0.382417
hsa_circ_	0.269361	0.382423
hsa_circ_	-0.42123	0.382438
hsa_circ_	0.124995	0.382466
hsa_circ_	-0.24487	0.382475
hsa_circ_	-0.25833	0.382476
hsa_circ_	-0.15008	0.382479
hsa_circ_	-0.15727	0.382486
hsa_circ_	-0.26962	0.382487
hsa_circ_	0.472418	0.382495
hsa_circ_	0.241065	0.382504
hsa_circ_	0.396401	0.382518
hsa_circ_	-0.13383	0.382531
hsa_circ_	-0.18718	0.38255
hsa_circ_	-0.32772	0.382595
hsa_circ_	0.37777	0.382606
hsa_circ_	-0.23885	0.382617
hsa_circ_	-0.16814	0.382617

hsa_circ_	-0.52025	0.382621
hsa_circ_	0.350341	0.382623
hsa_circ_	0.133441	0.382624
hsa_circ_	-0.13773	0.382624
hsa_circ_	0.144019	0.382637
hsa_circ_	-0.13548	0.382658
hsa_circ_	0.446245	0.382692
hsa_circ_	0.13772	0.382693
hsa_circ_	-0.33244	0.382701
hsa_circ_	0.563086	0.382724
hsa_circ_	0.152854	0.382724
hsa_circ_	0.566242	0.382726
hsa_circ_	0.649648	0.382734
hsa_circ_	-0.3383	0.382741
hsa_circ_	0.144436	0.38275
hsa_circ_	-0.42532	0.38276
hsa_circ_	0.336233	0.38276
hsa_circ_	0.248329	0.382769
hsa_circ_	0.302545	0.382775
hsa_circ_	0.148225	0.382783
hsa_circ_	0.385527	0.382839
hsa_circ_	-0.32812	0.382858
hsa_circ_	-0.15102	0.382873
hsa_circ_	0.164548	0.382877
hsa_circ_	-0.75269	0.382879
hsa_circ_	-0.54795	0.382885
hsa_circ_	-0.17432	0.382889
hsa_circ_	-0.34225	0.382909
hsa_circ_	-0.2146	0.38295
hsa_circ_	-0.30516	0.382962
hsa_circ_	-0.26542	0.38298
hsa_circ_	-0.38907	0.383012
hsa_circ_	0.526335	0.383031
hsa_circ_	0.336267	0.383054
hsa_circ_	0.17946	0.383058
hsa_circ_	-0.21702	0.383063
hsa_circ_	-0.11759	0.383065
hsa_circ_	0.180947	0.383071
hsa_circ_	-0.13291	0.383077
hsa_circ_	-0.21557	0.383123
hsa_circ_	0.163775	0.383126
hsa_circ_	-0.25166	0.38313
hsa_circ_	0.167573	0.383156
hsa_circ_	0.413064	0.383171
hsa_circ_	-0.24354	0.383182
hsa_circ_	-1.25039	0.383185
hsa_circ_	0.119724	0.38319
hsa_circ_	0.162625	0.383219
hsa_circ_	-0.34388	0.383226
hsa_circ_	-0.22331	0.383227
hsa_circ_	-0.22888	0.383248
hsa_circ_	0.130789	0.383261
hsa_circ_	0.150968	0.383274
hsa_circ_	0.582805	0.383274

hsa_circ_	0.329298	0.383281
hsa_circ_	0.238679	0.383287
hsa_circ_	-0.23359	0.38329
hsa_circ_	-0.13666	0.383308
hsa_circ_	-0.1475	0.383312
hsa_circ_	-0.16132	0.383321
hsa_circ_	-0.13166	0.383338
hsa_circ_	0.536351	0.383346
hsa_circ_	0.412789	0.383363
hsa_circ_	-0.12886	0.383365
hsa_circ_	0.408502	0.383375
hsa_circ_	0.145499	0.383385
hsa_circ_	-0.14339	0.383406
hsa_circ_	-0.13226	0.383408
hsa_circ_	0.146955	0.383413
hsa_circ_	-0.1665	0.383416
hsa_circ_	-0.31817	0.383417
hsa_circ_	-0.3985	0.383423
hsa_circ_	0.343728	0.383443
hsa_circ_	0.24075	0.383453
hsa_circ_	0.208924	0.383457
hsa_circ_	-0.13805	0.383477
hsa_circ_	-0.18315	0.383494
hsa_circ_	0.714598	0.383504
hsa_circ_	0.235743	0.383509
hsa_circ_	-0.53631	0.383512
hsa_circ_	-0.27679	0.383517
hsa_circ_	-0.19472	0.383536
hsa_circ_	0.203238	0.383543
hsa_circ_	0.18898	0.383556
hsa_circ_	0.19573	0.383577
hsa_circ_	-0.29266	0.383577
hsa_circ_	-0.15376	0.383593
hsa_circ_	0.151377	0.3836
hsa_circ_	-0.11893	0.383611
hsa_circ_	-0.15615	0.383617
hsa_circ_	0.253278	0.383631
hsa_circ_	-0.4423	0.383638
hsa_circ_	0.147716	0.383643
hsa_circ_	0.194991	0.383657
hsa_circ_	-0.17382	0.38366
hsa_circ_	-0.14889	0.383693
hsa_circ_	-0.20153	0.383695
hsa_circ_	0.114384	0.383702
hsa_circ_	-0.28506	0.383714
hsa_circ_	-0.35551	0.383756
hsa_circ_	0.70719	0.383794
hsa_circ_	0.220053	0.3838
hsa_circ_	0.154315	0.38381
hsa_circ_	0.282445	0.383852
hsa_circ_	-0.17062	0.383881
hsa_circ_	-0.121	0.383899
hsa_circ_	0.219061	0.383901
hsa_circ_	-0.13439	0.383905

hsa_circ_	-0.30046	0.38391
hsa_circ_	-0.14296	0.383913
hsa_circ_	-0.20401	0.383925
hsa_circ_	0.155004	0.383955
hsa_circ_	-0.17684	0.383959
hsa_circ_	0.422672	0.383964
hsa_circ_	-0.16801	0.38397
hsa_circ_	-0.16444	0.383976
hsa_circ_	-0.24003	0.383977
hsa_circ_	-0.2738	0.383985
hsa_circ_	-0.12622	0.383987
hsa_circ_	-0.24751	0.384007
hsa_circ_	0.149442	0.384011
hsa_circ_	0.199819	0.384029
hsa_circ_	-0.27025	0.384036
hsa_circ_	-0.36555	0.384044
hsa_circ_	0.413476	0.384047
hsa_circ_	0.423912	0.384048
hsa_circ_	-0.16087	0.384056
hsa_circ_	0.178776	0.384075
hsa_circ_	0.252239	0.384077
hsa_circ_	0.350147	0.384084
hsa_circ_	1.223378	0.38409
hsa_circ_	0.280324	0.384098
hsa_circ_	-0.16753	0.38412
hsa_circ_	-0.28246	0.384123
hsa_circ_	-0.19025	0.384142
hsa_circ_	-0.12717	0.384144
hsa_circ_	0.142586	0.384156
hsa_circ_	-0.31285	0.384161
hsa_circ_	0.257329	0.384164
hsa_circ_	-0.15585	0.384172
hsa_circ_	0.128971	0.384183
hsa_circ_	-0.302	0.384184
hsa_circ_	0.214058	0.384191
hsa_circ_	-0.48991	0.384215
hsa_circ_	0.180191	0.384245
hsa_circ_	-0.2357	0.384259
hsa_circ_	-0.13578	0.384288
hsa_circ_	0.161213	0.384297
hsa_circ_	-0.21887	0.384309
hsa_circ_	-0.1981	0.384334
hsa_circ_	-0.20123	0.384334
hsa_circ_	-0.13085	0.384352
hsa_circ_	0.227537	0.384353
hsa_circ_	-0.164	0.384357
hsa_circ_	0.184303	0.38438
hsa_circ_	-0.24269	0.384384
hsa_circ_	-0.57714	0.384402
hsa_circ_	-0.22093	0.384403
hsa_circ_	-0.16295	0.384418
hsa_circ_	-1.87487	0.38442
hsa_circ_	0.199333	0.384424
hsa_circ_	-0.1794	0.38445

hsa_circ_	0.142889	0.384471
hsa_circ_	-0.14358	0.384494
hsa_circ_	-0.134	0.384512
hsa_circ_	0.220843	0.384516
hsa_circ_	-0.4113	0.384522
hsa_circ_	0.160608	0.384539
hsa_circ_	-0.22793	0.384565
hsa_circ_	0.22164	0.384573
hsa_circ_	-0.16496	0.384624
hsa_circ_	-0.21241	0.384624
hsa_circ_	0.432347	0.384635
hsa_circ_	-0.33721	0.384639
hsa_circ_	0.239837	0.384639
hsa_circ_	0.446001	0.384643
hsa_circ_	0.140509	0.384654
hsa_circ_	-0.167	0.384668
hsa_circ_	-0.11665	0.384693
hsa_circ_	0.545052	0.384725
hsa_circ_	-0.55205	0.384756
hsa_circ_	0.436118	0.384771
hsa_circ_	-0.25006	0.384775
hsa_circ_	-0.21969	0.384779
hsa_circ_	0.424711	0.384784
hsa_circ_	-1.01633	0.384797
hsa_circ_	0.257079	0.384834
hsa_circ_	-0.11516	0.384843
hsa_circ_	-0.15331	0.384853
hsa_circ_	0.146976	0.384854
hsa_circ_	0.143819	0.384897
hsa_circ_	0.135709	0.384902
hsa_circ_	-0.18333	0.384903
hsa_circ_	0.201453	0.384907
hsa_circ_	-0.17679	0.384911
hsa_circ_	0.147051	0.384949
hsa_circ_	0.18865	0.384956
hsa_circ_	-0.1286	0.384956
hsa_circ_	-0.18388	0.384981
hsa_circ_	0.489425	0.385014
hsa_circ_	-0.16348	0.385018
hsa_circ_	0.152182	0.385025
hsa_circ_	-0.25053	0.385031
hsa_circ_	-0.12561	0.385035
hsa_circ_	-0.14792	0.385036
hsa_circ_	-0.35782	0.385041
hsa_circ_	0.184925	0.385055
hsa_circ_	-0.13773	0.385072
hsa_circ_	0.244858	0.385083
hsa_circ_	-0.15122	0.385092
hsa_circ_	-0.3759	0.385114
hsa_circ_	-0.34654	0.385142
hsa_circ_	-0.12817	0.385163
hsa_circ_	0.13069	0.385166
hsa_circ_	-0.77676	0.385172
hsa_circ_	-0.18637	0.385178

hsa_circ_	0.369929	0.385196
hsa_circ_	0.24241	0.385206
hsa_circ_	-0.13381	0.385208
hsa_circ_	-0.30738	0.385223
hsa_circ_	-0.27004	0.385232
hsa_circ_	-0.54576	0.385235
hsa_circ_	-0.54658	0.385253
hsa_circ_	0.447374	0.385253
hsa_circ_	0.211257	0.385266
hsa_circ_	-0.25273	0.38528
hsa_circ_	-0.12578	0.385292
hsa_circ_	-0.34739	0.385301
hsa_circ_	-0.1892	0.385316
hsa_circ_	0.161029	0.385332
hsa_circ_	0.663799	0.385347
hsa_circ_	-0.24945	0.385347
hsa_circ_	0.188245	0.385349
hsa_circ_	0.236235	0.385359
hsa_circ_	0.422532	0.385367
hsa_circ_	0.952147	0.385394
hsa_circ_	0.244754	0.385442
hsa_circ_	0.18358	0.385453
hsa_circ_	0.254421	0.385453
hsa_circ_	-0.17294	0.385466
hsa_circ_	0.266992	0.385516
hsa_circ_	-0.59645	0.385519
hsa_circ_	-0.14417	0.385523
hsa_circ_	0.499	0.385543
hsa_circ_	-0.22815	0.385577
hsa_circ_	0.168329	0.385586
hsa_circ_	-0.31639	0.385616
hsa_circ_	-0.31574	0.385616
hsa_circ_	-0.15193	0.385625
hsa_circ_	-0.446	0.385645
hsa_circ_	-0.18147	0.38565
hsa_circ_	-0.36244	0.385651
hsa_circ_	0.171881	0.385667
hsa_circ_	-0.16849	0.38568
hsa_circ_	-0.30437	0.385695
hsa_circ_	0.242784	0.385697
hsa_circ_	-0.11715	0.385699
hsa_circ_	-0.14097	0.385704
hsa_circ_	0.140345	0.385705
hsa_circ_	-0.20469	0.385731
hsa_circ_	0.146784	0.385749
hsa_circ_	-0.22081	0.385797
hsa_circ_	-0.1388	0.385797
hsa_circ_	0.206441	0.385815
hsa_circ_	-0.24612	0.385818
hsa_circ_	-0.16369	0.385843
hsa_circ_	-0.15034	0.385847
hsa_circ_	-0.24866	0.385848
hsa_circ_	0.596894	0.385857
hsa_circ_	-0.11813	0.385862

hsa_circ_	0.153867	0.38587
hsa_circ_	-0.30369	0.385886
hsa_circ_	-0.16235	0.385887
hsa_circ_	0.164758	0.385887
hsa_circ_	-0.23649	0.385902
hsa_circ_	0.647187	0.385918
hsa_circ_	0.155593	0.385924
hsa_circ_	-0.12816	0.38594
hsa_circ_	-0.53158	0.385955
hsa_circ_	-0.18994	0.385956
hsa_circ_	-0.12545	0.385965
hsa_circ_	-0.11617	0.385965
hsa_circ_	0.366755	0.386005
hsa_circ_	-0.17701	0.386006
hsa_circ_	0.216715	0.386019
hsa_circ_	-0.19542	0.386043
hsa_circ_	-0.45107	0.386047
hsa_circ_	0.172346	0.386079
hsa_circ_	0.397489	0.386108
hsa_circ_	0.350078	0.386138
hsa_circ_	-0.13434	0.386139
hsa_circ_	-0.30965	0.386185
hsa_circ_	0.119108	0.386191
hsa_circ_	-0.2315	0.386193
hsa_circ_	-0.54592	0.386208
hsa_circ_	0.193415	0.386234
hsa_circ_	-0.17861	0.386268
hsa_circ_	0.363628	0.386296
hsa_circ_	-0.48919	0.386312
hsa_circ_	-0.28212	0.386326
hsa_circ_	-0.16304	0.386333
hsa_circ_	0.139308	0.386344
hsa_circ_	0.525655	0.386347
hsa_circ_	0.383626	0.386352
hsa_circ_	-0.11741	0.386361
hsa_circ_	-0.15799	0.386367
hsa_circ_	-0.2411	0.386374
hsa_circ_	0.180945	0.386406
hsa_circ_	0.129973	0.386408
hsa_circ_	-0.2194	0.38641
hsa_circ_	-0.24154	0.386414
hsa_circ_	0.192521	0.386431
hsa_circ_	-0.28448	0.386431
hsa_circ_	0.175743	0.386437
hsa_circ_	-0.5851	0.38644
hsa_circ_	-0.38358	0.386473
hsa_circ_	0.141455	0.386479
hsa_circ_	0.139462	0.386501
hsa_circ_	0.327251	0.386507
hsa_circ_	-0.14299	0.38651
hsa_circ_	0.169858	0.386513
hsa_circ_	-0.15119	0.386536
hsa_circ_	0.325888	0.386545
hsa_circ_	-0.22811	0.386555

hsa_circ_	0.319191	0.386577
hsa_circ_	0.158999	0.386589
hsa_circ_	-0.43192	0.386594
hsa_circ_	0.139308	0.386604
hsa_circ_	0.128622	0.386618
hsa_circ_	0.174136	0.386639
hsa_circ_	0.205602	0.386647
hsa_circ_	0.16234	0.386654
hsa_circ_	-0.46023	0.386657
hsa_circ_	-0.49301	0.386674
hsa_circ_	-0.1165	0.386689
hsa_circ_	-0.13255	0.386691
hsa_circ_	0.306456	0.386715
hsa_circ_	0.122587	0.386729
hsa_circ_	0.155471	0.386744
hsa_circ_	-0.49198	0.386751
hsa_circ_	-0.23066	0.3868
hsa_circ_	-0.2328	0.386804
hsa_circ_	0.303093	0.386828
hsa_circ_	-0.12856	0.386828
hsa_circ_	0.452265	0.386832
hsa_circ_	-0.22957	0.386841
hsa_circ_	-0.21565	0.386844
hsa_circ_	-0.15446	0.386864
hsa_circ_	0.180367	0.386867
hsa_circ_	0.142031	0.386874
hsa_circ_	0.263244	0.386885
hsa_circ_	-0.39259	0.386888
hsa_circ_	-0.3088	0.386892
hsa_circ_	-0.4286	0.386898
hsa_circ_	-0.13236	0.386914
hsa_circ_	0.452307	0.386929
hsa_circ_	0.181822	0.386964
hsa_circ_	0.132964	0.386967
hsa_circ_	0.557495	0.386987
hsa_circ_	-0.14387	0.386995
hsa_circ_	0.189784	0.387002
hsa_circ_	0.139929	0.387004
hsa_circ_	0.160445	0.387037
hsa_circ_	-0.15053	0.387055
hsa_circ_	0.31394	0.387118
hsa_circ_	0.12862	0.38712
hsa_circ_	-0.21835	0.387129
hsa_circ_	-0.34284	0.387167
hsa_circ_	-0.19317	0.387171
hsa_circ_	0.472902	0.387178
hsa_circ_	0.390196	0.38721
hsa_circ_	0.236058	0.387212
hsa_circ_	0.160344	0.387231
hsa_circ_	-0.1725	0.387241
hsa_circ_	0.178851	0.38725
hsa_circ_	0.521364	0.387251
hsa_circ_	-0.16025	0.387257
hsa_circ_	0.160771	0.387259

hsa_circ_	-0.17786	0.387277
hsa_circ_	-0.17768	0.38729
hsa_circ_	0.15308	0.387291
hsa_circ_	-1.79182	0.387299
hsa_circ_	0.220875	0.38731
hsa_circ_	0.203349	0.387319
hsa_circ_	-0.28854	0.38736
hsa_circ_	-0.16607	0.387361
hsa_circ_	0.170529	0.387365
hsa_circ_	0.149328	0.387382
hsa_circ_	0.246112	0.387391
hsa_circ_	-0.33385	0.387394
hsa_circ_	0.153153	0.387414
hsa_circ_	-0.35682	0.38745
hsa_circ_	0.129215	0.387451
hsa_circ_	0.115178	0.387453
hsa_circ_	0.213575	0.387483
hsa_circ_	0.262662	0.387484
hsa_circ_	0.164461	0.387491
hsa_circ_	0.200408	0.3875
hsa_circ_	-0.11171	0.387512
hsa_circ_	0.288008	0.387512
hsa_circ_	-0.22821	0.387538
hsa_circ_	0.18075	0.387543
hsa_circ_	-0.23095	0.387565
hsa_circ_	0.201153	0.387571
hsa_circ_	0.18615	0.387584
hsa_circ_	-0.16673	0.387585
hsa_circ_	0.267173	0.387596
hsa_circ_	0.190753	0.387623
hsa_circ_	-0.24394	0.387641
hsa_circ_	0.217574	0.387642
hsa_circ_	-0.17994	0.387649
hsa_circ_	0.641656	0.387672
hsa_circ_	-0.68806	0.387737
hsa_circ_	0.52481	0.387758
hsa_circ_	0.170245	0.38776
hsa_circ_	-0.21512	0.387772
hsa_circ_	0.202057	0.387774
hsa_circ_	0.19393	0.38779
hsa_circ_	-0.14886	0.387792
hsa_circ_	0.118168	0.387793
hsa_circ_	-0.17644	0.387802
hsa_circ_	0.396226	0.387813
hsa_circ_	-0.31185	0.387816
hsa_circ_	-0.14961	0.387823
hsa_circ_	-0.1429	0.387835
hsa_circ_	0.140864	0.387849
hsa_circ_	-0.16128	0.387868
hsa_circ_	-0.27287	0.387874
hsa_circ_	-0.37375	0.387911
hsa_circ_	0.151063	0.387913
hsa_circ_	0.153422	0.387937
hsa_circ_	-0.5904	0.387947

hsa_circ_	0.365696	0.387952
hsa_circ_	0.187904	0.387971
hsa_circ_	-0.15552	0.387984
hsa_circ_	0.576681	0.388011
hsa_circ_	0.207413	0.388038
hsa_circ_	-0.27117	0.388058
hsa_circ_	-0.28102	0.388061
hsa_circ_	0.198533	0.388066
hsa_circ_	0.162629	0.388071
hsa_circ_	-0.23128	0.388076
hsa_circ_	0.194755	0.38809
hsa_circ_	-0.15806	0.388094
hsa_circ_	0.172665	0.388102
hsa_circ_	-0.69715	0.388105
hsa_circ_	-0.1551	0.388118
hsa_circ_	0.24503	0.388123
hsa_circ_	-0.15539	0.388124
hsa_circ_	-0.15067	0.388133
hsa_circ_	0.168456	0.388136
hsa_circ_	0.176869	0.388159
hsa_circ_	0.157462	0.388173
hsa_circ_	-0.11667	0.388178
hsa_circ_	0.227272	0.388187
hsa_circ_	-0.29079	0.38819
hsa_circ_	-0.19447	0.388204
hsa_circ_	0.136218	0.388212
hsa_circ_	-0.17902	0.388238
hsa_circ_	-0.14741	0.388247
hsa_circ_	-0.29261	0.388267
hsa_circ_	0.115701	0.388297
hsa_circ_	-0.17048	0.388317
hsa_circ_	0.558287	0.388322
hsa_circ_	0.265394	0.388341
hsa_circ_	-0.14142	0.388342
hsa_circ_	-0.34487	0.388344
hsa_circ_	-0.20449	0.388372
hsa_circ_	0.134323	0.38839
hsa_circ_	-0.51185	0.388406
hsa_circ_	-0.17893	0.388408
hsa_circ_	1.070933	0.38841
hsa_circ_	-0.16808	0.388411
hsa_circ_	0.405963	0.388431
hsa_circ_	-0.16667	0.388432
hsa_circ_	0.128544	0.388432
hsa_circ_	-0.12001	0.388443
hsa_circ_	-0.18078	0.38846
hsa_circ_	0.222723	0.388484
hsa_circ_	-0.42965	0.388487
hsa_circ_	0.202135	0.388492
hsa_circ_	-0.14709	0.388493
hsa_circ_	-0.14886	0.388505
hsa_circ_	-0.48617	0.38851
hsa_circ_	0.121747	0.388512
hsa_circ_	-0.20286	0.388533

hsa_circ_	-0.1927	0.388563
hsa_circ_	-0.67238	0.388569
hsa_circ_	-0.15341	0.388571
hsa_circ_	0.126772	0.388572
hsa_circ_	-0.13676	0.388585
hsa_circ_	-0.15456	0.388585
hsa_circ_	-0.40346	0.388598
hsa_circ_	0.209708	0.3886
hsa_circ_	0.282012	0.388602
hsa_circ_	0.123058	0.388606
hsa_circ_	-0.13964	0.388622
hsa_circ_	0.35266	0.388623
hsa_circ_	-0.14911	0.388634
hsa_circ_	0.132986	0.388649
hsa_circ_	-0.25791	0.388666
hsa_circ_	0.238754	0.388673
hsa_circ_	0.157914	0.388678
hsa_circ_	-0.40989	0.388684
hsa_circ_	-0.13073	0.38869
hsa_circ_	-0.12806	0.388691
hsa_circ_	0.116796	0.388696
hsa_circ_	-0.23242	0.388697
hsa_circ_	-0.13433	0.388764
hsa_circ_	0.117499	0.388771
hsa_circ_	-0.12706	0.388774
hsa_circ_	-0.13223	0.388774
hsa_circ_	-0.21975	0.38878
hsa_circ_	-0.13959	0.38878
hsa_circ_	0.12505	0.388785
hsa_circ_	-0.15966	0.388792
hsa_circ_	-0.47972	0.388826
hsa_circ_	0.126612	0.388857
hsa_circ_	-0.37314	0.388886
hsa_circ_	0.666704	0.388898
hsa_circ_	-0.18403	0.388899
hsa_circ_	0.555831	0.388908
hsa_circ_	0.398639	0.388922
hsa_circ_	-0.13441	0.388928
hsa_circ_	-0.14954	0.388929
hsa_circ_	-0.15186	0.388943
hsa_circ_	-0.28423	0.388949
hsa_circ_	0.178919	0.388952
hsa_circ_	-0.30006	0.388957
hsa_circ_	0.321148	0.388965
hsa_circ_	0.450347	0.388995
hsa_circ_	0.136552	0.388997
hsa_circ_	0.562442	0.389015
hsa_circ_	0.244	0.389016
hsa_circ_	0.236474	0.38903
hsa_circ_	0.195918	0.389031
hsa_circ_	-0.30682	0.389046
hsa_circ_	0.319958	0.389062
hsa_circ_	-0.29019	0.389063
hsa_circ_	-0.58144	0.389063

hsa_circ_	-0.29203	0.389082
hsa_circ_	-0.26305	0.389085
hsa_circ_	-0.1177	0.389088
hsa_circ_	0.259301	0.389097
hsa_circ_	0.229191	0.389123
hsa_circ_	-0.95047	0.389142
hsa_circ_	-0.13353	0.389149
hsa_circ_	0.181425	0.389167
hsa_circ_	-0.33672	0.389204
hsa_circ_	-0.13604	0.389221
hsa_circ_	0.123968	0.389253
hsa_circ_	-0.14907	0.389292
hsa_circ_	0.262824	0.389324
hsa_circ_	0.237934	0.389332
hsa_circ_	0.14996	0.389377
hsa_circ_	-0.12098	0.389398
hsa_circ_	-0.16535	0.389418
hsa_circ_	-0.13239	0.389431
hsa_circ_	-0.22589	0.389447
hsa_circ_	0.418939	0.389473
hsa_circ_	0.157978	0.389481
hsa_circ_	0.193401	0.389487
hsa_circ_	-0.19909	0.389499
hsa_circ_	0.279772	0.38951
hsa_circ_	-0.61869	0.389511
hsa_circ_	0.153092	0.389555
hsa_circ_	-0.13265	0.389557
hsa_circ_	-0.19925	0.389558
hsa_circ_	0.479529	0.389564
hsa_circ_	-0.3096	0.389586
hsa_circ_	-0.21965	0.389588
hsa_circ_	0.117783	0.389591
hsa_circ_	-0.31414	0.389611
hsa_circ_	-0.12048	0.389618
hsa_circ_	-0.13951	0.389652
hsa_circ_	0.159273	0.389657
hsa_circ_	-0.1361	0.389661
hsa_circ_	-0.58041	0.389679
hsa_circ_	-0.23372	0.389683
hsa_circ_	-0.37296	0.3897
hsa_circ_	-0.34986	0.389714
hsa_circ_	-0.13648	0.389728
hsa_circ_	0.417215	0.389773
hsa_circ_	-0.47561	0.389778
hsa_circ_	-0.15761	0.389782
hsa_circ_	-0.15218	0.38983
hsa_circ_	-0.15305	0.389832
hsa_circ_	0.24039	0.389833
hsa_circ_	-0.15065	0.389852
hsa_circ_	0.544413	0.389886
hsa_circ_	-0.16327	0.389887
hsa_circ_	0.445632	0.389889
hsa_circ_	-0.13541	0.389889
hsa_circ_	-0.2684	0.389897

hsa_circ_	-0.24828	0.389941
hsa_circ_	-0.2089	0.389949
hsa_circ_	-0.43912	0.389956
hsa_circ_	-0.15655	0.389975
hsa_circ_	-0.23675	0.38998
hsa_circ_	0.523335	0.389992
hsa_circ_	0.20629	0.390008
hsa_circ_	-0.12625	0.390016
hsa_circ_	0.169125	0.390019
hsa_circ_	-0.13875	0.390027
hsa_circ_	0.157475	0.390035
hsa_circ_	-0.13558	0.390036
hsa_circ_	0.462157	0.390046
hsa_circ_	-0.61803	0.390057
hsa_circ_	0.207304	0.390062
hsa_circ_	0.172905	0.390097
hsa_circ_	-0.21736	0.390113
hsa_circ_	0.603957	0.390114
hsa_circ_	0.315475	0.390115
hsa_circ_	-0.12552	0.390127
hsa_circ_	0.165485	0.39014
hsa_circ_	-0.35262	0.390161
hsa_circ_	0.185554	0.390164
hsa_circ_	0.233309	0.390181
hsa_circ_	0.146155	0.390189
hsa_circ_	-0.12501	0.390195
hsa_circ_	-0.13879	0.390211
hsa_circ_	-0.19465	0.390224
hsa_circ_	-0.13835	0.390238
hsa_circ_	0.20186	0.390242
hsa_circ_	0.235471	0.390279
hsa_circ_	-0.13052	0.390315
hsa_circ_	0.284358	0.390317
hsa_circ_	0.116392	0.390339
hsa_circ_	-0.2677	0.390349
hsa_circ_	0.182146	0.390376
hsa_circ_	-0.31053	0.39039
hsa_circ_	-0.26369	0.390392
hsa_circ_	0.315687	0.390422
hsa_circ_	-0.1207	0.390431
hsa_circ_	0.287913	0.390437
hsa_circ_	-0.14135	0.390449
hsa_circ_	0.212897	0.390458
hsa_circ_	0.423238	0.390468
hsa_circ_	-0.20121	0.390471
hsa_circ_	-0.99767	0.390487
hsa_circ_	0.154199	0.390494
hsa_circ_	-0.19808	0.390499
hsa_circ_	-0.14925	0.390508
hsa_circ_	0.156421	0.390524
hsa_circ_	0.131819	0.390525
hsa_circ_	0.176952	0.39055
hsa_circ_	-0.19518	0.390559
hsa_circ_	0.131757	0.390569

hsa_circ_	0.684568	0.390592
hsa_circ_	-0.14622	0.390597
hsa_circ_	0.176088	0.3906
hsa_circ_	0.234827	0.390611
hsa_circ_	-0.13104	0.390613
hsa_circ_	0.195658	0.39066
hsa_circ_	0.194523	0.390671
hsa_circ_	-0.13292	0.390677
hsa_circ_	0.311243	0.390679
hsa_circ_	-0.26942	0.390692
hsa_circ_	-0.19948	0.390693
hsa_circ_	0.298454	0.390726
hsa_circ_	-0.2228	0.390737
hsa_circ_	-0.16579	0.390801
hsa_circ_	-0.70396	0.390812
hsa_circ_	-0.28259	0.390813
hsa_circ_	-0.50564	0.390816
hsa_circ_	-0.12435	0.390819
hsa_circ_	0.179303	0.390839
hsa_circ_	0.188105	0.390841
hsa_circ_	-0.22035	0.390853
hsa_circ_	0.128182	0.390853
hsa_circ_	0.407608	0.390864
hsa_circ_	0.145185	0.390865
hsa_circ_	-0.19467	0.390897
hsa_circ_	-0.1321	0.390912
hsa_circ_	0.178904	0.390922
hsa_circ_	0.527589	0.390922
hsa_circ_	-0.23764	0.390936
hsa_circ_	-0.20937	0.390944
hsa_circ_	-0.26666	0.390967
hsa_circ_	0.145536	0.390984
hsa_circ_	-0.1627	0.391003
hsa_circ_	-0.19874	0.391007
hsa_circ_	-0.14191	0.391027
hsa_circ_	-0.1912	0.391061
hsa_circ_	-0.13484	0.391094
hsa_circ_	-0.1442	0.391098
hsa_circ_	-0.1922	0.391145
hsa_circ_	0.150205	0.391148
hsa_circ_	0.41985	0.391191
hsa_circ_	-0.21821	0.391198
hsa_circ_	0.205567	0.391218
hsa_circ_	-0.14364	0.391238
hsa_circ_	-0.37998	0.391264
hsa_circ_	-0.15871	0.391271
hsa_circ_	-0.12621	0.391303
hsa_circ_	0.266092	0.391312
hsa_circ_	0.655976	0.39133
hsa_circ_	-0.28835	0.391339
hsa_circ_	0.197804	0.391346
hsa_circ_	0.181694	0.391356
hsa_circ_	0.162885	0.391374
hsa_circ_	0.153603	0.391376

hsa_circ_	0.769668	0.39139
hsa_circ_	-0.12826	0.391401
hsa_circ_	0.169103	0.391403
hsa_circ_	-0.13502	0.391431
hsa_circ_	-0.1686	0.391443
hsa_circ_	-0.16991	0.391456
hsa_circ_	-0.25716	0.391464
hsa_circ_	-0.16046	0.391467
hsa_circ_	-0.16843	0.391473
hsa_circ_	0.638111	0.391484
hsa_circ_	0.483716	0.391487
hsa_circ_	0.193014	0.391495
hsa_circ_	-0.1294	0.391518
hsa_circ_	-0.11635	0.391546
hsa_circ_	-0.62751	0.391551
hsa_circ_	-0.16441	0.391553
hsa_circ_	-0.20918	0.391571
hsa_circ_	-0.14104	0.391576
hsa_circ_	0.154136	0.391584
hsa_circ_	0.248746	0.391587
hsa_circ_	0.153914	0.391598
hsa_circ_	0.229401	0.391609
hsa_circ_	0.531879	0.391624
hsa_circ_	-0.88671	0.391633
hsa_circ_	-0.17194	0.391658
hsa_circ_	0.484749	0.391662
hsa_circ_	0.219535	0.391665
hsa_circ_	-0.4942	0.39167
hsa_circ_	0.253918	0.391693
hsa_circ_	0.189303	0.391699
hsa_circ_	-0.56423	0.391704
hsa_circ_	-0.22854	0.391707
hsa_circ_	0.185558	0.391711
hsa_circ_	0.255302	0.391734
hsa_circ_	0.153231	0.391747
hsa_circ_	-0.21165	0.391763
hsa_circ_	0.363046	0.391764
hsa_circ_	-0.14878	0.391784
hsa_circ_	-0.12086	0.391789
hsa_circ_	-0.17856	0.391789
hsa_circ_	0.312044	0.391801
hsa_circ_	0.163412	0.391802
hsa_circ_	0.374611	0.391825
hsa_circ_	-0.1585	0.391865
hsa_circ_	0.121734	0.391893
hsa_circ_	0.168184	0.3919
hsa_circ_	-0.2076	0.391903
hsa_circ_	-0.14909	0.391906
hsa_circ_	-0.18272	0.39191
hsa_circ_	0.12298	0.39191
hsa_circ_	0.353284	0.391914
hsa_circ_	0.2923	0.391928
hsa_circ_	0.254792	0.39194
hsa_circ_	-0.1734	0.391941

hsa_circ_	0.205423	0.391969
hsa_circ_	-0.39018	0.391982
hsa_circ_	0.155646	0.391987
hsa_circ_	-0.55168	0.391987
hsa_circ_	-0.26123	0.391996
hsa_circ_	0.11852	0.391999
hsa_circ_	-0.26675	0.392012
hsa_circ_	-0.24735	0.392034
hsa_circ_	0.168512	0.392034
hsa_circ_	-0.11455	0.392044
hsa_circ_	-0.23726	0.39209
hsa_circ_	-0.13741	0.392098
hsa_circ_	0.265041	0.392103
hsa_circ_	-0.14232	0.392127
hsa_circ_	-0.12106	0.392157
hsa_circ_	0.234653	0.392178
hsa_circ_	-0.23354	0.392189
hsa_circ_	0.175682	0.392189
hsa_circ_	-0.2323	0.392198
hsa_circ_	0.165517	0.392205
hsa_circ_	-0.53951	0.392213
hsa_circ_	0.312688	0.392218
hsa_circ_	-0.16244	0.392219
hsa_circ_	-0.21761	0.39224
hsa_circ_	-0.16814	0.392243
hsa_circ_	0.140358	0.392243
hsa_circ_	0.184121	0.392246
hsa_circ_	0.121963	0.392269
hsa_circ_	-0.19835	0.392277
hsa_circ_	0.181972	0.392282
hsa_circ_	0.137345	0.392283
hsa_circ_	-0.14064	0.392311
hsa_circ_	-0.13418	0.392336
hsa_circ_	-0.18753	0.392357
hsa_circ_	-0.18196	0.392362
hsa_circ_	-0.21488	0.392399
hsa_circ_	-0.19217	0.3924
hsa_circ_	0.156224	0.39243
hsa_circ_	-0.13741	0.392457
hsa_circ_	0.364864	0.392463
hsa_circ_	-0.25382	0.392465
hsa_circ_	0.296415	0.392478
hsa_circ_	0.139084	0.392484
hsa_circ_	0.193896	0.39249
hsa_circ_	0.118638	0.392491
hsa_circ_	0.138444	0.392493
hsa_circ_	-0.11567	0.392515
hsa_circ_	0.156072	0.392543
hsa_circ_	-0.20171	0.392576
hsa_circ_	0.243393	0.392602
hsa_circ_	-0.30385	0.392618
hsa_circ_	-0.14288	0.392618
hsa_circ_	-0.2665	0.392623
hsa_circ_	0.146684	0.392624

hsa_circ_	-0.13083	0.392627
hsa_circ_	-0.19975	0.392637
hsa_circ_	-0.15318	0.392643
hsa_circ_	0.177058	0.392647
hsa_circ_	-0.2237	0.392649
hsa_circ_	-0.11404	0.392656
hsa_circ_	-0.19222	0.39266
hsa_circ_	0.250278	0.392666
hsa_circ_	-0.14216	0.392666
hsa_circ_	-0.1247	0.392688
hsa_circ_	0.113505	0.392704
hsa_circ_	-0.41464	0.392726
hsa_circ_	-0.40322	0.392726
hsa_circ_	0.365781	0.392768
hsa_circ_	-0.26385	0.392793
hsa_circ_	-0.26881	0.3928
hsa_circ_	0.279094	0.392842
hsa_circ_	-0.34174	0.392849
hsa_circ_	-0.3458	0.392861
hsa_circ_	0.186872	0.392864
hsa_circ_	-0.1217	0.392884
hsa_circ_	-0.13899	0.392891
hsa_circ_	0.12735	0.392901
hsa_circ_	-0.14021	0.392908
hsa_circ_	0.31438	0.39292
hsa_circ_	0.17853	0.39292
hsa_circ_	-0.11639	0.392934
hsa_circ_	-0.1945	0.392942
hsa_circ_	0.12147	0.392951
hsa_circ_	-0.22164	0.392956
hsa_circ_	-0.26709	0.392956
hsa_circ_	-0.2952	0.392972
hsa_circ_	0.314689	0.392981
hsa_circ_	0.235039	0.393054
hsa_circ_	-0.17654	0.393074
hsa_circ_	0.519941	0.393123
hsa_circ_	0.641597	0.393135
hsa_circ_	0.231609	0.393136
hsa_circ_	0.231986	0.393168
hsa_circ_	-0.38856	0.393198
hsa_circ_	0.524537	0.393199
hsa_circ_	-0.65028	0.393207
hsa_circ_	0.152303	0.393208
hsa_circ_	0.418486	0.393214
hsa_circ_	-0.25611	0.393257
hsa_circ_	-0.24254	0.393328
hsa_circ_	0.116741	0.39333
hsa_circ_	0.137832	0.393334
hsa_circ_	0.199729	0.393334
hsa_circ_	-0.25463	0.393349
hsa_circ_	-0.343	0.393362
hsa_circ_	-0.27377	0.393365
hsa_circ_	-0.25171	0.393369
hsa_circ_	0.447584	0.393378

hsa_circ_	0.36846	0.393381
hsa_circ_	0.294285	0.3934
hsa_circ_	-0.2865	0.393404
hsa_circ_	-0.22116	0.393411
hsa_circ_	-0.20666	0.393413
hsa_circ_	0.231066	0.393413
hsa_circ_	-0.13715	0.393418
hsa_circ_	-0.15548	0.393431
hsa_circ_	-0.20063	0.39346
hsa_circ_	0.124162	0.393465
hsa_circ_	-0.12258	0.393467
hsa_circ_	-1.00925	0.393497
hsa_circ_	0.181872	0.393499
hsa_circ_	-0.14409	0.393507
hsa_circ_	-0.28313	0.393527
hsa_circ_	-0.20755	0.393539
hsa_circ_	0.249831	0.393549
hsa_circ_	-0.15747	0.39356
hsa_circ_	-0.13945	0.393564
hsa_circ_	0.438149	0.393577
hsa_circ_	-0.27734	0.393588
hsa_circ_	-0.44391	0.393597
hsa_circ_	-0.19498	0.393603
hsa_circ_	0.623704	0.393625
hsa_circ_	-0.21618	0.393648
hsa_circ_	0.147526	0.393674
hsa_circ_	0.133491	0.393675
hsa_circ_	0.143359	0.393689
hsa_circ_	0.204252	0.393728
hsa_circ_	-0.2657	0.393764
hsa_circ_	-0.19875	0.393766
hsa_circ_	-0.19135	0.393767
hsa_circ_	0.319956	0.393808
hsa_circ_	0.18643	0.393816
hsa_circ_	0.146839	0.393824
hsa_circ_	0.135921	0.393825
hsa_circ_	-0.13151	0.393852
hsa_circ_	-0.12092	0.393856
hsa_circ_	-0.12523	0.393868
hsa_circ_	-0.33275	0.393875
hsa_circ_	-0.30031	0.393885
hsa_circ_	-0.27267	0.393894
hsa_circ_	-0.23186	0.393921
hsa_circ_	0.154132	0.393927
hsa_circ_	-0.12229	0.39393
hsa_circ_	-0.12773	0.39395
hsa_circ_	-1.50872	0.393962
hsa_circ_	-0.30564	0.393964
hsa_circ_	0.225043	0.393976
hsa_circ_	-0.25471	0.393979
hsa_circ_	0.217236	0.394038
hsa_circ_	-0.23102	0.394045
hsa_circ_	-0.17989	0.394046
hsa_circ_	-0.23058	0.394066

hsa_circ_	0.187106	0.394107
hsa_circ_	0.182568	0.394124
hsa_circ_	-0.19802	0.394124
hsa_circ_	0.166312	0.394133
hsa_circ_	-0.15234	0.394142
hsa_circ_	-0.2364	0.394148
hsa_circ_	0.707105	0.394163
hsa_circ_	-0.13162	0.39418
hsa_circ_	0.150059	0.394181
hsa_circ_	0.326404	0.394203
hsa_circ_	0.142327	0.394203
hsa_circ_	-0.10886	0.394204
hsa_circ_	0.187836	0.394211
hsa_circ_	0.17009	0.394214
hsa_circ_	0.468961	0.394216
hsa_circ_	-0.9573	0.394276
hsa_circ_	-0.12789	0.394303
hsa_circ_	0.131852	0.394309
hsa_circ_	0.566161	0.394314
hsa_circ_	0.180083	0.394328
hsa_circ_	-0.6415	0.394332
hsa_circ_	-0.11952	0.394333
hsa_circ_	-0.44925	0.394336
hsa_circ_	-0.58687	0.394351
hsa_circ_	0.171666	0.394352
hsa_circ_	0.247302	0.394371
hsa_circ_	-0.32641	0.394376
hsa_circ_	-0.67153	0.394387
hsa_circ_	0.14757	0.394392
hsa_circ_	0.141179	0.394397
hsa_circ_	0.616576	0.3944
hsa_circ_	-0.1807	0.394417
hsa_circ_	-0.17628	0.394427
hsa_circ_	0.21756	0.394431
hsa_circ_	-0.2507	0.39444
hsa_circ_	0.157426	0.394451
hsa_circ_	-0.28299	0.394456
hsa_circ_	0.159229	0.394486
hsa_circ_	0.209807	0.394504
hsa_circ_	0.126147	0.394507
hsa_circ_	-0.28591	0.394507
hsa_circ_	-0.41828	0.394527
hsa_circ_	-0.2417	0.394537
hsa_circ_	0.896861	0.394547
hsa_circ_	0.192105	0.394552
hsa_circ_	-0.13263	0.394561
hsa_circ_	0.188083	0.394567
hsa_circ_	0.163999	0.39457
hsa_circ_	-0.23977	0.394571
hsa_circ_	-0.4894	0.394579
hsa_circ_	-0.22478	0.394586
hsa_circ_	0.688507	0.394607
hsa_circ_	0.405579	0.394626
hsa_circ_	-0.20901	0.394628

hsa_circ_	-0.11646	0.394633
hsa_circ_	0.195297	0.394676
hsa_circ_	-0.14402	0.394689
hsa_circ_	-0.13883	0.394691
hsa_circ_	-0.14103	0.394725
hsa_circ_	-0.16993	0.394761
hsa_circ_	-0.40632	0.394777
hsa_circ_	-0.16226	0.394781
hsa_circ_	-0.12467	0.394784
hsa_circ_	-0.19027	0.394784
hsa_circ_	-0.15958	0.394786
hsa_circ_	-0.14047	0.394792
hsa_circ_	-0.15897	0.394794
hsa_circ_	-0.14432	0.394802
hsa_circ_	-0.13045	0.394815
hsa_circ_	-0.62022	0.394817
hsa_circ_	-0.33363	0.39482
hsa_circ_	-0.18281	0.394852
hsa_circ_	-0.17123	0.394882
hsa_circ_	0.430159	0.394911
hsa_circ_	0.242054	0.394918
hsa_circ_	-0.19796	0.39493
hsa_circ_	0.195994	0.394957
hsa_circ_	-0.16136	0.394961
hsa_circ_	0.213573	0.394974
hsa_circ_	0.120118	0.394987
hsa_circ_	0.12161	0.394994
hsa_circ_	0.128963	0.394994
hsa_circ_	-0.15588	0.394995
hsa_circ_	-0.21592	0.39501
hsa_circ_	0.170109	0.39504
hsa_circ_	0.189217	0.395046
hsa_circ_	-0.13085	0.395053
hsa_circ_	0.114278	0.395056
hsa_circ_	0.131544	0.395096
hsa_circ_	-0.19196	0.395118
hsa_circ_	-0.11721	0.395126
hsa_circ_	0.218634	0.395138
hsa_circ_	0.332893	0.395139
hsa_circ_	0.241279	0.39514
hsa_circ_	-0.31287	0.39514
hsa_circ_	-0.14859	0.395153
hsa_circ_	-0.25669	0.395171
hsa_circ_	0.153333	0.395182
hsa_circ_	-0.12621	0.395187
hsa_circ_	0.117061	0.395196
hsa_circ_	0.250875	0.395205
hsa_circ_	0.202584	0.395223
hsa_circ_	-0.12324	0.395264
hsa_circ_	0.194492	0.395264
hsa_circ_	-0.22552	0.395265
hsa_circ_	0.15948	0.395269
hsa_circ_	0.22363	0.395283
hsa_circ_	-0.24474	0.395293

hsa_circ_	0.156712	0.395306
hsa_circ_	-0.14189	0.395326
hsa_circ_	-0.17394	0.395335
hsa_circ_	0.150171	0.395335
hsa_circ_	-0.16885	0.395343
hsa_circ_	-0.56581	0.39536
hsa_circ_	0.141517	0.39537
hsa_circ_	0.150162	0.395375
hsa_circ_	0.160452	0.395388
hsa_circ_	0.392743	0.395397
hsa_circ_	-0.15401	0.395398
hsa_circ_	0.248314	0.395408
hsa_circ_	-0.2362	0.395413
hsa_circ_	-0.20447	0.39542
hsa_circ_	0.265363	0.395423
hsa_circ_	-0.26034	0.395434
hsa_circ_	-0.45687	0.395447
hsa_circ_	-0.26604	0.395454
hsa_circ_	0.373564	0.395454
hsa_circ_	-0.11112	0.395458
hsa_circ_	0.118598	0.395458
hsa_circ_	-0.12446	0.395502
hsa_circ_	0.122273	0.395505
hsa_circ_	-0.17013	0.395546
hsa_circ_	-0.2459	0.395553
hsa_circ_	0.332986	0.395554
hsa_circ_	-0.274	0.395555
hsa_circ_	-0.15415	0.395559
hsa_circ_	-0.16689	0.395573
hsa_circ_	-0.13245	0.395583
hsa_circ_	-0.45376	0.395583
hsa_circ_	-0.20256	0.395585
hsa_circ_	-0.16869	0.39559
hsa_circ_	-0.1224	0.395602
hsa_circ_	-0.13481	0.395604
hsa_circ_	0.160896	0.395606
hsa_circ_	-0.50939	0.395607
hsa_circ_	0.184609	0.395609
hsa_circ_	-0.3073	0.395624
hsa_circ_	-0.19281	0.395648
hsa_circ_	-0.19325	0.395673
hsa_circ_	-0.13753	0.395698
hsa_circ_	0.334426	0.395713
hsa_circ_	0.239774	0.395716
hsa_circ_	0.277517	0.395736
hsa_circ_	0.157828	0.395739
hsa_circ_	0.13958	0.395795
hsa_circ_	-0.14478	0.395822
hsa_circ_	-0.37445	0.395824
hsa_circ_	0.307532	0.395833
hsa_circ_	0.167044	0.395851
hsa_circ_	-0.17859	0.395853
hsa_circ_	-0.15295	0.395861
hsa_circ_	-0.31731	0.395865

hsa_circ_	0.171968	0.395866
hsa_circ_	0.208373	0.395872
hsa_circ_	0.266354	0.395873
hsa_circ_	-0.17561	0.395883
hsa_circ_	-0.18777	0.395885
hsa_circ_	0.642622	0.395896
hsa_circ_	-0.12947	0.395898
hsa_circ_	-0.13466	0.3959
hsa_circ_	-0.25555	0.395906
hsa_circ_	0.15016	0.39592
hsa_circ_	-0.51599	0.39592
hsa_circ_	0.171456	0.395925
hsa_circ_	0.166837	0.395928
hsa_circ_	-0.11847	0.39594
hsa_circ_	0.198481	0.395942
hsa_circ_	0.140141	0.395968
hsa_circ_	0.121148	0.395979
hsa_circ_	0.255861	0.39601
hsa_circ_	-0.34204	0.396016
hsa_circ_	0.162856	0.396055
hsa_circ_	-0.41345	0.396057
hsa_circ_	0.192111	0.396058
hsa_circ_	0.180671	0.396059
hsa_circ_	0.179339	0.396089
hsa_circ_	0.218417	0.396096
hsa_circ_	0.171196	0.396099
hsa_circ_	0.179991	0.396114
hsa_circ_	-0.24053	0.396123
hsa_circ_	-0.12549	0.396161
hsa_circ_	0.205502	0.396178
hsa_circ_	-0.14046	0.396178
hsa_circ_	0.615097	0.396181
hsa_circ_	-0.11713	0.396183
hsa_circ_	0.120403	0.396196
hsa_circ_	0.149183	0.396223
hsa_circ_	-0.45422	0.396227
hsa_circ_	-0.34562	0.396227
hsa_circ_	0.643882	0.396233
hsa_circ_	-0.49241	0.396242
hsa_circ_	0.171176	0.396248
hsa_circ_	-0.22084	0.396251
hsa_circ_	0.127482	0.396262
hsa_circ_	-0.23891	0.396276
hsa_circ_	0.276356	0.396288
hsa_circ_	0.142839	0.396288
hsa_circ_	0.22196	0.39629
hsa_circ_	-0.31522	0.396292
hsa_circ_	-0.11143	0.396293
hsa_circ_	0.360507	0.396316
hsa_circ_	-0.115	0.396322
hsa_circ_	-0.13621	0.396323
hsa_circ_	-0.11663	0.396333
hsa_circ_	-0.19683	0.396342
hsa_circ_	0.25402	0.396343

hsa_circ_	-0.14057	0.396349
hsa_circ_	0.243192	0.396358
hsa_circ_	-0.21447	0.396371
hsa_circ_	-0.20679	0.396373
hsa_circ_	0.154855	0.396397
hsa_circ_	-0.19539	0.396476
hsa_circ_	0.176933	0.396493
hsa_circ_	0.645437	0.396515
hsa_circ_	0.491903	0.396524
hsa_circ_	-0.30713	0.396556
hsa_circ_	-0.24386	0.396568
hsa_circ_	-0.11767	0.396608
hsa_circ_	0.199408	0.396609
hsa_circ_	0.259105	0.396627
hsa_circ_	-0.20995	0.396641
hsa_circ_	-0.15258	0.396653
hsa_circ_	0.228346	0.396655
hsa_circ_	0.128174	0.396656
hsa_circ_	-0.22298	0.396681
hsa_circ_	0.142566	0.396689
hsa_circ_	-0.11364	0.396691
hsa_circ_	0.241367	0.396692
hsa_circ_	-0.25775	0.396709
hsa_circ_	0.221742	0.396709
hsa_circ_	-0.34952	0.39672
hsa_circ_	-0.12079	0.396731
hsa_circ_	-0.13781	0.396741
hsa_circ_	0.487334	0.396777
hsa_circ_	-0.23854	0.396787
hsa_circ_	-0.16918	0.396814
hsa_circ_	0.160044	0.396831
hsa_circ_	-0.17021	0.396841
hsa_circ_	0.121791	0.396854
hsa_circ_	0.295382	0.396858
hsa_circ_	-0.12265	0.396863
hsa_circ_	-0.13301	0.396876
hsa_circ_	0.169317	0.396878
hsa_circ_	0.168143	0.396884
hsa_circ_	0.566907	0.396885
hsa_circ_	0.143124	0.396896
hsa_circ_	0.223752	0.396927
hsa_circ_	0.335436	0.39693
hsa_circ_	-0.14218	0.396931
hsa_circ_	-0.12463	0.39694
hsa_circ_	-0.17691	0.396942
hsa_circ_	-0.1269	0.396958
hsa_circ_	-0.26104	0.396991
hsa_circ_	0.314089	0.397021
hsa_circ_	-0.19272	0.397028
hsa_circ_	-0.2113	0.397038
hsa_circ_	0.211238	0.397038
hsa_circ_	-0.18205	0.397043
hsa_circ_	0.377007	0.397053
hsa_circ_	0.155219	0.397063

hsa_circ_	0.139639	0.397065
hsa_circ_	0.350546	0.397099
hsa_circ_	0.142428	0.397103
hsa_circ_	-0.1746	0.397113
hsa_circ_	-0.14291	0.397167
hsa_circ_	-0.42701	0.397175
hsa_circ_	-0.54402	0.397187
hsa_circ_	0.140858	0.397217
hsa_circ_	0.283191	0.397226
hsa_circ_	0.290934	0.397235
hsa_circ_	0.196291	0.397237
hsa_circ_	-0.17029	0.397256
hsa_circ_	-0.12428	0.397258
hsa_circ_	0.169988	0.397283
hsa_circ_	-0.15113	0.397302
hsa_circ_	0.186678	0.397304
hsa_circ_	-0.20165	0.397325
hsa_circ_	-0.26334	0.397342
hsa_circ_	-0.14211	0.397357
hsa_circ_	-0.42818	0.397363
hsa_circ_	-0.26398	0.397376
hsa_circ_	-0.1192	0.397385
hsa_circ_	-0.30318	0.397405
hsa_circ_	-0.20701	0.397417
hsa_circ_	-0.18851	0.397462
hsa_circ_	0.301893	0.397498
hsa_circ_	0.168886	0.397509
hsa_circ_	-0.91584	0.397523
hsa_circ_	0.133101	0.397576
hsa_circ_	0.523821	0.397576
hsa_circ_	-0.15476	0.397588
hsa_circ_	-0.31958	0.397593
hsa_circ_	0.24452	0.397604
hsa_circ_	0.151555	0.397612
hsa_circ_	0.413263	0.397618
hsa_circ_	-0.15488	0.397629
hsa_circ_	0.118043	0.39764
hsa_circ_	-0.37082	0.397645
hsa_circ_	-0.16527	0.39765
hsa_circ_	-0.25404	0.39766
hsa_circ_	-0.25861	0.397698
hsa_circ_	0.283868	0.397704
hsa_circ_	0.14923	0.397707
hsa_circ_	0.861254	0.397709
hsa_circ_	-0.38123	0.397717
hsa_circ_	-0.20484	0.39772
hsa_circ_	-0.20358	0.397721
hsa_circ_	0.563566	0.397728
hsa_circ_	-0.40029	0.397734
hsa_circ_	0.137645	0.39774
hsa_circ_	0.116424	0.397771
hsa_circ_	0.226277	0.397788
hsa_circ_	0.111869	0.397791
hsa_circ_	0.145459	0.397793

hsa_circ_	-0.60572	0.3978
hsa_circ_	0.140219	0.397833
hsa_circ_	0.558436	0.397837
hsa_circ_	0.162868	0.397882
hsa_circ_	-0.19301	0.397883
hsa_circ_	-1.39026	0.397894
hsa_circ_	0.170612	0.397901
hsa_circ_	-0.3831	0.397932
hsa_circ_	-0.87009	0.397959
hsa_circ_	-0.12687	0.397962
hsa_circ_	-0.67136	0.39797
hsa_circ_	0.141503	0.397972
hsa_circ_	-0.17541	0.397976
hsa_circ_	0.56681	0.397995
hsa_circ_	0.134241	0.39802
hsa_circ_	-0.15115	0.398027
hsa_circ_	0.153288	0.398032
hsa_circ_	-0.3602	0.398035
hsa_circ_	-0.21461	0.398061
hsa_circ_	-0.13074	0.398067
hsa_circ_	0.321241	0.398082
hsa_circ_	-0.13767	0.398086
hsa_circ_	-0.11866	0.398104
hsa_circ_	0.239994	0.398113
hsa_circ_	-0.26008	0.398197
hsa_circ_	-0.12163	0.398197
hsa_circ_	-0.57228	0.398208
hsa_circ_	0.264267	0.398208
hsa_circ_	0.369977	0.398209
hsa_circ_	-0.14122	0.398212
hsa_circ_	0.303441	0.39824
hsa_circ_	-0.30863	0.398245
hsa_circ_	0.142105	0.39826
hsa_circ_	0.156731	0.398304
hsa_circ_	0.292579	0.398317
hsa_circ_	-0.15126	0.398319
hsa_circ_	0.134392	0.39832
hsa_circ_	0.337251	0.398325
hsa_circ_	0.142857	0.39833
hsa_circ_	-0.57904	0.398334
hsa_circ_	0.442375	0.398338
hsa_circ_	-0.21083	0.398367
hsa_circ_	-0.16498	0.398377
hsa_circ_	0.191793	0.398393
hsa_circ_	0.405561	0.398411
hsa_circ_	0.174617	0.398411
hsa_circ_	0.12757	0.39842
hsa_circ_	0.164079	0.398439
hsa_circ_	-0.15002	0.398483
hsa_circ_	-0.18296	0.398519
hsa_circ_	0.193203	0.39852
hsa_circ_	0.227492	0.398522
hsa_circ_	-0.3044	0.398552
hsa_circ_	0.1444	0.398554

hsa_circ_	-0.74005	0.398636
hsa_circ_	-0.14413	0.398676
hsa_circ_	0.144465	0.398699
hsa_circ_	-0.82339	0.398708
hsa_circ_	-0.24637	0.398709
hsa_circ_	-0.17167	0.398713
hsa_circ_	0.319632	0.398732
hsa_circ_	0.169142	0.398751
hsa_circ_	0.163884	0.398768
hsa_circ_	0.186386	0.398777
hsa_circ_	-0.18335	0.398779
hsa_circ_	-0.53336	0.398799
hsa_circ_	-0.11596	0.398809
hsa_circ_	-0.43597	0.398821
hsa_circ_	-0.72817	0.398824
hsa_circ_	0.121197	0.398843
hsa_circ_	0.238613	0.398853
hsa_circ_	0.156339	0.398858
hsa_circ_	0.191432	0.398895
hsa_circ_	-0.1974	0.398902
hsa_circ_	-0.21931	0.398906
hsa_circ_	-0.50225	0.398926
hsa_circ_	0.471577	0.39894
hsa_circ_	-0.24661	0.398945
hsa_circ_	0.139485	0.398949
hsa_circ_	-0.76751	0.398962
hsa_circ_	0.201305	0.398962
hsa_circ_	-0.10927	0.398986
hsa_circ_	0.261298	0.399013
hsa_circ_	-0.18869	0.399038
hsa_circ_	0.177963	0.399045
hsa_circ_	0.177889	0.399053
hsa_circ_	-0.12631	0.399054
hsa_circ_	-0.48854	0.399063
hsa_circ_	-0.32913	0.399071
hsa_circ_	-0.13101	0.399087
hsa_circ_	0.229389	0.399104
hsa_circ_	0.478517	0.399122
hsa_circ_	0.165642	0.399131
hsa_circ_	0.494212	0.399152
hsa_circ_	0.215747	0.399194
hsa_circ_	0.402758	0.399195
hsa_circ_	-0.22547	0.399207
hsa_circ_	0.205262	0.399213
hsa_circ_	-0.30938	0.399214
hsa_circ_	0.40127	0.399222
hsa_circ_	0.252659	0.399225
hsa_circ_	-0.1327	0.39923
hsa_circ_	0.269713	0.399231
hsa_circ_	-0.11223	0.399234
hsa_circ_	0.140244	0.399234
hsa_circ_	-0.13619	0.399238
hsa_circ_	-0.15034	0.399281
hsa_circ_	-0.11235	0.399314

hsa_circ_	0.204389	0.399336
hsa_circ_	-0.12953	0.399354
hsa_circ_	-0.19215	0.399358
hsa_circ_	-0.56057	0.399374
hsa_circ_	0.352949	0.399381
hsa_circ_	-0.13984	0.399402
hsa_circ_	0.173944	0.399417
hsa_circ_	-0.2364	0.399421
hsa_circ_	0.124326	0.399433
hsa_circ_	0.485268	0.399448
hsa_circ_	0.16369	0.399498
hsa_circ_	0.240339	0.399511
hsa_circ_	-0.13937	0.399515
hsa_circ_	0.245558	0.399538
hsa_circ_	-0.11332	0.399538
hsa_circ_	0.165548	0.399544
hsa_circ_	0.311754	0.399581
hsa_circ_	0.156451	0.399596
hsa_circ_	0.161921	0.399623
hsa_circ_	-0.36266	0.399635
hsa_circ_	0.138996	0.399637
hsa_circ_	0.369705	0.399645
hsa_circ_	-0.18063	0.399656
hsa_circ_	0.300377	0.399658
hsa_circ_	0.163456	0.399685
hsa_circ_	-0.40546	0.399702
hsa_circ_	0.169704	0.399704
hsa_circ_	-0.17905	0.399707
hsa_circ_	-0.14268	0.399708
hsa_circ_	-0.1562	0.399711
hsa_circ_	-0.12179	0.399715
hsa_circ_	-0.15872	0.399716
hsa_circ_	0.138464	0.399719
hsa_circ_	-0.2229	0.39975
hsa_circ_	-0.1316	0.399802
hsa_circ_	0.349436	0.399804
hsa_circ_	0.197812	0.399827
hsa_circ_	0.295119	0.399829
hsa_circ_	0.111347	0.399856
hsa_circ_	0.321824	0.399946
hsa_circ_	0.201691	0.399981
hsa_circ_	-0.4021	0.400014
hsa_circ_	-0.23367	0.400015
hsa_circ_	-0.11922	0.400066
hsa_circ_	-0.22952	0.400078
hsa_circ_	-0.12114	0.40008
hsa_circ_	0.255848	0.400086
hsa_circ_	-0.19232	0.400089
hsa_circ_	-0.15382	0.40009
hsa_circ_	-0.10795	0.4001
hsa_circ_	-0.10751	0.400102
hsa_circ_	0.183455	0.400107
hsa_circ_	-0.11969	0.400126
hsa_circ_	-0.28414	0.40013

hsa_circ_	0.139888	0.400138
hsa_circ_	-0.18006	0.400158
hsa_circ_	-0.68905	0.400217
hsa_circ_	-0.13955	0.400221
hsa_circ_	0.448882	0.400225
hsa_circ_	0.166445	0.400228
hsa_circ_	-0.12866	0.400237
hsa_circ_	-0.4212	0.400255
hsa_circ_	0.142091	0.400256
hsa_circ_	0.153325	0.400276
hsa_circ_	-0.37071	0.400289
hsa_circ_	-0.15344	0.400289
hsa_circ_	-0.35413	0.400319
hsa_circ_	0.154103	0.400348
hsa_circ_	-0.15794	0.400382
hsa_circ_	0.406672	0.400387
hsa_circ_	0.181995	0.400398
hsa_circ_	0.174343	0.4004
hsa_circ_	0.121173	0.400406
hsa_circ_	0.39553	0.400407
hsa_circ_	-0.15281	0.400411
hsa_circ_	-0.44099	0.400419
hsa_circ_	-0.13814	0.400424
hsa_circ_	-0.1328	0.400475
hsa_circ_	0.19227	0.400482
hsa_circ_	0.135542	0.400487
hsa_circ_	0.491646	0.400487
hsa_circ_	-0.15231	0.400504
hsa_circ_	0.126352	0.400504
hsa_circ_	-0.14747	0.400516
hsa_circ_	-0.24336	0.400516
hsa_circ_	-0.15181	0.400523
hsa_circ_	-0.15355	0.400525
hsa_circ_	-0.2333	0.40054
hsa_circ_	0.282752	0.400581
hsa_circ_	-0.12162	0.400589
hsa_circ_	-0.12855	0.400603
hsa_circ_	0.140858	0.400606
hsa_circ_	0.139678	0.40062
hsa_circ_	-0.15079	0.400634
hsa_circ_	0.136425	0.400657
hsa_circ_	-0.55513	0.40067
hsa_circ_	-0.13682	0.400673
hsa_circ_	0.174432	0.400685
hsa_circ_	-0.42553	0.400688
hsa_circ_	-0.14499	0.400696
hsa_circ_	-0.1499	0.400701
hsa_circ_	0.196003	0.400716
hsa_circ_	-0.11337	0.400722
hsa_circ_	-0.5945	0.400727
hsa_circ_	-0.14348	0.400728
hsa_circ_	-0.45899	0.400736
hsa_circ_	0.157581	0.400745
hsa_circ_	-0.34056	0.40076

hsa_circ_	0.15846	0.400765
hsa_circ_	0.341179	0.400845
hsa_circ_	0.223976	0.400853
hsa_circ_	-0.16105	0.400879
hsa_circ_	-0.22517	0.40088
hsa_circ_	-0.15644	0.400887
hsa_circ_	-0.18121	0.400897
hsa_circ_	-0.32203	0.400917
hsa_circ_	-0.13934	0.400929
hsa_circ_	0.149515	0.400942
hsa_circ_	-0.15376	0.400954
hsa_circ_	-0.46115	0.400963
hsa_circ_	-0.10787	0.400978
hsa_circ_	-0.17961	0.401002
hsa_circ_	-0.14825	0.401032
hsa_circ_	0.144281	0.401044
hsa_circ_	-0.17203	0.401052
hsa_circ_	-0.33902	0.401074
hsa_circ_	-0.23441	0.401074
hsa_circ_	0.182015	0.401082
hsa_circ_	-0.14679	0.401091
hsa_circ_	-0.12548	0.401103
hsa_circ_	-0.68736	0.401116
hsa_circ_	-0.11194	0.401125
hsa_circ_	0.133009	0.401132
hsa_circ_	0.145167	0.401157
hsa_circ_	0.131184	0.401164
hsa_circ_	0.182635	0.401176
hsa_circ_	0.184172	0.401191
hsa_circ_	-0.17385	0.401194
hsa_circ_	0.593385	0.401239
hsa_circ_	-0.64702	0.40126
hsa_circ_	-0.21181	0.401284
hsa_circ_	0.197141	0.401302
hsa_circ_	-0.12643	0.401323
hsa_circ_	0.131061	0.401324
hsa_circ_	-0.1584	0.40133
hsa_circ_	0.145797	0.401337
hsa_circ_	0.223068	0.401387
hsa_circ_	-0.13574	0.401423
hsa_circ_	0.283635	0.401429
hsa_circ_	-0.10814	0.40144
hsa_circ_	-0.14968	0.401445
hsa_circ_	-0.48988	0.401451
hsa_circ_	0.426645	0.401463
hsa_circ_	0.237684	0.401514
hsa_circ_	0.365017	0.401519
hsa_circ_	0.285963	0.401552
hsa_circ_	-0.72296	0.401586
hsa_circ_	-0.26353	0.401586
hsa_circ_	-0.21129	0.401591
hsa_circ_	-0.17108	0.401596
hsa_circ_	0.251925	0.401601
hsa_circ_	-0.47385	0.401603

hsa_circ_	-0.40251	0.40162
hsa_circ_	-0.18505	0.401649
hsa_circ_	-0.44091	0.40166
hsa_circ_	-0.33698	0.401662
hsa_circ_	-0.15076	0.401669
hsa_circ_	-0.12335	0.401679
hsa_circ_	-0.17274	0.401708
hsa_circ_	0.16288	0.401709
hsa_circ_	0.229434	0.401713
hsa_circ_	0.113089	0.401719
hsa_circ_	0.250788	0.401735
hsa_circ_	-0.13842	0.401741
hsa_circ_	0.19401	0.401743
hsa_circ_	-0.20331	0.401743
hsa_circ_	0.196969	0.401792
hsa_circ_	0.153218	0.401802
hsa_circ_	-0.1516	0.401808
hsa_circ_	0.180359	0.401815
hsa_circ_	0.131609	0.401833
hsa_circ_	0.35756	0.40185
hsa_circ_	-0.20785	0.401871
hsa_circ_	-0.15889	0.401876
hsa_circ_	0.345921	0.401879
hsa_circ_	-0.13242	0.401893
hsa_circ_	-0.15763	0.401895
hsa_circ_	-0.21845	0.401898
hsa_circ_	0.178074	0.401899
hsa_circ_	-0.50511	0.401899
hsa_circ_	0.12048	0.401917
hsa_circ_	0.196298	0.401924
hsa_circ_	-0.29415	0.401933
hsa_circ_	0.217852	0.401941
hsa_circ_	-0.25171	0.401941
hsa_circ_	-0.13285	0.401947
hsa_circ_	-0.15725	0.401957
hsa_circ_	-0.20208	0.401965
hsa_circ_	0.184315	0.401978
hsa_circ_	0.162224	0.40198
hsa_circ_	0.14033	0.401999
hsa_circ_	0.527775	0.40202
hsa_circ_	-0.17203	0.402025
hsa_circ_	-0.36274	0.402053
hsa_circ_	-0.268	0.40208
hsa_circ_	-0.16998	0.402097
hsa_circ_	-0.13105	0.402126
hsa_circ_	0.117633	0.402134
hsa_circ_	-0.22114	0.402146
hsa_circ_	-0.16094	0.402155
hsa_circ_	0.131135	0.402156
hsa_circ_	-0.63489	0.402164
hsa_circ_	-0.11512	0.40217
hsa_circ_	0.229455	0.402192
hsa_circ_	-0.138	0.402196
hsa_circ_	-0.6924	0.402201

hsa_circ_	0.318935	0.402201
hsa_circ_	-0.35265	0.402211
hsa_circ_	-0.14531	0.402221
hsa_circ_	-0.12552	0.402224
hsa_circ_	-0.18123	0.402261
hsa_circ_	-0.33099	0.402293
hsa_circ_	-0.5954	0.402293
hsa_circ_	0.169049	0.402298
hsa_circ_	-0.36879	0.402298
hsa_circ_	0.544205	0.402312
hsa_circ_	0.146415	0.402328
hsa_circ_	0.291108	0.40233
hsa_circ_	0.179372	0.402334
hsa_circ_	-0.2216	0.402339
hsa_circ_	-0.12913	0.402342
hsa_circ_	0.158298	0.402352
hsa_circ_	-0.13466	0.402356
hsa_circ_	-0.21511	0.402358
hsa_circ_	-0.27563	0.402371
hsa_circ_	-0.21371	0.402392
hsa_circ_	0.390859	0.402415
hsa_circ_	0.494617	0.402429
hsa_circ_	-0.127	0.402448
hsa_circ_	0.13887	0.402453
hsa_circ_	-0.43077	0.402456
hsa_circ_	0.16917	0.402467
hsa_circ_	-0.11146	0.402478
hsa_circ_	-0.28362	0.402494
hsa_circ_	0.287259	0.402499
hsa_circ_	-0.28524	0.402499
hsa_circ_	-0.11852	0.402527
hsa_circ_	0.229465	0.402538
hsa_circ_	-0.12956	0.402547
hsa_circ_	0.113671	0.402564
hsa_circ_	0.131579	0.402565
hsa_circ_	-0.16574	0.402586
hsa_circ_	-0.15434	0.402619
hsa_circ_	0.477062	0.402622
hsa_circ_	-0.94162	0.402623
hsa_circ_	-0.14478	0.402633
hsa_circ_	-0.37273	0.402634
hsa_circ_	-0.26457	0.402641
hsa_circ_	0.625551	0.402645
hsa_circ_	-0.14559	0.402657
hsa_circ_	0.166783	0.402694
hsa_circ_	0.610851	0.4027
hsa_circ_	-0.13882	0.40274
hsa_circ_	0.142519	0.402741
hsa_circ_	0.605548	0.402789
hsa_circ_	-0.26548	0.402792
hsa_circ_	0.14751	0.402806
hsa_circ_	-0.16137	0.402815
hsa_circ_	0.243781	0.402829
hsa_circ_	0.193718	0.40283

hsa_circ_	0.178221	0.402844
hsa_circ_	-0.1275	0.402861
hsa_circ_	0.175778	0.40287
hsa_circ_	0.16661	0.402875
hsa_circ_	-0.13711	0.402918
hsa_circ_	-0.21954	0.402924
hsa_circ_	0.138252	0.402942
hsa_circ_	-0.16023	0.402955
hsa_circ_	-0.25458	0.402984
hsa_circ_	0.295208	0.403008
hsa_circ_	0.206024	0.403025
hsa_circ_	-0.19474	0.403029
hsa_circ_	-0.46453	0.40304
hsa_circ_	-0.32571	0.403048
hsa_circ_	-0.11052	0.403049
hsa_circ_	0.148481	0.403054
hsa_circ_	-0.16811	0.403068
hsa_circ_	0.309776	0.403075
hsa_circ_	0.176054	0.403075
hsa_circ_	0.173586	0.403077
hsa_circ_	0.146095	0.403078
hsa_circ_	-0.1652	0.403086
hsa_circ_	-0.38808	0.403093
hsa_circ_	0.120548	0.403101
hsa_circ_	0.157126	0.403109
hsa_circ_	0.205415	0.403121
hsa_circ_	-0.12008	0.403124
hsa_circ_	0.22021	0.403129
hsa_circ_	-0.20472	0.403144
hsa_circ_	-0.15024	0.403144
hsa_circ_	-0.26751	0.40315
hsa_circ_	-0.18131	0.403151
hsa_circ_	0.136644	0.403159
hsa_circ_	0.310718	0.403175
hsa_circ_	-0.14887	0.403179
hsa_circ_	-0.22382	0.403184
hsa_circ_	-0.13262	0.403185
hsa_circ_	-0.16479	0.403207
hsa_circ_	-0.14259	0.403221
hsa_circ_	0.136615	0.403227
hsa_circ_	-0.18992	0.403229
hsa_circ_	-0.14644	0.40325
hsa_circ_	-0.27065	0.403255
hsa_circ_	-0.19865	0.403268
hsa_circ_	-0.25872	0.403272
hsa_circ_	-0.15312	0.403277
hsa_circ_	0.188847	0.403295
hsa_circ_	0.215458	0.403297
hsa_circ_	-0.47745	0.403301
hsa_circ_	-0.12707	0.403316
hsa_circ_	0.169584	0.403317
hsa_circ_	0.193332	0.403317
hsa_circ_	0.360365	0.403328
hsa_circ_	-0.39498	0.403328

hsa_circ_	-0.16043	0.403372
hsa_circ_	-0.21505	0.403373
hsa_circ_	-0.20301	0.403375
hsa_circ_	0.153477	0.403379
hsa_circ_	-0.17091	0.403405
hsa_circ_	-1.89634	0.403417
hsa_circ_	0.11235	0.403439
hsa_circ_	0.587913	0.403447
hsa_circ_	0.167449	0.403465
hsa_circ_	0.218245	0.403487
hsa_circ_	0.22565	0.403496
hsa_circ_	-0.20534	0.403496
hsa_circ_	-0.12257	0.403522
hsa_circ_	0.125193	0.403524
hsa_circ_	0.210192	0.403527
hsa_circ_	0.150901	0.403542
hsa_circ_	0.171521	0.403561
hsa_circ_	0.419181	0.403562
hsa_circ_	-0.13265	0.403575
hsa_circ_	0.292238	0.403578
hsa_circ_	0.199918	0.403589
hsa_circ_	-0.18452	0.40359
hsa_circ_	-0.13852	0.4036
hsa_circ_	-0.1127	0.403607
hsa_circ_	0.191108	0.403619
hsa_circ_	0.319883	0.403621
hsa_circ_	-0.16092	0.403633
hsa_circ_	-0.13639	0.403662
hsa_circ_	0.150948	0.403682
hsa_circ_	-0.15415	0.40369
hsa_circ_	-0.15453	0.403703
hsa_circ_	0.401018	0.403715
hsa_circ_	0.246479	0.403721
hsa_circ_	0.382558	0.403725
hsa_circ_	0.275825	0.403728
hsa_circ_	0.197002	0.403734
hsa_circ_	0.230963	0.403755
hsa_circ_	-0.37634	0.40376
hsa_circ_	0.23358	0.40377
hsa_circ_	-0.1301	0.403795
hsa_circ_	0.191808	0.40387
hsa_circ_	0.126811	0.403882
hsa_circ_	-0.13023	0.403888
hsa_circ_	0.323977	0.403892
hsa_circ_	0.25909	0.4039
hsa_circ_	0.123539	0.4039
hsa_circ_	0.139834	0.403904
hsa_circ_	0.286708	0.403904
hsa_circ_	-0.21082	0.40392
hsa_circ_	-0.15995	0.403928
hsa_circ_	-0.34852	0.403931
hsa_circ_	-0.93505	0.40396
hsa_circ_	0.161975	0.403972
hsa_circ_	-0.15239	0.403982

hsa_circ_	0.174795	0.404007
hsa_circ_	-0.19961	0.40401
hsa_circ_	0.157931	0.404011
hsa_circ_	-0.14114	0.404025
hsa_circ_	-0.30046	0.404028
hsa_circ_	-0.19834	0.404037
hsa_circ_	0.211299	0.404046
hsa_circ_	-0.12453	0.40406
hsa_circ_	-0.26687	0.40411
hsa_circ_	-0.12105	0.404118
hsa_circ_	0.138392	0.40412
hsa_circ_	0.15299	0.404151
hsa_circ_	0.132945	0.404169
hsa_circ_	0.148372	0.404171
hsa_circ_	-0.16061	0.40418
hsa_circ_	0.271161	0.404213
hsa_circ_	-0.19479	0.404219
hsa_circ_	0.462295	0.404228
hsa_circ_	0.140221	0.404234
hsa_circ_	-0.12956	0.404236
hsa_circ_	0.173721	0.404264
hsa_circ_	0.244763	0.404267
hsa_circ_	-0.21008	0.404274
hsa_circ_	0.204554	0.404275
hsa_circ_	0.121862	0.404284
hsa_circ_	0.36004	0.404293
hsa_circ_	-0.17011	0.40431
hsa_circ_	-0.52935	0.404336
hsa_circ_	0.142516	0.404341
hsa_circ_	0.196125	0.404341
hsa_circ_	-0.18565	0.404352
hsa_circ_	-0.32821	0.404357
hsa_circ_	0.174268	0.404369
hsa_circ_	-0.16658	0.404392
hsa_circ_	0.344546	0.404399
hsa_circ_	-0.40615	0.404431
hsa_circ_	-0.14629	0.404432
hsa_circ_	-0.41099	0.40444
hsa_circ_	0.22888	0.404454
hsa_circ_	-0.36999	0.404456
hsa_circ_	-0.12683	0.404476
hsa_circ_	0.434322	0.4045
hsa_circ_	0.156739	0.404503
hsa_circ_	0.244219	0.404519
hsa_circ_	-0.75543	0.404536
hsa_circ_	0.17192	0.404555
hsa_circ_	0.928891	0.404556
hsa_circ_	0.29547	0.404589
hsa_circ_	-0.25618	0.404643
hsa_circ_	-0.33296	0.404643
hsa_circ_	0.162601	0.404644
hsa_circ_	-0.12041	0.40467
hsa_circ_	0.164556	0.404676
hsa_circ_	-0.11649	0.404676

hsa_circ_	0.150084	0.404682
hsa_circ_	0.489875	0.404685
hsa_circ_	-0.11467	0.404688
hsa_circ_	-0.42773	0.404704
hsa_circ_	-0.136	0.404711
hsa_circ_	-0.18647	0.404724
hsa_circ_	0.41604	0.404744
hsa_circ_	0.380581	0.404756
hsa_circ_	0.210895	0.404757
hsa_circ_	-0.18152	0.404774
hsa_circ_	0.1353	0.404777
hsa_circ_	0.121096	0.404796
hsa_circ_	0.126645	0.404813
hsa_circ_	-0.17014	0.404821
hsa_circ_	0.780713	0.404833
hsa_circ_	0.182306	0.404834
hsa_circ_	0.165948	0.404837
hsa_circ_	-0.13861	0.404847
hsa_circ_	-0.37451	0.404869
hsa_circ_	0.154671	0.404872
hsa_circ_	-0.29837	0.404873
hsa_circ_	-0.17164	0.404892
hsa_circ_	-0.15095	0.404923
hsa_circ_	-0.13747	0.404925
hsa_circ_	-0.51922	0.404926
hsa_circ_	-0.25714	0.40494
hsa_circ_	-0.34451	0.404946
hsa_circ_	-0.11274	0.404954
hsa_circ_	-0.2006	0.404964
hsa_circ_	0.15939	0.404975
hsa_circ_	-0.12905	0.404981
hsa_circ_	0.224848	0.405011
hsa_circ_	-0.10636	0.405017
hsa_circ_	0.130521	0.40502
hsa_circ_	-0.12645	0.405021
hsa_circ_	-0.19885	0.405034
hsa_circ_	-0.18894	0.40506
hsa_circ_	-0.23008	0.40508
hsa_circ_	-0.14937	0.405085
hsa_circ_	0.165446	0.405089
hsa_circ_	0.158914	0.405089
hsa_circ_	-0.12588	0.405095
hsa_circ_	-0.16521	0.405112
hsa_circ_	0.210977	0.405113
hsa_circ_	-0.16174	0.405119
hsa_circ_	-0.15314	0.405131
hsa_circ_	-0.42438	0.405145
hsa_circ_	-0.18792	0.40517
hsa_circ_	0.190974	0.405191
hsa_circ_	0.14011	0.405203
hsa_circ_	0.493845	0.405216
hsa_circ_	0.225086	0.405219
hsa_circ_	0.178881	0.405224
hsa_circ_	0.24261	0.405252

hsa_circ_	0.11981	0.405252
hsa_circ_	-0.17059	0.405254
hsa_circ_	-0.16168	0.405255
hsa_circ_	-0.21902	0.405262
hsa_circ_	-0.21211	0.405269
hsa_circ_	0.141607	0.405295
hsa_circ_	-0.15204	0.405297
hsa_circ_	0.164182	0.405301
hsa_circ_	0.303805	0.405303
hsa_circ_	-0.29699	0.405309
hsa_circ_	-0.18322	0.40533
hsa_circ_	0.207083	0.405331
hsa_circ_	0.113283	0.405344
hsa_circ_	-0.31302	0.405371
hsa_circ_	-0.12583	0.405378
hsa_circ_	-0.24778	0.405408
hsa_circ_	-0.13564	0.405436
hsa_circ_	0.12607	0.405443
hsa_circ_	-0.24465	0.405447
hsa_circ_	0.207888	0.405456
hsa_circ_	-0.60201	0.405464
hsa_circ_	0.242822	0.405464
hsa_circ_	-0.15119	0.405466
hsa_circ_	-0.11303	0.405471
hsa_circ_	-0.63054	0.405472
hsa_circ_	-0.17903	0.405473
hsa_circ_	-0.14518	0.405502
hsa_circ_	-0.27665	0.405522
hsa_circ_	0.148758	0.405523
hsa_circ_	-0.19204	0.405523
hsa_circ_	0.21678	0.405541
hsa_circ_	-0.20302	0.405554
hsa_circ_	-0.10776	0.405575
hsa_circ_	0.145748	0.405583
hsa_circ_	0.169779	0.405664
hsa_circ_	0.205517	0.405675
hsa_circ_	0.204497	0.405679
hsa_circ_	0.518301	0.405696
hsa_circ_	-0.17453	0.405701
hsa_circ_	-0.24307	0.405711
hsa_circ_	-0.17229	0.405728
hsa_circ_	0.183069	0.405729
hsa_circ_	0.145789	0.405759
hsa_circ_	-0.12027	0.405768
hsa_circ_	-0.13281	0.405783
hsa_circ_	-0.40909	0.405789
hsa_circ_	-0.5998	0.405791
hsa_circ_	-0.19783	0.405799
hsa_circ_	0.227611	0.405804
hsa_circ_	-0.17037	0.405816
hsa_circ_	-0.21782	0.40582
hsa_circ_	0.233685	0.405848
hsa_circ_	-0.18647	0.40586
hsa_circ_	-0.11914	0.405861

hsa_circ_	0.143328	0.405862
hsa_circ_	-0.12471	0.405876
hsa_circ_	-0.23068	0.405879
hsa_circ_	0.117993	0.405889
hsa_circ_	-0.17601	0.40589
hsa_circ_	0.127326	0.405948
hsa_circ_	-0.12802	0.406008
hsa_circ_	-0.16522	0.406011
hsa_circ_	0.155423	0.406035
hsa_circ_	-0.16884	0.406039
hsa_circ_	-0.18625	0.40604
hsa_circ_	-0.1321	0.406053
hsa_circ_	-0.12235	0.406059
hsa_circ_	0.277451	0.406072
hsa_circ_	0.236986	0.40609
hsa_circ_	0.237192	0.406096
hsa_circ_	0.249353	0.406099
hsa_circ_	0.329114	0.406106
hsa_circ_	0.306028	0.406111
hsa_circ_	-0.24159	0.406112
hsa_circ_	-0.12301	0.406118
hsa_circ_	-0.3733	0.406118
hsa_circ_	-0.34737	0.406131
hsa_circ_	0.266607	0.406138
hsa_circ_	0.181424	0.406151
hsa_circ_	0.1302	0.406156
hsa_circ_	-0.2383	0.406156
hsa_circ_	-0.38265	0.406161
hsa_circ_	-0.12666	0.406162
hsa_circ_	0.489233	0.406164
hsa_circ_	0.167756	0.406192
hsa_circ_	0.640765	0.406221
hsa_circ_	0.243952	0.406239
hsa_circ_	0.286063	0.406247
hsa_circ_	-0.15776	0.406269
hsa_circ_	-0.22497	0.406275
hsa_circ_	0.203304	0.406281
hsa_circ_	-0.1374	0.406335
hsa_circ_	-0.14199	0.406335
hsa_circ_	0.281302	0.406352
hsa_circ_	0.169454	0.40638
hsa_circ_	0.182742	0.406406
hsa_circ_	-0.11814	0.40641
hsa_circ_	-0.14388	0.406413
hsa_circ_	-0.11802	0.406454
hsa_circ_	0.145849	0.406468
hsa_circ_	0.154367	0.406474
hsa_circ_	-0.14293	0.406487
hsa_circ_	-0.12046	0.406488
hsa_circ_	-0.24131	0.406497
hsa_circ_	0.256559	0.406507
hsa_circ_	0.230373	0.406523
hsa_circ_	0.156537	0.406527
hsa_circ_	0.651155	0.406532

hsa_circ_	-0.15777	0.406578
hsa_circ_	0.150687	0.406581
hsa_circ_	0.313526	0.406592
hsa_circ_	-0.45006	0.406598
hsa_circ_	-0.24911	0.406602
hsa_circ_	0.119427	0.406606
hsa_circ_	0.211175	0.406608
hsa_circ_	0.133287	0.406627
hsa_circ_	0.177054	0.406631
hsa_circ_	0.268633	0.406637
hsa_circ_	0.166393	0.406647
hsa_circ_	0.125305	0.406667
hsa_circ_	0.141852	0.406669
hsa_circ_	0.217462	0.406706
hsa_circ_	0.202369	0.406709
hsa_circ_	-0.21948	0.406733
hsa_circ_	-0.18914	0.406748
hsa_circ_	0.138003	0.406763
hsa_circ_	-0.15073	0.406769
hsa_circ_	-0.18266	0.40677
hsa_circ_	0.146917	0.406785
hsa_circ_	-0.17688	0.406814
hsa_circ_	-0.15497	0.406836
hsa_circ_	0.257717	0.406844
hsa_circ_	0.327751	0.406868
hsa_circ_	-0.97522	0.406907
hsa_circ_	0.191149	0.406919
hsa_circ_	0.339283	0.406971
hsa_circ_	-0.12937	0.406978
hsa_circ_	0.240204	0.406996
hsa_circ_	-0.19263	0.407027
hsa_circ_	0.16273	0.407033
hsa_circ_	-0.16697	0.407096
hsa_circ_	0.195535	0.407128
hsa_circ_	-0.33761	0.407131
hsa_circ_	-0.17664	0.407132
hsa_circ_	0.282584	0.407136
hsa_circ_	-0.23422	0.407141
hsa_circ_	-0.42544	0.407149
hsa_circ_	-0.37396	0.407149
hsa_circ_	0.141179	0.407158
hsa_circ_	0.146547	0.40716
hsa_circ_	-0.29141	0.40719
hsa_circ_	0.21994	0.4072
hsa_circ_	-0.36015	0.407211
hsa_circ_	0.124059	0.407219
hsa_circ_	-0.13548	0.407226
hsa_circ_	-0.15918	0.407234
hsa_circ_	0.133373	0.407242
hsa_circ_	0.130032	0.407243
hsa_circ_	0.429842	0.407247
hsa_circ_	0.255051	0.40726
hsa_circ_	0.439101	0.407268
hsa_circ_	-0.21606	0.407269

hsa_circ_	0.2002	0.407271
hsa_circ_	0.389249	0.407286
hsa_circ_	-0.15788	0.407288
hsa_circ_	0.116251	0.4073
hsa_circ_	0.115652	0.407307
hsa_circ_	-0.26556	0.407316
hsa_circ_	0.136778	0.407336
hsa_circ_	0.121448	0.407338
hsa_circ_	-0.22234	0.407339
hsa_circ_	0.122471	0.407354
hsa_circ_	0.222517	0.407369
hsa_circ_	-0.34297	0.407395
hsa_circ_	-0.13853	0.407402
hsa_circ_	0.118077	0.407403
hsa_circ_	-0.29639	0.407418
hsa_circ_	0.258245	0.407466
hsa_circ_	0.204956	0.407471
hsa_circ_	0.160402	0.407503
hsa_circ_	-0.16702	0.407504
hsa_circ_	0.30046	0.407513
hsa_circ_	0.425451	0.407517
hsa_circ_	0.188028	0.407536
hsa_circ_	0.554223	0.407553
hsa_circ_	0.194413	0.407567
hsa_circ_	0.141863	0.407569
hsa_circ_	-0.13579	0.407582
hsa_circ_	0.15716	0.407628
hsa_circ_	-0.27165	0.407649
hsa_circ_	0.19641	0.407658
hsa_circ_	0.135156	0.407663
hsa_circ_	0.152421	0.407679
hsa_circ_	-0.12136	0.407716
hsa_circ_	0.124402	0.407725
hsa_circ_	-0.26421	0.407757
hsa_circ_	-0.13957	0.40777
hsa_circ_	-0.17551	0.407771
hsa_circ_	-0.26547	0.407776
hsa_circ_	0.195144	0.407779
hsa_circ_	-0.4788	0.407781
hsa_circ_	-0.11836	0.407813
hsa_circ_	-0.18715	0.407815
hsa_circ_	0.455266	0.407817
hsa_circ_	-0.18051	0.407828
hsa_circ_	0.11796	0.40783
hsa_circ_	0.288109	0.407831
hsa_circ_	0.148989	0.40784
hsa_circ_	0.187115	0.407851
hsa_circ_	-0.28309	0.40787
hsa_circ_	-0.1323	0.407875
hsa_circ_	0.250247	0.407884
hsa_circ_	0.201866	0.407915
hsa_circ_	0.357233	0.407935
hsa_circ_	0.169734	0.407958
hsa_circ_	-0.65284	0.407979

hsa_circ_	-0.14993	0.407981
hsa_circ_	-0.39288	0.407993
hsa_circ_	-0.1513	0.408051
hsa_circ_	-0.22129	0.408052
hsa_circ_	-0.14248	0.408068
hsa_circ_	-0.17064	0.408088
hsa_circ_	0.187085	0.408091
hsa_circ_	-0.22103	0.40812
hsa_circ_	0.147265	0.408151
hsa_circ_	-0.1878	0.408161
hsa_circ_	-0.22409	0.408171
hsa_circ_	-0.16992	0.408184
hsa_circ_	0.222927	0.408184
hsa_circ_	-0.20214	0.408188
hsa_circ_	0.14624	0.408229
hsa_circ_	-0.15935	0.408305
hsa_circ_	-0.12198	0.408322
hsa_circ_	0.13447	0.408324
hsa_circ_	-0.51488	0.408325
hsa_circ_	-0.32419	0.408336
hsa_circ_	0.193399	0.40835
hsa_circ_	0.151615	0.408362
hsa_circ_	0.213194	0.408365
hsa_circ_	-0.13263	0.408373
hsa_circ_	-0.15226	0.408373
hsa_circ_	-0.14125	0.408419
hsa_circ_	-0.21015	0.40845
hsa_circ_	-0.20918	0.408456
hsa_circ_	0.142127	0.408461
hsa_circ_	0.121754	0.408486
hsa_circ_	-0.17844	0.408488
hsa_circ_	-0.1165	0.408499
hsa_circ_	0.138283	0.408503
hsa_circ_	0.191138	0.408514
hsa_circ_	-0.34764	0.408537
hsa_circ_	-0.11305	0.408572
hsa_circ_	-0.33872	0.408574
hsa_circ_	-0.10679	0.408579
hsa_circ_	-0.15468	0.408603
hsa_circ_	-0.22027	0.40863
hsa_circ_	-0.25602	0.408638
hsa_circ_	0.133628	0.408664
hsa_circ_	-0.23834	0.408666
hsa_circ_	-0.13678	0.408668
hsa_circ_	0.140689	0.408713
hsa_circ_	-0.20498	0.408715
hsa_circ_	0.800546	0.40872
hsa_circ_	-0.20714	0.408723
hsa_circ_	-0.1271	0.408724
hsa_circ_	-0.1974	0.40873
hsa_circ_	0.374731	0.408735
hsa_circ_	0.230001	0.408736
hsa_circ_	0.156739	0.408752
hsa_circ_	0.19405	0.408752

hsa_circ_	-0.40762	0.40876
hsa_circ_	0.10929	0.408772
hsa_circ_	-0.13944	0.408777
hsa_circ_	-0.37202	0.408792
hsa_circ_	-0.16387	0.408815
hsa_circ_	-0.37152	0.408817
hsa_circ_	0.165333	0.408827
hsa_circ_	-0.11043	0.408849
hsa_circ_	-0.13412	0.408851
hsa_circ_	-0.16575	0.408863
hsa_circ_	-0.16395	0.408864
hsa_circ_	-0.18081	0.408871
hsa_circ_	-0.18481	0.408891
hsa_circ_	-0.30967	0.408942
hsa_circ_	-0.12167	0.408943
hsa_circ_	0.258353	0.408944
hsa_circ_	0.152947	0.408987
hsa_circ_	-0.11446	0.409004
hsa_circ_	0.142892	0.409031
hsa_circ_	0.146069	0.409032
hsa_circ_	-0.43629	0.409032
hsa_circ_	0.156802	0.40904
hsa_circ_	0.186998	0.409041
hsa_circ_	-0.13022	0.409048
hsa_circ_	-0.11556	0.409061
hsa_circ_	0.59204	0.40907
hsa_circ_	-0.27198	0.409088
hsa_circ_	0.229752	0.409089
hsa_circ_	-0.22622	0.409094
hsa_circ_	0.16644	0.409118
hsa_circ_	0.384866	0.409118
hsa_circ_	-0.15156	0.409153
hsa_circ_	0.164053	0.409182
hsa_circ_	-0.18903	0.409203
hsa_circ_	-0.22735	0.409204
hsa_circ_	-0.37948	0.409213
hsa_circ_	0.142263	0.409214
hsa_circ_	0.444321	0.409218
hsa_circ_	0.410155	0.409218
hsa_circ_	-0.20134	0.409227
hsa_circ_	-0.24259	0.40923
hsa_circ_	-0.67049	0.409231
hsa_circ_	0.298376	0.409234
hsa_circ_	0.192015	0.409242
hsa_circ_	-0.17824	0.409245
hsa_circ_	0.220295	0.409253
hsa_circ_	0.265725	0.409255
hsa_circ_	-0.16008	0.409265
hsa_circ_	-0.15434	0.409266
hsa_circ_	-0.15531	0.409273
hsa_circ_	0.138101	0.409276
hsa_circ_	0.300584	0.409288
hsa_circ_	0.387202	0.409305
hsa_circ_	-0.24097	0.40931

hsa_circ_	-0.14827	0.409311
hsa_circ_	-0.2119	0.40933
hsa_circ_	-0.16302	0.409344
hsa_circ_	-0.16188	0.409349
hsa_circ_	0.347802	0.409351
hsa_circ_	0.500545	0.409365
hsa_circ_	-0.40674	0.409387
hsa_circ_	-0.15863	0.409391
hsa_circ_	-0.25016	0.409412
hsa_circ_	-0.13698	0.409419
hsa_circ_	0.435921	0.409437
hsa_circ_	-0.37273	0.409438
hsa_circ_	-0.27149	0.409471
hsa_circ_	-0.38836	0.409475
hsa_circ_	0.259106	0.409489
hsa_circ_	-0.22351	0.409494
hsa_circ_	0.144457	0.409512
hsa_circ_	0.215854	0.409524
hsa_circ_	-0.17183	0.409537
hsa_circ_	0.237697	0.409538
hsa_circ_	-0.12982	0.409573
hsa_circ_	-0.13901	0.40958
hsa_circ_	-0.26254	0.409594
hsa_circ_	0.505523	0.409616
hsa_circ_	0.247012	0.409633
hsa_circ_	-0.28033	0.409654
hsa_circ_	0.299509	0.409655
hsa_circ_	-0.15652	0.409658
hsa_circ_	-0.15652	0.409659
hsa_circ_	-0.20615	0.409671
hsa_circ_	0.156697	0.409676
hsa_circ_	0.171131	0.409681
hsa_circ_	-0.22064	0.409692
hsa_circ_	0.333878	0.409708
hsa_circ_	0.156101	0.409711
hsa_circ_	0.341071	0.409727
hsa_circ_	-0.17195	0.409755
hsa_circ_	0.310441	0.409777
hsa_circ_	0.408332	0.409791
hsa_circ_	0.158173	0.409802
hsa_circ_	0.224712	0.409813
hsa_circ_	0.243591	0.409823
hsa_circ_	0.689426	0.409823
hsa_circ_	0.21861	0.409849
hsa_circ_	0.143174	0.409853
hsa_circ_	0.165576	0.409883
hsa_circ_	0.119124	0.409884
hsa_circ_	-0.25327	0.409888
hsa_circ_	0.148137	0.409891
hsa_circ_	-0.12552	0.409895
hsa_circ_	0.198516	0.40991
hsa_circ_	0.133323	0.409943
hsa_circ_	0.489231	0.409943
hsa_circ_	-0.14084	0.409947

hsa_circ_	0.140351	0.409952
hsa_circ_	-0.12787	0.409954
hsa_circ_	-1.74423	0.40996
hsa_circ_	0.133479	0.409971
hsa_circ_	-0.12961	0.409983
hsa_circ_	-0.42637	0.40999
hsa_circ_	0.187745	0.41001
hsa_circ_	-0.15896	0.410012
hsa_circ_	-0.11401	0.410022
hsa_circ_	-0.54956	0.410035
hsa_circ_	-0.42631	0.410037
hsa_circ_	0.348463	0.410045
hsa_circ_	-0.3641	0.410068
hsa_circ_	-0.17335	0.410084
hsa_circ_	-0.24709	0.410086
hsa_circ_	-0.57774	0.410146
hsa_circ_	0.281137	0.410178
hsa_circ_	-0.20307	0.410185
hsa_circ_	-0.146	0.410212
hsa_circ_	-0.1619	0.410226
hsa_circ_	-0.49293	0.410251
hsa_circ_	0.269569	0.410264
hsa_circ_	0.228916	0.410279
hsa_circ_	0.16837	0.410283
hsa_circ_	-0.52076	0.410298
hsa_circ_	0.184775	0.410312
hsa_circ_	-0.17098	0.410333
hsa_circ_	0.244389	0.410354
hsa_circ_	-0.36943	0.410391
hsa_circ_	-0.21865	0.410401
hsa_circ_	0.151032	0.410409
hsa_circ_	-0.4482	0.41043
hsa_circ_	-0.20116	0.410443
hsa_circ_	-0.17409	0.410475
hsa_circ_	-0.17632	0.410479
hsa_circ_	-0.16835	0.410511
hsa_circ_	0.231939	0.410512
hsa_circ_	-0.19936	0.410513
hsa_circ_	0.146467	0.410525
hsa_circ_	-0.12711	0.410528
hsa_circ_	-0.20817	0.410536
hsa_circ_	0.336921	0.410543
hsa_circ_	0.118065	0.410546
hsa_circ_	-0.1446	0.410549
hsa_circ_	-0.26024	0.410549
hsa_circ_	-0.2189	0.410555
hsa_circ_	-0.1319	0.41056
hsa_circ_	-0.12854	0.410566
hsa_circ_	0.200088	0.410619
hsa_circ_	0.15289	0.410657
hsa_circ_	-0.20532	0.410664
hsa_circ_	-0.13714	0.410726
hsa_circ_	-0.13845	0.410731
hsa_circ_	0.147472	0.410773

hsa_circ_	0.227682	0.410779
hsa_circ_	0.162937	0.410779
hsa_circ_	-0.38148	0.410797
hsa_circ_	-0.13205	0.410822
hsa_circ_	0.274622	0.410827
hsa_circ_	-0.20545	0.410834
hsa_circ_	0.146501	0.410834
hsa_circ_	-0.50656	0.410839
hsa_circ_	0.161008	0.410844
hsa_circ_	0.117531	0.410852
hsa_circ_	0.212656	0.410853
hsa_circ_	0.355845	0.410854
hsa_circ_	-0.16023	0.410857
hsa_circ_	0.122829	0.410874
hsa_circ_	-0.29682	0.410885
hsa_circ_	0.13881	0.410887
hsa_circ_	-0.12522	0.410889
hsa_circ_	0.167953	0.410918
hsa_circ_	-0.84162	0.410931
hsa_circ_	0.132798	0.410934
hsa_circ_	0.148875	0.410948
hsa_circ_	-0.18017	0.410949
hsa_circ_	0.20429	0.410998
hsa_circ_	0.560632	0.41101
hsa_circ_	-0.45033	0.411027
hsa_circ_	0.18687	0.411027
hsa_circ_	-0.37053	0.411036
hsa_circ_	-0.13948	0.411054
hsa_circ_	0.194019	0.411094
hsa_circ_	0.12886	0.411095
hsa_circ_	0.506584	0.411118
hsa_circ_	-0.15063	0.411122
hsa_circ_	0.20513	0.411159
hsa_circ_	-0.11335	0.411165
hsa_circ_	-0.17193	0.41119
hsa_circ_	0.192477	0.411206
hsa_circ_	0.106532	0.411225
hsa_circ_	0.11526	0.411236
hsa_circ_	-0.18032	0.41124
hsa_circ_	-0.11924	0.411241
hsa_circ_	0.174059	0.411249
hsa_circ_	0.117947	0.411259
hsa_circ_	0.22597	0.41126
hsa_circ_	0.310933	0.411291
hsa_circ_	-0.1766	0.411321
hsa_circ_	0.27056	0.411323
hsa_circ_	-0.14522	0.411331
hsa_circ_	-0.11091	0.411361
hsa_circ_	0.114328	0.411369
hsa_circ_	-0.47287	0.411405
hsa_circ_	0.519978	0.411438
hsa_circ_	-0.1709	0.411457
hsa_circ_	0.286621	0.411469
hsa_circ_	0.231017	0.411479

hsa_circ_	-0.13876	0.411504
hsa_circ_	-0.15238	0.411506
hsa_circ_	-0.29205	0.411525
hsa_circ_	0.198408	0.411528
hsa_circ_	-0.17135	0.411535
hsa_circ_	0.25375	0.411545
hsa_circ_	-0.12579	0.411554
hsa_circ_	-0.13234	0.411563
hsa_circ_	0.291623	0.411566
hsa_circ_	-0.16805	0.411584
hsa_circ_	0.139345	0.411586
hsa_circ_	0.111995	0.411587
hsa_circ_	-0.10983	0.411605
hsa_circ_	0.246976	0.41161
hsa_circ_	0.275297	0.411622
hsa_circ_	0.15463	0.411634
hsa_circ_	0.13022	0.411652
hsa_circ_	-0.17548	0.411657
hsa_circ_	0.143673	0.411703
hsa_circ_	0.211992	0.411704
hsa_circ_	-0.41922	0.41171
hsa_circ_	0.269711	0.411725
hsa_circ_	0.273997	0.411726
hsa_circ_	-0.20449	0.411745
hsa_circ_	0.123447	0.41175
hsa_circ_	-0.1166	0.411759
hsa_circ_	0.291724	0.411761
hsa_circ_	-0.15992	0.411792
hsa_circ_	0.237742	0.411804
hsa_circ_	-0.21564	0.411808
hsa_circ_	-0.16148	0.411828
hsa_circ_	0.440496	0.411832
hsa_circ_	0.23061	0.411836
hsa_circ_	0.280952	0.41184
hsa_circ_	0.436972	0.411874
hsa_circ_	-0.14615	0.411889
hsa_circ_	0.314706	0.411893
hsa_circ_	0.469398	0.411903
hsa_circ_	0.139191	0.411905
hsa_circ_	-0.54282	0.411906
hsa_circ_	-0.11913	0.411939
hsa_circ_	0.119784	0.411947
hsa_circ_	0.171198	0.411955
hsa_circ_	0.168326	0.411971
hsa_circ_	-0.17402	0.411986
hsa_circ_	-0.15938	0.412
hsa_circ_	-0.15566	0.412004
hsa_circ_	0.155811	0.412018
hsa_circ_	-0.1852	0.412023
hsa_circ_	-0.24672	0.412024
hsa_circ_	-0.17695	0.412042
hsa_circ_	0.38562	0.41205
hsa_circ_	-0.11388	0.412056
hsa_circ_	-0.18074	0.412064

hsa_circ_	-0.18693	0.412091
hsa_circ_	0.244632	0.412108
hsa_circ_	0.312218	0.412118
hsa_circ_	-0.13199	0.41212
hsa_circ_	-0.20119	0.412124
hsa_circ_	0.115214	0.412127
hsa_circ_	-0.12005	0.412146
hsa_circ_	-0.12827	0.412195
hsa_circ_	-0.10729	0.41221
hsa_circ_	0.128988	0.412218
hsa_circ_	-0.11369	0.412218
hsa_circ_	-0.43459	0.412241
hsa_circ_	-0.14354	0.412242
hsa_circ_	-0.26092	0.412244
hsa_circ_	0.386176	0.412245
hsa_circ_	0.205115	0.412252
hsa_circ_	0.112126	0.412274
hsa_circ_	0.12591	0.412283
hsa_circ_	-0.24023	0.412295
hsa_circ_	-0.16163	0.412319
hsa_circ_	-0.12622	0.412327
hsa_circ_	0.174728	0.412345
hsa_circ_	0.205015	0.41236
hsa_circ_	-0.38357	0.412383
hsa_circ_	0.143112	0.412404
hsa_circ_	0.178152	0.412409
hsa_circ_	-0.27407	0.412417
hsa_circ_	-0.25685	0.412423
hsa_circ_	-0.15259	0.412424
hsa_circ_	-0.13186	0.412428
hsa_circ_	-0.14128	0.412436
hsa_circ_	-0.11196	0.412439
hsa_circ_	-0.12427	0.412443
hsa_circ_	2.50776	0.412443
hsa_circ_	0.601817	0.412443
hsa_circ_	-0.15149	0.412454
hsa_circ_	-0.16219	0.412457
hsa_circ_	0.170724	0.412542
hsa_circ_	0.200998	0.412542
hsa_circ_	-0.1352	0.412543
hsa_circ_	-0.261	0.412559
hsa_circ_	0.19341	0.412562
hsa_circ_	0.336513	0.412596
hsa_circ_	0.12585	0.412596
hsa_circ_	-0.14605	0.412622
hsa_circ_	-0.20334	0.412642
hsa_circ_	-0.14239	0.412668
hsa_circ_	0.1653	0.412676
hsa_circ_	-0.11761	0.412693
hsa_circ_	-0.23583	0.412695
hsa_circ_	-0.11444	0.412708
hsa_circ_	0.353504	0.412711
hsa_circ_	0.144882	0.412711
hsa_circ_	-0.25255	0.41272

hsa_circ_	-0.6225	0.41273
hsa_circ_	0.208488	0.412735
hsa_circ_	0.205678	0.412741
hsa_circ_	-0.14597	0.412748
hsa_circ_	-0.20933	0.41276
hsa_circ_	-0.52588	0.412775
hsa_circ_	0.184134	0.41279
hsa_circ_	0.312572	0.412857
hsa_circ_	-0.17281	0.412865
hsa_circ_	-0.16989	0.412866
hsa_circ_	0.13799	0.412886
hsa_circ_	0.143771	0.41292
hsa_circ_	0.458497	0.412947
hsa_circ_	0.105119	0.412962
hsa_circ_	0.164501	0.412982
hsa_circ_	-0.12022	0.412999
hsa_circ_	0.221039	0.413004
hsa_circ_	-0.15161	0.413008
hsa_circ_	-0.14698	0.413013
hsa_circ_	-0.13932	0.413034
hsa_circ_	0.18491	0.413046
hsa_circ_	0.342268	0.413078
hsa_circ_	0.203182	0.41308
hsa_circ_	0.148969	0.413101
hsa_circ_	-0.17895	0.413105
hsa_circ_	-0.12621	0.413109
hsa_circ_	-0.25436	0.413134
hsa_circ_	0.224613	0.413134
hsa_circ_	0.2326	0.413152
hsa_circ_	0.126132	0.413157
hsa_circ_	-0.13439	0.41317
hsa_circ_	-0.15152	0.413212
hsa_circ_	-0.17385	0.413217
hsa_circ_	-0.16377	0.41324
hsa_circ_	-0.73663	0.413246
hsa_circ_	0.180389	0.413248
hsa_circ_	0.192531	0.413251
hsa_circ_	-0.31243	0.413271
hsa_circ_	-0.17919	0.413275
hsa_circ_	-0.10569	0.413276
hsa_circ_	-0.12944	0.413296
hsa_circ_	-0.13537	0.413303
hsa_circ_	-0.15137	0.41331
hsa_circ_	-0.36245	0.41332
hsa_circ_	0.11156	0.413324
hsa_circ_	0.174502	0.413328
hsa_circ_	-0.21655	0.413341
hsa_circ_	-0.12439	0.413349
hsa_circ_	-0.21237	0.413354
hsa_circ_	0.151078	0.413362
hsa_circ_	-0.18054	0.413371
hsa_circ_	0.119053	0.413376
hsa_circ_	-0.109	0.413385
hsa_circ_	-0.52497	0.413395

hsa_circ_	0.274637	0.413395
hsa_circ_	-0.20048	0.413397
hsa_circ_	0.366866	0.413404
hsa_circ_	-0.12127	0.413404
hsa_circ_	0.239059	0.413443
hsa_circ_	0.190378	0.413462
hsa_circ_	0.354906	0.413462
hsa_circ_	0.226663	0.413471
hsa_circ_	0.162407	0.413473
hsa_circ_	0.146016	0.413499
hsa_circ_	-0.1271	0.4135
hsa_circ_	-0.12582	0.413503
hsa_circ_	0.302643	0.413539
hsa_circ_	0.139252	0.413542
hsa_circ_	-0.23048	0.413552
hsa_circ_	0.186676	0.413562
hsa_circ_	-0.22299	0.413562
hsa_circ_	-0.25037	0.413614
hsa_circ_	0.344855	0.413618
hsa_circ_	-0.17694	0.41363
hsa_circ_	0.146723	0.413651
hsa_circ_	0.117642	0.413655
hsa_circ_	-0.12016	0.413667
hsa_circ_	-0.22018	0.413668
hsa_circ_	0.319582	0.413668
hsa_circ_	-0.21804	0.413668
hsa_circ_	-0.35593	0.413672
hsa_circ_	-0.37961	0.413684
hsa_circ_	-0.25317	0.413687
hsa_circ_	-0.13618	0.413687
hsa_circ_	-0.15903	0.413698
hsa_circ_	0.288905	0.413711
hsa_circ_	0.147247	0.413725
hsa_circ_	-0.13469	0.413773
hsa_circ_	-0.50587	0.413784
hsa_circ_	-0.14956	0.413788
hsa_circ_	-0.33841	0.41379
hsa_circ_	0.137885	0.413798
hsa_circ_	-0.46541	0.413809
hsa_circ_	-0.4408	0.413831
hsa_circ_	0.15109	0.413832
hsa_circ_	-0.4377	0.413837
hsa_circ_	-0.2057	0.413839
hsa_circ_	0.118417	0.413846
hsa_circ_	-0.16168	0.413865
hsa_circ_	0.171314	0.413872
hsa_circ_	0.214596	0.413873
hsa_circ_	-0.14966	0.413891
hsa_circ_	0.149687	0.4139
hsa_circ_	0.129328	0.41392
hsa_circ_	0.208315	0.41394
hsa_circ_	-0.18964	0.413951
hsa_circ_	0.123552	0.413974
hsa_circ_	-0.24329	0.414032

hsa_circ_	-0.59555	0.414067
hsa_circ_	0.683059	0.414091
hsa_circ_	-0.19382	0.414094
hsa_circ_	0.121264	0.414096
hsa_circ_	-0.60669	0.414106
hsa_circ_	-0.23282	0.414129
hsa_circ_	-0.13462	0.414145
hsa_circ_	-0.14401	0.414152
hsa_circ_	-0.13886	0.414165
hsa_circ_	0.110222	0.41417
hsa_circ_	-0.22748	0.414178
hsa_circ_	-0.23742	0.414179
hsa_circ_	-0.23733	0.414188
hsa_circ_	-0.43546	0.41419
hsa_circ_	-0.21064	0.414211
hsa_circ_	-0.51595	0.414225
hsa_circ_	-0.39163	0.414227
hsa_circ_	-0.47454	0.414234
hsa_circ_	0.165682	0.414237
hsa_circ_	-0.83923	0.414243
hsa_circ_	0.423962	0.414243
hsa_circ_	-0.20299	0.41425
hsa_circ_	-0.24873	0.414311
hsa_circ_	0.131249	0.414317
hsa_circ_	0.241487	0.414323
hsa_circ_	0.253649	0.414328
hsa_circ_	-0.16878	0.414351
hsa_circ_	0.35739	0.414351
hsa_circ_	-0.14618	0.414369
hsa_circ_	-0.15034	0.414387
hsa_circ_	0.216317	0.414388
hsa_circ_	0.266724	0.41439
hsa_circ_	0.368003	0.414404
hsa_circ_	-0.331	0.414421
hsa_circ_	0.106381	0.414424
hsa_circ_	-0.18721	0.414434
hsa_circ_	0.199631	0.414438
hsa_circ_	0.35281	0.414471
hsa_circ_	0.1924	0.414475
hsa_circ_	0.349919	0.414476
hsa_circ_	-0.1241	0.414495
hsa_circ_	-0.22668	0.414496
hsa_circ_	-0.13717	0.414502
hsa_circ_	0.158225	0.414507
hsa_circ_	-0.36115	0.41451
hsa_circ_	-0.13366	0.414526
hsa_circ_	0.128369	0.414528
hsa_circ_	0.125291	0.414537
hsa_circ_	0.263096	0.414538
hsa_circ_	-0.12722	0.414539
hsa_circ_	-0.13183	0.414543
hsa_circ_	-0.24343	0.414591
hsa_circ_	0.256211	0.414604
hsa_circ_	0.128281	0.414612

hsa_circ_	-0.24286	0.414623
hsa_circ_	-0.16659	0.414624
hsa_circ_	-0.10483	0.41467
hsa_circ_	-0.12605	0.414689
hsa_circ_	-0.47454	0.414706
hsa_circ_	-0.39704	0.414713
hsa_circ_	0.239169	0.41472
hsa_circ_	0.148146	0.414728
hsa_circ_	0.132271	0.414735
hsa_circ_	-0.18813	0.414754
hsa_circ_	-0.11663	0.414758
hsa_circ_	-0.18318	0.41476
hsa_circ_	-0.35282	0.4148
hsa_circ_	-0.15377	0.414805
hsa_circ_	0.209136	0.414808
hsa_circ_	0.174032	0.414812
hsa_circ_	0.300746	0.414815
hsa_circ_	0.280143	0.414822
hsa_circ_	0.408051	0.414823
hsa_circ_	-0.43449	0.414824
hsa_circ_	0.142334	0.414829
hsa_circ_	-0.23048	0.414843
hsa_circ_	-0.26093	0.414854
hsa_circ_	-0.21146	0.414877
hsa_circ_	0.177314	0.414877
hsa_circ_	0.288564	0.414906
hsa_circ_	0.579801	0.414911
hsa_circ_	-0.14258	0.414919
hsa_circ_	0.140801	0.414922
hsa_circ_	0.307375	0.414941
hsa_circ_	0.13323	0.414946
hsa_circ_	0.139621	0.414957
hsa_circ_	-0.23886	0.414959
hsa_circ_	-0.43115	0.414993
hsa_circ_	-0.24597	0.414997
hsa_circ_	-0.12706	0.415009
hsa_circ_	0.158001	0.415012
hsa_circ_	-0.17679	0.415017
hsa_circ_	0.122998	0.415017
hsa_circ_	-0.18409	0.415021
hsa_circ_	-0.38411	0.415045
hsa_circ_	0.141719	0.415055
hsa_circ_	-0.28771	0.415068
hsa_circ_	0.473928	0.41508
hsa_circ_	-0.38188	0.415103
hsa_circ_	-0.20672	0.415121
hsa_circ_	0.221003	0.415135
hsa_circ_	0.236758	0.415167
hsa_circ_	0.318931	0.415181
hsa_circ_	-0.26556	0.415182
hsa_circ_	0.279609	0.415183
hsa_circ_	-0.26089	0.415187
hsa_circ_	0.105182	0.415191
hsa_circ_	0.110014	0.415202

hsa_circ_	-0.15871	0.415228
hsa_circ_	-0.12652	0.415235
hsa_circ_	-0.10883	0.415236
hsa_circ_	0.752003	0.415257
hsa_circ_	0.399657	0.415259
hsa_circ_	-0.169	0.415296
hsa_circ_	0.190338	0.415302
hsa_circ_	0.253222	0.415304
hsa_circ_	-0.62071	0.415311
hsa_circ_	0.107984	0.415318
hsa_circ_	0.216793	0.415319
hsa_circ_	-0.11505	0.415337
hsa_circ_	0.106363	0.415345
hsa_circ_	0.641952	0.41536
hsa_circ_	-0.50308	0.415368
hsa_circ_	0.152939	0.415368
hsa_circ_	-0.13979	0.415387
hsa_circ_	-0.40335	0.415396
hsa_circ_	-0.13737	0.415408
hsa_circ_	-0.21377	0.41541
hsa_circ_	0.370744	0.415438
hsa_circ_	-0.12077	0.415458
hsa_circ_	-0.11276	0.415485
hsa_circ_	0.194756	0.415496
hsa_circ_	-0.55231	0.415505
hsa_circ_	0.163416	0.415519
hsa_circ_	-0.12699	0.415548
hsa_circ_	-0.2312	0.415555
hsa_circ_	0.137381	0.415561
hsa_circ_	0.128234	0.415563
hsa_circ_	-0.21454	0.415571
hsa_circ_	0.131365	0.415581
hsa_circ_	0.471036	0.41562
hsa_circ_	-0.13138	0.415643
hsa_circ_	-0.32021	0.415661
hsa_circ_	-0.16652	0.415687
hsa_circ_	0.200383	0.41569
hsa_circ_	0.357446	0.415693
hsa_circ_	-0.13554	0.415703
hsa_circ_	-0.30329	0.415734
hsa_circ_	-0.18492	0.415735
hsa_circ_	0.106609	0.415747
hsa_circ_	0.129143	0.415748
hsa_circ_	-0.11679	0.415749
hsa_circ_	0.384652	0.415786
hsa_circ_	-0.76678	0.415792
hsa_circ_	0.117241	0.415794
hsa_circ_	-0.1478	0.41583
hsa_circ_	-0.20479	0.415841
hsa_circ_	-0.28136	0.415846
hsa_circ_	-0.12871	0.415864
hsa_circ_	0.502374	0.415875
hsa_circ_	-0.25396	0.415881
hsa_circ_	-0.18129	0.415892

hsa_circ_	-0.2439	0.415903
hsa_circ_	-0.13945	0.415904
hsa_circ_	-0.39906	0.415915
hsa_circ_	-0.12308	0.415957
hsa_circ_	-0.15952	0.415963
hsa_circ_	-0.15615	0.415963
hsa_circ_	-0.18899	0.415977
hsa_circ_	0.190571	0.415997
hsa_circ_	0.163031	0.415999
hsa_circ_	-0.19131	0.416014
hsa_circ_	-0.16505	0.416038
hsa_circ_	-0.14104	0.416055
hsa_circ_	-0.34126	0.416057
hsa_circ_	-0.24446	0.416066
hsa_circ_	0.14711	0.416087
hsa_circ_	-0.19451	0.41609
hsa_circ_	0.326607	0.416097
hsa_circ_	0.196884	0.416117
hsa_circ_	0.156291	0.416123
hsa_circ_	0.377823	0.416128
hsa_circ_	0.376779	0.416157
hsa_circ_	-0.22787	0.416183
hsa_circ_	0.15048	0.416189
hsa_circ_	-0.12241	0.416202
hsa_circ_	-0.42641	0.416206
hsa_circ_	-0.1537	0.416219
hsa_circ_	0.621755	0.416229
hsa_circ_	0.14216	0.416237
hsa_circ_	0.155849	0.416242
hsa_circ_	-0.20311	0.41627
hsa_circ_	0.136865	0.416273
hsa_circ_	-0.21021	0.416277
hsa_circ_	0.16625	0.416287
hsa_circ_	-0.17901	0.416331
hsa_circ_	-0.17505	0.416333
hsa_circ_	-0.15809	0.416337
hsa_circ_	0.269836	0.416383
hsa_circ_	0.155127	0.416385
hsa_circ_	0.371446	0.416416
hsa_circ_	-0.40091	0.416425
hsa_circ_	-0.21825	0.41643
hsa_circ_	-0.14227	0.416445
hsa_circ_	-0.16471	0.416447
hsa_circ_	0.114201	0.416453
hsa_circ_	-0.18017	0.416466
hsa_circ_	-0.15792	0.416468
hsa_circ_	-0.13584	0.41647
hsa_circ_	0.3156	0.416471
hsa_circ_	0.204479	0.416473
hsa_circ_	0.161778	0.416475
hsa_circ_	0.134944	0.416483
hsa_circ_	0.19505	0.416494
hsa_circ_	-0.15563	0.416496
hsa_circ_	0.237051	0.416518

hsa_circ_	0.15572	0.416539
hsa_circ_	0.14496	0.416558
hsa_circ_	0.110529	0.416564
hsa_circ_	0.168072	0.41657
hsa_circ_	0.189917	0.416582
hsa_circ_	-0.19525	0.416583
hsa_circ_	0.15708	0.416588
hsa_circ_	-0.18396	0.416596
hsa_circ_	0.160829	0.416606
hsa_circ_	-0.1246	0.416612
hsa_circ_	0.120515	0.416616
hsa_circ_	0.338892	0.416622
hsa_circ_	-0.16179	0.416631
hsa_circ_	-0.1225	0.416642
hsa_circ_	0.118004	0.416646
hsa_circ_	0.137361	0.416652
hsa_circ_	0.206414	0.416664
hsa_circ_	0.129728	0.416675
hsa_circ_	-0.15303	0.416689
hsa_circ_	-0.18995	0.416749
hsa_circ_	-0.29288	0.41676
hsa_circ_	0.280908	0.416776
hsa_circ_	0.164363	0.416816
hsa_circ_	-0.31146	0.416818
hsa_circ_	-0.21173	0.41682
hsa_circ_	0.174032	0.416824
hsa_circ_	0.249944	0.416828
hsa_circ_	0.249434	0.416842
hsa_circ_	-0.22131	0.416864
hsa_circ_	-0.3813	0.416866
hsa_circ_	0.243664	0.416889
hsa_circ_	-0.33452	0.416897
hsa_circ_	-0.33064	0.416899
hsa_circ_	0.431913	0.416906
hsa_circ_	-0.22972	0.416907
hsa_circ_	0.497053	0.416916
hsa_circ_	0.127268	0.416923
hsa_circ_	0.14605	0.416928
hsa_circ_	0.133216	0.416951
hsa_circ_	-0.14187	0.416969
hsa_circ_	0.354938	0.41701
hsa_circ_	0.135588	0.417011
hsa_circ_	0.160747	0.417014
hsa_circ_	0.291105	0.417034
hsa_circ_	0.156347	0.417072
hsa_circ_	0.175536	0.417076
hsa_circ_	0.222171	0.417093
hsa_circ_	-0.22928	0.417096
hsa_circ_	-0.12706	0.417099
hsa_circ_	-0.76787	0.417107
hsa_circ_	-0.15246	0.417109
hsa_circ_	-0.16857	0.417139
hsa_circ_	-0.57336	0.417145
hsa_circ_	-0.11766	0.417155

hsa_circ_	-0.28877	0.417156
hsa_circ_	-0.12596	0.417175
hsa_circ_	-0.82173	0.417185
hsa_circ_	0.136999	0.417194
hsa_circ_	0.15829	0.417216
hsa_circ_	0.14161	0.417217
hsa_circ_	-0.16261	0.417221
hsa_circ_	0.491034	0.417253
hsa_circ_	0.152747	0.417288
hsa_circ_	0.205201	0.41729
hsa_circ_	0.171602	0.417295
hsa_circ_	-0.22686	0.417304
hsa_circ_	-0.36923	0.417338
hsa_circ_	-0.1395	0.417363
hsa_circ_	0.165266	0.417364
hsa_circ_	0.196179	0.417365
hsa_circ_	0.344983	0.417373
hsa_circ_	-0.28544	0.417386
hsa_circ_	-0.22144	0.417408
hsa_circ_	-0.13956	0.417413
hsa_circ_	0.681732	0.417419
hsa_circ_	0.144054	0.417426
hsa_circ_	0.142036	0.417447
hsa_circ_	-0.56636	0.417468
hsa_circ_	0.289086	0.417496
hsa_circ_	-0.57849	0.417498
hsa_circ_	0.317861	0.417499
hsa_circ_	-0.14522	0.417511
hsa_circ_	-0.13828	0.417524
hsa_circ_	0.127251	0.417525
hsa_circ_	-0.22548	0.417531
hsa_circ_	-0.20042	0.417546
hsa_circ_	0.115176	0.417564
hsa_circ_	0.124493	0.417588
hsa_circ_	0.638563	0.417619
hsa_circ_	0.29904	0.417638
hsa_circ_	-0.6236	0.417653
hsa_circ_	-0.15735	0.417656
hsa_circ_	-0.11885	0.417674
hsa_circ_	-0.33787	0.41769
hsa_circ_	-0.21228	0.41773
hsa_circ_	0.13983	0.417732
hsa_circ_	0.166933	0.41775
hsa_circ_	0.120343	0.417756
hsa_circ_	0.276422	0.417771
hsa_circ_	0.34656	0.417786
hsa_circ_	-0.29684	0.417793
hsa_circ_	-0.12364	0.417803
hsa_circ_	0.17178	0.417816
hsa_circ_	0.129578	0.41782
hsa_circ_	0.576592	0.417855
hsa_circ_	-0.12345	0.417891
hsa_circ_	-0.11714	0.417911
hsa_circ_	0.122711	0.417932

hsa_circ_	0.295118	0.417974
hsa_circ_	-0.17029	0.418023
hsa_circ_	-0.27267	0.418037
hsa_circ_	0.42269	0.418041
hsa_circ_	-0.58651	0.418056
hsa_circ_	-0.16878	0.418071
hsa_circ_	0.206007	0.418073
hsa_circ_	-0.16199	0.418073
hsa_circ_	-0.24083	0.418086
hsa_circ_	0.22004	0.418108
hsa_circ_	-0.10487	0.418145
hsa_circ_	-0.42615	0.41816
hsa_circ_	0.276813	0.418167
hsa_circ_	-0.49951	0.418174
hsa_circ_	-0.15957	0.418177
hsa_circ_	-0.11294	0.418177
hsa_circ_	0.134673	0.418197
hsa_circ_	-0.12212	0.418219
hsa_circ_	0.135637	0.418221
hsa_circ_	-0.13474	0.418243
hsa_circ_	0.120829	0.41826
hsa_circ_	-0.20038	0.418276
hsa_circ_	-0.43671	0.418285
hsa_circ_	0.173521	0.418286
hsa_circ_	-0.12811	0.418286
hsa_circ_	0.412341	0.418297
hsa_circ_	-0.17472	0.418299
hsa_circ_	0.114971	0.418299
hsa_circ_	-0.23072	0.4183
hsa_circ_	-0.28255	0.418343
hsa_circ_	0.325356	0.418366
hsa_circ_	-0.3482	0.418369
hsa_circ_	-0.21194	0.41837
hsa_circ_	-0.20347	0.418372
hsa_circ_	0.309372	0.418377
hsa_circ_	-0.45705	0.418386
hsa_circ_	-0.34969	0.418389
hsa_circ_	-0.14709	0.418393
hsa_circ_	0.133513	0.418439
hsa_circ_	0.209711	0.418446
hsa_circ_	-0.38177	0.41846
hsa_circ_	0.200341	0.418467
hsa_circ_	-0.13708	0.418483
hsa_circ_	0.103896	0.418484
hsa_circ_	0.286459	0.418484
hsa_circ_	0.190013	0.418513
hsa_circ_	-0.16933	0.41854
hsa_circ_	-0.14806	0.41855
hsa_circ_	0.138909	0.418561
hsa_circ_	-0.23056	0.41858
hsa_circ_	-0.20076	0.418605
hsa_circ_	0.188056	0.418607
hsa_circ_	0.254545	0.418608
hsa_circ_	-0.19196	0.418638

hsa_circ_	-0.12016	0.418666
hsa_circ_	0.217519	0.418671
hsa_circ_	-0.46334	0.41869
hsa_circ_	0.122865	0.418709
hsa_circ_	-0.25212	0.418733
hsa_circ_	-0.12905	0.418736
hsa_circ_	-0.13966	0.418736
hsa_circ_	-0.13508	0.418743
hsa_circ_	-0.12627	0.418747
hsa_circ_	0.184336	0.418773
hsa_circ_	-0.53309	0.418781
hsa_circ_	0.151229	0.418791
hsa_circ_	-0.22696	0.4188
hsa_circ_	0.186854	0.418808
hsa_circ_	0.166248	0.418826
hsa_circ_	0.193781	0.418834
hsa_circ_	-0.11927	0.418838
hsa_circ_	-0.19936	0.418859
hsa_circ_	-0.22967	0.418912
hsa_circ_	-0.22289	0.418925
hsa_circ_	0.120717	0.418943
hsa_circ_	0.149877	0.418954
hsa_circ_	-0.1541	0.418977
hsa_circ_	-0.19866	0.418979
hsa_circ_	-0.11501	0.418989
hsa_circ_	0.130532	0.419
hsa_circ_	-0.25139	0.419
hsa_circ_	-0.19329	0.419028
hsa_circ_	0.13642	0.419029
hsa_circ_	0.310933	0.41906
hsa_circ_	0.171577	0.419067
hsa_circ_	0.242535	0.41909
hsa_circ_	0.142623	0.419098
hsa_circ_	-0.17166	0.41911
hsa_circ_	-1.74174	0.41915
hsa_circ_	0.529686	0.419221
hsa_circ_	0.142977	0.419236
hsa_circ_	0.115397	0.419238
hsa_circ_	0.227438	0.419238
hsa_circ_	0.19981	0.419243
hsa_circ_	-0.11813	0.419243
hsa_circ_	-0.12668	0.419246
hsa_circ_	-0.13436	0.419258
hsa_circ_	-0.14098	0.419261
hsa_circ_	-0.13995	0.419291
hsa_circ_	0.192809	0.419295
hsa_circ_	0.226994	0.419311
hsa_circ_	0.1776	0.419313
hsa_circ_	0.125346	0.419335
hsa_circ_	-0.12176	0.419338
hsa_circ_	-0.19282	0.419345
hsa_circ_	0.165148	0.419378
hsa_circ_	-0.41872	0.419382
hsa_circ_	0.172132	0.419402

hsa_circ_	0.173919	0.41942
hsa_circ_	-0.15106	0.41942
hsa_circ_	-0.30615	0.419433
hsa_circ_	0.115078	0.419462
hsa_circ_	-0.38945	0.419471
hsa_circ_	-0.10822	0.419493
hsa_circ_	0.20078	0.419503
hsa_circ_	0.145663	0.419523
hsa_circ_	-0.12177	0.419523
hsa_circ_	0.149343	0.419531
hsa_circ_	0.140767	0.419552
hsa_circ_	-0.19111	0.419556
hsa_circ_	0.170514	0.419585
hsa_circ_	0.254812	0.419593
hsa_circ_	-0.15461	0.419594
hsa_circ_	-0.11027	0.419599
hsa_circ_	0.148008	0.41962
hsa_circ_	0.300122	0.419639
hsa_circ_	0.207044	0.41966
hsa_circ_	-0.18225	0.419677
hsa_circ_	-0.21333	0.419682
hsa_circ_	-0.11199	0.419692
hsa_circ_	0.18619	0.419712
hsa_circ_	0.124247	0.419747
hsa_circ_	0.13883	0.419765
hsa_circ_	0.264591	0.419775
hsa_circ_	-0.25621	0.419788
hsa_circ_	-0.14456	0.419788
hsa_circ_	0.118612	0.419789
hsa_circ_	-0.17052	0.419804
hsa_circ_	0.155035	0.419809
hsa_circ_	-0.1332	0.419826
hsa_circ_	-0.11026	0.419836
hsa_circ_	-0.1165	0.419866
hsa_circ_	-0.19989	0.419869
hsa_circ_	0.398652	0.419875
hsa_circ_	0.176999	0.419927
hsa_circ_	-0.13335	0.419935
hsa_circ_	0.143326	0.41994
hsa_circ_	-0.2803	0.419946
hsa_circ_	-0.13555	0.41996
hsa_circ_	-0.24899	0.419962
hsa_circ_	0.128607	0.419975
hsa_circ_	0.113624	0.419983
hsa_circ_	0.527286	0.419992
hsa_circ_	-0.29241	0.420003
hsa_circ_	0.138813	0.420003
hsa_circ_	0.554892	0.420057
hsa_circ_	0.263697	0.420065
hsa_circ_	-0.43732	0.420107
hsa_circ_	-0.47819	0.420124
hsa_circ_	-0.15981	0.420126
hsa_circ_	-0.11682	0.420148
hsa_circ_	-0.16663	0.42018

hsa_circ_	0.112357	0.42018
hsa_circ_	0.26737	0.420213
hsa_circ_	0.338389	0.420221
hsa_circ_	0.1879	0.420232
hsa_circ_	-0.21965	0.420258
hsa_circ_	0.162132	0.420292
hsa_circ_	0.193197	0.420299
hsa_circ_	-0.23186	0.420305
hsa_circ_	0.12454	0.420314
hsa_circ_	-0.16164	0.420318
hsa_circ_	-0.10631	0.420326
hsa_circ_	-0.33941	0.420336
hsa_circ_	-0.34648	0.420342
hsa_circ_	0.478671	0.420342
hsa_circ_	-0.20906	0.420363
hsa_circ_	-0.11801	0.420367
hsa_circ_	0.18272	0.420375
hsa_circ_	-0.15707	0.420387
hsa_circ_	0.129283	0.420409
hsa_circ_	-0.15185	0.420421
hsa_circ_	0.113487	0.420422
hsa_circ_	0.251316	0.420446
hsa_circ_	0.197682	0.420448
hsa_circ_	0.563765	0.420448
hsa_circ_	0.107449	0.420453
hsa_circ_	-0.17915	0.420457
hsa_circ_	-0.16475	0.4205
hsa_circ_	-0.21925	0.420523
hsa_circ_	-0.36326	0.420525
hsa_circ_	-0.1293	0.420568
hsa_circ_	0.229981	0.420568
hsa_circ_	0.104844	0.420575
hsa_circ_	-0.10756	0.420584
hsa_circ_	0.191383	0.420593
hsa_circ_	-0.5613	0.420598
hsa_circ_	-0.1283	0.420603
hsa_circ_	0.155118	0.420603
hsa_circ_	-0.21561	0.420625
hsa_circ_	-0.11382	0.420636
hsa_circ_	0.216514	0.420652
hsa_circ_	0.422868	0.42066
hsa_circ_	0.179449	0.420667
hsa_circ_	-0.1614	0.420689
hsa_circ_	0.132108	0.420718
hsa_circ_	0.304819	0.420721
hsa_circ_	-0.1306	0.420735
hsa_circ_	-0.12026	0.420749
hsa_circ_	-0.32281	0.420749
hsa_circ_	0.11262	0.420756
hsa_circ_	-0.20201	0.42078
hsa_circ_	-0.13895	0.420799
hsa_circ_	-0.20513	0.420803
hsa_circ_	-0.15688	0.420818
hsa_circ_	-0.12601	0.420822

hsa_circ_	0.253569	0.420843
hsa_circ_	-0.18675	0.420873
hsa_circ_	-0.11498	0.420877
hsa_circ_	-0.12104	0.420894
hsa_circ_	0.231768	0.420915
hsa_circ_	0.252858	0.420933
hsa_circ_	0.209957	0.420936
hsa_circ_	0.123432	0.420951
hsa_circ_	-0.11999	0.420961
hsa_circ_	-1.0198	0.420983
hsa_circ_	-0.139	0.420984
hsa_circ_	0.175464	0.420988
hsa_circ_	-0.21551	0.420997
hsa_circ_	0.491279	0.421021
hsa_circ_	0.274967	0.421083
hsa_circ_	-0.12198	0.421097
hsa_circ_	0.237418	0.421106
hsa_circ_	-0.28911	0.421111
hsa_circ_	-0.55613	0.421147
hsa_circ_	-0.51612	0.421153
hsa_circ_	0.148022	0.421195
hsa_circ_	0.243081	0.421212
hsa_circ_	-0.29122	0.421237
hsa_circ_	-0.1327	0.42124
hsa_circ_	-0.46515	0.421256
hsa_circ_	-0.12481	0.421276
hsa_circ_	-0.26592	0.42128
hsa_circ_	0.383679	0.421298
hsa_circ_	-0.18651	0.421299
hsa_circ_	-0.11472	0.421415
hsa_circ_	-0.14875	0.421415
hsa_circ_	0.171286	0.421417
hsa_circ_	0.265872	0.421417
hsa_circ_	-0.19875	0.421421
hsa_circ_	0.426739	0.421427
hsa_circ_	-0.50879	0.421429
hsa_circ_	-0.32271	0.421437
hsa_circ_	-0.10986	0.421454
hsa_circ_	0.156064	0.421481
hsa_circ_	-0.1528	0.421493
hsa_circ_	-0.12148	0.421494
hsa_circ_	-0.28673	0.421496
hsa_circ_	0.189252	0.421509
hsa_circ_	-0.2755	0.421535
hsa_circ_	-0.31534	0.421538
hsa_circ_	-0.15081	0.421565
hsa_circ_	-0.21945	0.421566
hsa_circ_	0.398685	0.421592
hsa_circ_	-0.16314	0.421599
hsa_circ_	0.14188	0.421613
hsa_circ_	0.149639	0.421613
hsa_circ_	0.121599	0.421621
hsa_circ_	0.238238	0.421623
hsa_circ_	0.46959	0.421624

hsa_circ_	0.138824	0.421633
hsa_circ_	0.146613	0.421648
hsa_circ_	0.393769	0.421649
hsa_circ_	-0.15491	0.421654
hsa_circ_	-0.28558	0.42168
hsa_circ_	0.12651	0.421683
hsa_circ_	-0.16546	0.421684
hsa_circ_	0.19529	0.421702
hsa_circ_	-0.13479	0.421711
hsa_circ_	-0.17818	0.421724
hsa_circ_	0.112376	0.42175
hsa_circ_	-0.14564	0.421762
hsa_circ_	-0.11701	0.421766
hsa_circ_	0.180551	0.421771
hsa_circ_	-0.44066	0.421777
hsa_circ_	0.165244	0.421825
hsa_circ_	-0.11527	0.421838
hsa_circ_	0.161679	0.421845
hsa_circ_	-0.26023	0.421848
hsa_circ_	-0.35246	0.42186
hsa_circ_	-0.26165	0.421886
hsa_circ_	-0.23101	0.421901
hsa_circ_	0.180661	0.421915
hsa_circ_	0.131269	0.421933
hsa_circ_	0.547718	0.421945
hsa_circ_	-0.15342	0.421946
hsa_circ_	0.129493	0.421948
hsa_circ_	-0.13656	0.421949
hsa_circ_	0.146582	0.42195
hsa_circ_	-0.33712	0.421969
hsa_circ_	0.145022	0.421973
hsa_circ_	0.18063	0.422
hsa_circ_	0.104791	0.422005
hsa_circ_	-0.10923	0.422008
hsa_circ_	-1.11924	0.422023
hsa_circ_	-0.40507	0.42203
hsa_circ_	-0.10345	0.422038
hsa_circ_	-0.30428	0.422042
hsa_circ_	-0.14467	0.422044
hsa_circ_	-0.2351	0.422045
hsa_circ_	0.118309	0.422048
hsa_circ_	0.123743	0.422063
hsa_circ_	-0.13489	0.422075
hsa_circ_	0.403423	0.422131
hsa_circ_	-0.1237	0.422161
hsa_circ_	0.207447	0.422171
hsa_circ_	-0.62261	0.422174
hsa_circ_	0.144925	0.422192
hsa_circ_	0.298352	0.422213
hsa_circ_	-0.21705	0.422228
hsa_circ_	-0.24877	0.422231
hsa_circ_	-0.11753	0.422261
hsa_circ_	-0.28611	0.422271
hsa_circ_	-0.21893	0.422298

hsa_circ_	0.124881	0.42231
hsa_circ_	0.132849	0.42231
hsa_circ_	-0.20549	0.422314
hsa_circ_	-0.11993	0.422339
hsa_circ_	-0.45368	0.422349
hsa_circ_	0.292982	0.422386
hsa_circ_	0.167024	0.422402
hsa_circ_	0.592234	0.422413
hsa_circ_	0.119801	0.422422
hsa_circ_	-0.16884	0.422435
hsa_circ_	-0.15636	0.422444
hsa_circ_	-0.42683	0.422445
hsa_circ_	-0.19983	0.422457
hsa_circ_	-0.13251	0.42246
hsa_circ_	0.167091	0.42247
hsa_circ_	0.230337	0.422487
hsa_circ_	0.105438	0.422521
hsa_circ_	0.124538	0.422533
hsa_circ_	-0.25672	0.422533
hsa_circ_	0.426666	0.422534
hsa_circ_	0.144762	0.422536
hsa_circ_	-0.33062	0.422555
hsa_circ_	-0.11005	0.422559
hsa_circ_	-0.11444	0.422562
hsa_circ_	-0.20219	0.42258
hsa_circ_	-0.32342	0.422598
hsa_circ_	-0.18567	0.422599
hsa_circ_	-0.14222	0.422658
hsa_circ_	0.141611	0.422669
hsa_circ_	0.243439	0.422701
hsa_circ_	0.178621	0.422705
hsa_circ_	0.20477	0.422724
hsa_circ_	-0.72446	0.42278
hsa_circ_	-1.00365	0.422791
hsa_circ_	0.426453	0.422791
hsa_circ_	0.116472	0.422828
hsa_circ_	-0.11823	0.422833
hsa_circ_	0.233188	0.422836
hsa_circ_	0.282876	0.422867
hsa_circ_	0.190408	0.422872
hsa_circ_	-0.12992	0.422882
hsa_circ_	-0.11149	0.422883
hsa_circ_	0.364634	0.4229
hsa_circ_	-0.14536	0.422919
hsa_circ_	-0.17076	0.422936
hsa_circ_	0.11573	0.422948
hsa_circ_	0.280466	0.42296
hsa_circ_	-0.18416	0.422965
hsa_circ_	-0.11246	0.422966
hsa_circ_	-0.21547	0.422977
hsa_circ_	-0.11053	0.422978
hsa_circ_	0.127796	0.422991
hsa_circ_	-0.12918	0.422995
hsa_circ_	0.183442	0.422998

hsa_circ_	-0.15566	0.423008
hsa_circ_	-0.59491	0.423022
hsa_circ_	0.34486	0.423044
hsa_circ_	0.233878	0.423053
hsa_circ_	0.322385	0.423054
hsa_circ_	-0.12361	0.423058
hsa_circ_	-0.11023	0.42306
hsa_circ_	-0.11079	0.423073
hsa_circ_	0.184188	0.423076
hsa_circ_	-0.19072	0.423084
hsa_circ_	-0.32483	0.423124
hsa_circ_	-0.15257	0.423127
hsa_circ_	0.141065	0.423131
hsa_circ_	0.111578	0.42314
hsa_circ_	0.26786	0.423148
hsa_circ_	0.161928	0.423148
hsa_circ_	0.111391	0.423153
hsa_circ_	-0.12806	0.423158
hsa_circ_	-0.35831	0.423169
hsa_circ_	0.181642	0.42319
hsa_circ_	-0.12863	0.42319
hsa_circ_	0.202316	0.423204
hsa_circ_	-0.31914	0.423241
hsa_circ_	0.112494	0.423252
hsa_circ_	-0.1821	0.423264
hsa_circ_	0.166641	0.423271
hsa_circ_	0.10773	0.423277
hsa_circ_	0.116343	0.423279
hsa_circ_	0.315921	0.423287
hsa_circ_	0.253173	0.423294
hsa_circ_	-0.17786	0.423324
hsa_circ_	-0.13811	0.423329
hsa_circ_	-0.51013	0.423336
hsa_circ_	-0.1871	0.423339
hsa_circ_	-0.11592	0.423343
hsa_circ_	-0.32588	0.423372
hsa_circ_	0.637949	0.423376
hsa_circ_	0.111014	0.423382
hsa_circ_	0.294922	0.423404
hsa_circ_	-0.11859	0.423413
hsa_circ_	0.202573	0.423416
hsa_circ_	-0.16311	0.423422
hsa_circ_	-0.10865	0.423432
hsa_circ_	-0.131	0.423453
hsa_circ_	-0.14478	0.423464
hsa_circ_	-0.12957	0.423473
hsa_circ_	-0.14147	0.423474
hsa_circ_	-0.22268	0.423488
hsa_circ_	0.148016	0.423496
hsa_circ_	0.174596	0.423496
hsa_circ_	0.145633	0.423501
hsa_circ_	-0.11862	0.423528
hsa_circ_	0.363795	0.423552
hsa_circ_	-0.12074	0.423558

hsa_circ_	0.192473	0.423585
hsa_circ_	0.153575	0.423604
hsa_circ_	-0.14378	0.423633
hsa_circ_	-0.26634	0.423635
hsa_circ_	-0.1155	0.423651
hsa_circ_	0.223193	0.42368
hsa_circ_	-0.13405	0.423706
hsa_circ_	0.494773	0.423712
hsa_circ_	0.283619	0.423718
hsa_circ_	-0.16882	0.423729
hsa_circ_	0.128187	0.423751
hsa_circ_	-0.18189	0.423756
hsa_circ_	-0.62457	0.423771
hsa_circ_	-0.17541	0.423771
hsa_circ_	-0.26765	0.423784
hsa_circ_	-0.10511	0.4238
hsa_circ_	-0.15394	0.423848
hsa_circ_	-0.13231	0.423879
hsa_circ_	-0.83204	0.423883
hsa_circ_	-0.18047	0.423896
hsa_circ_	0.18981	0.423929
hsa_circ_	0.124599	0.42393
hsa_circ_	-0.1585	0.42393
hsa_circ_	-0.16821	0.423938
hsa_circ_	0.129955	0.423948
hsa_circ_	-0.44337	0.423952
hsa_circ_	-0.31145	0.423968
hsa_circ_	-0.13384	0.423972
hsa_circ_	-0.1998	0.423987
hsa_circ_	-0.15815	0.423987
hsa_circ_	0.181977	0.423989
hsa_circ_	0.284932	0.423997
hsa_circ_	0.168963	0.424002
hsa_circ_	-0.23062	0.424003
hsa_circ_	-0.22311	0.424005
hsa_circ_	0.175545	0.424024
hsa_circ_	0.248353	0.424026
hsa_circ_	-0.1202	0.424028
hsa_circ_	0.372259	0.424048
hsa_circ_	-0.22088	0.424084
hsa_circ_	-0.13023	0.424108
hsa_circ_	-0.16535	0.424109
hsa_circ_	-0.21872	0.424118
hsa_circ_	-0.18253	0.424145
hsa_circ_	-0.1948	0.424155
hsa_circ_	0.144627	0.424183
hsa_circ_	0.21273	0.424204
hsa_circ_	-0.22423	0.424205
hsa_circ_	-0.19093	0.424212
hsa_circ_	0.148231	0.424237
hsa_circ_	0.636979	0.424247
hsa_circ_	0.130654	0.424256
hsa_circ_	0.514773	0.424257
hsa_circ_	0.455449	0.424261

hsa_circ_	0.157967	0.424261
hsa_circ_	-0.19571	0.42428
hsa_circ_	0.265534	0.424287
hsa_circ_	-0.15301	0.424298
hsa_circ_	-0.15627	0.424375
hsa_circ_	0.229118	0.424377
hsa_circ_	0.275298	0.424406
hsa_circ_	-0.18752	0.424414
hsa_circ_	0.148944	0.424441
hsa_circ_	-0.19144	0.424446
hsa_circ_	0.126107	0.42445
hsa_circ_	-0.12774	0.42445
hsa_circ_	-0.12134	0.424466
hsa_circ_	-0.14243	0.424471
hsa_circ_	0.109986	0.424495
hsa_circ_	0.212524	0.424525
hsa_circ_	-0.2882	0.424533
hsa_circ_	-0.45191	0.424536
hsa_circ_	0.192263	0.424537
hsa_circ_	-0.13364	0.424546
hsa_circ_	0.227964	0.424556
hsa_circ_	-0.19947	0.424586
hsa_circ_	0.139222	0.42459
hsa_circ_	-0.24107	0.424599
hsa_circ_	0.174587	0.424617
hsa_circ_	-0.13542	0.424636
hsa_circ_	-0.13659	0.424655
hsa_circ_	-0.13196	0.424667
hsa_circ_	0.606991	0.424711
hsa_circ_	0.287924	0.424713
hsa_circ_	0.304398	0.424716
hsa_circ_	-0.19088	0.424757
hsa_circ_	0.153602	0.424765
hsa_circ_	-0.18608	0.424772
hsa_circ_	-0.60391	0.424792
hsa_circ_	0.260098	0.424801
hsa_circ_	-0.11697	0.424806
hsa_circ_	-0.16981	0.424828
hsa_circ_	0.147808	0.424844
hsa_circ_	-0.11257	0.424861
hsa_circ_	0.456338	0.42487
hsa_circ_	0.11042	0.424879
hsa_circ_	0.123301	0.424888
hsa_circ_	-0.11995	0.424894
hsa_circ_	0.178896	0.424954
hsa_circ_	0.138986	0.424996
hsa_circ_	-0.19433	0.425
hsa_circ_	-0.32015	0.425005
hsa_circ_	0.27176	0.425008
hsa_circ_	-0.22336	0.425016
hsa_circ_	-0.11526	0.425024
hsa_circ_	0.271109	0.425039
hsa_circ_	-0.15464	0.425046
hsa_circ_	-0.47035	0.425049

hsa_circ_	0.147338	0.42507
hsa_circ_	-0.21568	0.425093
hsa_circ_	0.161371	0.425097
hsa_circ_	-0.20444	0.425098
hsa_circ_	-0.23951	0.425103
hsa_circ_	0.303657	0.425131
hsa_circ_	-0.17761	0.42514
hsa_circ_	-0.2145	0.425145
hsa_circ_	0.137003	0.425155
hsa_circ_	-0.37511	0.425173
hsa_circ_	-0.1488	0.425186
hsa_circ_	-0.17172	0.425229
hsa_circ_	-0.16134	0.425229
hsa_circ_	-0.15989	0.425235
hsa_circ_	0.119462	0.425293
hsa_circ_	-0.16711	0.425296
hsa_circ_	-0.12602	0.425301
hsa_circ_	-0.26153	0.425324
hsa_circ_	-0.20231	0.42534
hsa_circ_	0.253981	0.425342
hsa_circ_	0.229958	0.425342
hsa_circ_	0.132473	0.425353
hsa_circ_	-0.13819	0.425355
hsa_circ_	-0.13094	0.425361
hsa_circ_	0.143272	0.425364
hsa_circ_	-0.18486	0.42539
hsa_circ_	-0.18975	0.425421
hsa_circ_	0.18888	0.425429
hsa_circ_	0.11244	0.425433
hsa_circ_	-0.14839	0.425458
hsa_circ_	-0.16185	0.425477
hsa_circ_	0.171364	0.425479
hsa_circ_	-0.23315	0.425494
hsa_circ_	-0.21189	0.425534
hsa_circ_	0.419278	0.425539
hsa_circ_	0.257595	0.425549
hsa_circ_	0.375383	0.425572
hsa_circ_	0.15624	0.425579
hsa_circ_	-0.12168	0.425595
hsa_circ_	0.556835	0.425603
hsa_circ_	0.301142	0.42561
hsa_circ_	0.363748	0.425626
hsa_circ_	0.434385	0.425631
hsa_circ_	0.144642	0.425648
hsa_circ_	0.138683	0.425656
hsa_circ_	-0.3098	0.425665
hsa_circ_	-0.22788	0.425669
hsa_circ_	-0.13563	0.425669
hsa_circ_	0.161334	0.425673
hsa_circ_	-0.24667	0.425681
hsa_circ_	-0.26452	0.425687
hsa_circ_	0.123453	0.425687
hsa_circ_	0.503188	0.425707
hsa_circ_	-0.11997	0.425707

hsa_circ_	-0.24486	0.425711
hsa_circ_	-0.41165	0.425738
hsa_circ_	-0.14664	0.42574
hsa_circ_	-0.26169	0.425768
hsa_circ_	-0.18188	0.425791
hsa_circ_	-0.18481	0.425807
hsa_circ_	0.160631	0.425807
hsa_circ_	0.156481	0.425812
hsa_circ_	-0.37971	0.425813
hsa_circ_	-0.41876	0.425821
hsa_circ_	0.48758	0.425829
hsa_circ_	-0.12878	0.425832
hsa_circ_	-0.30647	0.425845
hsa_circ_	0.171036	0.425859
hsa_circ_	-0.22689	0.425862
hsa_circ_	0.130077	0.425866
hsa_circ_	-0.43388	0.425878
hsa_circ_	0.332426	0.425892
hsa_circ_	0.229054	0.425907
hsa_circ_	0.410214	0.425923
hsa_circ_	0.15464	0.425924
hsa_circ_	-0.2134	0.425929
hsa_circ_	-0.23724	0.425958
hsa_circ_	-0.151	0.425963
hsa_circ_	0.4355	0.425967
hsa_circ_	0.227212	0.425978
hsa_circ_	0.236968	0.425995
hsa_circ_	0.132866	0.426004
hsa_circ_	-0.13732	0.426018
hsa_circ_	0.172873	0.426054
hsa_circ_	-0.16715	0.426087
hsa_circ_	0.183056	0.426087
hsa_circ_	0.220338	0.426099
hsa_circ_	-0.29749	0.426107
hsa_circ_	0.145667	0.426131
hsa_circ_	0.230602	0.426168
hsa_circ_	0.596893	0.426181
hsa_circ_	-0.21159	0.426188
hsa_circ_	0.176637	0.426205
hsa_circ_	-0.33122	0.426209
hsa_circ_	0.185973	0.426259
hsa_circ_	-1.85179	0.426265
hsa_circ_	0.149274	0.426287
hsa_circ_	0.637276	0.426302
hsa_circ_	0.13126	0.426311
hsa_circ_	-0.11034	0.426316
hsa_circ_	0.146764	0.426319
hsa_circ_	-0.61206	0.426324
hsa_circ_	-0.13632	0.426344
hsa_circ_	0.192595	0.426344
hsa_circ_	-0.12533	0.426354
hsa_circ_	0.289368	0.426359
hsa_circ_	-0.14522	0.426361
hsa_circ_	0.306862	0.426371

hsa_circ_	0.140962	0.426374
hsa_circ_	-0.20794	0.426377
hsa_circ_	-0.28518	0.426377
hsa_circ_	-0.56089	0.426382
hsa_circ_	-0.14223	0.426415
hsa_circ_	0.182768	0.42642
hsa_circ_	0.233345	0.426425
hsa_circ_	0.117311	0.426441
hsa_circ_	-0.19329	0.426444
hsa_circ_	0.469419	0.426454
hsa_circ_	-0.22245	0.426486
hsa_circ_	0.115472	0.426547
hsa_circ_	0.125649	0.42655
hsa_circ_	-0.17895	0.426566
hsa_circ_	0.161279	0.426602
hsa_circ_	-0.16732	0.426636
hsa_circ_	0.159751	0.426646
hsa_circ_	0.268767	0.426648
hsa_circ_	0.247364	0.426653
hsa_circ_	-0.19732	0.42666
hsa_circ_	0.146585	0.426664
hsa_circ_	-0.13905	0.426689
hsa_circ_	0.498978	0.426693
hsa_circ_	0.149151	0.426694
hsa_circ_	0.365944	0.42672
hsa_circ_	-0.36181	0.426727
hsa_circ_	-0.13837	0.426741
hsa_circ_	-0.384	0.426747
hsa_circ_	0.11841	0.426759
hsa_circ_	-0.35959	0.426764
hsa_circ_	-0.25794	0.426767
hsa_circ_	0.131358	0.426772
hsa_circ_	-0.19298	0.426797
hsa_circ_	0.158518	0.426803
hsa_circ_	0.370189	0.426806
hsa_circ_	-0.12107	0.426817
hsa_circ_	0.205193	0.426824
hsa_circ_	0.131417	0.426857
hsa_circ_	0.331646	0.426868
hsa_circ_	-0.27817	0.426894
hsa_circ_	-0.61588	0.426907
hsa_circ_	-0.16865	0.426907
hsa_circ_	0.120372	0.426911
hsa_circ_	-0.27419	0.426917
hsa_circ_	-0.96449	0.426919
hsa_circ_	-0.32627	0.426942
hsa_circ_	-0.15322	0.426948
hsa_circ_	-0.189	0.426981
hsa_circ_	-0.13036	0.426981
hsa_circ_	-0.15337	0.426994
hsa_circ_	-0.11476	0.427001
hsa_circ_	-0.34761	0.427004
hsa_circ_	-0.26366	0.427016
hsa_circ_	0.363166	0.427025

hsa_circ_	0.124331	0.427062
hsa_circ_	-0.54741	0.427067
hsa_circ_	0.132833	0.427071
hsa_circ_	-0.12539	0.427077
hsa_circ_	-0.12858	0.427083
hsa_circ_	-0.1569	0.427091
hsa_circ_	-0.95252	0.427101
hsa_circ_	-0.10821	0.427118
hsa_circ_	-0.41286	0.427141
hsa_circ_	-0.15088	0.427159
hsa_circ_	0.147474	0.427179
hsa_circ_	-0.12677	0.427187
hsa_circ_	-0.48704	0.427239
hsa_circ_	0.21728	0.427258
hsa_circ_	0.616656	0.42727
hsa_circ_	-0.34928	0.427304
hsa_circ_	0.165218	0.42731
hsa_circ_	0.116679	0.427318
hsa_circ_	-0.1482	0.427321
hsa_circ_	0.318873	0.427377
hsa_circ_	0.206327	0.427383
hsa_circ_	0.132805	0.427407
hsa_circ_	-0.12825	0.427413
hsa_circ_	-0.11226	0.427421
hsa_circ_	-0.41315	0.427422
hsa_circ_	0.173012	0.427424
hsa_circ_	-0.3082	0.427424
hsa_circ_	0.158544	0.427426
hsa_circ_	-0.16182	0.427456
hsa_circ_	-0.22188	0.427458
hsa_circ_	-0.12954	0.427467
hsa_circ_	0.221001	0.427475
hsa_circ_	-0.20476	0.427475
hsa_circ_	-0.41463	0.427488
hsa_circ_	-0.12559	0.427498
hsa_circ_	0.141084	0.427528
hsa_circ_	0.186306	0.427534
hsa_circ_	0.147054	0.427538
hsa_circ_	0.292946	0.427544
hsa_circ_	0.192158	0.427553
hsa_circ_	0.110088	0.427555
hsa_circ_	-0.22841	0.427558
hsa_circ_	0.26476	0.427566
hsa_circ_	-0.10401	0.427574
hsa_circ_	-0.18651	0.427589
hsa_circ_	-0.17416	0.427602
hsa_circ_	0.162602	0.427605
hsa_circ_	-0.65497	0.427612
hsa_circ_	-0.178	0.42762
hsa_circ_	-0.16238	0.427629
hsa_circ_	0.207387	0.427646
hsa_circ_	0.178529	0.427649
hsa_circ_	0.16294	0.427679
hsa_circ_	0.480991	0.427685

hsa_circ_	0.202169	0.427692
hsa_circ_	0.180252	0.427702
hsa_circ_	-0.22192	0.427732
hsa_circ_	0.219771	0.427833
hsa_circ_	0.328869	0.427844
hsa_circ_	0.426277	0.427851
hsa_circ_	0.159575	0.427859
hsa_circ_	-0.1307	0.427863
hsa_circ_	-0.21077	0.427888
hsa_circ_	0.146107	0.427897
hsa_circ_	-0.262	0.427909
hsa_circ_	-0.41369	0.42791
hsa_circ_	0.202757	0.427983
hsa_circ_	0.307554	0.427984
hsa_circ_	0.268337	0.427999
hsa_circ_	0.200404	0.428026
hsa_circ_	0.171318	0.428036
hsa_circ_	0.131736	0.428049
hsa_circ_	0.150768	0.428065
hsa_circ_	0.204673	0.428081
hsa_circ_	-0.11955	0.428131
hsa_circ_	0.165317	0.428139
hsa_circ_	0.138202	0.428143
hsa_circ_	0.41474	0.42816
hsa_circ_	0.183068	0.428163
hsa_circ_	-0.18631	0.428169
hsa_circ_	-0.11737	0.428174
hsa_circ_	-0.16687	0.428191
hsa_circ_	-0.24626	0.428195
hsa_circ_	0.172696	0.428205
hsa_circ_	0.2019	0.428216
hsa_circ_	0.168052	0.428218
hsa_circ_	-0.24186	0.428221
hsa_circ_	-0.1838	0.428239
hsa_circ_	-0.16739	0.428244
hsa_circ_	0.347826	0.428258
hsa_circ_	-0.10863	0.428266
hsa_circ_	0.129845	0.428268
hsa_circ_	-0.11772	0.428273
hsa_circ_	-0.12564	0.428289
hsa_circ_	0.116701	0.428293
hsa_circ_	-0.26742	0.428304
hsa_circ_	-0.21442	0.428309
hsa_circ_	-0.16471	0.428323
hsa_circ_	-0.24741	0.428323
hsa_circ_	0.227977	0.428333
hsa_circ_	-0.15316	0.428349
hsa_circ_	-0.32718	0.428367
hsa_circ_	0.183147	0.428373
hsa_circ_	-0.16346	0.42838
hsa_circ_	-0.14188	0.4284
hsa_circ_	-0.42362	0.428411
hsa_circ_	0.176104	0.428425
hsa_circ_	0.206141	0.428425

hsa_circ_	-0.20132	0.428439
hsa_circ_	-0.22799	0.428461
hsa_circ_	-0.15109	0.428464
hsa_circ_	-0.13588	0.428511
hsa_circ_	0.174299	0.42852
hsa_circ_	-0.21899	0.428527
hsa_circ_	-0.13385	0.428535
hsa_circ_	-0.22444	0.428548
hsa_circ_	0.37721	0.428574
hsa_circ_	0.124006	0.428574
hsa_circ_	-0.13488	0.428583
hsa_circ_	-0.11231	0.428583
hsa_circ_	0.16874	0.4286
hsa_circ_	-2.27762	0.428605
hsa_circ_	-0.2099	0.42862
hsa_circ_	-0.17231	0.428641
hsa_circ_	0.164165	0.428645
hsa_circ_	-0.1472	0.428667
hsa_circ_	-0.39584	0.428697
hsa_circ_	-0.1327	0.428698
hsa_circ_	-0.18827	0.428699
hsa_circ_	-0.38767	0.428712
hsa_circ_	0.490549	0.428733
hsa_circ_	0.175224	0.428746
hsa_circ_	-0.34616	0.428759
hsa_circ_	-0.69131	0.42877
hsa_circ_	-0.14997	0.42877
hsa_circ_	0.11072	0.428784
hsa_circ_	-0.22225	0.428789
hsa_circ_	0.131674	0.428794
hsa_circ_	0.235988	0.428796
hsa_circ_	-0.13966	0.428798
hsa_circ_	-0.10582	0.428801
hsa_circ_	-0.32078	0.428808
hsa_circ_	-0.22243	0.428809
hsa_circ_	0.144831	0.428809
hsa_circ_	-0.4656	0.428816
hsa_circ_	0.375426	0.42882
hsa_circ_	0.357318	0.428827
hsa_circ_	-0.37311	0.428833
hsa_circ_	-0.18009	0.428841
hsa_circ_	-0.12598	0.428853
hsa_circ_	-0.15841	0.428858
hsa_circ_	-0.14438	0.428865
hsa_circ_	0.405107	0.428868
hsa_circ_	0.192542	0.428885
hsa_circ_	0.19398	0.428895
hsa_circ_	0.167462	0.428928
hsa_circ_	0.623477	0.428929
hsa_circ_	0.426572	0.428933
hsa_circ_	-0.37962	0.428937
hsa_circ_	-0.19375	0.428956
hsa_circ_	0.134677	0.428961
hsa_circ_	-0.20823	0.429

hsa_circ_	-0.14003	0.429005
hsa_circ_	-0.16402	0.429049
hsa_circ_	0.156073	0.429054
hsa_circ_	-0.10744	0.429057
hsa_circ_	-0.18892	0.429113
hsa_circ_	-0.12356	0.429117
hsa_circ_	0.118105	0.429126
hsa_circ_	0.158996	0.429128
hsa_circ_	0.184718	0.429135
hsa_circ_	0.115995	0.429137
hsa_circ_	-0.20117	0.429139
hsa_circ_	0.12892	0.429155
hsa_circ_	-0.23287	0.42917
hsa_circ_	0.119655	0.42918
hsa_circ_	-0.109	0.429197
hsa_circ_	-0.31993	0.429213
hsa_circ_	0.263452	0.429217
hsa_circ_	-0.16337	0.429222
hsa_circ_	-0.15837	0.429234
hsa_circ_	-0.22056	0.42924
hsa_circ_	0.200835	0.429248
hsa_circ_	-0.20631	0.429263
hsa_circ_	0.197232	0.429281
hsa_circ_	0.27138	0.429299
hsa_circ_	-0.15571	0.429307
hsa_circ_	0.261529	0.429332
hsa_circ_	0.260081	0.42934
hsa_circ_	0.11218	0.429351
hsa_circ_	-0.27538	0.429353
hsa_circ_	0.224488	0.429353
hsa_circ_	-0.145	0.429358
hsa_circ_	-0.15356	0.429391
hsa_circ_	0.263553	0.429394
hsa_circ_	0.130634	0.429399
hsa_circ_	-0.29947	0.42941
hsa_circ_	-0.14001	0.429422
hsa_circ_	0.134339	0.429427
hsa_circ_	-0.1478	0.429431
hsa_circ_	0.106427	0.429447
hsa_circ_	-0.24189	0.429449
hsa_circ_	-0.16879	0.429458
hsa_circ_	0.241846	0.429525
hsa_circ_	0.159577	0.429529
hsa_circ_	0.232856	0.42953
hsa_circ_	-0.12374	0.429541
hsa_circ_	-0.22957	0.429546
hsa_circ_	0.267654	0.429558
hsa_circ_	0.498777	0.429573
hsa_circ_	0.119518	0.429577
hsa_circ_	0.53319	0.429584
hsa_circ_	-0.15473	0.429607
hsa_circ_	0.137227	0.429608
hsa_circ_	0.234086	0.429644
hsa_circ_	-0.23963	0.429649

hsa_circ_	0.464833	0.429657
hsa_circ_	-0.34016	0.429665
hsa_circ_	0.112408	0.429692
hsa_circ_	-0.16212	0.4297
hsa_circ_	-0.34088	0.429703
hsa_circ_	-0.1124	0.429709
hsa_circ_	-0.45612	0.42971
hsa_circ_	-0.17849	0.429714
hsa_circ_	-0.15217	0.429722
hsa_circ_	-0.13506	0.429735
hsa_circ_	-0.10599	0.429738
hsa_circ_	-0.16222	0.429747
hsa_circ_	-0.10964	0.429749
hsa_circ_	-0.1984	0.429752
hsa_circ_	-0.11267	0.429754
hsa_circ_	0.270318	0.429788
hsa_circ_	0.233389	0.429795
hsa_circ_	-0.40629	0.42982
hsa_circ_	-0.3392	0.429836
hsa_circ_	0.358828	0.429843
hsa_circ_	-0.22487	0.429884
hsa_circ_	0.128607	0.429901
hsa_circ_	0.166195	0.429907
hsa_circ_	-0.18936	0.429911
hsa_circ_	-0.41793	0.429925
hsa_circ_	-0.29292	0.429925
hsa_circ_	0.320932	0.429928
hsa_circ_	-0.11807	0.429947
hsa_circ_	0.113758	0.429953
hsa_circ_	0.185161	0.429955
hsa_circ_	0.156442	0.429987
hsa_circ_	-0.19922	0.42999
hsa_circ_	0.110529	0.430004
hsa_circ_	-0.14445	0.430006
hsa_circ_	-0.12922	0.430012
hsa_circ_	-0.15042	0.430053
hsa_circ_	0.4514	0.430066
hsa_circ_	-0.20093	0.430086
hsa_circ_	-0.1142	0.43009
hsa_circ_	0.216417	0.430105
hsa_circ_	0.169264	0.430126
hsa_circ_	-0.1943	0.430155
hsa_circ_	-0.14536	0.430161
hsa_circ_	-0.12363	0.430165
hsa_circ_	-0.12826	0.430172
hsa_circ_	0.13895	0.430176
hsa_circ_	-0.13617	0.430179
hsa_circ_	-0.27475	0.430183
hsa_circ_	0.162004	0.430194
hsa_circ_	0.1434	0.430206
hsa_circ_	-0.16422	0.430215
hsa_circ_	0.149215	0.430222
hsa_circ_	0.208524	0.430227
hsa_circ_	-0.20351	0.430229

hsa_circ_	-0.14058	0.430232
hsa_circ_	-0.12928	0.430237
hsa_circ_	-0.10024	0.430243
hsa_circ_	-0.14167	0.430252
hsa_circ_	0.130704	0.430272
hsa_circ_	-0.18054	0.430273
hsa_circ_	0.240311	0.430337
hsa_circ_	0.120333	0.430342
hsa_circ_	0.165796	0.430344
hsa_circ_	0.183114	0.430384
hsa_circ_	-0.41271	0.430392
hsa_circ_	-0.25221	0.430425
hsa_circ_	-0.11242	0.430447
hsa_circ_	-0.3292	0.430459
hsa_circ_	-0.1365	0.430461
hsa_circ_	0.146417	0.430467
hsa_circ_	0.129434	0.43047
hsa_circ_	-0.182	0.430495
hsa_circ_	0.121799	0.430517
hsa_circ_	-0.11083	0.430528
hsa_circ_	-0.13613	0.430556
hsa_circ_	0.105046	0.430581
hsa_circ_	0.136771	0.430626
hsa_circ_	0.535035	0.430644
hsa_circ_	0.153746	0.430654
hsa_circ_	0.167977	0.430656
hsa_circ_	0.147811	0.430683
hsa_circ_	-0.12003	0.430714
hsa_circ_	0.233213	0.430724
hsa_circ_	0.20715	0.430732
hsa_circ_	-0.24006	0.430809
hsa_circ_	0.140078	0.430819
hsa_circ_	0.119422	0.430822
hsa_circ_	-0.11572	0.43084
hsa_circ_	-0.21785	0.430852
hsa_circ_	-0.22828	0.430867
hsa_circ_	0.520647	0.430868
hsa_circ_	-0.17361	0.430889
hsa_circ_	-0.12754	0.430893
hsa_circ_	-0.15834	0.430916
hsa_circ_	-0.18957	0.430917
hsa_circ_	-0.27541	0.430919
hsa_circ_	0.185079	0.430939
hsa_circ_	0.177865	0.430989
hsa_circ_	-0.101	0.431029
hsa_circ_	-0.18579	0.43104
hsa_circ_	-0.11441	0.431049
hsa_circ_	-0.30502	0.431051
hsa_circ_	-0.1421	0.431061
hsa_circ_	0.13175	0.431066
hsa_circ_	0.116337	0.431073
hsa_circ_	-0.12611	0.431088
hsa_circ_	0.127686	0.431099
hsa_circ_	-0.1196	0.431139

hsa_circ_	-0.19495	0.431151
hsa_circ_	0.128152	0.431157
hsa_circ_	-0.16475	0.431194
hsa_circ_	-0.12862	0.431196
hsa_circ_	-0.13891	0.431208
hsa_circ_	-0.14119	0.431214
hsa_circ_	-0.14059	0.431224
hsa_circ_	-0.11505	0.431264
hsa_circ_	0.130153	0.431265
hsa_circ_	0.143085	0.431342
hsa_circ_	-0.14373	0.431345
hsa_circ_	0.236631	0.431379
hsa_circ_	-0.42461	0.431435
hsa_circ_	-0.46827	0.431451
hsa_circ_	0.422266	0.431458
hsa_circ_	0.135269	0.43147
hsa_circ_	0.194719	0.431478
hsa_circ_	0.137246	0.431489
hsa_circ_	-0.22837	0.431492
hsa_circ_	0.127859	0.431502
hsa_circ_	-0.13151	0.431516
hsa_circ_	-0.14607	0.431533
hsa_circ_	0.142251	0.431537
hsa_circ_	0.145972	0.43155
hsa_circ_	0.21371	0.431559
hsa_circ_	0.125141	0.431576
hsa_circ_	0.417729	0.43158
hsa_circ_	-0.31229	0.431581
hsa_circ_	0.12162	0.431584
hsa_circ_	0.199596	0.4316
hsa_circ_	-0.18066	0.431605
hsa_circ_	0.172429	0.431623
hsa_circ_	-0.13584	0.431627
hsa_circ_	0.11022	0.431634
hsa_circ_	0.178268	0.431638
hsa_circ_	-0.23153	0.431665
hsa_circ_	0.34961	0.431673
hsa_circ_	-0.16715	0.431699
hsa_circ_	-0.13078	0.431712
hsa_circ_	-0.16994	0.431728
hsa_circ_	-0.1193	0.431736
hsa_circ_	-0.32177	0.431751
hsa_circ_	0.188436	0.431752
hsa_circ_	-0.23667	0.431787
hsa_circ_	-0.17575	0.431797
hsa_circ_	-0.16202	0.431833
hsa_circ_	0.139105	0.431852
hsa_circ_	0.15286	0.431862
hsa_circ_	0.139676	0.431865
hsa_circ_	-0.12168	0.431866
hsa_circ_	0.112233	0.43187
hsa_circ_	-0.28879	0.431887
hsa_circ_	0.119834	0.431888
hsa_circ_	-0.13582	0.431897

hsa_circ_	-0.26452	0.431899
hsa_circ_	-0.24547	0.431903
hsa_circ_	0.170677	0.431938
hsa_circ_	-0.18045	0.431938
hsa_circ_	-0.10702	0.431963
hsa_circ_	-0.68665	0.431973
hsa_circ_	0.377246	0.431984
hsa_circ_	-0.14417	0.431988
hsa_circ_	-0.12975	0.431998
hsa_circ_	0.595897	0.431999
hsa_circ_	0.133563	0.432015
hsa_circ_	0.163786	0.432027
hsa_circ_	0.22311	0.432027
hsa_circ_	0.428033	0.432056
hsa_circ_	-0.21449	0.432059
hsa_circ_	-0.59687	0.432066
hsa_circ_	0.185686	0.432081
hsa_circ_	-0.15382	0.432106
hsa_circ_	0.172811	0.432107
hsa_circ_	-0.12182	0.432114
hsa_circ_	-0.10439	0.432117
hsa_circ_	-0.16252	0.432147
hsa_circ_	-0.14738	0.43218
hsa_circ_	0.115765	0.432191
hsa_circ_	-0.14746	0.432191
hsa_circ_	0.123246	0.432194
hsa_circ_	-0.20361	0.432224
hsa_circ_	-0.24221	0.432225
hsa_circ_	-0.19517	0.432226
hsa_circ_	-0.12089	0.432243
hsa_circ_	0.23747	0.432254
hsa_circ_	-0.18147	0.432283
hsa_circ_	-0.13006	0.432285
hsa_circ_	0.263577	0.432287
hsa_circ_	-0.15682	0.43229
hsa_circ_	-0.19726	0.432292
hsa_circ_	0.286117	0.432305
hsa_circ_	-0.12306	0.432308
hsa_circ_	-0.12049	0.432329
hsa_circ_	-0.12909	0.43233
hsa_circ_	0.322509	0.432367
hsa_circ_	0.26783	0.432375
hsa_circ_	0.258682	0.43242
hsa_circ_	0.123461	0.432429
hsa_circ_	-0.19167	0.432431
hsa_circ_	0.309977	0.43244
hsa_circ_	0.182177	0.432442
hsa_circ_	-0.32475	0.432453
hsa_circ_	0.20068	0.432465
hsa_circ_	-0.11753	0.432465
hsa_circ_	0.107362	0.432486
hsa_circ_	0.151686	0.432493
hsa_circ_	-0.13758	0.432494
hsa_circ_	-0.2054	0.432515

hsa_circ_	0.150742	0.432532
hsa_circ_	0.131144	0.432563
hsa_circ_	-0.21202	0.432563
hsa_circ_	-0.28984	0.432569
hsa_circ_	-0.15481	0.432573
hsa_circ_	-0.56532	0.432581
hsa_circ_	-0.1604	0.432611
hsa_circ_	-0.09985	0.432633
hsa_circ_	0.165587	0.432635
hsa_circ_	-0.11495	0.432646
hsa_circ_	-0.38839	0.43265
hsa_circ_	-0.11868	0.43265
hsa_circ_	0.51342	0.432701
hsa_circ_	0.12721	0.432705
hsa_circ_	-0.34806	0.432711
hsa_circ_	0.202442	0.43273
hsa_circ_	0.162847	0.432736
hsa_circ_	0.402762	0.43274
hsa_circ_	-0.17004	0.43276
hsa_circ_	-0.29598	0.432765
hsa_circ_	-0.13625	0.432769
hsa_circ_	-0.26223	0.432774
hsa_circ_	0.177718	0.432788
hsa_circ_	-0.13737	0.432788
hsa_circ_	-0.40685	0.432806
hsa_circ_	-0.639	0.432811
hsa_circ_	-0.11903	0.432827
hsa_circ_	0.11401	0.432844
hsa_circ_	0.132546	0.432848
hsa_circ_	0.180879	0.432854
hsa_circ_	-0.20699	0.43286
hsa_circ_	0.113673	0.432861
hsa_circ_	-0.1936	0.432901
hsa_circ_	-0.11577	0.432938
hsa_circ_	0.20106	0.432972
hsa_circ_	0.12748	0.432976
hsa_circ_	-0.13411	0.432984
hsa_circ_	-0.10997	0.433009
hsa_circ_	-0.34156	0.433017
hsa_circ_	0.127926	0.433022
hsa_circ_	0.107043	0.433026
hsa_circ_	-0.15385	0.433043
hsa_circ_	-0.35392	0.43306
hsa_circ_	0.161192	0.433086
hsa_circ_	0.187457	0.433097
hsa_circ_	-0.28392	0.433097
hsa_circ_	0.507627	0.433102
hsa_circ_	0.160741	0.433106
hsa_circ_	-0.11436	0.433128
hsa_circ_	0.119617	0.43313
hsa_circ_	0.134439	0.433145
hsa_circ_	0.134229	0.433148
hsa_circ_	0.14887	0.433151
hsa_circ_	-0.59697	0.433154

hsa_circ_	0.116601	0.433157
hsa_circ_	0.706591	0.433177
hsa_circ_	-0.34081	0.433177
hsa_circ_	-0.13086	0.433191
hsa_circ_	-0.24903	0.433196
hsa_circ_	-0.152	0.4332
hsa_circ_	0.281156	0.433205
hsa_circ_	0.227868	0.433209
hsa_circ_	-0.17684	0.433219
hsa_circ_	-0.18185	0.433223
hsa_circ_	-0.4524	0.43323
hsa_circ_	-0.14178	0.433234
hsa_circ_	0.165034	0.43325
hsa_circ_	-0.11189	0.433252
hsa_circ_	0.153761	0.433263
hsa_circ_	0.306621	0.433266
hsa_circ_	0.118532	0.433271
hsa_circ_	0.148759	0.433286
hsa_circ_	0.407464	0.433286
hsa_circ_	0.141976	0.433297
hsa_circ_	-0.13016	0.433319
hsa_circ_	-0.37842	0.43332
hsa_circ_	0.113123	0.433323
hsa_circ_	0.226276	0.433334
hsa_circ_	-0.6774	0.433346
hsa_circ_	0.318388	0.433355
hsa_circ_	-0.13635	0.433361
hsa_circ_	-0.69773	0.433368
hsa_circ_	0.165779	0.43339
hsa_circ_	-0.33629	0.433397
hsa_circ_	0.120158	0.433402
hsa_circ_	0.105563	0.433409
hsa_circ_	0.333162	0.433433
hsa_circ_	-0.13036	0.433436
hsa_circ_	-0.1669	0.433453
hsa_circ_	0.396417	0.433468
hsa_circ_	-0.1284	0.433472
hsa_circ_	0.314484	0.433484
hsa_circ_	-0.22215	0.433501
hsa_circ_	-0.1906	0.433504
hsa_circ_	0.106424	0.433522
hsa_circ_	-0.26851	0.433523
hsa_circ_	0.116202	0.43353
hsa_circ_	0.333229	0.433533
hsa_circ_	0.49478	0.433535
hsa_circ_	-0.36029	0.433573
hsa_circ_	-0.12128	0.433578
hsa_circ_	-0.16571	0.43361
hsa_circ_	-0.18679	0.433619
hsa_circ_	0.12379	0.433624
hsa_circ_	0.150068	0.433648
hsa_circ_	-0.42729	0.433648
hsa_circ_	-0.38227	0.433654
hsa_circ_	-0.3336	0.433667

hsa_circ_	0.120611	0.433676
hsa_circ_	0.199886	0.433679
hsa_circ_	0.312145	0.433694
hsa_circ_	0.156039	0.433708
hsa_circ_	-0.14625	0.433713
hsa_circ_	0.135827	0.43372
hsa_circ_	-0.22411	0.433735
hsa_circ_	0.183505	0.433738
hsa_circ_	0.117304	0.43375
hsa_circ_	0.251587	0.433759
hsa_circ_	-0.32616	0.433772
hsa_circ_	-0.20013	0.433778
hsa_circ_	0.198945	0.433802
hsa_circ_	0.389621	0.433809
hsa_circ_	0.147158	0.43383
hsa_circ_	-0.1338	0.433858
hsa_circ_	0.452062	0.433862
hsa_circ_	-0.29265	0.433871
hsa_circ_	-0.36325	0.433882
hsa_circ_	-0.42	0.433907
hsa_circ_	0.273398	0.433914
hsa_circ_	-0.11108	0.433922
hsa_circ_	-0.17351	0.433943
hsa_circ_	-0.16201	0.433943
hsa_circ_	0.22874	0.43395
hsa_circ_	-0.40673	0.433986
hsa_circ_	-0.16136	0.434002
hsa_circ_	0.121384	0.434005
hsa_circ_	0.128907	0.434047
hsa_circ_	0.206511	0.434048
hsa_circ_	0.203616	0.434066
hsa_circ_	0.125227	0.434078
hsa_circ_	0.162605	0.434105
hsa_circ_	-0.24822	0.434118
hsa_circ_	-0.1279	0.434149
hsa_circ_	0.23753	0.434149
hsa_circ_	-0.34563	0.434168
hsa_circ_	0.109839	0.434172
hsa_circ_	0.165974	0.434176
hsa_circ_	-0.15245	0.434181
hsa_circ_	-1.0505	0.434185
hsa_circ_	-0.56172	0.434189
hsa_circ_	0.377631	0.434199
hsa_circ_	0.179614	0.434205
hsa_circ_	-0.32604	0.434223
hsa_circ_	0.162653	0.434235
hsa_circ_	0.572264	0.434244
hsa_circ_	-0.12117	0.434247
hsa_circ_	0.469275	0.43425
hsa_circ_	0.150747	0.434269
hsa_circ_	-0.16202	0.434279
hsa_circ_	0.479031	0.43429
hsa_circ_	0.22775	0.434299
hsa_circ_	-0.11201	0.434301

hsa_circ_	0.233981	0.434307
hsa_circ_	-0.24583	0.434323
hsa_circ_	-0.61479	0.434336
hsa_circ_	0.183477	0.434353
hsa_circ_	0.124571	0.434356
hsa_circ_	-0.14764	0.434366
hsa_circ_	0.135972	0.434366
hsa_circ_	0.135245	0.43438
hsa_circ_	0.158047	0.434381
hsa_circ_	0.19779	0.434434
hsa_circ_	0.106894	0.434441
hsa_circ_	0.401989	0.43445
hsa_circ_	0.12152	0.434469
hsa_circ_	0.123896	0.434491
hsa_circ_	0.150156	0.434494
hsa_circ_	0.104577	0.434498
hsa_circ_	-0.11658	0.43453
hsa_circ_	0.208756	0.43454
hsa_circ_	-0.16059	0.434549
hsa_circ_	-0.20175	0.434552
hsa_circ_	-0.34578	0.434557
hsa_circ_	-0.28055	0.434561
hsa_circ_	-0.12122	0.434568
hsa_circ_	0.18117	0.434582
hsa_circ_	0.331518	0.434588
hsa_circ_	0.197387	0.434623
hsa_circ_	-0.17634	0.434624
hsa_circ_	0.405495	0.434627
hsa_circ_	-0.20332	0.43463
hsa_circ_	-0.17514	0.434665
hsa_circ_	-0.10317	0.434665
hsa_circ_	0.131772	0.434667
hsa_circ_	-0.21946	0.434684
hsa_circ_	-0.1309	0.43469
hsa_circ_	-0.12764	0.43472
hsa_circ_	0.166285	0.434738
hsa_circ_	0.126303	0.434761
hsa_circ_	0.182	0.43477
hsa_circ_	0.19261	0.434783
hsa_circ_	-0.17602	0.434797
hsa_circ_	0.164712	0.4348
hsa_circ_	0.137609	0.434814
hsa_circ_	-0.14879	0.434816
hsa_circ_	0.187881	0.434829
hsa_circ_	0.179597	0.434832
hsa_circ_	-0.12055	0.434865
hsa_circ_	-0.13806	0.434873
hsa_circ_	0.605051	0.434876
hsa_circ_	-0.14148	0.434883
hsa_circ_	0.134058	0.434894
hsa_circ_	0.243699	0.434896
hsa_circ_	0.509371	0.434901
hsa_circ_	0.126332	0.434901
hsa_circ_	-0.21703	0.434911

hsa_circ_	-0.15661	0.434912
hsa_circ_	0.18486	0.434919
hsa_circ_	-0.16006	0.434951
hsa_circ_	0.165212	0.434956
hsa_circ_	0.339216	0.434979
hsa_circ_	-0.47896	0.434989
hsa_circ_	0.174841	0.434994
hsa_circ_	-0.15669	0.435004
hsa_circ_	-0.13429	0.435006
hsa_circ_	0.277346	0.435046
hsa_circ_	0.182955	0.435047
hsa_circ_	-0.46174	0.435054
hsa_circ_	-0.96155	0.435064
hsa_circ_	0.145393	0.435077
hsa_circ_	-0.10326	0.435101
hsa_circ_	-0.45105	0.435107
hsa_circ_	-0.57262	0.435117
hsa_circ_	-0.24803	0.435132
hsa_circ_	-0.11869	0.435175
hsa_circ_	-0.41415	0.435177
hsa_circ_	0.22341	0.435196
hsa_circ_	-0.22579	0.435201
hsa_circ_	-0.16283	0.435206
hsa_circ_	-0.10194	0.435222
hsa_circ_	-0.30297	0.435234
hsa_circ_	0.14295	0.435258
hsa_circ_	0.151165	0.43528
hsa_circ_	-0.17568	0.43529
hsa_circ_	-0.15727	0.435329
hsa_circ_	-0.11115	0.43535
hsa_circ_	-0.41255	0.435369
hsa_circ_	-0.13934	0.435375
hsa_circ_	-0.24727	0.43538
hsa_circ_	-0.13264	0.435392
hsa_circ_	-0.15767	0.435395
hsa_circ_	-0.20218	0.435397
hsa_circ_	-0.27998	0.435479
hsa_circ_	-0.13242	0.435489
hsa_circ_	0.179413	0.43549
hsa_circ_	-0.22576	0.43551
hsa_circ_	-0.17555	0.43552
hsa_circ_	0.141172	0.435533
hsa_circ_	-0.17306	0.435541
hsa_circ_	-0.1623	0.435555
hsa_circ_	-0.11233	0.435565
hsa_circ_	-0.14929	0.43557
hsa_circ_	-0.22803	0.435595
hsa_circ_	-0.19861	0.435602
hsa_circ_	-0.13518	0.435623
hsa_circ_	0.168417	0.435645
hsa_circ_	-0.32787	0.435652
hsa_circ_	0.150039	0.435658
hsa_circ_	-0.1637	0.435672
hsa_circ_	0.17936	0.435675

hsa_circ_	-0.12143	0.435683
hsa_circ_	0.378642	0.435693
hsa_circ_	0.131792	0.435707
hsa_circ_	-0.15998	0.435717
hsa_circ_	-0.42426	0.43572
hsa_circ_	-0.21575	0.43573
hsa_circ_	0.236469	0.435792
hsa_circ_	0.190569	0.435812
hsa_circ_	-0.12264	0.435818
hsa_circ_	-0.15814	0.43584
hsa_circ_	-0.18877	0.435854
hsa_circ_	0.206199	0.435862
hsa_circ_	0.171825	0.435864
hsa_circ_	-0.18863	0.435872
hsa_circ_	-0.21841	0.435872
hsa_circ_	0.161473	0.435913
hsa_circ_	0.14725	0.435933
hsa_circ_	0.23124	0.435937
hsa_circ_	-0.3867	0.435939
hsa_circ_	0.353283	0.435959
hsa_circ_	0.120382	0.435964
hsa_circ_	-0.21052	0.435967
hsa_circ_	0.147808	0.435971
hsa_circ_	0.216508	0.435973
hsa_circ_	-0.4532	0.435975
hsa_circ_	0.177077	0.435975
hsa_circ_	-0.12994	0.435985
hsa_circ_	-0.11863	0.436005
hsa_circ_	-0.21783	0.436007
hsa_circ_	0.421295	0.436014
hsa_circ_	-0.30912	0.436018
hsa_circ_	0.619491	0.43606
hsa_circ_	0.181744	0.436066
hsa_circ_	-0.18953	0.436083
hsa_circ_	0.382761	0.436092
hsa_circ_	-0.12057	0.436097
hsa_circ_	-0.12146	0.436099
hsa_circ_	-0.2184	0.436114
hsa_circ_	0.107117	0.436117
hsa_circ_	0.195424	0.436133
hsa_circ_	-0.13546	0.436141
hsa_circ_	-0.13745	0.436182
hsa_circ_	0.114757	0.436198
hsa_circ_	0.552142	0.43621
hsa_circ_	-0.18081	0.436235
hsa_circ_	-0.30609	0.436236
hsa_circ_	0.118436	0.436241
hsa_circ_	0.22306	0.436255
hsa_circ_	0.554763	0.436255
hsa_circ_	0.175698	0.436262
hsa_circ_	0.12315	0.436272
hsa_circ_	0.166503	0.436273
hsa_circ_	0.115307	0.43629
hsa_circ_	-0.10374	0.436295

hsa_circ_	0.201822	0.436353
hsa_circ_	-0.16715	0.43636
hsa_circ_	-0.30178	0.436393
hsa_circ_	0.158757	0.436403
hsa_circ_	0.1418	0.436418
hsa_circ_	-0.26208	0.436437
hsa_circ_	0.280057	0.436453
hsa_circ_	0.206056	0.436456
hsa_circ_	0.148278	0.436472
hsa_circ_	0.161861	0.436536
hsa_circ_	0.137949	0.43654
hsa_circ_	0.314474	0.436541
hsa_circ_	-0.2279	0.436571
hsa_circ_	-0.12823	0.436601
hsa_circ_	-0.45806	0.436625
hsa_circ_	-0.1221	0.436635
hsa_circ_	0.176348	0.436637
hsa_circ_	-0.18415	0.436644
hsa_circ_	-0.16864	0.436651
hsa_circ_	-0.33019	0.436663
hsa_circ_	-0.16113	0.43667
hsa_circ_	-0.12184	0.436683
hsa_circ_	0.201067	0.436686
hsa_circ_	-0.17495	0.436686
hsa_circ_	-0.19866	0.436726
hsa_circ_	-0.45617	0.436751
hsa_circ_	0.165936	0.436777
hsa_circ_	0.494502	0.436777
hsa_circ_	-0.31065	0.43679
hsa_circ_	0.244679	0.436795
hsa_circ_	0.1483	0.436814
hsa_circ_	-0.10407	0.436821
hsa_circ_	0.107488	0.436836
hsa_circ_	0.35147	0.436845
hsa_circ_	0.212961	0.436896
hsa_circ_	0.179087	0.436898
hsa_circ_	0.139829	0.436907
hsa_circ_	0.29278	0.436957
hsa_circ_	-0.11491	0.436959
hsa_circ_	-0.26172	0.43697
hsa_circ_	-0.14574	0.436974
hsa_circ_	-0.1788	0.436985
hsa_circ_	0.129947	0.437003
hsa_circ_	-0.33886	0.437005
hsa_circ_	-0.10735	0.437013
hsa_circ_	0.128278	0.437016
hsa_circ_	-0.17077	0.437031
hsa_circ_	-0.17293	0.437035
hsa_circ_	0.16148	0.437049
hsa_circ_	-0.10748	0.437055
hsa_circ_	-0.29951	0.437065
hsa_circ_	0.127326	0.437073
hsa_circ_	0.36582	0.43708
hsa_circ_	-0.20157	0.437084

hsa_circ_	-0.45451	0.437086
hsa_circ_	0.132983	0.437113
hsa_circ_	-0.16105	0.437115
hsa_circ_	0.239426	0.43712
hsa_circ_	-0.11889	0.437121
hsa_circ_	-0.42296	0.437144
hsa_circ_	-0.1985	0.437148
hsa_circ_	-0.11083	0.437155
hsa_circ_	0.345015	0.437182
hsa_circ_	0.361709	0.437186
hsa_circ_	0.291297	0.437187
hsa_circ_	0.12592	0.437241
hsa_circ_	0.187219	0.437248
hsa_circ_	0.103267	0.437256
hsa_circ_	0.304148	0.437279
hsa_circ_	0.279946	0.437281
hsa_circ_	0.413538	0.437286
hsa_circ_	-0.27967	0.437297
hsa_circ_	0.159327	0.43731
hsa_circ_	-0.29964	0.437331
hsa_circ_	-0.14088	0.437338
hsa_circ_	-0.15296	0.43734
hsa_circ_	0.173519	0.437358
hsa_circ_	-0.11565	0.43737
hsa_circ_	-0.12688	0.437391
hsa_circ_	-0.60297	0.437403
hsa_circ_	0.240377	0.43741
hsa_circ_	-0.27198	0.437422
hsa_circ_	-0.12906	0.437432
hsa_circ_	0.160006	0.437439
hsa_circ_	0.207763	0.437444
hsa_circ_	-0.11767	0.43745
hsa_circ_	0.204003	0.437485
hsa_circ_	0.173864	0.437493
hsa_circ_	-0.15004	0.437507
hsa_circ_	-0.31743	0.437513
hsa_circ_	-0.18738	0.437518
hsa_circ_	0.15415	0.437521
hsa_circ_	0.242743	0.437526
hsa_circ_	-0.15853	0.437533
hsa_circ_	0.157056	0.437541
hsa_circ_	0.255921	0.437557
hsa_circ_	-0.40004	0.437567
hsa_circ_	-0.16436	0.437571
hsa_circ_	0.454397	0.437572
hsa_circ_	-0.10988	0.437578
hsa_circ_	0.137432	0.437579
hsa_circ_	0.121393	0.43758
hsa_circ_	-0.11818	0.437586
hsa_circ_	0.232305	0.437635
hsa_circ_	0.167439	0.437653
hsa_circ_	0.275861	0.437664
hsa_circ_	-0.12182	0.437665
hsa_circ_	0.209327	0.437692

hsa_circ_	-0.15237	0.437695
hsa_circ_	0.14795	0.437747
hsa_circ_	0.181886	0.437749
hsa_circ_	-0.1101	0.437757
hsa_circ_	0.579414	0.437758
hsa_circ_	-0.13444	0.437782
hsa_circ_	-0.12895	0.437791
hsa_circ_	0.128171	0.4378
hsa_circ_	-0.22468	0.437809
hsa_circ_	0.300887	0.437817
hsa_circ_	0.349001	0.437838
hsa_circ_	-0.30669	0.437838
hsa_circ_	0.756579	0.437842
hsa_circ_	-0.10795	0.437864
hsa_circ_	-0.10038	0.437872
hsa_circ_	-0.12842	0.437874
hsa_circ_	0.10226	0.437882
hsa_circ_	-0.2019	0.437894
hsa_circ_	-0.1779	0.437903
hsa_circ_	0.119205	0.437909
hsa_circ_	-0.24899	0.437915
hsa_circ_	0.176861	0.437918
hsa_circ_	0.291558	0.437928
hsa_circ_	-0.21532	0.43795
hsa_circ_	-0.1279	0.437991
hsa_circ_	0.180549	0.438
hsa_circ_	0.172761	0.438003
hsa_circ_	-0.10578	0.438036
hsa_circ_	0.220207	0.438049
hsa_circ_	-0.34397	0.438057
hsa_circ_	-0.50391	0.438059
hsa_circ_	0.441409	0.438062
hsa_circ_	0.146127	0.438078
hsa_circ_	0.131466	0.438083
hsa_circ_	0.202382	0.43809
hsa_circ_	-0.12093	0.438127
hsa_circ_	0.206825	0.438169
hsa_circ_	0.356899	0.438177
hsa_circ_	-0.30595	0.438192
hsa_circ_	-0.14736	0.43821
hsa_circ_	-0.14668	0.438215
hsa_circ_	-0.45484	0.438259
hsa_circ_	0.151167	0.438301
hsa_circ_	-0.10167	0.438309
hsa_circ_	0.118769	0.438314
hsa_circ_	0.15487	0.438363
hsa_circ_	-0.18886	0.438365
hsa_circ_	-0.15464	0.438388
hsa_circ_	-0.10283	0.438395
hsa_circ_	0.181357	0.4384
hsa_circ_	-0.1129	0.438402
hsa_circ_	0.107241	0.438405
hsa_circ_	-0.17783	0.438428
hsa_circ_	0.143444	0.438434

hsa_circ_	-0.15535	0.438446
hsa_circ_	0.127684	0.438453
hsa_circ_	0.190961	0.438454
hsa_circ_	0.211098	0.438456
hsa_circ_	-0.16625	0.438484
hsa_circ_	0.225427	0.438486
hsa_circ_	0.164701	0.438498
hsa_circ_	-0.12568	0.4385
hsa_circ_	0.598285	0.438503
hsa_circ_	0.253861	0.438527
hsa_circ_	-0.14256	0.438566
hsa_circ_	0.161965	0.438571
hsa_circ_	0.145272	0.438575
hsa_circ_	0.200225	0.438578
hsa_circ_	-0.4869	0.43858
hsa_circ_	0.11417	0.438581
hsa_circ_	-0.15519	0.438583
hsa_circ_	-0.21138	0.438593
hsa_circ_	0.201242	0.4386
hsa_circ_	0.120697	0.438619
hsa_circ_	-0.16556	0.438634
hsa_circ_	-0.11063	0.438693
hsa_circ_	-0.10765	0.438694
hsa_circ_	0.117345	0.438695
hsa_circ_	0.157122	0.438709
hsa_circ_	0.213244	0.438714
hsa_circ_	-0.19089	0.43874
hsa_circ_	0.158507	0.43875
hsa_circ_	-0.20549	0.438781
hsa_circ_	-0.26679	0.438798
hsa_circ_	0.367465	0.438799
hsa_circ_	0.338505	0.438801
hsa_circ_	0.21231	0.438805
hsa_circ_	-0.25299	0.438809
hsa_circ_	-0.11745	0.438811
hsa_circ_	-0.16324	0.438812
hsa_circ_	0.148792	0.438816
hsa_circ_	0.117006	0.43882
hsa_circ_	-0.1478	0.438823
hsa_circ_	-0.25062	0.438834
hsa_circ_	0.16923	0.43884
hsa_circ_	0.141287	0.438854
hsa_circ_	-0.25068	0.438855
hsa_circ_	0.150018	0.438884
hsa_circ_	0.23348	0.438909
hsa_circ_	-0.32582	0.438936
hsa_circ_	0.107408	0.43897
hsa_circ_	-0.37458	0.438971
hsa_circ_	0.155607	0.438978
hsa_circ_	-0.37526	0.438986
hsa_circ_	-0.13246	0.438991
hsa_circ_	-0.16546	0.439003
hsa_circ_	-0.24529	0.439015
hsa_circ_	0.220635	0.439029

hsa_circ_	-0.09828	0.439033
hsa_circ_	0.131968	0.439036
hsa_circ_	0.47091	0.439051
hsa_circ_	0.184555	0.439051
hsa_circ_	0.334713	0.439053
hsa_circ_	0.112357	0.439058
hsa_circ_	-0.11858	0.43906
hsa_circ_	0.289094	0.439062
hsa_circ_	-0.65667	0.439068
hsa_circ_	0.552024	0.439113
hsa_circ_	0.105475	0.439122
hsa_circ_	0.204826	0.439125
hsa_circ_	0.569648	0.439142
hsa_circ_	-0.12829	0.43918
hsa_circ_	0.2635	0.439181
hsa_circ_	-0.16441	0.439205
hsa_circ_	-0.43304	0.43921
hsa_circ_	-0.15382	0.439213
hsa_circ_	-0.31046	0.439231
hsa_circ_	-0.32976	0.439242
hsa_circ_	-0.12249	0.439257
hsa_circ_	0.140578	0.43926
hsa_circ_	0.115901	0.439261
hsa_circ_	-0.60007	0.439272
hsa_circ_	-0.14786	0.439273
hsa_circ_	-0.41717	0.439277
hsa_circ_	-0.33842	0.439289
hsa_circ_	0.119137	0.43929
hsa_circ_	-0.54649	0.43933
hsa_circ_	0.104873	0.439341
hsa_circ_	-0.34832	0.439342
hsa_circ_	-0.26729	0.439347
hsa_circ_	0.379491	0.439352
hsa_circ_	0.228944	0.439358
hsa_circ_	0.141779	0.439361
hsa_circ_	-0.18928	0.439374
hsa_circ_	0.306078	0.43939
hsa_circ_	-0.11813	0.43941
hsa_circ_	0.149425	0.439443
hsa_circ_	-0.27098	0.439448
hsa_circ_	-0.16392	0.439475
hsa_circ_	-0.15322	0.439492
hsa_circ_	-0.22444	0.439505
hsa_circ_	-0.11132	0.439513
hsa_circ_	-0.26096	0.439516
hsa_circ_	-0.13952	0.439516
hsa_circ_	0.185614	0.439529
hsa_circ_	0.19437	0.439552
hsa_circ_	-0.30433	0.439564
hsa_circ_	-0.14967	0.43957
hsa_circ_	0.107321	0.43958
hsa_circ_	0.352468	0.439582
hsa_circ_	0.255979	0.439586
hsa_circ_	-0.15162	0.439587

hsa_circ_	0.235471	0.43959
hsa_circ_	0.232302	0.439613
hsa_circ_	-0.29622	0.439623
hsa_circ_	0.179482	0.439624
hsa_circ_	-0.14249	0.439646
hsa_circ_	0.153114	0.439669
hsa_circ_	-0.11809	0.439677
hsa_circ_	-0.118	0.439696
hsa_circ_	-0.31557	0.439705
hsa_circ_	-0.23631	0.439731
hsa_circ_	0.189223	0.439731
hsa_circ_	-0.13994	0.43976
hsa_circ_	-0.39222	0.439761
hsa_circ_	0.113019	0.43977
hsa_circ_	-0.31338	0.439794
hsa_circ_	-0.12386	0.439811
hsa_circ_	-0.18806	0.439816
hsa_circ_	-0.46638	0.439825
hsa_circ_	-0.30882	0.439859
hsa_circ_	0.141614	0.439861
hsa_circ_	0.460246	0.439873
hsa_circ_	0.264054	0.439882
hsa_circ_	0.256076	0.439919
hsa_circ_	-0.47861	0.439935
hsa_circ_	0.151822	0.439942
hsa_circ_	0.12258	0.439944
hsa_circ_	0.179826	0.439949
hsa_circ_	0.133011	0.439958
hsa_circ_	0.314703	0.439987
hsa_circ_	-0.47318	0.440029
hsa_circ_	0.294736	0.440055
hsa_circ_	0.130386	0.440085
hsa_circ_	-0.13938	0.44009
hsa_circ_	0.280695	0.440095
hsa_circ_	0.161384	0.440103
hsa_circ_	0.387622	0.44011
hsa_circ_	0.304405	0.440145
hsa_circ_	0.113837	0.440147
hsa_circ_	0.192456	0.440152
hsa_circ_	-0.16342	0.440153
hsa_circ_	-0.18967	0.440155
hsa_circ_	-0.36547	0.440169
hsa_circ_	-0.12866	0.44017
hsa_circ_	0.125861	0.440183
hsa_circ_	0.197194	0.440235
hsa_circ_	-0.21145	0.440255
hsa_circ_	-0.12854	0.440265
hsa_circ_	0.251939	0.440273
hsa_circ_	0.439652	0.440284
hsa_circ_	-0.36655	0.440299
hsa_circ_	-0.39723	0.4403
hsa_circ_	-0.17317	0.440312
hsa_circ_	0.262206	0.440319
hsa_circ_	0.123199	0.440324

hsa_circ_	-0.31541	0.440349
hsa_circ_	-0.13966	0.440349
hsa_circ_	-0.44822	0.440352
hsa_circ_	0.124748	0.440366
hsa_circ_	-0.1364	0.440375
hsa_circ_	0.172501	0.440387
hsa_circ_	0.187143	0.440388
hsa_circ_	0.329257	0.440397
hsa_circ_	0.215108	0.440409
hsa_circ_	0.109261	0.440433
hsa_circ_	0.120843	0.440435
hsa_circ_	0.143676	0.440439
hsa_circ_	-0.10869	0.440444
hsa_circ_	-0.15929	0.44045
hsa_circ_	-0.36408	0.440463
hsa_circ_	0.127254	0.440467
hsa_circ_	-0.15316	0.440475
hsa_circ_	-0.15019	0.44048
hsa_circ_	0.115496	0.440506
hsa_circ_	-0.15104	0.440507
hsa_circ_	-0.40757	0.440523
hsa_circ_	0.361751	0.440552
hsa_circ_	0.144978	0.440571
hsa_circ_	0.303759	0.440592
hsa_circ_	0.151554	0.440599
hsa_circ_	0.245017	0.4406
hsa_circ_	-0.26499	0.440603
hsa_circ_	0.166429	0.440612
hsa_circ_	-0.3699	0.440644
hsa_circ_	-0.10639	0.4407
hsa_circ_	-0.34984	0.44071
hsa_circ_	-0.13972	0.440715
hsa_circ_	-0.10567	0.440727
hsa_circ_	0.119499	0.440733
hsa_circ_	0.450304	0.440744
hsa_circ_	0.13194	0.440752
hsa_circ_	0.12339	0.440754
hsa_circ_	0.145955	0.440754
hsa_circ_	0.252628	0.440754
hsa_circ_	-0.41305	0.440757
hsa_circ_	0.215395	0.440757
hsa_circ_	-0.19194	0.440763
hsa_circ_	0.254463	0.440774
hsa_circ_	-0.17681	0.440775
hsa_circ_	0.165602	0.440798
hsa_circ_	0.302065	0.440805
hsa_circ_	0.155962	0.440851
hsa_circ_	-0.29724	0.440877
hsa_circ_	-0.11448	0.440883
hsa_circ_	-0.23846	0.440887
hsa_circ_	0.179602	0.440902
hsa_circ_	0.153219	0.440915
hsa_circ_	0.208825	0.44095
hsa_circ_	-0.39556	0.440952

hsa_circ_	0.36084	0.440954
hsa_circ_	0.239948	0.440955
hsa_circ_	-0.09921	0.440955
hsa_circ_	-0.10969	0.440961
hsa_circ_	-0.16296	0.440973
hsa_circ_	-0.12798	0.44098
hsa_circ_	-0.20715	0.440991
hsa_circ_	-0.52306	0.44102
hsa_circ_	-0.16648	0.441037
hsa_circ_	0.113687	0.441047
hsa_circ_	0.264531	0.441059
hsa_circ_	0.149409	0.441065
hsa_circ_	-0.18158	0.44107
hsa_circ_	0.223306	0.441076
hsa_circ_	0.155833	0.441083
hsa_circ_	0.282858	0.441088
hsa_circ_	0.220698	0.441092
hsa_circ_	-0.12244	0.441103
hsa_circ_	0.454656	0.441116
hsa_circ_	0.284248	0.441116
hsa_circ_	0.295231	0.44113
hsa_circ_	0.125772	0.441167
hsa_circ_	0.374197	0.441182
hsa_circ_	0.185628	0.441189
hsa_circ_	0.455519	0.441198
hsa_circ_	-0.13794	0.441205
hsa_circ_	-0.23793	0.441239
hsa_circ_	0.133511	0.441255
hsa_circ_	-0.11257	0.441282
hsa_circ_	0.237391	0.441286
hsa_circ_	0.313333	0.441293
hsa_circ_	-0.13769	0.441295
hsa_circ_	-0.17977	0.441309
hsa_circ_	-0.13874	0.441343
hsa_circ_	0.170852	0.441366
hsa_circ_	-0.54813	0.44137
hsa_circ_	0.326019	0.441376
hsa_circ_	0.200125	0.441377
hsa_circ_	-0.10641	0.44138
hsa_circ_	-0.39294	0.44138
hsa_circ_	-0.14061	0.441382
hsa_circ_	0.200105	0.441389
hsa_circ_	0.141658	0.441404
hsa_circ_	-0.14345	0.441418
hsa_circ_	-0.15443	0.441481
hsa_circ_	0.215219	0.441486
hsa_circ_	0.141099	0.441497
hsa_circ_	-0.29713	0.441512
hsa_circ_	0.133327	0.441524
hsa_circ_	0.335665	0.441528
hsa_circ_	-0.354	0.441534
hsa_circ_	0.148422	0.441536
hsa_circ_	-0.15036	0.441537
hsa_circ_	0.346809	0.441551

hsa_circ_	0.198931	0.441566
hsa_circ_	-0.11253	0.441585
hsa_circ_	-0.11267	0.441587
hsa_circ_	-0.27057	0.441594
hsa_circ_	0.10599	0.441618
hsa_circ_	0.188184	0.441644
hsa_circ_	-0.15261	0.441654
hsa_circ_	0.230946	0.441655
hsa_circ_	-0.16771	0.441706
hsa_circ_	0.130989	0.441737
hsa_circ_	0.147299	0.441742
hsa_circ_	-0.16868	0.441747
hsa_circ_	-0.15703	0.441749
hsa_circ_	-0.2646	0.441777
hsa_circ_	-0.15734	0.441808
hsa_circ_	-0.16213	0.441831
hsa_circ_	-0.12678	0.441832
hsa_circ_	-0.37342	0.441856
hsa_circ_	0.170016	0.441867
hsa_circ_	0.196091	0.441867
hsa_circ_	0.161205	0.441904
hsa_circ_	-0.41767	0.441909
hsa_circ_	0.164826	0.441928
hsa_circ_	-0.13499	0.441931
hsa_circ_	-0.17124	0.441945
hsa_circ_	0.196002	0.44196
hsa_circ_	0.50179	0.441976
hsa_circ_	-0.23171	0.441984
hsa_circ_	-0.26092	0.441997
hsa_circ_	-0.41649	0.441998
hsa_circ_	0.344931	0.442014
hsa_circ_	0.303424	0.442015
hsa_circ_	0.13153	0.442022
hsa_circ_	-0.38986	0.442029
hsa_circ_	0.147943	0.442039
hsa_circ_	0.164397	0.44204
hsa_circ_	-0.20744	0.442041
hsa_circ_	0.139207	0.442073
hsa_circ_	-0.15923	0.44209
hsa_circ_	-0.15856	0.442106
hsa_circ_	0.394703	0.44212
hsa_circ_	0.099127	0.442124
hsa_circ_	0.116517	0.442131
hsa_circ_	0.336575	0.442158
hsa_circ_	-0.30737	0.442173
hsa_circ_	-0.11805	0.442191
hsa_circ_	-0.14279	0.442214
hsa_circ_	-0.13386	0.442221
hsa_circ_	-0.26179	0.442233
hsa_circ_	-0.44529	0.442237
hsa_circ_	-0.20257	0.442249
hsa_circ_	-0.1384	0.442252
hsa_circ_	-0.23719	0.442269
hsa_circ_	-0.14175	0.442286

hsa_circ_	0.228188	0.442293
hsa_circ_	0.369229	0.442304
hsa_circ_	-0.17935	0.442319
hsa_circ_	0.155531	0.442322
hsa_circ_	-0.10606	0.442323
hsa_circ_	0.112566	0.442326
hsa_circ_	0.113886	0.442326
hsa_circ_	0.13407	0.442332
hsa_circ_	0.116414	0.442363
hsa_circ_	0.125632	0.442401
hsa_circ_	-0.13387	0.442413
hsa_circ_	0.149508	0.442442
hsa_circ_	0.158311	0.442454
hsa_circ_	0.116017	0.442456
hsa_circ_	0.107127	0.442462
hsa_circ_	0.253869	0.442487
hsa_circ_	-0.1933	0.442509
hsa_circ_	0.235755	0.44253
hsa_circ_	-0.11392	0.442537
hsa_circ_	0.482739	0.44254
hsa_circ_	0.144999	0.442553
hsa_circ_	-0.20459	0.442554
hsa_circ_	-0.17725	0.442571
hsa_circ_	0.305719	0.442579
hsa_circ_	0.119414	0.442584
hsa_circ_	-0.10995	0.442598
hsa_circ_	-0.14243	0.442611
hsa_circ_	-0.20246	0.442633
hsa_circ_	-0.25297	0.442644
hsa_circ_	0.280188	0.442648
hsa_circ_	-0.16026	0.442665
hsa_circ_	0.202767	0.442668
hsa_circ_	-0.10405	0.442674
hsa_circ_	-0.10452	0.442682
hsa_circ_	-0.41513	0.442684
hsa_circ_	0.1531	0.442696
hsa_circ_	0.345531	0.442696
hsa_circ_	0.218368	0.442708
hsa_circ_	0.150962	0.442719
hsa_circ_	-0.18388	0.442724
hsa_circ_	0.14258	0.442736
hsa_circ_	-0.49834	0.442742
hsa_circ_	0.624178	0.442746
hsa_circ_	-0.12563	0.442746
hsa_circ_	-0.24517	0.442796
hsa_circ_	-0.39076	0.442846
hsa_circ_	-0.11824	0.442854
hsa_circ_	-0.11003	0.442928
hsa_circ_	-0.12523	0.442947
hsa_circ_	0.145271	0.442994
hsa_circ_	-0.46489	0.443007
hsa_circ_	0.136505	0.443031
hsa_circ_	0.127348	0.443047
hsa_circ_	0.225171	0.443104

hsa_circ_	0.104538	0.443111
hsa_circ_	0.17307	0.443116
hsa_circ_	0.197498	0.44312
hsa_circ_	-0.14061	0.443143
hsa_circ_	0.284615	0.443144
hsa_circ_	-0.19	0.443145
hsa_circ_	0.201403	0.443178
hsa_circ_	-0.1077	0.443182
hsa_circ_	-1.29128	0.443194
hsa_circ_	-1.62824	0.443202
hsa_circ_	0.14703	0.443211
hsa_circ_	0.125958	0.443229
hsa_circ_	-0.11114	0.443235
hsa_circ_	-0.13889	0.443236
hsa_circ_	-0.32472	0.443236
hsa_circ_	0.111825	0.443247
hsa_circ_	0.152022	0.443252
hsa_circ_	-0.44358	0.443299
hsa_circ_	-0.33109	0.443302
hsa_circ_	0.142295	0.443338
hsa_circ_	-0.13394	0.443341
hsa_circ_	0.124029	0.44335
hsa_circ_	0.135522	0.443352
hsa_circ_	0.107014	0.443371
hsa_circ_	0.173151	0.443385
hsa_circ_	-0.29522	0.44342
hsa_circ_	0.212107	0.443423
hsa_circ_	-0.24611	0.443454
hsa_circ_	0.128811	0.44346
hsa_circ_	0.144868	0.443505
hsa_circ_	-0.34172	0.443505
hsa_circ_	0.174197	0.443509
hsa_circ_	-0.15312	0.443535
hsa_circ_	0.108116	0.443551
hsa_circ_	-0.11505	0.443564
hsa_circ_	-0.16229	0.443564
hsa_circ_	0.200898	0.443572
hsa_circ_	-0.15361	0.443584
hsa_circ_	0.170398	0.443602
hsa_circ_	0.121261	0.443646
hsa_circ_	-0.12245	0.443649
hsa_circ_	-0.19469	0.443671
hsa_circ_	0.405503	0.443672
hsa_circ_	-0.2198	0.443672
hsa_circ_	-0.20694	0.443682
hsa_circ_	-0.11446	0.443686
hsa_circ_	-0.12312	0.443707
hsa_circ_	0.101675	0.443717
hsa_circ_	-0.34299	0.443735
hsa_circ_	0.168374	0.443735
hsa_circ_	-0.1571	0.443736
hsa_circ_	0.134037	0.443744
hsa_circ_	0.147795	0.443749
hsa_circ_	0.130469	0.44377

hsa_circ_	-0.14316	0.443776
hsa_circ_	-0.26304	0.443777
hsa_circ_	-0.12272	0.443787
hsa_circ_	-0.2304	0.443815
hsa_circ_	0.187189	0.443816
hsa_circ_	0.360457	0.443842
hsa_circ_	-0.23321	0.44386
hsa_circ_	-0.15016	0.443862
hsa_circ_	-0.39506	0.44388
hsa_circ_	0.168514	0.443893
hsa_circ_	-0.32481	0.443903
hsa_circ_	-0.10452	0.443915
hsa_circ_	0.15407	0.443925
hsa_circ_	-0.11406	0.443937
hsa_circ_	0.120528	0.443938
hsa_circ_	0.159166	0.443942
hsa_circ_	-0.44538	0.443951
hsa_circ_	-0.10638	0.443953
hsa_circ_	0.364475	0.443954
hsa_circ_	-0.28892	0.443958
hsa_circ_	-0.19476	0.443965
hsa_circ_	0.279447	0.443981
hsa_circ_	-0.15629	0.443987
hsa_circ_	0.114216	0.443998
hsa_circ_	0.193828	0.444013
hsa_circ_	0.180248	0.444019
hsa_circ_	-0.18807	0.444021
hsa_circ_	0.342653	0.444022
hsa_circ_	0.149349	0.44403
hsa_circ_	0.310111	0.444055
hsa_circ_	0.130407	0.444072
hsa_circ_	0.162003	0.444076
hsa_circ_	0.239608	0.444077
hsa_circ_	0.412854	0.444083
hsa_circ_	0.257071	0.444096
hsa_circ_	0.112777	0.444128
hsa_circ_	-0.26882	0.444198
hsa_circ_	-0.10316	0.444202
hsa_circ_	-0.35416	0.444207
hsa_circ_	0.241005	0.444213
hsa_circ_	-0.38454	0.444221
hsa_circ_	-0.27646	0.444224
hsa_circ_	-0.14452	0.444239
hsa_circ_	-0.34765	0.44425
hsa_circ_	0.14082	0.44425
hsa_circ_	-0.36119	0.444263
hsa_circ_	0.105098	0.444285
hsa_circ_	0.124733	0.444297
hsa_circ_	-0.10499	0.444316
hsa_circ_	-0.19481	0.444333
hsa_circ_	0.125639	0.444352
hsa_circ_	-0.26153	0.444358
hsa_circ_	-0.14128	0.444404
hsa_circ_	0.13456	0.444406

hsa_circ_	-0.16366	0.444419
hsa_circ_	0.104479	0.44442
hsa_circ_	-0.20112	0.444424
hsa_circ_	-0.10835	0.44444
hsa_circ_	-0.10361	0.444446
hsa_circ_	0.465566	0.444507
hsa_circ_	0.139717	0.444509
hsa_circ_	-0.30223	0.444509
hsa_circ_	-0.12707	0.444524
hsa_circ_	0.497692	0.444528
hsa_circ_	-0.21727	0.44453
hsa_circ_	-0.11516	0.444558
hsa_circ_	-0.12226	0.444603
hsa_circ_	0.126082	0.444607
hsa_circ_	0.164019	0.444614
hsa_circ_	-0.12238	0.444623
hsa_circ_	-0.21101	0.44463
hsa_circ_	-0.26835	0.44464
hsa_circ_	-0.11314	0.444644
hsa_circ_	-0.21471	0.444647
hsa_circ_	-0.16953	0.444699
hsa_circ_	-0.1103	0.444732
hsa_circ_	-0.10969	0.444764
hsa_circ_	0.32112	0.444768
hsa_circ_	-0.18965	0.444806
hsa_circ_	-0.12254	0.444826
hsa_circ_	-0.16952	0.444861
hsa_circ_	-0.17508	0.444867
hsa_circ_	0.139995	0.444868
hsa_circ_	0.22402	0.44487
hsa_circ_	0.245004	0.444872
hsa_circ_	0.136125	0.444913
hsa_circ_	0.195965	0.444919
hsa_circ_	0.329231	0.444934
hsa_circ_	0.098894	0.444935
hsa_circ_	-0.13412	0.444952
hsa_circ_	0.13489	0.44499
hsa_circ_	0.124356	0.444992
hsa_circ_	0.113653	0.444998
hsa_circ_	0.280069	0.445008
hsa_circ_	0.286307	0.445009
hsa_circ_	-0.13006	0.445012
hsa_circ_	0.143965	0.445018
hsa_circ_	-0.18735	0.445025
hsa_circ_	0.276802	0.445043
hsa_circ_	-0.11095	0.445052
hsa_circ_	-0.30778	0.445056
hsa_circ_	0.222107	0.445066
hsa_circ_	0.79967	0.445076
hsa_circ_	-0.11145	0.445082
hsa_circ_	-0.28305	0.44509
hsa_circ_	-0.13365	0.445095
hsa_circ_	0.645986	0.445111
hsa_circ_	-0.30814	0.445127

hsa_circ_	-0.37395	0.445133
hsa_circ_	0.208889	0.445137
hsa_circ_	0.292709	0.445143
hsa_circ_	0.235545	0.445169
hsa_circ_	0.127349	0.445172
hsa_circ_	0.187282	0.445186
hsa_circ_	-0.21105	0.445187
hsa_circ_	0.190204	0.445198
hsa_circ_	-0.12184	0.445199
hsa_circ_	-0.27669	0.445203
hsa_circ_	0.280745	0.445224
hsa_circ_	0.406929	0.445224
hsa_circ_	0.310294	0.445246
hsa_circ_	-0.14502	0.445254
hsa_circ_	0.12851	0.445258
hsa_circ_	-0.1863	0.445288
hsa_circ_	-0.18416	0.445291
hsa_circ_	-0.1953	0.44531
hsa_circ_	-0.21679	0.445336
hsa_circ_	-0.25081	0.44534
hsa_circ_	-0.10904	0.44534
hsa_circ_	-0.18533	0.445352
hsa_circ_	0.178417	0.445353
hsa_circ_	-0.13515	0.445355
hsa_circ_	0.111675	0.445372
hsa_circ_	-0.20068	0.445374
hsa_circ_	0.658821	0.4454
hsa_circ_	-0.12948	0.445408
hsa_circ_	-0.1235	0.445423
hsa_circ_	-0.15514	0.445432
hsa_circ_	-0.1252	0.445449
hsa_circ_	-0.11663	0.445474
hsa_circ_	-0.1517	0.445506
hsa_circ_	0.270833	0.445539
hsa_circ_	0.387196	0.44555
hsa_circ_	0.344056	0.445554
hsa_circ_	-0.17337	0.445594
hsa_circ_	-0.14124	0.445594
hsa_circ_	-0.12853	0.445595
hsa_circ_	-0.20507	0.445605
hsa_circ_	0.402143	0.44562
hsa_circ_	0.567732	0.445677
hsa_circ_	0.420843	0.445677
hsa_circ_	-0.18915	0.445678
hsa_circ_	-0.35219	0.44568
hsa_circ_	-0.09908	0.445697
hsa_circ_	0.158977	0.445703
hsa_circ_	-0.12848	0.445733
hsa_circ_	-0.15586	0.445746
hsa_circ_	0.211138	0.445754
hsa_circ_	0.153351	0.445795
hsa_circ_	-0.10032	0.445796
hsa_circ_	-0.80726	0.445826
hsa_circ_	0.390494	0.445854

hsa_circ_	0.199878	0.445882
hsa_circ_	-0.13443	0.445893
hsa_circ_	-0.10878	0.445905
hsa_circ_	-0.14513	0.445907
hsa_circ_	-0.61792	0.445908
hsa_circ_	0.146262	0.445913
hsa_circ_	-0.14532	0.445944
hsa_circ_	0.153513	0.445958
hsa_circ_	0.212996	0.445977
hsa_circ_	-0.10332	0.446001
hsa_circ_	-0.75159	0.446016
hsa_circ_	0.131045	0.446037
hsa_circ_	0.179636	0.446127
hsa_circ_	0.197949	0.446153
hsa_circ_	0.389477	0.446162
hsa_circ_	0.13849	0.446164
hsa_circ_	-0.52341	0.446168
hsa_circ_	0.274473	0.446173
hsa_circ_	-0.20228	0.446206
hsa_circ_	-0.13758	0.446219
hsa_circ_	-0.17366	0.44625
hsa_circ_	-0.14606	0.44626
hsa_circ_	-0.18082	0.446311
hsa_circ_	-0.22378	0.446327
hsa_circ_	0.166464	0.446334
hsa_circ_	-0.29221	0.446337
hsa_circ_	0.155249	0.446369
hsa_circ_	-0.10997	0.446413
hsa_circ_	0.420721	0.446413
hsa_circ_	0.255774	0.446416
hsa_circ_	-0.34792	0.44643
hsa_circ_	0.105445	0.446434
hsa_circ_	0.195595	0.446456
hsa_circ_	-0.21606	0.446474
hsa_circ_	0.176892	0.446484
hsa_circ_	-0.13367	0.446486
hsa_circ_	-0.14796	0.446489
hsa_circ_	-0.14672	0.446489
hsa_circ_	-0.20914	0.446506
hsa_circ_	-0.14117	0.446508
hsa_circ_	0.16015	0.446511
hsa_circ_	0.13431	0.44652
hsa_circ_	0.110052	0.446534
hsa_circ_	-0.11638	0.446541
hsa_circ_	0.101512	0.446571
hsa_circ_	-0.46616	0.446575
hsa_circ_	0.10715	0.446598
hsa_circ_	-0.0965	0.44663
hsa_circ_	-0.20412	0.446634
hsa_circ_	0.119255	0.446655
hsa_circ_	0.162369	0.446674
hsa_circ_	-0.21638	0.446694
hsa_circ_	-0.21544	0.446697
hsa_circ_	-0.11075	0.446713

hsa_circ_	-0.18338	0.446743
hsa_circ_	0.204196	0.446745
hsa_circ_	0.19983	0.446782
hsa_circ_	-0.20383	0.446786
hsa_circ_	-0.20522	0.446788
hsa_circ_	0.136877	0.446795
hsa_circ_	-0.48112	0.446809
hsa_circ_	-0.36207	0.446812
hsa_circ_	0.276272	0.44684
hsa_circ_	0.171943	0.446846
hsa_circ_	0.115011	0.446854
hsa_circ_	-0.1496	0.446885
hsa_circ_	-0.11967	0.446893
hsa_circ_	0.141544	0.446908
hsa_circ_	0.13432	0.446917
hsa_circ_	0.207372	0.446928
hsa_circ_	0.198784	0.446939
hsa_circ_	0.397608	0.446974
hsa_circ_	0.557917	0.446989
hsa_circ_	0.743363	0.446995
hsa_circ_	-0.11542	0.447006
hsa_circ_	-0.15121	0.447007
hsa_circ_	-0.28245	0.44703
hsa_circ_	0.169882	0.447066
hsa_circ_	-0.10271	0.447076
hsa_circ_	-0.10687	0.447076
hsa_circ_	0.218165	0.44711
hsa_circ_	0.170085	0.447123
hsa_circ_	0.141917	0.447125
hsa_circ_	0.167547	0.447128
hsa_circ_	0.101729	0.447131
hsa_circ_	-0.13772	0.447193
hsa_circ_	-0.10906	0.447194
hsa_circ_	-0.19293	0.447208
hsa_circ_	-0.12881	0.447216
hsa_circ_	-0.43433	0.44725
hsa_circ_	-0.12971	0.447256
hsa_circ_	-0.17903	0.447291
hsa_circ_	0.117322	0.447292
hsa_circ_	0.142635	0.447298
hsa_circ_	0.147373	0.447299
hsa_circ_	-0.17144	0.4473
hsa_circ_	0.180336	0.447307
hsa_circ_	-0.38136	0.447327
hsa_circ_	-0.31788	0.447329
hsa_circ_	-0.13104	0.447385
hsa_circ_	-0.14163	0.447411
hsa_circ_	-0.37453	0.447426
hsa_circ_	0.195468	0.447443
hsa_circ_	0.173284	0.447452
hsa_circ_	0.198556	0.447475
hsa_circ_	-0.22033	0.447481
hsa_circ_	-0.10759	0.447486
hsa_circ_	0.17371	0.447504

hsa_circ_	-0.20474	0.447526
hsa_circ_	-0.42963	0.447528
hsa_circ_	0.111752	0.44755
hsa_circ_	-0.15353	0.447571
hsa_circ_	0.185519	0.447579
hsa_circ_	-0.22318	0.447583
hsa_circ_	-0.2624	0.447598
hsa_circ_	-0.11756	0.447603
hsa_circ_	-0.95236	0.447604
hsa_circ_	0.123778	0.44763
hsa_circ_	-0.2787	0.447634
hsa_circ_	-0.26428	0.447645
hsa_circ_	-0.15521	0.447696
hsa_circ_	0.186578	0.447711
hsa_circ_	-0.17305	0.447723
hsa_circ_	-0.20484	0.447741
hsa_circ_	-0.42622	0.447804
hsa_circ_	-0.14836	0.447816
hsa_circ_	0.214454	0.44782
hsa_circ_	0.2117	0.447854
hsa_circ_	0.267409	0.447868
hsa_circ_	0.106033	0.447879
hsa_circ_	-0.14294	0.447899
hsa_circ_	-0.39362	0.447901
hsa_circ_	-0.12656	0.447903
hsa_circ_	-0.12318	0.447904
hsa_circ_	0.124119	0.447917
hsa_circ_	0.098798	0.447922
hsa_circ_	0.137381	0.447946
hsa_circ_	-0.18401	0.447946
hsa_circ_	-0.2362	0.447952
hsa_circ_	-0.3915	0.447973
hsa_circ_	0.204944	0.447979
hsa_circ_	0.229658	0.447994
hsa_circ_	-0.15315	0.447997
hsa_circ_	-0.12365	0.448008
hsa_circ_	-0.2421	0.448029
hsa_circ_	-0.1295	0.44805
hsa_circ_	0.30031	0.448054
hsa_circ_	-0.51285	0.448058
hsa_circ_	0.105373	0.448066
hsa_circ_	0.411724	0.448067
hsa_circ_	-0.47682	0.448095
hsa_circ_	-0.6385	0.448126
hsa_circ_	-0.21035	0.448153
hsa_circ_	0.361992	0.448169
hsa_circ_	-0.20244	0.448184
hsa_circ_	-0.15016	0.44819
hsa_circ_	0.169372	0.448198
hsa_circ_	0.396579	0.448223
hsa_circ_	-0.13997	0.448254
hsa_circ_	-0.16376	0.448269
hsa_circ_	-0.22077	0.448269
hsa_circ_	-0.23436	0.448293

hsa_circ_	0.101642	0.448311
hsa_circ_	-0.1627	0.448318
hsa_circ_	0.106089	0.44833
hsa_circ_	-0.19668	0.44833
hsa_circ_	-0.20518	0.448358
hsa_circ_	0.215685	0.44837
hsa_circ_	0.276521	0.44841
hsa_circ_	-0.49696	0.448426
hsa_circ_	-0.11842	0.448432
hsa_circ_	0.237103	0.44846
hsa_circ_	0.131504	0.448469
hsa_circ_	-0.32799	0.448476
hsa_circ_	0.13615	0.44848
hsa_circ_	0.110558	0.448506
hsa_circ_	0.141114	0.448532
hsa_circ_	-0.33419	0.448539
hsa_circ_	-0.21407	0.448555
hsa_circ_	-0.14494	0.448559
hsa_circ_	-0.19735	0.448582
hsa_circ_	-0.13447	0.448592
hsa_circ_	0.518657	0.448603
hsa_circ_	-0.1833	0.448681
hsa_circ_	0.480449	0.448692
hsa_circ_	0.100923	0.448695
hsa_circ_	-0.46955	0.448699
hsa_circ_	-0.22781	0.448709
hsa_circ_	-0.103	0.44873
hsa_circ_	-0.17104	0.448734
hsa_circ_	-0.14615	0.44874
hsa_circ_	0.151321	0.448757
hsa_circ_	-0.11346	0.448782
hsa_circ_	-0.47261	0.448815
hsa_circ_	0.100676	0.448823
hsa_circ_	0.320563	0.44883
hsa_circ_	0.123169	0.448871
hsa_circ_	-0.38684	0.448897
hsa_circ_	-0.26572	0.448904
hsa_circ_	0.213199	0.448929
hsa_circ_	0.130314	0.448933
hsa_circ_	0.48372	0.448953
hsa_circ_	-0.10939	0.448965
hsa_circ_	-0.12092	0.448977
hsa_circ_	-0.14479	0.448995
hsa_circ_	-0.10363	0.448996
hsa_circ_	0.174242	0.448997
hsa_circ_	-0.16254	0.449006
hsa_circ_	-0.1494	0.449012
hsa_circ_	-0.14743	0.449031
hsa_circ_	-0.11283	0.449042
hsa_circ_	-0.11767	0.449061
hsa_circ_	0.127704	0.449132
hsa_circ_	-0.11908	0.449132
hsa_circ_	0.124896	0.449137
hsa_circ_	-0.10746	0.449144

hsa_circ_	0.139149	0.449152
hsa_circ_	0.396668	0.449219
hsa_circ_	0.312336	0.449224
hsa_circ_	-0.10365	0.449227
hsa_circ_	0.141998	0.449232
hsa_circ_	0.187537	0.449239
hsa_circ_	-0.1373	0.449257
hsa_circ_	-0.13462	0.449267
hsa_circ_	0.45841	0.449326
hsa_circ_	-0.47168	0.449342
hsa_circ_	-0.14496	0.449345
hsa_circ_	-0.12942	0.449363
hsa_circ_	-0.16195	0.449369
hsa_circ_	0.143817	0.449399
hsa_circ_	-0.12126	0.4494
hsa_circ_	0.140449	0.449402
hsa_circ_	0.149331	0.449403
hsa_circ_	-0.51245	0.449411
hsa_circ_	0.139404	0.449416
hsa_circ_	0.157279	0.449434
hsa_circ_	-0.14165	0.449436
hsa_circ_	0.428332	0.44944
hsa_circ_	-0.12903	0.449443
hsa_circ_	0.446558	0.449464
hsa_circ_	-0.13413	0.449472
hsa_circ_	-0.18302	0.449487
hsa_circ_	-0.10493	0.449507
hsa_circ_	0.19774	0.449509
hsa_circ_	0.203476	0.44951
hsa_circ_	-0.18691	0.449545
hsa_circ_	-0.36629	0.449572
hsa_circ_	-0.38842	0.449591
hsa_circ_	-0.1472	0.449595
hsa_circ_	-0.25322	0.449597
hsa_circ_	-0.1877	0.449603
hsa_circ_	-0.10539	0.449608
hsa_circ_	0.245852	0.449611
hsa_circ_	-0.20261	0.449634
hsa_circ_	-0.28581	0.449645
hsa_circ_	-0.11341	0.449654
hsa_circ_	0.395576	0.44966
hsa_circ_	0.131951	0.449664
hsa_circ_	0.129312	0.449694
hsa_circ_	0.158174	0.449697
hsa_circ_	0.132374	0.449697
hsa_circ_	-0.15129	0.449703
hsa_circ_	0.203944	0.449708
hsa_circ_	0.101451	0.449734
hsa_circ_	0.222758	0.449744
hsa_circ_	-0.16651	0.449784
hsa_circ_	-0.12444	0.449796
hsa_circ_	-0.14127	0.449799
hsa_circ_	0.290684	0.4498
hsa_circ_	-0.17677	0.449803

hsa_circ_	0.121517	0.449814
hsa_circ_	0.309187	0.449817
hsa_circ_	0.240628	0.449818
hsa_circ_	0.155431	0.449831
hsa_circ_	0.547257	0.449836
hsa_circ_	0.459021	0.449838
hsa_circ_	0.121695	0.44984
hsa_circ_	0.129118	0.449841
hsa_circ_	0.362033	0.449851
hsa_circ_	0.307043	0.449876
hsa_circ_	0.463862	0.449876
hsa_circ_	0.123282	0.449883
hsa_circ_	0.108937	0.449902
hsa_circ_	-0.19247	0.449905
hsa_circ_	0.130532	0.44992
hsa_circ_	0.116206	0.449923
hsa_circ_	0.500941	0.449934
hsa_circ_	0.211921	0.44996
hsa_circ_	0.202279	0.44999
hsa_circ_	-0.1762	0.449993
hsa_circ_	-0.18351	0.450003
hsa_circ_	-0.6152	0.450007
hsa_circ_	-0.19106	0.450022
hsa_circ_	-0.11142	0.450056
hsa_circ_	-0.20656	0.450069
hsa_circ_	0.112635	0.450072
hsa_circ_	0.428676	0.450079
hsa_circ_	0.606154	0.450092
hsa_circ_	0.170001	0.450151
hsa_circ_	0.198081	0.450158
hsa_circ_	0.178368	0.450164
hsa_circ_	0.107632	0.450165
hsa_circ_	0.417448	0.450174
hsa_circ_	-0.13319	0.450184
hsa_circ_	0.167857	0.450195
hsa_circ_	-0.36913	0.450224
hsa_circ_	-0.10857	0.450226
hsa_circ_	0.152062	0.450236
hsa_circ_	0.254749	0.450238
hsa_circ_	0.12277	0.45025
hsa_circ_	-0.53993	0.450255
hsa_circ_	0.116618	0.450255
hsa_circ_	0.264056	0.450261
hsa_circ_	-0.22422	0.450307
hsa_circ_	-0.12164	0.450322
hsa_circ_	-0.61809	0.450322
hsa_circ_	-0.15999	0.45033
hsa_circ_	0.189633	0.450335
hsa_circ_	0.337809	0.450374
hsa_circ_	-0.32748	0.450377
hsa_circ_	0.214521	0.450379
hsa_circ_	-0.26732	0.45039
hsa_circ_	0.179031	0.450396
hsa_circ_	0.443673	0.450404

hsa_circ_	0.15826	0.450417
hsa_circ_	-0.12644	0.450419
hsa_circ_	-0.25609	0.450433
hsa_circ_	-0.2584	0.450436
hsa_circ_	0.122265	0.450444
hsa_circ_	-0.12888	0.450448
hsa_circ_	-0.35445	0.450453
hsa_circ_	-0.45576	0.450453
hsa_circ_	0.192497	0.450455
hsa_circ_	-0.1355	0.450458
hsa_circ_	-0.16732	0.450481
hsa_circ_	-0.11151	0.450497
hsa_circ_	-0.14457	0.450518
hsa_circ_	0.147052	0.450576
hsa_circ_	-0.27303	0.450594
hsa_circ_	0.396658	0.450603
hsa_circ_	-0.14213	0.450605
hsa_circ_	0.182785	0.450612
hsa_circ_	0.105983	0.450617
hsa_circ_	-0.12272	0.450638
hsa_circ_	-0.21078	0.45064
hsa_circ_	-0.23473	0.450663
hsa_circ_	0.294738	0.450665
hsa_circ_	-0.17701	0.450669
hsa_circ_	-0.10487	0.450673
hsa_circ_	-0.11389	0.450676
hsa_circ_	0.326762	0.450701
hsa_circ_	0.449	0.450702
hsa_circ_	0.18922	0.450704
hsa_circ_	0.300711	0.450706
hsa_circ_	-0.11447	0.450709
hsa_circ_	0.973843	0.450748
hsa_circ_	-0.10858	0.450749
hsa_circ_	-0.14263	0.450751
hsa_circ_	-0.33792	0.450752
hsa_circ_	-0.14141	0.450767
hsa_circ_	-0.11351	0.450772
hsa_circ_	0.146376	0.450787
hsa_circ_	-0.1603	0.450805
hsa_circ_	-0.13678	0.450822
hsa_circ_	-0.10696	0.450846
hsa_circ_	0.1402	0.450863
hsa_circ_	-0.10861	0.450885
hsa_circ_	0.200261	0.45092
hsa_circ_	0.183686	0.450944
hsa_circ_	-0.14241	0.450949
hsa_circ_	-0.16491	0.450982
hsa_circ_	-0.37637	0.450987
hsa_circ_	-0.10082	0.451023
hsa_circ_	-0.24067	0.451051
hsa_circ_	-0.1141	0.451052
hsa_circ_	-0.66093	0.451056
hsa_circ_	-0.29206	0.451066
hsa_circ_	-0.1021	0.451114

hsa_circ_	0.359429	0.451134
hsa_circ_	0.308795	0.451161
hsa_circ_	0.155246	0.451169
hsa_circ_	-0.42666	0.451189
hsa_circ_	-0.12551	0.451195
hsa_circ_	-0.12308	0.451199
hsa_circ_	0.12193	0.45121
hsa_circ_	0.152254	0.451234
hsa_circ_	0.136911	0.451237
hsa_circ_	0.17345	0.451249
hsa_circ_	-0.15312	0.451252
hsa_circ_	-0.32204	0.451258
hsa_circ_	0.462131	0.451273
hsa_circ_	0.174695	0.451294
hsa_circ_	0.111326	0.451302
hsa_circ_	0.144731	0.451306
hsa_circ_	0.196276	0.451311
hsa_circ_	0.109531	0.451312
hsa_circ_	0.108063	0.451312
hsa_circ_	-0.47175	0.451326
hsa_circ_	-0.11524	0.451329
hsa_circ_	0.266298	0.451349
hsa_circ_	-0.44817	0.451382
hsa_circ_	-0.12618	0.451385
hsa_circ_	2.073336	0.451407
hsa_circ_	0.242602	0.451432
hsa_circ_	0.130883	0.451449
hsa_circ_	0.132539	0.45146
hsa_circ_	-0.24281	0.451479
hsa_circ_	0.101445	0.45149
hsa_circ_	-0.2436	0.451498
hsa_circ_	0.145283	0.451508
hsa_circ_	0.161761	0.45152
hsa_circ_	0.358656	0.451561
hsa_circ_	-0.23708	0.451567
hsa_circ_	-0.15852	0.451583
hsa_circ_	-0.29146	0.451586
hsa_circ_	-0.19038	0.451594
hsa_circ_	0.289202	0.451625
hsa_circ_	-0.26447	0.451631
hsa_circ_	-0.13897	0.451685
hsa_circ_	0.256931	0.451693
hsa_circ_	0.146902	0.451709
hsa_circ_	-0.22142	0.451736
hsa_circ_	-0.15396	0.451743
hsa_circ_	-0.23522	0.451762
hsa_circ_	0.178689	0.451774
hsa_circ_	0.113437	0.451786
hsa_circ_	-0.23323	0.451788
hsa_circ_	0.134874	0.451811
hsa_circ_	0.124559	0.451817
hsa_circ_	-0.15729	0.451839
hsa_circ_	-0.382	0.451846
hsa_circ_	-0.18491	0.451872

hsa_circ_	-0.22205	0.451888
hsa_circ_	0.140751	0.45192
hsa_circ_	0.15122	0.451933
hsa_circ_	-0.13949	0.451937
hsa_circ_	-0.35462	0.451941
hsa_circ_	0.281466	0.451943
hsa_circ_	-0.20695	0.451961
hsa_circ_	-0.30952	0.451965
hsa_circ_	0.10569	0.451987
hsa_circ_	-0.10612	0.452001
hsa_circ_	0.131822	0.452001
hsa_circ_	-0.35379	0.452005
hsa_circ_	-0.28025	0.452035
hsa_circ_	0.327579	0.452043
hsa_circ_	0.393344	0.452045
hsa_circ_	0.458561	0.45205
hsa_circ_	-0.18082	0.452051
hsa_circ_	0.122508	0.452052
hsa_circ_	-0.20801	0.452055
hsa_circ_	-0.1695	0.452075
hsa_circ_	0.23301	0.452084
hsa_circ_	-0.30338	0.452101
hsa_circ_	0.161335	0.452128
hsa_circ_	-0.64323	0.45225
hsa_circ_	0.252092	0.452256
hsa_circ_	0.171744	0.452273
hsa_circ_	-0.1389	0.452289
hsa_circ_	0.125998	0.4523
hsa_circ_	-0.14335	0.45232
hsa_circ_	0.177581	0.452321
hsa_circ_	0.170904	0.452345
hsa_circ_	0.193729	0.452379
hsa_circ_	0.152099	0.452391
hsa_circ_	0.13284	0.452402
hsa_circ_	-0.11267	0.452412
hsa_circ_	0.120649	0.452416
hsa_circ_	-0.25481	0.452416
hsa_circ_	-0.22675	0.452418
hsa_circ_	-0.17975	0.452436
hsa_circ_	0.20461	0.45244
hsa_circ_	0.122747	0.452454
hsa_circ_	0.165838	0.45248
hsa_circ_	0.389736	0.452498
hsa_circ_	0.148627	0.452502
hsa_circ_	0.102596	0.452516
hsa_circ_	-0.51418	0.452531
hsa_circ_	-0.26062	0.452539
hsa_circ_	0.17272	0.452543
hsa_circ_	0.143326	0.452572
hsa_circ_	0.160177	0.45259
hsa_circ_	-0.57385	0.452614
hsa_circ_	-0.15126	0.452625
hsa_circ_	-0.13911	0.45264
hsa_circ_	-0.10114	0.452664

hsa_circ_	-0.13234	0.452668
hsa_circ_	-0.16912	0.452675
hsa_circ_	0.497972	0.452675
hsa_circ_	0.106673	0.452681
hsa_circ_	-0.28774	0.452695
hsa_circ_	0.214095	0.452739
hsa_circ_	-0.22939	0.452754
hsa_circ_	-0.23807	0.452782
hsa_circ_	0.099827	0.452797
hsa_circ_	0.15939	0.452804
hsa_circ_	-0.11367	0.45283
hsa_circ_	0.476581	0.452833
hsa_circ_	-0.16901	0.45287
hsa_circ_	-0.60959	0.452871
hsa_circ_	0.104083	0.452875
hsa_circ_	-0.14223	0.452881
hsa_circ_	-0.13962	0.452906
hsa_circ_	0.123971	0.452912
hsa_circ_	0.103301	0.452922
hsa_circ_	0.202157	0.452956
hsa_circ_	-0.13138	0.452959
hsa_circ_	-0.144	0.452962
hsa_circ_	0.405841	0.452968
hsa_circ_	-0.11238	0.452968
hsa_circ_	-0.13694	0.45297
hsa_circ_	-0.10247	0.45297
hsa_circ_	0.163471	0.452988
hsa_circ_	-0.12438	0.453008
hsa_circ_	-0.90172	0.453015
hsa_circ_	-0.1432	0.453019
hsa_circ_	0.329895	0.45302
hsa_circ_	-0.11485	0.453031
hsa_circ_	-0.17864	0.453032
hsa_circ_	-0.1275	0.453116
hsa_circ_	0.169342	0.45315
hsa_circ_	-0.17865	0.45315
hsa_circ_	0.145957	0.453219
hsa_circ_	-0.21387	0.45323
hsa_circ_	0.100096	0.453238
hsa_circ_	0.120695	0.453244
hsa_circ_	-0.15264	0.453264
hsa_circ_	-0.33251	0.453265
hsa_circ_	0.190315	0.453308
hsa_circ_	-0.15186	0.453327
hsa_circ_	0.161739	0.453341
hsa_circ_	0.125219	0.453379
hsa_circ_	0.140668	0.453387
hsa_circ_	0.134983	0.453404
hsa_circ_	-0.49209	0.453409
hsa_circ_	0.125933	0.453432
hsa_circ_	0.130571	0.453433
hsa_circ_	0.211036	0.453435
hsa_circ_	0.149956	0.453456
hsa_circ_	-0.18183	0.453458

hsa_circ_	-0.30953	0.453474
hsa_circ_	-0.20242	0.453484
hsa_circ_	0.169523	0.453494
hsa_circ_	0.109445	0.453498
hsa_circ_	-0.0978	0.453504
hsa_circ_	0.166389	0.453521
hsa_circ_	-0.15764	0.453528
hsa_circ_	-0.12636	0.45353
hsa_circ_	-0.47143	0.45354
hsa_circ_	0.360422	0.453546
hsa_circ_	-0.15973	0.453552
hsa_circ_	0.118783	0.453557
hsa_circ_	-0.30007	0.453577
hsa_circ_	0.113144	0.453582
hsa_circ_	0.232675	0.453588
hsa_circ_	0.146992	0.453602
hsa_circ_	-0.22139	0.453637
hsa_circ_	-0.0966	0.453647
hsa_circ_	0.131827	0.453658
hsa_circ_	-0.47245	0.453673
hsa_circ_	0.112785	0.453681
hsa_circ_	0.550364	0.453685
hsa_circ_	0.233559	0.453713
hsa_circ_	0.21254	0.453729
hsa_circ_	0.277188	0.453757
hsa_circ_	0.257083	0.453796
hsa_circ_	-0.19631	0.45381
hsa_circ_	0.203384	0.453812
hsa_circ_	-0.15105	0.453839
hsa_circ_	0.524469	0.453847
hsa_circ_	-0.1468	0.453871
hsa_circ_	0.153058	0.45388
hsa_circ_	0.217704	0.453906
hsa_circ_	0.343335	0.453918
hsa_circ_	0.195324	0.453931
hsa_circ_	-0.11651	0.453946
hsa_circ_	0.134317	0.453967
hsa_circ_	-0.17596	0.453985
hsa_circ_	-0.2191	0.454002
hsa_circ_	-0.43211	0.454004
hsa_circ_	-0.11483	0.454011
hsa_circ_	0.279771	0.454036
hsa_circ_	0.233745	0.454037
hsa_circ_	-0.42294	0.454044
hsa_circ_	0.128012	0.454044
hsa_circ_	-0.15193	0.454045
hsa_circ_	-0.10437	0.454052
hsa_circ_	-0.23371	0.454067
hsa_circ_	-0.55964	0.454076
hsa_circ_	0.108427	0.454097
hsa_circ_	-0.24841	0.454117
hsa_circ_	0.159478	0.454118
hsa_circ_	0.303377	0.454128
hsa_circ_	0.119194	0.454132

hsa_circ_	-0.12279	0.454143
hsa_circ_	0.307729	0.454143
hsa_circ_	0.148841	0.454145
hsa_circ_	0.167154	0.454155
hsa_circ_	-0.17659	0.454175
hsa_circ_	-0.12234	0.454177
hsa_circ_	0.115915	0.454184
hsa_circ_	0.241185	0.454187
hsa_circ_	0.267946	0.454203
hsa_circ_	-0.14161	0.454204
hsa_circ_	-0.39692	0.454209
hsa_circ_	-0.25323	0.45425
hsa_circ_	-0.20616	0.454262
hsa_circ_	0.215458	0.45427
hsa_circ_	0.205416	0.454284
hsa_circ_	0.194833	0.4543
hsa_circ_	-0.25658	0.454304
hsa_circ_	0.158878	0.454321
hsa_circ_	-0.13962	0.454329
hsa_circ_	-0.16017	0.454336
hsa_circ_	-0.65438	0.454347
hsa_circ_	0.240384	0.454363
hsa_circ_	0.101271	0.45437
hsa_circ_	-0.37714	0.454384
hsa_circ_	-0.09817	0.454398
hsa_circ_	0.114448	0.454404
hsa_circ_	0.230633	0.454422
hsa_circ_	-0.15731	0.454422
hsa_circ_	-0.27083	0.454423
hsa_circ_	0.136468	0.454448
hsa_circ_	0.178008	0.454484
hsa_circ_	-0.2042	0.454507
hsa_circ_	-0.11784	0.454515
hsa_circ_	0.722057	0.454515
hsa_circ_	-0.17199	0.454522
hsa_circ_	0.103207	0.454525
hsa_circ_	0.159206	0.454528
hsa_circ_	-0.44055	0.454573
hsa_circ_	0.131076	0.45459
hsa_circ_	-0.30335	0.454607
hsa_circ_	0.114699	0.454609
hsa_circ_	-0.15977	0.454614
hsa_circ_	-0.33091	0.45462
hsa_circ_	-0.19081	0.454626
hsa_circ_	-0.28469	0.454644
hsa_circ_	-0.10038	0.454647
hsa_circ_	-0.19229	0.454669
hsa_circ_	0.172737	0.454679
hsa_circ_	0.162779	0.454691
hsa_circ_	-0.40321	0.454705
hsa_circ_	-0.11829	0.454711
hsa_circ_	-0.81841	0.454711
hsa_circ_	-0.18476	0.454724
hsa_circ_	-0.28587	0.454733

hsa_circ_	-0.19195	0.454741
hsa_circ_	0.170535	0.45475
hsa_circ_	0.167921	0.454757
hsa_circ_	0.138046	0.454765
hsa_circ_	-0.18456	0.454766
hsa_circ_	-0.11595	0.454796
hsa_circ_	-0.17759	0.454826
hsa_circ_	0.240682	0.454843
hsa_circ_	-0.19659	0.454847
hsa_circ_	-0.19388	0.45485
hsa_circ_	-0.27464	0.454856
hsa_circ_	0.125602	0.454868
hsa_circ_	0.200855	0.454879
hsa_circ_	-0.16911	0.454895
hsa_circ_	0.286017	0.454915
hsa_circ_	-0.14802	0.454954
hsa_circ_	0.145395	0.454959
hsa_circ_	0.356784	0.454974
hsa_circ_	0.140061	0.454982
hsa_circ_	0.217284	0.454987
hsa_circ_	-0.11791	0.454989
hsa_circ_	-0.11229	0.455002
hsa_circ_	0.155619	0.45501
hsa_circ_	-0.14185	0.455027
hsa_circ_	0.301223	0.455037
hsa_circ_	-0.18261	0.455043
hsa_circ_	0.12035	0.455056
hsa_circ_	-0.28832	0.455057
hsa_circ_	-0.13627	0.455061
hsa_circ_	0.404942	0.455074
hsa_circ_	-0.50966	0.455076
hsa_circ_	-0.12059	0.455079
hsa_circ_	-0.11701	0.455092
hsa_circ_	-0.34127	0.455093
hsa_circ_	-0.09463	0.455121
hsa_circ_	-0.21989	0.455121
hsa_circ_	0.139035	0.455149
hsa_circ_	-0.30614	0.455161
hsa_circ_	-0.15409	0.455171
hsa_circ_	-0.10791	0.455178
hsa_circ_	-0.12184	0.455188
hsa_circ_	0.132438	0.455192
hsa_circ_	-0.16825	0.455205
hsa_circ_	-0.13873	0.455214
hsa_circ_	0.130782	0.455215
hsa_circ_	0.151415	0.455221
hsa_circ_	-0.24737	0.455233
hsa_circ_	-0.13279	0.455249
hsa_circ_	1.069845	0.455275
hsa_circ_	-0.14703	0.45528
hsa_circ_	-0.18241	0.45528
hsa_circ_	0.174572	0.455292
hsa_circ_	-0.32296	0.45532
hsa_circ_	-0.14441	0.455325

hsa_circ_	-0.12351	0.455325
hsa_circ_	0.302471	0.455336
hsa_circ_	-0.12382	0.455357
hsa_circ_	0.302052	0.455362
hsa_circ_	-0.3081	0.455376
hsa_circ_	0.102321	0.455421
hsa_circ_	0.117854	0.455437
hsa_circ_	0.158303	0.455439
hsa_circ_	-0.35435	0.455484
hsa_circ_	-0.12661	0.455507
hsa_circ_	-0.96127	0.455516
hsa_circ_	-0.12497	0.455537
hsa_circ_	0.329199	0.455538
hsa_circ_	0.267321	0.455554
hsa_circ_	0.149299	0.45556
hsa_circ_	0.207415	0.455563
hsa_circ_	0.114493	0.455579
hsa_circ_	-0.20004	0.455579
hsa_circ_	-0.32411	0.455595
hsa_circ_	-0.28483	0.45561
hsa_circ_	-0.41542	0.455622
hsa_circ_	-0.20013	0.455624
hsa_circ_	-0.14039	0.455632
hsa_circ_	-0.57452	0.455637
hsa_circ_	0.118528	0.45565
hsa_circ_	-0.19264	0.45568
hsa_circ_	-0.38728	0.455689
hsa_circ_	-0.21368	0.455732
hsa_circ_	0.143625	0.455746
hsa_circ_	0.111214	0.455749
hsa_circ_	-0.10067	0.455753
hsa_circ_	0.21103	0.455756
hsa_circ_	0.106019	0.455768
hsa_circ_	0.464184	0.455781
hsa_circ_	-0.4497	0.455786
hsa_circ_	-0.11648	0.455809
hsa_circ_	0.391661	0.455837
hsa_circ_	0.164703	0.455847
hsa_circ_	-0.10399	0.455865
hsa_circ_	-0.13727	0.455866
hsa_circ_	0.14631	0.455866
hsa_circ_	-0.13261	0.45589
hsa_circ_	0.241729	0.455909
hsa_circ_	0.150749	0.455921
hsa_circ_	-0.18073	0.455944
hsa_circ_	-0.16299	0.455965
hsa_circ_	-0.10529	0.456033
hsa_circ_	-0.13779	0.456042
hsa_circ_	-0.15954	0.456071
hsa_circ_	0.234693	0.456075
hsa_circ_	0.202665	0.456088
hsa_circ_	0.197633	0.456088
hsa_circ_	-0.28934	0.456112
hsa_circ_	0.09884	0.456126

hsa_circ_	0.159795	0.456127
hsa_circ_	0.132095	0.456135
hsa_circ_	-0.17871	0.456152
hsa_circ_	0.119031	0.456153
hsa_circ_	-0.16958	0.456177
hsa_circ_	0.146394	0.456179
hsa_circ_	0.120594	0.456197
hsa_circ_	0.251989	0.456211
hsa_circ_	0.244891	0.456215
hsa_circ_	-0.23225	0.456255
hsa_circ_	-0.20844	0.456283
hsa_circ_	-0.1785	0.456292
hsa_circ_	-0.12988	0.456295
hsa_circ_	0.143024	0.456302
hsa_circ_	0.472761	0.456303
hsa_circ_	0.171505	0.456305
hsa_circ_	0.305424	0.456307
hsa_circ_	-0.43267	0.45632
hsa_circ_	0.162326	0.456337
hsa_circ_	-0.11669	0.456352
hsa_circ_	0.125189	0.456372
hsa_circ_	-0.11	0.456378
hsa_circ_	0.114143	0.456394
hsa_circ_	0.818945	0.456422
hsa_circ_	0.237376	0.456435
hsa_circ_	-0.10462	0.456453
hsa_circ_	-0.12514	0.456475
hsa_circ_	0.235176	0.456477
hsa_circ_	0.26898	0.45649
hsa_circ_	-0.18942	0.456497
hsa_circ_	0.187112	0.456499
hsa_circ_	0.201844	0.456501
hsa_circ_	-0.31288	0.456509
hsa_circ_	0.151523	0.456527
hsa_circ_	0.165878	0.456528
hsa_circ_	0.127478	0.456545
hsa_circ_	0.103757	0.456547
hsa_circ_	0.652291	0.456548
hsa_circ_	-0.13897	0.456574
hsa_circ_	0.220572	0.456582
hsa_circ_	0.379316	0.456593
hsa_circ_	-0.15962	0.456605
hsa_circ_	0.190565	0.456616
hsa_circ_	0.127809	0.456635
hsa_circ_	-0.14779	0.456636
hsa_circ_	-0.11371	0.456648
hsa_circ_	-0.16965	0.456651
hsa_circ_	-0.10754	0.456652
hsa_circ_	0.469048	0.456677
hsa_circ_	0.226013	0.456701
hsa_circ_	-0.22694	0.456711
hsa_circ_	0.33686	0.456738
hsa_circ_	-0.2936	0.456758
hsa_circ_	-0.17821	0.456763

hsa_circ_	0.223108	0.456769
hsa_circ_	0.244817	0.456786
hsa_circ_	0.568833	0.456793
hsa_circ_	0.158805	0.456794
hsa_circ_	0.12543	0.456801
hsa_circ_	0.154396	0.456868
hsa_circ_	-0.42924	0.456888
hsa_circ_	-0.14803	0.456893
hsa_circ_	0.108287	0.456894
hsa_circ_	0.109111	0.456919
hsa_circ_	0.156991	0.456922
hsa_circ_	0.101867	0.456926
hsa_circ_	-0.16641	0.45695
hsa_circ_	-0.31611	0.456955
hsa_circ_	0.218694	0.456968
hsa_circ_	-0.11903	0.456987
hsa_circ_	0.284156	0.456992
hsa_circ_	0.201832	0.457011
hsa_circ_	0.180632	0.457014
hsa_circ_	0.190662	0.457016
hsa_circ_	-0.36468	0.457046
hsa_circ_	0.125297	0.457047
hsa_circ_	0.738876	0.457061
hsa_circ_	-0.12343	0.457075
hsa_circ_	-0.17805	0.457096
hsa_circ_	-0.11105	0.457129
hsa_circ_	0.112233	0.457134
hsa_circ_	-0.22432	0.457141
hsa_circ_	0.173341	0.457147
hsa_circ_	0.121485	0.45717
hsa_circ_	-0.16755	0.457172
hsa_circ_	0.415366	0.457216
hsa_circ_	0.229726	0.45722
hsa_circ_	-0.13554	0.457235
hsa_circ_	-0.11413	0.457235
hsa_circ_	0.252497	0.457237
hsa_circ_	-0.10446	0.457261
hsa_circ_	0.132195	0.457263
hsa_circ_	-0.10921	0.457265
hsa_circ_	-0.32112	0.457265
hsa_circ_	0.252763	0.457265
hsa_circ_	0.124584	0.457267
hsa_circ_	-0.37169	0.457267
hsa_circ_	0.399709	0.457271
hsa_circ_	-0.2202	0.457277
hsa_circ_	-0.19876	0.457279
hsa_circ_	0.175931	0.45728
hsa_circ_	-0.41945	0.457315
hsa_circ_	-0.1597	0.45732
hsa_circ_	0.126443	0.457324
hsa_circ_	-0.11736	0.457335
hsa_circ_	0.155488	0.457378
hsa_circ_	-0.59187	0.457404
hsa_circ_	-0.16687	0.457409

hsa_circ_	0.165289	0.457415
hsa_circ_	0.096198	0.45742
hsa_circ_	0.26788	0.457443
hsa_circ_	-0.3421	0.457447
hsa_circ_	-0.24052	0.457475
hsa_circ_	0.145432	0.457475
hsa_circ_	0.122985	0.457485
hsa_circ_	-0.15105	0.457492
hsa_circ_	0.119863	0.457503
hsa_circ_	-0.12144	0.457505
hsa_circ_	0.254665	0.457513
hsa_circ_	0.141935	0.457525
hsa_circ_	0.29955	0.457532
hsa_circ_	0.285707	0.457533
hsa_circ_	-0.41214	0.457534
hsa_circ_	-0.13226	0.457538
hsa_circ_	0.141768	0.457551
hsa_circ_	-0.14958	0.457558
hsa_circ_	-0.28071	0.457577
hsa_circ_	0.324162	0.45758
hsa_circ_	0.114652	0.4576
hsa_circ_	0.235012	0.457619
hsa_circ_	0.433307	0.457634
hsa_circ_	-0.1397	0.457639
hsa_circ_	0.463766	0.457657
hsa_circ_	-0.11176	0.457659
hsa_circ_	-0.17426	0.45766
hsa_circ_	0.180756	0.457662
hsa_circ_	0.335814	0.457691
hsa_circ_	0.558253	0.457697
hsa_circ_	-0.1421	0.457732
hsa_circ_	-0.11393	0.457738
hsa_circ_	-0.14393	0.457773
hsa_circ_	0.215079	0.457811
hsa_circ_	0.141113	0.45782
hsa_circ_	0.168674	0.457838
hsa_circ_	-0.22425	0.457861
hsa_circ_	-0.44678	0.457886
hsa_circ_	-0.30718	0.457904
hsa_circ_	0.104446	0.457905
hsa_circ_	0.102894	0.45791
hsa_circ_	0.366919	0.457926
hsa_circ_	0.174434	0.457961
hsa_circ_	-0.47254	0.457972
hsa_circ_	-0.11337	0.457984
hsa_circ_	-0.18366	0.458021
hsa_circ_	0.263773	0.458023
hsa_circ_	0.136587	0.458031
hsa_circ_	0.174004	0.458047
hsa_circ_	0.11027	0.458054
hsa_circ_	0.127862	0.458076
hsa_circ_	0.3654	0.458089
hsa_circ_	-0.27796	0.458093
hsa_circ_	-0.13534	0.458101

hsa_circ_	0.130982	0.458119
hsa_circ_	-1.51645	0.458129
hsa_circ_	-0.16224	0.458141
hsa_circ_	0.210493	0.458145
hsa_circ_	-0.24969	0.458148
hsa_circ_	0.110752	0.458149
hsa_circ_	0.107215	0.45815
hsa_circ_	-0.14312	0.458193
hsa_circ_	-0.12341	0.458193
hsa_circ_	0.144807	0.458218
hsa_circ_	0.117594	0.458225
hsa_circ_	-0.24453	0.458228
hsa_circ_	-0.59017	0.458259
hsa_circ_	-0.14729	0.45826
hsa_circ_	-0.11002	0.458264
hsa_circ_	-0.2092	0.458265
hsa_circ_	-0.51936	0.458273
hsa_circ_	-0.11736	0.458323
hsa_circ_	-0.14451	0.458333
hsa_circ_	-0.12823	0.458352
hsa_circ_	0.136377	0.45837
hsa_circ_	0.446901	0.458377
hsa_circ_	0.124081	0.45839
hsa_circ_	0.195942	0.458397
hsa_circ_	-0.30224	0.458436
hsa_circ_	-0.20098	0.458447
hsa_circ_	-0.12546	0.458459
hsa_circ_	-0.12503	0.45846
hsa_circ_	-0.15782	0.458482
hsa_circ_	0.306326	0.458492
hsa_circ_	-1.41453	0.458498
hsa_circ_	0.124033	0.458499
hsa_circ_	-0.2899	0.45853
hsa_circ_	-0.19666	0.458549
hsa_circ_	-0.33385	0.458563
hsa_circ_	0.124351	0.458566
hsa_circ_	-1.09914	0.458622
hsa_circ_	0.159071	0.458623
hsa_circ_	-0.138	0.458627
hsa_circ_	0.496856	0.458645
hsa_circ_	-0.17417	0.458646
hsa_circ_	-0.18687	0.45867
hsa_circ_	-0.17241	0.458691
hsa_circ_	-0.17938	0.458693
hsa_circ_	0.140374	0.458706
hsa_circ_	-0.37047	0.458737
hsa_circ_	-0.15826	0.458748
hsa_circ_	-0.33202	0.458754
hsa_circ_	-0.18994	0.458763
hsa_circ_	0.122087	0.458773
hsa_circ_	0.147152	0.458776
hsa_circ_	0.180338	0.458779
hsa_circ_	-0.13564	0.458786
hsa_circ_	0.197097	0.458813

hsa_circ_	0.129889	0.458817
hsa_circ_	0.292158	0.458819
hsa_circ_	0.173421	0.45882
hsa_circ_	-0.09954	0.458849
hsa_circ_	-0.12588	0.458851
hsa_circ_	0.176295	0.45886
hsa_circ_	0.11145	0.458924
hsa_circ_	0.117257	0.458931
hsa_circ_	-0.31465	0.458943
hsa_circ_	-0.16955	0.458957
hsa_circ_	-0.15836	0.458972
hsa_circ_	-0.11818	0.458977
hsa_circ_	-0.1199	0.458999
hsa_circ_	0.176655	0.459024
hsa_circ_	0.239242	0.459027
hsa_circ_	-0.19855	0.459034
hsa_circ_	0.349911	0.459066
hsa_circ_	0.124607	0.459072
hsa_circ_	-0.16745	0.459091
hsa_circ_	-0.25618	0.459103
hsa_circ_	-0.2284	0.459103
hsa_circ_	0.132783	0.459116
hsa_circ_	-0.3259	0.459117
hsa_circ_	0.167937	0.459125
hsa_circ_	0.279078	0.459143
hsa_circ_	0.301212	0.459152
hsa_circ_	-0.10887	0.459156
hsa_circ_	0.149845	0.459184
hsa_circ_	0.126784	0.459195
hsa_circ_	-1.15572	0.459199
hsa_circ_	-0.2446	0.459205
hsa_circ_	-0.1545	0.459211
hsa_circ_	0.471116	0.459219
hsa_circ_	-0.1646	0.459227
hsa_circ_	0.119226	0.45923
hsa_circ_	0.114572	0.459239
hsa_circ_	0.202181	0.459281
hsa_circ_	0.12976	0.459331
hsa_circ_	-0.18345	0.45934
hsa_circ_	0.131292	0.459349
hsa_circ_	-0.13019	0.459349
hsa_circ_	-0.11021	0.459371
hsa_circ_	-0.10021	0.45938
hsa_circ_	-0.10913	0.459396
hsa_circ_	-0.10603	0.459414
hsa_circ_	0.118456	0.459424
hsa_circ_	0.250605	0.459447
hsa_circ_	0.251831	0.459465
hsa_circ_	0.138316	0.4595
hsa_circ_	-0.17673	0.45951
hsa_circ_	-0.17056	0.459518
hsa_circ_	0.113173	0.459519
hsa_circ_	-0.13554	0.459523
hsa_circ_	0.104456	0.459537

hsa_circ_	-0.1892	0.459551
hsa_circ_	-0.16847	0.459557
hsa_circ_	-0.14997	0.459569
hsa_circ_	-0.84085	0.459575
hsa_circ_	-0.13247	0.459581
hsa_circ_	-0.15021	0.459602
hsa_circ_	0.294503	0.459603
hsa_circ_	0.193522	0.459662
hsa_circ_	0.201464	0.459666
hsa_circ_	0.11896	0.459681
hsa_circ_	-0.60142	0.459685
hsa_circ_	-0.15698	0.459689
hsa_circ_	-0.16036	0.459692
hsa_circ_	-0.47333	0.459701
hsa_circ_	0.424265	0.459703
hsa_circ_	0.348758	0.459765
hsa_circ_	-0.36348	0.459779
hsa_circ_	0.165549	0.459786
hsa_circ_	-0.16993	0.459803
hsa_circ_	-0.10666	0.459809
hsa_circ_	0.147014	0.459813
hsa_circ_	0.130367	0.459813
hsa_circ_	-0.1168	0.459844
hsa_circ_	0.127325	0.459852
hsa_circ_	0.158939	0.459853
hsa_circ_	0.226682	0.459853
hsa_circ_	-0.20062	0.459862
hsa_circ_	0.134814	0.459865
hsa_circ_	-0.31557	0.459883
hsa_circ_	-0.10558	0.459886
hsa_circ_	-0.10838	0.459895
hsa_circ_	-0.19981	0.459916
hsa_circ_	0.227718	0.459965
hsa_circ_	-0.3946	0.460028
hsa_circ_	0.187141	0.460034
hsa_circ_	-0.1221	0.460038
hsa_circ_	-0.12811	0.460051
hsa_circ_	0.252501	0.460056
hsa_circ_	-0.10821	0.460062
hsa_circ_	-0.1427	0.46008
hsa_circ_	-0.21277	0.460099
hsa_circ_	0.427253	0.460106
hsa_circ_	0.125141	0.460196
hsa_circ_	0.123783	0.46022
hsa_circ_	0.276558	0.460227
hsa_circ_	0.427604	0.460243
hsa_circ_	-0.10672	0.460244
hsa_circ_	0.228957	0.460252
hsa_circ_	-0.39418	0.460268
hsa_circ_	-0.14469	0.460309
hsa_circ_	-0.18207	0.460315
hsa_circ_	-0.25509	0.460321
hsa_circ_	0.308676	0.460321
hsa_circ_	-0.42982	0.460329

hsa_circ_	0.109043	0.460344
hsa_circ_	0.106514	0.460366
hsa_circ_	0.139014	0.460395
hsa_circ_	0.145286	0.460449
hsa_circ_	-0.13982	0.46045
hsa_circ_	-0.30376	0.460454
hsa_circ_	0.233785	0.46046
hsa_circ_	0.101436	0.460486
hsa_circ_	-0.11735	0.460494
hsa_circ_	-0.39738	0.460498
hsa_circ_	0.18327	0.460504
hsa_circ_	-0.22514	0.460521
hsa_circ_	0.106703	0.460526
hsa_circ_	-0.1152	0.460527
hsa_circ_	-0.48326	0.460546
hsa_circ_	-0.17338	0.460548
hsa_circ_	-0.38786	0.46055
hsa_circ_	-0.26601	0.46058
hsa_circ_	0.272654	0.460582
hsa_circ_	0.114794	0.460593
hsa_circ_	-0.2505	0.460602
hsa_circ_	-0.12338	0.460608
hsa_circ_	-0.12005	0.460636
hsa_circ_	0.172332	0.460646
hsa_circ_	0.115994	0.460728
hsa_circ_	0.146761	0.460739
hsa_circ_	0.19629	0.460741
hsa_circ_	-0.12873	0.460754
hsa_circ_	0.248037	0.460756
hsa_circ_	-0.21642	0.460757
hsa_circ_	-0.39235	0.460797
hsa_circ_	-0.13303	0.460807
hsa_circ_	-0.14863	0.46083
hsa_circ_	0.436455	0.46083
hsa_circ_	-0.11568	0.460838
hsa_circ_	0.12636	0.460842
hsa_circ_	0.143741	0.460849
hsa_circ_	-0.39218	0.460852
hsa_circ_	0.130945	0.460865
hsa_circ_	0.167908	0.460866
hsa_circ_	-0.2095	0.460873
hsa_circ_	-0.31478	0.460879
hsa_circ_	0.104146	0.460903
hsa_circ_	0.118762	0.46094
hsa_circ_	0.448861	0.460941
hsa_circ_	0.245693	0.460981
hsa_circ_	0.484163	0.461005
hsa_circ_	-0.14271	0.461026
hsa_circ_	-0.09565	0.461032
hsa_circ_	-0.67156	0.461041
hsa_circ_	-0.65179	0.461063
hsa_circ_	0.099457	0.461065
hsa_circ_	-0.20064	0.461074
hsa_circ_	0.12765	0.461099

hsa_circ_	0.307183	0.461103
hsa_circ_	0.425715	0.461107
hsa_circ_	-0.14149	0.461146
hsa_circ_	0.158066	0.461151
hsa_circ_	-0.11743	0.461156
hsa_circ_	0.164927	0.461181
hsa_circ_	-0.25821	0.461202
hsa_circ_	0.120173	0.461204
hsa_circ_	-0.12482	0.461228
hsa_circ_	-0.47006	0.461241
hsa_circ_	-0.13025	0.461241
hsa_circ_	0.161478	0.461286
hsa_circ_	-0.40885	0.461305
hsa_circ_	0.117247	0.461308
hsa_circ_	-0.15884	0.46131
hsa_circ_	0.157624	0.461323
hsa_circ_	0.159856	0.461327
hsa_circ_	-0.36477	0.461363
hsa_circ_	0.206634	0.46137
hsa_circ_	0.097246	0.461378
hsa_circ_	0.132633	0.4614
hsa_circ_	0.094432	0.461434
hsa_circ_	-0.15178	0.461447
hsa_circ_	0.128412	0.461457
hsa_circ_	-0.12327	0.461466
hsa_circ_	-0.1279	0.461467
hsa_circ_	-0.13968	0.461494
hsa_circ_	-0.27364	0.461537
hsa_circ_	-1.50053	0.46154
hsa_circ_	0.116747	0.461571
hsa_circ_	0.294521	0.461578
hsa_circ_	-0.20253	0.461588
hsa_circ_	-0.12308	0.461616
hsa_circ_	0.182217	0.461619
hsa_circ_	0.103325	0.461624
hsa_circ_	-0.16241	0.461647
hsa_circ_	0.176459	0.461652
hsa_circ_	0.155074	0.461674
hsa_circ_	-0.14797	0.461675
hsa_circ_	0.369905	0.461719
hsa_circ_	0.165741	0.461723
hsa_circ_	0.68185	0.461725
hsa_circ_	0.261076	0.461726
hsa_circ_	-0.1312	0.461727
hsa_circ_	0.106191	0.461738
hsa_circ_	0.408137	0.461739
hsa_circ_	0.168881	0.461759
hsa_circ_	0.147314	0.461796
hsa_circ_	-0.18323	0.4618
hsa_circ_	0.389195	0.461806
hsa_circ_	-0.23599	0.461836
hsa_circ_	0.340963	0.461847
hsa_circ_	0.098129	0.461848
hsa_circ_	-0.176	0.461854

hsa_circ_	-0.20395	0.461868
hsa_circ_	-0.1734	0.461881
hsa_circ_	-0.1034	0.461894
hsa_circ_	-0.10325	0.461903
hsa_circ_	0.140148	0.461903
hsa_circ_	0.1731	0.461928
hsa_circ_	0.316937	0.461933
hsa_circ_	0.243996	0.461958
hsa_circ_	-0.16954	0.461964
hsa_circ_	0.174801	0.46197
hsa_circ_	-0.22807	0.461991
hsa_circ_	-0.37427	0.461993
hsa_circ_	-0.09996	0.462
hsa_circ_	-0.30052	0.462006
hsa_circ_	0.177385	0.462009
hsa_circ_	0.180155	0.462014
hsa_circ_	-0.12897	0.462043
hsa_circ_	-0.21603	0.46205
hsa_circ_	0.285505	0.462053
hsa_circ_	0.182629	0.462062
hsa_circ_	0.14259	0.462096
hsa_circ_	0.168733	0.46214
hsa_circ_	0.60793	0.462151
hsa_circ_	0.479272	0.462169
hsa_circ_	0.119513	0.462204
hsa_circ_	-0.17436	0.462217
hsa_circ_	-0.15405	0.462226
hsa_circ_	-0.16508	0.462234
hsa_circ_	0.251638	0.462252
hsa_circ_	0.120568	0.462261
hsa_circ_	0.143747	0.46229
hsa_circ_	0.127806	0.462353
hsa_circ_	0.113711	0.462354
hsa_circ_	0.22443	0.462358
hsa_circ_	0.15107	0.462395
hsa_circ_	-0.18883	0.462397
hsa_circ_	0.237135	0.462411
hsa_circ_	-0.11706	0.462417
hsa_circ_	-0.13693	0.462454
hsa_circ_	0.123197	0.462457
hsa_circ_	-0.11692	0.462463
hsa_circ_	0.152434	0.462479
hsa_circ_	0.117285	0.462483
hsa_circ_	-0.15976	0.462514
hsa_circ_	-0.33263	0.462537
hsa_circ_	0.12857	0.462546
hsa_circ_	-0.15127	0.462565
hsa_circ_	-0.3693	0.462574
hsa_circ_	0.150181	0.462576
hsa_circ_	-0.10794	0.462576
hsa_circ_	0.140294	0.462606
hsa_circ_	-0.32265	0.462613
hsa_circ_	-0.11746	0.462621
hsa_circ_	0.183071	0.462661

hsa_circ_	-0.98419	0.462673
hsa_circ_	-0.1076	0.462689
hsa_circ_	-0.18357	0.462692
hsa_circ_	0.112835	0.462715
hsa_circ_	-0.10115	0.462719
hsa_circ_	0.102047	0.46272
hsa_circ_	0.300881	0.462724
hsa_circ_	-0.15642	0.46273
hsa_circ_	0.16004	0.462732
hsa_circ_	-0.32521	0.462752
hsa_circ_	0.209993	0.462783
hsa_circ_	-0.48404	0.462785
hsa_circ_	0.195904	0.462794
hsa_circ_	0.109329	0.462796
hsa_circ_	-0.54817	0.462818
hsa_circ_	0.105989	0.46283
hsa_circ_	0.284145	0.462833
hsa_circ_	-0.25915	0.462846
hsa_circ_	0.152627	0.462853
hsa_circ_	0.157373	0.462886
hsa_circ_	0.104458	0.462887
hsa_circ_	0.129636	0.462903
hsa_circ_	0.420296	0.462916
hsa_circ_	0.114495	0.462918
hsa_circ_	-0.18684	0.462962
hsa_circ_	0.215915	0.46298
hsa_circ_	-0.11245	0.462984
hsa_circ_	-0.1089	0.462993
hsa_circ_	-0.13561	0.463019
hsa_circ_	0.169047	0.463022
hsa_circ_	-0.15066	0.463028
hsa_circ_	0.125053	0.463041
hsa_circ_	0.219319	0.463043
hsa_circ_	0.104365	0.463051
hsa_circ_	-0.18944	0.46308
hsa_circ_	0.257508	0.46312
hsa_circ_	-0.12158	0.463133
hsa_circ_	0.201737	0.463174
hsa_circ_	0.115729	0.463208
hsa_circ_	0.185303	0.463214
hsa_circ_	-0.10643	0.463217
hsa_circ_	0.139346	0.463221
hsa_circ_	-0.10798	0.463236
hsa_circ_	0.247561	0.463245
hsa_circ_	-0.10741	0.463259
hsa_circ_	0.12859	0.463288
hsa_circ_	0.393195	0.4633
hsa_circ_	-0.19372	0.46331
hsa_circ_	0.145749	0.463316
hsa_circ_	-0.1182	0.463323
hsa_circ_	-0.2299	0.463344
hsa_circ_	0.422184	0.46342
hsa_circ_	0.34771	0.463442
hsa_circ_	-0.24649	0.463447

hsa_circ_	0.226785	0.463447
hsa_circ_	0.202491	0.463464
hsa_circ_	-0.14952	0.463466
hsa_circ_	-0.12488	0.463467
hsa_circ_	-0.14297	0.46347
hsa_circ_	0.142635	0.463484
hsa_circ_	-0.19495	0.463491
hsa_circ_	-0.37863	0.4635
hsa_circ_	0.29043	0.463508
hsa_circ_	0.153557	0.463533
hsa_circ_	-0.22271	0.463599
hsa_circ_	0.186943	0.463603
hsa_circ_	0.125542	0.463612
hsa_circ_	-0.16778	0.463621
hsa_circ_	-0.10899	0.463638
hsa_circ_	0.173272	0.463642
hsa_circ_	-0.23053	0.463648
hsa_circ_	-0.11419	0.463667
hsa_circ_	0.160545	0.463671
hsa_circ_	0.154187	0.463686
hsa_circ_	0.122957	0.463697
hsa_circ_	-0.12809	0.46372
hsa_circ_	-0.11389	0.463746
hsa_circ_	-0.13875	0.463748
hsa_circ_	-0.27249	0.46375
hsa_circ_	-0.12943	0.463806
hsa_circ_	-0.11423	0.463819
hsa_circ_	-0.29099	0.463835
hsa_circ_	0.24968	0.463844
hsa_circ_	0.124914	0.463848
hsa_circ_	0.513888	0.463852
hsa_circ_	-0.41672	0.463861
hsa_circ_	-0.15313	0.46387
hsa_circ_	0.111821	0.463872
hsa_circ_	0.236712	0.463875
hsa_circ_	-0.12351	0.463878
hsa_circ_	0.262407	0.4639
hsa_circ_	-0.19606	0.463901
hsa_circ_	-0.13549	0.46392
hsa_circ_	0.160347	0.463928
hsa_circ_	0.117342	0.463936
hsa_circ_	0.150534	0.463948
hsa_circ_	0.10334	0.463973
hsa_circ_	-0.11106	0.463983
hsa_circ_	-0.41846	0.463994
hsa_circ_	-0.18503	0.464009
hsa_circ_	-0.17017	0.464047
hsa_circ_	-0.28417	0.464069
hsa_circ_	-0.25807	0.464086
hsa_circ_	0.158493	0.464087
hsa_circ_	-0.15097	0.464105
hsa_circ_	0.226879	0.464137
hsa_circ_	0.165765	0.464138
hsa_circ_	-0.11797	0.46415

hsa_circ_	0.407694	0.464175
hsa_circ_	-0.09473	0.464204
hsa_circ_	0.117735	0.46421
hsa_circ_	-0.14143	0.464244
hsa_circ_	0.123638	0.464257
hsa_circ_	0.645016	0.464263
hsa_circ_	0.165586	0.464265
hsa_circ_	-0.09273	0.464293
hsa_circ_	0.198427	0.464293
hsa_circ_	0.199449	0.464316
hsa_circ_	-0.18676	0.464317
hsa_circ_	0.138299	0.464318
hsa_circ_	0.547565	0.464324
hsa_circ_	-0.11395	0.464334
hsa_circ_	-0.19669	0.464335
hsa_circ_	-0.54673	0.464345
hsa_circ_	-0.29884	0.464348
hsa_circ_	0.358938	0.464349
hsa_circ_	0.21452	0.464373
hsa_circ_	-0.15977	0.464381
hsa_circ_	0.207248	0.464398
hsa_circ_	0.190102	0.464416
hsa_circ_	-0.3187	0.464423
hsa_circ_	0.141187	0.464423
hsa_circ_	0.21714	0.464429
hsa_circ_	0.108484	0.464462
hsa_circ_	-0.10558	0.464483
hsa_circ_	0.125234	0.464494
hsa_circ_	0.763093	0.464504
hsa_circ_	0.142215	0.464514
hsa_circ_	0.559947	0.464542
hsa_circ_	-0.22392	0.464552
hsa_circ_	-0.41219	0.464555
hsa_circ_	-0.11912	0.464628
hsa_circ_	-0.16323	0.464638
hsa_circ_	-0.23413	0.464647
hsa_circ_	0.546588	0.464673
hsa_circ_	-0.17852	0.464678
hsa_circ_	0.135413	0.464685
hsa_circ_	0.135457	0.464697
hsa_circ_	0.168663	0.464711
hsa_circ_	-0.12725	0.464726
hsa_circ_	-0.16111	0.464752
hsa_circ_	-0.41181	0.464769
hsa_circ_	-0.11617	0.46481
hsa_circ_	0.358149	0.464814
hsa_circ_	0.10887	0.464822
hsa_circ_	0.153224	0.464859
hsa_circ_	0.227523	0.464872
hsa_circ_	0.09766	0.464875
hsa_circ_	-0.43125	0.464913
hsa_circ_	0.358288	0.464927
hsa_circ_	-0.17374	0.464933
hsa_circ_	-0.15447	0.46496

hsa_circ_	-0.11603	0.464982
hsa_circ_	-0.10817	0.464984
hsa_circ_	0.124384	0.464994
hsa_circ_	0.109585	0.465056
hsa_circ_	-0.11257	0.465064
hsa_circ_	0.112797	0.465076
hsa_circ_	-0.15425	0.465086
hsa_circ_	-0.12408	0.465096
hsa_circ_	-0.15407	0.465103
hsa_circ_	-0.14112	0.465114
hsa_circ_	0.138845	0.465119
hsa_circ_	0.199884	0.46513
hsa_circ_	-0.17498	0.465142
hsa_circ_	0.384311	0.465155
hsa_circ_	-0.0982	0.465158
hsa_circ_	-0.15322	0.465166
hsa_circ_	-0.17199	0.465179
hsa_circ_	0.137247	0.46519
hsa_circ_	-0.20976	0.465196
hsa_circ_	0.112291	0.465204
hsa_circ_	0.670515	0.465221
hsa_circ_	0.203579	0.465227
hsa_circ_	-0.69812	0.465262
hsa_circ_	-0.27065	0.465264
hsa_circ_	-0.62625	0.465265
hsa_circ_	0.128577	0.465273
hsa_circ_	0.294907	0.46529
hsa_circ_	-0.20969	0.465327
hsa_circ_	-0.12151	0.465332
hsa_circ_	0.110383	0.465333
hsa_circ_	-0.20043	0.465355
hsa_circ_	0.539804	0.465355
hsa_circ_	0.109918	0.465365
hsa_circ_	0.175113	0.465368
hsa_circ_	0.097157	0.46539
hsa_circ_	0.225041	0.465407
hsa_circ_	0.259142	0.465413
hsa_circ_	-0.1356	0.465432
hsa_circ_	-0.095	0.465437
hsa_circ_	-0.10609	0.465448
hsa_circ_	0.119515	0.465456
hsa_circ_	0.104776	0.465469
hsa_circ_	-0.12385	0.465497
hsa_circ_	-0.19008	0.46553
hsa_circ_	0.115149	0.465545
hsa_circ_	-0.48599	0.465581
hsa_circ_	0.207307	0.465596
hsa_circ_	0.125887	0.465596
hsa_circ_	-0.10441	0.465606
hsa_circ_	0.234148	0.465624
hsa_circ_	0.466004	0.465633
hsa_circ_	0.115814	0.465642
hsa_circ_	0.360255	0.465642
hsa_circ_	0.446272	0.465648

hsa_circ_	-0.09386	0.465655
hsa_circ_	-0.56661	0.465666
hsa_circ_	0.134196	0.465676
hsa_circ_	-0.29439	0.465704
hsa_circ_	0.128552	0.465712
hsa_circ_	-0.1279	0.46572
hsa_circ_	0.304448	0.465735
hsa_circ_	0.381467	0.465737
hsa_circ_	-0.19279	0.465745
hsa_circ_	-0.11566	0.465745
hsa_circ_	-0.12547	0.465762
hsa_circ_	-0.18534	0.465773
hsa_circ_	-0.12678	0.465808
hsa_circ_	0.111252	0.465817
hsa_circ_	0.263836	0.465821
hsa_circ_	0.244565	0.46583
hsa_circ_	-0.10835	0.465832
hsa_circ_	-0.22535	0.465834
hsa_circ_	0.169149	0.465854
hsa_circ_	-0.33757	0.465859
hsa_circ_	0.098909	0.46587
hsa_circ_	0.169117	0.465883
hsa_circ_	0.14994	0.465885
hsa_circ_	0.329051	0.465902
hsa_circ_	0.170845	0.465918
hsa_circ_	0.452267	0.465936
hsa_circ_	-0.32896	0.465948
hsa_circ_	-0.22598	0.465957
hsa_circ_	-0.15058	0.465963
hsa_circ_	0.151674	0.465971
hsa_circ_	0.124689	0.465972
hsa_circ_	-0.09745	0.465981
hsa_circ_	0.190504	0.465994
hsa_circ_	-0.10857	0.465998
hsa_circ_	0.312934	0.466007
hsa_circ_	-0.19202	0.466018
hsa_circ_	0.117878	0.46604
hsa_circ_	-0.131	0.466042
hsa_circ_	-0.10523	0.466087
hsa_circ_	0.125468	0.466099
hsa_circ_	-0.20057	0.466104
hsa_circ_	-0.45163	0.466122
hsa_circ_	-0.17754	0.466125
hsa_circ_	-0.11134	0.466126
hsa_circ_	-0.2344	0.46613
hsa_circ_	-0.19069	0.466147
hsa_circ_	-0.15996	0.466156
hsa_circ_	-0.18916	0.466167
hsa_circ_	-0.11884	0.466172
hsa_circ_	-0.36183	0.466184
hsa_circ_	0.138238	0.466199
hsa_circ_	0.1043	0.466206
hsa_circ_	-0.16297	0.466237
hsa_circ_	0.12926	0.466242

hsa_circ_	0.13689	0.466244
hsa_circ_	0.272871	0.46627
hsa_circ_	-0.22963	0.466284
hsa_circ_	-0.14369	0.466291
hsa_circ_	-0.20263	0.466296
hsa_circ_	-0.17267	0.466309
hsa_circ_	0.163966	0.466314
hsa_circ_	0.181766	0.466392
hsa_circ_	0.129659	0.4664
hsa_circ_	-0.19823	0.466404
hsa_circ_	-0.22617	0.46641
hsa_circ_	0.175577	0.466436
hsa_circ_	0.105879	0.466499
hsa_circ_	-0.49397	0.466503
hsa_circ_	0.168219	0.466504
hsa_circ_	0.320168	0.466506
hsa_circ_	-0.13049	0.466572
hsa_circ_	-0.09996	0.466572
hsa_circ_	0.198249	0.466578
hsa_circ_	0.139249	0.466579
hsa_circ_	0.323518	0.466592
hsa_circ_	0.100631	0.466603
hsa_circ_	-0.43086	0.466608
hsa_circ_	0.127677	0.466609
hsa_circ_	0.109323	0.466615
hsa_circ_	-0.25578	0.46666
hsa_circ_	0.411311	0.466675
hsa_circ_	0.108239	0.466692
hsa_circ_	-0.12441	0.466734
hsa_circ_	0.148061	0.466735
hsa_circ_	0.138354	0.466757
hsa_circ_	0.209385	0.466781
hsa_circ_	0.117757	0.466787
hsa_circ_	0.157854	0.466789
hsa_circ_	0.344266	0.466805
hsa_circ_	0.154205	0.466812
hsa_circ_	0.162867	0.466845
hsa_circ_	-0.22346	0.466869
hsa_circ_	0.135321	0.466874
hsa_circ_	0.119304	0.466895
hsa_circ_	0.10593	0.466916
hsa_circ_	-0.30849	0.466928
hsa_circ_	-0.10712	0.466931
hsa_circ_	-0.22182	0.466932
hsa_circ_	-0.1197	0.46694
hsa_circ_	-0.10156	0.466944
hsa_circ_	0.11882	0.466958
hsa_circ_	0.134715	0.466965
hsa_circ_	-0.32007	0.467002
hsa_circ_	0.109224	0.467004
hsa_circ_	0.320217	0.467044
hsa_circ_	0.121443	0.467054
hsa_circ_	-0.14341	0.467055
hsa_circ_	-0.28636	0.467065

hsa_circ_	-0.09621	0.467083
hsa_circ_	0.435303	0.467091
hsa_circ_	-0.22671	0.467092
hsa_circ_	0.2321	0.467137
hsa_circ_	0.147125	0.467176
hsa_circ_	0.217674	0.467181
hsa_circ_	-0.09858	0.467193
hsa_circ_	0.200857	0.467209
hsa_circ_	-0.10538	0.467257
hsa_circ_	-0.75316	0.467262
hsa_circ_	0.10384	0.467276
hsa_circ_	0.131031	0.467295
hsa_circ_	-0.1474	0.467295
hsa_circ_	0.124158	0.467331
hsa_circ_	-0.28089	0.467332
hsa_circ_	-0.13805	0.467361
hsa_circ_	-0.13419	0.467374
hsa_circ_	-0.19416	0.467379
hsa_circ_	0.189677	0.46741
hsa_circ_	0.156503	0.467425
hsa_circ_	-0.16302	0.467515
hsa_circ_	0.101482	0.46752
hsa_circ_	0.110589	0.467523
hsa_circ_	-0.26104	0.467526
hsa_circ_	0.510344	0.467544
hsa_circ_	0.199165	0.467569
hsa_circ_	-0.12057	0.467582
hsa_circ_	0.148866	0.467584
hsa_circ_	-0.10085	0.467596
hsa_circ_	0.47273	0.467608
hsa_circ_	0.444506	0.467636
hsa_circ_	0.208894	0.467654
hsa_circ_	-0.15068	0.46767
hsa_circ_	0.151583	0.467679
hsa_circ_	-0.45107	0.467682
hsa_circ_	-0.18493	0.46769
hsa_circ_	0.446462	0.467694
hsa_circ_	-0.11431	0.467701
hsa_circ_	0.139249	0.467704
hsa_circ_	-0.14517	0.467723
hsa_circ_	-0.18795	0.467743
hsa_circ_	0.116071	0.46775
hsa_circ_	0.207283	0.467756
hsa_circ_	-0.10566	0.467759
hsa_circ_	0.442563	0.467805
hsa_circ_	-0.12059	0.467822
hsa_circ_	0.135619	0.467825
hsa_circ_	-0.42182	0.467827
hsa_circ_	0.242873	0.46784
hsa_circ_	-0.11302	0.467841
hsa_circ_	-0.17378	0.467842
hsa_circ_	0.147727	0.46786
hsa_circ_	0.109726	0.467871
hsa_circ_	-0.24595	0.467871

hsa_circ_	-0.18624	0.467888
hsa_circ_	0.106955	0.467934
hsa_circ_	0.170339	0.467949
hsa_circ_	-0.11204	0.467951
hsa_circ_	0.206515	0.467976
hsa_circ_	0.197276	0.467986
hsa_circ_	0.125137	0.467988
hsa_circ_	-0.12833	0.467994
hsa_circ_	-0.10645	0.468001
hsa_circ_	0.140056	0.468028
hsa_circ_	-0.19923	0.468091
hsa_circ_	-0.15466	0.468103
hsa_circ_	0.135242	0.468112
hsa_circ_	0.129814	0.468114
hsa_circ_	-0.33595	0.468117
hsa_circ_	0.224426	0.468144
hsa_circ_	-0.29114	0.468149
hsa_circ_	0.222675	0.468154
hsa_circ_	-0.11736	0.468161
hsa_circ_	-0.61096	0.468193
hsa_circ_	0.527543	0.468195
hsa_circ_	0.146813	0.468213
hsa_circ_	0.097423	0.468218
hsa_circ_	0.142252	0.468232
hsa_circ_	0.195911	0.468247
hsa_circ_	0.162961	0.46825
hsa_circ_	0.108768	0.468263
hsa_circ_	0.158048	0.468263
hsa_circ_	-0.57223	0.468315
hsa_circ_	-0.28507	0.468327
hsa_circ_	-0.41075	0.468342
hsa_circ_	0.109299	0.468346
hsa_circ_	0.63119	0.468346
hsa_circ_	-0.17205	0.468355
hsa_circ_	0.178979	0.468358
hsa_circ_	0.217035	0.468373
hsa_circ_	-0.21478	0.468379
hsa_circ_	-0.11924	0.468383
hsa_circ_	-0.24482	0.468384
hsa_circ_	0.108763	0.468395
hsa_circ_	-0.16368	0.46845
hsa_circ_	-0.17285	0.468451
hsa_circ_	-0.13483	0.468453
hsa_circ_	-0.37634	0.468456
hsa_circ_	0.462252	0.468458
hsa_circ_	-0.20749	0.468465
hsa_circ_	-0.10761	0.468472
hsa_circ_	-0.11091	0.468476
hsa_circ_	0.094138	0.468487
hsa_circ_	-0.10382	0.468532
hsa_circ_	-0.16803	0.468536
hsa_circ_	-0.1909	0.468538
hsa_circ_	-0.16016	0.468543
hsa_circ_	0.125822	0.46857

hsa_circ_	-0.19372	0.468578
hsa_circ_	0.214584	0.468596
hsa_circ_	-0.34709	0.468604
hsa_circ_	-0.41012	0.468643
hsa_circ_	-0.10888	0.468652
hsa_circ_	-0.28354	0.468655
hsa_circ_	-0.15885	0.46866
hsa_circ_	-0.29141	0.468671
hsa_circ_	-0.25772	0.468691
hsa_circ_	-0.10076	0.468694
hsa_circ_	-0.28086	0.468738
hsa_circ_	0.338933	0.468749
hsa_circ_	-0.17471	0.468754
hsa_circ_	0.276988	0.468758
hsa_circ_	-0.16967	0.468771
hsa_circ_	-0.24535	0.468784
hsa_circ_	0.4485	0.468788
hsa_circ_	0.127812	0.468797
hsa_circ_	0.129783	0.468798
hsa_circ_	-0.42755	0.468805
hsa_circ_	0.147246	0.468813
hsa_circ_	-0.183	0.468816
hsa_circ_	-0.15364	0.468828
hsa_circ_	-0.10076	0.468854
hsa_circ_	0.260201	0.468857
hsa_circ_	0.244915	0.468878
hsa_circ_	0.519534	0.468898
hsa_circ_	0.13347	0.468916
hsa_circ_	-0.16778	0.468978
hsa_circ_	-0.35481	0.468979
hsa_circ_	-0.10537	0.468982
hsa_circ_	-0.13819	0.468989
hsa_circ_	-0.12863	0.468991
hsa_circ_	-0.20301	0.468995
hsa_circ_	0.530719	0.469007
hsa_circ_	-0.09621	0.469017
hsa_circ_	-0.20129	0.46902
hsa_circ_	-0.12033	0.469044
hsa_circ_	0.179945	0.469087
hsa_circ_	-0.35855	0.46912
hsa_circ_	-0.15424	0.469133
hsa_circ_	-0.13112	0.469137
hsa_circ_	0.299	0.469141
hsa_circ_	0.181991	0.469142
hsa_circ_	-0.24283	0.469143
hsa_circ_	0.151906	0.469156
hsa_circ_	0.144723	0.469161
hsa_circ_	0.166897	0.469164
hsa_circ_	0.130441	0.469172
hsa_circ_	-0.12222	0.469174
hsa_circ_	0.100525	0.469188
hsa_circ_	0.274029	0.469202
hsa_circ_	-0.16969	0.469213
hsa_circ_	0.13382	0.469216

hsa_circ_	0.110324	0.469222
hsa_circ_	-0.55789	0.469252
hsa_circ_	-0.12832	0.469261
hsa_circ_	0.138084	0.469268
hsa_circ_	-0.46228	0.469291
hsa_circ_	-0.1113	0.469315
hsa_circ_	-0.18162	0.469323
hsa_circ_	0.332101	0.469324
hsa_circ_	-0.16725	0.46935
hsa_circ_	-0.09968	0.469361
hsa_circ_	0.168511	0.469361
hsa_circ_	-0.1	0.469369
hsa_circ_	-0.09591	0.469383
hsa_circ_	0.116224	0.469414
hsa_circ_	-0.09891	0.469414
hsa_circ_	0.135396	0.469415
hsa_circ_	0.133527	0.469429
hsa_circ_	0.121715	0.469431
hsa_circ_	-0.1392	0.469443
hsa_circ_	0.260352	0.46945
hsa_circ_	-0.23296	0.469484
hsa_circ_	-0.18248	0.469494
hsa_circ_	0.504196	0.469514
hsa_circ_	-0.24828	0.469541
hsa_circ_	-0.32939	0.469598
hsa_circ_	0.222152	0.46961
hsa_circ_	-0.11593	0.469617
hsa_circ_	0.144855	0.469631
hsa_circ_	-0.11447	0.46965
hsa_circ_	0.179776	0.469657
hsa_circ_	-0.46762	0.469696
hsa_circ_	-0.24612	0.469706
hsa_circ_	0.139217	0.469716
hsa_circ_	0.17133	0.469718
hsa_circ_	-0.17042	0.469726
hsa_circ_	0.148754	0.469778
hsa_circ_	-0.1004	0.469792
hsa_circ_	0.285716	0.469818
hsa_circ_	-0.23175	0.469819
hsa_circ_	-0.23182	0.469869
hsa_circ_	0.204545	0.469885
hsa_circ_	0.159224	0.469895
hsa_circ_	0.181953	0.469901
hsa_circ_	-0.25549	0.469929
hsa_circ_	0.150233	0.469959
hsa_circ_	0.155027	0.469996
hsa_circ_	-0.12493	0.47005
hsa_circ_	-0.50116	0.47006
hsa_circ_	0.203392	0.470064
hsa_circ_	-0.16484	0.470099
hsa_circ_	0.138448	0.470112
hsa_circ_	-0.16076	0.47012
hsa_circ_	-0.09667	0.470134
hsa_circ_	-0.14266	0.470143

hsa_circ_	0.121945	0.470149
hsa_circ_	-0.34975	0.470168
hsa_circ_	0.203825	0.470179
hsa_circ_	0.124307	0.470198
hsa_circ_	-0.12411	0.470213
hsa_circ_	-0.13529	0.47024
hsa_circ_	-0.22731	0.470279
hsa_circ_	-0.17515	0.470281
hsa_circ_	-0.2951	0.470287
hsa_circ_	0.182645	0.470287
hsa_circ_	-0.09736	0.4703
hsa_circ_	-0.38428	0.470356
hsa_circ_	0.163427	0.470359
hsa_circ_	0.303882	0.470362
hsa_circ_	0.527597	0.470371
hsa_circ_	-0.32199	0.47038
hsa_circ_	0.163256	0.470392
hsa_circ_	0.141348	0.470392
hsa_circ_	-0.2325	0.470403
hsa_circ_	-0.15561	0.470418
hsa_circ_	0.174144	0.470421
hsa_circ_	-0.18284	0.470422
hsa_circ_	0.170428	0.470435
hsa_circ_	0.327133	0.47045
hsa_circ_	-0.22875	0.470455
hsa_circ_	0.104826	0.470471
hsa_circ_	0.46706	0.470478
hsa_circ_	0.113744	0.47048
hsa_circ_	0.313747	0.4705
hsa_circ_	0.229643	0.470503
hsa_circ_	-0.11554	0.470517
hsa_circ_	-0.12974	0.470518
hsa_circ_	-0.3436	0.47055
hsa_circ_	0.327966	0.470554
hsa_circ_	-0.13201	0.470565
hsa_circ_	-0.24555	0.470582
hsa_circ_	0.126838	0.470583
hsa_circ_	-0.10713	0.470589
hsa_circ_	-0.37422	0.470605
hsa_circ_	-0.70642	0.470613
hsa_circ_	-0.17481	0.470614
hsa_circ_	0.147818	0.470646
hsa_circ_	0.142734	0.470646
hsa_circ_	-0.1717	0.470661
hsa_circ_	-0.30818	0.470703
hsa_circ_	-0.12636	0.470716
hsa_circ_	-0.09942	0.470717
hsa_circ_	0.098714	0.470724
hsa_circ_	0.236975	0.470736
hsa_circ_	-0.09326	0.470744
hsa_circ_	0.116703	0.470776
hsa_circ_	-0.13852	0.470777
hsa_circ_	0.190294	0.47078
hsa_circ_	-0.09413	0.470789

hsa_circ_	0.214899	0.470803
hsa_circ_	0.197574	0.470822
hsa_circ_	0.144929	0.470827
hsa_circ_	0.217215	0.470845
hsa_circ_	-0.27368	0.470861
hsa_circ_	0.138677	0.470867
hsa_circ_	-0.12073	0.47087
hsa_circ_	0.117412	0.470906
hsa_circ_	0.500889	0.470913
hsa_circ_	-0.1893	0.470917
hsa_circ_	0.108259	0.470922
hsa_circ_	0.126058	0.470922
hsa_circ_	0.221107	0.470923
hsa_circ_	-0.21357	0.470929
hsa_circ_	-0.10232	0.470934
hsa_circ_	0.143103	0.471001
hsa_circ_	0.13208	0.471008
hsa_circ_	0.253485	0.471014
hsa_circ_	-0.48057	0.471027
hsa_circ_	0.122695	0.471052
hsa_circ_	0.264588	0.471065
hsa_circ_	-0.18392	0.471078
hsa_circ_	0.246654	0.471084
hsa_circ_	0.175614	0.471084
hsa_circ_	0.13041	0.471101
hsa_circ_	-0.16441	0.471105
hsa_circ_	-0.10684	0.471123
hsa_circ_	-0.17955	0.471124
hsa_circ_	-0.13135	0.471125
hsa_circ_	0.180009	0.471154
hsa_circ_	-0.38461	0.471161
hsa_circ_	0.12934	0.471167
hsa_circ_	-0.20245	0.471197
hsa_circ_	0.303763	0.471201
hsa_circ_	-0.1321	0.471206
hsa_circ_	-0.40639	0.471217
hsa_circ_	-0.15892	0.471234
hsa_circ_	-0.21474	0.47124
hsa_circ_	0.12829	0.471248
hsa_circ_	-0.10058	0.471251
hsa_circ_	0.158506	0.471274
hsa_circ_	-0.13885	0.471276
hsa_circ_	0.107547	0.471283
hsa_circ_	-0.10564	0.471286
hsa_circ_	-0.27882	0.471324
hsa_circ_	-0.23414	0.471341
hsa_circ_	-0.17971	0.471359
hsa_circ_	0.116127	0.471405
hsa_circ_	0.62929	0.471407
hsa_circ_	0.182762	0.471447
hsa_circ_	0.163274	0.471468
hsa_circ_	0.312846	0.471479
hsa_circ_	-0.21101	0.471493
hsa_circ_	0.10885	0.471494

hsa_circ_	-0.10348	0.471496
hsa_circ_	-0.14517	0.471498
hsa_circ_	0.16795	0.471513
hsa_circ_	0.204301	0.47152
hsa_circ_	0.317499	0.471532
hsa_circ_	-0.12297	0.471534
hsa_circ_	-0.09927	0.471538
hsa_circ_	-0.09246	0.47154
hsa_circ_	0.343652	0.471544
hsa_circ_	0.111607	0.471558
hsa_circ_	-0.14119	0.471558
hsa_circ_	0.205921	0.471566
hsa_circ_	-0.1602	0.471608
hsa_circ_	-0.46913	0.471613
hsa_circ_	0.184294	0.47163
hsa_circ_	-0.13707	0.471642
hsa_circ_	-0.12326	0.471652
hsa_circ_	0.154043	0.471663
hsa_circ_	0.225243	0.471708
hsa_circ_	0.16805	0.471711
hsa_circ_	-0.10263	0.471722
hsa_circ_	0.117133	0.471738
hsa_circ_	-0.29448	0.47175
hsa_circ_	-0.19624	0.47178
hsa_circ_	-0.33537	0.471786
hsa_circ_	-0.20895	0.471789
hsa_circ_	-0.18123	0.471797
hsa_circ_	-0.13376	0.471858
hsa_circ_	-0.10102	0.471862
hsa_circ_	0.213439	0.471867
hsa_circ_	0.101115	0.471868
hsa_circ_	0.131223	0.471871
hsa_circ_	-0.15577	0.471885
hsa_circ_	0.150588	0.471887
hsa_circ_	-0.36096	0.471898
hsa_circ_	-0.33741	0.471907
hsa_circ_	0.113242	0.47191
hsa_circ_	0.155125	0.471926
hsa_circ_	0.171513	0.471927
hsa_circ_	-0.18723	0.471934
hsa_circ_	-0.13942	0.471938
hsa_circ_	0.181401	0.471939
hsa_circ_	0.157016	0.47194
hsa_circ_	-0.11471	0.471964
hsa_circ_	-0.31924	0.471969
hsa_circ_	0.130348	0.471977
hsa_circ_	0.412315	0.471977
hsa_circ_	-0.22718	0.471982
hsa_circ_	-0.14073	0.471983
hsa_circ_	-0.1106	0.471992
hsa_circ_	-0.60256	0.472
hsa_circ_	0.111221	0.472016
hsa_circ_	-0.15099	0.472043
hsa_circ_	0.1061	0.472052

hsa_circ_	0.097455	0.472058
hsa_circ_	0.094728	0.472067
hsa_circ_	-0.16343	0.472091
hsa_circ_	-0.12468	0.472096
hsa_circ_	0.130456	0.472116
hsa_circ_	-0.28612	0.472132
hsa_circ_	-0.43886	0.472139
hsa_circ_	-0.14931	0.472165
hsa_circ_	0.175915	0.472218
hsa_circ_	-0.10824	0.472228
hsa_circ_	-0.12934	0.472243
hsa_circ_	-0.23504	0.472248
hsa_circ_	0.098843	0.472295
hsa_circ_	0.135513	0.472308
hsa_circ_	0.106472	0.472335
hsa_circ_	-0.40149	0.472335
hsa_circ_	-0.25864	0.472369
hsa_circ_	0.131726	0.472374
hsa_circ_	0.167704	0.472375
hsa_circ_	-0.12475	0.472377
hsa_circ_	0.123541	0.472433
hsa_circ_	-0.14625	0.472484
hsa_circ_	0.256782	0.472488
hsa_circ_	0.281426	0.472526
hsa_circ_	-0.1004	0.47253
hsa_circ_	0.14879	0.472544
hsa_circ_	-0.10903	0.472555
hsa_circ_	-0.15244	0.472561
hsa_circ_	-0.32972	0.472585
hsa_circ_	-0.27316	0.472601
hsa_circ_	0.305591	0.47263
hsa_circ_	-0.13812	0.47263
hsa_circ_	-0.19604	0.472706
hsa_circ_	0.169972	0.472765
hsa_circ_	0.162313	0.472766
hsa_circ_	-0.21918	0.472775
hsa_circ_	0.139194	0.472793
hsa_circ_	-0.39112	0.472864
hsa_circ_	-0.13782	0.472874
hsa_circ_	-0.14037	0.472882
hsa_circ_	-0.44227	0.472893
hsa_circ_	-0.14679	0.472895
hsa_circ_	-0.15382	0.472903
hsa_circ_	0.252339	0.472915
hsa_circ_	0.213362	0.472922
hsa_circ_	0.172283	0.472926
hsa_circ_	0.181919	0.472936
hsa_circ_	-0.37331	0.472936
hsa_circ_	-0.31194	0.472994
hsa_circ_	-0.16223	0.472997
hsa_circ_	0.138336	0.473027
hsa_circ_	-0.13314	0.473037
hsa_circ_	-0.11351	0.473061
hsa_circ_	0.187558	0.473074

hsa_circ_	-0.18412	0.473075
hsa_circ_	-0.30934	0.473135
hsa_circ_	0.586655	0.473152
hsa_circ_	-0.21162	0.473157
hsa_circ_	0.129805	0.47321
hsa_circ_	-0.61367	0.473214
hsa_circ_	-0.10876	0.473219
hsa_circ_	-0.45823	0.473225
hsa_circ_	0.131291	0.473235
hsa_circ_	-0.41933	0.473251
hsa_circ_	0.114817	0.473255
hsa_circ_	0.145214	0.473293
hsa_circ_	-0.16587	0.473322
hsa_circ_	-0.21008	0.473347
hsa_circ_	0.134074	0.473388
hsa_circ_	-0.15419	0.473401
hsa_circ_	-0.18963	0.473406
hsa_circ_	0.103429	0.473418
hsa_circ_	0.13445	0.473423
hsa_circ_	0.170347	0.47348
hsa_circ_	0.193869	0.473485
hsa_circ_	-0.10588	0.473491
hsa_circ_	0.123514	0.47353
hsa_circ_	-0.16986	0.473573
hsa_circ_	-0.6273	0.473575
hsa_circ_	0.379637	0.473583
hsa_circ_	-0.10815	0.473592
hsa_circ_	-0.42269	0.473639
hsa_circ_	-0.20546	0.473654
hsa_circ_	-0.32874	0.473654
hsa_circ_	-0.24446	0.47367
hsa_circ_	-0.27456	0.473693
hsa_circ_	-0.15403	0.473695
hsa_circ_	-0.14717	0.473708
hsa_circ_	0.484306	0.473715
hsa_circ_	-0.18431	0.473721
hsa_circ_	0.259512	0.473762
hsa_circ_	0.125197	0.473779
hsa_circ_	-0.17468	0.473784
hsa_circ_	-0.16459	0.473795
hsa_circ_	0.144398	0.473807
hsa_circ_	-0.17822	0.473808
hsa_circ_	0.18791	0.473828
hsa_circ_	0.127418	0.473835
hsa_circ_	0.155565	0.473875
hsa_circ_	-0.12309	0.473884
hsa_circ_	0.117589	0.473902
hsa_circ_	-0.13115	0.47394
hsa_circ_	0.091397	0.473949
hsa_circ_	0.103769	0.473953
hsa_circ_	-0.18054	0.47396
hsa_circ_	-0.12061	0.473962
hsa_circ_	-0.10367	0.473962
hsa_circ_	0.113055	0.473974

hsa_circ_	-0.16104	0.473976
hsa_circ_	-0.2123	0.473978
hsa_circ_	-0.09923	0.47399
hsa_circ_	0.18131	0.473993
hsa_circ_	-0.54818	0.474003
hsa_circ_	0.497235	0.474046
hsa_circ_	-0.09318	0.474075
hsa_circ_	0.385525	0.474079
hsa_circ_	0.25703	0.474083
hsa_circ_	0.116578	0.474096
hsa_circ_	-0.43778	0.4741
hsa_circ_	-0.21367	0.474122
hsa_circ_	0.329728	0.474193
hsa_circ_	-0.147	0.474194
hsa_circ_	0.511163	0.474199
hsa_circ_	0.185135	0.474214
hsa_circ_	0.156524	0.474214
hsa_circ_	0.131658	0.474216
hsa_circ_	0.141271	0.47422
hsa_circ_	0.192484	0.474227
hsa_circ_	-0.3147	0.474232
hsa_circ_	-0.09318	0.474236
hsa_circ_	0.196054	0.474252
hsa_circ_	-0.12937	0.474263
hsa_circ_	-0.15833	0.474265
hsa_circ_	0.129843	0.474275
hsa_circ_	-0.10569	0.474279
hsa_circ_	0.605563	0.474288
hsa_circ_	0.120611	0.474293
hsa_circ_	0.104852	0.47432
hsa_circ_	0.605499	0.474324
hsa_circ_	-0.37898	0.474326
hsa_circ_	-0.15875	0.474339
hsa_circ_	-0.23008	0.474352
hsa_circ_	0.195808	0.47439
hsa_circ_	0.395202	0.47441
hsa_circ_	-0.11523	0.47445
hsa_circ_	0.138367	0.474451
hsa_circ_	0.162468	0.474467
hsa_circ_	-0.16723	0.474468
hsa_circ_	-0.13511	0.474486
hsa_circ_	-0.15852	0.47451
hsa_circ_	0.169086	0.47454
hsa_circ_	-0.2844	0.474562
hsa_circ_	0.231119	0.474563
hsa_circ_	0.27255	0.474634
hsa_circ_	0.152807	0.474667
hsa_circ_	0.142166	0.474678
hsa_circ_	-0.26763	0.474691
hsa_circ_	0.120546	0.474692
hsa_circ_	0.328126	0.474693
hsa_circ_	-0.13981	0.47471
hsa_circ_	0.28975	0.474713
hsa_circ_	-0.28721	0.474726

hsa_circ_	0.162882	0.474729
hsa_circ_	0.127572	0.47473
hsa_circ_	0.186394	0.47473
hsa_circ_	-0.12814	0.474743
hsa_circ_	-0.09451	0.47479
hsa_circ_	-0.09784	0.47481
hsa_circ_	0.147719	0.474819
hsa_circ_	0.118229	0.474838
hsa_circ_	-0.10928	0.474845
hsa_circ_	0.157232	0.474877
hsa_circ_	0.13799	0.474892
hsa_circ_	0.113616	0.474902
hsa_circ_	-0.25979	0.474904
hsa_circ_	0.224554	0.474908
hsa_circ_	0.394694	0.474913
hsa_circ_	0.144939	0.474925
hsa_circ_	0.110085	0.474927
hsa_circ_	0.123489	0.474939
hsa_circ_	-0.14754	0.474973
hsa_circ_	0.169843	0.474979
hsa_circ_	0.222797	0.474998
hsa_circ_	0.15212	0.475012
hsa_circ_	-0.12323	0.475014
hsa_circ_	0.127862	0.475037
hsa_circ_	0.104041	0.475045
hsa_circ_	-0.11257	0.475117
hsa_circ_	0.272982	0.475121
hsa_circ_	-0.11733	0.475123
hsa_circ_	0.258065	0.475166
hsa_circ_	-0.42678	0.47518
hsa_circ_	-0.55686	0.475181
hsa_circ_	-0.1117	0.475208
hsa_circ_	0.10878	0.475213
hsa_circ_	-0.19173	0.475231
hsa_circ_	0.222043	0.47525
hsa_circ_	-0.16483	0.475253
hsa_circ_	-0.1344	0.475281
hsa_circ_	0.181794	0.475298
hsa_circ_	-0.10061	0.475301
hsa_circ_	0.128015	0.475302
hsa_circ_	-0.216	0.475304
hsa_circ_	0.156833	0.475308
hsa_circ_	-0.31433	0.475367
hsa_circ_	-0.20239	0.475381
hsa_circ_	-0.30176	0.475383
hsa_circ_	0.151361	0.4754
hsa_circ_	0.19917	0.475405
hsa_circ_	0.16705	0.475415
hsa_circ_	0.108423	0.475426
hsa_circ_	0.207848	0.475429
hsa_circ_	-0.14578	0.475437
hsa_circ_	-1.43324	0.475443
hsa_circ_	0.164919	0.475453
hsa_circ_	-0.11012	0.475459

hsa_circ_	-0.09584	0.475465
hsa_circ_	0.346769	0.475478
hsa_circ_	0.184847	0.475484
hsa_circ_	-0.11028	0.475485
hsa_circ_	0.331338	0.475489
hsa_circ_	0.113137	0.475502
hsa_circ_	-0.15682	0.475524
hsa_circ_	-0.12321	0.475539
hsa_circ_	-0.1577	0.475557
hsa_circ_	-0.12305	0.475599
hsa_circ_	-0.26411	0.475606
hsa_circ_	-0.61813	0.475625
hsa_circ_	0.18598	0.475646
hsa_circ_	0.130506	0.475655
hsa_circ_	-0.12981	0.475657
hsa_circ_	0.156336	0.47566
hsa_circ_	-0.19788	0.475678
hsa_circ_	-0.10842	0.475696
hsa_circ_	-0.51893	0.4757
hsa_circ_	-0.15494	0.475701
hsa_circ_	-0.17883	0.475702
hsa_circ_	0.116674	0.475705
hsa_circ_	-0.18329	0.475707
hsa_circ_	-0.19292	0.475726
hsa_circ_	-0.11882	0.47574
hsa_circ_	-0.09858	0.475776
hsa_circ_	0.33144	0.475783
hsa_circ_	-0.41685	0.47581
hsa_circ_	0.134593	0.475833
hsa_circ_	-0.26406	0.475847
hsa_circ_	-0.11702	0.475851
hsa_circ_	-0.11504	0.475856
hsa_circ_	0.134692	0.475859
hsa_circ_	-0.12245	0.475861
hsa_circ_	0.385685	0.475879
hsa_circ_	-0.10141	0.475887
hsa_circ_	-0.14551	0.475925
hsa_circ_	-0.33642	0.475959
hsa_circ_	0.134735	0.475977
hsa_circ_	-0.43969	0.475983
hsa_circ_	0.127981	0.475991
hsa_circ_	-0.17509	0.475995
hsa_circ_	0.390192	0.475996
hsa_circ_	0.100164	0.475998
hsa_circ_	-0.59529	0.476001
hsa_circ_	-0.20131	0.476009
hsa_circ_	-0.17218	0.476029
hsa_circ_	-0.09629	0.476035
hsa_circ_	-0.14307	0.476044
hsa_circ_	0.141035	0.476058
hsa_circ_	-0.11257	0.476066
hsa_circ_	0.689028	0.476076
hsa_circ_	-0.51803	0.476091
hsa_circ_	-0.11394	0.476092

hsa_circ_	0.112355	0.476097
hsa_circ_	-0.26833	0.476098
hsa_circ_	-0.15488	0.476111
hsa_circ_	-0.10653	0.476119
hsa_circ_	0.137735	0.476123
hsa_circ_	0.283837	0.476137
hsa_circ_	0.374874	0.476155
hsa_circ_	-0.22392	0.476179
hsa_circ_	0.222694	0.476194
hsa_circ_	0.222481	0.476215
hsa_circ_	-0.21327	0.476241
hsa_circ_	-0.16373	0.476247
hsa_circ_	-0.13984	0.476317
hsa_circ_	-0.30304	0.47633
hsa_circ_	-0.16398	0.476348
hsa_circ_	0.552748	0.476362
hsa_circ_	0.312827	0.476376
hsa_circ_	0.23688	0.47639
hsa_circ_	0.137763	0.476402
hsa_circ_	-0.16593	0.476406
hsa_circ_	-0.15953	0.476412
hsa_circ_	0.204143	0.476423
hsa_circ_	0.119759	0.47645
hsa_circ_	0.301279	0.476452
hsa_circ_	-0.12569	0.476459
hsa_circ_	0.391429	0.476501
hsa_circ_	0.273025	0.476506
hsa_circ_	0.166448	0.47651
hsa_circ_	-0.12418	0.47651
hsa_circ_	0.144815	0.476526
hsa_circ_	-0.24395	0.476551
hsa_circ_	-0.11988	0.476551
hsa_circ_	-0.14657	0.476557
hsa_circ_	0.373738	0.476563
hsa_circ_	0.106392	0.476566
hsa_circ_	0.128852	0.476579
hsa_circ_	0.124141	0.476586
hsa_circ_	-0.21588	0.476597
hsa_circ_	-0.1154	0.476599
hsa_circ_	-0.13526	0.4766
hsa_circ_	0.203913	0.476614
hsa_circ_	-0.18311	0.476628
hsa_circ_	0.380942	0.476647
hsa_circ_	-0.10134	0.476661
hsa_circ_	0.356037	0.476666
hsa_circ_	-0.24914	0.476672
hsa_circ_	-0.2947	0.476675
hsa_circ_	0.141588	0.476677
hsa_circ_	-0.1414	0.476707
hsa_circ_	0.134097	0.476713
hsa_circ_	0.129481	0.476714
hsa_circ_	0.230464	0.476727
hsa_circ_	-0.1788	0.476743
hsa_circ_	-0.13991	0.47675

hsa_circ_	0.160198	0.476752
hsa_circ_	-0.151	0.476754
hsa_circ_	-0.37781	0.47676
hsa_circ_	0.268004	0.476775
hsa_circ_	-0.1155	0.476782
hsa_circ_	-0.13343	0.476787
hsa_circ_	-0.14784	0.476789
hsa_circ_	-0.2469	0.47679
hsa_circ_	-0.1033	0.476791
hsa_circ_	-0.19499	0.476793
hsa_circ_	-0.19873	0.4768
hsa_circ_	0.091899	0.476848
hsa_circ_	-0.09941	0.476905
hsa_circ_	-0.15656	0.476905
hsa_circ_	-0.16809	0.47691
hsa_circ_	-0.09304	0.476923
hsa_circ_	0.248479	0.476934
hsa_circ_	0.230346	0.476945
hsa_circ_	-0.10713	0.476958
hsa_circ_	-0.13254	0.476958
hsa_circ_	0.316003	0.476964
hsa_circ_	0.118285	0.476973
hsa_circ_	0.10326	0.476973
hsa_circ_	0.129914	0.477049
hsa_circ_	-0.19166	0.477062
hsa_circ_	0.126597	0.477081
hsa_circ_	-0.09102	0.477084
hsa_circ_	0.119906	0.477085
hsa_circ_	-0.15199	0.477086
hsa_circ_	0.188698	0.477097
hsa_circ_	0.156078	0.477111
hsa_circ_	-0.11957	0.477111
hsa_circ_	-0.18026	0.47712
hsa_circ_	-0.32659	0.477154
hsa_circ_	-0.13304	0.477158
hsa_circ_	0.129527	0.477167
hsa_circ_	0.133628	0.477168
hsa_circ_	0.1319	0.477177
hsa_circ_	0.096924	0.477193
hsa_circ_	-0.1702	0.477198
hsa_circ_	-0.10668	0.477205
hsa_circ_	-0.11561	0.477227
hsa_circ_	-0.16948	0.477234
hsa_circ_	-0.13028	0.477242
hsa_circ_	-0.18676	0.477262
hsa_circ_	0.151327	0.477271
hsa_circ_	0.146239	0.477278
hsa_circ_	0.261769	0.47731
hsa_circ_	-0.19978	0.477317
hsa_circ_	0.300462	0.477322
hsa_circ_	-0.1517	0.477328
hsa_circ_	0.138121	0.477331
hsa_circ_	0.155396	0.477335
hsa_circ_	0.115782	0.477346

hsa_circ_	0.137064	0.477365
hsa_circ_	-0.10267	0.477365
hsa_circ_	0.154704	0.477383
hsa_circ_	-0.16385	0.477406
hsa_circ_	0.170514	0.477436
hsa_circ_	0.166933	0.477439
hsa_circ_	0.364204	0.477444
hsa_circ_	-0.12293	0.47746
hsa_circ_	0.227208	0.477462
hsa_circ_	0.122129	0.477477
hsa_circ_	-0.1502	0.477493
hsa_circ_	-0.50092	0.477504
hsa_circ_	0.373707	0.477521
hsa_circ_	-0.17582	0.477522
hsa_circ_	-0.19218	0.477548
hsa_circ_	-0.14039	0.477549
hsa_circ_	0.147609	0.477569
hsa_circ_	-0.1408	0.477571
hsa_circ_	-0.13025	0.477584
hsa_circ_	0.297109	0.477585
hsa_circ_	0.142831	0.477623
hsa_circ_	-0.37861	0.477625
hsa_circ_	-0.16896	0.477626
hsa_circ_	-0.32581	0.477665
hsa_circ_	0.162591	0.477666
hsa_circ_	0.374195	0.477669
hsa_circ_	-0.18396	0.477692
hsa_circ_	-0.10183	0.477705
hsa_circ_	-0.17114	0.477722
hsa_circ_	0.129022	0.477724
hsa_circ_	-0.15765	0.477743
hsa_circ_	-0.13771	0.47778
hsa_circ_	-0.25498	0.477808
hsa_circ_	-0.12666	0.477822
hsa_circ_	-1.03978	0.477841
hsa_circ_	-0.33612	0.477844
hsa_circ_	0.176661	0.477851
hsa_circ_	-0.11753	0.477856
hsa_circ_	-0.14654	0.477875
hsa_circ_	0.2107	0.477881
hsa_circ_	0.237181	0.477882
hsa_circ_	0.346068	0.477884
hsa_circ_	0.122669	0.477908
hsa_circ_	-0.15669	0.477911
hsa_circ_	0.178929	0.477911
hsa_circ_	0.2362	0.477918
hsa_circ_	0.195338	0.477934
hsa_circ_	0.290399	0.477937
hsa_circ_	0.482266	0.477962
hsa_circ_	0.129807	0.477987
hsa_circ_	0.364775	0.477991
hsa_circ_	-0.13744	0.478019
hsa_circ_	-0.21527	0.478036
hsa_circ_	0.17512	0.478044

hsa_circ_	-0.10122	0.478085
hsa_circ_	-0.27913	0.478086
hsa_circ_	0.239039	0.478087
hsa_circ_	0.120443	0.478097
hsa_circ_	-0.32651	0.478136
hsa_circ_	-0.17306	0.478146
hsa_circ_	-0.09443	0.478148
hsa_circ_	-0.17177	0.478153
hsa_circ_	0.179413	0.47816
hsa_circ_	0.269723	0.478167
hsa_circ_	-0.161	0.478181
hsa_circ_	-0.43995	0.478184
hsa_circ_	-0.18183	0.478202
hsa_circ_	0.148609	0.478214
hsa_circ_	-0.26233	0.478218
hsa_circ_	0.156299	0.478227
hsa_circ_	-0.14763	0.478233
hsa_circ_	-0.13751	0.478239
hsa_circ_	-0.0911	0.478256
hsa_circ_	-0.20373	0.478273
hsa_circ_	0.169886	0.478303
hsa_circ_	-0.16704	0.478313
hsa_circ_	-0.11877	0.478321
hsa_circ_	0.157432	0.478324
hsa_circ_	0.234924	0.478329
hsa_circ_	-0.0948	0.478336
hsa_circ_	0.103392	0.47834
hsa_circ_	-0.13841	0.478346
hsa_circ_	-0.21594	0.478377
hsa_circ_	-0.10196	0.478382
hsa_circ_	0.260539	0.478403
hsa_circ_	0.220983	0.478411
hsa_circ_	-0.11566	0.47842
hsa_circ_	-0.13421	0.478425
hsa_circ_	0.180116	0.478432
hsa_circ_	0.131081	0.478449
hsa_circ_	0.246698	0.478522
hsa_circ_	0.408282	0.478528
hsa_circ_	-0.11109	0.47854
hsa_circ_	-0.15101	0.47854
hsa_circ_	0.178506	0.478568
hsa_circ_	0.153385	0.478574
hsa_circ_	-0.12581	0.478575
hsa_circ_	-0.16002	0.478578
hsa_circ_	-1.07544	0.478588
hsa_circ_	-0.21623	0.478609
hsa_circ_	-0.19308	0.478613
hsa_circ_	0.209631	0.478631
hsa_circ_	-0.12386	0.478646
hsa_circ_	0.145862	0.478656
hsa_circ_	0.115916	0.47871
hsa_circ_	-0.10171	0.478716
hsa_circ_	-0.1358	0.478745
hsa_circ_	0.214711	0.478752

hsa_circ_	-0.22204	0.478798
hsa_circ_	0.371932	0.478799
hsa_circ_	0.358274	0.478807
hsa_circ_	-0.11005	0.478819
hsa_circ_	-0.09748	0.478821
hsa_circ_	0.122092	0.478822
hsa_circ_	-0.18774	0.478831
hsa_circ_	0.211544	0.478833
hsa_circ_	-0.08956	0.478839
hsa_circ_	-0.11931	0.478862
hsa_circ_	-0.16507	0.478875
hsa_circ_	-0.42154	0.478881
hsa_circ_	0.100502	0.47889
hsa_circ_	0.147031	0.478926
hsa_circ_	-0.16876	0.478948
hsa_circ_	-0.10987	0.478983
hsa_circ_	-0.10018	0.478993
hsa_circ_	-0.15659	0.479019
hsa_circ_	0.475185	0.479019
hsa_circ_	0.115524	0.479019
hsa_circ_	-0.49815	0.479027
hsa_circ_	-0.21649	0.479037
hsa_circ_	0.105862	0.47904
hsa_circ_	0.10007	0.479044
hsa_circ_	-0.13505	0.479054
hsa_circ_	-0.10446	0.479055
hsa_circ_	0.185228	0.479059
hsa_circ_	-0.11382	0.479067
hsa_circ_	0.326786	0.479087
hsa_circ_	0.171674	0.479092
hsa_circ_	0.124247	0.479097
hsa_circ_	-0.17165	0.479098
hsa_circ_	0.18569	0.479112
hsa_circ_	0.142423	0.479125
hsa_circ_	0.129583	0.479164
hsa_circ_	0.204799	0.479164
hsa_circ_	0.150015	0.479178
hsa_circ_	0.259239	0.479181
hsa_circ_	0.186368	0.479207
hsa_circ_	0.213262	0.479207
hsa_circ_	-0.23764	0.479208
hsa_circ_	-0.14883	0.479245
hsa_circ_	0.171511	0.479249
hsa_circ_	-0.23654	0.479257
hsa_circ_	0.118615	0.479264
hsa_circ_	0.120153	0.47928
hsa_circ_	-0.12796	0.479283
hsa_circ_	-0.18542	0.479287
hsa_circ_	-0.20678	0.479287
hsa_circ_	-0.19615	0.479326
hsa_circ_	0.229233	0.479328
hsa_circ_	-0.17258	0.479373
hsa_circ_	0.154634	0.479379
hsa_circ_	0.128655	0.479386

hsa_circ_	-0.14036	0.479386
hsa_circ_	-0.22144	0.479387
hsa_circ_	-0.1284	0.47941
hsa_circ_	-0.10953	0.479414
hsa_circ_	0.191619	0.47944
hsa_circ_	0.21738	0.479448
hsa_circ_	-0.13449	0.479457
hsa_circ_	0.176566	0.479474
hsa_circ_	-0.14609	0.479492
hsa_circ_	-0.20925	0.479499
hsa_circ_	0.355022	0.479503
hsa_circ_	-0.08989	0.479503
hsa_circ_	0.109214	0.479512
hsa_circ_	-0.15728	0.47953
hsa_circ_	-0.22505	0.479534
hsa_circ_	-0.43602	0.479537
hsa_circ_	0.26563	0.47955
hsa_circ_	-0.16405	0.479551
hsa_circ_	-0.15118	0.479575
hsa_circ_	0.224322	0.479586
hsa_circ_	-0.10646	0.479597
hsa_circ_	-0.11476	0.479624
hsa_circ_	-0.16583	0.479644
hsa_circ_	0.127802	0.479648
hsa_circ_	0.170369	0.479653
hsa_circ_	-0.14919	0.479655
hsa_circ_	-0.09818	0.479692
hsa_circ_	0.460136	0.479708
hsa_circ_	0.109651	0.479711
hsa_circ_	0.134008	0.479717
hsa_circ_	0.278733	0.47974
hsa_circ_	-0.11765	0.479743
hsa_circ_	-0.28917	0.479746
hsa_circ_	-0.37186	0.479748
hsa_circ_	-0.16975	0.479765
hsa_circ_	0.151584	0.479781
hsa_circ_	-0.33956	0.479784
hsa_circ_	-0.13256	0.479788
hsa_circ_	-0.18417	0.479808
hsa_circ_	0.142716	0.479814
hsa_circ_	0.14969	0.479818
hsa_circ_	-0.16932	0.47983
hsa_circ_	0.100258	0.479836
hsa_circ_	0.20501	0.479836
hsa_circ_	0.259256	0.479863
hsa_circ_	0.23646	0.47989
hsa_circ_	0.419595	0.479954
hsa_circ_	-0.12949	0.479955
hsa_circ_	0.315248	0.47996
hsa_circ_	-0.13033	0.479975
hsa_circ_	-0.16895	0.479991
hsa_circ_	-0.09284	0.479991
hsa_circ_	-0.3157	0.479997
hsa_circ_	-0.17558	0.480004

hsa_circ_	-0.18674	0.480019
hsa_circ_	-0.24815	0.48002
hsa_circ_	-0.12467	0.480024
hsa_circ_	-0.16313	0.480036
hsa_circ_	0.125717	0.48004
hsa_circ_	0.275319	0.480052
hsa_circ_	0.122659	0.480056
hsa_circ_	-0.10637	0.480059
hsa_circ_	-0.09283	0.48014
hsa_circ_	0.1291	0.48014
hsa_circ_	-0.2626	0.480144
hsa_circ_	-0.21322	0.48018
hsa_circ_	-0.20001	0.480195
hsa_circ_	-0.09796	0.480208
hsa_circ_	0.437791	0.480212
hsa_circ_	-0.90701	0.480217
hsa_circ_	-0.10648	0.480237
hsa_circ_	-0.27919	0.480246
hsa_circ_	0.142644	0.480264
hsa_circ_	0.224138	0.480274
hsa_circ_	0.127772	0.480301
hsa_circ_	-0.17025	0.480306
hsa_circ_	0.201208	0.480325
hsa_circ_	0.174587	0.480336
hsa_circ_	-0.30363	0.480378
hsa_circ_	-0.11173	0.480403
hsa_circ_	0.092775	0.480403
hsa_circ_	-0.132	0.480408
hsa_circ_	-0.20029	0.480418
hsa_circ_	0.093348	0.480424
hsa_circ_	-0.1039	0.480452
hsa_circ_	0.131168	0.480477
hsa_circ_	0.131676	0.480478
hsa_circ_	-0.45643	0.480491
hsa_circ_	0.119226	0.480493
hsa_circ_	-0.3342	0.480501
hsa_circ_	-0.38854	0.480517
hsa_circ_	-0.18274	0.480517
hsa_circ_	0.114087	0.480519
hsa_circ_	0.171246	0.48055
hsa_circ_	-0.10422	0.480578
hsa_circ_	-0.09725	0.480581
hsa_circ_	-0.11214	0.480589
hsa_circ_	-0.12939	0.480591
hsa_circ_	-0.15872	0.480602
hsa_circ_	-0.10133	0.480645
hsa_circ_	0.154144	0.480648
hsa_circ_	-0.23898	0.480661
hsa_circ_	-0.19414	0.480667
hsa_circ_	-0.10825	0.480676
hsa_circ_	-0.15115	0.480688
hsa_circ_	-0.14796	0.480701
hsa_circ_	-0.14919	0.480708
hsa_circ_	0.114342	0.480732

hsa_circ_	-0.21263	0.480735
hsa_circ_	0.571157	0.480737
hsa_circ_	0.299438	0.480742
hsa_circ_	-0.16609	0.480751
hsa_circ_	0.264326	0.480757
hsa_circ_	0.164841	0.480774
hsa_circ_	0.102309	0.480777
hsa_circ_	-0.09655	0.480805
hsa_circ_	-0.16821	0.480811
hsa_circ_	0.208119	0.480811
hsa_circ_	0.234954	0.480814
hsa_circ_	-0.1132	0.480816
hsa_circ_	-0.111	0.480819
hsa_circ_	0.279089	0.480849
hsa_circ_	-0.24585	0.480852
hsa_circ_	0.204912	0.480879
hsa_circ_	0.103493	0.4809
hsa_circ_	0.114386	0.480907
hsa_circ_	0.136371	0.480919
hsa_circ_	0.148157	0.480935
hsa_circ_	0.101645	0.480938
hsa_circ_	0.34951	0.480942
hsa_circ_	0.249691	0.480944
hsa_circ_	-0.11024	0.480956
hsa_circ_	-0.16817	0.480965
hsa_circ_	-0.12263	0.480965
hsa_circ_	-0.1606	0.480978
hsa_circ_	0.145491	0.480981
hsa_circ_	0.10677	0.481
hsa_circ_	0.111723	0.481009
hsa_circ_	0.116564	0.481025
hsa_circ_	0.321919	0.481049
hsa_circ_	-0.10694	0.481055
hsa_circ_	0.117273	0.481067
hsa_circ_	-0.13154	0.481073
hsa_circ_	-0.15053	0.481079
hsa_circ_	-0.13041	0.481099
hsa_circ_	0.335982	0.481106
hsa_circ_	0.156651	0.481108
hsa_circ_	-0.14526	0.481109
hsa_circ_	-0.0966	0.481126
hsa_circ_	-0.15637	0.481138
hsa_circ_	-0.1567	0.481156
hsa_circ_	0.107304	0.481168
hsa_circ_	0.122438	0.481173
hsa_circ_	-0.10959	0.481175
hsa_circ_	-0.1519	0.481184
hsa_circ_	-0.12076	0.48119
hsa_circ_	0.107103	0.481204
hsa_circ_	0.315946	0.481234
hsa_circ_	0.223036	0.481242
hsa_circ_	0.211809	0.481258
hsa_circ_	-0.47295	0.481265
hsa_circ_	-0.16152	0.481277

hsa_circ_	-0.34613	0.481331
hsa_circ_	-0.13989	0.48134
hsa_circ_	-0.13367	0.481361
hsa_circ_	0.12832	0.481363
hsa_circ_	0.285477	0.481378
hsa_circ_	-0.21433	0.481395
hsa_circ_	-0.34136	0.481401
hsa_circ_	0.201817	0.481403
hsa_circ_	-0.28265	0.481424
hsa_circ_	-0.16883	0.481448
hsa_circ_	-0.16145	0.481468
hsa_circ_	-0.16519	0.481514
hsa_circ_	0.298123	0.481531
hsa_circ_	0.163165	0.481538
hsa_circ_	-0.2211	0.481539
hsa_circ_	-0.11048	0.48156
hsa_circ_	-0.13068	0.481562
hsa_circ_	-0.36475	0.48157
hsa_circ_	-0.09302	0.481575
hsa_circ_	0.111746	0.481621
hsa_circ_	0.120061	0.481634
hsa_circ_	-0.29214	0.481646
hsa_circ_	-0.25781	0.481646
hsa_circ_	0.138	0.48165
hsa_circ_	-0.11892	0.481654
hsa_circ_	-0.33788	0.481658
hsa_circ_	-0.10611	0.481672
hsa_circ_	-0.19291	0.481675
hsa_circ_	0.158839	0.481722
hsa_circ_	0.142804	0.481751
hsa_circ_	-0.21329	0.481757
hsa_circ_	-0.22312	0.481808
hsa_circ_	0.098087	0.481811
hsa_circ_	0.11196	0.481816
hsa_circ_	-0.11387	0.481824
hsa_circ_	0.120722	0.481831
hsa_circ_	-0.30391	0.481842
hsa_circ_	-0.12407	0.481843
hsa_circ_	-0.14535	0.481847
hsa_circ_	0.100988	0.48185
hsa_circ_	-0.10265	0.481856
hsa_circ_	-0.68141	0.481864
hsa_circ_	-0.24098	0.481881
hsa_circ_	-0.10179	0.481892
hsa_circ_	0.10518	0.481893
hsa_circ_	-0.20953	0.481909
hsa_circ_	-0.16558	0.481909
hsa_circ_	-0.12054	0.481912
hsa_circ_	0.173745	0.481914
hsa_circ_	0.158919	0.481919
hsa_circ_	0.198937	0.481932
hsa_circ_	-0.17786	0.481934
hsa_circ_	-0.2282	0.481948
hsa_circ_	0.170647	0.481949

hsa_circ_	-0.0998	0.481967
hsa_circ_	-0.71526	0.481968
hsa_circ_	-0.22891	0.481972
hsa_circ_	-0.1814	0.481978
hsa_circ_	0.151333	0.482018
hsa_circ_	0.098695	0.482027
hsa_circ_	-0.25429	0.482037
hsa_circ_	-0.1982	0.482073
hsa_circ_	0.192402	0.482079
hsa_circ_	0.279653	0.482083
hsa_circ_	-0.11976	0.48209
hsa_circ_	0.105635	0.482091
hsa_circ_	0.314567	0.482135
hsa_circ_	0.116565	0.482147
hsa_circ_	0.250709	0.482154
hsa_circ_	0.110136	0.482155
hsa_circ_	-0.33737	0.482155
hsa_circ_	0.199757	0.482169
hsa_circ_	0.164925	0.48217
hsa_circ_	-0.20778	0.482184
hsa_circ_	-0.36162	0.482193
hsa_circ_	-0.17934	0.482195
hsa_circ_	-0.12041	0.482208
hsa_circ_	0.268811	0.482215
hsa_circ_	-0.13996	0.482222
hsa_circ_	-0.12616	0.482245
hsa_circ_	0.345487	0.482245
hsa_circ_	0.369785	0.482261
hsa_circ_	-0.32746	0.482292
hsa_circ_	0.148376	0.482313
hsa_circ_	-0.26346	0.482336
hsa_circ_	-0.1075	0.48235
hsa_circ_	-0.22391	0.482362
hsa_circ_	0.42083	0.482371
hsa_circ_	-0.26301	0.482374
hsa_circ_	0.113755	0.48238
hsa_circ_	0.134672	0.482383
hsa_circ_	-0.36251	0.482388
hsa_circ_	0.331181	0.482403
hsa_circ_	-0.11706	0.482413
hsa_circ_	0.174034	0.482434
hsa_circ_	0.115578	0.482439
hsa_circ_	-0.15792	0.48244
hsa_circ_	-0.53597	0.48245
hsa_circ_	-0.35984	0.482456
hsa_circ_	0.159254	0.482479
hsa_circ_	-0.14248	0.482493
hsa_circ_	0.193371	0.482509
hsa_circ_	0.163032	0.48251
hsa_circ_	0.095358	0.482534
hsa_circ_	0.154333	0.482554
hsa_circ_	-0.55367	0.482557
hsa_circ_	0.107093	0.482562
hsa_circ_	0.198923	0.482563

hsa_circ_	0.291501	0.482581
hsa_circ_	-0.09843	0.482582
hsa_circ_	0.284818	0.482583
hsa_circ_	0.242392	0.482592
hsa_circ_	0.118782	0.482606
hsa_circ_	0.110221	0.482615
hsa_circ_	-0.09627	0.482622
hsa_circ_	0.261071	0.482624
hsa_circ_	-0.17882	0.482657
hsa_circ_	0.141909	0.482657
hsa_circ_	-0.17205	0.482663
hsa_circ_	0.091745	0.482679
hsa_circ_	0.273132	0.482692
hsa_circ_	0.15369	0.482702
hsa_circ_	-0.35174	0.482702
hsa_circ_	0.12885	0.482749
hsa_circ_	0.224726	0.482761
hsa_circ_	0.209954	0.48278
hsa_circ_	0.165959	0.482827
hsa_circ_	-0.17078	0.48284
hsa_circ_	0.168603	0.48285
hsa_circ_	0.158316	0.482865
hsa_circ_	-0.10747	0.482871
hsa_circ_	-0.11681	0.482875
hsa_circ_	-0.15565	0.482883
hsa_circ_	-0.18674	0.482894
hsa_circ_	0.419065	0.482916
hsa_circ_	-0.4725	0.482923
hsa_circ_	-0.52091	0.482924
hsa_circ_	-0.14498	0.482924
hsa_circ_	0.168217	0.482927
hsa_circ_	-0.31374	0.482935
hsa_circ_	-0.11222	0.482951
hsa_circ_	0.194238	0.482963
hsa_circ_	-0.09713	0.482963
hsa_circ_	0.111895	0.482986
hsa_circ_	0.091325	0.483011
hsa_circ_	-0.15087	0.483014
hsa_circ_	0.115721	0.483014
hsa_circ_	0.326741	0.48302
hsa_circ_	-0.11892	0.483021
hsa_circ_	-0.18111	0.483026
hsa_circ_	-0.19674	0.483039
hsa_circ_	-0.12534	0.483041
hsa_circ_	0.189938	0.483043
hsa_circ_	-0.24647	0.483054
hsa_circ_	0.117922	0.483072
hsa_circ_	0.119875	0.483121
hsa_circ_	-0.13024	0.483125
hsa_circ_	0.1144	0.483146
hsa_circ_	-0.35081	0.483175
hsa_circ_	0.144272	0.483191
hsa_circ_	0.173436	0.483197
hsa_circ_	0.145799	0.483205

hsa_circ_	-0.21358	0.483206
hsa_circ_	-0.16426	0.483206
hsa_circ_	0.131803	0.483213
hsa_circ_	-0.14842	0.483213
hsa_circ_	-0.1022	0.483219
hsa_circ_	-0.20019	0.483233
hsa_circ_	-0.20331	0.483261
hsa_circ_	-0.09418	0.483271
hsa_circ_	0.256401	0.483273
hsa_circ_	0.101492	0.483298
hsa_circ_	0.116422	0.483301
hsa_circ_	-0.18932	0.483304
hsa_circ_	-0.1457	0.48331
hsa_circ_	-0.11567	0.483319
hsa_circ_	-0.23678	0.483324
hsa_circ_	-0.13307	0.48333
hsa_circ_	-0.11402	0.483339
hsa_circ_	0.246835	0.483354
hsa_circ_	-0.15062	0.483358
hsa_circ_	0.449092	0.483393
hsa_circ_	0.095416	0.483394
hsa_circ_	0.140844	0.483396
hsa_circ_	-0.43345	0.483409
hsa_circ_	0.142919	0.483414
hsa_circ_	-0.1001	0.483433
hsa_circ_	-0.11944	0.483473
hsa_circ_	-0.21267	0.483474
hsa_circ_	-0.17116	0.483497
hsa_circ_	-0.20369	0.483505
hsa_circ_	0.095991	0.483509
hsa_circ_	0.097896	0.483539
hsa_circ_	0.121121	0.483552
hsa_circ_	0.121401	0.483555
hsa_circ_	0.109825	0.483561
hsa_circ_	-0.14589	0.48359
hsa_circ_	-0.21451	0.483614
hsa_circ_	0.124861	0.483652
hsa_circ_	-0.20825	0.483655
hsa_circ_	0.119535	0.483677
hsa_circ_	0.146335	0.483677
hsa_circ_	-0.41824	0.483696
hsa_circ_	-0.29301	0.4837
hsa_circ_	-0.41673	0.483725
hsa_circ_	-0.21808	0.483731
hsa_circ_	0.170819	0.483735
hsa_circ_	0.295473	0.483738
hsa_circ_	0.107023	0.483759
hsa_circ_	-0.11425	0.483794
hsa_circ_	-0.23546	0.483809
hsa_circ_	0.110709	0.483819
hsa_circ_	0.118335	0.483831
hsa_circ_	0.106715	0.483833
hsa_circ_	-0.15739	0.483834
hsa_circ_	-0.17706	0.483886

hsa_circ_	-0.17541	0.483909
hsa_circ_	0.408827	0.483909
hsa_circ_	-0.16965	0.483915
hsa_circ_	0.343372	0.483922
hsa_circ_	0.146316	0.483982
hsa_circ_	0.171925	0.484012
hsa_circ_	0.141566	0.484051
hsa_circ_	-0.13783	0.484087
hsa_circ_	0.243596	0.484113
hsa_circ_	-0.3083	0.484118
hsa_circ_	0.199064	0.484122
hsa_circ_	-0.12974	0.484154
hsa_circ_	-0.22595	0.484164
hsa_circ_	-0.94413	0.484173
hsa_circ_	0.230658	0.484206
hsa_circ_	0.449557	0.484233
hsa_circ_	0.173192	0.484242
hsa_circ_	-0.09891	0.484248
hsa_circ_	-0.11783	0.484251
hsa_circ_	-0.25974	0.484265
hsa_circ_	0.10849	0.484271
hsa_circ_	-0.12215	0.484288
hsa_circ_	-0.16922	0.484294
hsa_circ_	0.343195	0.484311
hsa_circ_	0.122444	0.484321
hsa_circ_	-0.17898	0.484331
hsa_circ_	-0.15227	0.484336
hsa_circ_	-0.11149	0.484341
hsa_circ_	-0.14546	0.484341
hsa_circ_	0.318147	0.484343
hsa_circ_	0.441191	0.484363
hsa_circ_	0.121587	0.484383
hsa_circ_	0.226331	0.484413
hsa_circ_	0.166761	0.484421
hsa_circ_	-0.27468	0.484425
hsa_circ_	-0.16322	0.484429
hsa_circ_	0.111238	0.484457
hsa_circ_	0.123891	0.484463
hsa_circ_	0.118922	0.484467
hsa_circ_	-0.09855	0.484467
hsa_circ_	0.153704	0.484469
hsa_circ_	-0.45479	0.484476
hsa_circ_	0.171082	0.484487
hsa_circ_	0.194389	0.484489
hsa_circ_	-0.42524	0.48449
hsa_circ_	-0.13393	0.484491
hsa_circ_	-0.21076	0.484497
hsa_circ_	0.126072	0.484513
hsa_circ_	0.110609	0.484514
hsa_circ_	0.191305	0.484521
hsa_circ_	0.155122	0.484524
hsa_circ_	0.688752	0.484526
hsa_circ_	0.114039	0.484554
hsa_circ_	0.390303	0.484558

hsa_circ_	0.124426	0.484561
hsa_circ_	0.533746	0.484576
hsa_circ_	-0.11728	0.484584
hsa_circ_	0.156471	0.484623
hsa_circ_	-0.13428	0.484643
hsa_circ_	0.114389	0.484656
hsa_circ_	0.302829	0.484663
hsa_circ_	-0.22869	0.484671
hsa_circ_	-0.36427	0.484677
hsa_circ_	-0.15368	0.484681
hsa_circ_	-0.15486	0.484683
hsa_circ_	-0.16507	0.484705
hsa_circ_	0.201013	0.484708
hsa_circ_	-0.10235	0.484715
hsa_circ_	-0.23815	0.484731
hsa_circ_	0.182518	0.484785
hsa_circ_	-0.51384	0.4848
hsa_circ_	-0.10882	0.484809
hsa_circ_	0.143335	0.484837
hsa_circ_	-0.24275	0.484861
hsa_circ_	-0.13724	0.484862
hsa_circ_	0.113862	0.484891
hsa_circ_	-0.10588	0.484891
hsa_circ_	-0.2397	0.484907
hsa_circ_	-0.2777	0.484928
hsa_circ_	0.348477	0.48494
hsa_circ_	-0.10852	0.484956
hsa_circ_	0.13947	0.484985
hsa_circ_	-0.1625	0.484985
hsa_circ_	-0.10485	0.484989
hsa_circ_	-0.14408	0.48501
hsa_circ_	-0.18638	0.485015
hsa_circ_	0.225952	0.485048
hsa_circ_	-0.23128	0.485059
hsa_circ_	-0.34397	0.485077
hsa_circ_	-0.10412	0.485079
hsa_circ_	-0.1451	0.485083
hsa_circ_	-0.09227	0.485099
hsa_circ_	-0.3325	0.485101
hsa_circ_	-0.09316	0.485104
hsa_circ_	-0.32408	0.485112
hsa_circ_	0.147321	0.48512
hsa_circ_	0.117146	0.485212
hsa_circ_	0.264118	0.485223
hsa_circ_	0.119336	0.485228
hsa_circ_	-0.14642	0.485231
hsa_circ_	0.172441	0.485234
hsa_circ_	0.204447	0.485236
hsa_circ_	-0.22926	0.48525
hsa_circ_	0.15521	0.485256
hsa_circ_	0.230407	0.485261
hsa_circ_	-0.13283	0.485277
hsa_circ_	0.096821	0.485278
hsa_circ_	0.099331	0.48528

hsa_circ_	0.197941	0.485285
hsa_circ_	-0.12692	0.485291
hsa_circ_	-0.20519	0.4853
hsa_circ_	0.183636	0.485302
hsa_circ_	0.165201	0.485305
hsa_circ_	-0.12777	0.485315
hsa_circ_	-0.30379	0.48532
hsa_circ_	-0.16184	0.485325
hsa_circ_	-0.10554	0.485337
hsa_circ_	-0.14648	0.485353
hsa_circ_	-0.09297	0.48536
hsa_circ_	0.143595	0.485372
hsa_circ_	-0.15671	0.485382
hsa_circ_	-0.12347	0.485397
hsa_circ_	0.225044	0.485409
hsa_circ_	-0.14409	0.485416
hsa_circ_	-0.09168	0.485435
hsa_circ_	-0.11594	0.485439
hsa_circ_	-0.14318	0.485477
hsa_circ_	-0.28958	0.485497
hsa_circ_	-0.1444	0.485503
hsa_circ_	-0.24009	0.485513
hsa_circ_	-0.42867	0.485517
hsa_circ_	0.113783	0.485518
hsa_circ_	0.339094	0.485541
hsa_circ_	0.14789	0.485557
hsa_circ_	0.248077	0.485574
hsa_circ_	0.283308	0.485582
hsa_circ_	-0.10324	0.485588
hsa_circ_	-0.3587	0.485597
hsa_circ_	-0.10764	0.485611
hsa_circ_	-0.1145	0.485628
hsa_circ_	-0.21047	0.485631
hsa_circ_	0.331018	0.485636
hsa_circ_	-0.11947	0.48564
hsa_circ_	0.223676	0.48564
hsa_circ_	-0.24037	0.485651
hsa_circ_	-0.27885	0.485667
hsa_circ_	-0.12303	0.485676
hsa_circ_	0.162667	0.485693
hsa_circ_	-0.10203	0.485697
hsa_circ_	0.115314	0.485715
hsa_circ_	0.399946	0.485738
hsa_circ_	0.180953	0.485772
hsa_circ_	-0.18818	0.485777
hsa_circ_	-0.17547	0.485785
hsa_circ_	-0.8943	0.485788
hsa_circ_	-0.32138	0.485815
hsa_circ_	-0.13963	0.485838
hsa_circ_	-0.09515	0.485844
hsa_circ_	0.211606	0.485845
hsa_circ_	0.141271	0.48591
hsa_circ_	-0.0952	0.485917
hsa_circ_	0.134429	0.485943

hsa_circ_	-0.41609	0.485978
hsa_circ_	-0.17469	0.486001
hsa_circ_	0.120495	0.486007
hsa_circ_	-0.25329	0.486009
hsa_circ_	0.148753	0.48601
hsa_circ_	0.22581	0.486046
hsa_circ_	-0.10376	0.486063
hsa_circ_	0.092067	0.486077
hsa_circ_	0.421629	0.486092
hsa_circ_	-0.47291	0.486118
hsa_circ_	-0.11069	0.486124
hsa_circ_	0.166734	0.486138
hsa_circ_	-0.45479	0.486154
hsa_circ_	0.117706	0.486163
hsa_circ_	0.236174	0.486187
hsa_circ_	0.288638	0.486189
hsa_circ_	-0.55837	0.486197
hsa_circ_	-0.09355	0.486219
hsa_circ_	0.179911	0.486227
hsa_circ_	-0.13577	0.486235
hsa_circ_	-0.21912	0.486246
hsa_circ_	-0.35208	0.486253
hsa_circ_	-0.09954	0.486256
hsa_circ_	0.143228	0.486264
hsa_circ_	0.111974	0.486276
hsa_circ_	0.128853	0.486281
hsa_circ_	-0.10635	0.486304
hsa_circ_	-0.16932	0.486319
hsa_circ_	-0.09305	0.486325
hsa_circ_	0.136833	0.486334
hsa_circ_	0.109879	0.486338
hsa_circ_	0.195068	0.48636
hsa_circ_	-0.2327	0.486372
hsa_circ_	-0.12051	0.486386
hsa_circ_	-0.33947	0.486413
hsa_circ_	0.366999	0.486438
hsa_circ_	-0.27327	0.486445
hsa_circ_	-0.13171	0.486492
hsa_circ_	0.105393	0.486498
hsa_circ_	0.442049	0.486504
hsa_circ_	0.272116	0.486526
hsa_circ_	-0.11823	0.48653
hsa_circ_	-0.11342	0.486569
hsa_circ_	0.122465	0.486577
hsa_circ_	0.398124	0.486581
hsa_circ_	-0.13743	0.486682
hsa_circ_	-0.35709	0.486684
hsa_circ_	-0.25275	0.486697
hsa_circ_	-0.14091	0.486718
hsa_circ_	-0.09978	0.486725
hsa_circ_	0.174089	0.486751
hsa_circ_	-0.2719	0.486758
hsa_circ_	-0.16842	0.486768
hsa_circ_	-0.1455	0.486809

hsa_circ_	-0.08815	0.486819
hsa_circ_	0.147022	0.486827
hsa_circ_	-0.10872	0.486841
hsa_circ_	0.326507	0.486841
hsa_circ_	0.18543	0.486851
hsa_circ_	-0.14385	0.486855
hsa_circ_	-0.21365	0.486862
hsa_circ_	0.228761	0.486873
hsa_circ_	0.130326	0.48688
hsa_circ_	0.103282	0.486883
hsa_circ_	0.206988	0.486888
hsa_circ_	-0.15548	0.486903
hsa_circ_	-0.13506	0.486914
hsa_circ_	0.144243	0.486924
hsa_circ_	0.108445	0.486925
hsa_circ_	-0.28377	0.486926
hsa_circ_	0.341387	0.486976
hsa_circ_	0.1029	0.487005
hsa_circ_	0.118933	0.487059
hsa_circ_	-0.14082	0.487116
hsa_circ_	-0.10025	0.487142
hsa_circ_	0.146505	0.487145
hsa_circ_	0.33552	0.487156
hsa_circ_	0.160986	0.487172
hsa_circ_	0.207591	0.48718
hsa_circ_	-0.25951	0.487218
hsa_circ_	0.315284	0.48723
hsa_circ_	-0.20951	0.48724
hsa_circ_	-0.09462	0.487245
hsa_circ_	-0.27209	0.487268
hsa_circ_	0.259724	0.487269
hsa_circ_	0.128959	0.48727
hsa_circ_	-0.09521	0.487278
hsa_circ_	-0.1333	0.487308
hsa_circ_	-0.25447	0.487318
hsa_circ_	-0.09048	0.487324
hsa_circ_	0.164984	0.487395
hsa_circ_	0.307143	0.487413
hsa_circ_	0.126837	0.487419
hsa_circ_	-0.26582	0.487445
hsa_circ_	0.203803	0.48745
hsa_circ_	-0.47666	0.487455
hsa_circ_	-0.10285	0.487455
hsa_circ_	-0.7484	0.487497
hsa_circ_	0.124471	0.487519
hsa_circ_	0.165905	0.487531
hsa_circ_	0.347235	0.487537
hsa_circ_	0.104381	0.487572
hsa_circ_	-0.09947	0.487586
hsa_circ_	0.115014	0.487595
hsa_circ_	-0.11978	0.487617
hsa_circ_	-0.31657	0.487649
hsa_circ_	-0.10313	0.487661
hsa_circ_	-0.38662	0.487674

hsa_circ_	0.264947	0.487708
hsa_circ_	-0.10207	0.487726
hsa_circ_	-0.16314	0.487734
hsa_circ_	0.100188	0.487752
hsa_circ_	-0.17243	0.487791
hsa_circ_	0.197191	0.487794
hsa_circ_	0.145735	0.487829
hsa_circ_	0.141342	0.487835
hsa_circ_	-0.13002	0.487848
hsa_circ_	-0.20404	0.48786
hsa_circ_	-0.13203	0.487864
hsa_circ_	0.197498	0.48787
hsa_circ_	0.131433	0.487902
hsa_circ_	-0.12072	0.487923
hsa_circ_	0.319385	0.487927
hsa_circ_	0.130477	0.487991
hsa_circ_	-0.12094	0.488017
hsa_circ_	0.598744	0.488026
hsa_circ_	0.100036	0.488035
hsa_circ_	0.195696	0.488045
hsa_circ_	-0.20233	0.488065
hsa_circ_	0.22391	0.488073
hsa_circ_	-0.17267	0.488086
hsa_circ_	0.133886	0.488103
hsa_circ_	-0.46882	0.488106
hsa_circ_	0.239861	0.488106
hsa_circ_	0.095085	0.488106
hsa_circ_	-0.10226	0.488126
hsa_circ_	0.112847	0.488128
hsa_circ_	0.100195	0.488143
hsa_circ_	-0.15055	0.488147
hsa_circ_	-0.53783	0.488149
hsa_circ_	-0.25079	0.488153
hsa_circ_	0.138369	0.488186
hsa_circ_	-0.14186	0.488205
hsa_circ_	-0.13454	0.488219
hsa_circ_	0.116363	0.488226
hsa_circ_	-0.18831	0.488228
hsa_circ_	-0.2086	0.488237
hsa_circ_	-0.38297	0.488241
hsa_circ_	-0.09837	0.488244
hsa_circ_	-0.17686	0.488263
hsa_circ_	0.206508	0.488266
hsa_circ_	0.099222	0.488285
hsa_circ_	-0.22487	0.48829
hsa_circ_	0.259455	0.4883
hsa_circ_	0.103963	0.488302
hsa_circ_	-0.31874	0.488304
hsa_circ_	0.096258	0.488318
hsa_circ_	-0.13124	0.48834
hsa_circ_	-0.12895	0.488349
hsa_circ_	0.172342	0.488354
hsa_circ_	-0.17389	0.48836
hsa_circ_	0.183574	0.488363

hsa_circ_	-0.21098	0.488367
hsa_circ_	0.13017	0.488371
hsa_circ_	-0.27044	0.488378
hsa_circ_	-0.10327	0.488394
hsa_circ_	-0.23014	0.488397
hsa_circ_	0.115967	0.488415
hsa_circ_	0.120747	0.488417
hsa_circ_	-0.10395	0.488465
hsa_circ_	-0.233	0.488496
hsa_circ_	-0.21572	0.4885
hsa_circ_	-0.25056	0.488504
hsa_circ_	0.103956	0.488548
hsa_circ_	-0.09826	0.488555
hsa_circ_	0.323949	0.488557
hsa_circ_	0.165647	0.488606
hsa_circ_	0.131746	0.488607
hsa_circ_	0.293054	0.48862
hsa_circ_	-0.10982	0.488631
hsa_circ_	-0.29473	0.488633
hsa_circ_	0.14127	0.48864
hsa_circ_	-0.10231	0.488642
hsa_circ_	0.311882	0.488653
hsa_circ_	-0.16119	0.488667
hsa_circ_	-0.12092	0.488667
hsa_circ_	0.181703	0.488684
hsa_circ_	-0.1707	0.488685
hsa_circ_	0.109573	0.488693
hsa_circ_	-0.11455	0.488703
hsa_circ_	0.253867	0.488708
hsa_circ_	0.132538	0.488718
hsa_circ_	0.108998	0.488737
hsa_circ_	-0.1567	0.488742
hsa_circ_	-0.15051	0.488763
hsa_circ_	-0.30683	0.488791
hsa_circ_	0.10091	0.488809
hsa_circ_	0.09831	0.488817
hsa_circ_	0.150959	0.488823
hsa_circ_	-0.11859	0.48883
hsa_circ_	0.109782	0.488849
hsa_circ_	-0.38412	0.488917
hsa_circ_	-0.30139	0.488925
hsa_circ_	-0.16942	0.488929
hsa_circ_	0.121899	0.488934
hsa_circ_	0.148326	0.488964
hsa_circ_	-0.0968	0.488979
hsa_circ_	0.147418	0.48898
hsa_circ_	0.139177	0.489032
hsa_circ_	0.131998	0.489034
hsa_circ_	0.112724	0.489035
hsa_circ_	-0.11372	0.48905
hsa_circ_	-0.17404	0.489133
hsa_circ_	0.149322	0.489136
hsa_circ_	-0.23217	0.489136
hsa_circ_	-0.42805	0.489136

hsa_circ_	0.114854	0.489143
hsa_circ_	-0.11476	0.489163
hsa_circ_	-0.18089	0.489164
hsa_circ_	-0.14997	0.489169
hsa_circ_	-0.11352	0.489187
hsa_circ_	0.305364	0.489187
hsa_circ_	0.319576	0.489187
hsa_circ_	0.130674	0.489188
hsa_circ_	-0.12523	0.489189
hsa_circ_	0.106943	0.489206
hsa_circ_	0.187817	0.489231
hsa_circ_	0.442647	0.489233
hsa_circ_	-0.13862	0.489261
hsa_circ_	0.11411	0.489262
hsa_circ_	-0.11773	0.489277
hsa_circ_	0.109136	0.489282
hsa_circ_	0.132053	0.489291
hsa_circ_	-0.11115	0.489299
hsa_circ_	0.211949	0.489308
hsa_circ_	0.271268	0.489316
hsa_circ_	-0.37728	0.48932
hsa_circ_	0.135525	0.489327
hsa_circ_	0.217712	0.48934
hsa_circ_	0.157367	0.48936
hsa_circ_	0.310778	0.489392
hsa_circ_	-0.17925	0.489406
hsa_circ_	0.256733	0.489407
hsa_circ_	0.168538	0.489426
hsa_circ_	0.105483	0.489427
hsa_circ_	-0.13979	0.48943
hsa_circ_	0.148373	0.489433
hsa_circ_	0.328576	0.489443
hsa_circ_	-0.1966	0.489446
hsa_circ_	0.193923	0.489453
hsa_circ_	0.478552	0.489514
hsa_circ_	0.127516	0.489518
hsa_circ_	-0.18873	0.489533
hsa_circ_	0.100648	0.489539
hsa_circ_	-0.14255	0.489565
hsa_circ_	-0.11589	0.489594
hsa_circ_	-0.0924	0.489612
hsa_circ_	0.268596	0.489632
hsa_circ_	-0.14262	0.489663
hsa_circ_	0.136874	0.489681
hsa_circ_	0.11647	0.489682
hsa_circ_	-0.45406	0.489687
hsa_circ_	0.105475	0.489689
hsa_circ_	-0.1123	0.489698
hsa_circ_	-0.27531	0.4897
hsa_circ_	0.110232	0.489702
hsa_circ_	-0.20637	0.489719
hsa_circ_	0.112895	0.48972
hsa_circ_	0.106703	0.489739
hsa_circ_	0.147604	0.489778

hsa_circ_	0.286239	0.489792
hsa_circ_	-0.43055	0.489832
hsa_circ_	0.124456	0.489842
hsa_circ_	0.113745	0.489854
hsa_circ_	0.119554	0.489883
hsa_circ_	-0.28218	0.489908
hsa_circ_	0.290263	0.48994
hsa_circ_	-0.11857	0.489944
hsa_circ_	-0.83197	0.489956
hsa_circ_	-0.10592	0.490006
hsa_circ_	0.474594	0.490007
hsa_circ_	-0.18891	0.490044
hsa_circ_	-0.15961	0.490059
hsa_circ_	-0.10313	0.490064
hsa_circ_	0.145717	0.490071
hsa_circ_	0.534034	0.490075
hsa_circ_	0.37966	0.490095
hsa_circ_	0.17449	0.490108
hsa_circ_	-0.12081	0.490115
hsa_circ_	0.112988	0.490117
hsa_circ_	-0.14783	0.490128
hsa_circ_	-0.11942	0.490164
hsa_circ_	0.169173	0.490173
hsa_circ_	0.143966	0.490182
hsa_circ_	-0.47981	0.490196
hsa_circ_	0.108431	0.490197
hsa_circ_	-0.37109	0.490198
hsa_circ_	0.311535	0.490199
hsa_circ_	0.095694	0.490244
hsa_circ_	0.195185	0.490292
hsa_circ_	-0.10599	0.490295
hsa_circ_	-0.09987	0.490299
hsa_circ_	-0.24207	0.4903
hsa_circ_	-0.20213	0.490311
hsa_circ_	-0.09739	0.490316
hsa_circ_	0.500826	0.490323
hsa_circ_	-0.10277	0.490327
hsa_circ_	-0.31904	0.490338
hsa_circ_	-0.10289	0.490352
hsa_circ_	-0.24828	0.490367
hsa_circ_	0.130243	0.490375
hsa_circ_	-0.09889	0.490427
hsa_circ_	-0.13192	0.490437
hsa_circ_	-0.11703	0.49045
hsa_circ_	-0.08909	0.490465
hsa_circ_	-0.39988	0.490492
hsa_circ_	0.330224	0.490495
hsa_circ_	0.377907	0.4905
hsa_circ_	-0.29662	0.490506
hsa_circ_	0.179588	0.490509
hsa_circ_	0.340874	0.490516
hsa_circ_	-0.08703	0.490551
hsa_circ_	0.112777	0.490574
hsa_circ_	-0.46935	0.490576

hsa_circ_	0.164693	0.49058
hsa_circ_	0.208624	0.490581
hsa_circ_	-0.09253	0.490592
hsa_circ_	0.096791	0.490603
hsa_circ_	0.104505	0.490604
hsa_circ_	-0.09649	0.490644
hsa_circ_	0.12151	0.490654
hsa_circ_	-0.13408	0.490659
hsa_circ_	-0.17412	0.490664
hsa_circ_	-0.10016	0.490671
hsa_circ_	0.157811	0.490675
hsa_circ_	0.410408	0.49072
hsa_circ_	-0.13722	0.490721
hsa_circ_	-0.37661	0.490753
hsa_circ_	-0.12713	0.49076
hsa_circ_	0.34644	0.490784
hsa_circ_	-0.18249	0.490789
hsa_circ_	0.094825	0.490802
hsa_circ_	-0.10776	0.490803
hsa_circ_	-0.21448	0.490808
hsa_circ_	-0.21934	0.49082
hsa_circ_	-0.10249	0.490851
hsa_circ_	0.126215	0.490852
hsa_circ_	-0.1247	0.490855
hsa_circ_	-0.10243	0.490878
hsa_circ_	-0.19256	0.49089
hsa_circ_	0.095322	0.490905
hsa_circ_	-0.2324	0.490907
hsa_circ_	-0.11846	0.49092
hsa_circ_	0.717353	0.490931
hsa_circ_	0.308955	0.490941
hsa_circ_	-0.10174	0.490941
hsa_circ_	0.12031	0.490977
hsa_circ_	0.389658	0.49098
hsa_circ_	0.481578	0.491004
hsa_circ_	0.167363	0.491016
hsa_circ_	0.200362	0.491043
hsa_circ_	0.089253	0.491057
hsa_circ_	-0.16029	0.491073
hsa_circ_	0.355043	0.491098
hsa_circ_	-0.09758	0.491104
hsa_circ_	-0.11176	0.491128
hsa_circ_	0.179568	0.491163
hsa_circ_	-0.2476	0.49117
hsa_circ_	-0.38247	0.491187
hsa_circ_	0.152955	0.491195
hsa_circ_	-0.21787	0.491214
hsa_circ_	0.201763	0.491215
hsa_circ_	-0.31448	0.491231
hsa_circ_	0.375753	0.491296
hsa_circ_	-0.09723	0.491324
hsa_circ_	-0.31433	0.491334
[truncated: 385,209 more chars]
